# Overexpression of *PTPRCAP* inhibits biological function of lung adenocarcinoma through apoptosis pathway

**DOI:** 10.1371/journal.pone.0337223

**Published:** 2025-12-18

**Authors:** Yuting Yang, Baoshan Zhao, Yingjie Jiang, Xiaoli Han, Jingtao Huang, Guangrui Sun, Zongying Liang

**Affiliations:** 1 Department of Thoracic Surgery, Affiliated Hospital of Chengde Medical University, Chengde, Hebei Province, China; 2 Department of Education Division, Affiliated Hospital of Chengde Medical University, Chengde, Hebei Province, China; 3 Hebei Key Laboratory Of Panvascular Disease, Chengde, Hebei Province, China; Guangdong Medical University, CHINA

## Abstract

**Background:**

*PTPRCAP* (protein tyrosine phosphatase receptor C-associated protein) has been implicated in tumor suppression in several malignancies; however, its role in lung adenocarcinoma (LUAD) remains unclear. This study aimed to investigate the expression profile and functional significance of *PTPRCAP* in LUAD.

**Methods:**

Forty-five pairs of LUAD and adjacent non-tumor tissues were collected from patients undergoing surgery at Chengde Medical University Affiliated Hospital. *PTPRCAP* mRNA and protein levels were quantified by RT-qPCR and immunohistochemistry (IHC), respectively, and correlated with clinicopathological features. A549 and H1299 LUAD cell lines and BEAS-2B normal bronchial epithelial cells were used for in vitro assays. *PTPRCAP* was overexpressed via plasmid transfection (OE group) and compared with vector-transfected controls (Vector group). Functional assays included CCK-8 proliferation, scratch wound healing, Transwell migration/invasion, and Annexin V-PE apoptosis assays. Apoptosis-related proteins (Bax, Bcl-2, and cleaved caspase-3) were evaluated by Western blot. The effect of overexpression PTPRCAP on tumor growth was observed through nude mouse xenografts.

**Results:**

*PTPRCAP* mRNA and protein levels were significantly lower in LUAD tissues than in adjacent non-tumor tissues (P < 0.05). Low PTPRCAP expression correlated with advanced TNM stage and poor differentiation (P < 0.05). In vitro, *PTPRCAP* expression was markedly reduced in A549 and H1299 cells compared with BEAS-2B. Overexpression of *PTPRCAP* significantly suppressed proliferation, migration, and invasion (P < 0.001), and increased apoptosis rates in both cell lines (P < 0.01). Mechanistically, PTPRCAP upregulation elevated pro-apoptotic Bax and cleaved caspase-3 while downregulating anti-apoptotic Bcl-2 (P < 0.05). In vivo xenograft experiments demonstrated that overexpression PTPRCAP inhibited tumor growth in nude mice (P < 0.001).

**Conclusions:**

*PTPRCAP* is downregulated in LUAD and acts as a tumor suppressor by promoting apoptosis and inhibiting proliferation, migration, and invasion. These findings suggest *PTPRCAP* as a potential therapeutic target for LUAD.

## Introduction

Lung cancer is one of the main causes of cancer-related deaths worldwide, with its incidence and mortality rates remaining high, seriously threatening human health. Among the various pathological subtypes of lung cancer, lung adenocarcinoma is the most common type, accounting for approximately 40% of all lung cancer cases [1,2].In the early stage, patients with lung adenocarcinoma often lack specific symptoms, making it difficult to detect the disease in time. In the middle and advanced stages, lung adenocarcinoma is prone to local infiltration and distant metastasis, which significantly increases the treatment difficulty and the 5-year overall survival rate of patients is less than 20% [3]. In recent years, with the continuous progress of modern medical biotechnology and the in-depth integration of multiple disciplines, the treatment of lung adenocarcinoma has ushered in more approaches and hopes. Molecular biological targeted therapy, as a modern medical approach for treating cancer, has pointed out the direction for the treatment of lung adenocarcinoma [4]. Therefore, in-depth exploration of the molecular biological pathogenesis of lung adenocarcinoma holds significant scientific and clinical value for the development of targeted therapeutic strategies [5]. Protein tyrosine phosphatase receptor-associated protein (PTPRCAP), also known as CD45-AP, is a key regulator of the protein tyrosine phosphatase PTPRC (protein tyrosine phosphatase receptor type C/CD45). This interaction is crucial for maintaining stable CD45 expression, a member of the survival phosphatase family implicated in various cellular processes [6]. While PTPRCAP’s role has been explored in other malignancies—for instance, its aberrant expression is linked to clinicopathological features and prognosis in hepatocellular carcinoma [7,8]—its function in LUAD is poorly defined. Interestingly, a genetic variation in the PTPRCAP promoter (rs869736) is associated with increased susceptibility to diffuse-type gastric cancer by potentially elevating PTPRCAP expression [9], suggesting a context-dependent role in tumorigenesis. Given these findings in other cancers and the established importance of signaling pathways regulated by CD45 in lymphocyte activation and potentially in cancer biology, we hypothesized that PTPRCAP may function as a significant modulator of LUAD progression. Therefore, this study aims to systematically investigate the expression profile of PTPRCAP in LUAD, assess its clinical relevance, and explore its impact on LUAD cell biological functions, with a specific focus on the apoptotic pathway, to evaluate its potential as a therapeutic target.

## Methods

### Tissues and cells

Forty-five tissue specimens were obtained from patients with histologically confirmed lung adenocarcinoma who underwent thoracic surgery at the Affiliated Hospital of Chengde Medical University between 01/12/2023 and 31/06/2025. All specimens were immediately snap-frozen in liquid nitrogen and stored at -80°C until analysis. Among them, 27 were female and 18 were male; 28 cases were ≥ 60 years old and 17 cases were < 60 years old, with an average age of (62.29 ± 7. 45) years old; 21 cases were in stage I, 21 cases were in stage II and 3 cases were in stage III; 3 cases were poorly differentiated, 42 cases were moderately differentiated and 3 cases were well differentiated; 3 cases of lymphatic metastases.Inclusion criteria:① Patients with lung adenocarcinoma confirmed by pathology;② No anti-tumor treatment (radiotherapy, chemotherapy, immunotherapy or anti-tumor ready-for-use traditional Chinese medicine treatment) has been performed before taking the specimen;③ Patients who have not had any other malignancies.Exclusion criteria:① Patients with incomplete data and or other malignant tumors;② Patients who had undergone anti-tumor treatment such as radiotherapy and chemotherapy before surgery. Human lung adenocarcinoma cells A549, H1299 and normal lung epithelial cells BEAS-2B were derived from the central laboratory of Affiliated Hospital of Chengde Medical University. This study was conducted in accordance with the Declaration of Helsinki and was approved by the Ethics Committee of the Affiliated Hospital of Chengde Medical University (Approval Number: CYFYLL2022268). Informed consent was obtained from all participants prior to their inclusion in the study. The consent was obtained in written form.

### Source of reagents

Penicillin and streptomycin (Shanghai Xiaopeng Biotechnology Co., LTD.) *PTPRCAP* overexpression plasmid (Nanjing Jingpusai Biotechnology Co., LTD.) Lipofectamine 3000 transfection reagent (Invitrogen, USA). *PTPRCAP* primers, GAPDH primers, reverse transcription kits and real-time fluorescence quantitative polymerase chain reaction (RT-qPCR) kits (Tiangen Biochemical Technology (Beijing) Co., LTD.). Matrix adhesive (Biozellen Company, USA); Apoptosis detection Kit (TONBO Biosciences) CCK-8 Kit (Shandong Sikejie Biotechnology Co., LTD.) Antibody: *PTPRCAP* Antibody (Wuhan Sanying Biotechnology Co., LTD.) GAPDH Antibody (Wuhan Sewell Biotechnology Co., LTD.) BAX, Caspase-3 and Bcl-2 antibodies (Wuhan Aibotaike Biotechnology Co., LTD.).

### Cell culture, transfection, and grouping

BEAS-2B, A549 and H1299 cells were resuscitated and subcultured in RPMI-1640 medium containing 10% fetal bovine serum (FBS) and 1% penicillin/streptomycin mixture. Cells were cultured in a constant temperature incubator at 37°C with 5% CO₂ until the cell density approached 80%. Subsequently, A549 and H1299 cells were inoculated into 6-well plates, with an inoculation volume of 3 × 10⁵ cells per well. When the cell density reached 80%, the *PTPRCAP* overexpression plasmid (pLenti-CMV-PTPRCAP-EF1-GFP-Puro) and the control plasmid (pLenti-CMV-GFP-Puro) were transfected into A549 and H1299 cells respectively using Lipofectamine 3000 transfection reagent. The experiment was divided into two groups: the experimental group (OE) was transfected with the *PTPRCAP* overexpression plasmid, and the negative control group (Vector) was transfected with the empty plasmid. Following transfection, the cells were cultured under standard conditions for 48 hours. Successful transfection was confirmed by observing widespread GFP fluorescence under a microscope prior to subsequent experiments.

### Real-time fluorescence quantitative polymerase reaction (RT-qPCR)

Tissues were homogenized at low temperature using a tissue grinder to preserve RNA integrity. Total RNA was subsequently extracted from both cells and tissues using the Trizol reagent, ensuring high yield and purity. The extracted RNA was then reverse-transcribed into cDNA using a reverse transcription kit, following the manufacturer’s protocol. Real-time quantitative PCR (RT-qPCR) was performed using a fluorescence quantitative kit, with the system configuration and reaction conditions strictly adhering to the kit instructions. Relative gene expression levels were calculated using the 2^-ΔΔct^ method, which normalized the target gene expression against a reference gene and allowed for accurate comparisons across samples. *PTPRCAP* forward primer sequence 5’-CAGGACACACAGACTATGACCACG-3; Reverse primer sequence 5’-GTCACTG TCTCTGGCTTCCTCA-3’. *GAPDH* forward primer sequence 5’ ´-CGACCACTTTGACAAGCTCA-3’, reverse primer sequence 5’ ´-AGGGGTCTACATGGCAACTG-3’ ´.

### Immunohistochemistry

Tissue sections were dewaxed, followed by antigen retrieval after membrane rupture, and then incubated in a Hydrogen Peroxide Block for 10 minutes. After cooling, the sections were blocked with goat serum. The primary antibody (PTPRCAP, diluted 1:100) was added and incubated overnight at 4°C. The sections were then incubated with the secondary antibody at room temperature for 30 minutes, followed by DAB color development and hematoxylin counterstaining. The sections were subsequently dehydrated, sealed with gum, and examined under a microscope. For scoring, the percentage of positive cells was categorized as follows: 0 points for 0% ~ 5%, 1 point for 6% ~ 24%, 2 points for 25% ~ 49%, 3 points for 50% ~ 74%, and 4 points for 75% ~ 100%. Staining intensity was scored on a scale of 0 ~ 3: 0 for negative, 1 for weak, 2 for moderate, and 3 for strong staining. The Immunoreactive Score (IRS) was calculated using the formula IRS = PP*SI, where PP represents the percentage of positive cells and SI denotes the staining intensity grade. IRS ≤ 2 was classified as low expression, and >2 as high expression.

### Western blot experiment

Total protein was extracted from each group of cells and its concentration was determined. Protein samples were prepared for electrophoresis and subjected to SDS-PAGE gel electrophoresis. The separated proteins were transferred onto a methanol-activated PVDF membrane using a 400 mA constant current power supply. After transfer, the membrane was blocked with 5% skimmed milk powder at room temperature for 2 hours. Unbound milk powder was washed off with TBST buffer solution. The membrane was then incubated with primary antibodies (PTPRCAP, GAPDH, Bax, Caspase 3, Bcl-2) overnight at 4°C. The following day, the membrane was rewarmed on a shaker for 1 hour and washed three times with TBST buffer, each for 10 minutes. The secondary antibody was added and incubated on a shaker for 1 hour, followed by three 10-minute washes with TBST. The membrane was then exposed and developed using a C300 developer. Band intensities were measured and analyzed using ImageJ software, and the gray values of the target genes were normalized to the internal reference gene for statistical analysis.

### CCK-8 assay to detect cell proliferation activity

The cell suspension was counted and seeded into 96-well plates at a density of 2,500 cells per 100 µL well, with six replicate wells established for each group. Cells were cultured for 0, 24, 48, and 72 hours, respectively. After each time point, 10 µL of CCK-8 reagent was added to each well and incubated at 37°C for 2 hours. The absorbance (OD value) was then measured at 450 nm using an ELISA reader to assess cell proliferation.

### Scratch healing experiment

A549 cells and H1299 cells were seeded into 6-well plates, with three replicate wells for each group. When the cell density reached 80%, transfection was performed. After covering the bottom of the well, a vertical scratch was made using the tip of a 10 µL pipette. Floating cells were removed by washing with PBS, and the basic culture medium was added to continue culturing. The scratch area was photographed under an inverted microscope at 0 hours and 24 hours to evaluate cell migration ability.

### Transwell cell Migration Assay

Cells were collected 48 hours after transfection and adjusted to a density of 3 × 10⁴ cells per well. They were then resuspended in 200 µL of culture medium containing 1% fetal bovine serum (FBS) and seeded into the upper chamber of a Transwell insert. The lower chamber was filled with 700 µL of culture medium containing 20% FBS. After 24 hours of incubation, the culture medium was discarded, and the cells were washed twice with PBS. The cells were then fixed with methanol for 30 minutes, followed by two washes with PBS. The cells were stained with 1% crystal violet for 10 minutes, and non-migrating cells on the upper surface of the membrane were removed using a cotton swab. The cells were washed twice more with PBS, and the number of migrated cells was counted under an inverted microscope.

### Transwell cell Invasion Assay

The upper chamber of the Transwell insert was pre-coated with Matrigel matrix and allowed to solidify. A cell suspension with a density of 5 × 10⁴ cells per well was then seeded into the upper chamber, and the lower chamber was filled with 700 µL of culture medium containing 20% FBS. After 24 hours of incubation, the culture medium was discarded, and the subsequent steps were the same as those in the migration experiment.

### Apoptosis assay

Cells from each transfection group were harvested using EDTA-free trypsin to avoid interference with apoptosis markers. They were washed twice with PBS and centrifuged at 1000 rpm for 5 minutes to pellet the cells. The supernatant was discarded, and the cell pellet was resuspended in 100 µL of binding buffer. Each sample was stained with 5 µL of Annexin V-PE and 5 µL of 7-AAD, incubated in the dark for 15 minutes to allow binding to apoptotic cells. The samples were then diluted with 400 µL of binding buffer and analyzed using flow cytometry to quantify the apoptosis rate.

### Animal Experiments

A total of ten male BALB/c-nu mice (aged 6 ~ 8 weeks; weight range: 20 ~ 25g) were selected. The mice were housed in a pathogen-free facility with controlled conditions: 55% humidity, 26°C temperature, and a 12-hour light/dark cycle, with free access to food and water. The mice were randomly divided into two groups: the PTPTCAP overexpression-transfected group (OE) and the negative control group (Vector) (n = 5 per group).A total of 5 × 10⁶ A549 cells were subcutaneously injected into the right anterior limb of each mouse. After inoculation, a cotton swab was applied to prevent fluid leakage. The nude mice were maintained under the same housing conditions. Tumor formation was assessed one week post-inoculation, and tumor growth was monitored thereafter.Starting from the confirmed tumor formation, tumor volume was measured weekly. After four weeks, the mice were euthanized, and the tumors were excised and weighed. All animal experiments were approved by the Animal Welfare and Research Ethics Committee of the Affiliated Hospital of Chengde Medical University. The studies were conducted in compliance with the institutional ethical guidelines of Chengde Medical University and the AVMA Guidelines.

### Statistical analysis

Statistical analyses were performed using GraphPad Prism 10.0. Normally distributed measurement data are expressed as mean ± standard deviation. Group differences were assessed as follows: between two groups using an unpaired Student’s t-test; among multiple groups using one-way or two-way ANOVA, followed by an appropriate post hoc test. For data that violated the assumption of normality, the Mann-Whitney U test was used. Categorical data, reported as [cases (%)], were analyzed by Fisher’s exact test. The level of statistical significance was defined as *P < 0.05, **P < 0.01, ***P < 0.001, and ****P < 0.0001.

## Results

### Expression of PTPRCAP and its clinical relevance in lung adenocarcinoma

**Fig 1:**
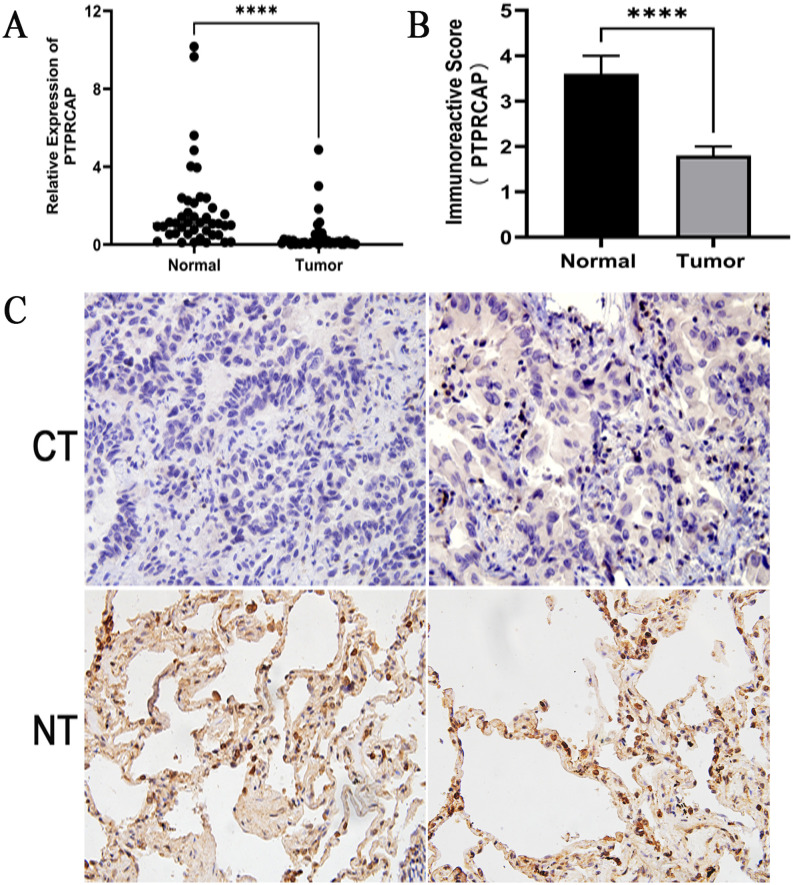
PTPRCAP expression is downregulated in lung adenocarcinoma tissues. The expression levels of PTPRCAP were compared between lung adenocarcinoma tissues (CT) and adjacent normal lung tissues (NT) from 45 patients. **(A)** PTPRCAP mRNA expression levels were quantified by qRT-PCR. **(B, C)** PTPRCAP protein expression was detected by immunohistochemical (IHC) analysis. Quantitative analysis of the immunoreactivity score (IRS) is presented in **(B)**, and representative IHC images are shown in **(C)**. Data in **(A)** and **(B)** are presented as box and whisker plots showing the median with interquartile range. ****P < 0.0001. Statistical significance was determined by the Mann-Whitney U test.

**Fig 2:**
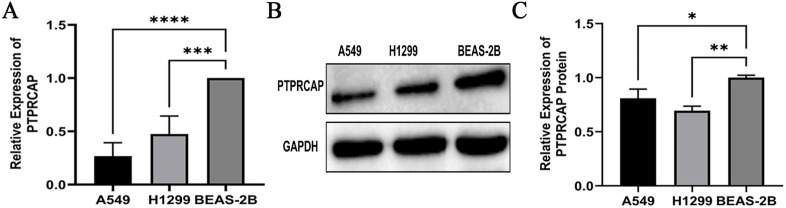
PTPRCAP expression is downregulated in lung cancer cell lines. The expression levels of PTPRCAP were compared between the lung epithelial cell line BEAS-2B and the lung cancer cell lines A549 and H1299. **(A)** PTPRCAP mRNA expression levels were quantified by qRT-PCR and normalized to GAPDH. **(B, C)** PTPRCAP protein expression levels were detected by western blot analysis, with GAPDH serving as a loading control. Quantitative analysis of protein levels is shown in **(C)**. Data are presented as mean ± SD. *P < 0.05, **P < 0.01, ***P < 0.001, ****P < 0.0001. Statistical significance was determined by one-way ANOVA.

RT-qPCR analysis revealed a significant downregulation of PTPRCAP mRNA in lung adenocarcinoma tissues (median: 0.107, IQR: 0.053–0.288) compared to matched adjacent non-tumor tissues (median: 1.084, IQR: 0.535–2.166), representing an approximate tenfold decrease (Mann-Whitney U = 257, P < 0.0001; n = 45 patient samples; Fig 1A). Consistent with the mRNA findings, immunohistochemical analysis demonstrated a substantially lower positive expression rate of PTPRCAP protein in tumor tissues (22.22%, 10/45) relative to paracancerous tissues (93.33%, 42/45), with a concomitantly reduced immunoreactivity score (IRS) (P < 0.0001, Fig 2). Clinico-pathological correlation analysis indicated that the positive expression rate of PTPRCAP protein was not significantly associated with patient age, gender, or lymph node metastasis status (all P > 0.05), but showed significant associations with TNM stage and histological differentiation grade (P < 0.05; Table 1).

**Table 1:** Association between PTPRCAP expression and clinicopathological characteristics of patients with lung adenocarcinoma.

Clinicopathological Characteristic	Total(n = 45)	PTPRCAP High Expression(n = 10)	PTPRCAP Low Expression(n = 35)	*P* value
Gender				0.489
Male	18	5(27.78%)	13(72.22%)	
Female	27	5(18.50%)	22(81.48%)	
Age(years)				0.719
≥60	28	7(25.00%)	21(75.00%)	
< 60	17	3(17.65%)	14(82.35%)	
TNM stage				0.009**
Ⅰ stage	23	9(39.13%)	5(60.87%)	
Ⅱ stage	22	1(4.55%)	5(95.45%)	
Histodifferentiation				0.017*
Well	3	3(100.00%)	0(0.00%)	
Moderately	39	7(17.95%)	32(82.05%)	
Poorly	3	0(0.00%)	3(100.00%)	
Lymphatic metastasis				>0.999
Metastasis	6	1(16.70%)	5(27.80%)	
No metastasis	39	9(23.10%)	5(27.80%)	

*P* values were calculated using the Fisher’s exact test for categorical variables. A *P* value of less than 0.05 was considered statistically significant.*P < 0.05, **P < 0.01

### Expression of *PTPRCAP* in lung cancer cell lines

The expression of PTPRCAP was significantly downregulated in lung cancer cell lines compared to the lung epithelial cell line BEAS-2B. At the mRNA level, quantitative RT-PCR analysis revealed that PTPRCAP expression was substantially lower in both A549 and H1299 cells than in BEAS-2B cells (Fig 2A). This reduction was confirmed at the protein level by western blot analysis (Fig 2B and 2C).

### Transfection efficiency of *PTPRCAP* in A549 and H1299 cells

**Fig 3:**
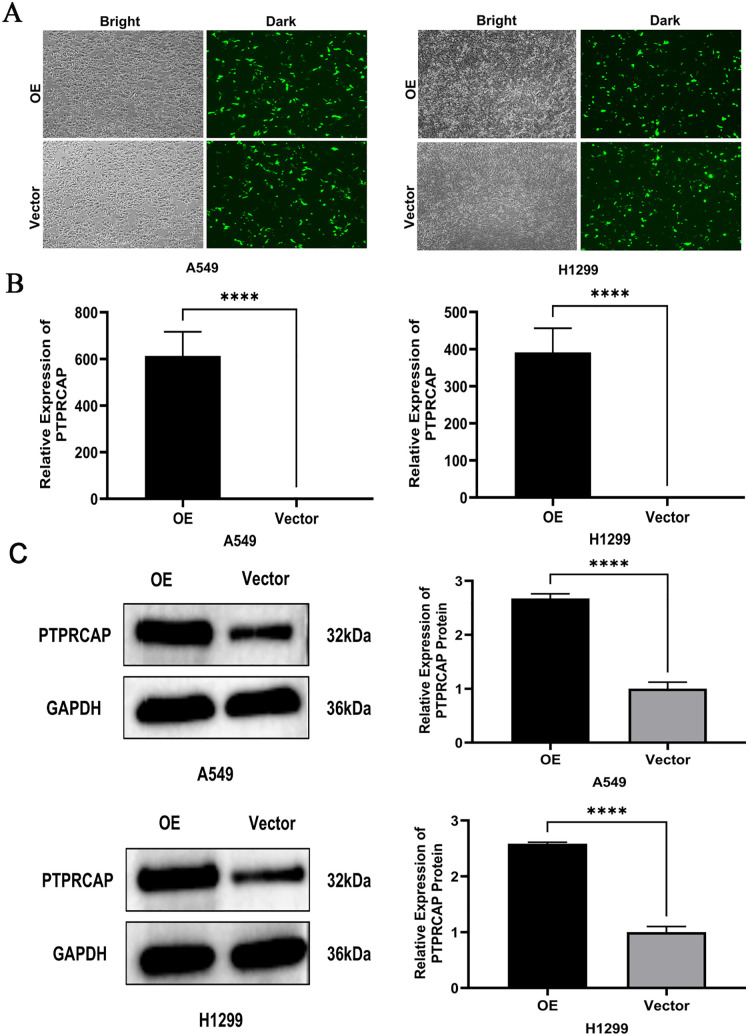
Transfection efficiency of PTPRCAP in A549 and H1299 cells. **(A)** Representative fluorescence microscopy images confirming transfection efficiency in A549 and H1299 cells transfected with a GFP-tagged plasmid. **(B,C)** The success of PTPRCAP overexpression was verified by measuring **(B)** mRNA expression levels using qRT-PCR and **(C)** protein expression levels using western blot analysis. Data are presented as mean ± SD. ****P < 0.0001. Statistical significance was determined by Student’s t-test. OE, cells transfected with PTPRCAP overexpression plasmids; Vector, cells transfected with empty plasmid.

After transfection of overexpression plasmid and control plasmid, the transfection efficiency was initially revealed under fluorescence microscope (Fig 3A); RT-qPCR results showed that the expression of *PTPRCAP* in the OE group in A549 and H1299 cells was significantly higher than that in the Vector group, and the difference was statistically significant (P < 0.001, n = 3, Fig 3B), indicating that overexpression model was successfully constructed in A549 cells and H1299 cells; After transfection of the overexpression plasmid, the Western blot results showed that the protein expression of *PTPRCAP* in the OE group was significantly higher than that in the Vector group in the two cells, and the difference was statistically significant (P < 0.001, n = 3, Fig 3C).

### Effect of overexpression of *PTPRCAP* on proliferation ability of A549 and H1299 cells

**Fig 4:**
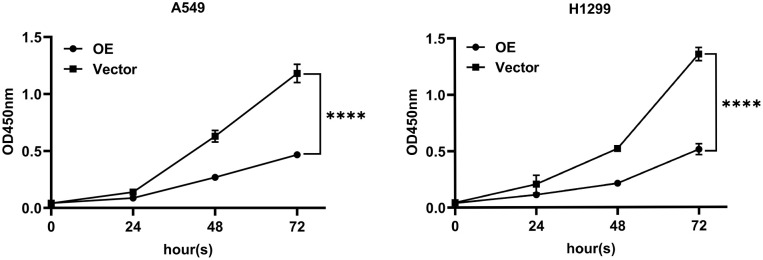
PTPRCAP overexpression inhibits the proliferation of A549 and H1299 cells. Cell proliferation was assessed by CCK-8 assay at 24, 48 and 72 hours post-seeding. The proliferative capacity of both A549 and H1299 cells was significantly inhibited in the OE group compared to the Vector group. Data are presented as mean ± SD. ****P < 0.0001. Statistical significance was determined by two-way ANOVA. OE, cells transfected with PTPRCAP overexpression plasmids; Vector, cells transfected with empty plasmid.

The results of CCK8 experiment showed that compared with the Vector group, the proliferation ability of A549 and H1299 cells in the OE group was significantly reduced (P < 0.001, n = 3, Fig 4).

### Effect of overexpression of PTPRCAP on the migration ability of A549 and H1299 cells

**Fig 5:**
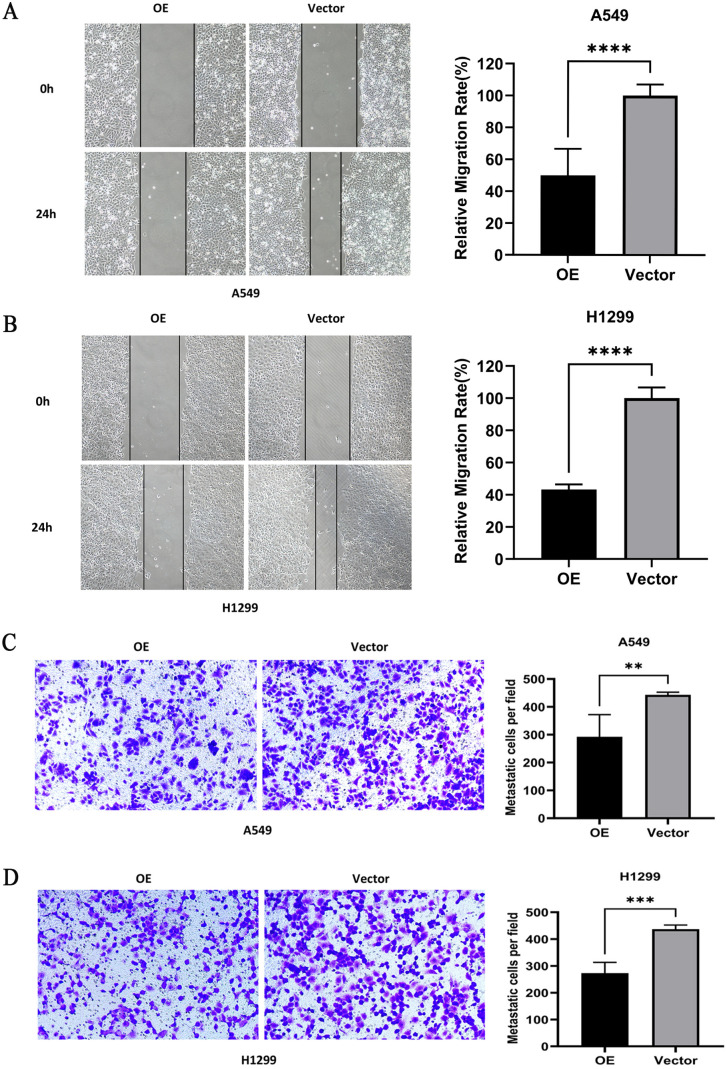
Overexpression of PTPRCAP inhibits the migration of A549 and H1299 cells. Cell migration was assessed using wound healing and Transwell assays following transfection. **(A, B)** Wound healing assays for A549 and H1299 cells. **(C, D)** Transwell migration assays (crystal violet staining) for A549 and H1299 cells. Quantitative data demonstrate that PTPRCAP overexpression significantly impaired cell migration compared to the Vector group. Data are presented as mean ± SD. **P < 0.01, ***P < 0.001, ****P < 0.0001. Statistical significance was determined by Student’s t-test. OE: cells transfected with PTPRCAP overexpression plasmids; Vector: cells transfected with empty plasmid.

The results of scratch healing experiment showed that the migration ability of OE group in A549 and H1299 cells were significantly weaker than that of Vector group (P < 0.001, n = 4, Fig 5A and 5B); The results of transwell chamber migration experiment showed that compared with the Vector group, the number of cell migration in the OE group in A549 and H1299 cells was significantly reduced (P < 0.01, n = 4, Fig 5C and 5D).

### Effect of overexpression of *PTPRCAP* on invasion ability of A549 and H1299 cells

**Fig 6:**
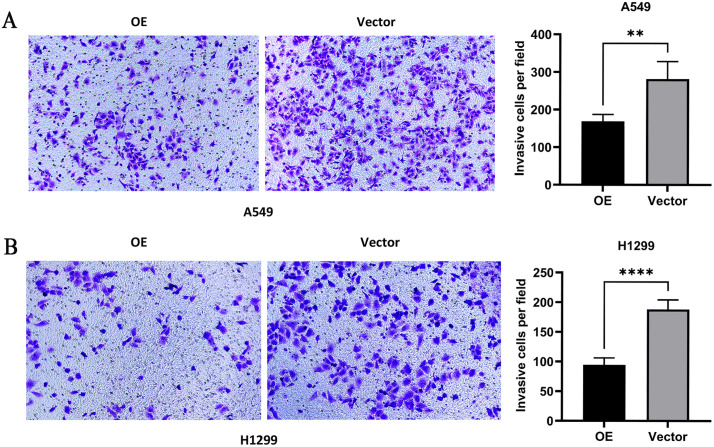
PTPRCAP overexpression inhibits the invasion of A549 and H1299 cells. The invasive capability of cells was assessed using Matrigel invasion assays following transfection. Representative images and quantitative analysis show that PTPRCAP overexpression significantly suppressed the invasion of both **(A)** A549 and **(B)** H1299 cells compared to the Vector group. Data are presented as mean ± SD. **P < 0.01, ****P < 0.0001. Statistical significance was determined by Student’s t-test. OE: cells transfected with PTPRCAP overexpression plasmids; Vector: cells transfected with empty plasmid.

The results of Transwell chamber invasion experiment showed that compared with the Vector group, the number of cell invasion and penetration of membranes in the OE group in A549 and H1299 cells was significantly reduced (P < 0.01, Fig 6A and 6B).

### Effect of overexpression of *PTPRCAP* on apoptosis of A549 and H1299 cells

**Fig 7:**
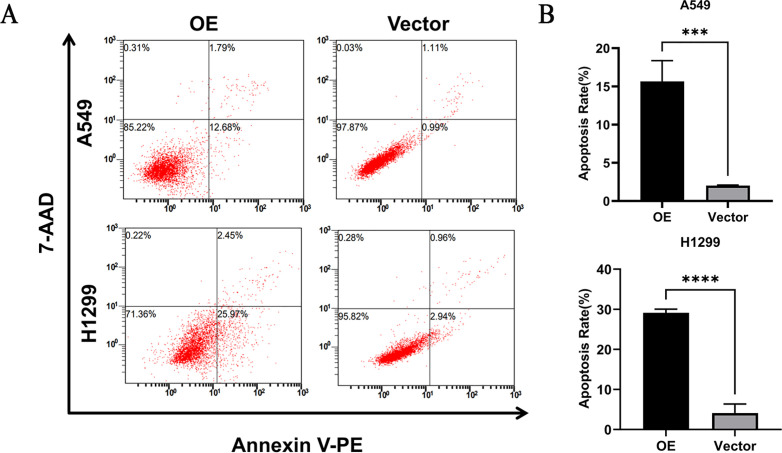
Effect of *PTPRCAP* overexpression on apoptosis of lung adenocarcinoma cells. **(A)** Flow cytometry analysis of apoptosis in PTPRCAP-overexpressing A549 and H1299 cells. **(B)** Flow cytometric comparison of apoptosis rates in PTPRCAP-overexpressing lung adenocarcinoma cells. Data are presented as mean ± SD. ***P < 0.001, ****P < 0.0001. Statistical significance was determined by Student’s t-test. OE: cells transfected with PTPRCAP overexpression plasmids; Vector: cells transfected with empty plasmid.

The results of flow cytometry experiments showed that the apoptotic ability of OE group cells in A549 and H1299 cells was significantly higher than that in Vector group (P < 0.01, n = 3, Fig 7A and 7B).

### Overexpression *PTPRCAP* activates the apoptosis signaling pathway in A549 and H1299 cells

**Fig 8:**
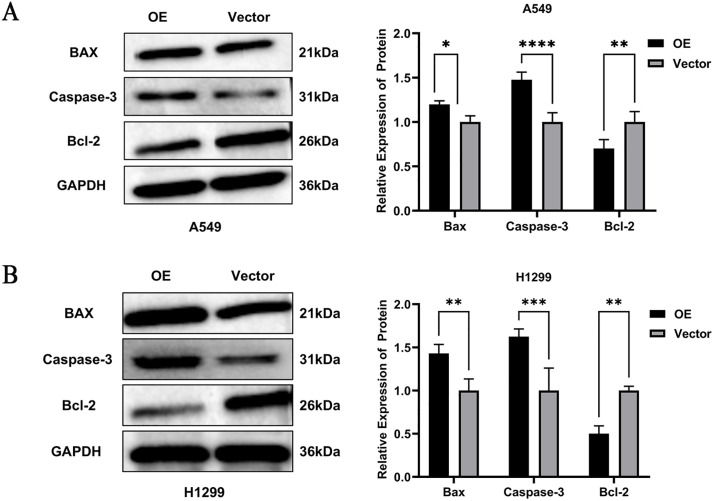
Overexpression PTPRCAP activates the apoptosis signaling pathway. **(A)**. Western blot analysis of BAX and Caspase-3 and Bcl-2 in the A549 cells following transfection with OE or Voctor. **(B)** Overexpression PTPRCAP significantly inhibited the protein levels of Bcl-2 compared with the vector groups, but the expression level of BAX and Caspase-3 increased in H1299 cells. Data are presented as mean ± SD. ***P < 0.001, ****P < 0.0001. Statistical significance was determined by Student’s t-test. OE: cells transfected with PTPRCAP overexpression plasmids; Vector: cells transfected with empty plasmid.

Western blot analysis was performed to examine the expression levels of key proteins components in the apoptosis signaling pathway. The results demonstrated that upregulation of PTPRCAP expression in lung adenocarcinoma A549 cells significantly inhibited the protein levels of Bcl-2 compared with the vector groups, but the expression level of BAX and Caspase-3 increased (P < 0.05, n = 3, Fig 8A). The results in H1299 cells were consistent with those in A549 cells(P < 0.05, n = 3, Fig 8B).These findings collectively indicate that PTPRCAP overexpression directly activates the apoptosis signaling pathway, thereby inhibiting tumor cell proliferation, invasion and migration capabilities,promoting cell apoptosis.

### Overexpression *PTPRCAP* inhibits lung adenocarcinoma tumors growth in vivo

**Fig 9:**
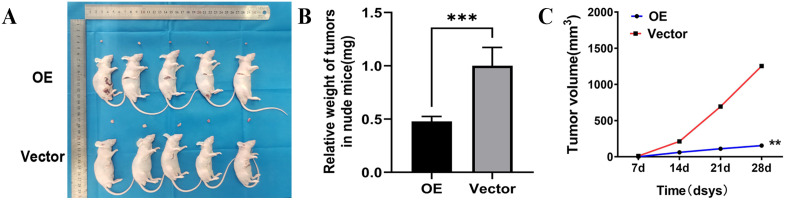
Overexpression PTPRCAP inhibits A549 cell tumor formation and growth. **(A)** Images of the xenograft tumors from mice injected with OE or Vector cells. Following injection of the A549 cells for 28 days, the mice were sacrificed and the xenograft tumors were collected. **(B)** The weight of the subcutaneous xenografts tumors was lower from mice injected with OE. **(C)** The tumor volume of OE group was smaller than Vector group.(Nude mice each group n = 5, Two groups).**P < 0.01, ***P < 0.001. OE, cells transfected with PTPRCAP overexpression plasmids; Vector, cells transfected with empty plasmid.

To confirm whether overexpression PTPRCAP may serve the same function in the suppression of tumor growth in vivo, a subcutaneous xenograft model was used. Consistent with the in vitro results, upregulation of PTPRCAP expression significantly reduced the tumor weight (P < 0.01, Fig.9A and 9B) and markedly decreased the tumor volume compared with the mice injected with control cells (P < 0.01, Fig 9A and 9C).

## Discussion

Lung cancer, as a malignant tumor with extremely high morbidity and mortality rates worldwide, poses a serious threat to human health [10]. Among the various histological types of lung cancer, lung adenocarcinoma (LUAD) is the most common one, accounting for a significant proportion of lung cancer cases. Despite considerable progress in the treatment of lung adenocarcinoma in recent years, including the comprehensive application of multiple approaches such as surgery, radiotherapy, chemotherapy, targeted therapy, and immunotherapy, the treatment outcomes still pose challenges. Particularly for patients with stage IA lung adenocarcinoma, even after comprehensive treatment, the 5-year recurrence-free survival rate only reaches 63% to 81% [11]. This data indicates that despite the continuous enrichment of treatment methods, some patients still face the risk of recurrence. Therefore, in-depth exploration of the molecular mechanisms of lung cancer, especially those related to non-small cell lung cancer (NSCLC), is of extremely significant importance for developing more effective treatment strategies, improving the long-term survival rate and quality of life of patients.

*PTPRCAP* (Protein Tyrosine Phosphatase Receptor C-type Associated Protein), also known as CD45-AP or LPAP, is an important transmembrane phosphorylated protein. It plays a crucial role in maintaining the stable expression of the tyrosine phosphatase PTPRC/CD45(protein tyrosine phosphatase receptor type C/CD45) by specifically binding to it. The PTP(protein tyrosine phosphatase) family members encoded by the PTPRC gene have complex dual roles in tumor biology: on the one hand, they can act as tumor suppressors, preventing tumor progression; on the other hand, they may also act as oncogenes, promoting tumor development [12]. Further research has revealed a potential link between the secondary allele of rs869736 in the promoter region of the *PTPRCAP* gene and the susceptibility to diffuse-type gastric cancer. Specifically, this allele may affect the risk of gastric cancer by increasing the expression level of *PTPRCAP*. The T allele enhances the activity of the promoter and binds to nuclear proteins, significantly increasing the expression level of *PTPRCAP*, which may increase the risk of gastric cancer. This discovery provides a new perspective for understanding the role of *PTPRCAP* in tumor development. Additionally, *PTPRCAP* may also promote tumor progression through multiple mechanisms. It may activate the SRC family kinases (SFKs) and interfere with the cell adhesion mediated by E-cadherin, promoting the invasion and metastasis of tumor cells [9]. Recent studies have continuously revealed the role of *PTPRCAP* in various cancers. In the TCGA cohort study, DDR deficiency exhibited unique immune characteristics, closely related to the specific immune characteristics of ovarian cancer. Particularly in ovarian cancer with DNA repair system defects, the expression of *PTPRCAP* significantly increased, suggesting that *PTPRCAP* may play an important role in the immune response of ovarian cancer [13]. In the field of breast cancer, researchers such as MARCHETTI [14] have identified genes positively correlated with the disease-free survival rate of patients with triple-negative breast cancer through bioinformatics methods. *PTPRCAP* was among them. Further RT-PCR and Western blot experiments confirmed that *PTPRCAP* was expressed at a lower level in triple-negative breast cancer cell lines, providing experimental support for its role in breast cancer.In the field of lung adenocarcinoma, researchers such as GILLETTE [15] conducted a comprehensive proteogenomic study on a prospectively collected cohort of lung adenocarcinoma cases. They discovered evidence that 120 proteins, including *PTPRCAP*, are regulated by DNA methylation, providing new insights into the biology of LUAD. Furthermore, the stem cell index based on mRNA expression (mRNAsi) serves as an indicator for quantifying the similarity between tumors and stem cells, reflecting the stem cell characteristics of the transcriptome. Recent studies have shown that *PTPRCAP* is the only gene that is associated with the stemness index in both peripheral blood and lung cancer tumor tissues. This further highlights the potential importance of *PTPRCAP* in lung cancer, but its specific role requires further investigation [16]. Although research on the occurrence and development of lung cancer is ongoing, the expression and role of *PTPRCAP* in lung cancer have not yet reached a clear conclusion.

Abnormalities in the apoptosis pathway play a crucial role in the occurrence of tumors. Apoptosis is a natural process in the cell life cycle, but the dysregulation of its regulatory mechanism is often closely related to the occurrence and development of tumors. Bax is a representative pro-apoptotic gene, which plays a key role in the initiation of apoptosis. Bcl-2 is a key protein that inhibits apoptosis, and it prevents the occurrence of apoptosis by interacting with pro-apoptotic proteins. Caspase-3 is the most important apoptotic execution protein in the Caspase cascade reaction, which is activated in the final stage of apoptosis, leading to the disintegration and death of the cell [17]. Studies have shown that BAX is highly expressed in a variety of cancers and is closely associated with poor prognosis in nine types of cancers [18]. This indicates that the abnormal expression of BAX may be an important tumor marker, and its role in cell apoptosis cannot be ignored. Numerous studies have also revealed that proteins related to the apoptotic pathway, such as BAX, Bcl-2 and Caspase-3, play significant roles in the occurrence of malignant tumors such as esophageal cancer [19], breast cancer [20], pancreatic cancer [21] and prostate cancer [22]. These research results further emphasize the significance of the apoptotic pathway in tumor biology. In lung cancer, the apoptotic pathway also plays an important role. Lung cancer is one of the most common malignant tumors worldwide. Its high incidence and mortality rates make it a major challenge in the field of public health. Abnormalities in the apoptotic pathway play a key role in the occurrence and development of lung cancer. For instance, the research conducted by LI [23] et al. revealed that Ligusticum chuanxiong can increase the ratio of Bax/Bcl-2, promote the overexpression of Caspase-9, further activate the downstream protein Caspase-3, and ultimately lead to the apoptosis of A549 cells. This discovery has revealed the potential application value of Ligusticum chuanxiong in the treatment of lung cancer, and also provided new ideas for studying the role of the apoptotic pathway in lung cancer.The latest research indicates that the NADPH oxidase inhibitor roboxin (AP) can significantly reduce the survival ability of lung cancer A549 cells, induce cell apoptosis, and simultaneously increase the expression of Bax and Caspase-3, while reducing the expression of Bcl-2 [24]. These results suggest that lobbietin may exert its anti-tumor effect by regulating the apoptotic pathway, providing a new direction for targeted therapy of lung cancer. However, at present, no research has clearly defined the specific mechanism of action of *PTPRCAP* in lung cancer.

This study provides evidence that PTPRCAP acts as a potential tumor suppressor in lung adenocarcinoma (LUAD). We found that PTPRCAP expression is significantly downregulated in LUAD tissues and cell lines compared to normal counterparts. Functionally, restoring PTPRCAP expression in LUAD cells (A549 and H1299) markedly suppressed proliferation, migration, and invasion, while promoting apoptosis. These findings position PTPRCAP as a significant negative regulator of LUAD aggressiveness. In this study, we intentionally selected two LUAD cell lines with distinct genetic backgrounds and phenotypic characteristics—A549 (p53 wild-type, epithelial-like) and H1299 (p53-null, mesenchymal-like)—for functional validation. This selection was based on the following important considerations: First, LUAD patient tumors themselves are highly heterogeneous, exhibiting significant differences particularly in TP53 mutation status and the degree of epithelial-mesenchymal transition (EMT). p53 is a key regulator of cellular stress responses (including DNA damage and oncogene activation), and its status profoundly influences cell proliferation, apoptosis, and response to therapy. The EMT status, meanwhile, is closely associated with cell migration, invasion, and stem cell-like properties. By replicating the experiments in these two cell models representing different LUAD subgroups, we were able to assess the generality and robustness of the effects of PTPRCAP overexpression. It is compelling that despite the fundamental differences between A549 and H1299 cells, PTPRCAP overexpression consistently inhibited proliferation, migration, and invasion, while promoting apoptosis, in both. This result strongly suggests that the tumor-suppressive function of PTPRCAP is independent of p53 status and not strictly constrained by the baseline EMT phenotype of the cells. This significantly enhances the reliability and clinical relevance of our conclusions, indicating that therapeutic strategies targeting PTPRCAP could be applicable to a broader spectrum of LUAD patient populations, not limited to a specific molecular subtype.

A key insight from our work is the involvement of the apoptotic pathway. Flow cytometry analysis confirmed a significant increase in apoptosis upon PTPRCAP overexpression. Western blot analysis further revealed that this pro-apoptotic effect was associated with an upregulation of the pro-apoptotic protein BAX and the executioner caspase, Caspase-3, alongside a downregulation of the anti-apoptotic protein Bcl-2. This shift in the balance towards apoptosis execution provides a plausible mechanism for the observed tumor-suppressive effects of PTPRCAP. While the precise upstream signaling linking PTPRCAP to this apoptotic axis requires further investigation, our data strongly suggest that modulating the BAX/Bcl-2 ratio and activating Caspase-3 is a critical downstream consequence of PTPRCAP activity in LUAD cells.

Interpreting our results in the broader context of PTPRCAP biology requires careful consideration. PTPRCAP is best characterized for its role in lymphocyte activation, where it binds to and stabilizes CD45 (PTPRC), a key regulator of T- and B-cell receptor signaling [12]. Targeting PTPRCAP for LUAD treatment raises the question of its effect on this pathway. While our study did not directly assess lymphocyte function, the tumor-suppressive effects we observed in LUAD cells appear to be mediated through an intrinsic apoptotic mechanism within the cancer cells themselves. It is possible that PTPRCAP has cell-type-specific functions. In lymphocytes, it supports CD45’s role in immune activation, whereas in LUAD epithelial cells, its presence may engage different signaling networks that ultimately converge on apoptosis. Future studies should explore whether PTPRCAP expression in LUAD influences tumor-immune interactions, but our current findings highlight a direct, cell-autonomous role in inhibiting cancer cell survival.

The observation of low PTPRCAP expression in LUAD prompts the question of its underlying cause. A known minor allele of rs869736 in the PTPRCAP promoter is associated with increased PTPRCAP expression and higher risk for diffuse-type gastric cancer [9]. Our study, however, focused on the functional consequences of PTPRCAP expression and did not investigate the genetic variations responsible for its low expression in LUAD. This remains an important area for future research. The downregulation we observed could be due to other mechanisms, such as promoter hypermethylation, as suggested by proteogenomic studies linking PTPRCAP regulation to DNA methylation in LUAD [15], or through alterations in transcriptional regulators distinct from those affected by rs869736. Elucidating the precise epigenetic or genetic basis for PTPRCAP suppression in LUAD is a crucial next step to understand its deregulation fully.

Our study observed low expression levels of both PTPRCAP mRNA and protein in LUAD tissues, suggesting that its downregulation may occur at the transcriptional level. However, this does not exclude the possibility of defects in post-transcriptional regulatory mechanisms. The low expression of PTPRCAP may result from the combined effects of multiple mechanisms: Transcriptional-level regulation: As discussed earlier, promoter hypermethylation is a common mechanism leading to gene silencing. Additionally, alterations in transcription factor activity (e.g., activation of repressors or inactivation of activators) may directly lead to reduced mRNA transcription. Post-transcriptional regulation: Even if mRNA is transcribed, its stability, nuclear export, and translation efficiency are finely regulated. microRNAs (miRNAs) or long non-coding RNAs (lncRNAs) may bind to the 3’-untranslated region (3’-UTR) of PTPRCAP mRNA, promoting its degradation or inhibiting its translation, thereby leading to a further disproportionate decrease in protein expression relative to mRNA levels. Our data showing low levels of both mRNA and protein tend to suggest that the primary issue lies at the transcriptional level, but post-transcriptional regulation may exacerbate this phenomenon. Protein-level regulation: The PTPRCAP protein itself may be rapidly degraded via the ubiquitin-proteasome pathway or autophagy-lysosome pathway, resulting in the maintenance of low steady-state protein levels. As this study primarily focused on the expression levels and functional effects of PTPRCAP, the specific regulatory mechanisms mentioned above have not been thoroughly investigated. Therefore, elucidating the dominant mechanism behind PTPRCAP downregulation in LUAD—whether it is transcriptional inhibition (e.g., methylation), post-transcriptional regulation (e.g., miRNA action), or accelerated protein degradation—represents an important direction for future research. Determining the primary mechanism will be crucial for developing therapeutic strategies to restore its expression (e.g., using demethylating agents or targeting specific miRNAs).

Our study has limitations. The sample size was relatively small, and only two cell lines were used for functional assays. Future work should validate these findings in larger cohorts and a broader panel of LUAD models. Furthermore, generating PTPRCAP-knockout models would provide complementary evidence to our gain-of-function approach. Perhaps most importantly, the specific signaling pathways upstream of the apoptotic cascade that are modulated by PTPRCAP in LUAD cells remain to be identified. Investigating the interactome of PTPRCAP in lung cancer cells will be essential to map the molecular network through which it exerts its tumor-suppressive effects.

In conclusion, our results demonstrate that PTPRCAP is frequently downregulated in LUAD and that its re-expression inhibits malignant phenotypes, at least in part, by activating the intrinsic apoptotic pathway. These findings propose PTPRCAP as a potential novel biomarker and a candidate therapeutic target for LUAD. Future research should focus on delineating the precise mechanism of its action and the reasons for its loss in LUAD, paving the way for targeted strategies to reactivate this pathway.

## Supporting information

S1 Fig Tumor formation in nude mice. (JPG)

S1 File A549-H1299-Apoptosis. (PDF)

S2 File Raw data of Western Blots Images. (PDF)

S3 File A549-Scratch original image. (PDF)

S4 File H1299-Scratch original image. (PDF)

S5 File Green fluorescence transfection efficiency. (PDF)

S6 File OE-A549-Transwell invasion original image. (ZIP)

S7 File OE-A549-Transwell migration original image. (ZIP)

S8 File OE-H1299-Transwell invasion original image. (ZIP)

S9 File OE-H1299-Transwell migration original image. (ZIP)

S10 File OE-PCR basic verification. (ZIP)

S11 File PCR of lung adenocarcinoma tissue. (ZIP)

S12 File Statistical file. (ZIP)

S13 File IHC image-1. (ZIP)

S14 File IHC image-2. (ZIP)

S15 File IHC image-3. (ZIP)

S16 File IHC image-4. (ZIP)

S17 File IHC image-5. (ZIP)

S18 File IHC image-6. (ZIP)

S19 File IHC image-7. (ZIP)

S20 File IHC image-8. (ZIP)

S21 File IHC image-9. (ZIP)

S22 File IHC image-10. (ZIP)

S23 File IHC image-11. (ZIP)

S24 File IHC image-12. (ZIP)

S25 File IHC image-13. (ZIP)

S26 File IHC image-14. (ZIP)

S27 File IHC image-15. (ZIP)

S28 File IHC image-16. (ZIP)

S29 File IHC image-17. (ZIP)

S30 File IHC image-18. (ZIP)

S31 File IHC image-19. (ZIP)

S32 File IHC image-20. (ZIP)

S33 File IHC image-21. (ZIP)

S34 File IHC image-22. (ZIP)

S35 File IHC image-23. (ZIP)

S36 File IHC image-24. (ZIP)

S37 File IHC image-25. (ZIP)

S38 File IHC image-26. (ZIP)

S39 File IHC image-27. (ZIP)

S40 File IHC image-28. (ZIP)

S41 File IHC image-29. (ZIP)

S42 File IHC image-30. (ZIP)

S43 File IHC image-31. (ZIP)

S44 File IHC image-32. (ZIP)

S45 File IHC image-33. (ZIP)

S46 File IHC image-34. (ZIP)

S47 File IHC image-35. (ZIP)

S48 File IHC image-36. (ZIP)

S49 File IHC image-37. (ZIP)

S50 File IHC image-38. (ZIP)

S51 File IHC image-39. (ZIP)

S52 File IHC image-40. (ZIP)

S53 File IHC image-41. (ZIP)

S54 File IHC image-42. (ZIP)

S55 File IHC image-43. (ZIP)

S56 File IHC image-44. (ZIP)

S57 File IHC image-45. (ZIP)
