# Supplementary figures and images for: Overexpression of PTPRCAP inhibits biological function of lung adenocarcinoma through apoptosis pathway (part 1 of 5)
Source: PLoS One. 2025 Dec 18;20(12):e0337223. doi: 10.1371/journal.pone.0337223 (PMC12716888; doi:10.1371/journal.pone.0337223)

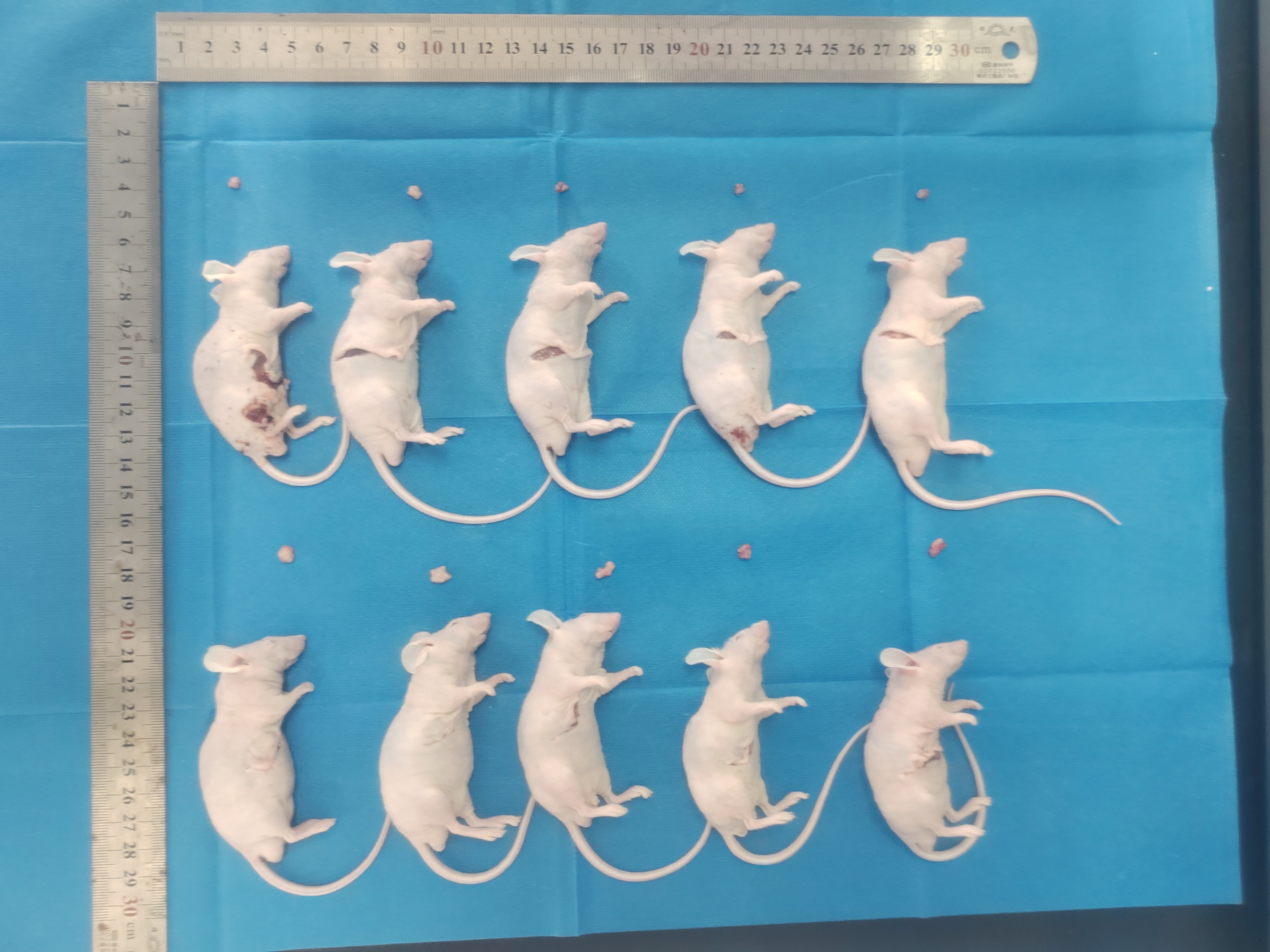

Supplement: S1 Fig — (JPG) [file pone.0337223.s001.jpg]

# A549-OE

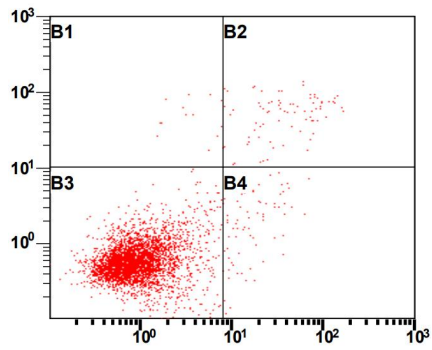

|    |      |       |       |
|----|------|-------|-------|
| B1 | 31   | 0.31  | 0.31  |
| B2 | 179  | 1.79  | 1.79  |
| B3 | 8522 | 85.22 | 85.22 |
| B4 | 1268 | 12.68 | 12.68 |

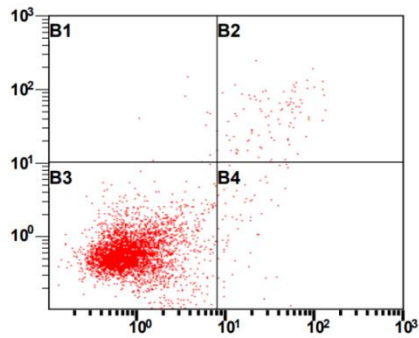

|    |      |       |       |
|----|------|-------|-------|
| B1 | 23   | 0.23  | 0.23  |
| B2 | 165  | 1.65  | 1.65  |
| B3 | 8601 | 86.01 | 86.01 |
| B4 | 1211 | 12.11 | 12.11 |

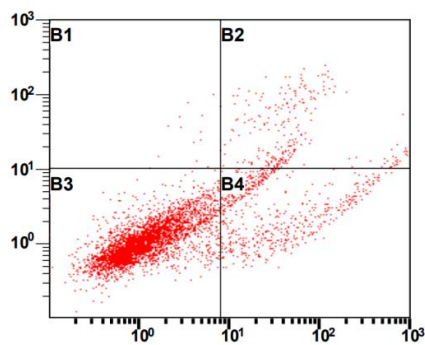

|    |      |       |       |
|----|------|-------|-------|
| B1 | 29   | 0.29  | 0.29  |
| B2 | 613  | 6.13  | 6.13  |
| B3 | 8095 | 80.95 | 80.95 |
| B4 | 1263 | 12.63 | 12.63 |

# A549-Vector

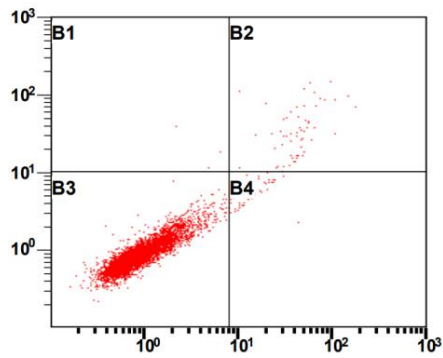

|    |      |       |       |
|----|------|-------|-------|
| B1 | 3    | 0.03  | 0.03  |
| B2 | 111  | 1.11  | 1.11  |
| B3 | 9787 | 97.87 | 97.87 |
| B4 | 99   | 0.99  | 0.99  |

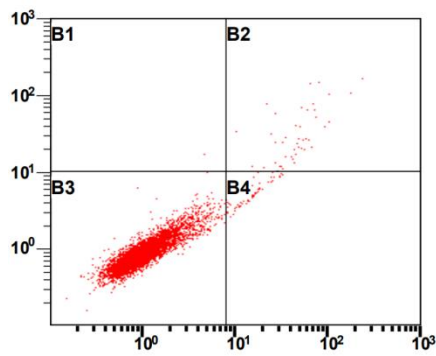

|    |      |       |       |
|----|------|-------|-------|
| B1 | 5    | 0.05  | 0.05  |
| B2 | 91   | 0.91  | 0.91  |
| B3 | 9793 | 97.93 | 97.93 |
| B4 | 111  | 1.11  | 1.11  |

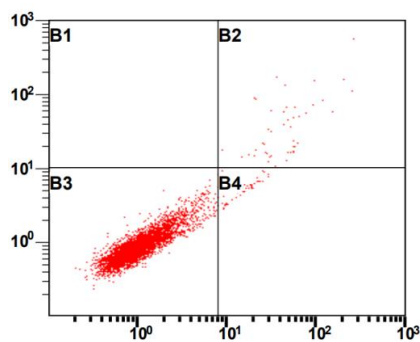

|    |      |       |       |
|----|------|-------|-------|
| B1 | 4    | 0.04  | 0.04  |
| B2 | 84   | 0.84  | 0.84  |
| B3 | 9799 | 97.99 | 97.99 |
| B4 | 113  | 1.13  | 1.13  |

# H1299-OE

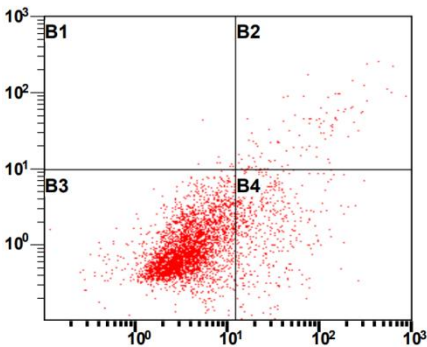

|    |      |       |       |
|----|------|-------|-------|
| B1 | 22   | 0.22  | 0.22  |
| B2 | 245  | 2.45  | 2.45  |
| B3 | 7136 | 71.36 | 71.36 |
| B4 | 2597 | 25.97 | 25.97 |

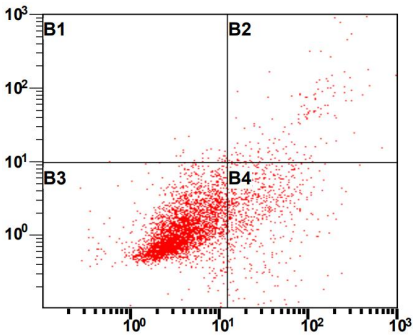

|    |      |       |       |
|----|------|-------|-------|
| B1 | 66   | 0.66  | 0.66  |
| B2 | 363  | 3.63  | 3.63  |
| B3 | 7052 | 70.52 | 70.52 |
| B4 | 2519 | 25.19 | 25.19 |

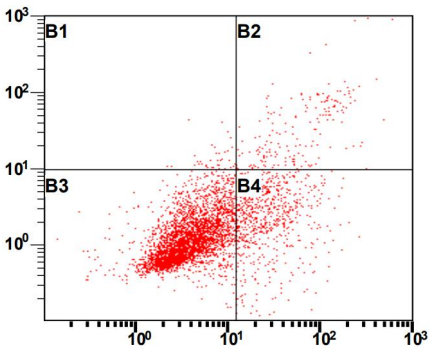

|    |      |       |       |
|----|------|-------|-------|
| B1 | 67   | 0.67  | 0.67  |
| B2 | 400  | 4.00  | 4.00  |
| B3 | 6921 | 69.21 | 69.21 |
| B4 | 2612 | 26.12 | 26.12 |

# H1299-Vector

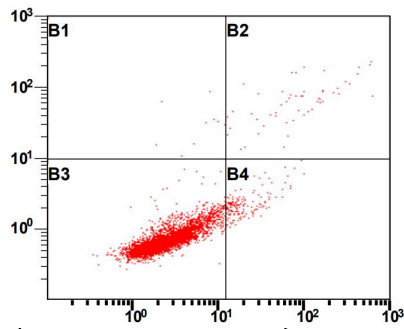

|    |      |       |       |
|----|------|-------|-------|
| B1 | 28   | 0.28  | 0.28  |
| B2 | 96   | 0.96  | 0.96  |
| B3 | 9582 | 95.82 | 95.82 |
| B4 | 294  | 2.94  | 2.94  |

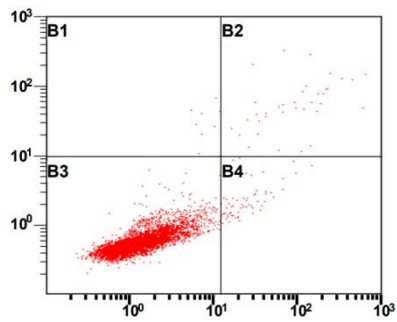

|    |      |       |       |
|----|------|-------|-------|
| B1 | 14   | 0.14  | 0.14  |
| B2 | 76   | 0.76  | 0.76  |
| B3 | 9788 | 97.88 | 97.88 |
| B4 | 122  | 1.22  | 1.22  |

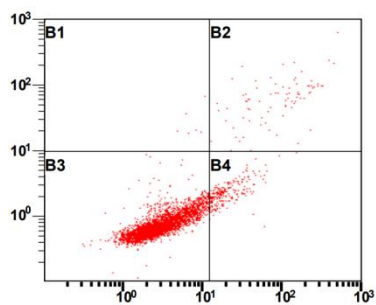

|    |      |       |       |
|----|------|-------|-------|
| B1 | 22   | 0.22  | 0.22  |
| B2 | 155  | 1.55  | 1.55  |
| B3 | 9332 | 93.32 | 93.32 |
| B4 | 491  | 4.91  | 4.91  |

Supplement: S1 File — (PDF) [file pone.0337223.s002.pdf]

**Fig 2B**

**PTPRCAP 32kDa**

**group: A549 H1299 BEAS-2B**

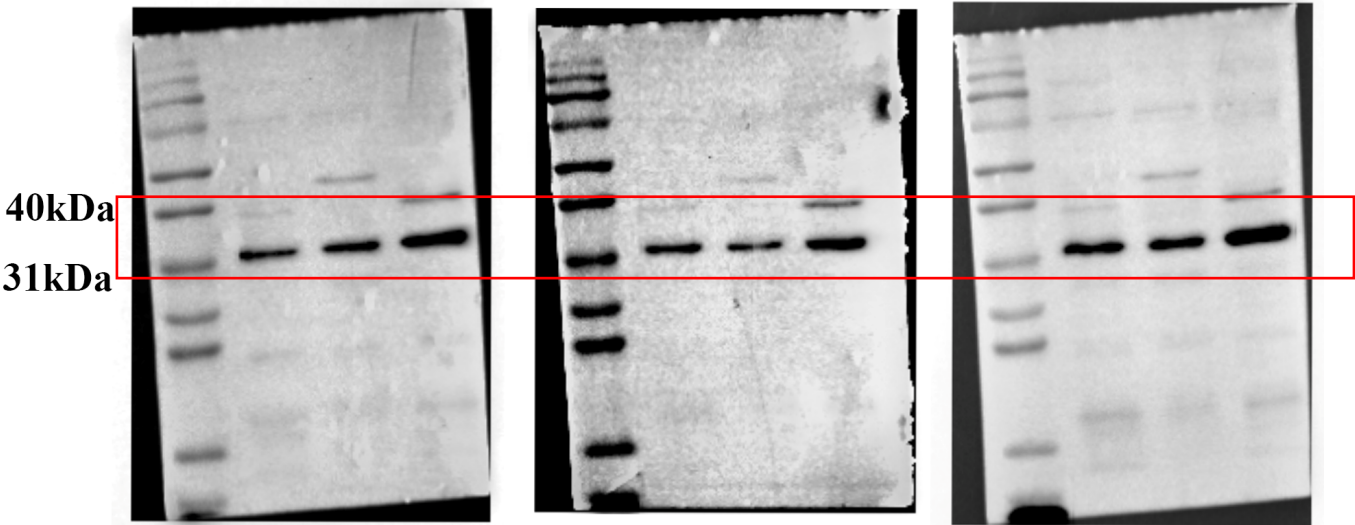

**GAPDH 36kDa**

**group: A549 H1299 BEAS-2B**

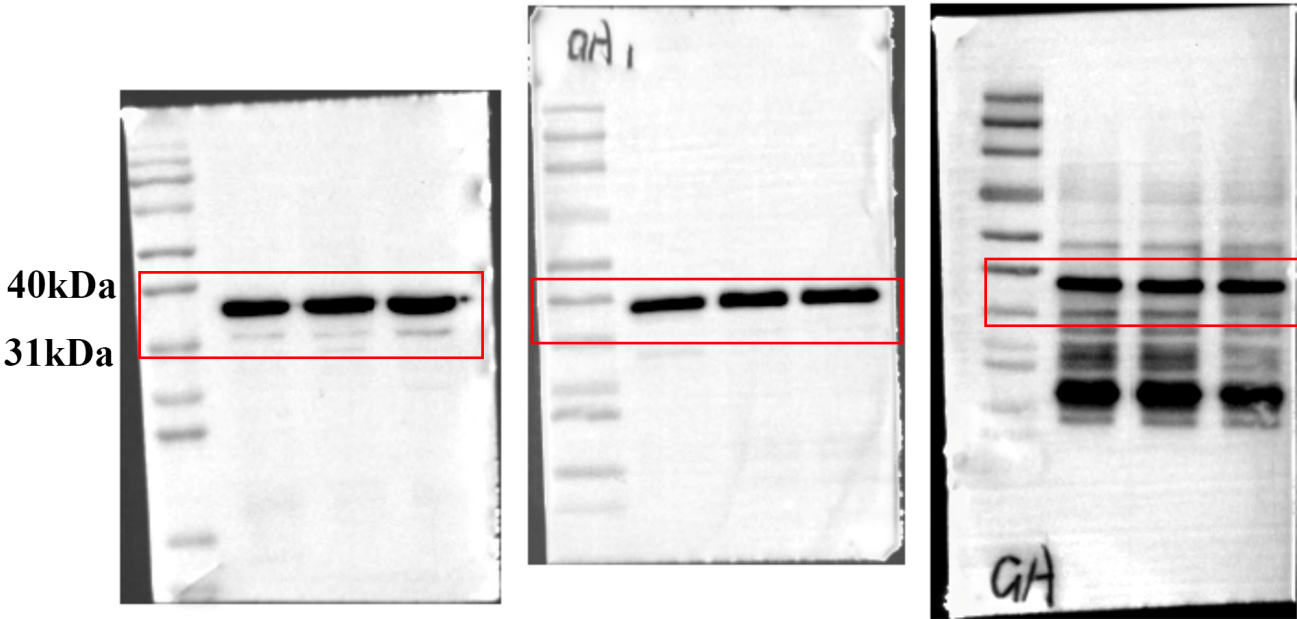

**Fig 8A**

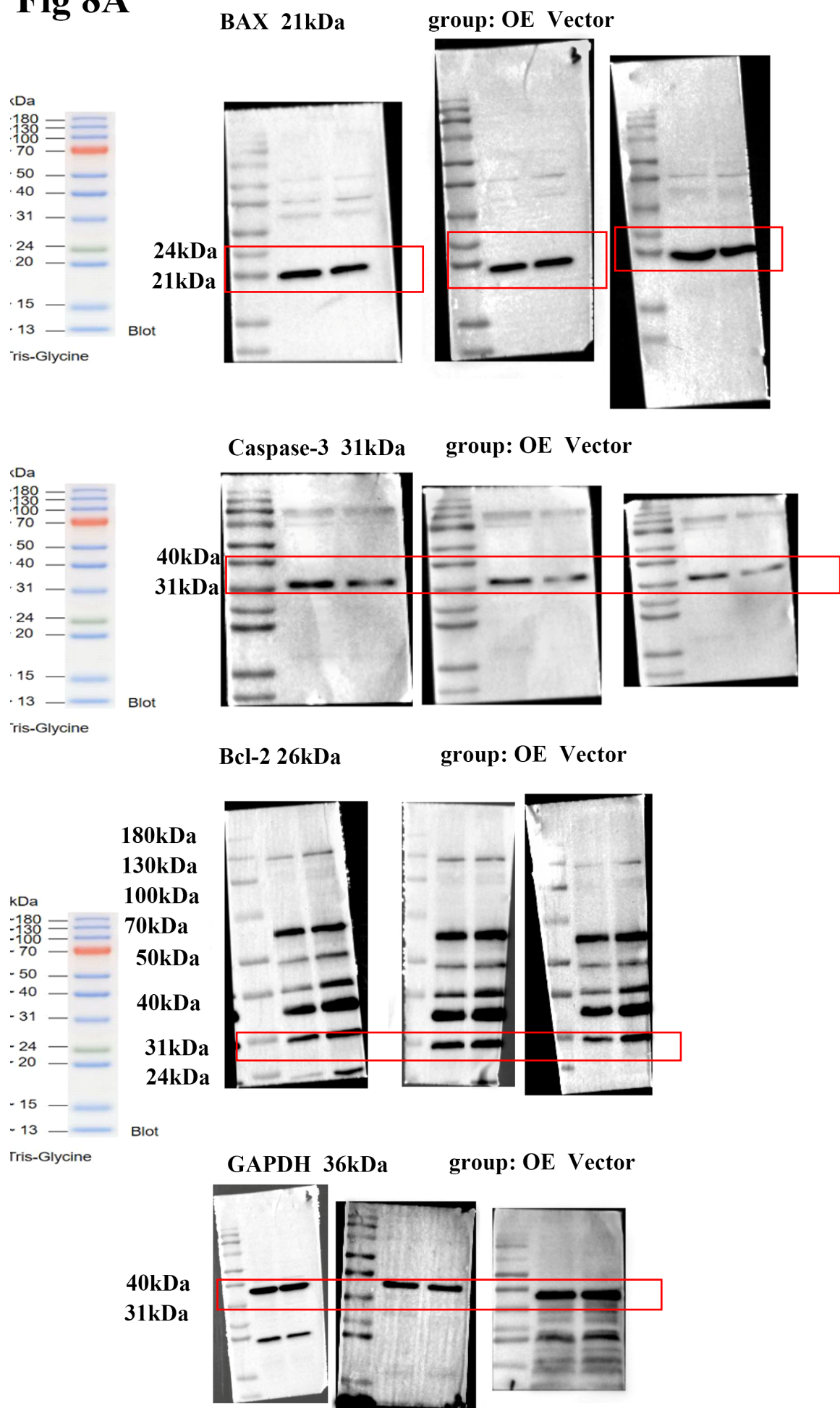

**Fig 8B**

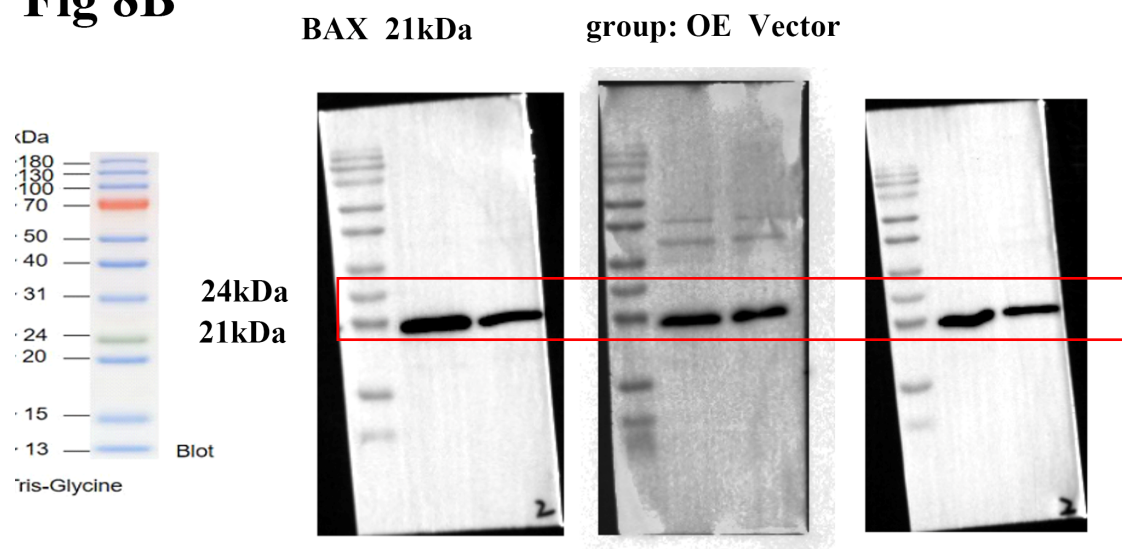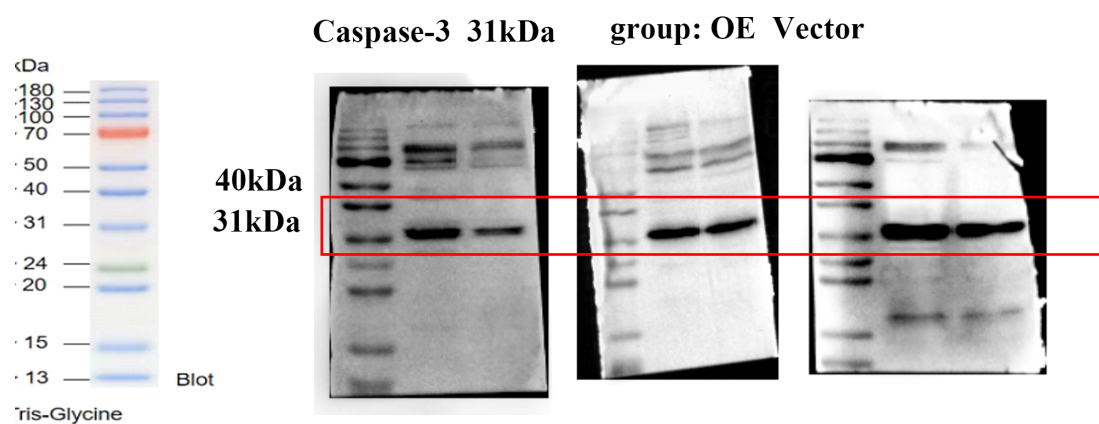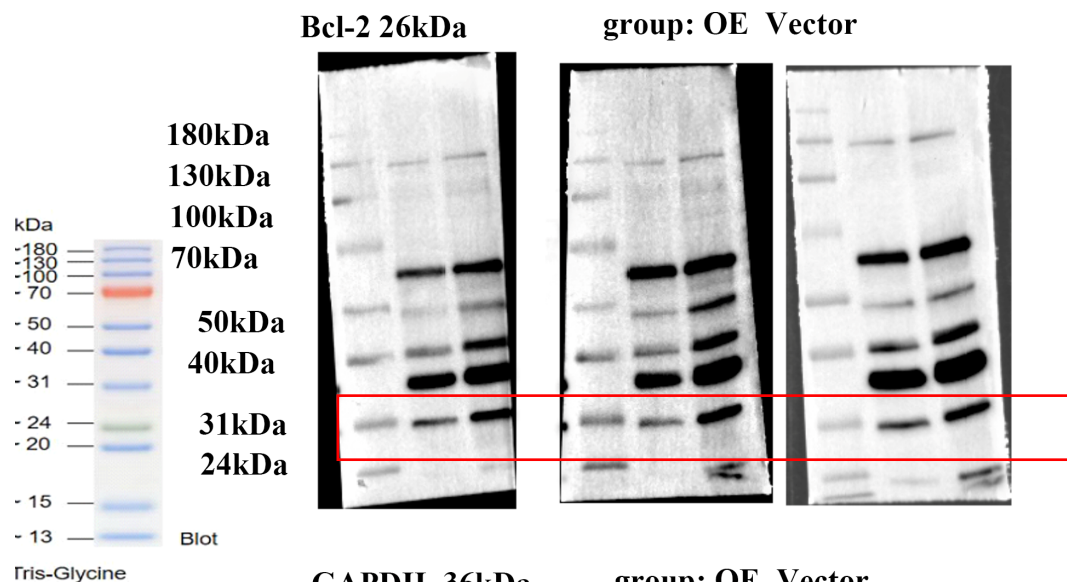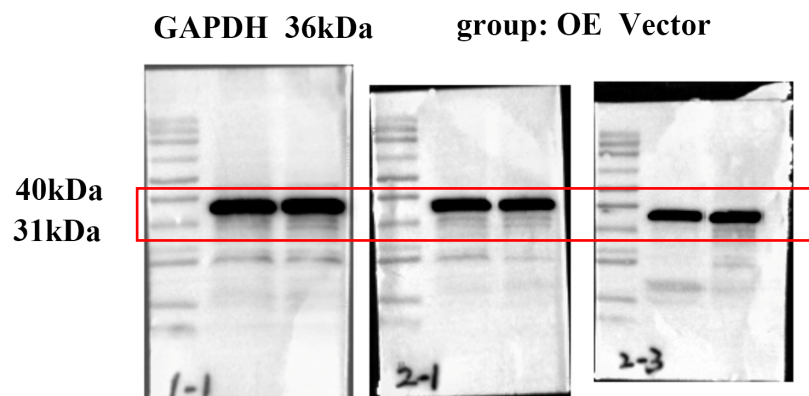

Supplement: S2 File — (PDF) [file pone.0337223.s003.pdf]

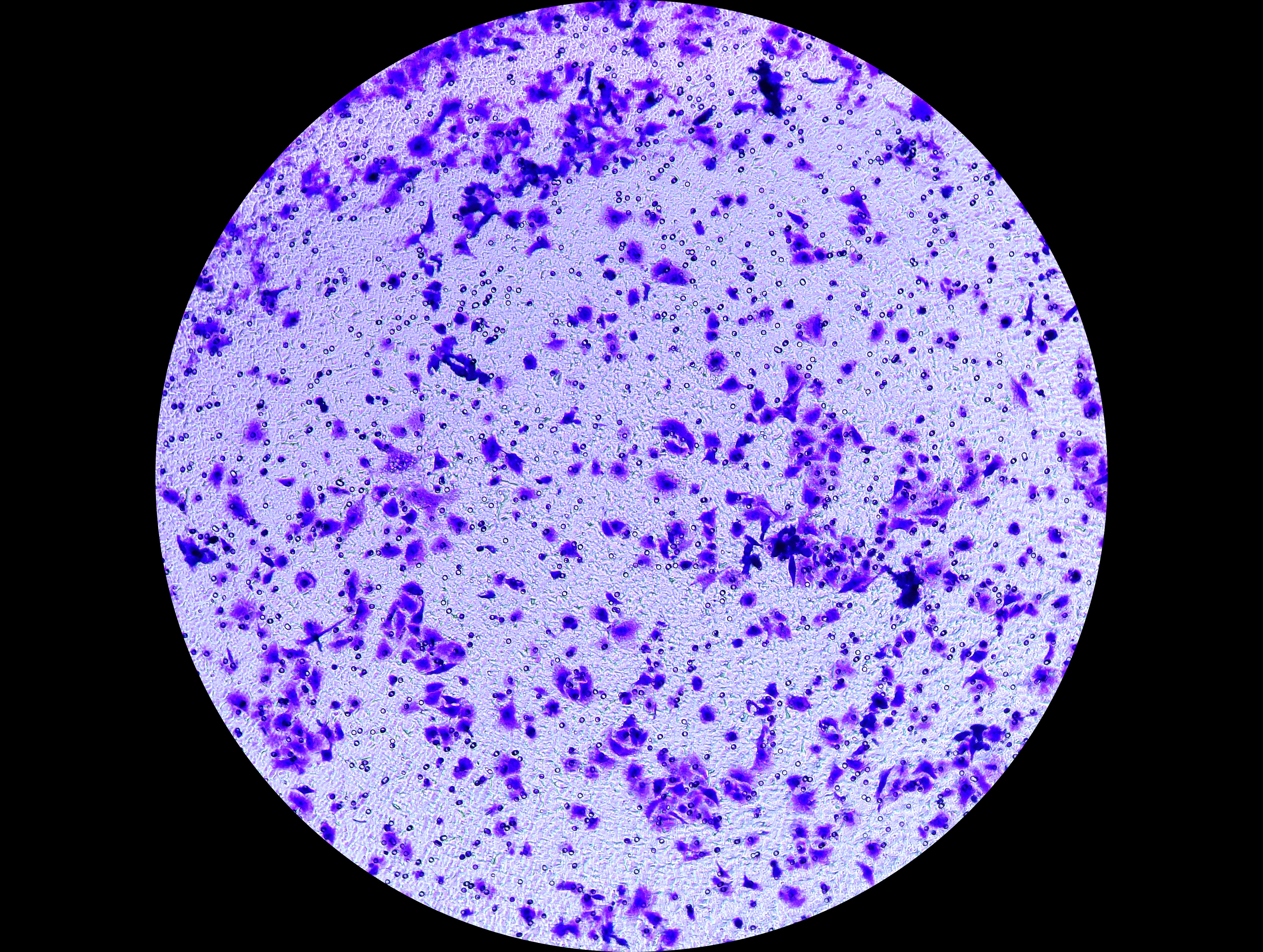

Supplement: S6 File — (ZIP) [file pone.0337223.s007.zip › OE-A549-Transwell invasion original image/A549-侵袭 -OV (1).JPG]

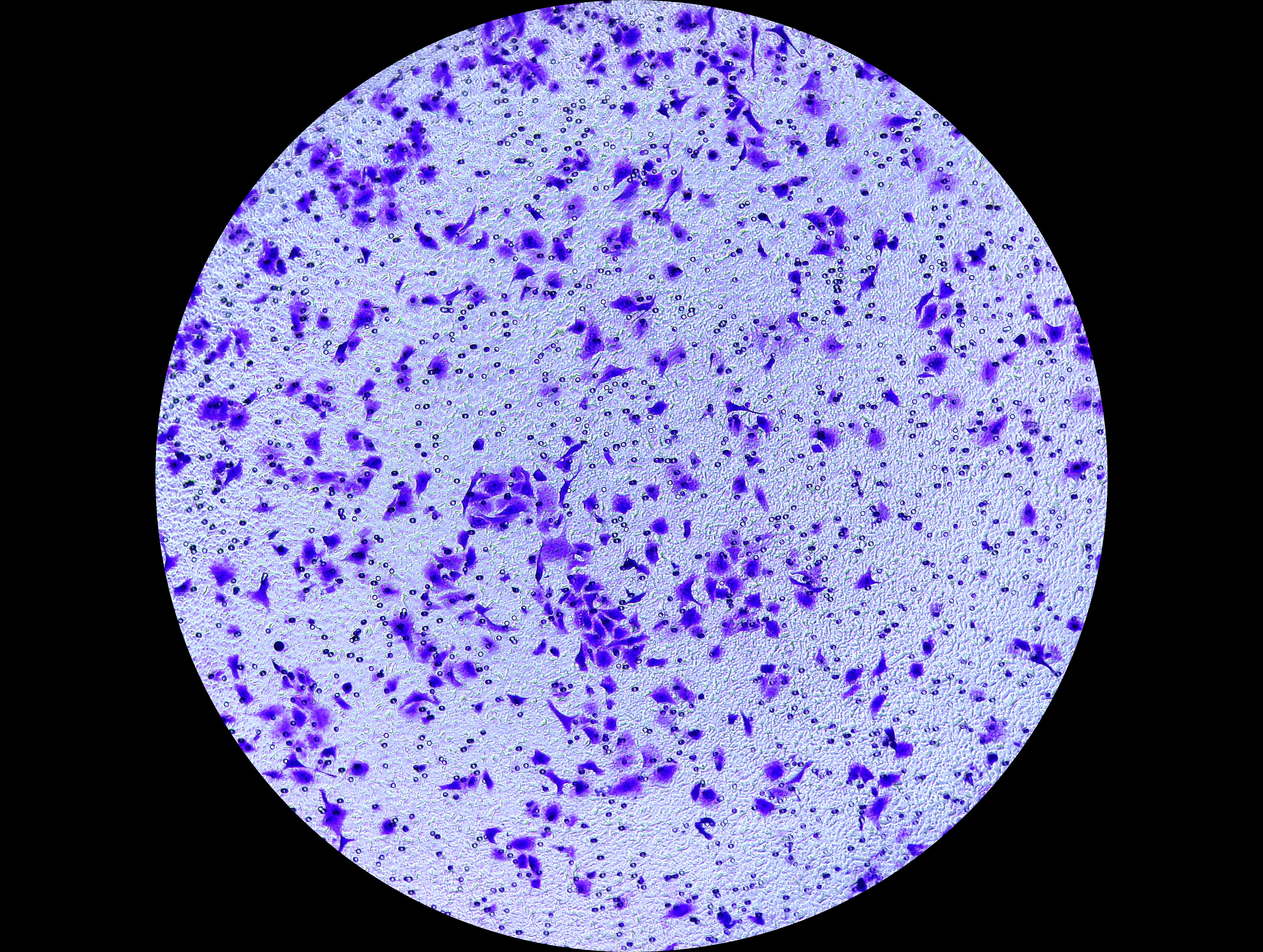

Supplement: S6 File — (ZIP) [file pone.0337223.s007.zip › OE-A549-Transwell invasion original image/A549-侵袭 -OV (2).JPG]

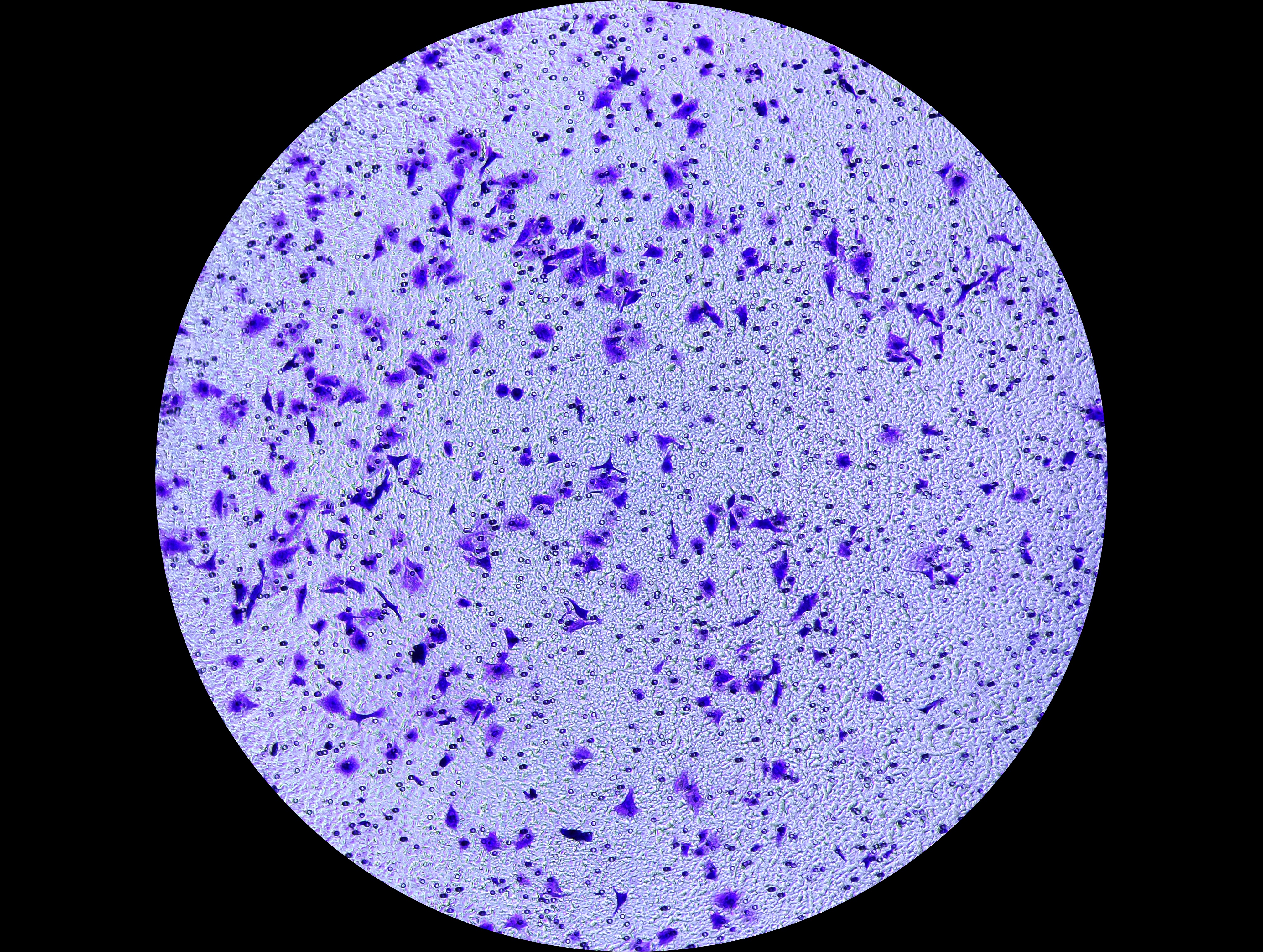

Supplement: S6 File — (ZIP) [file pone.0337223.s007.zip › OE-A549-Transwell invasion original image/A549-侵袭 -OV (3).JPG]

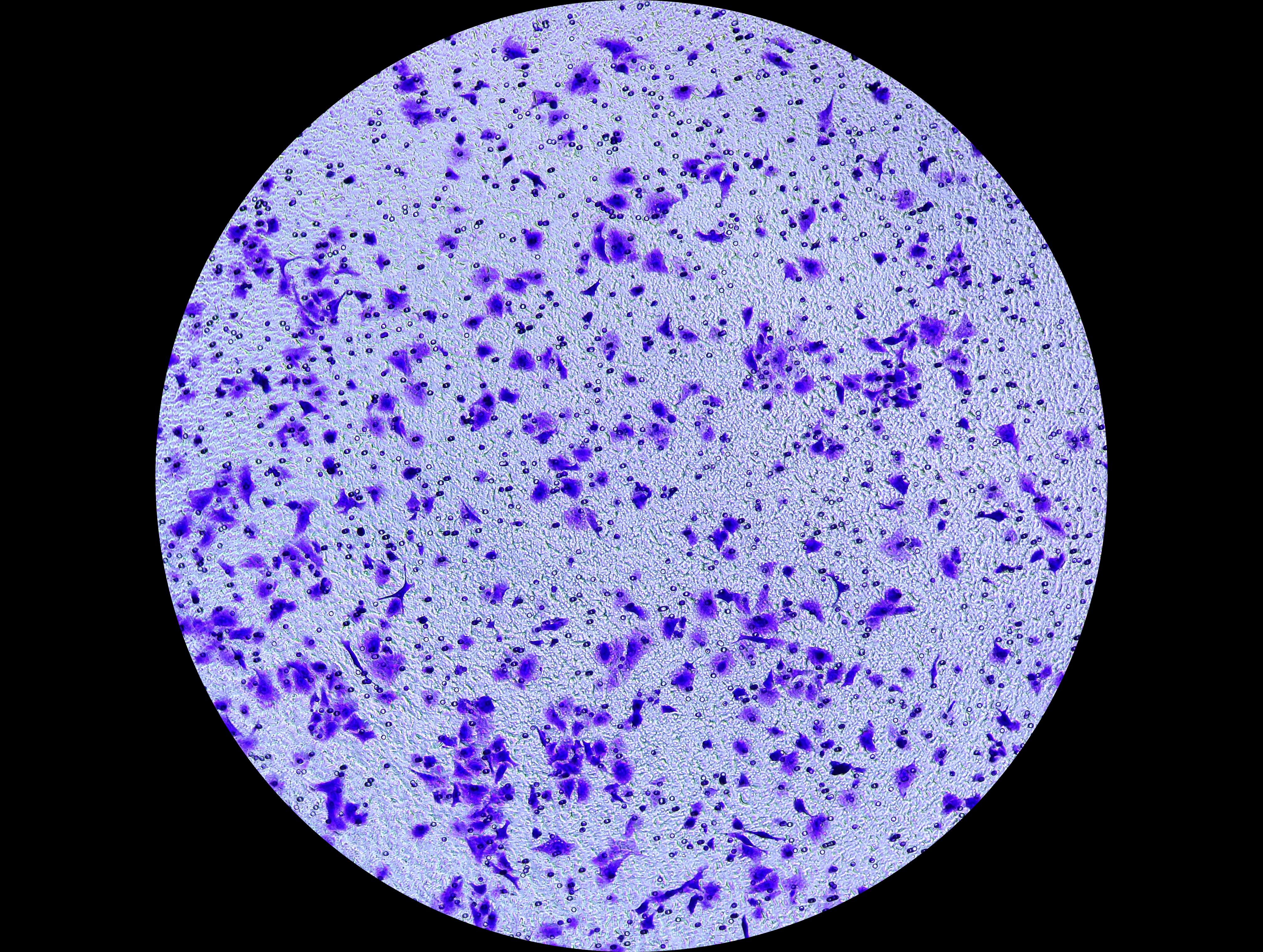

Supplement: S6 File — (ZIP) [file pone.0337223.s007.zip › OE-A549-Transwell invasion original image/A549-侵袭 -OV (4).JPG]

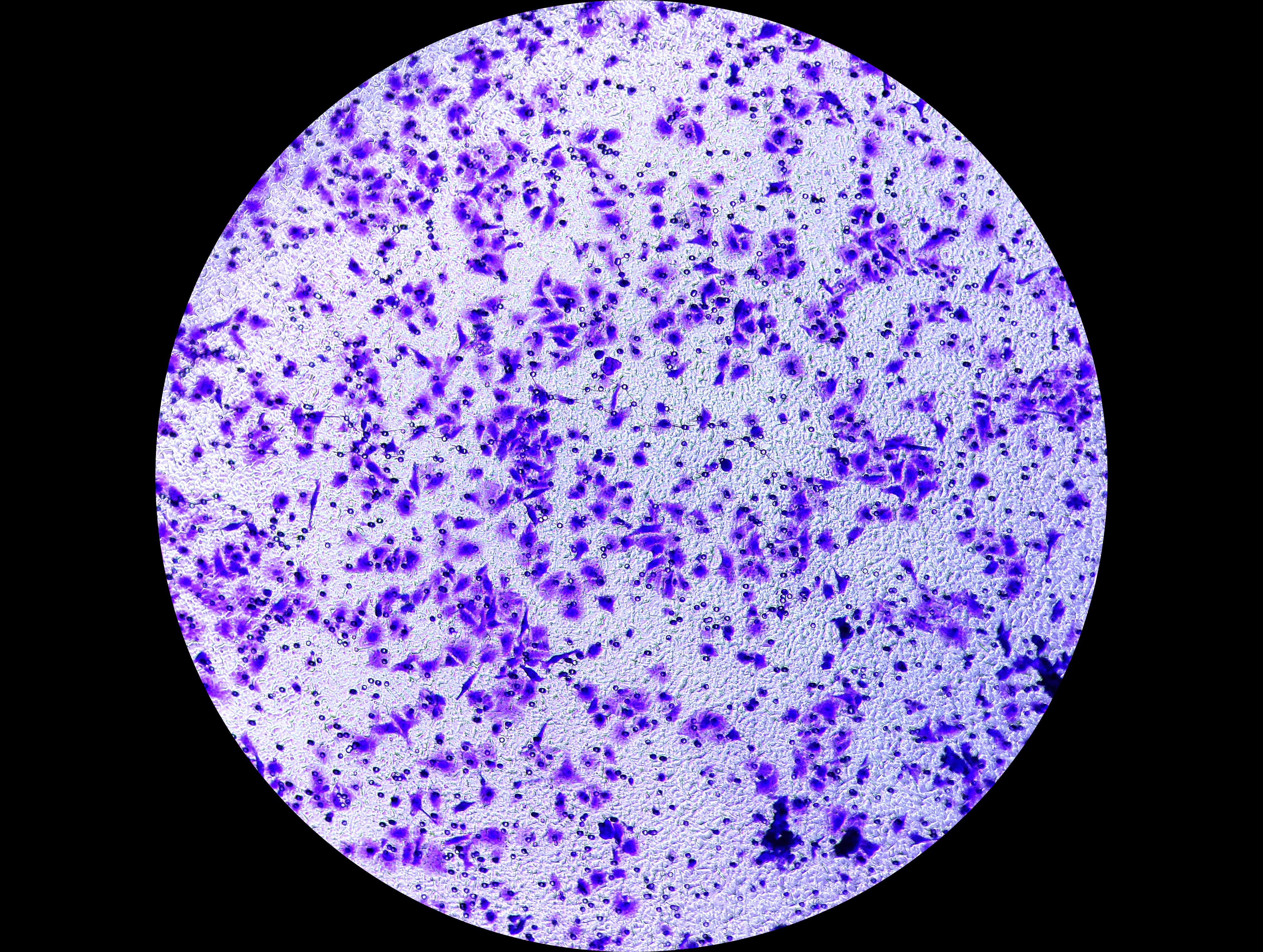

Supplement: S6 File — (ZIP) [file pone.0337223.s007.zip › OE-A549-Transwell invasion original image/A549-侵袭-OV -NC(1).JPG]

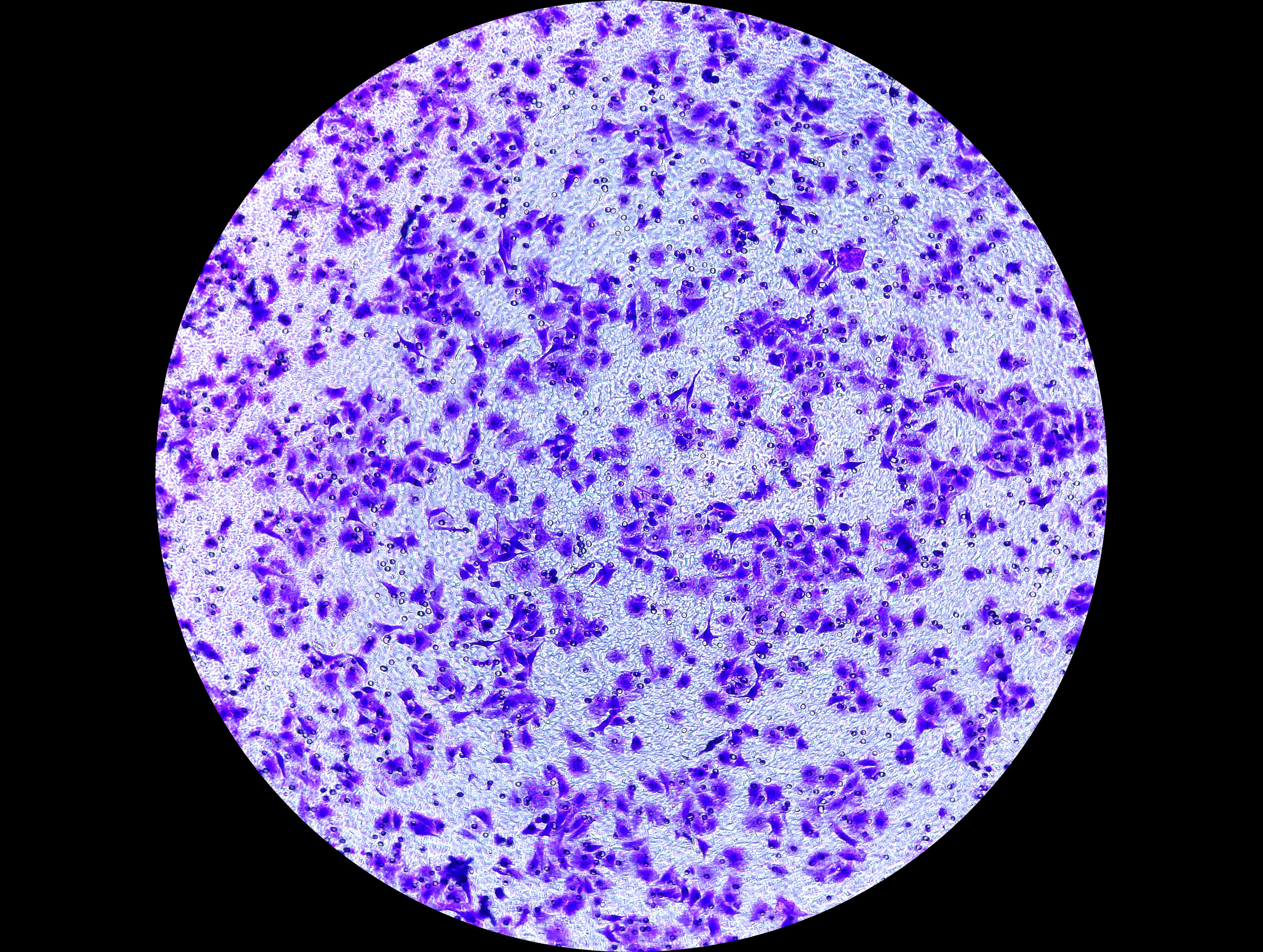

Supplement: S6 File — (ZIP) [file pone.0337223.s007.zip › OE-A549-Transwell invasion original image/A549-侵袭-OV -NC(2).JPG]

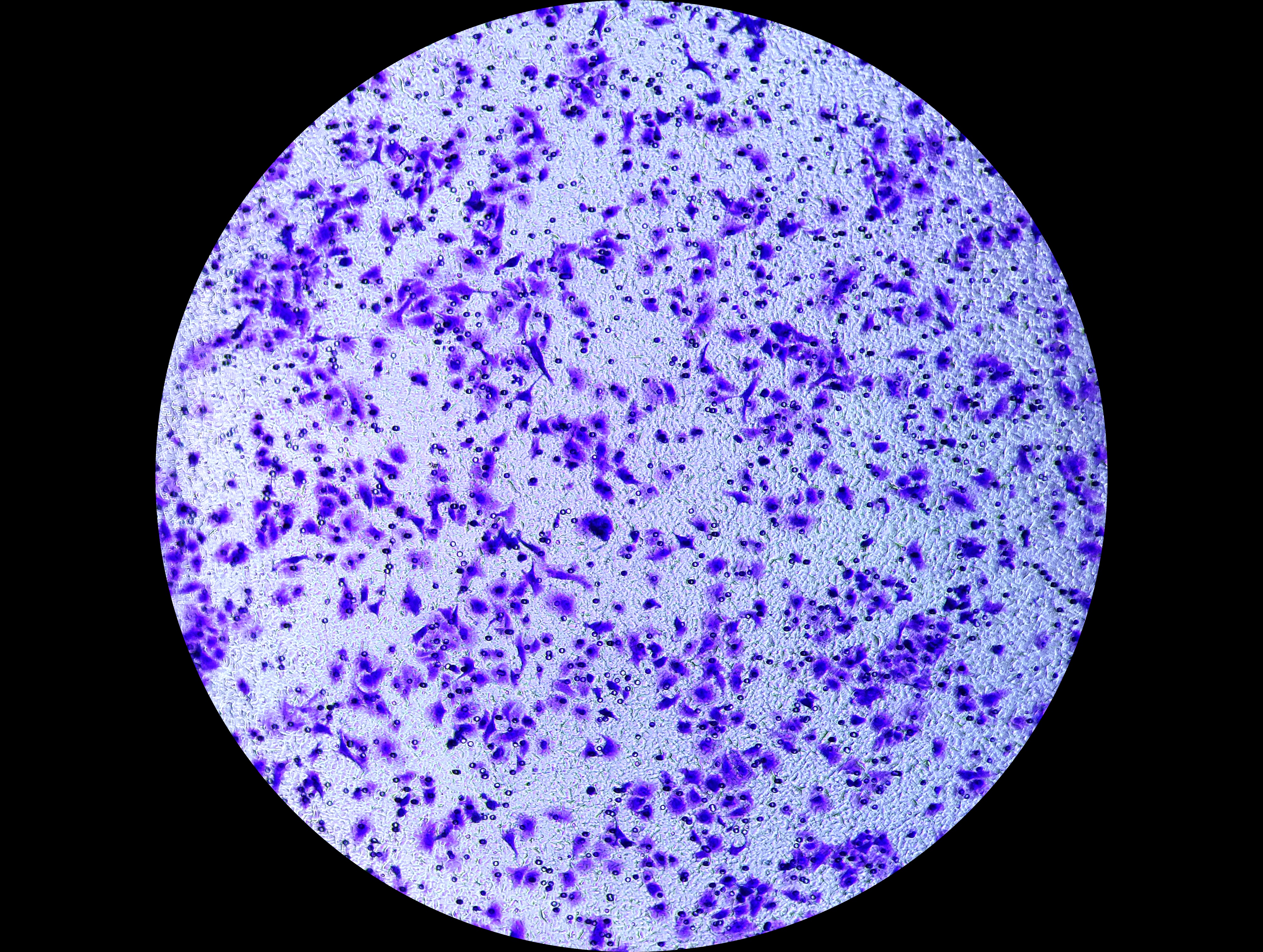

Supplement: S6 File — (ZIP) [file pone.0337223.s007.zip › OE-A549-Transwell invasion original image/A549-侵袭-OV -NC(3).JPG]

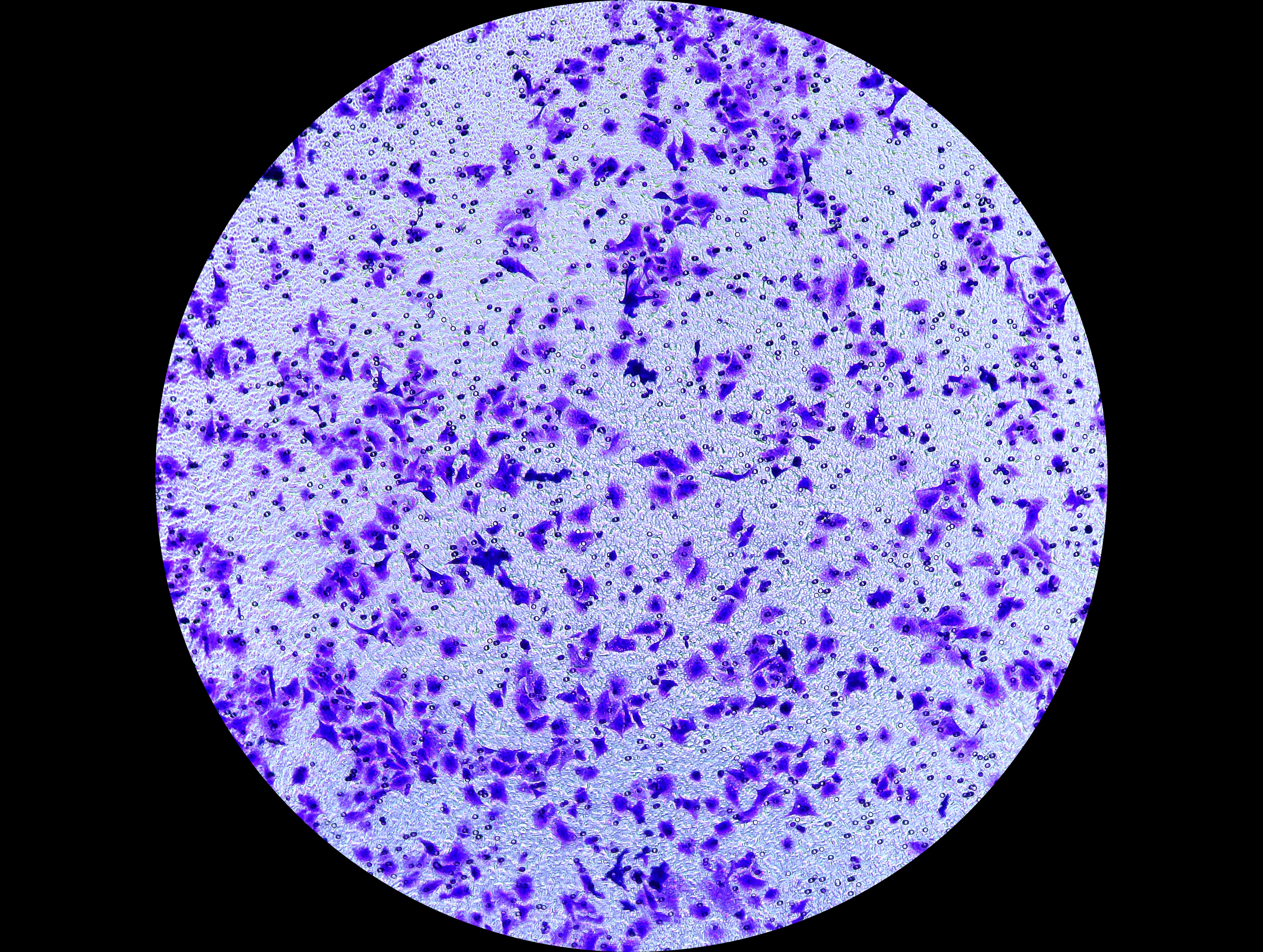

Supplement: S6 File — (ZIP) [file pone.0337223.s007.zip › OE-A549-Transwell invasion original image/A549-侵袭-OV -NC(4).JPG]

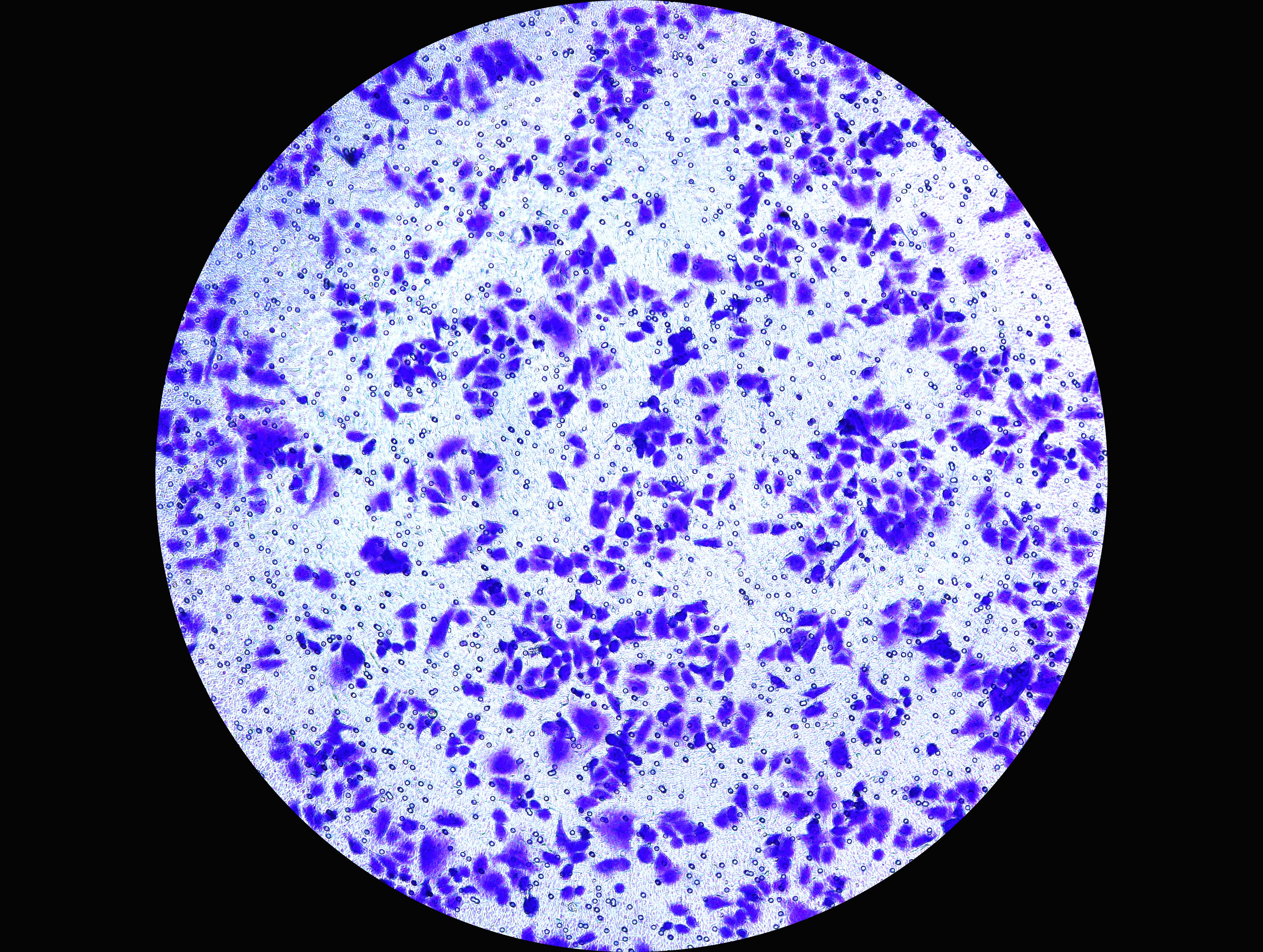

Supplement: S7 File — (ZIP) [file pone.0337223.s008.zip › OE-A549-Transwell migration original image/A549-迁移-OV (1).JPG]

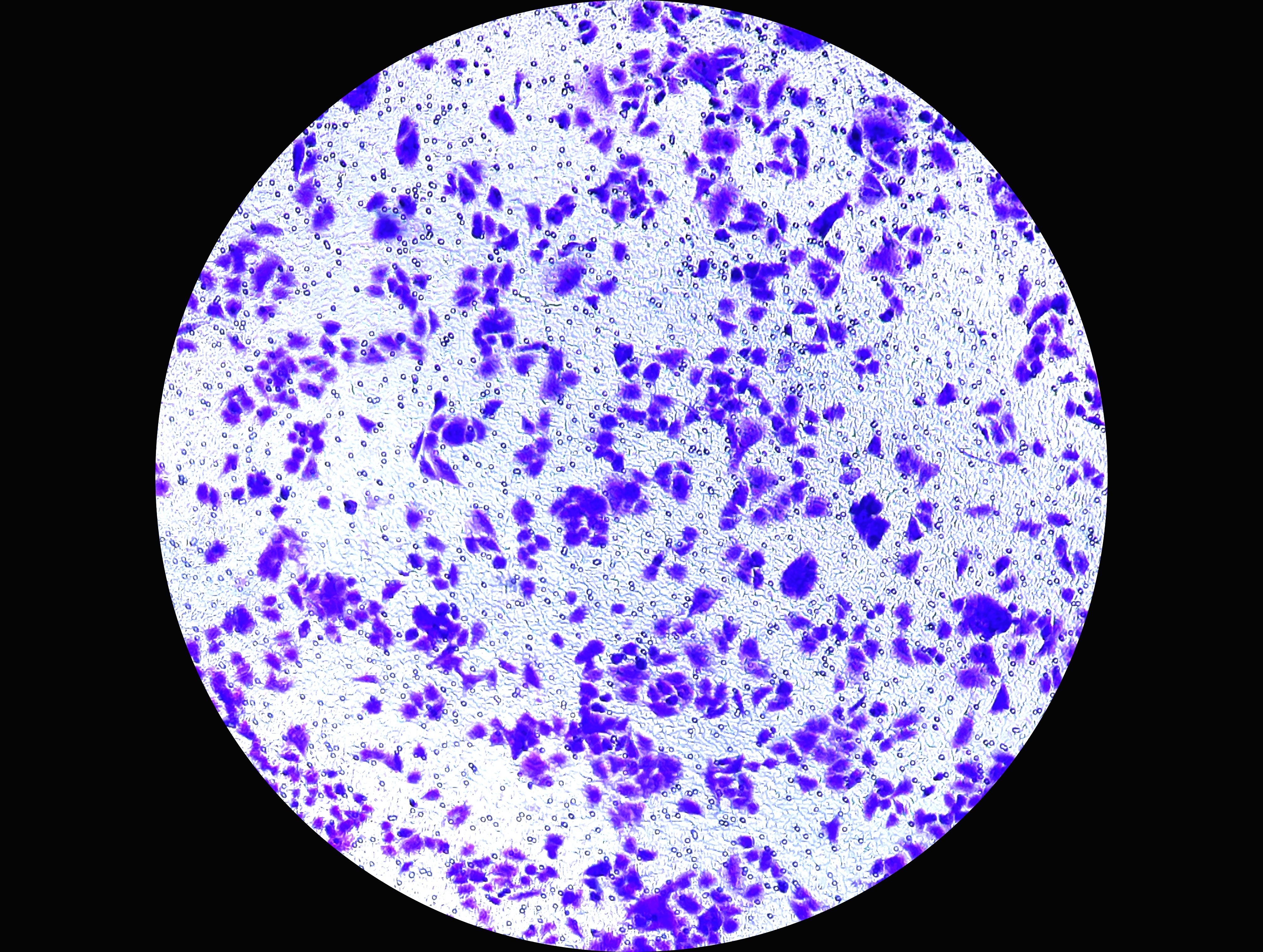

Supplement: S7 File — (ZIP) [file pone.0337223.s008.zip › OE-A549-Transwell migration original image/A549-迁移-OV (2).JPG]

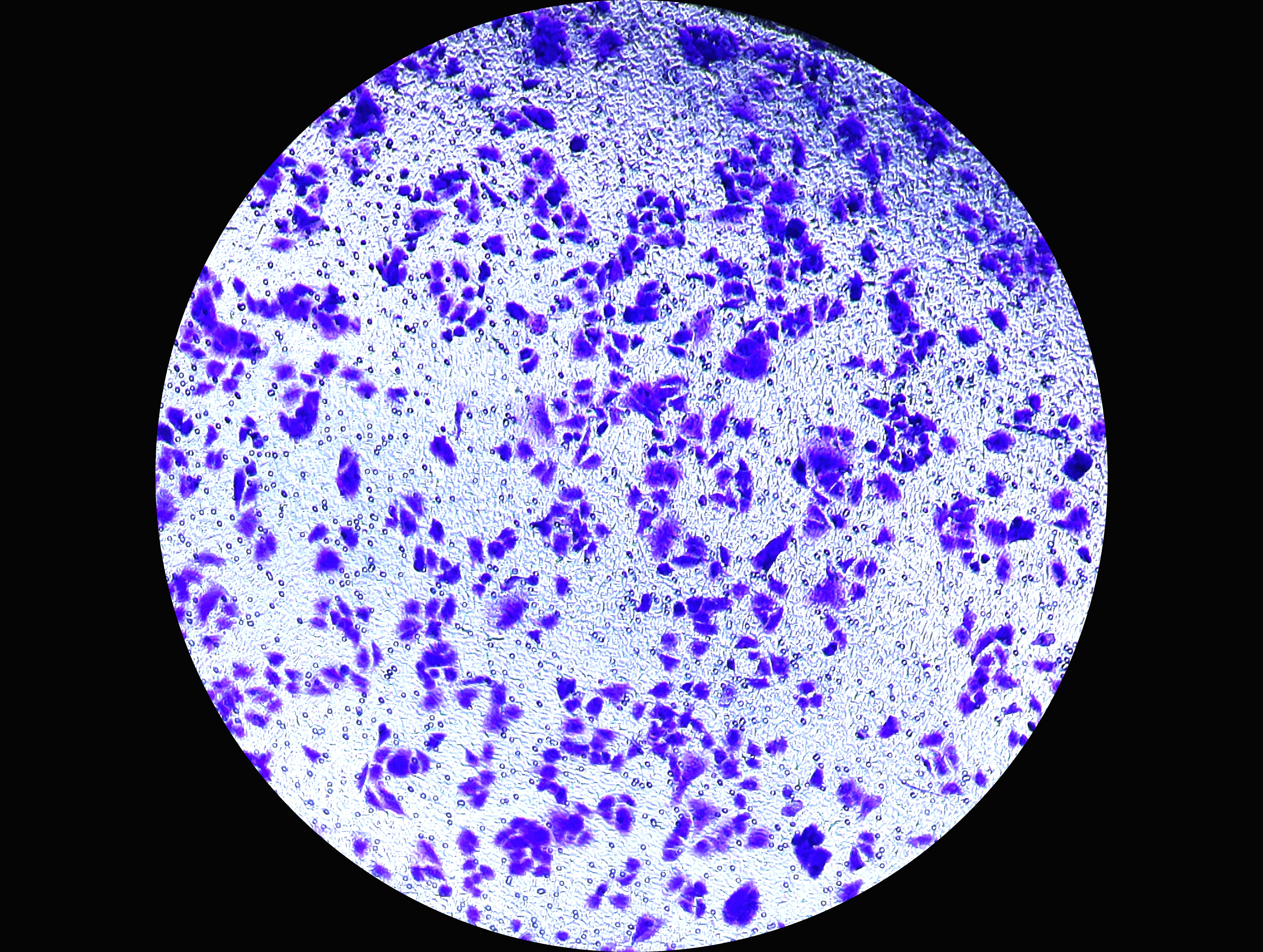

Supplement: S7 File — (ZIP) [file pone.0337223.s008.zip › OE-A549-Transwell migration original image/A549-迁移-OV (3).JPG]

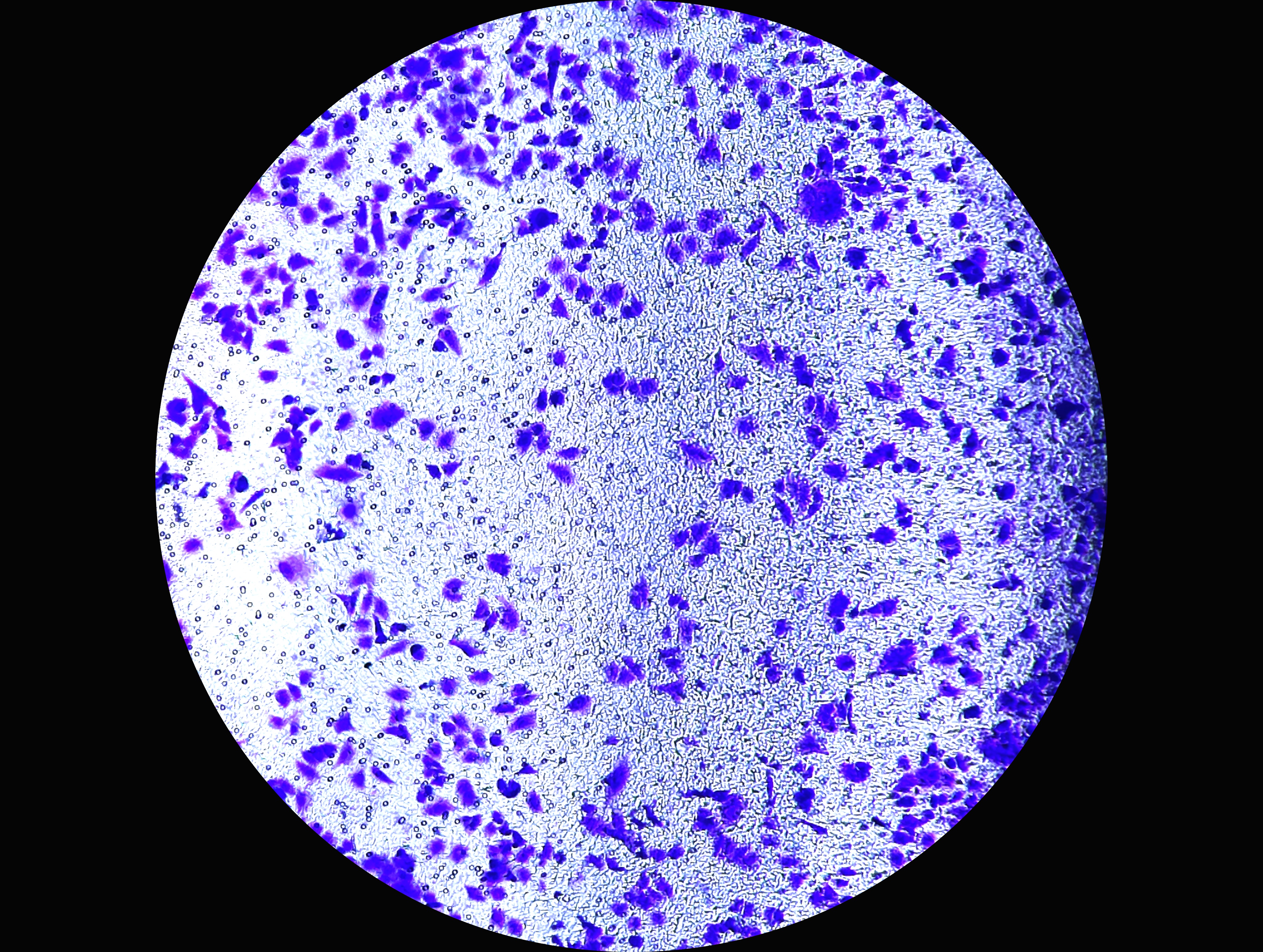

Supplement: S7 File — (ZIP) [file pone.0337223.s008.zip › OE-A549-Transwell migration original image/A549-迁移-OV (4).JPG]

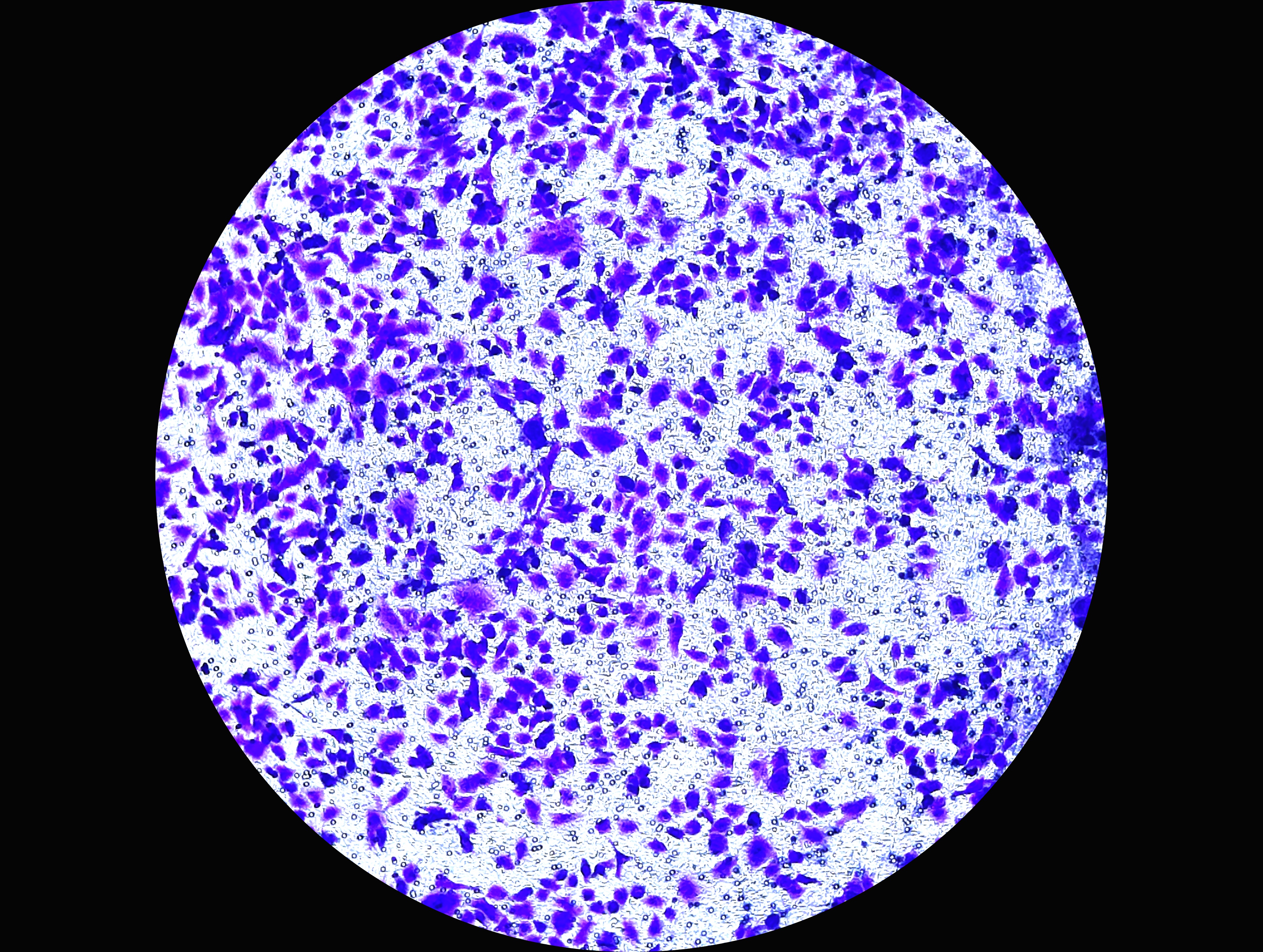

Supplement: S7 File — (ZIP) [file pone.0337223.s008.zip › OE-A549-Transwell migration original image/A549-迁移-OV-NC (1).JPG]

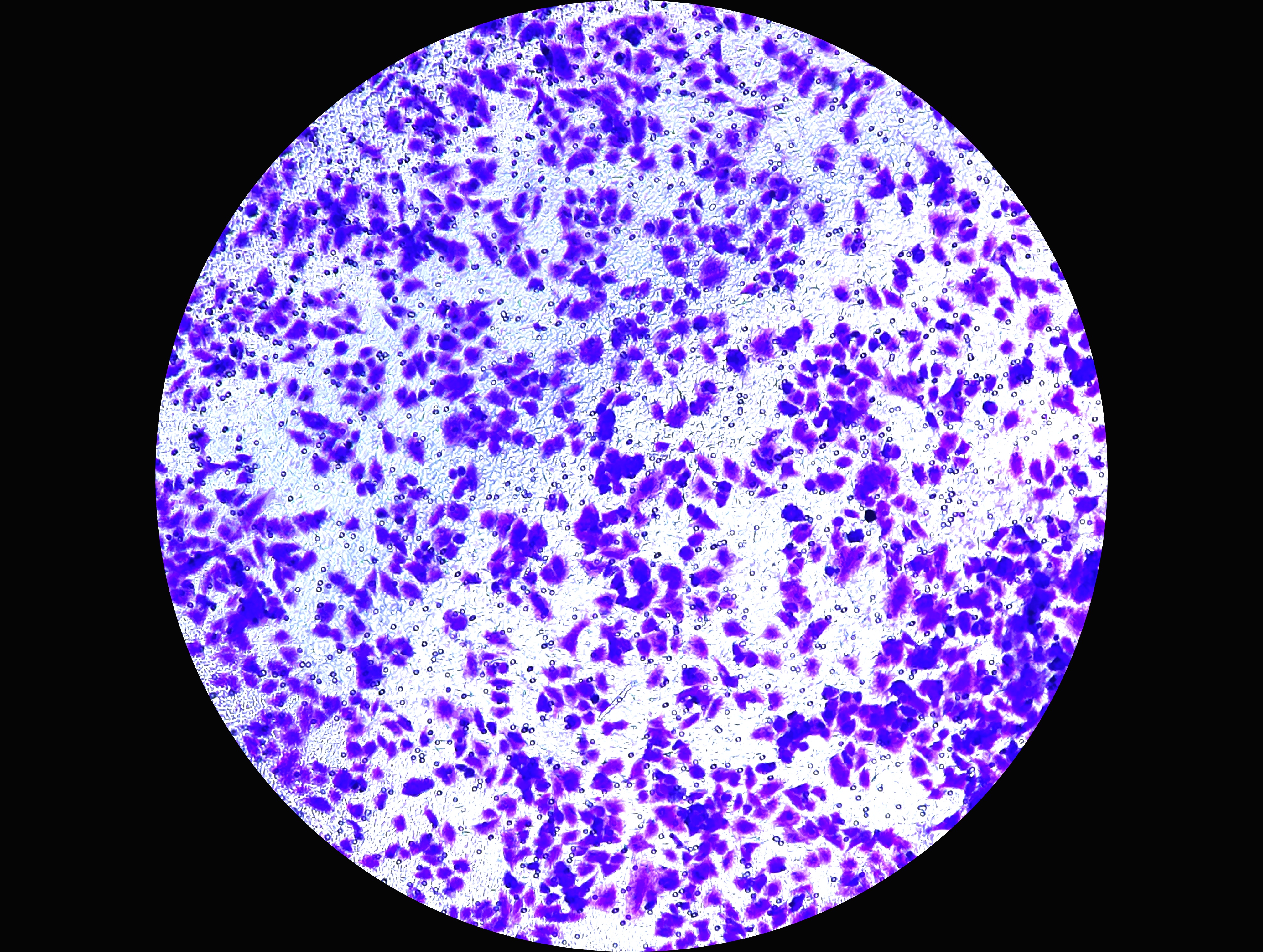

Supplement: S7 File — (ZIP) [file pone.0337223.s008.zip › OE-A549-Transwell migration original image/A549-迁移-OV-NC (2).JPG]

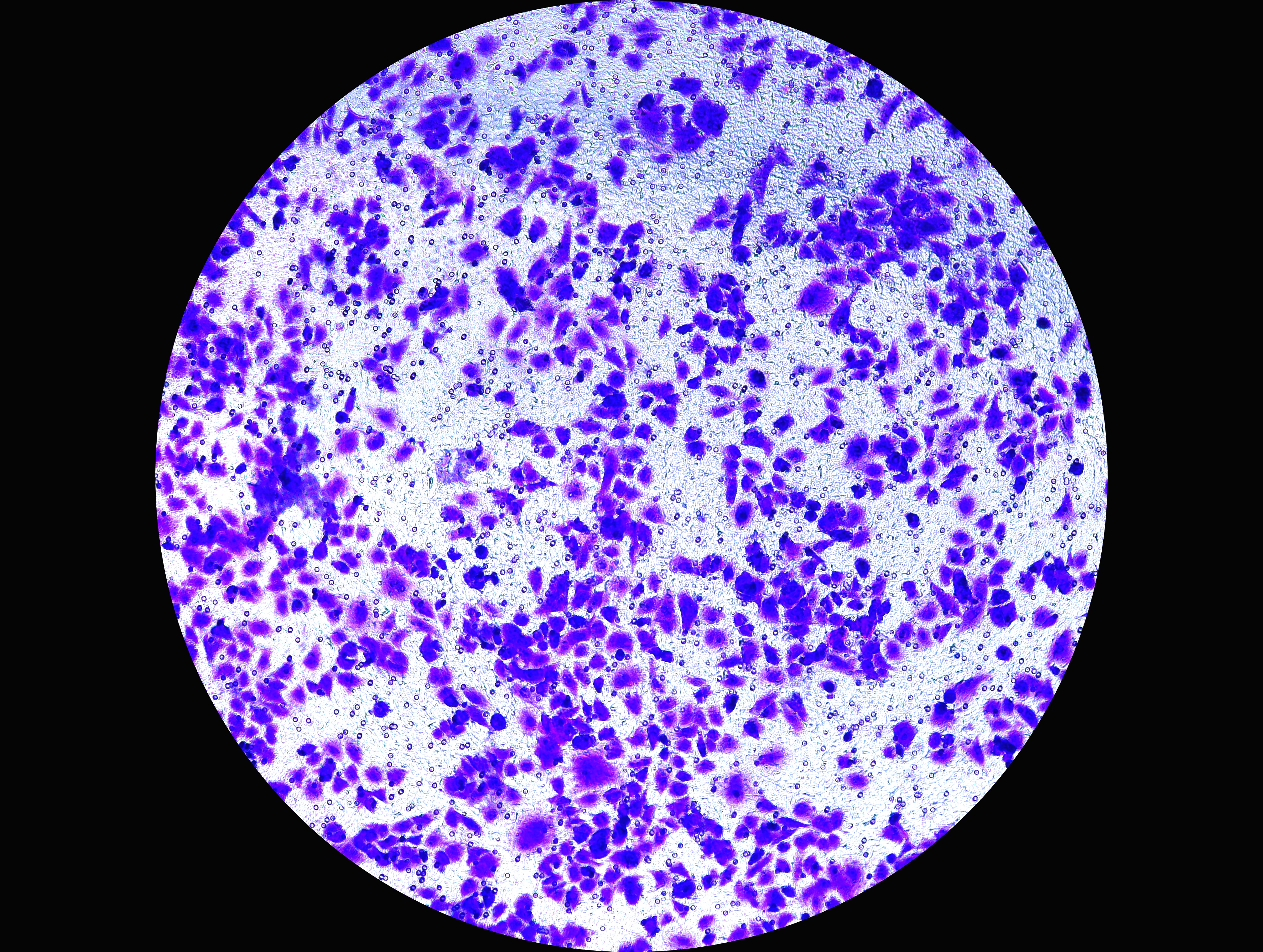

Supplement: S7 File — (ZIP) [file pone.0337223.s008.zip › OE-A549-Transwell migration original image/A549-迁移-OV-NC (3).JPG]

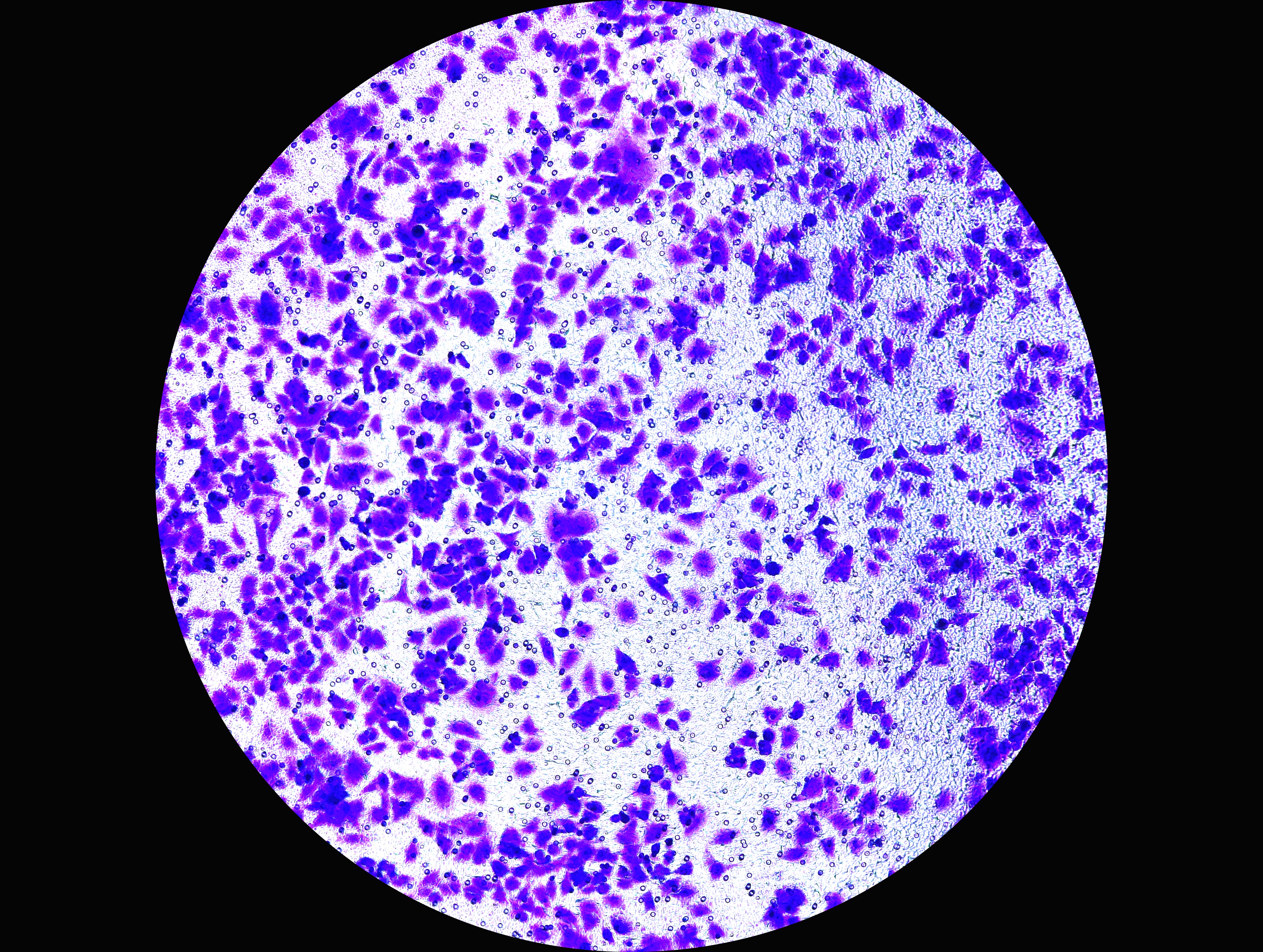

Supplement: S7 File — (ZIP) [file pone.0337223.s008.zip › OE-A549-Transwell migration original image/A549-迁移-OV-NC (4).JPG]

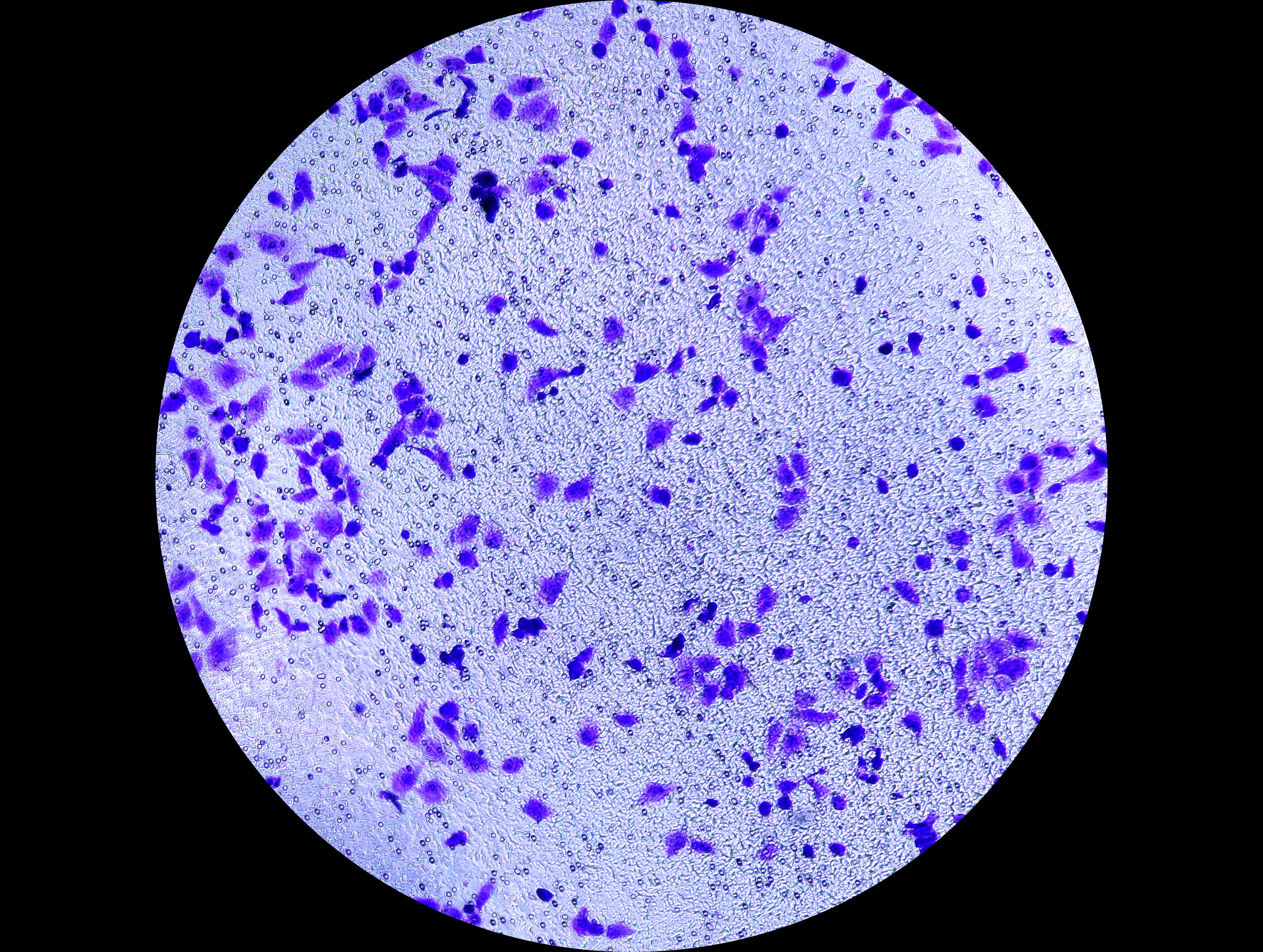

Supplement: S8 File — (ZIP) [file pone.0337223.s009.zip › OE-H1299-Transwell invasion original image/H1299-侵袭-OV (1).JPG]

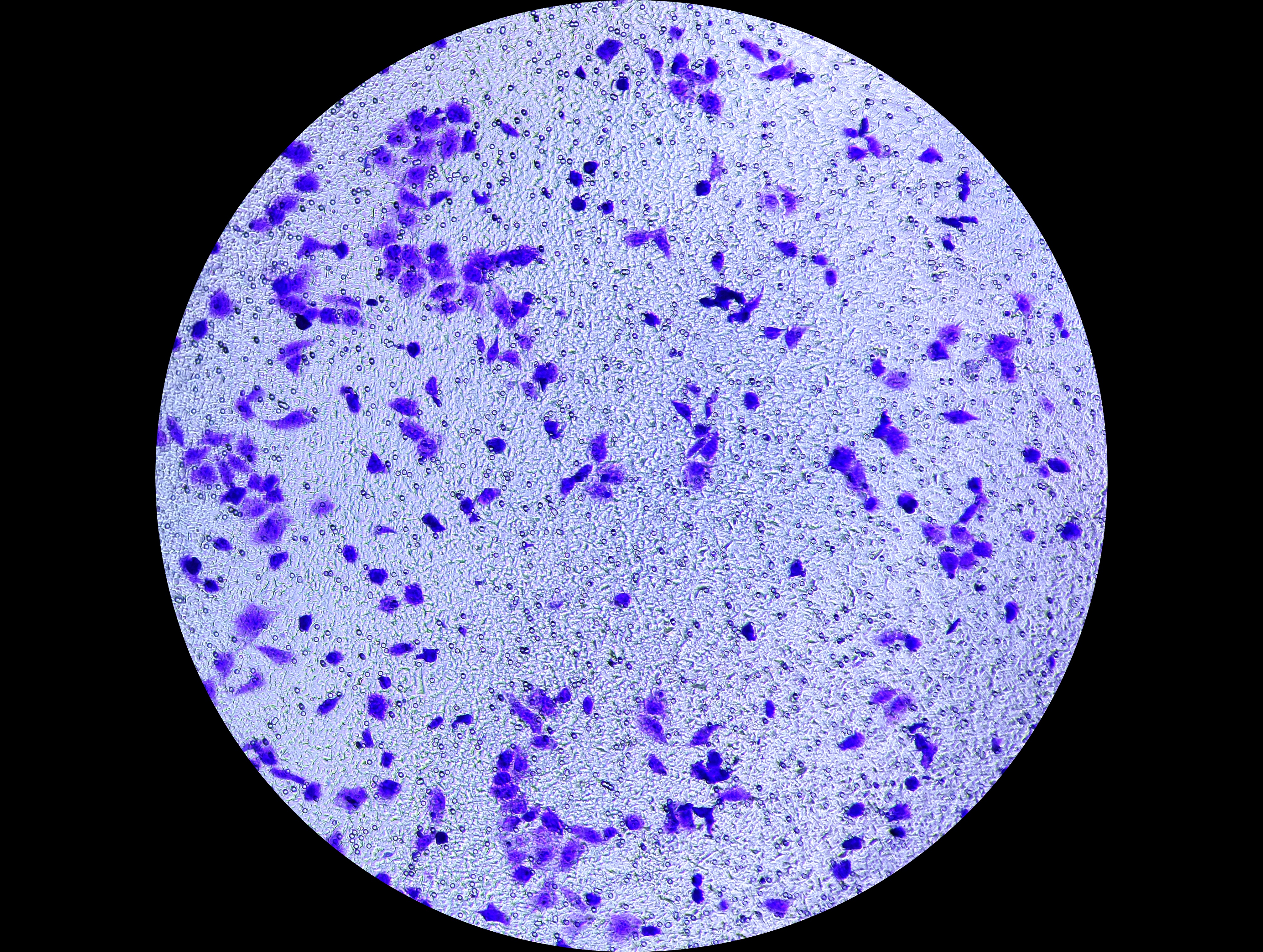

Supplement: S8 File — (ZIP) [file pone.0337223.s009.zip › OE-H1299-Transwell invasion original image/H1299-侵袭-OV (2).JPG]

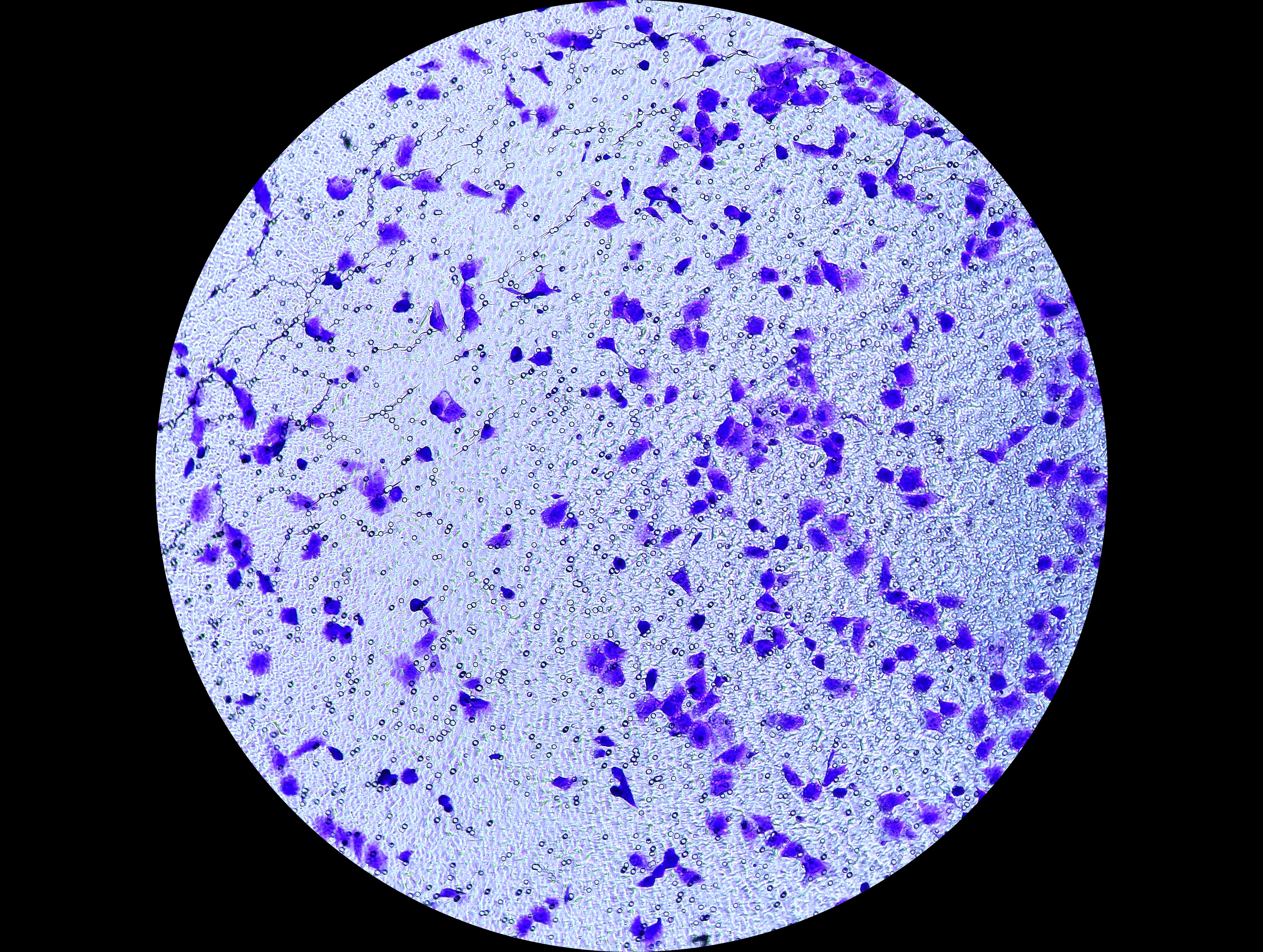

Supplement: S8 File — (ZIP) [file pone.0337223.s009.zip › OE-H1299-Transwell invasion original image/H1299-侵袭-OV (3).JPG]

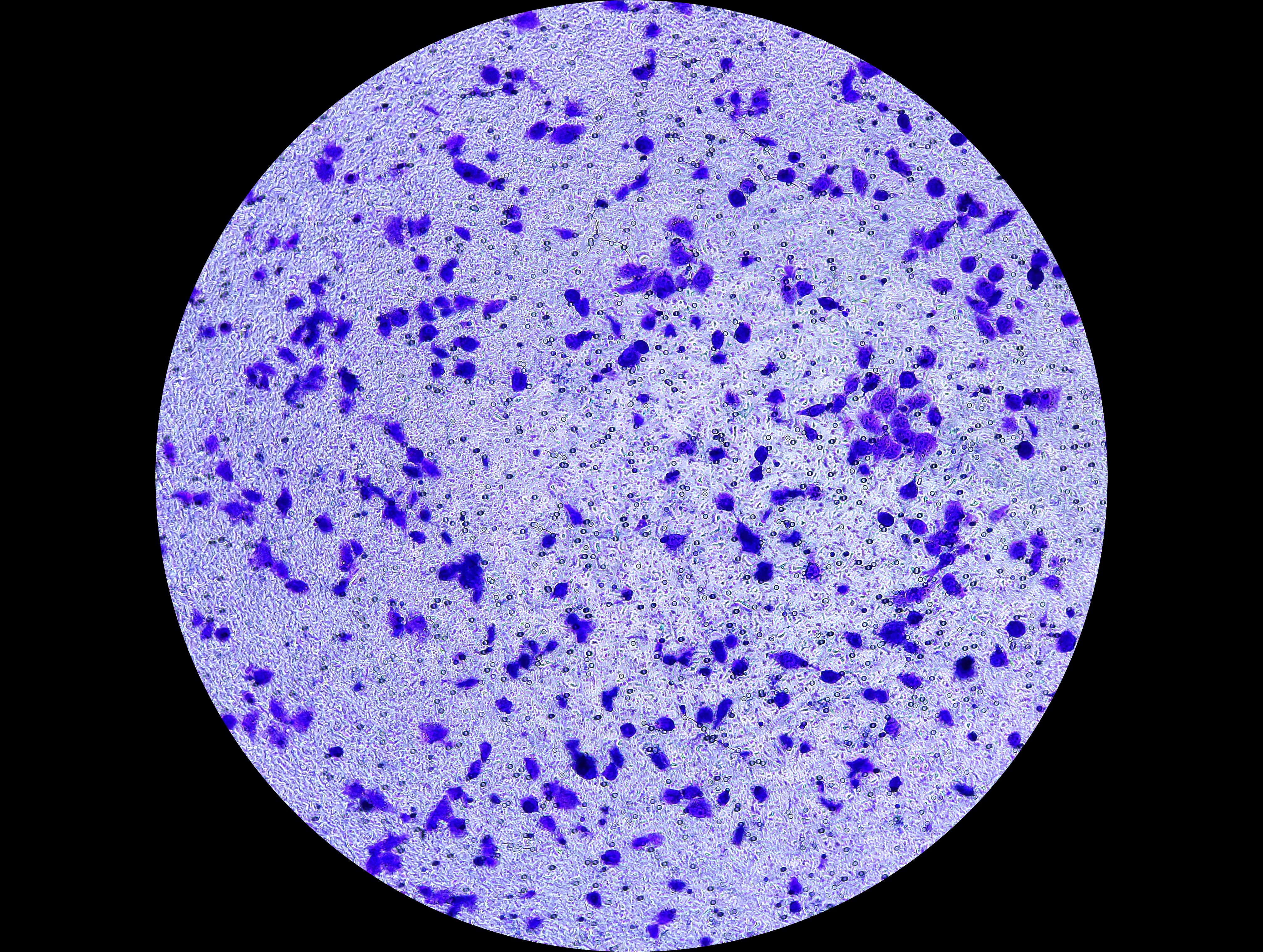

Supplement: S8 File — (ZIP) [file pone.0337223.s009.zip › OE-H1299-Transwell invasion original image/H1299-侵袭-OV (4).JPG]

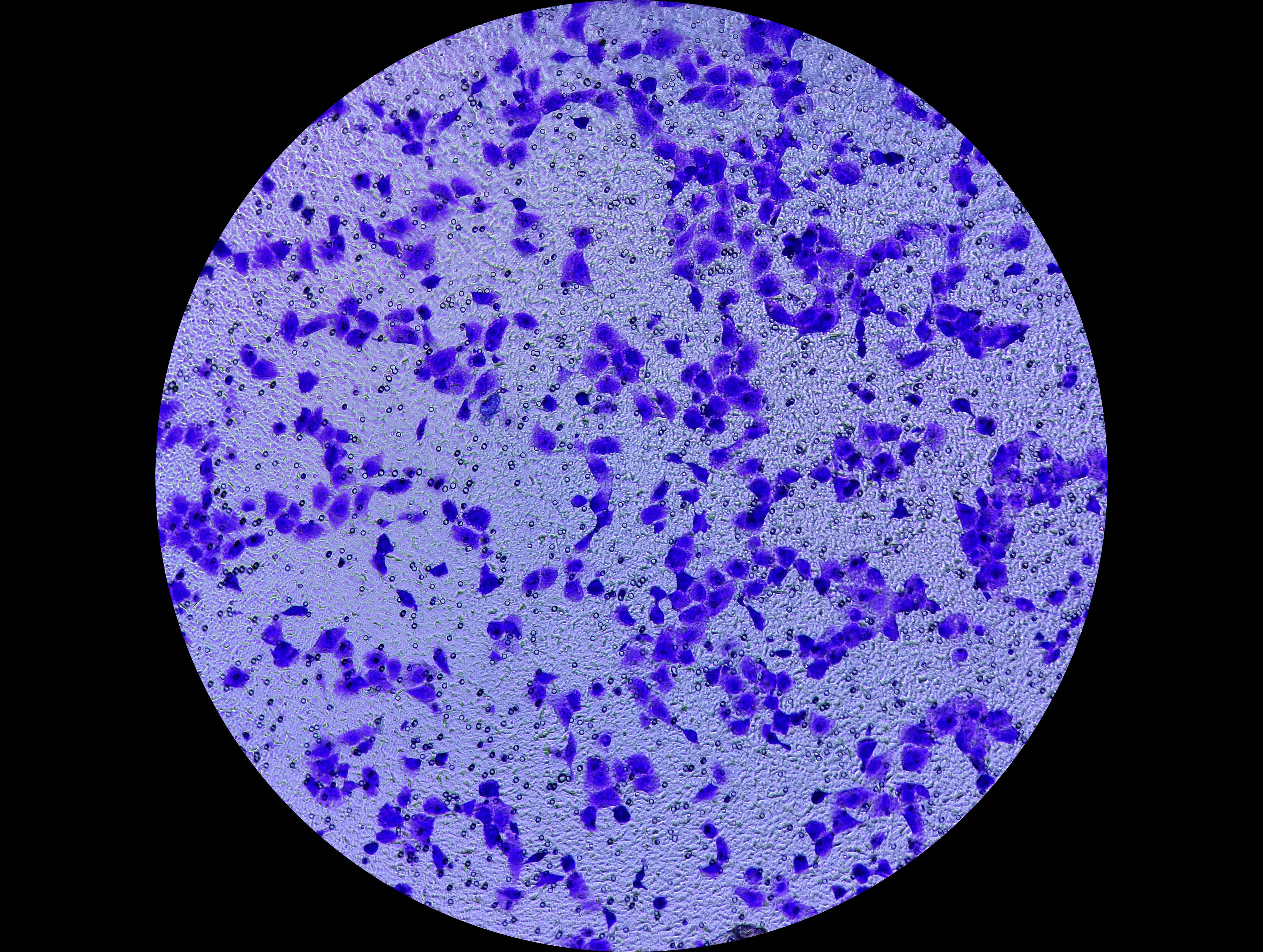

Supplement: S8 File — (ZIP) [file pone.0337223.s009.zip › OE-H1299-Transwell invasion original image/H1299-侵袭-OV-NC (1).JPG]

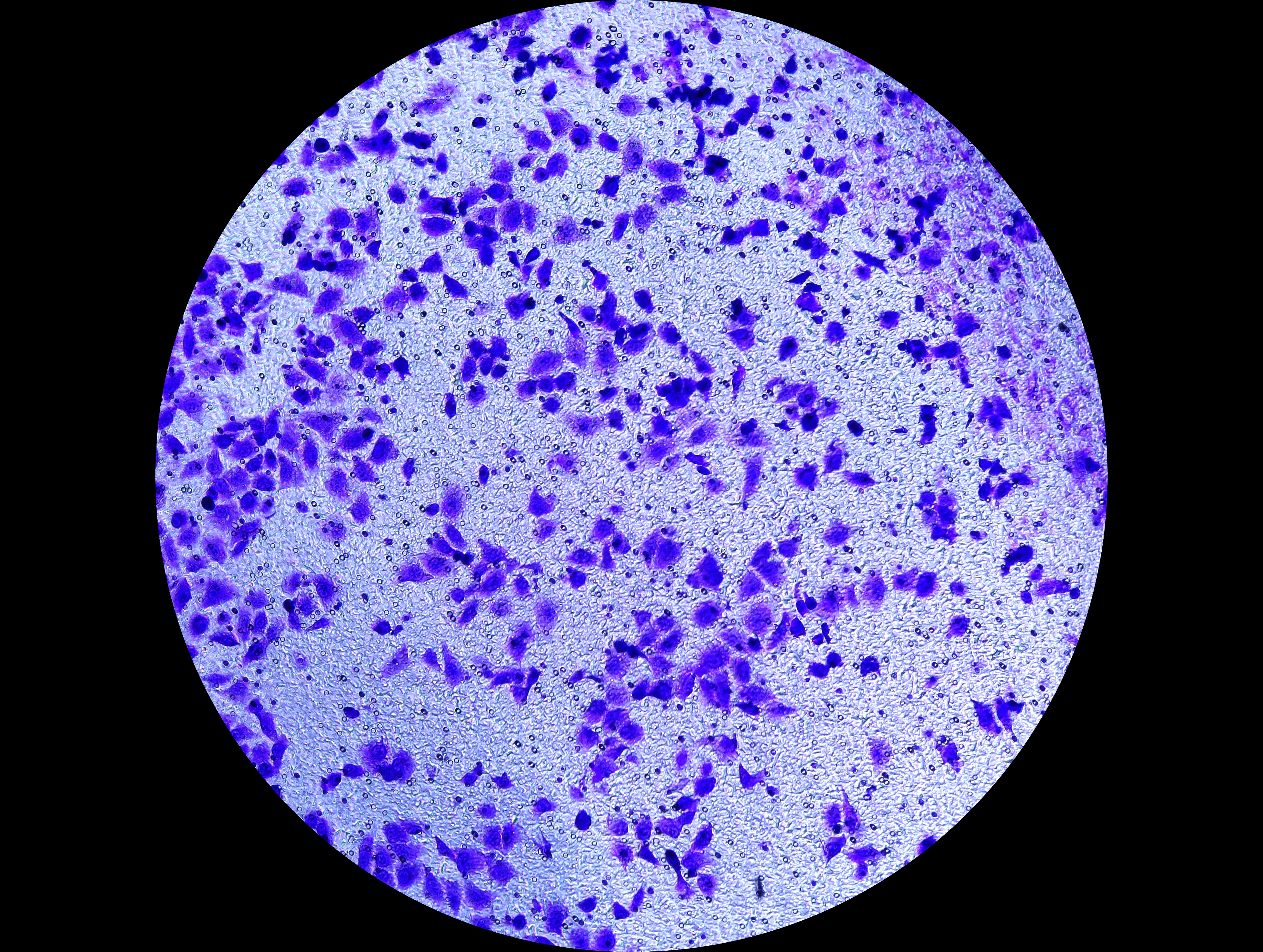

Supplement: S8 File — (ZIP) [file pone.0337223.s009.zip › OE-H1299-Transwell invasion original image/H1299-侵袭-OV-NC (2).JPG]

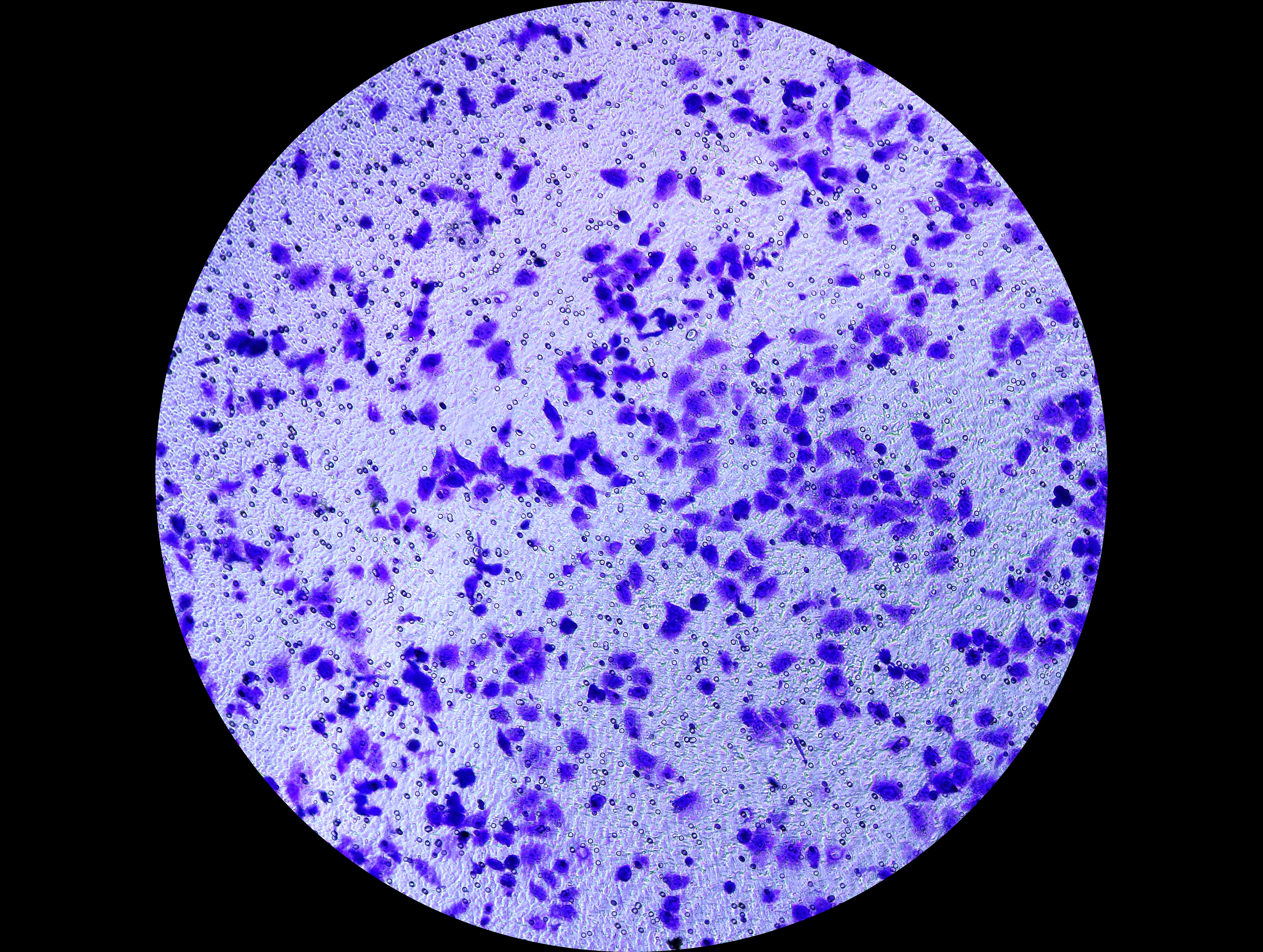

Supplement: S8 File — (ZIP) [file pone.0337223.s009.zip › OE-H1299-Transwell invasion original image/H1299-侵袭-OV-NC (3).JPG]

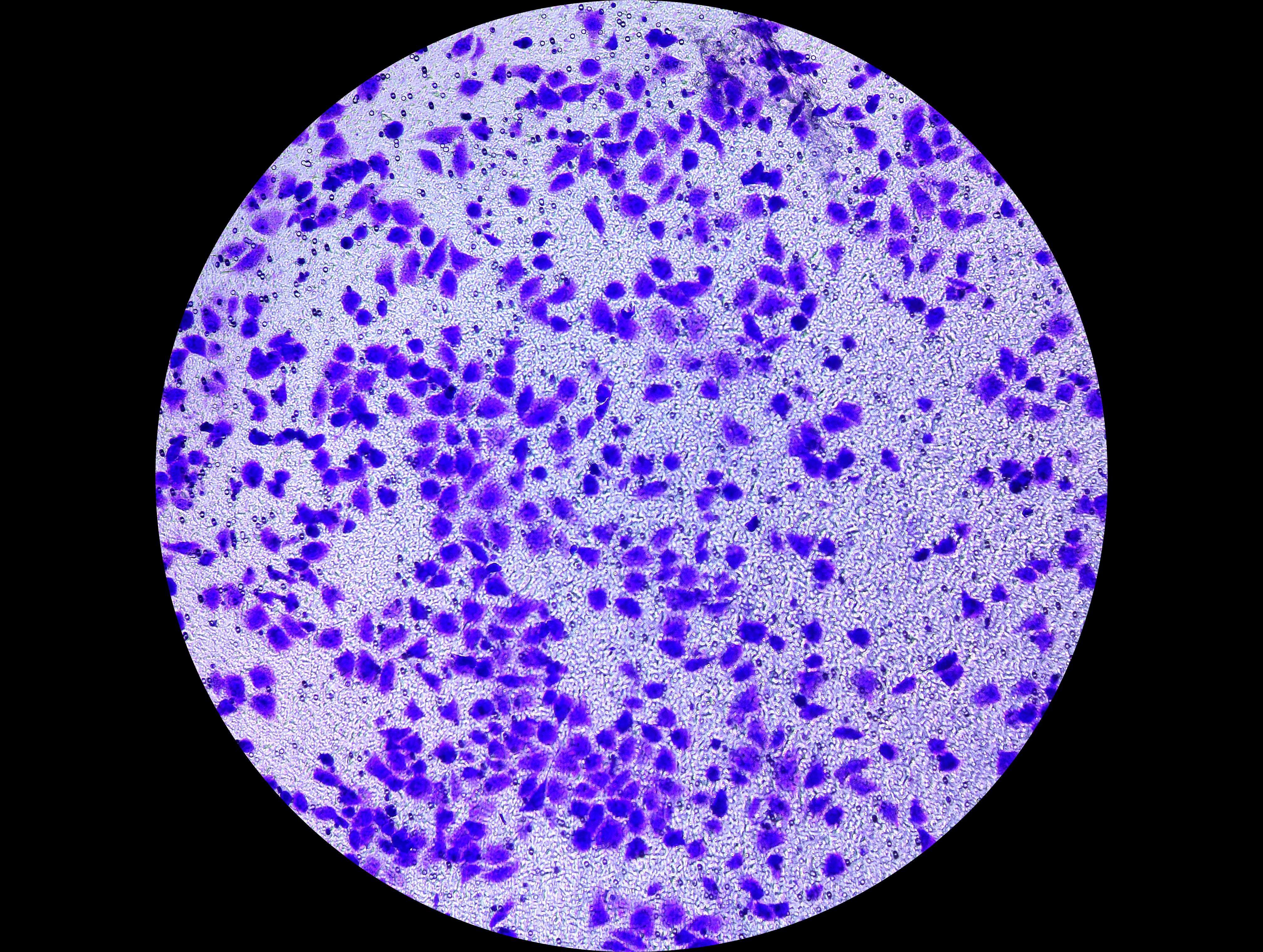

Supplement: S8 File — (ZIP) [file pone.0337223.s009.zip › OE-H1299-Transwell invasion original image/H1299-侵袭-OV-NC (4).JPG]

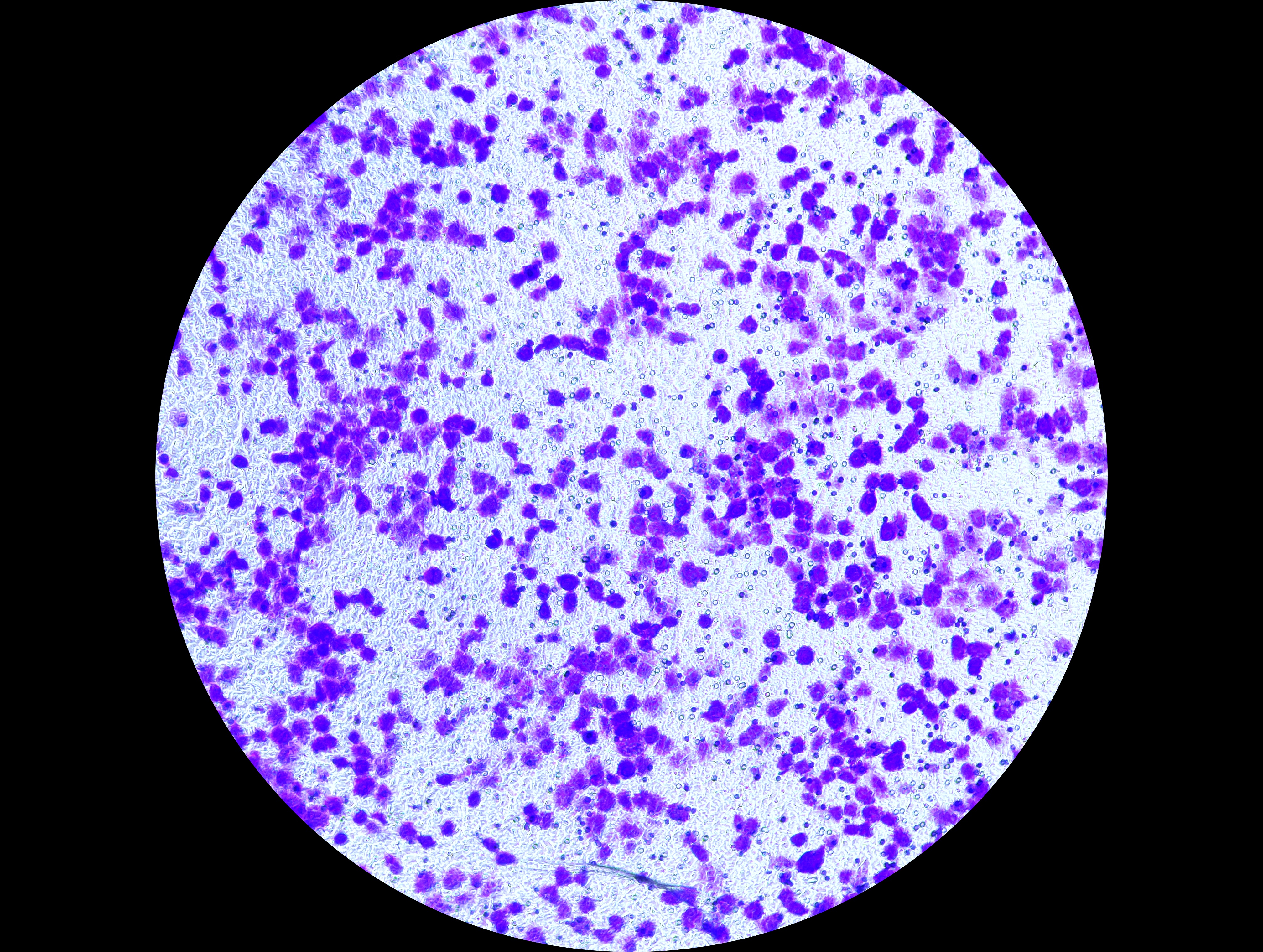

Supplement: S9 File — (ZIP) [file pone.0337223.s010.zip › OE-H1299-Transwell migration original image/H1299-迁移 -OV-NC (1).JPG]

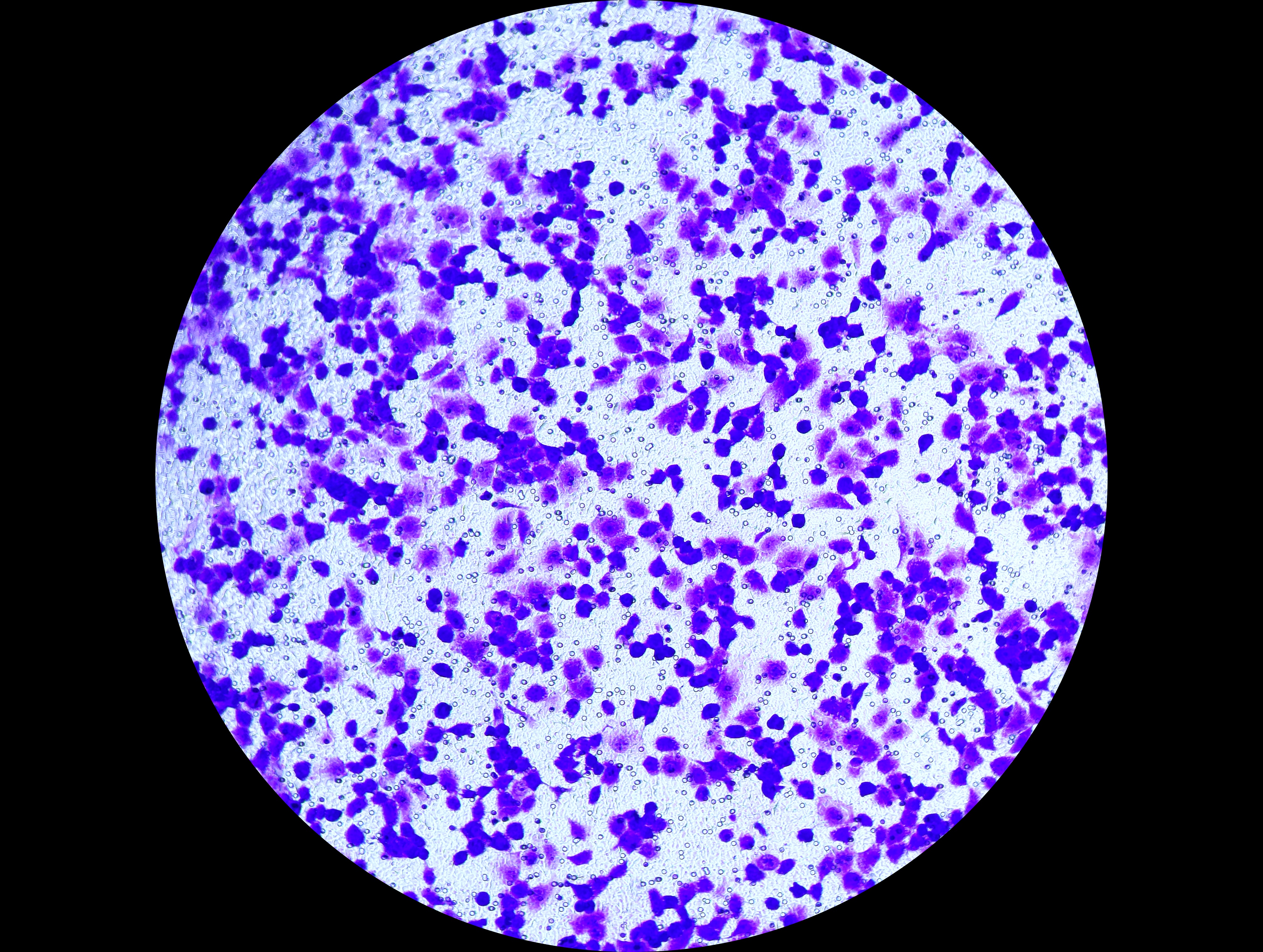

Supplement: S9 File — (ZIP) [file pone.0337223.s010.zip › OE-H1299-Transwell migration original image/H1299-迁移 -OV-NC (2).JPG]

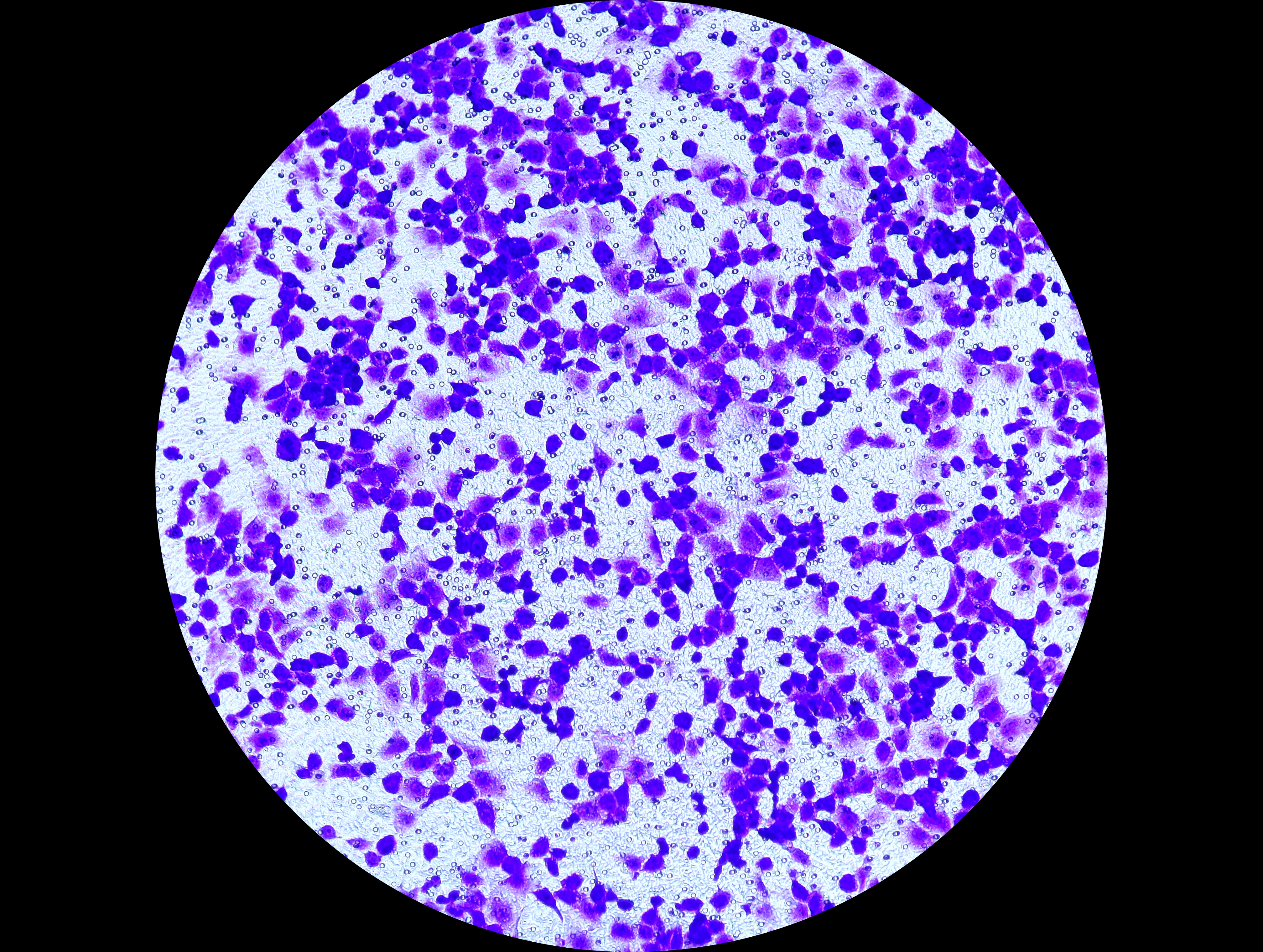

Supplement: S9 File — (ZIP) [file pone.0337223.s010.zip › OE-H1299-Transwell migration original image/H1299-迁移 -OV-NC (3).JPG]

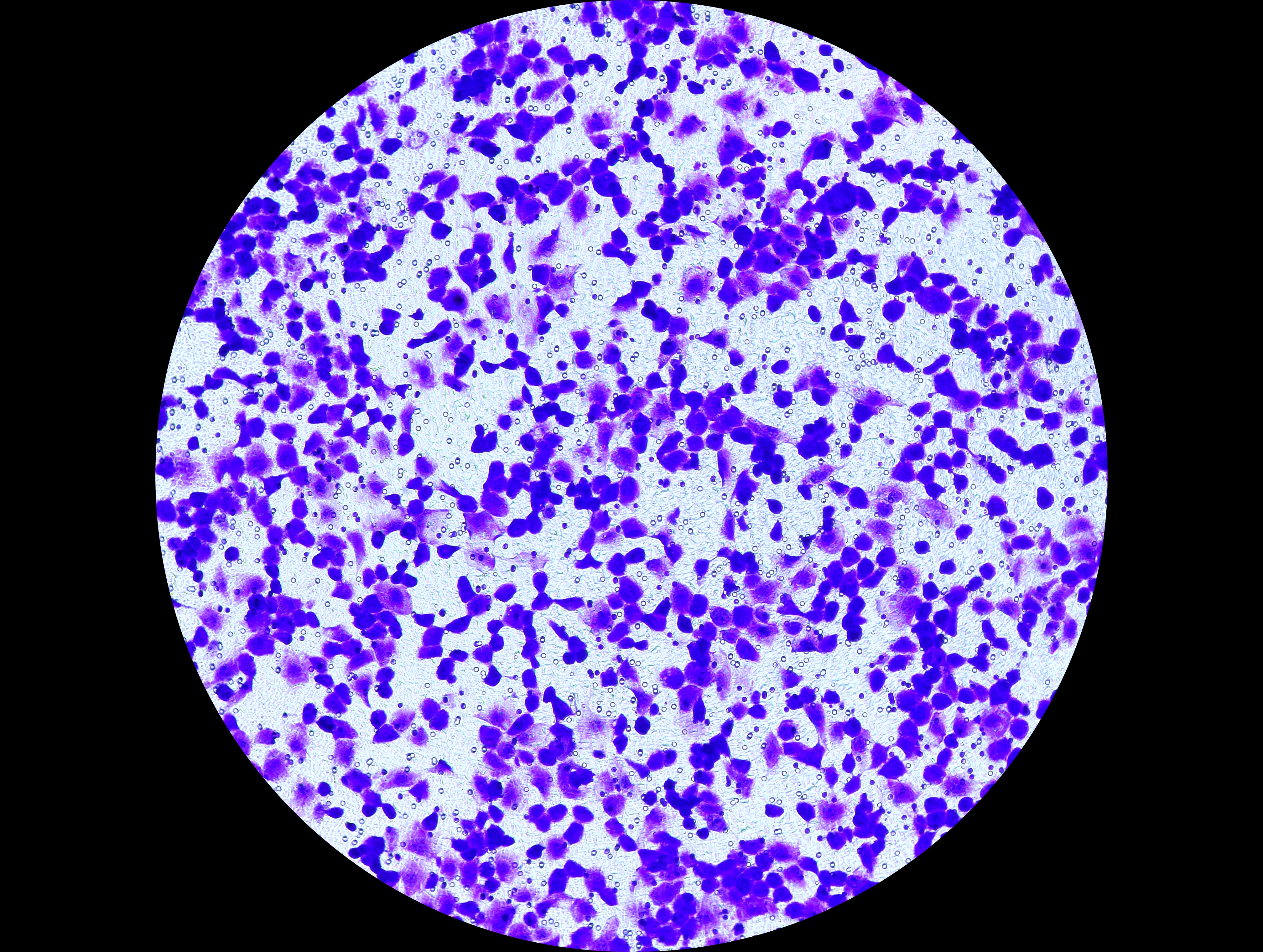

Supplement: S9 File — (ZIP) [file pone.0337223.s010.zip › OE-H1299-Transwell migration original image/H1299-迁移 -OV-NC (4).JPG]

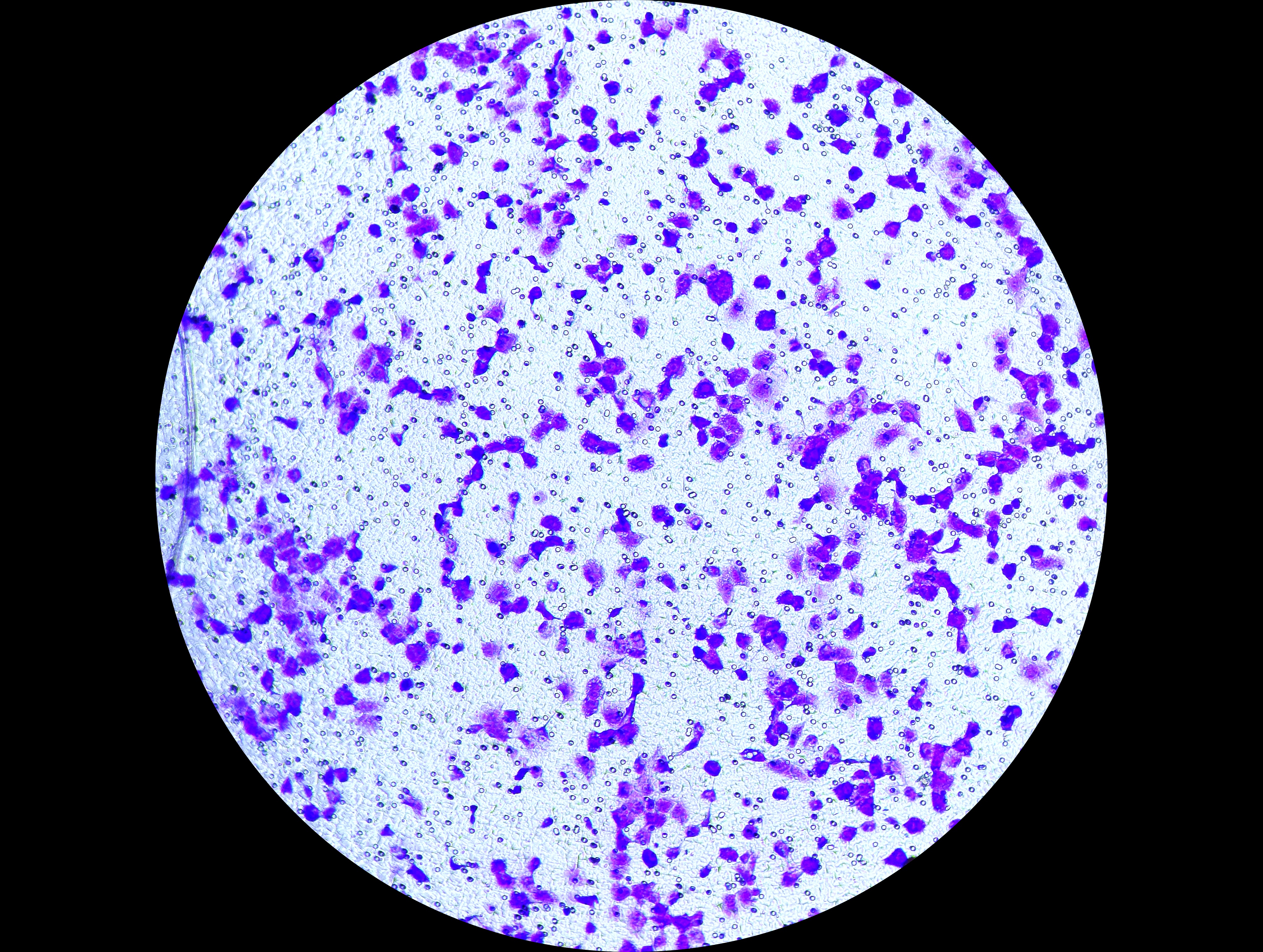

Supplement: S9 File — (ZIP) [file pone.0337223.s010.zip › OE-H1299-Transwell migration original image/H1299-迁移-OV (1).JPG]

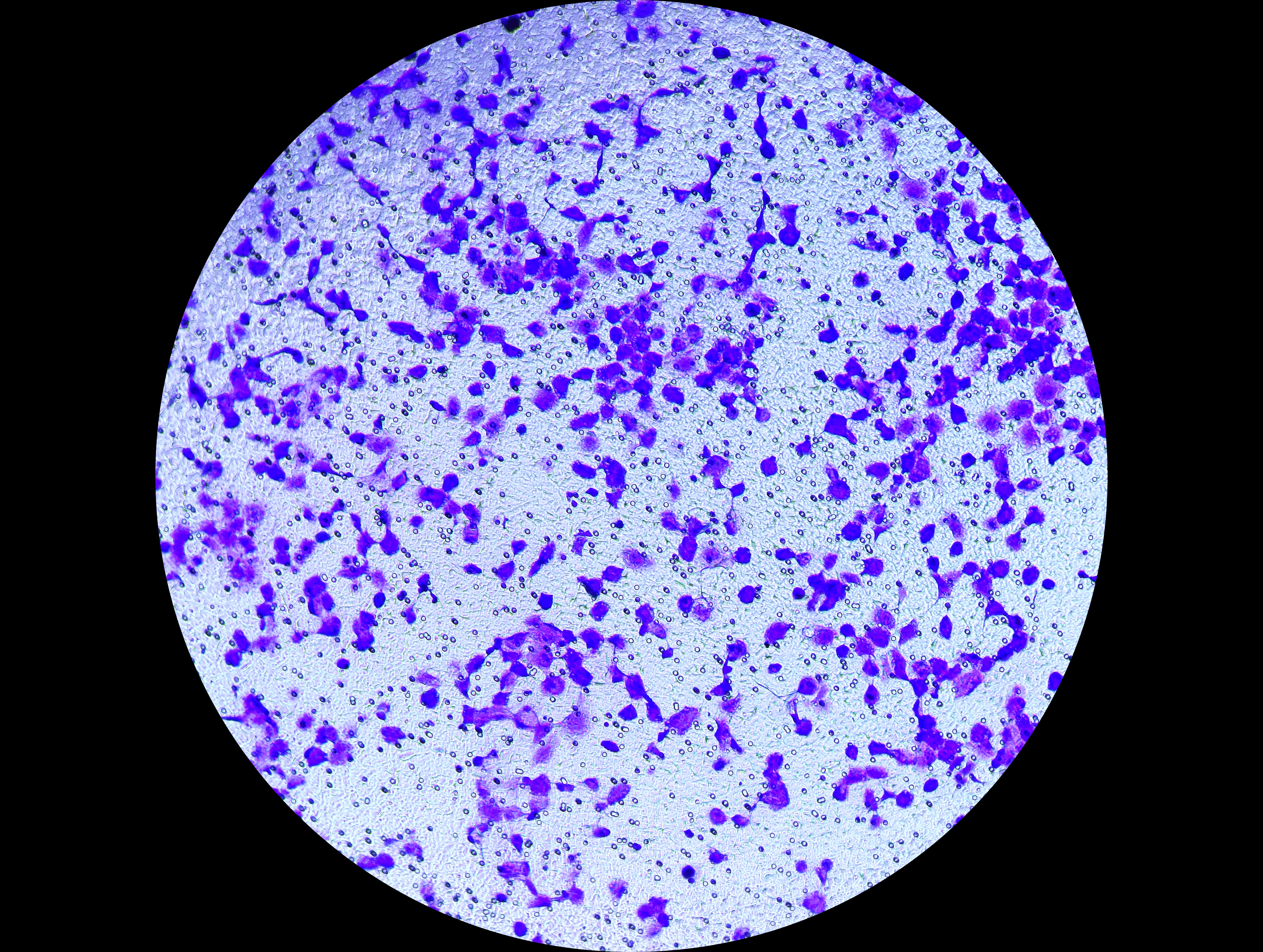

Supplement: S9 File — (ZIP) [file pone.0337223.s010.zip › OE-H1299-Transwell migration original image/H1299-迁移-OV (2).JPG]

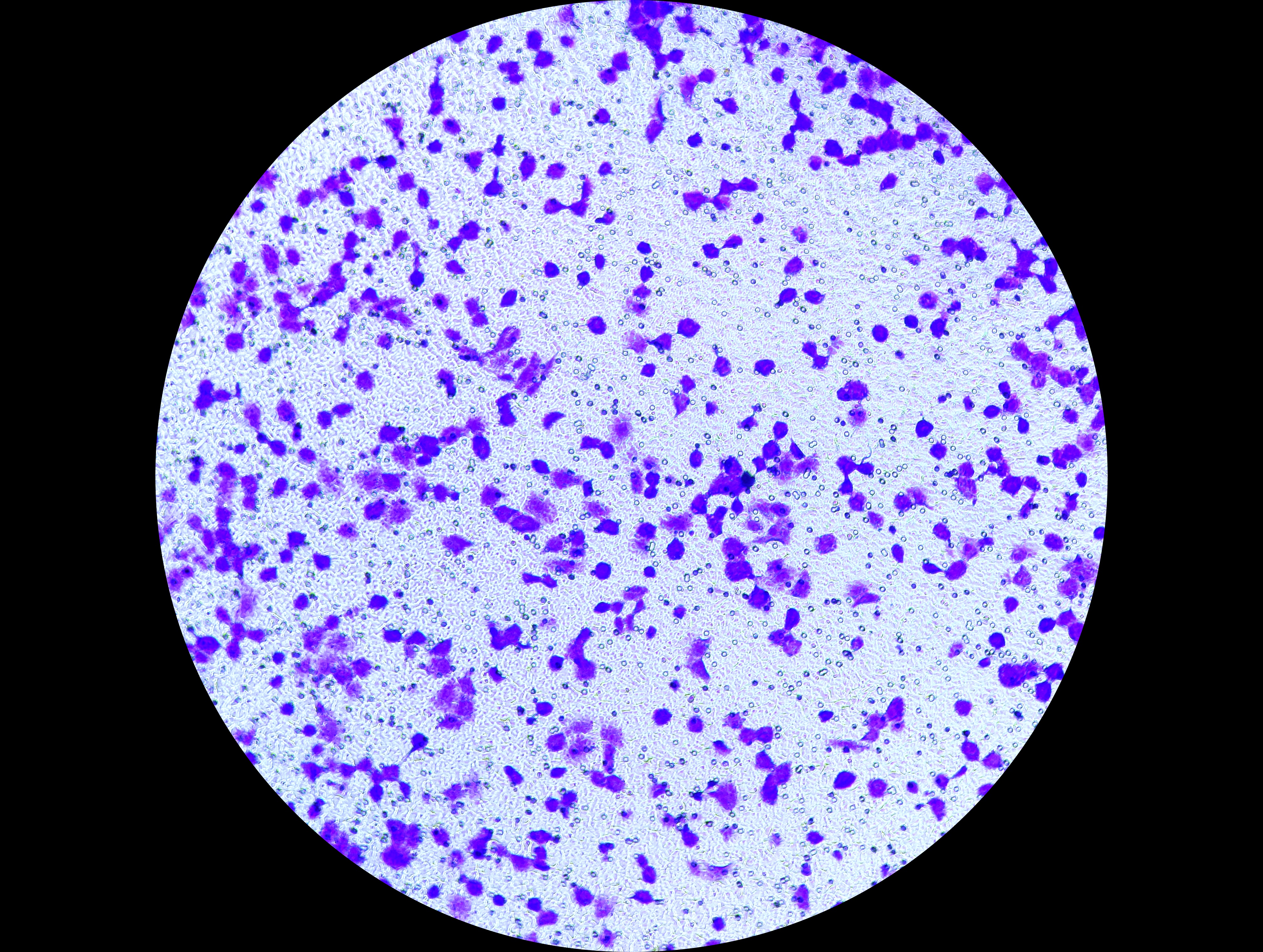

Supplement: S9 File — (ZIP) [file pone.0337223.s010.zip › OE-H1299-Transwell migration original image/H1299-迁移-OV (3).JPG]

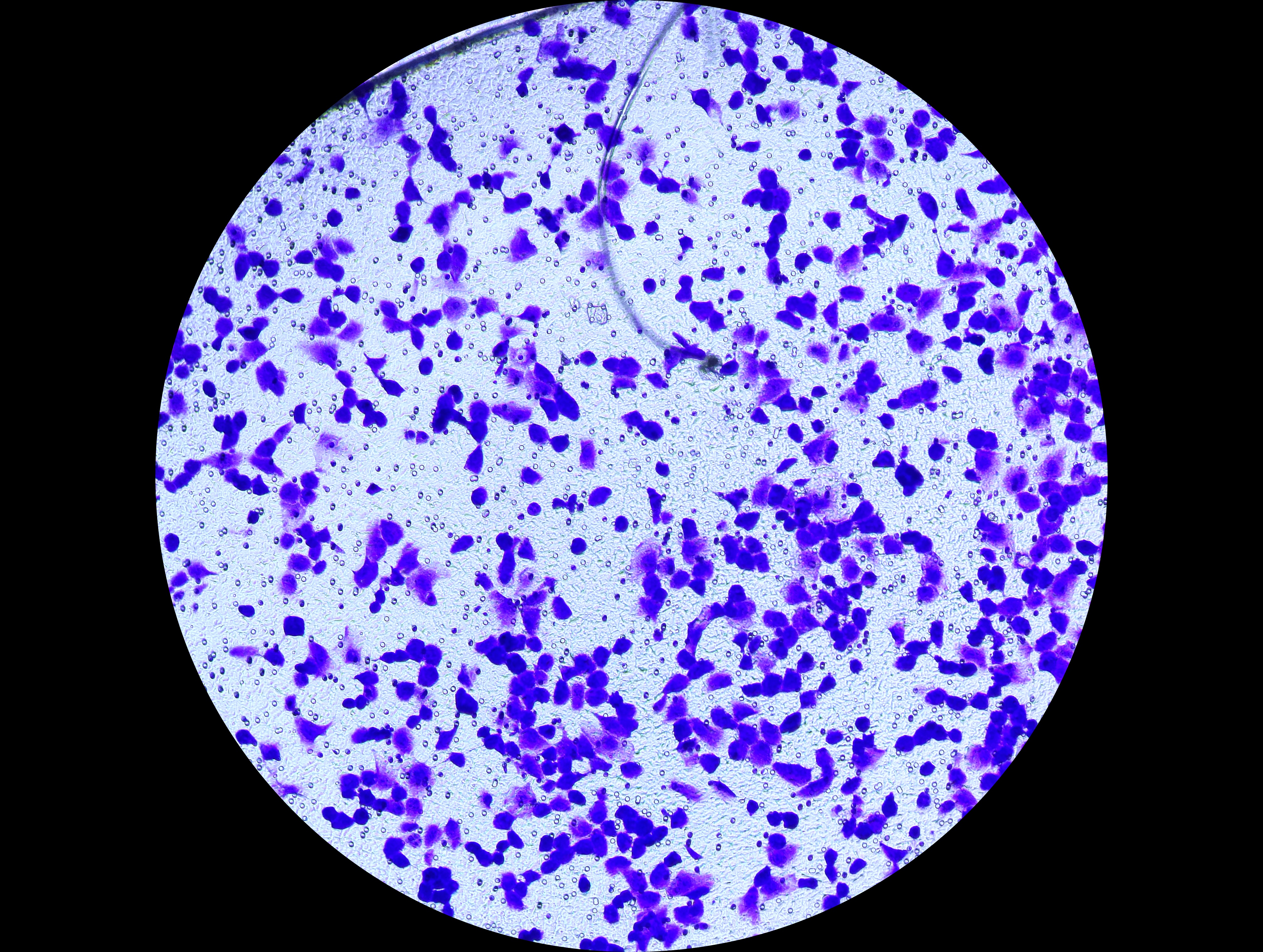

Supplement: S9 File — (ZIP) [file pone.0337223.s010.zip › OE-H1299-Transwell migration original image/H1299-迁移-OV (4).JPG]

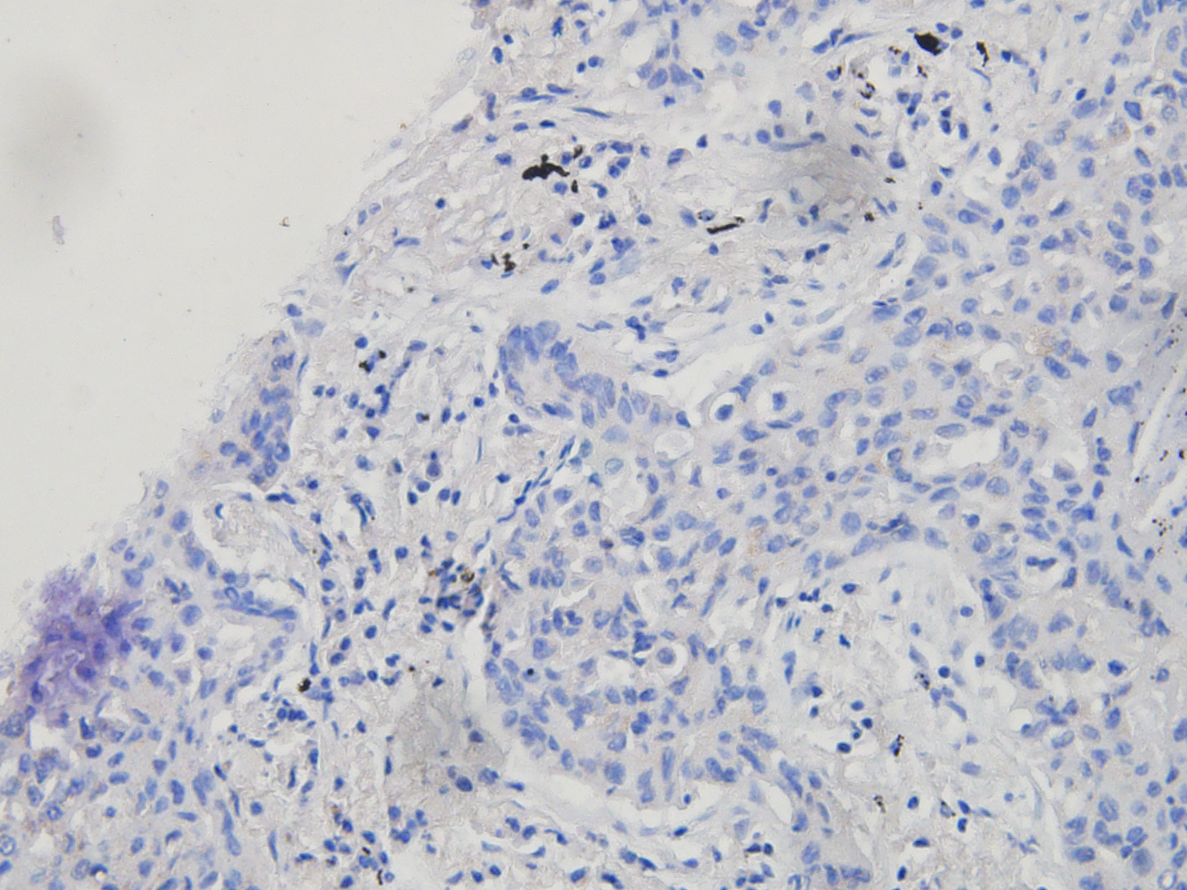

Supplement: S13 File — (ZIP) [file pone.0337223.s014.zip › 461763-400x-CA-N/461763-400x--CA (1).tif]

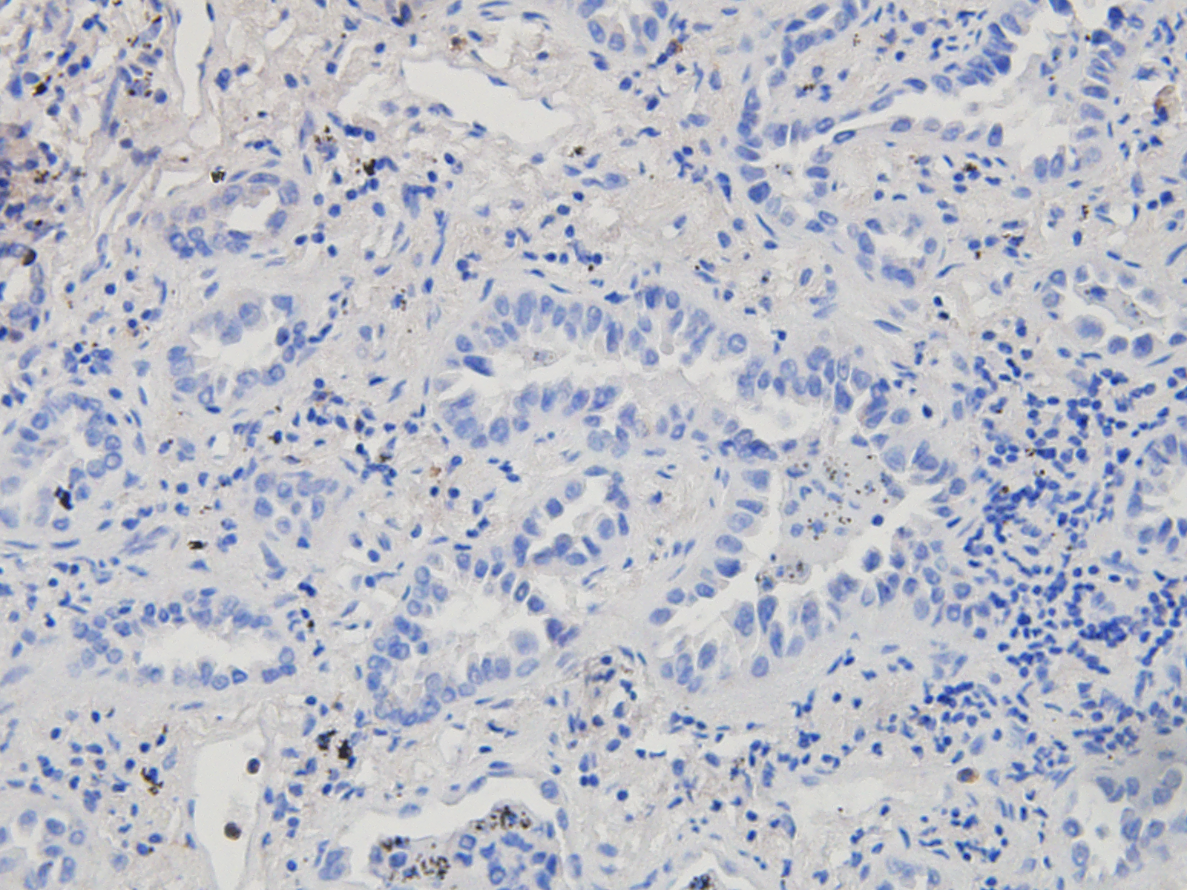

Supplement: S13 File — (ZIP) [file pone.0337223.s014.zip › 461763-400x-CA-N/461763-400x--CA (2).tif]

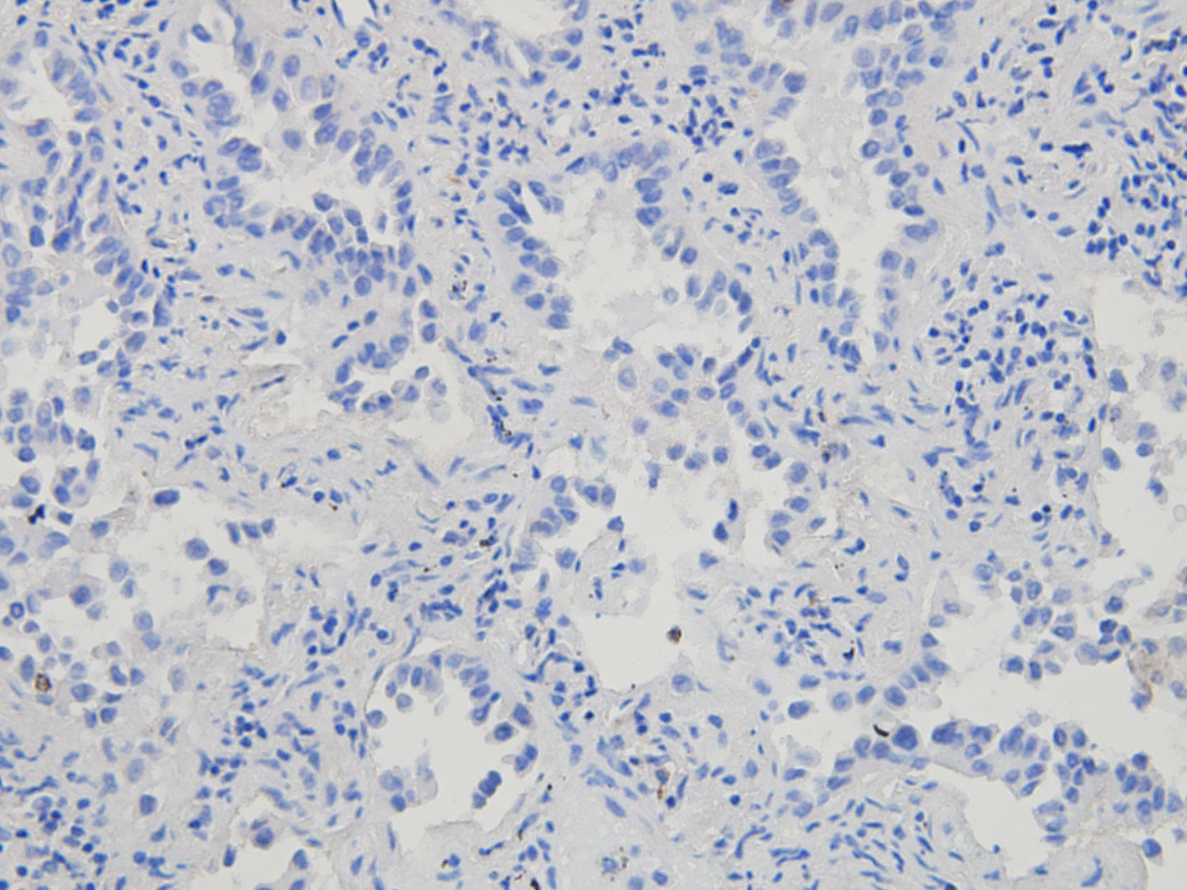

Supplement: S13 File — (ZIP) [file pone.0337223.s014.zip › 461763-400x-CA-N/461763-400x--CA (3).tif]

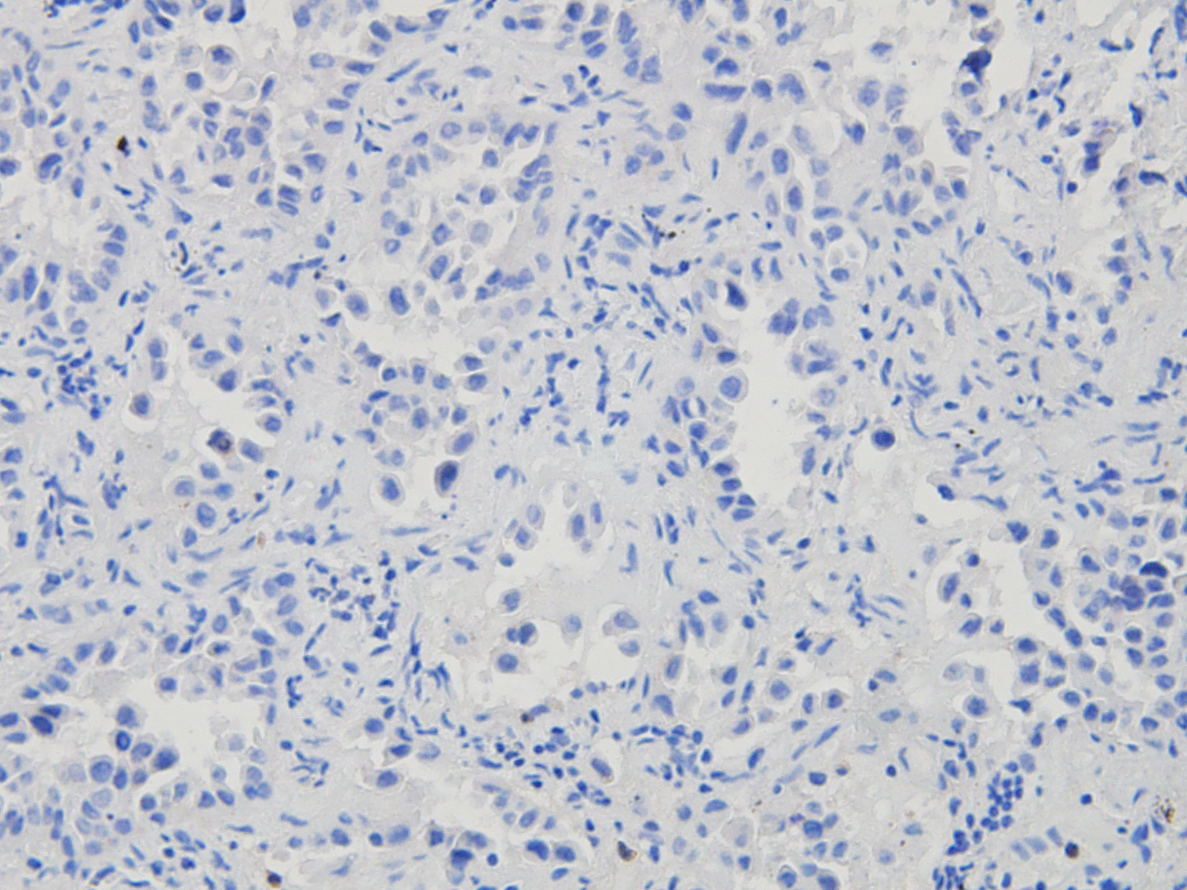

Supplement: S13 File — (ZIP) [file pone.0337223.s014.zip › 461763-400x-CA-N/461763-400x--CA (4).tif]

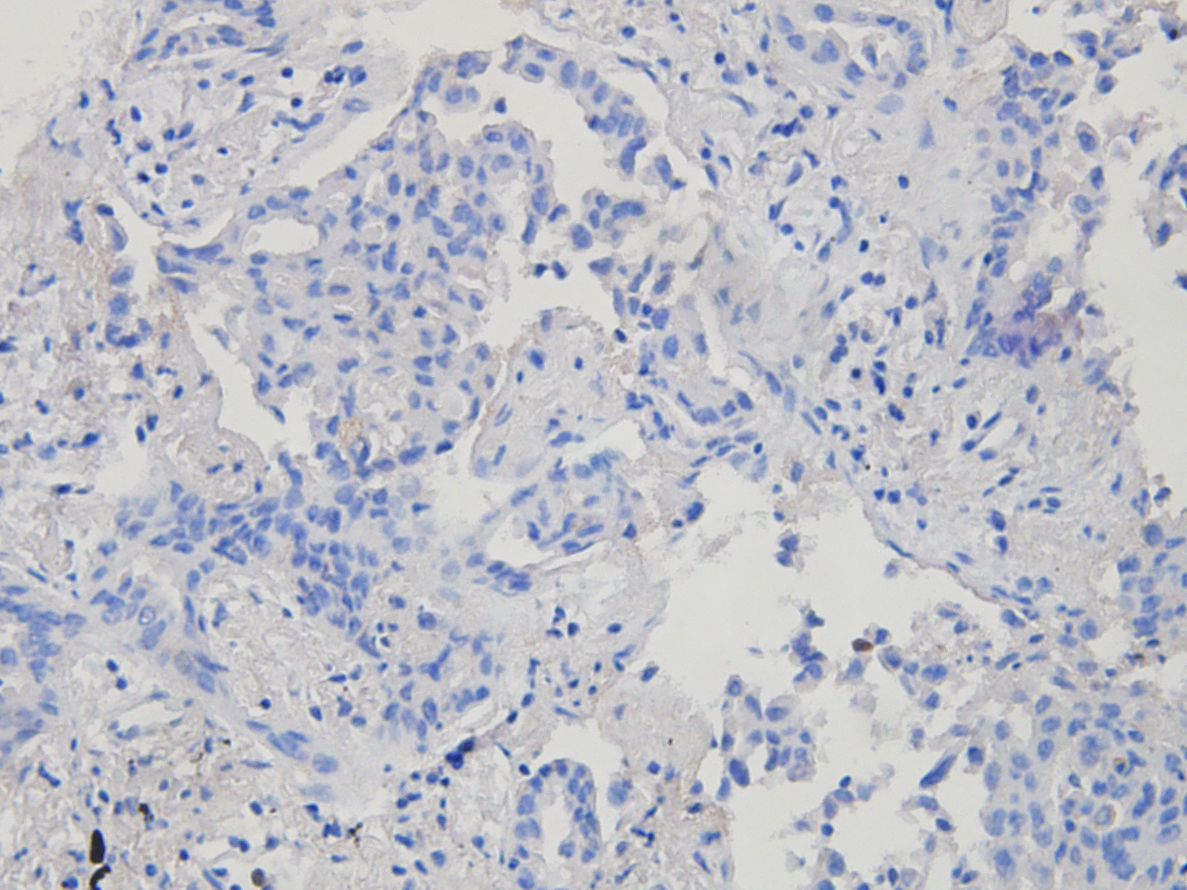

Supplement: S13 File — (ZIP) [file pone.0337223.s014.zip › 461763-400x-CA-N/461763-400x--CA (5).tif]

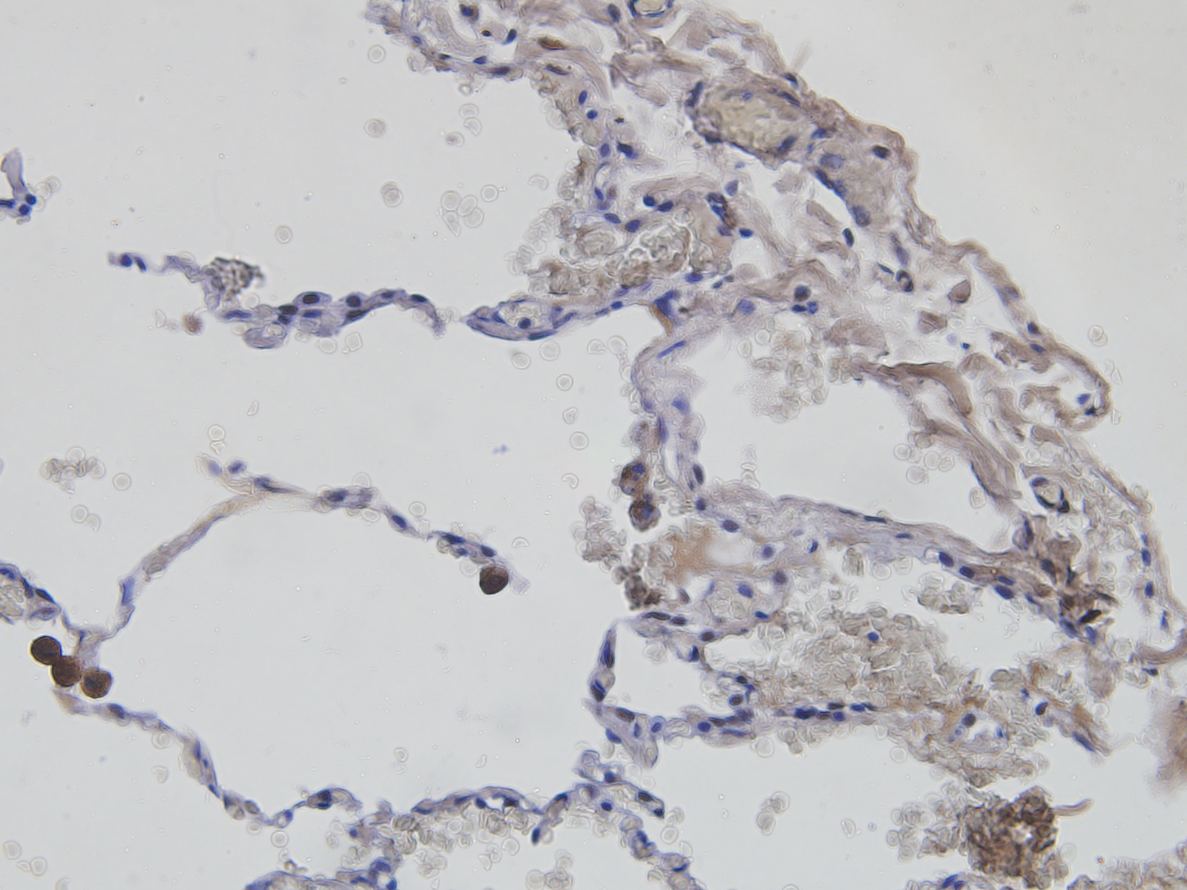

Supplement: S13 File — (ZIP) [file pone.0337223.s014.zip › 461763-400x-CA-N/461763-400x-N (1).tif]

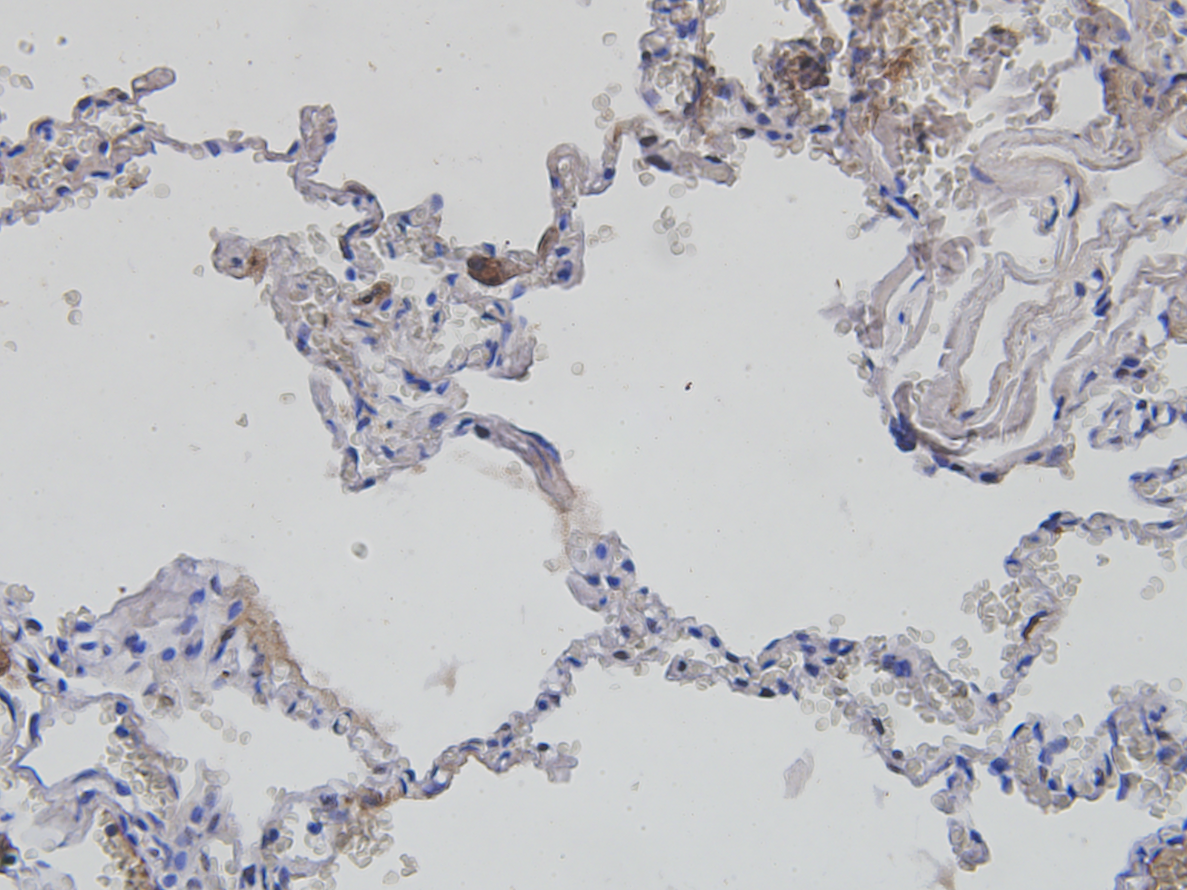

Supplement: S13 File — (ZIP) [file pone.0337223.s014.zip › 461763-400x-CA-N/461763-400x-N (2).tif]

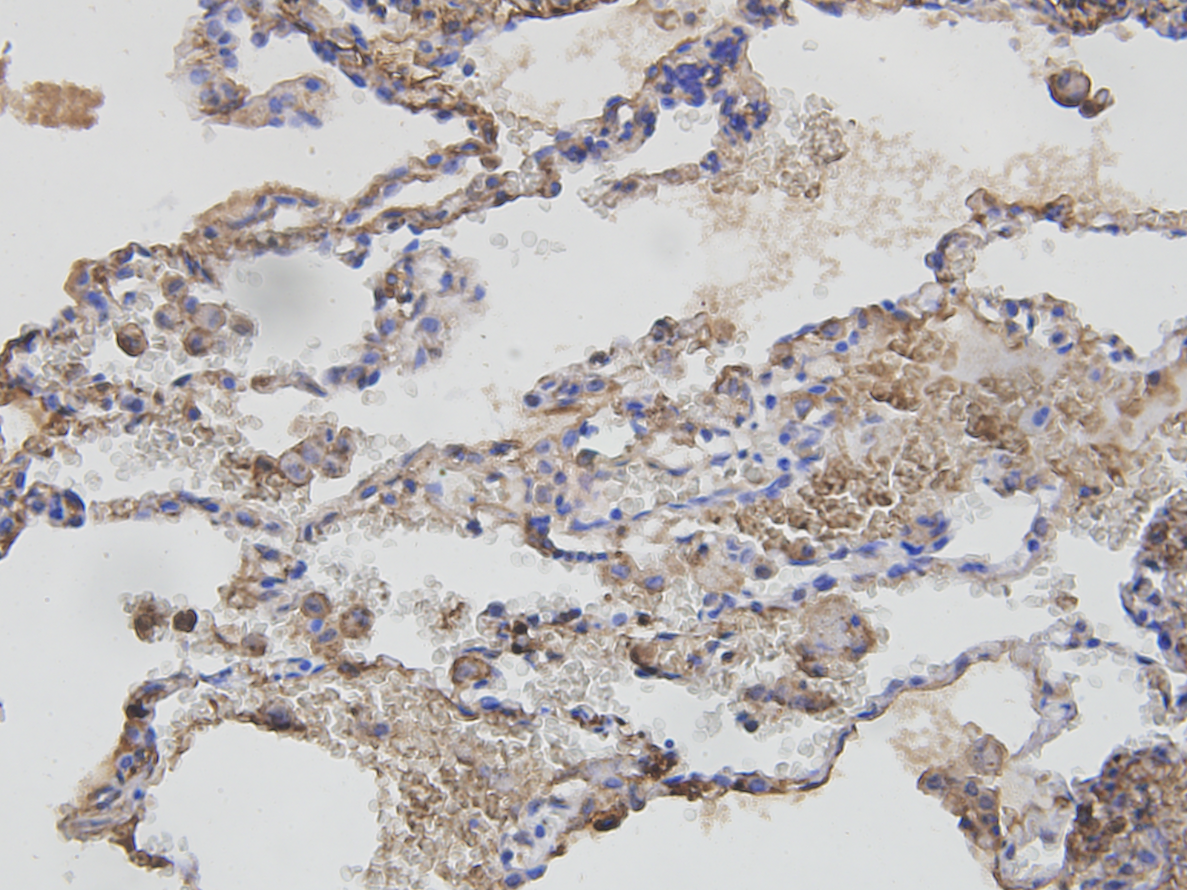

Supplement: S13 File — (ZIP) [file pone.0337223.s014.zip › 461763-400x-CA-N/461763-400x-N (3).tif]

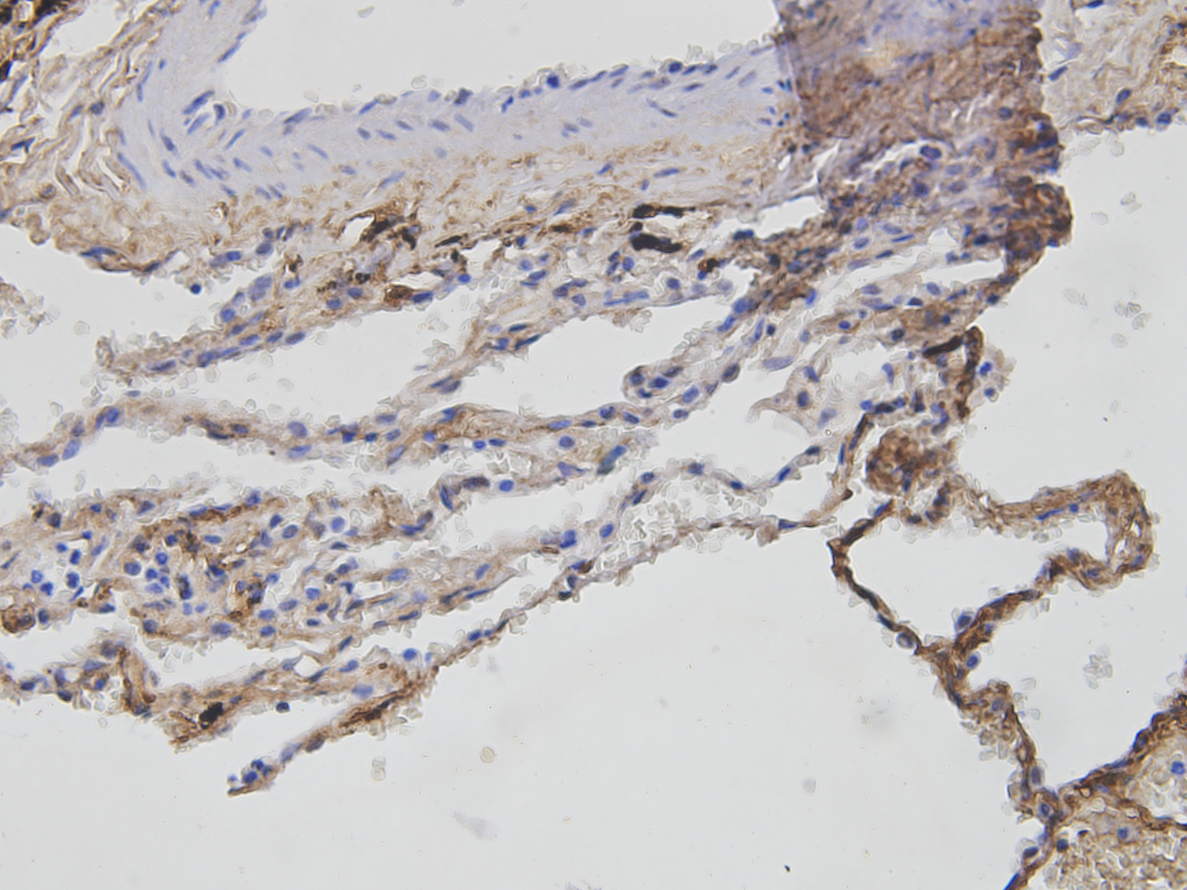

Supplement: S13 File — (ZIP) [file pone.0337223.s014.zip › 461763-400x-CA-N/461763-400x-N (4).tif]

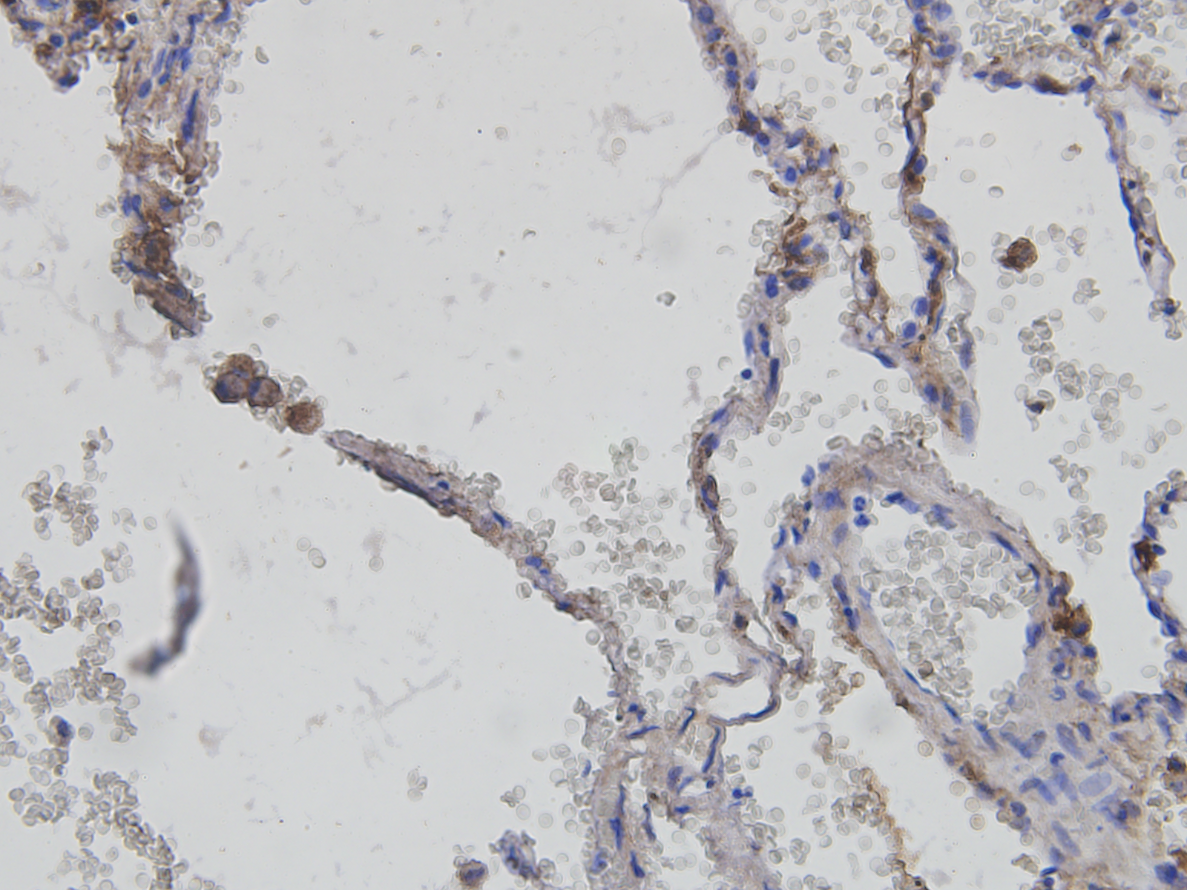

Supplement: S13 File — (ZIP) [file pone.0337223.s014.zip › 461763-400x-CA-N/461763-400x-N (5).tif]

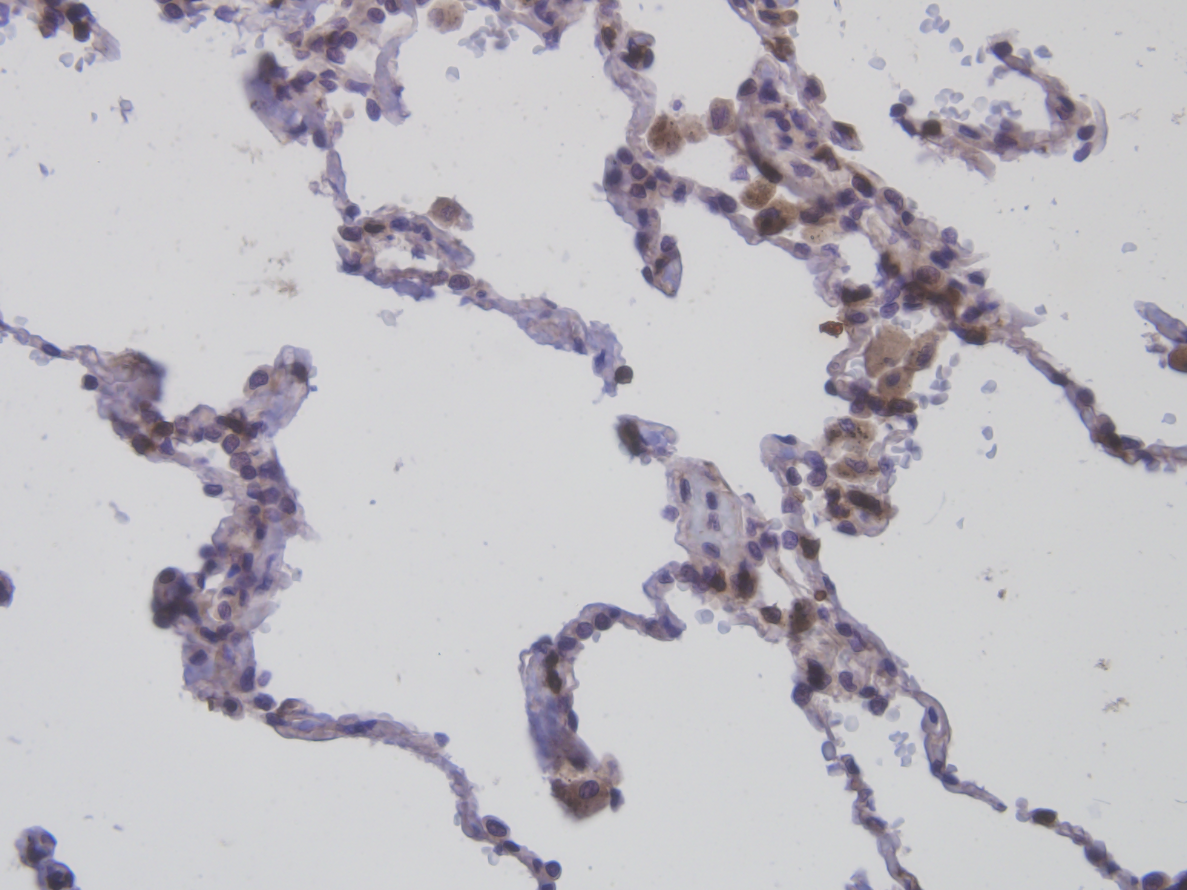

Supplement: S14 File — (ZIP) [file pone.0337223.s015.zip › 461765-400X-CA-N/461765-400X--N (1).tif]

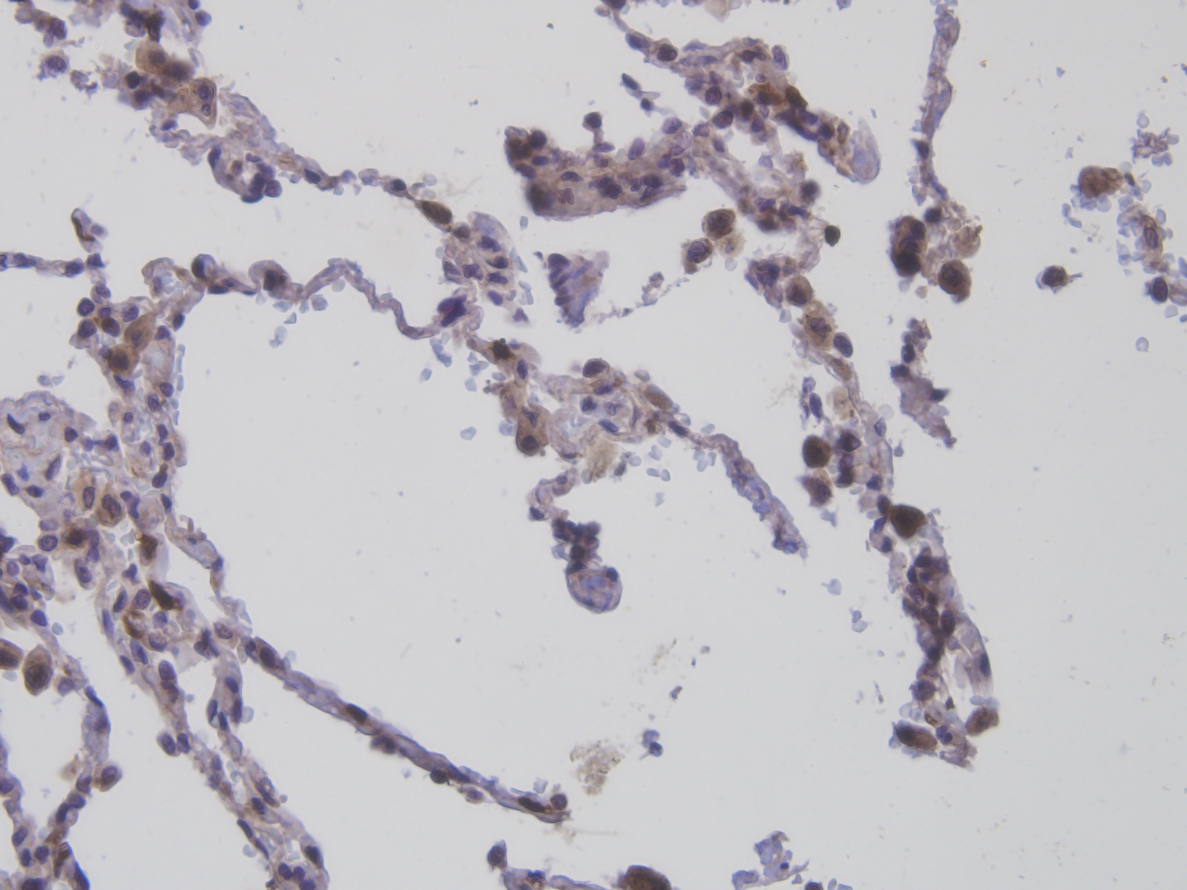

Supplement: S14 File — (ZIP) [file pone.0337223.s015.zip › 461765-400X-CA-N/461765-400X--N (2).tif]

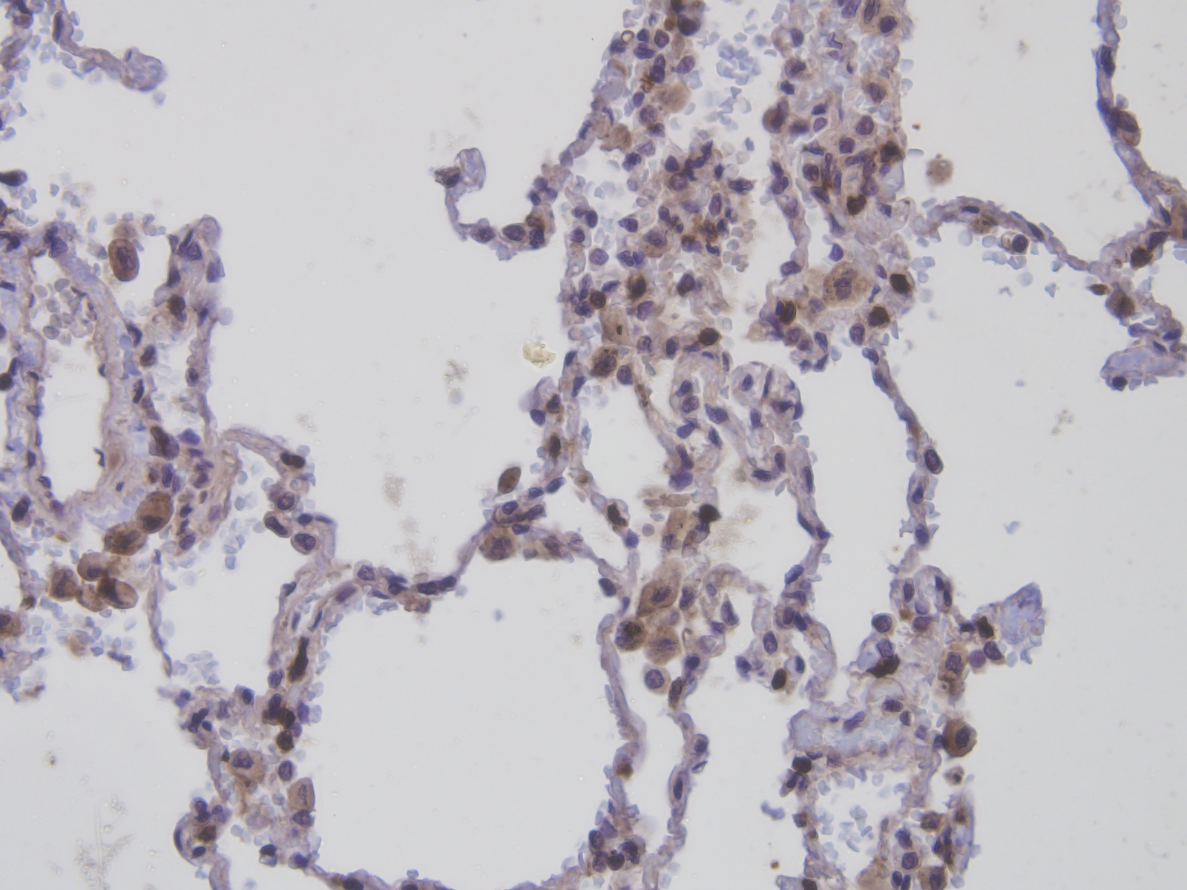

Supplement: S14 File — (ZIP) [file pone.0337223.s015.zip › 461765-400X-CA-N/461765-400X--N (3).tif]

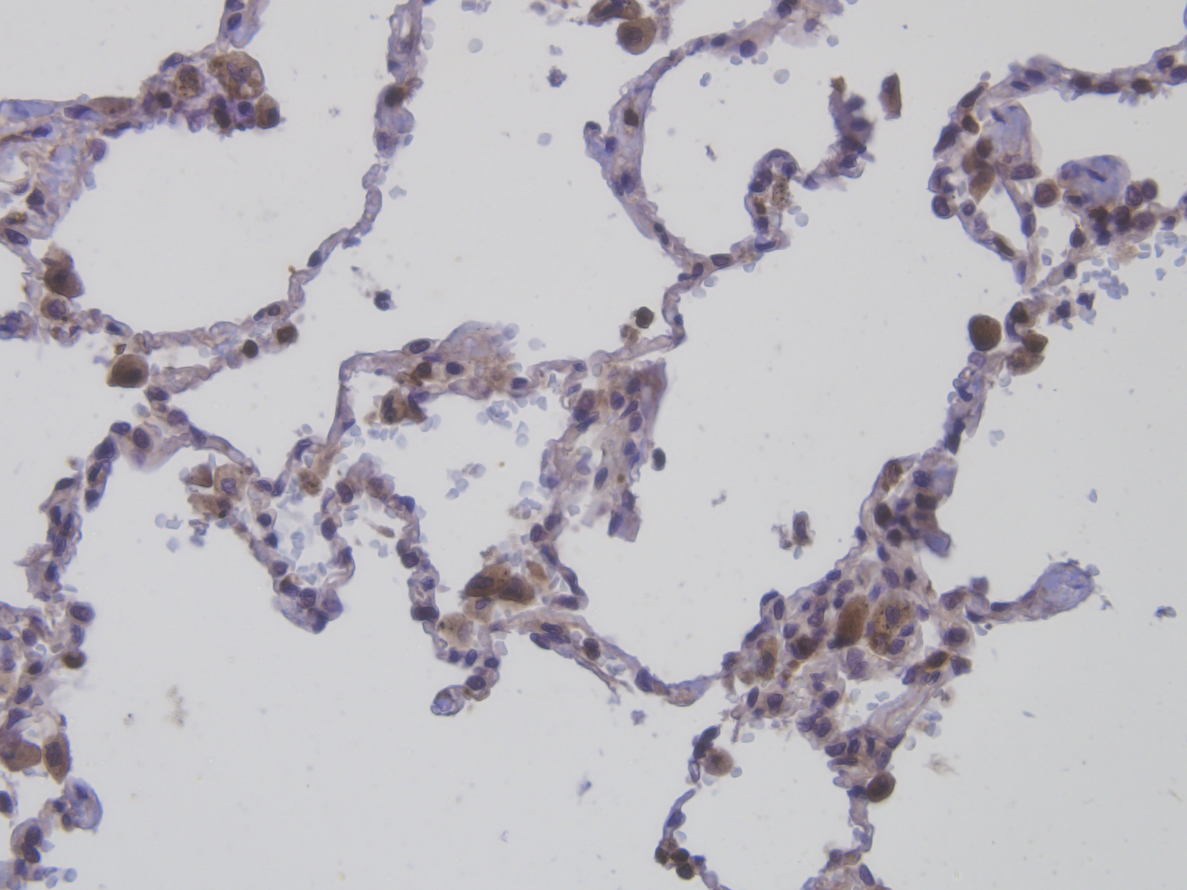

Supplement: S14 File — (ZIP) [file pone.0337223.s015.zip › 461765-400X-CA-N/461765-400X--N (4).tif]

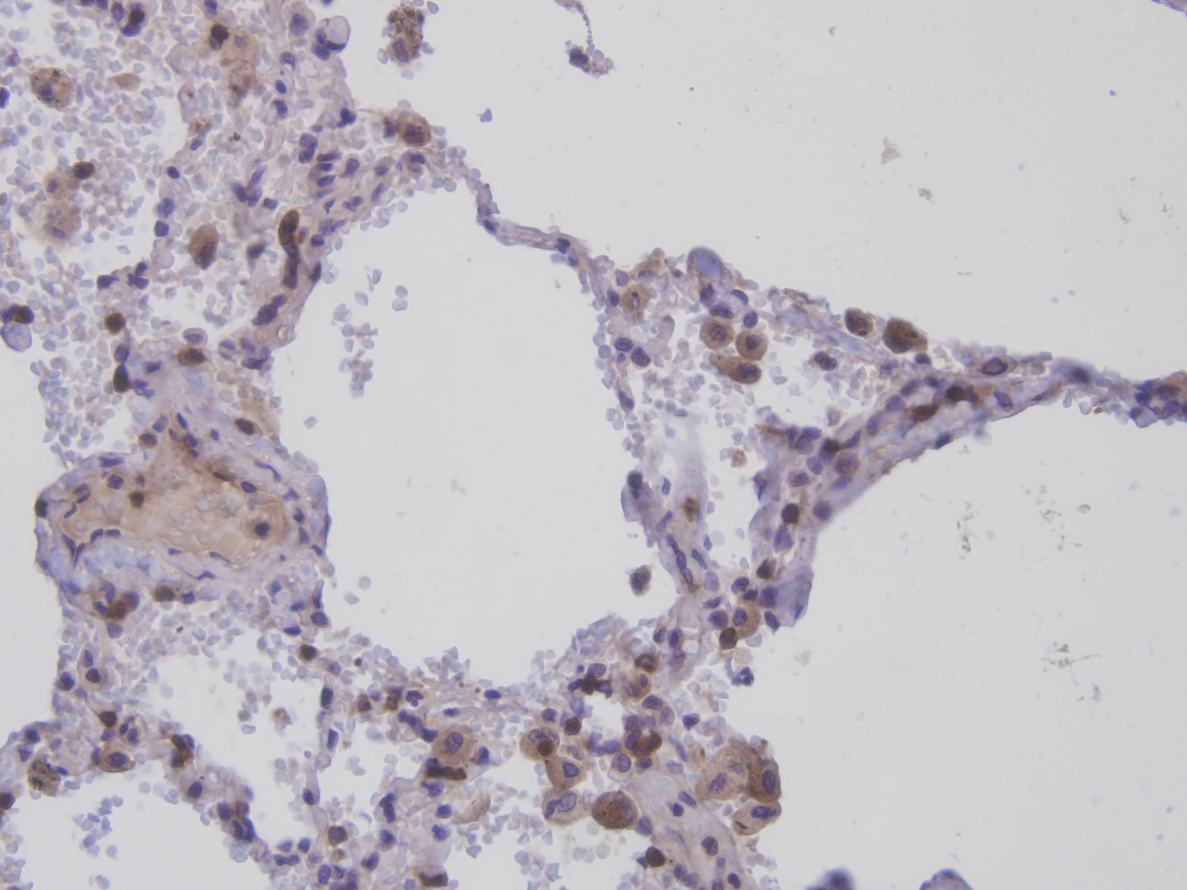

Supplement: S14 File — (ZIP) [file pone.0337223.s015.zip › 461765-400X-CA-N/461765-400X--N (5).tif]

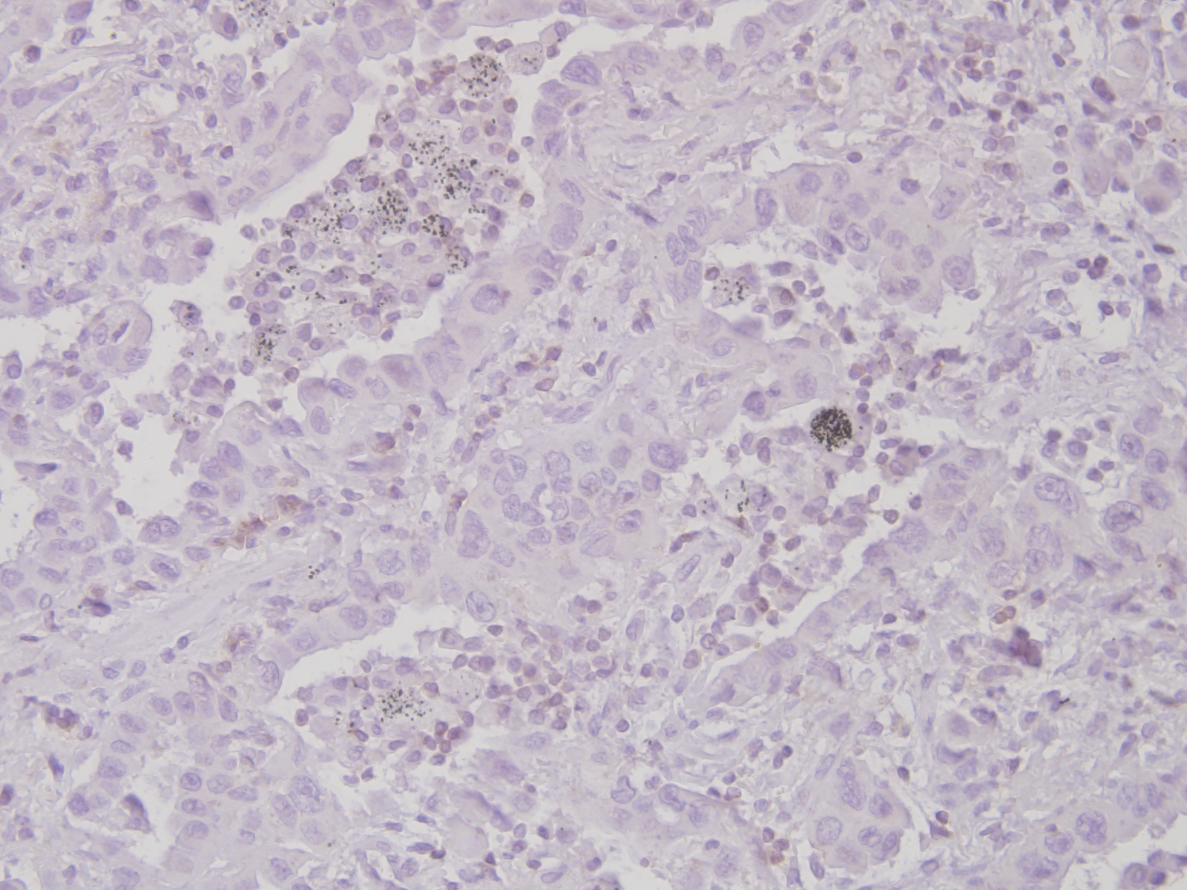

Supplement: S14 File — (ZIP) [file pone.0337223.s015.zip › 461765-400X-CA-N/461765-400X-CA (1).tif]

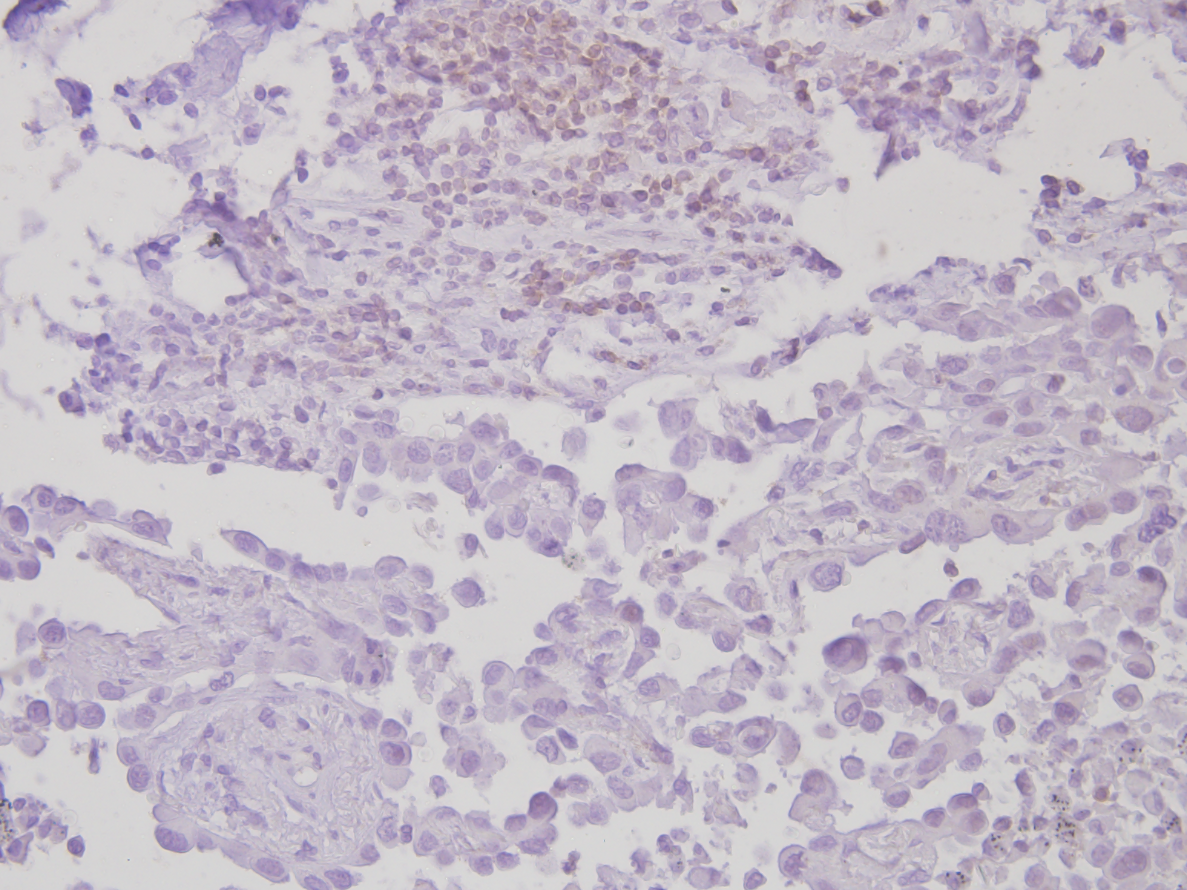

Supplement: S14 File — (ZIP) [file pone.0337223.s015.zip › 461765-400X-CA-N/461765-400X-CA (2).tif]

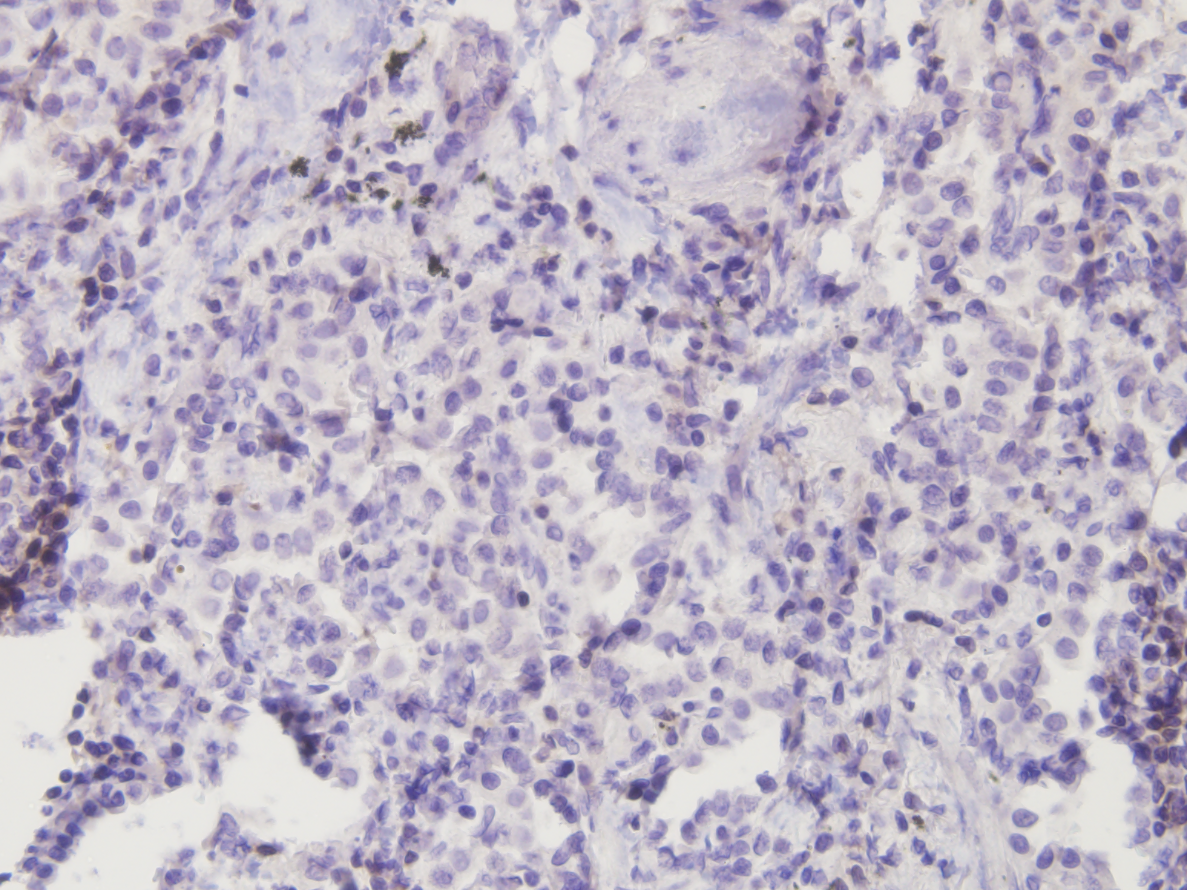

Supplement: S14 File — (ZIP) [file pone.0337223.s015.zip › 461765-400X-CA-N/461765-400X-CA (3).tif]

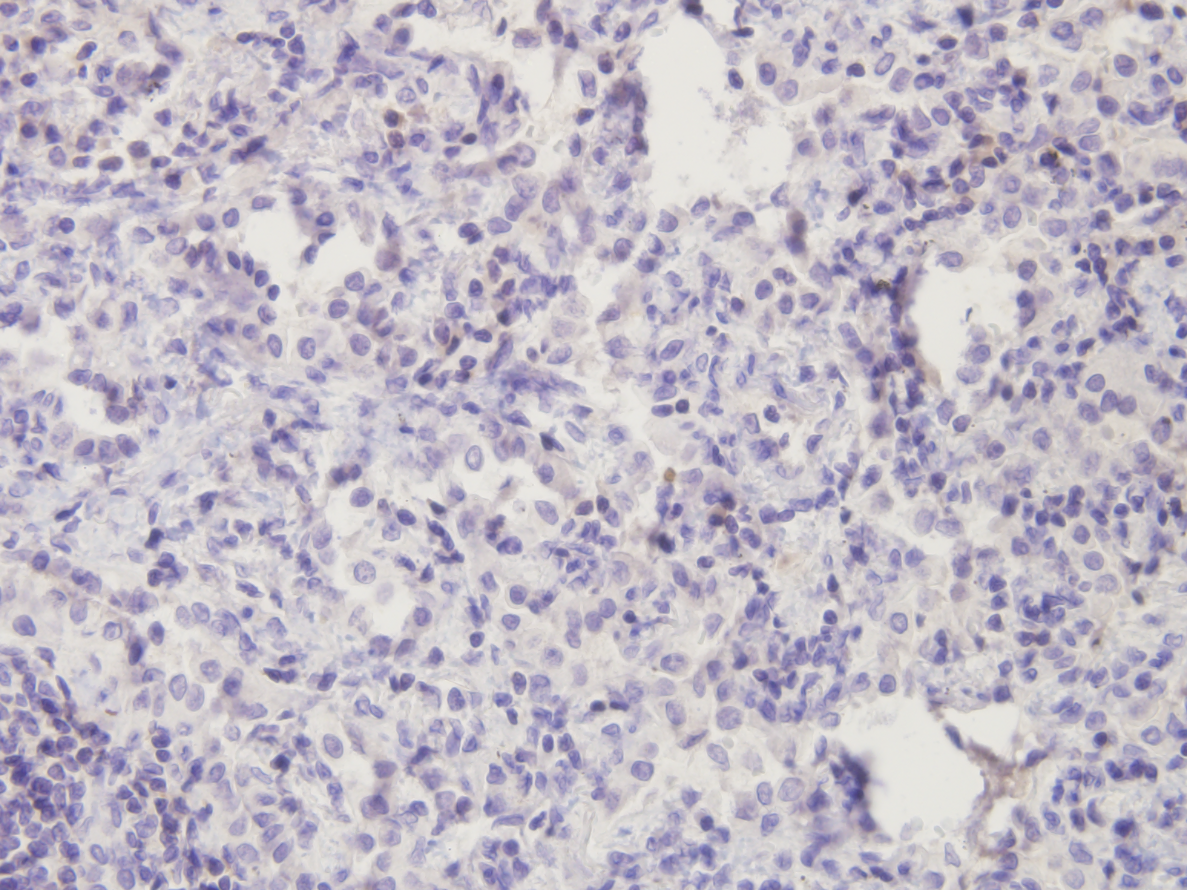

Supplement: S14 File — (ZIP) [file pone.0337223.s015.zip › 461765-400X-CA-N/461765-400X-CA (4).tif]

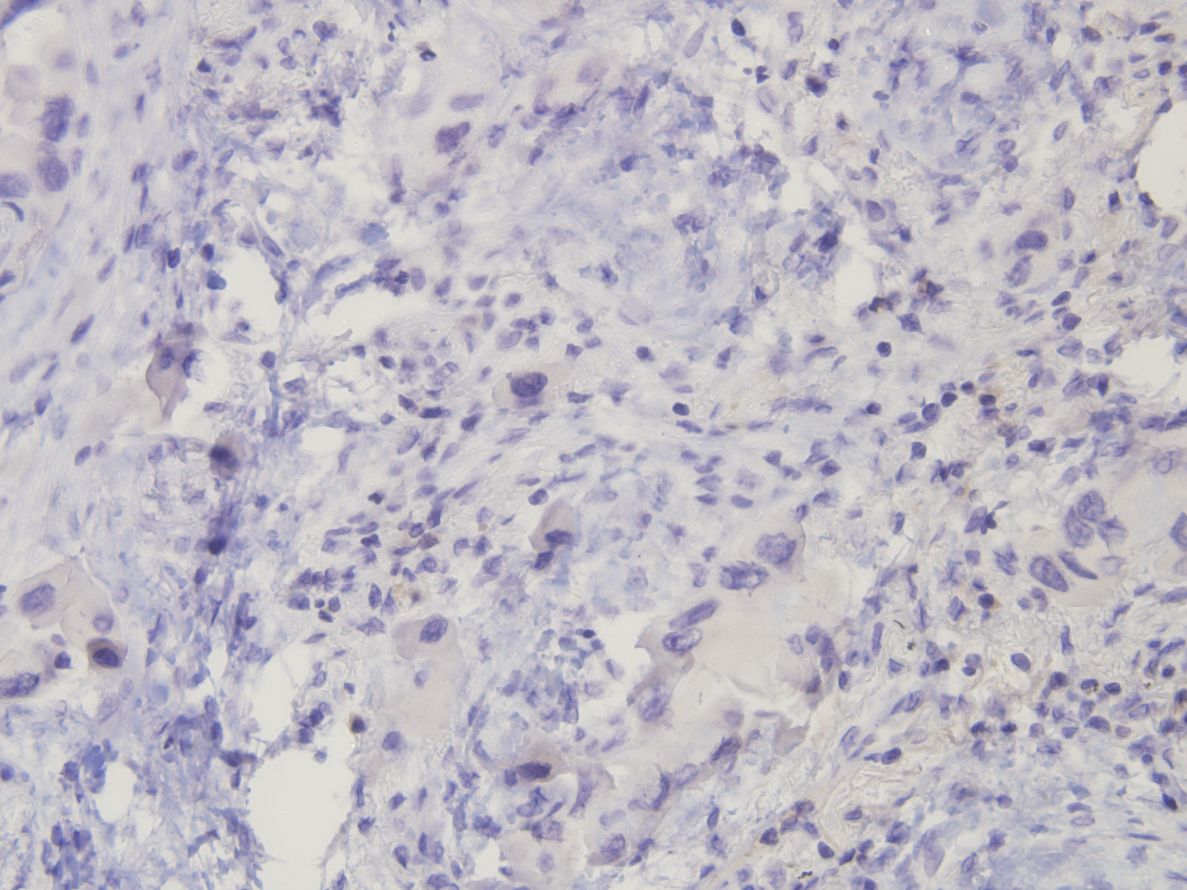

Supplement: S14 File — (ZIP) [file pone.0337223.s015.zip › 461765-400X-CA-N/461765-400X-CA (5).tif]

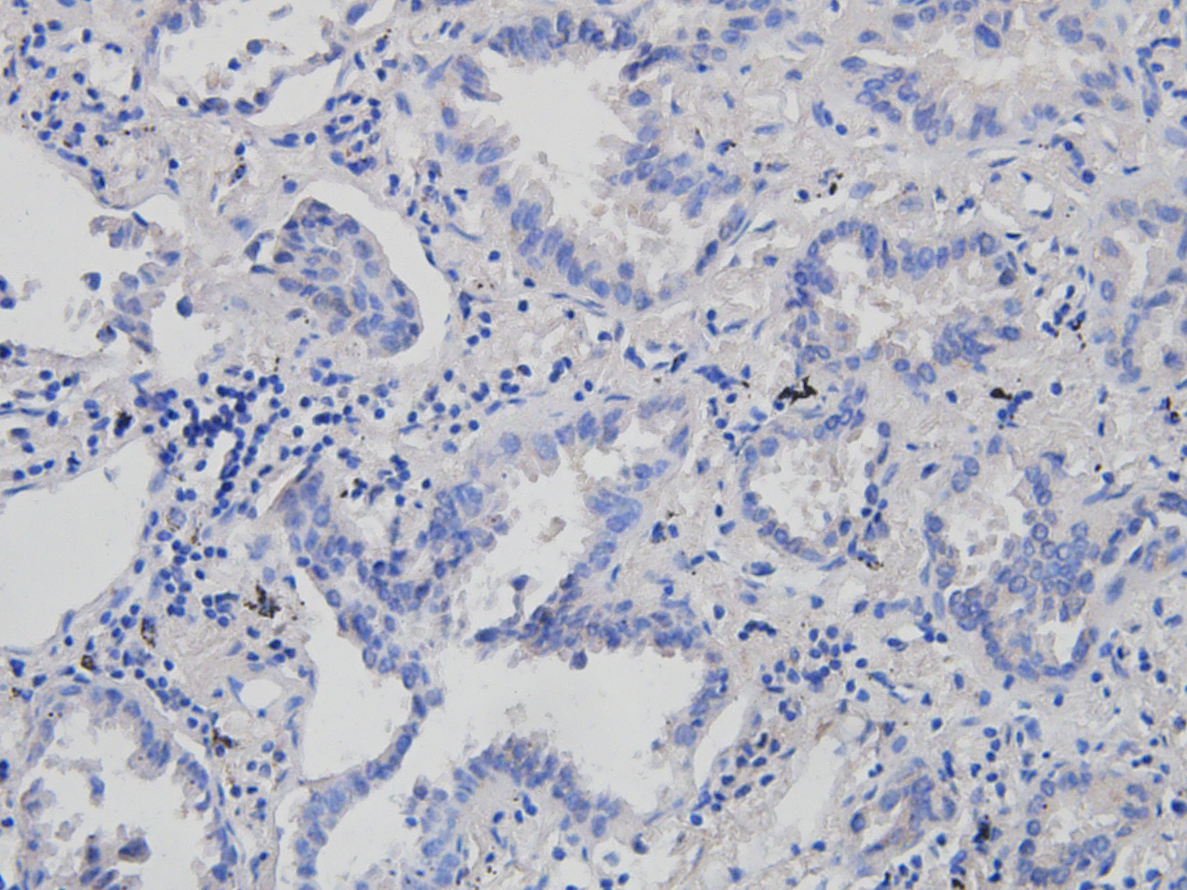

Supplement: S15 File — (ZIP) [file pone.0337223.s016.zip › 461991-400x-CA-N/461991-400x-CA (1).tif]

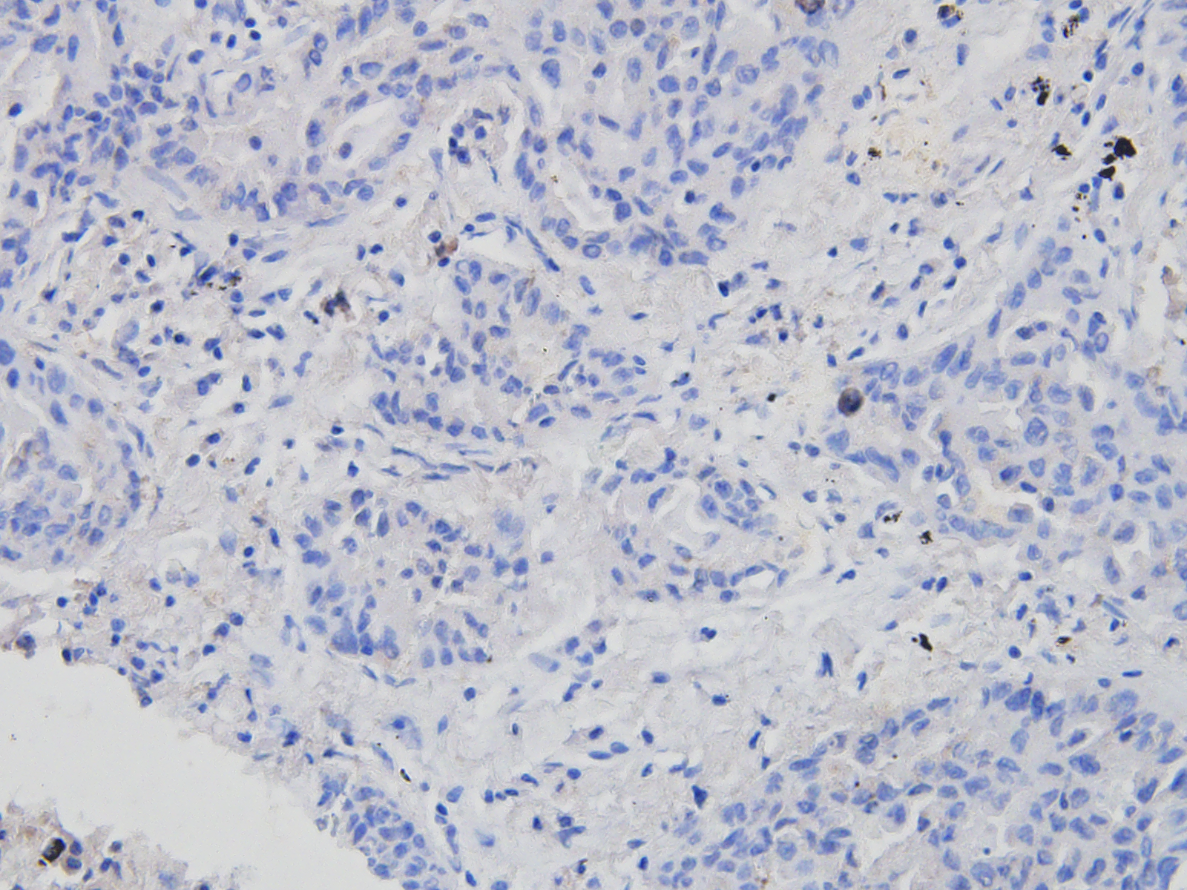

Supplement: S15 File — (ZIP) [file pone.0337223.s016.zip › 461991-400x-CA-N/461991-400x-CA (2).tif]

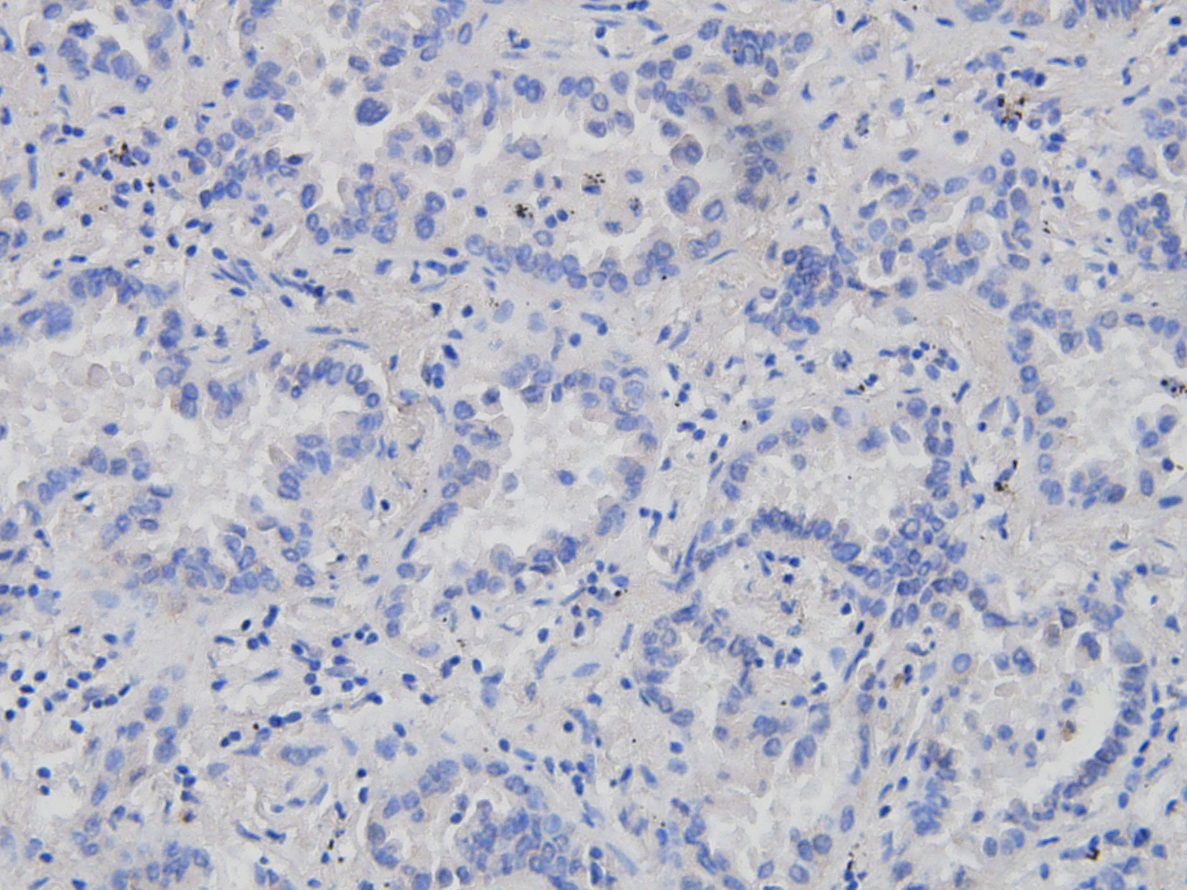

Supplement: S15 File — (ZIP) [file pone.0337223.s016.zip › 461991-400x-CA-N/461991-400x-CA (3).tif]

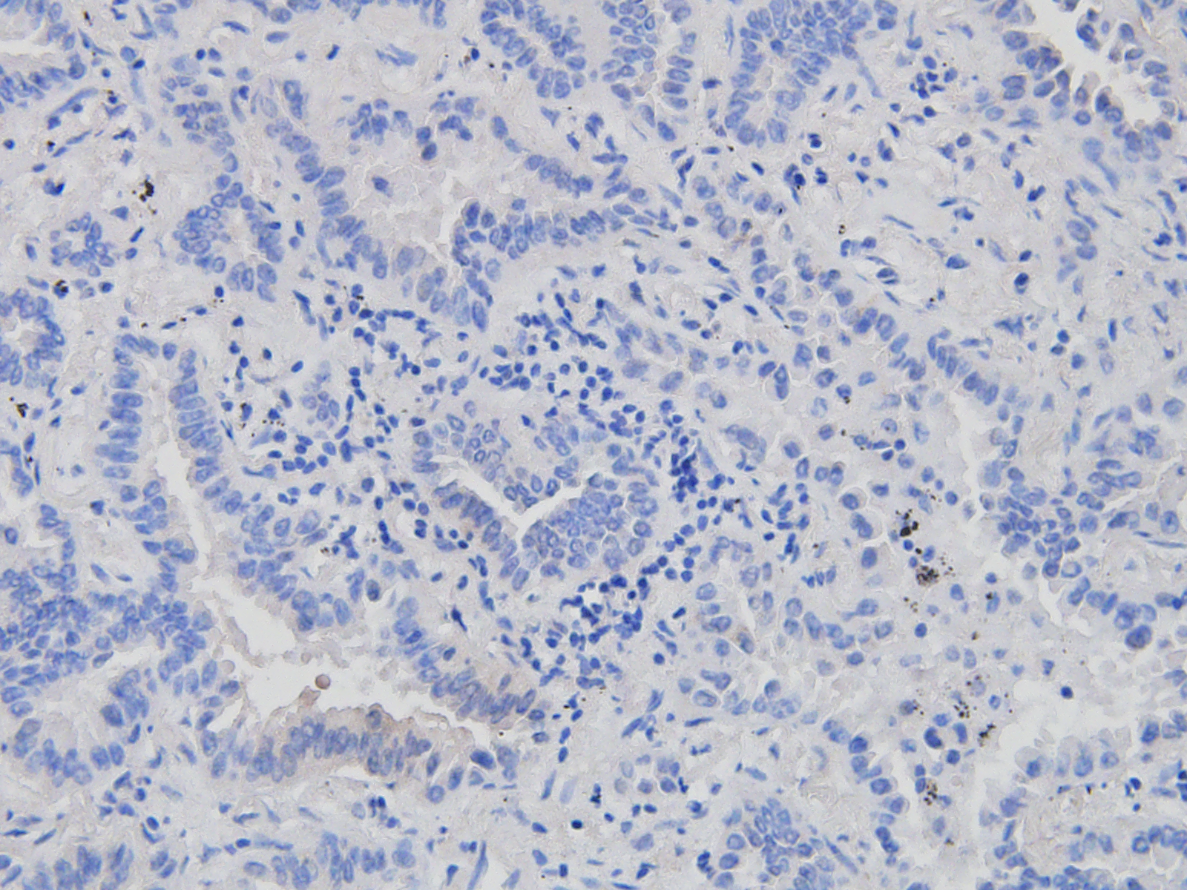

Supplement: S15 File — (ZIP) [file pone.0337223.s016.zip › 461991-400x-CA-N/461991-400x-CA (4).tif]

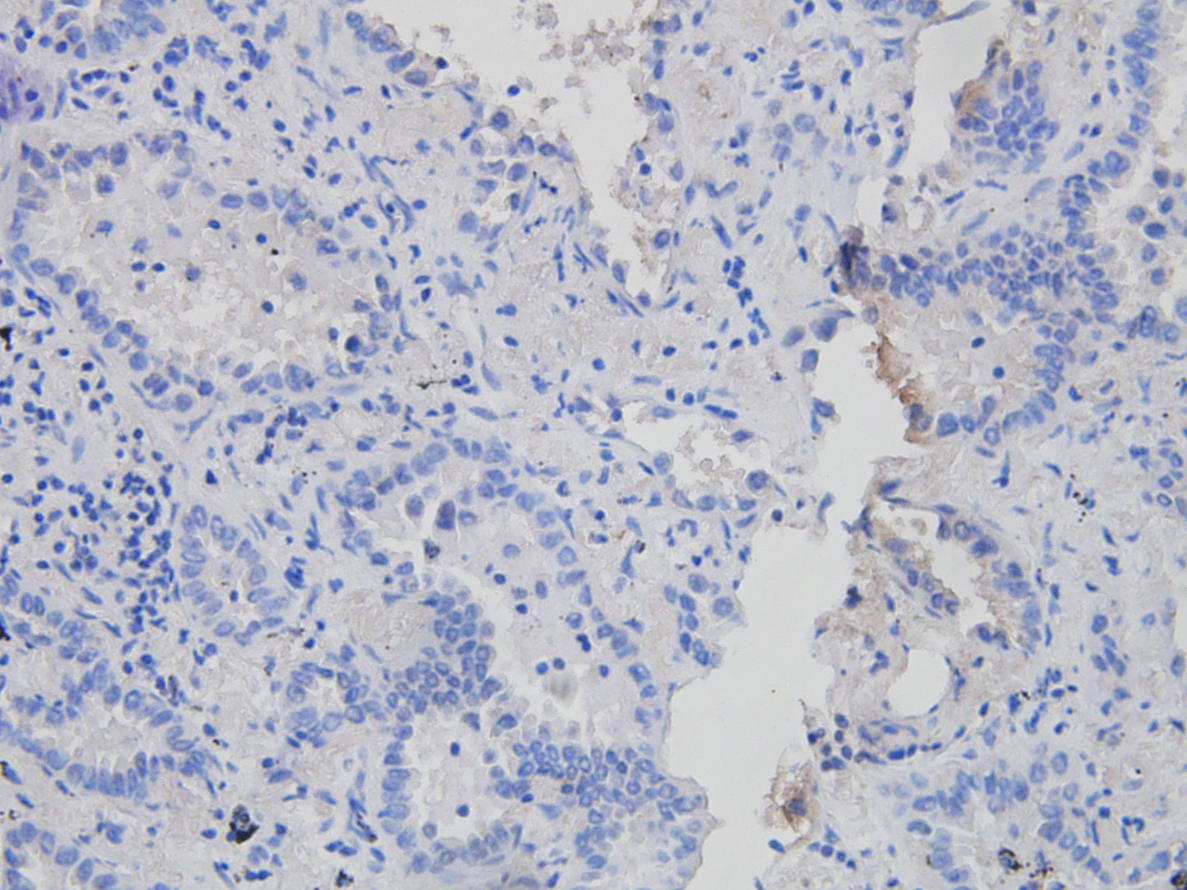

Supplement: S15 File — (ZIP) [file pone.0337223.s016.zip › 461991-400x-CA-N/461991-400x-CA (5).tif]

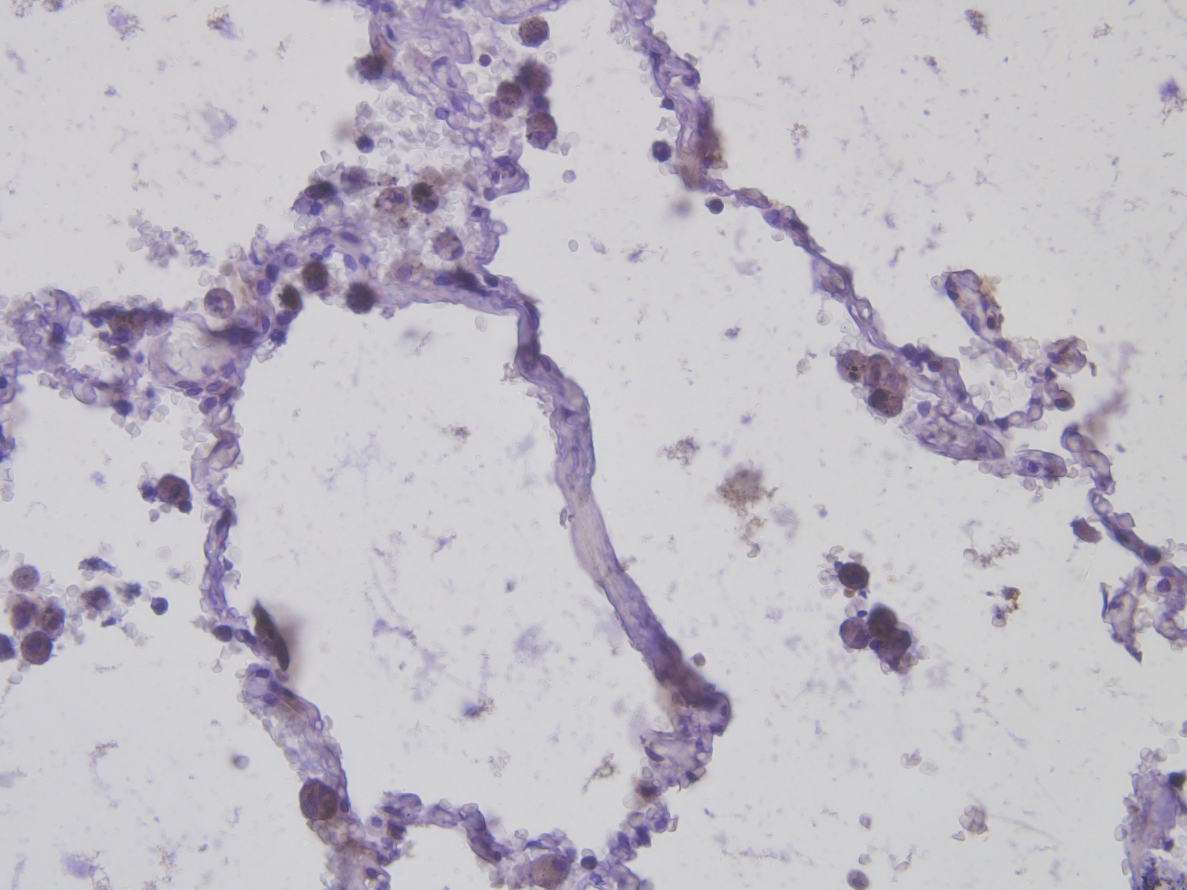

Supplement: S15 File — (ZIP) [file pone.0337223.s016.zip › 461991-400x-CA-N/461991-400x-N (1).tif]

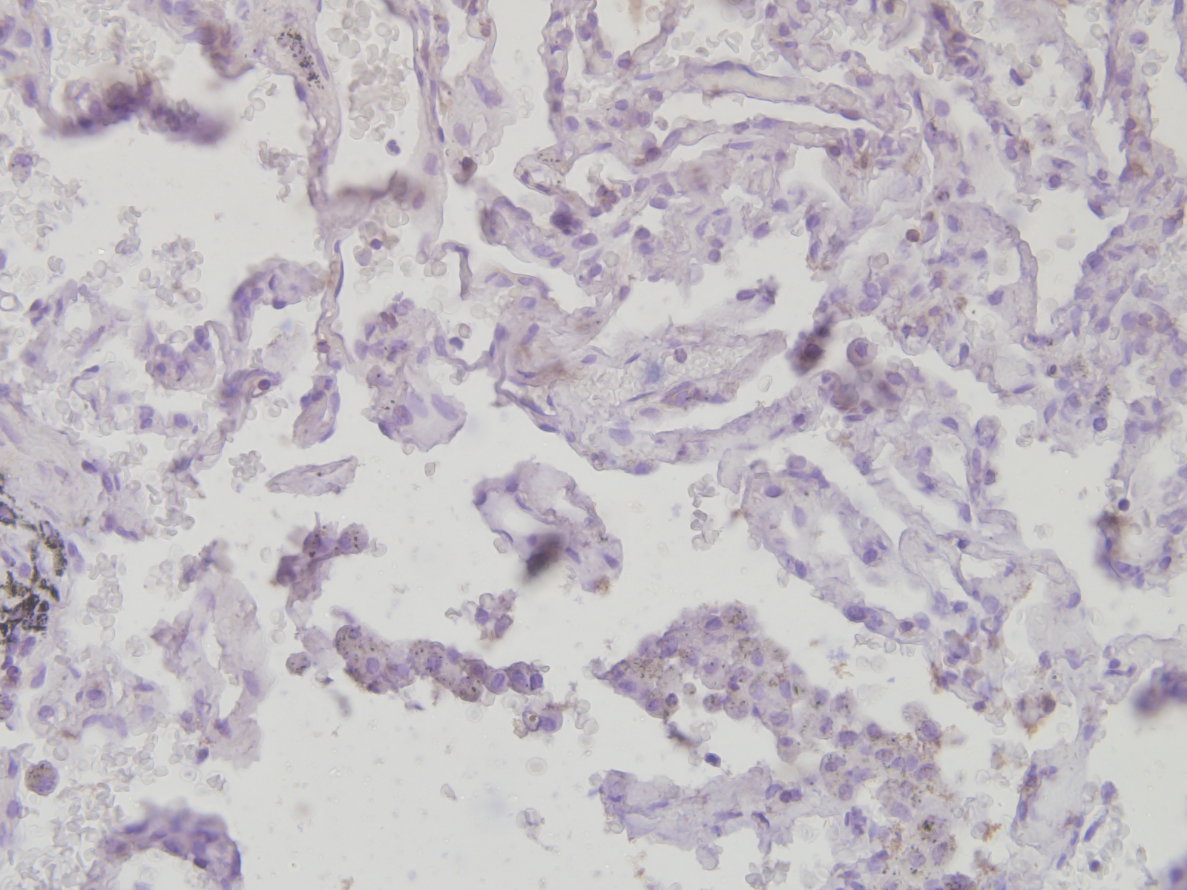

Supplement: S15 File — (ZIP) [file pone.0337223.s016.zip › 461991-400x-CA-N/461991-400x-N (2).tif]

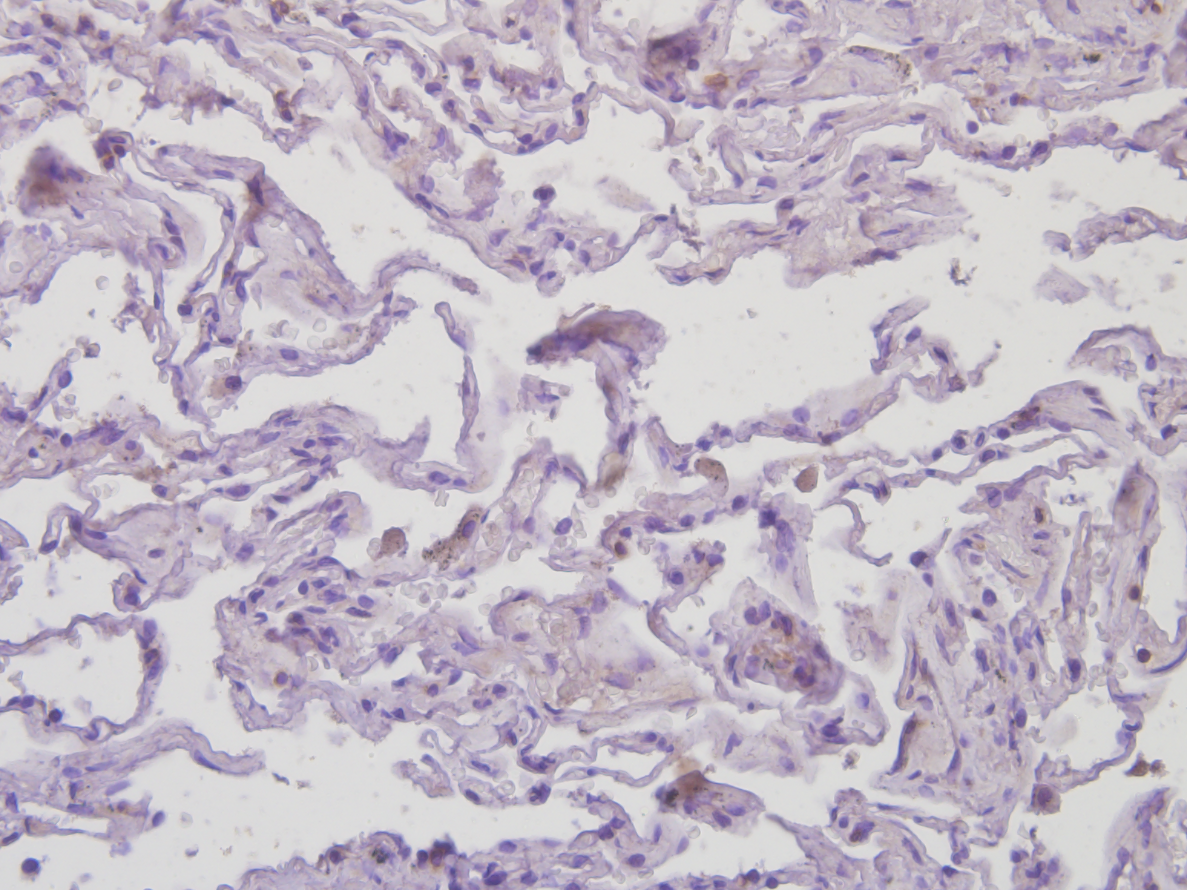

Supplement: S15 File — (ZIP) [file pone.0337223.s016.zip › 461991-400x-CA-N/461991-400x-N (3).tif]

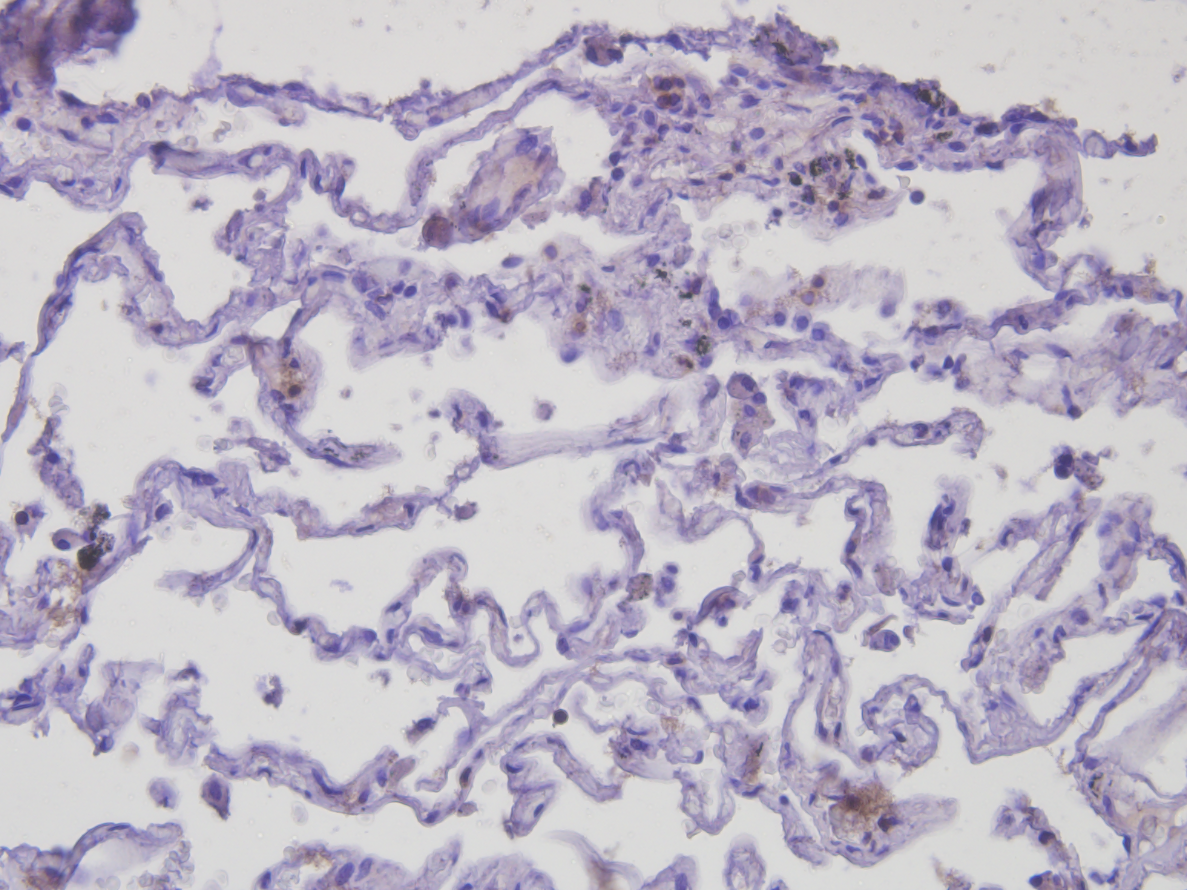

Supplement: S15 File — (ZIP) [file pone.0337223.s016.zip › 461991-400x-CA-N/461991-400x-N (4).tif]

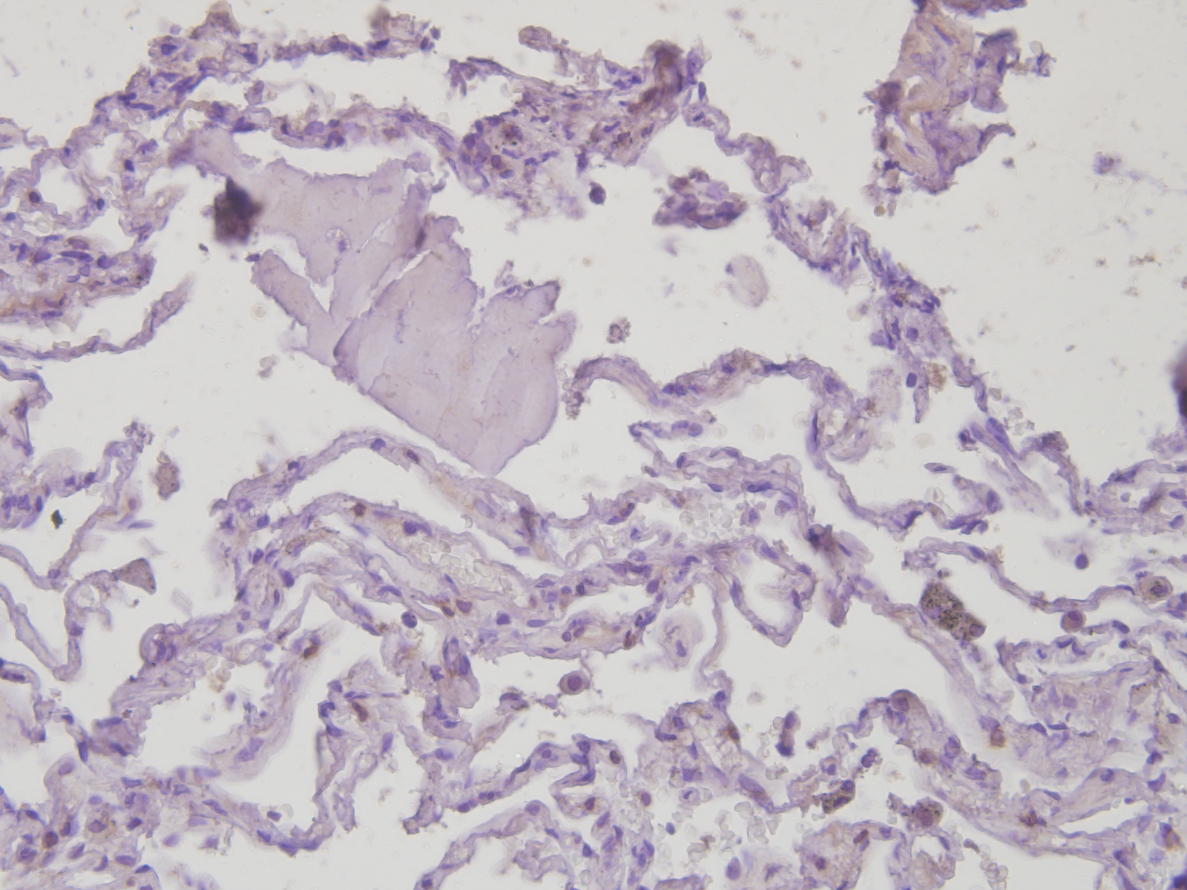

Supplement: S15 File — (ZIP) [file pone.0337223.s016.zip › 461991-400x-CA-N/461991-400x-N (5).tif]

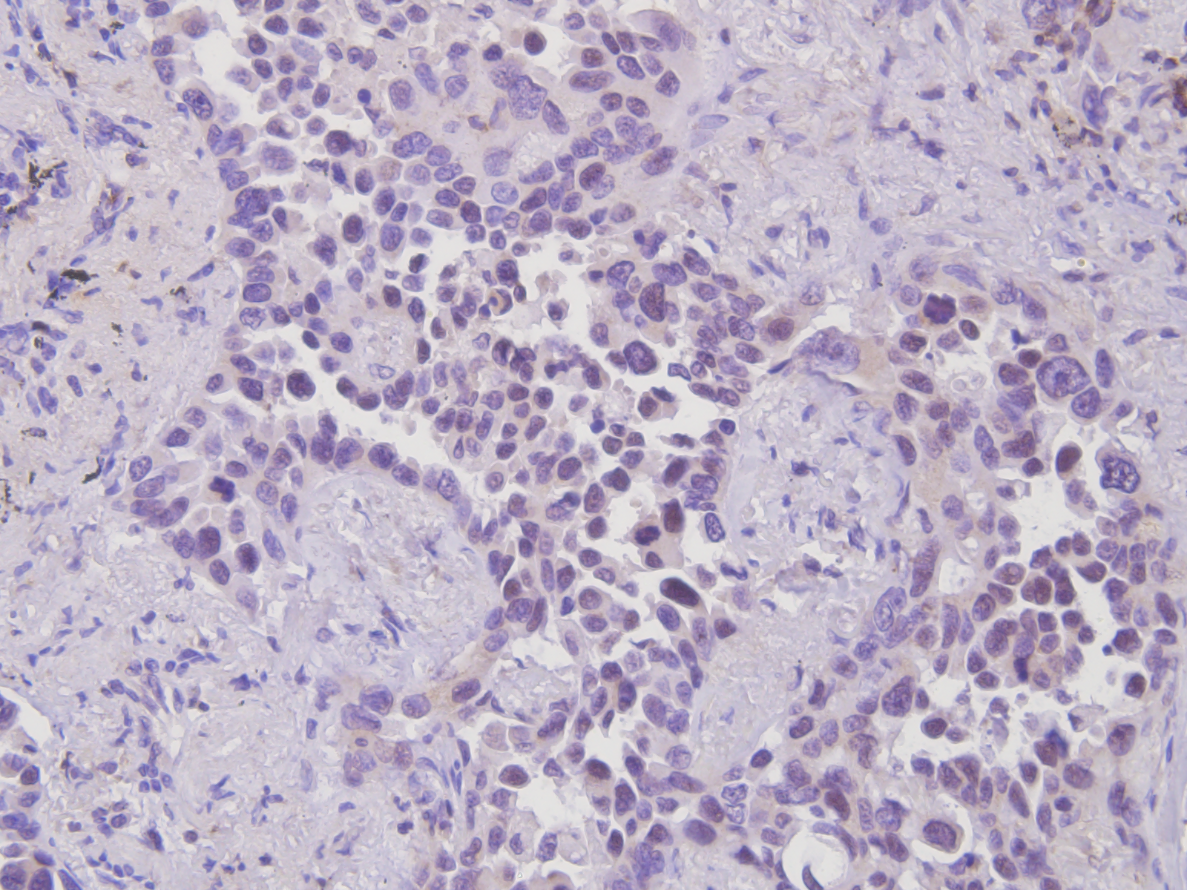

Supplement: S16 File — (ZIP) [file pone.0337223.s017.zip › 462473-400X-CA-N/462473-400X-CA (1).tif]

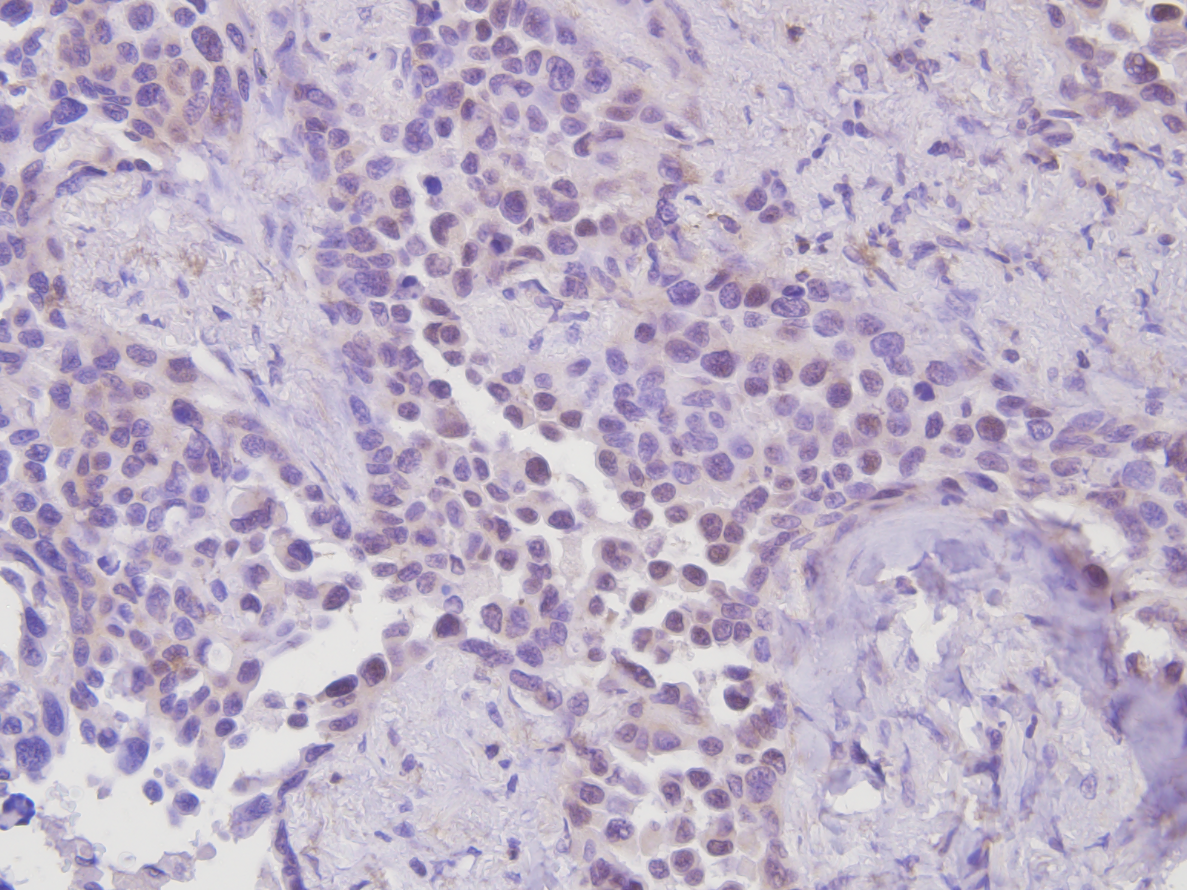

Supplement: S16 File — (ZIP) [file pone.0337223.s017.zip › 462473-400X-CA-N/462473-400X-CA (2).tif]

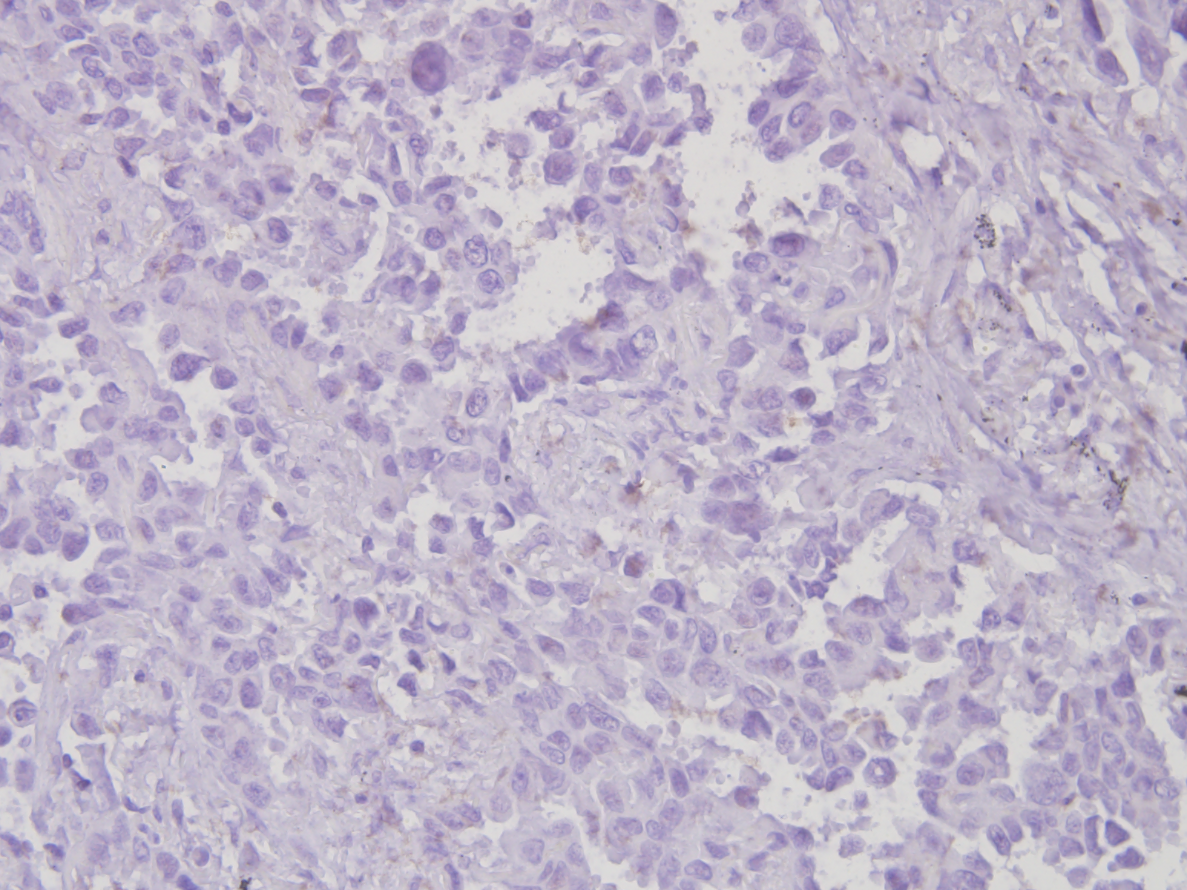

Supplement: S16 File — (ZIP) [file pone.0337223.s017.zip › 462473-400X-CA-N/462473-400X-CA (3).tif]

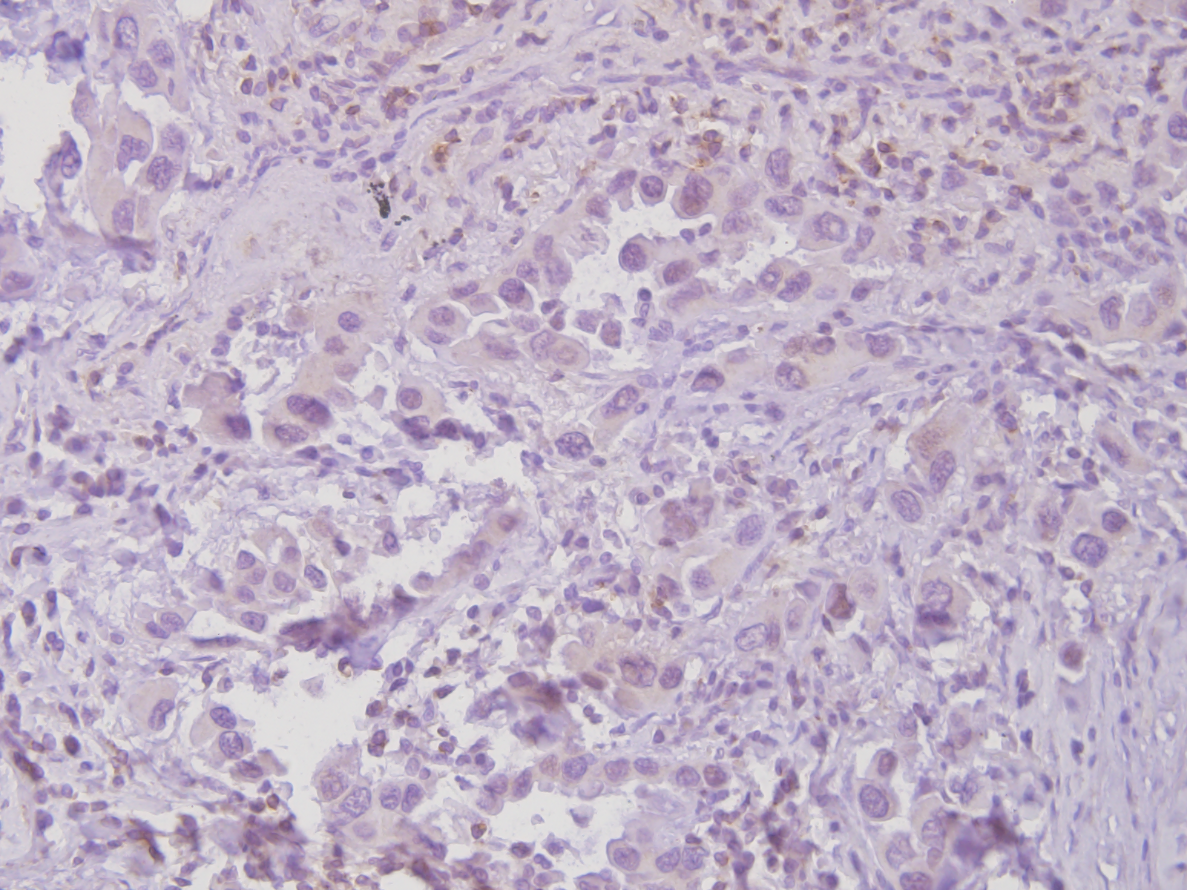

Supplement: S16 File — (ZIP) [file pone.0337223.s017.zip › 462473-400X-CA-N/462473-400X-CA (4).tif]

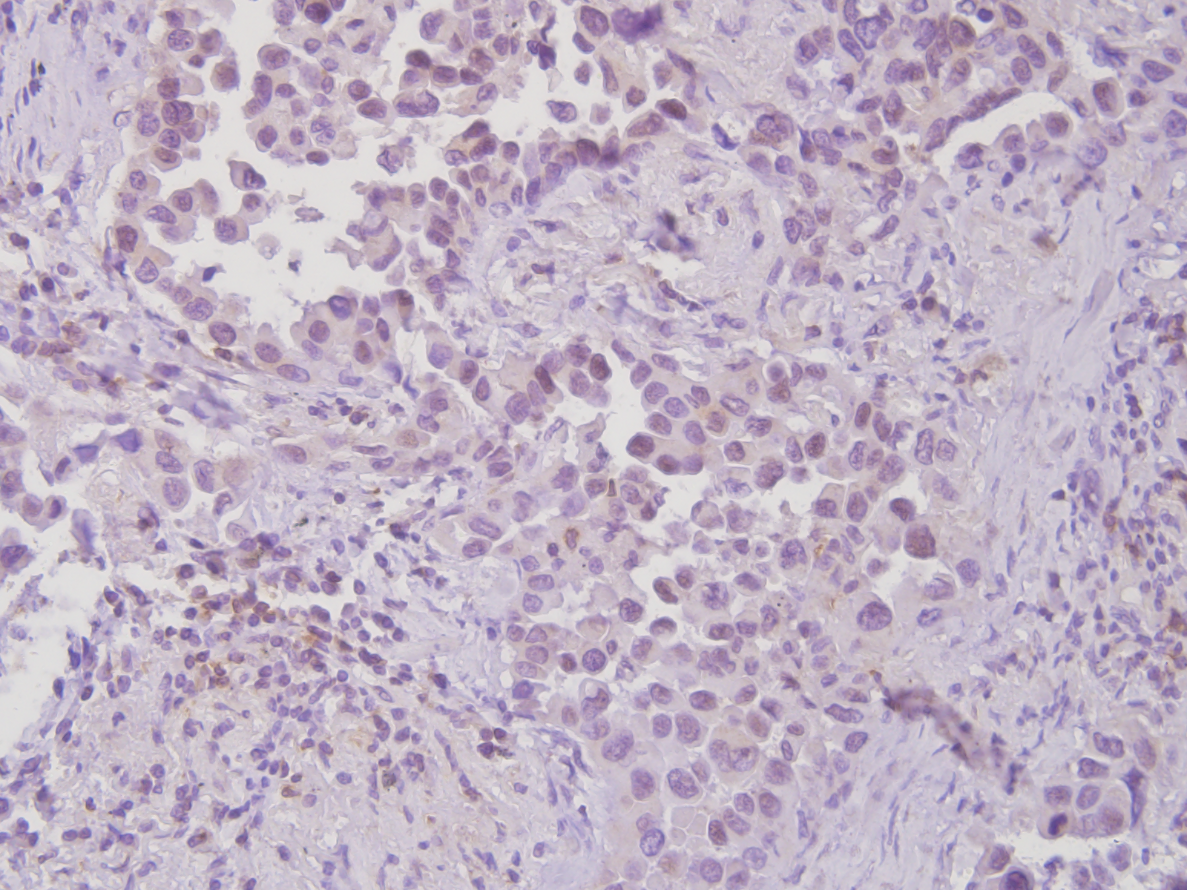

Supplement: S16 File — (ZIP) [file pone.0337223.s017.zip › 462473-400X-CA-N/462473-400X-CA (5).tif]

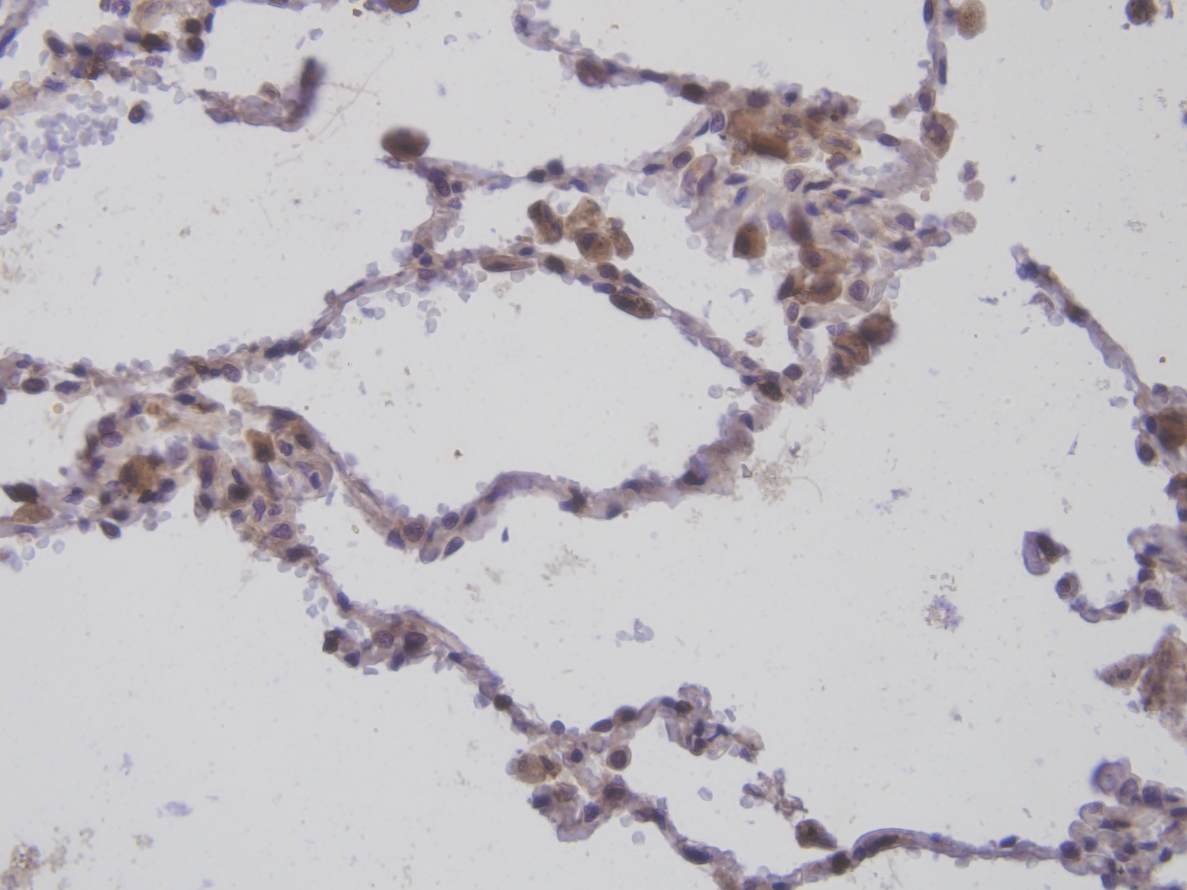

Supplement: S16 File — (ZIP) [file pone.0337223.s017.zip › 462473-400X-CA-N/462473-400X-N (1).tif]

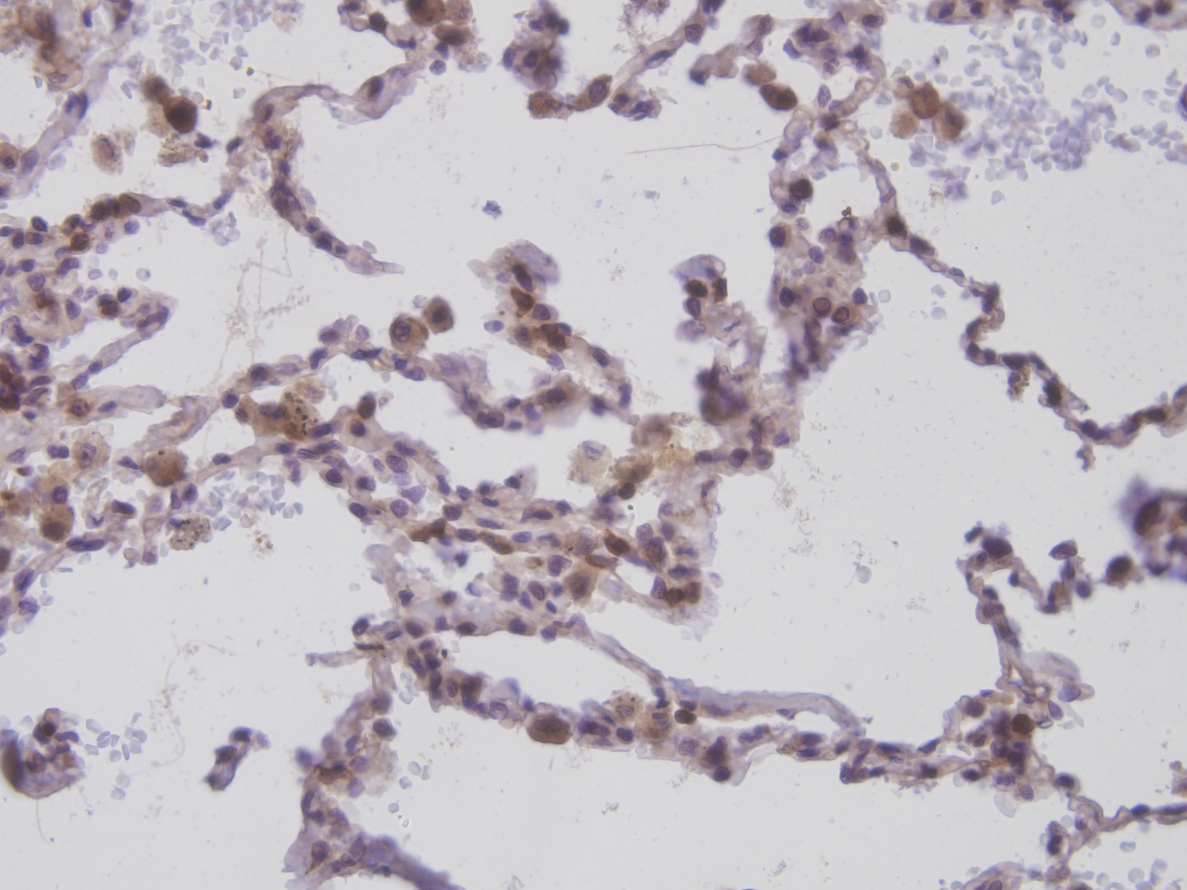

Supplement: S16 File — (ZIP) [file pone.0337223.s017.zip › 462473-400X-CA-N/462473-400X-N (2).tif]

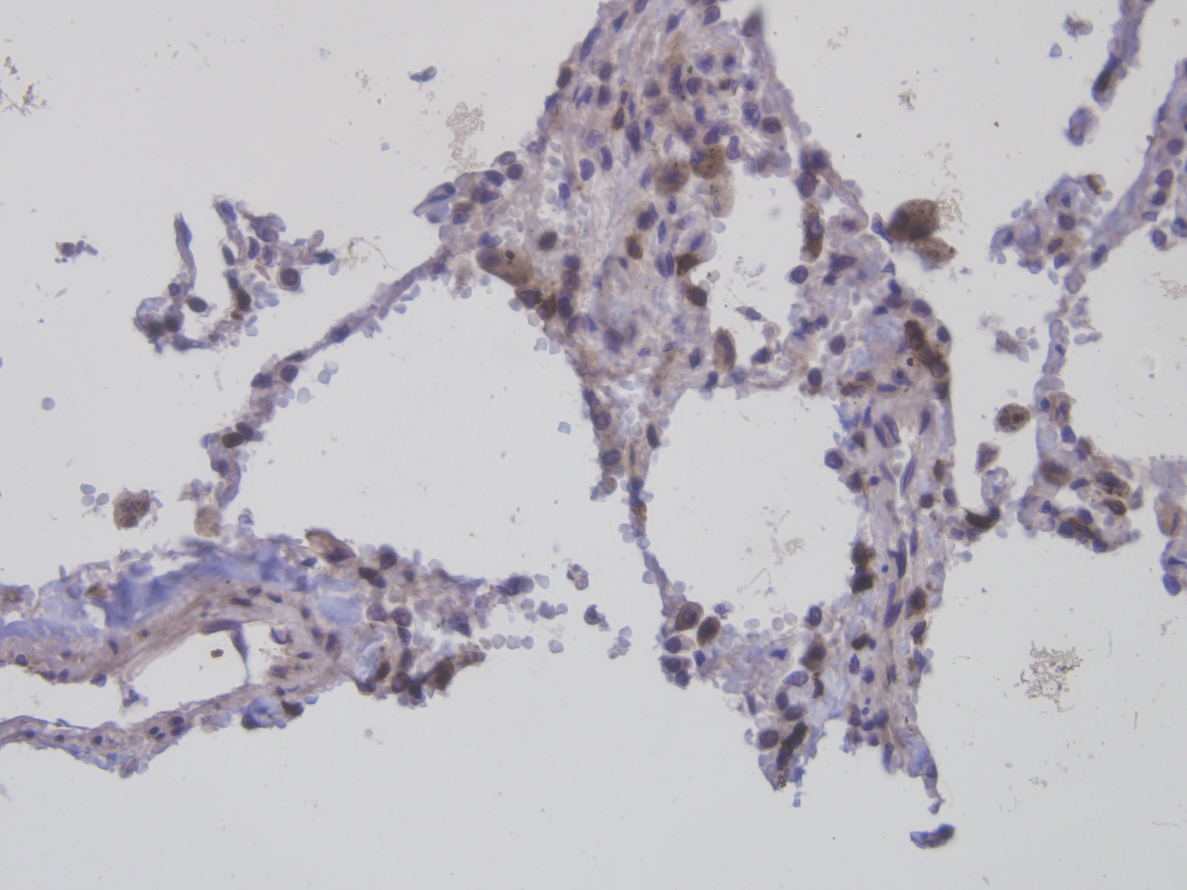

Supplement: S16 File — (ZIP) [file pone.0337223.s017.zip › 462473-400X-CA-N/462473-400X-N (3).tif]

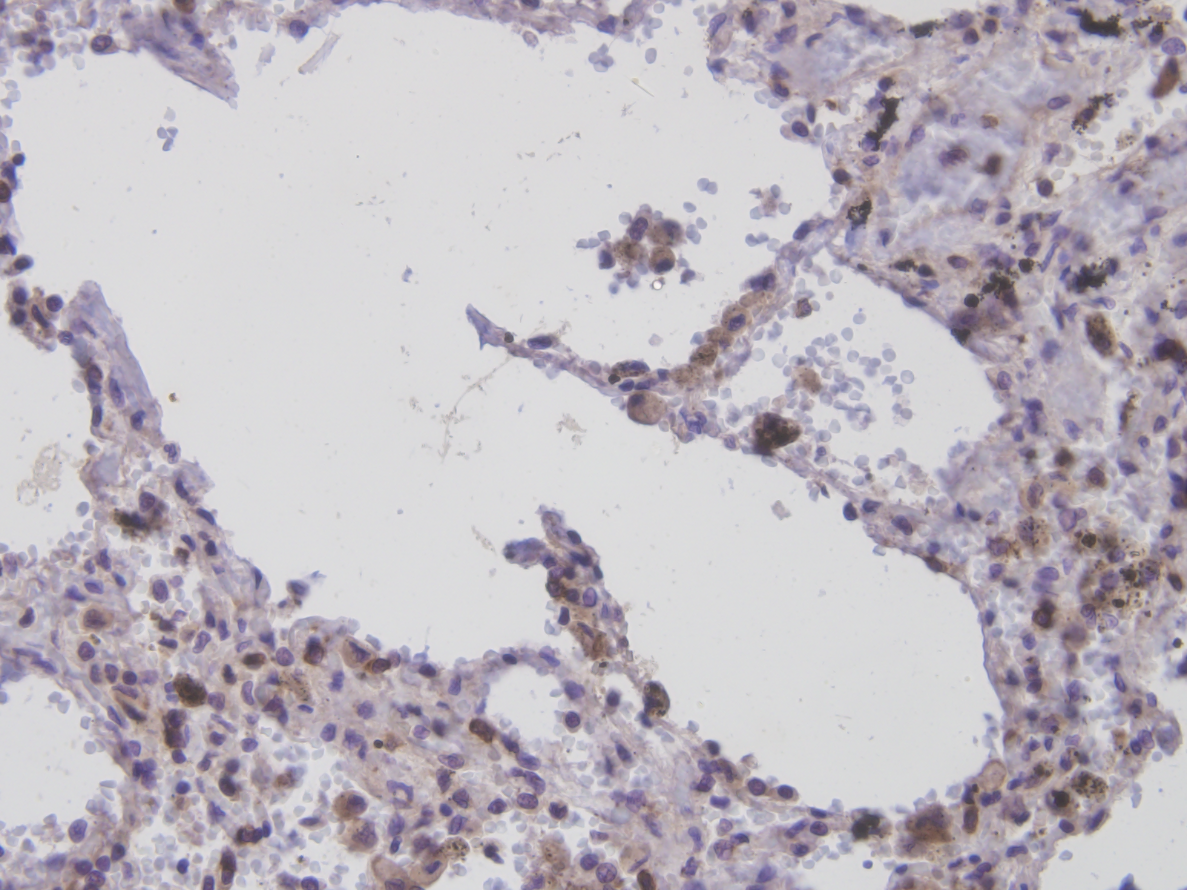

Supplement: S16 File — (ZIP) [file pone.0337223.s017.zip › 462473-400X-CA-N/462473-400X-N (4).tif]

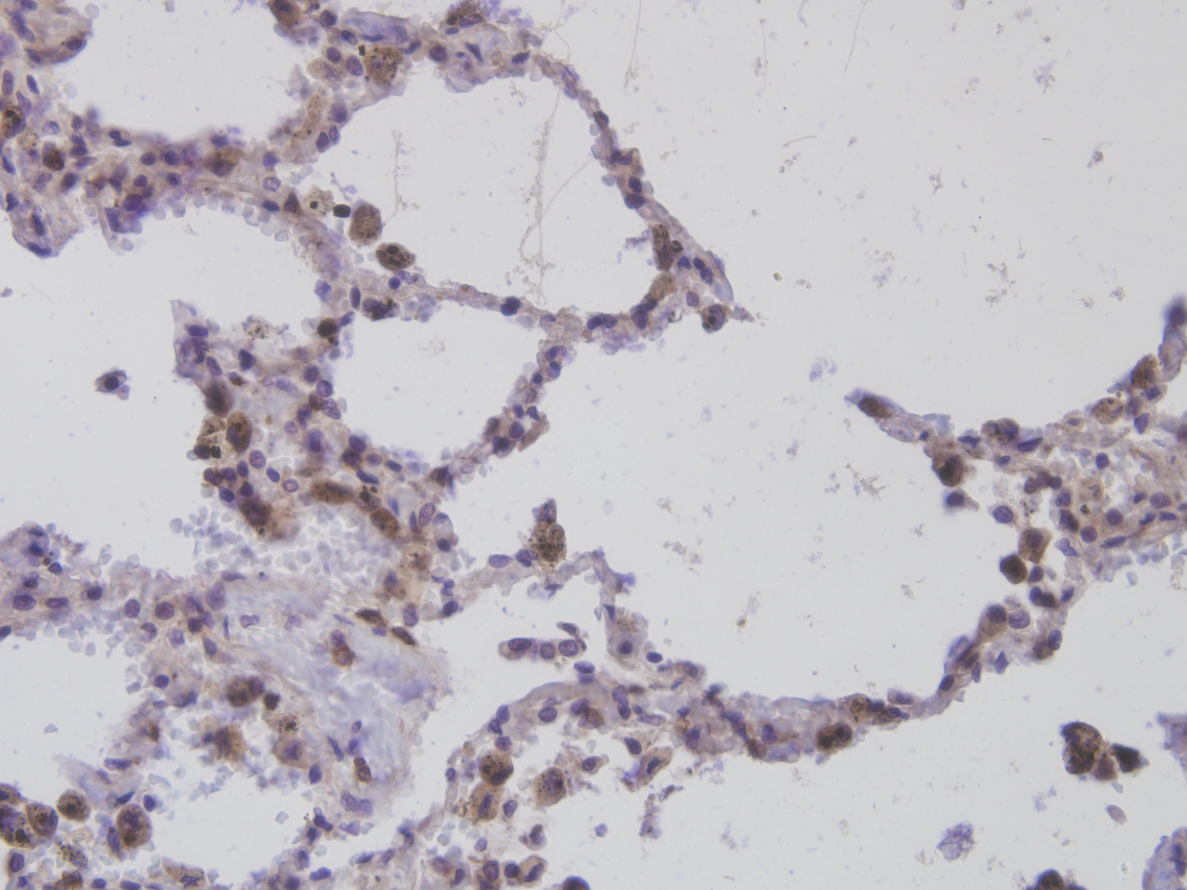

Supplement: S16 File — (ZIP) [file pone.0337223.s017.zip › 462473-400X-CA-N/462473-400X-N (5).tif]

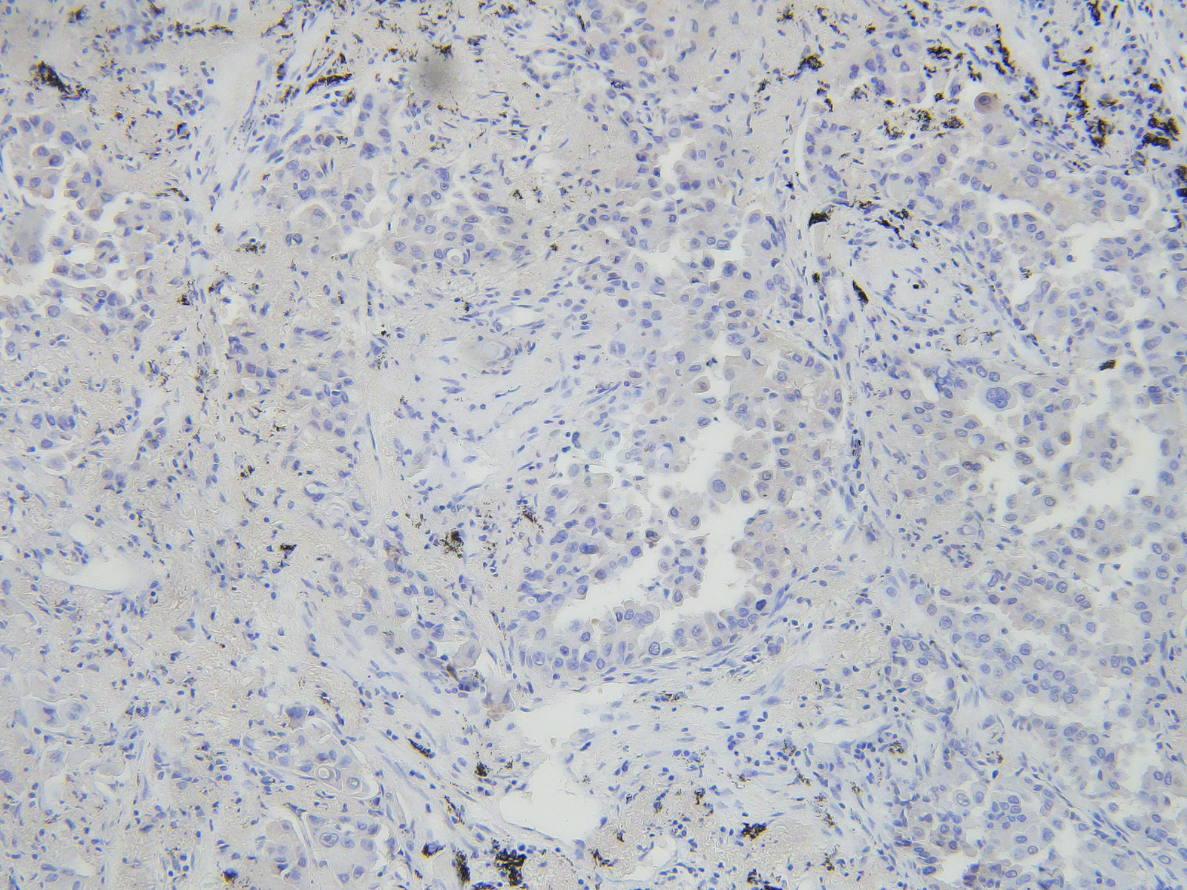

Supplement: S17 File — (ZIP) [file pone.0337223.s018.zip › 462603-400X-N-CA-/462603-400X-CA (1).tif]

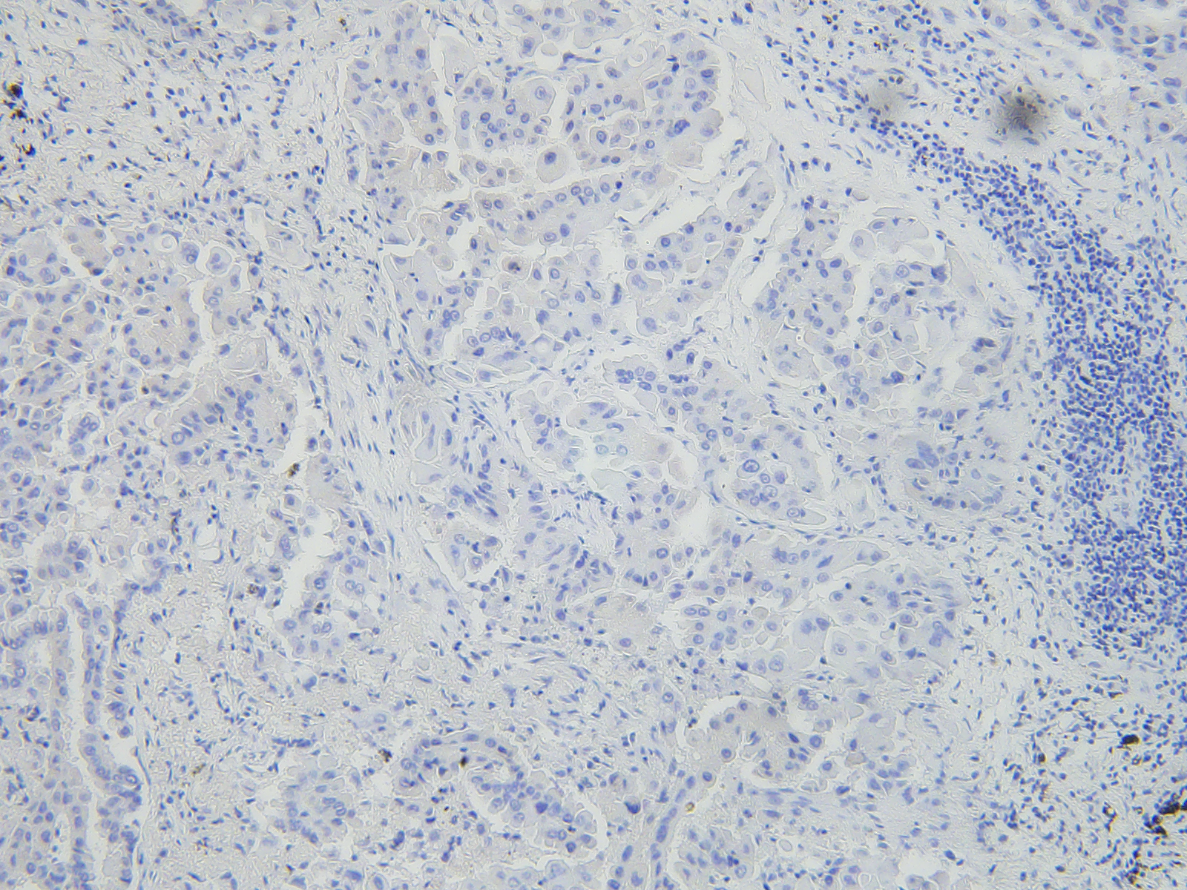

Supplement: S17 File — (ZIP) [file pone.0337223.s018.zip › 462603-400X-N-CA-/462603-400X-CA (2).tif]

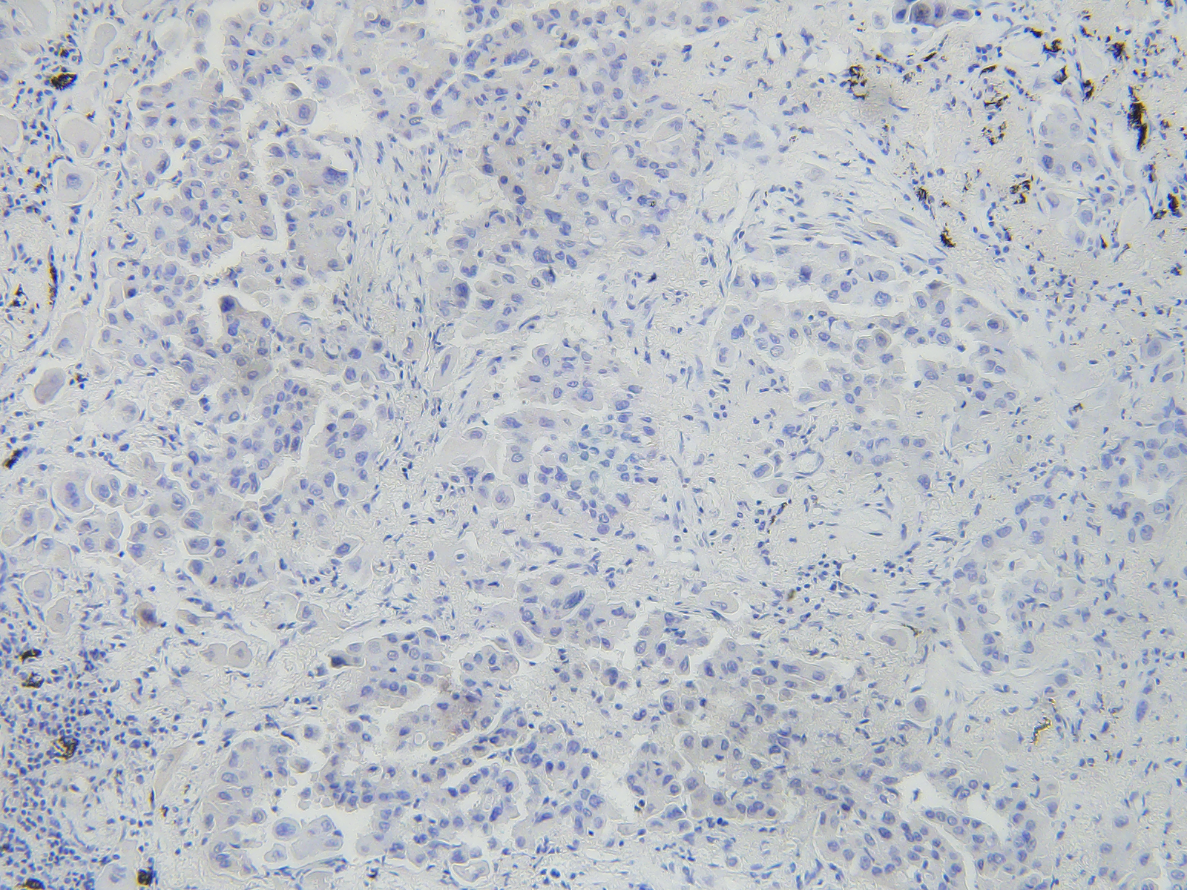

Supplement: S17 File — (ZIP) [file pone.0337223.s018.zip › 462603-400X-N-CA-/462603-400X-CA (3).tif]

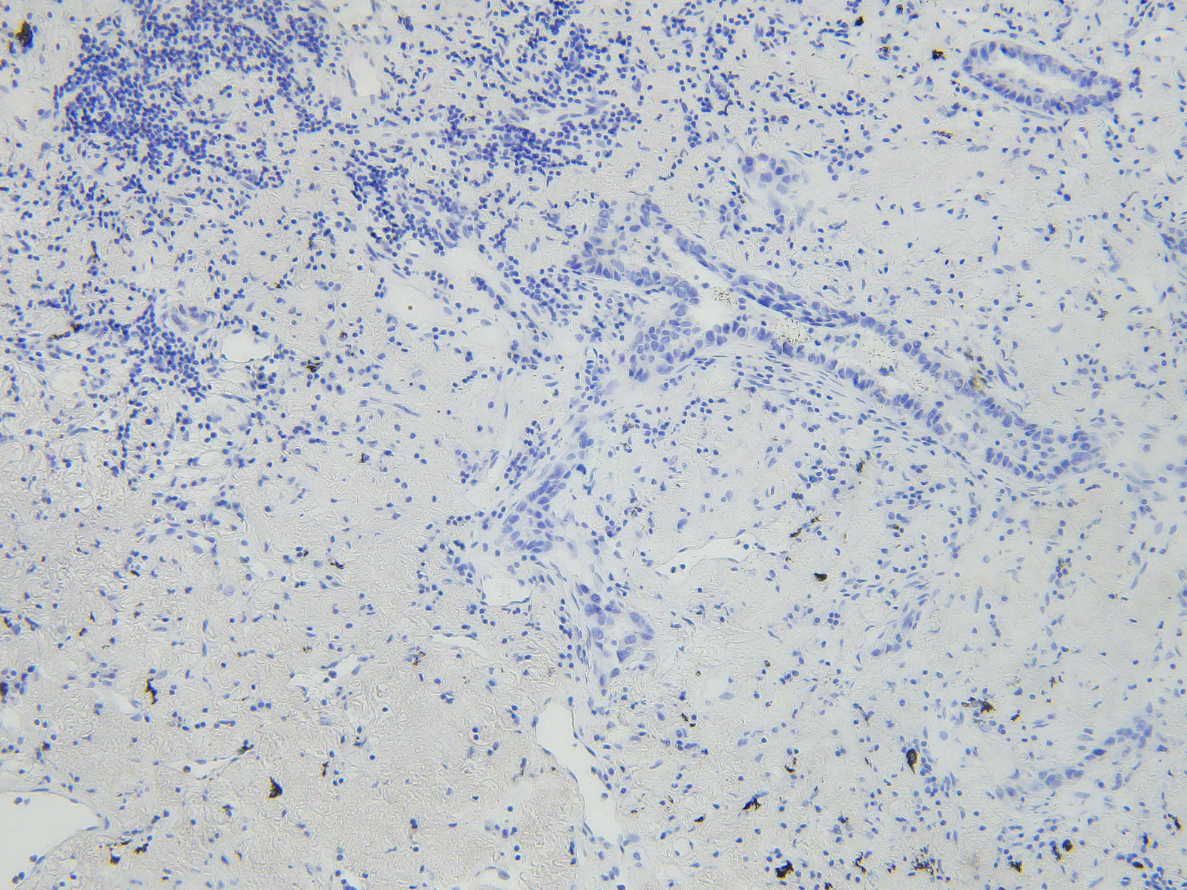

Supplement: S17 File — (ZIP) [file pone.0337223.s018.zip › 462603-400X-N-CA-/462603-400X-CA (4).tif]

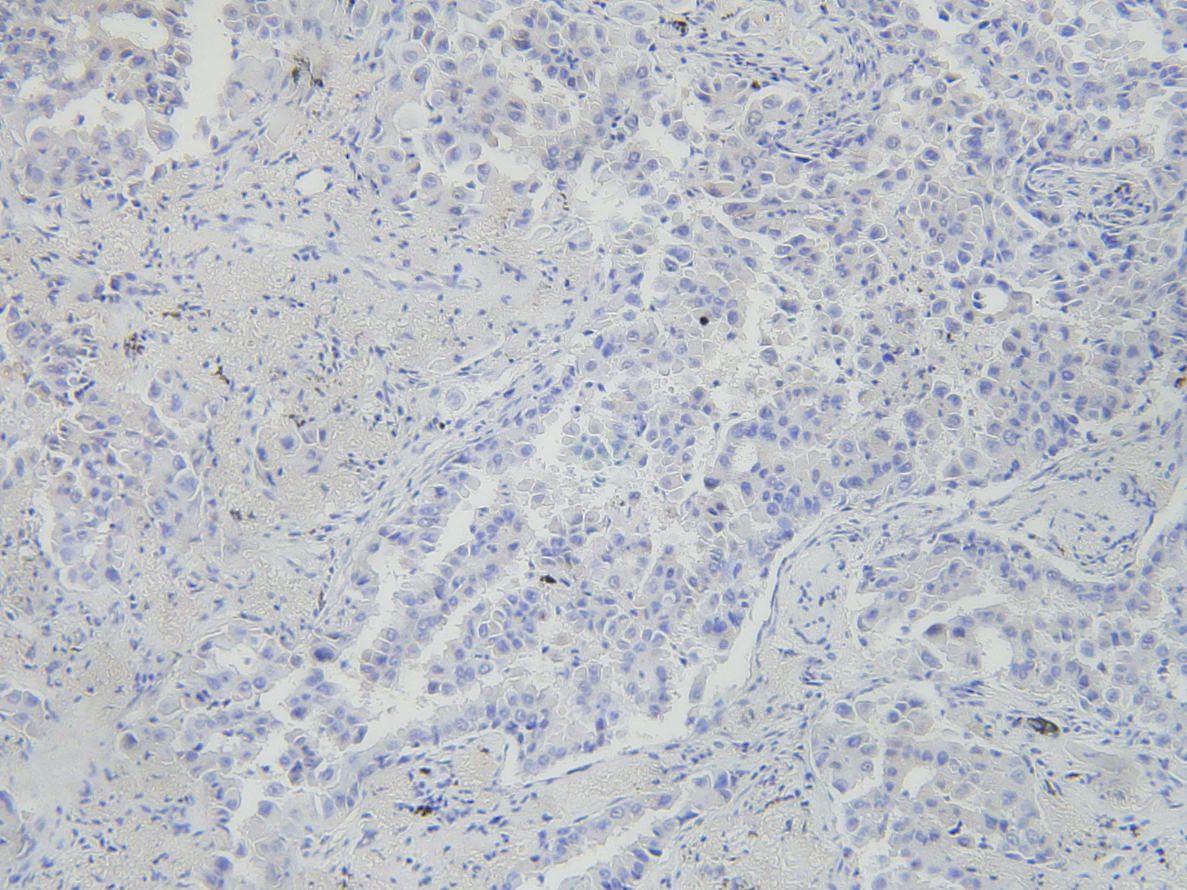

Supplement: S17 File — (ZIP) [file pone.0337223.s018.zip › 462603-400X-N-CA-/462603-400X-CA (5).tif]

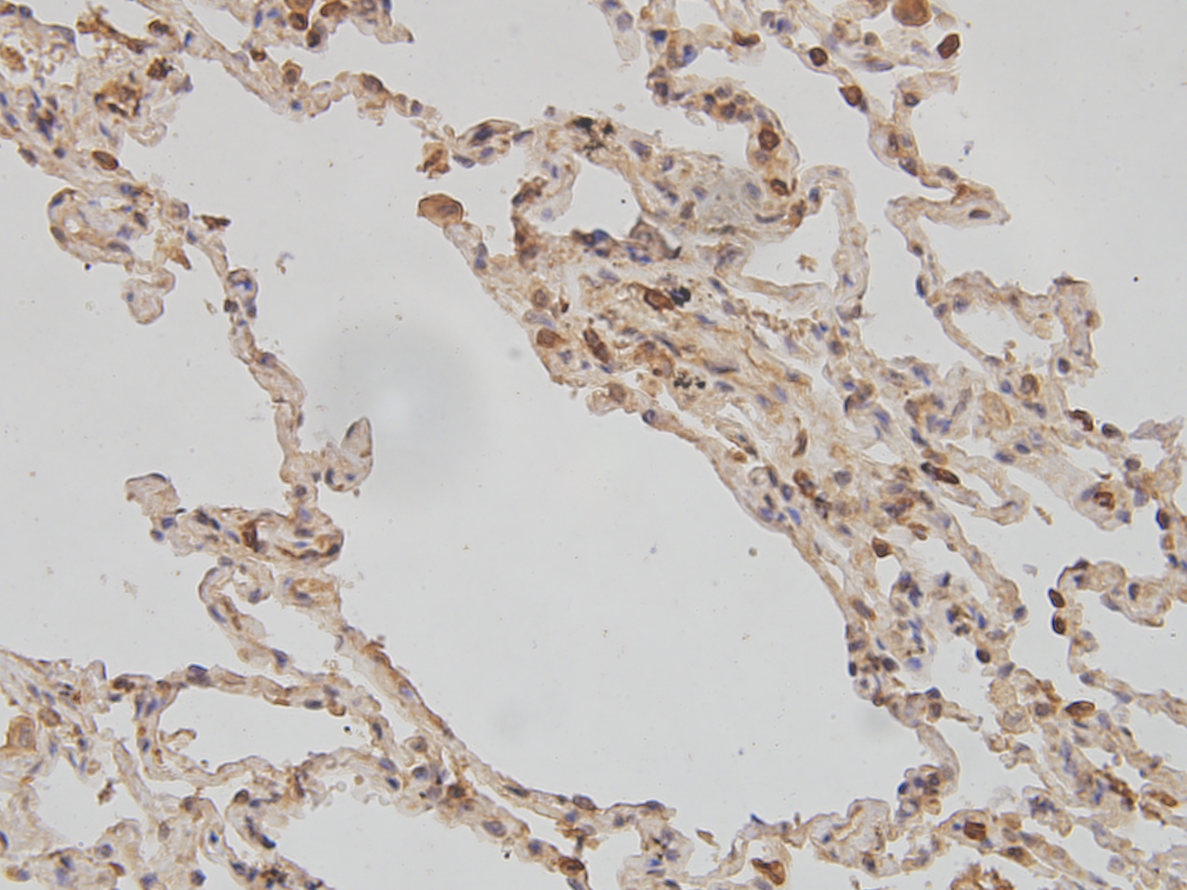

Supplement: S17 File — (ZIP) [file pone.0337223.s018.zip › 462603-400X-N-CA-/462603-400X-N (1).tif]

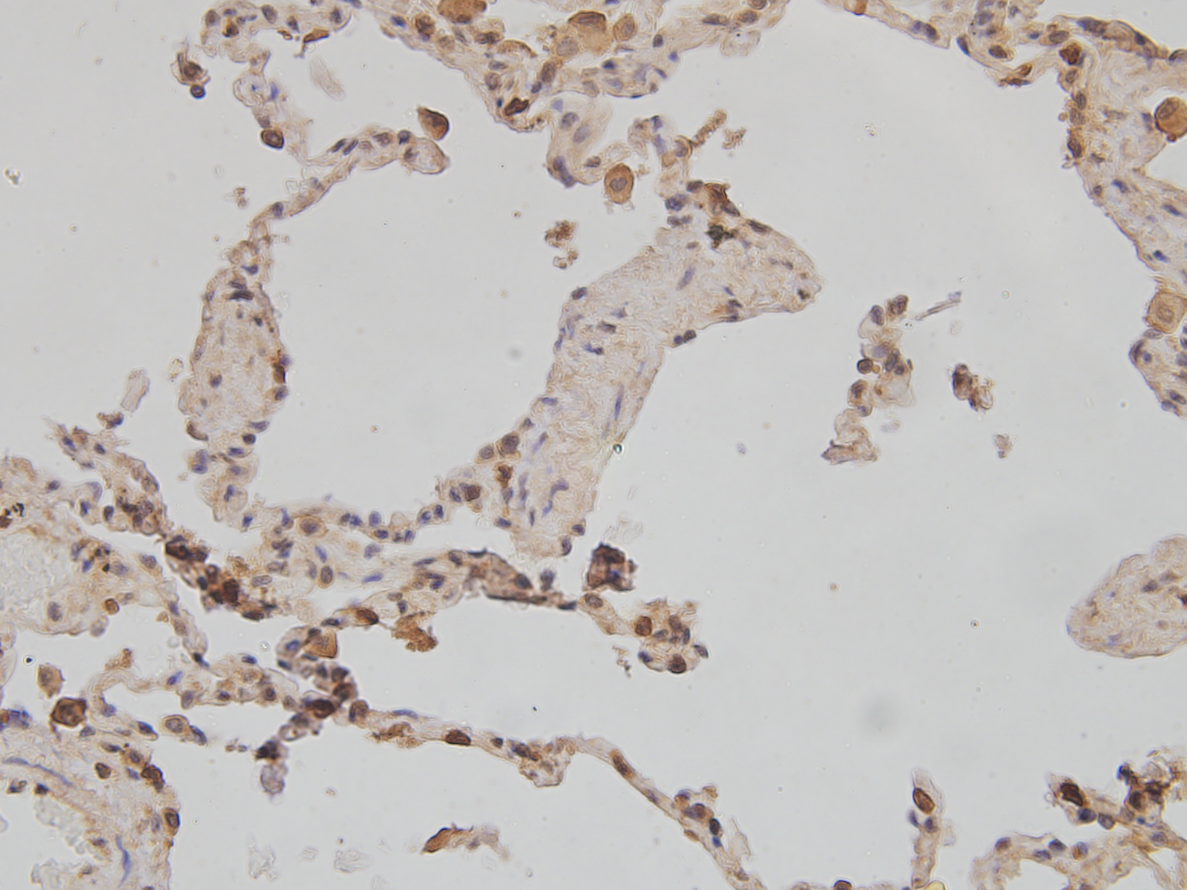

Supplement: S17 File — (ZIP) [file pone.0337223.s018.zip › 462603-400X-N-CA-/462603-400X-N (2).tif]

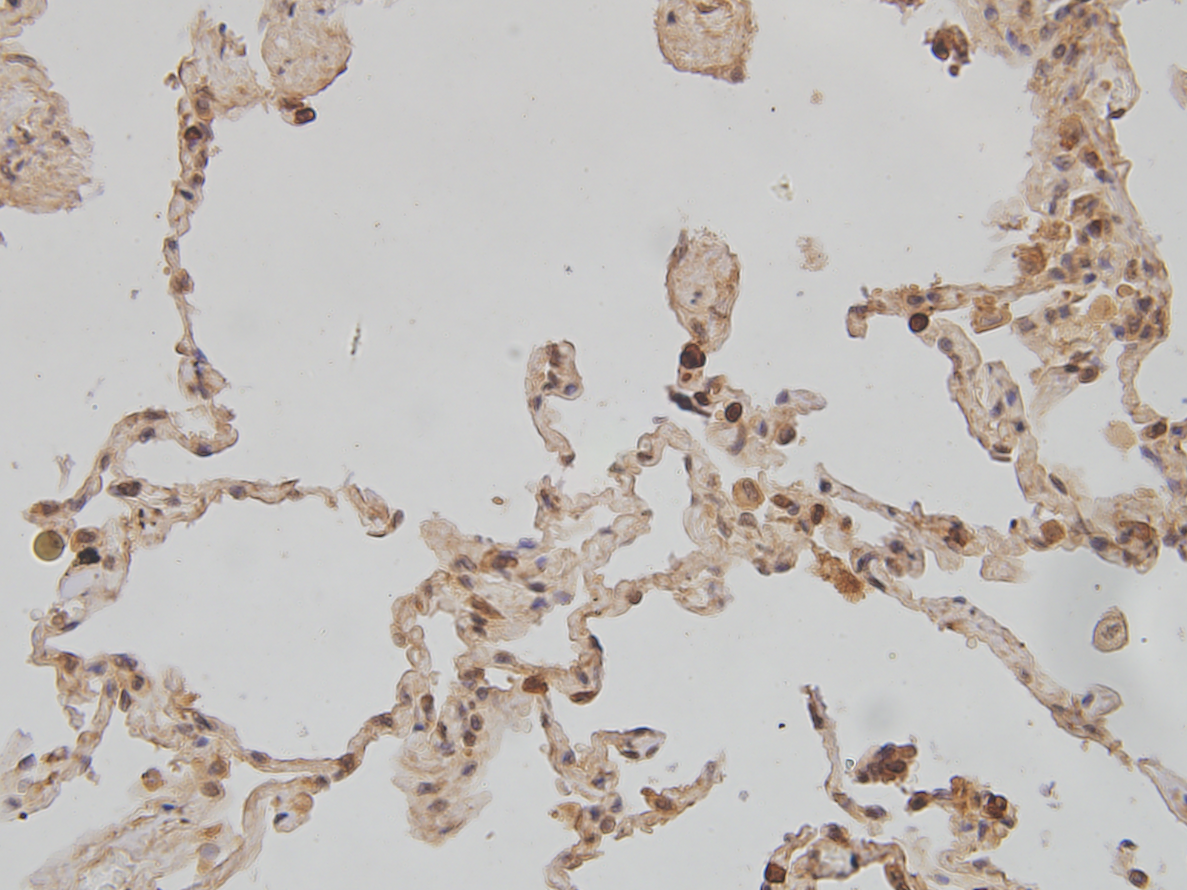

Supplement: S17 File — (ZIP) [file pone.0337223.s018.zip › 462603-400X-N-CA-/462603-400X-N (3).tif]

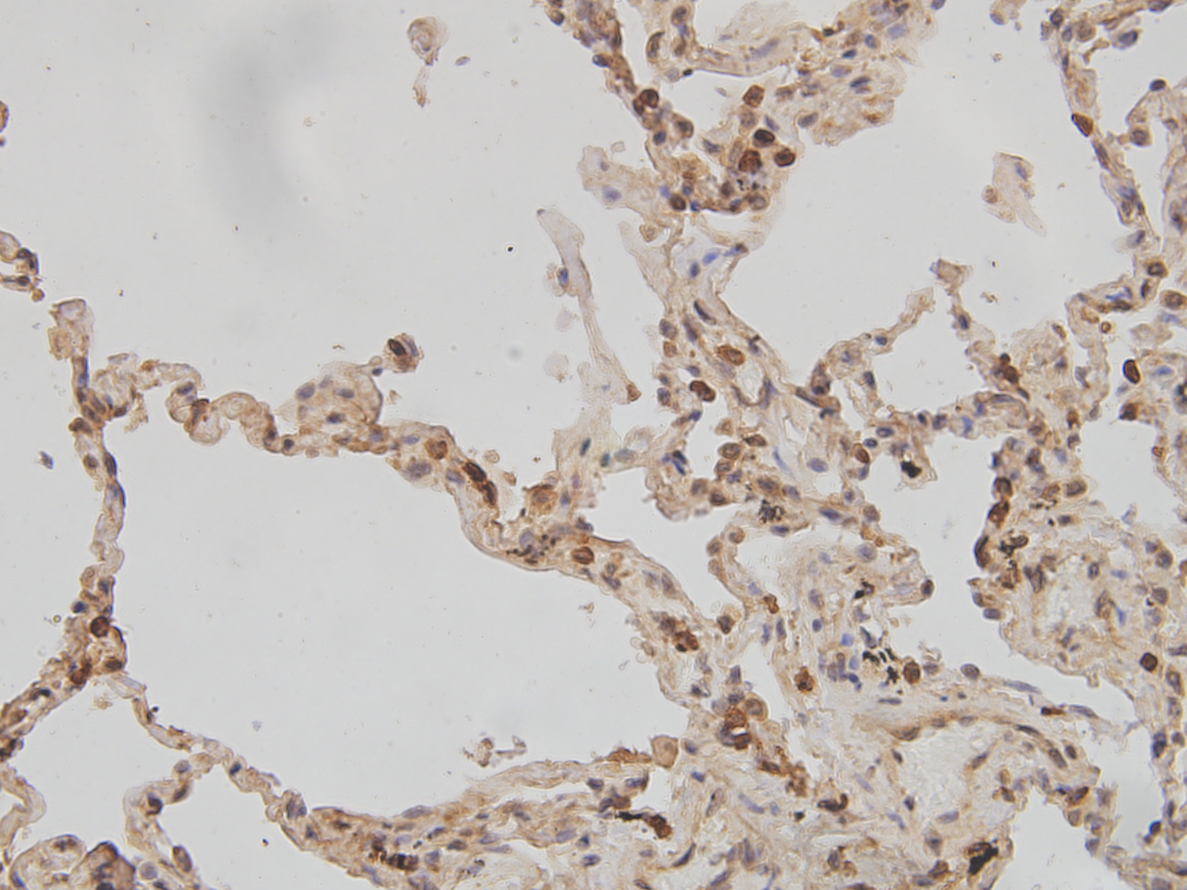

Supplement: S17 File — (ZIP) [file pone.0337223.s018.zip › 462603-400X-N-CA-/462603-400X-N (4).tif]

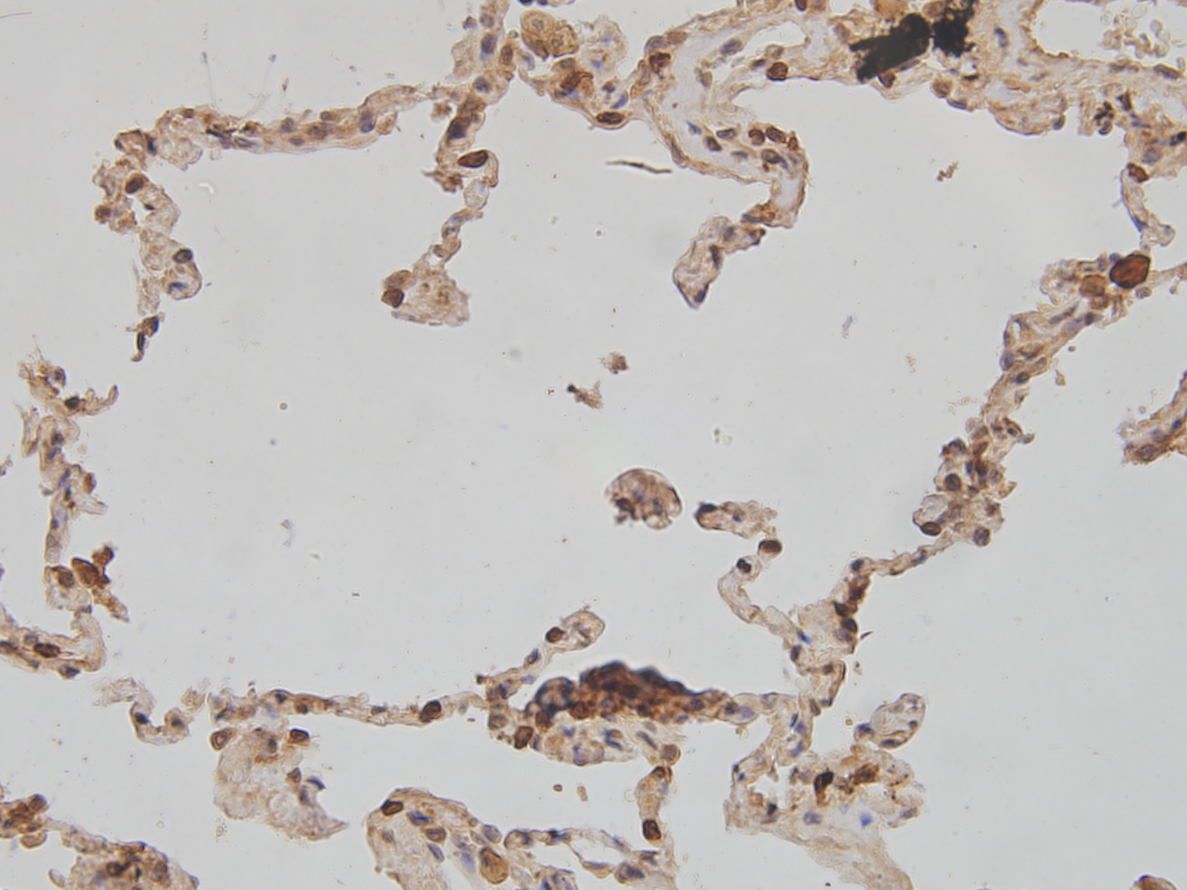

Supplement: S17 File — (ZIP) [file pone.0337223.s018.zip › 462603-400X-N-CA-/462603-400X-N (5).tif]

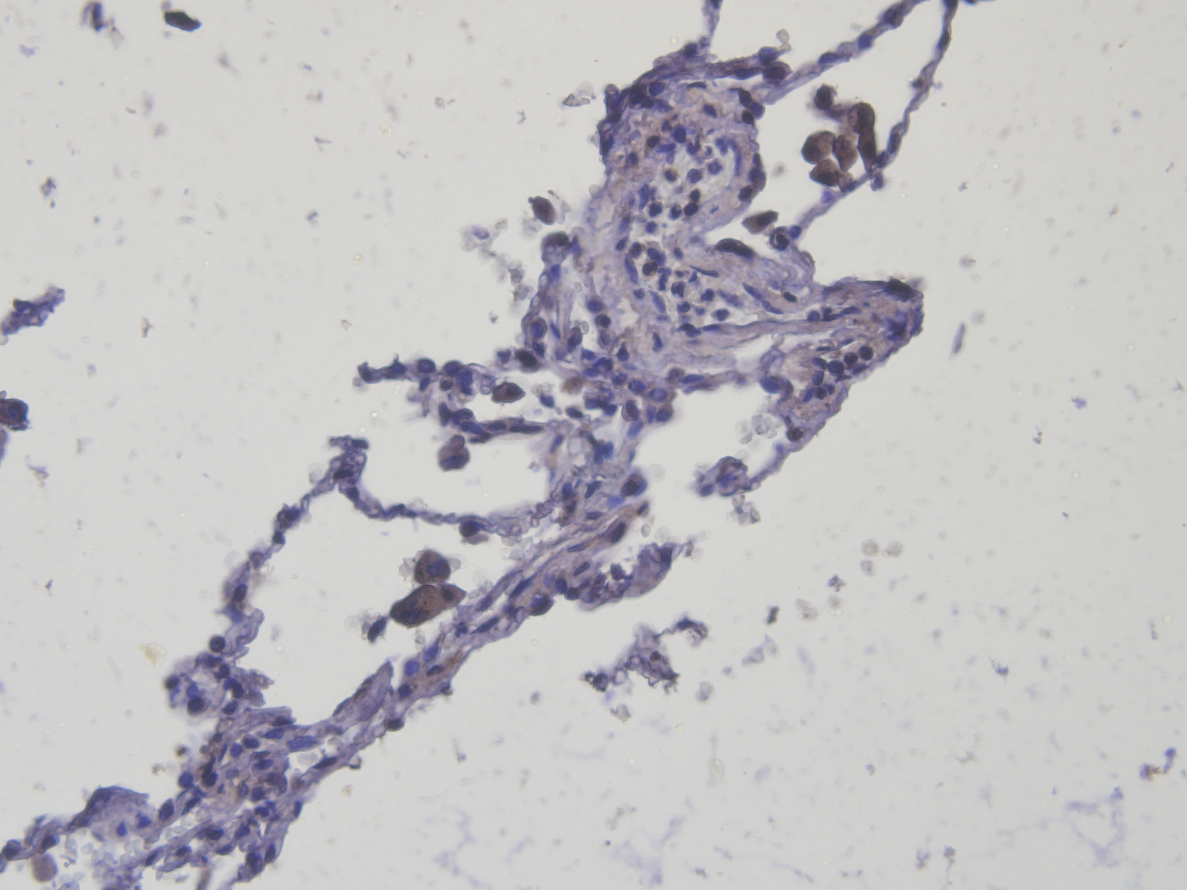

Supplement: S18 File — (ZIP) [file pone.0337223.s019.zip › 462938-400X-N-CA/462938-400X-- N (1).tif]

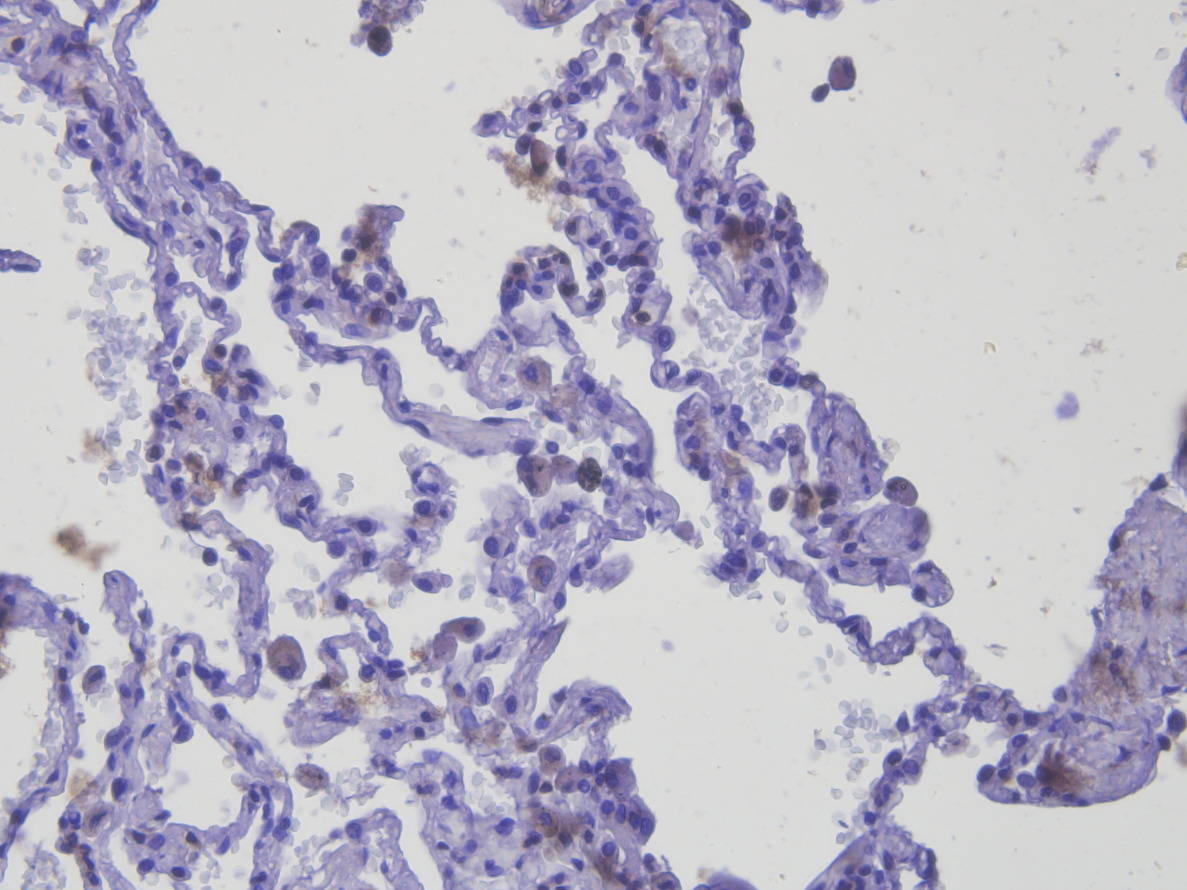

Supplement: S18 File — (ZIP) [file pone.0337223.s019.zip › 462938-400X-N-CA/462938-400X-- N (2).tif]

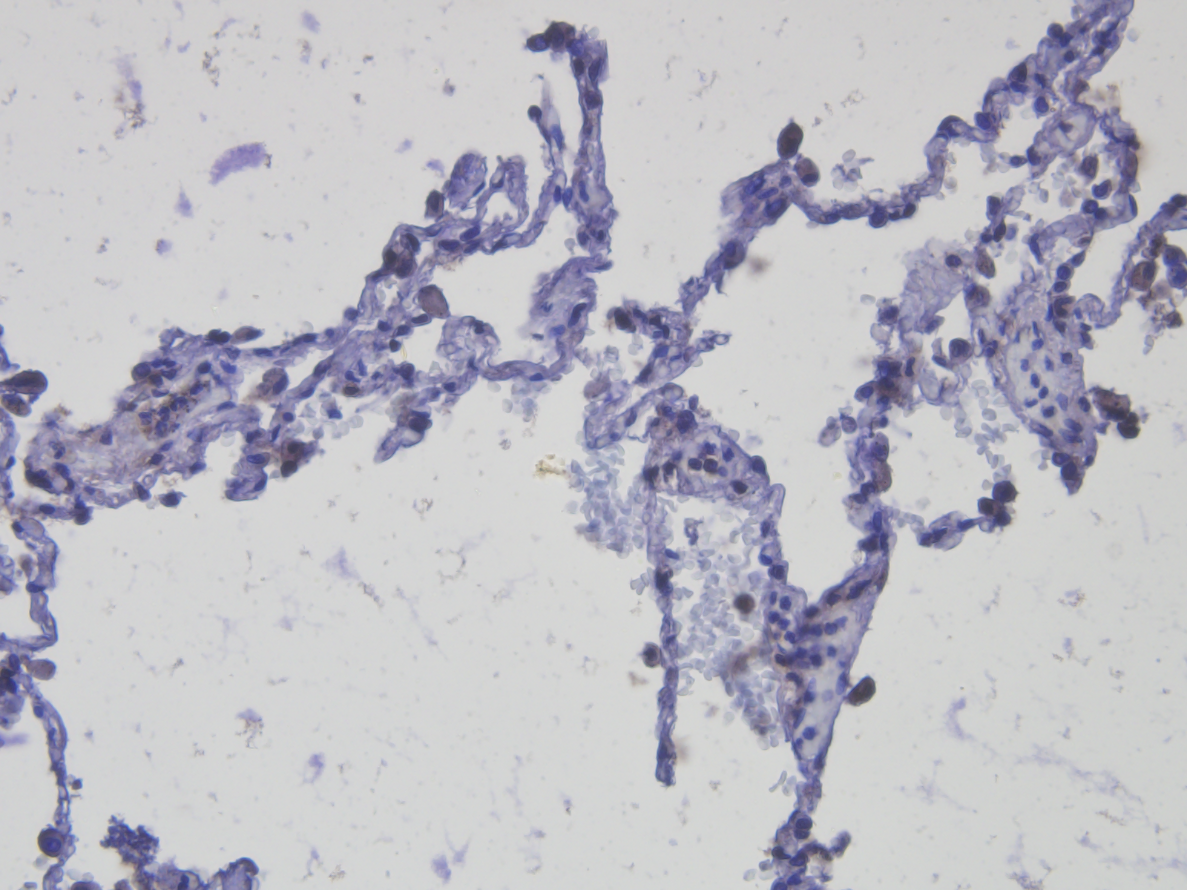

Supplement: S18 File — (ZIP) [file pone.0337223.s019.zip › 462938-400X-N-CA/462938-400X-- N (3).tif]

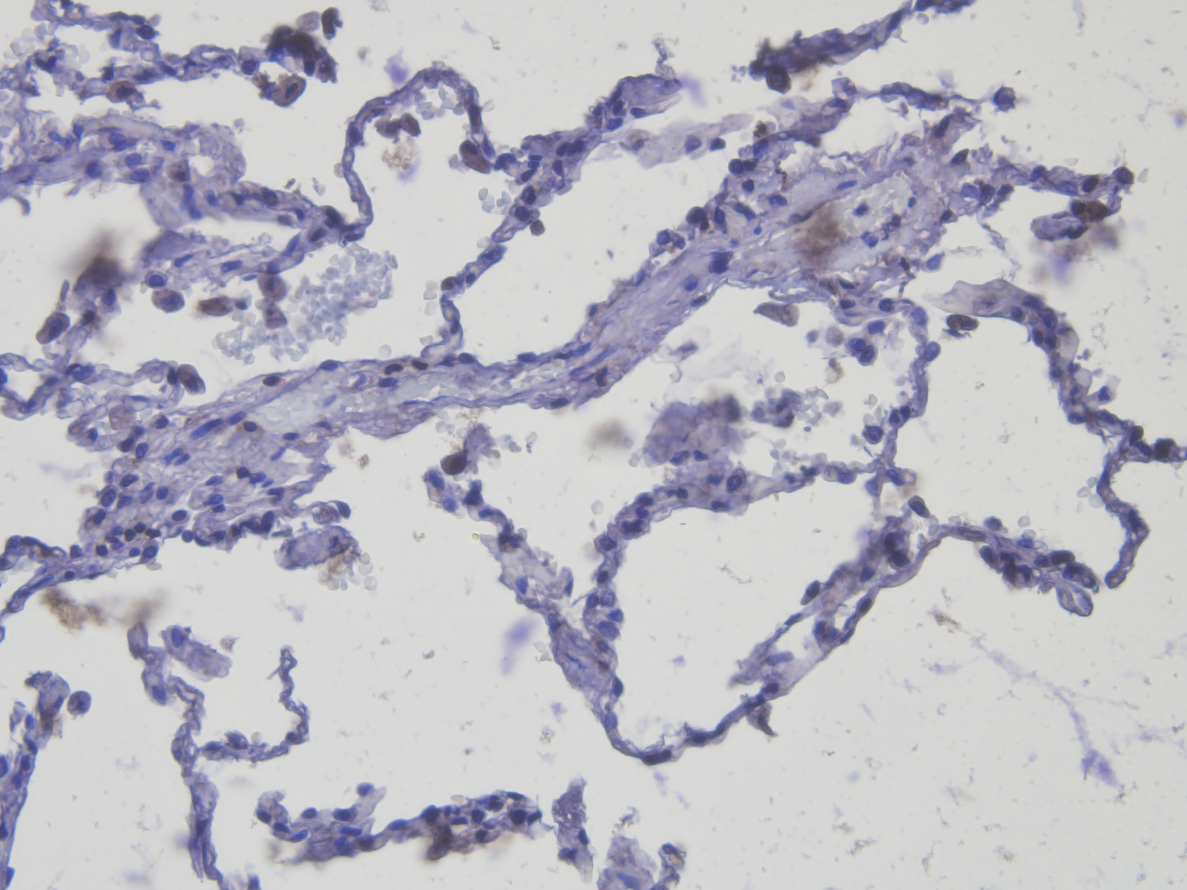

Supplement: S18 File — (ZIP) [file pone.0337223.s019.zip › 462938-400X-N-CA/462938-400X-- N (4).tif]

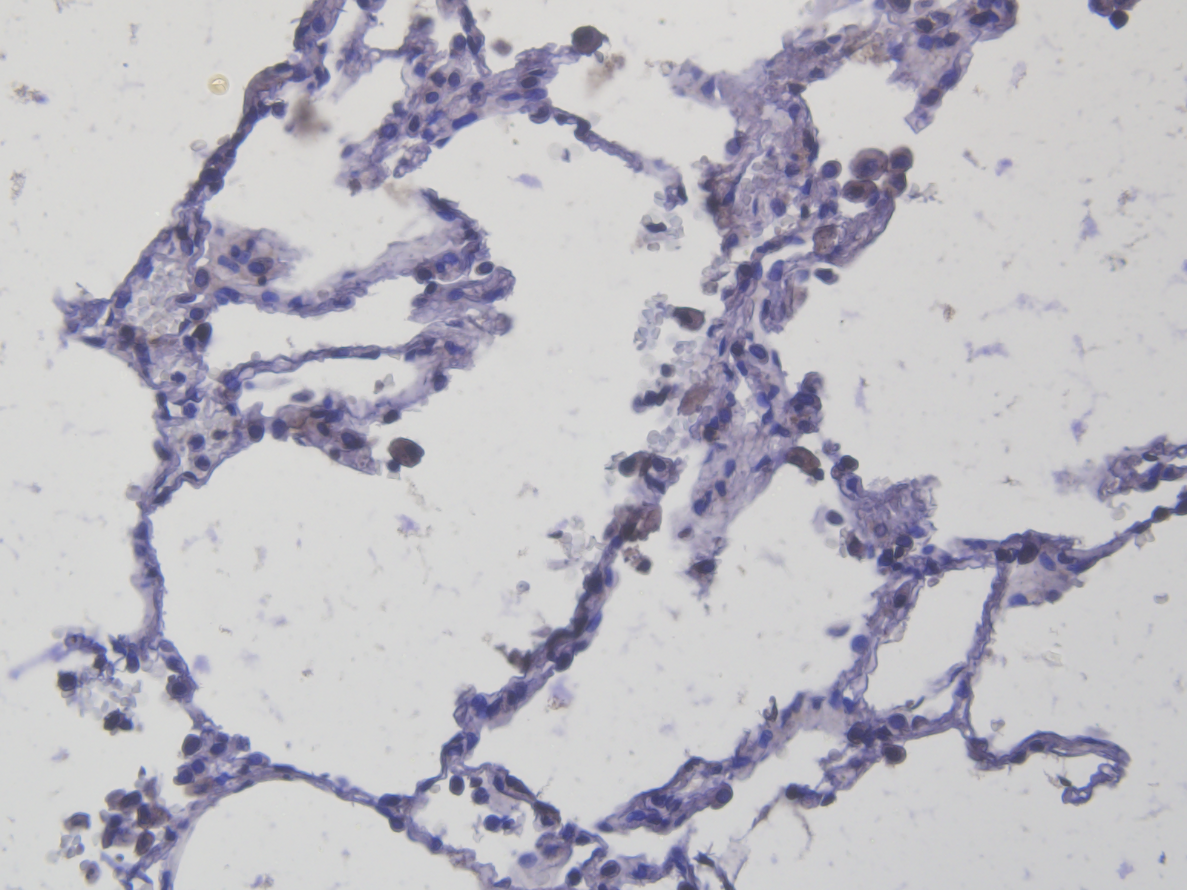

Supplement: S18 File — (ZIP) [file pone.0337223.s019.zip › 462938-400X-N-CA/462938-400X-- N (5).tif]

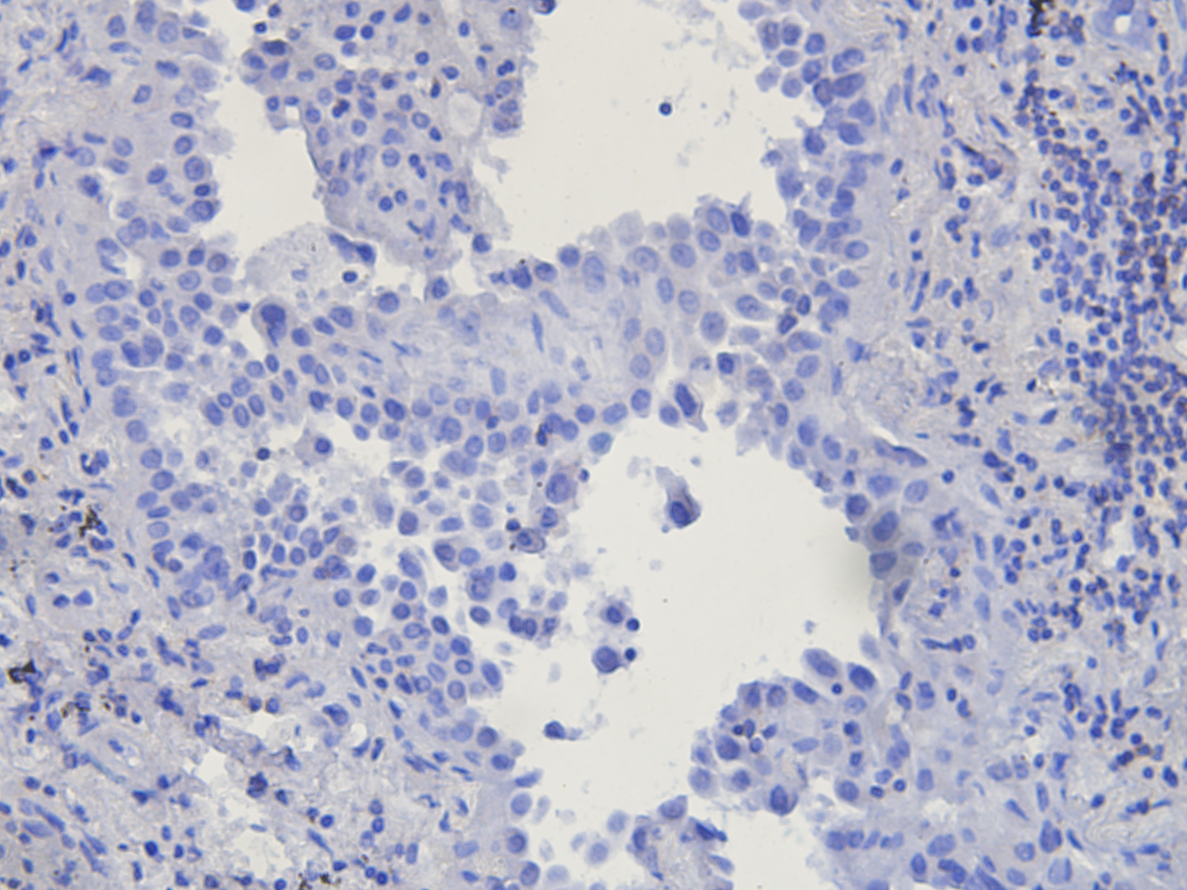

Supplement: S18 File — (ZIP) [file pone.0337223.s019.zip › 462938-400X-N-CA/462938-400X-CA (1).tif]

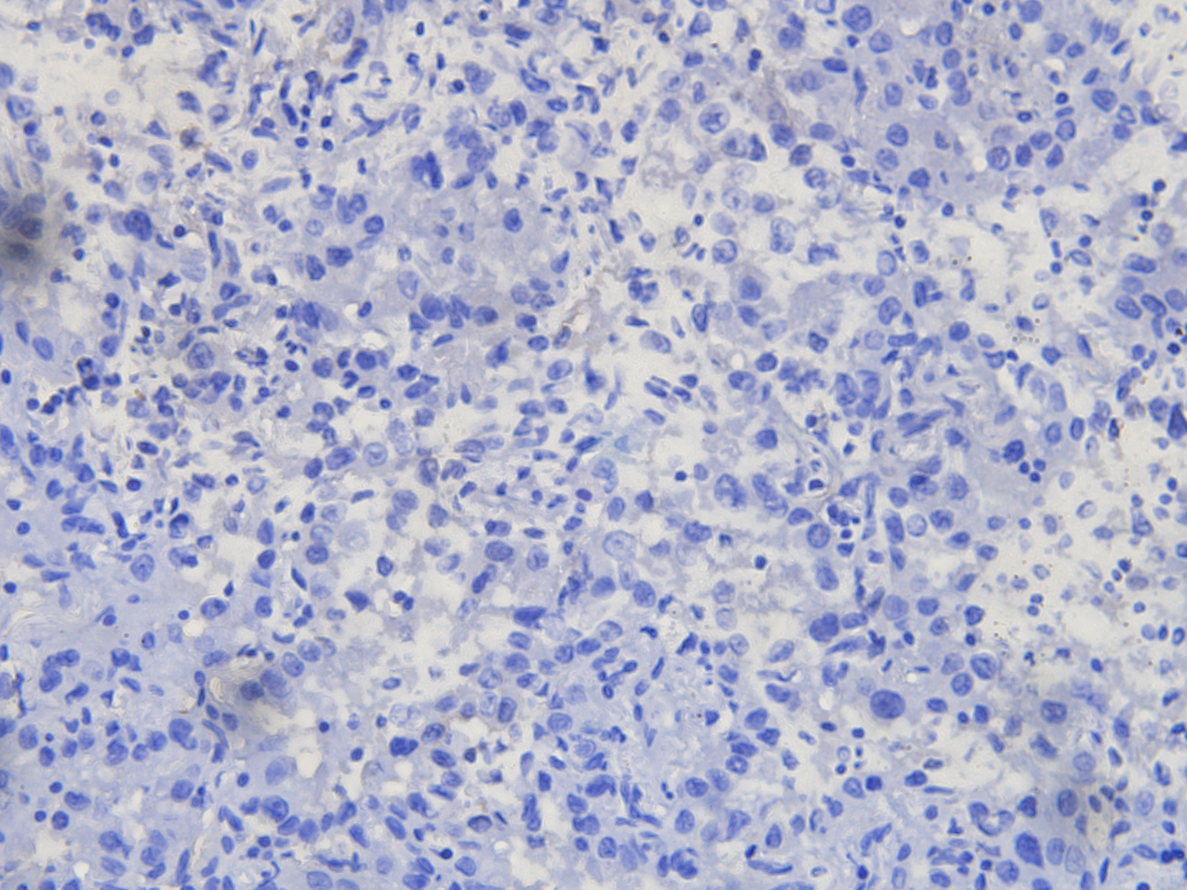

Supplement: S18 File — (ZIP) [file pone.0337223.s019.zip › 462938-400X-N-CA/462938-400X-CA (2).tif]

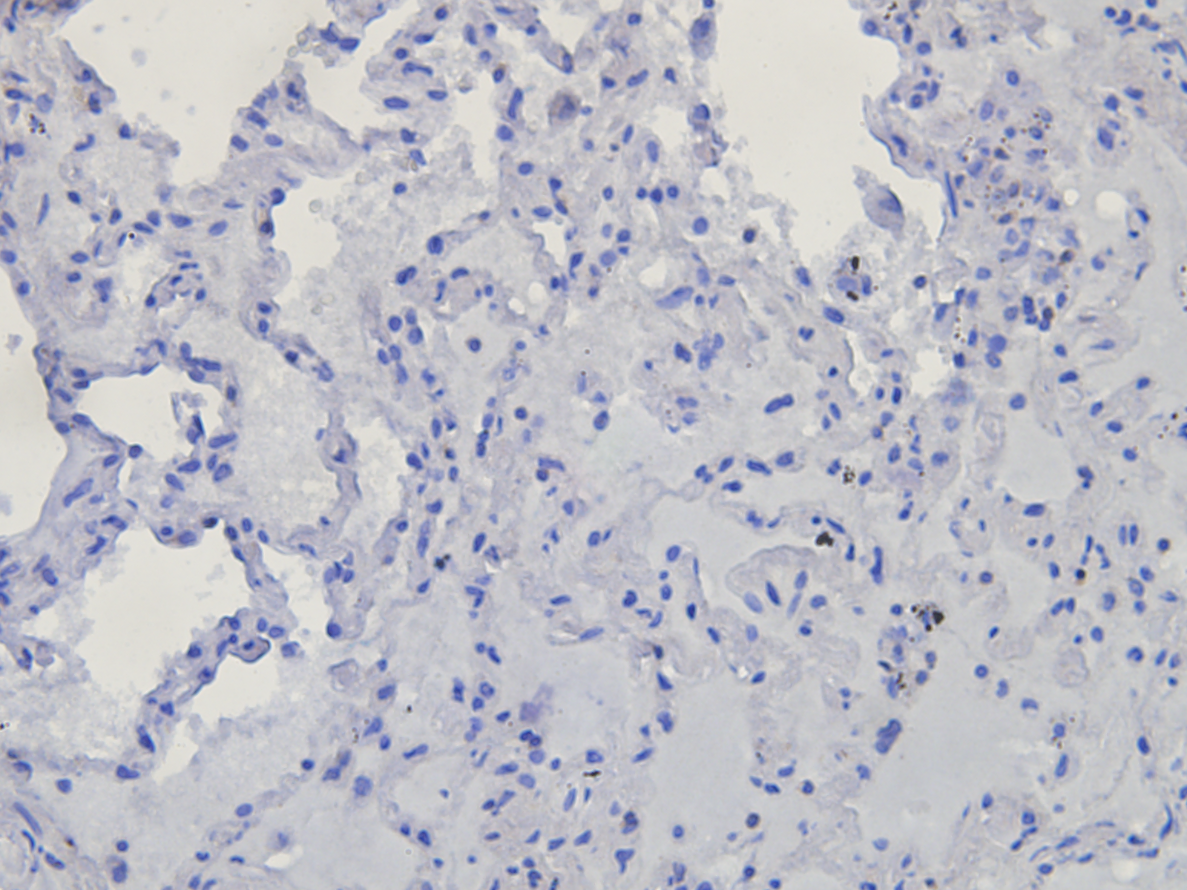

Supplement: S18 File — (ZIP) [file pone.0337223.s019.zip › 462938-400X-N-CA/462938-400X-CA (3).tif]

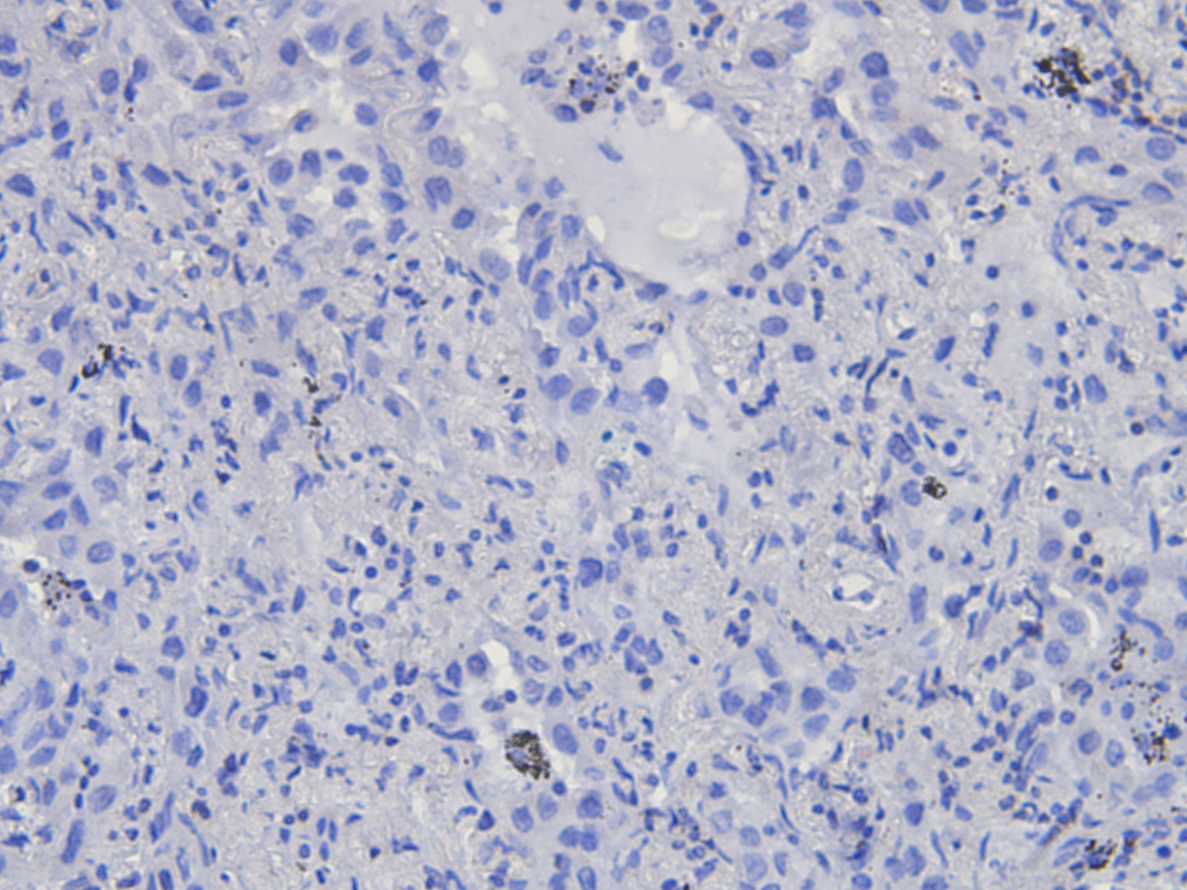

Supplement: S18 File — (ZIP) [file pone.0337223.s019.zip › 462938-400X-N-CA/462938-400X-CA (4).tif]

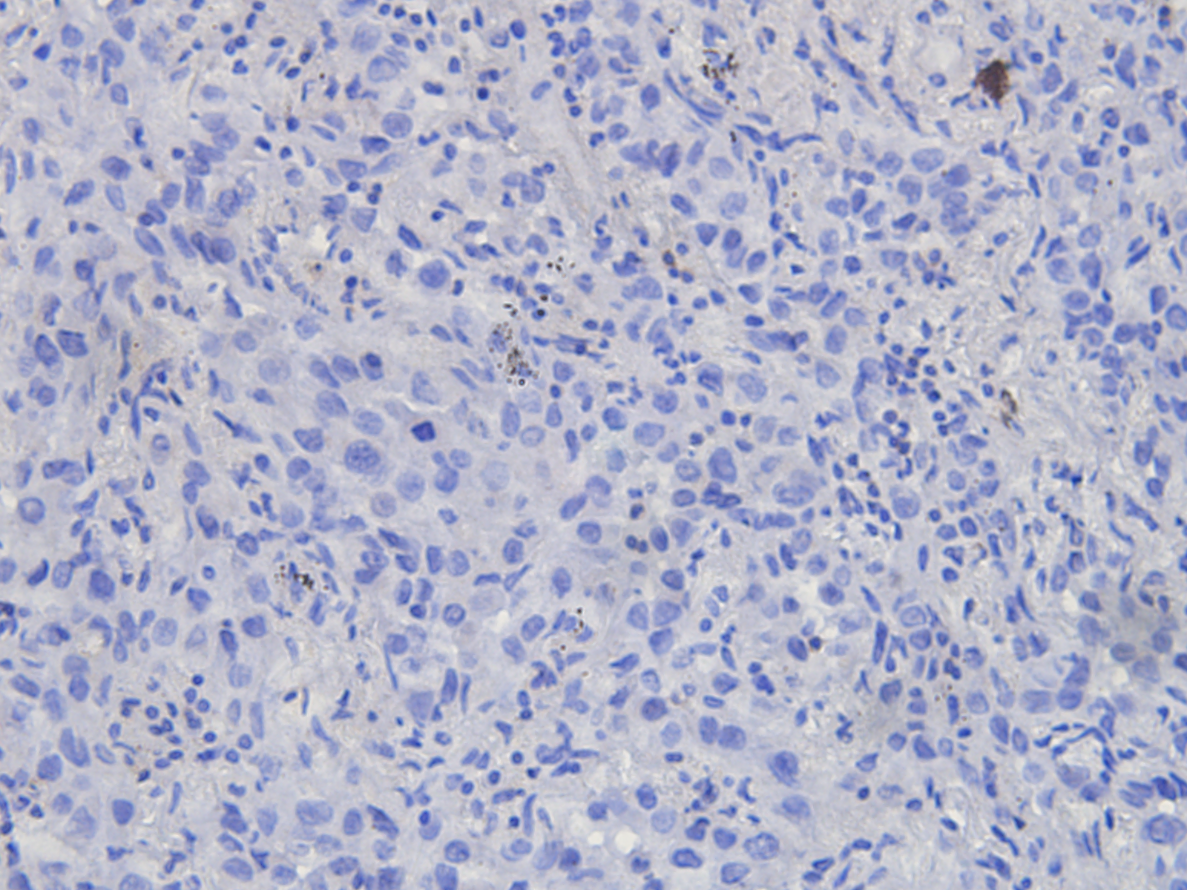

Supplement: S18 File — (ZIP) [file pone.0337223.s019.zip › 462938-400X-N-CA/462938-400X-CA (5).tif]

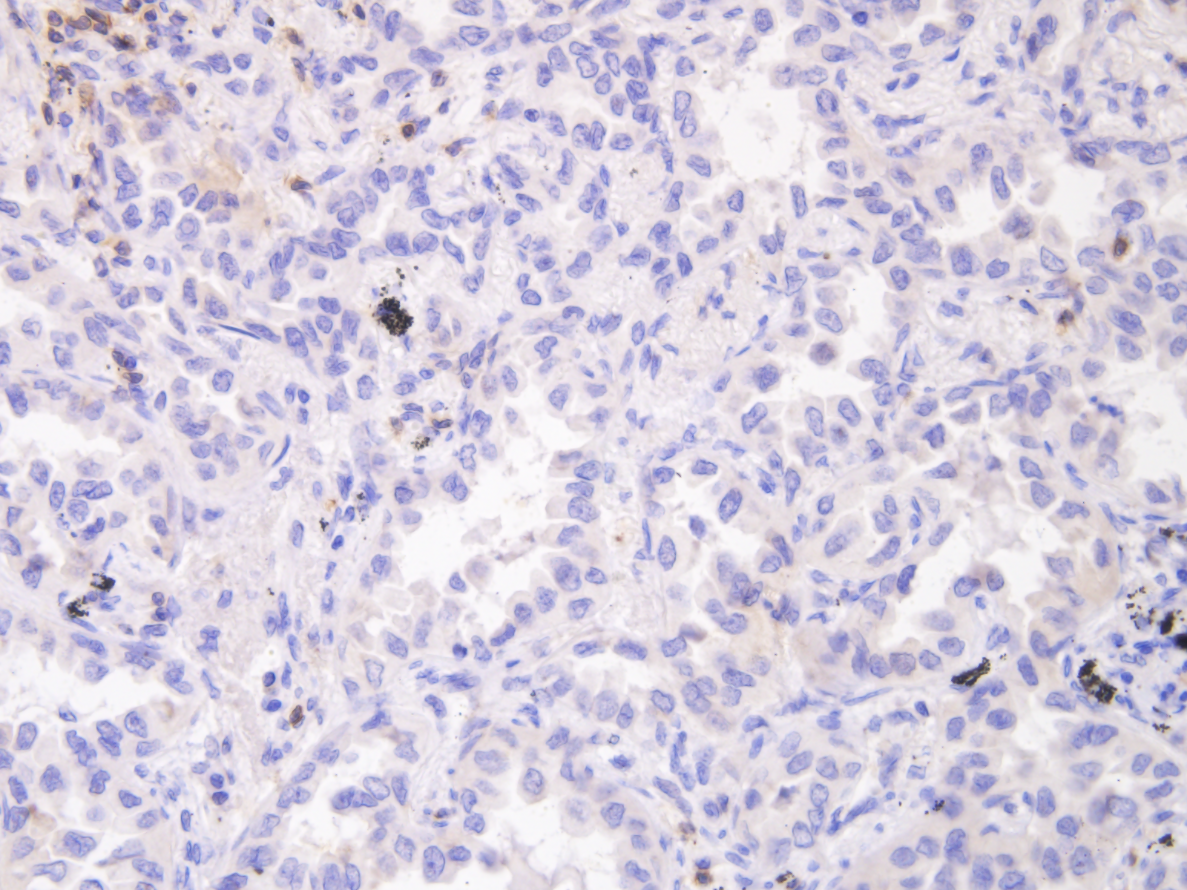

Supplement: S19 File — (ZIP) [file pone.0337223.s020.zip › 464554-400X-N-CA/464554-400X-CA (1).tif]

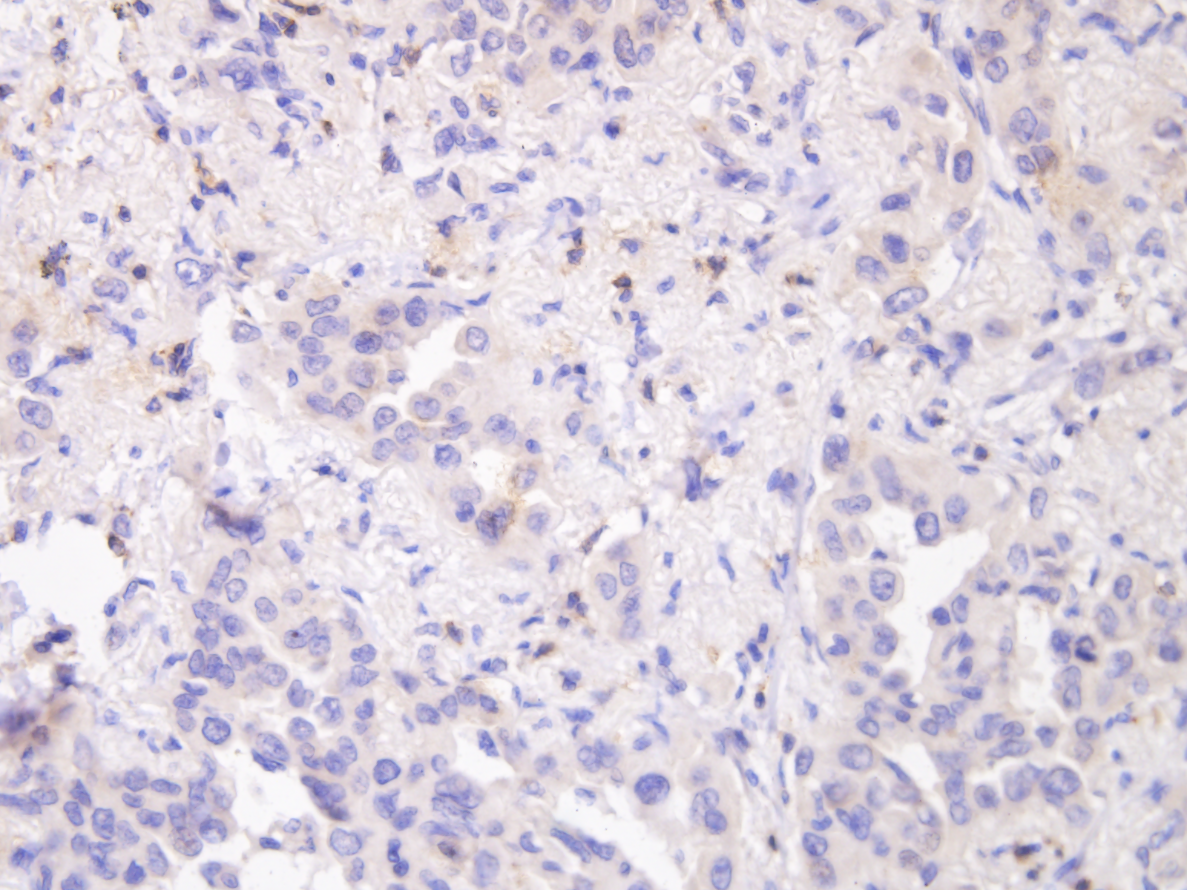

Supplement: S19 File — (ZIP) [file pone.0337223.s020.zip › 464554-400X-N-CA/464554-400X-CA (2).tif]

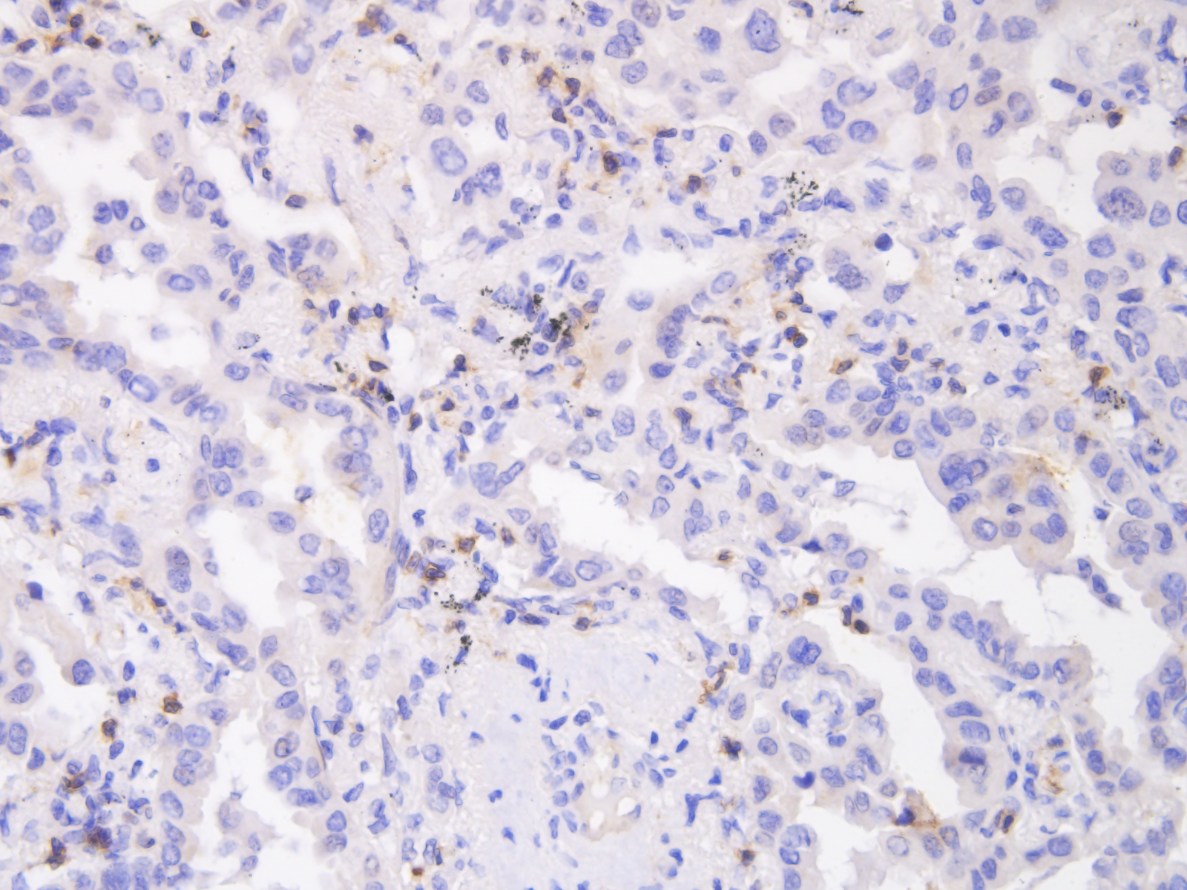

Supplement: S19 File — (ZIP) [file pone.0337223.s020.zip › 464554-400X-N-CA/464554-400X-CA (3).tif]

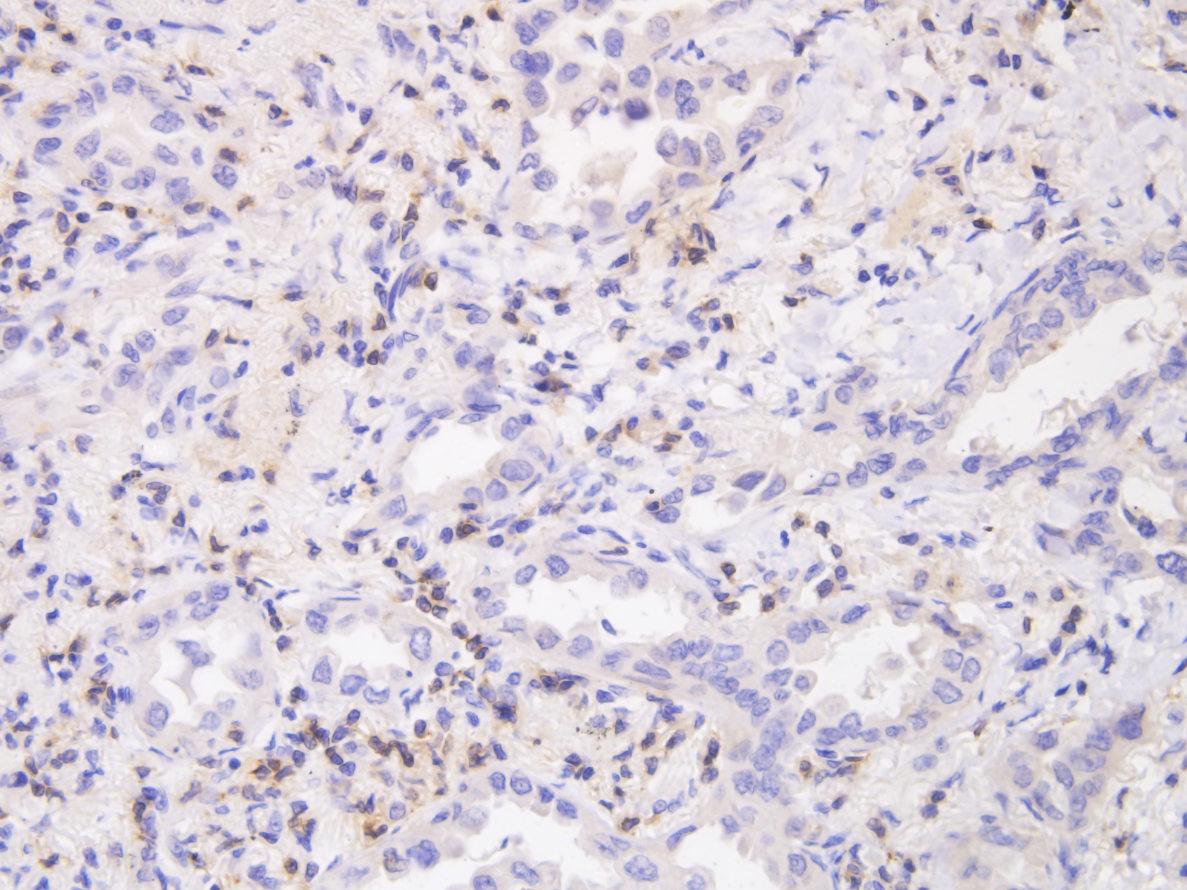

Supplement: S19 File — (ZIP) [file pone.0337223.s020.zip › 464554-400X-N-CA/464554-400X-CA (4).tif]

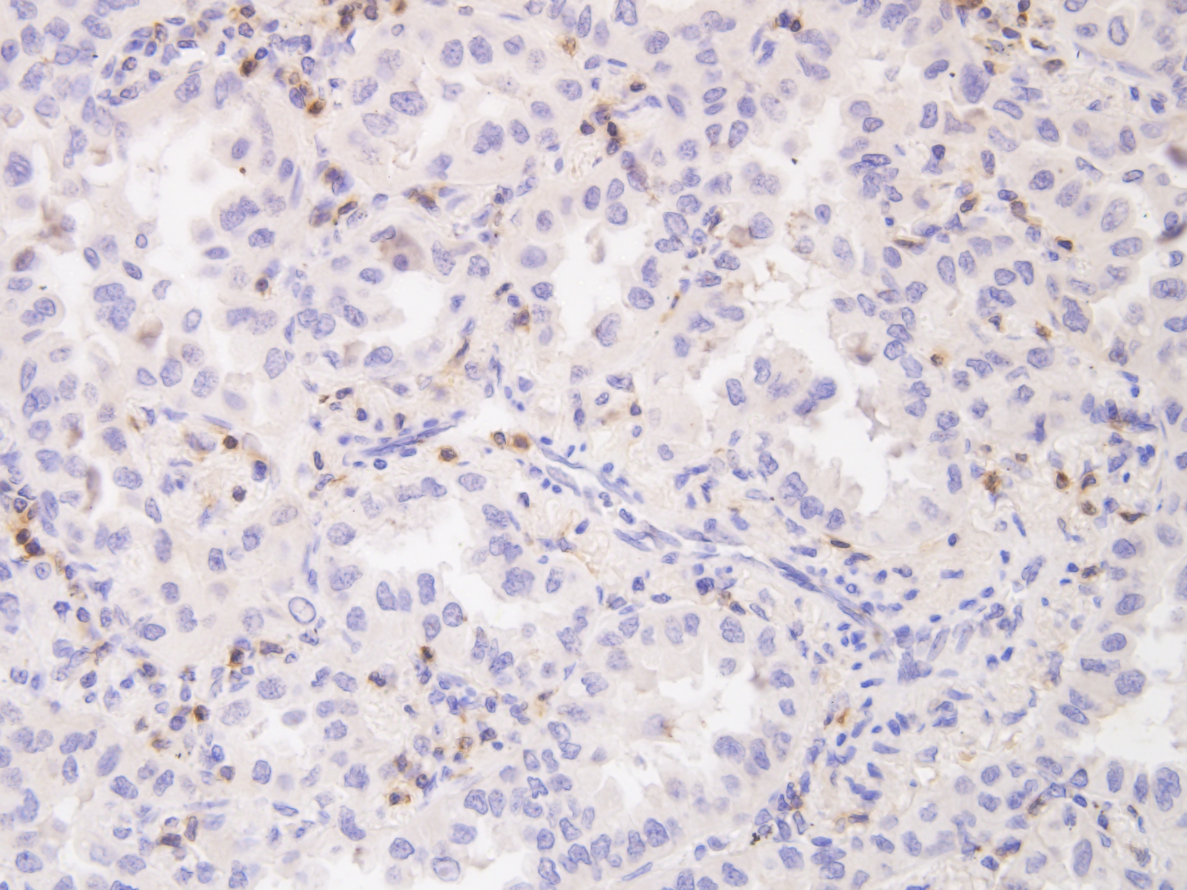

Supplement: S19 File — (ZIP) [file pone.0337223.s020.zip › 464554-400X-N-CA/464554-400X-CA (5).tif]
