# Supplementary material for: Overexpression of PTPRCAP inhibits biological function of lung adenocarcinoma through apoptosis pathway
Source: PLoS One. 2025 Dec 18;20(12):e0337223. doi: 10.1371/journal.pone.0337223 (PMC12716888; doi:10.1371/journal.pone.0337223)

H1299-OE-Scratch original image-0h

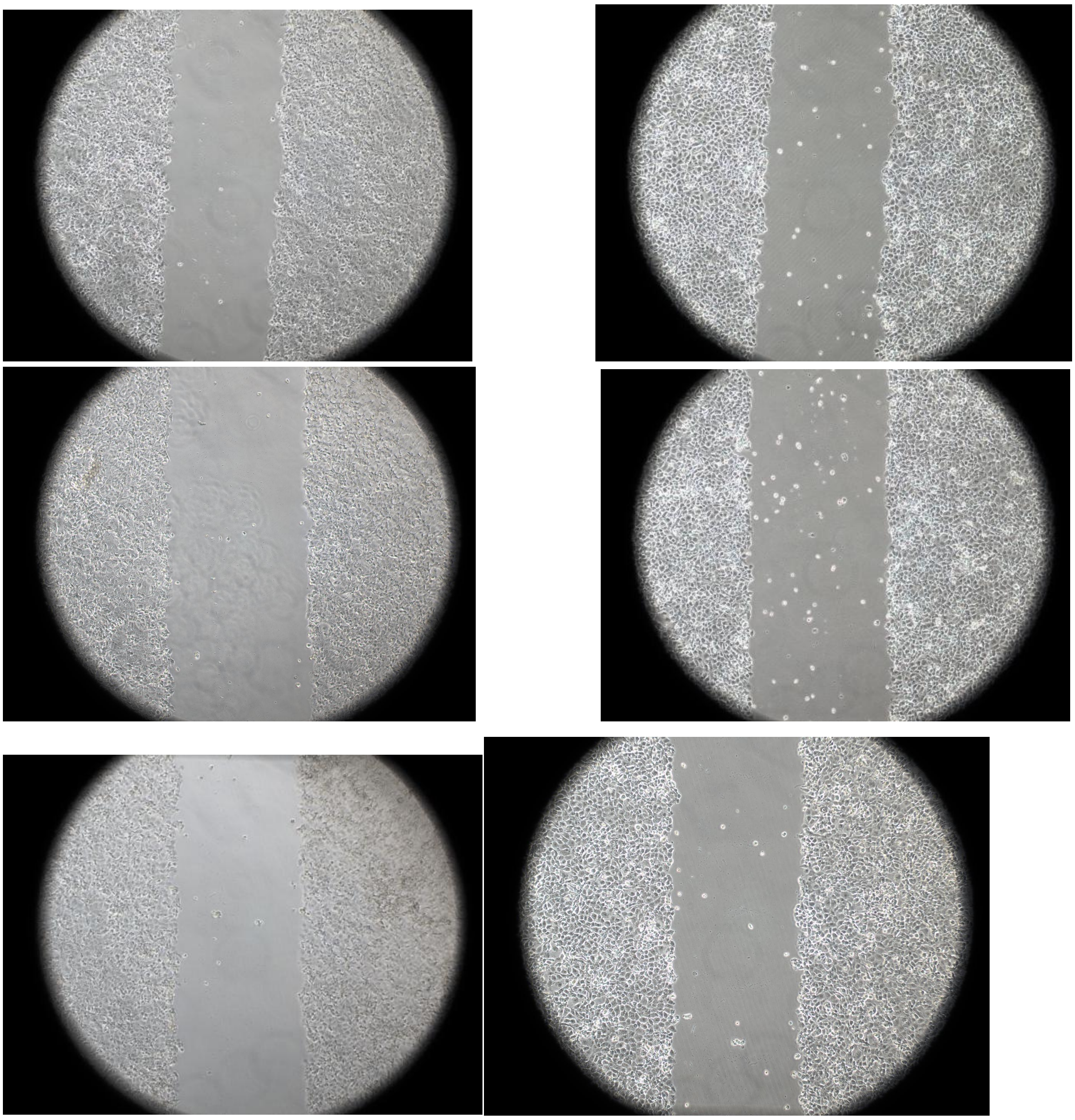

H1299-Vector-Scratch original image-0h

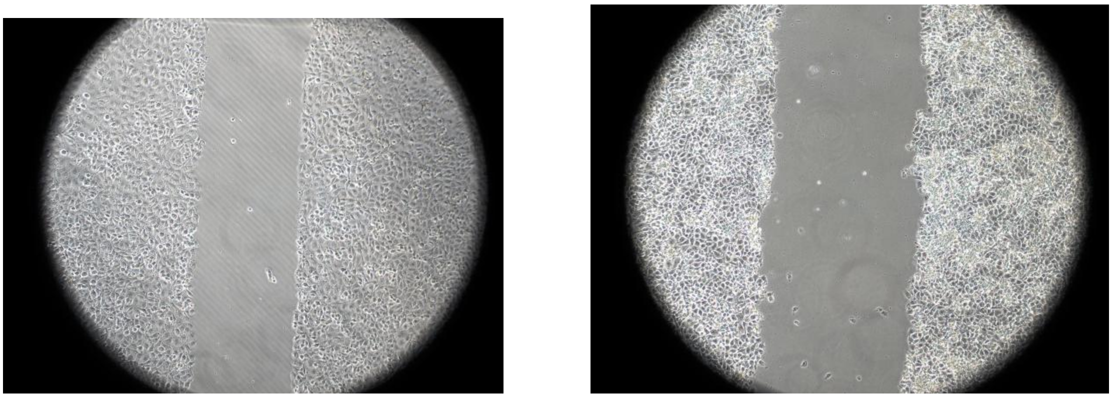

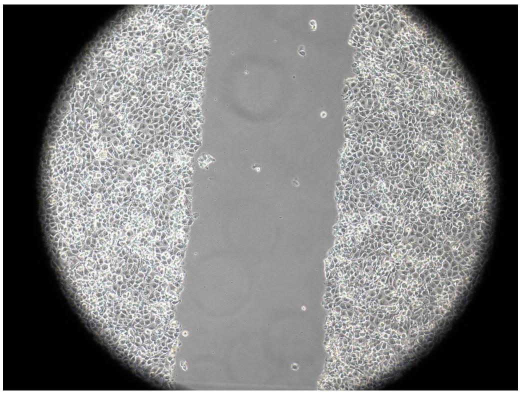
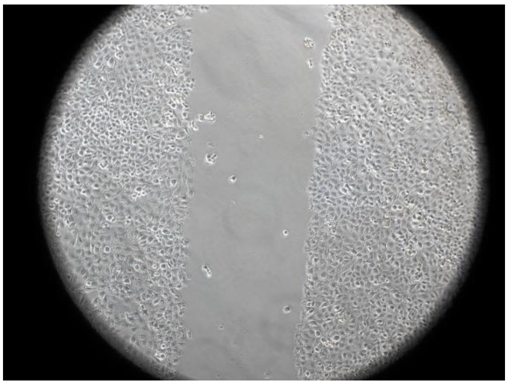
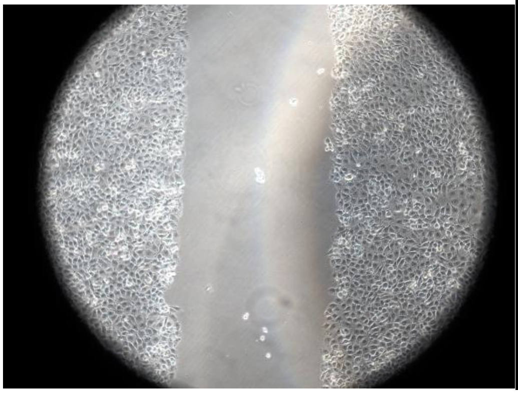
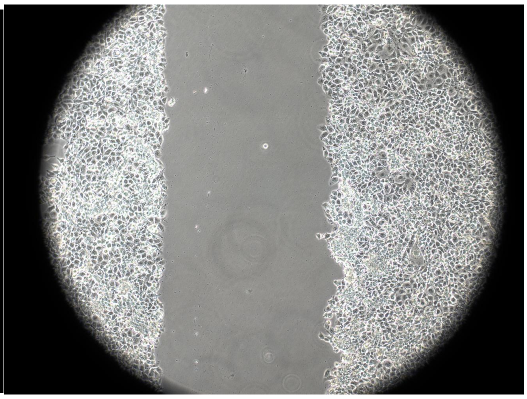

H1299-OE-Scratch original image-24h

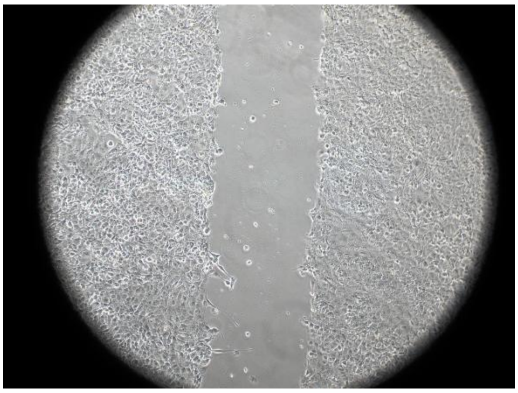
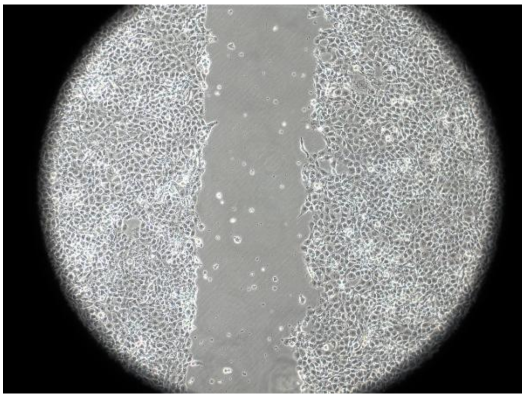
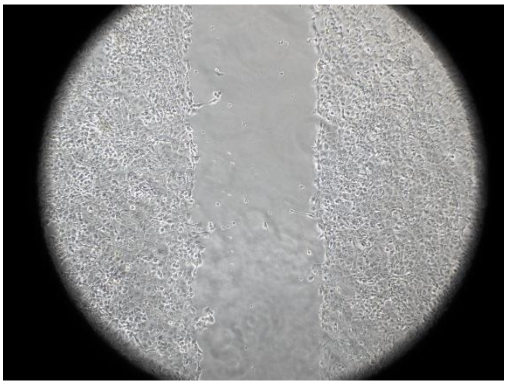
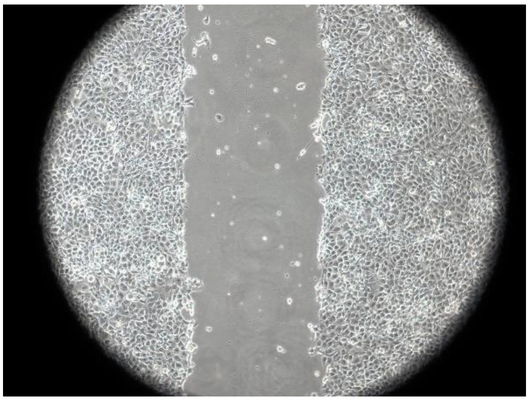

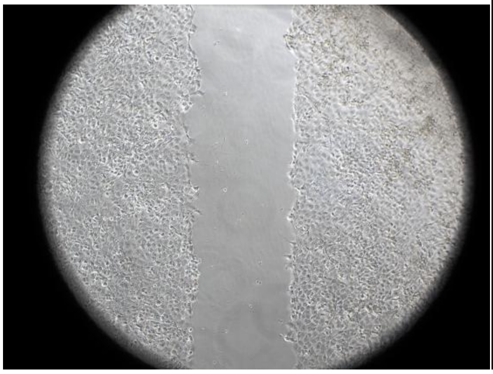
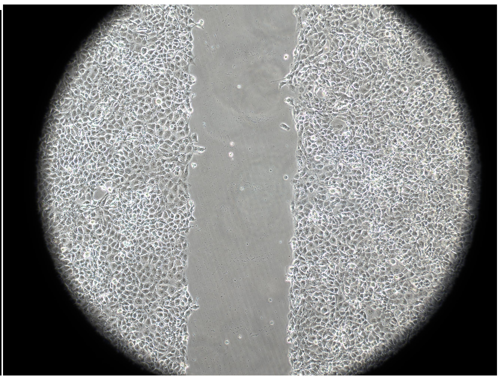

H1299-Vector-Scratch original image-24h

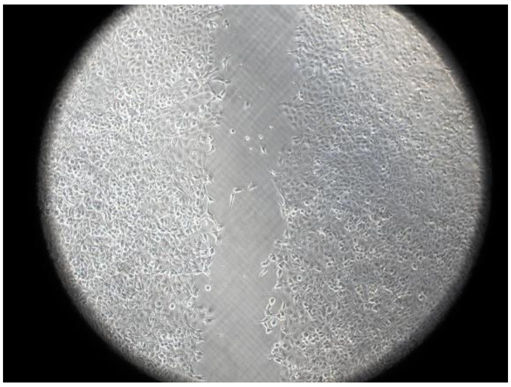
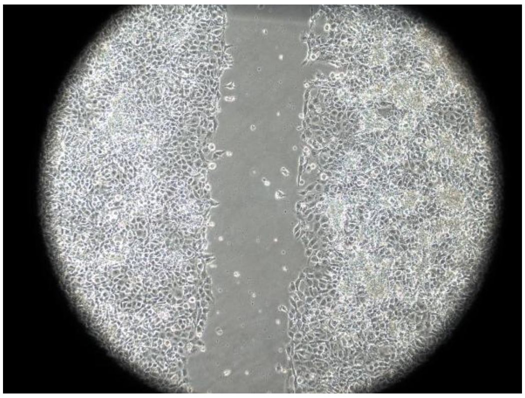
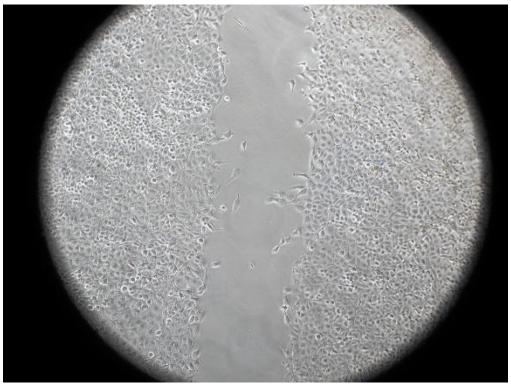
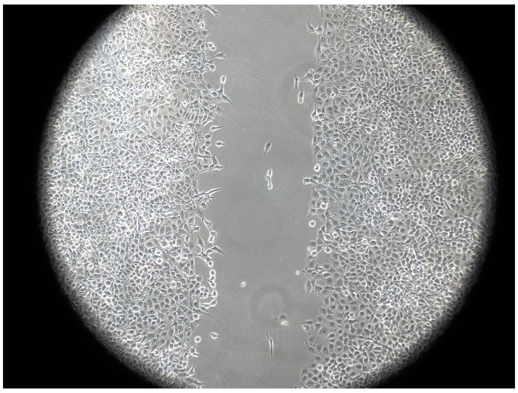
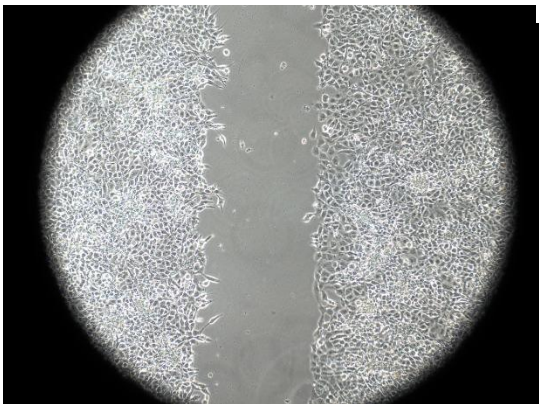
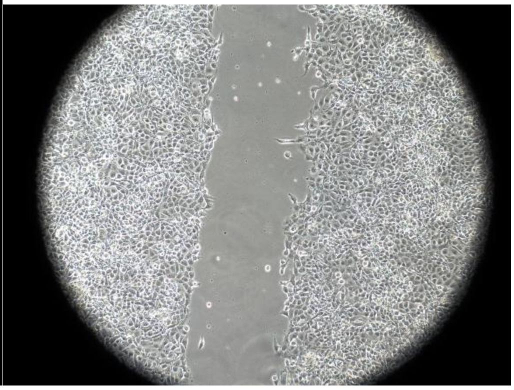

Supplement: S4 File — (PDF) [file pone.0337223.s005.pdf]
