# Supplementary material for: Overexpression of PTPRCAP inhibits biological function of lung adenocarcinoma through apoptosis pathway
Source: PLoS One. 2025 Dec 18;20(12):e0337223. doi: 10.1371/journal.pone.0337223 (PMC12716888; doi:10.1371/journal.pone.0337223)

A549-OE-Green fluorescence transfection efficiency.

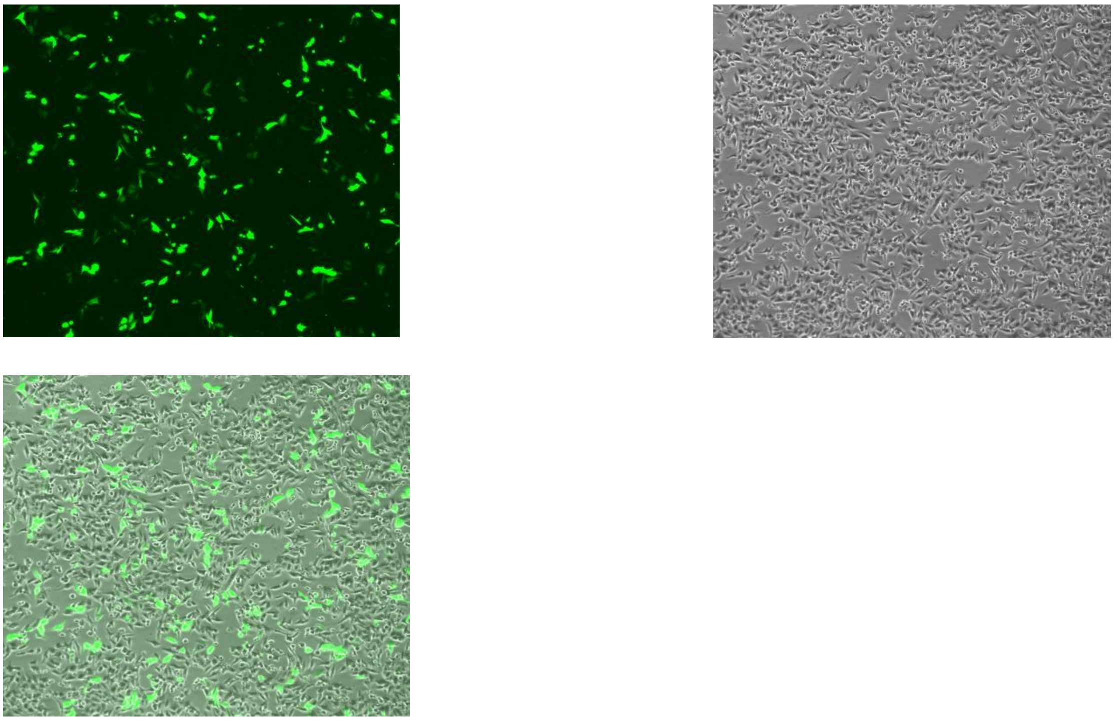

A549-Vector-Green fluorescence transfection efficiency.

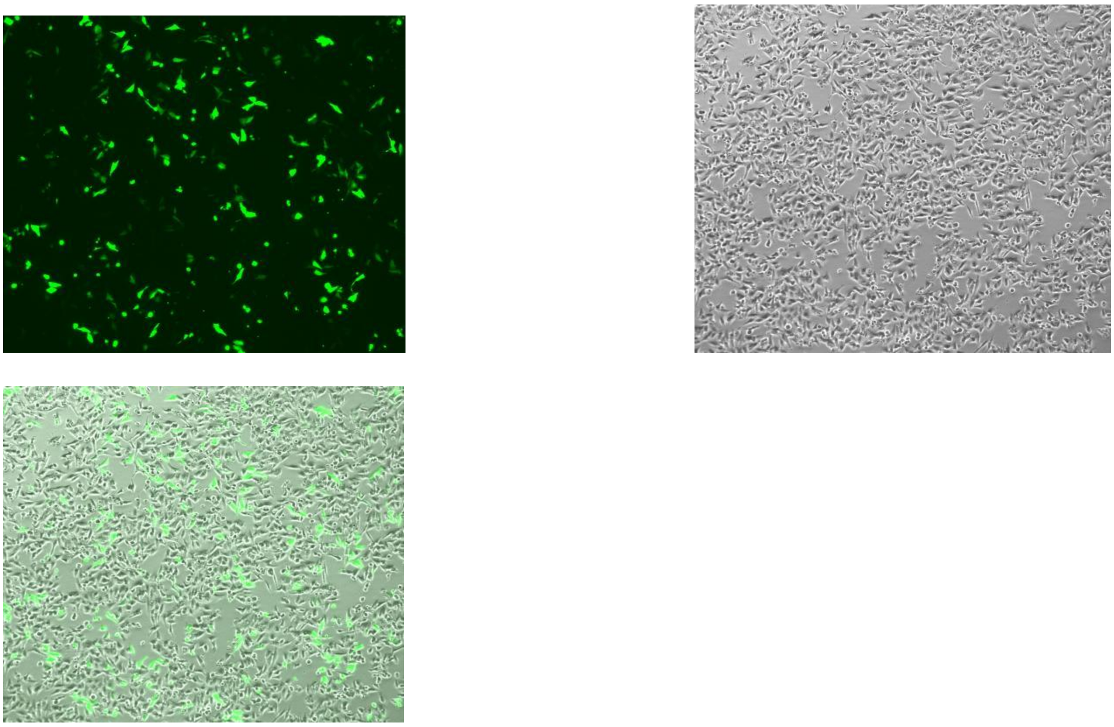

H1299-OE-Green fluorescence transfection efficiency.

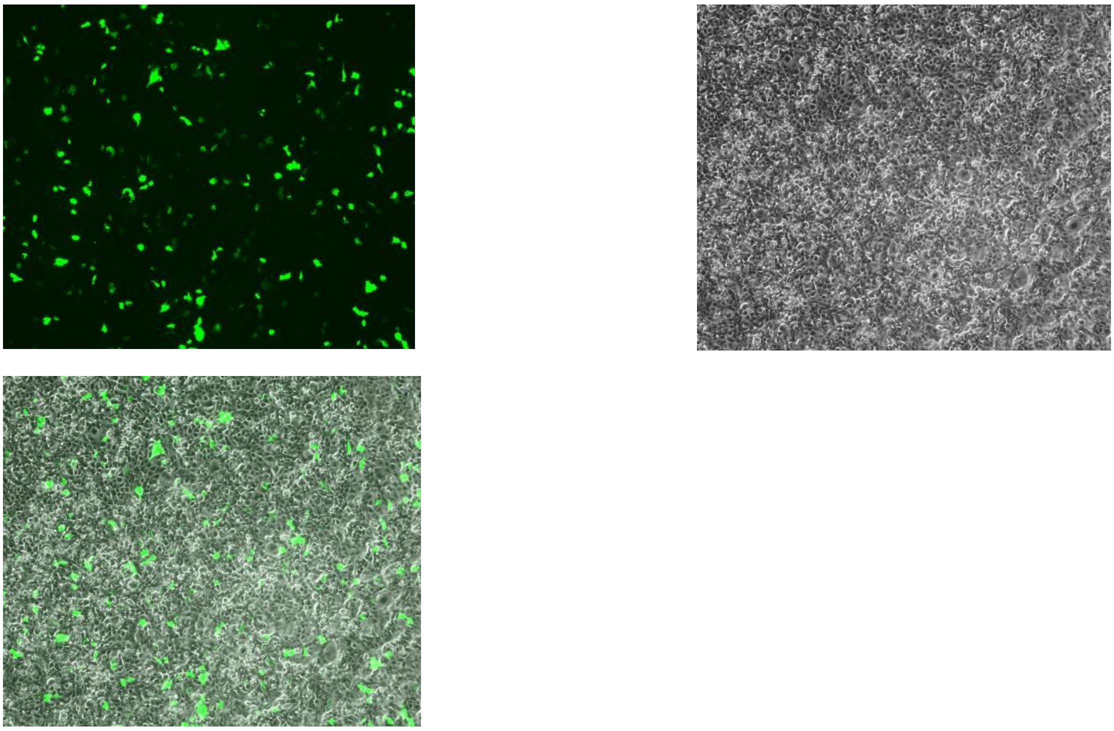

H1299-Vector-Green fluorescence transfection efficiency.

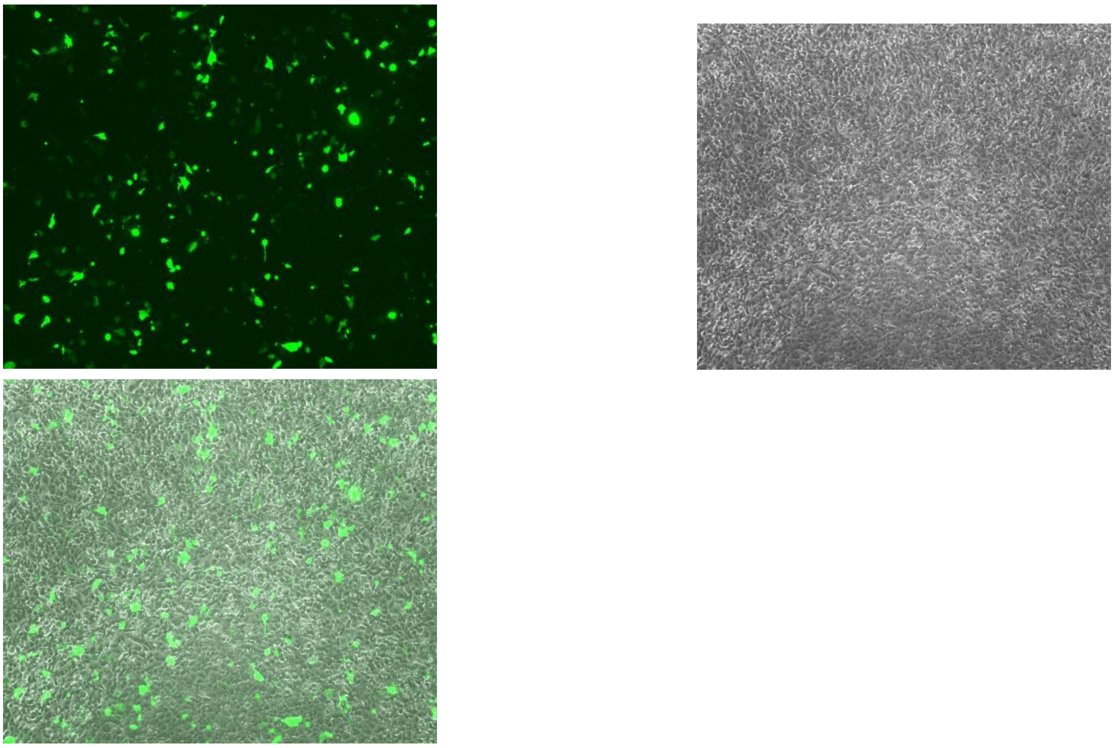

Supplement: S5 File — (PDF) [file pone.0337223.s006.pdf]
