# Supplementary figures and images for: Overexpression of PTPRCAP inhibits biological function of lung adenocarcinoma through apoptosis pathway (part 2 of 5)
Source: PLoS One. 2025 Dec 18;20(12):e0337223. doi: 10.1371/journal.pone.0337223 (PMC12716888; doi:10.1371/journal.pone.0337223)

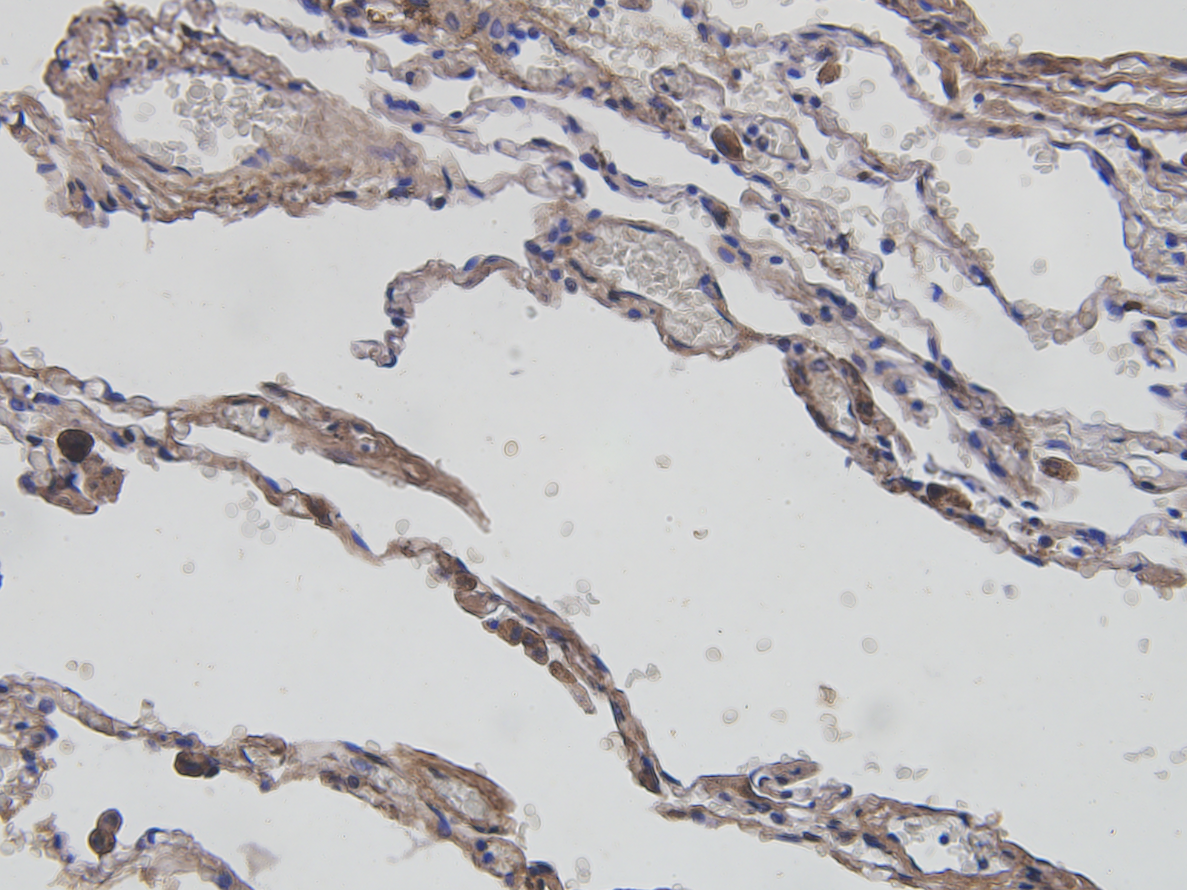

Supplement: S19 File — (ZIP) [file pone.0337223.s020.zip › 464554-400X-N-CA/464554-400X-N (1).tif]

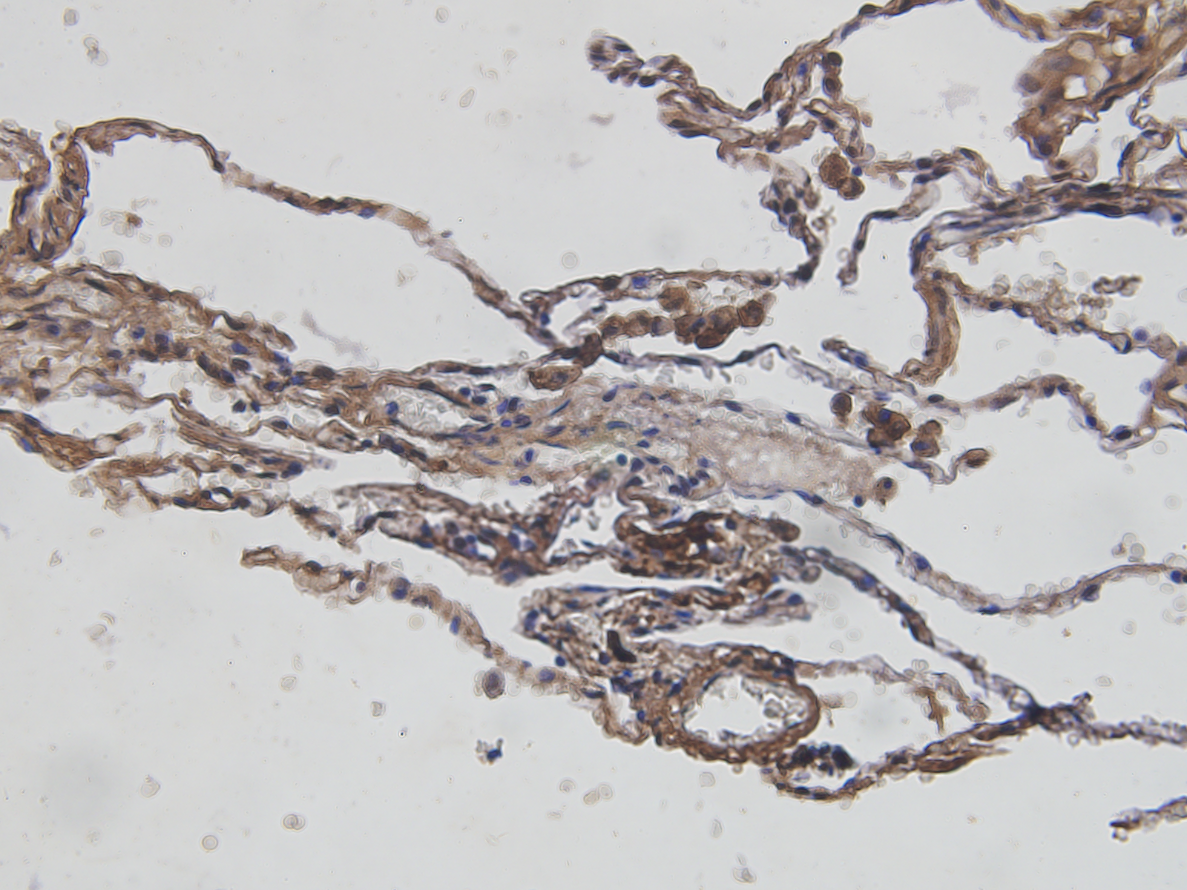

Supplement: S19 File — (ZIP) [file pone.0337223.s020.zip › 464554-400X-N-CA/464554-400X-N (2).tif]

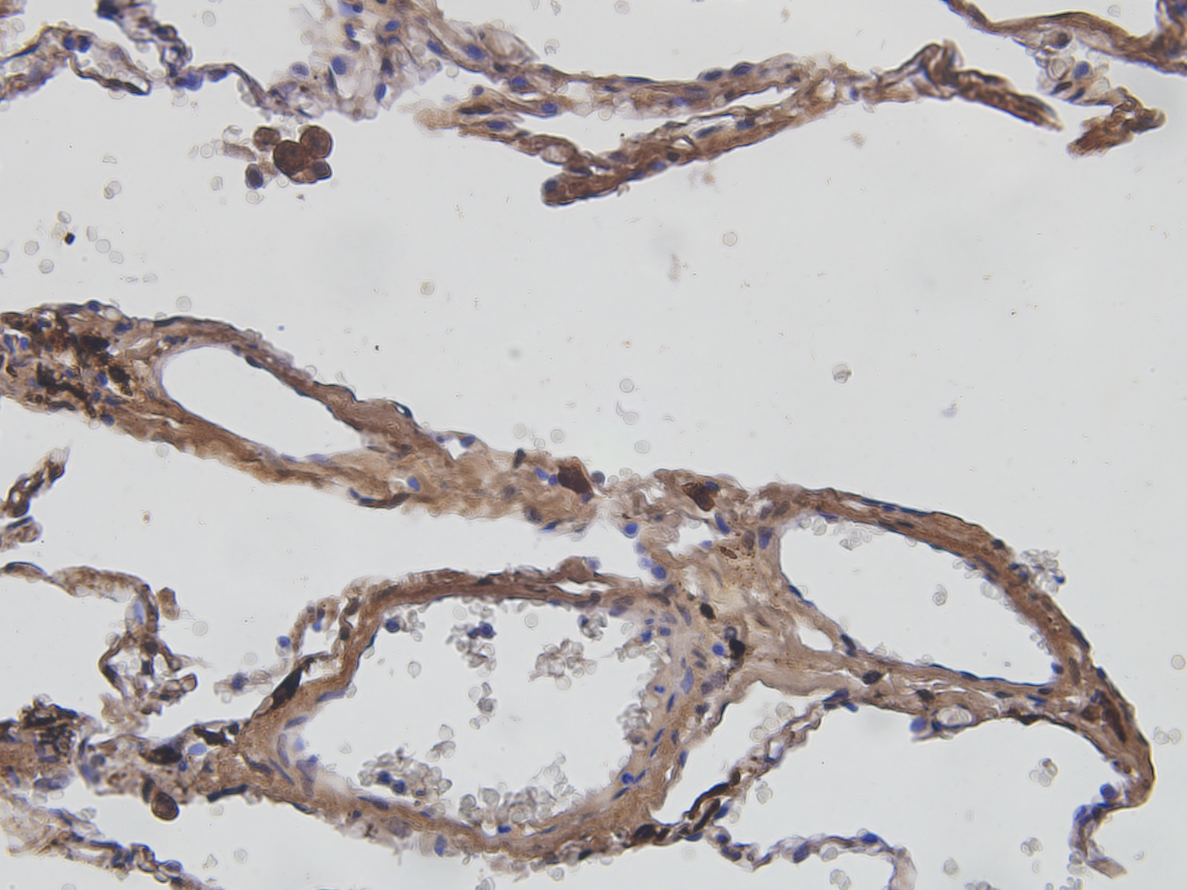

Supplement: S19 File — (ZIP) [file pone.0337223.s020.zip › 464554-400X-N-CA/464554-400X-N (3).tif]

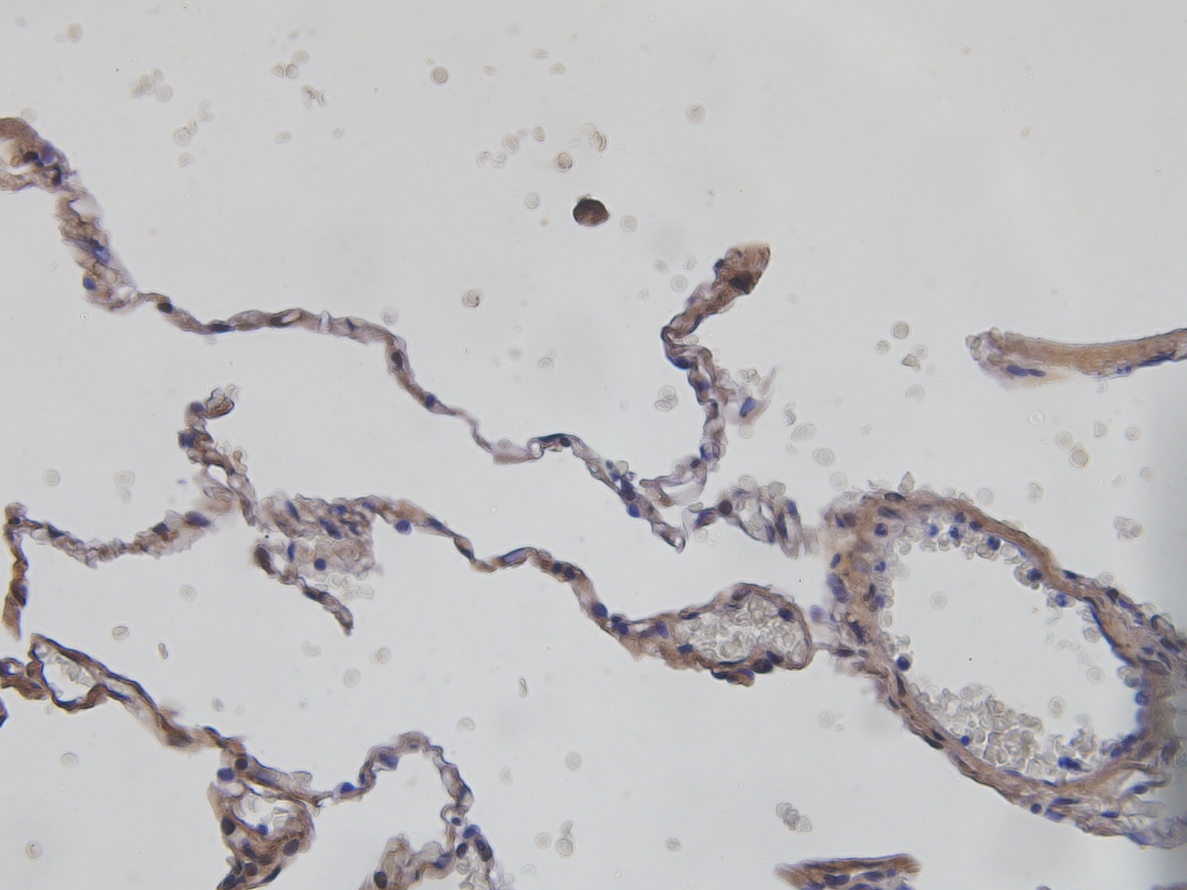

Supplement: S19 File — (ZIP) [file pone.0337223.s020.zip › 464554-400X-N-CA/464554-400X-N (4).tif]

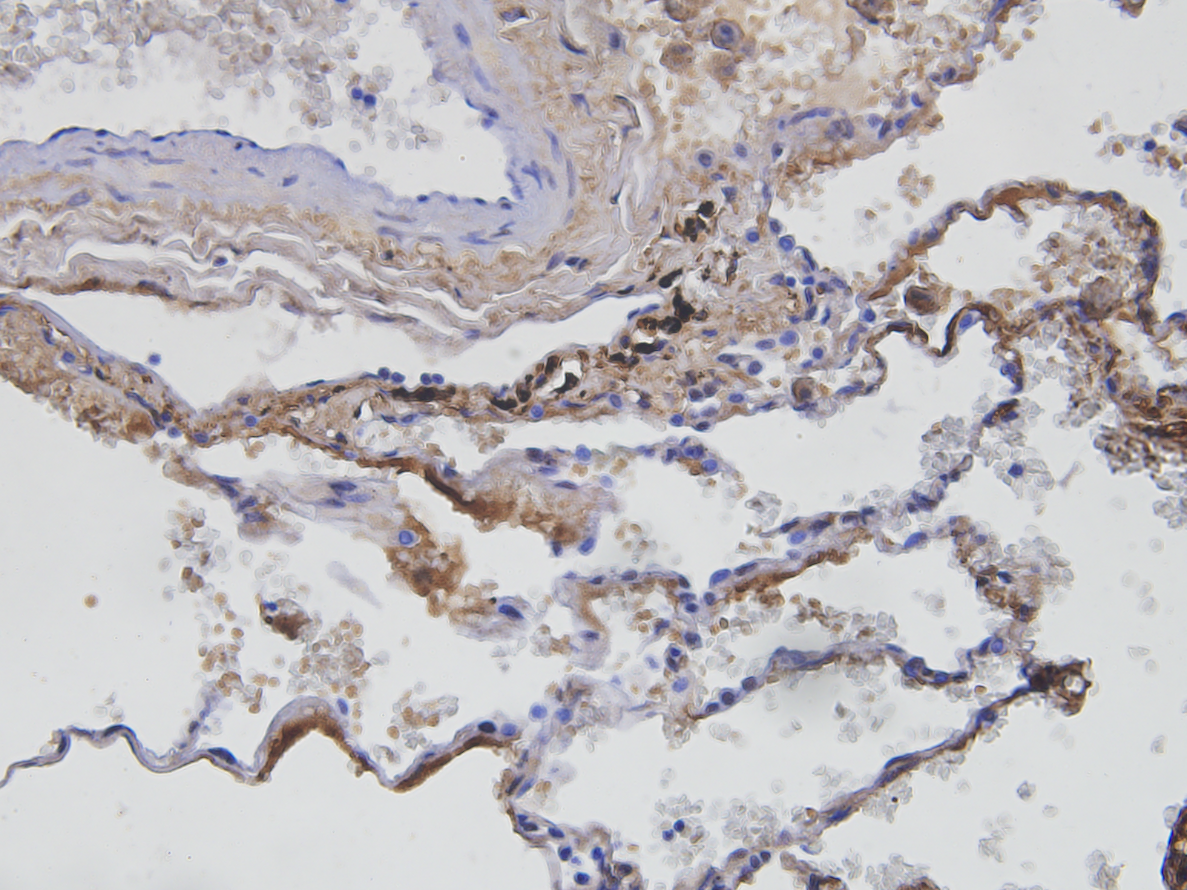

Supplement: S19 File — (ZIP) [file pone.0337223.s020.zip › 464554-400X-N-CA/464554-400X-N (5).tif]

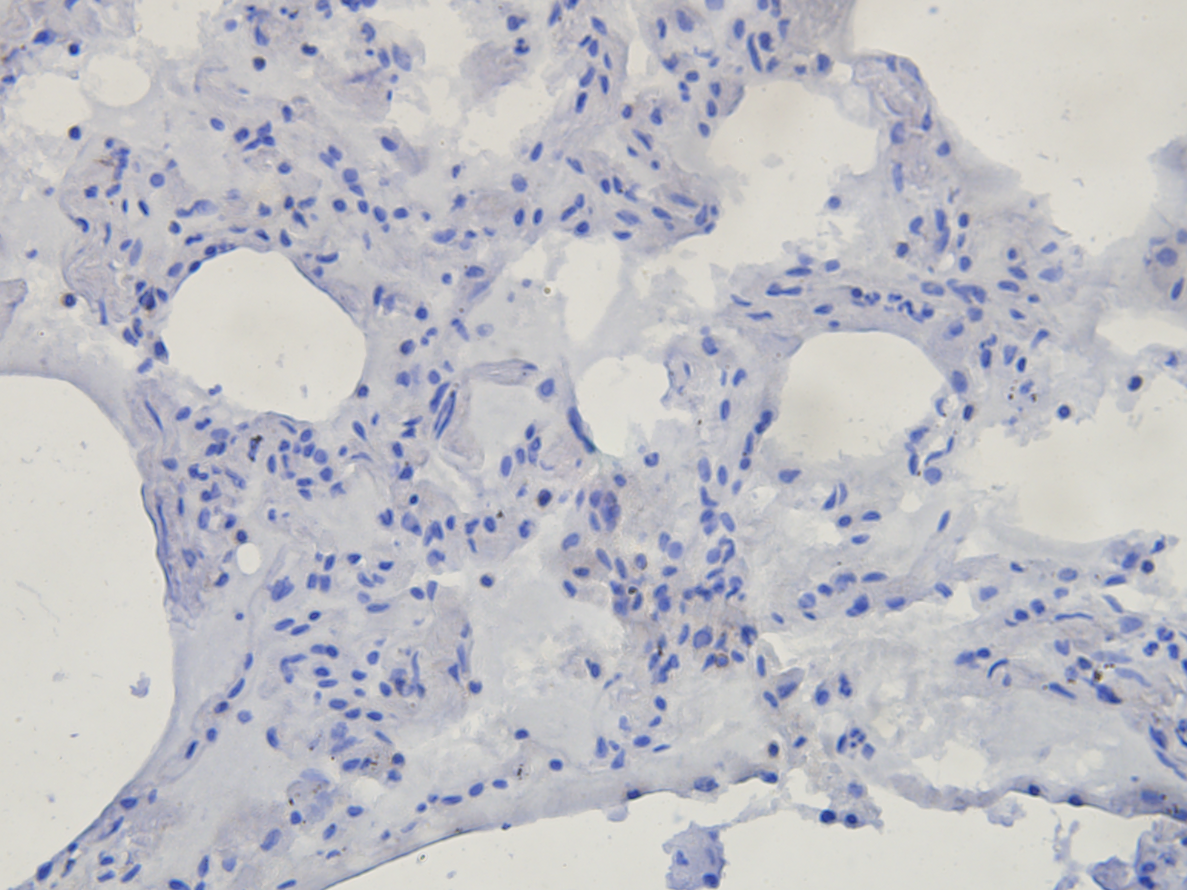

Supplement: S20 File — (ZIP) [file pone.0337223.s021.zip › 465281-400X-N-CA/465281-400X-CA (1).tif]

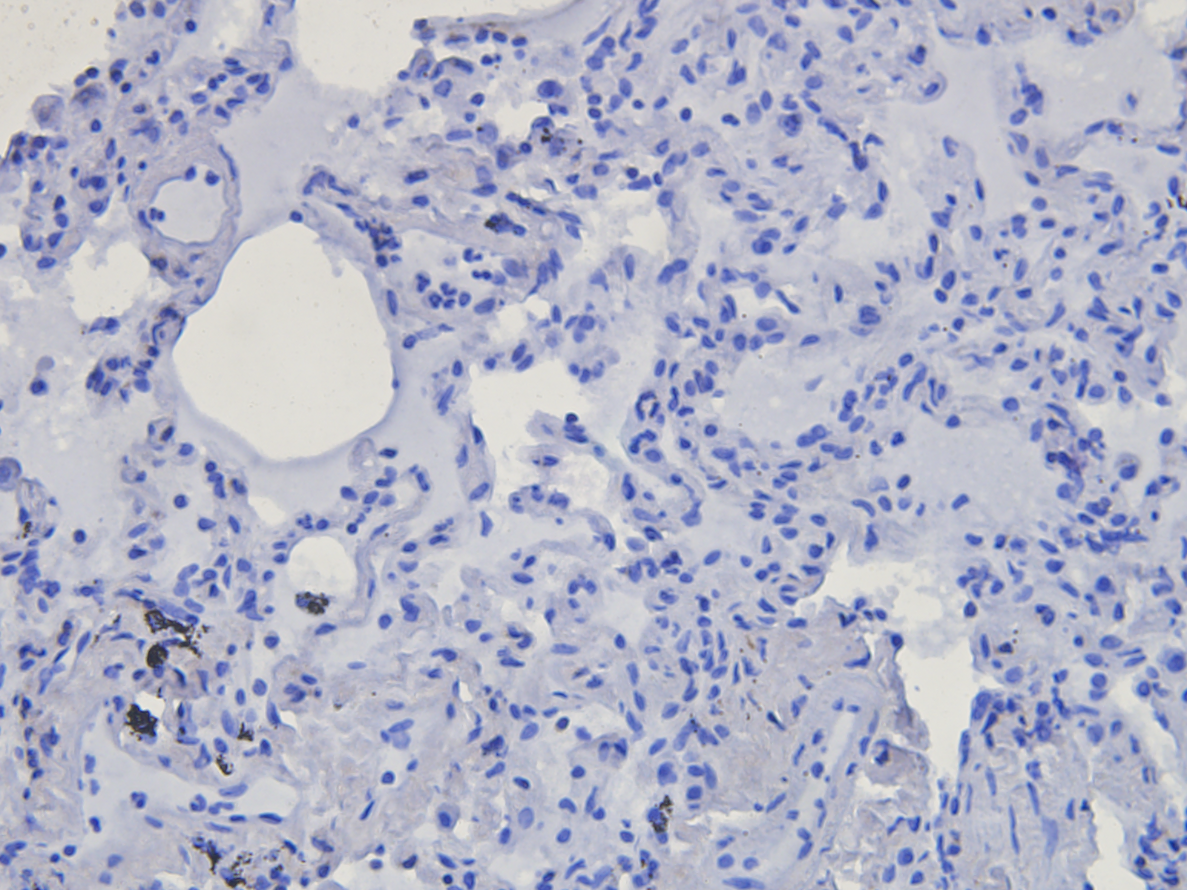

Supplement: S20 File — (ZIP) [file pone.0337223.s021.zip › 465281-400X-N-CA/465281-400X-CA (2).tif]

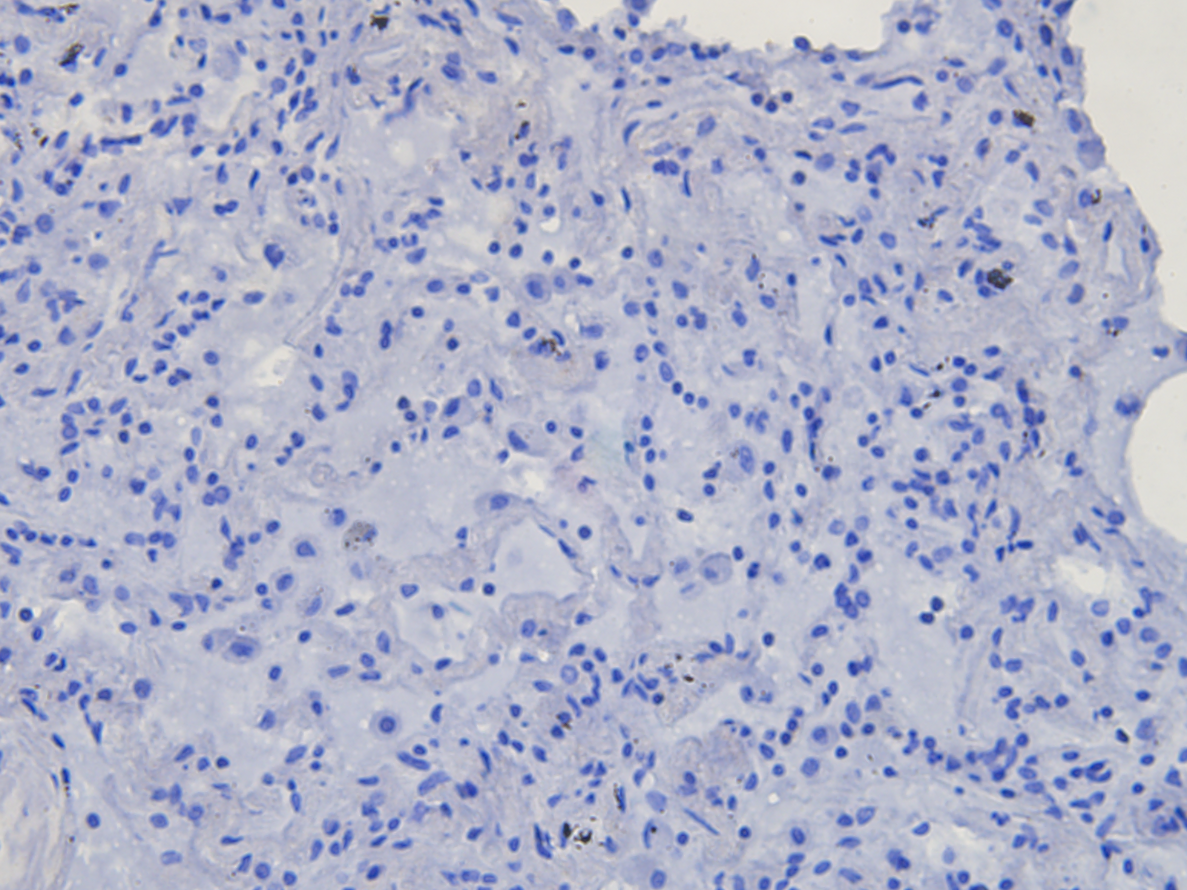

Supplement: S20 File — (ZIP) [file pone.0337223.s021.zip › 465281-400X-N-CA/465281-400X-CA (3).tif]

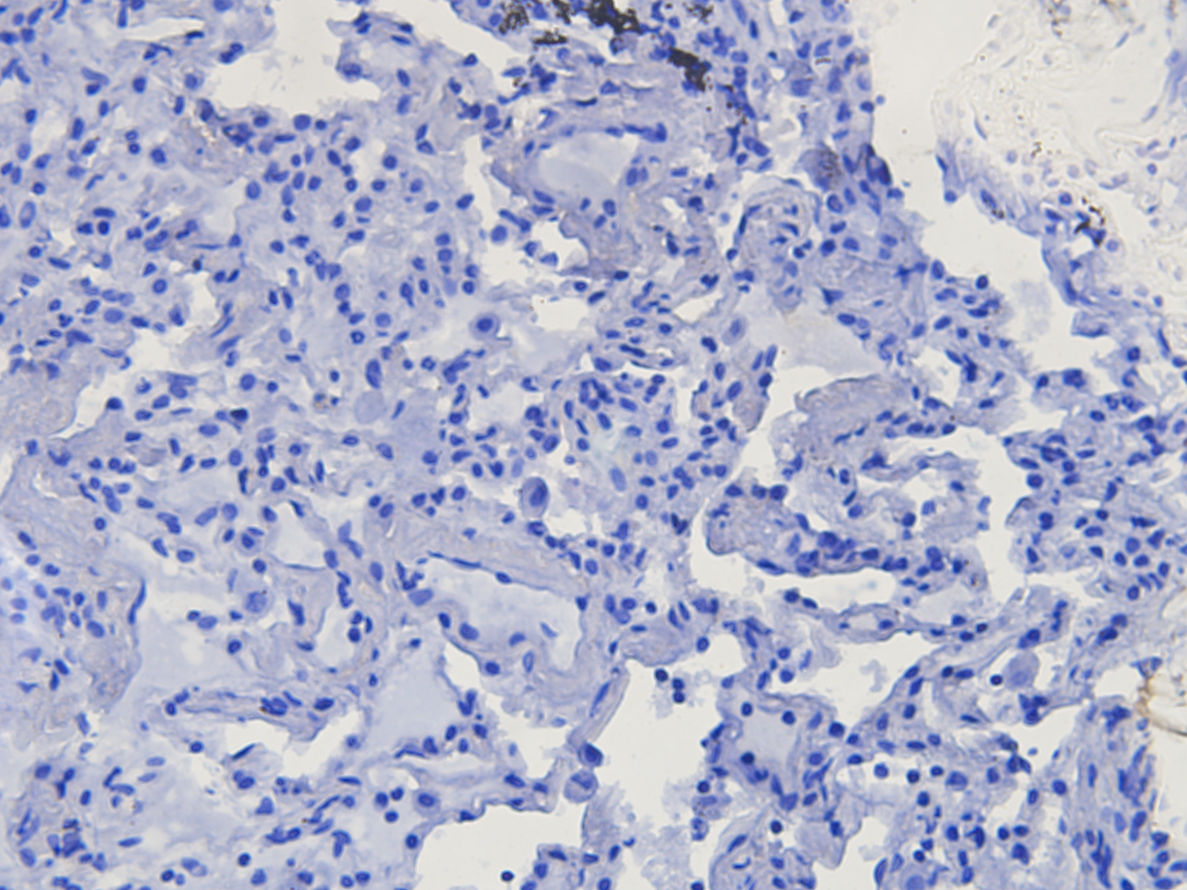

Supplement: S20 File — (ZIP) [file pone.0337223.s021.zip › 465281-400X-N-CA/465281-400X-CA (4).tif]

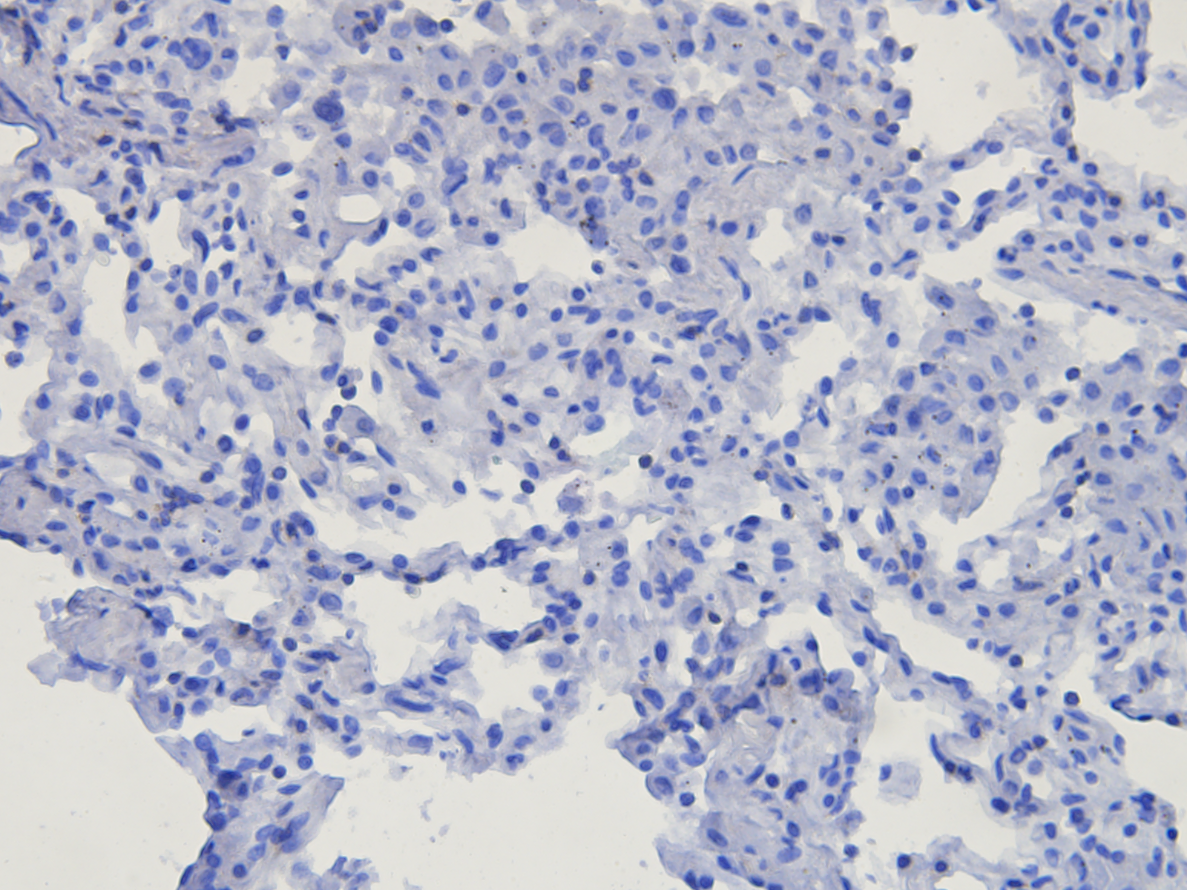

Supplement: S20 File — (ZIP) [file pone.0337223.s021.zip › 465281-400X-N-CA/465281-400X-CA (5).tif]

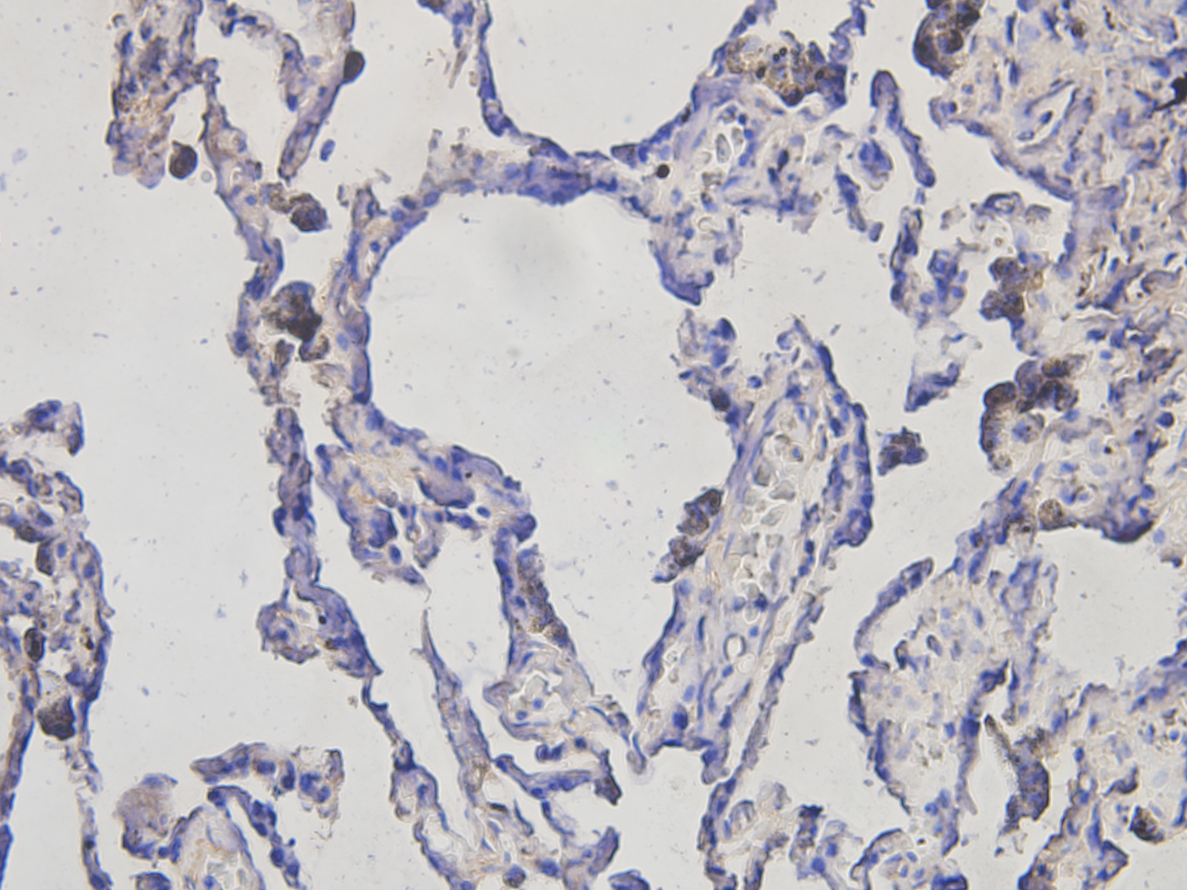

Supplement: S20 File — (ZIP) [file pone.0337223.s021.zip › 465281-400X-N-CA/465281-400X-N (1).tif]

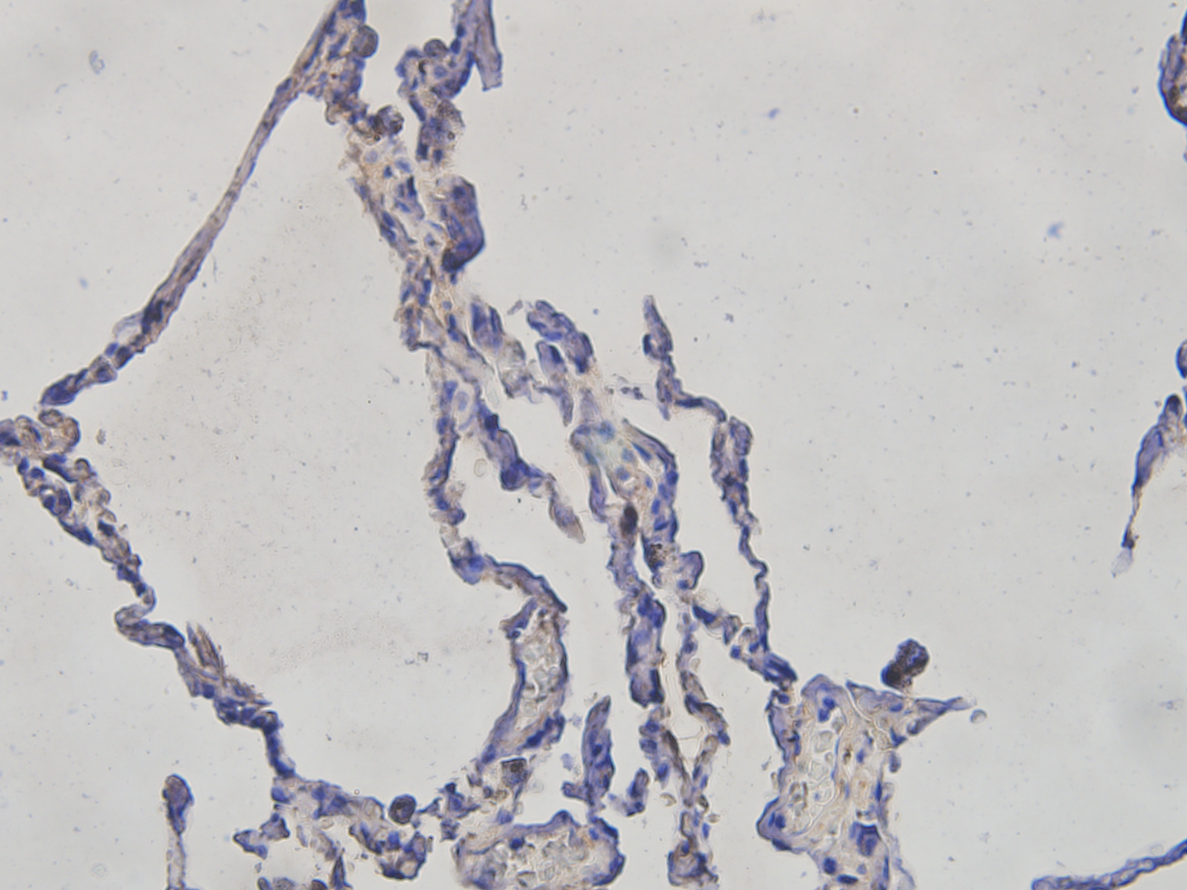

Supplement: S20 File — (ZIP) [file pone.0337223.s021.zip › 465281-400X-N-CA/465281-400X-N (2).tif]

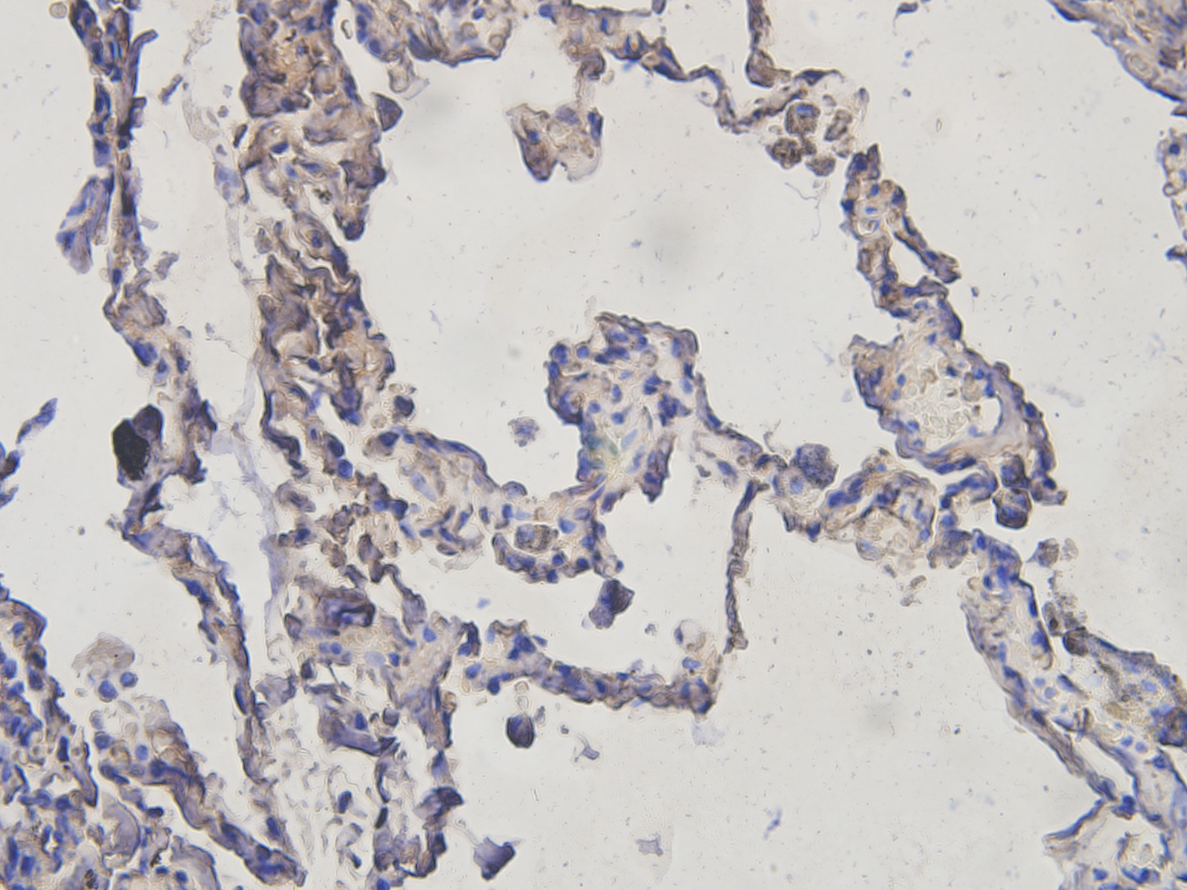

Supplement: S20 File — (ZIP) [file pone.0337223.s021.zip › 465281-400X-N-CA/465281-400X-N (3).tif]

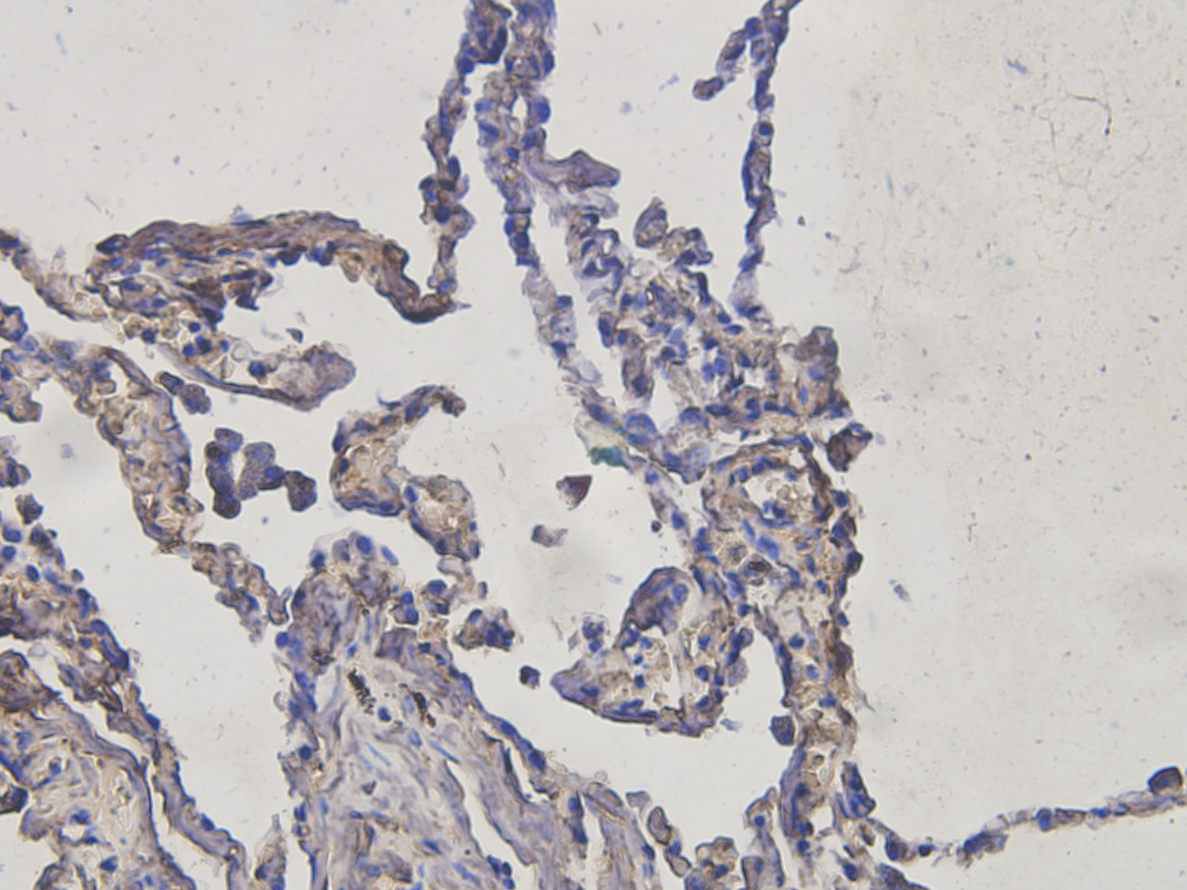

Supplement: S20 File — (ZIP) [file pone.0337223.s021.zip › 465281-400X-N-CA/465281-400X-N (4).tif]

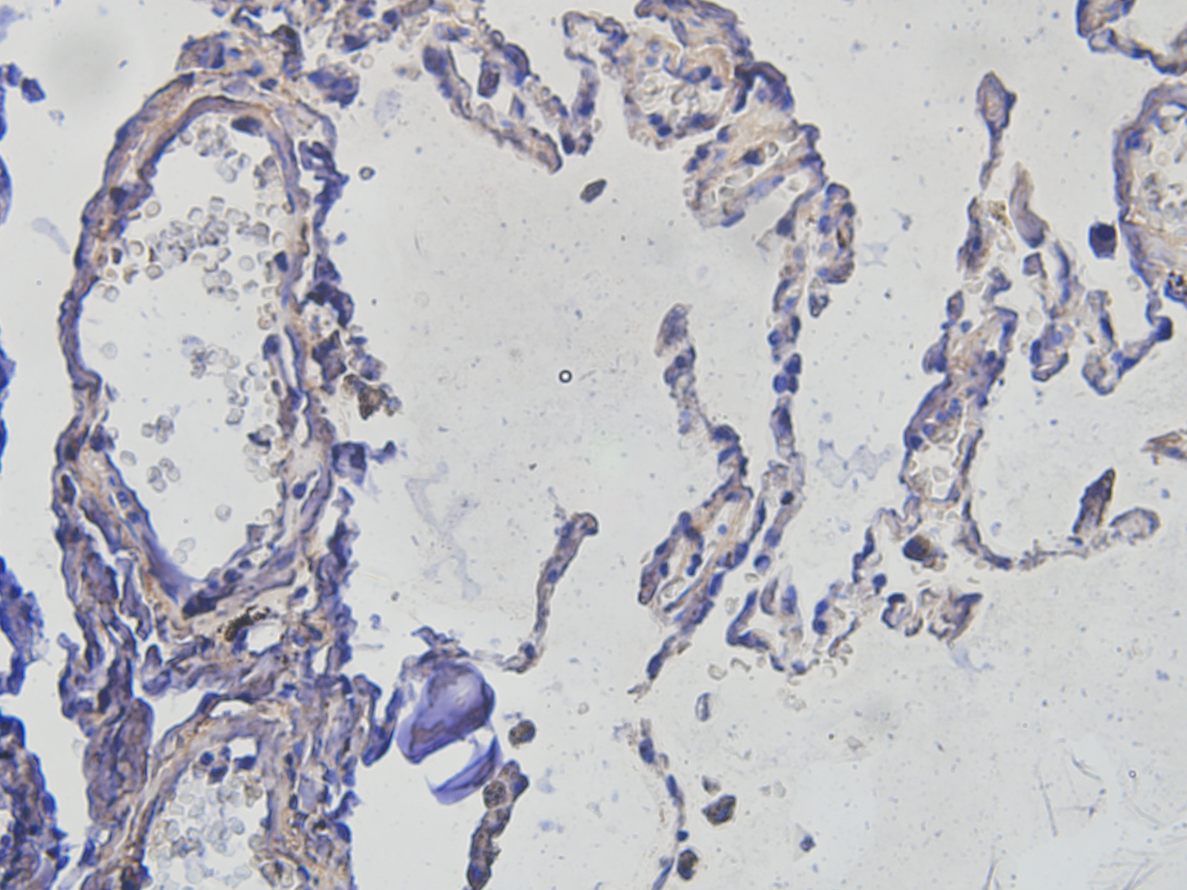

Supplement: S20 File — (ZIP) [file pone.0337223.s021.zip › 465281-400X-N-CA/465281-400X-N (5).tif]

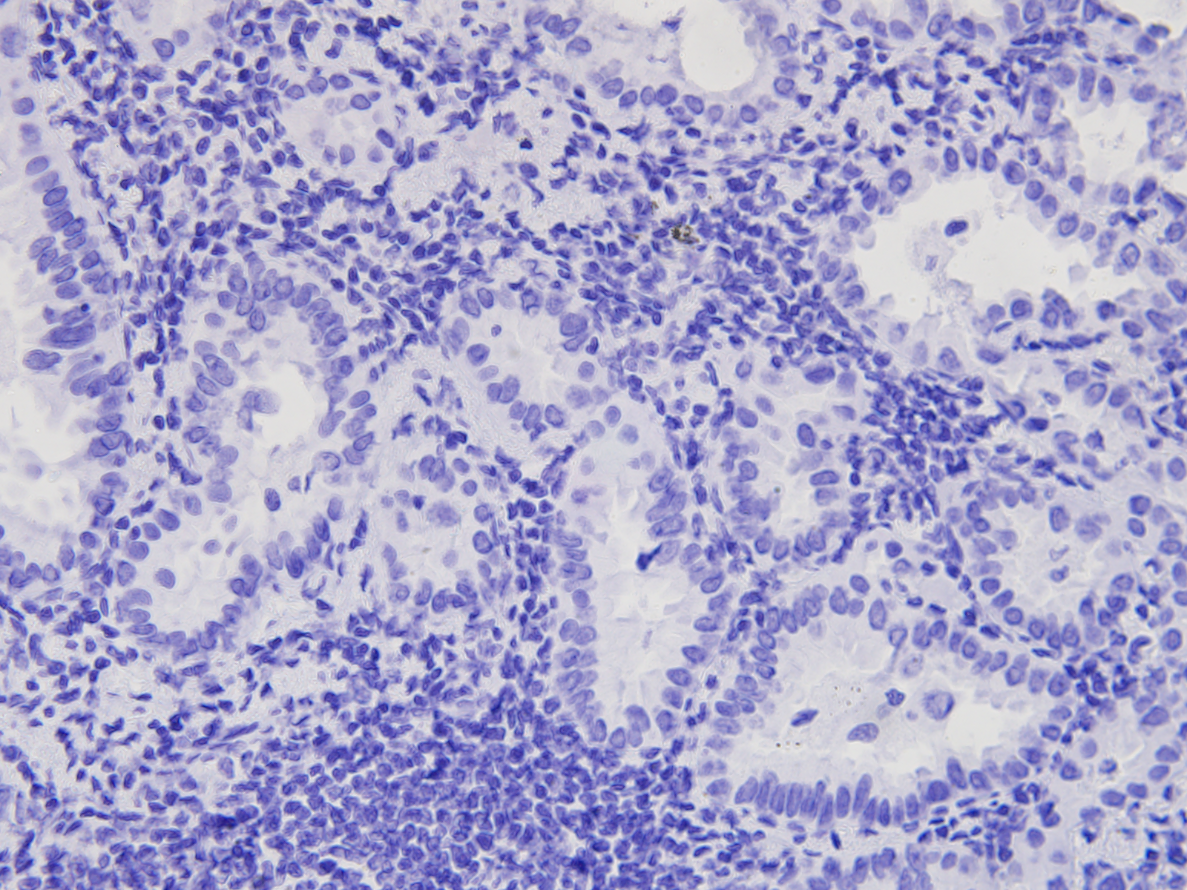

Supplement: S21 File — (ZIP) [file pone.0337223.s022.zip › 465866-400X-CA-N/465866-400X-CA (1).tif]

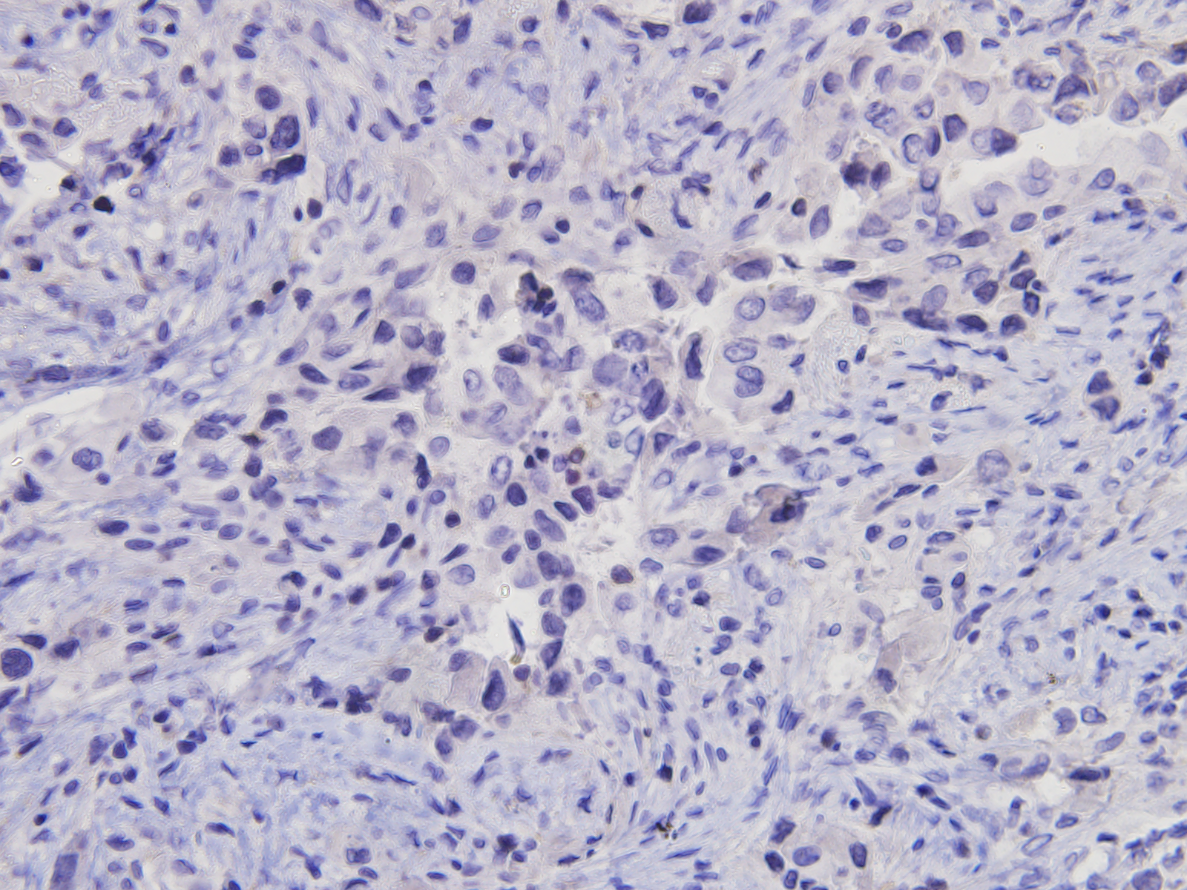

Supplement: S21 File — (ZIP) [file pone.0337223.s022.zip › 465866-400X-CA-N/465866-400X-CA (2).tif]

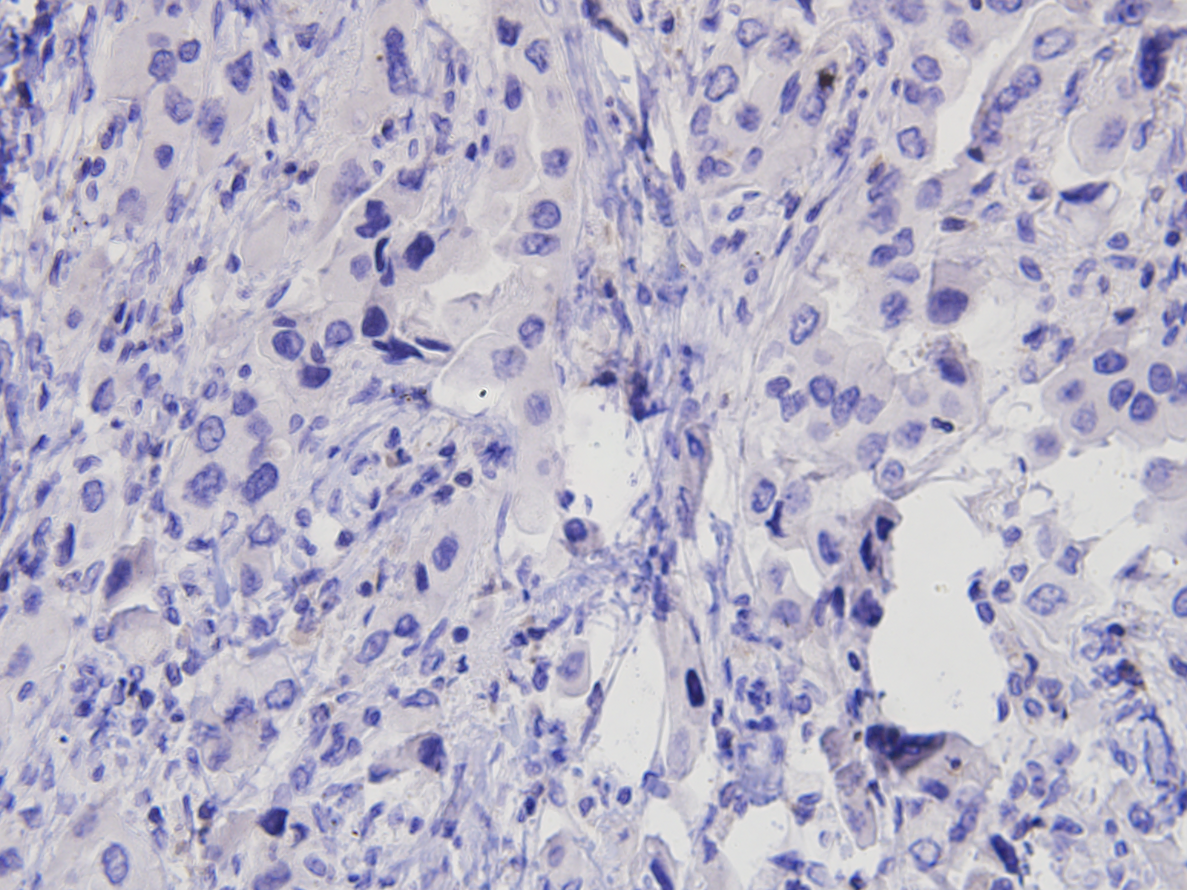

Supplement: S21 File — (ZIP) [file pone.0337223.s022.zip › 465866-400X-CA-N/465866-400X-CA (3).tif]

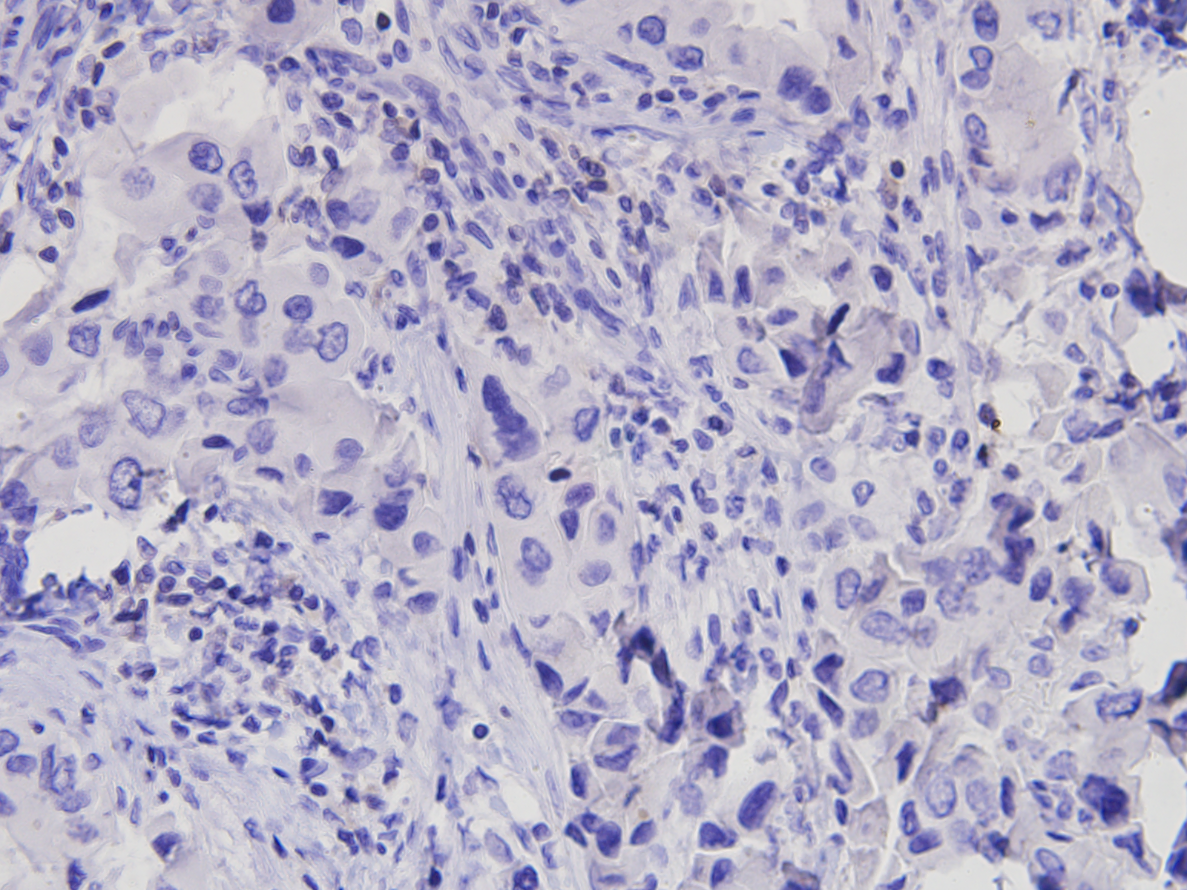

Supplement: S21 File — (ZIP) [file pone.0337223.s022.zip › 465866-400X-CA-N/465866-400X-CA (4).tif]

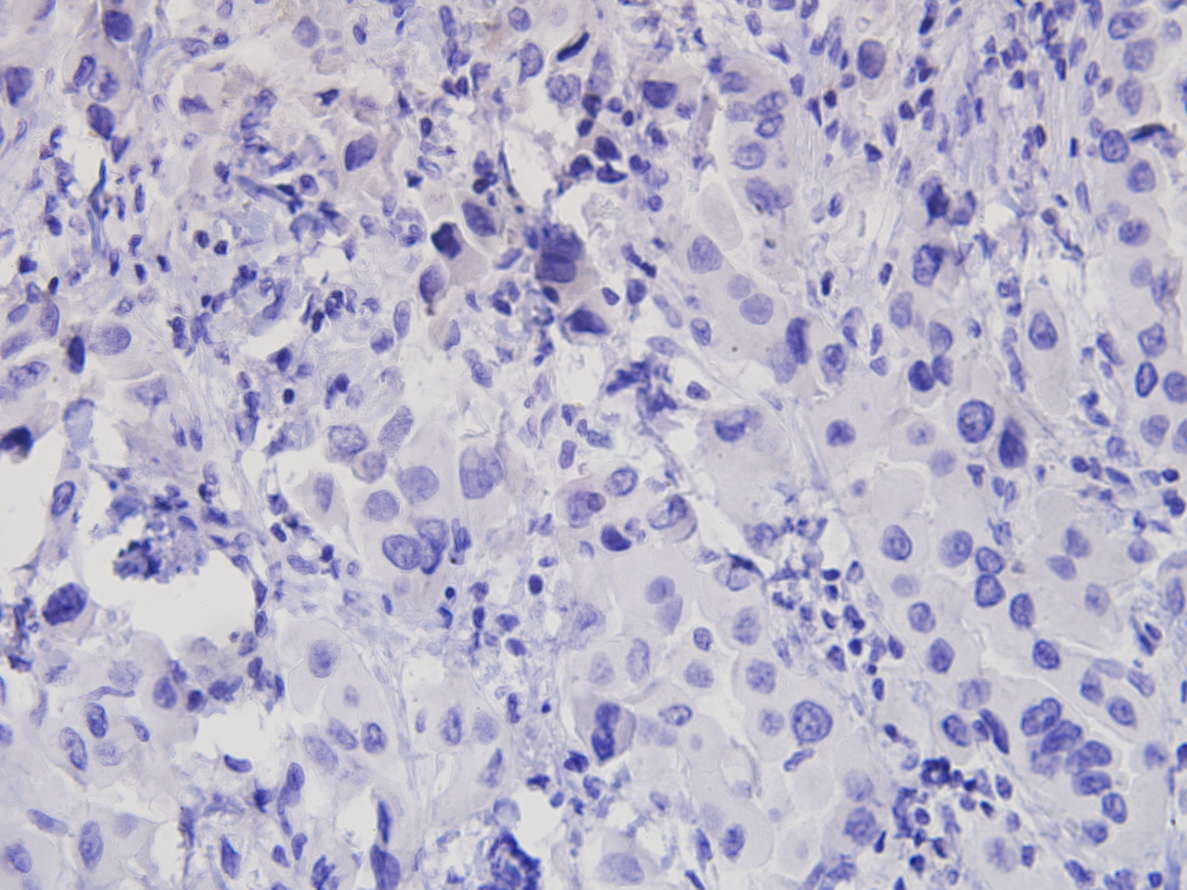

Supplement: S21 File — (ZIP) [file pone.0337223.s022.zip › 465866-400X-CA-N/465866-400X-CA (5).tif]

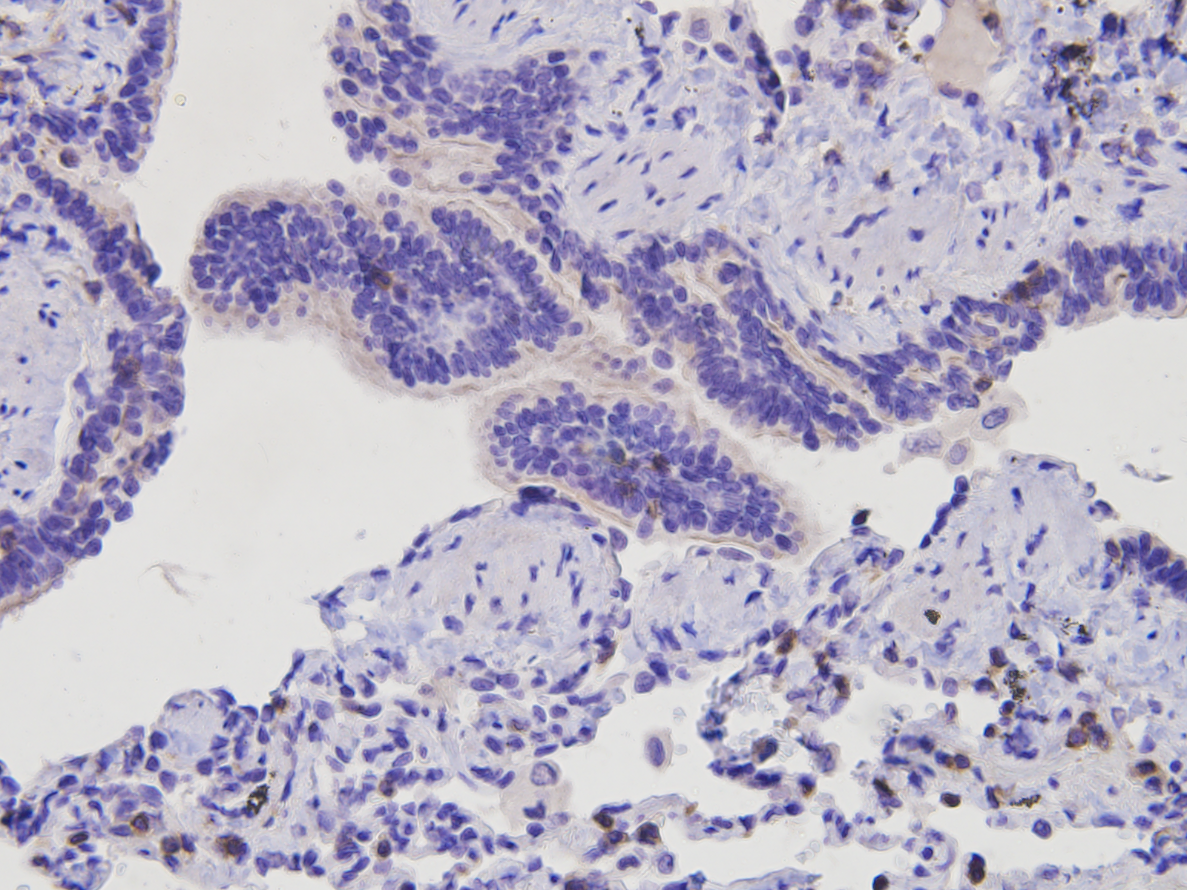

Supplement: S21 File — (ZIP) [file pone.0337223.s022.zip › 465866-400X-CA-N/465866-400X-N (1).tif]

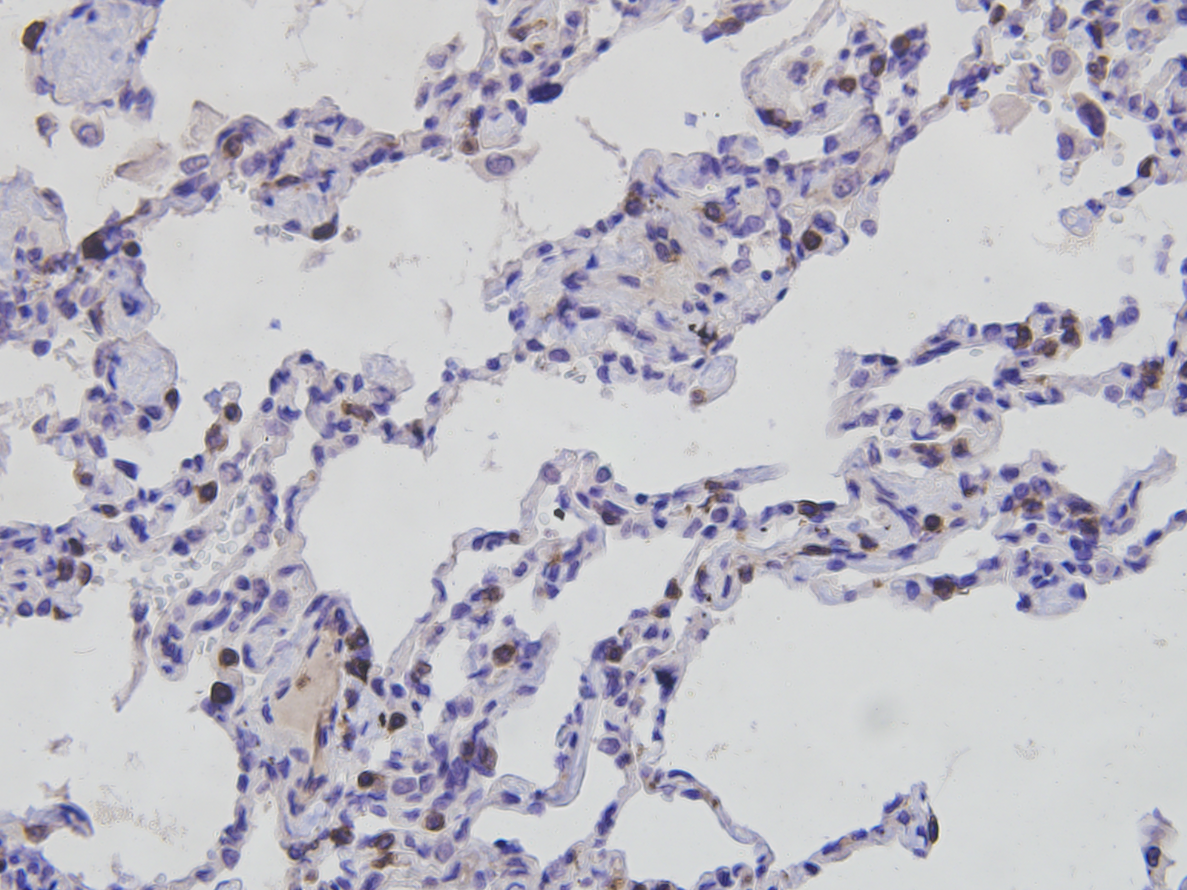

Supplement: S21 File — (ZIP) [file pone.0337223.s022.zip › 465866-400X-CA-N/465866-400X-N (2).tif]

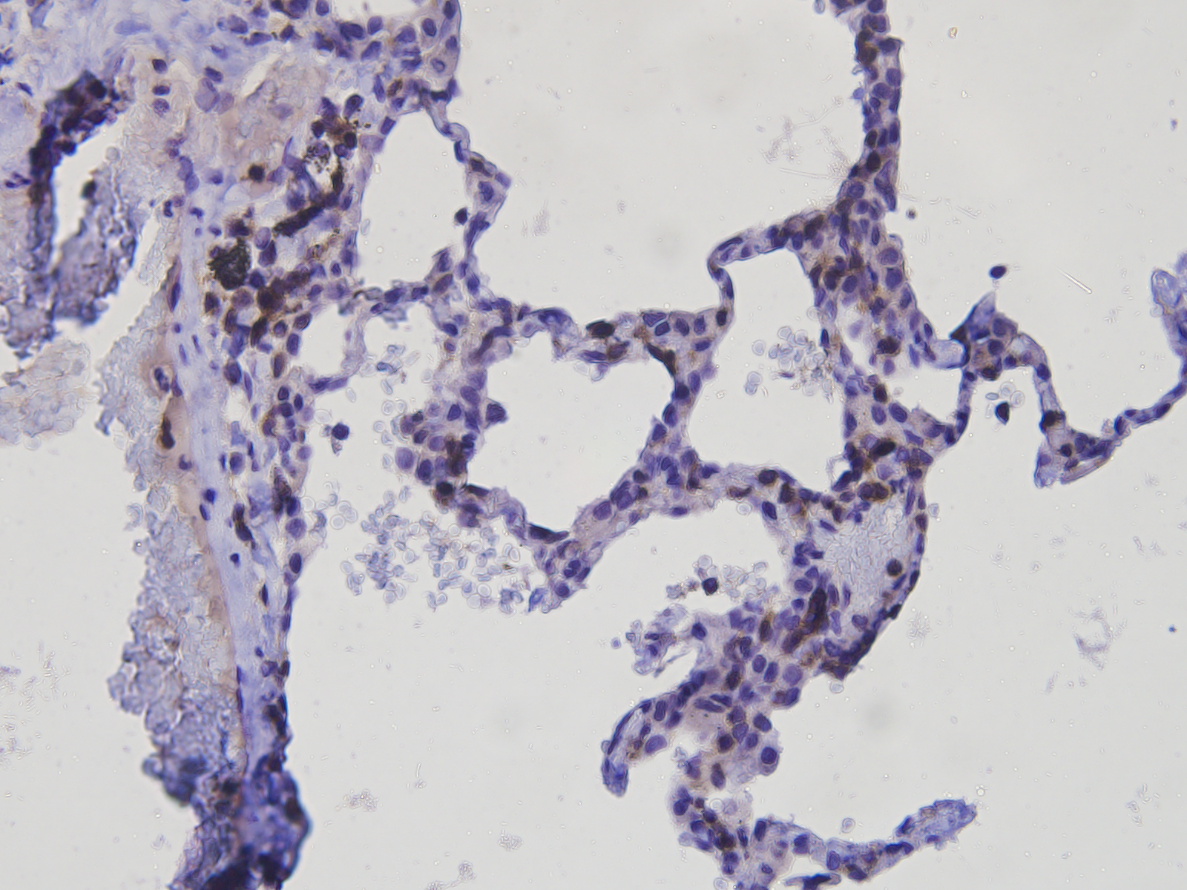

Supplement: S21 File — (ZIP) [file pone.0337223.s022.zip › 465866-400X-CA-N/465866-400X-N (3).tif]

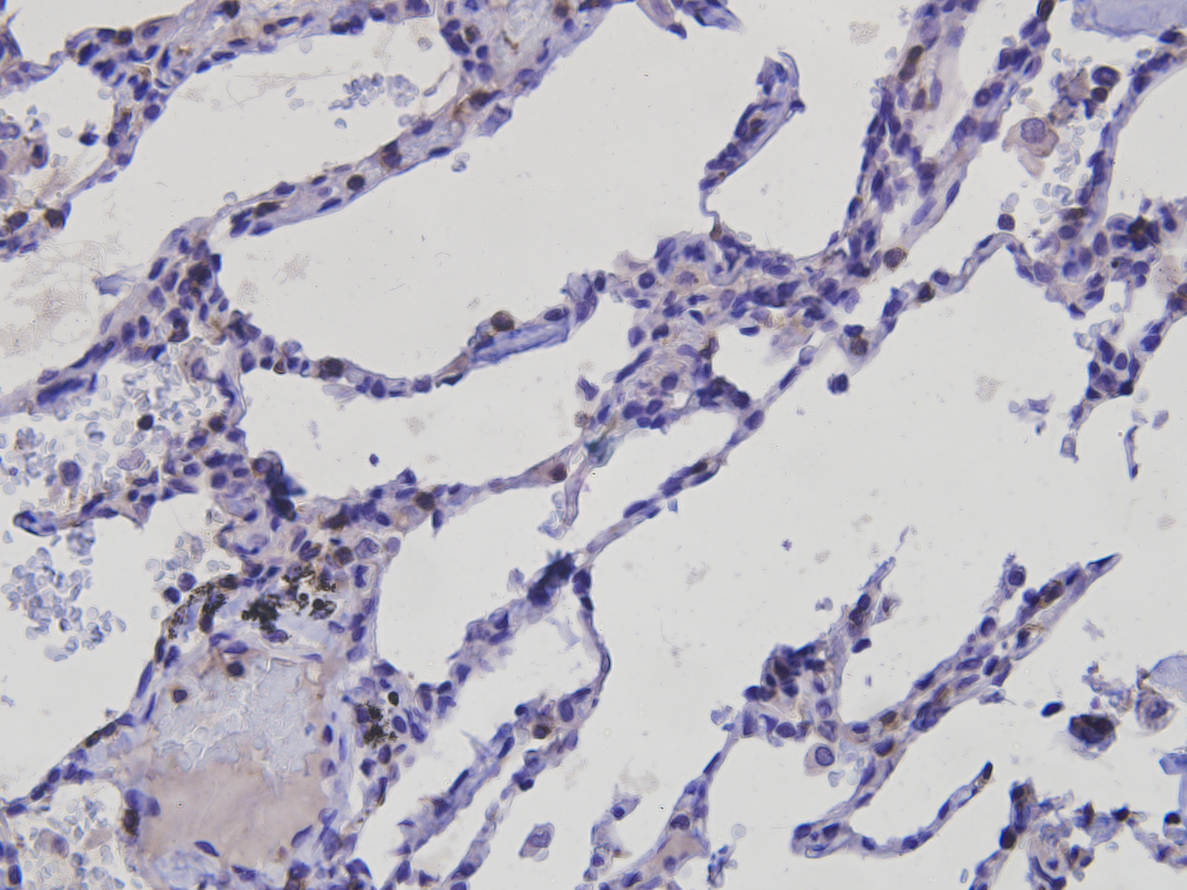

Supplement: S21 File — (ZIP) [file pone.0337223.s022.zip › 465866-400X-CA-N/465866-400X-N (4).tif]

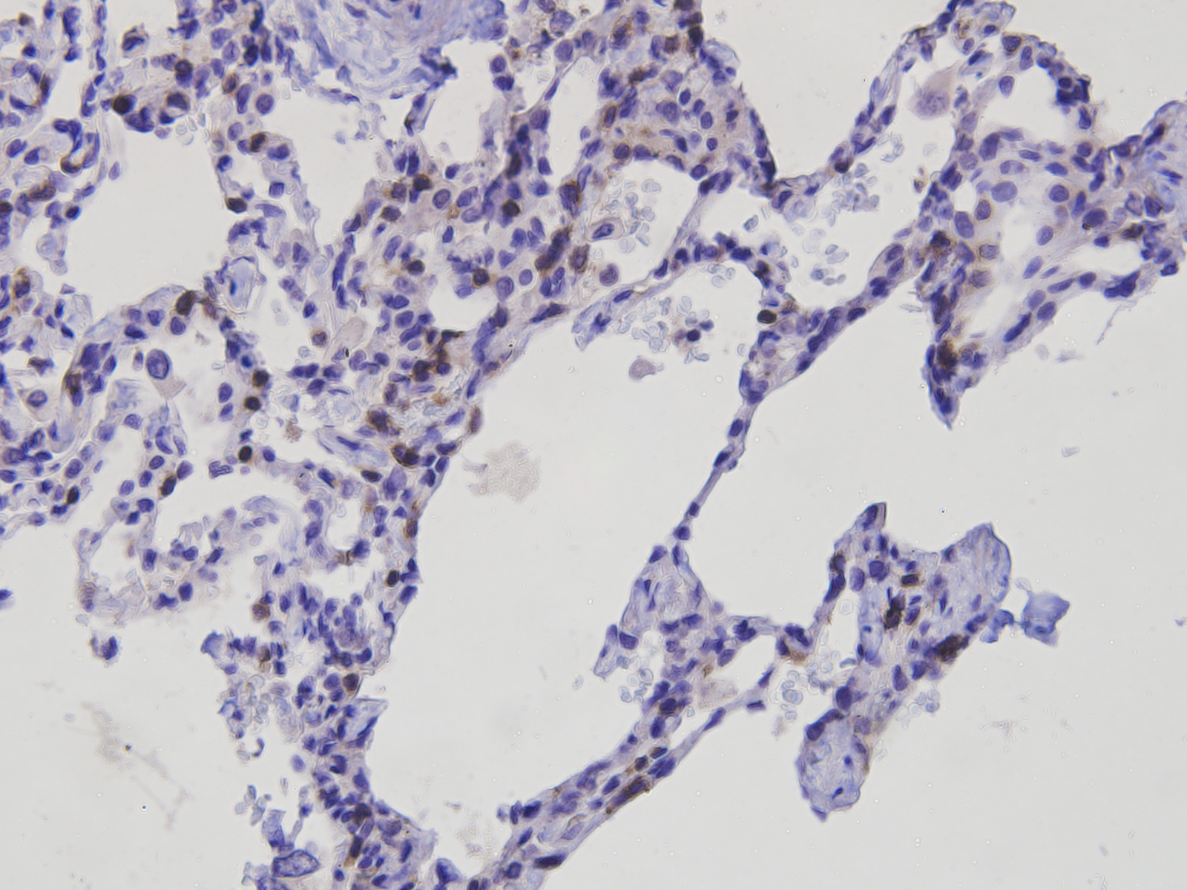

Supplement: S21 File — (ZIP) [file pone.0337223.s022.zip › 465866-400X-CA-N/465866-400X-N (5).tif]

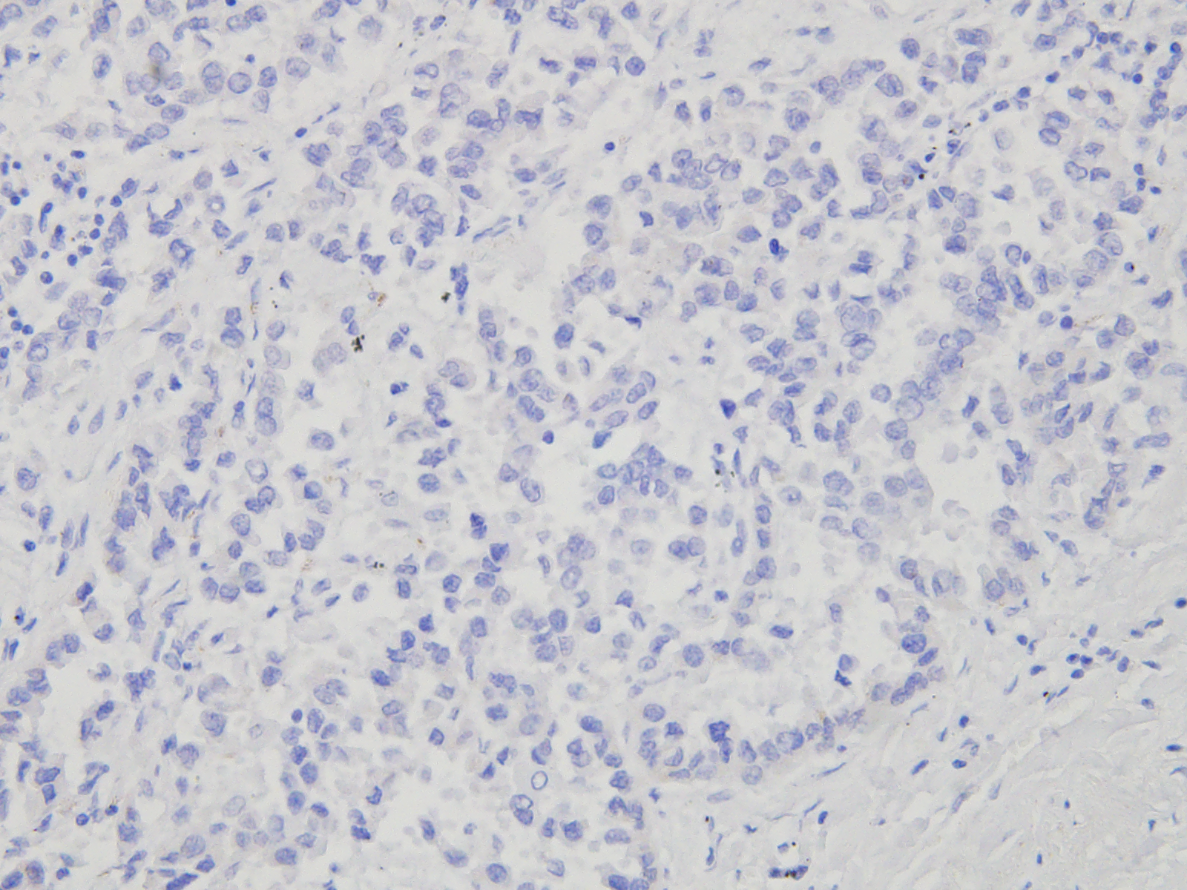

Supplement: S22 File — (ZIP) [file pone.0337223.s023.zip › 467204-400x-CA-N/467204-400x-CA (1).tif]

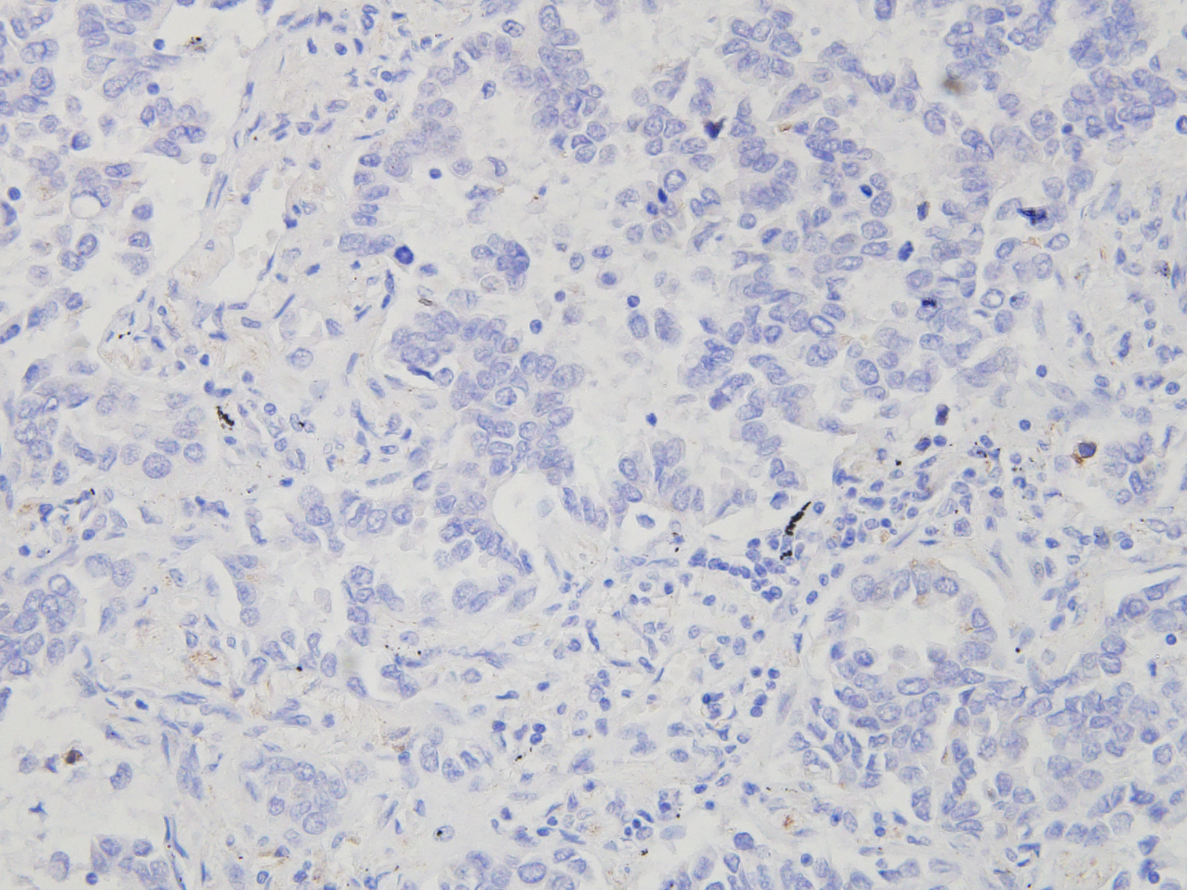

Supplement: S22 File — (ZIP) [file pone.0337223.s023.zip › 467204-400x-CA-N/467204-400x-CA (2).tif]

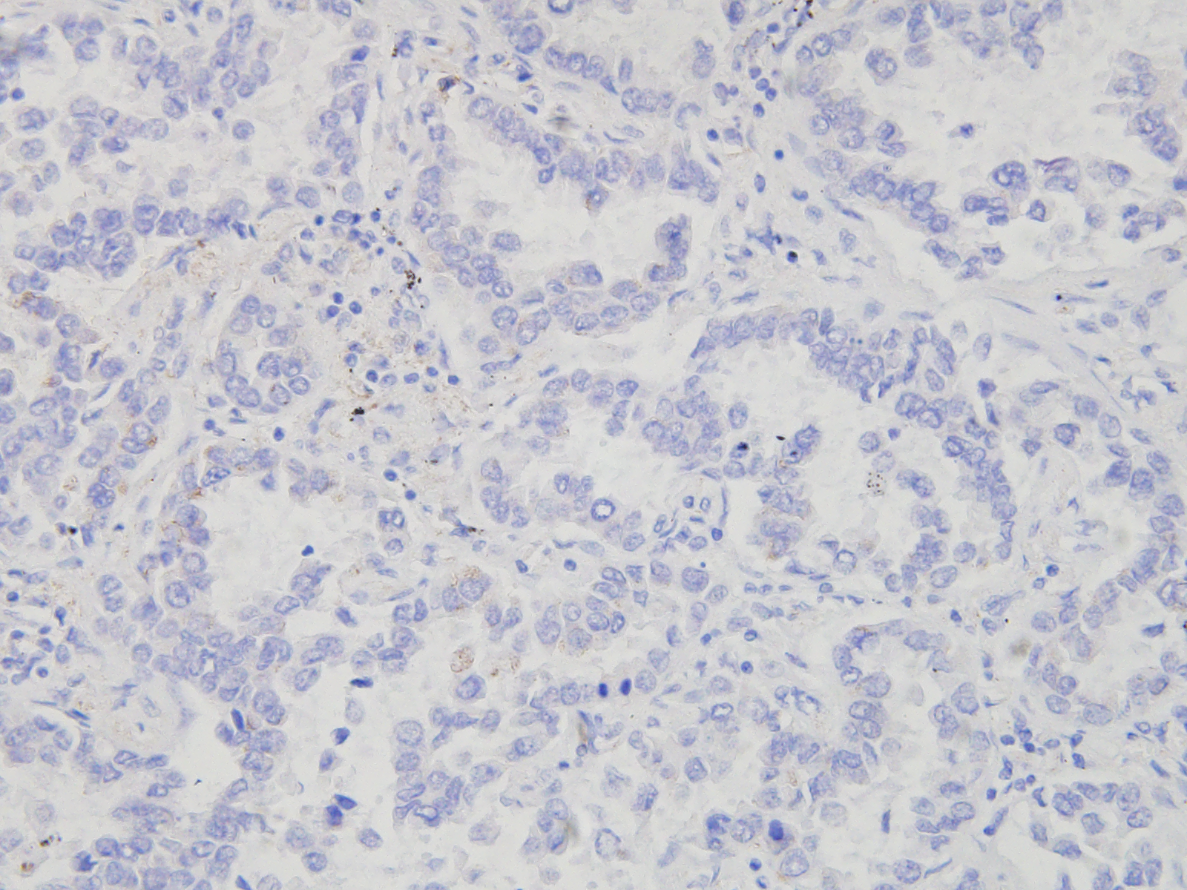

Supplement: S22 File — (ZIP) [file pone.0337223.s023.zip › 467204-400x-CA-N/467204-400x-CA (3).tif]

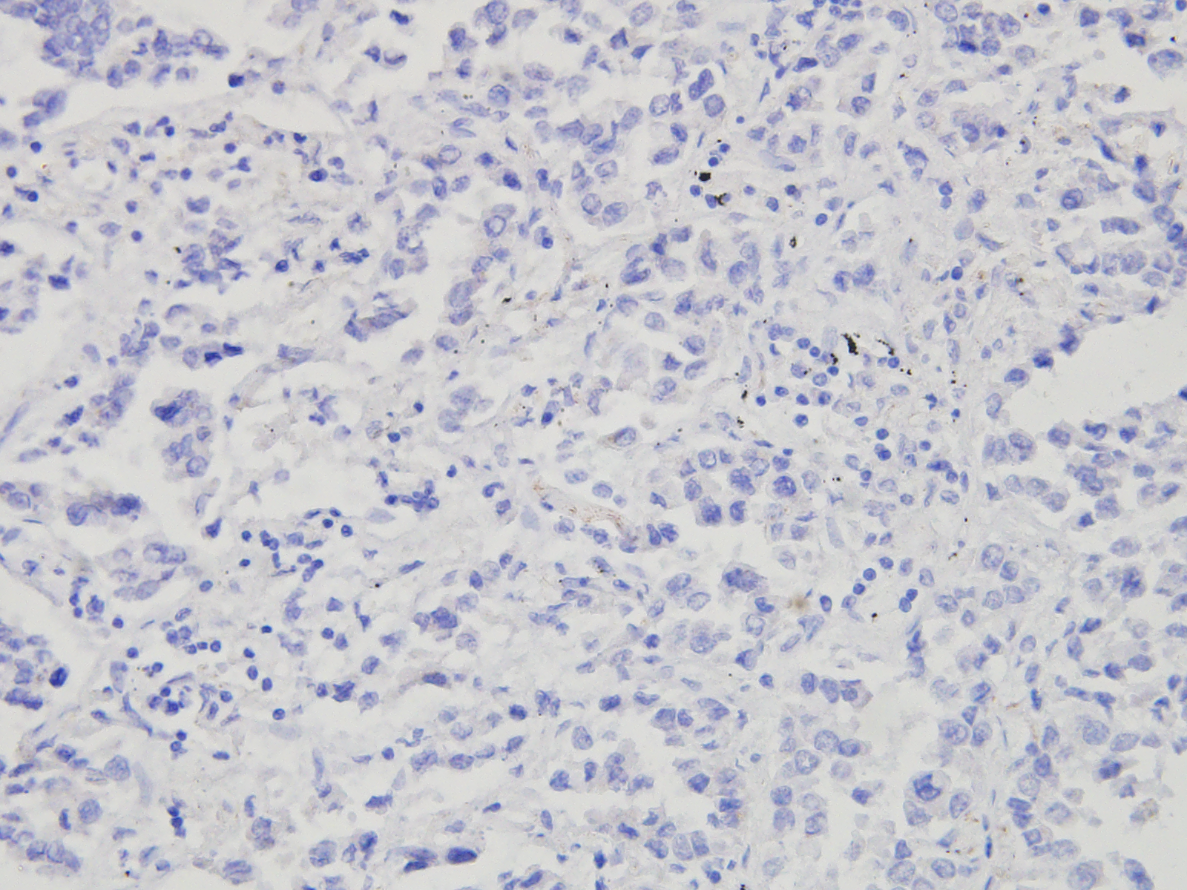

Supplement: S22 File — (ZIP) [file pone.0337223.s023.zip › 467204-400x-CA-N/467204-400x-CA (4).tif]

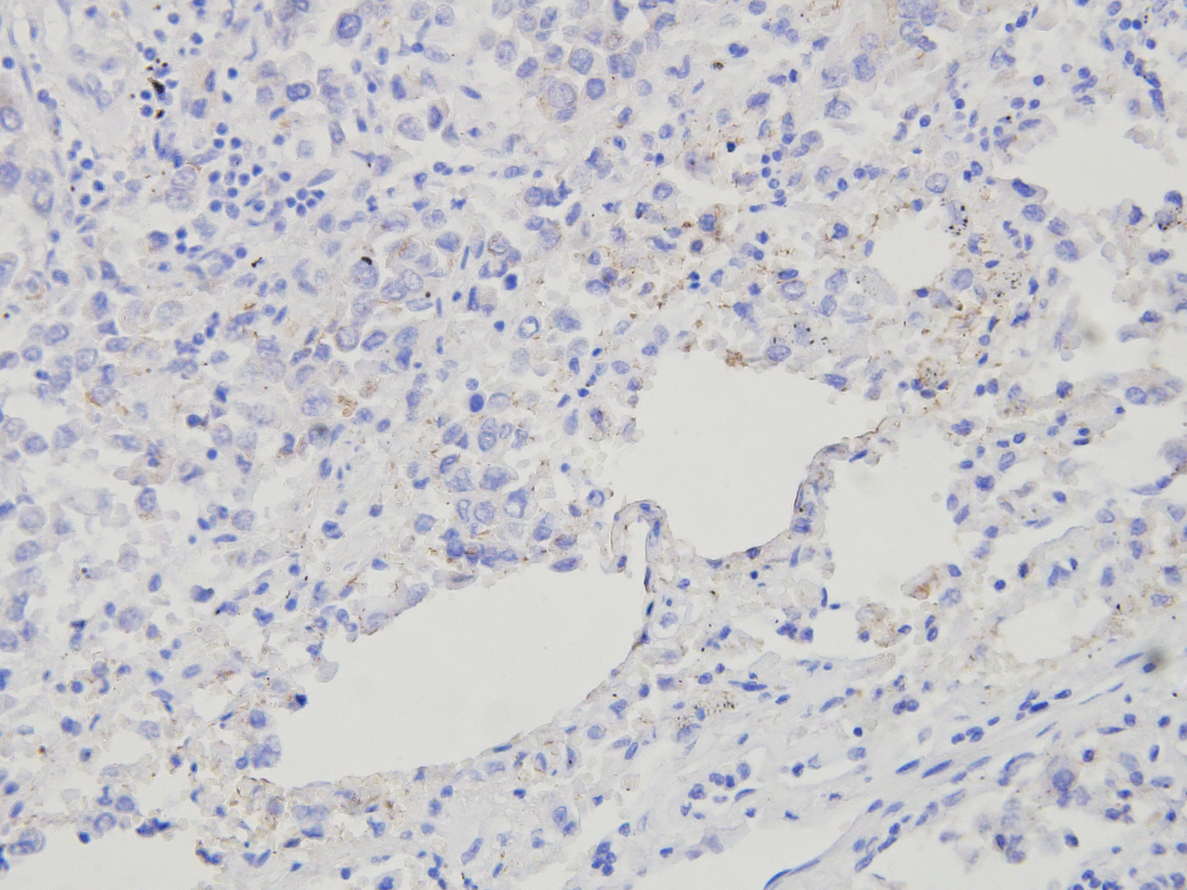

Supplement: S22 File — (ZIP) [file pone.0337223.s023.zip › 467204-400x-CA-N/467204-400x-CA (5).tif]

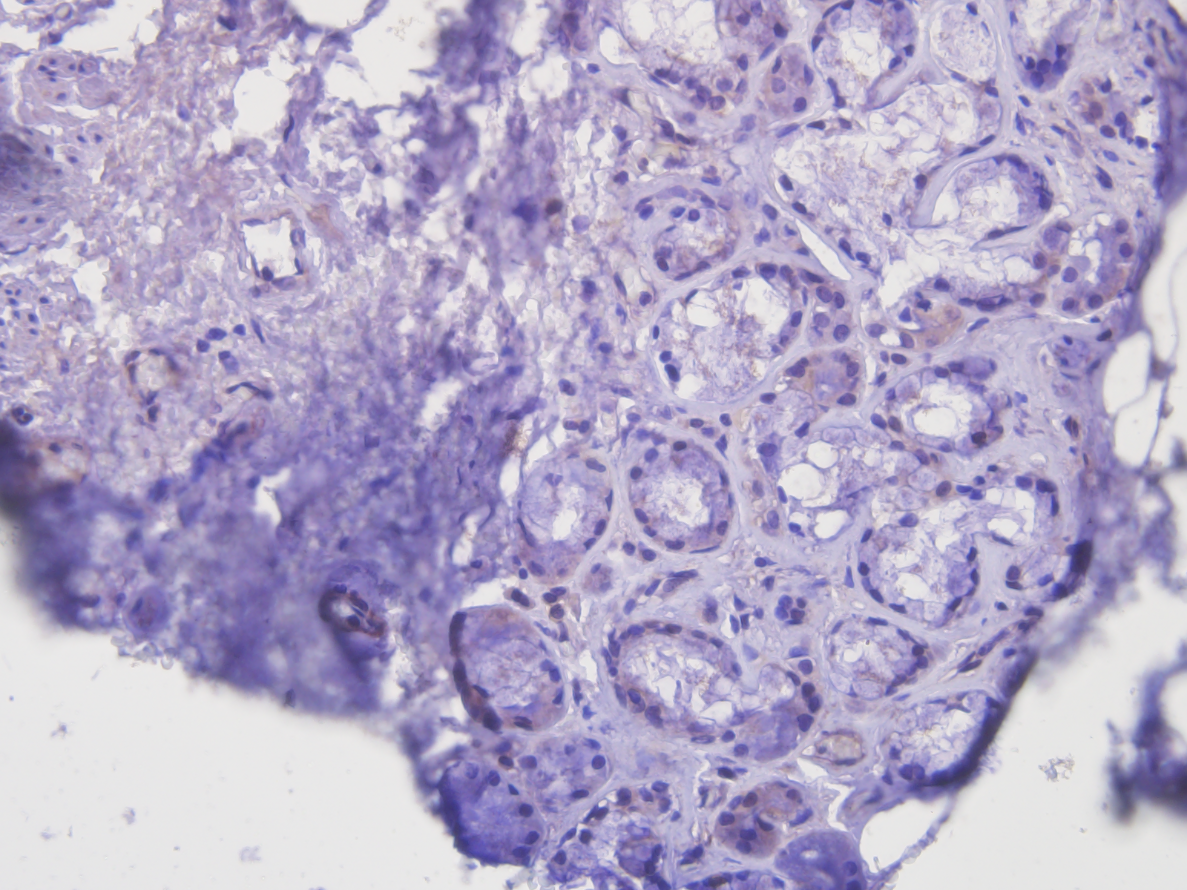

Supplement: S22 File — (ZIP) [file pone.0337223.s023.zip › 467204-400x-CA-N/467204-400x-N (1).tif]

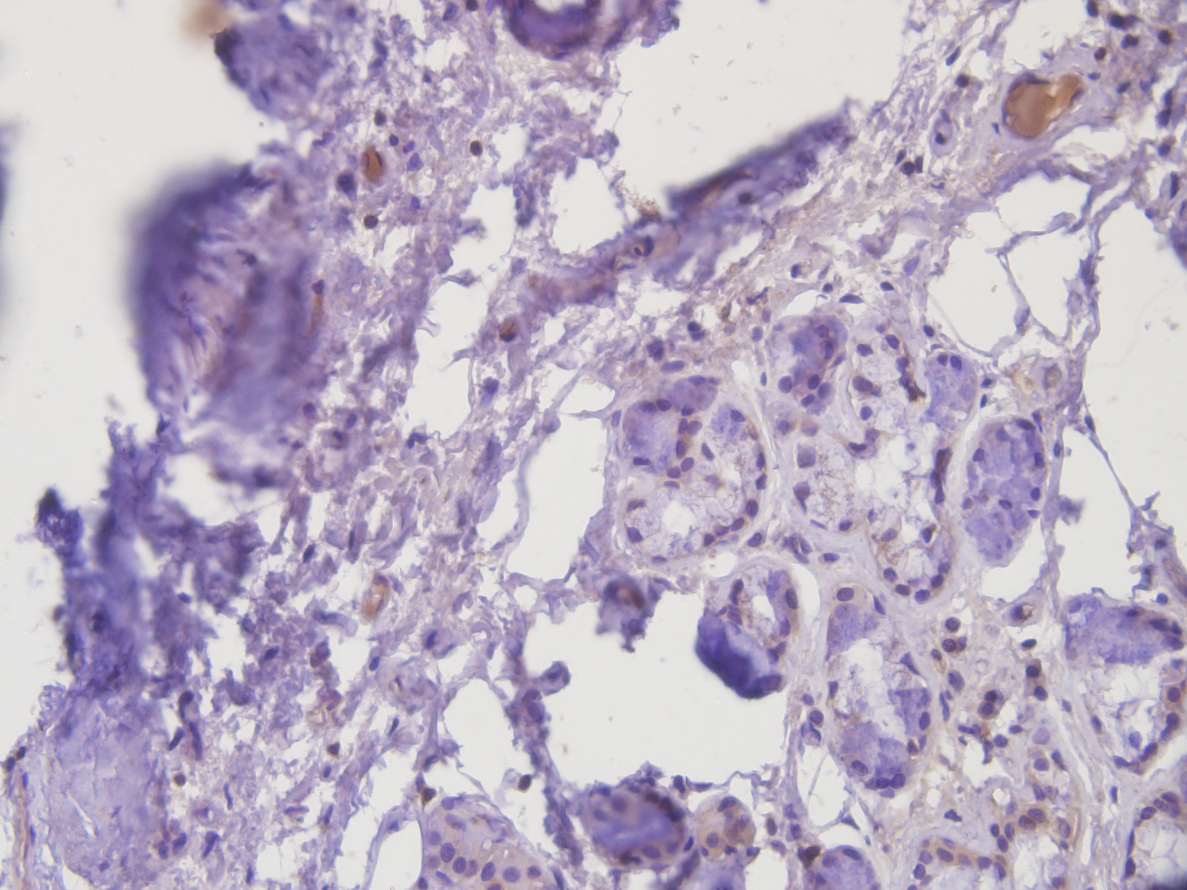

Supplement: S22 File — (ZIP) [file pone.0337223.s023.zip › 467204-400x-CA-N/467204-400x-N (2).tif]

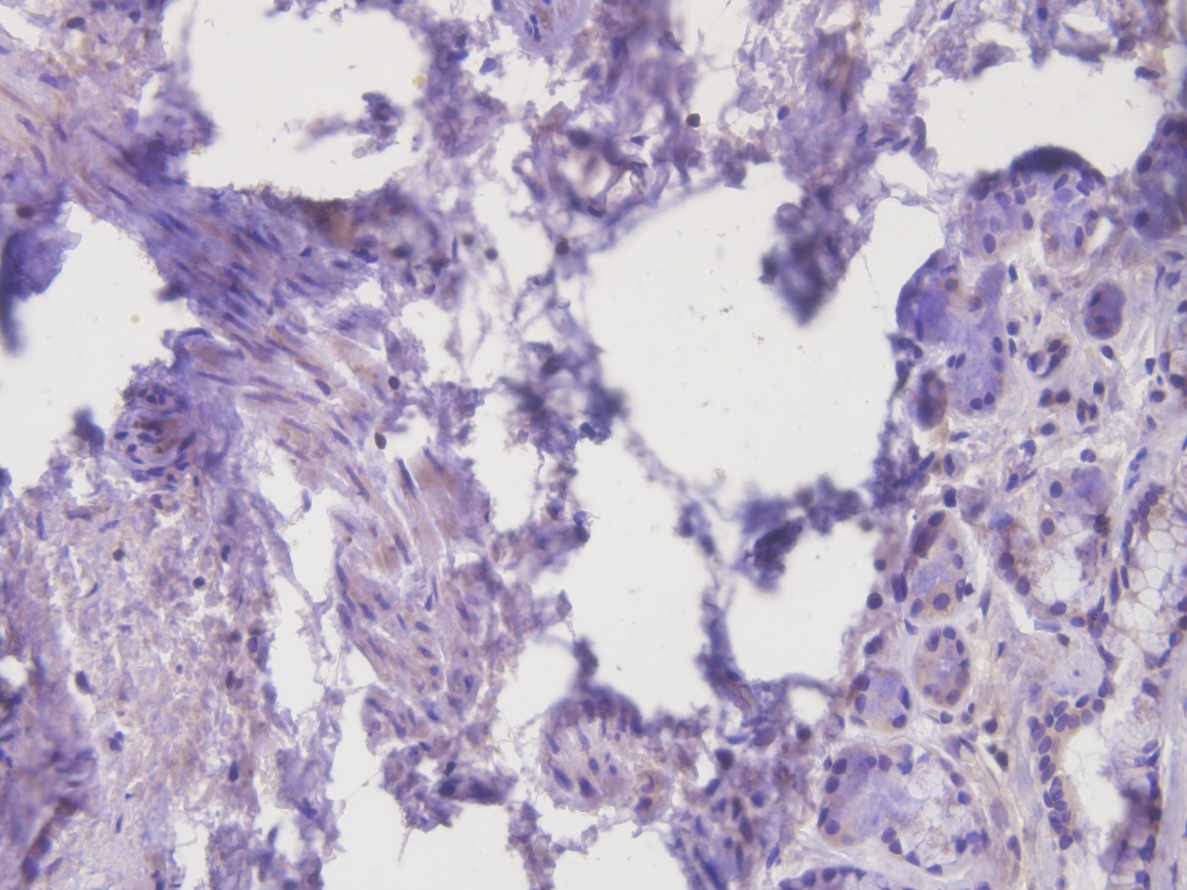

Supplement: S22 File — (ZIP) [file pone.0337223.s023.zip › 467204-400x-CA-N/467204-400x-N (3).tif]

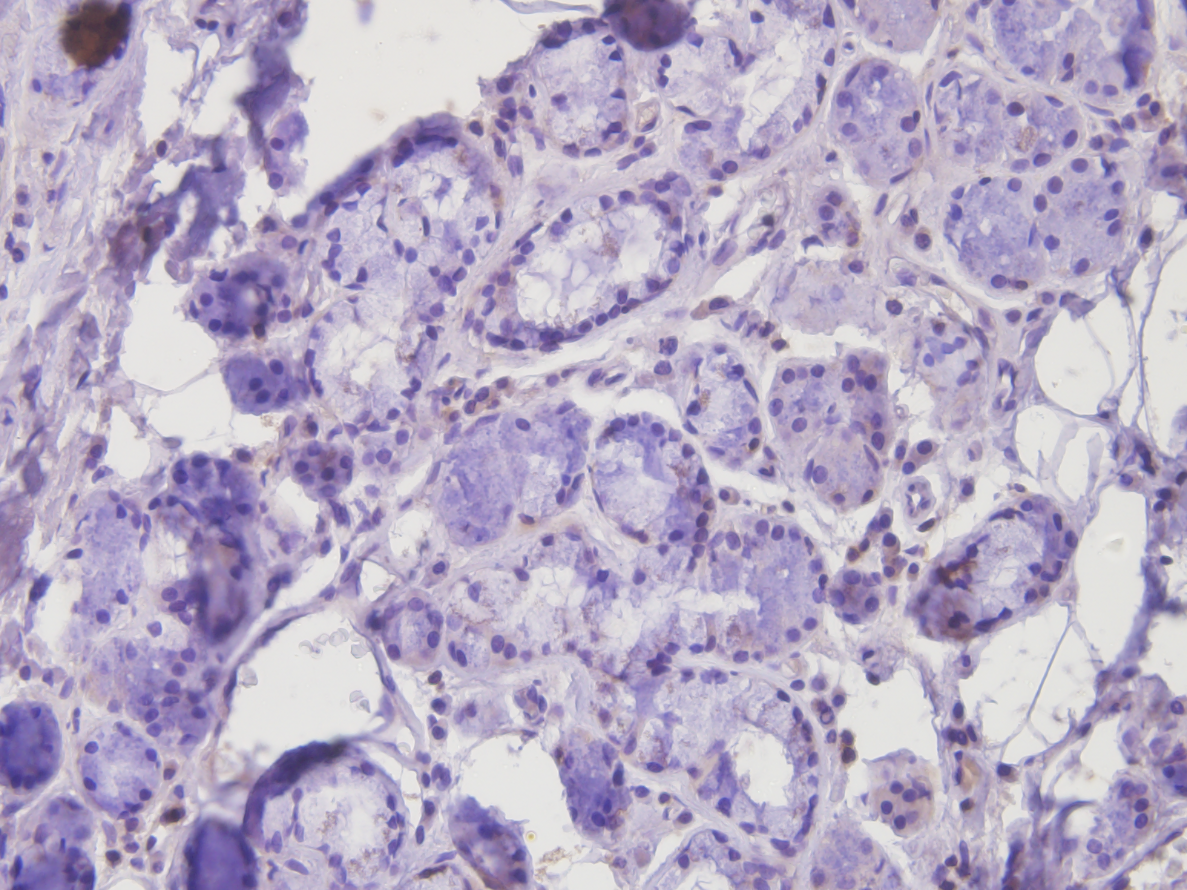

Supplement: S22 File — (ZIP) [file pone.0337223.s023.zip › 467204-400x-CA-N/467204-400x-N (4).tif]

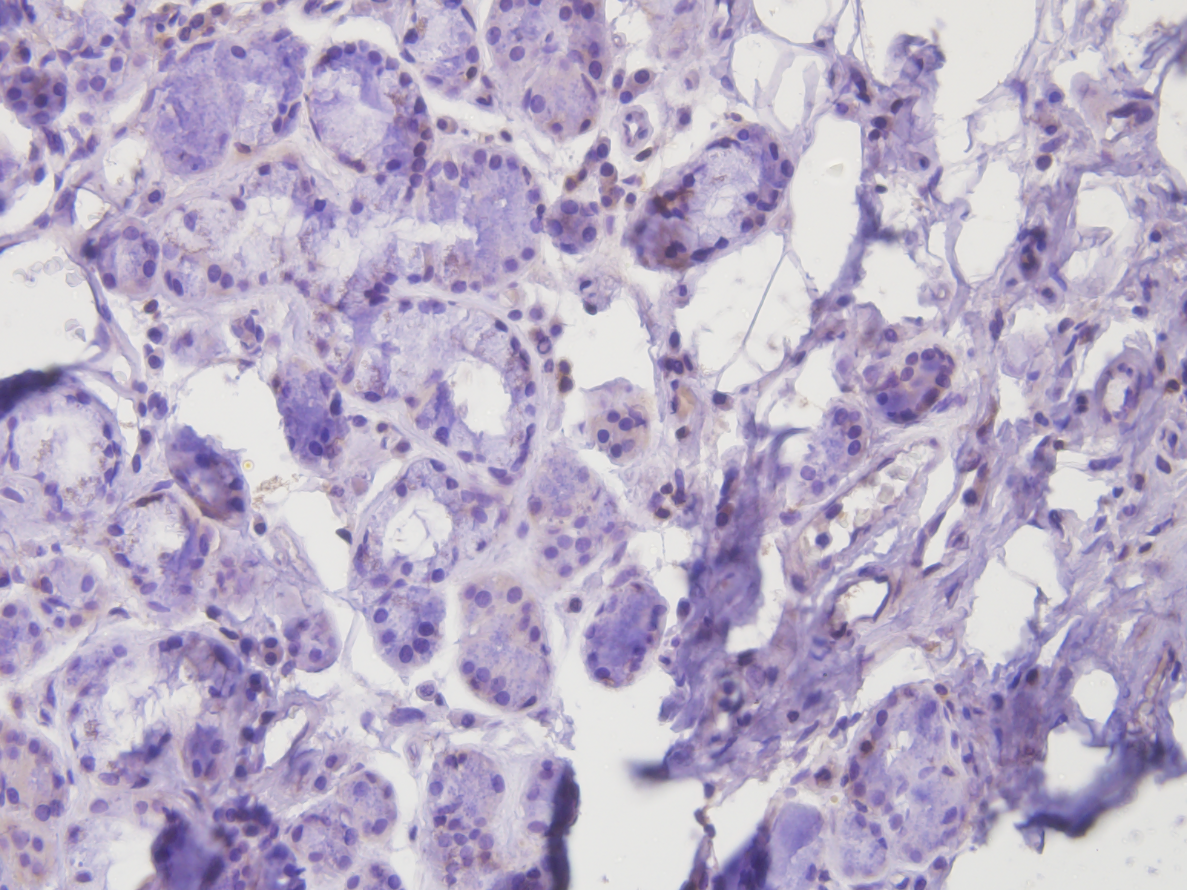

Supplement: S22 File — (ZIP) [file pone.0337223.s023.zip › 467204-400x-CA-N/467204-400x-N (5).tif]

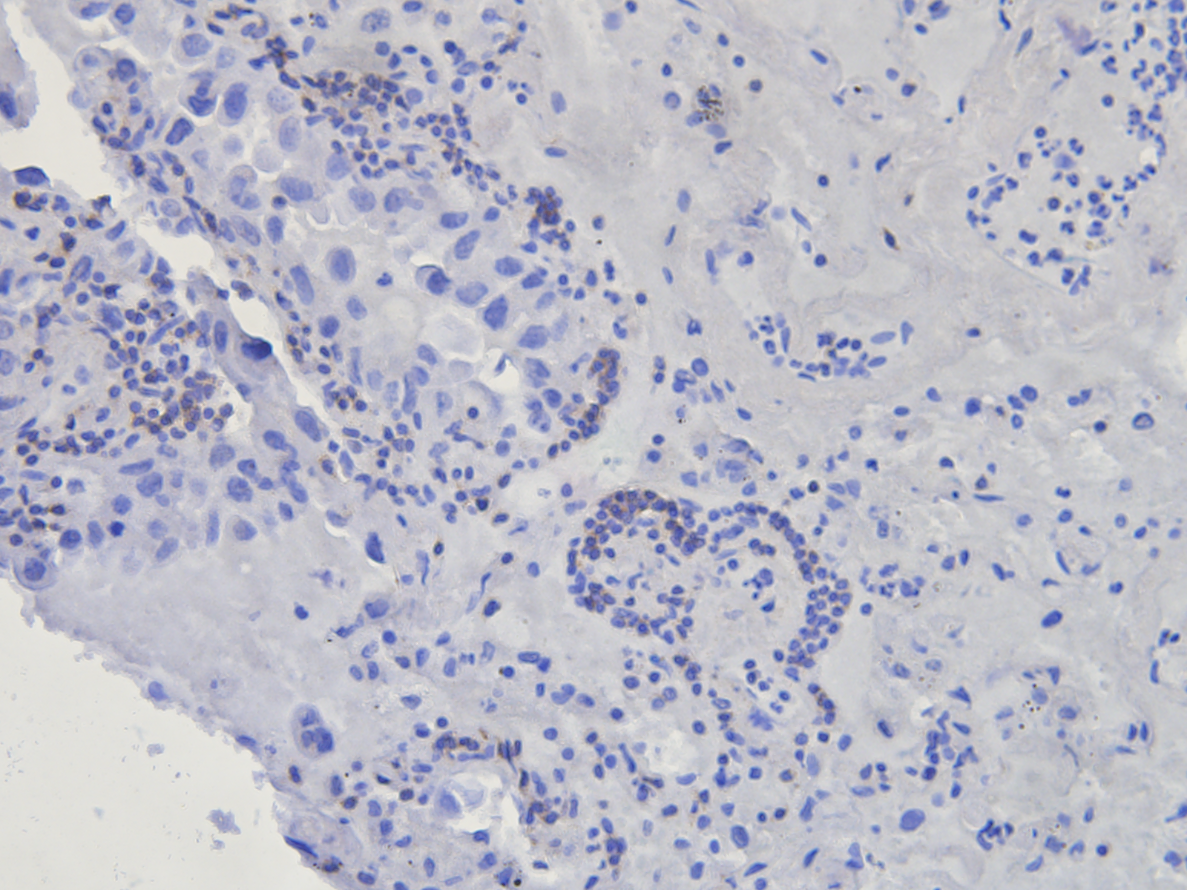

Supplement: S23 File — (ZIP) [file pone.0337223.s024.zip › 467564-400X-CA-N/467564-400X-CA (1).tif]

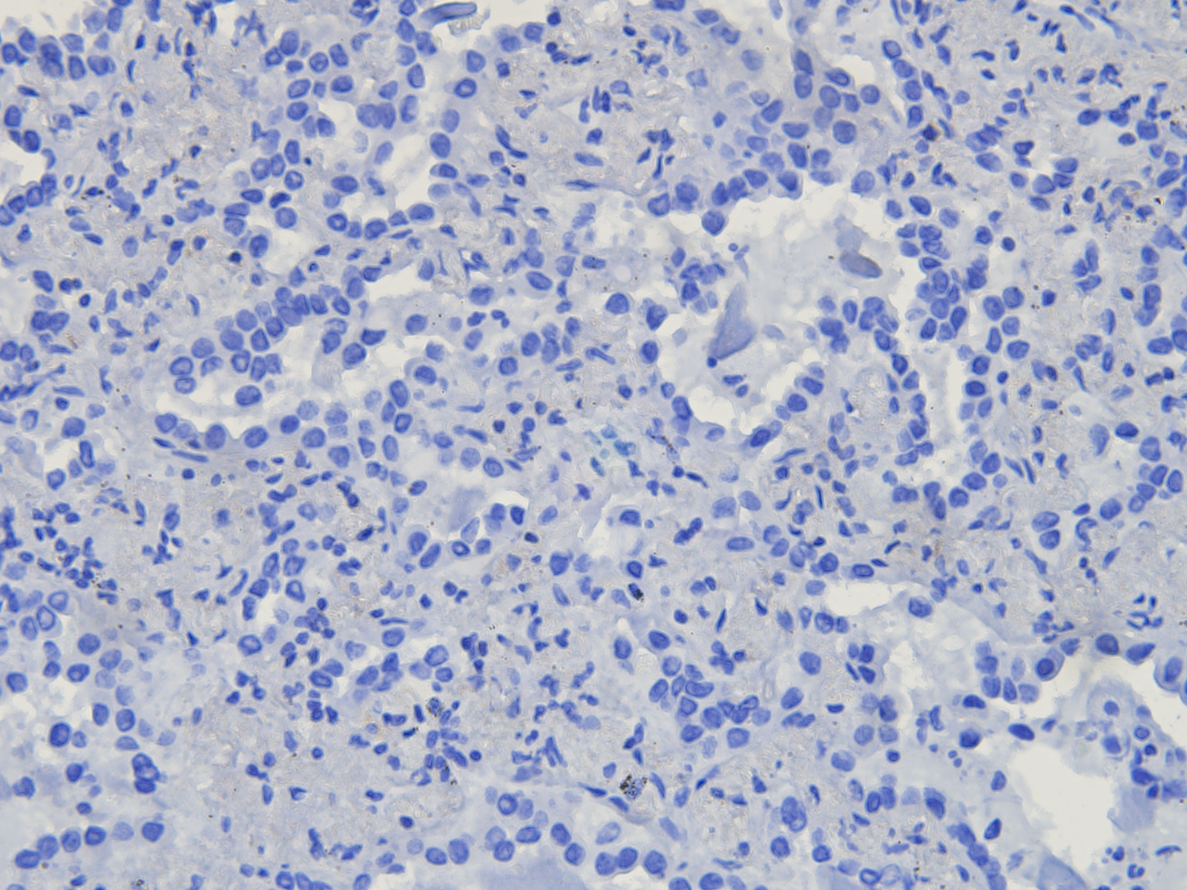

Supplement: S23 File — (ZIP) [file pone.0337223.s024.zip › 467564-400X-CA-N/467564-400X-CA (2).tif]

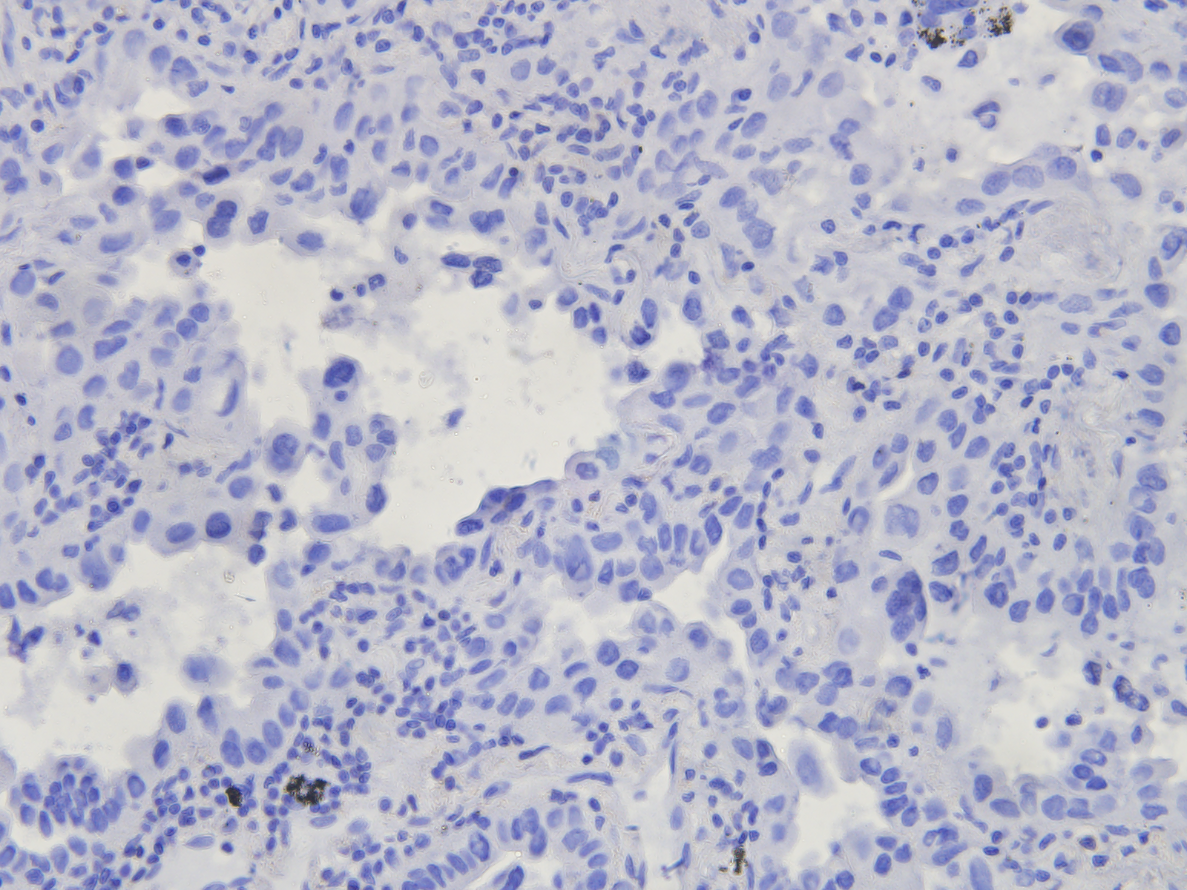

Supplement: S23 File — (ZIP) [file pone.0337223.s024.zip › 467564-400X-CA-N/467564-400X-CA (3).tif]

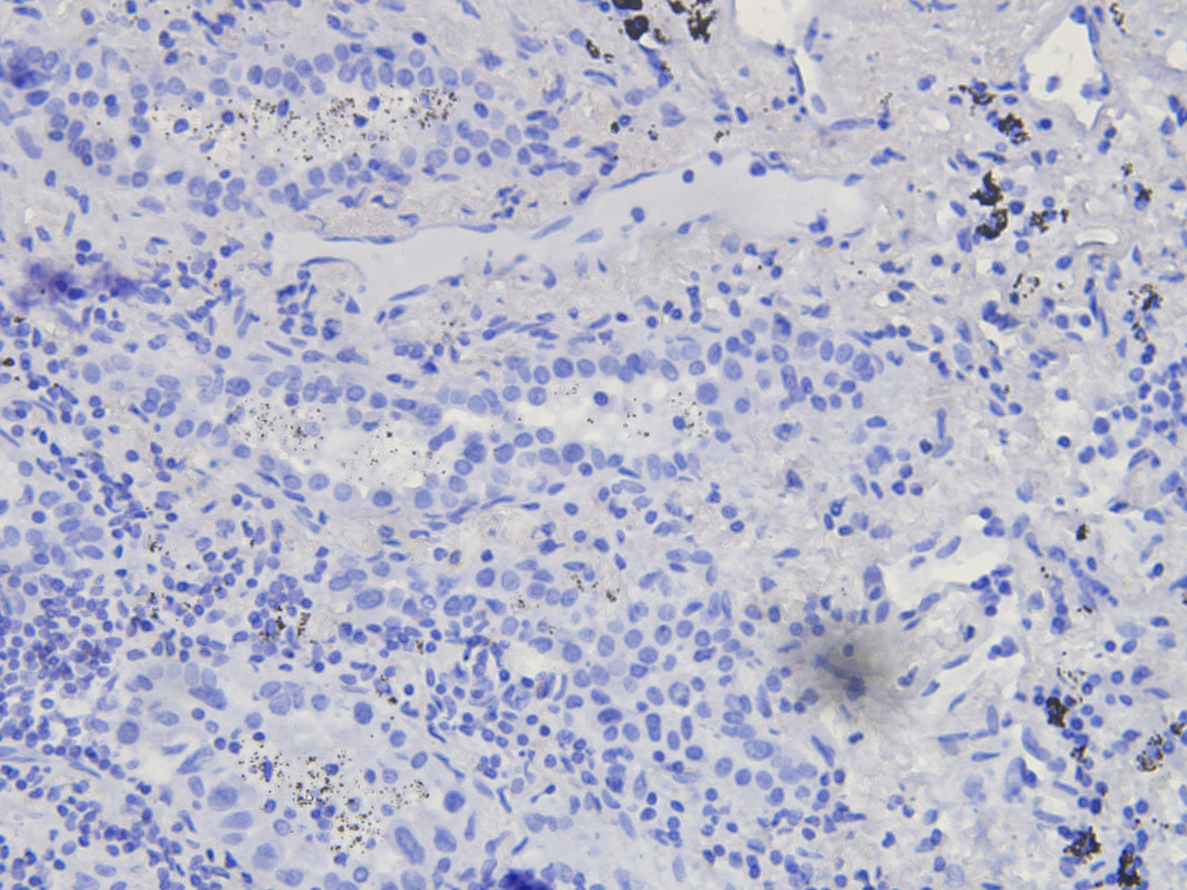

Supplement: S23 File — (ZIP) [file pone.0337223.s024.zip › 467564-400X-CA-N/467564-400X-CA (4).tif]

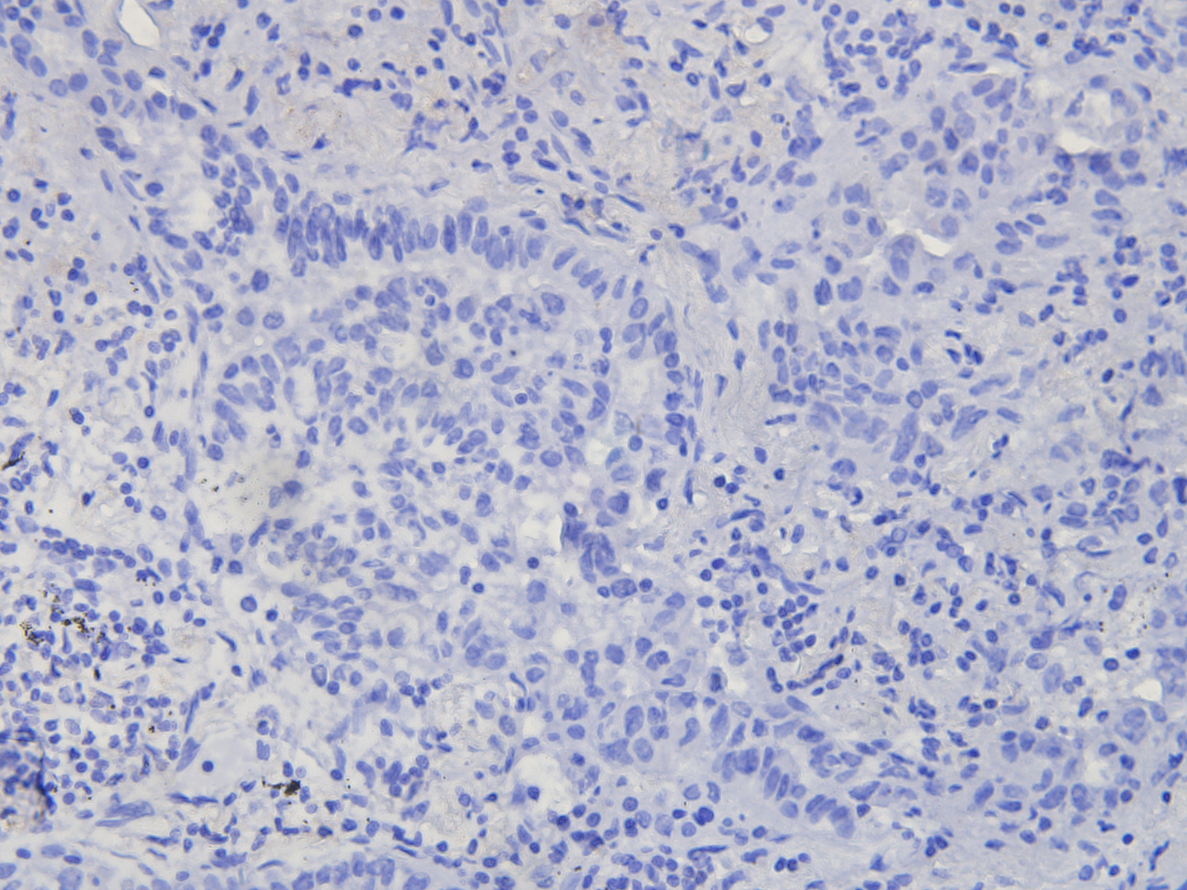

Supplement: S23 File — (ZIP) [file pone.0337223.s024.zip › 467564-400X-CA-N/467564-400X-CA (6).tif]

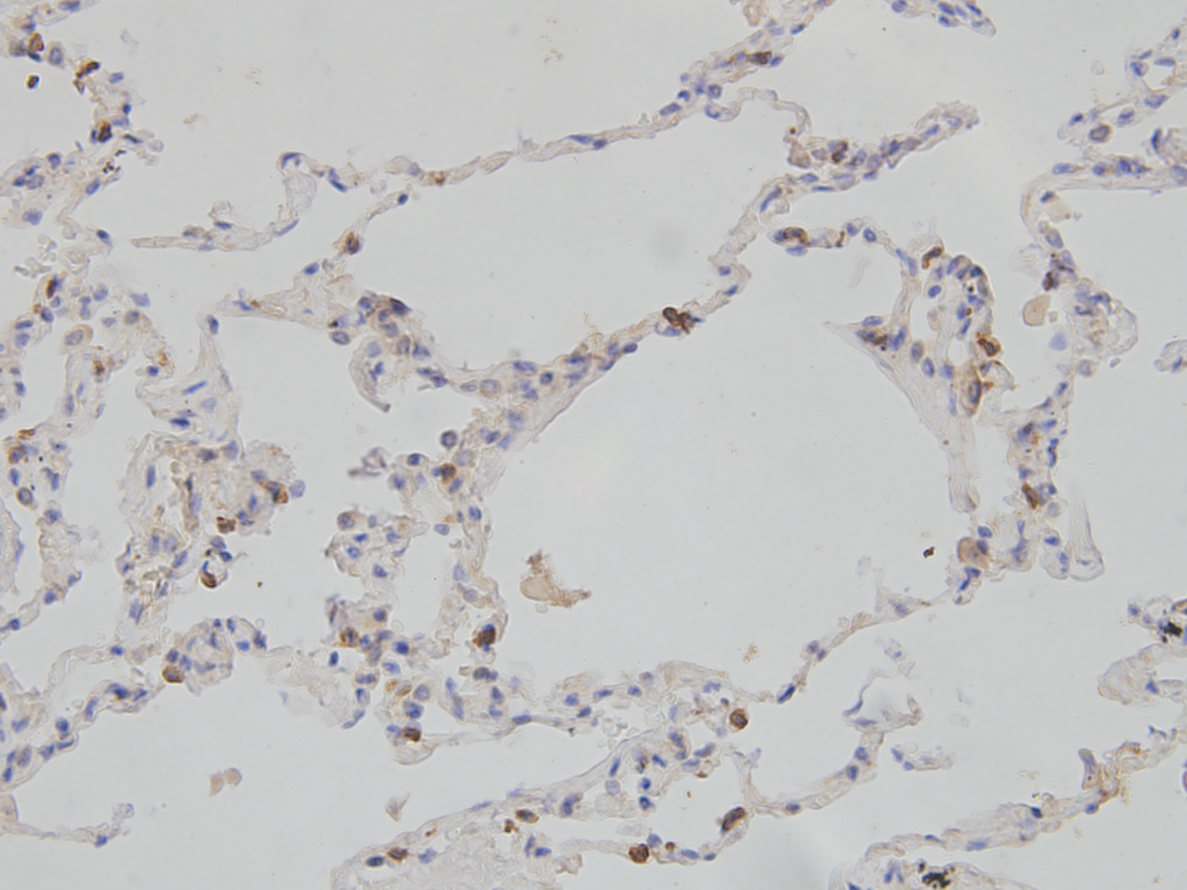

Supplement: S23 File — (ZIP) [file pone.0337223.s024.zip › 467564-400X-CA-N/467564-400X-N (1).tif]

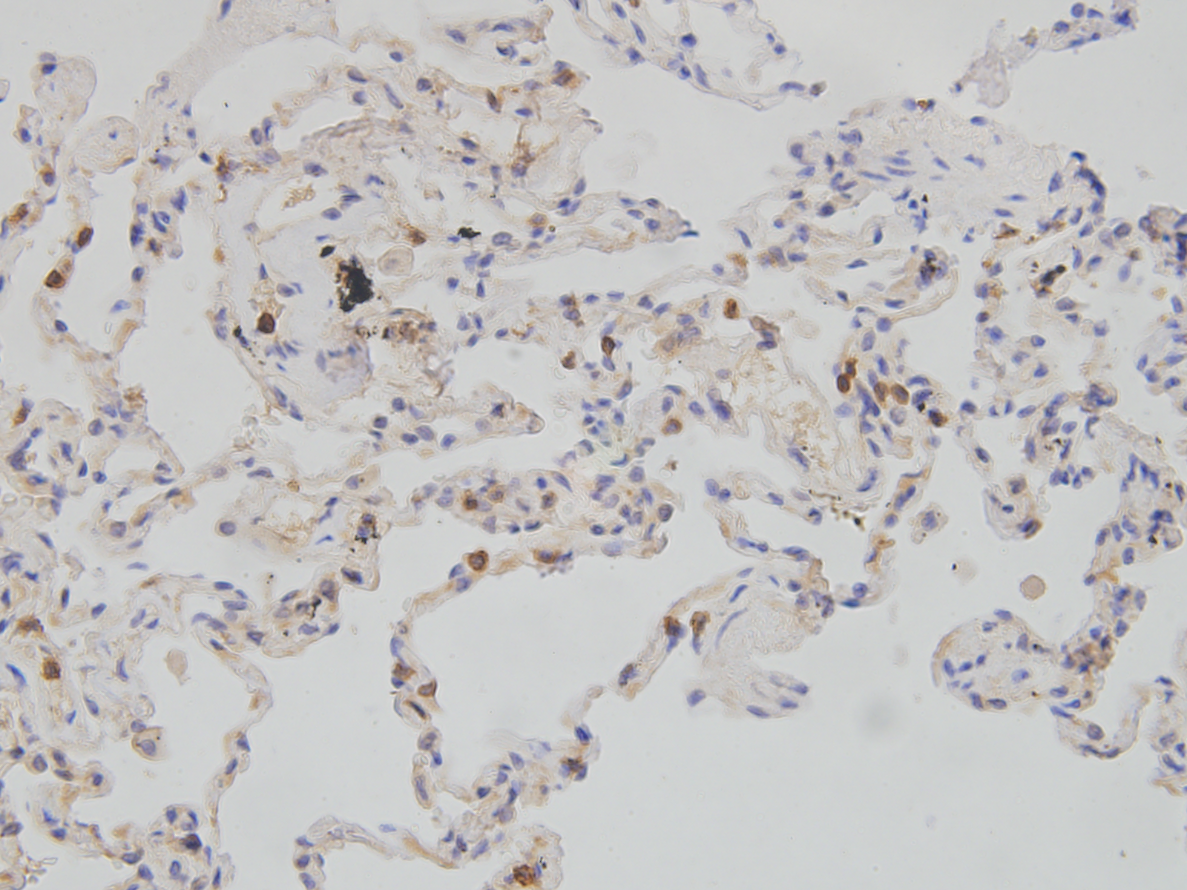

Supplement: S23 File — (ZIP) [file pone.0337223.s024.zip › 467564-400X-CA-N/467564-400X-N (2).tif]

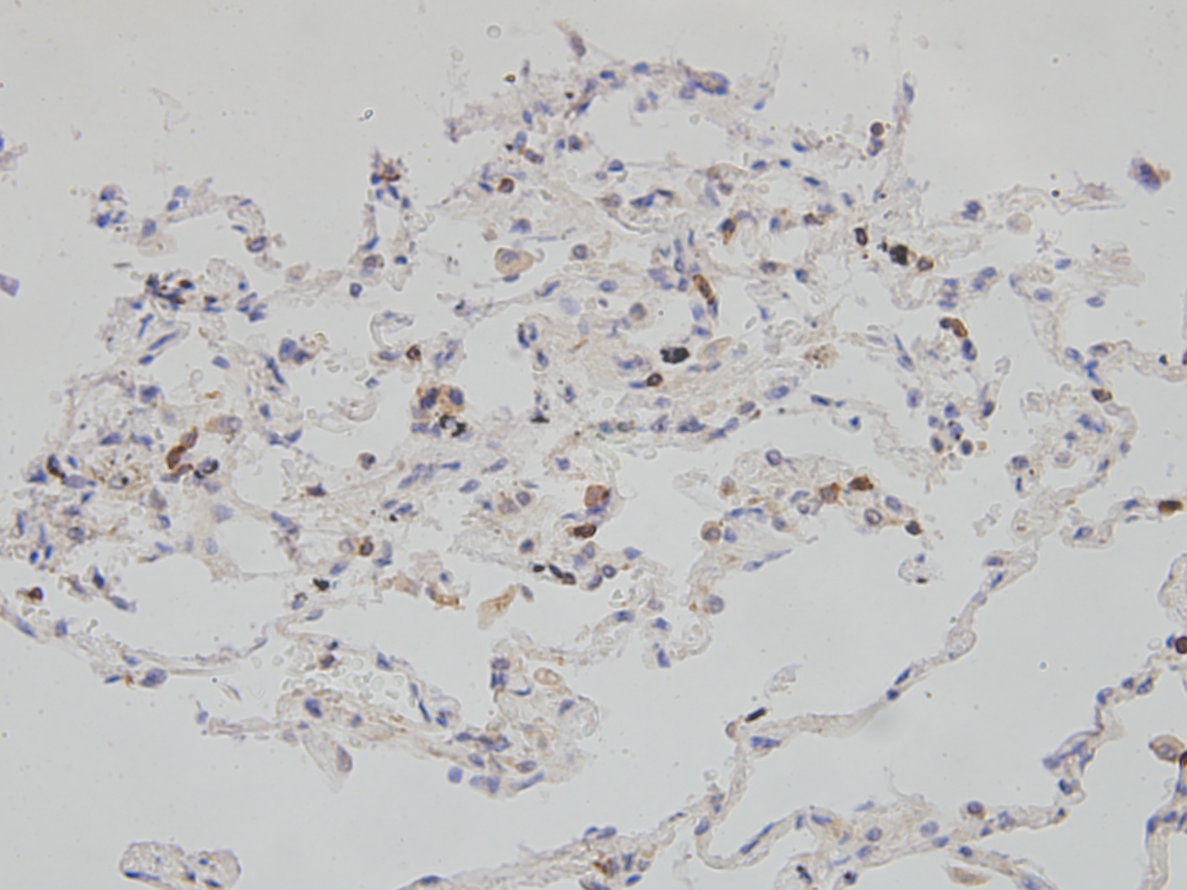

Supplement: S23 File — (ZIP) [file pone.0337223.s024.zip › 467564-400X-CA-N/467564-400X-N (3).tif]

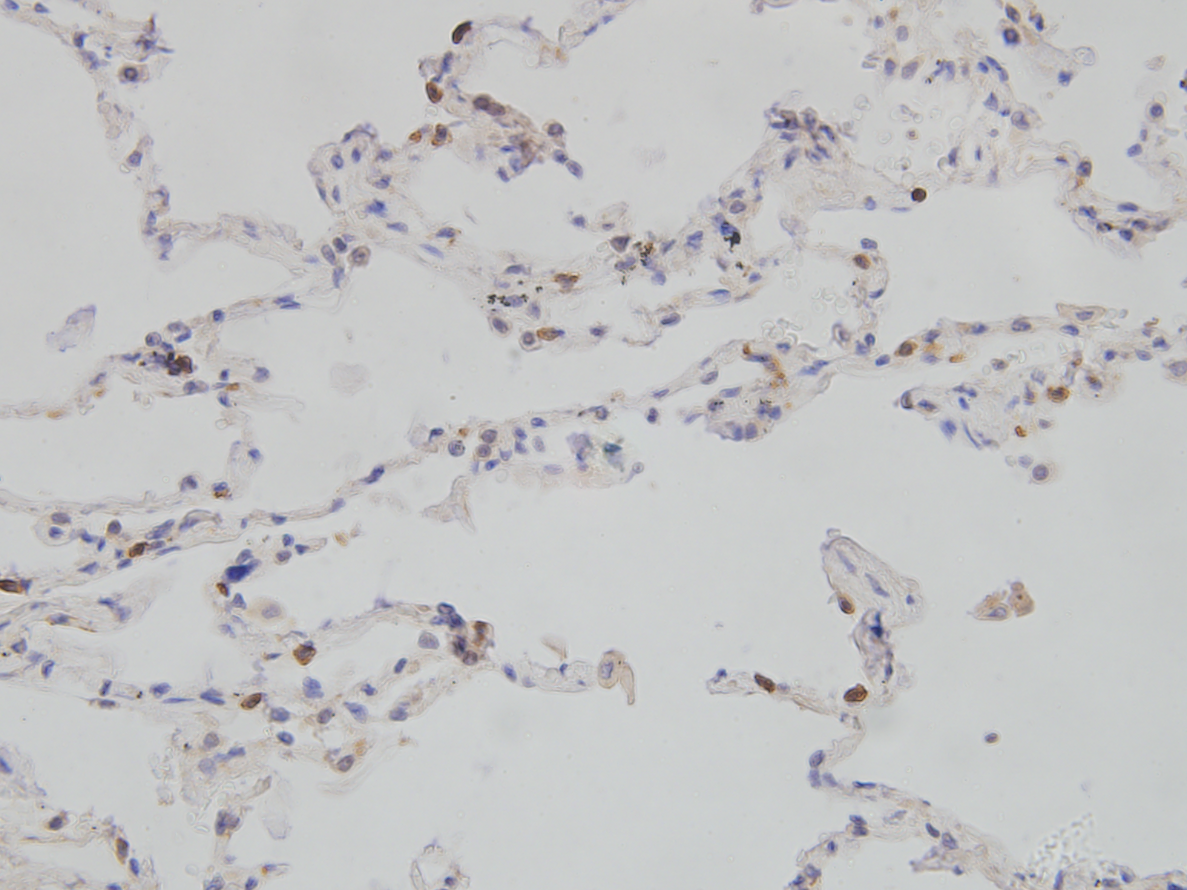

Supplement: S23 File — (ZIP) [file pone.0337223.s024.zip › 467564-400X-CA-N/467564-400X-N (4).tif]

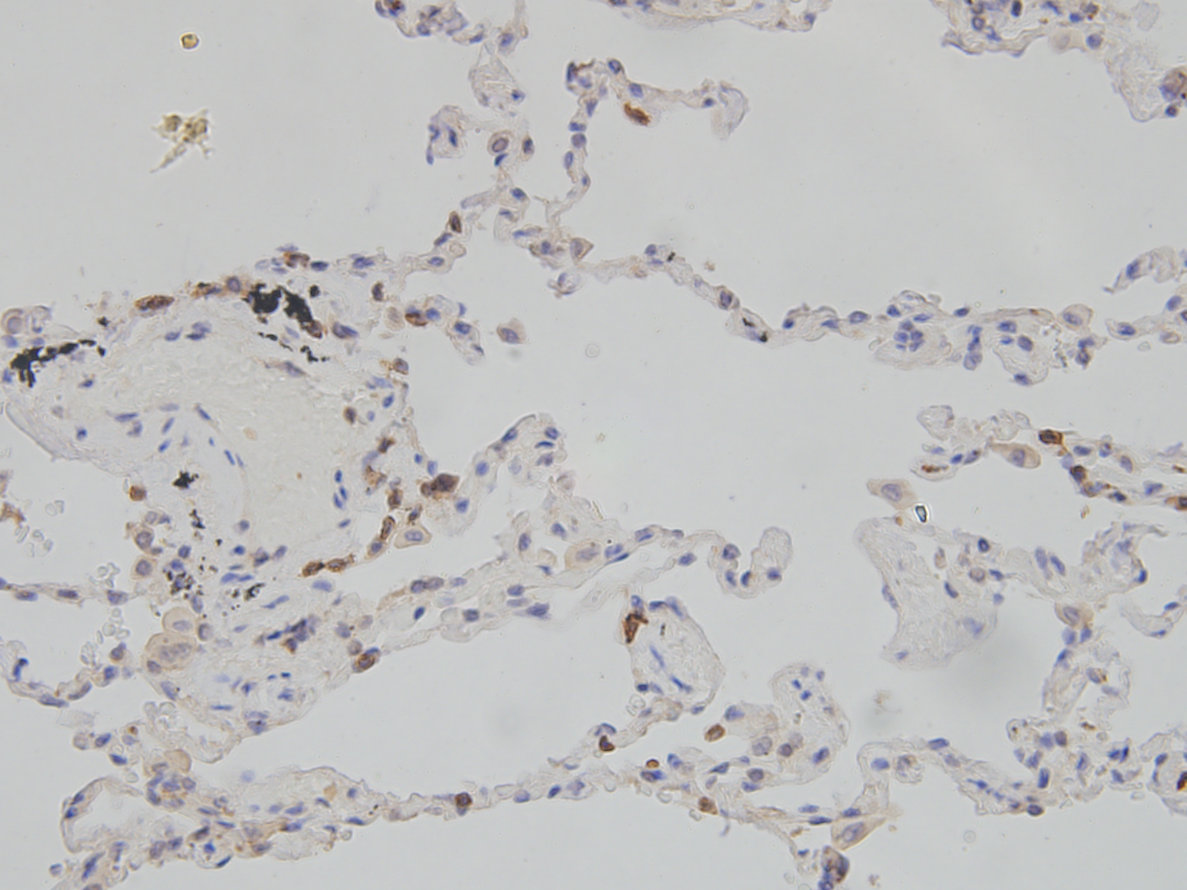

Supplement: S23 File — (ZIP) [file pone.0337223.s024.zip › 467564-400X-CA-N/467564-400X-N (5).tif]

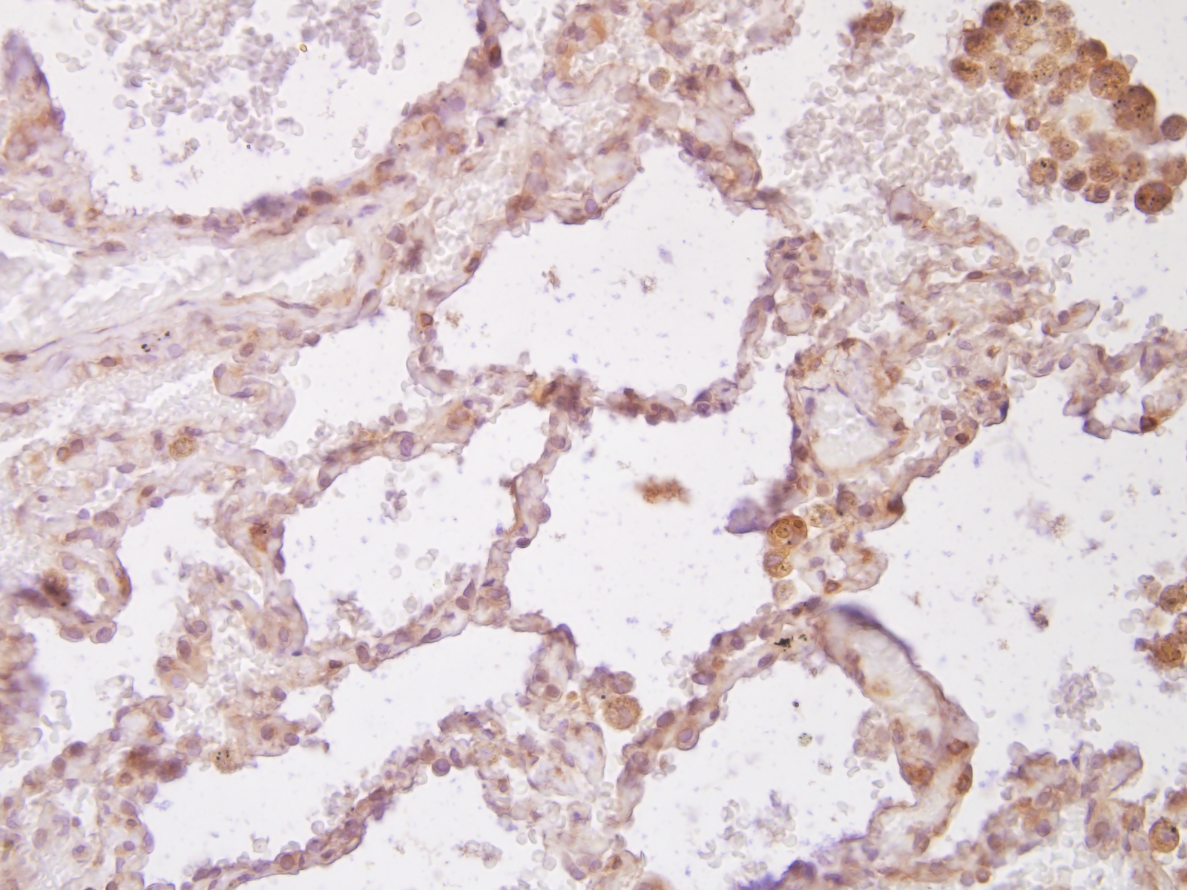

Supplement: S24 File — (ZIP) [file pone.0337223.s025.zip › 467893-400X-CA-N/467893-400X- N (1).tif]

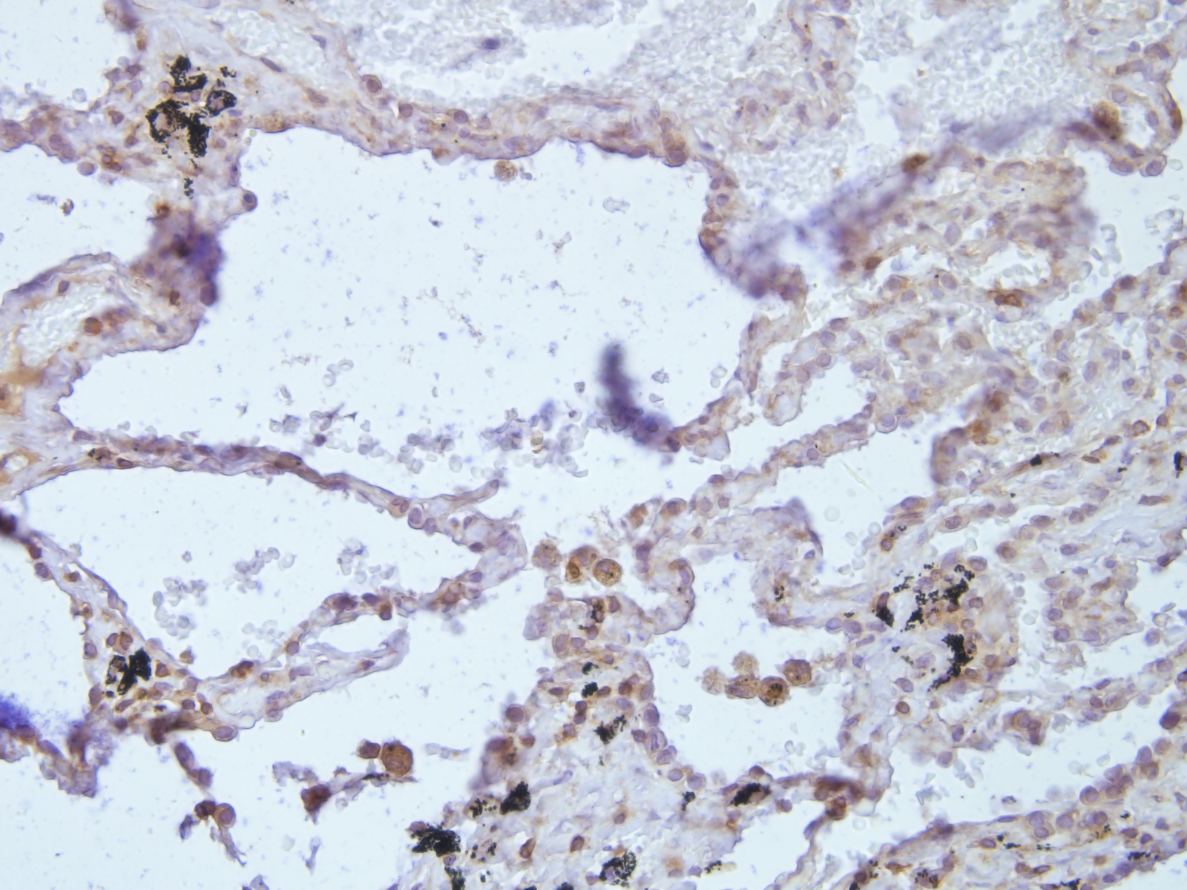

Supplement: S24 File — (ZIP) [file pone.0337223.s025.zip › 467893-400X-CA-N/467893-400X- N (2).tif]

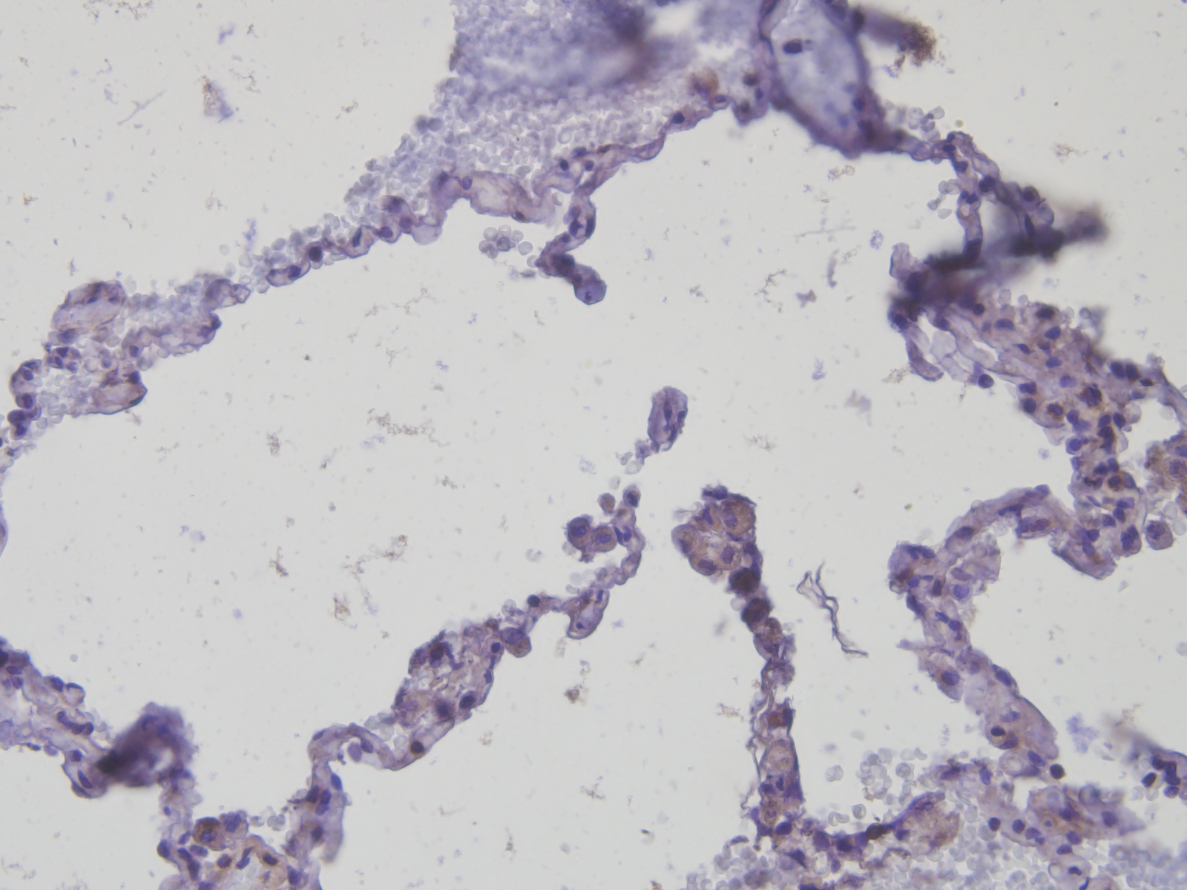

Supplement: S24 File — (ZIP) [file pone.0337223.s025.zip › 467893-400X-CA-N/467893-400X- N (3).tif]

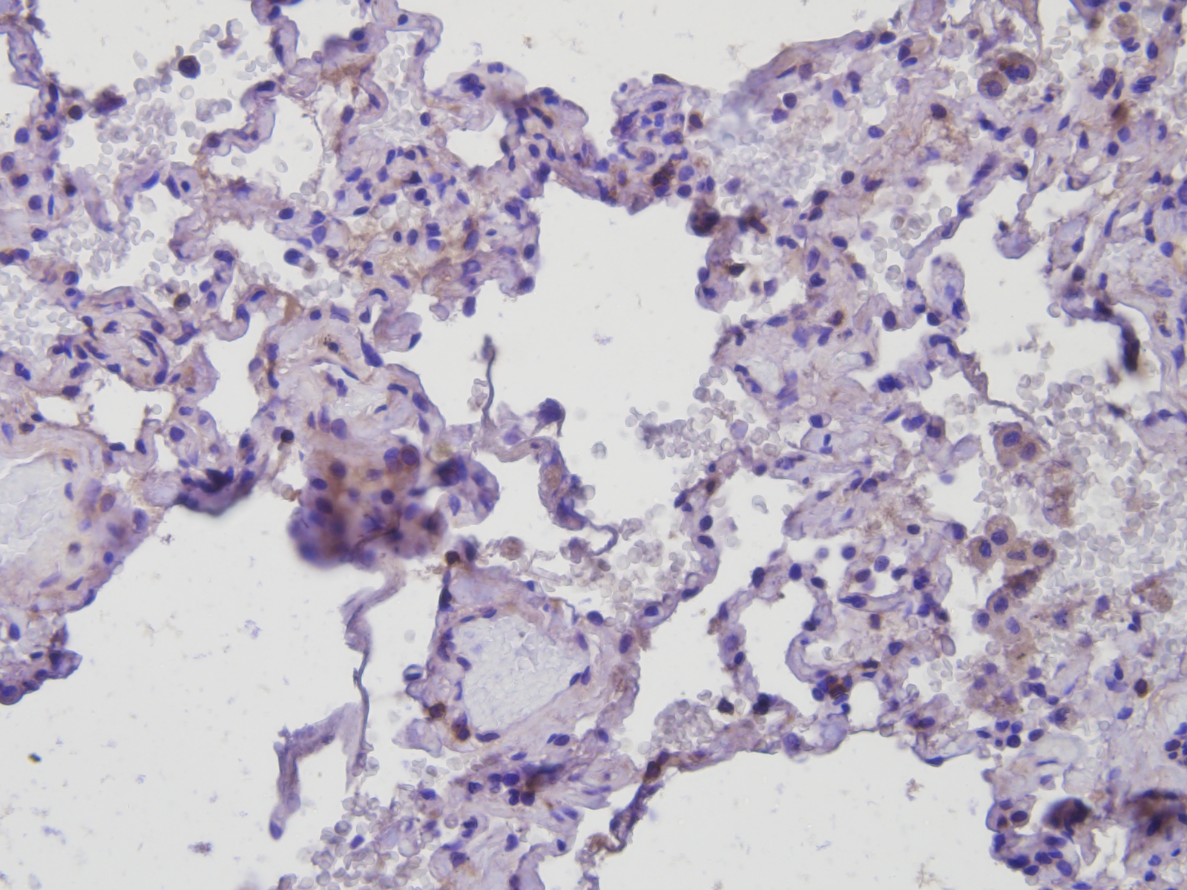

Supplement: S24 File — (ZIP) [file pone.0337223.s025.zip › 467893-400X-CA-N/467893-400X- N (4).tif]

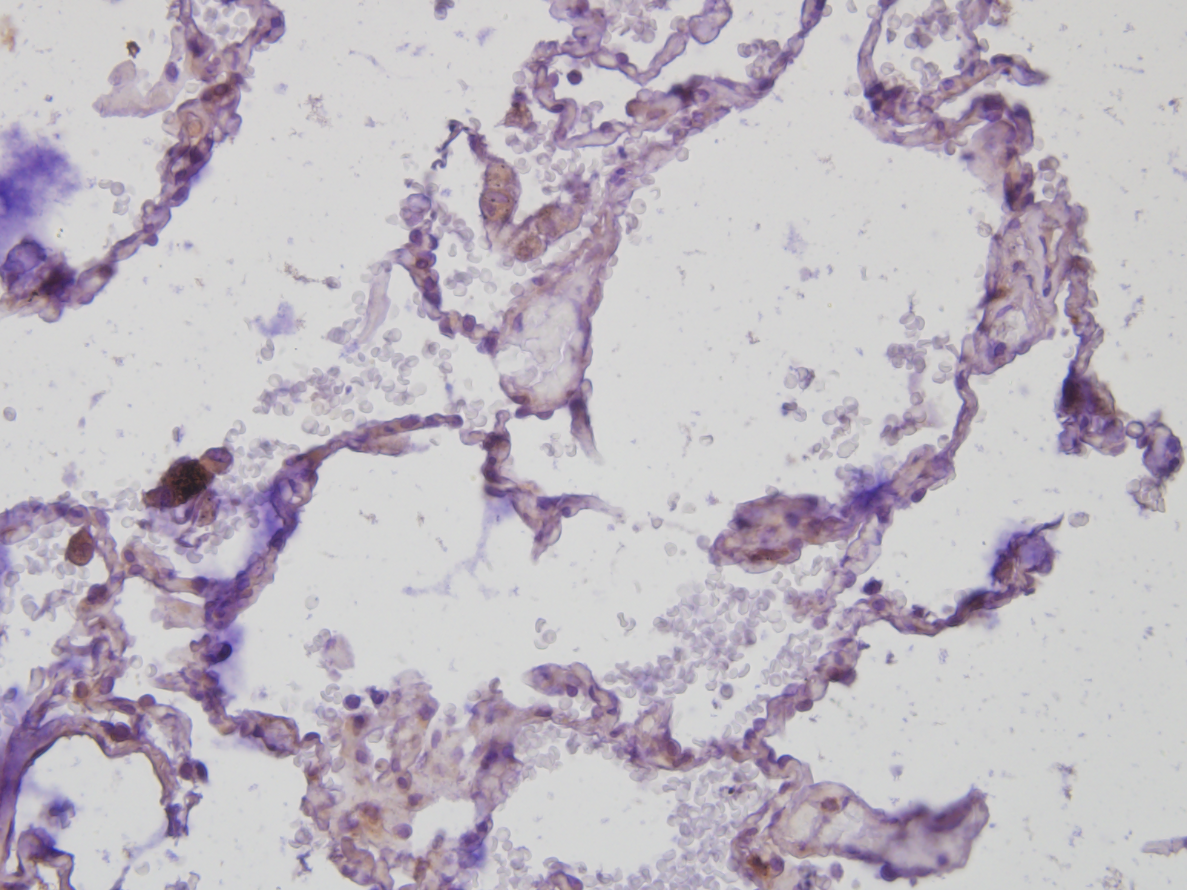

Supplement: S24 File — (ZIP) [file pone.0337223.s025.zip › 467893-400X-CA-N/467893-400X- N (5).tif]

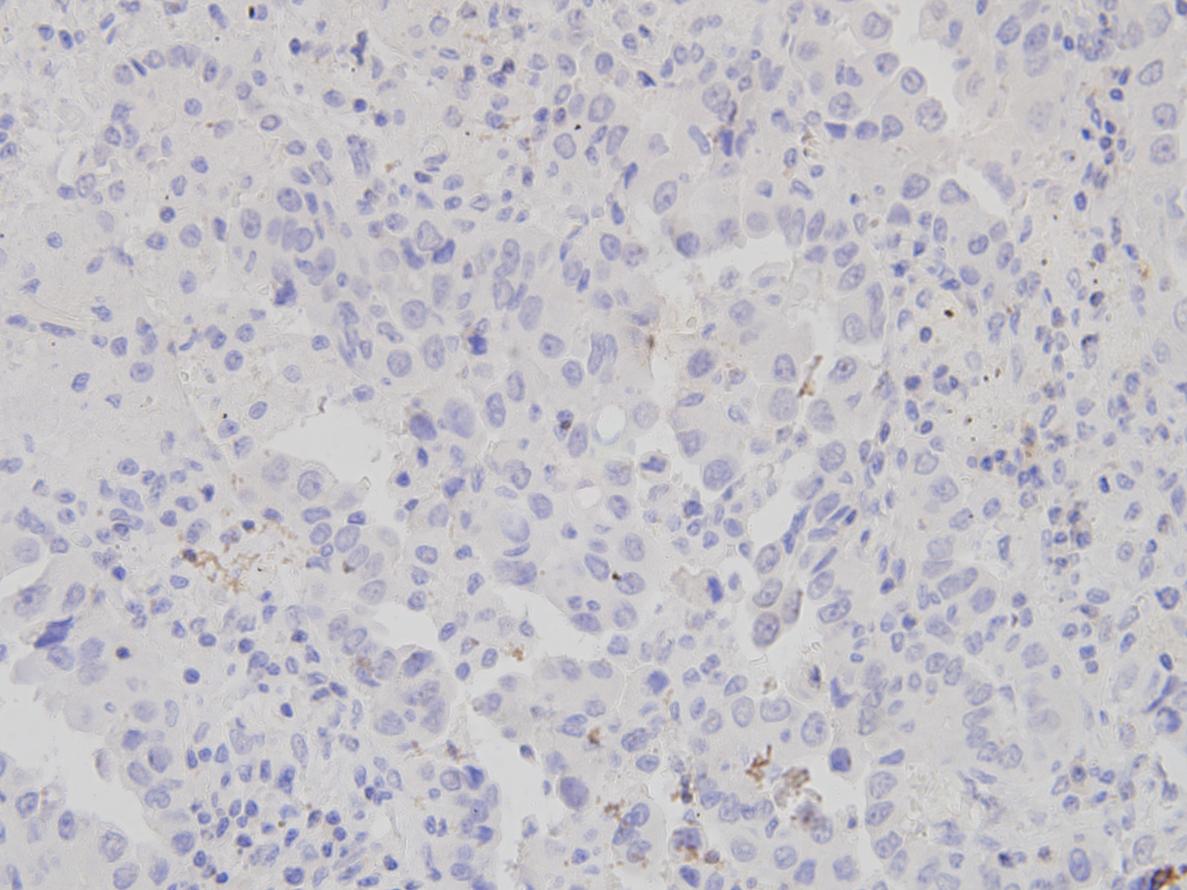

Supplement: S24 File — (ZIP) [file pone.0337223.s025.zip › 467893-400X-CA-N/467893-400X-CA (1).tif]

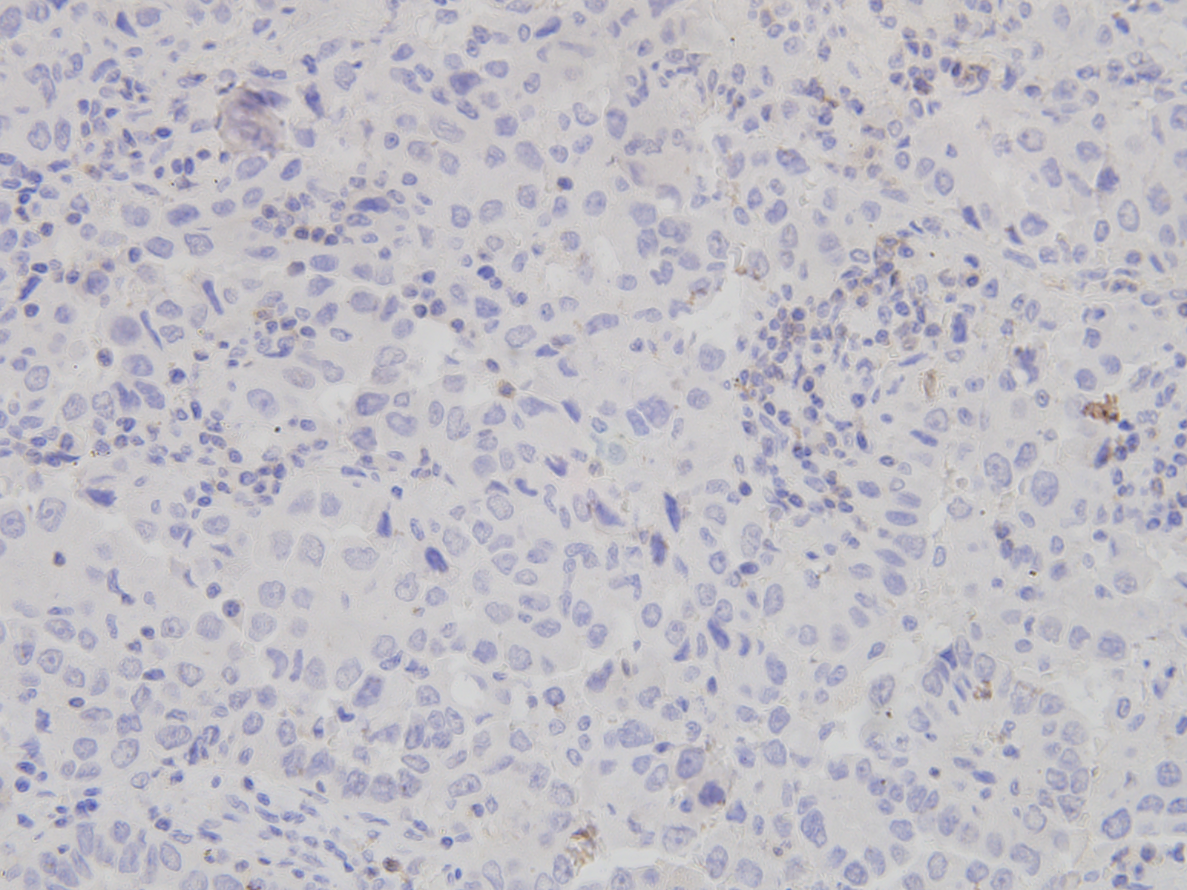

Supplement: S24 File — (ZIP) [file pone.0337223.s025.zip › 467893-400X-CA-N/467893-400X-CA (2).tif]

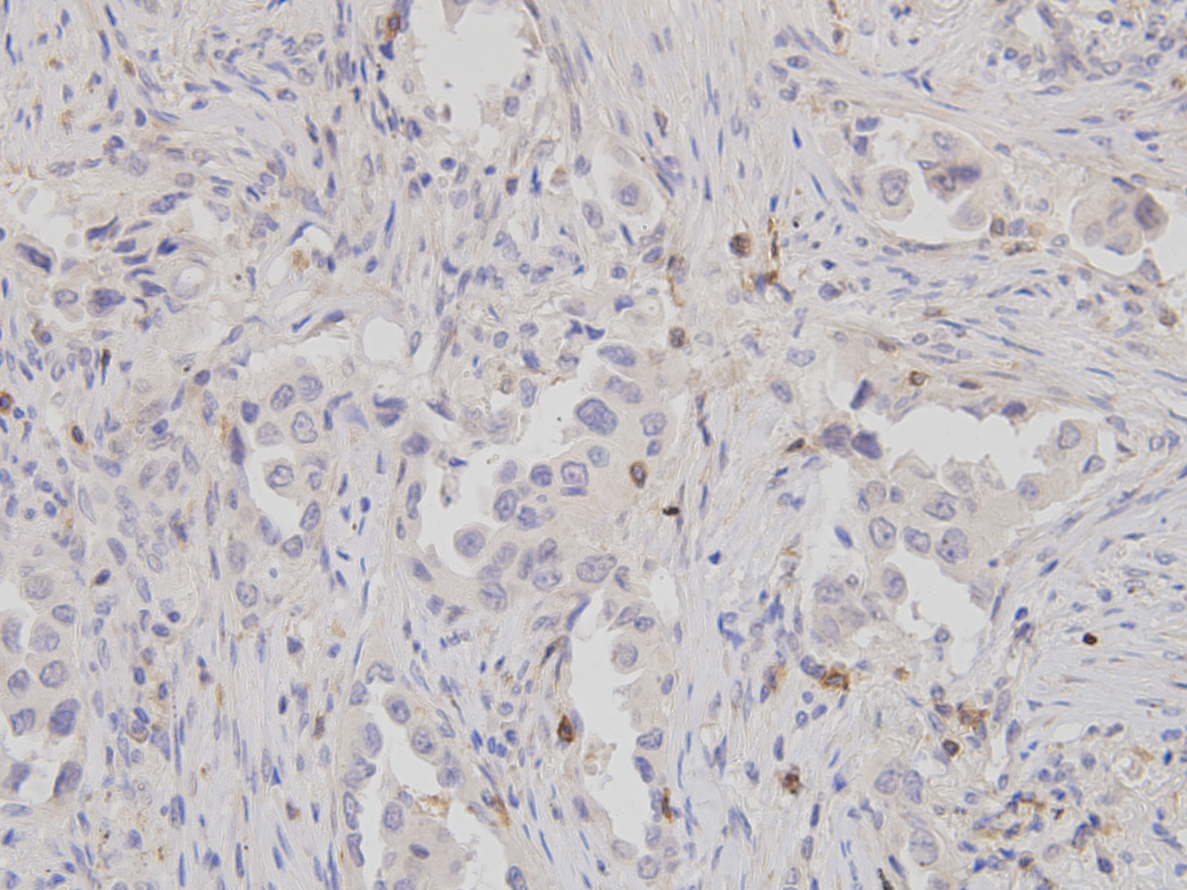

Supplement: S24 File — (ZIP) [file pone.0337223.s025.zip › 467893-400X-CA-N/467893-400X-CA (3).tif]

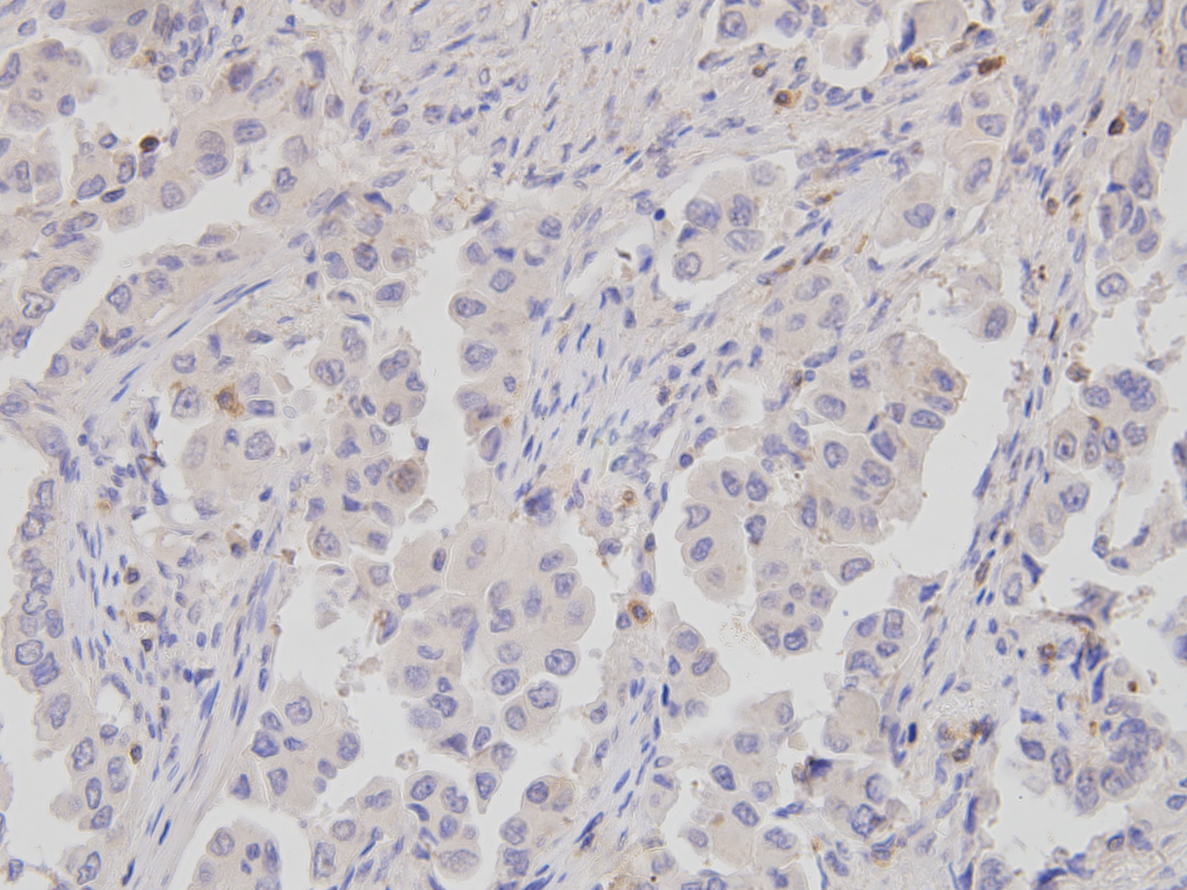

Supplement: S24 File — (ZIP) [file pone.0337223.s025.zip › 467893-400X-CA-N/467893-400X-CA (4).tif]

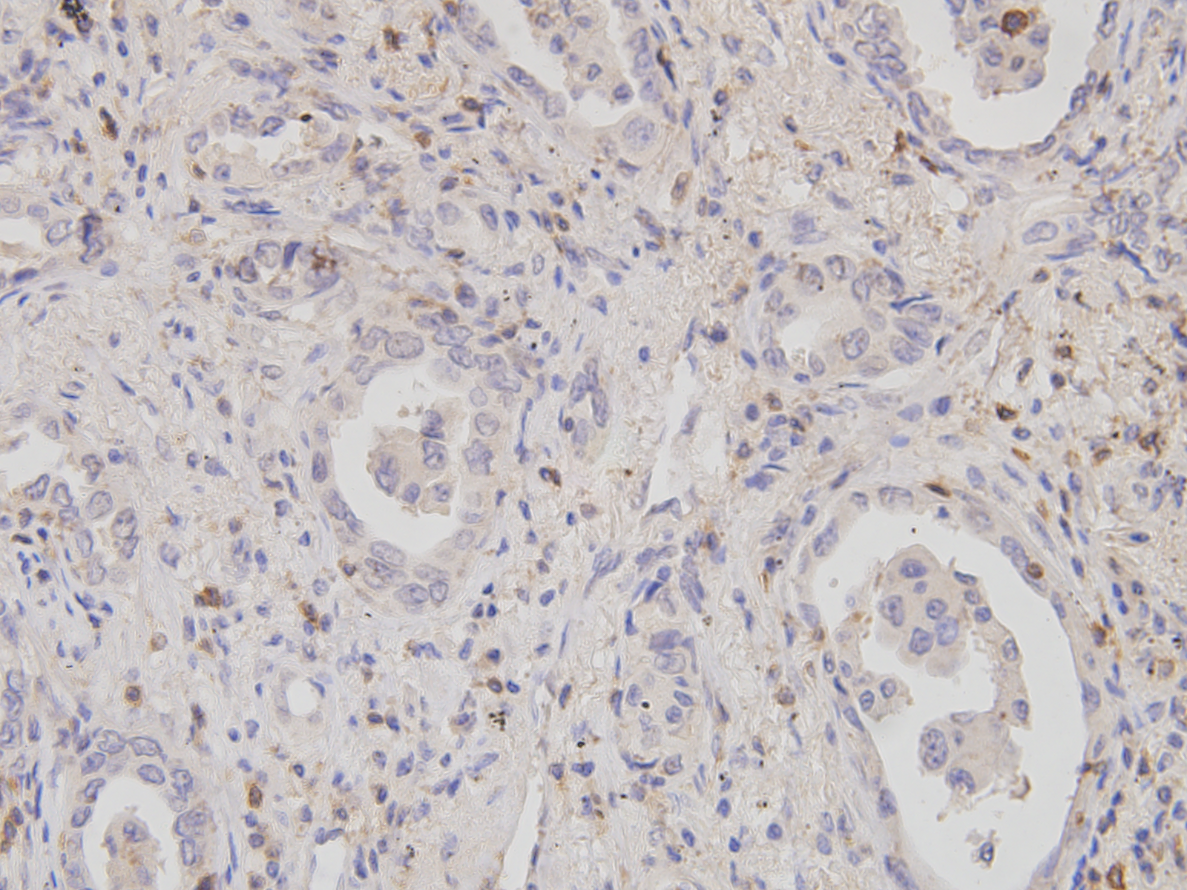

Supplement: S24 File — (ZIP) [file pone.0337223.s025.zip › 467893-400X-CA-N/467893-400X-CA (5).tif]

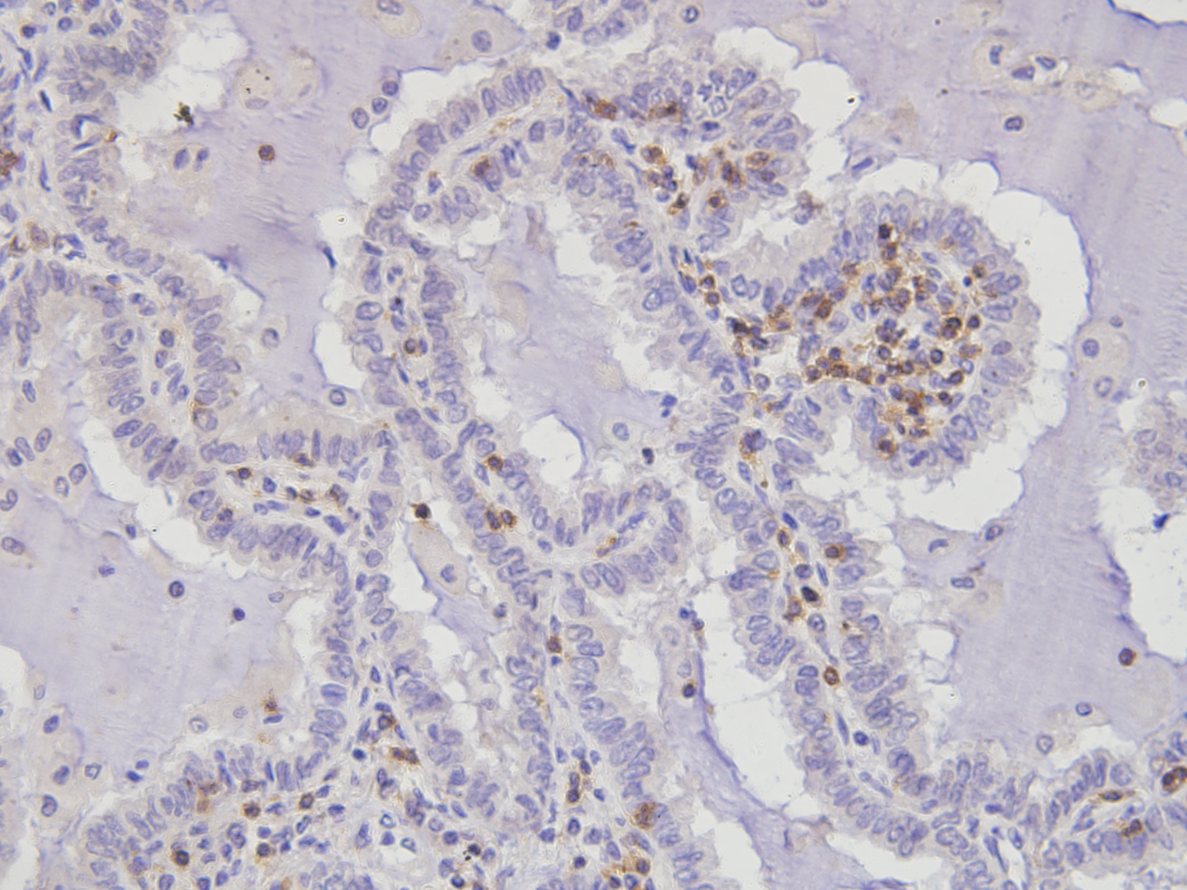

Supplement: S25 File — (ZIP) [file pone.0337223.s026.zip › 468077-400X-CA-N-/468077-400X-CA (1).tif]

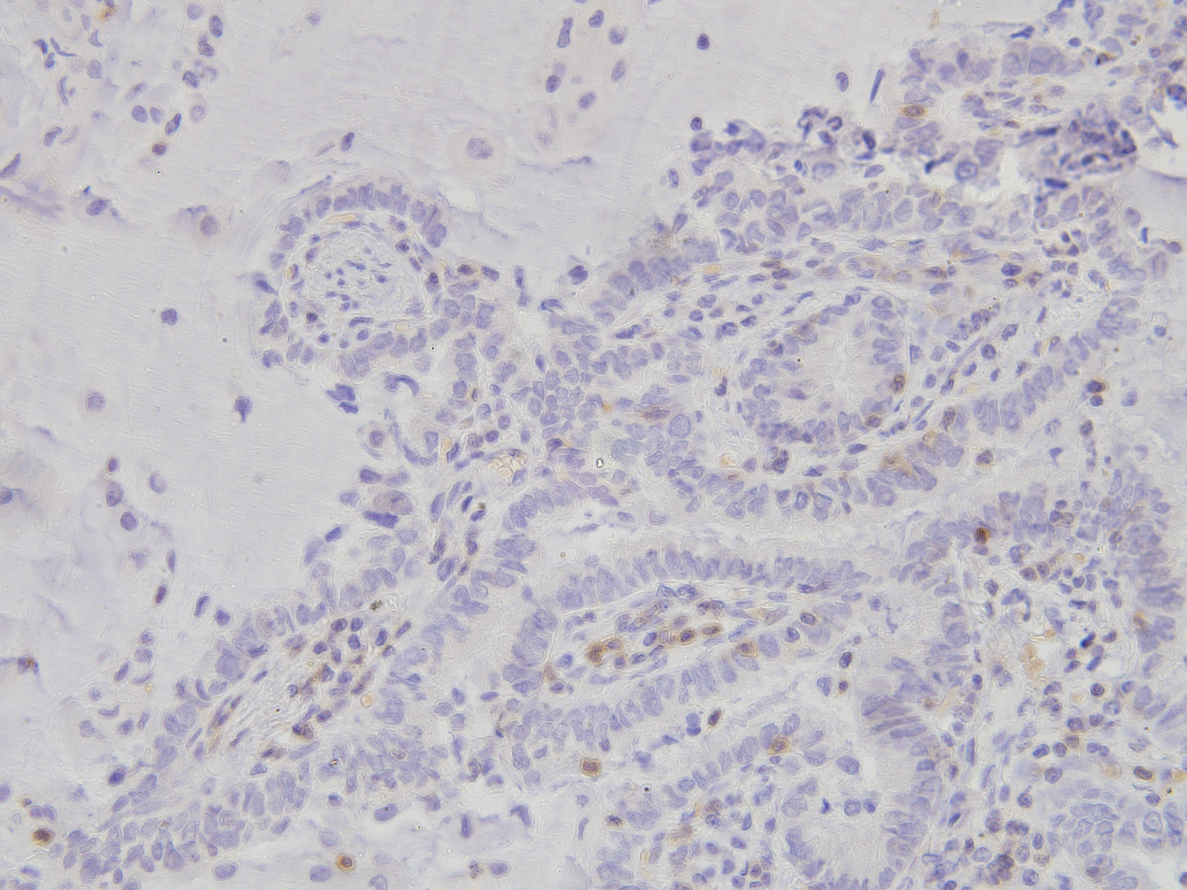

Supplement: S25 File — (ZIP) [file pone.0337223.s026.zip › 468077-400X-CA-N-/468077-400X-CA (2).tif]

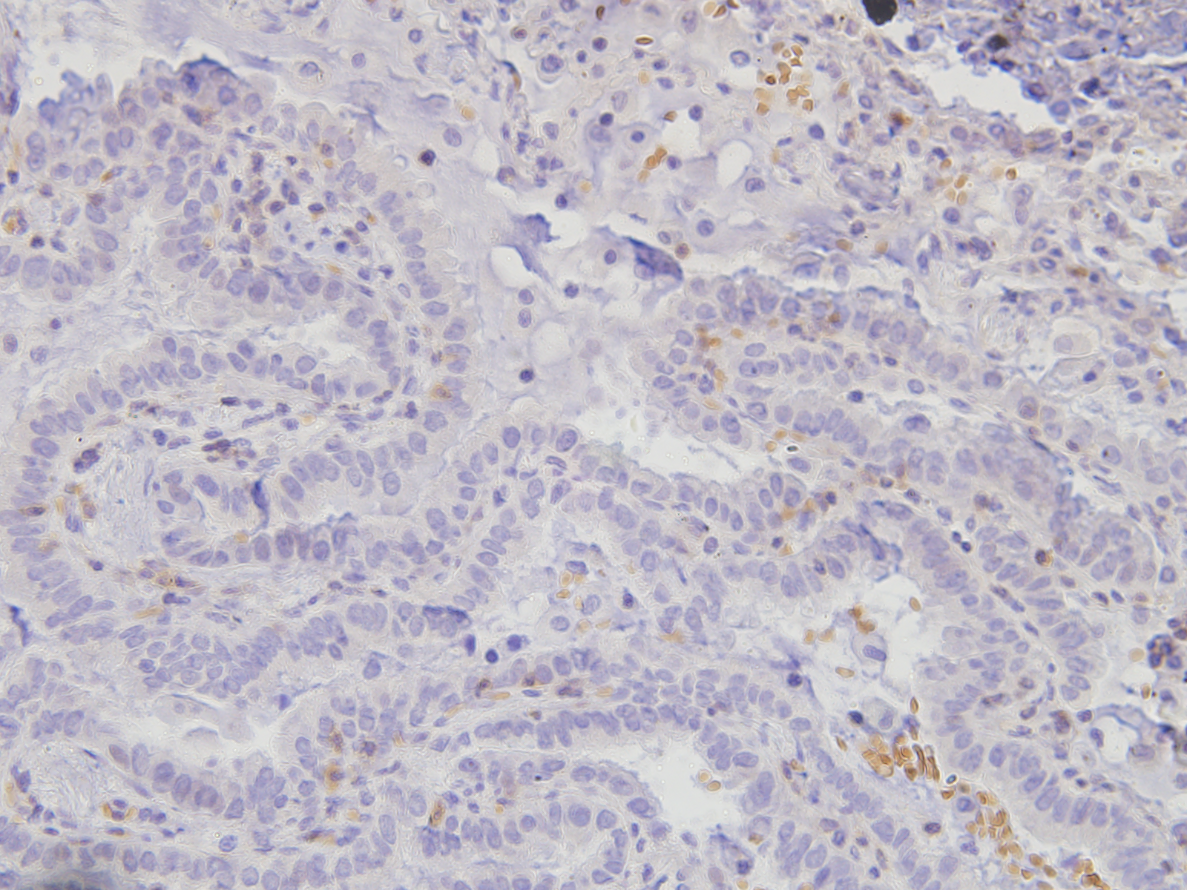

Supplement: S25 File — (ZIP) [file pone.0337223.s026.zip › 468077-400X-CA-N-/468077-400X-CA (3).tif]

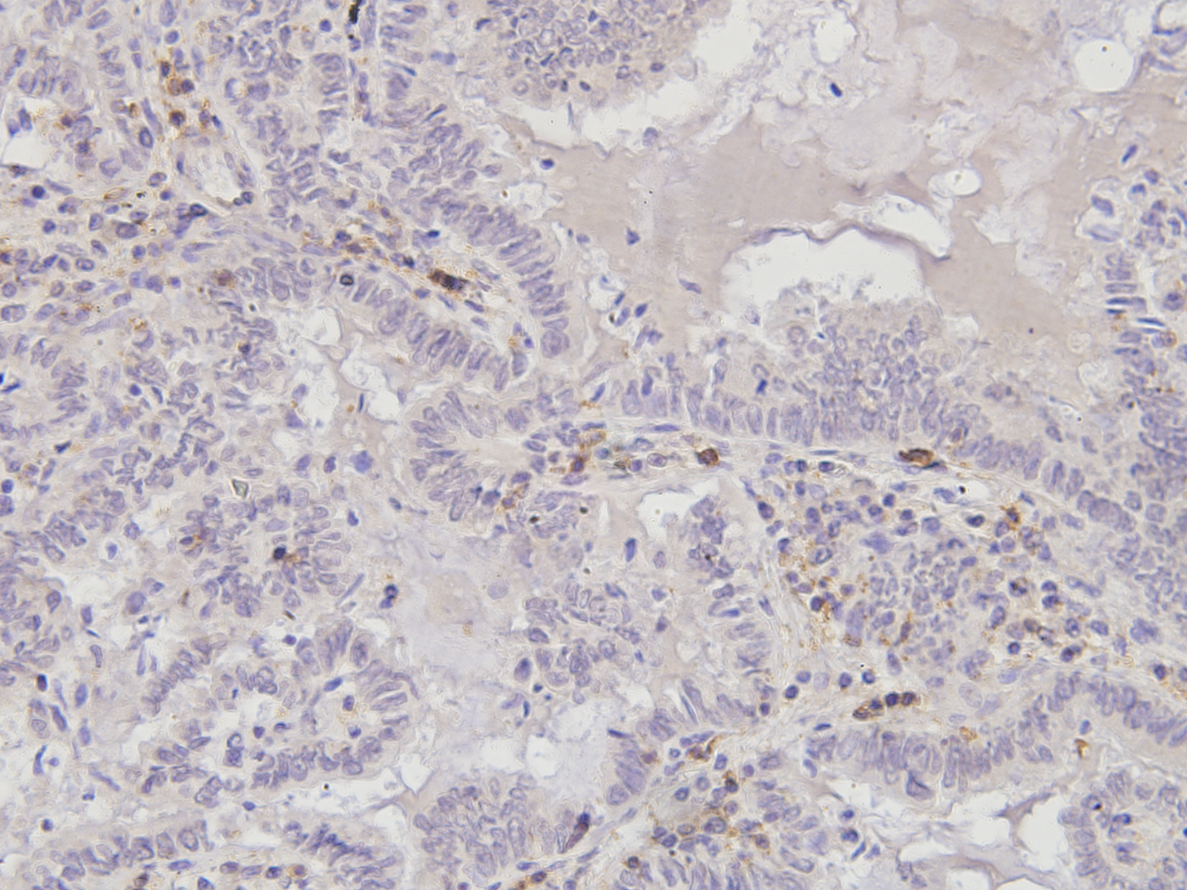

Supplement: S25 File — (ZIP) [file pone.0337223.s026.zip › 468077-400X-CA-N-/468077-400X-CA (4).tif]

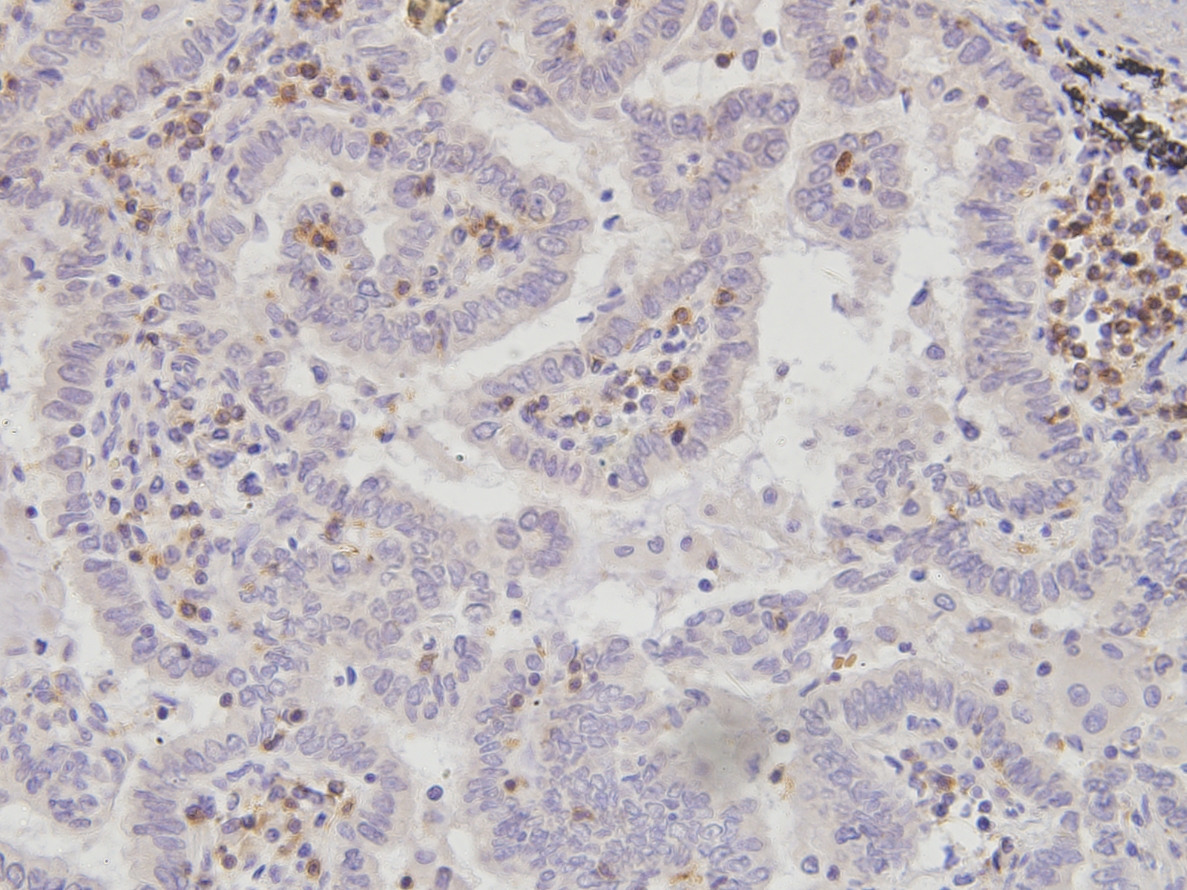

Supplement: S25 File — (ZIP) [file pone.0337223.s026.zip › 468077-400X-CA-N-/468077-400X-CA (5).tif]

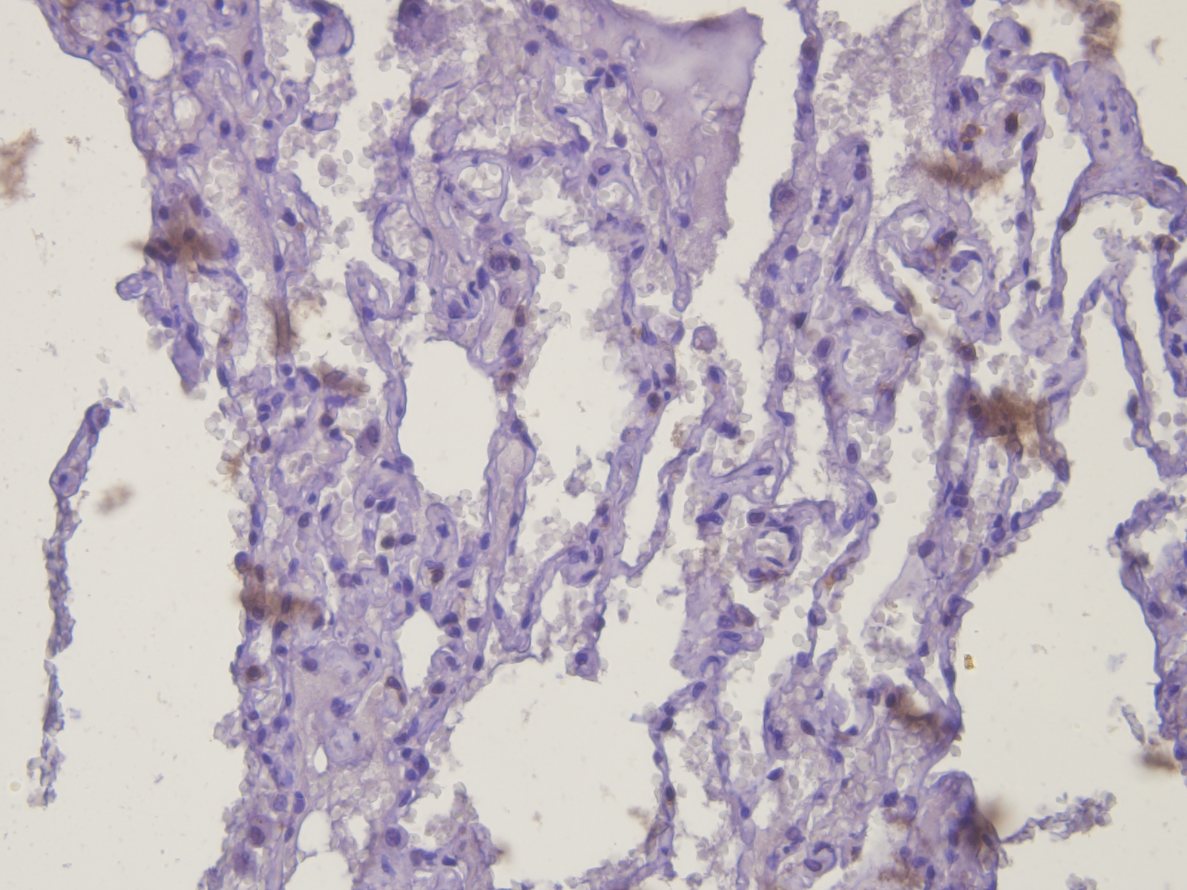

Supplement: S25 File — (ZIP) [file pone.0337223.s026.zip › 468077-400X-CA-N-/468077-400X-N (1).tif]

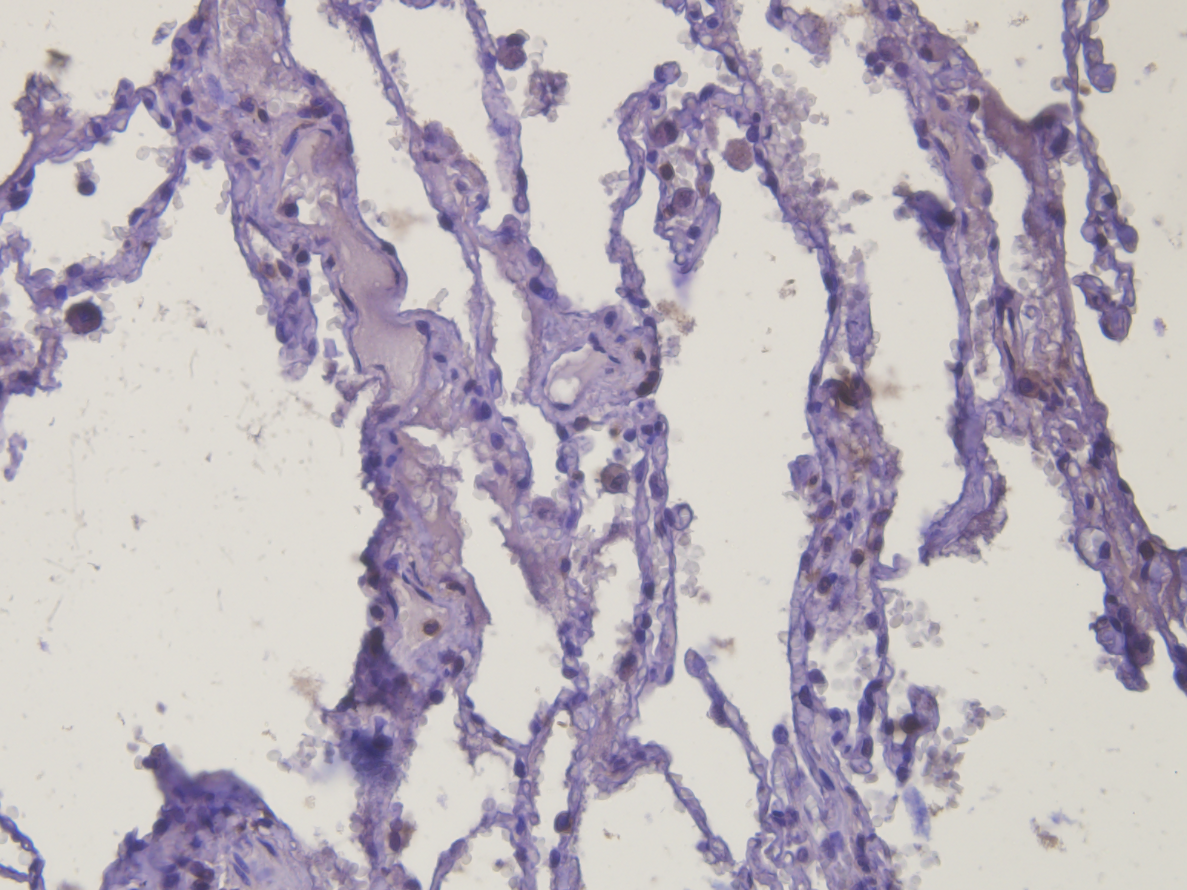

Supplement: S25 File — (ZIP) [file pone.0337223.s026.zip › 468077-400X-CA-N-/468077-400X-N (2).tif]

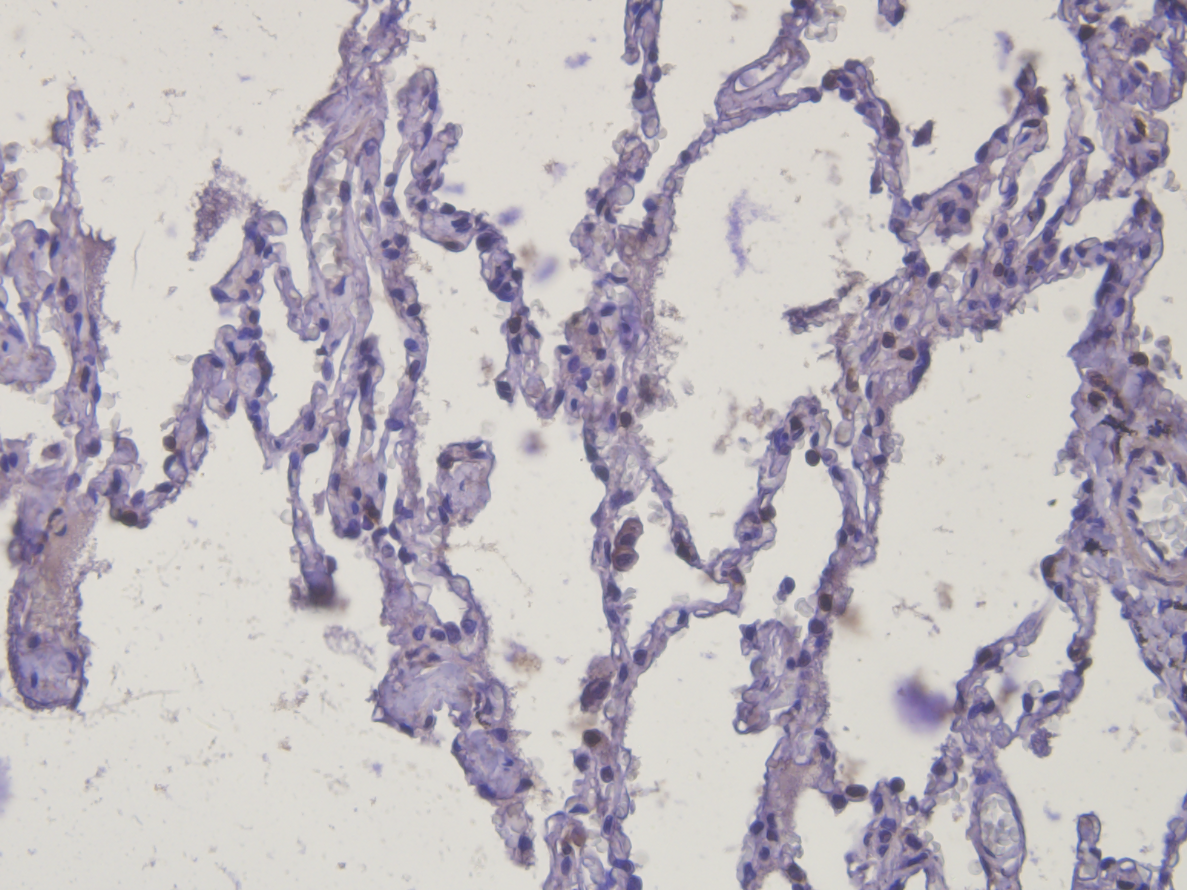

Supplement: S25 File — (ZIP) [file pone.0337223.s026.zip › 468077-400X-CA-N-/468077-400X-N (3).tif]

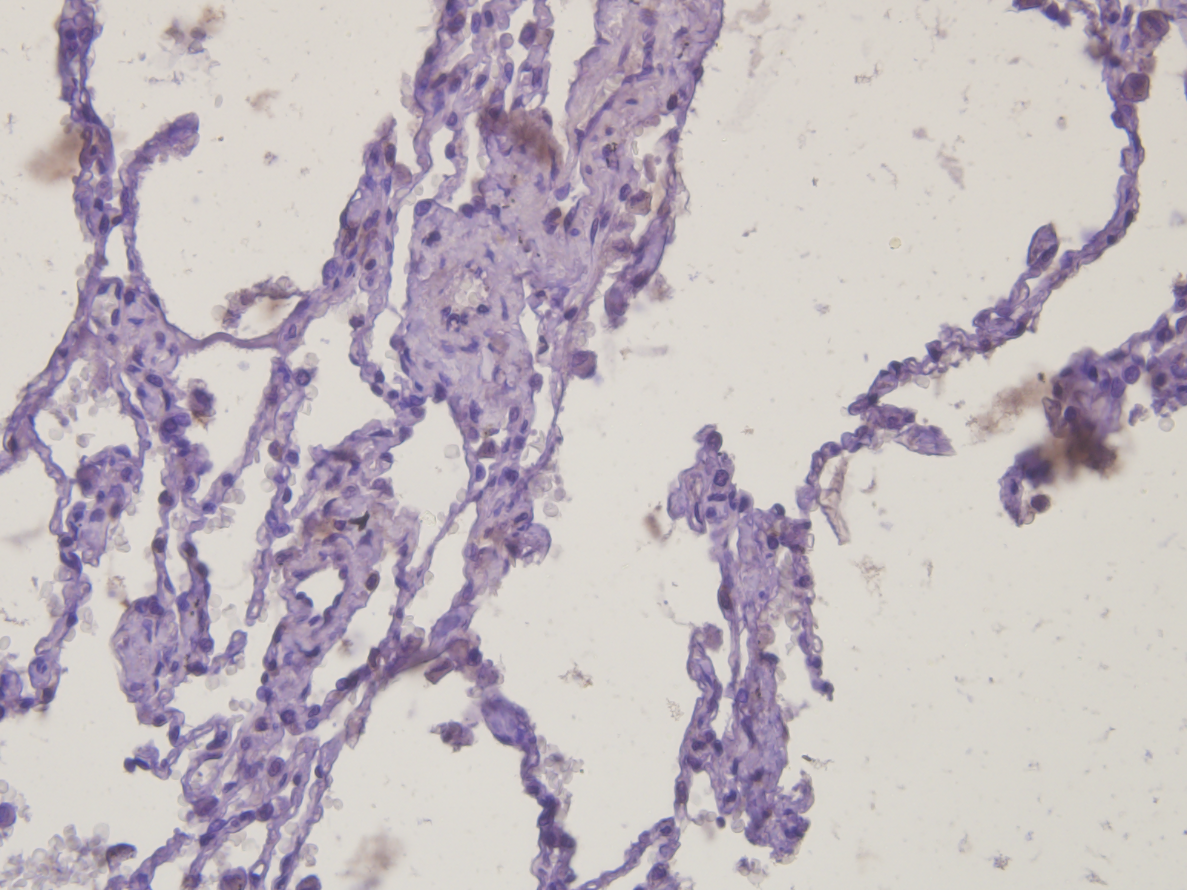

Supplement: S25 File — (ZIP) [file pone.0337223.s026.zip › 468077-400X-CA-N-/468077-400X-N (4).tif]

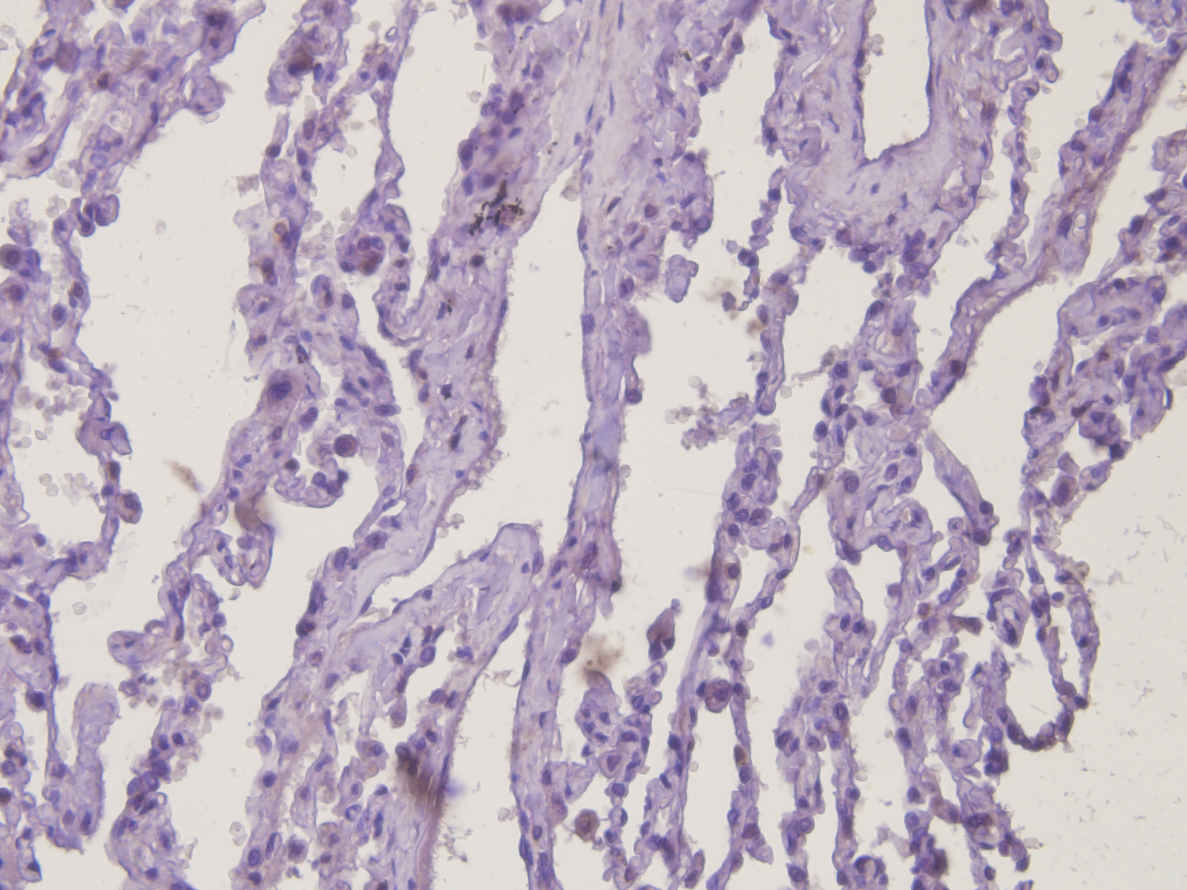

Supplement: S25 File — (ZIP) [file pone.0337223.s026.zip › 468077-400X-CA-N-/468077-400X-N (5).tif]

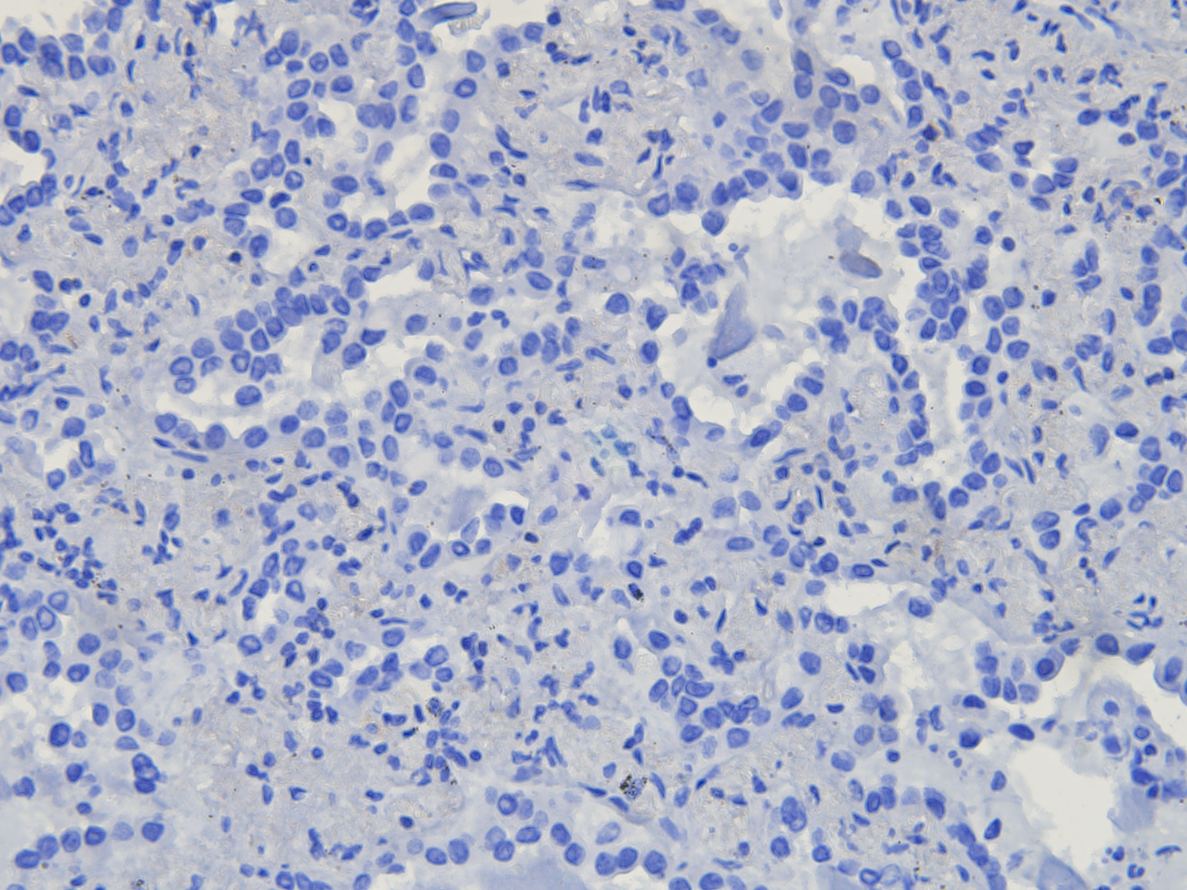

Supplement: S26 File — (ZIP) [file pone.0337223.s027.zip › 469144-400X-CA-N/469144-400x-ca (1).tif]

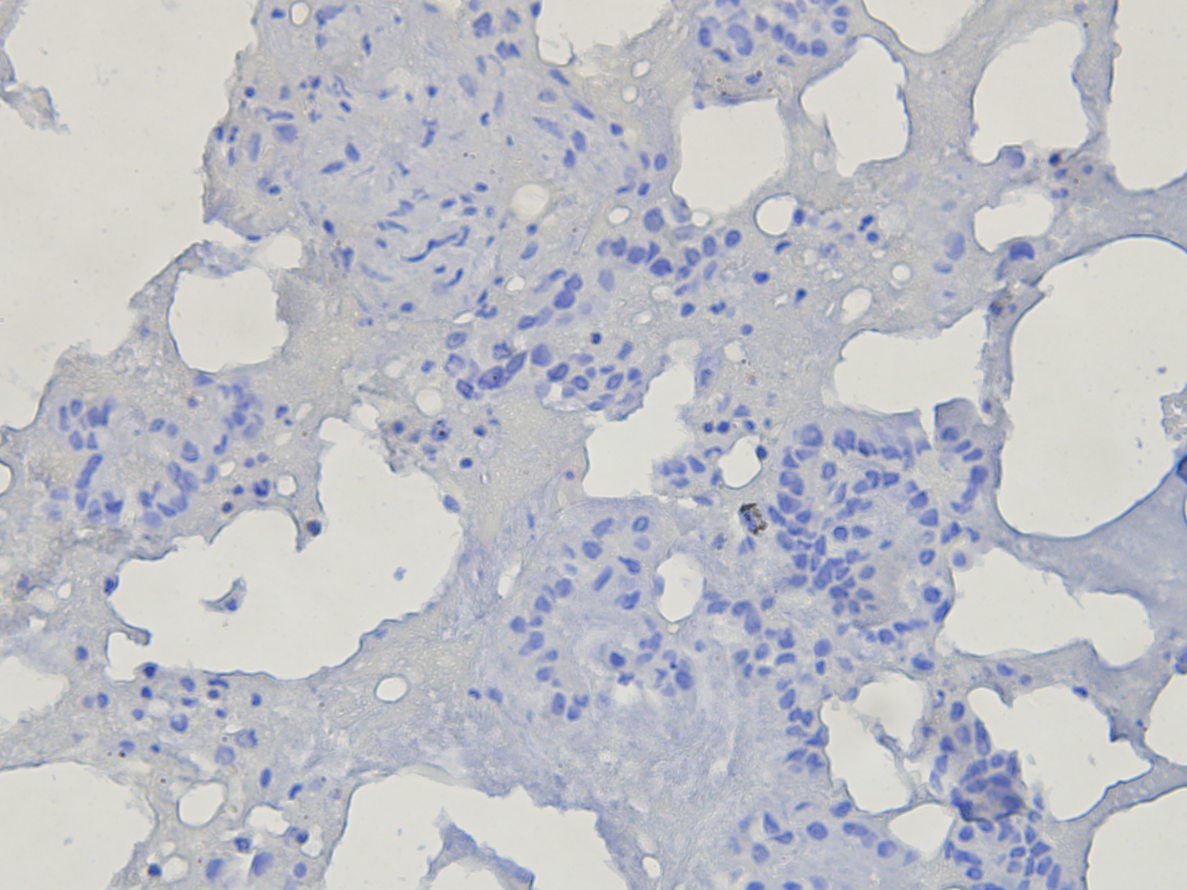

Supplement: S26 File — (ZIP) [file pone.0337223.s027.zip › 469144-400X-CA-N/469144-400x-ca (2).tif]

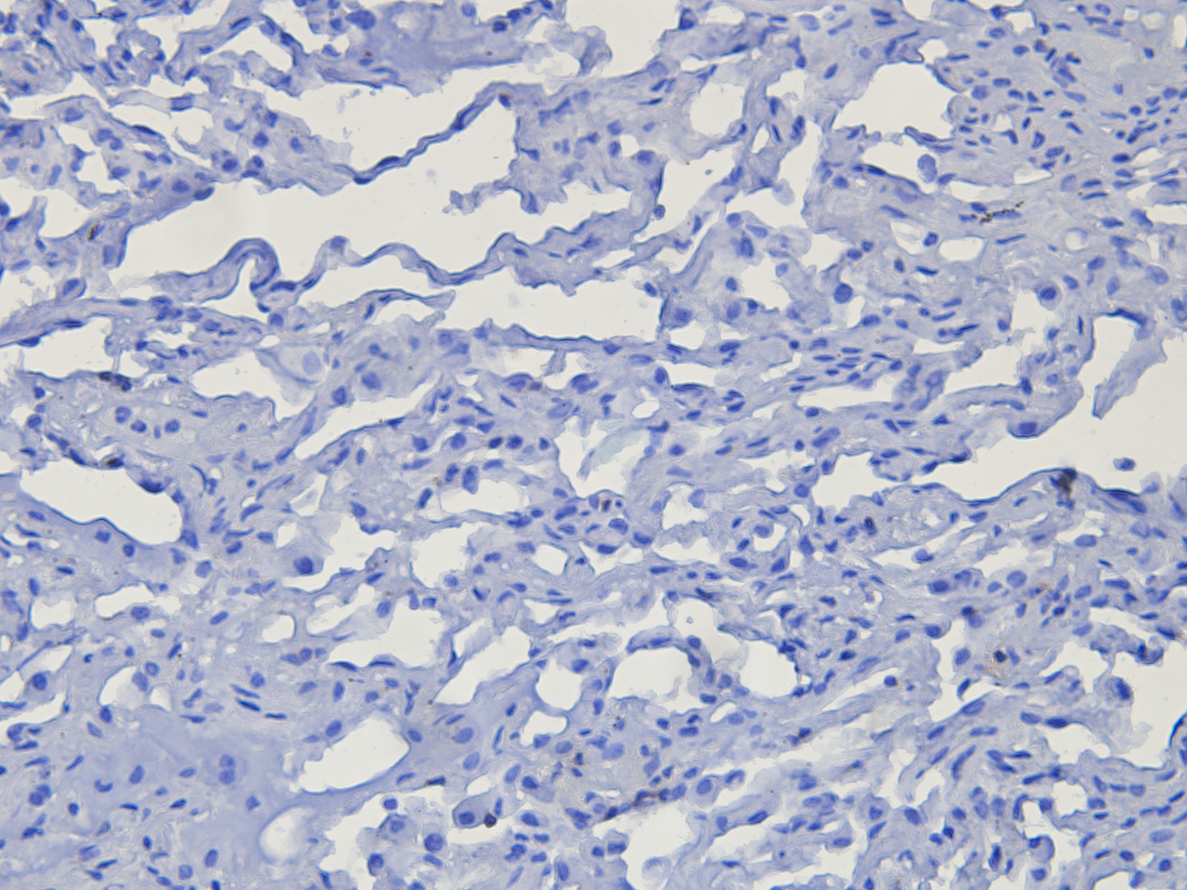

Supplement: S26 File — (ZIP) [file pone.0337223.s027.zip › 469144-400X-CA-N/469144-400x-ca (3).tif]

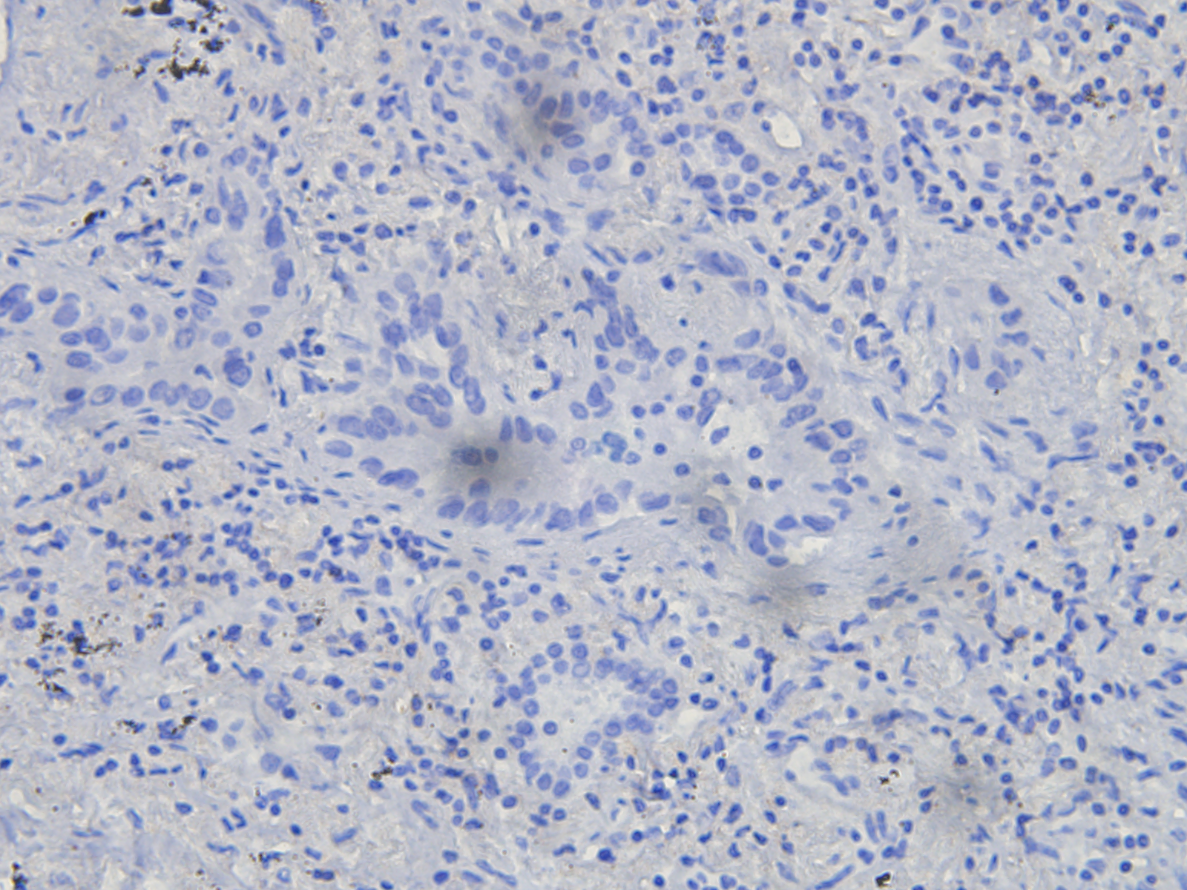

Supplement: S26 File — (ZIP) [file pone.0337223.s027.zip › 469144-400X-CA-N/469144-400x-ca (4).tif]

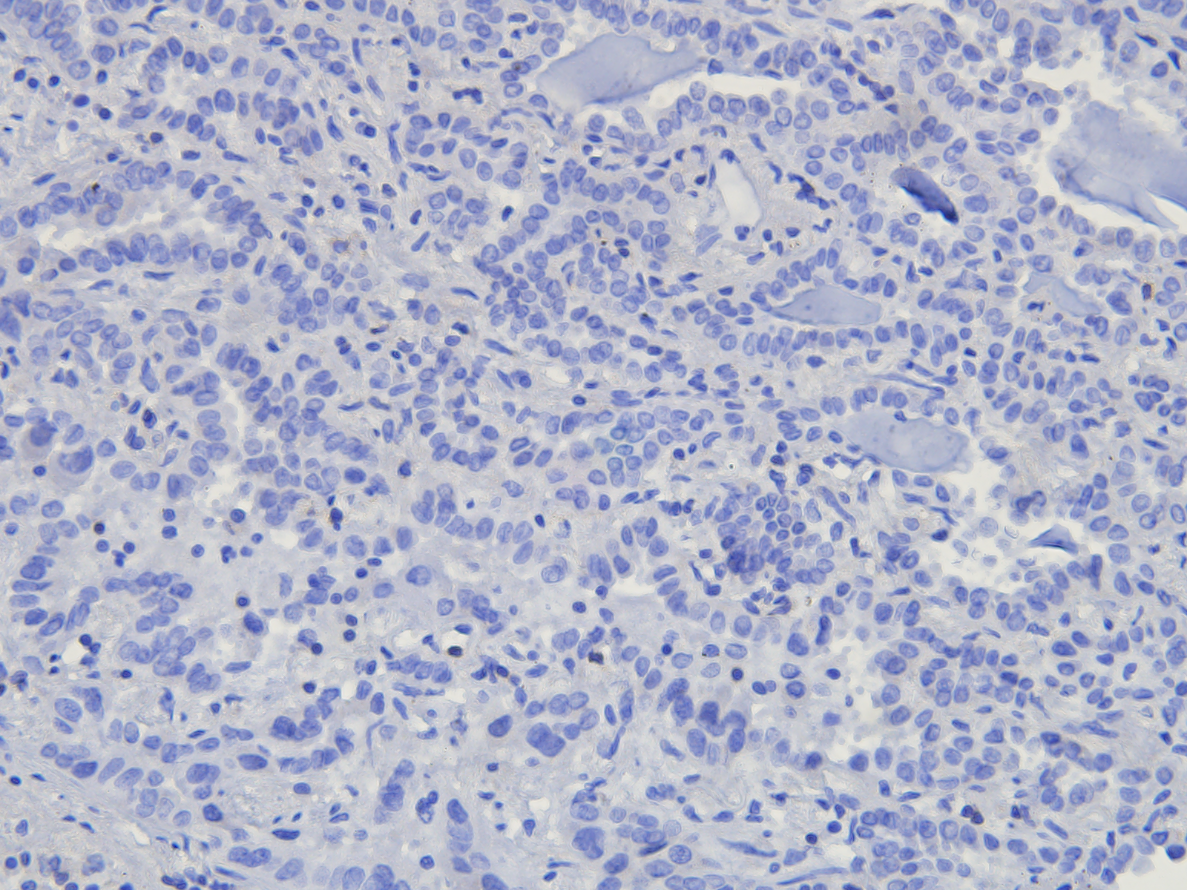

Supplement: S26 File — (ZIP) [file pone.0337223.s027.zip › 469144-400X-CA-N/469144-400x-ca (5).tif]

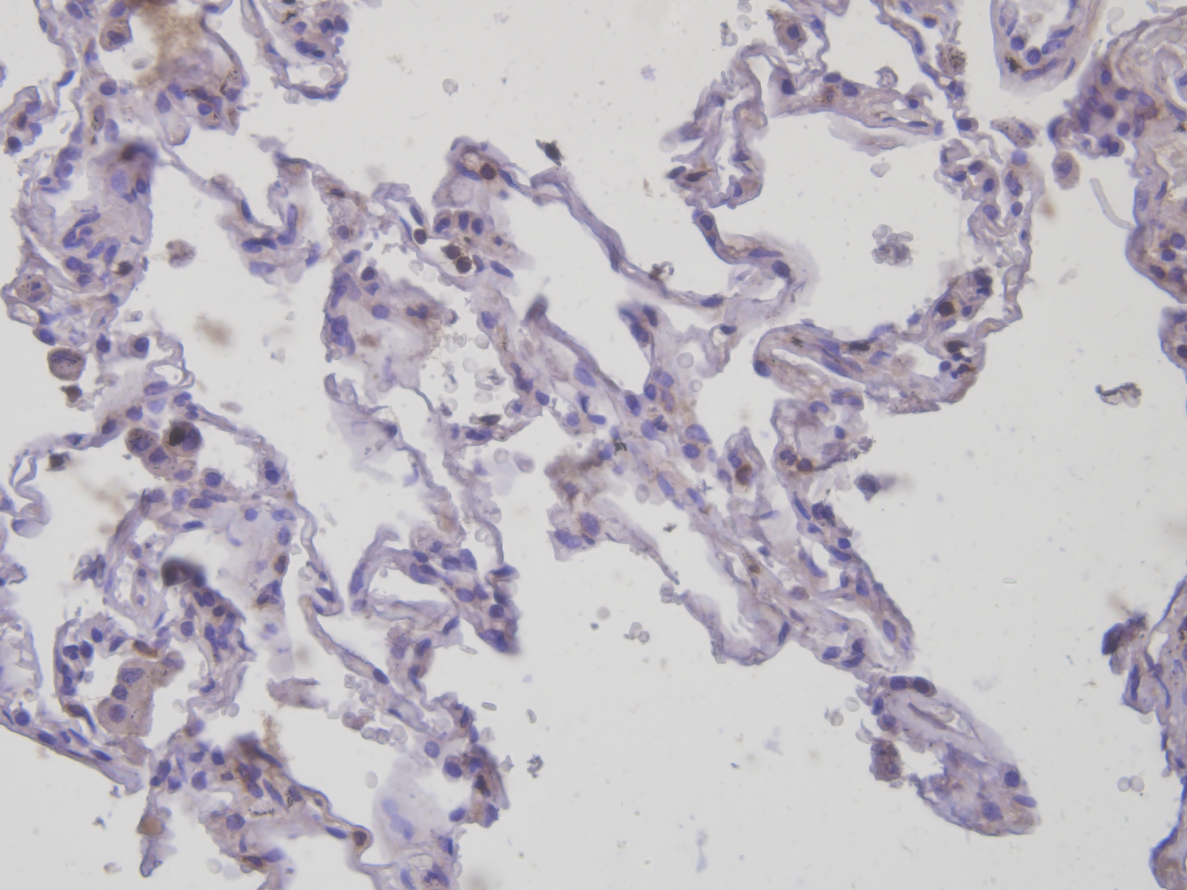

Supplement: S26 File — (ZIP) [file pone.0337223.s027.zip › 469144-400X-CA-N/469144-400x-n (1).tif]

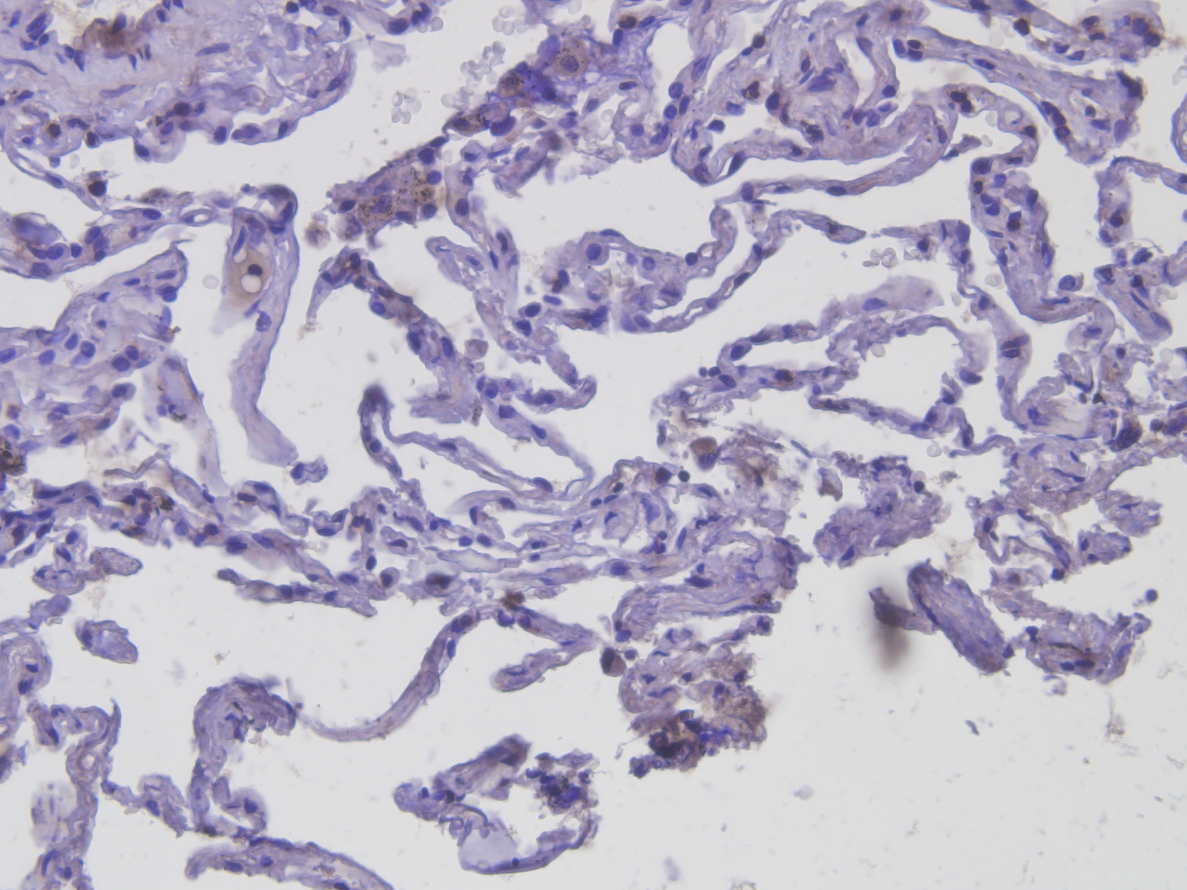

Supplement: S26 File — (ZIP) [file pone.0337223.s027.zip › 469144-400X-CA-N/469144-400x-n (2).tif]

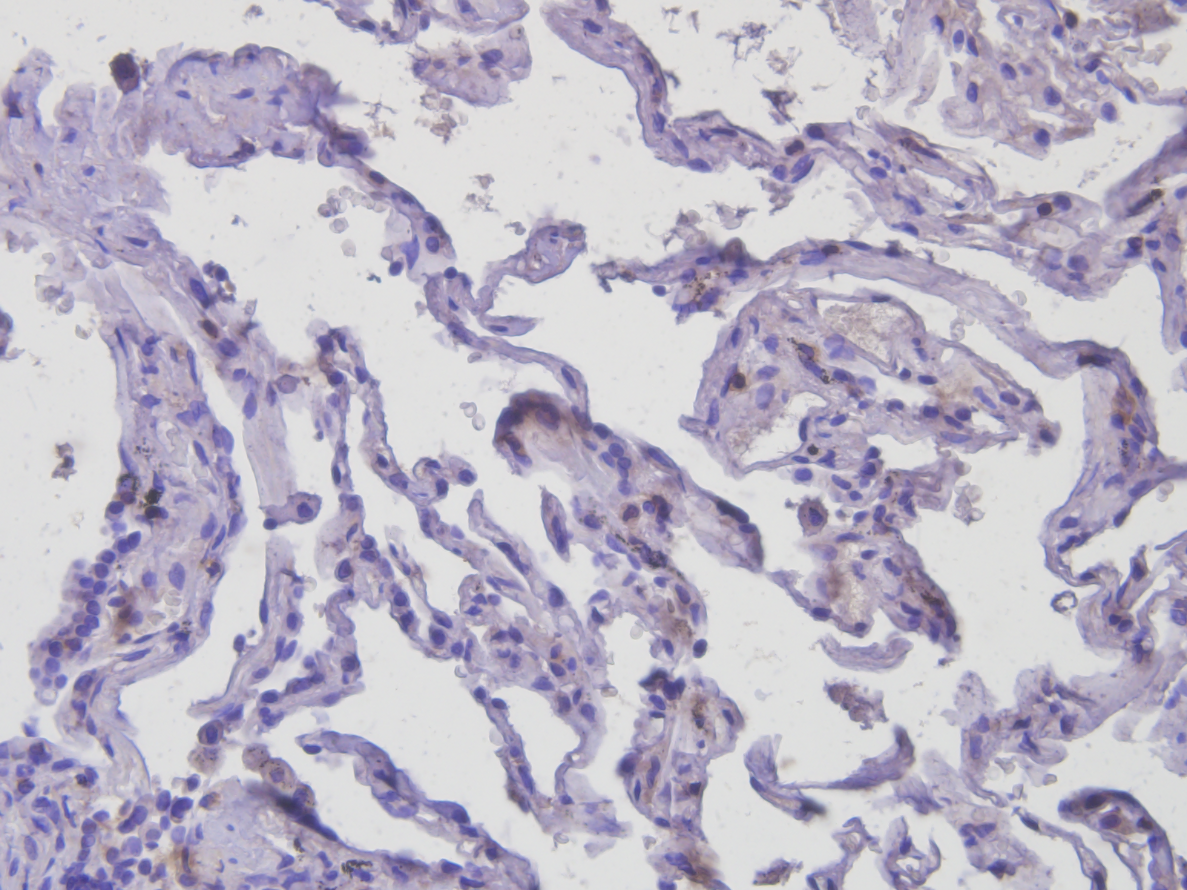

Supplement: S26 File — (ZIP) [file pone.0337223.s027.zip › 469144-400X-CA-N/469144-400x-n (3).tif]

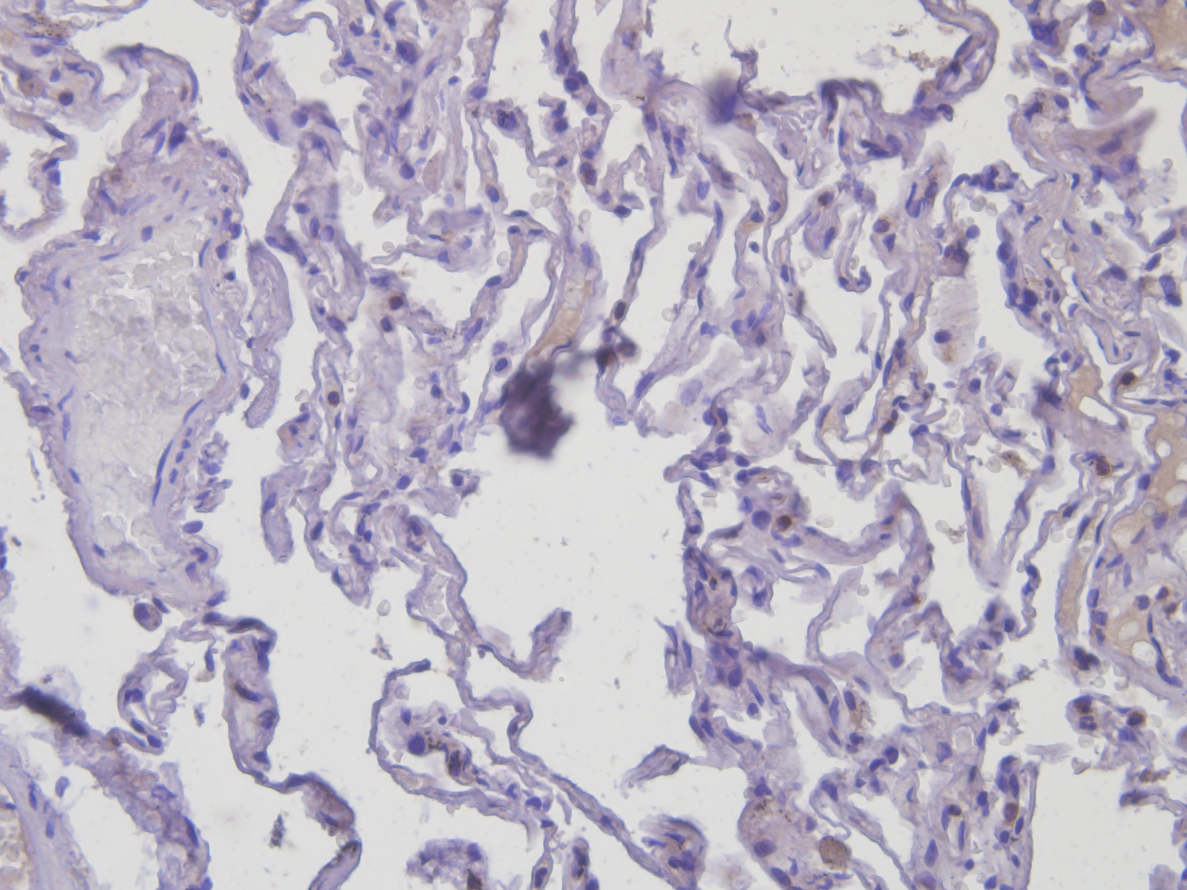

Supplement: S26 File — (ZIP) [file pone.0337223.s027.zip › 469144-400X-CA-N/469144-400x-n (4).tif]

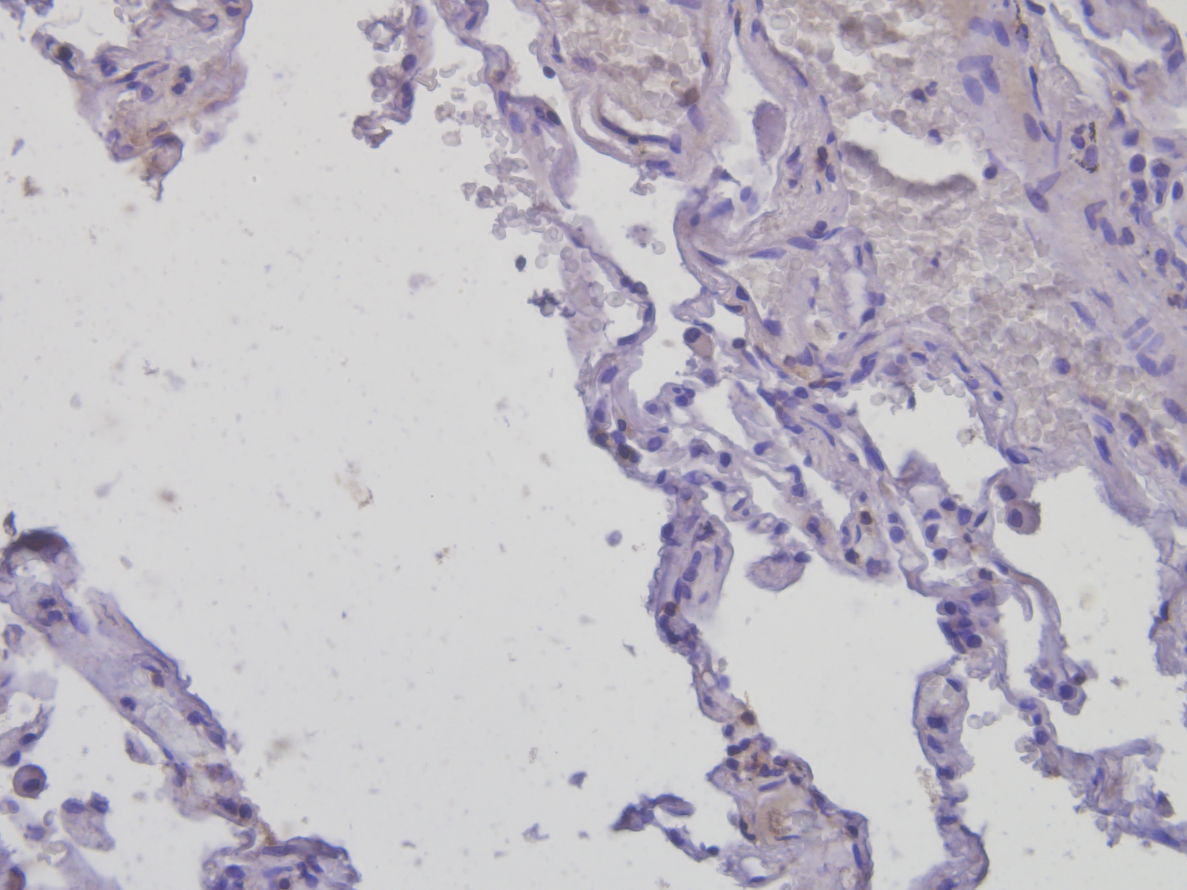

Supplement: S26 File — (ZIP) [file pone.0337223.s027.zip › 469144-400X-CA-N/469144-400x-n (5).tif]

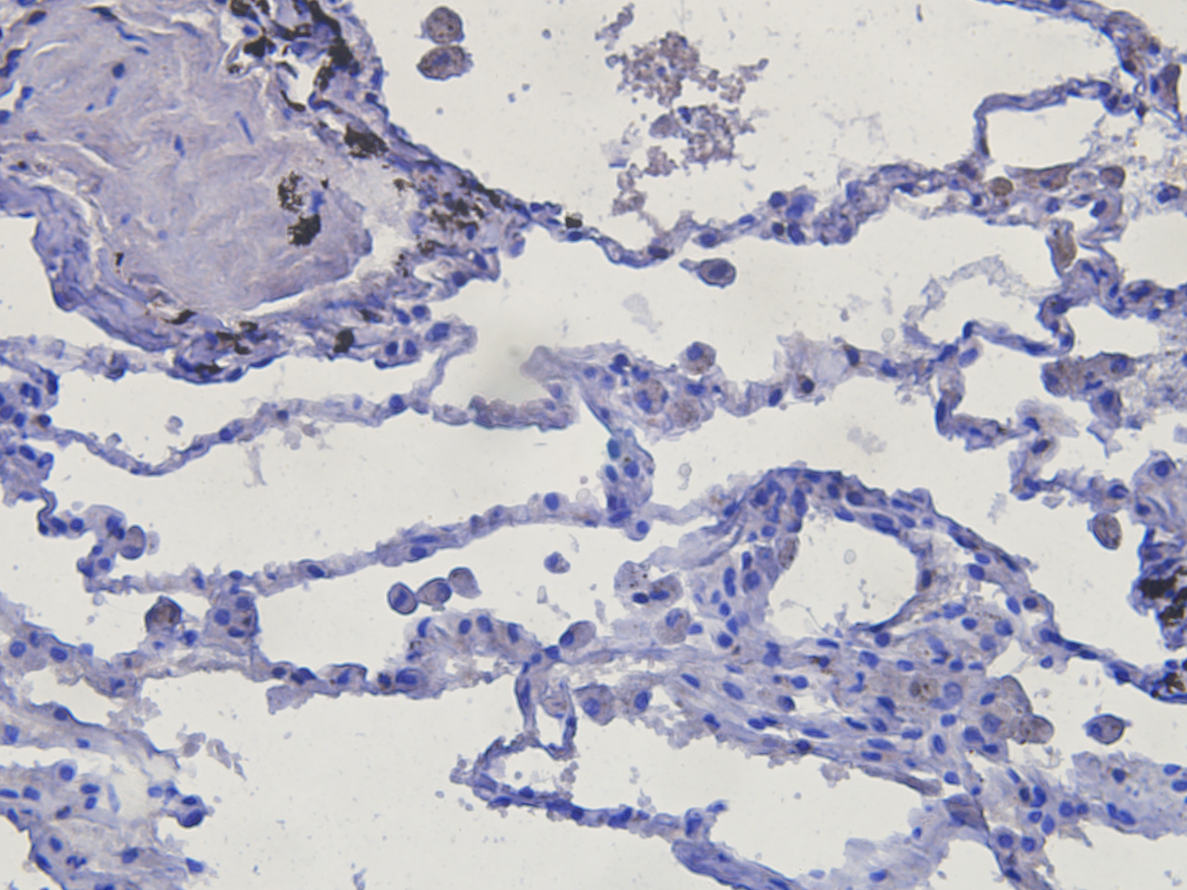

Supplement: S27 File — (ZIP) [file pone.0337223.s028.zip › 469316-400X-CA-N/469316-ca (1).tif]

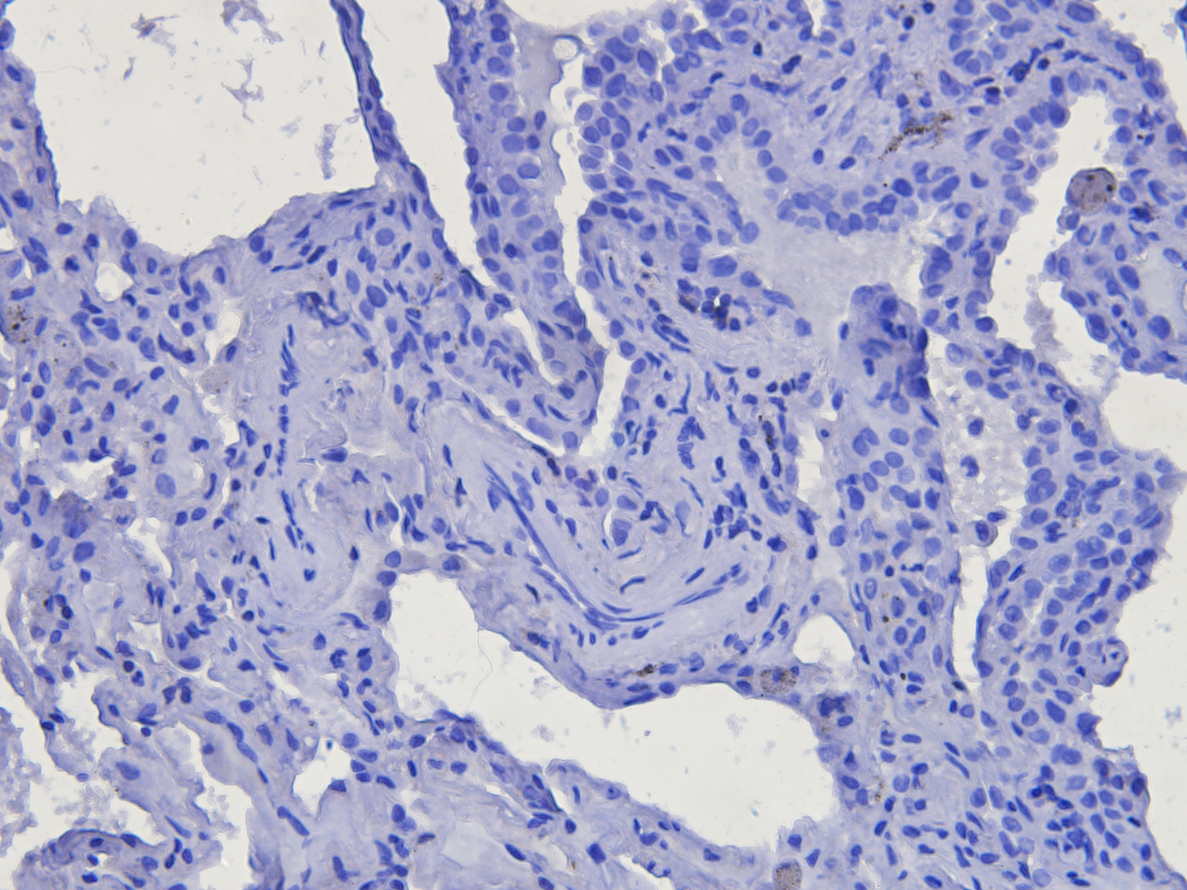

Supplement: S27 File — (ZIP) [file pone.0337223.s028.zip › 469316-400X-CA-N/469316-ca (2).tif]

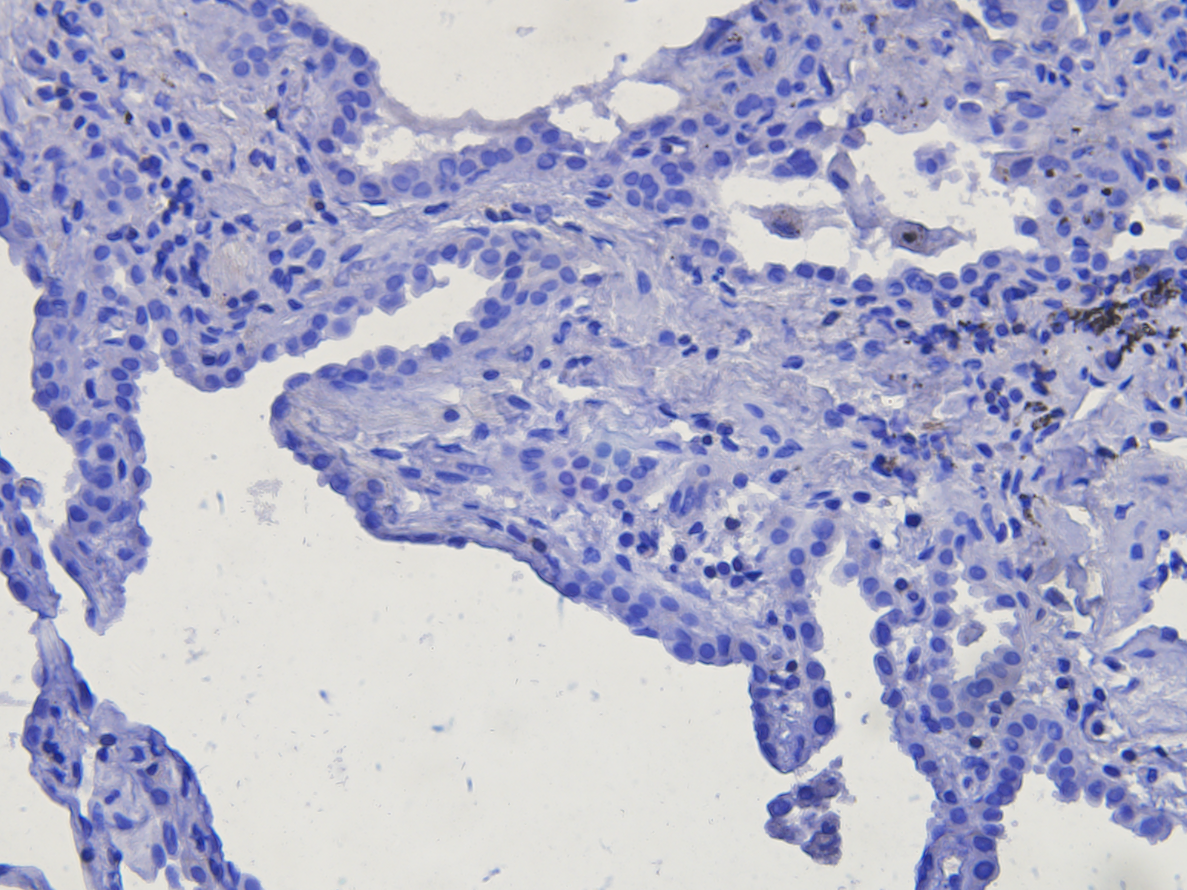

Supplement: S27 File — (ZIP) [file pone.0337223.s028.zip › 469316-400X-CA-N/469316-ca (3).tif]

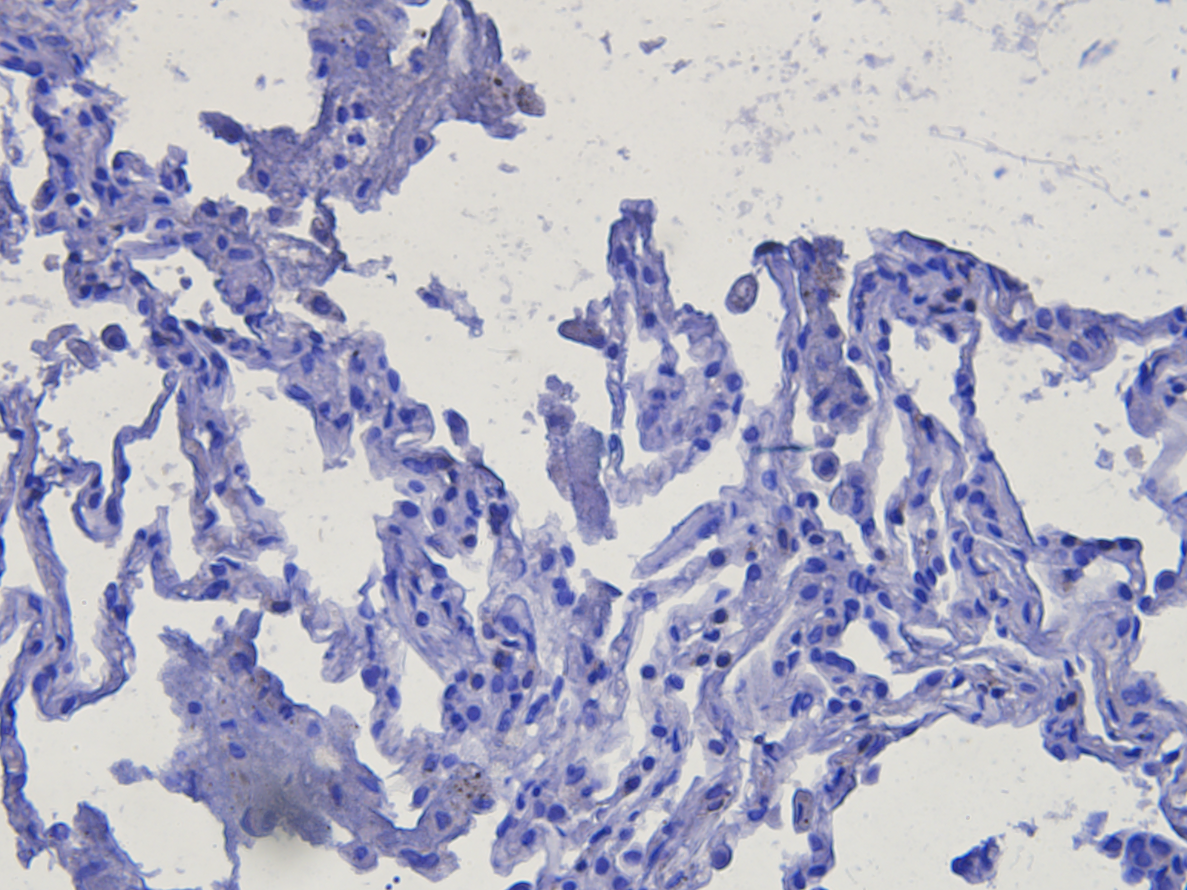

Supplement: S27 File — (ZIP) [file pone.0337223.s028.zip › 469316-400X-CA-N/469316-ca (4).tif]

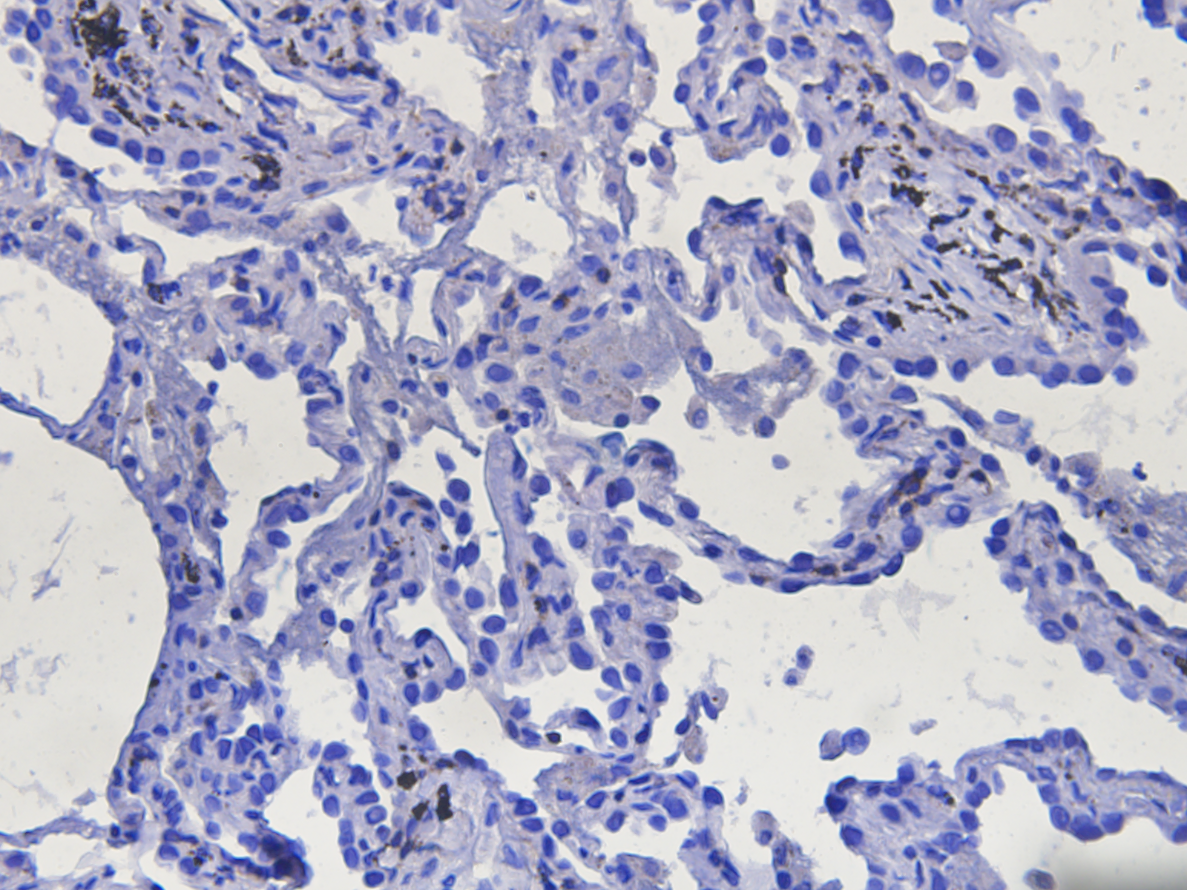

Supplement: S27 File — (ZIP) [file pone.0337223.s028.zip › 469316-400X-CA-N/469316-ca (5).tif]

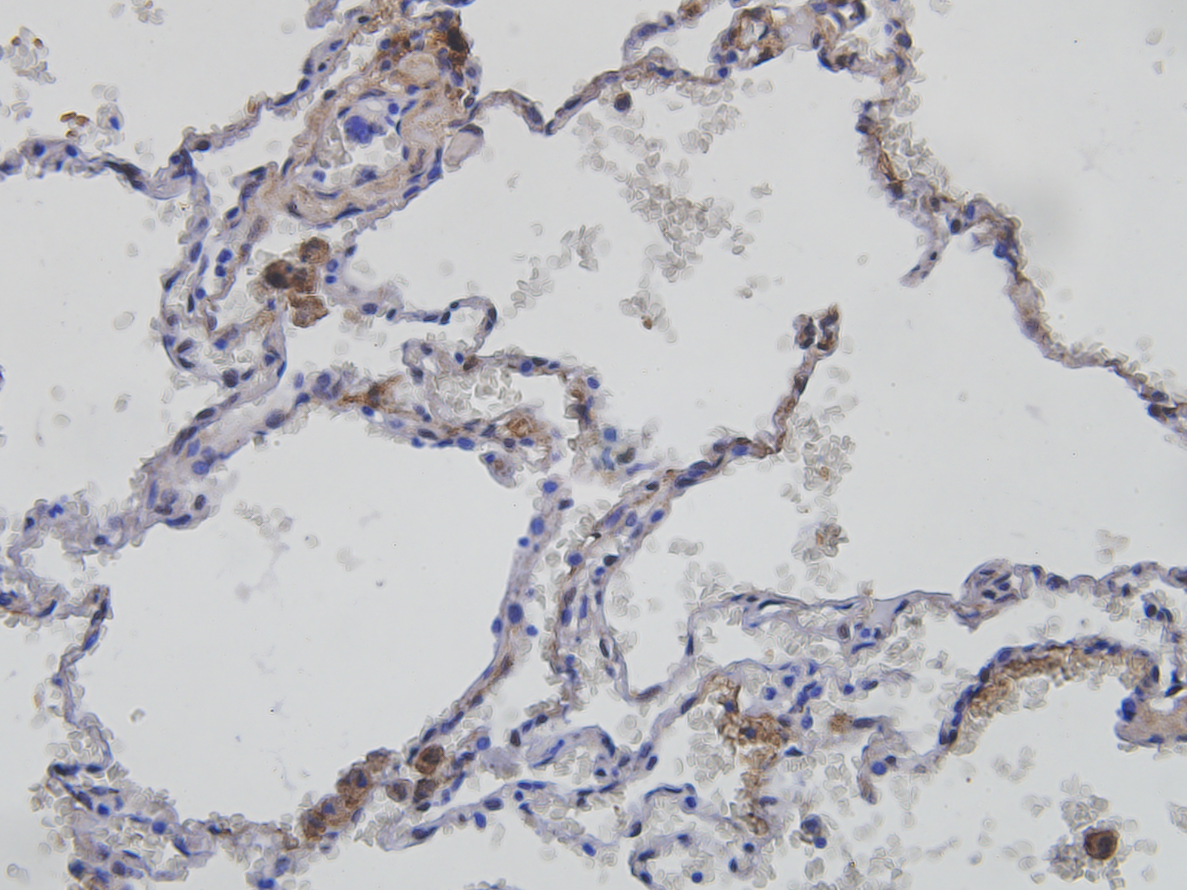

Supplement: S27 File — (ZIP) [file pone.0337223.s028.zip › 469316-400X-CA-N/469316-n (1).tif]

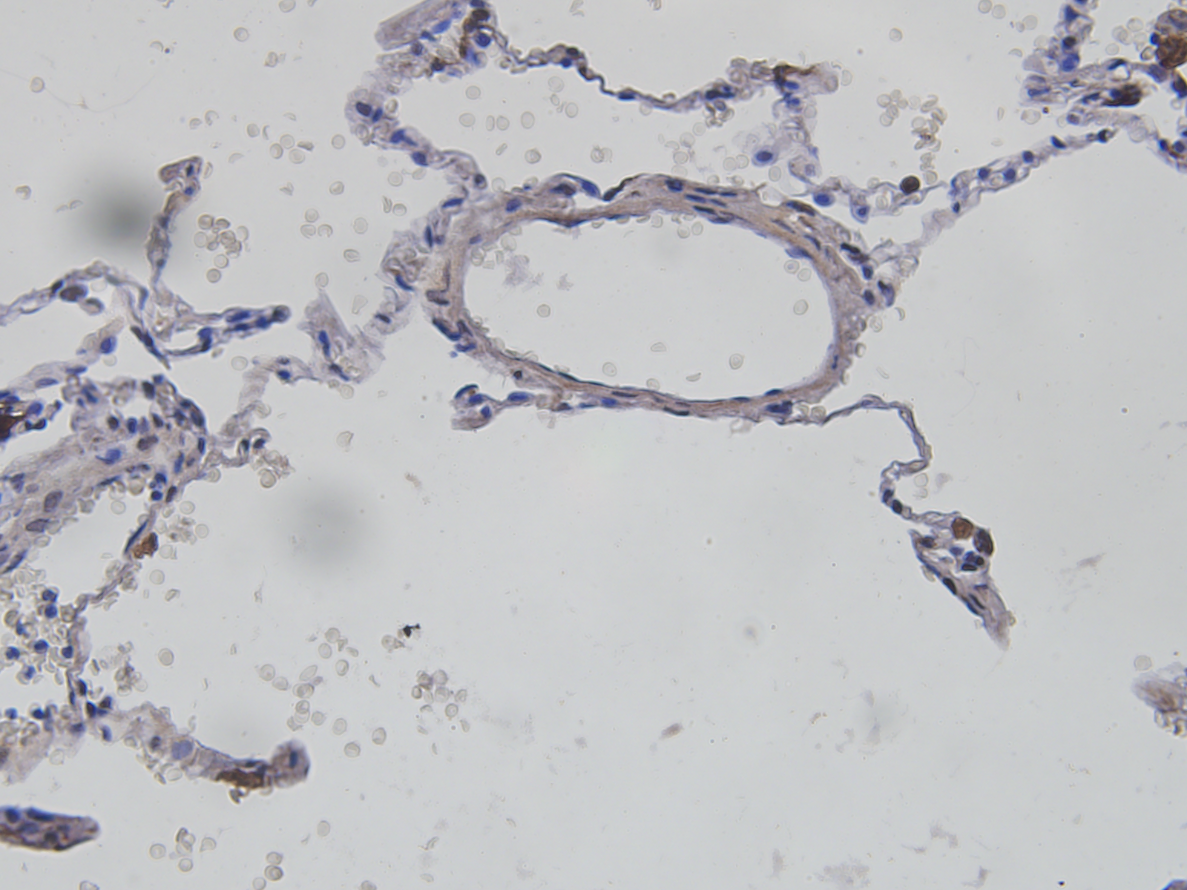

Supplement: S27 File — (ZIP) [file pone.0337223.s028.zip › 469316-400X-CA-N/469316-n (2).tif]

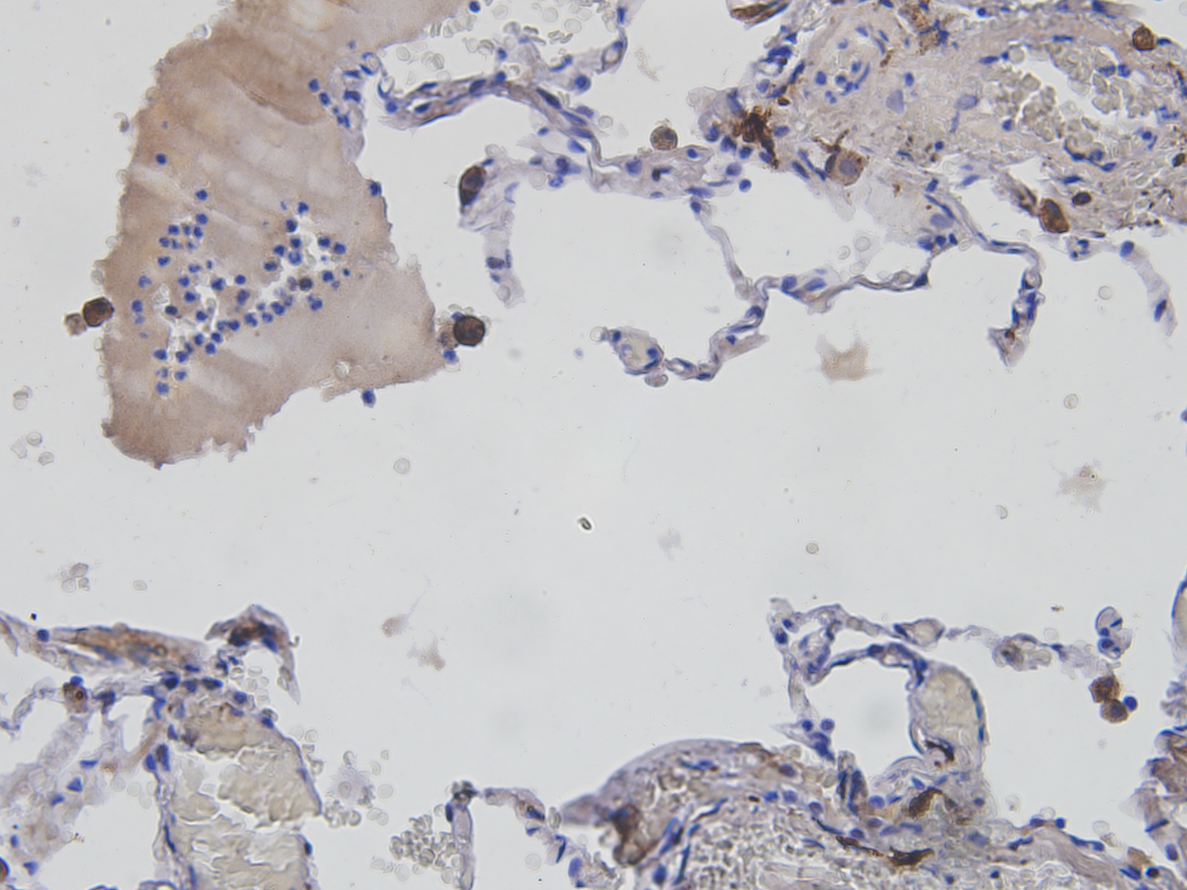

Supplement: S27 File — (ZIP) [file pone.0337223.s028.zip › 469316-400X-CA-N/469316-n (3).tif]

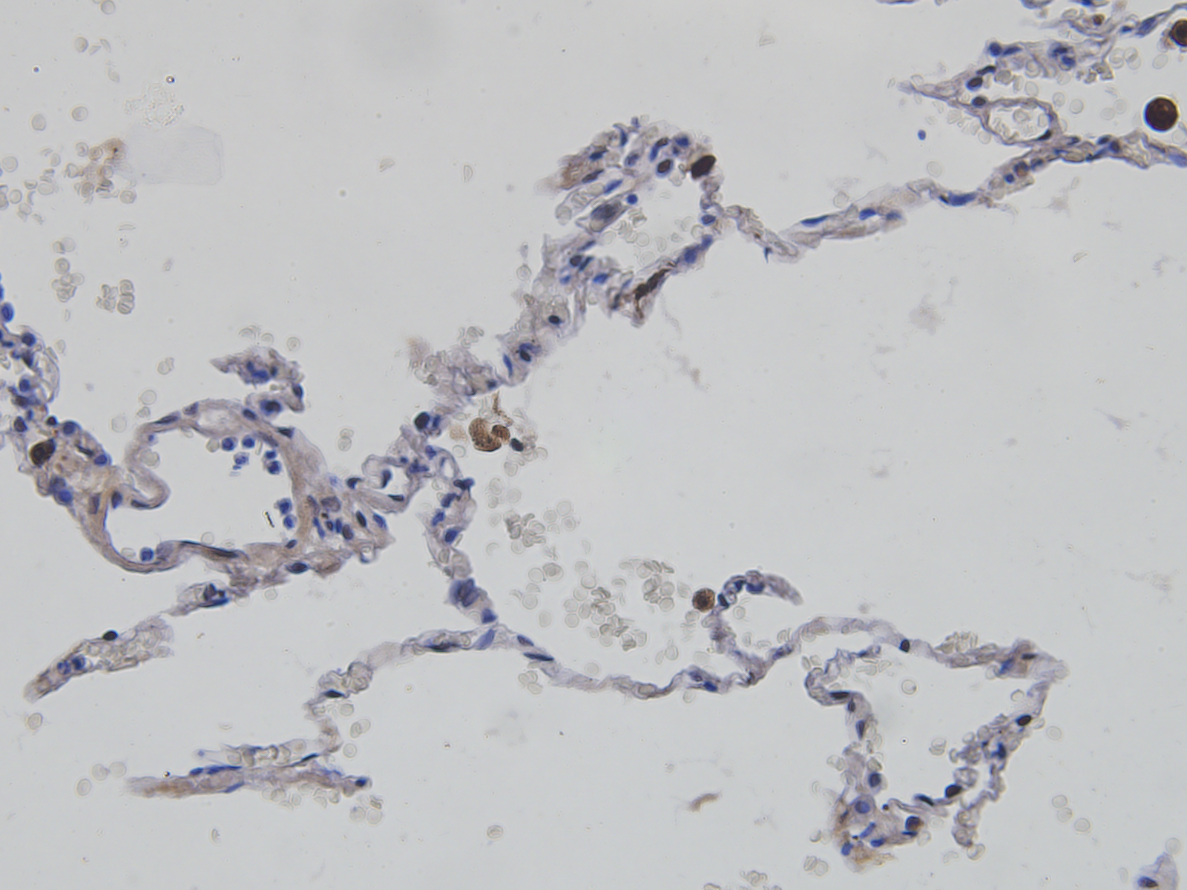

Supplement: S27 File — (ZIP) [file pone.0337223.s028.zip › 469316-400X-CA-N/469316-n (4).tif]

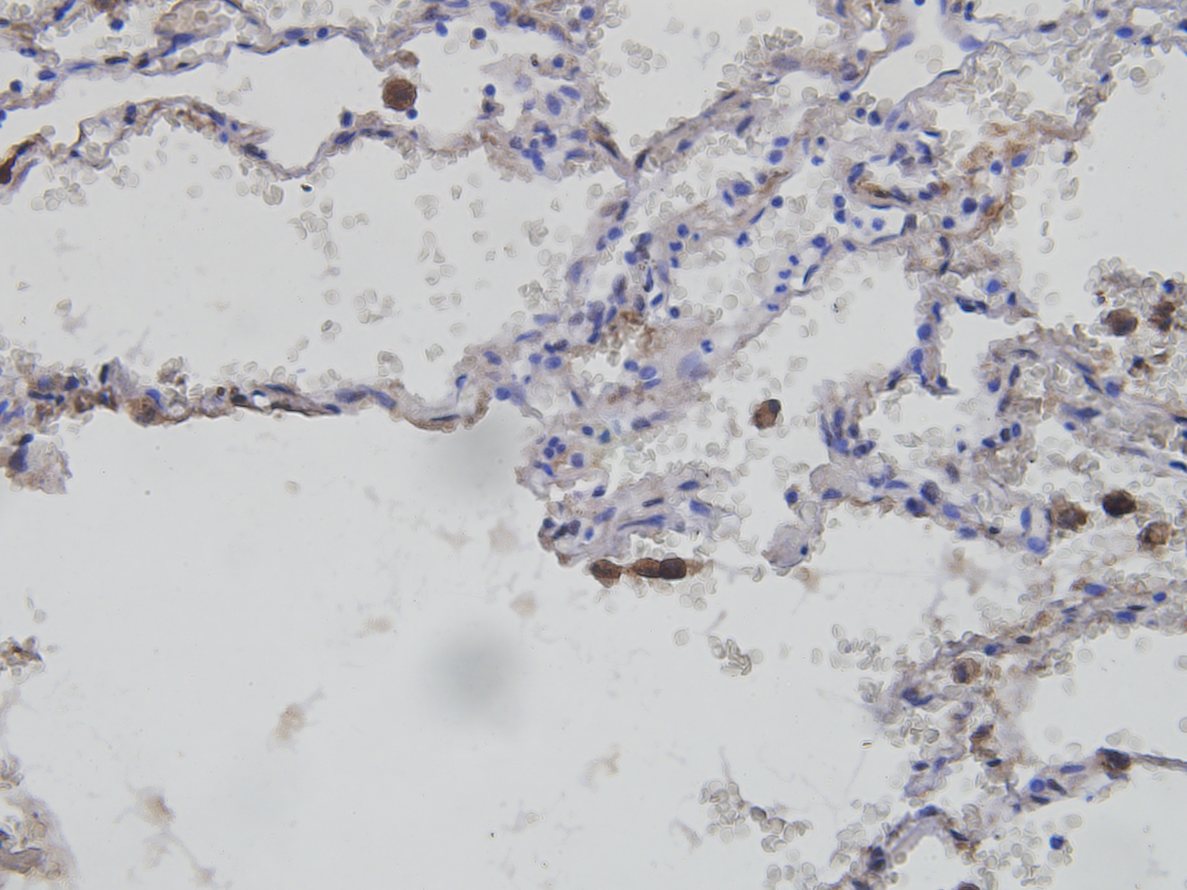

Supplement: S27 File — (ZIP) [file pone.0337223.s028.zip › 469316-400X-CA-N/469316-n (5).tif]

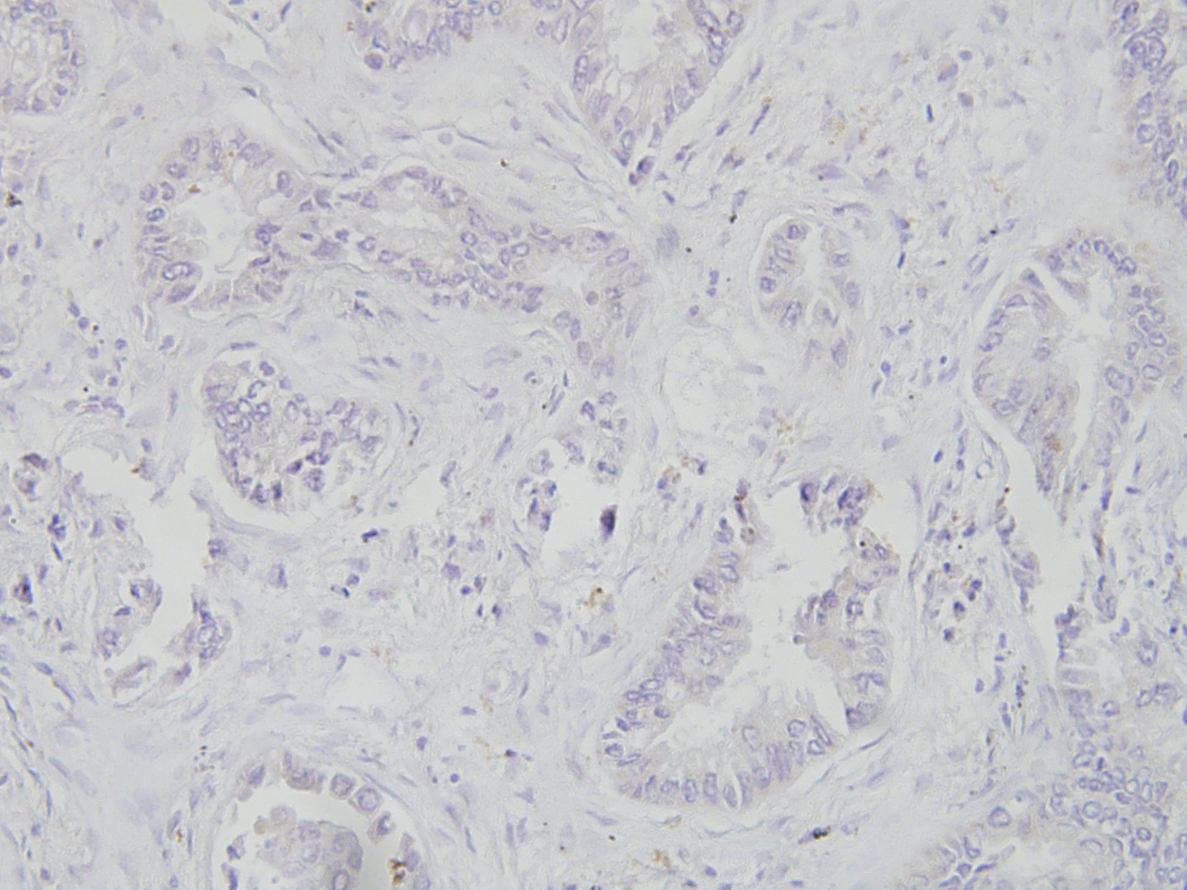

Supplement: S28 File — (ZIP) [file pone.0337223.s029.zip › 474453-400X-N-CA/474453-ca (1).tif]

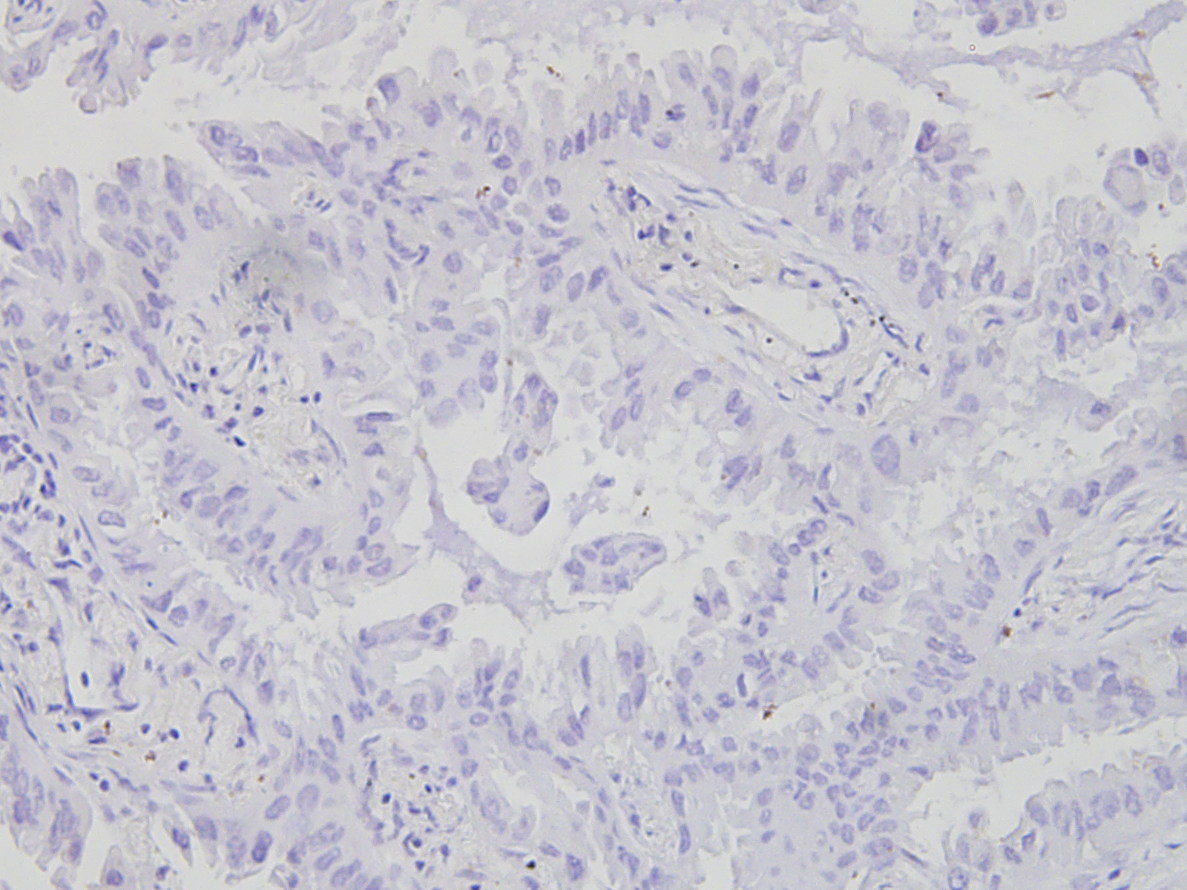

Supplement: S28 File — (ZIP) [file pone.0337223.s029.zip › 474453-400X-N-CA/474453-ca (2).tif]

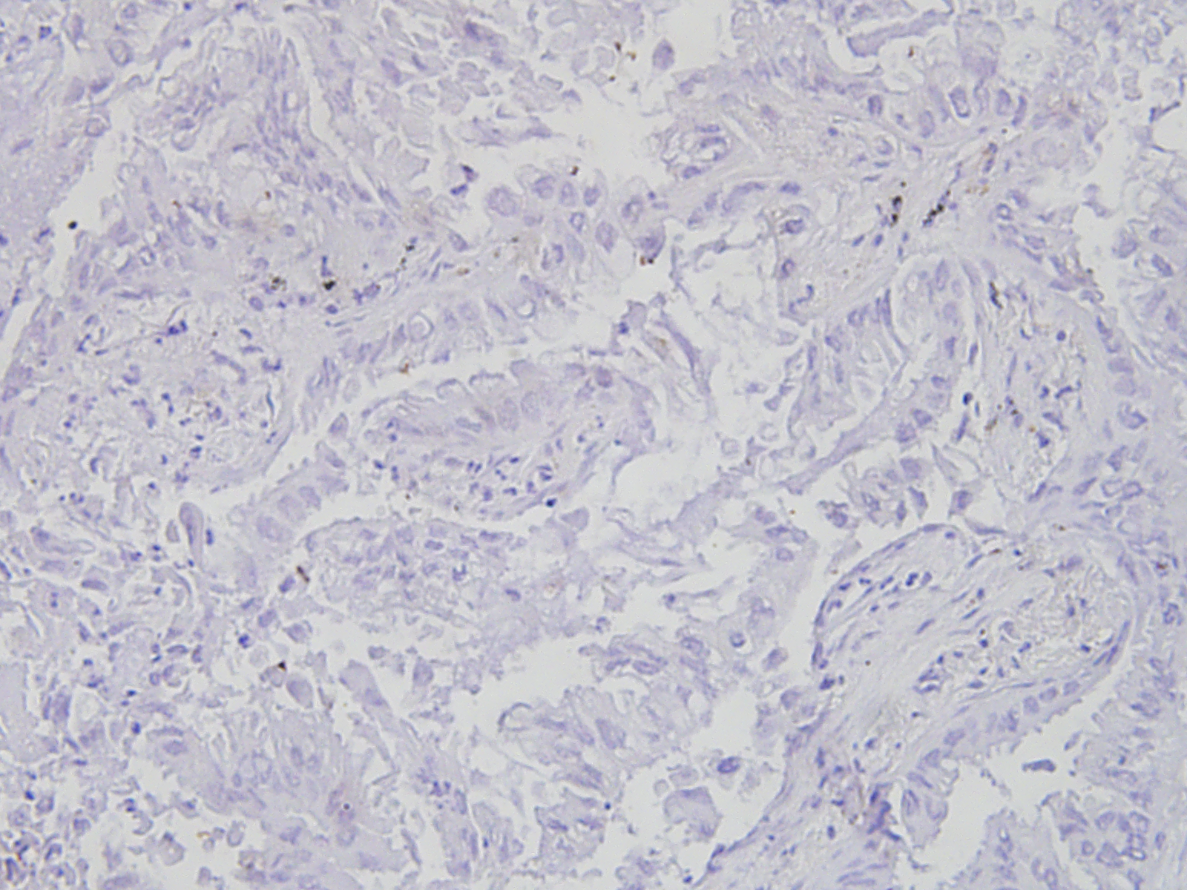

Supplement: S28 File — (ZIP) [file pone.0337223.s029.zip › 474453-400X-N-CA/474453-ca (3).tif]

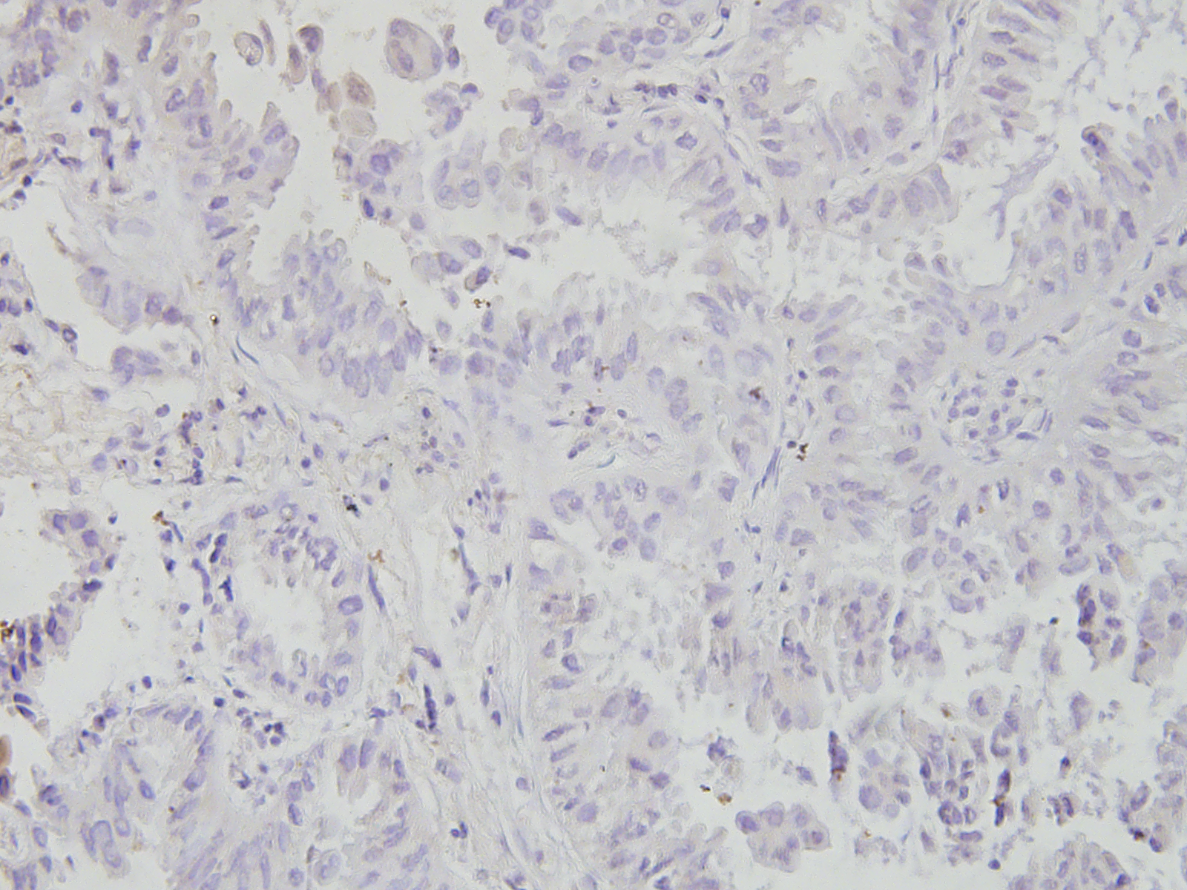

Supplement: S28 File — (ZIP) [file pone.0337223.s029.zip › 474453-400X-N-CA/474453-ca (4).tif]

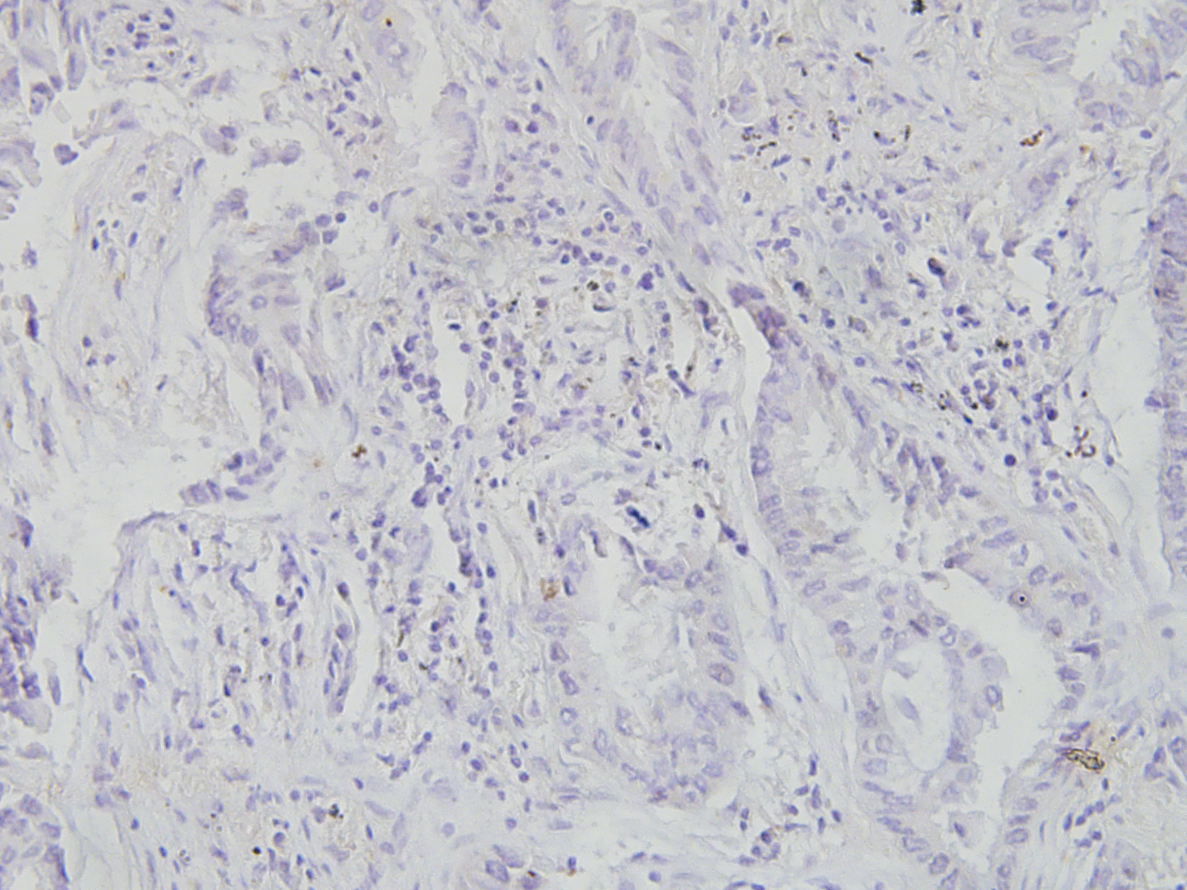

Supplement: S28 File — (ZIP) [file pone.0337223.s029.zip › 474453-400X-N-CA/474453-ca (5).tif]

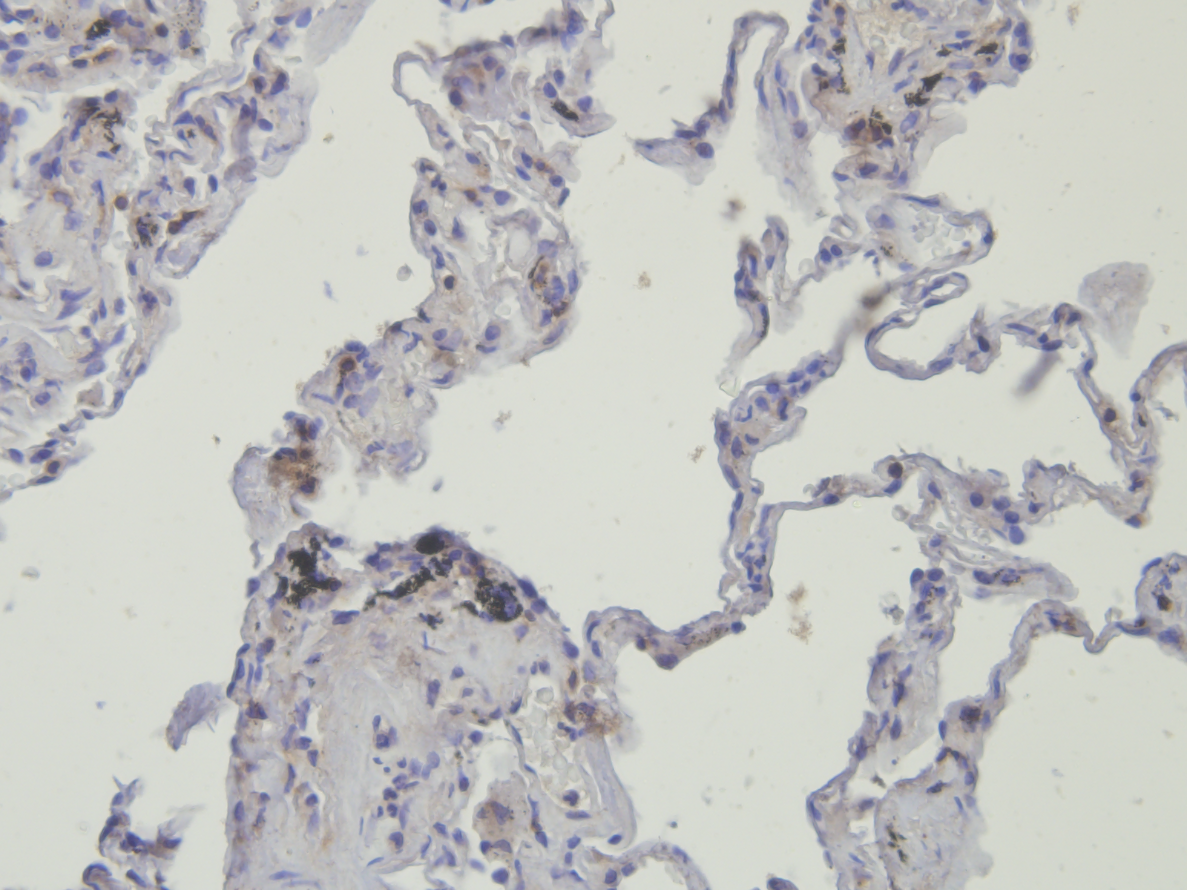

Supplement: S28 File — (ZIP) [file pone.0337223.s029.zip › 474453-400X-N-CA/474453-n (1).tif]

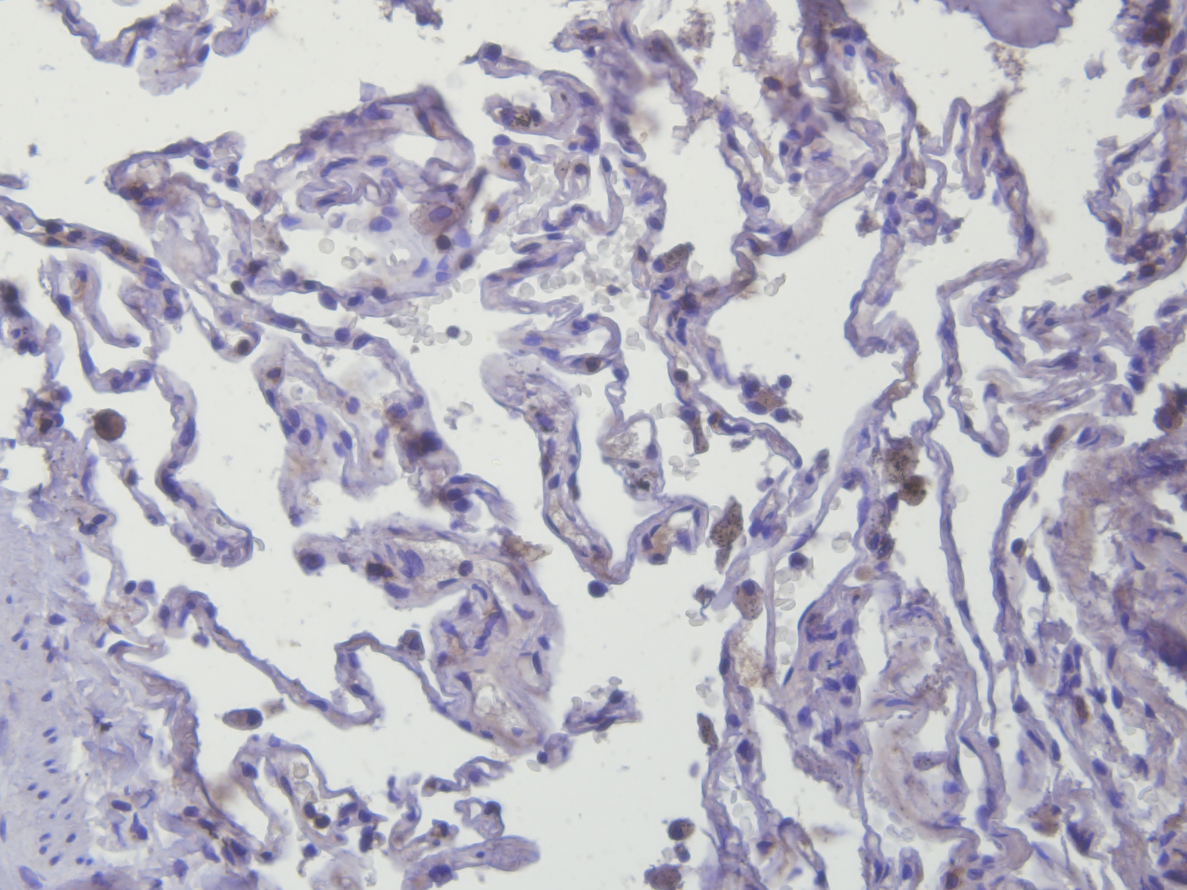

Supplement: S28 File — (ZIP) [file pone.0337223.s029.zip › 474453-400X-N-CA/474453-n (2).tif]

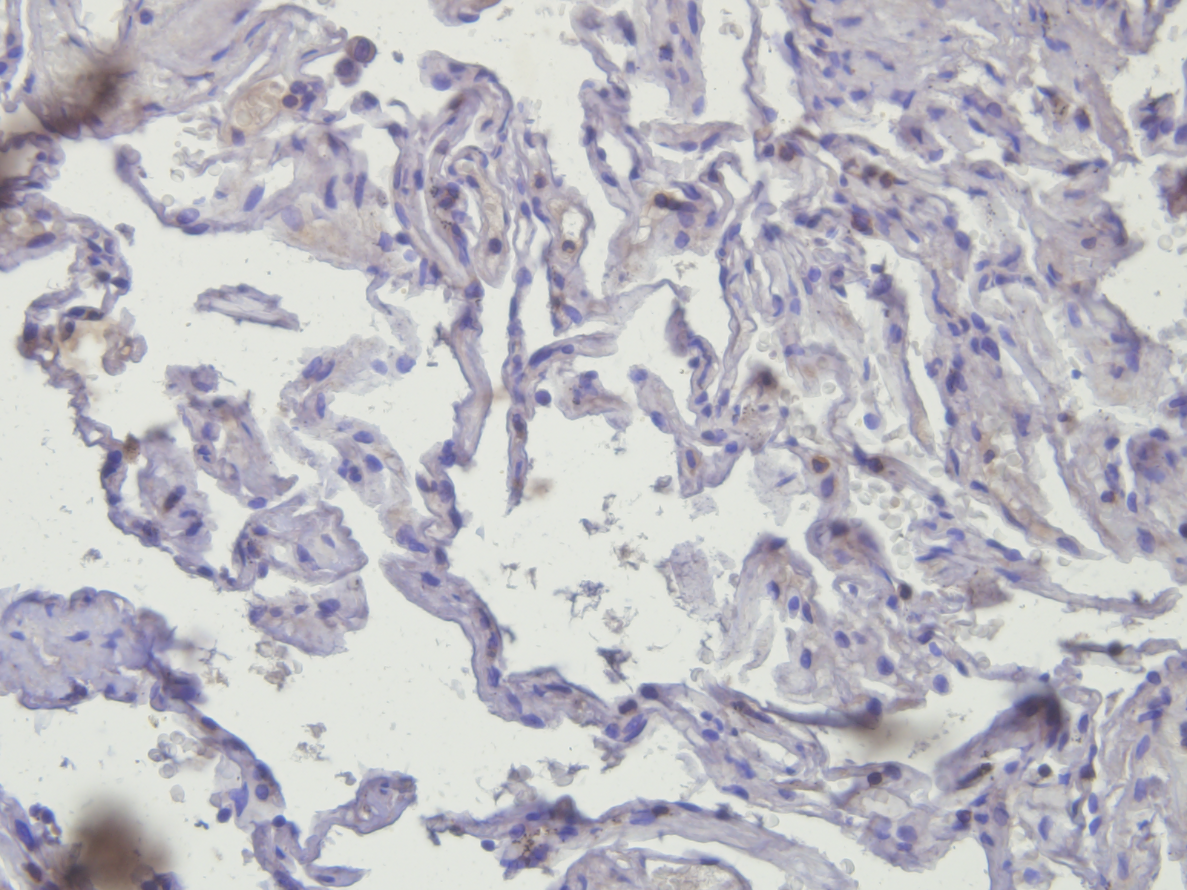

Supplement: S28 File — (ZIP) [file pone.0337223.s029.zip › 474453-400X-N-CA/474453-n (3).tif]

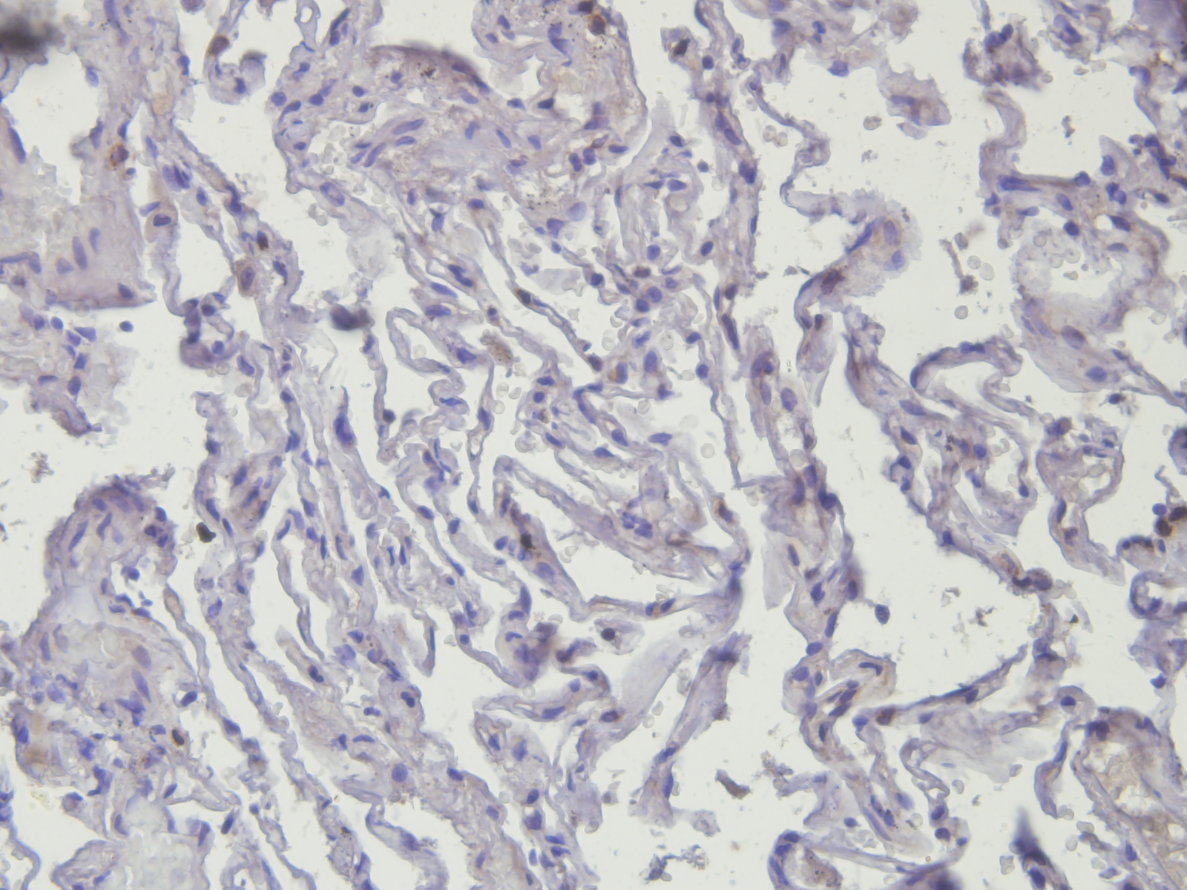

Supplement: S28 File — (ZIP) [file pone.0337223.s029.zip › 474453-400X-N-CA/474453-n (4).tif]

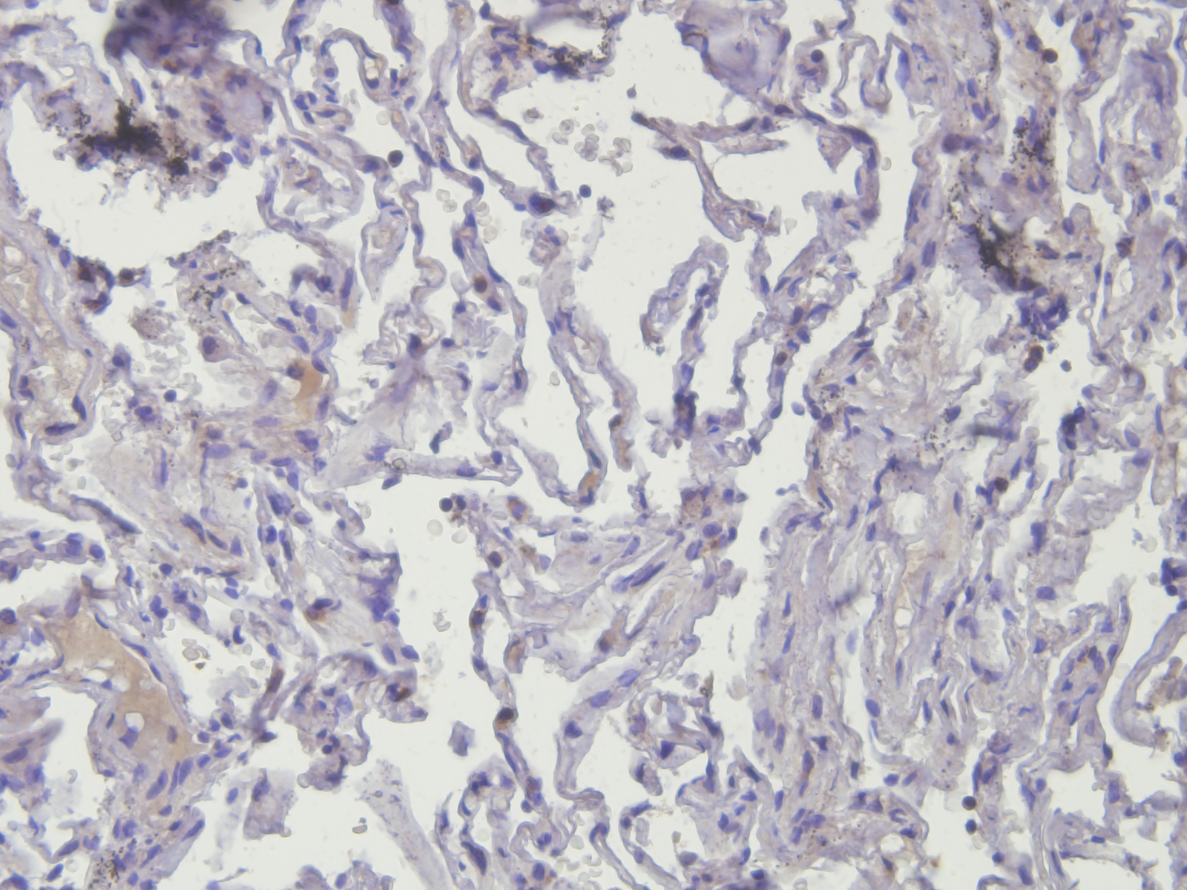

Supplement: S28 File — (ZIP) [file pone.0337223.s029.zip › 474453-400X-N-CA/474453-n (5).tif]

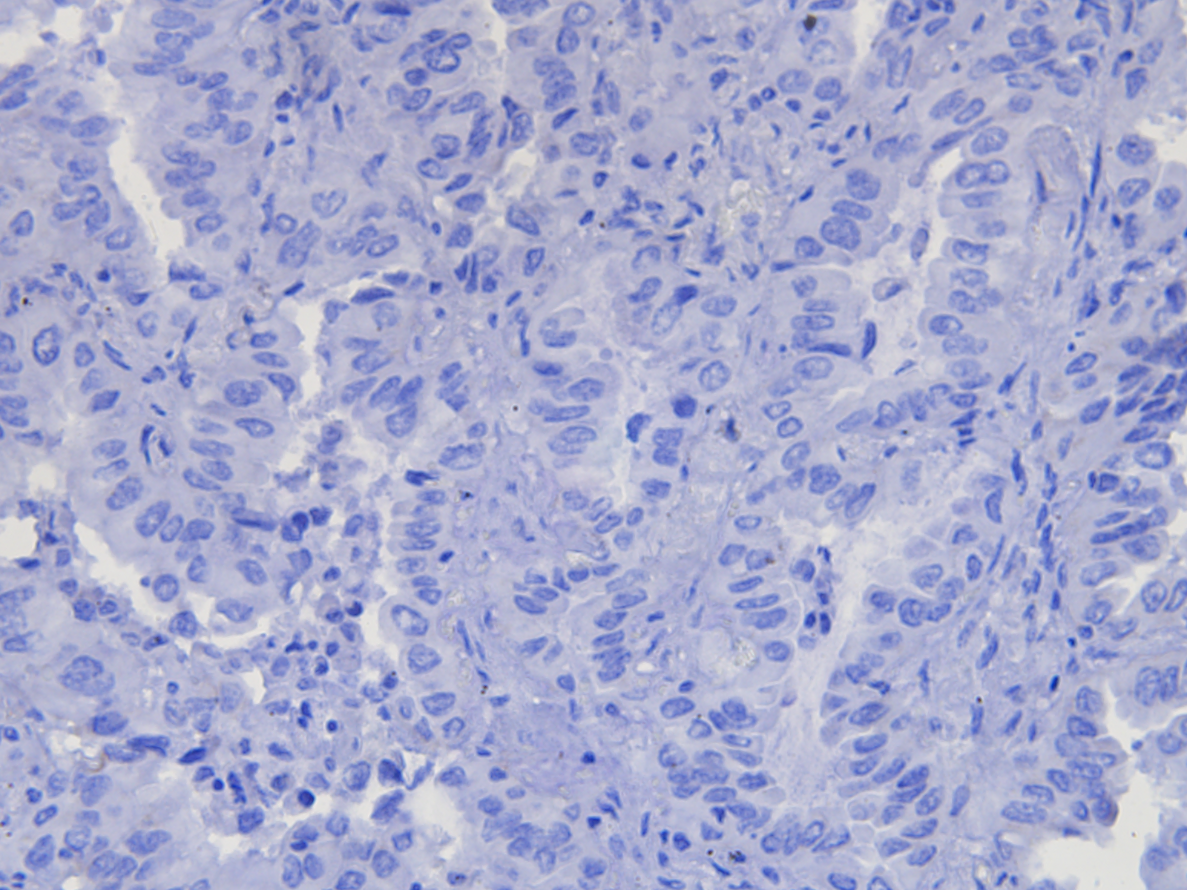

Supplement: S29 File — (ZIP) [file pone.0337223.s030.zip › 475114-400X-CA-N/475114-400X-CA (1).tif]

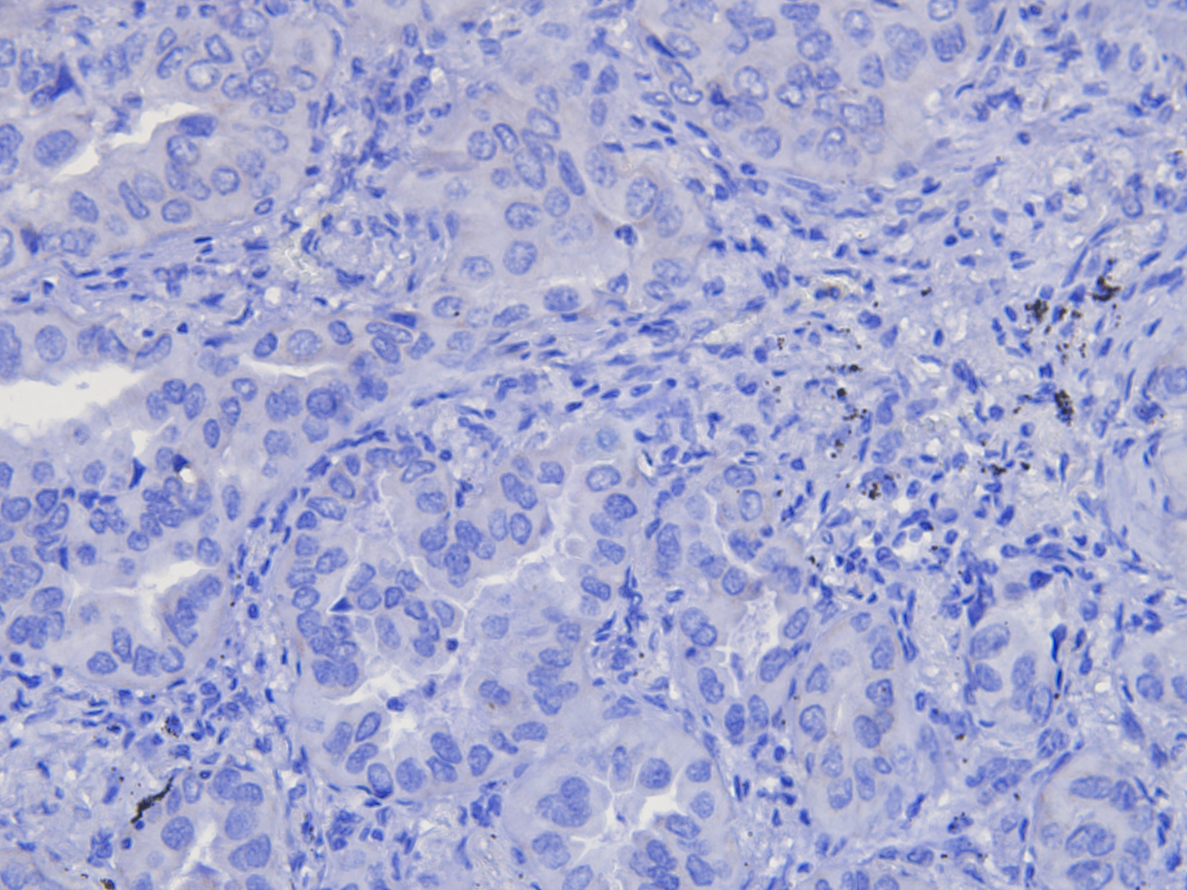

Supplement: S29 File — (ZIP) [file pone.0337223.s030.zip › 475114-400X-CA-N/475114-400X-CA (2).tif]

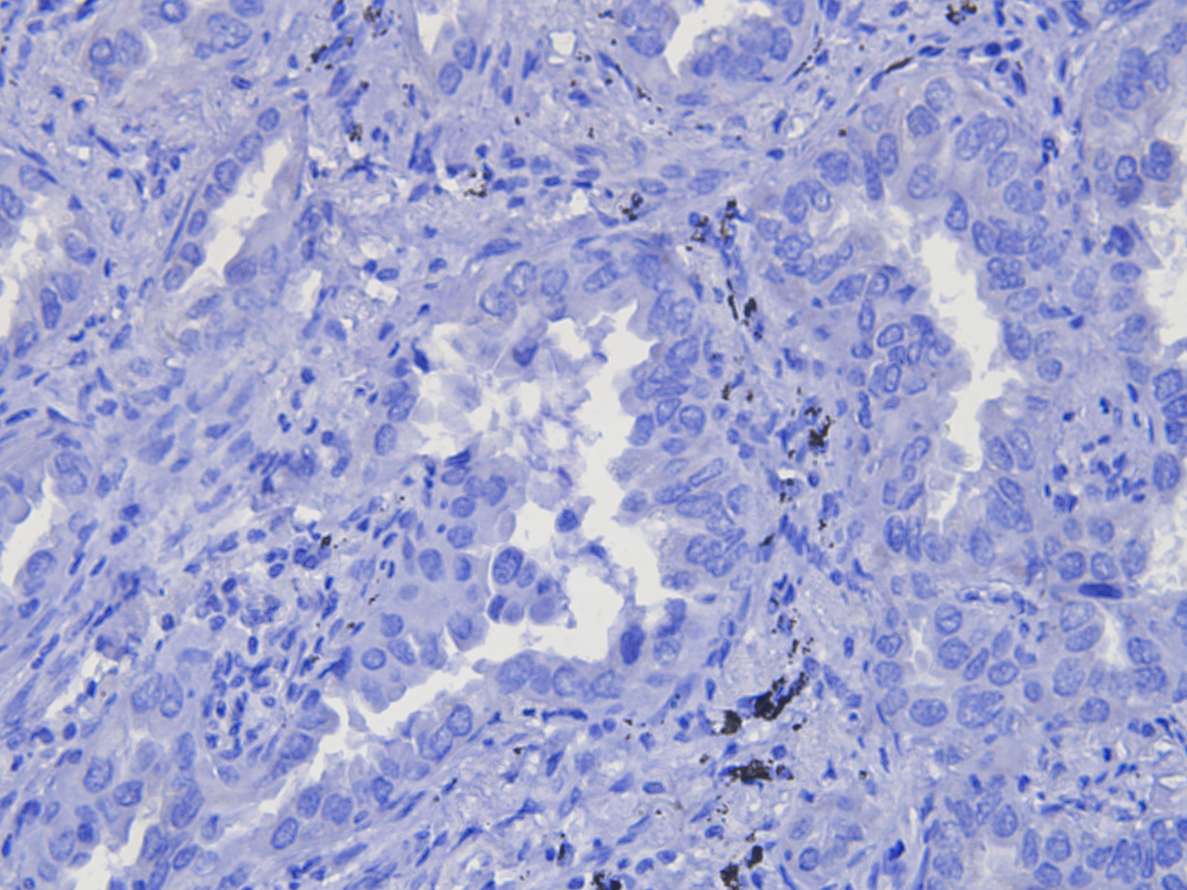

Supplement: S29 File — (ZIP) [file pone.0337223.s030.zip › 475114-400X-CA-N/475114-400X-CA (3).tif]

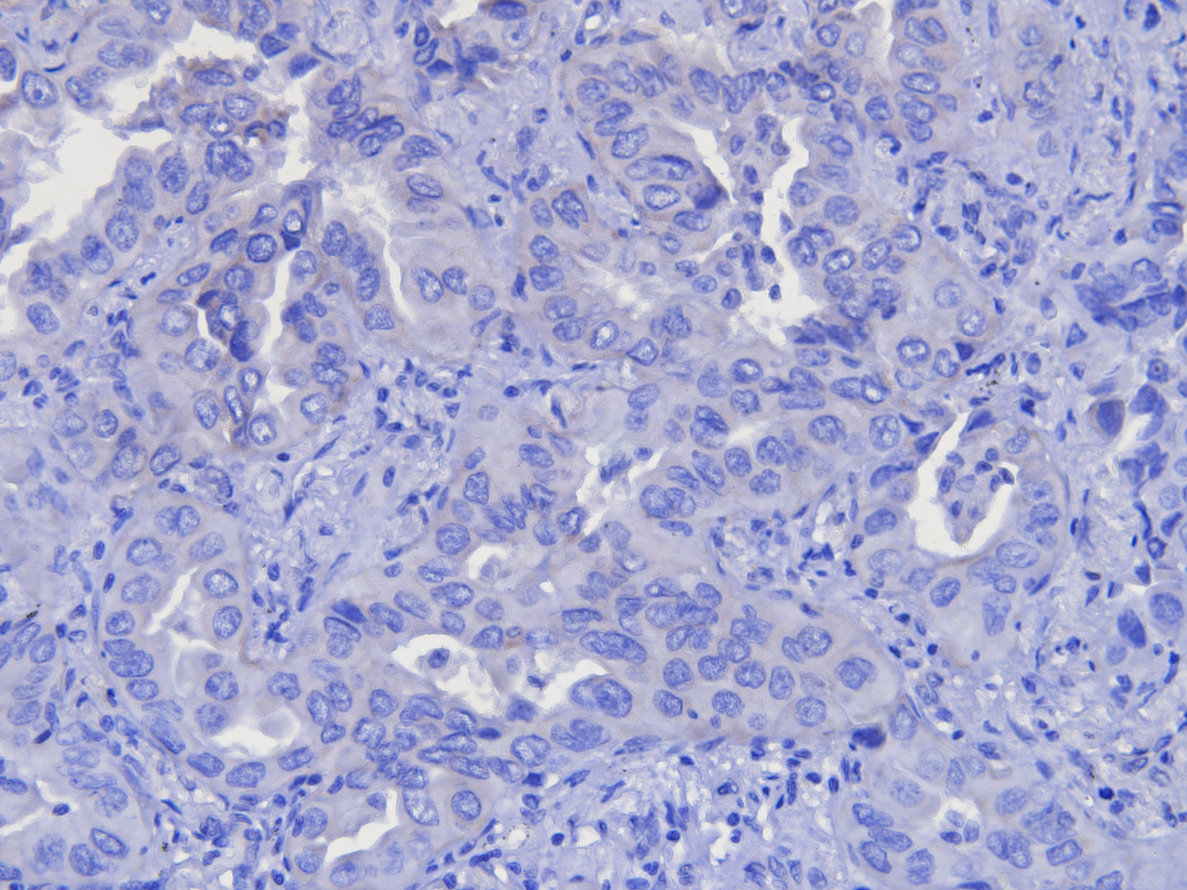

Supplement: S29 File — (ZIP) [file pone.0337223.s030.zip › 475114-400X-CA-N/475114-400X-CA (4).tif]

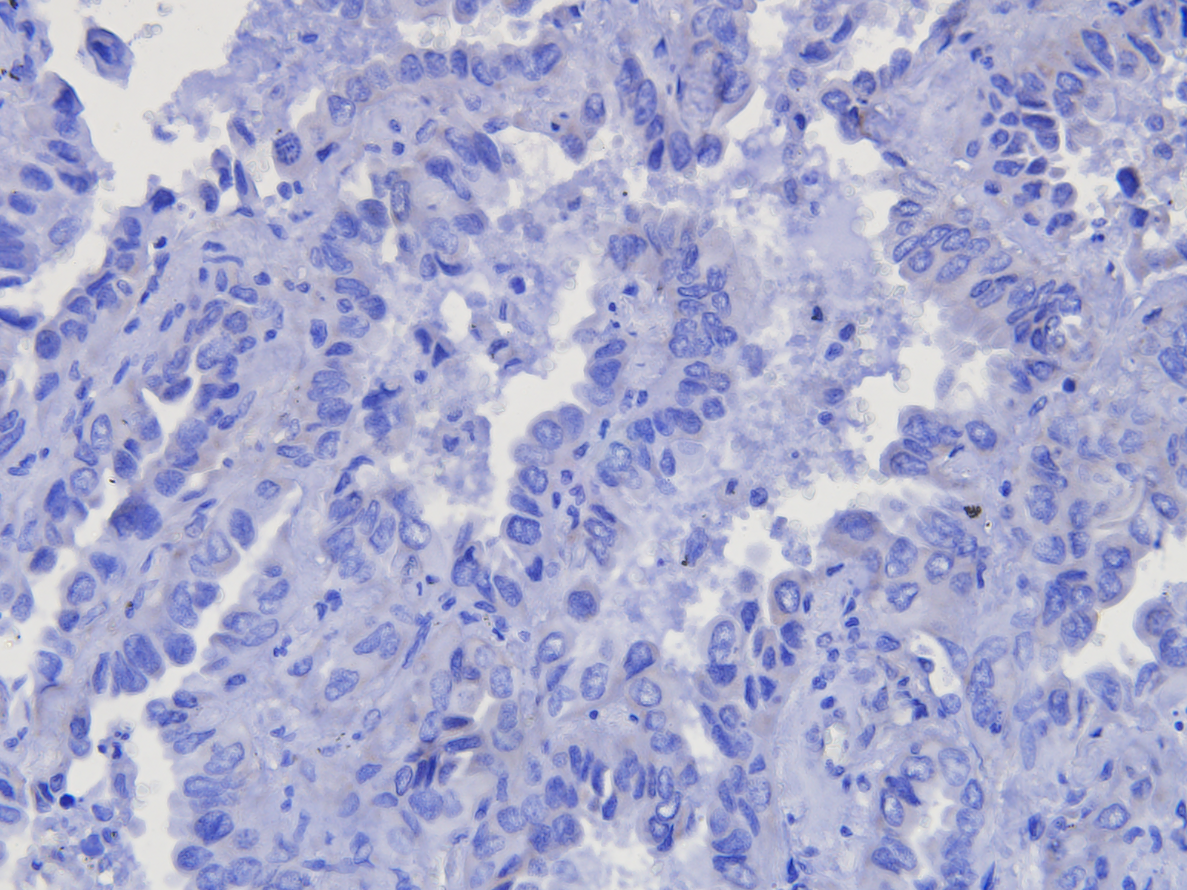

Supplement: S29 File — (ZIP) [file pone.0337223.s030.zip › 475114-400X-CA-N/475114-400X-CA (5).tif]
