# Supplementary figures and images for: Overexpression of PTPRCAP inhibits biological function of lung adenocarcinoma through apoptosis pathway (part 3 of 5)
Source: PLoS One. 2025 Dec 18;20(12):e0337223. doi: 10.1371/journal.pone.0337223 (PMC12716888; doi:10.1371/journal.pone.0337223)

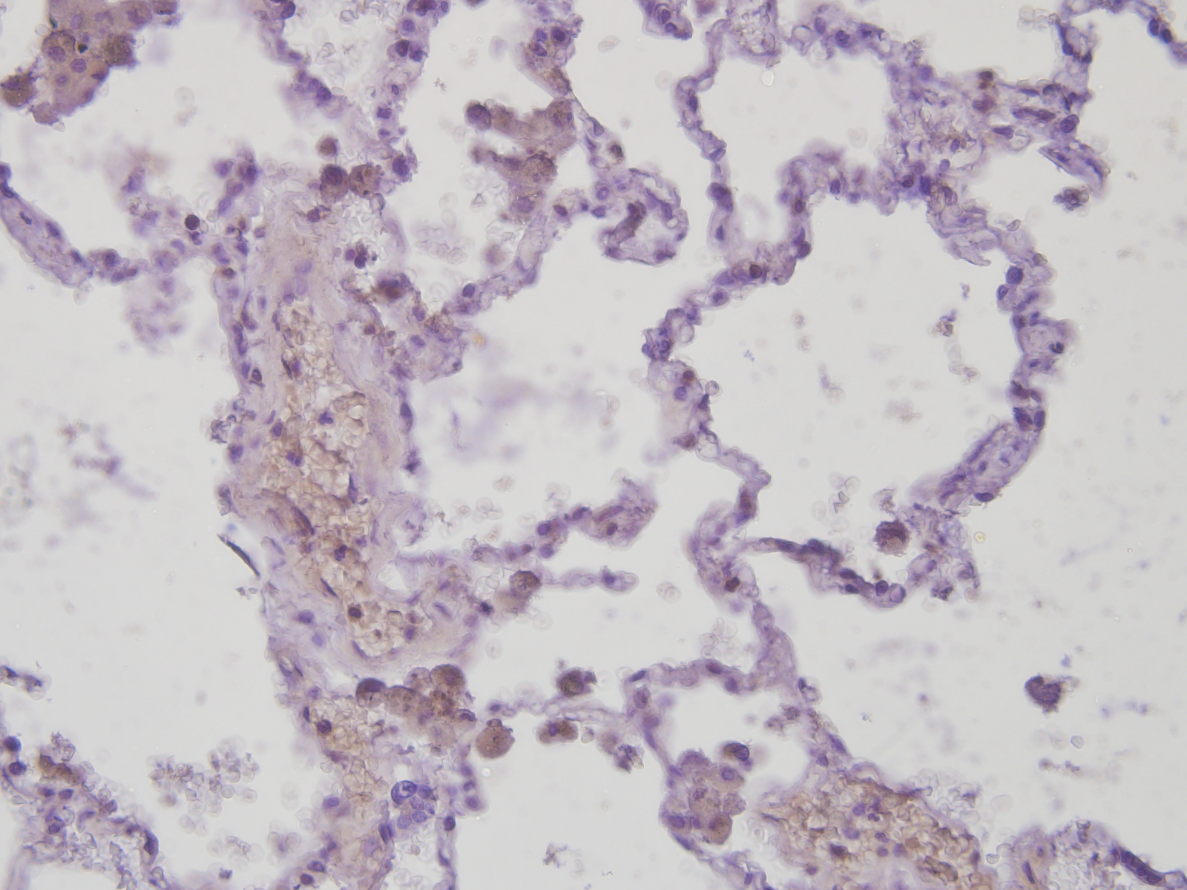

Supplement: S29 File — (ZIP) [file pone.0337223.s030.zip › 475114-400X-CA-N/475114-400X-N (1).tif]

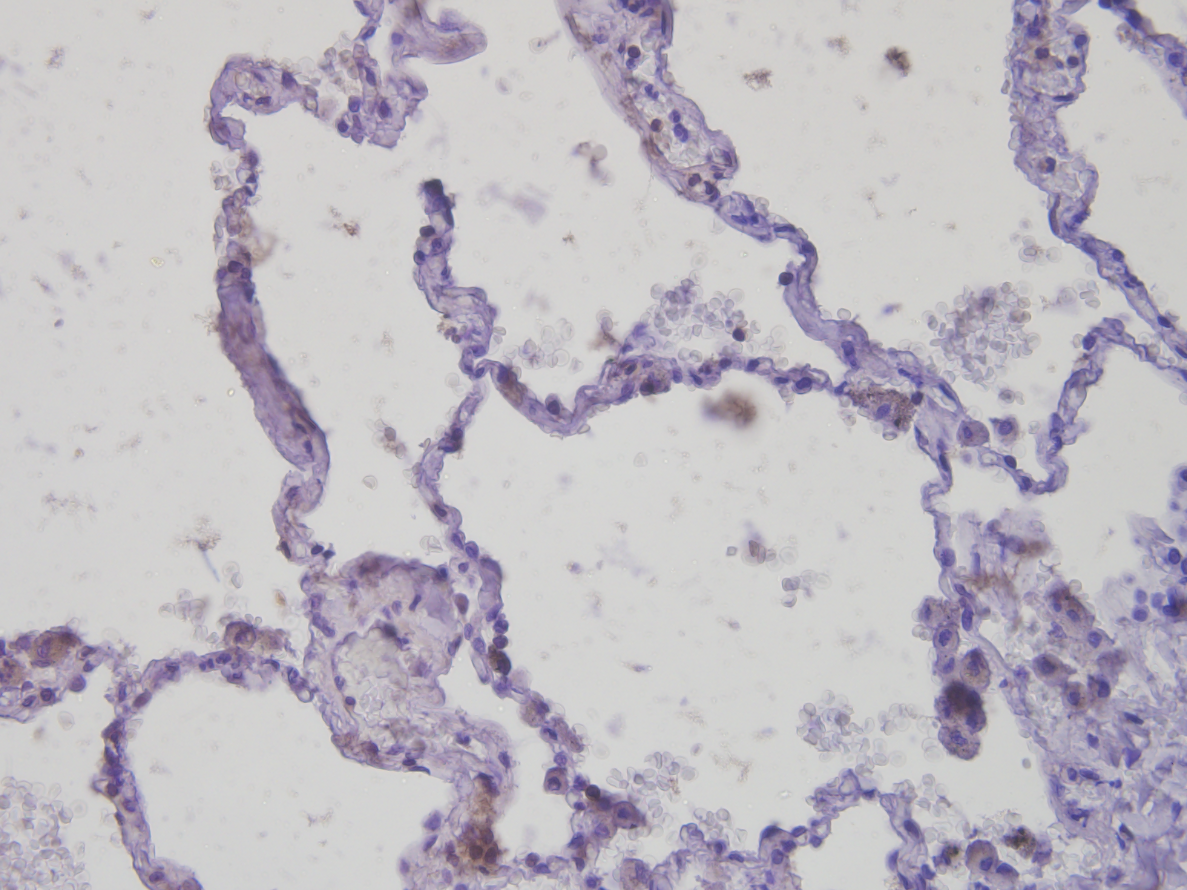

Supplement: S29 File — (ZIP) [file pone.0337223.s030.zip › 475114-400X-CA-N/475114-400X-N (2).tif]

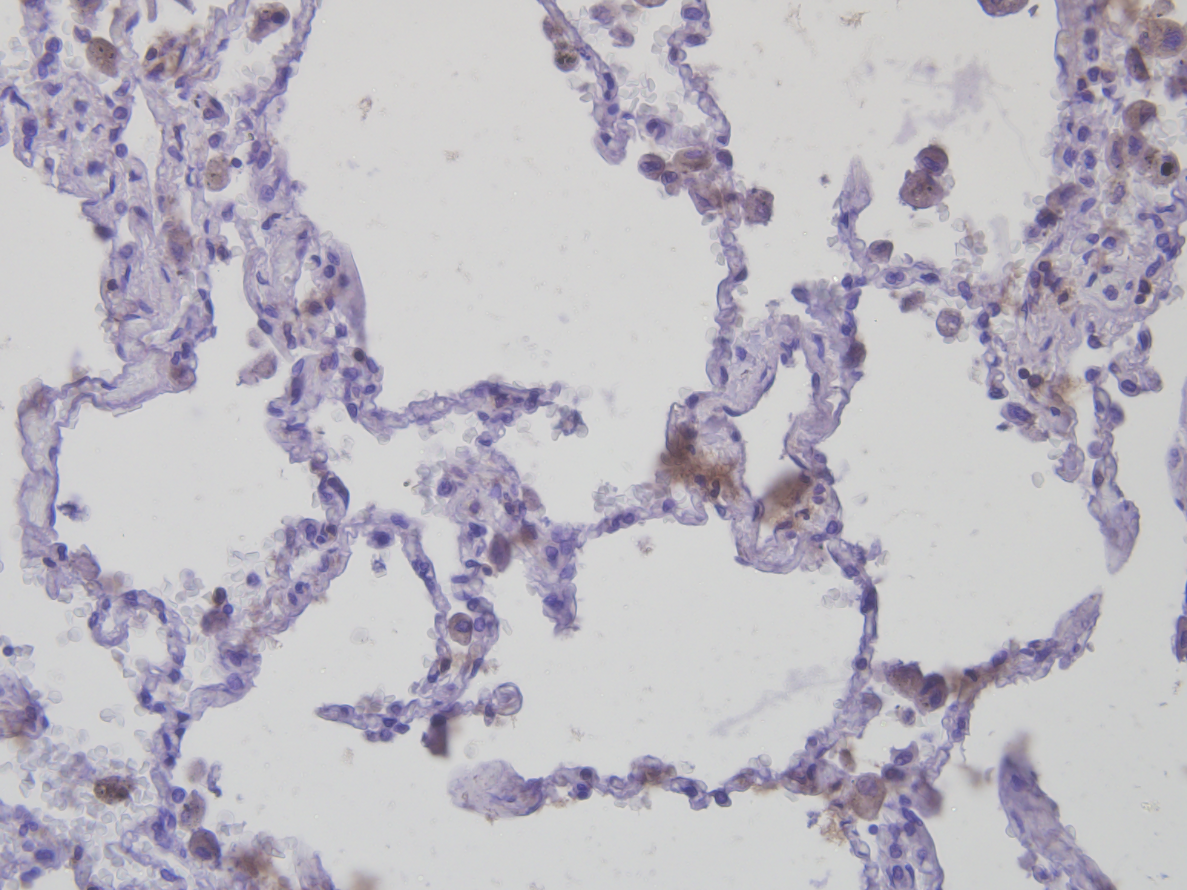

Supplement: S29 File — (ZIP) [file pone.0337223.s030.zip › 475114-400X-CA-N/475114-400X-N (3).tif]

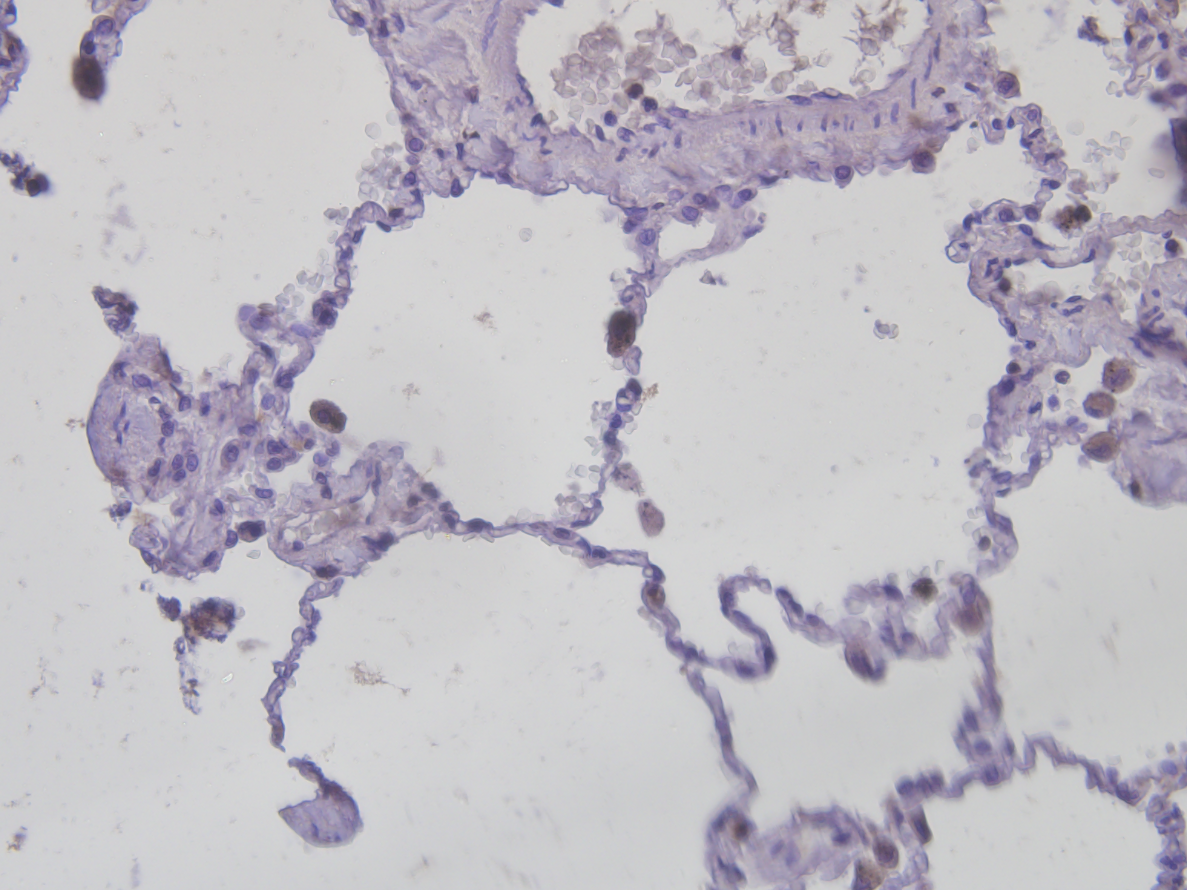

Supplement: S29 File — (ZIP) [file pone.0337223.s030.zip › 475114-400X-CA-N/475114-400X-N (4).tif]

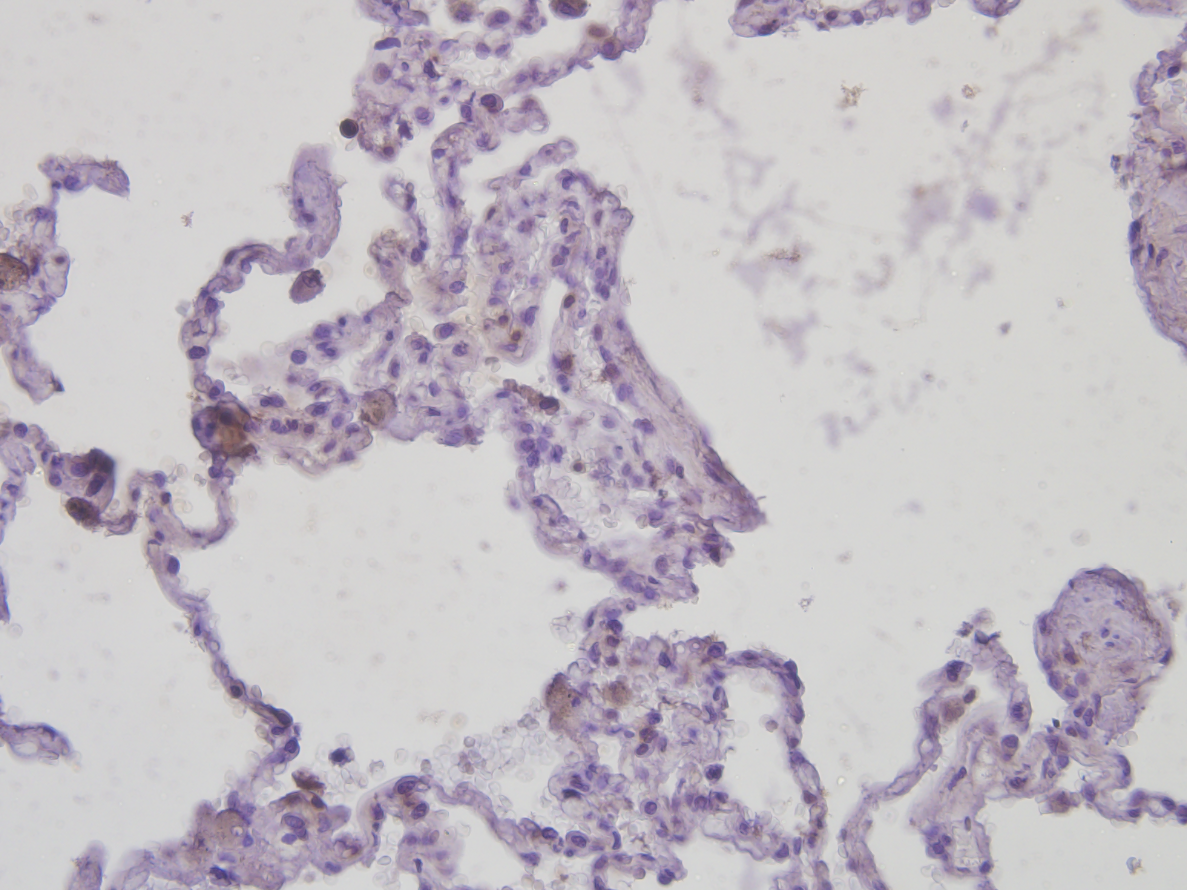

Supplement: S29 File — (ZIP) [file pone.0337223.s030.zip › 475114-400X-CA-N/475114-400X-N (5).tif]

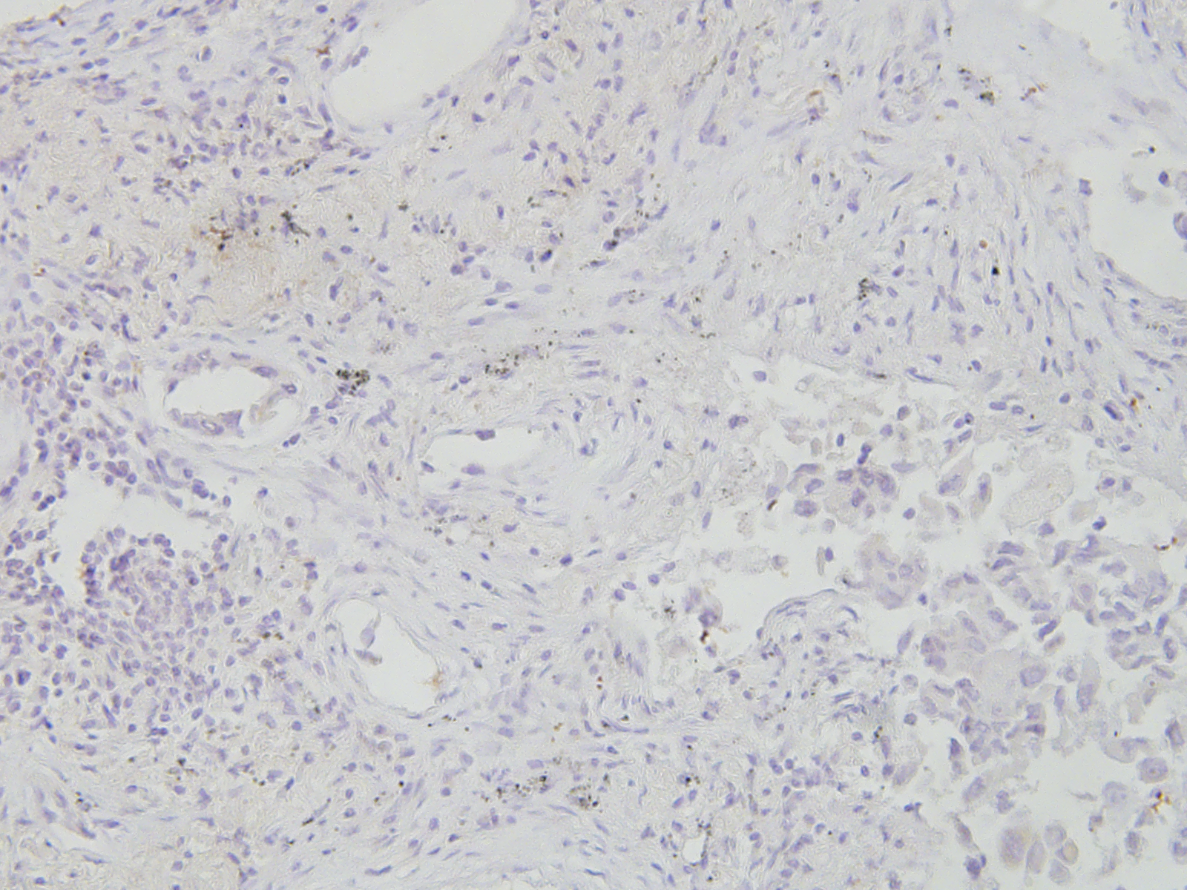

Supplement: S30 File — (ZIP) [file pone.0337223.s031.zip › 475772-400X-ca-N/475772-400X-CA (1).tif]

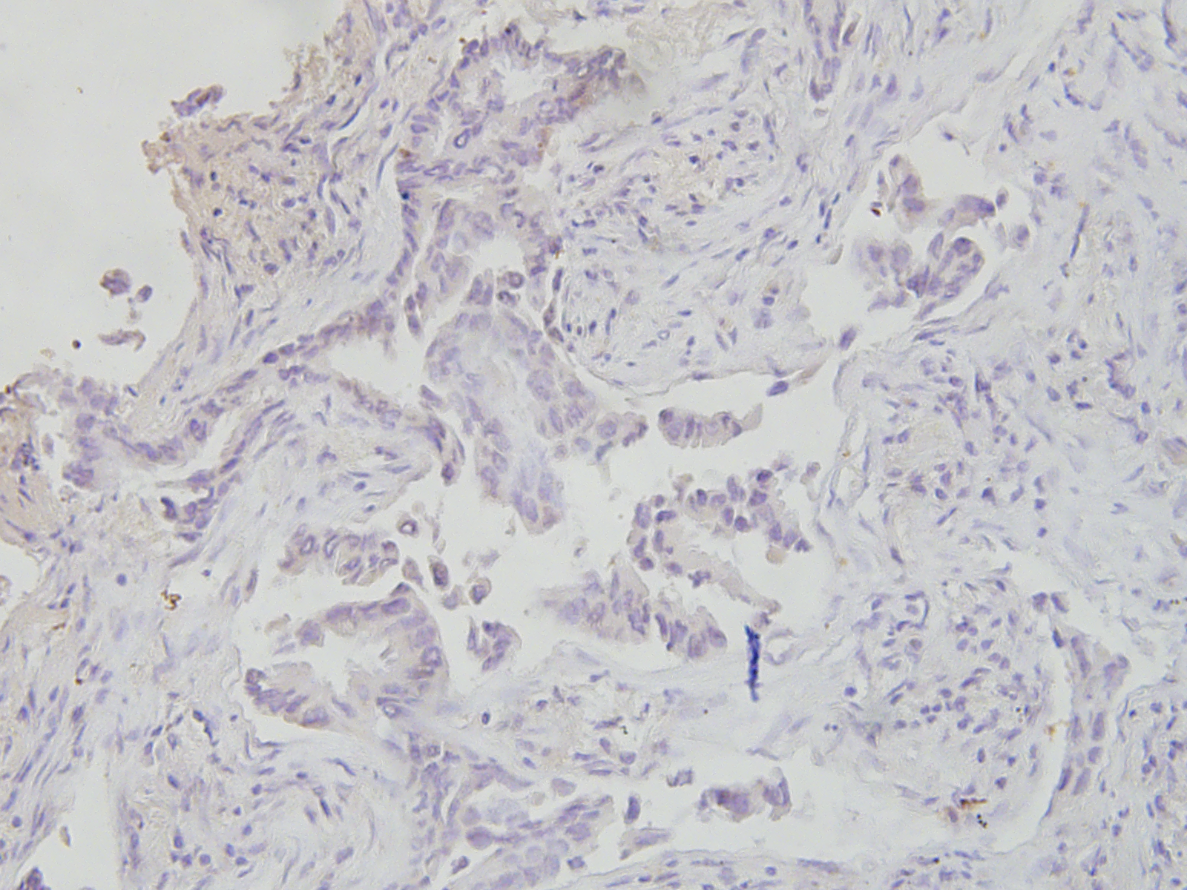

Supplement: S30 File — (ZIP) [file pone.0337223.s031.zip › 475772-400X-ca-N/475772-400X-CA (2).tif]

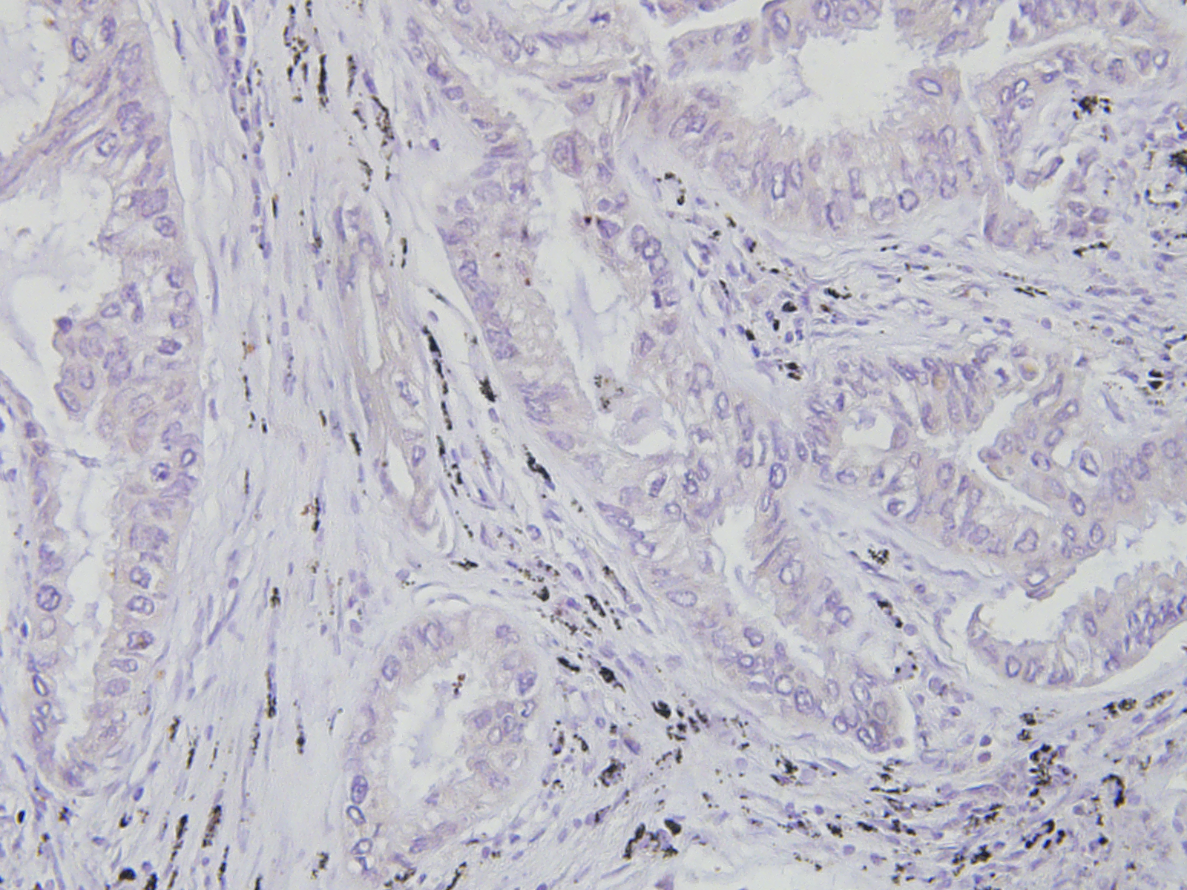

Supplement: S30 File — (ZIP) [file pone.0337223.s031.zip › 475772-400X-ca-N/475772-400X-CA (3).tif]

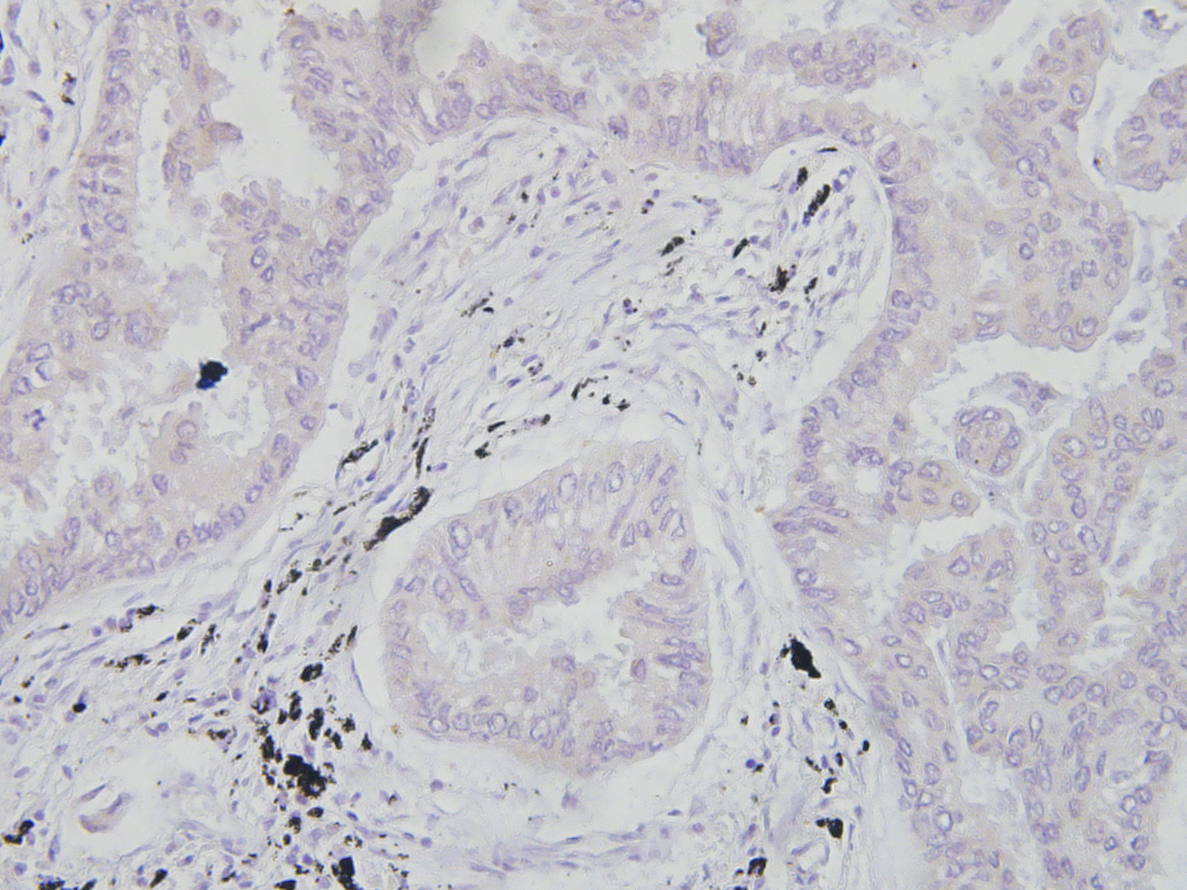

Supplement: S30 File — (ZIP) [file pone.0337223.s031.zip › 475772-400X-ca-N/475772-400X-CA (4).tif]

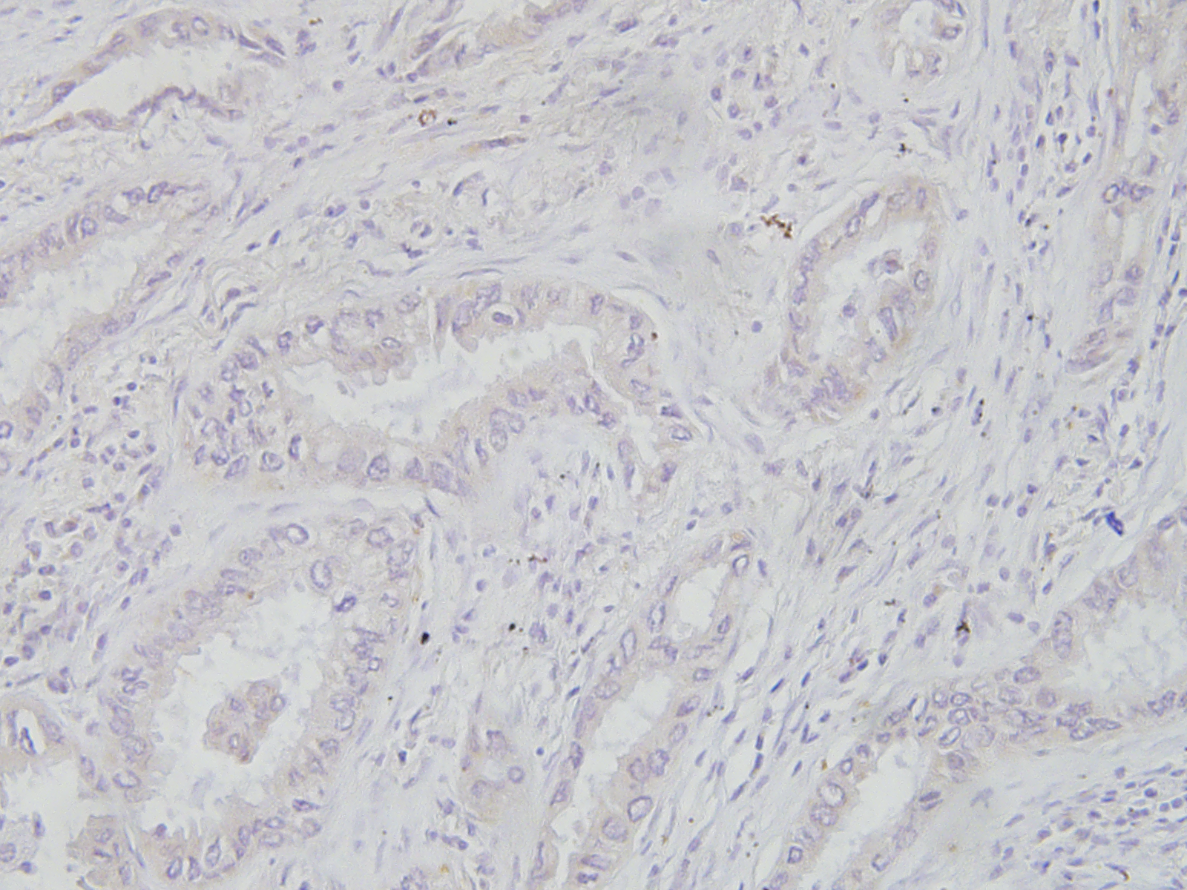

Supplement: S30 File — (ZIP) [file pone.0337223.s031.zip › 475772-400X-ca-N/475772-400X-CA (5).tif]

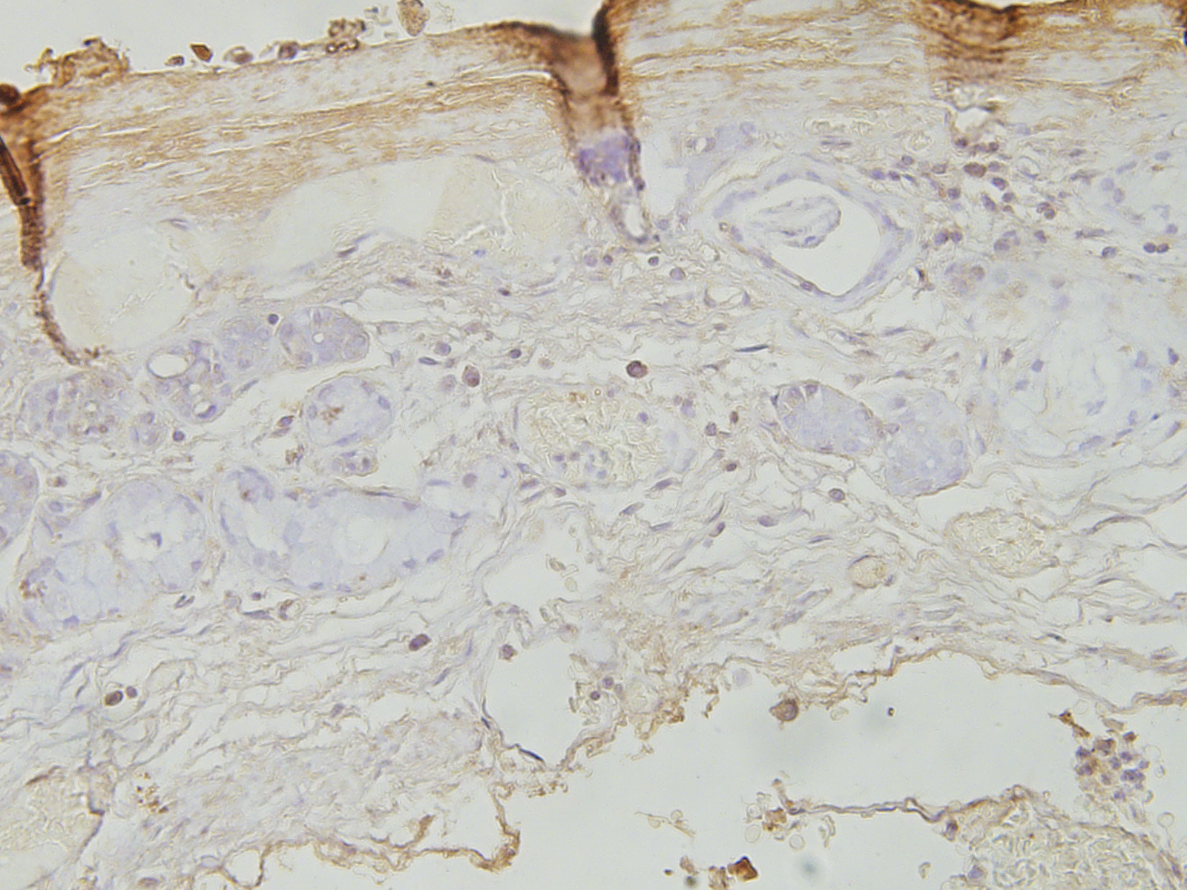

Supplement: S30 File — (ZIP) [file pone.0337223.s031.zip › 475772-400X-ca-N/475772-400X-N (1).tif]

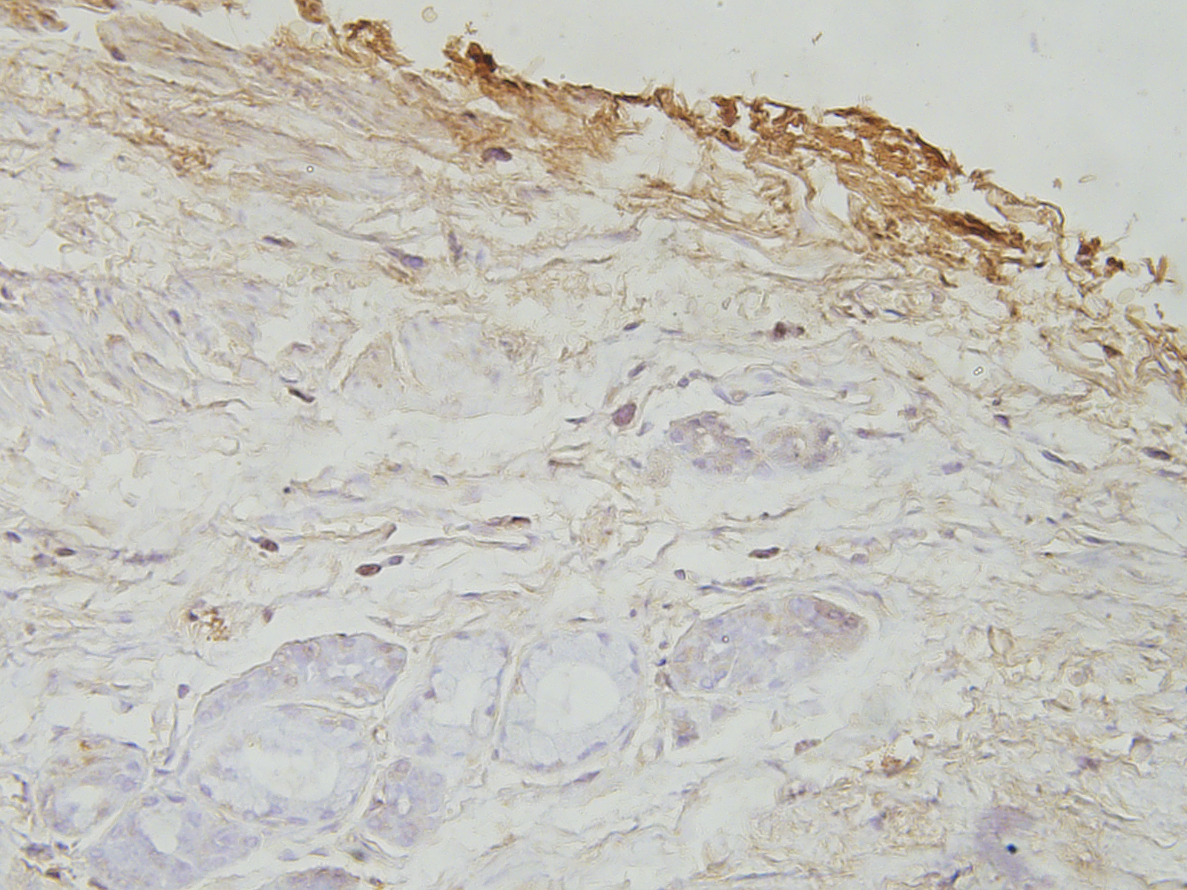

Supplement: S30 File — (ZIP) [file pone.0337223.s031.zip › 475772-400X-ca-N/475772-400X-n (2).tif]

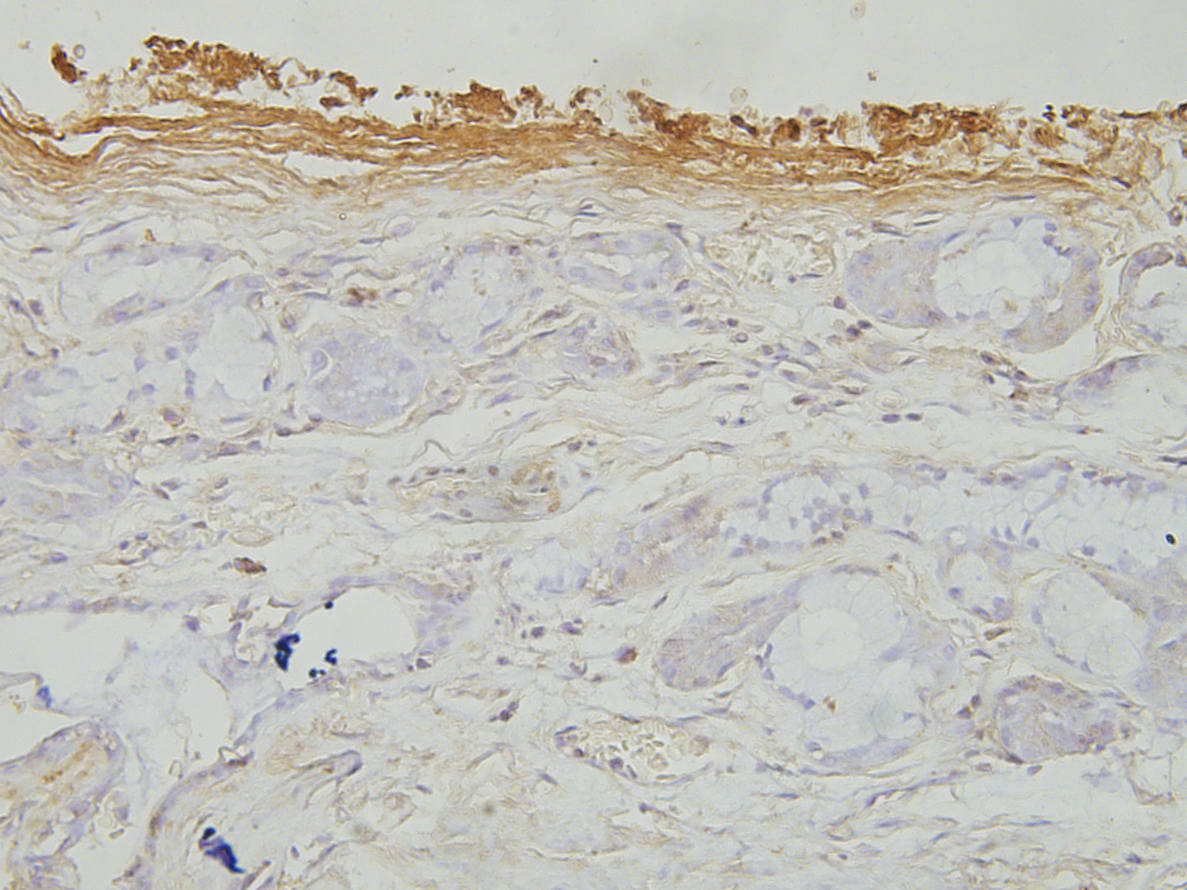

Supplement: S30 File — (ZIP) [file pone.0337223.s031.zip › 475772-400X-ca-N/475772-400X-n (3).tif]

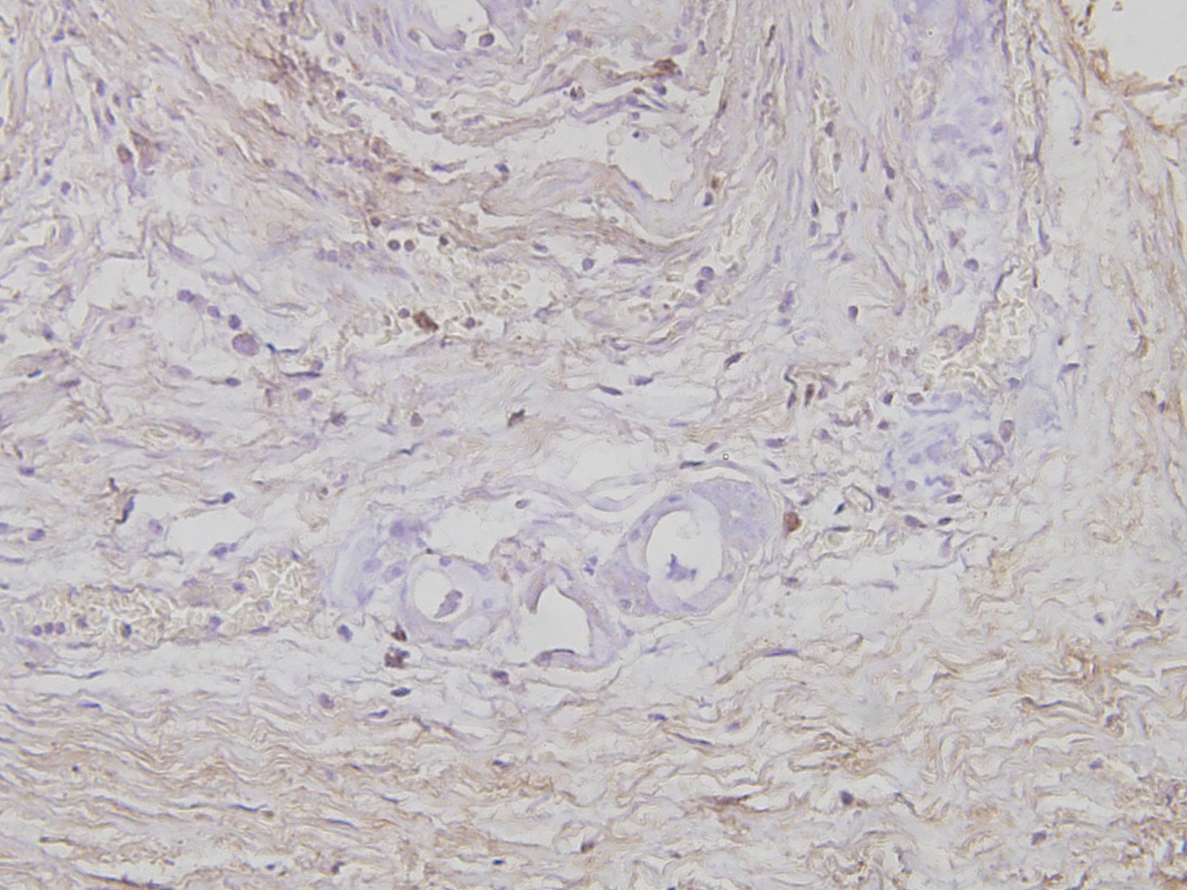

Supplement: S30 File — (ZIP) [file pone.0337223.s031.zip › 475772-400X-ca-N/475772-400X-n (4).tif]

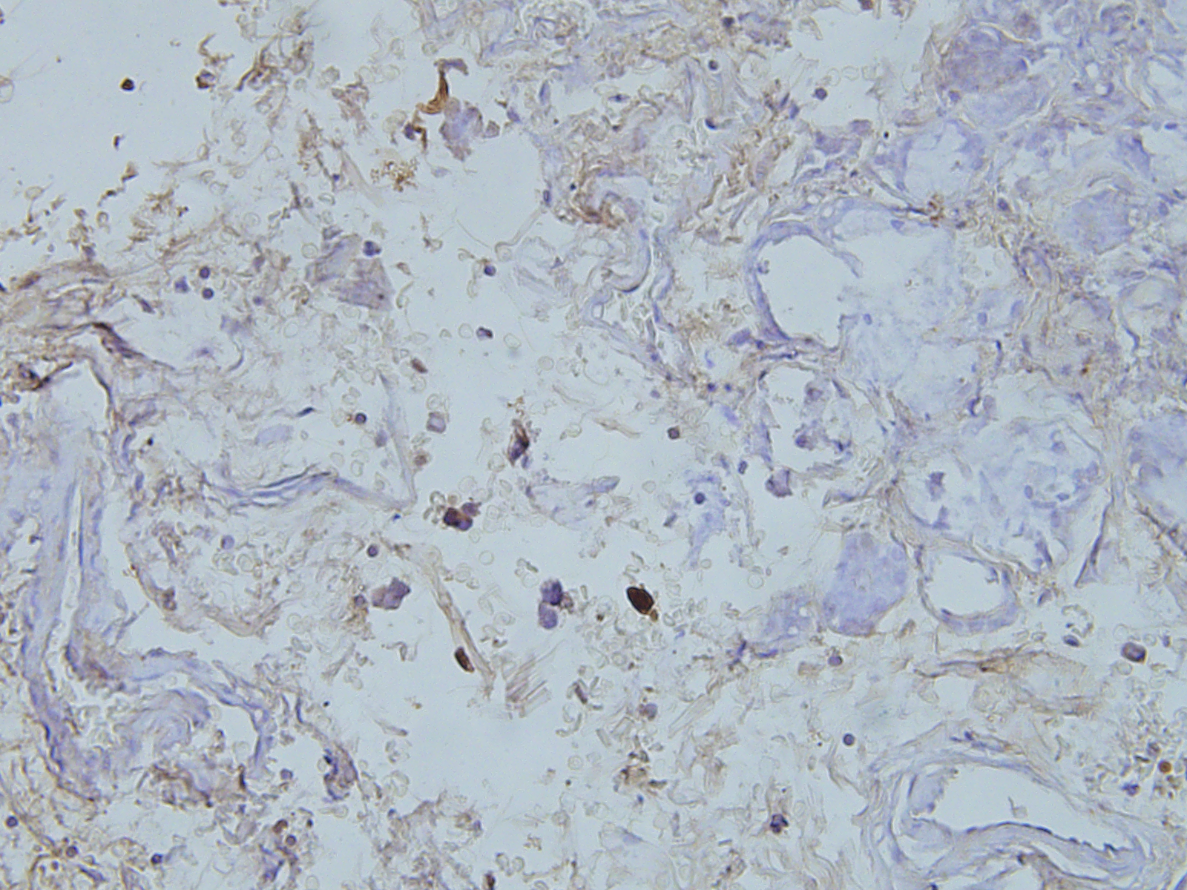

Supplement: S30 File — (ZIP) [file pone.0337223.s031.zip › 475772-400X-ca-N/475772-400X-n (5).tif]

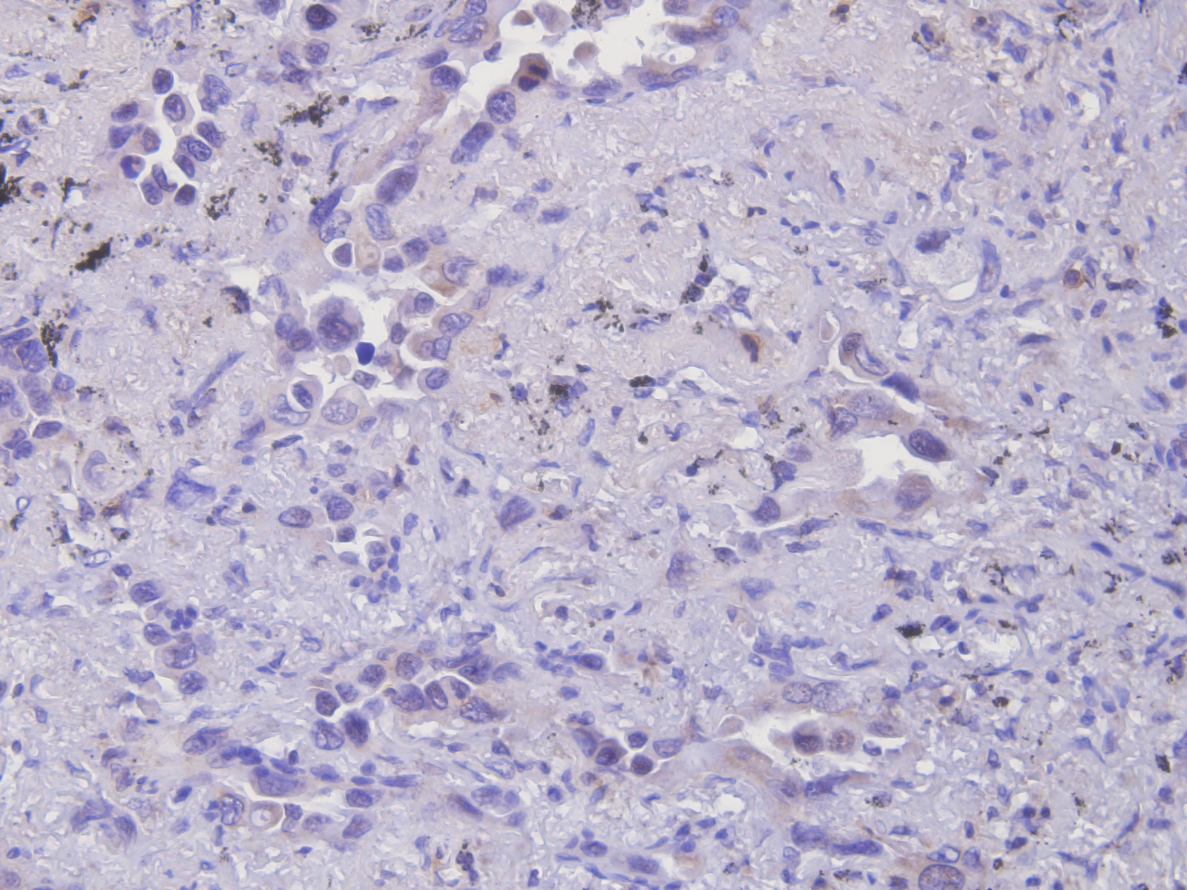

Supplement: S31 File — (ZIP) [file pone.0337223.s032.zip › 478627-400X-CA-N/478627-400X-CA (1).tif]

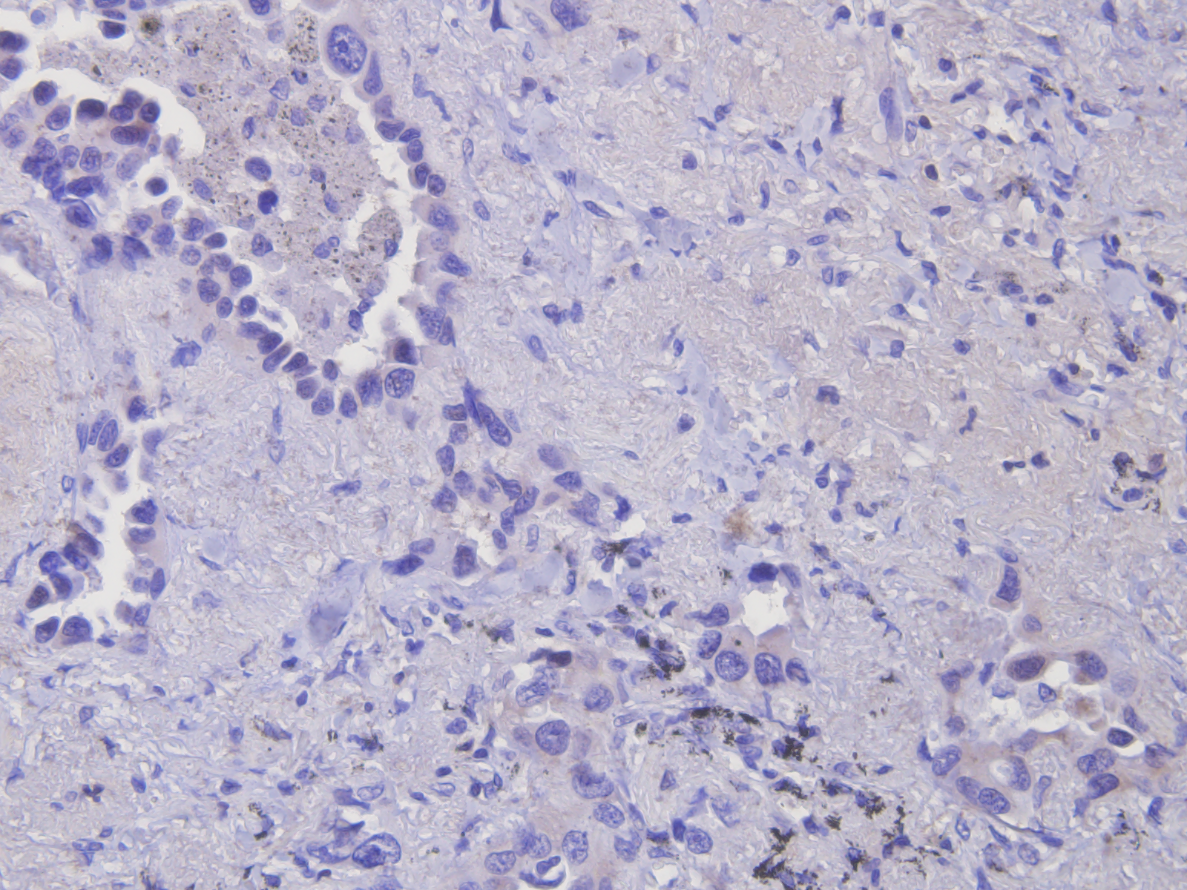

Supplement: S31 File — (ZIP) [file pone.0337223.s032.zip › 478627-400X-CA-N/478627-400X-CA (2).tif]

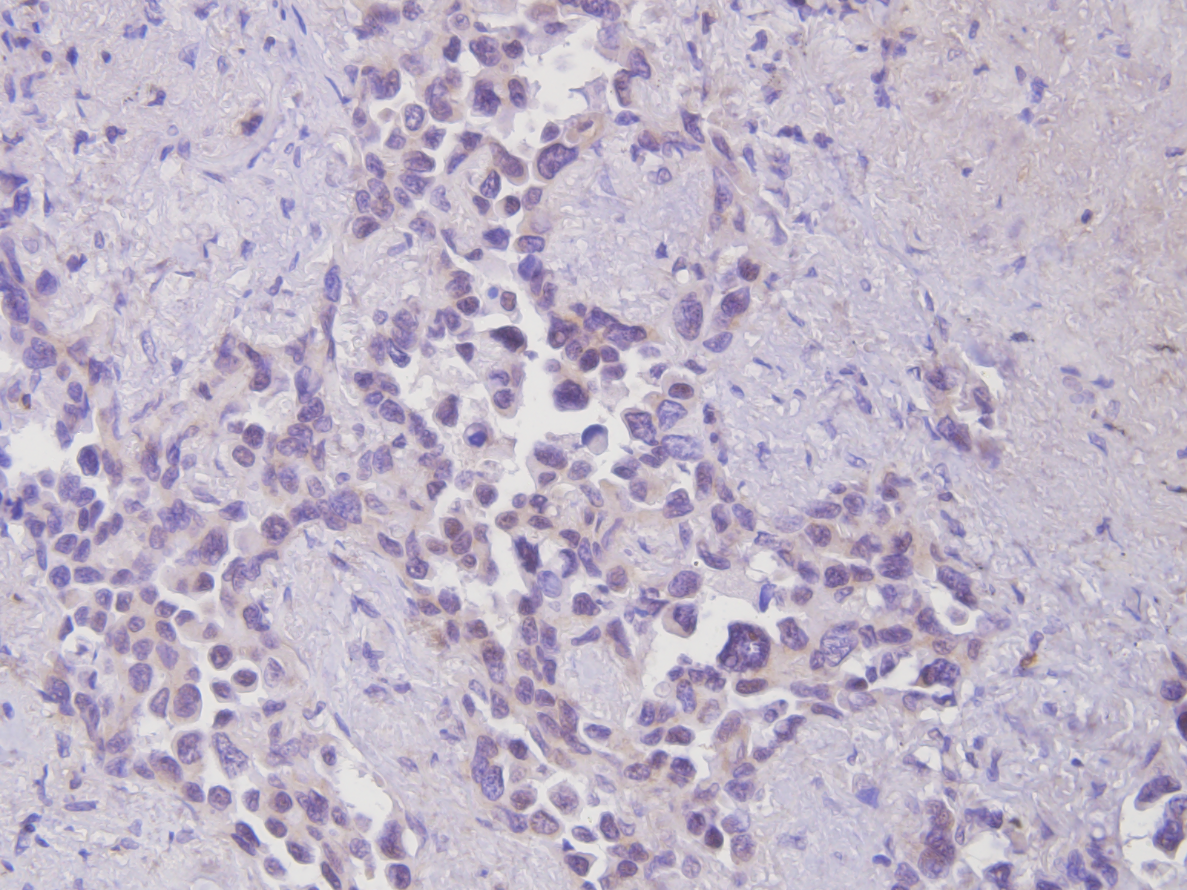

Supplement: S31 File — (ZIP) [file pone.0337223.s032.zip › 478627-400X-CA-N/478627-400X-CA (3).tif]

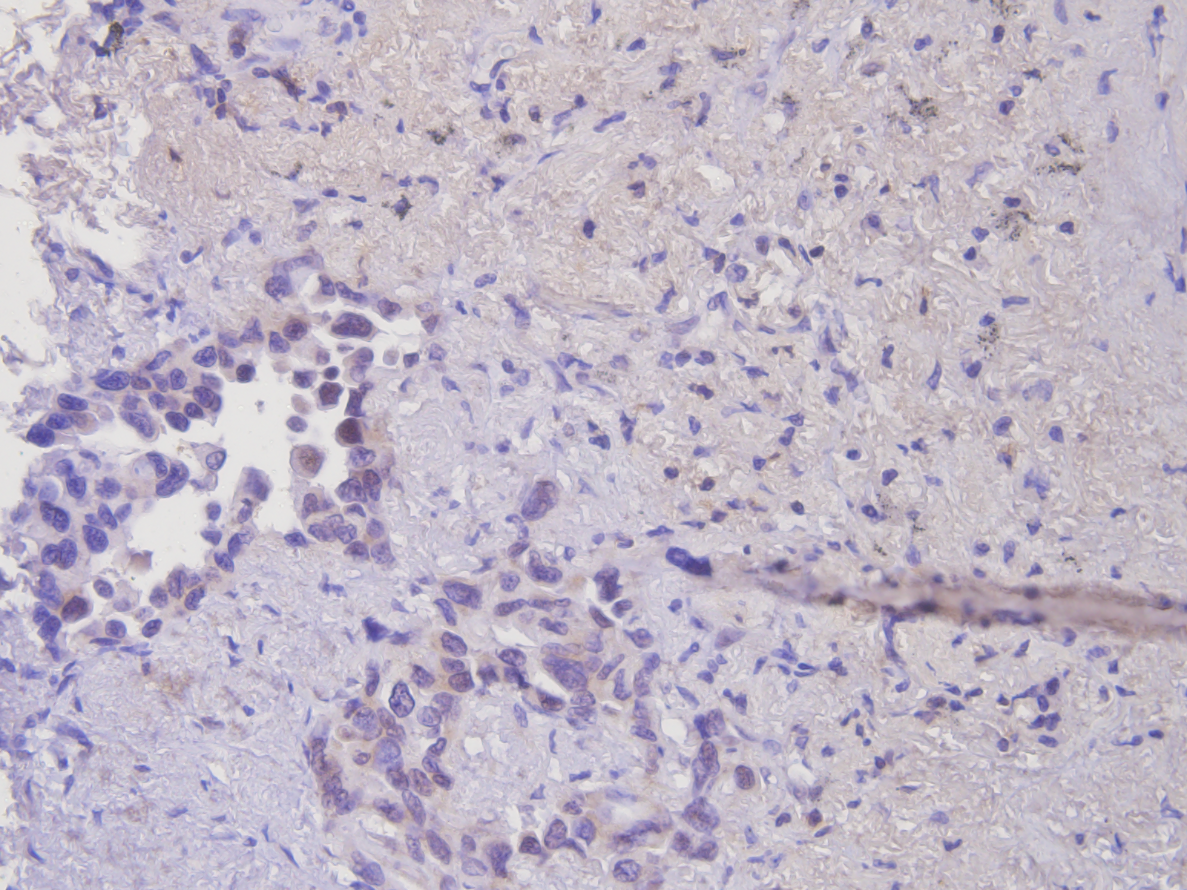

Supplement: S31 File — (ZIP) [file pone.0337223.s032.zip › 478627-400X-CA-N/478627-400X-CA (4).tif]

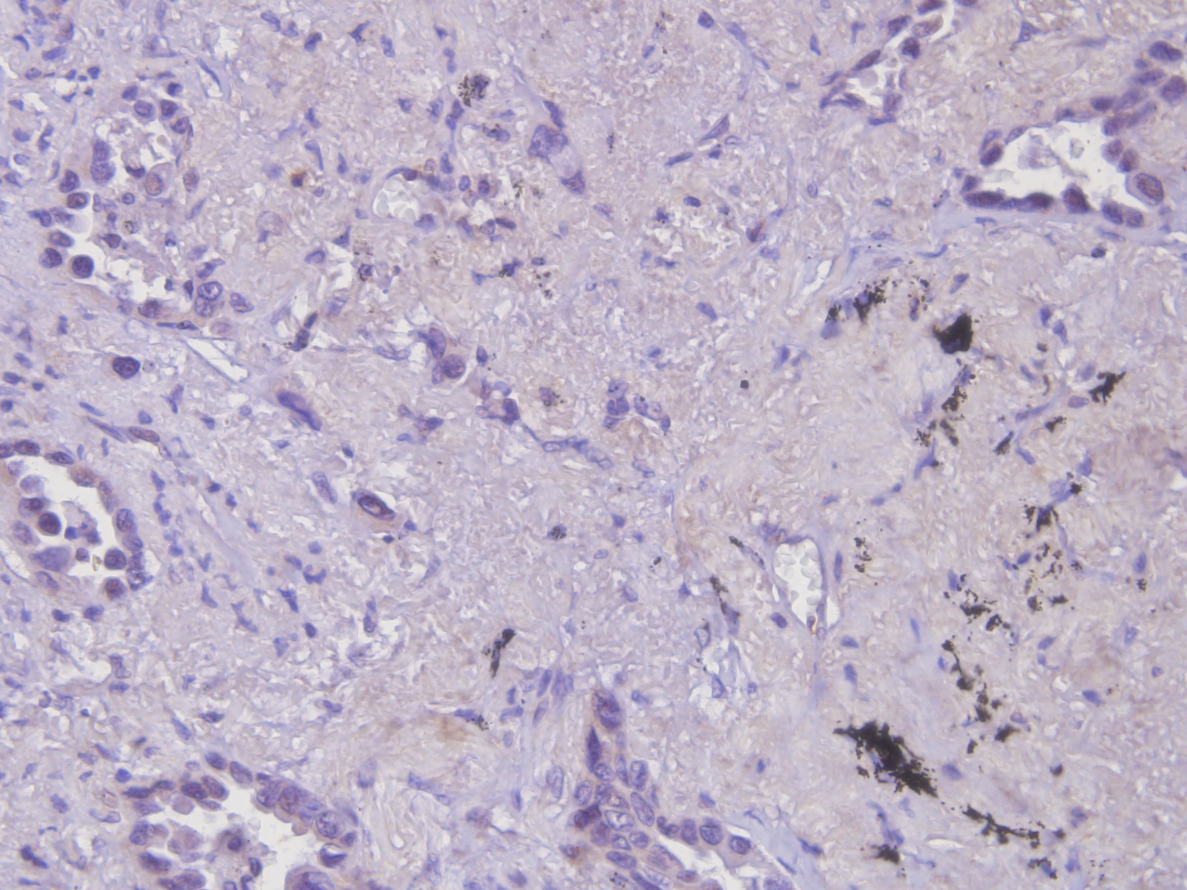

Supplement: S31 File — (ZIP) [file pone.0337223.s032.zip › 478627-400X-CA-N/478627-400X-CA (5).tif]

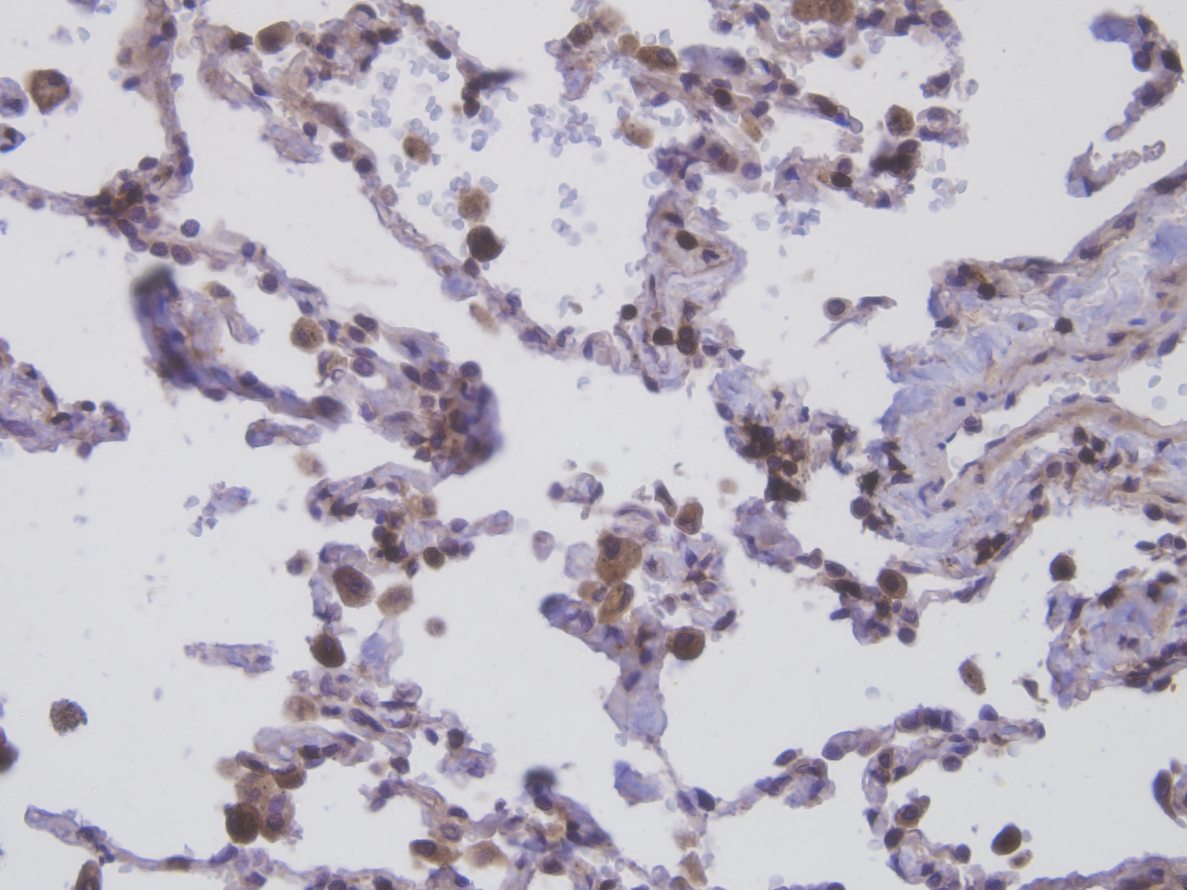

Supplement: S31 File — (ZIP) [file pone.0337223.s032.zip › 478627-400X-CA-N/478627-400X-N (1).tif]

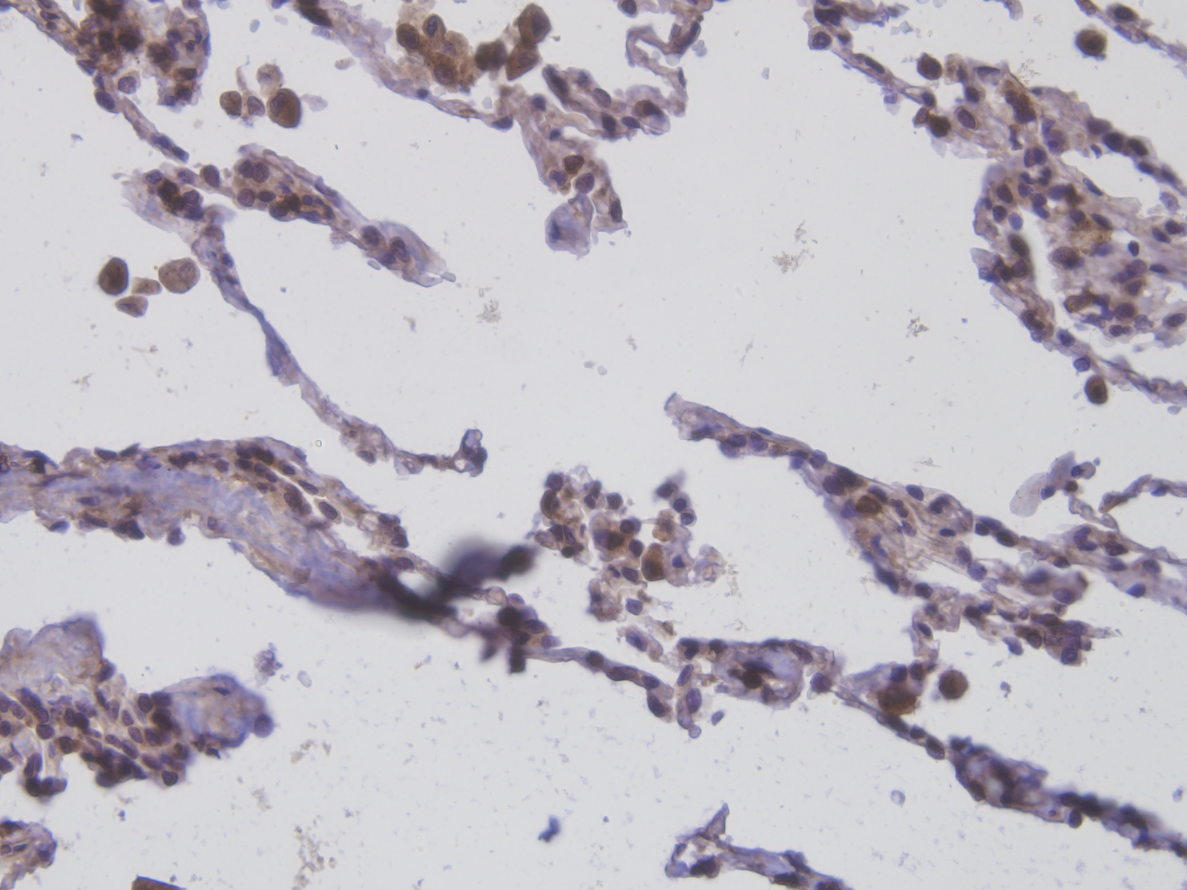

Supplement: S31 File — (ZIP) [file pone.0337223.s032.zip › 478627-400X-CA-N/478627-400X-N (2).tif]

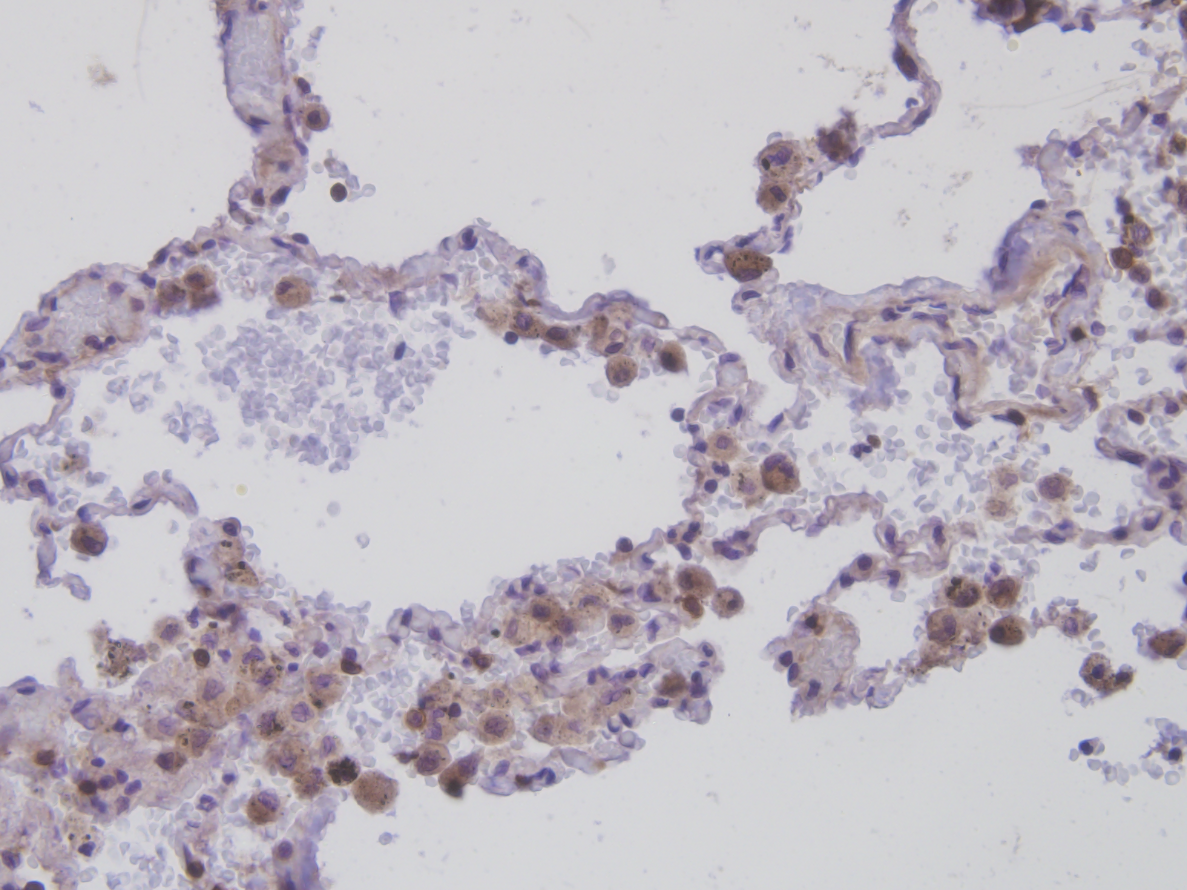

Supplement: S31 File — (ZIP) [file pone.0337223.s032.zip › 478627-400X-CA-N/478627-400X-N (3).tif]

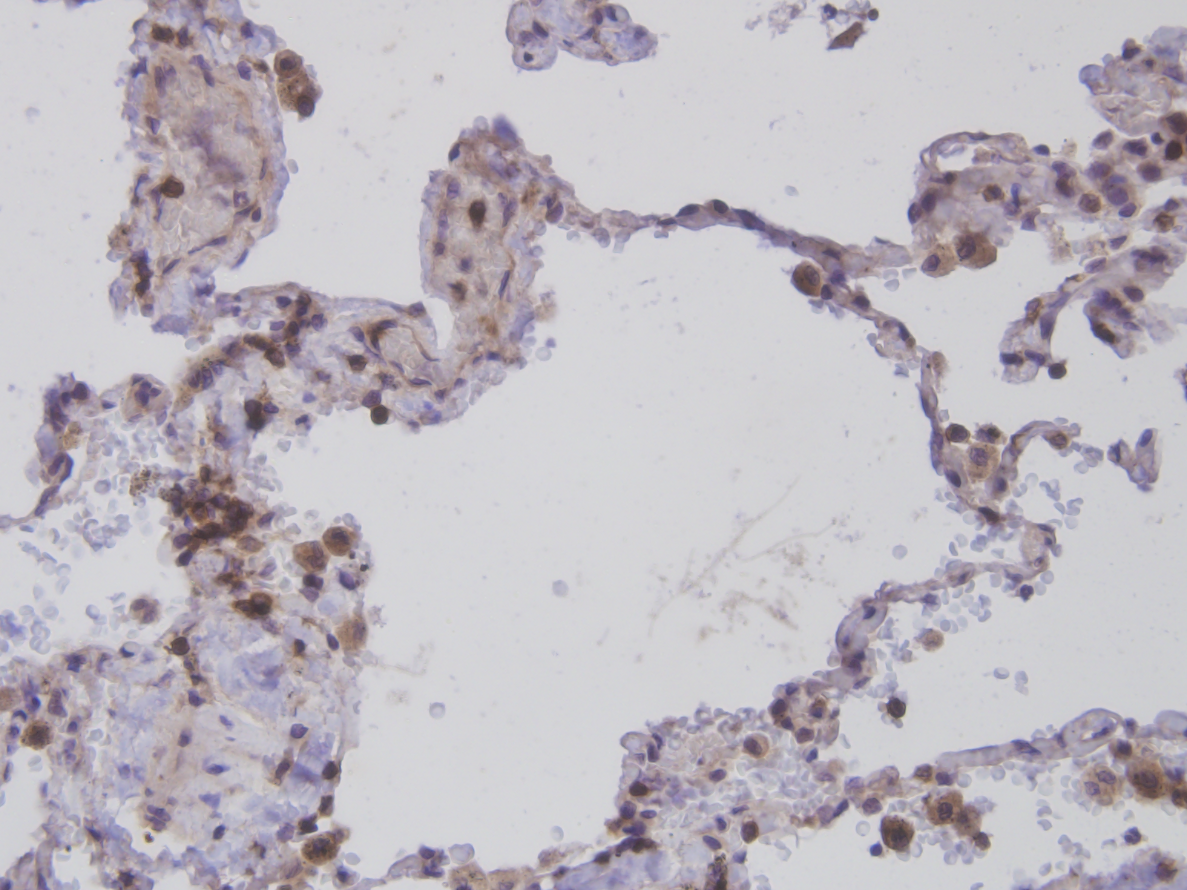

Supplement: S31 File — (ZIP) [file pone.0337223.s032.zip › 478627-400X-CA-N/478627-400X-N (4).tif]

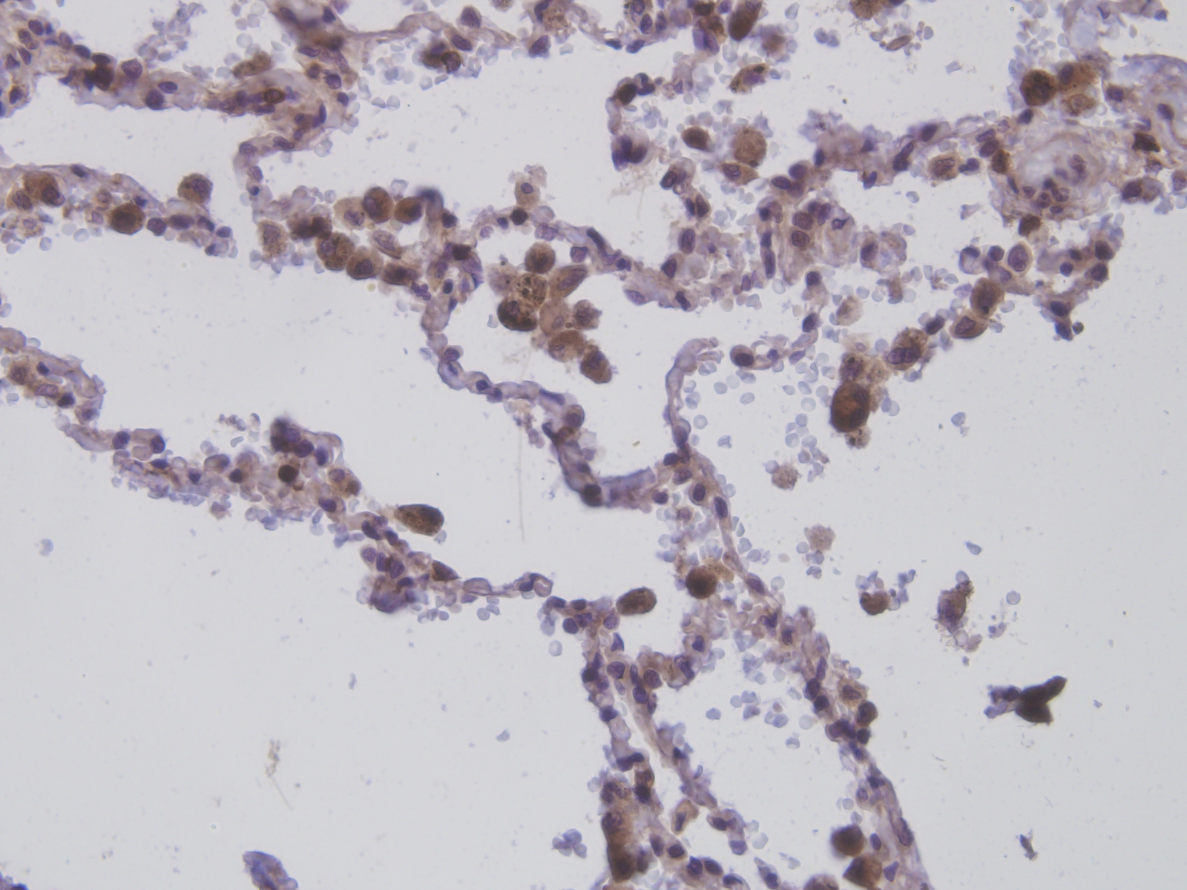

Supplement: S31 File — (ZIP) [file pone.0337223.s032.zip › 478627-400X-CA-N/478627-400X-N (5).tif]

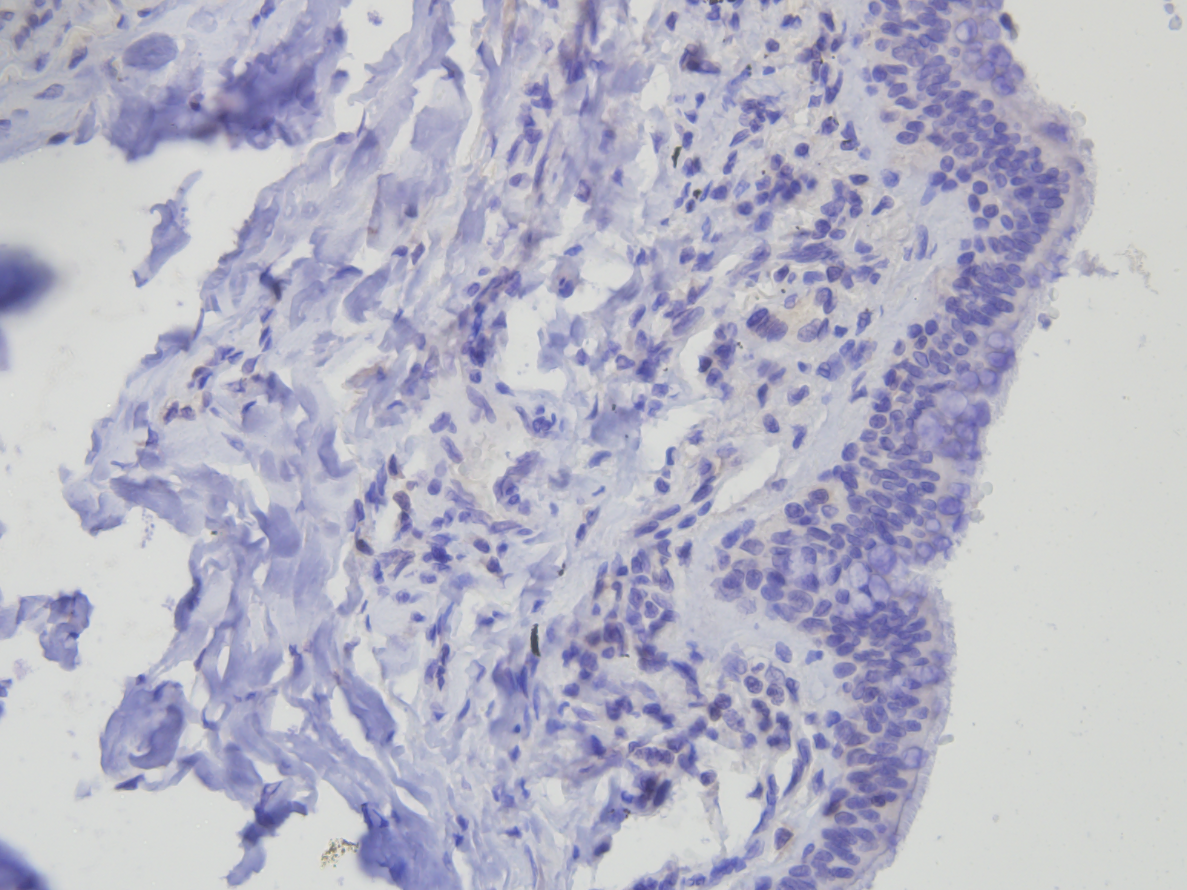

Supplement: S32 File — (ZIP) [file pone.0337223.s033.zip › 481080-400X-CA-N/481080-400X-CA (1).tif]

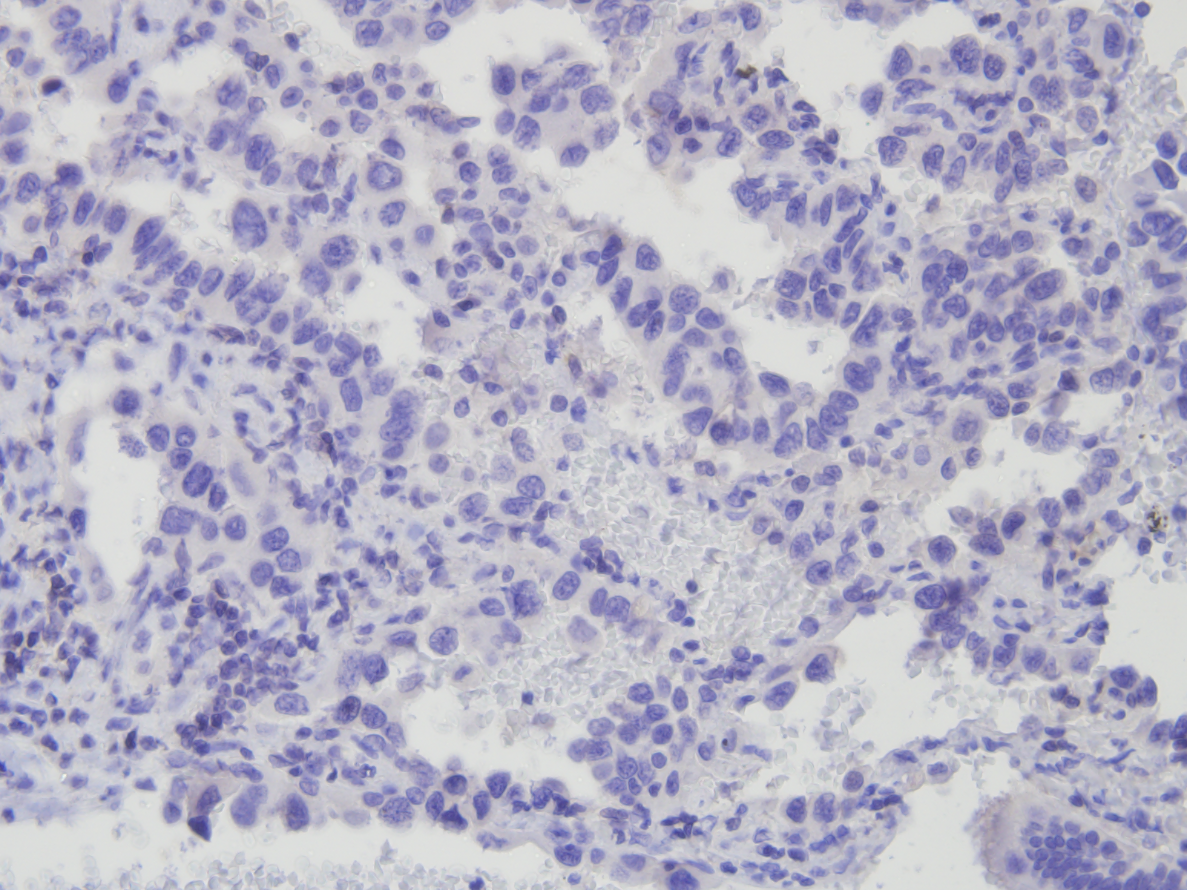

Supplement: S32 File — (ZIP) [file pone.0337223.s033.zip › 481080-400X-CA-N/481080-400X-CA (2).tif]

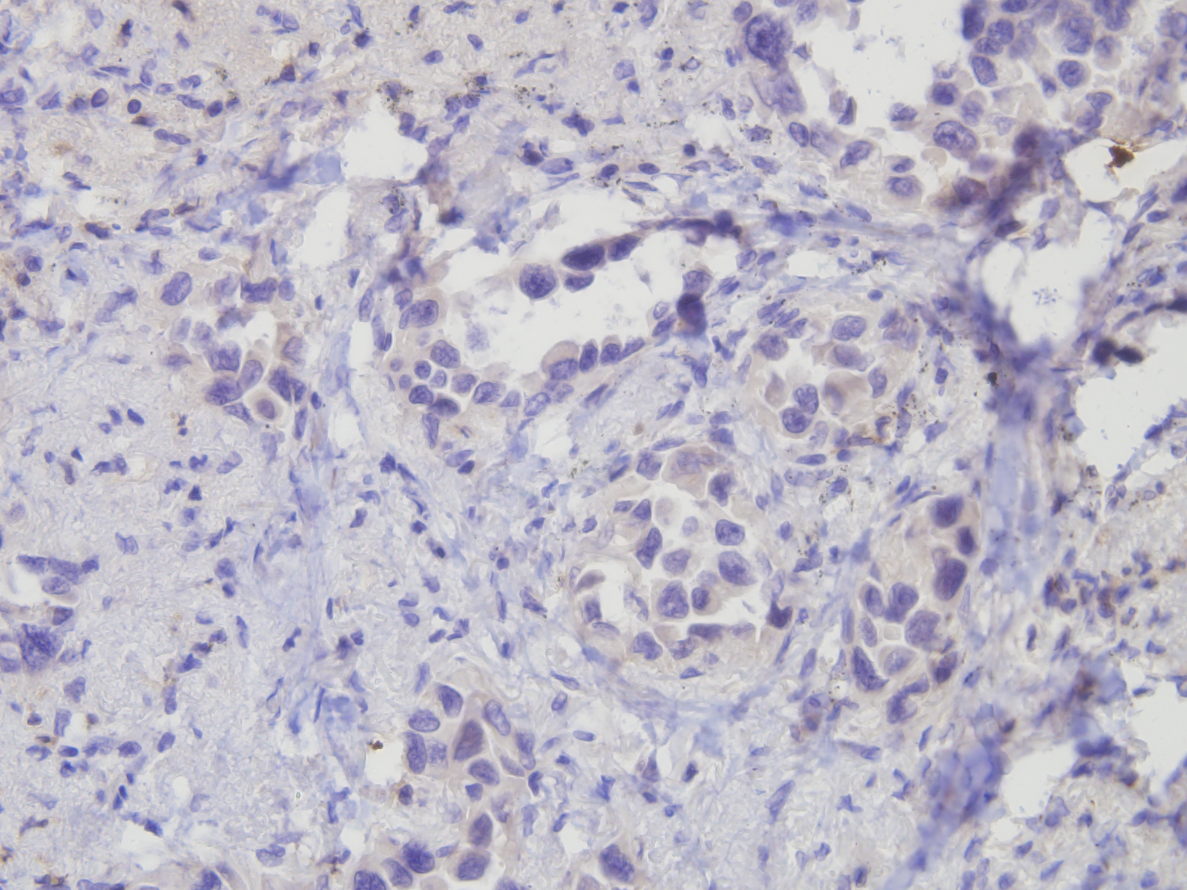

Supplement: S32 File — (ZIP) [file pone.0337223.s033.zip › 481080-400X-CA-N/481080-400X-CA (3).tif]

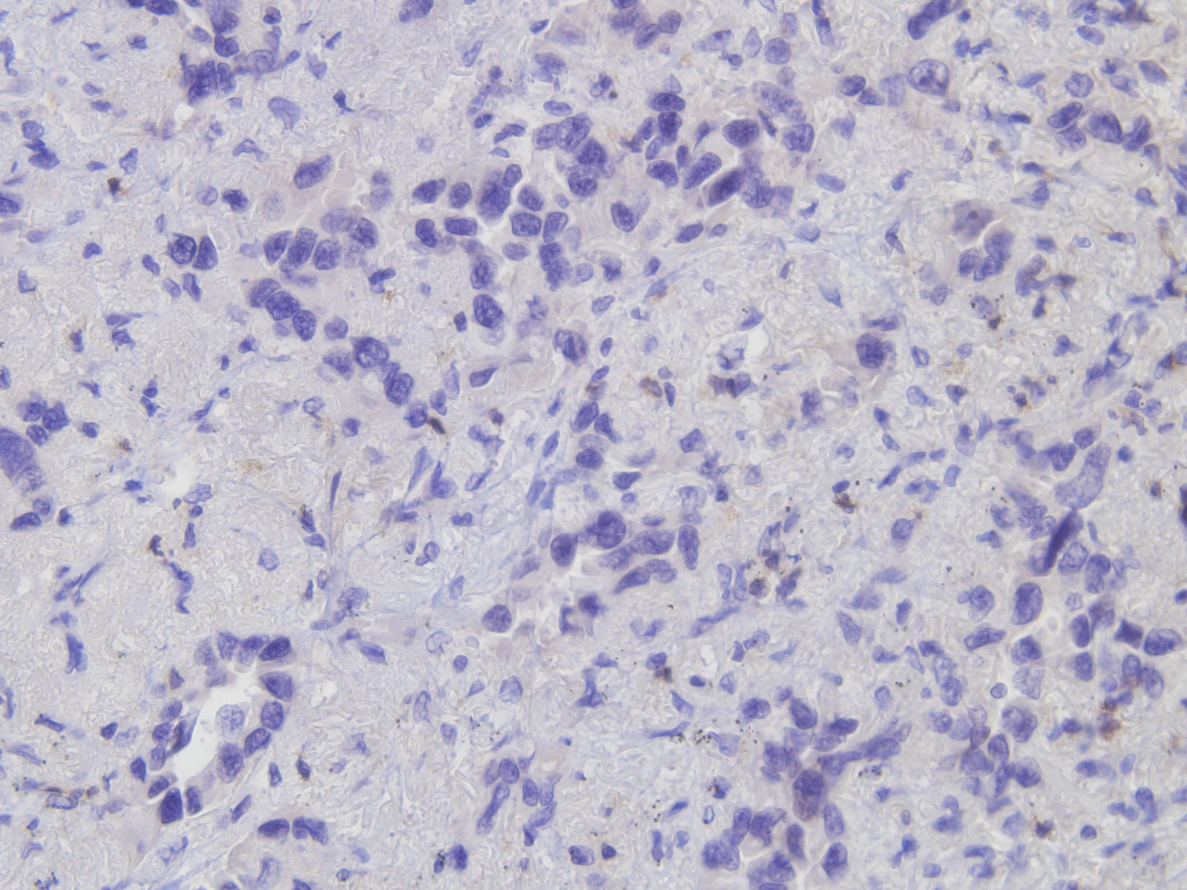

Supplement: S32 File — (ZIP) [file pone.0337223.s033.zip › 481080-400X-CA-N/481080-400X-CA (4).tif]

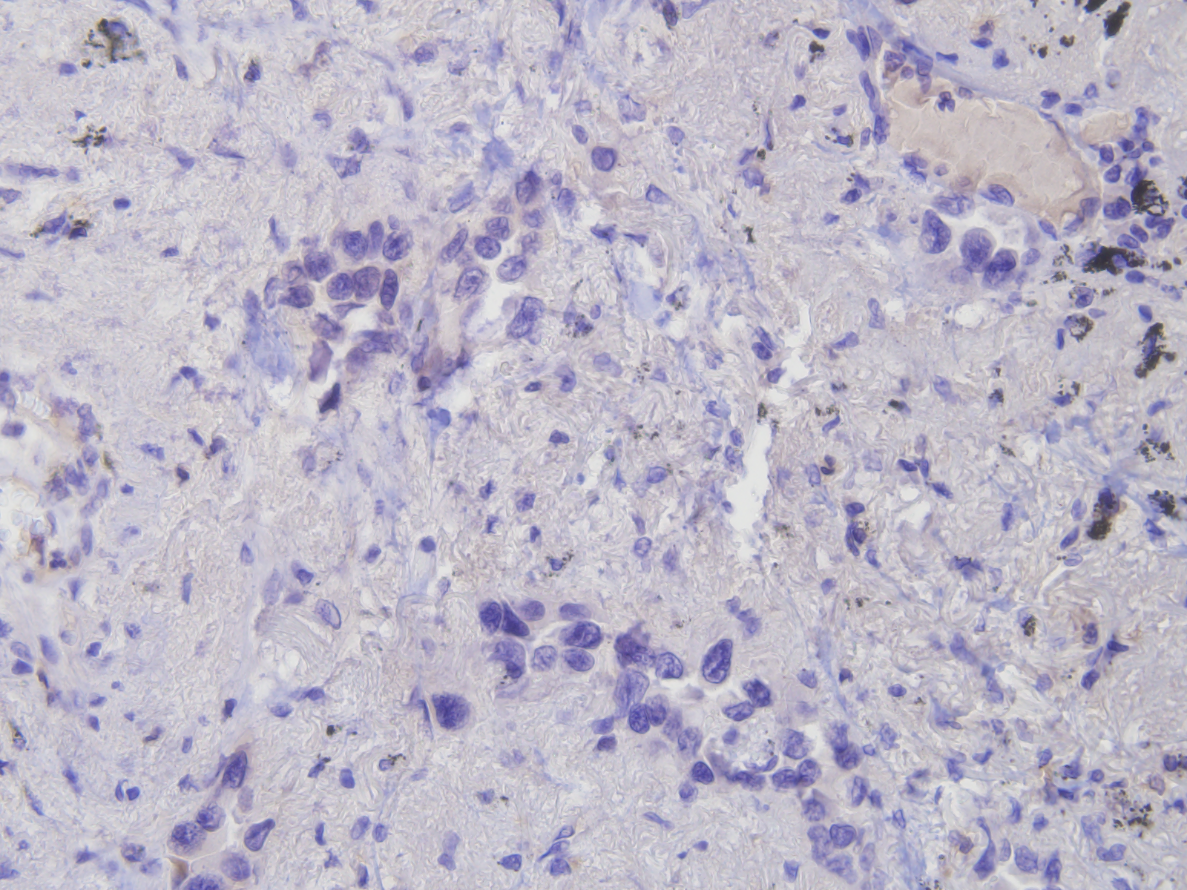

Supplement: S32 File — (ZIP) [file pone.0337223.s033.zip › 481080-400X-CA-N/481080-400X-CA (5).tif]

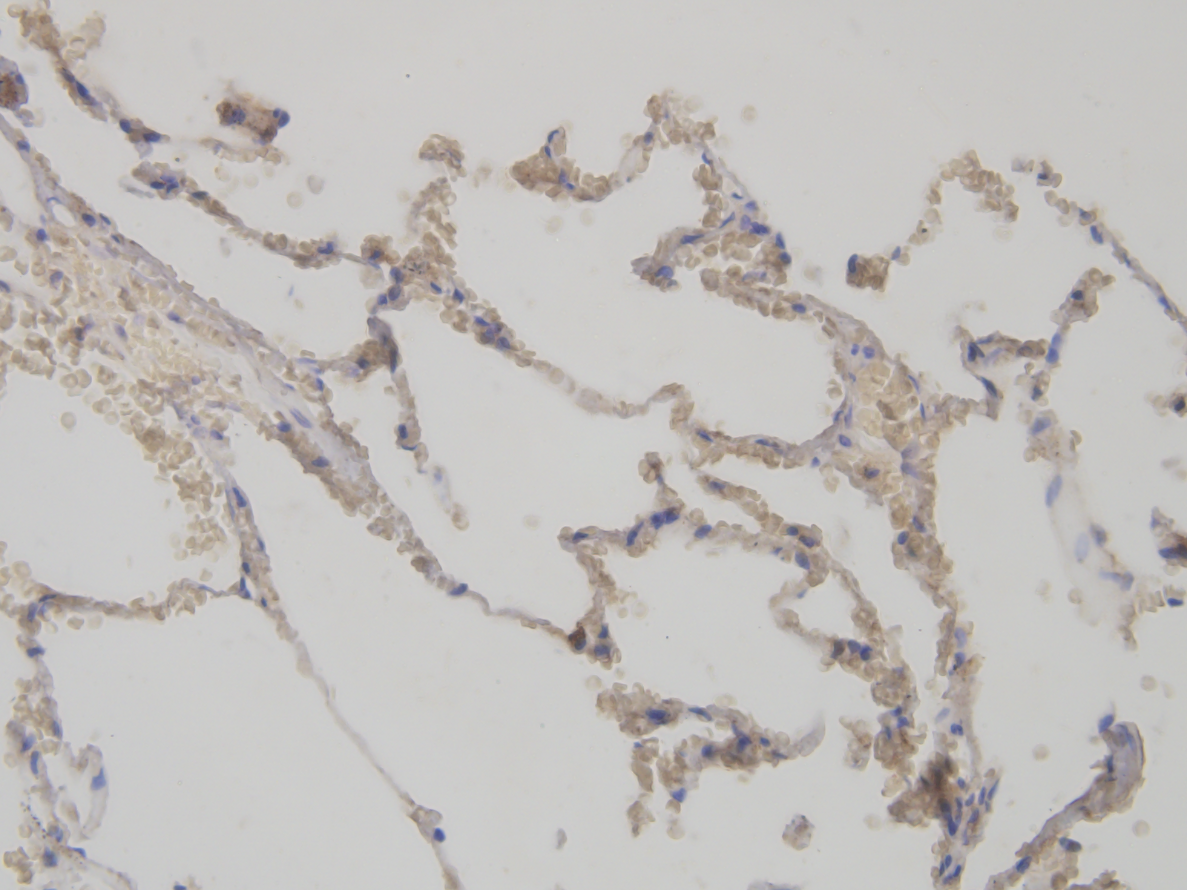

Supplement: S32 File — (ZIP) [file pone.0337223.s033.zip › 481080-400X-CA-N/481080-400X-N (1).tif]

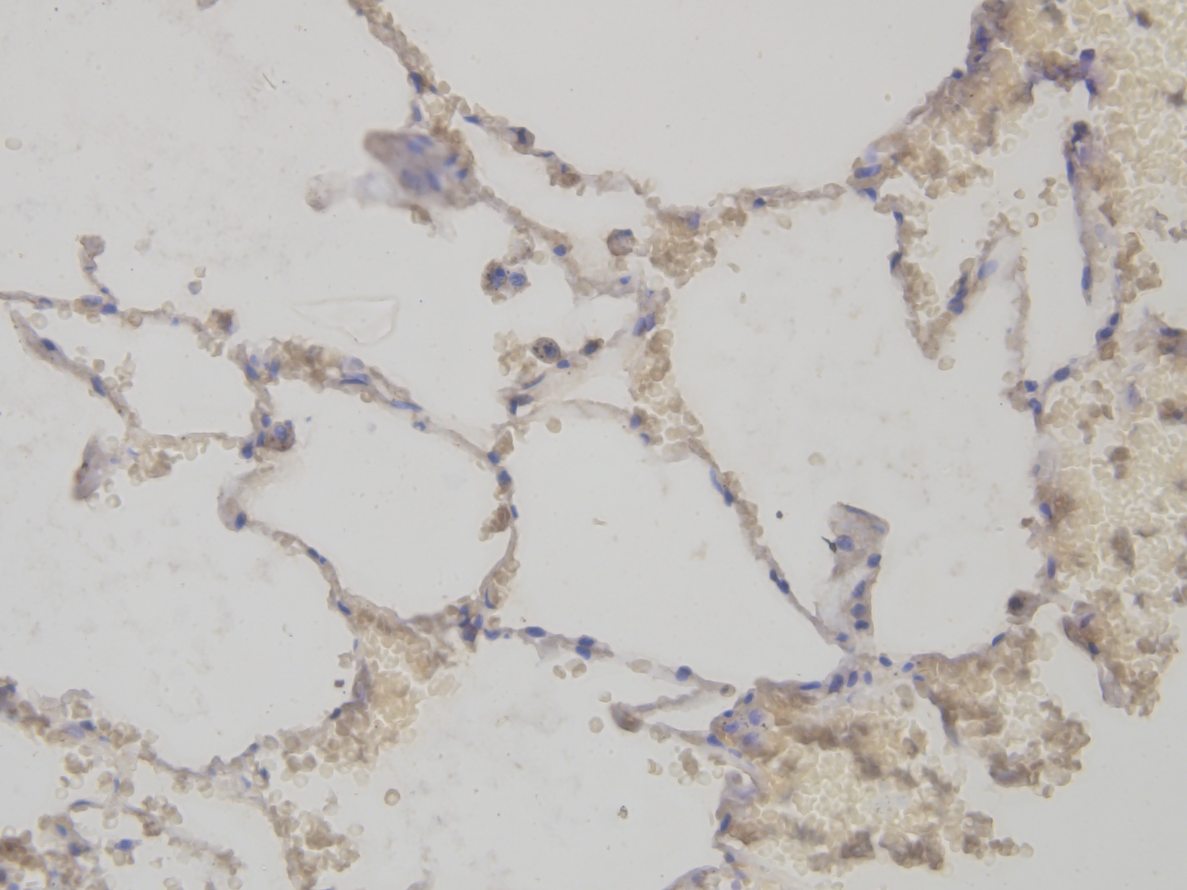

Supplement: S32 File — (ZIP) [file pone.0337223.s033.zip › 481080-400X-CA-N/481080-400X-N (2).tif]

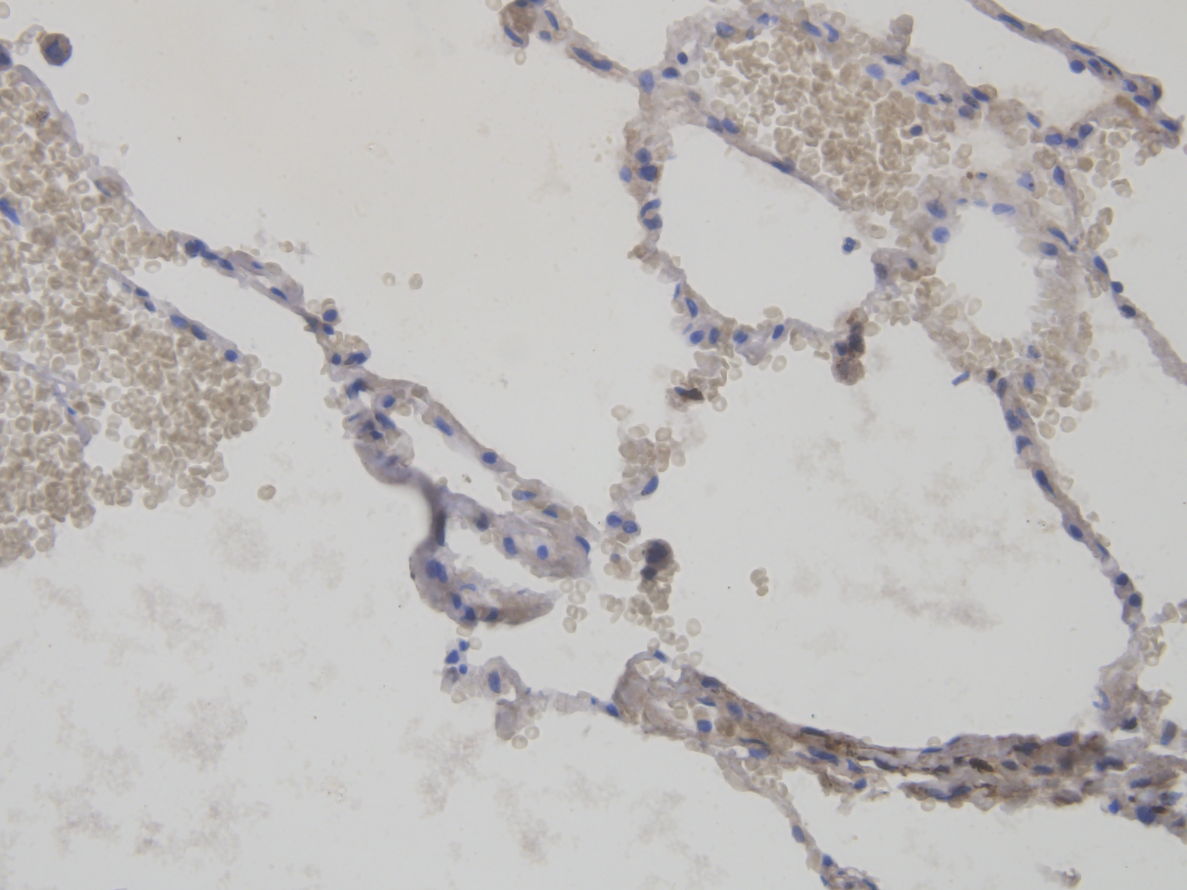

Supplement: S32 File — (ZIP) [file pone.0337223.s033.zip › 481080-400X-CA-N/481080-400X-N (3).tif]

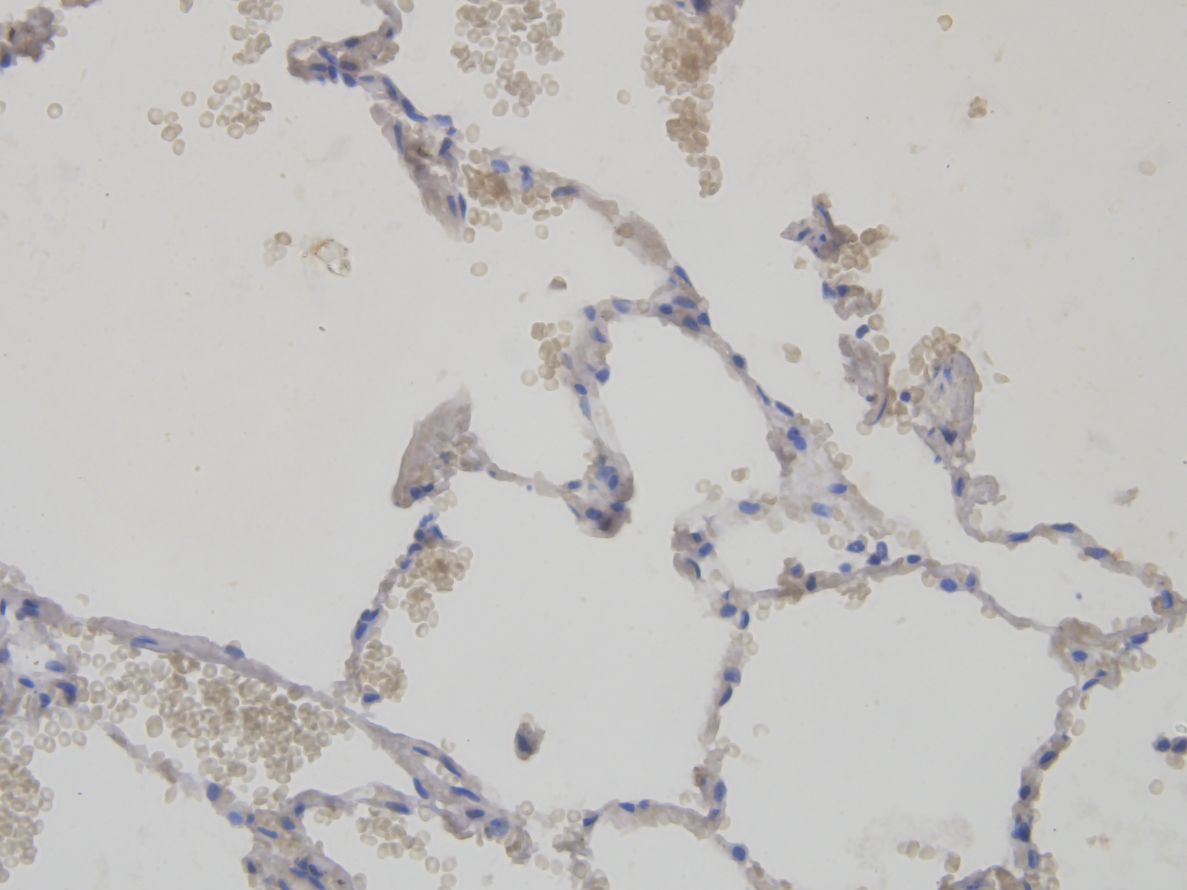

Supplement: S32 File — (ZIP) [file pone.0337223.s033.zip › 481080-400X-CA-N/481080-400X-N (4).tif]

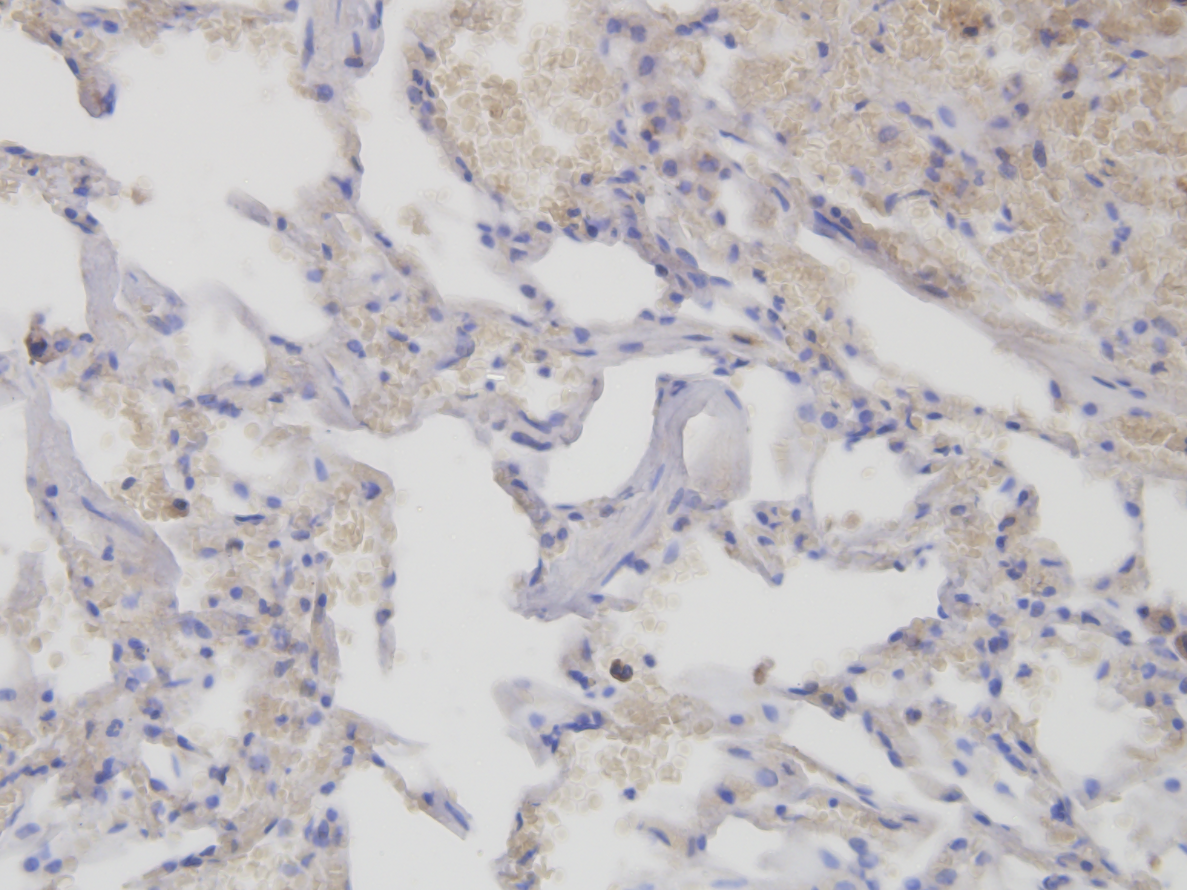

Supplement: S32 File — (ZIP) [file pone.0337223.s033.zip › 481080-400X-CA-N/481080-400X-N (5).tif]

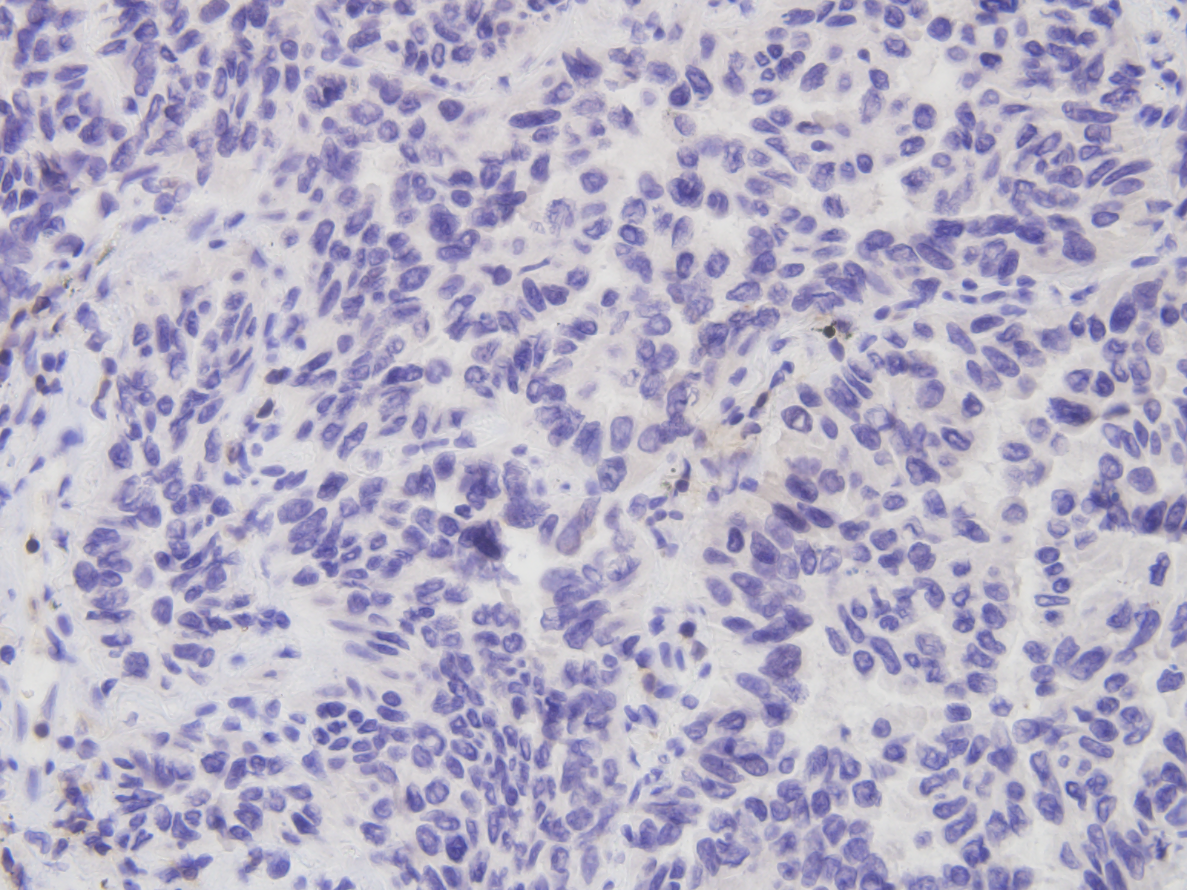

Supplement: S33 File — (ZIP) [file pone.0337223.s034.zip › 484686-400X-CA-N/484686-400X-CA (1).tif]

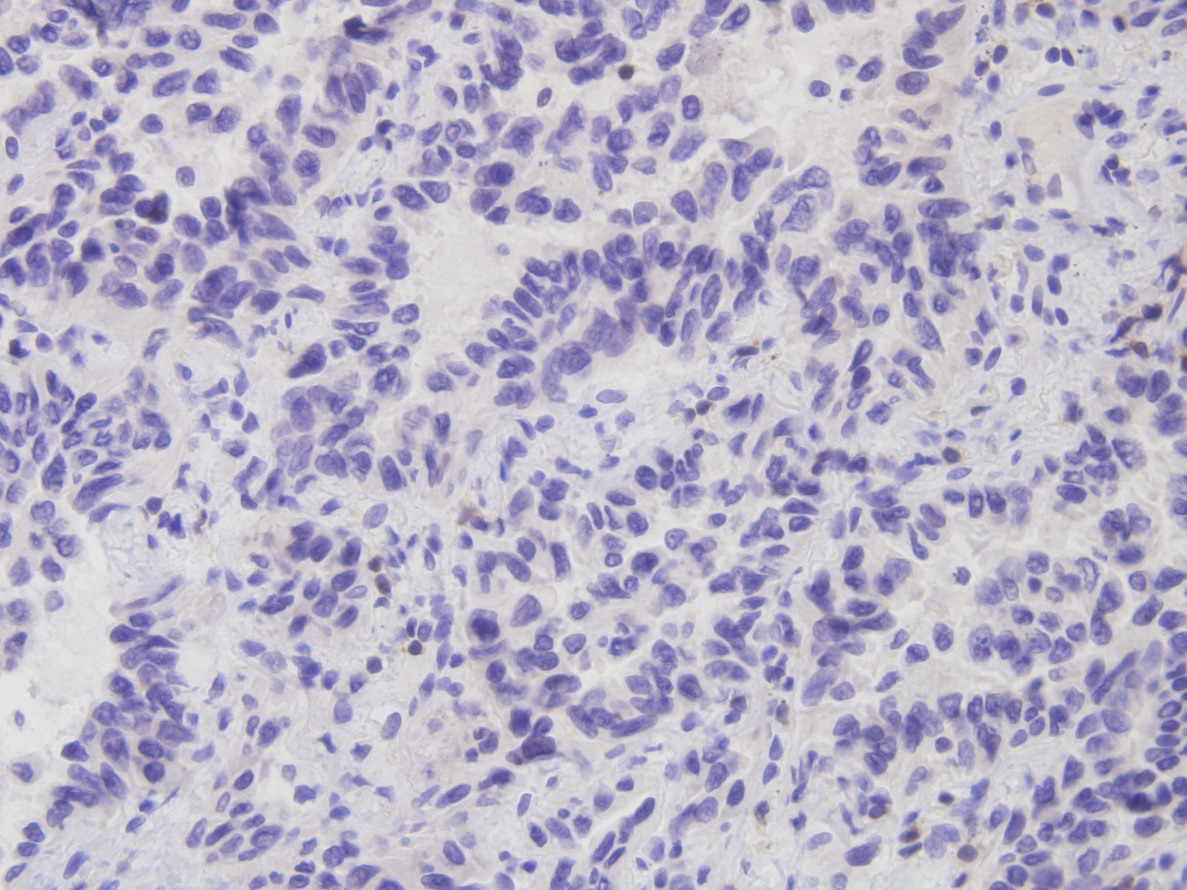

Supplement: S33 File — (ZIP) [file pone.0337223.s034.zip › 484686-400X-CA-N/484686-400X-CA (2).tif]

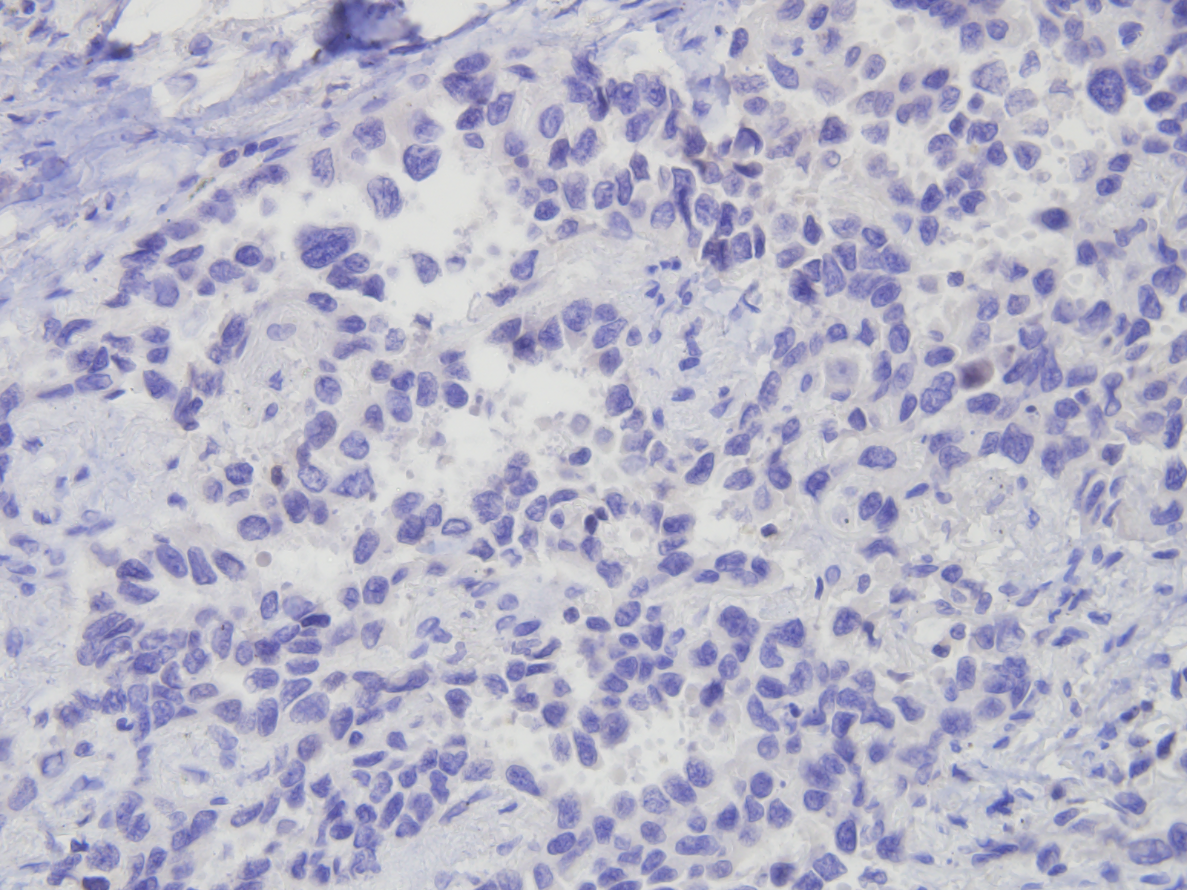

Supplement: S33 File — (ZIP) [file pone.0337223.s034.zip › 484686-400X-CA-N/484686-400X-CA (3).tif]

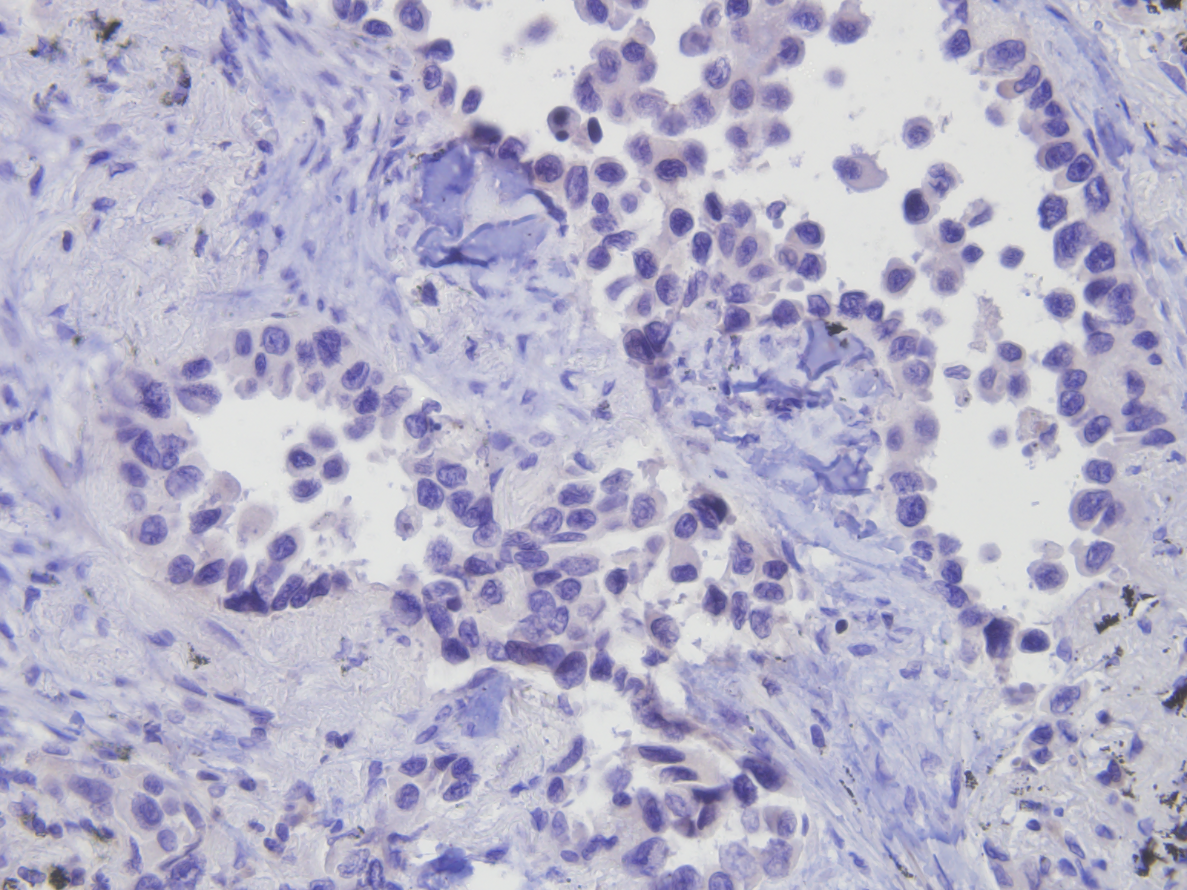

Supplement: S33 File — (ZIP) [file pone.0337223.s034.zip › 484686-400X-CA-N/484686-400X-CA (4).tif]

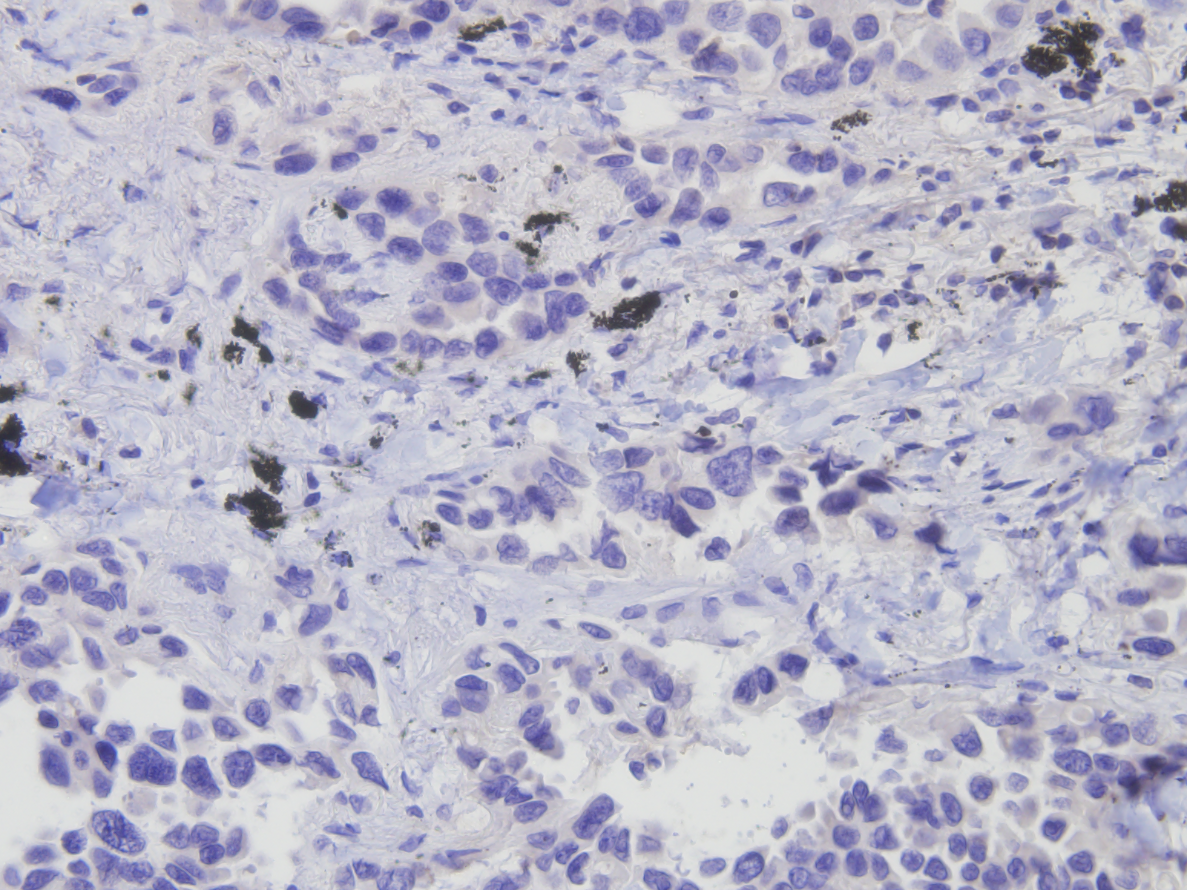

Supplement: S33 File — (ZIP) [file pone.0337223.s034.zip › 484686-400X-CA-N/484686-400X-CA (5).tif]

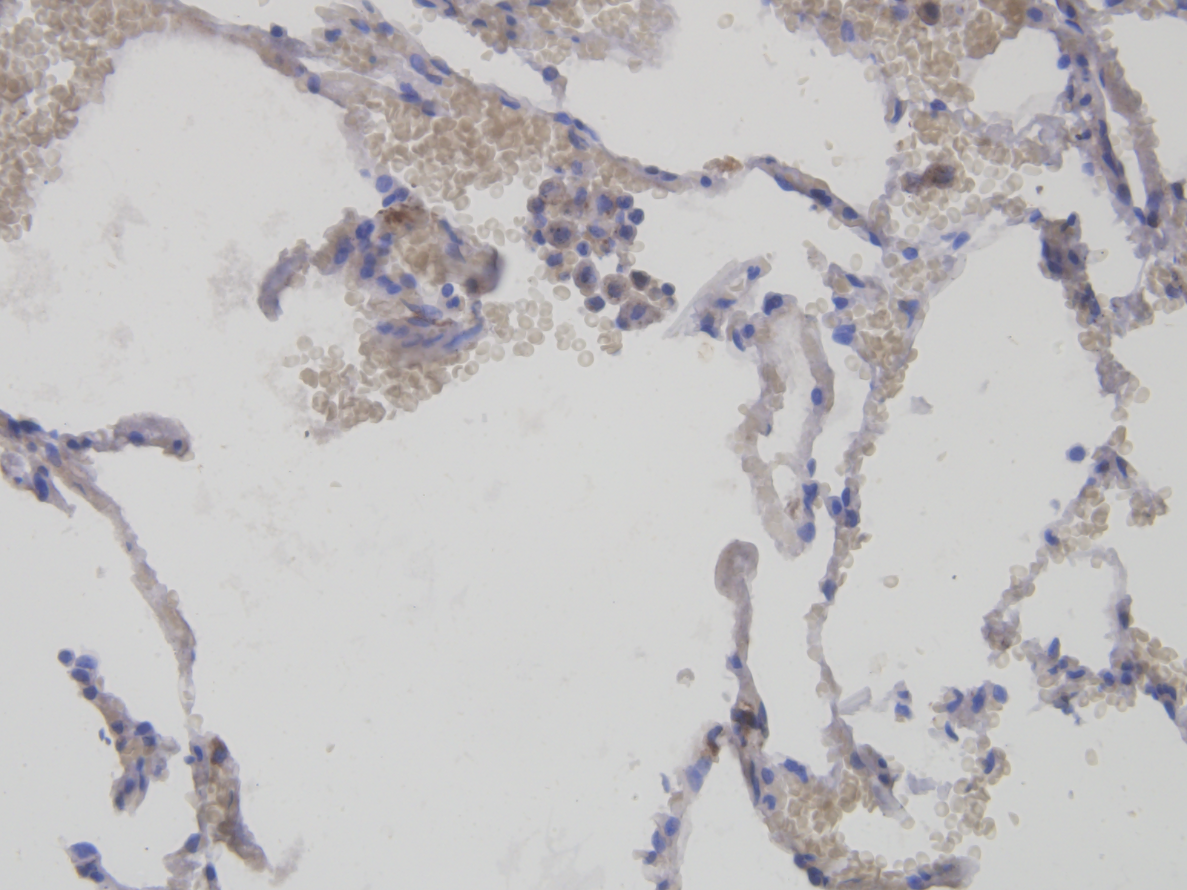

Supplement: S33 File — (ZIP) [file pone.0337223.s034.zip › 484686-400X-CA-N/484686-400X-N (1).tif]

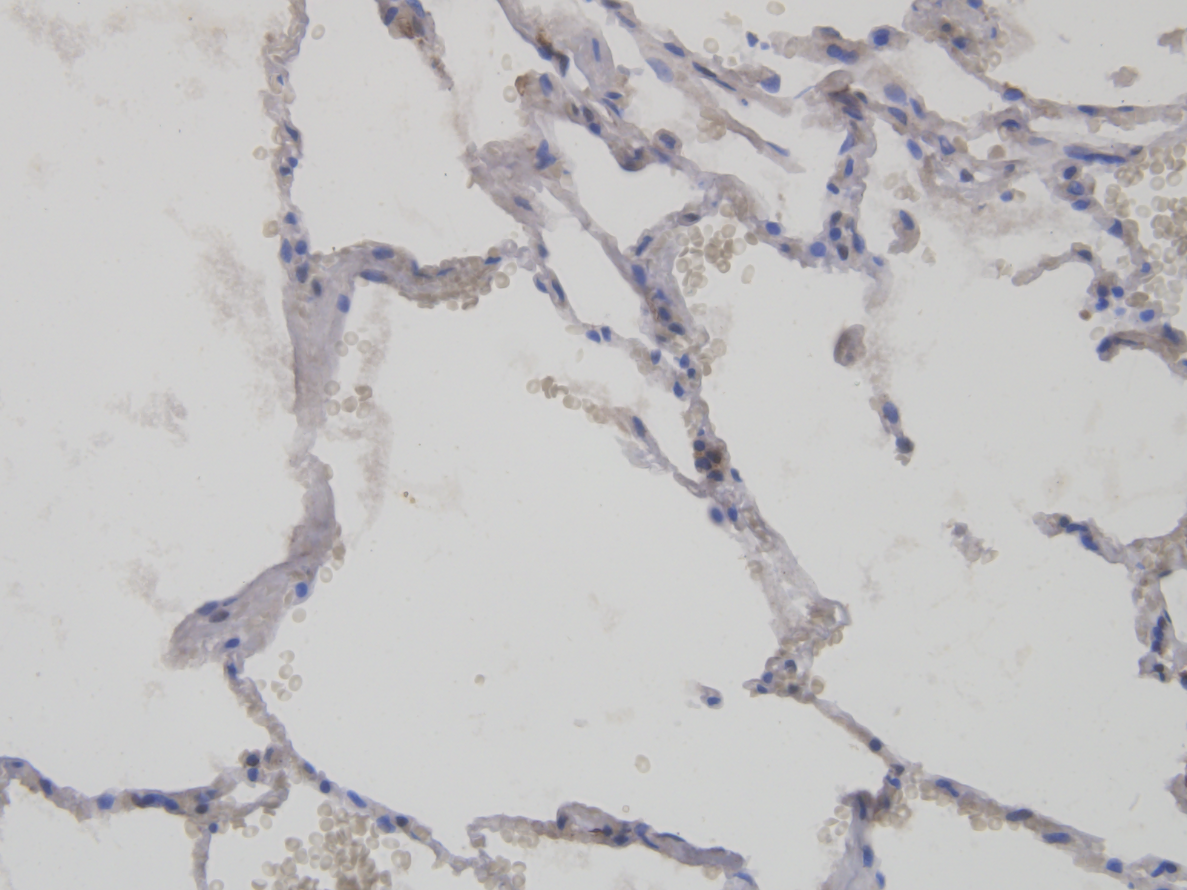

Supplement: S33 File — (ZIP) [file pone.0337223.s034.zip › 484686-400X-CA-N/484686-400X-N (2).tif]

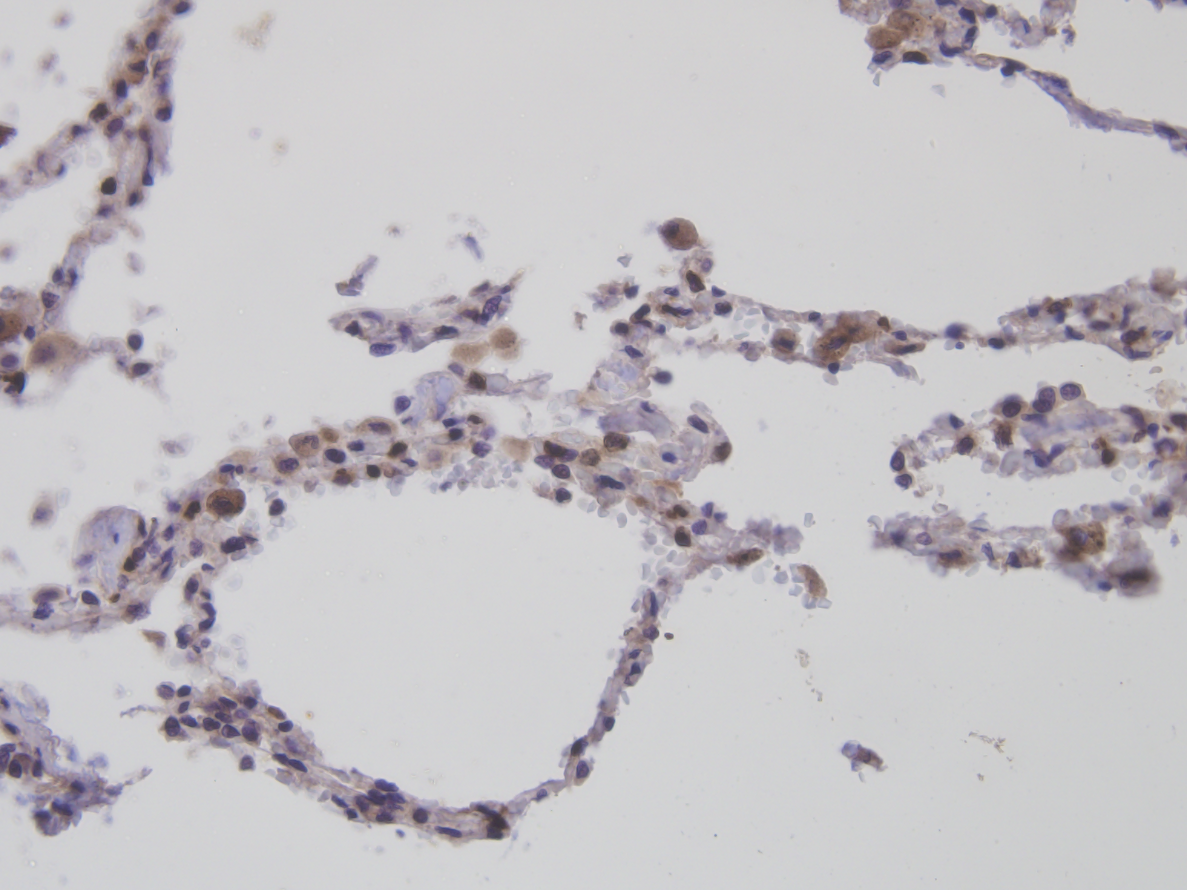

Supplement: S33 File — (ZIP) [file pone.0337223.s034.zip › 484686-400X-CA-N/484686-400X-N (3).tif]

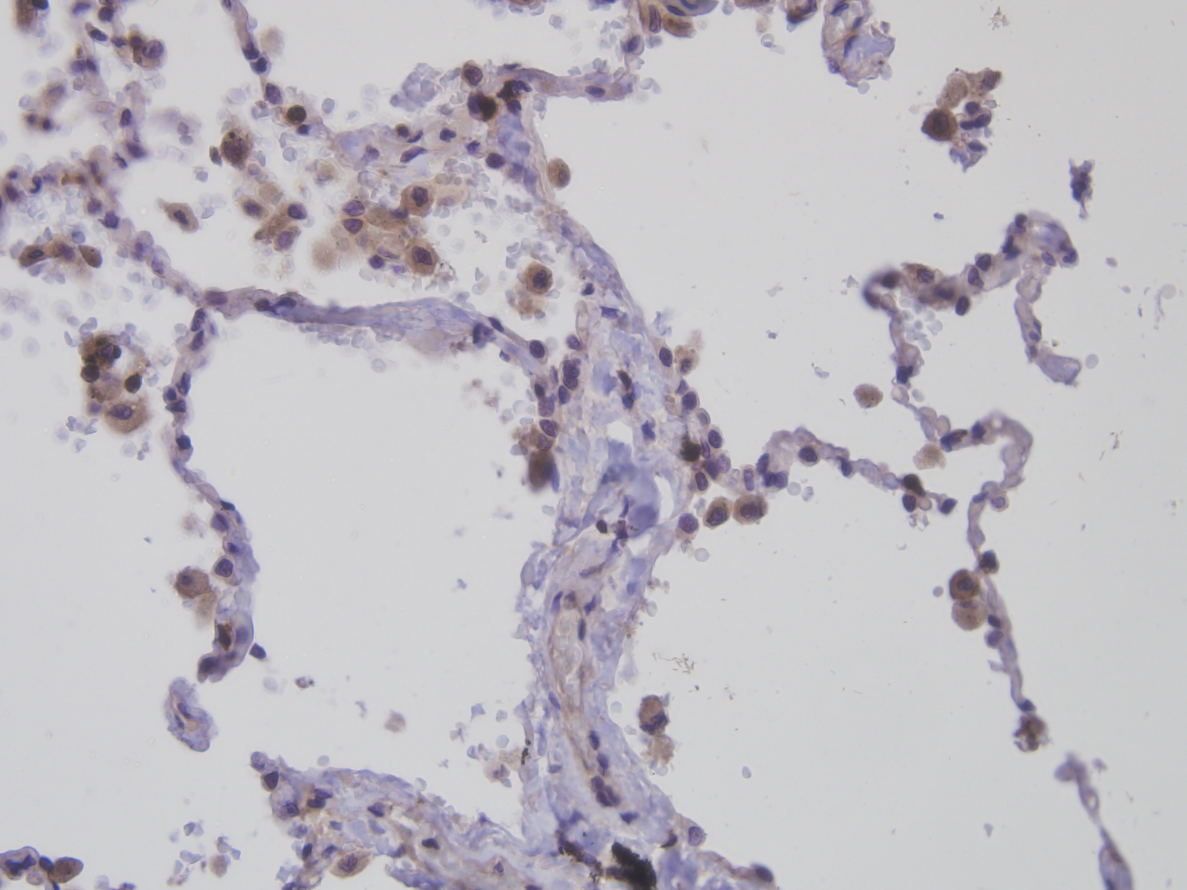

Supplement: S33 File — (ZIP) [file pone.0337223.s034.zip › 484686-400X-CA-N/484686-400X-N (4).tif]

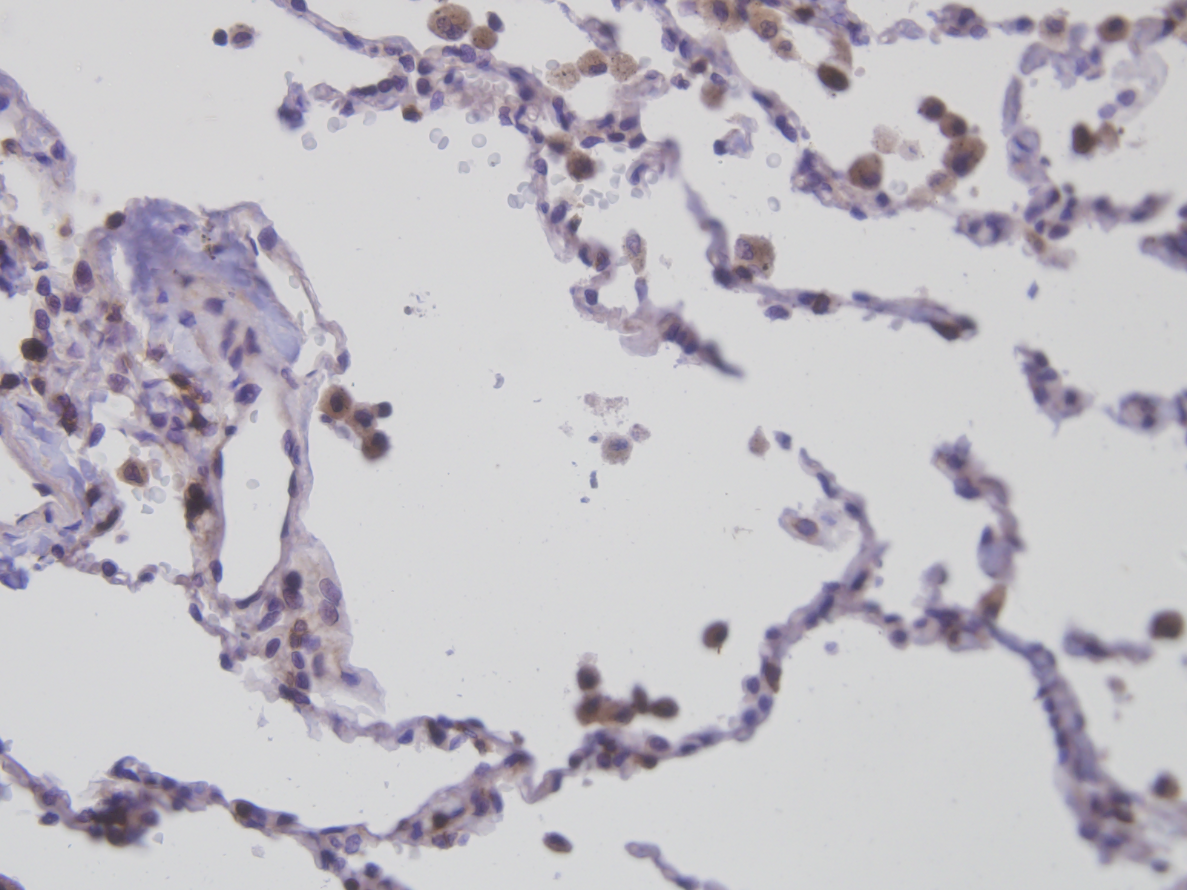

Supplement: S33 File — (ZIP) [file pone.0337223.s034.zip › 484686-400X-CA-N/484686-400X-N (5).tif]

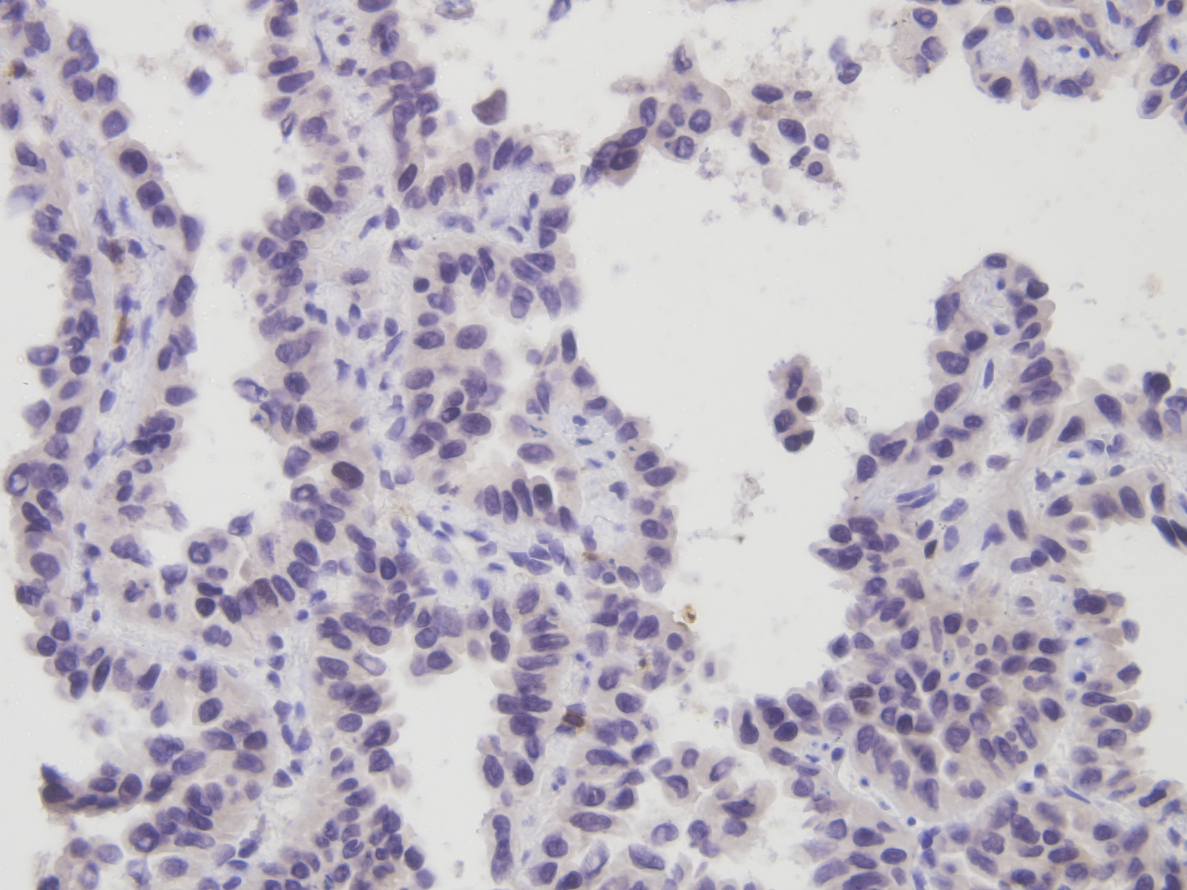

Supplement: S34 File — (ZIP) [file pone.0337223.s035.zip › 486066-400X-CA-N/486066-400X-CA (1).tif]

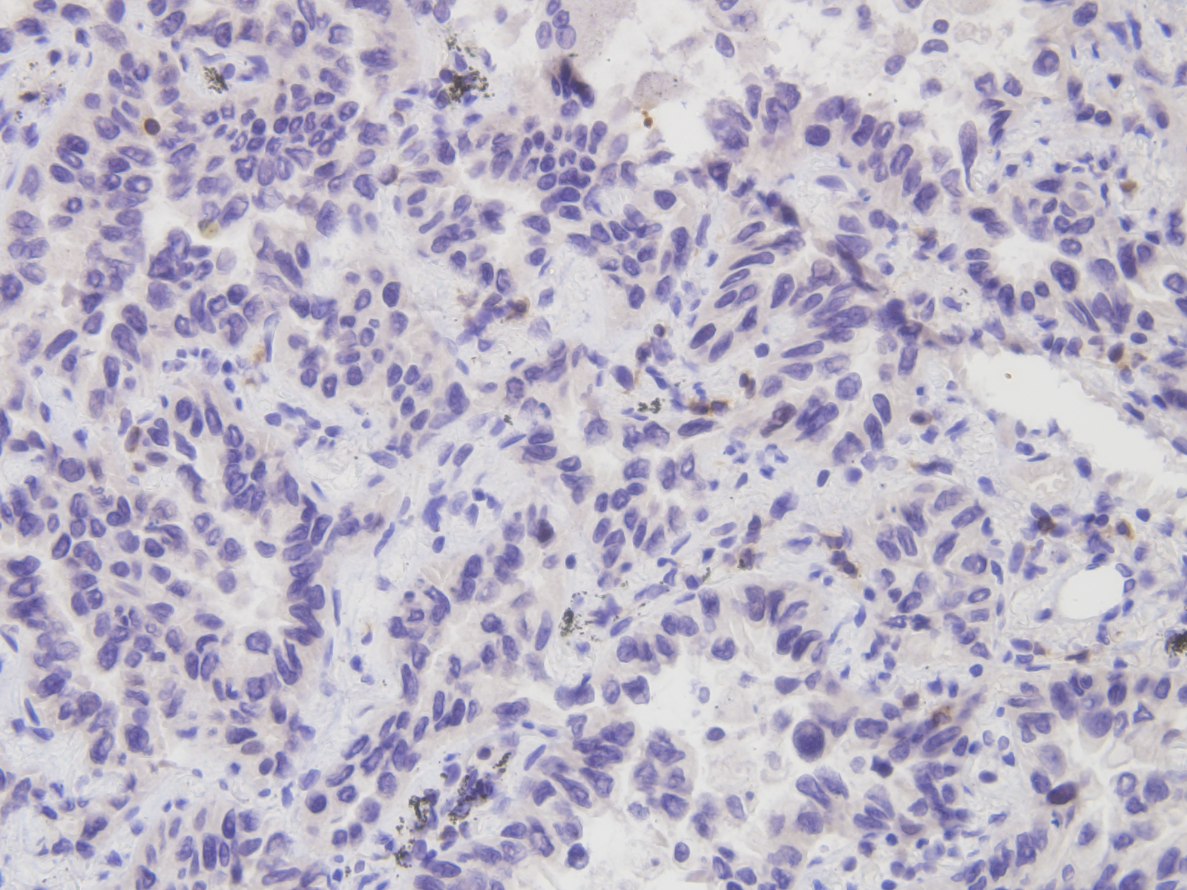

Supplement: S34 File — (ZIP) [file pone.0337223.s035.zip › 486066-400X-CA-N/486066-400X-CA (2).tif]

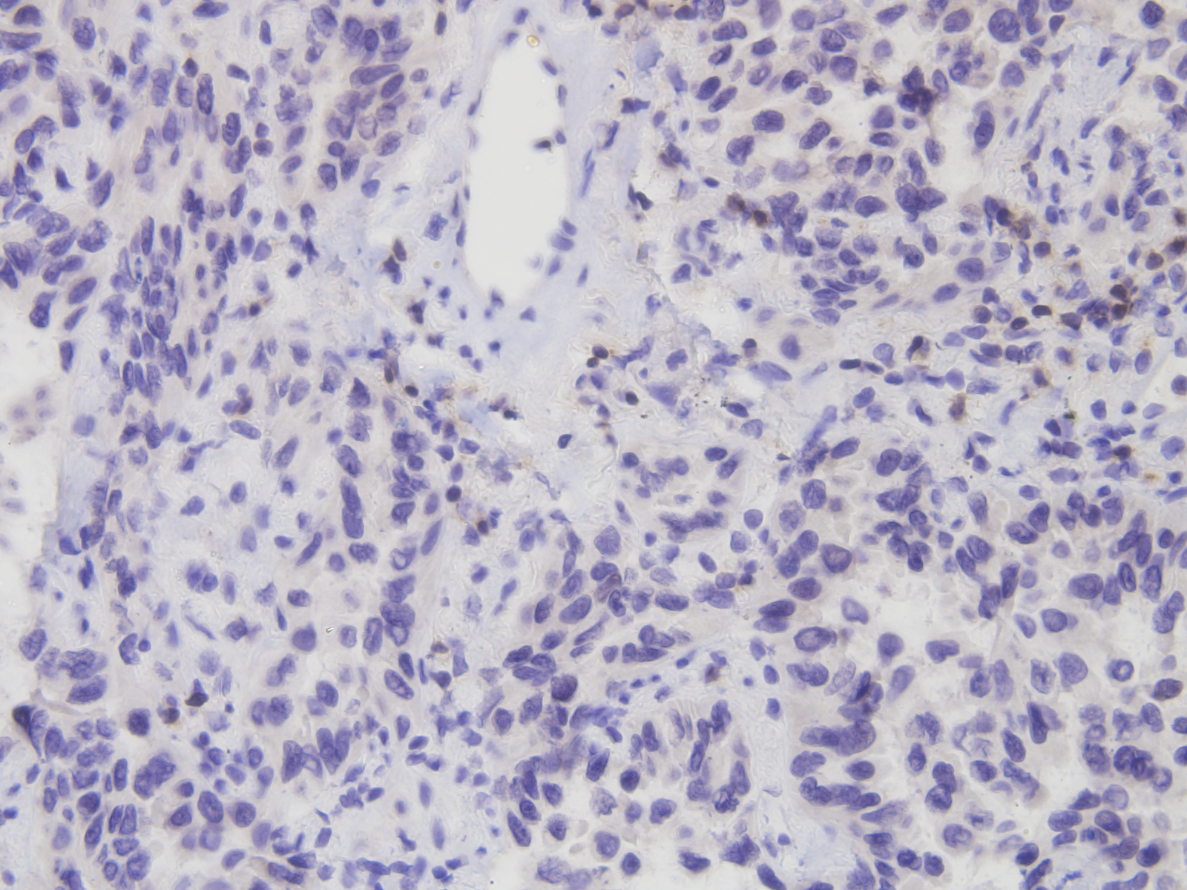

Supplement: S34 File — (ZIP) [file pone.0337223.s035.zip › 486066-400X-CA-N/486066-400X-CA (3).tif]

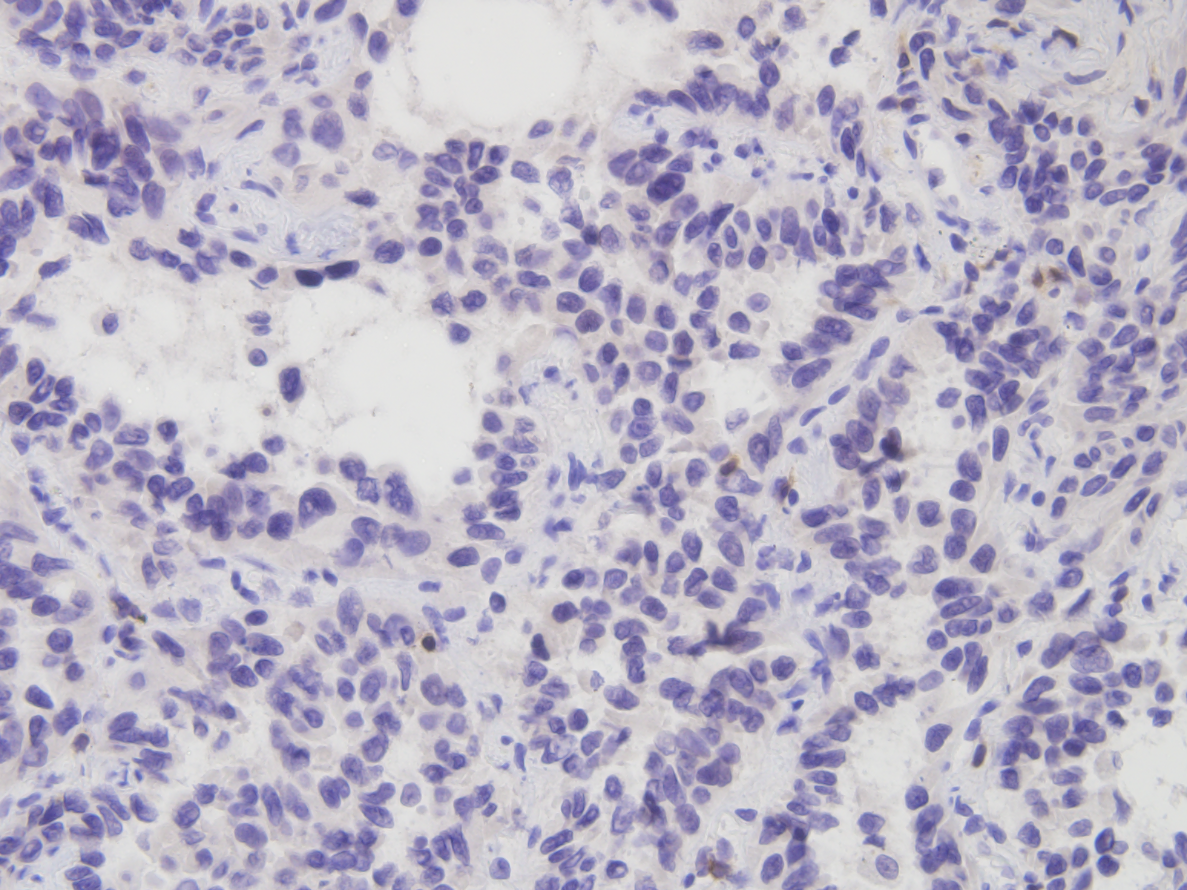

Supplement: S34 File — (ZIP) [file pone.0337223.s035.zip › 486066-400X-CA-N/486066-400X-CA (4).tif]

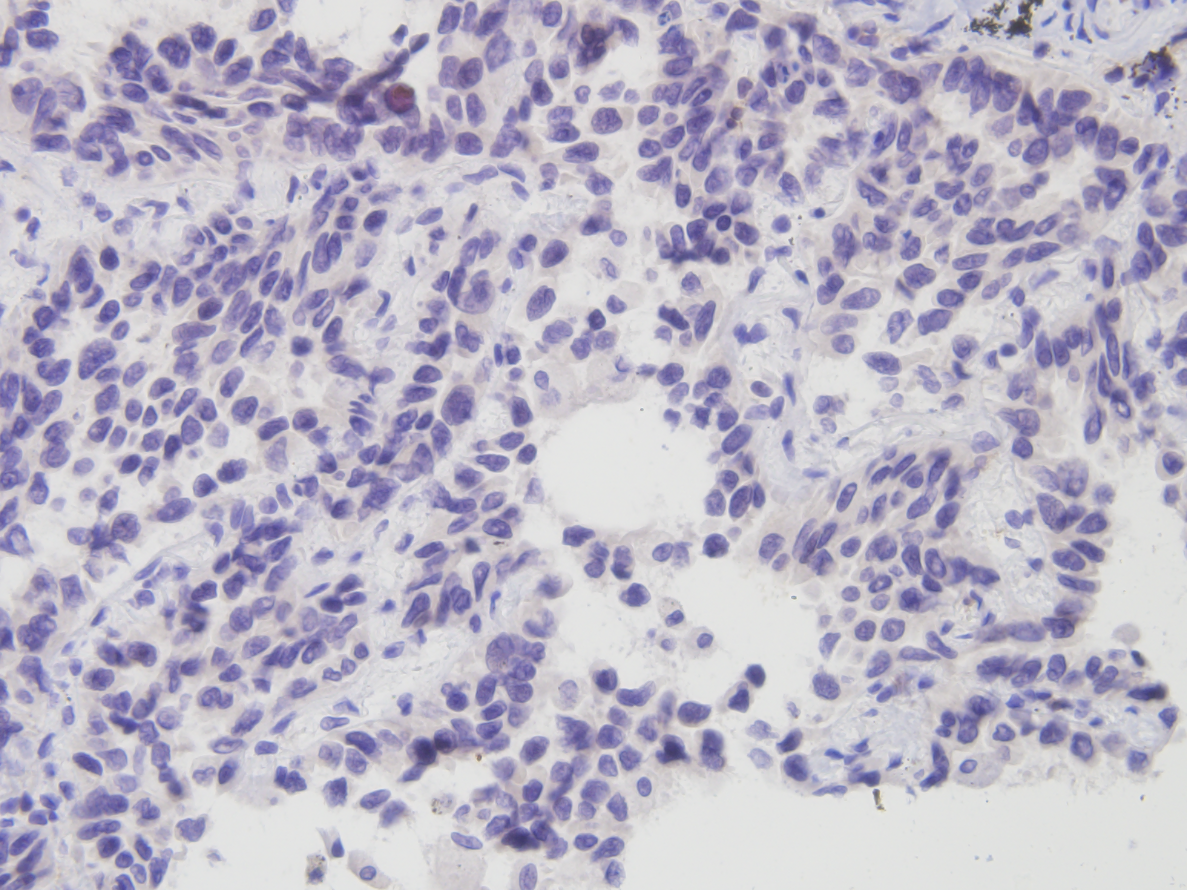

Supplement: S34 File — (ZIP) [file pone.0337223.s035.zip › 486066-400X-CA-N/486066-400X-CA (5).tif]

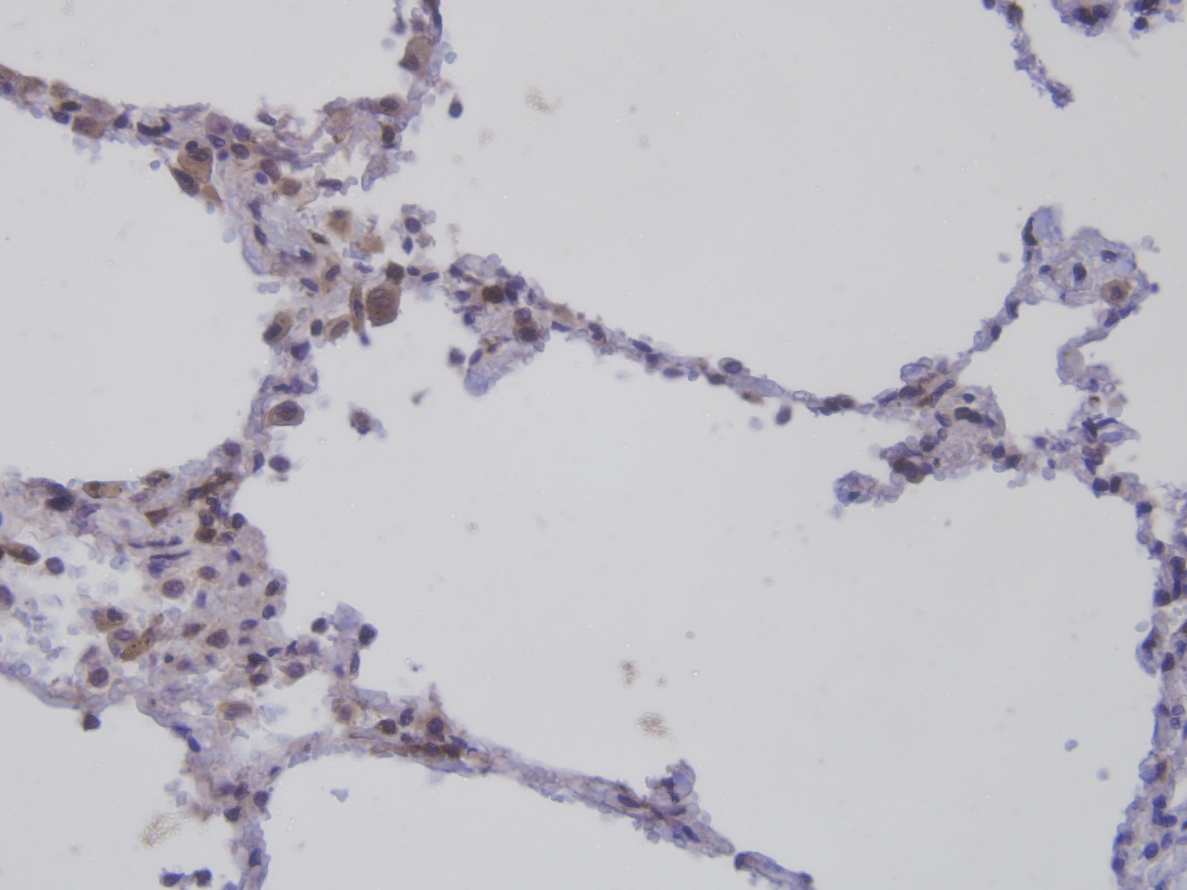

Supplement: S34 File — (ZIP) [file pone.0337223.s035.zip › 486066-400X-CA-N/486066-400X-N (1).tif]

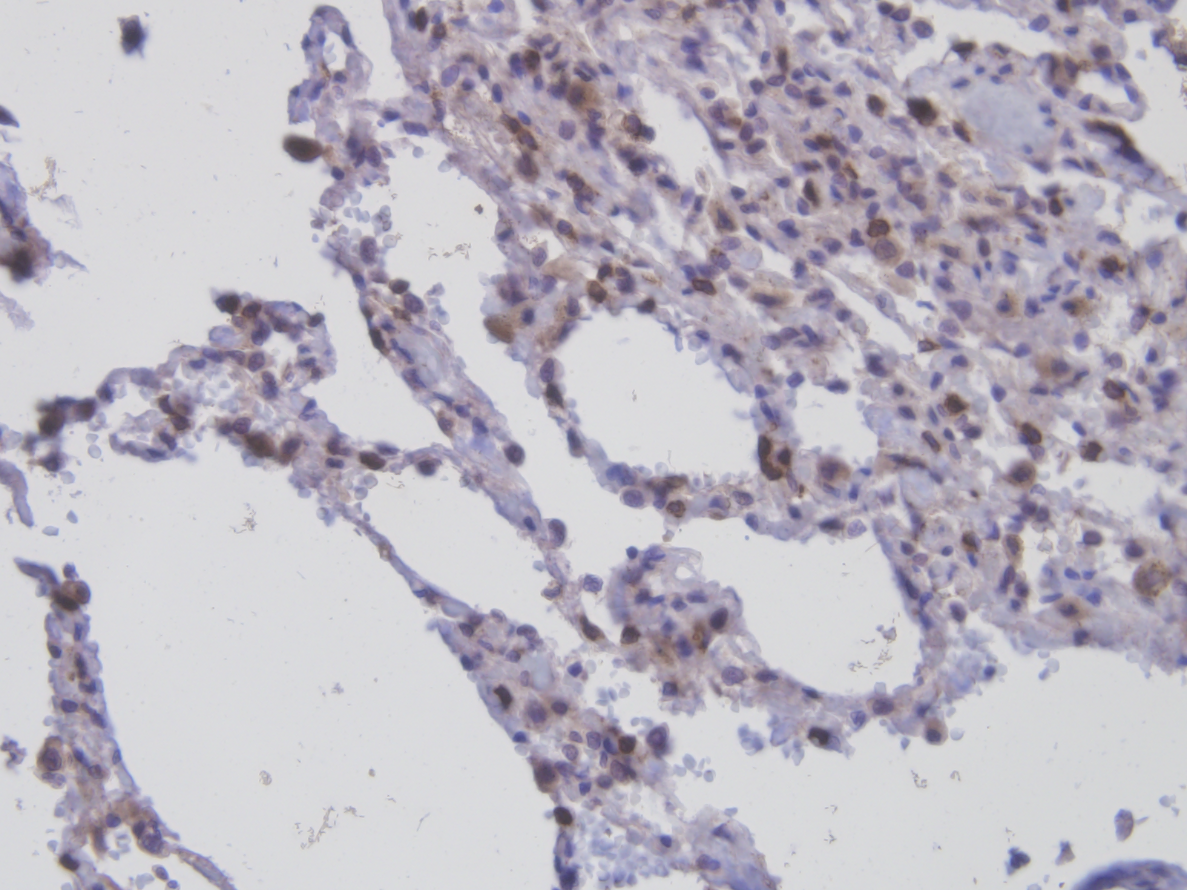

Supplement: S34 File — (ZIP) [file pone.0337223.s035.zip › 486066-400X-CA-N/486066-400X-N (2).tif]

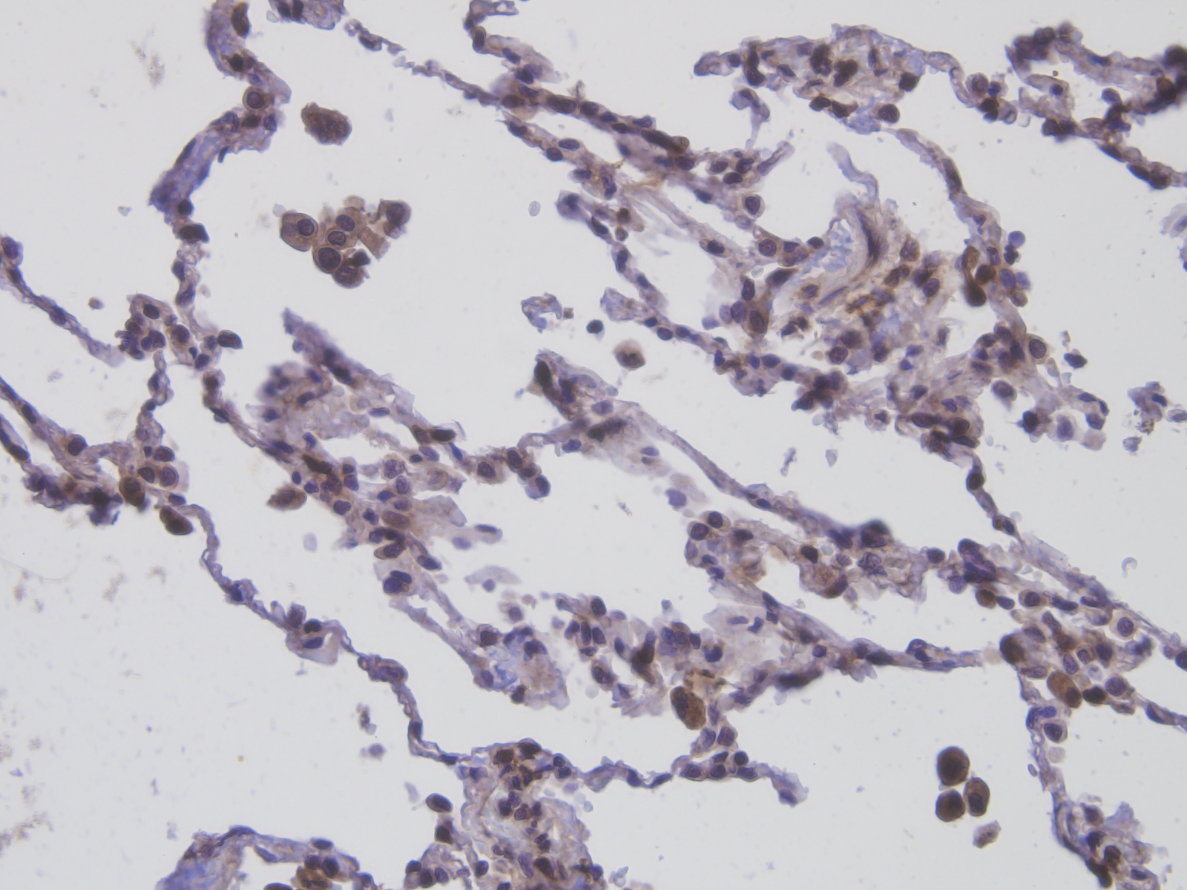

Supplement: S34 File — (ZIP) [file pone.0337223.s035.zip › 486066-400X-CA-N/486066-400X-N (3).tif]

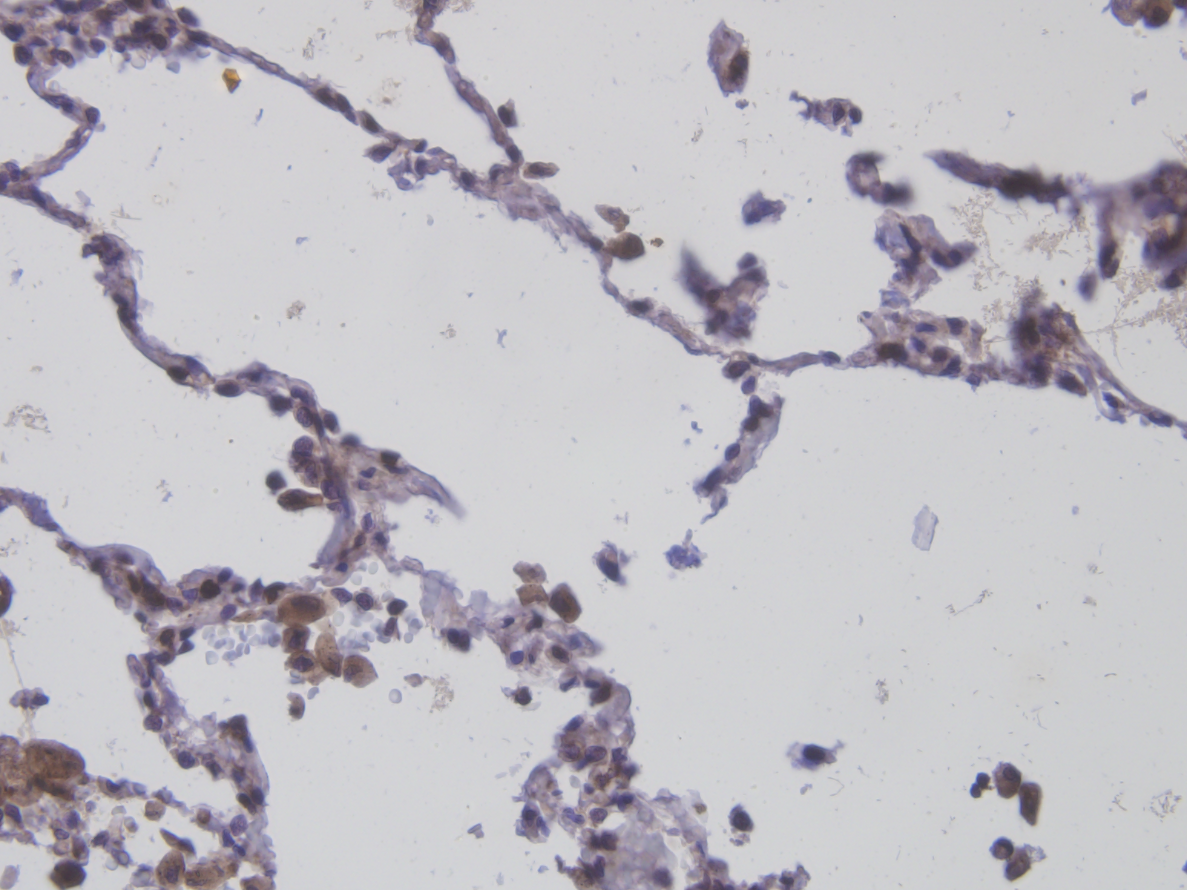

Supplement: S34 File — (ZIP) [file pone.0337223.s035.zip › 486066-400X-CA-N/486066-400X-N (4).tif]

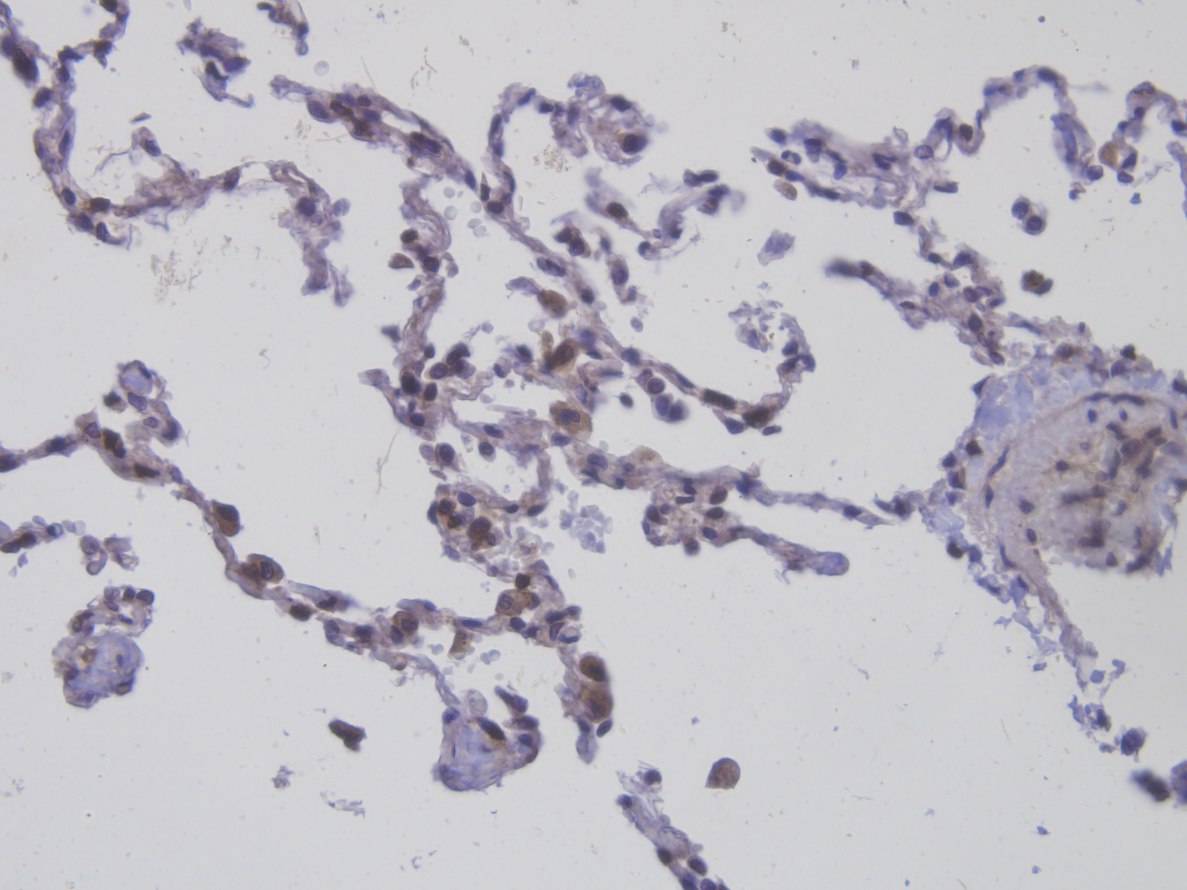

Supplement: S34 File — (ZIP) [file pone.0337223.s035.zip › 486066-400X-CA-N/486066-400X-N (5).tif]

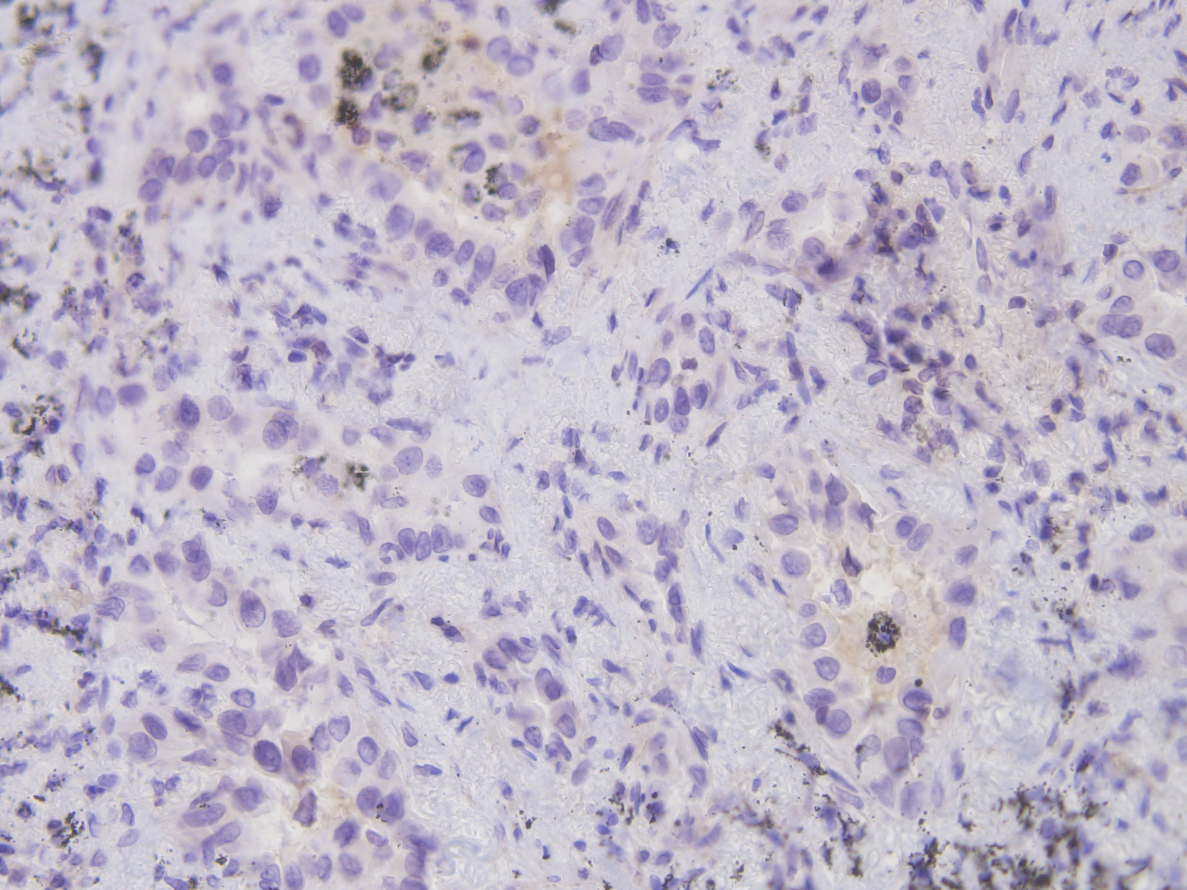

Supplement: S35 File — (ZIP) [file pone.0337223.s036.zip › 486371-400-CA-N/486371-400-CA (1).tif]

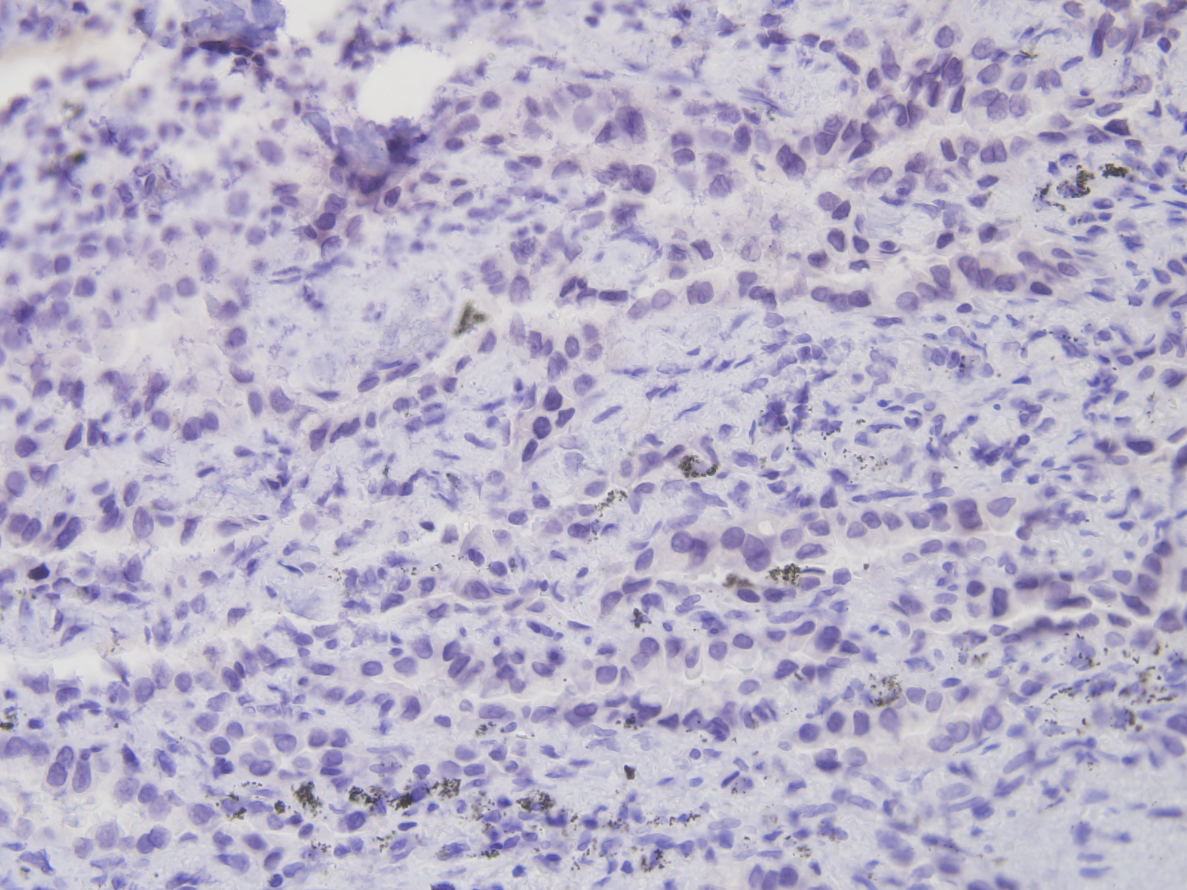

Supplement: S35 File — (ZIP) [file pone.0337223.s036.zip › 486371-400-CA-N/486371-400-CA (2).tif]

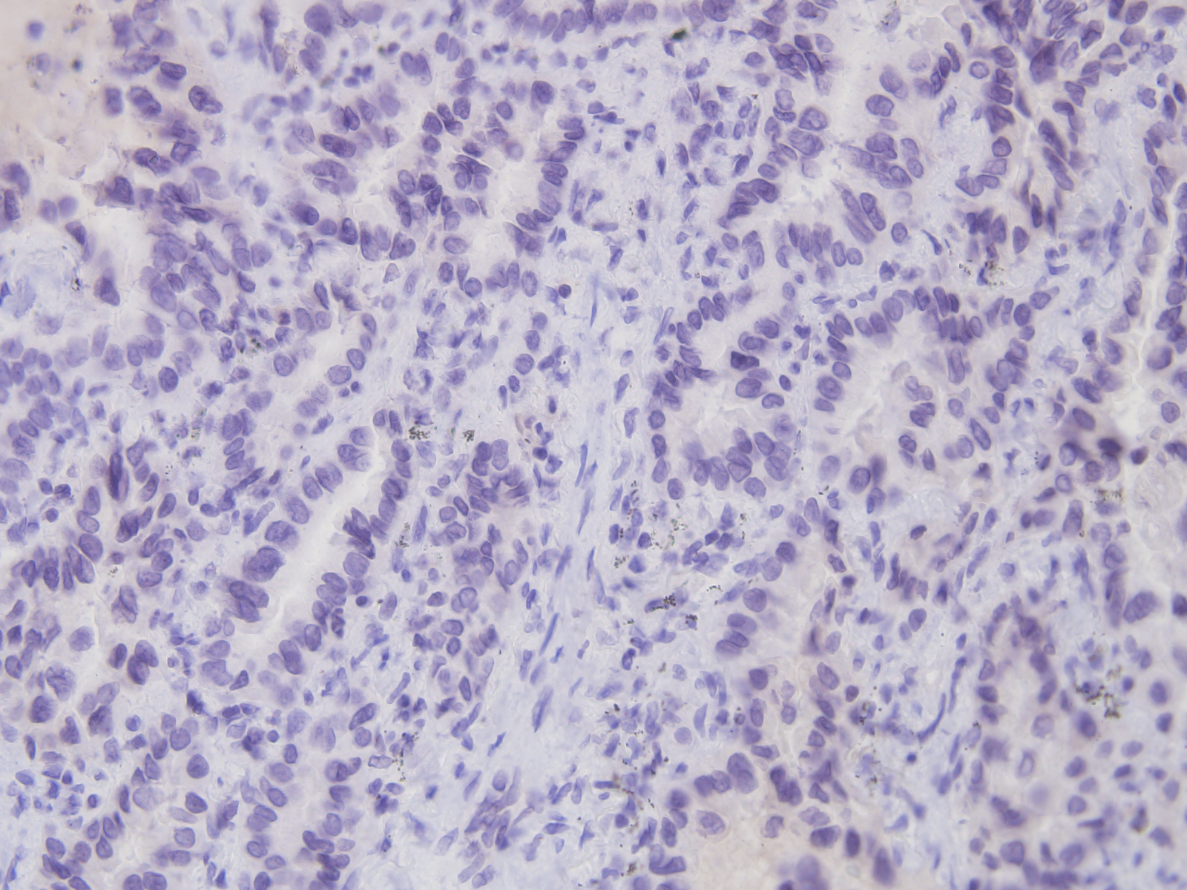

Supplement: S35 File — (ZIP) [file pone.0337223.s036.zip › 486371-400-CA-N/486371-400-CA (3).tif]

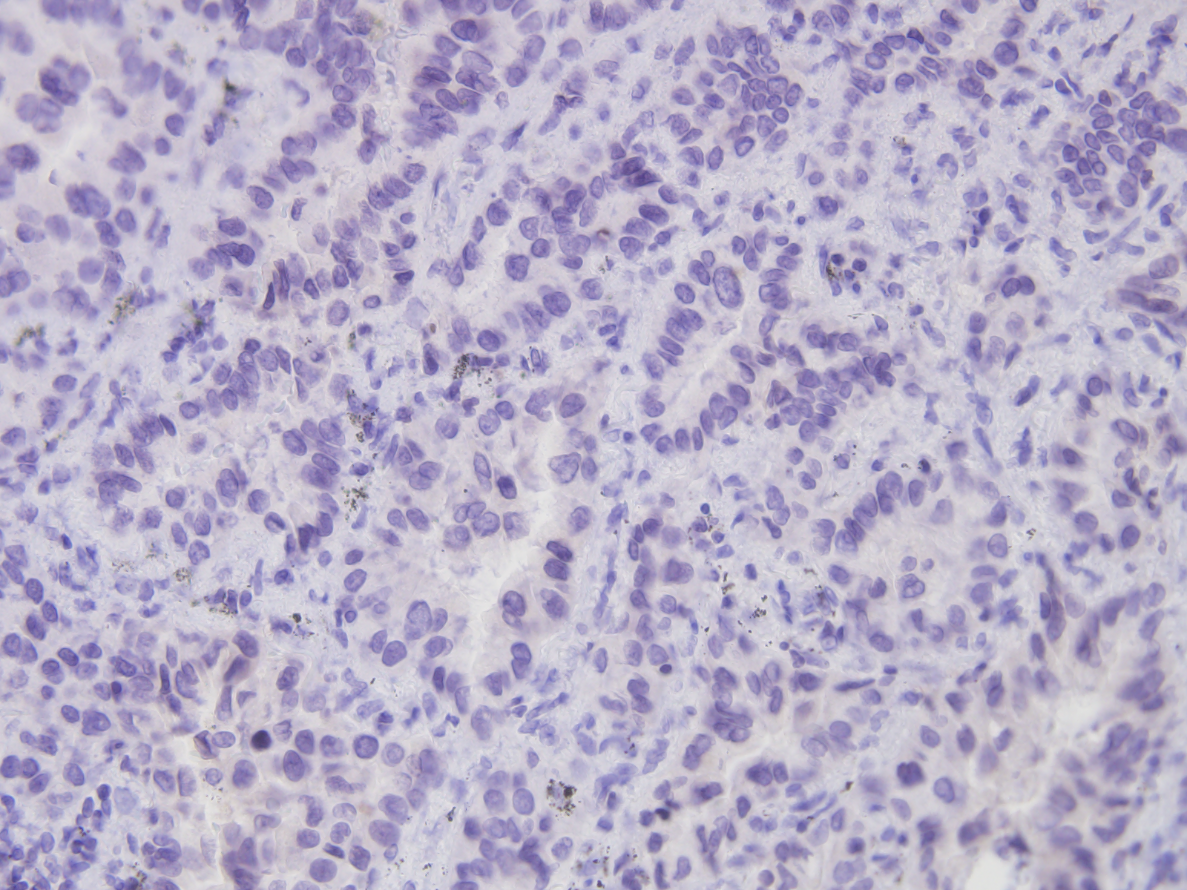

Supplement: S35 File — (ZIP) [file pone.0337223.s036.zip › 486371-400-CA-N/486371-400-CA (4).tif]

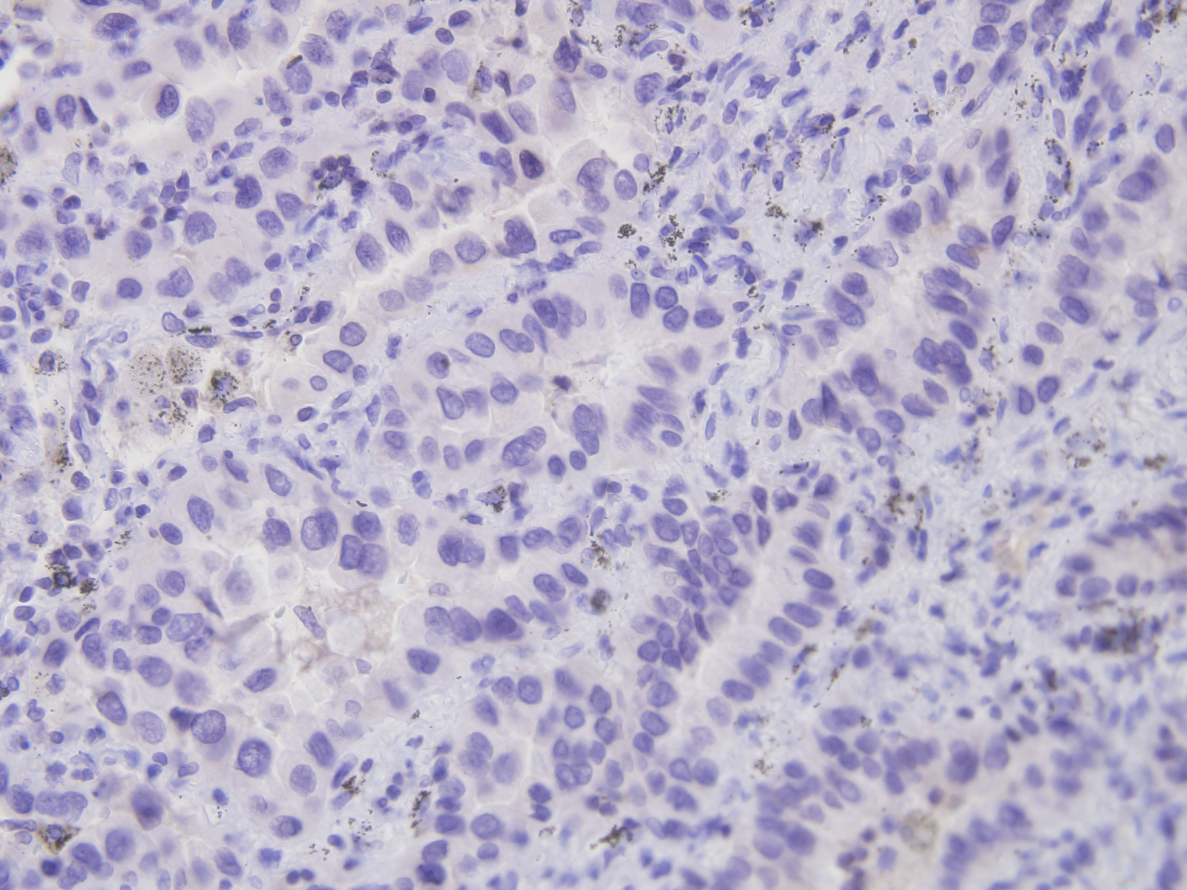

Supplement: S35 File — (ZIP) [file pone.0337223.s036.zip › 486371-400-CA-N/486371-400-CA (5).tif]

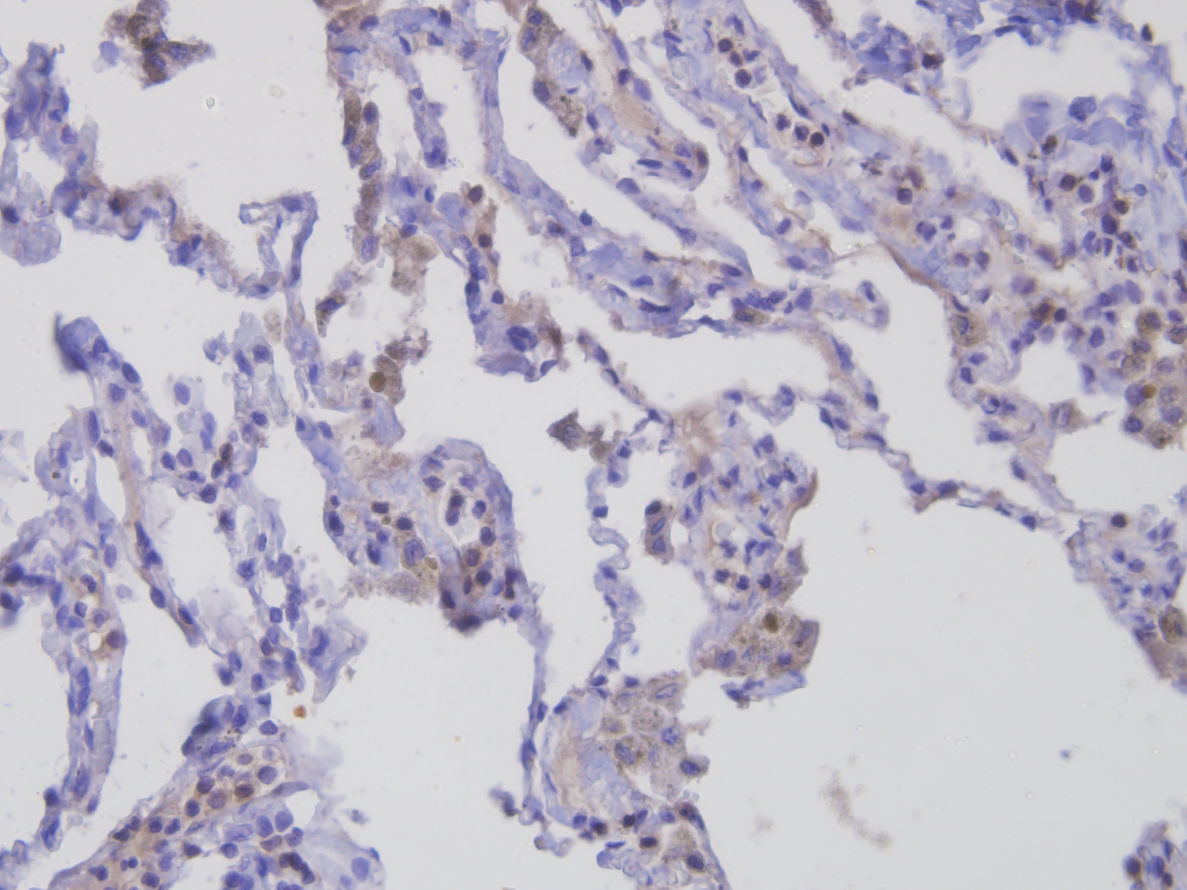

Supplement: S35 File — (ZIP) [file pone.0337223.s036.zip › 486371-400-CA-N/486371-400-N (1).tif]

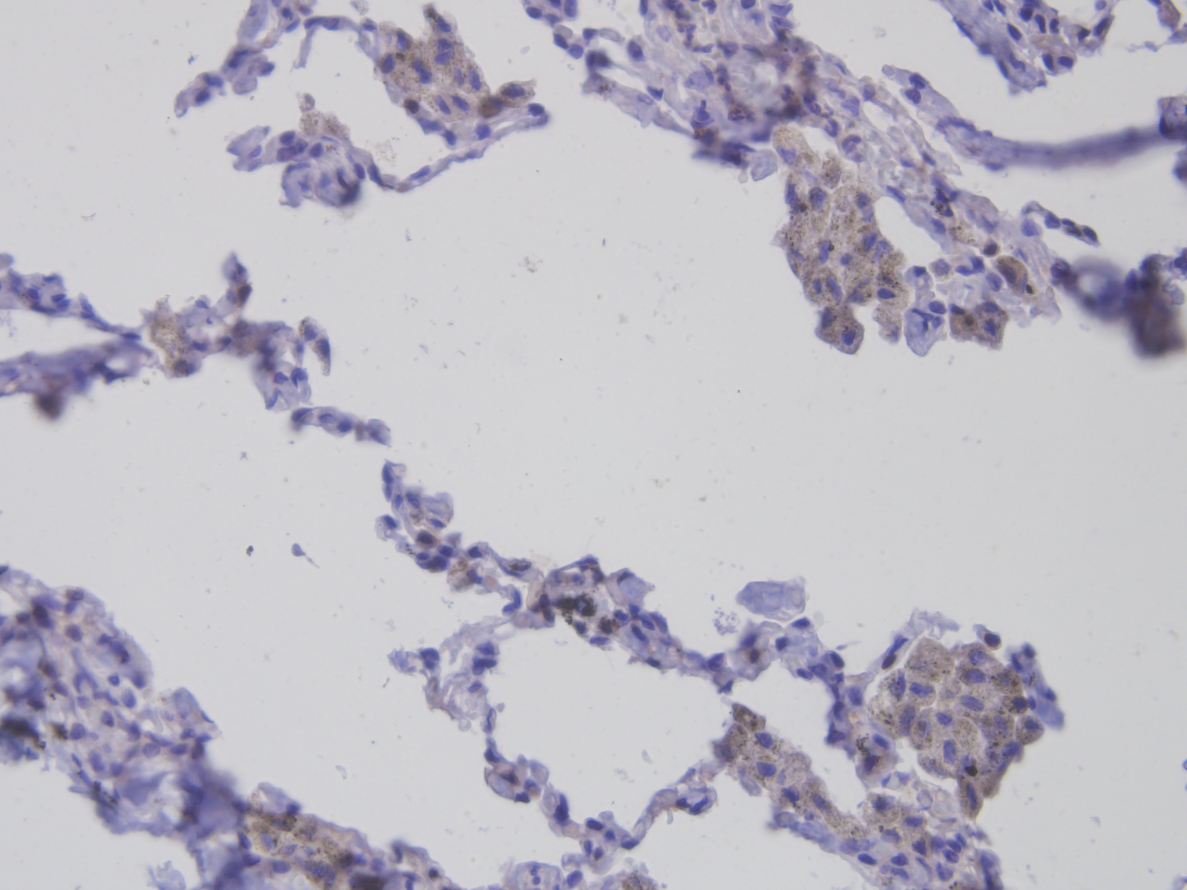

Supplement: S35 File — (ZIP) [file pone.0337223.s036.zip › 486371-400-CA-N/486371-400-N (2).tif]

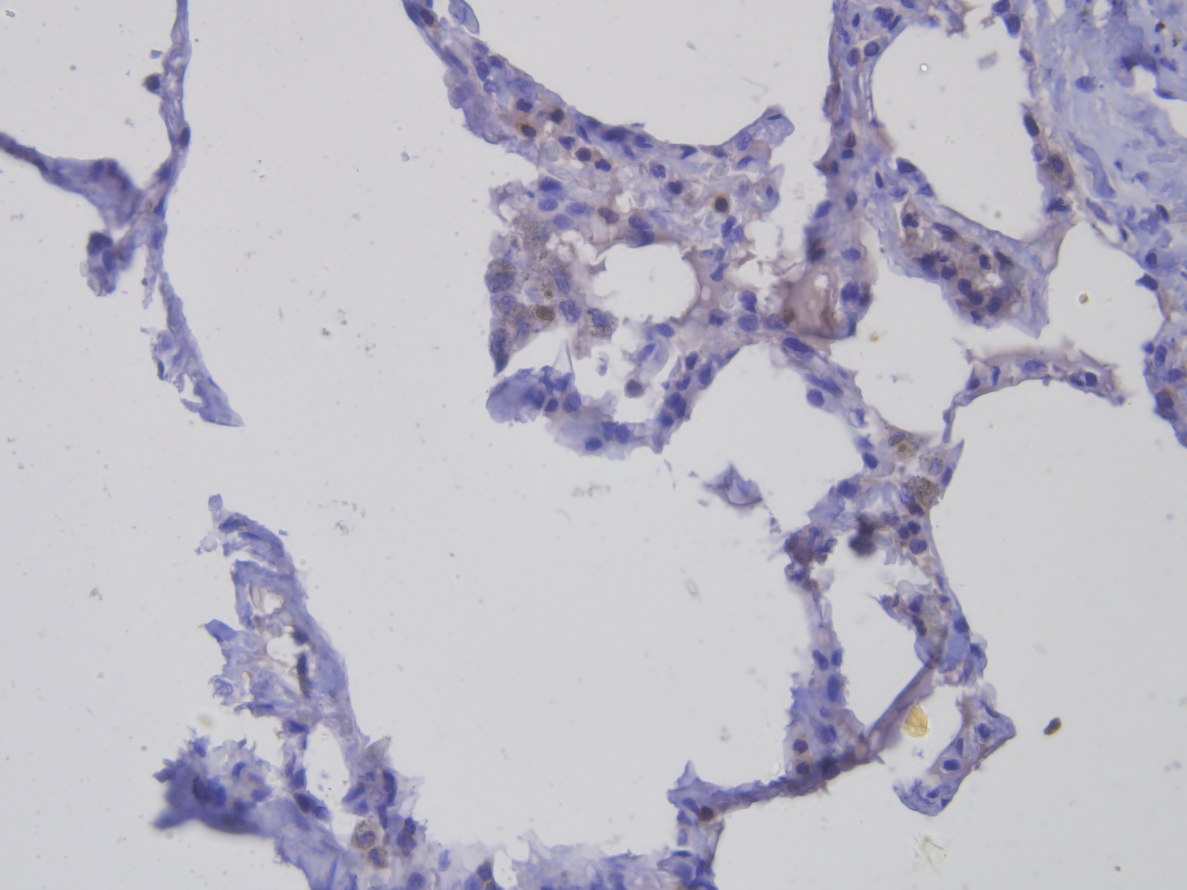

Supplement: S35 File — (ZIP) [file pone.0337223.s036.zip › 486371-400-CA-N/486371-400-N (3).tif]

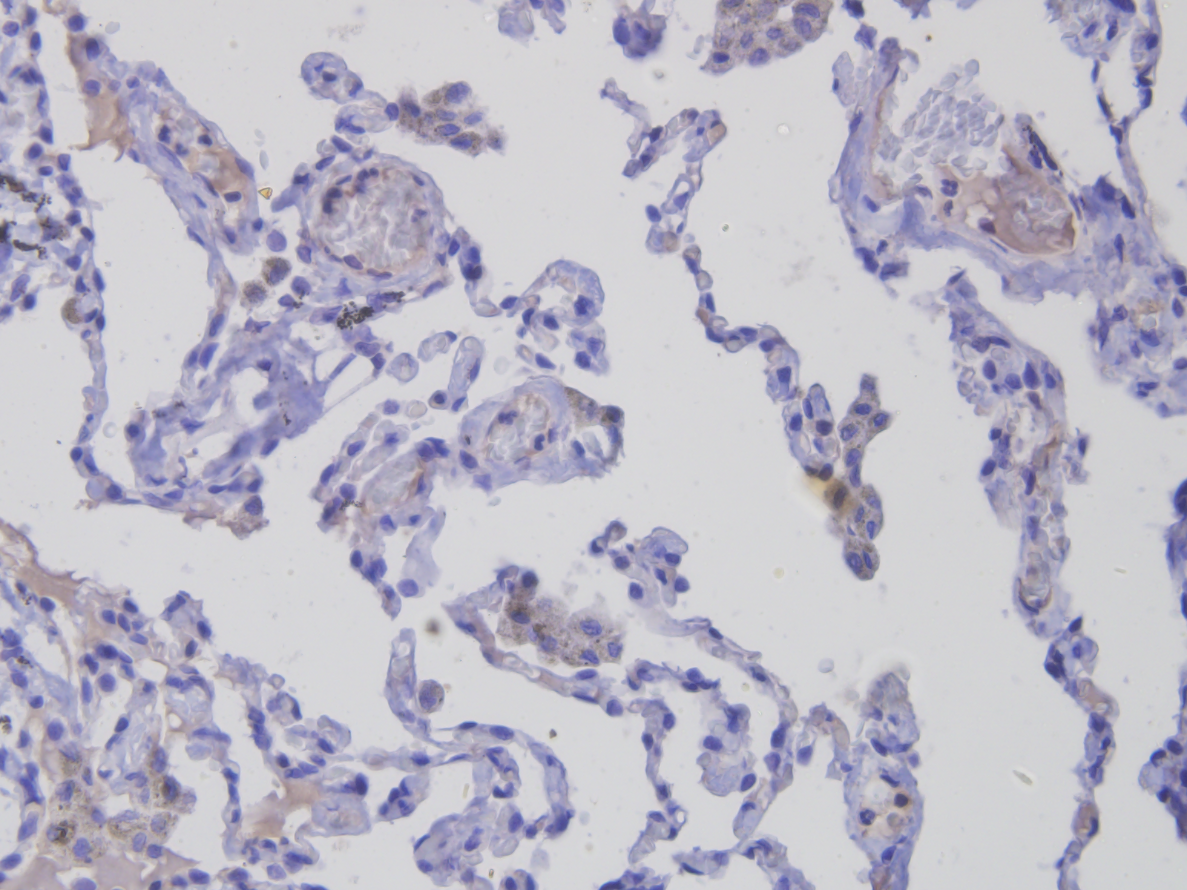

Supplement: S35 File — (ZIP) [file pone.0337223.s036.zip › 486371-400-CA-N/486371-400-N (4).tif]

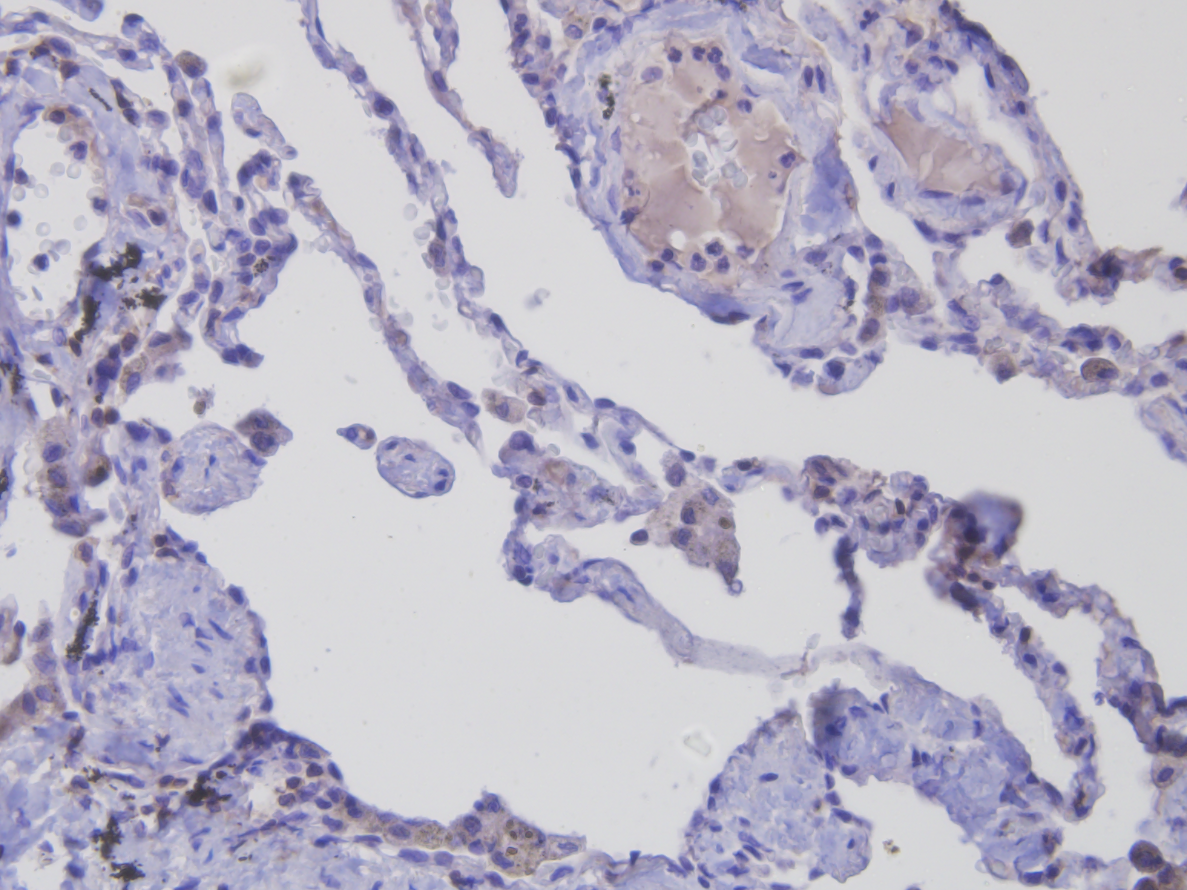

Supplement: S35 File — (ZIP) [file pone.0337223.s036.zip › 486371-400-CA-N/486371-400-N (5).tif]

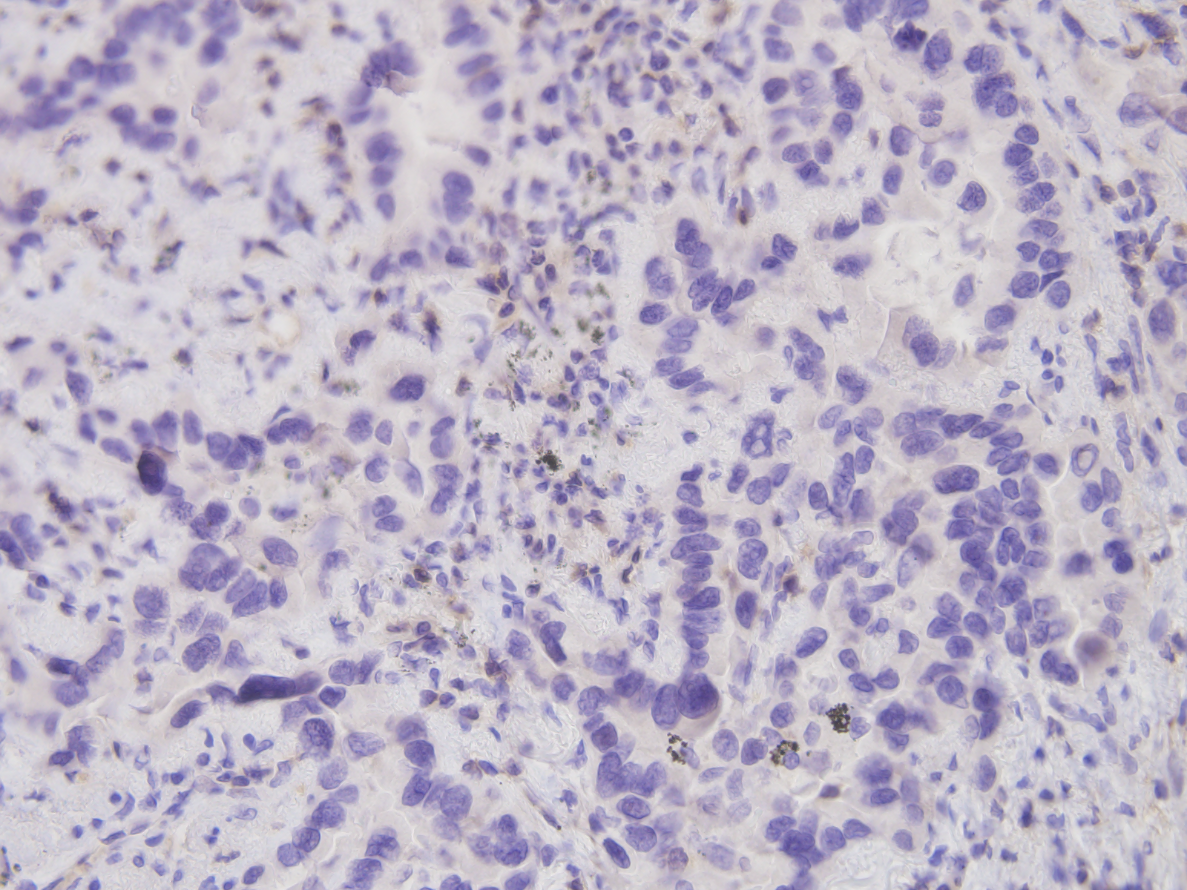

Supplement: S36 File — (ZIP) [file pone.0337223.s037.zip › 487298-400-CA-N/487298-400-CA (1).tif]

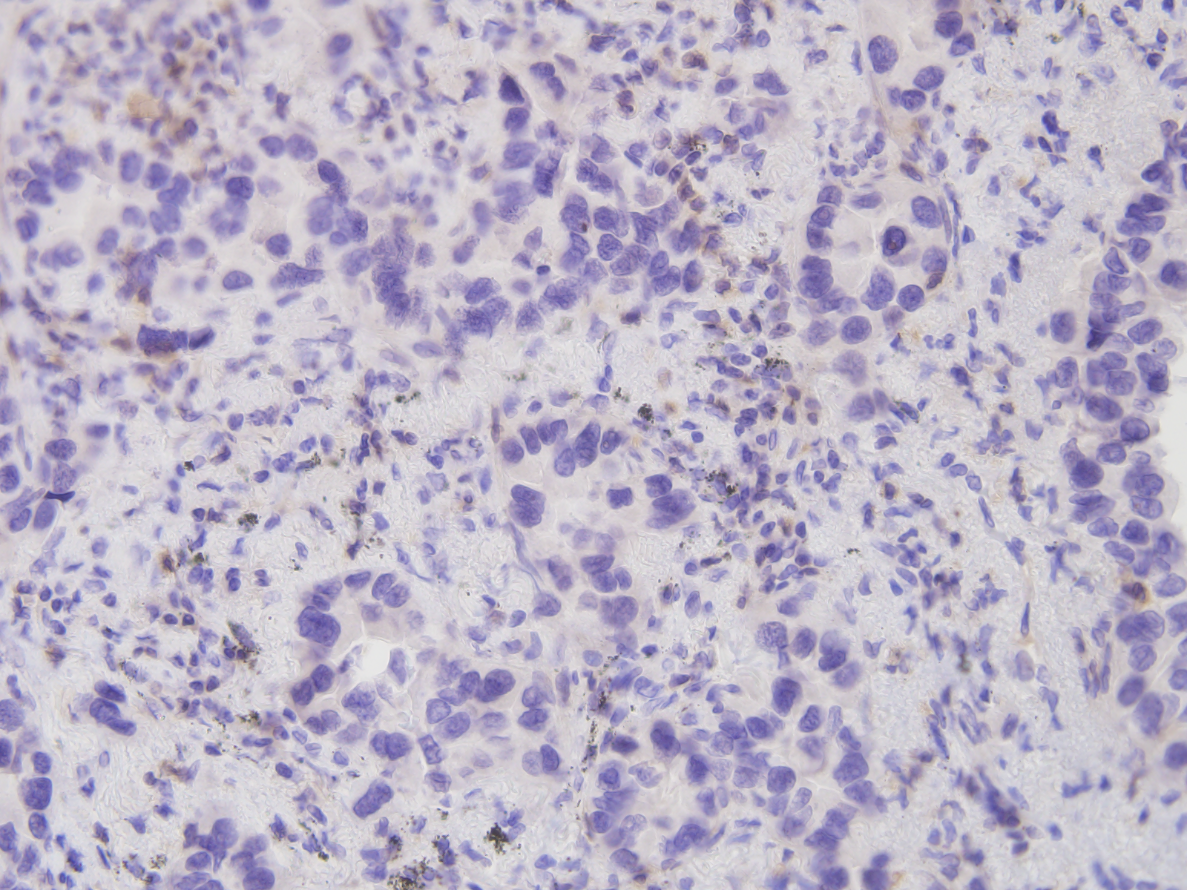

Supplement: S36 File — (ZIP) [file pone.0337223.s037.zip › 487298-400-CA-N/487298-400-CA (2).tif]

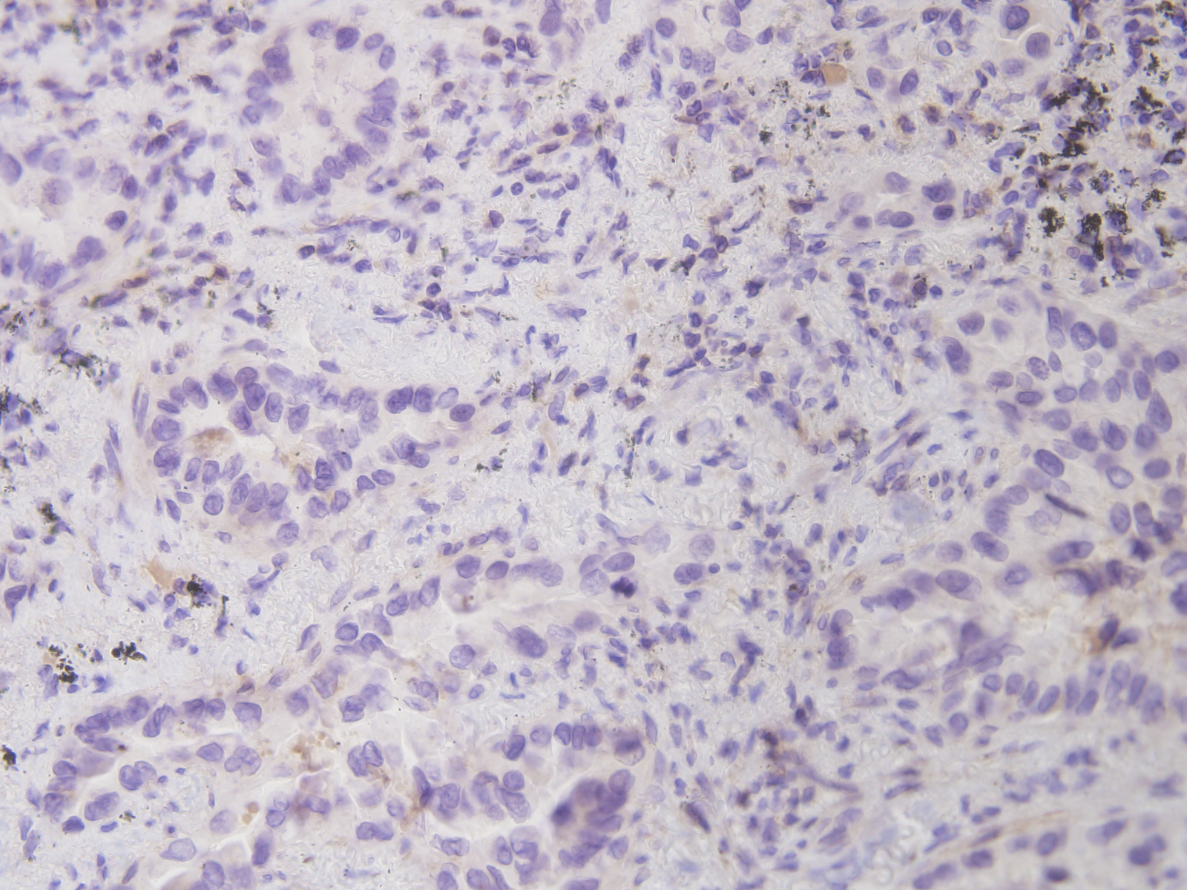

Supplement: S36 File — (ZIP) [file pone.0337223.s037.zip › 487298-400-CA-N/487298-400-CA (3).tif]

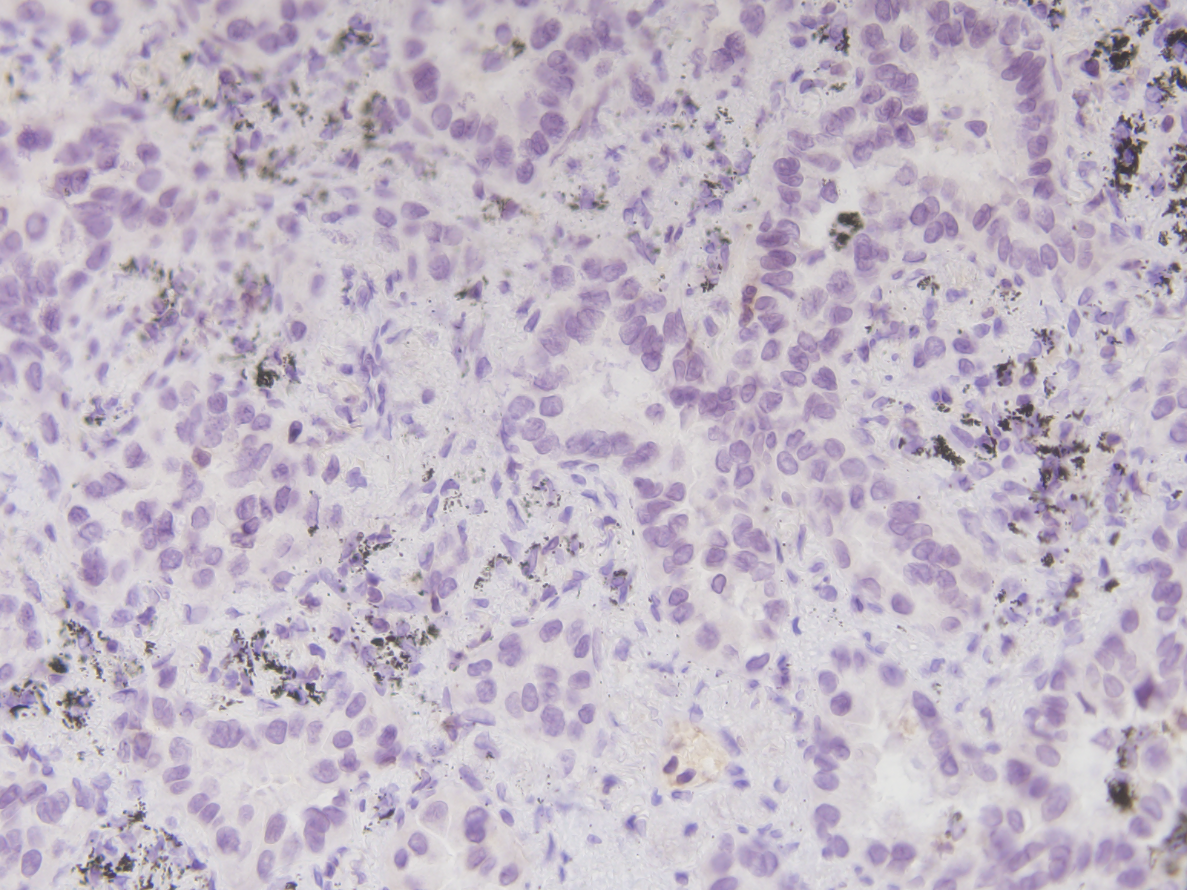

Supplement: S36 File — (ZIP) [file pone.0337223.s037.zip › 487298-400-CA-N/487298-400-CA (4).tif]

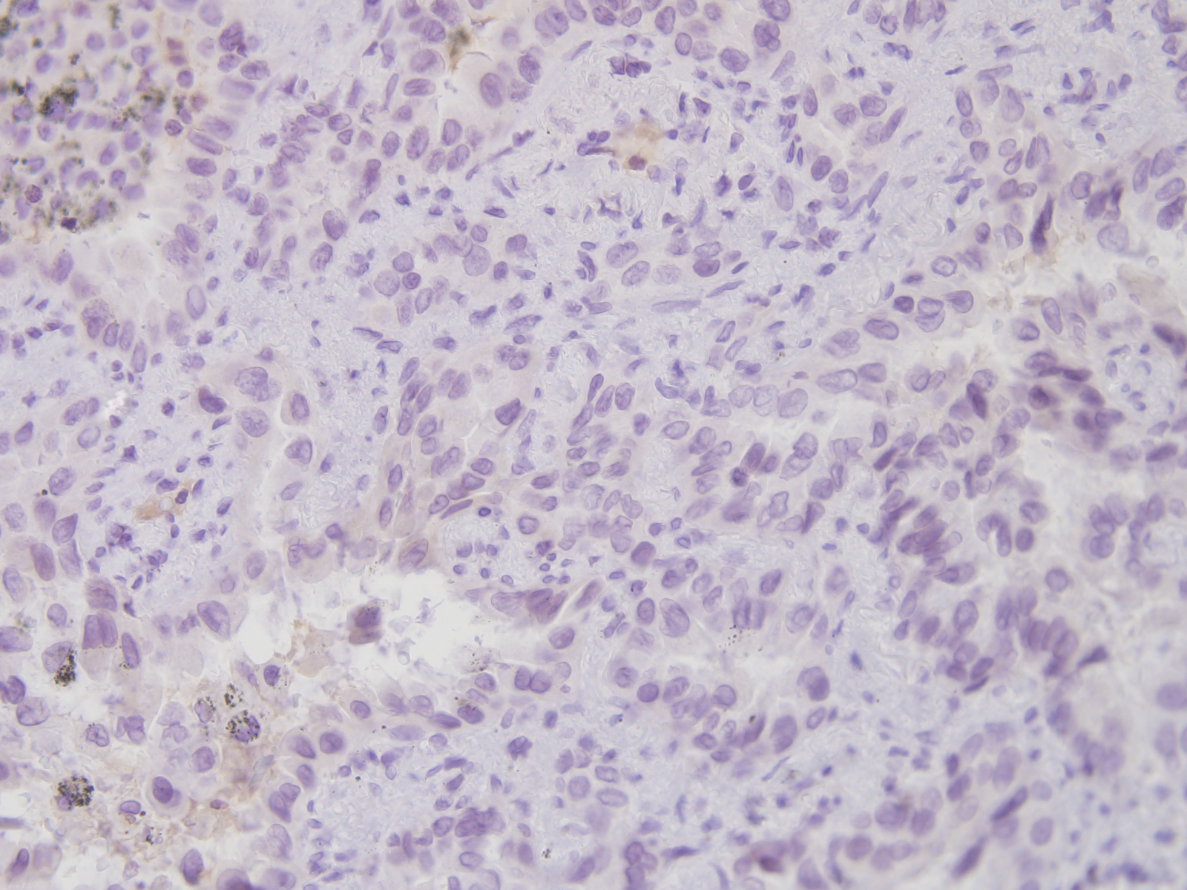

Supplement: S36 File — (ZIP) [file pone.0337223.s037.zip › 487298-400-CA-N/487298-400-CA (5).tif]

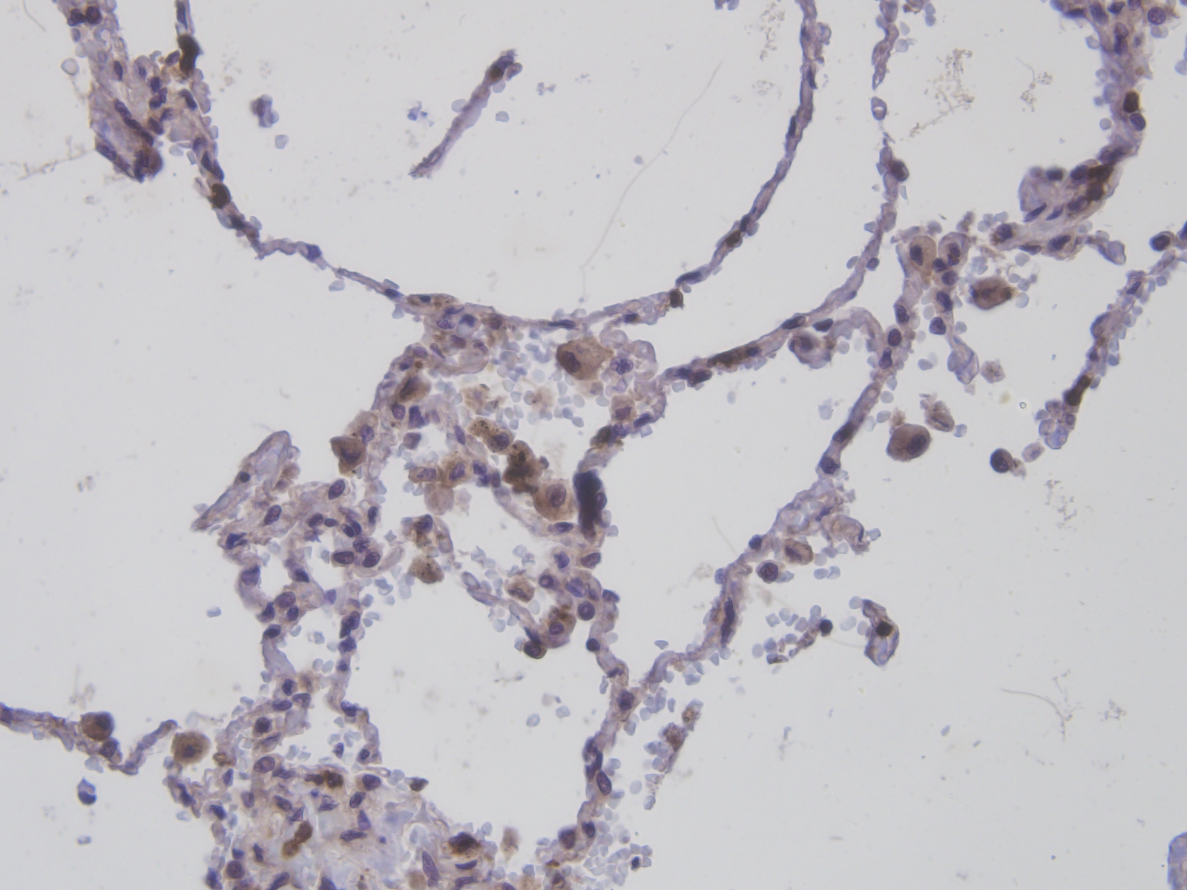

Supplement: S36 File — (ZIP) [file pone.0337223.s037.zip › 487298-400-CA-N/487298-400-N (1).tif]

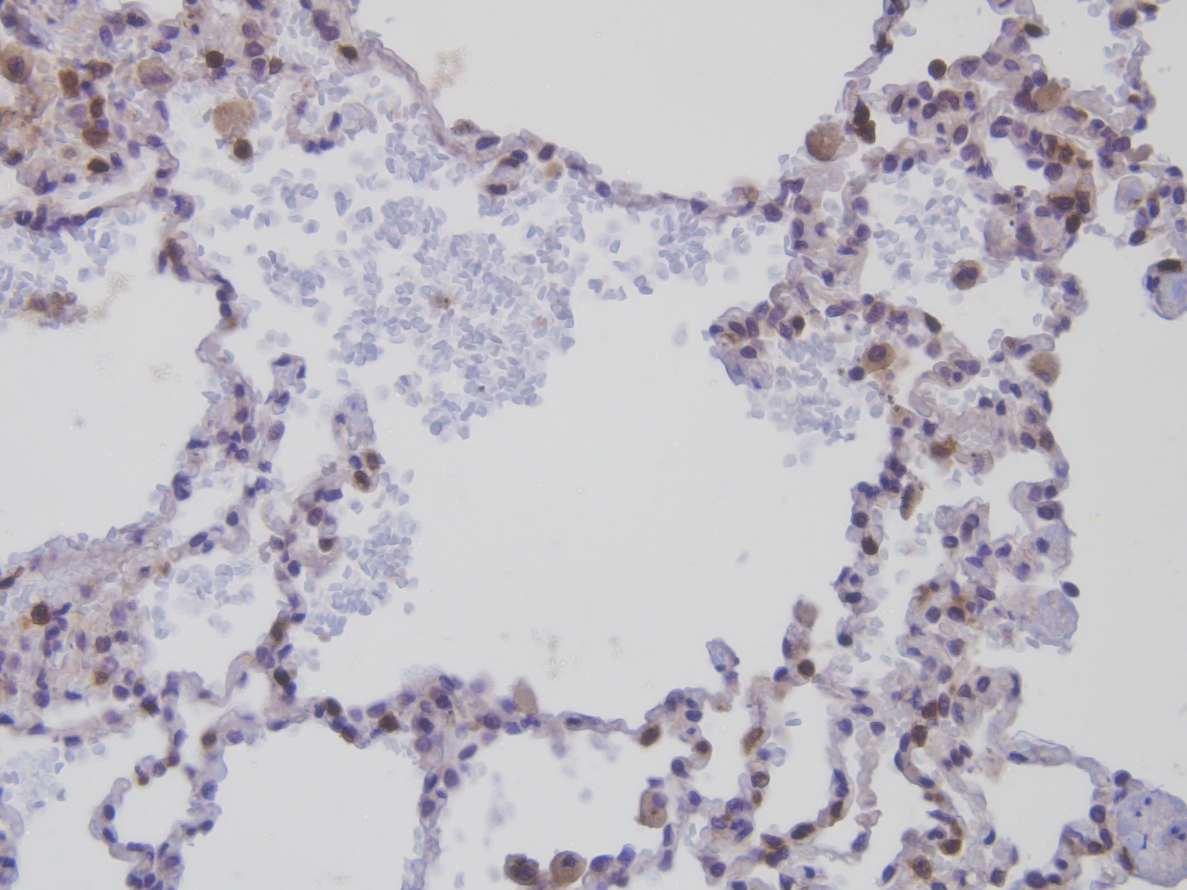

Supplement: S36 File — (ZIP) [file pone.0337223.s037.zip › 487298-400-CA-N/487298-400-N (2).tif]

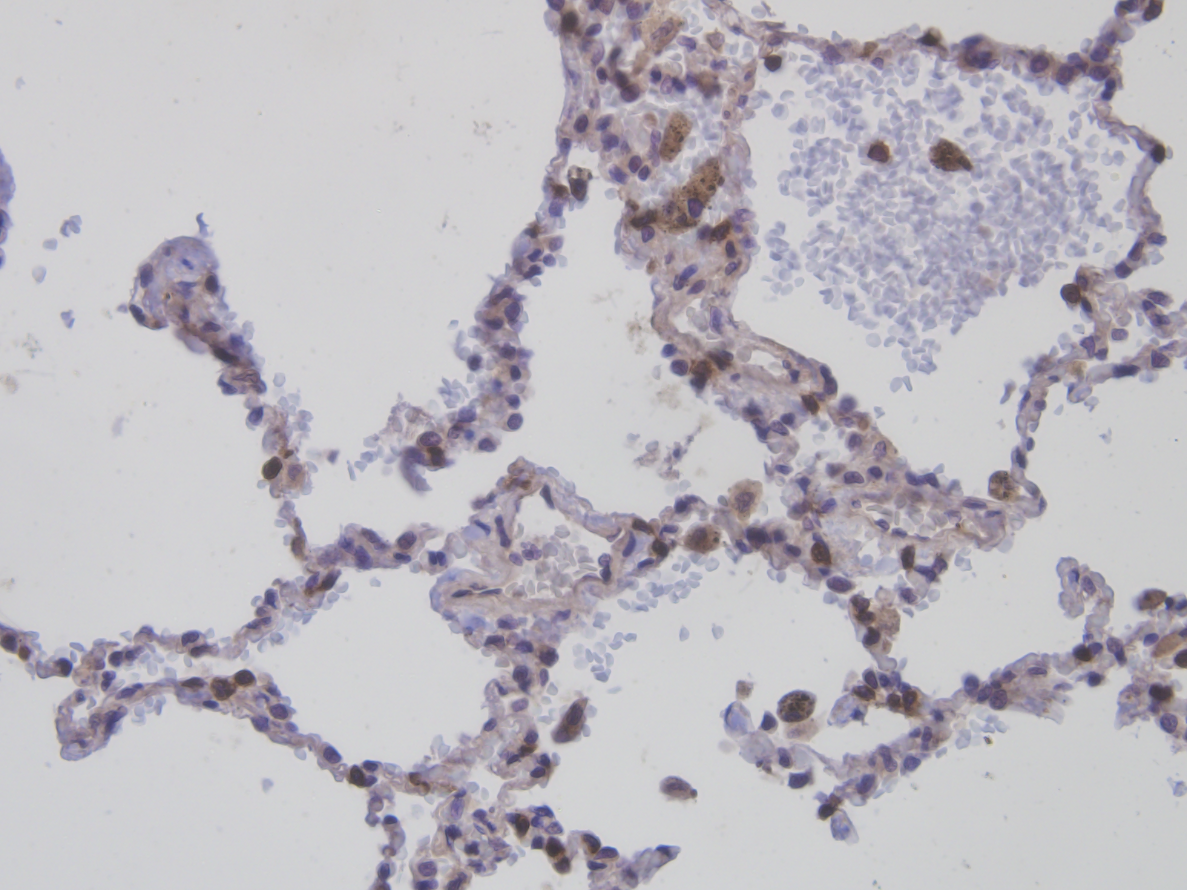

Supplement: S36 File — (ZIP) [file pone.0337223.s037.zip › 487298-400-CA-N/487298-400-N (3).tif]

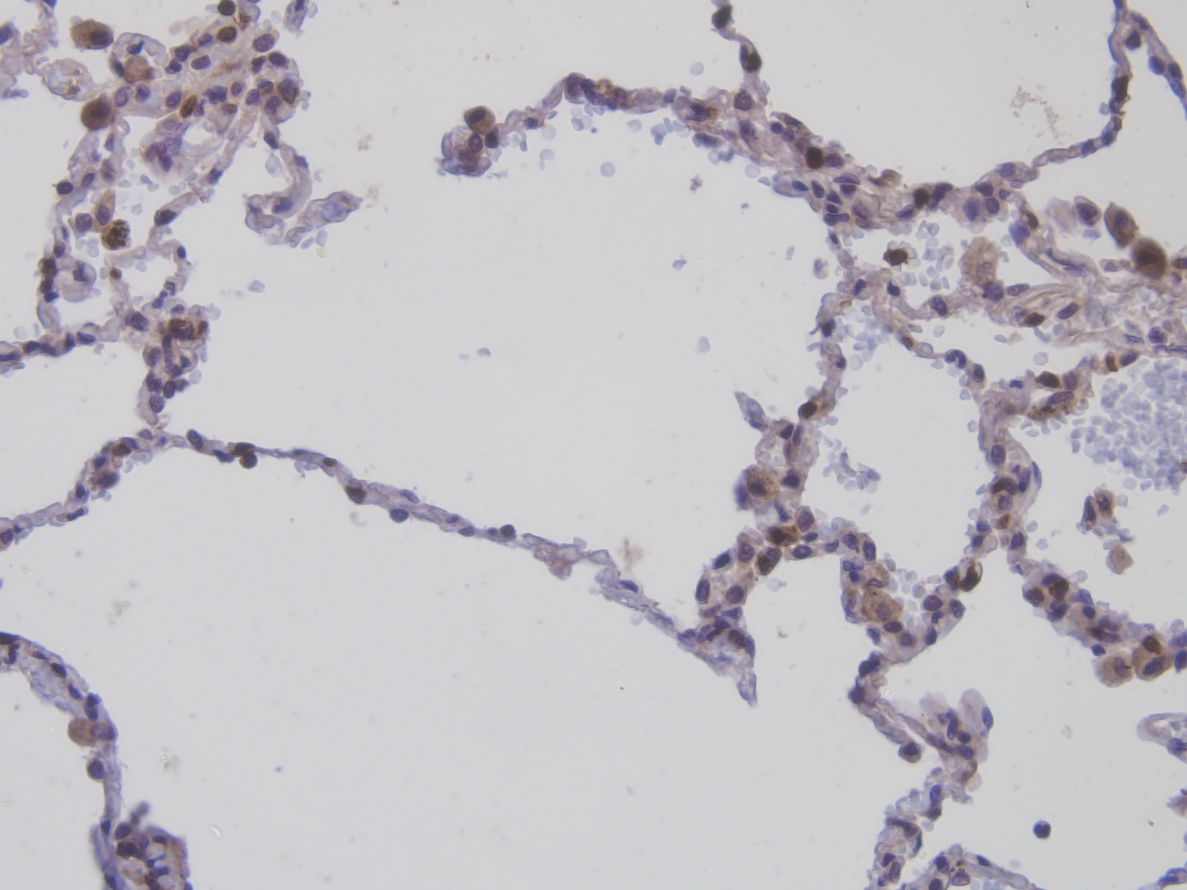

Supplement: S36 File — (ZIP) [file pone.0337223.s037.zip › 487298-400-CA-N/487298-400-N (4).tif]

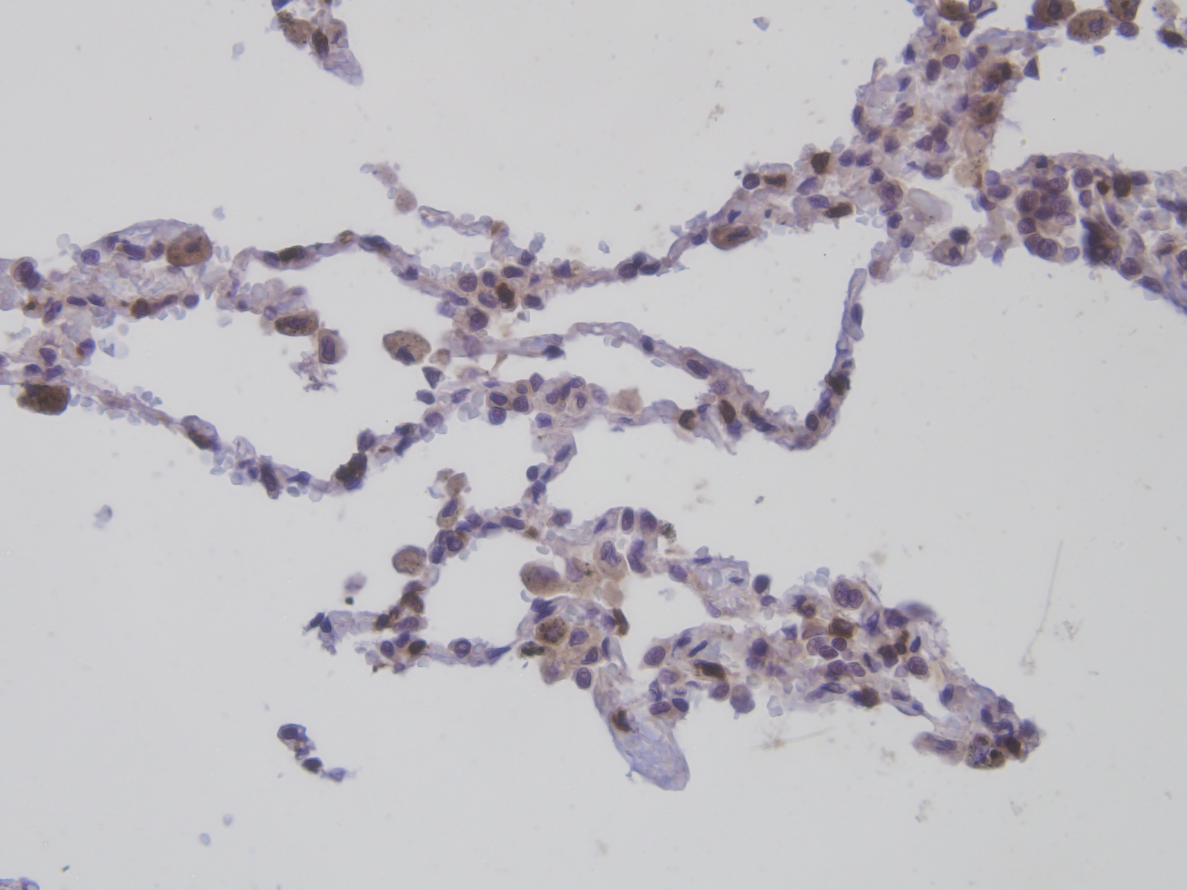

Supplement: S36 File — (ZIP) [file pone.0337223.s037.zip › 487298-400-CA-N/487298-400-N (5).tif]

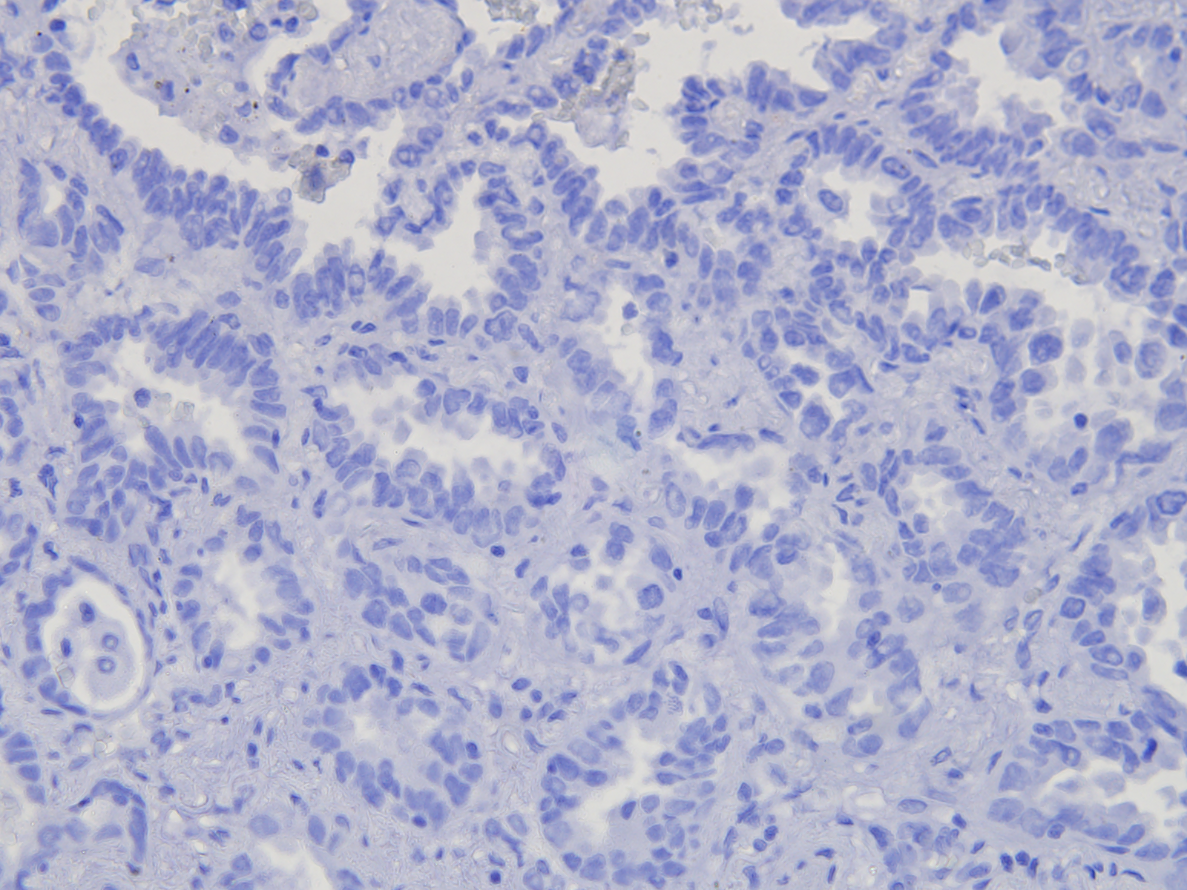

Supplement: S37 File — (ZIP) [file pone.0337223.s038.zip › 488524-400X-CA-N/488524-ca (1).tif]

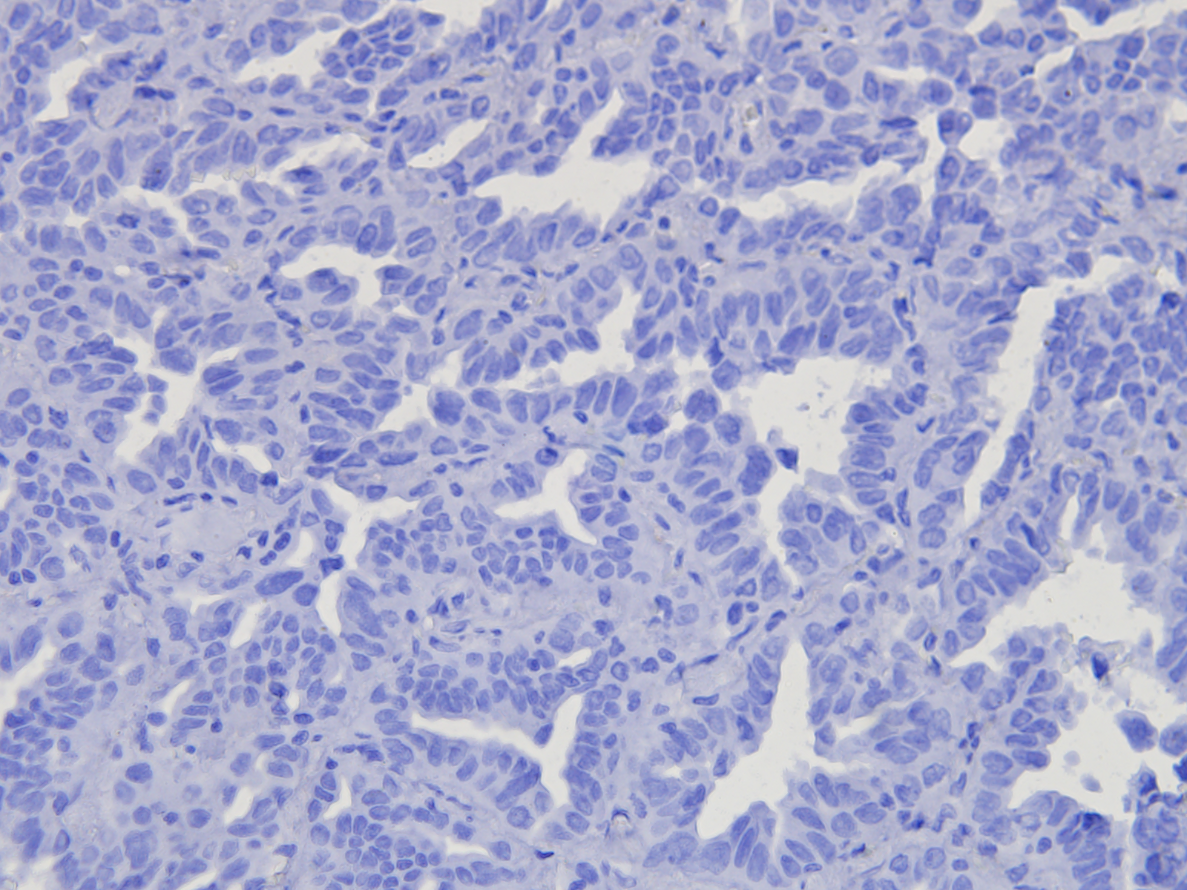

Supplement: S37 File — (ZIP) [file pone.0337223.s038.zip › 488524-400X-CA-N/488524-ca (2).tif]

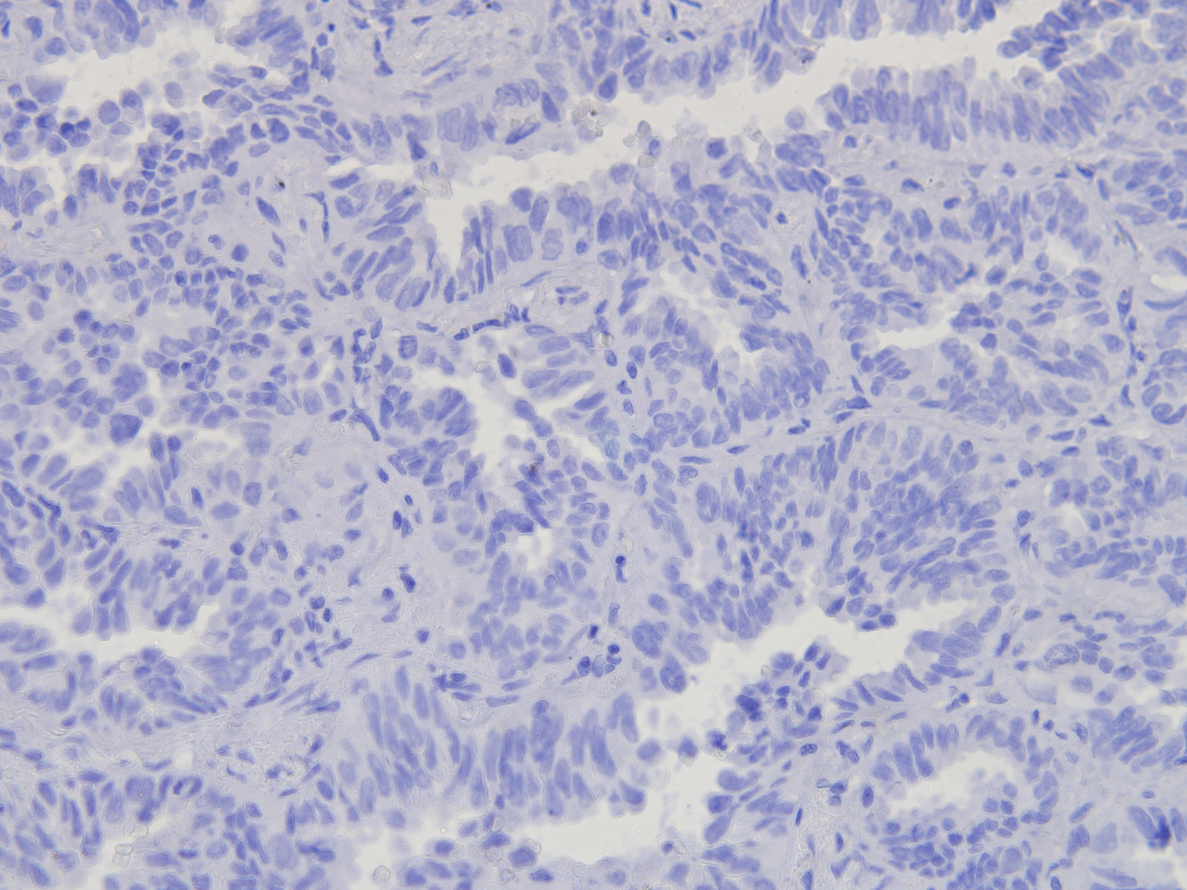

Supplement: S37 File — (ZIP) [file pone.0337223.s038.zip › 488524-400X-CA-N/488524-ca (3).tif]

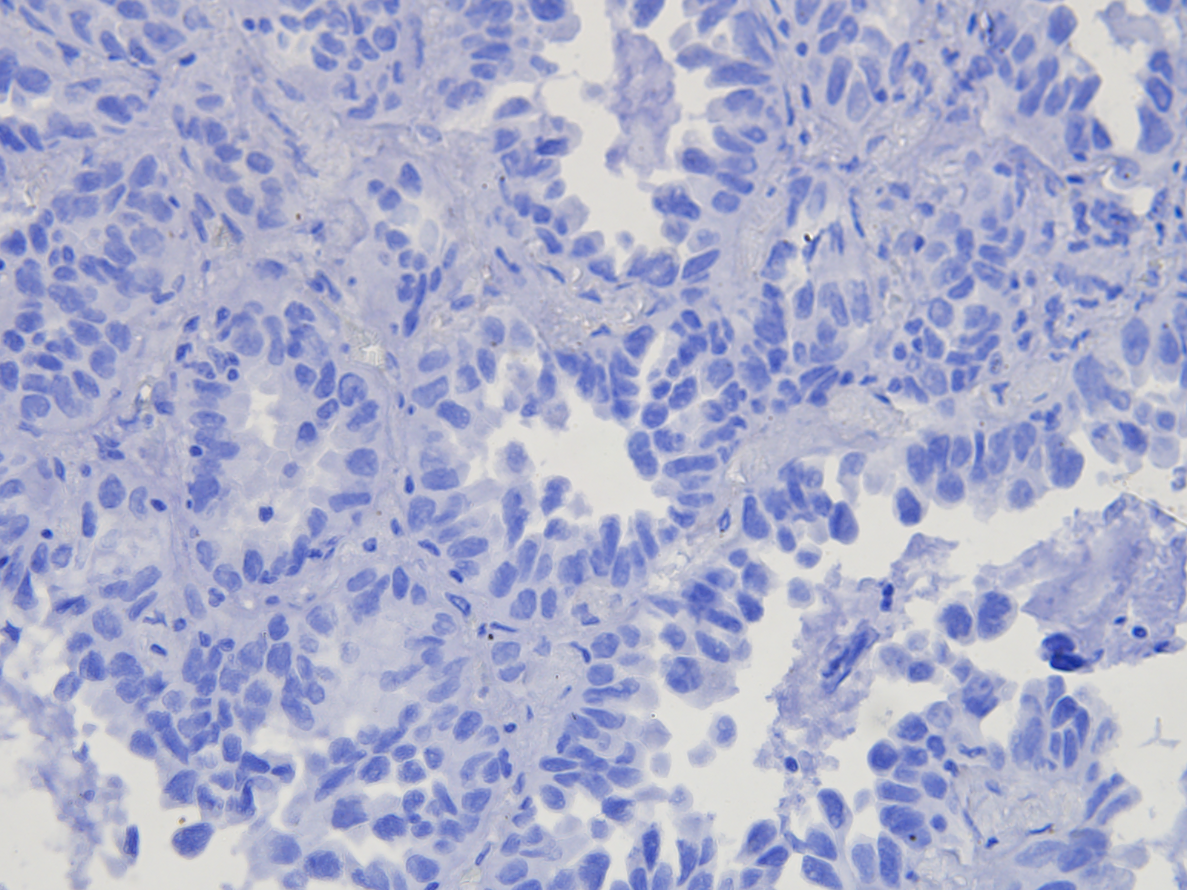

Supplement: S37 File — (ZIP) [file pone.0337223.s038.zip › 488524-400X-CA-N/488524-ca (4).tif]

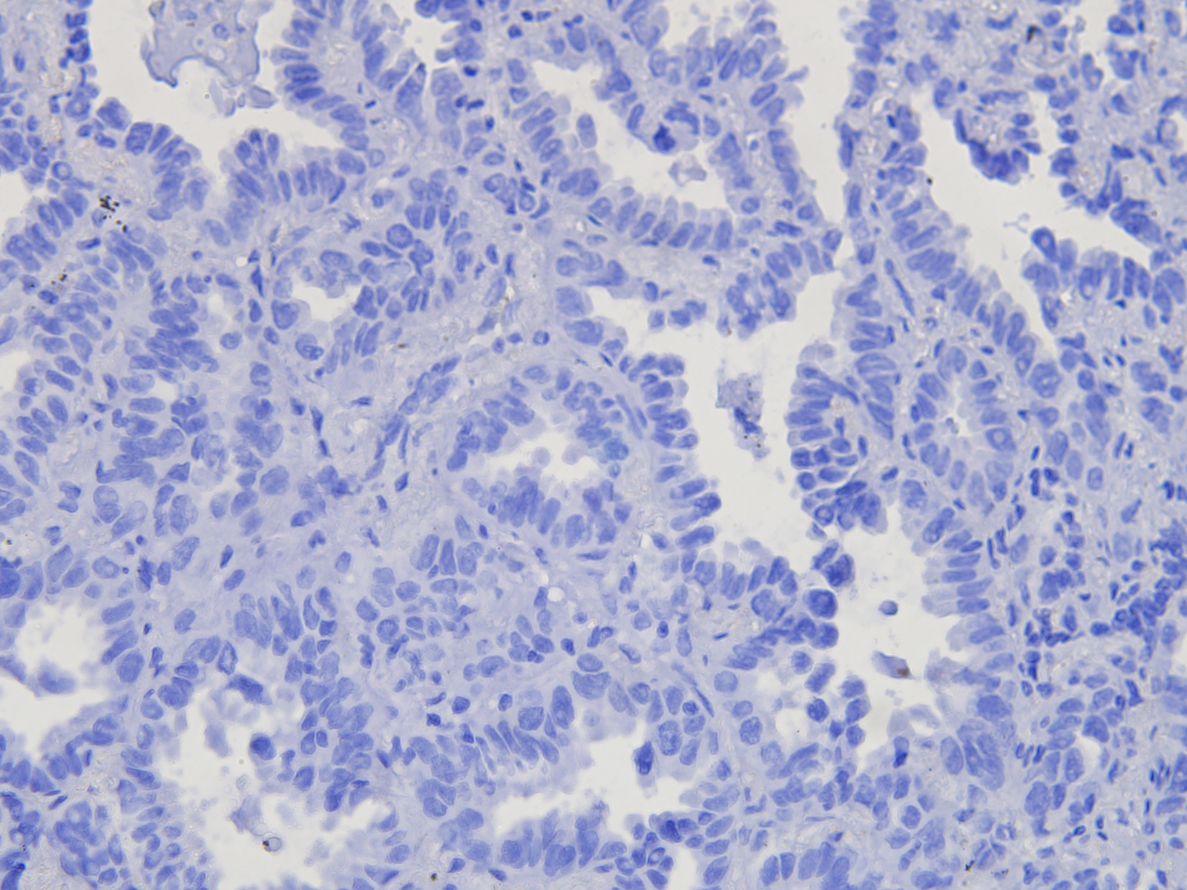

Supplement: S37 File — (ZIP) [file pone.0337223.s038.zip › 488524-400X-CA-N/488524-ca (5).tif]

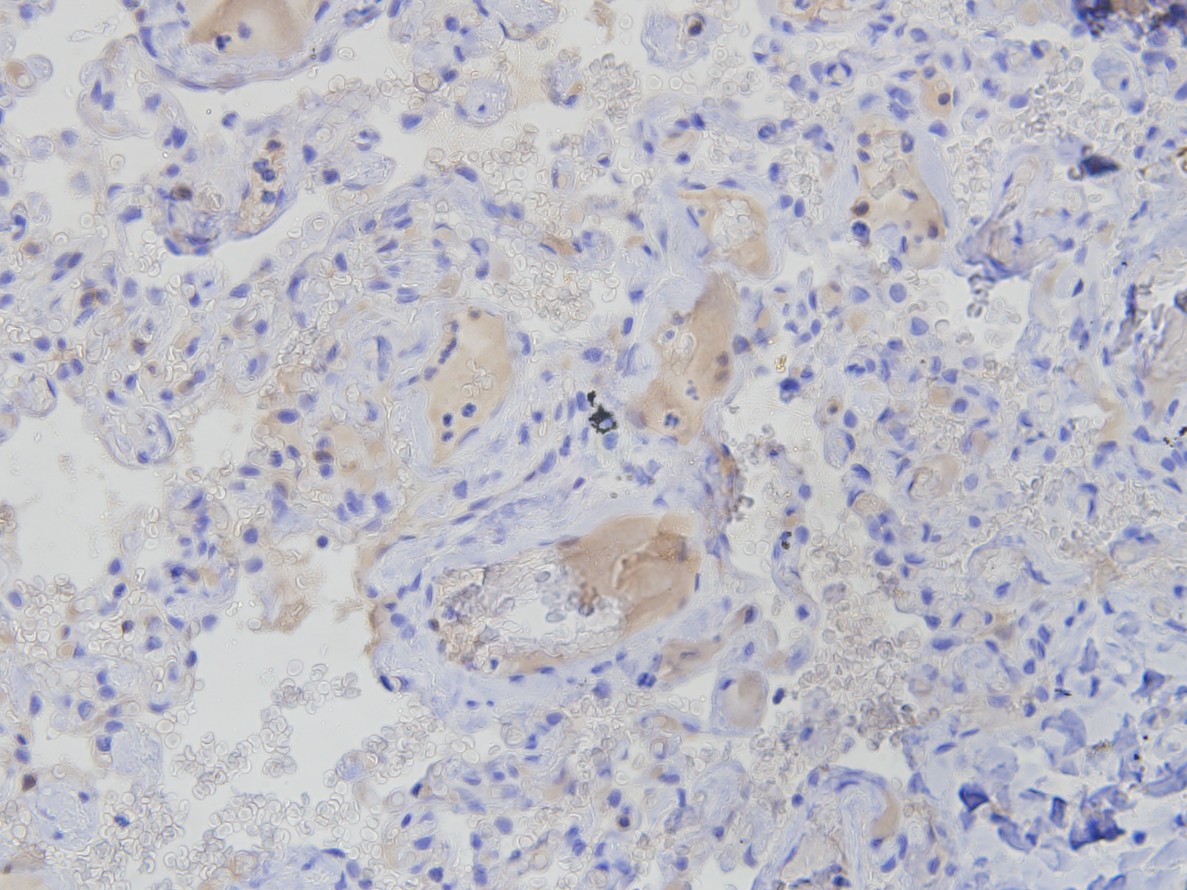

Supplement: S37 File — (ZIP) [file pone.0337223.s038.zip › 488524-400X-CA-N/488524-n (1).tif]

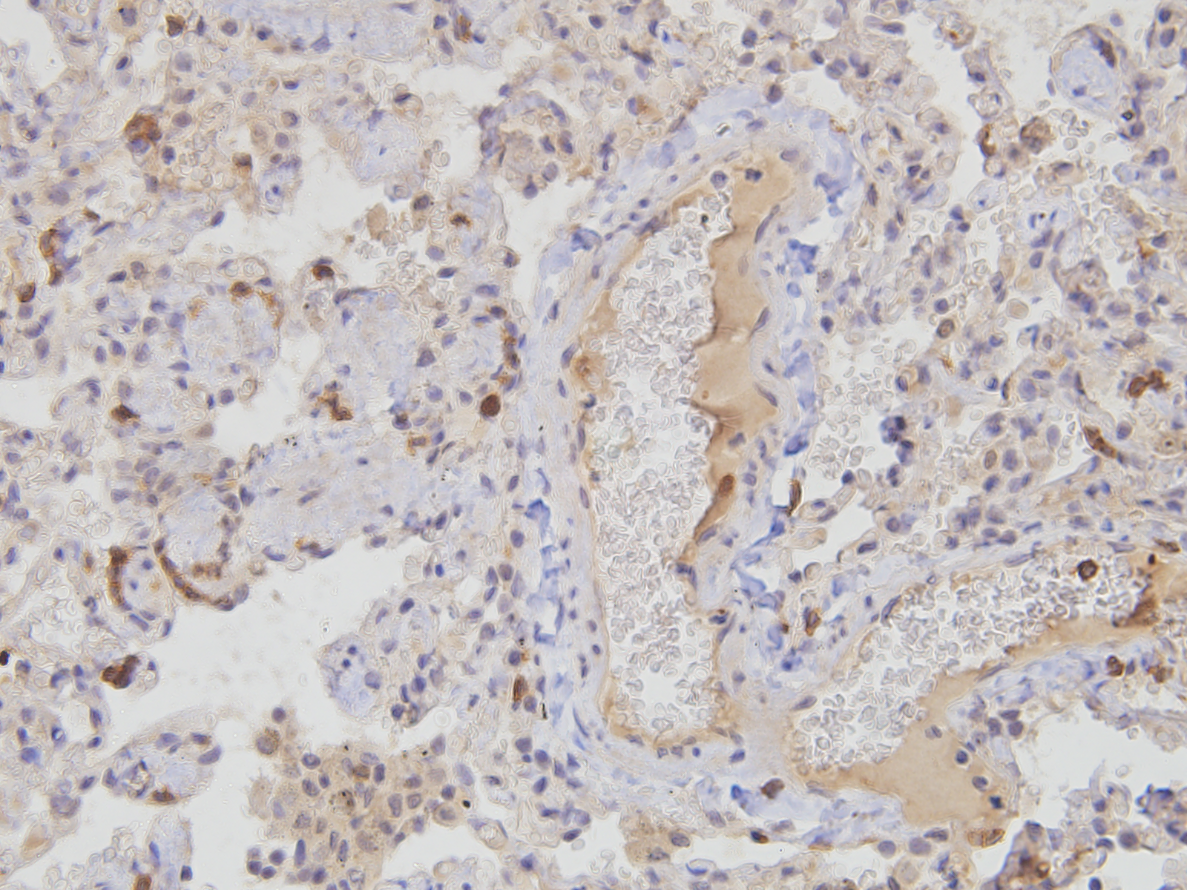

Supplement: S37 File — (ZIP) [file pone.0337223.s038.zip › 488524-400X-CA-N/488524-n (2).tif]

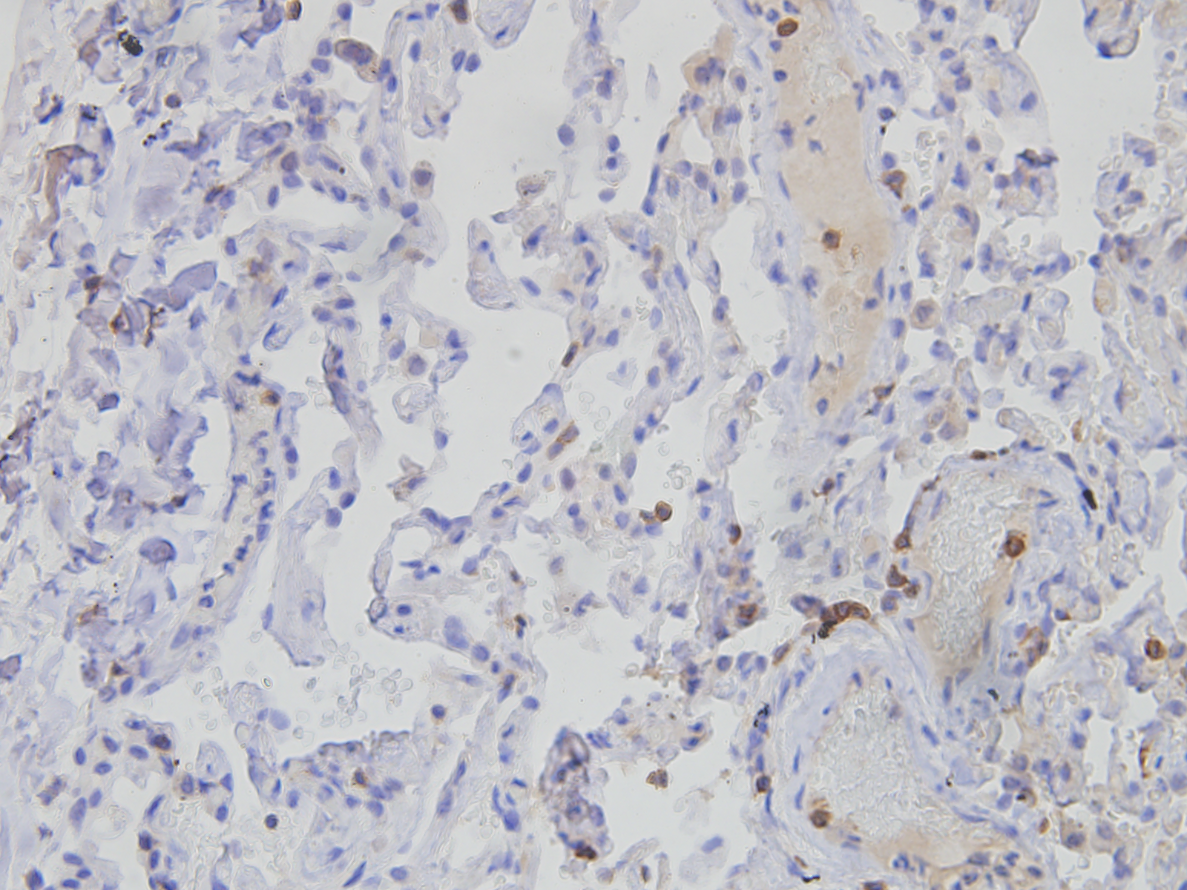

Supplement: S37 File — (ZIP) [file pone.0337223.s038.zip › 488524-400X-CA-N/488524-n (3).tif]

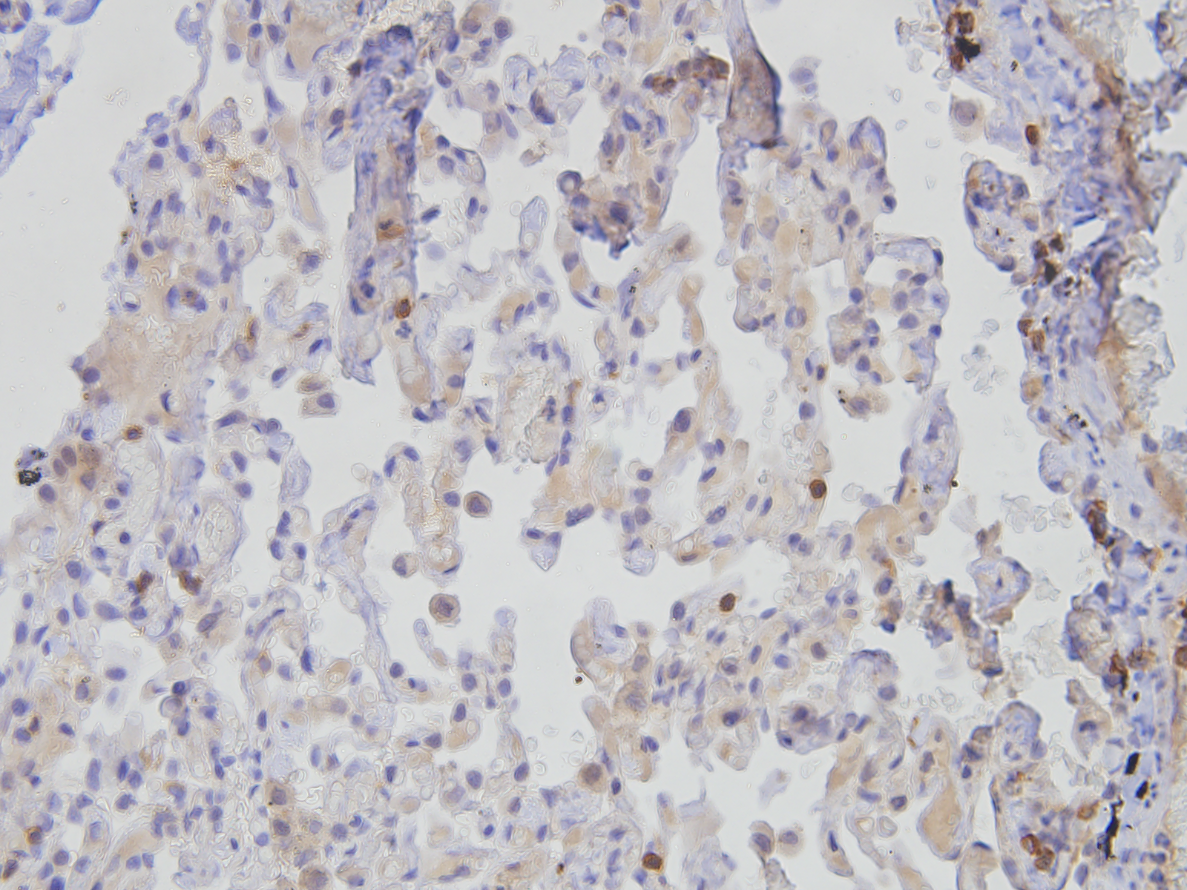

Supplement: S37 File — (ZIP) [file pone.0337223.s038.zip › 488524-400X-CA-N/488524-n (4).tif]

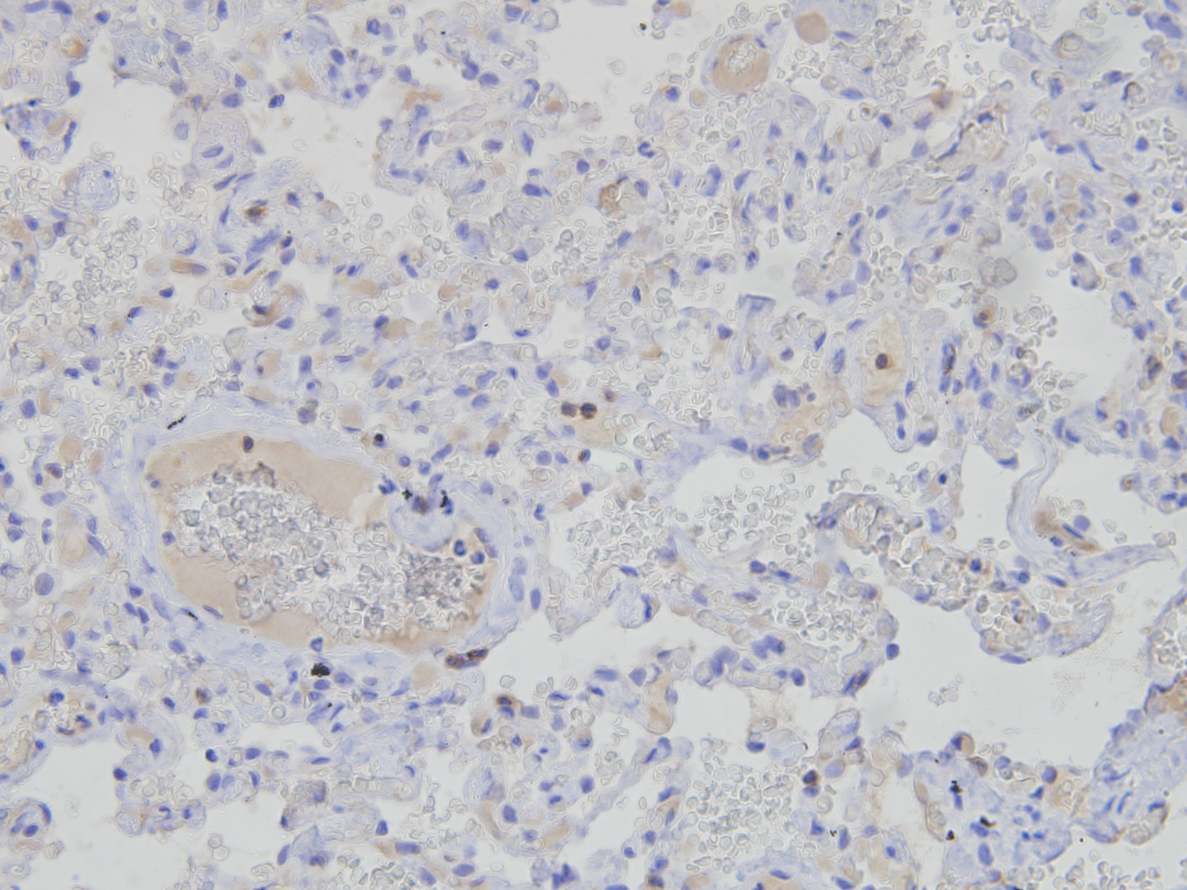

Supplement: S37 File — (ZIP) [file pone.0337223.s038.zip › 488524-400X-CA-N/488524-n (5).tif]

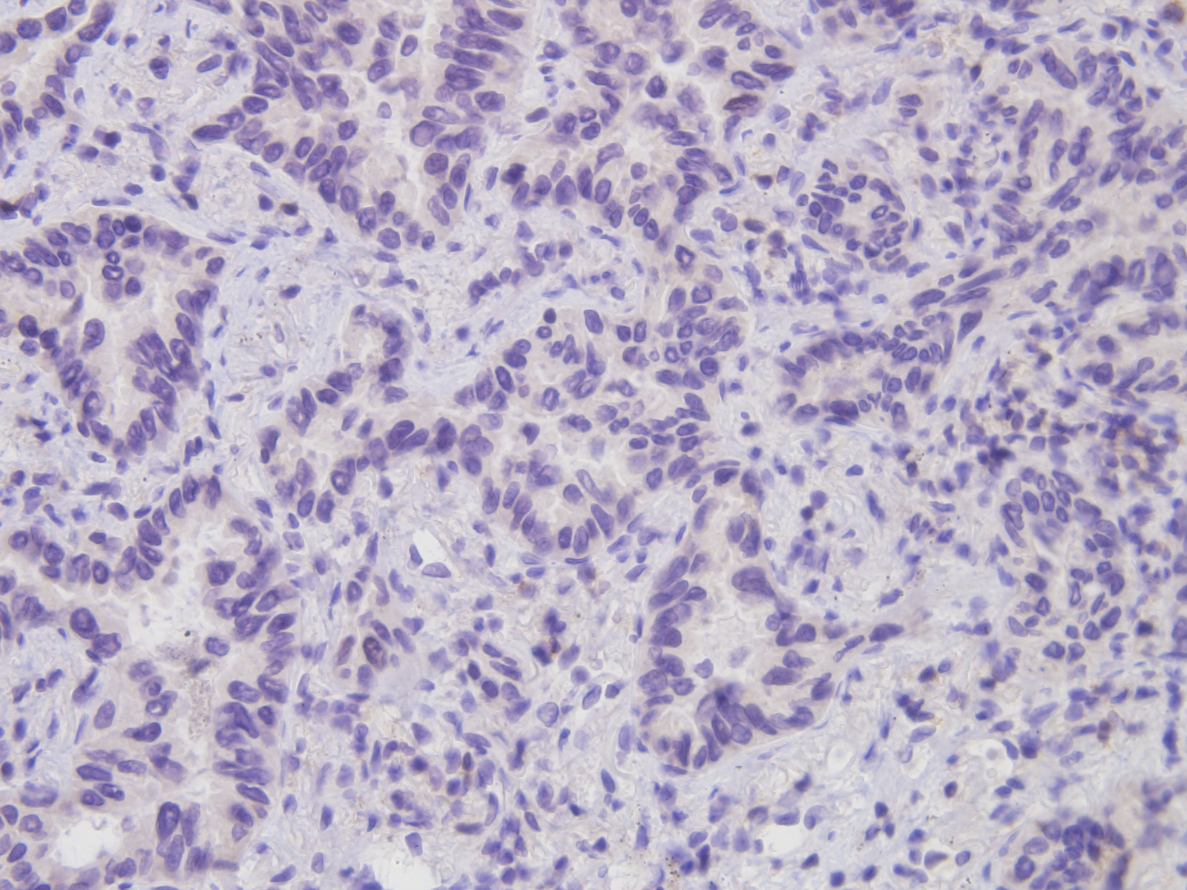

Supplement: S38 File — (ZIP) [file pone.0337223.s039.zip › 488702-400X-N-CA/488702-400X-CA (1).tif]

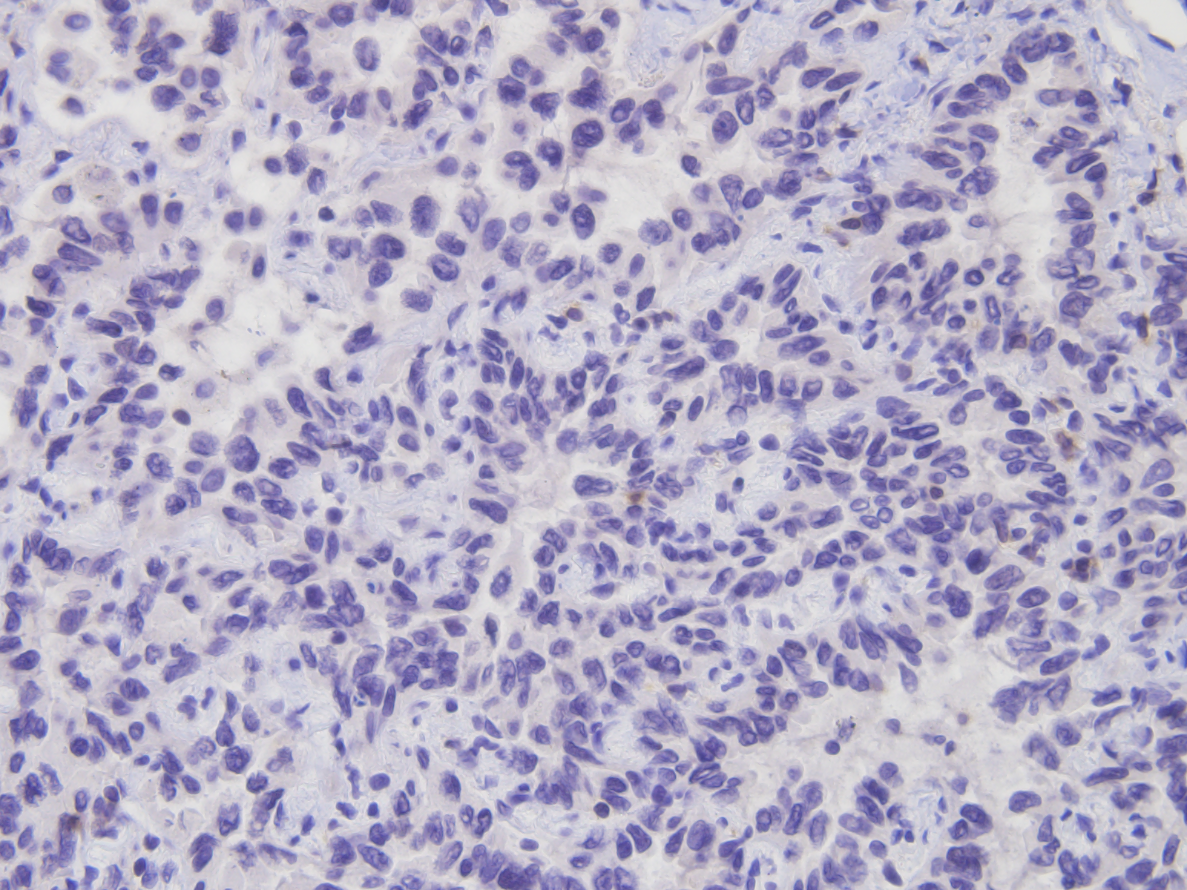

Supplement: S38 File — (ZIP) [file pone.0337223.s039.zip › 488702-400X-N-CA/488702-400X-CA (2).tif]

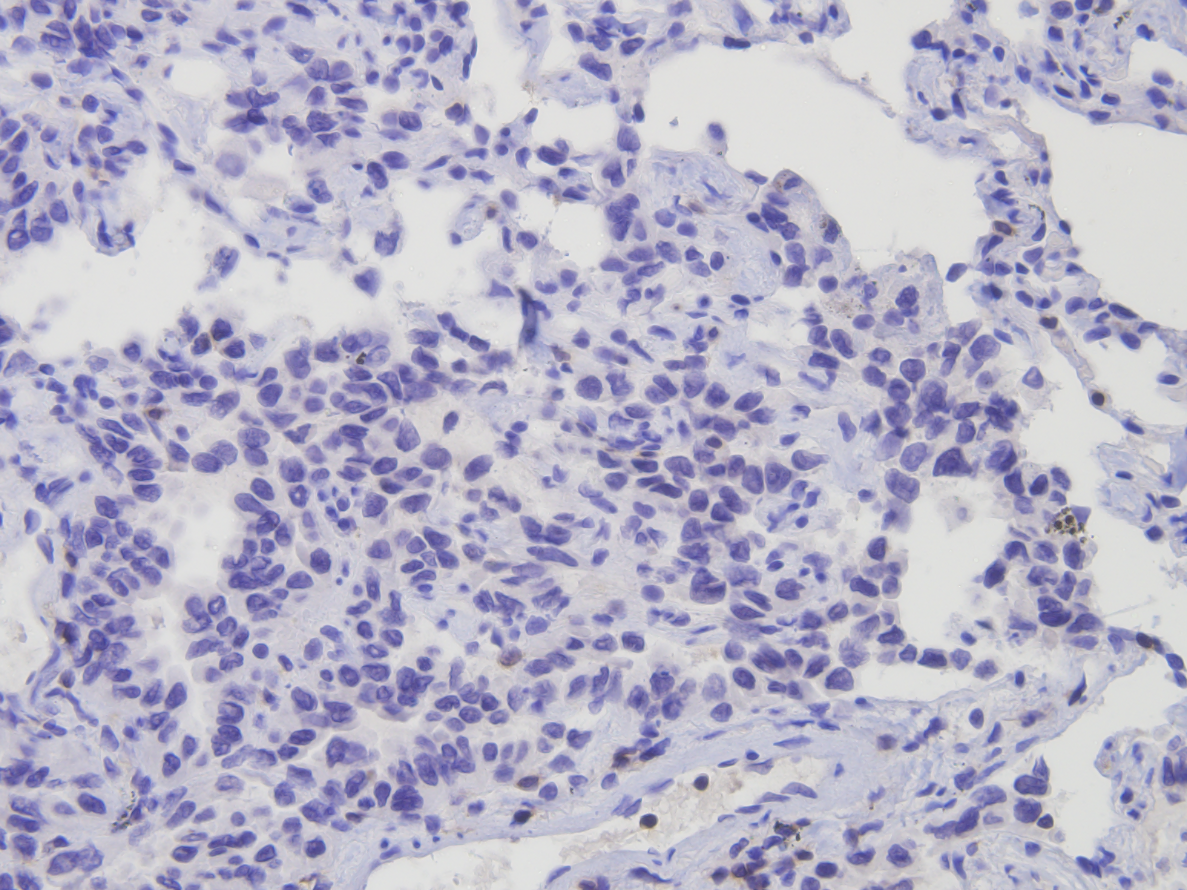

Supplement: S38 File — (ZIP) [file pone.0337223.s039.zip › 488702-400X-N-CA/488702-400X-CA (3).tif]

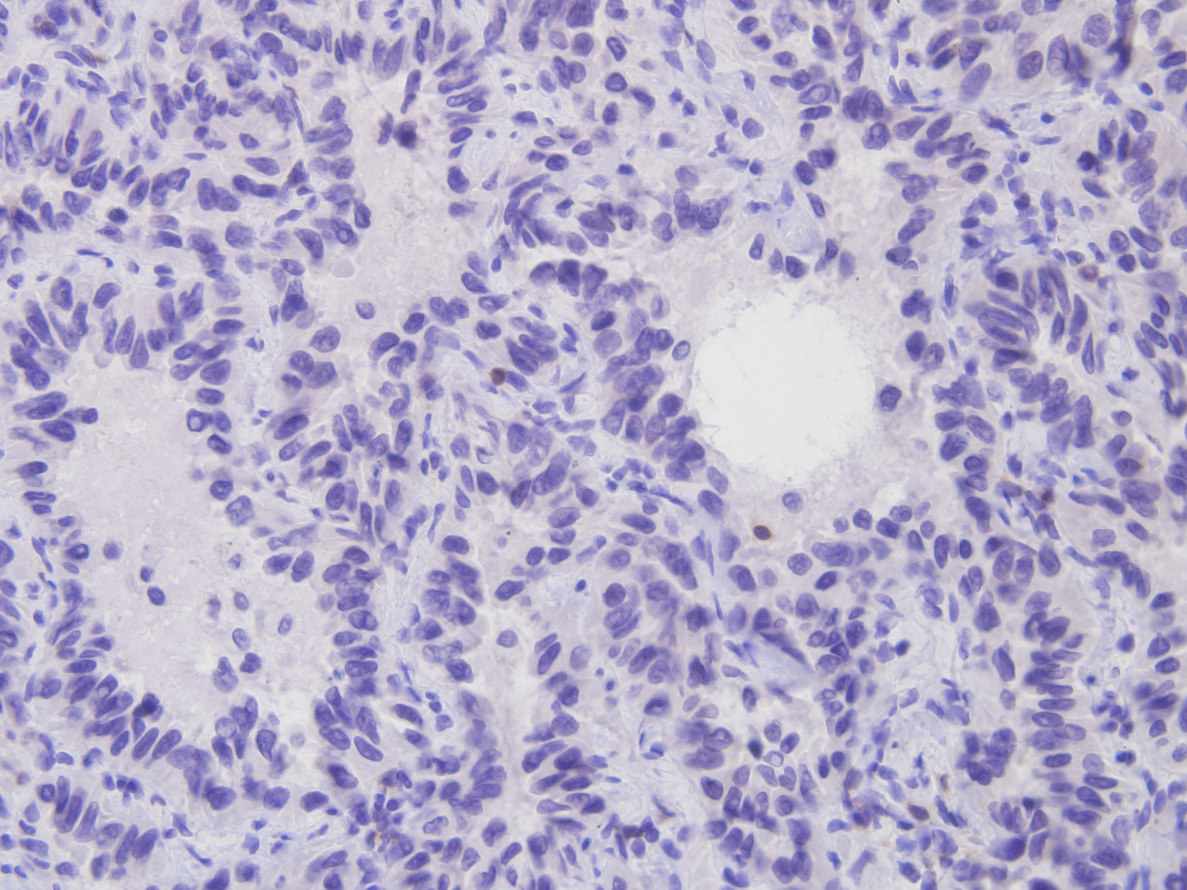

Supplement: S38 File — (ZIP) [file pone.0337223.s039.zip › 488702-400X-N-CA/488702-400X-CA (4).tif]

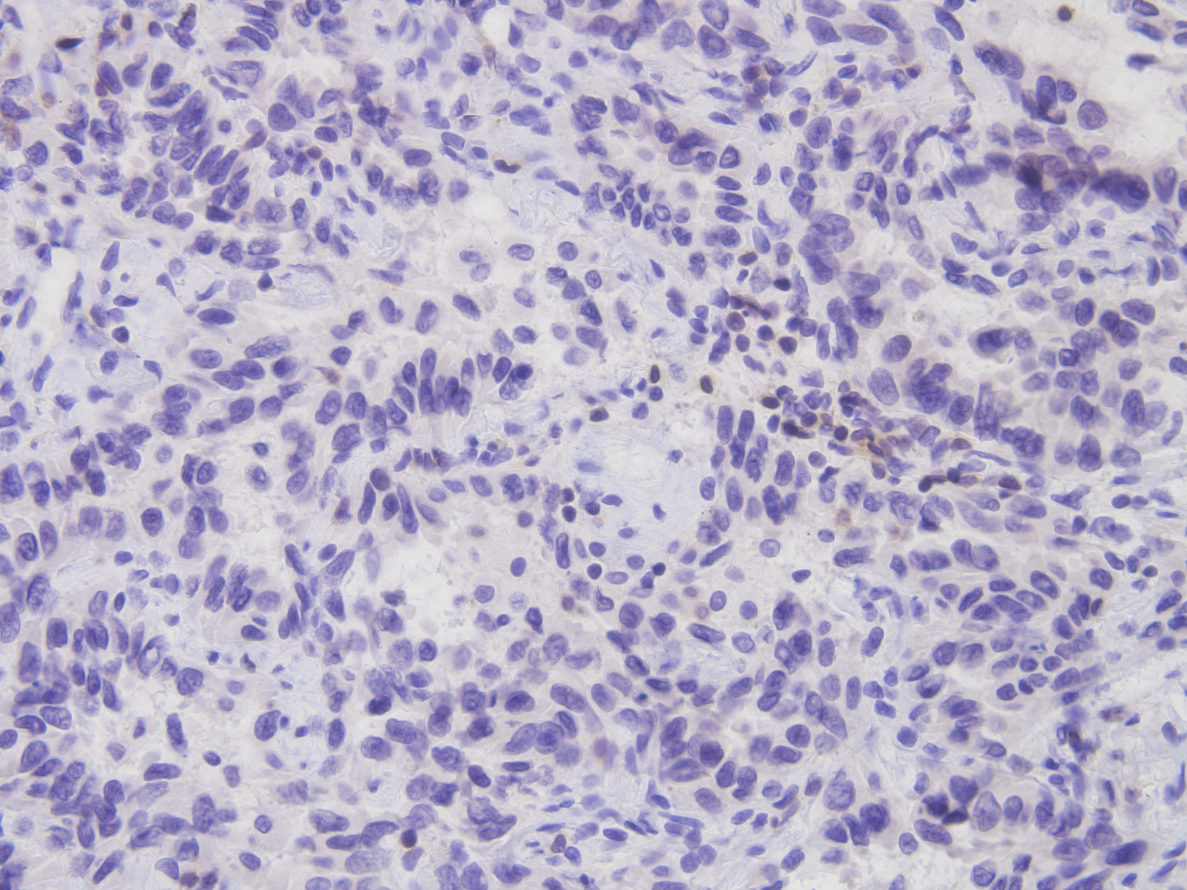

Supplement: S38 File — (ZIP) [file pone.0337223.s039.zip › 488702-400X-N-CA/488702-400X-CA (5).tif]

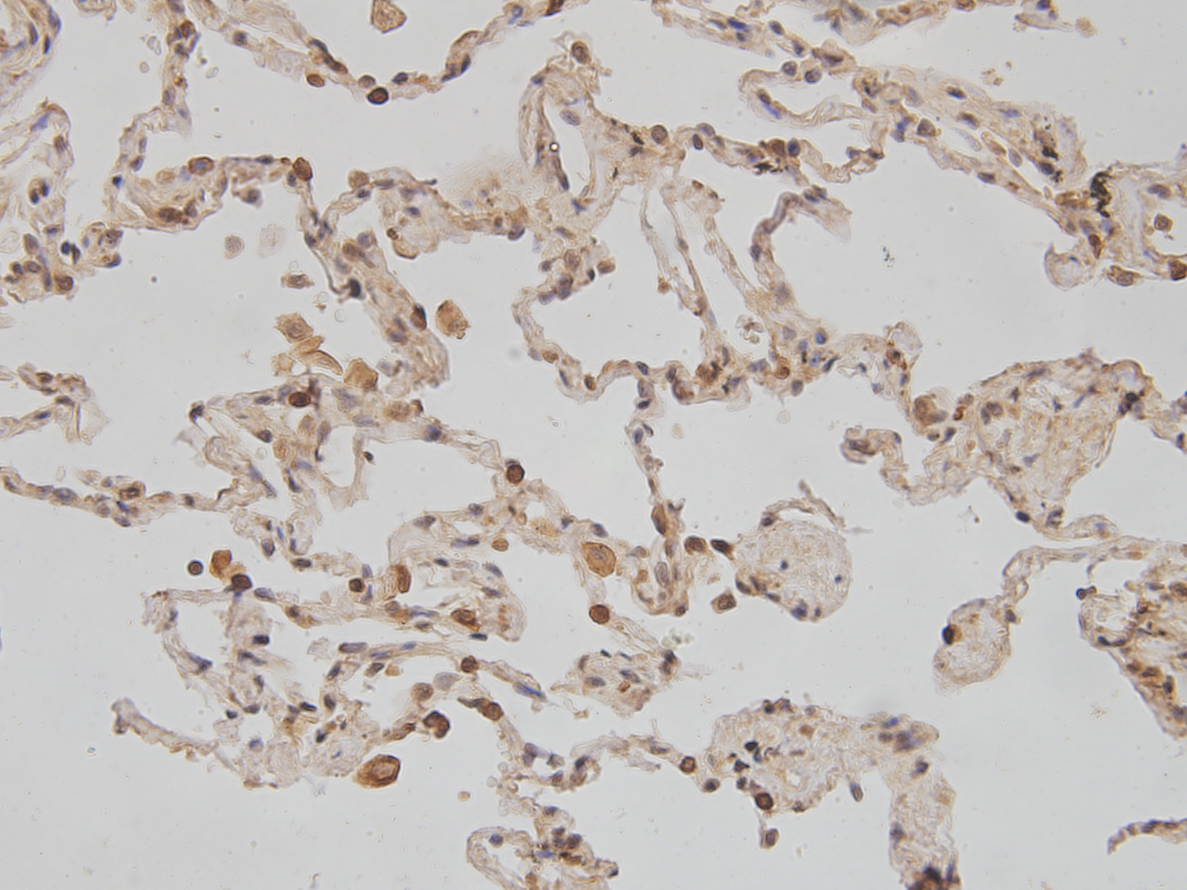

Supplement: S38 File — (ZIP) [file pone.0337223.s039.zip › 488702-400X-N-CA/488702-400X-N (1).tif]

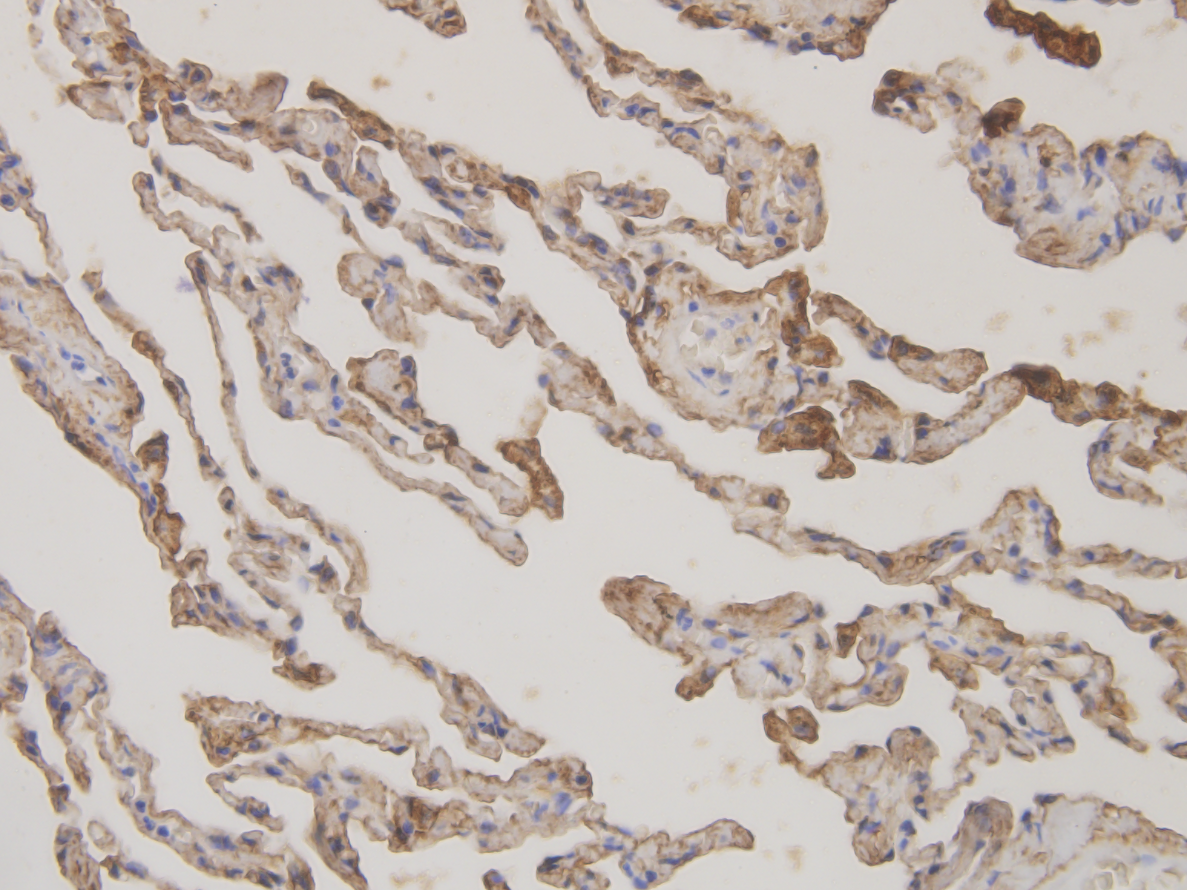

Supplement: S38 File — (ZIP) [file pone.0337223.s039.zip › 488702-400X-N-CA/488702-400X-N (2).tif]

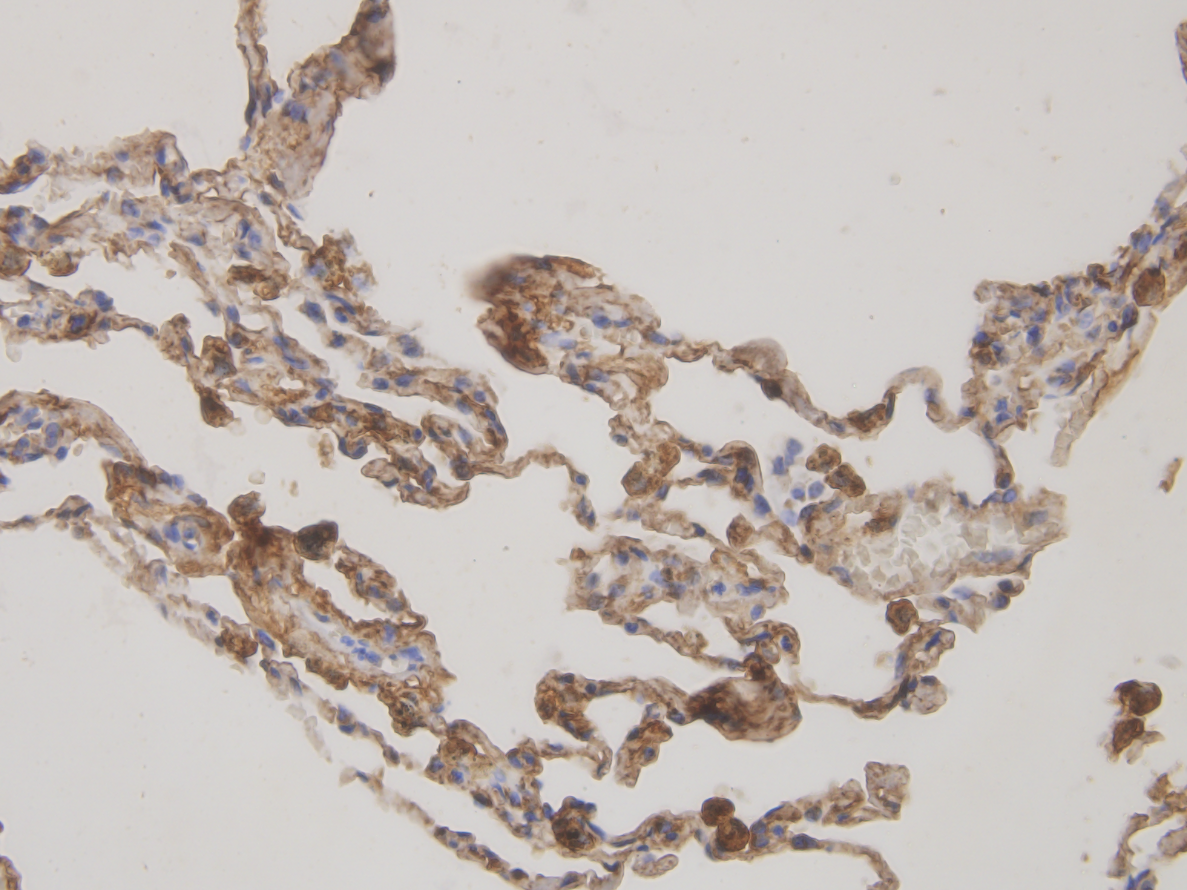

Supplement: S38 File — (ZIP) [file pone.0337223.s039.zip › 488702-400X-N-CA/488702-400X-N (3).tif]

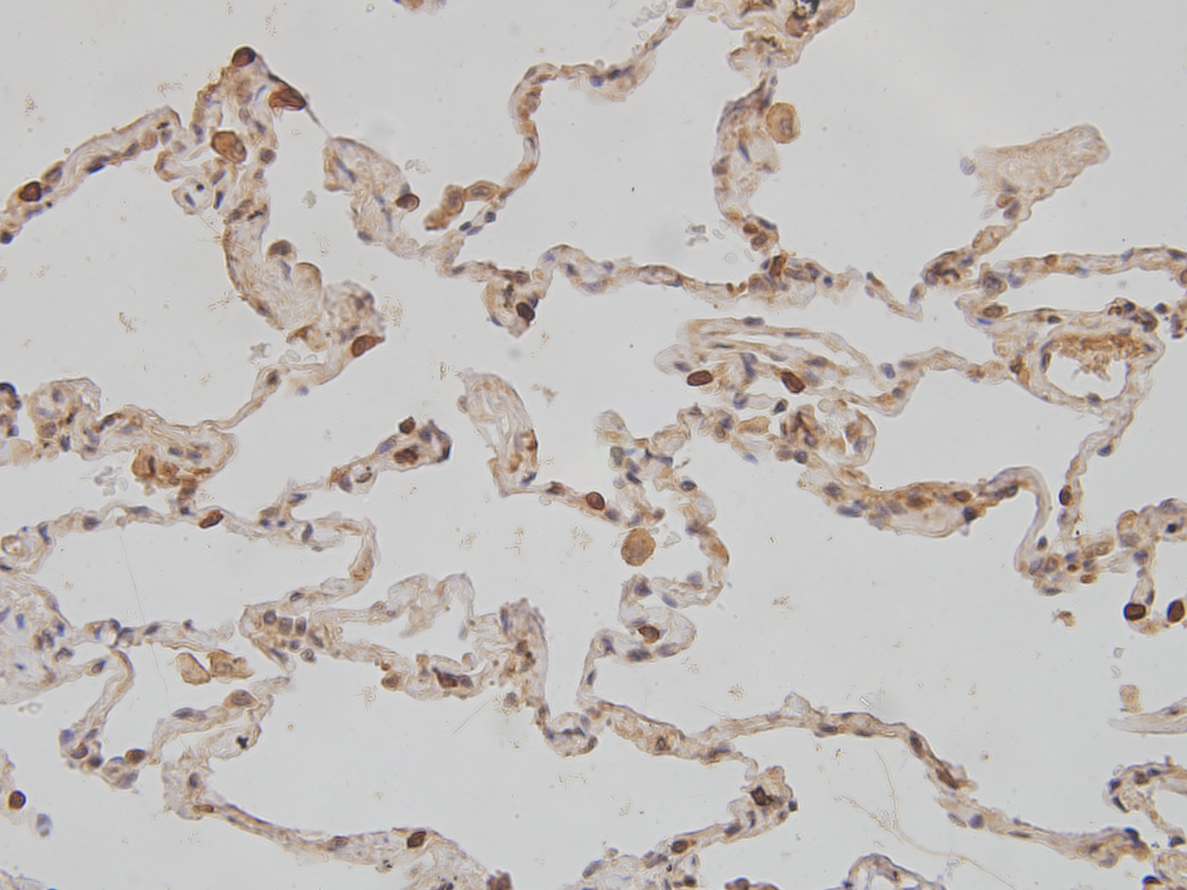

Supplement: S38 File — (ZIP) [file pone.0337223.s039.zip › 488702-400X-N-CA/488702-400X-N (4).tif]

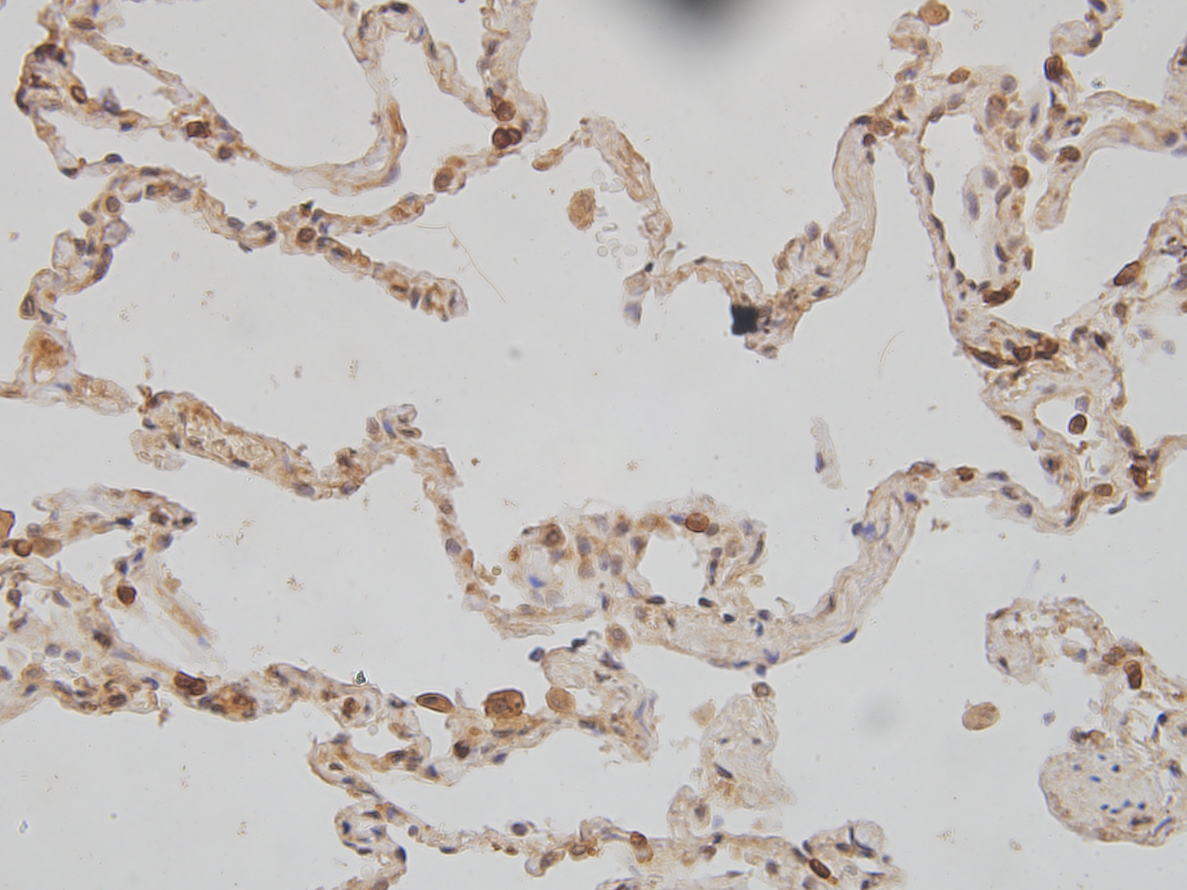

Supplement: S38 File — (ZIP) [file pone.0337223.s039.zip › 488702-400X-N-CA/488702-400X-N (5).tif]

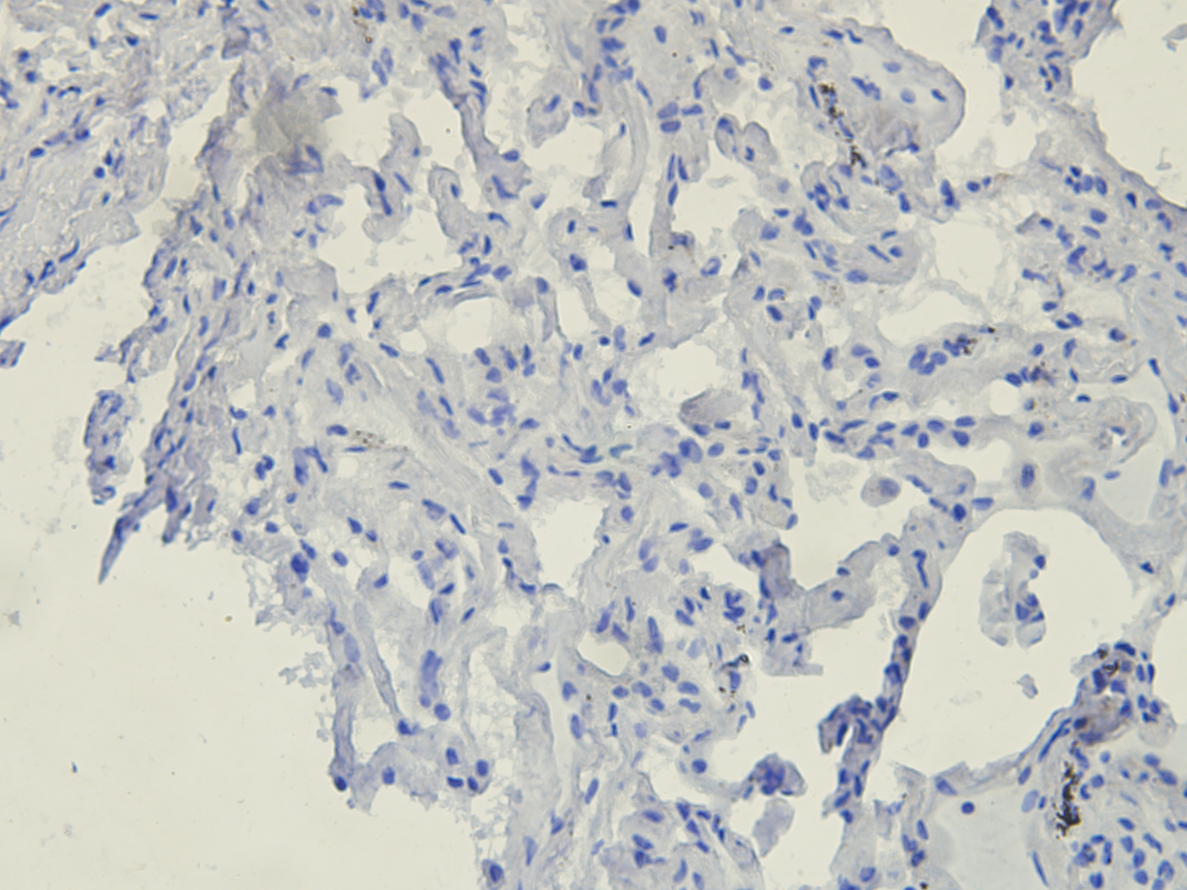

Supplement: S39 File — (ZIP) [file pone.0337223.s040.zip › 489990-400X-CA-N/489990-ca (1).tif]

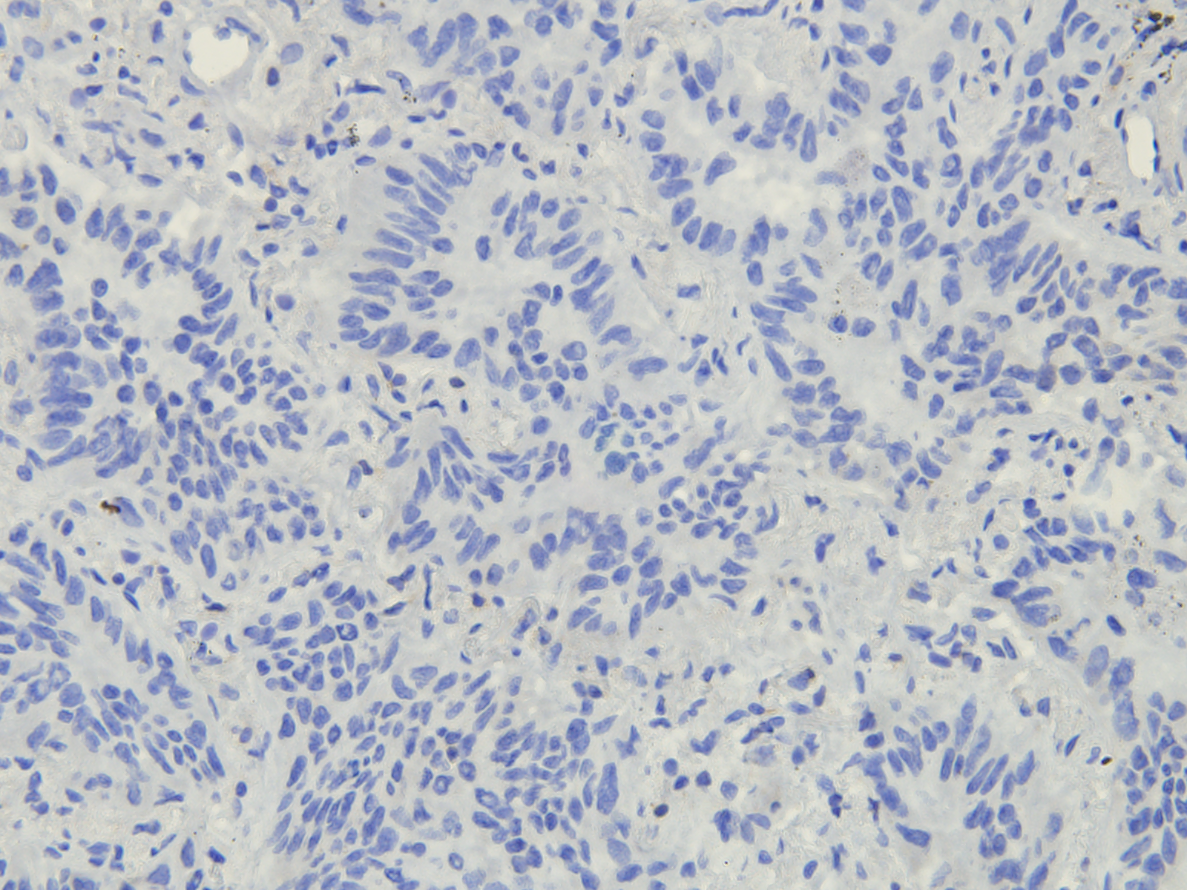

Supplement: S39 File — (ZIP) [file pone.0337223.s040.zip › 489990-400X-CA-N/489990-ca (2).tif]

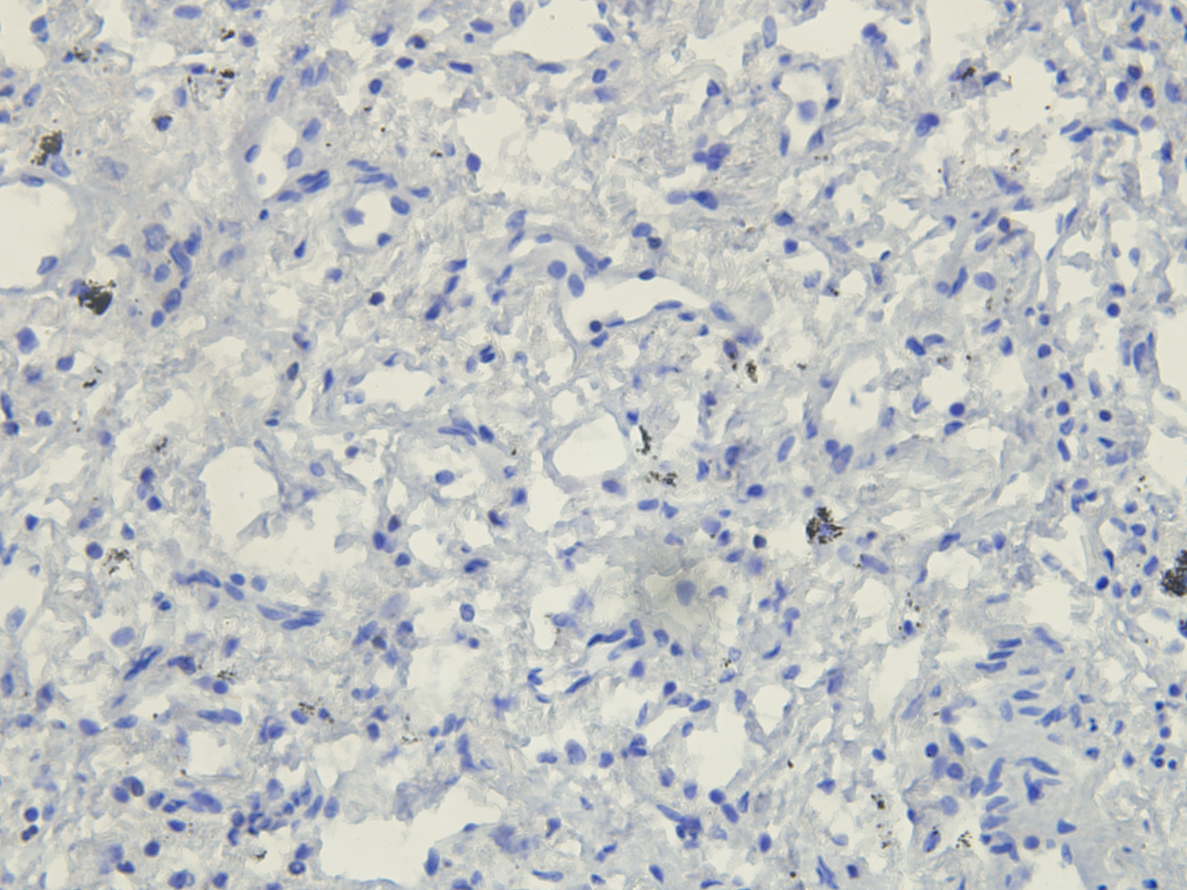

Supplement: S39 File — (ZIP) [file pone.0337223.s040.zip › 489990-400X-CA-N/489990-ca (3).tif]

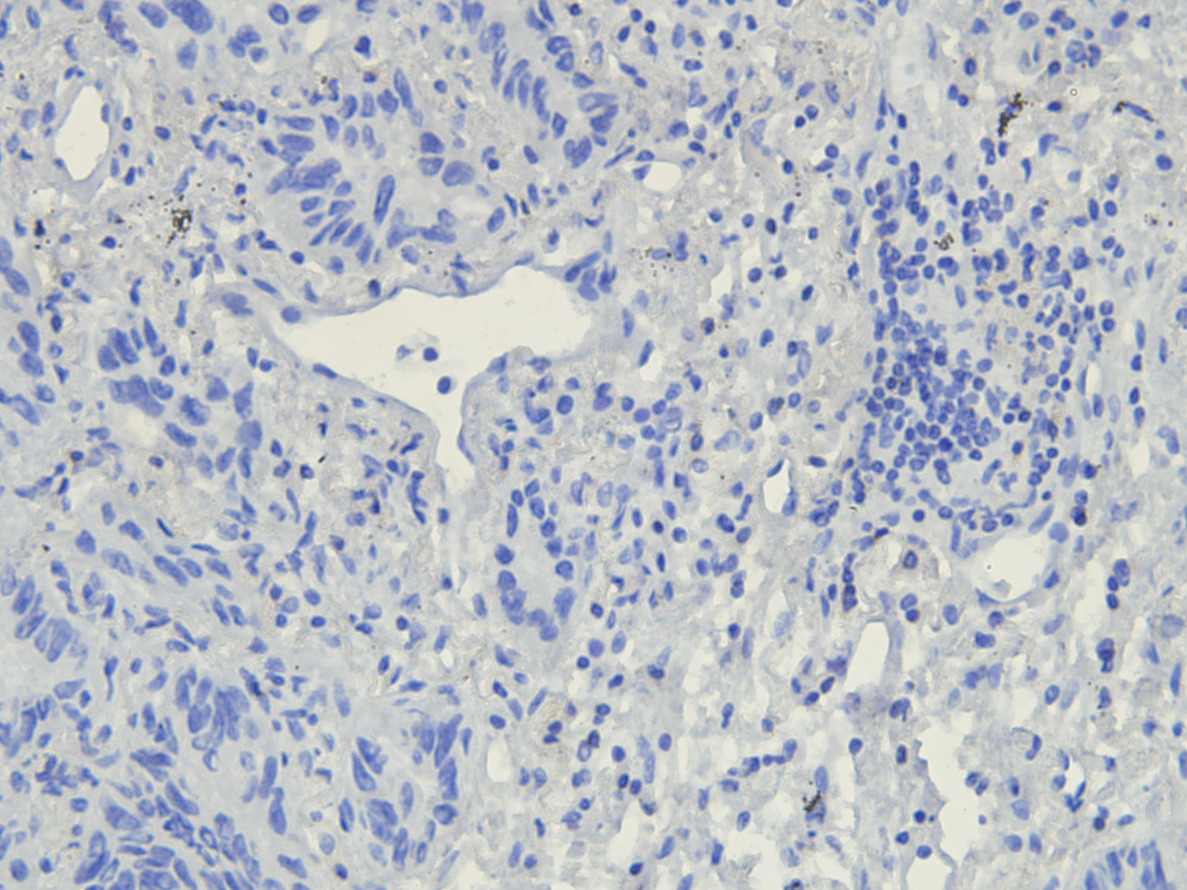

Supplement: S39 File — (ZIP) [file pone.0337223.s040.zip › 489990-400X-CA-N/489990-ca (4).tif]

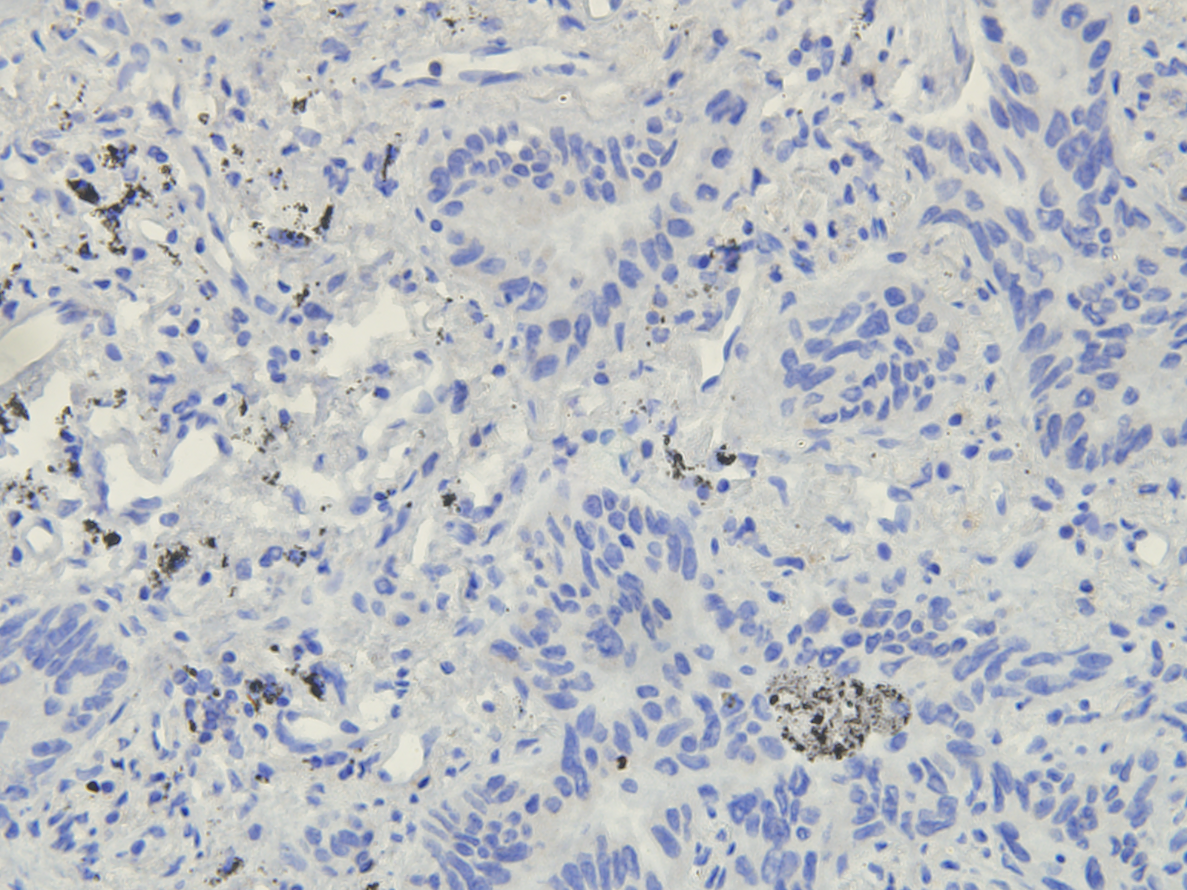

Supplement: S39 File — (ZIP) [file pone.0337223.s040.zip › 489990-400X-CA-N/489990-ca (5).tif]
