# Supplementary figures and images for: Overexpression of PTPRCAP inhibits biological function of lung adenocarcinoma through apoptosis pathway (part 4 of 5)
Source: PLoS One. 2025 Dec 18;20(12):e0337223. doi: 10.1371/journal.pone.0337223 (PMC12716888; doi:10.1371/journal.pone.0337223)

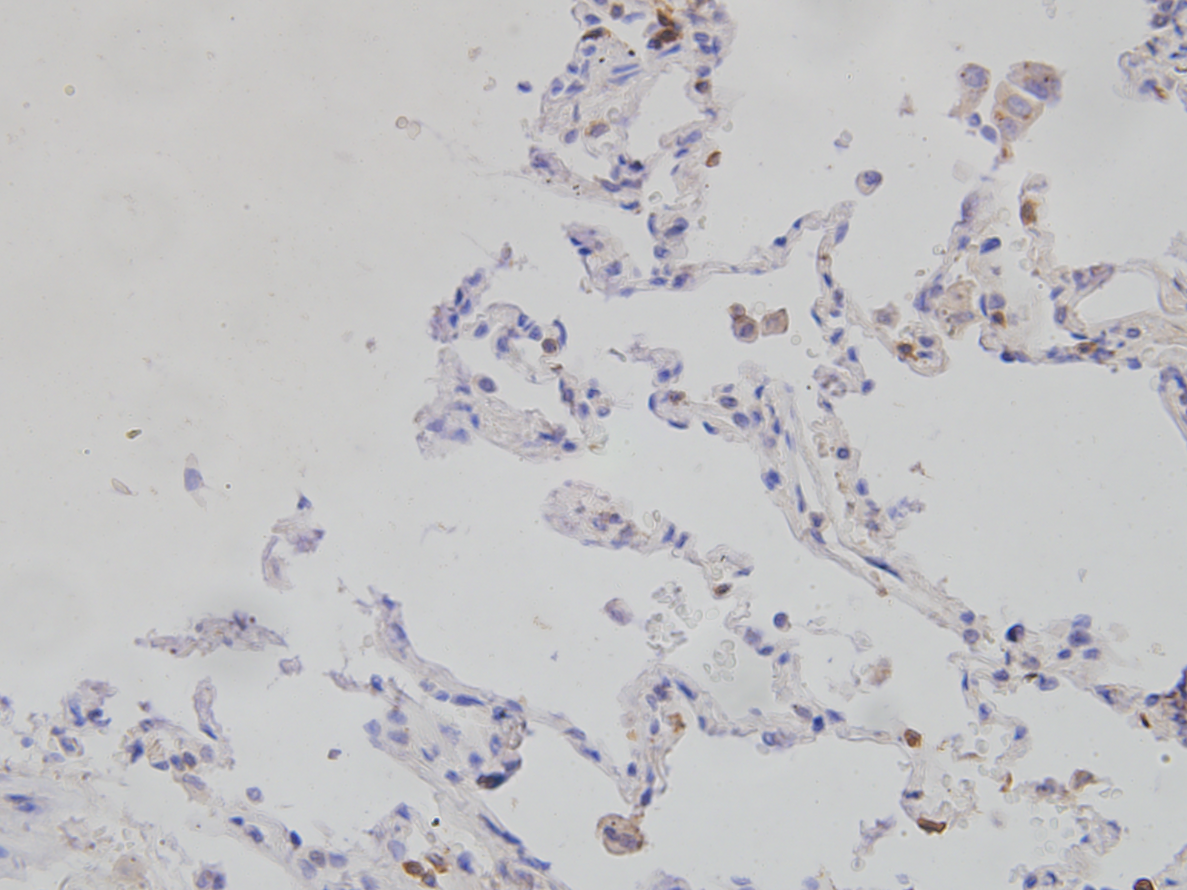

Supplement: S39 File — (ZIP) [file pone.0337223.s040.zip › 489990-400X-CA-N/489990-n (1).tif]

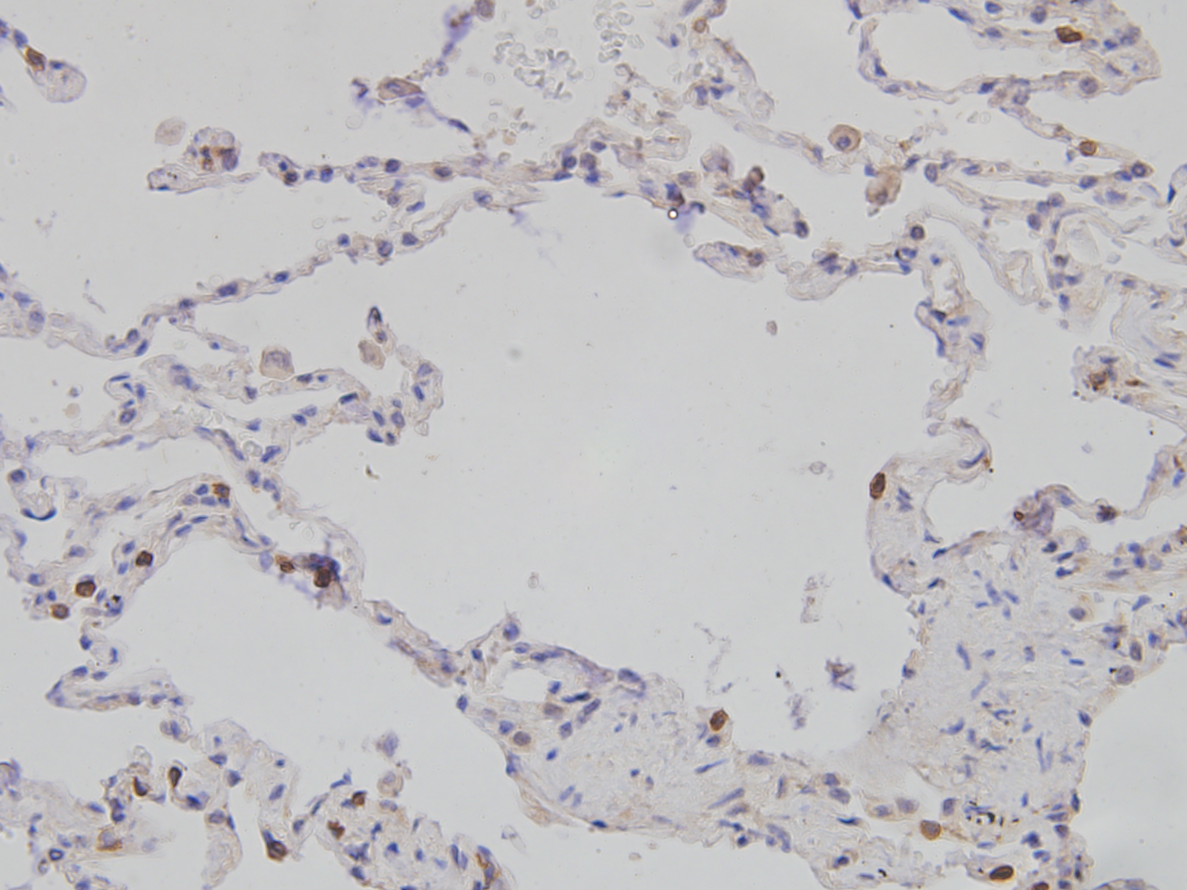

Supplement: S39 File — (ZIP) [file pone.0337223.s040.zip › 489990-400X-CA-N/489990-n (2).tif]

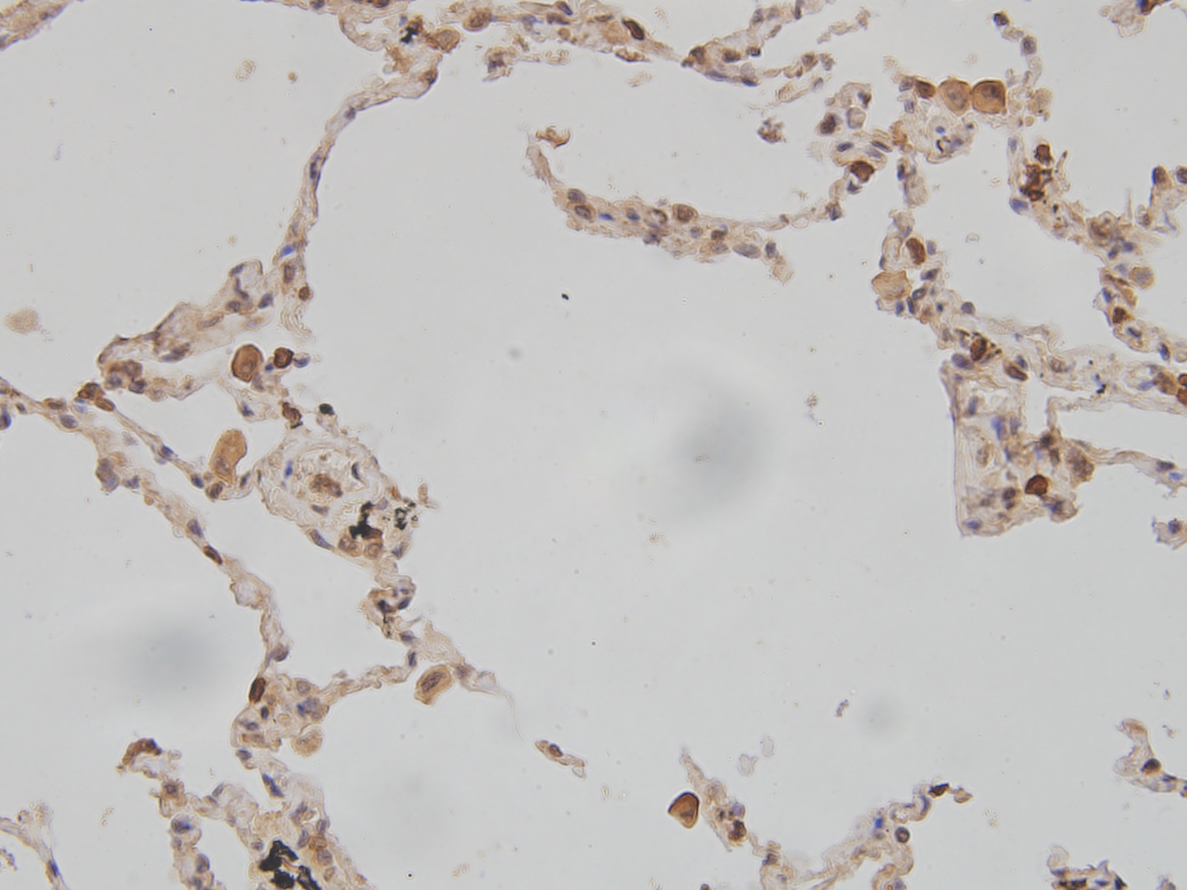

Supplement: S39 File — (ZIP) [file pone.0337223.s040.zip › 489990-400X-CA-N/489990-n (3).tif]

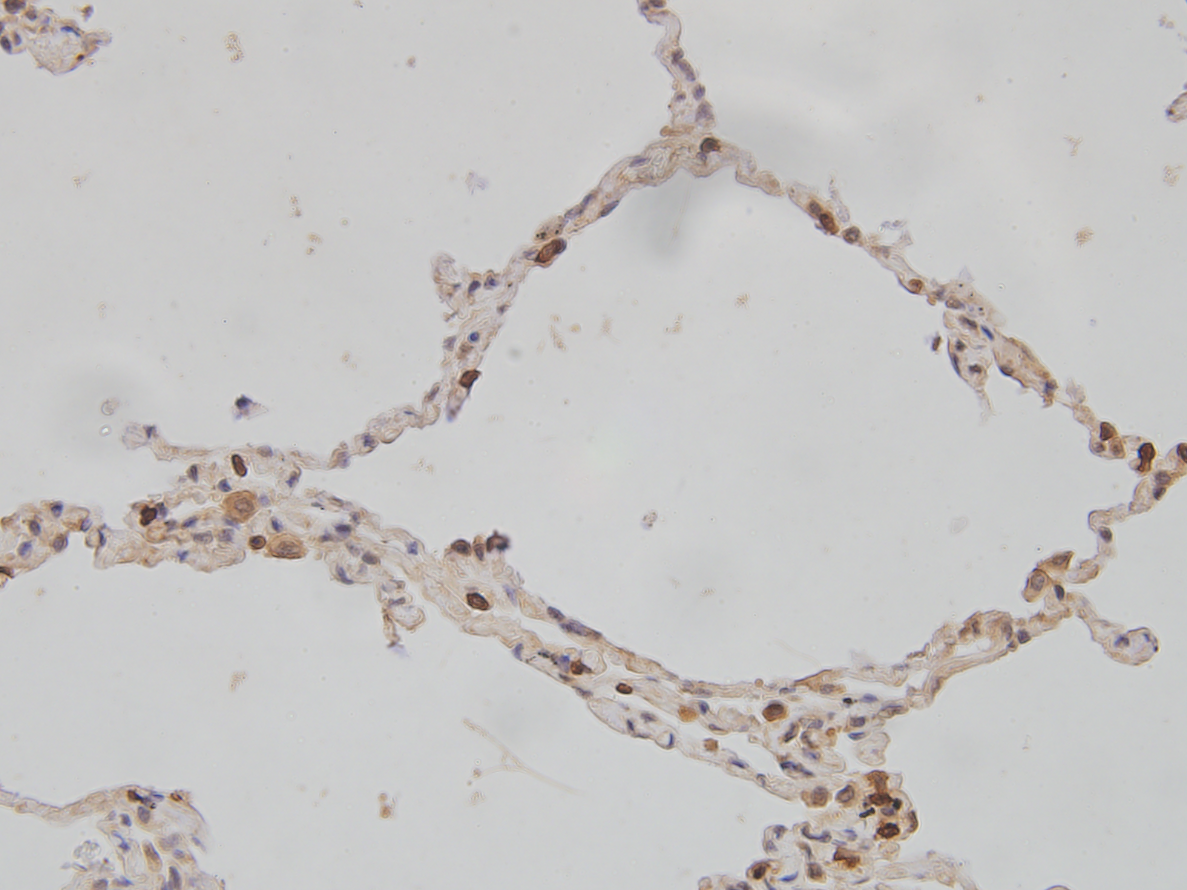

Supplement: S39 File — (ZIP) [file pone.0337223.s040.zip › 489990-400X-CA-N/489990-n (4).tif]

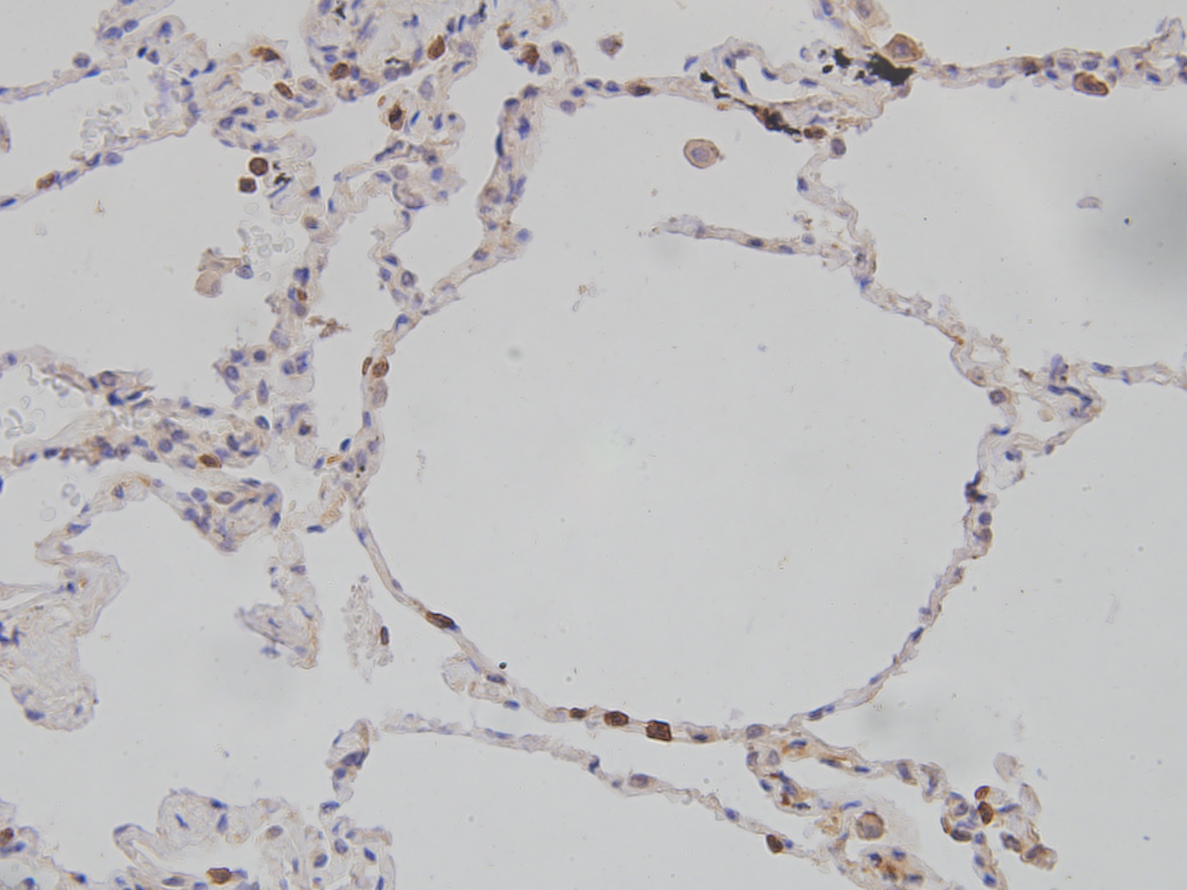

Supplement: S39 File — (ZIP) [file pone.0337223.s040.zip › 489990-400X-CA-N/489990-n (5).tif]

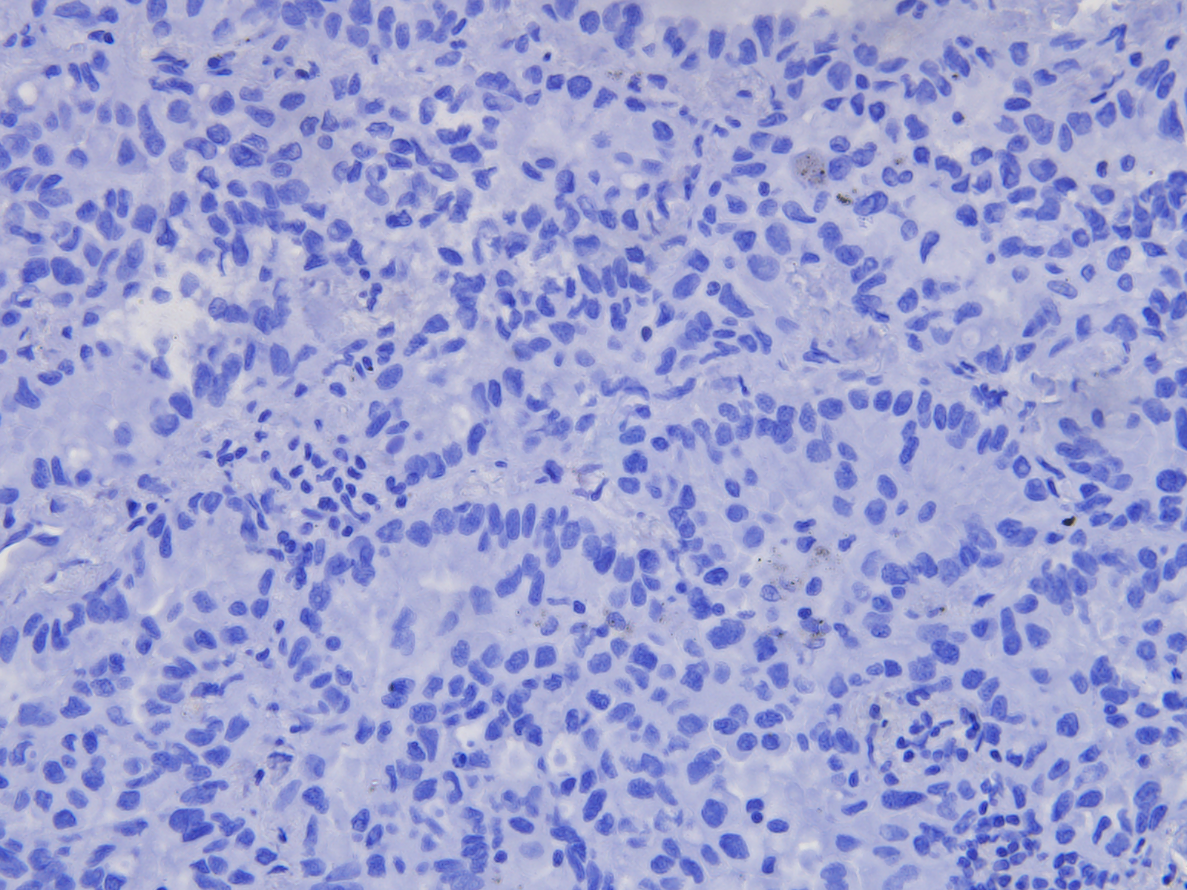

Supplement: S40 File — (ZIP) [file pone.0337223.s041.zip › 490129-400X-CA-N/490129-CA (1).tif]

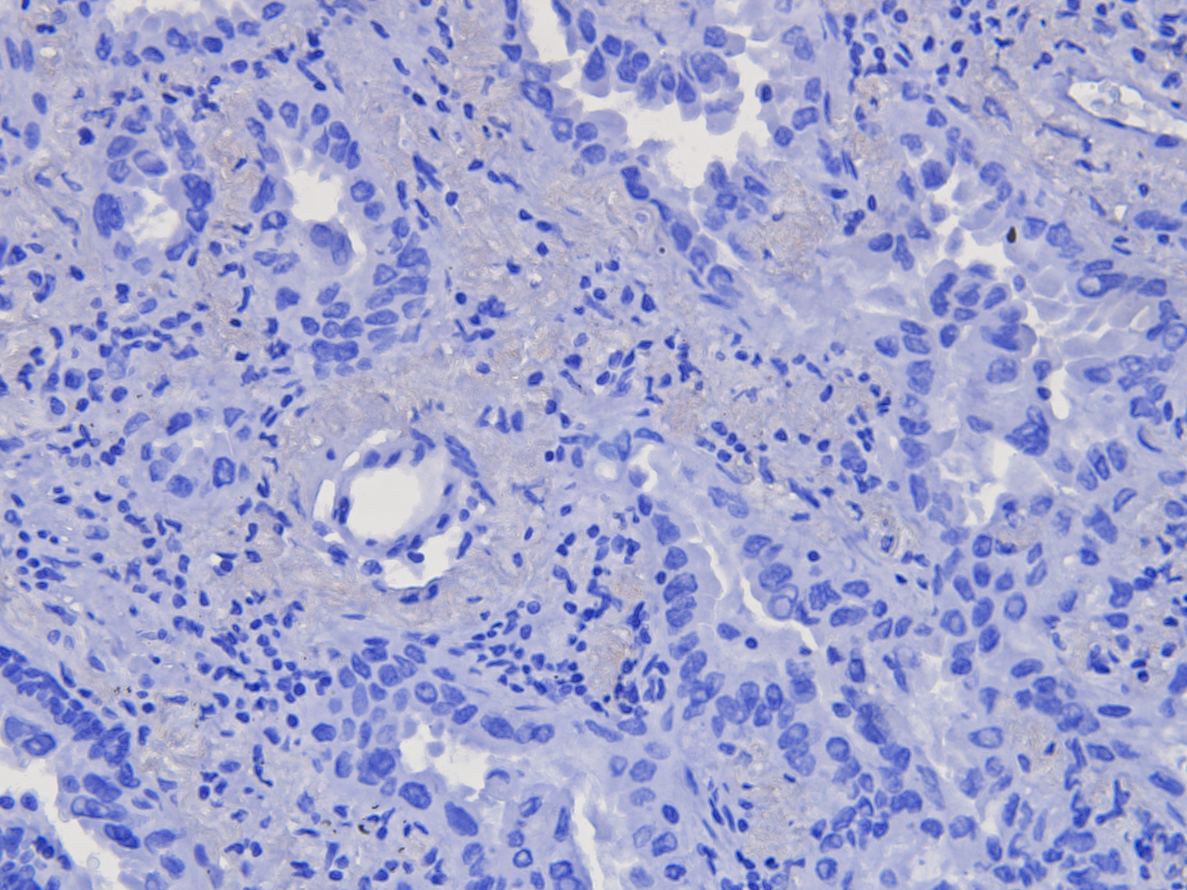

Supplement: S40 File — (ZIP) [file pone.0337223.s041.zip › 490129-400X-CA-N/490129-CA (2).tif]

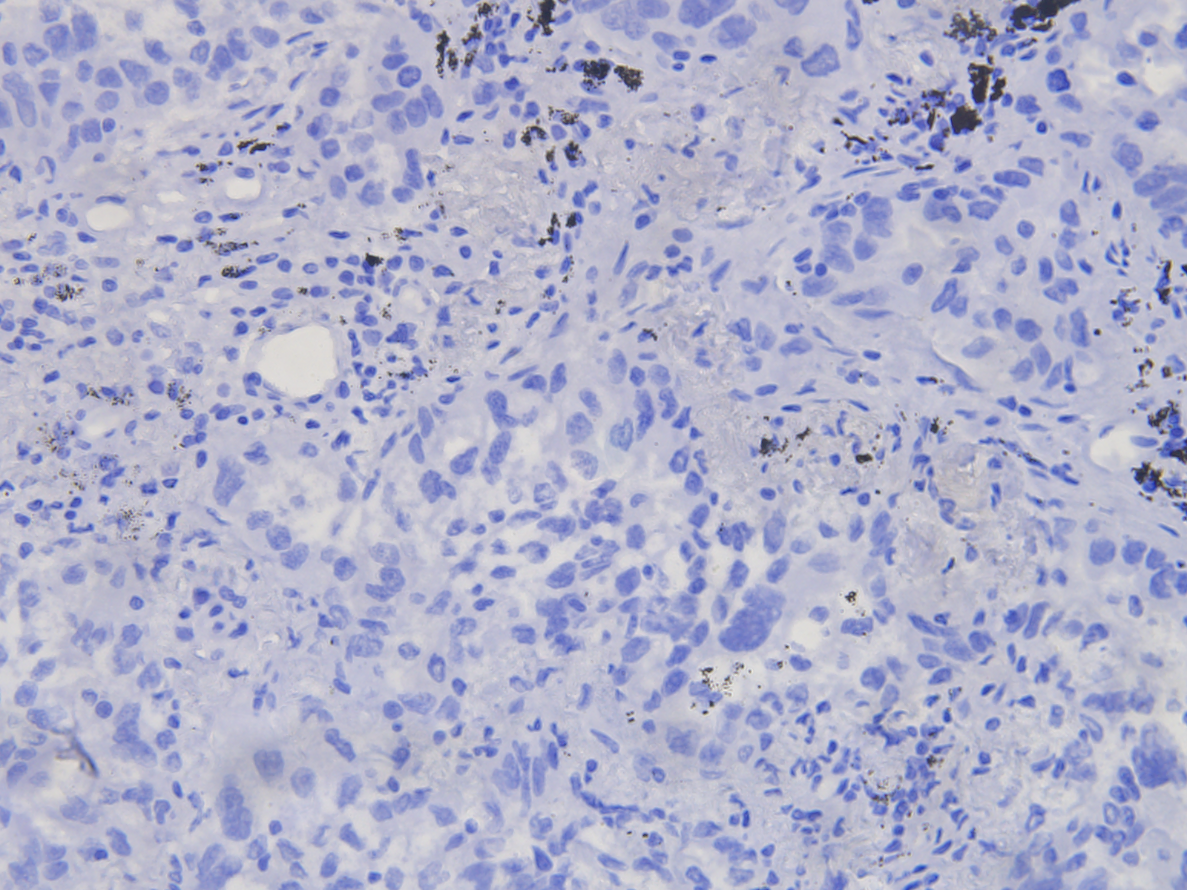

Supplement: S40 File — (ZIP) [file pone.0337223.s041.zip › 490129-400X-CA-N/490129-CA (3).tif]

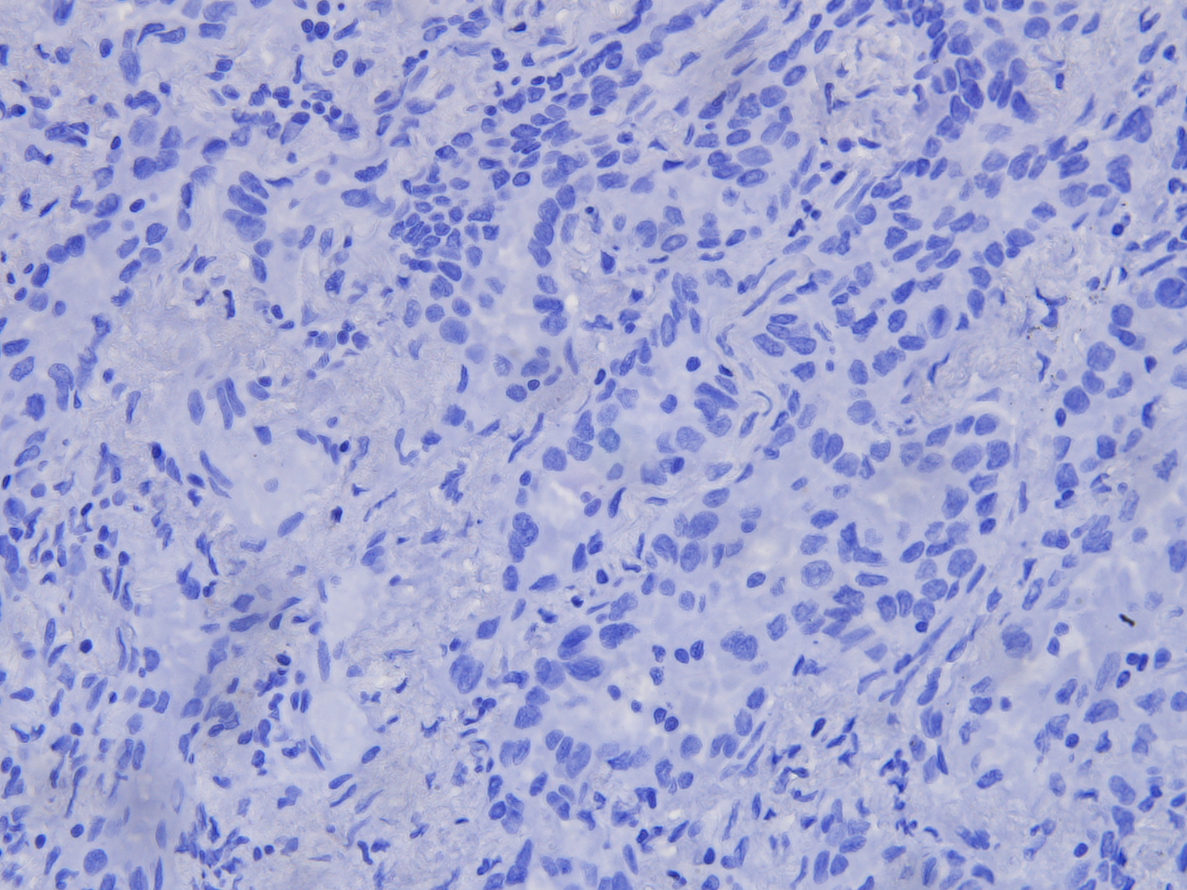

Supplement: S40 File — (ZIP) [file pone.0337223.s041.zip › 490129-400X-CA-N/490129-CA (4).tif]

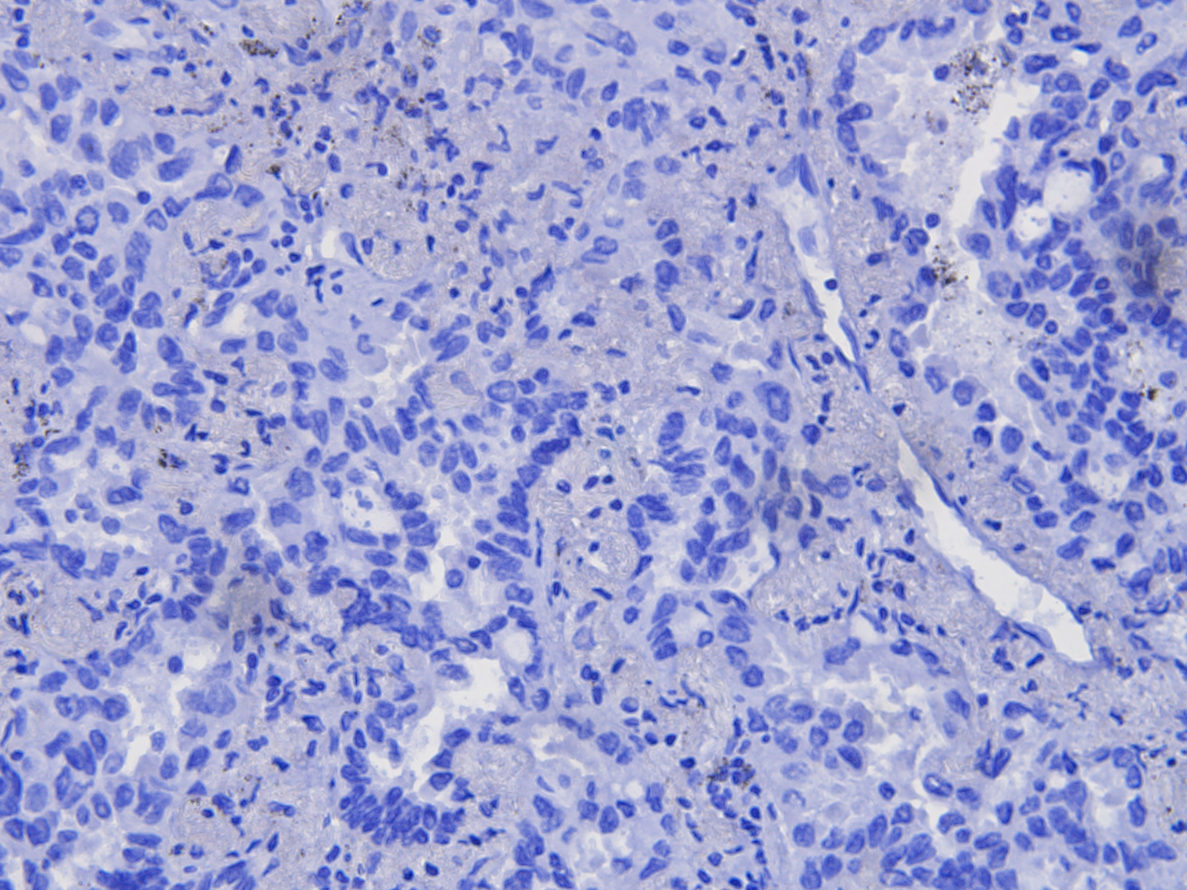

Supplement: S40 File — (ZIP) [file pone.0337223.s041.zip › 490129-400X-CA-N/490129-CA (5).tif]

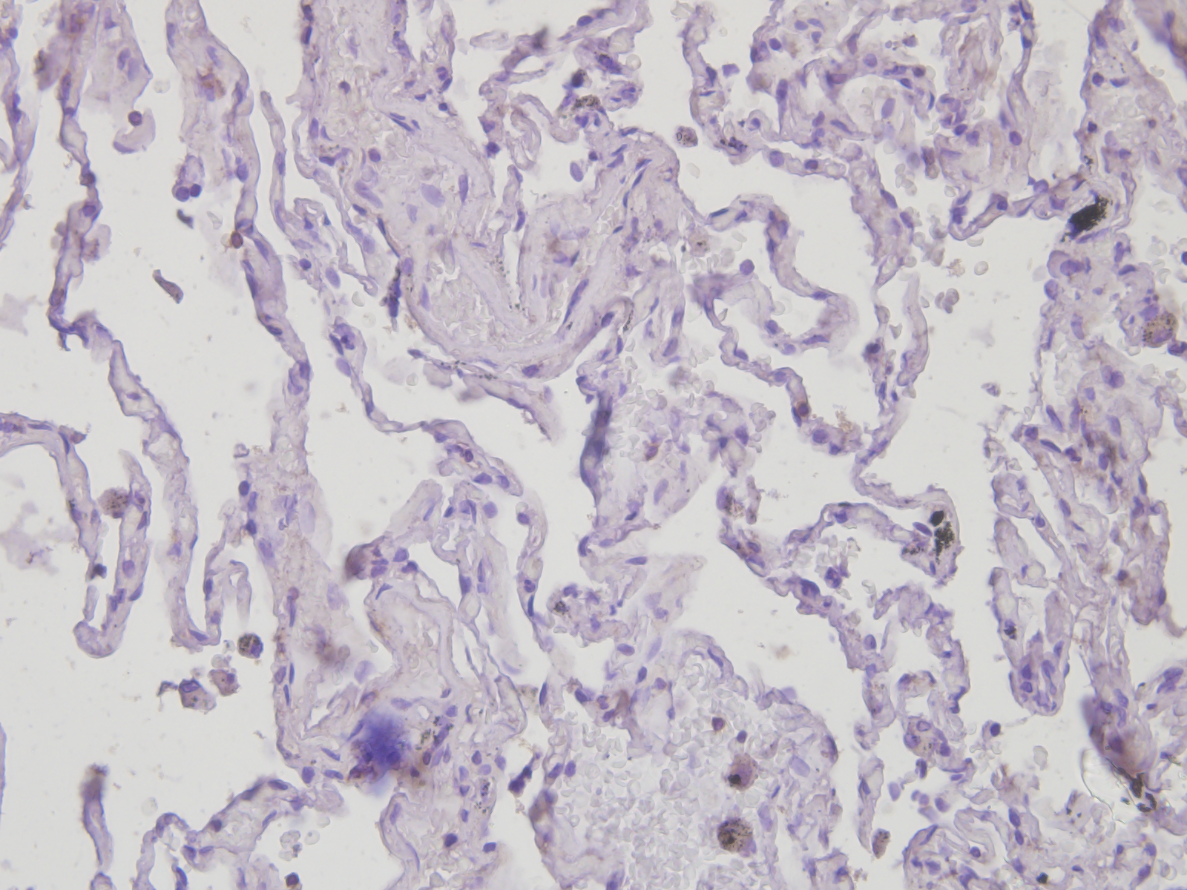

Supplement: S40 File — (ZIP) [file pone.0337223.s041.zip › 490129-400X-CA-N/490129-n (1).tif]

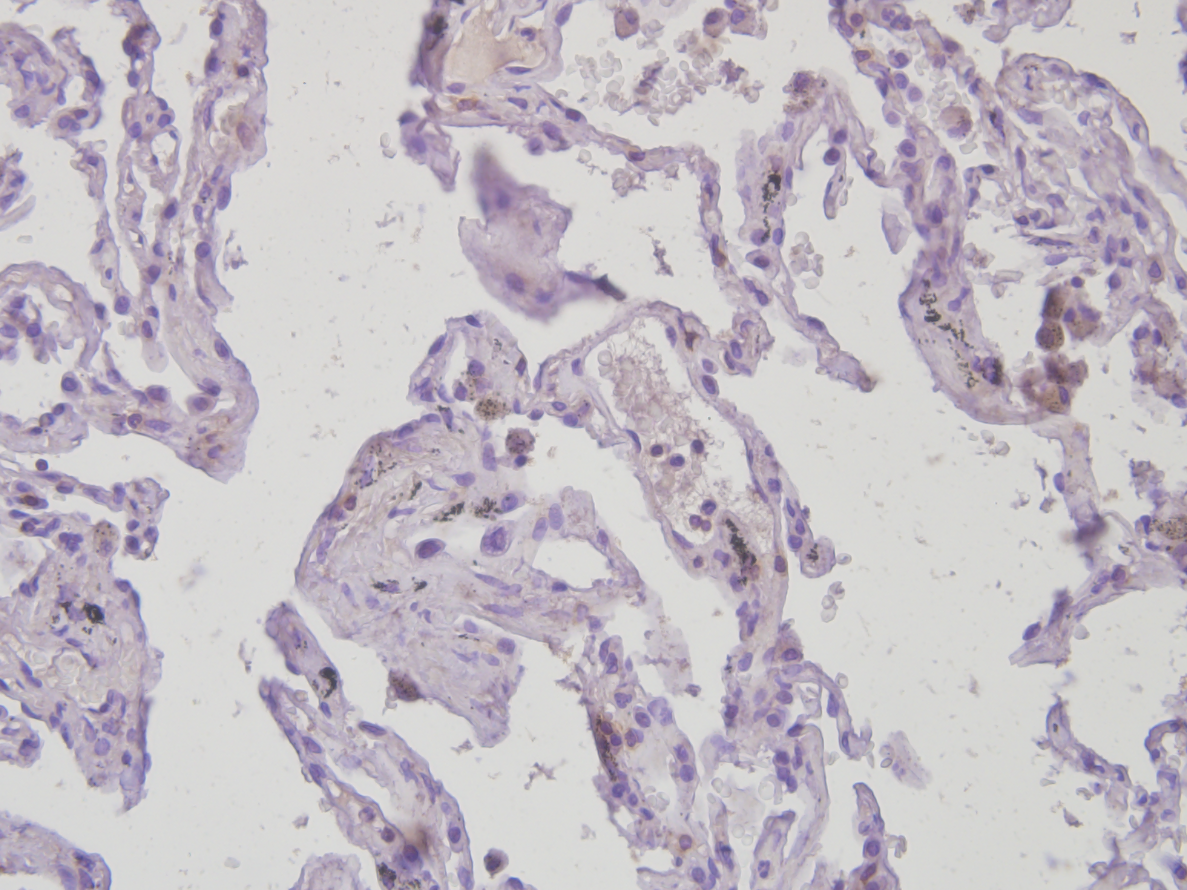

Supplement: S40 File — (ZIP) [file pone.0337223.s041.zip › 490129-400X-CA-N/490129-n (2).tif]

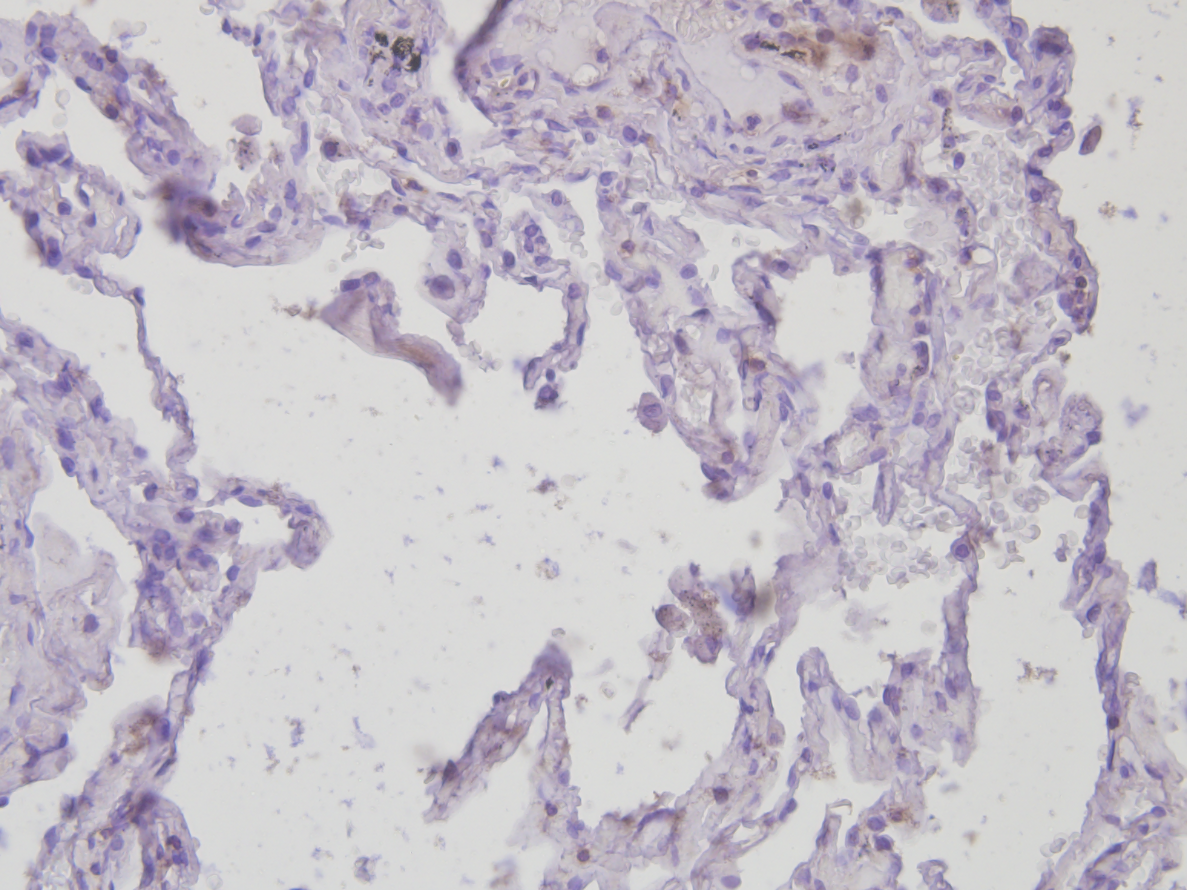

Supplement: S40 File — (ZIP) [file pone.0337223.s041.zip › 490129-400X-CA-N/490129-n (3).tif]

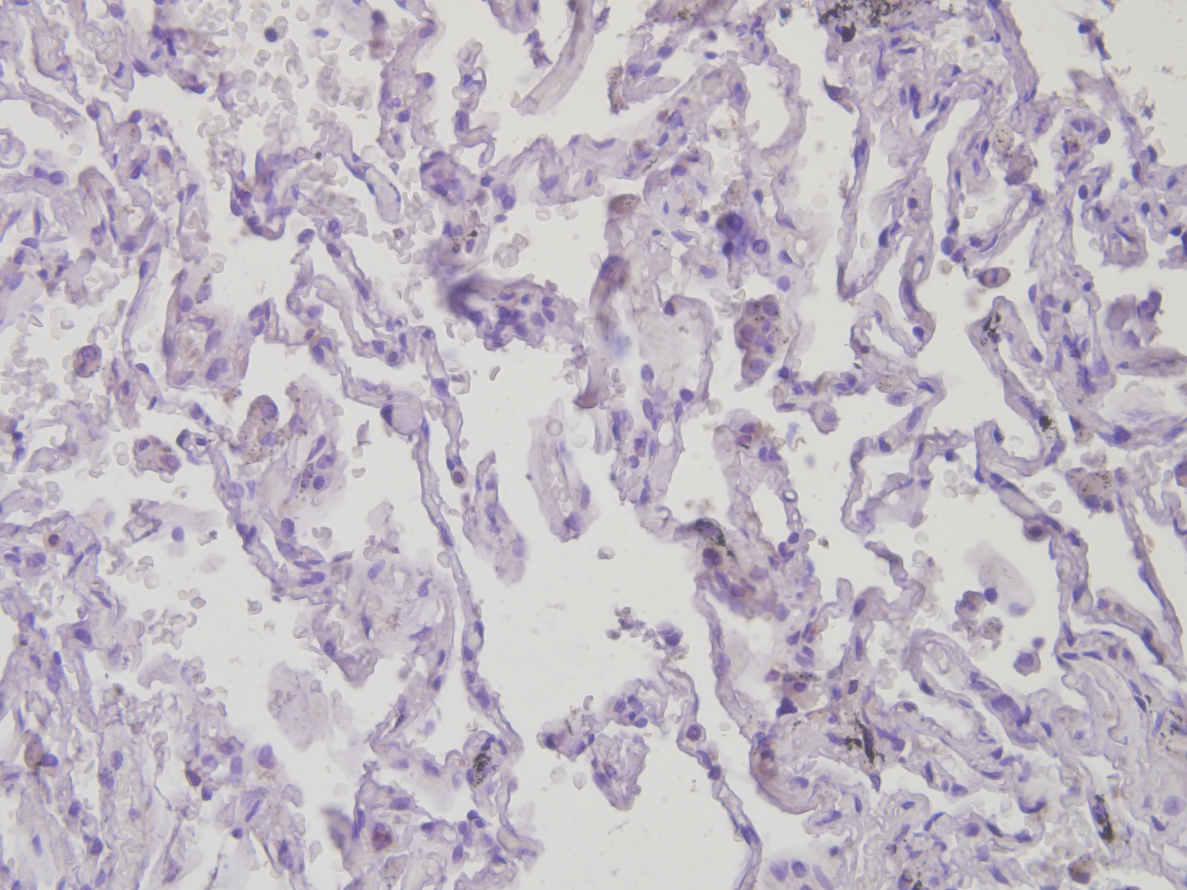

Supplement: S40 File — (ZIP) [file pone.0337223.s041.zip › 490129-400X-CA-N/490129-n (4).tif]

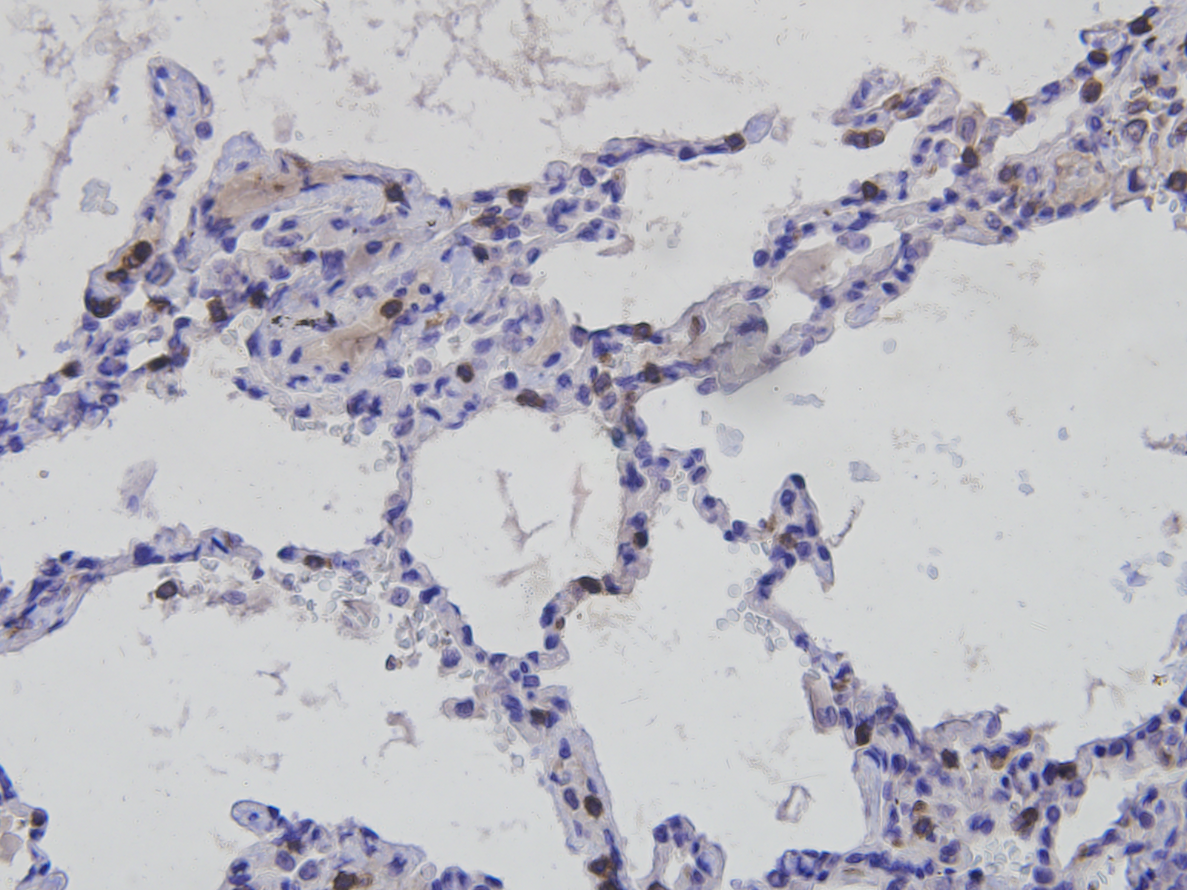

Supplement: S40 File — (ZIP) [file pone.0337223.s041.zip › 490129-400X-CA-N/490129-n(5).tif]

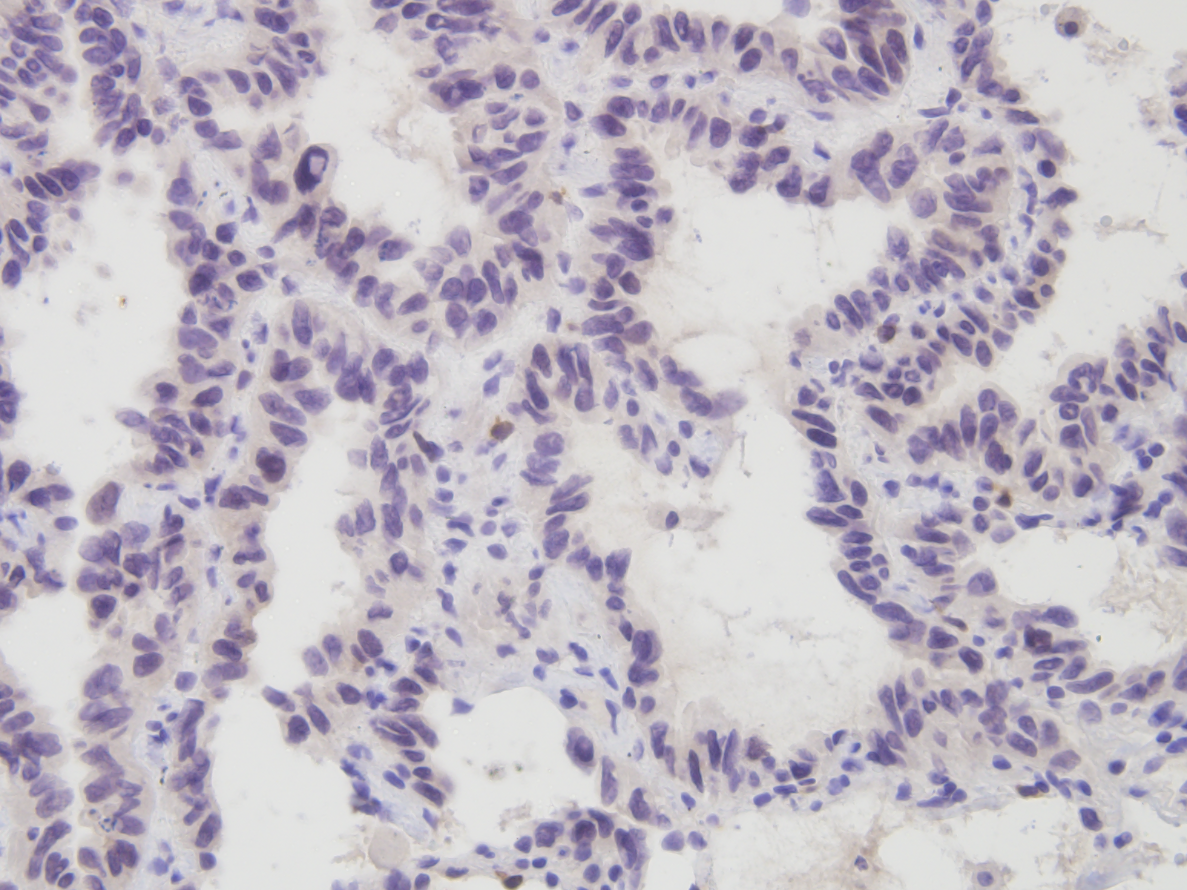

Supplement: S41 File — (ZIP) [file pone.0337223.s042.zip › 491617-400-CA-N/491617-400-CA (1).tif]

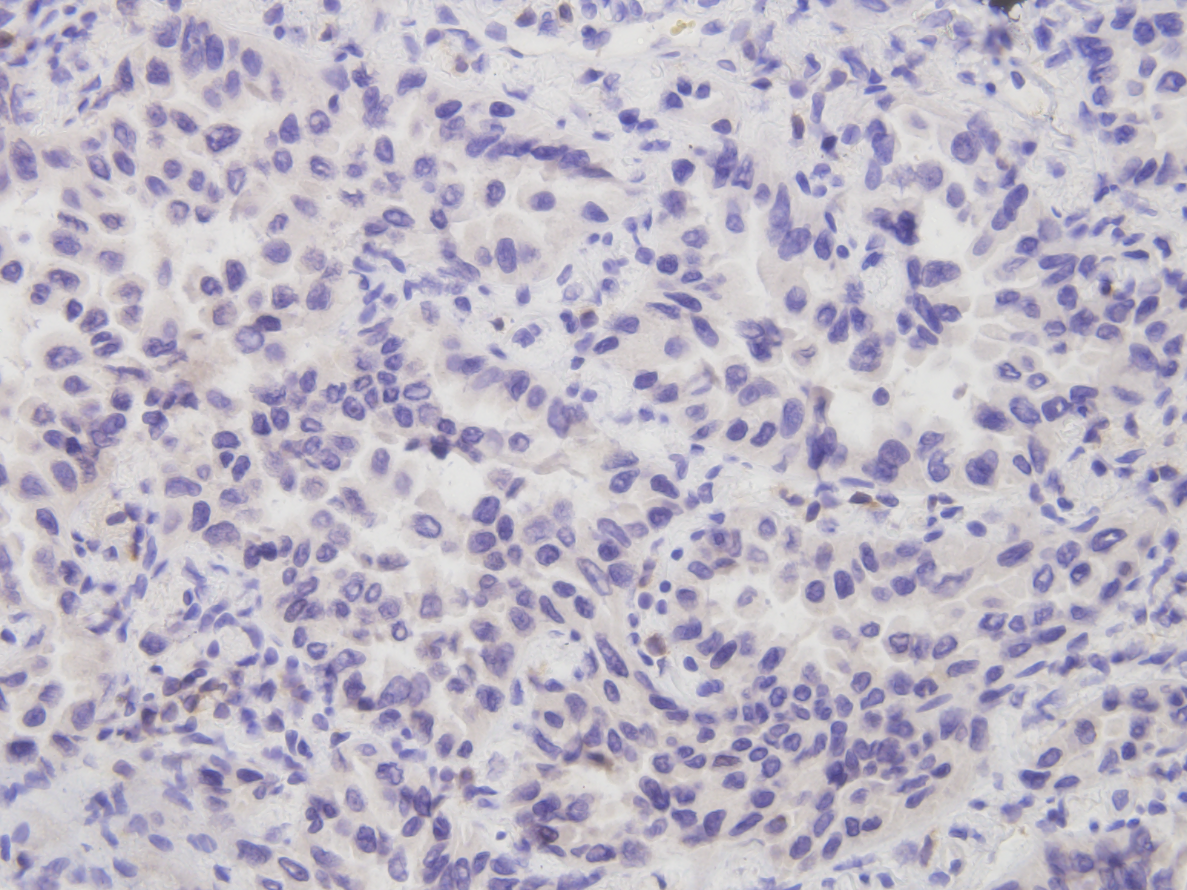

Supplement: S41 File — (ZIP) [file pone.0337223.s042.zip › 491617-400-CA-N/491617-400-CA (2).tif]

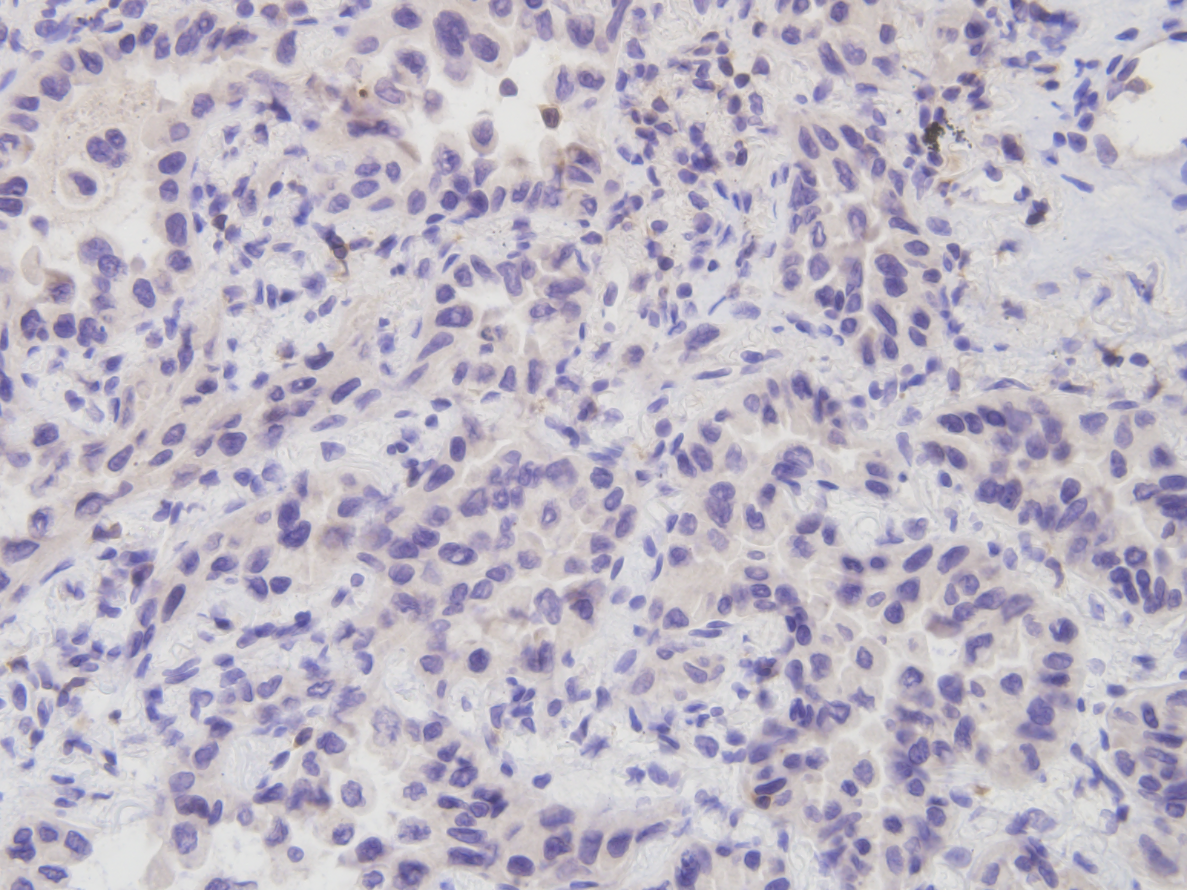

Supplement: S41 File — (ZIP) [file pone.0337223.s042.zip › 491617-400-CA-N/491617-400-CA (3).tif]

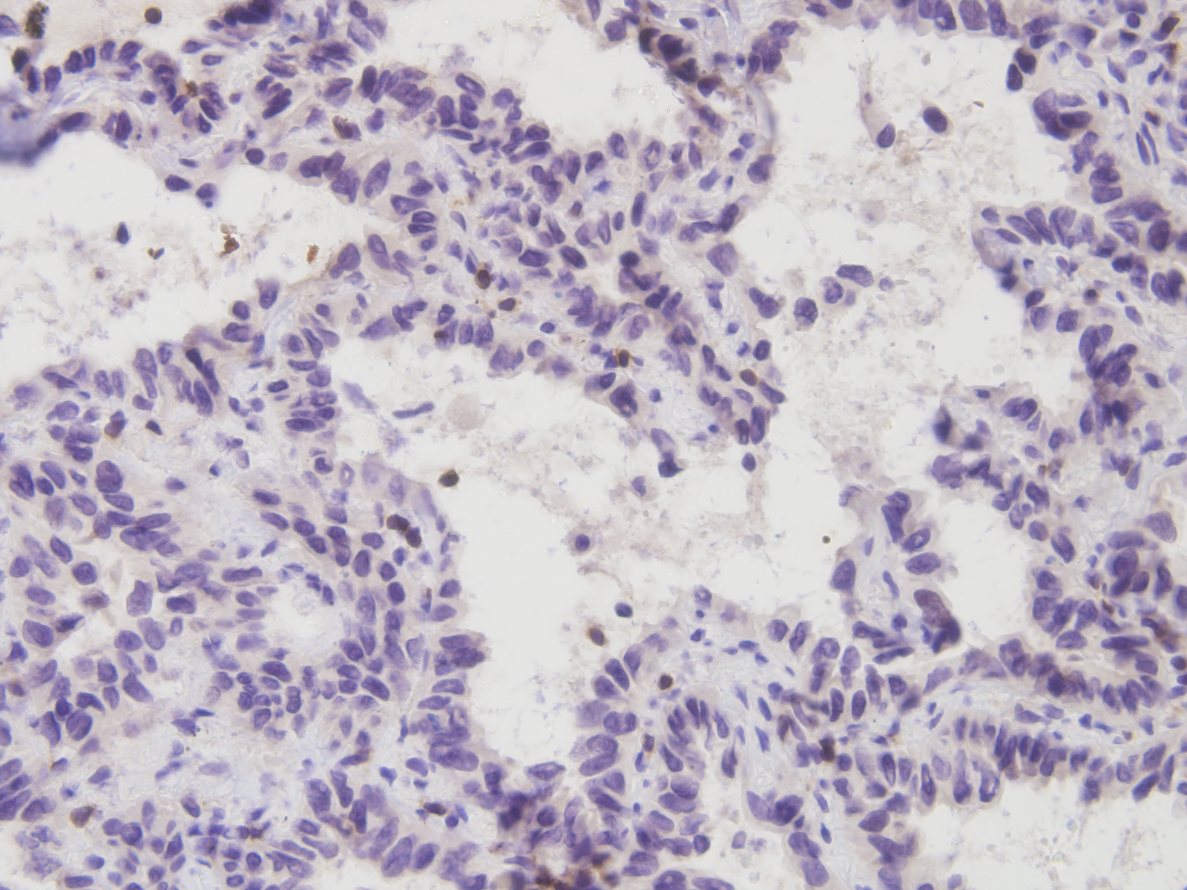

Supplement: S41 File — (ZIP) [file pone.0337223.s042.zip › 491617-400-CA-N/491617-400-CA (4).tif]

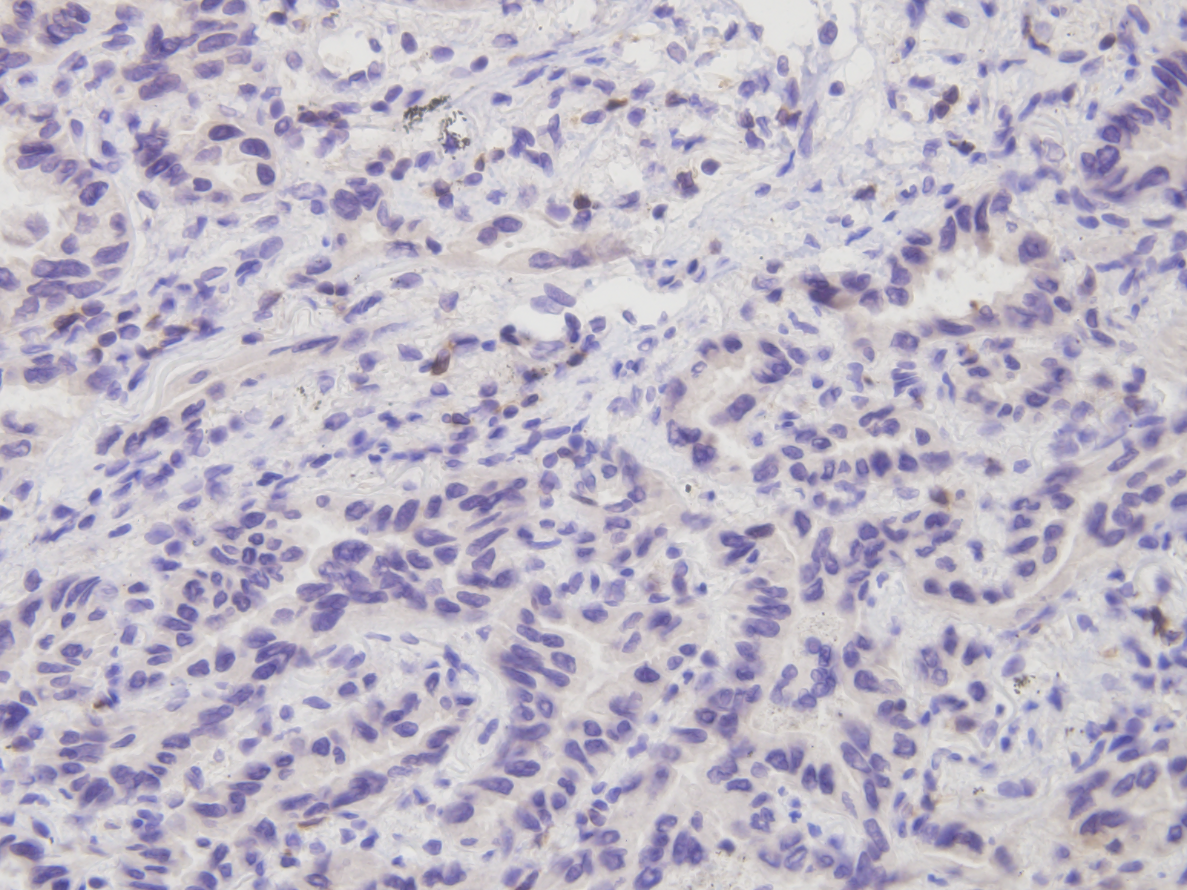

Supplement: S41 File — (ZIP) [file pone.0337223.s042.zip › 491617-400-CA-N/491617-400-CA (5).tif]

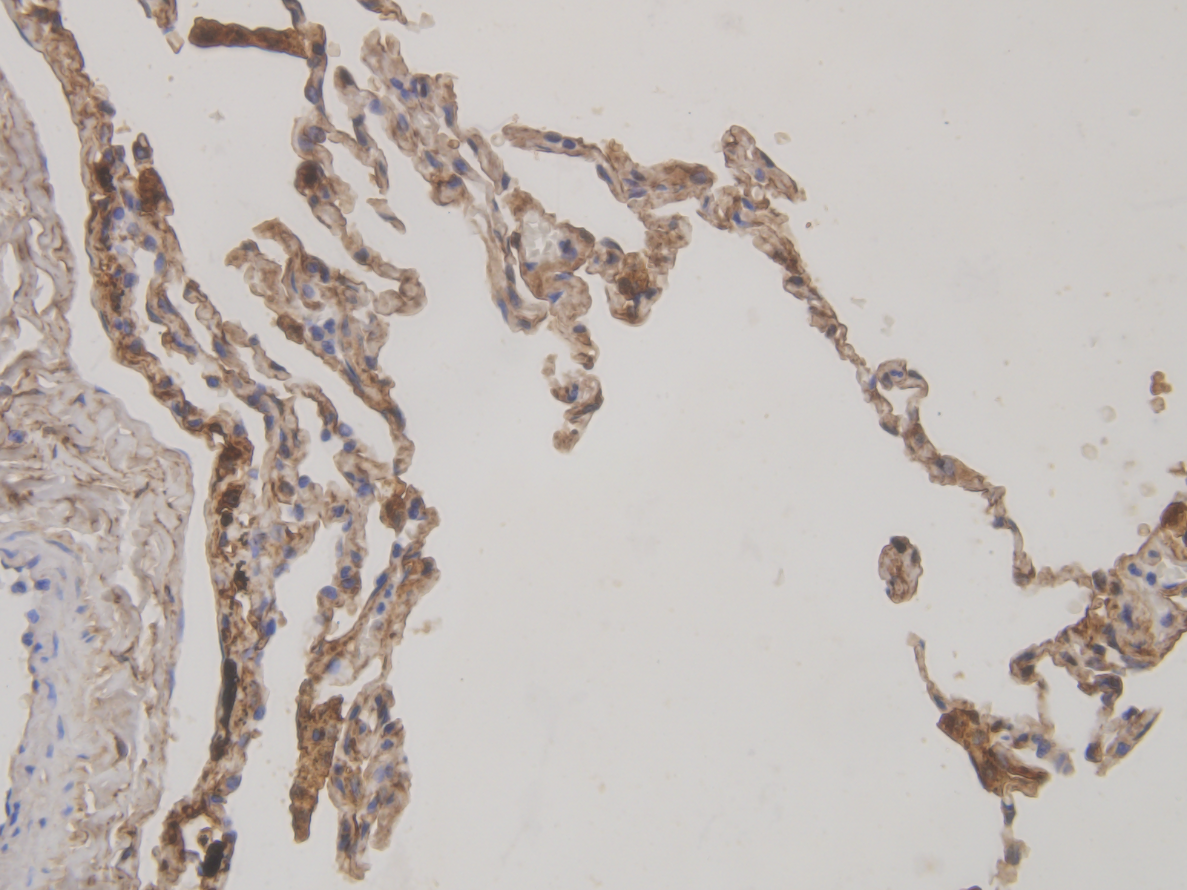

Supplement: S41 File — (ZIP) [file pone.0337223.s042.zip › 491617-400-CA-N/491617-400-N (1).tif]

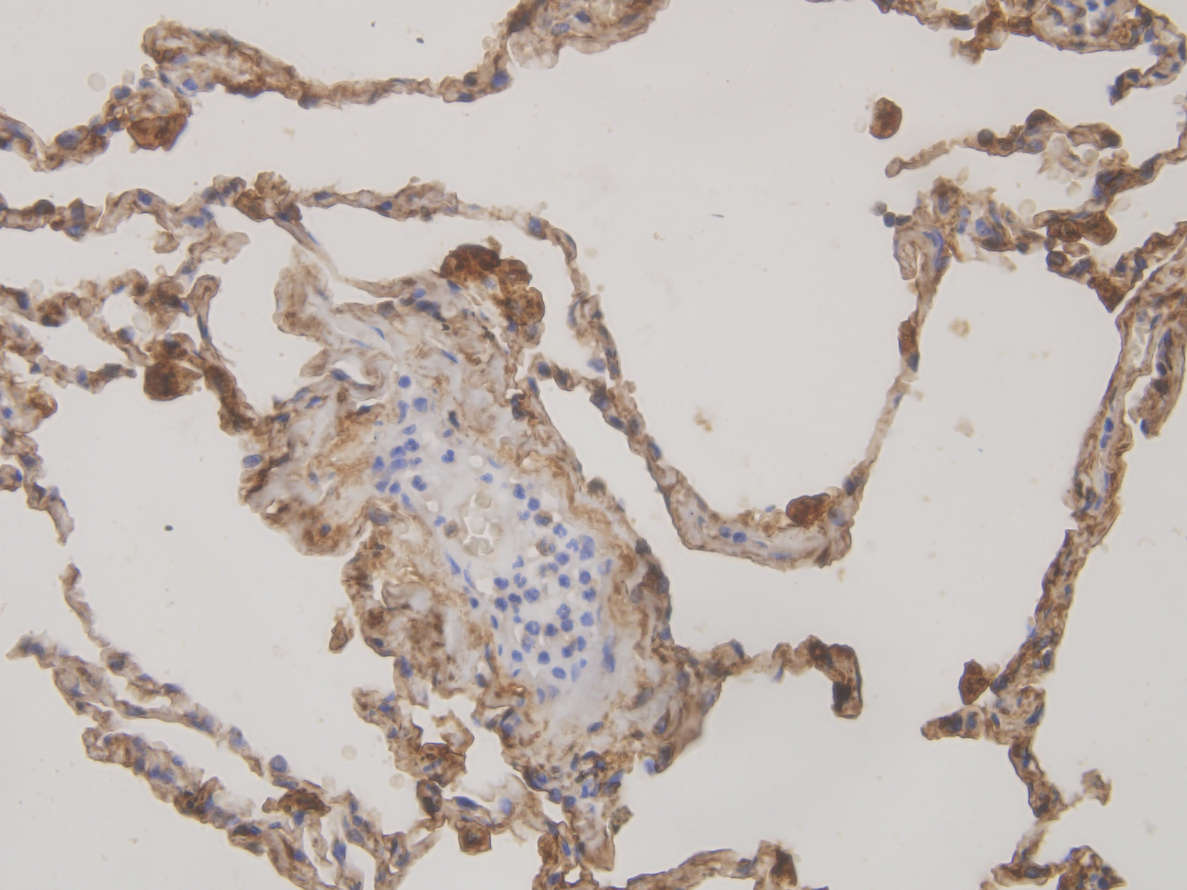

Supplement: S41 File — (ZIP) [file pone.0337223.s042.zip › 491617-400-CA-N/491617-400-N (2).tif]

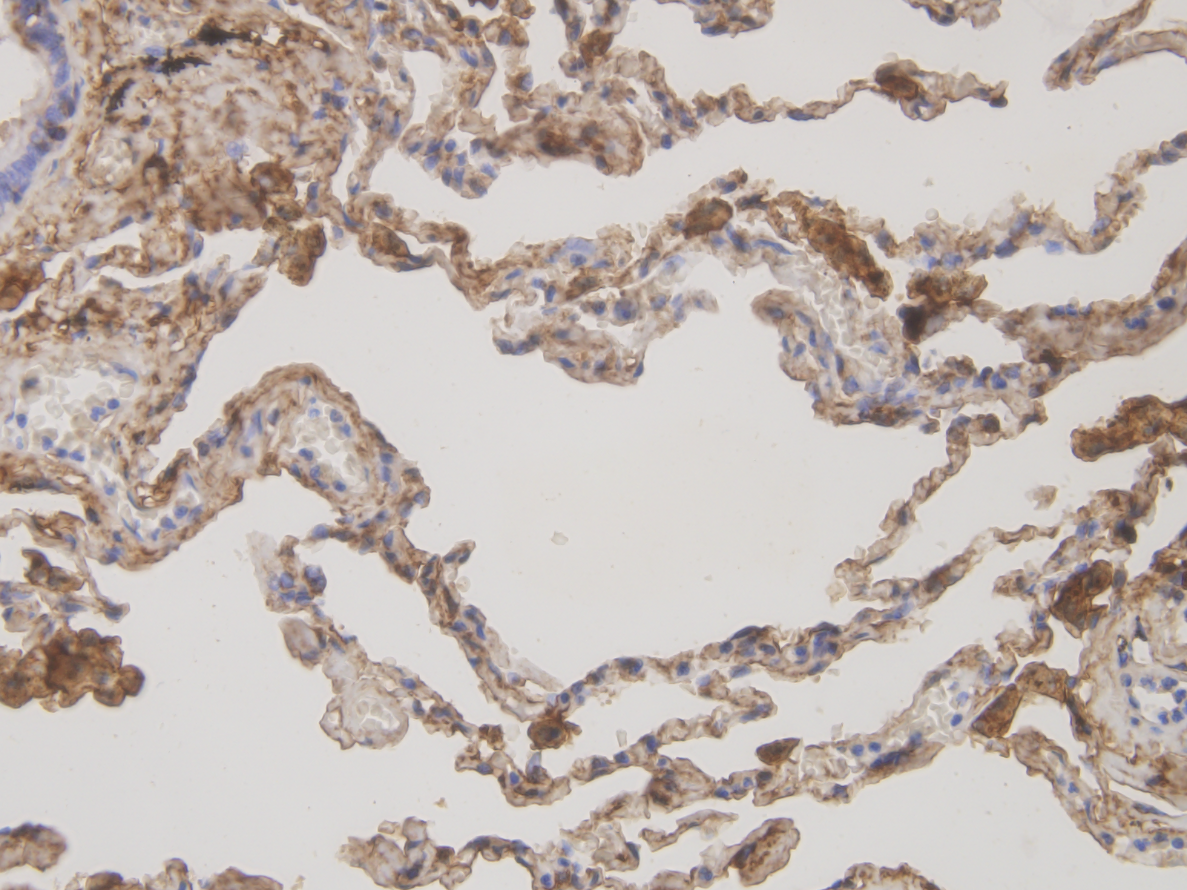

Supplement: S41 File — (ZIP) [file pone.0337223.s042.zip › 491617-400-CA-N/491617-400-N (3).tif]

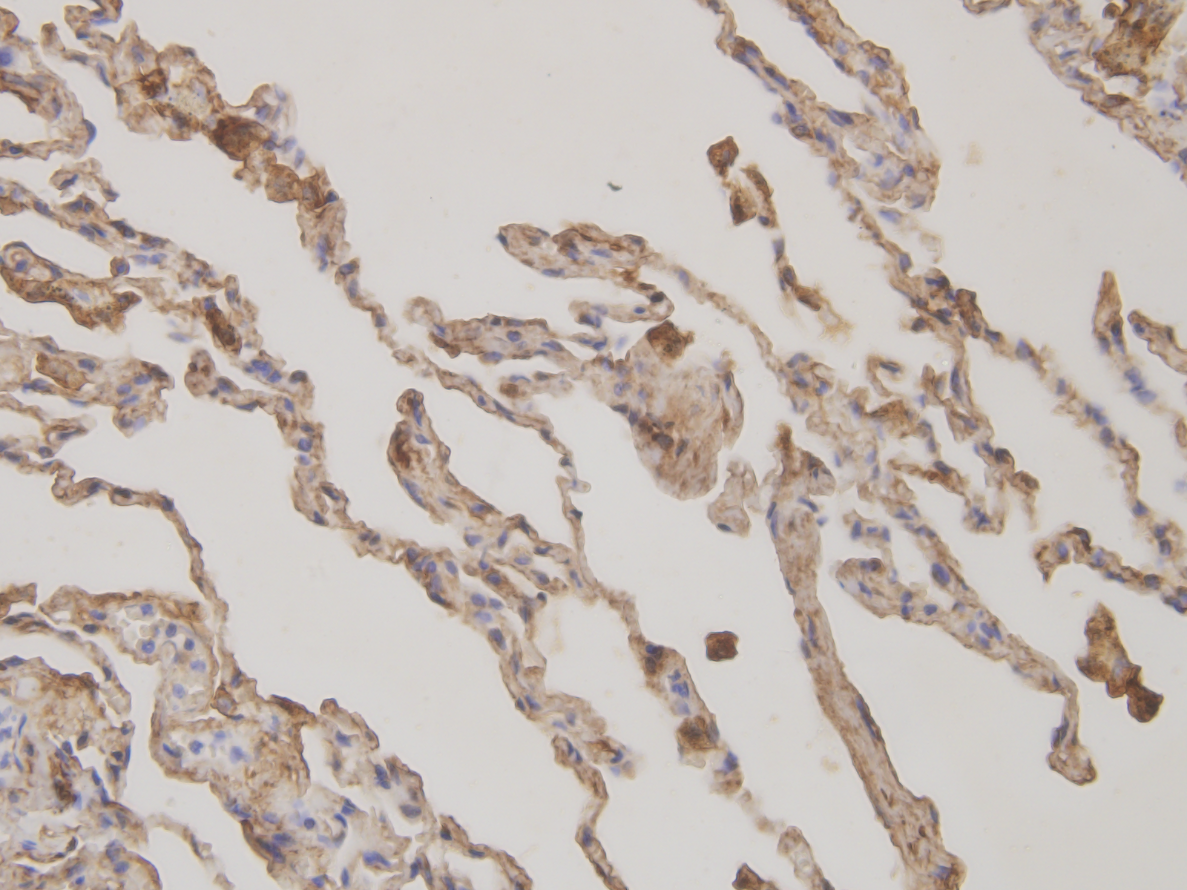

Supplement: S41 File — (ZIP) [file pone.0337223.s042.zip › 491617-400-CA-N/491617-400-N (4).tif]

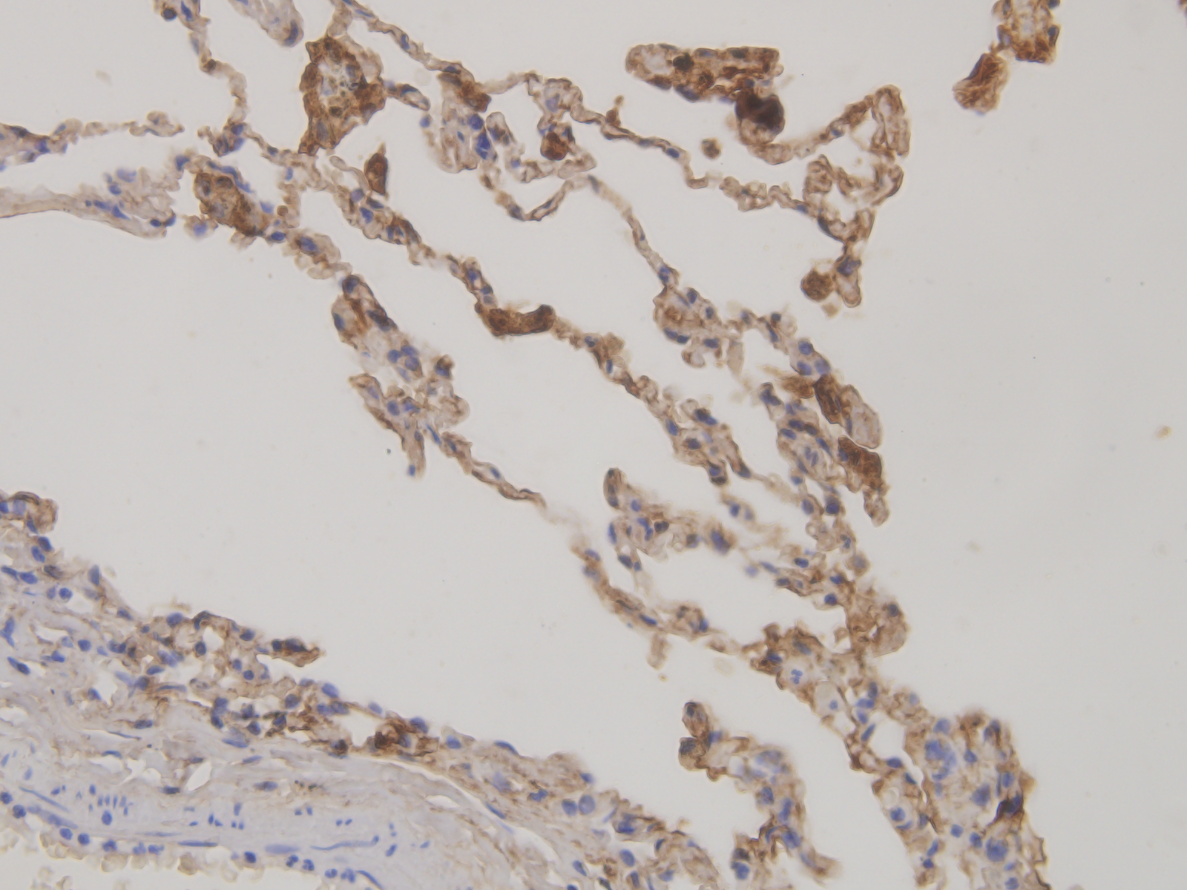

Supplement: S41 File — (ZIP) [file pone.0337223.s042.zip › 491617-400-CA-N/491617-400-N (5).tif]

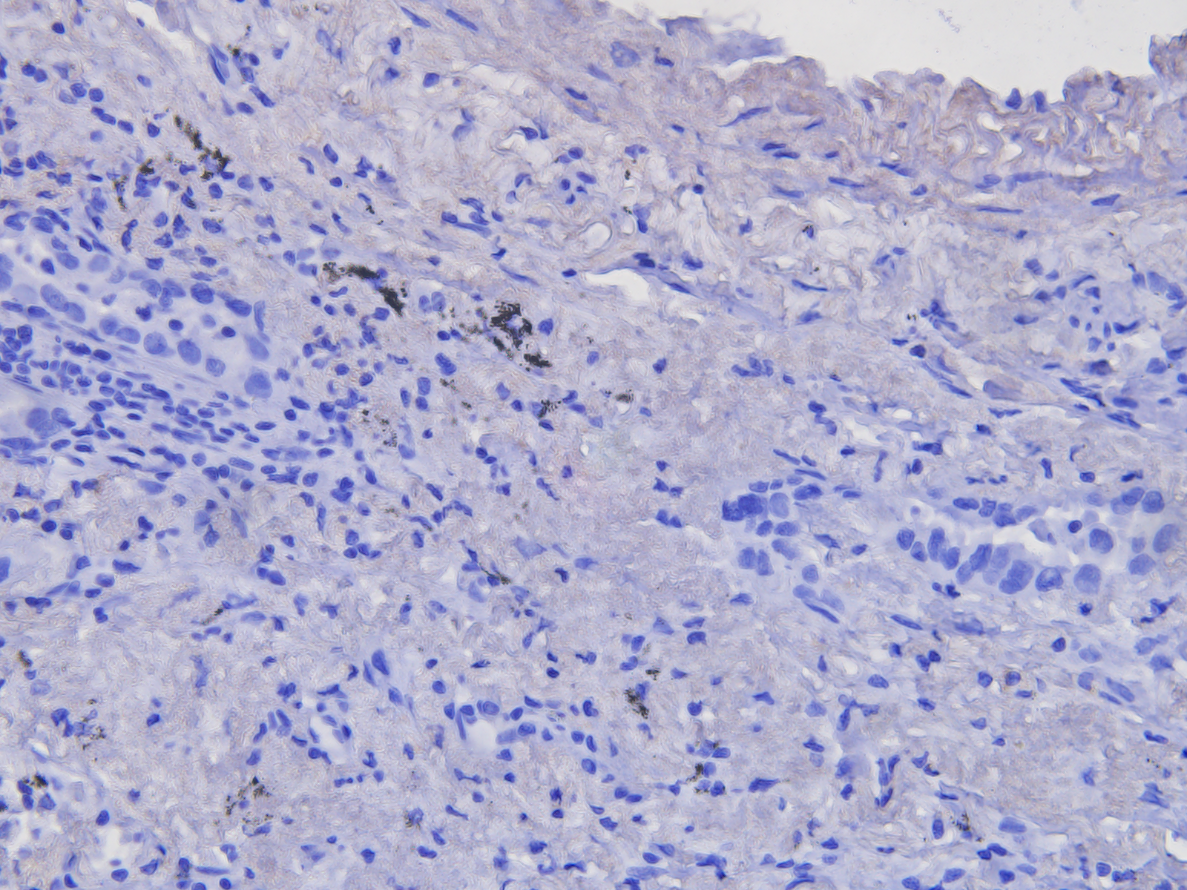

Supplement: S42 File — (ZIP) [file pone.0337223.s043.zip › 492150-400X-CA-N/492150-ca (1).tif]

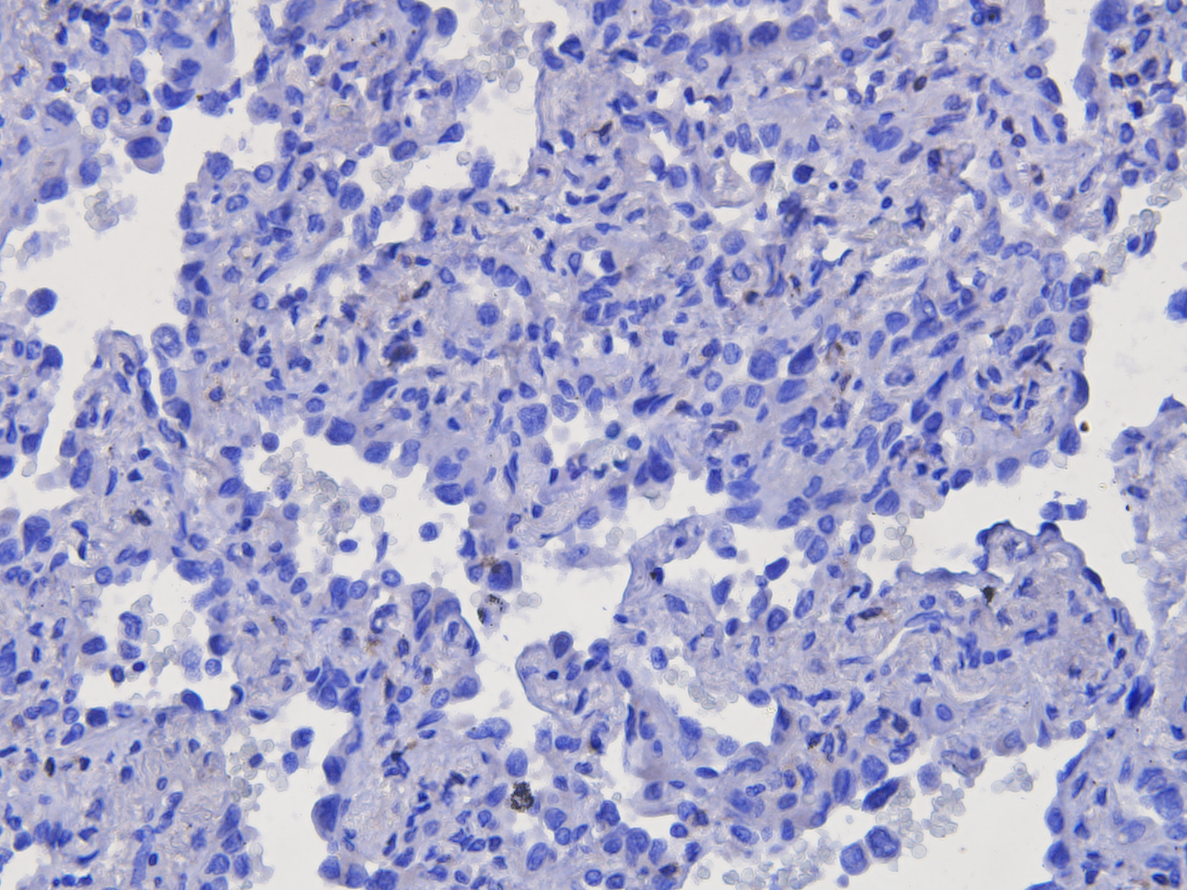

Supplement: S42 File — (ZIP) [file pone.0337223.s043.zip › 492150-400X-CA-N/492150-ca (2).tif]

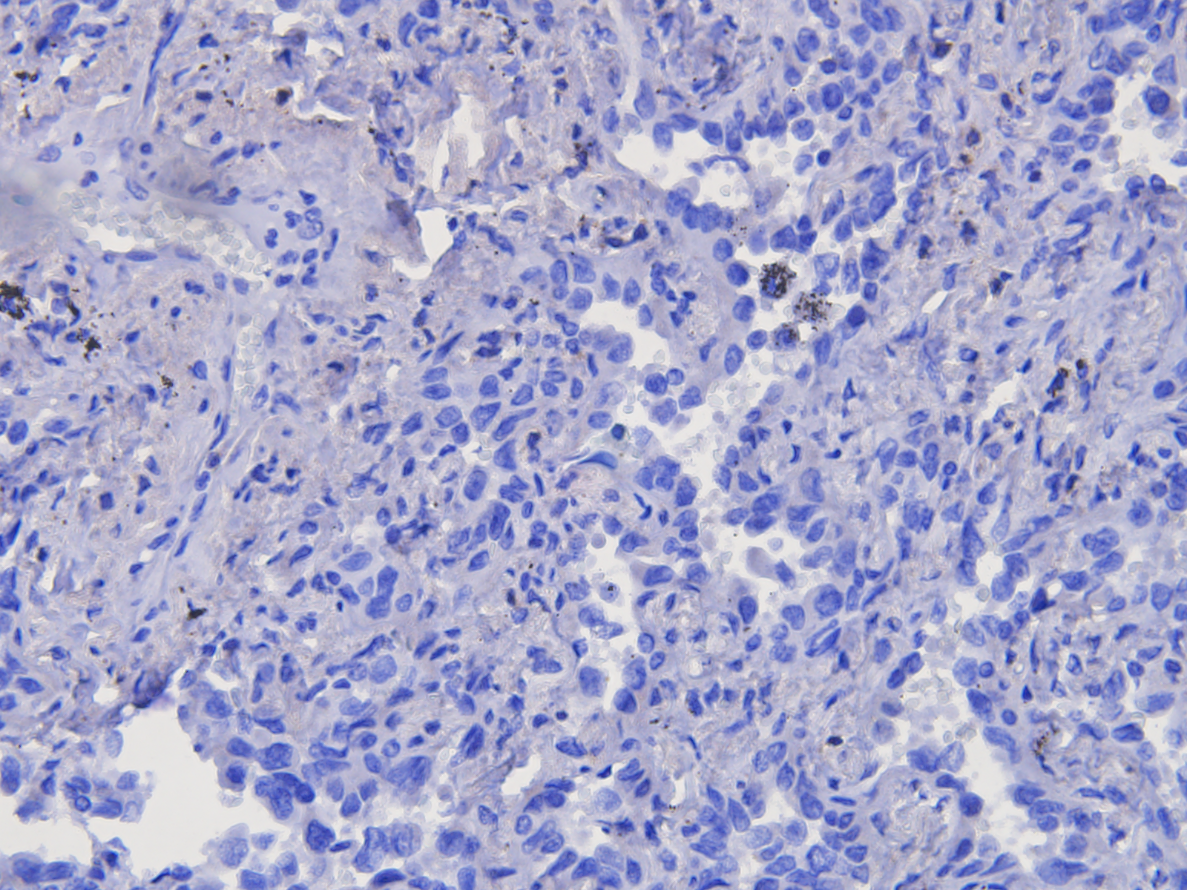

Supplement: S42 File — (ZIP) [file pone.0337223.s043.zip › 492150-400X-CA-N/492150-ca (3).tif]

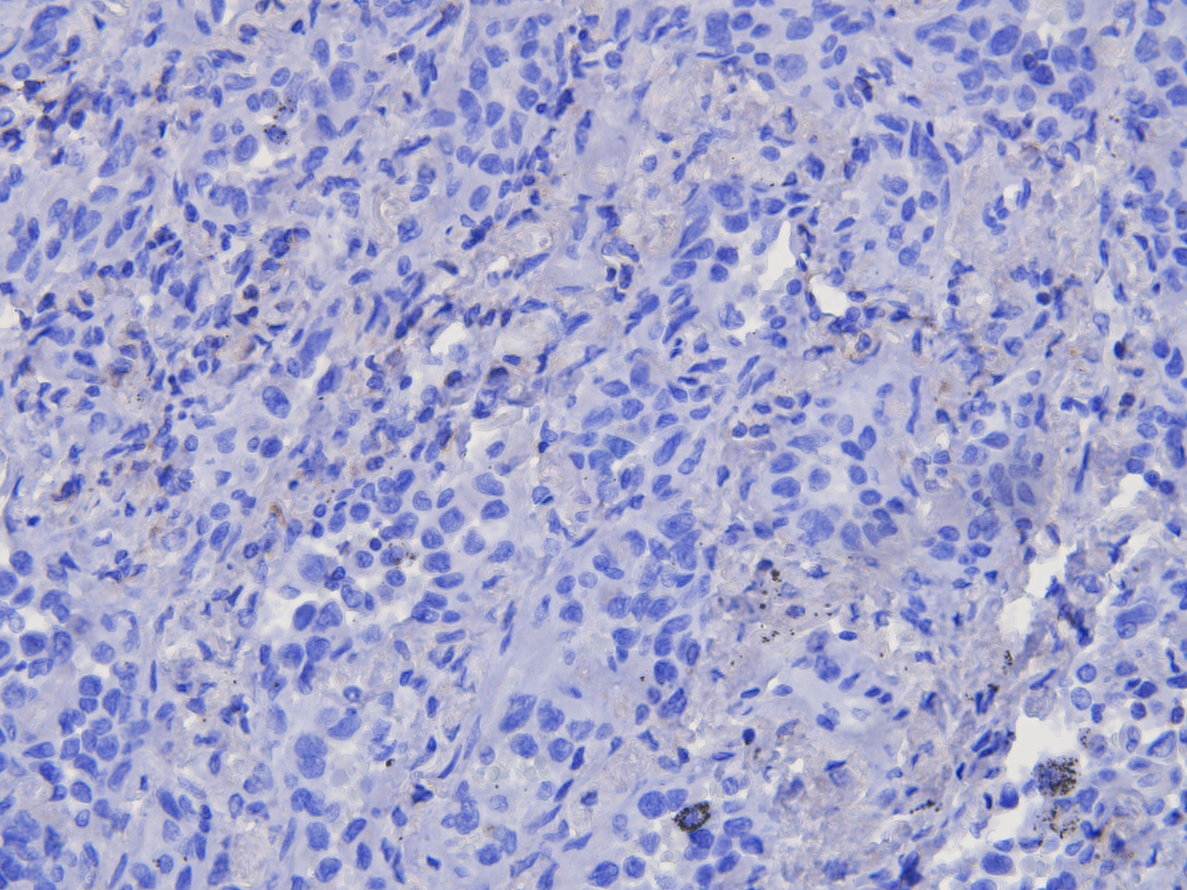

Supplement: S42 File — (ZIP) [file pone.0337223.s043.zip › 492150-400X-CA-N/492150-ca (4).tif]

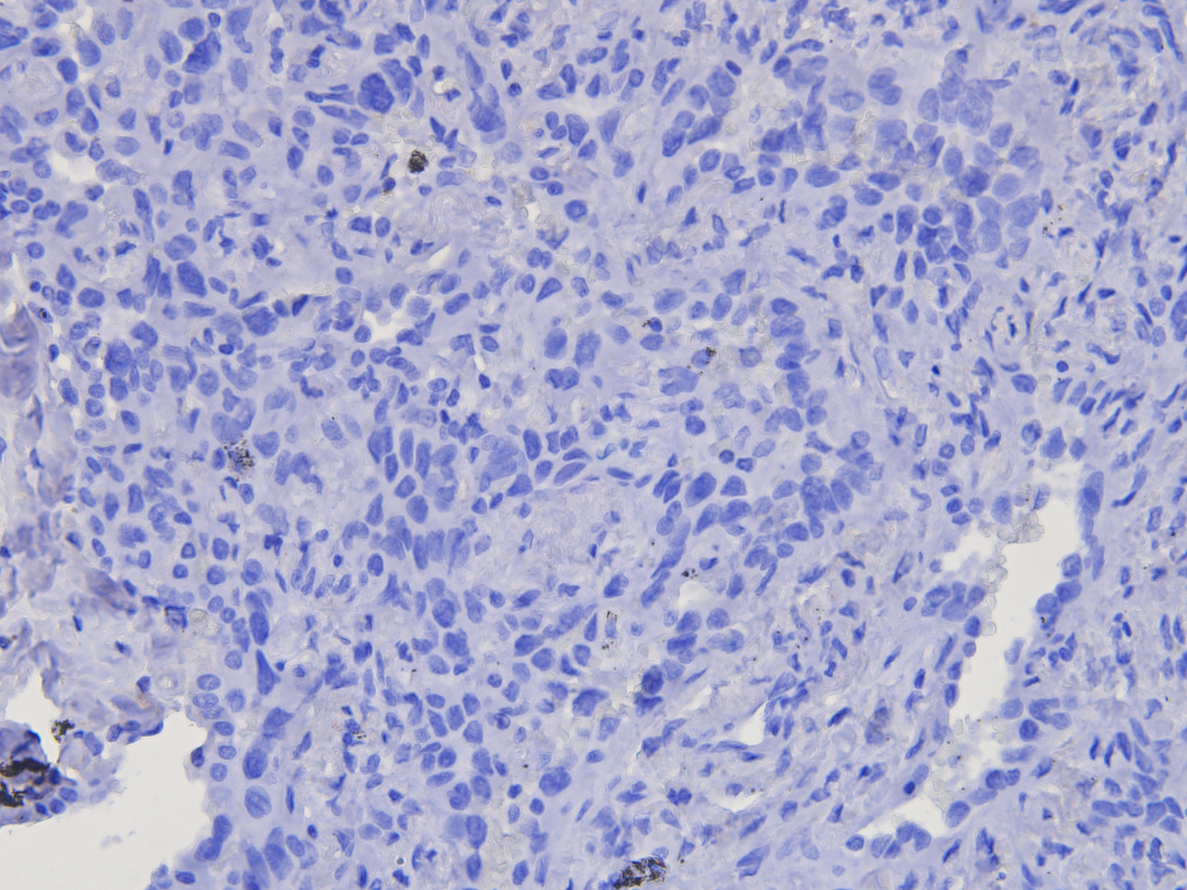

Supplement: S42 File — (ZIP) [file pone.0337223.s043.zip › 492150-400X-CA-N/492150-ca (5).tif]

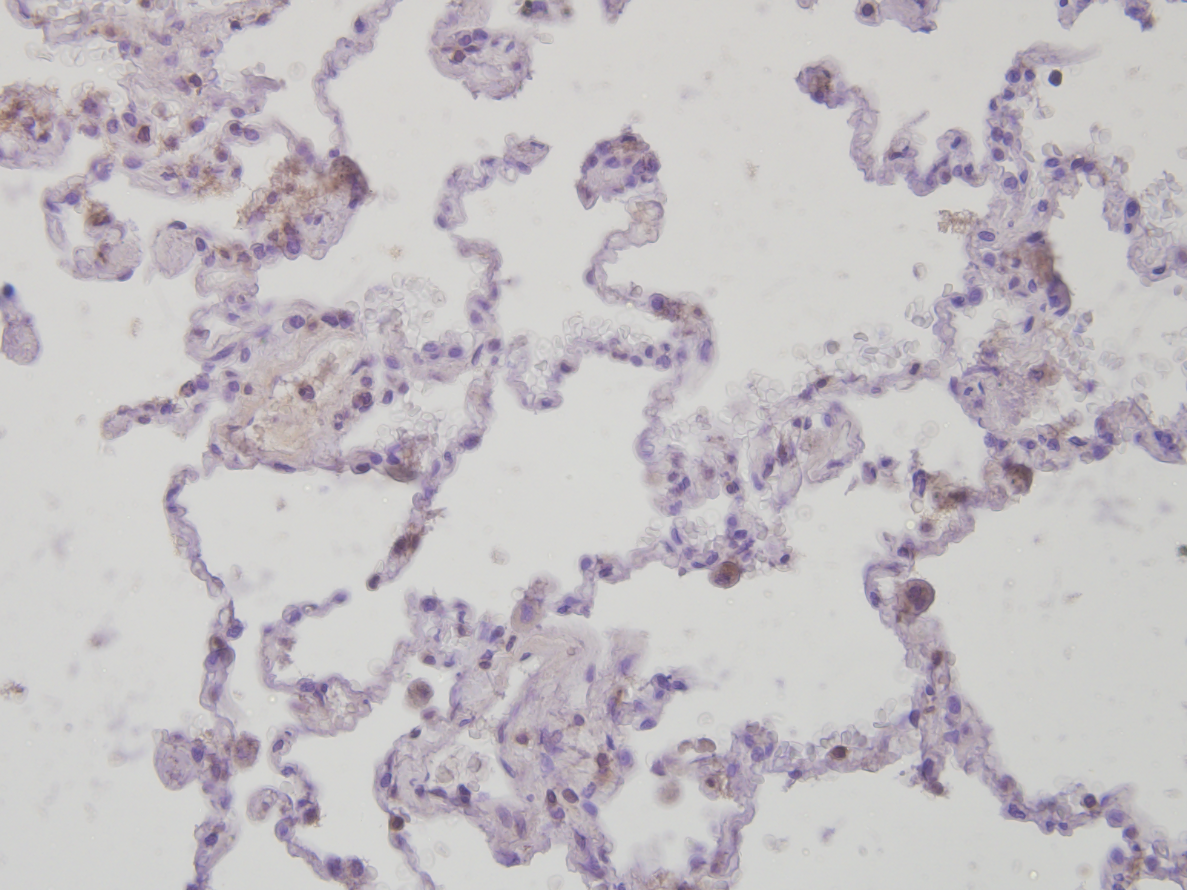

Supplement: S42 File — (ZIP) [file pone.0337223.s043.zip › 492150-400X-CA-N/492150-n (1).tif]

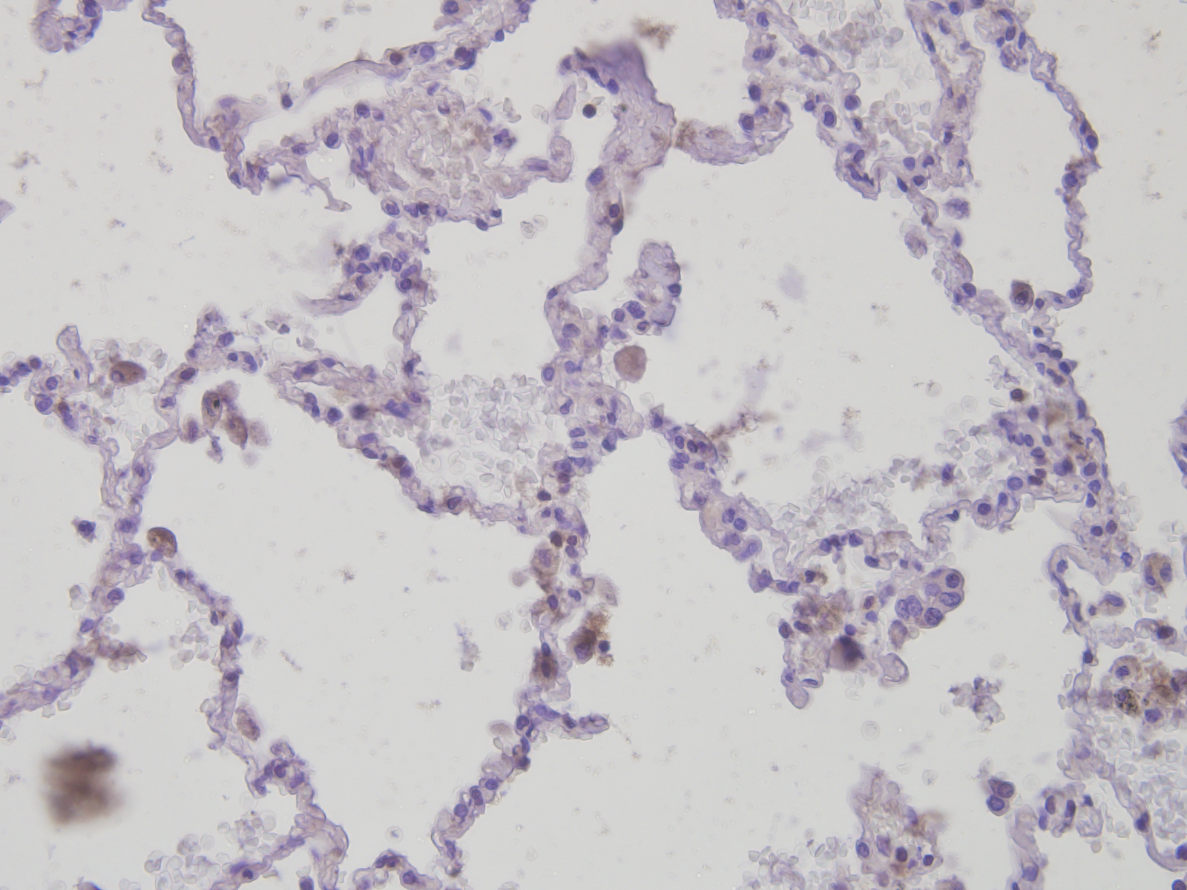

Supplement: S42 File — (ZIP) [file pone.0337223.s043.zip › 492150-400X-CA-N/492150-n (2).tif]

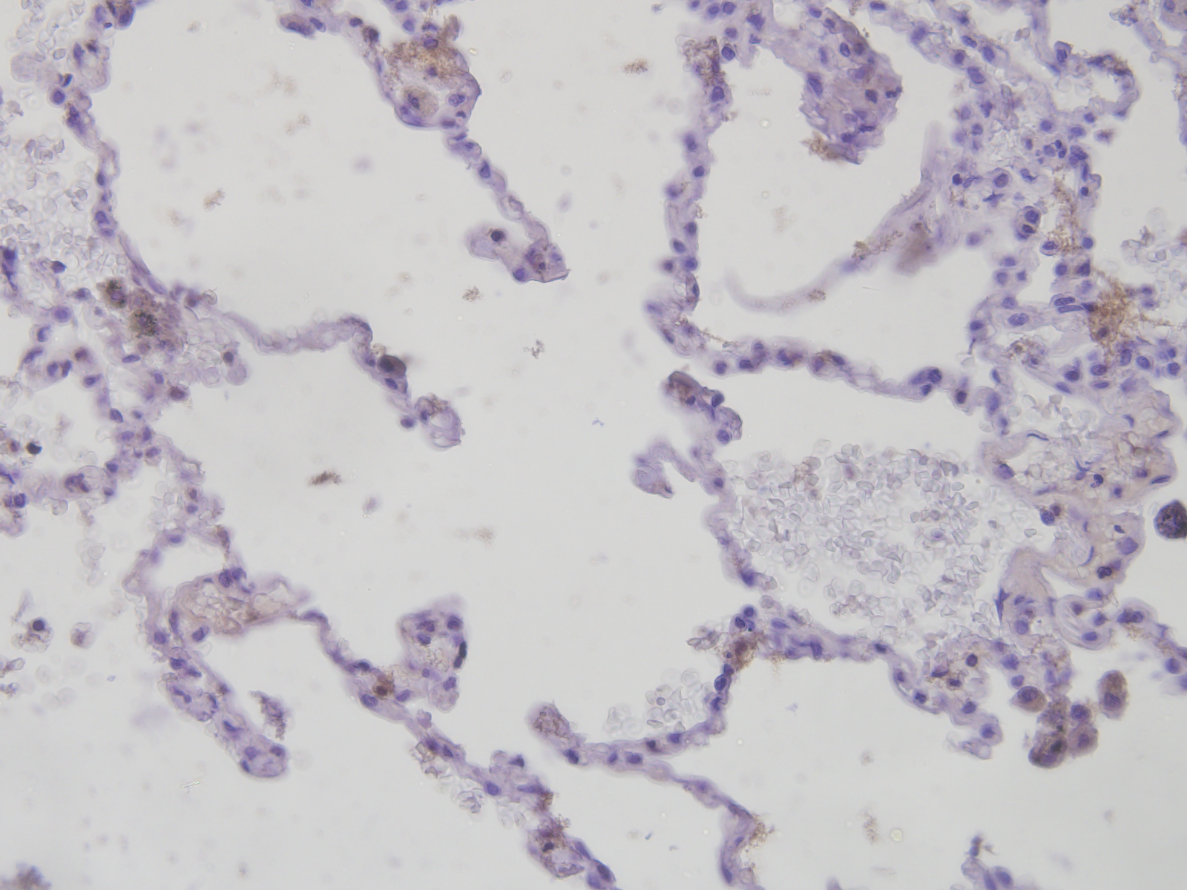

Supplement: S42 File — (ZIP) [file pone.0337223.s043.zip › 492150-400X-CA-N/492150-n (3).tif]

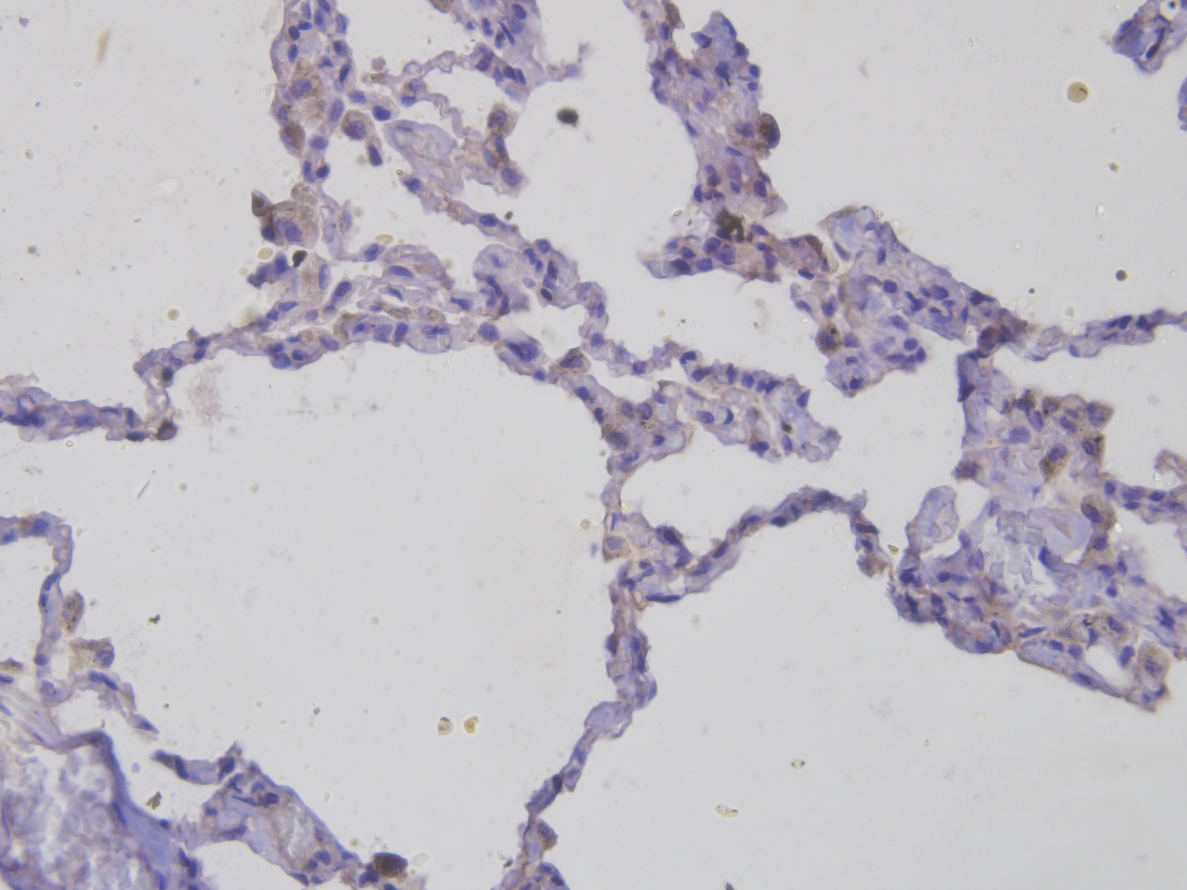

Supplement: S42 File — (ZIP) [file pone.0337223.s043.zip › 492150-400X-CA-N/492150-n (4).tif]

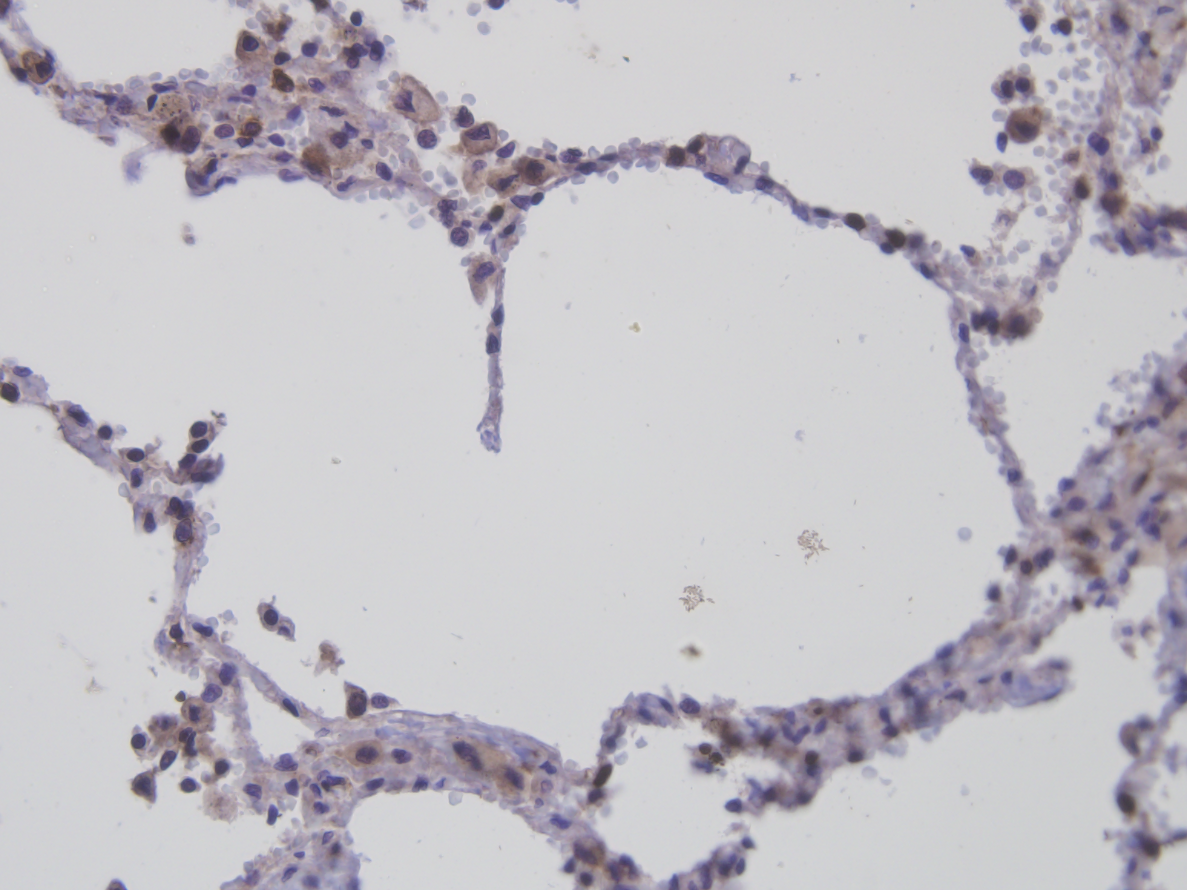

Supplement: S42 File — (ZIP) [file pone.0337223.s043.zip › 492150-400X-CA-N/492150-n (5).tif]

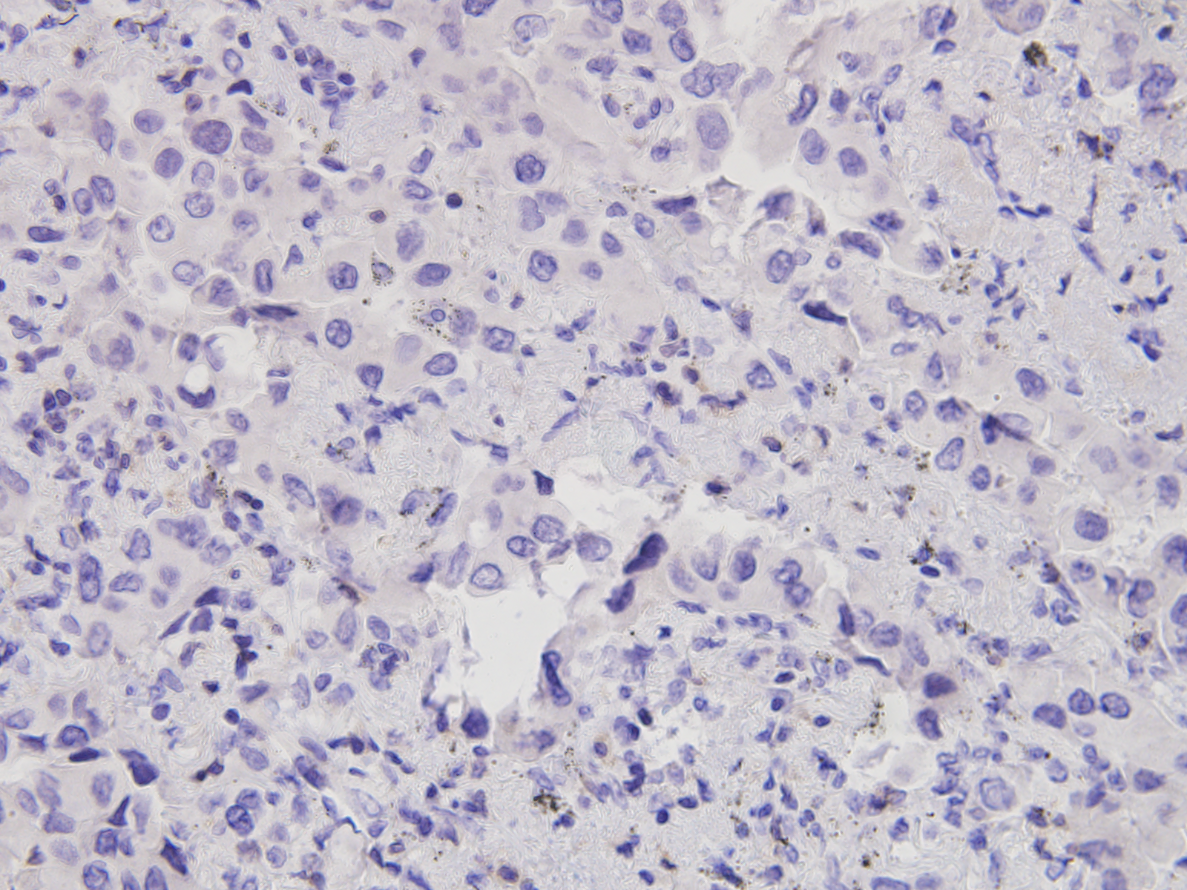

Supplement: S43 File — (ZIP) [file pone.0337223.s044.zip › 496547-400X-CA-N/496547-400X-CA (1).tif]

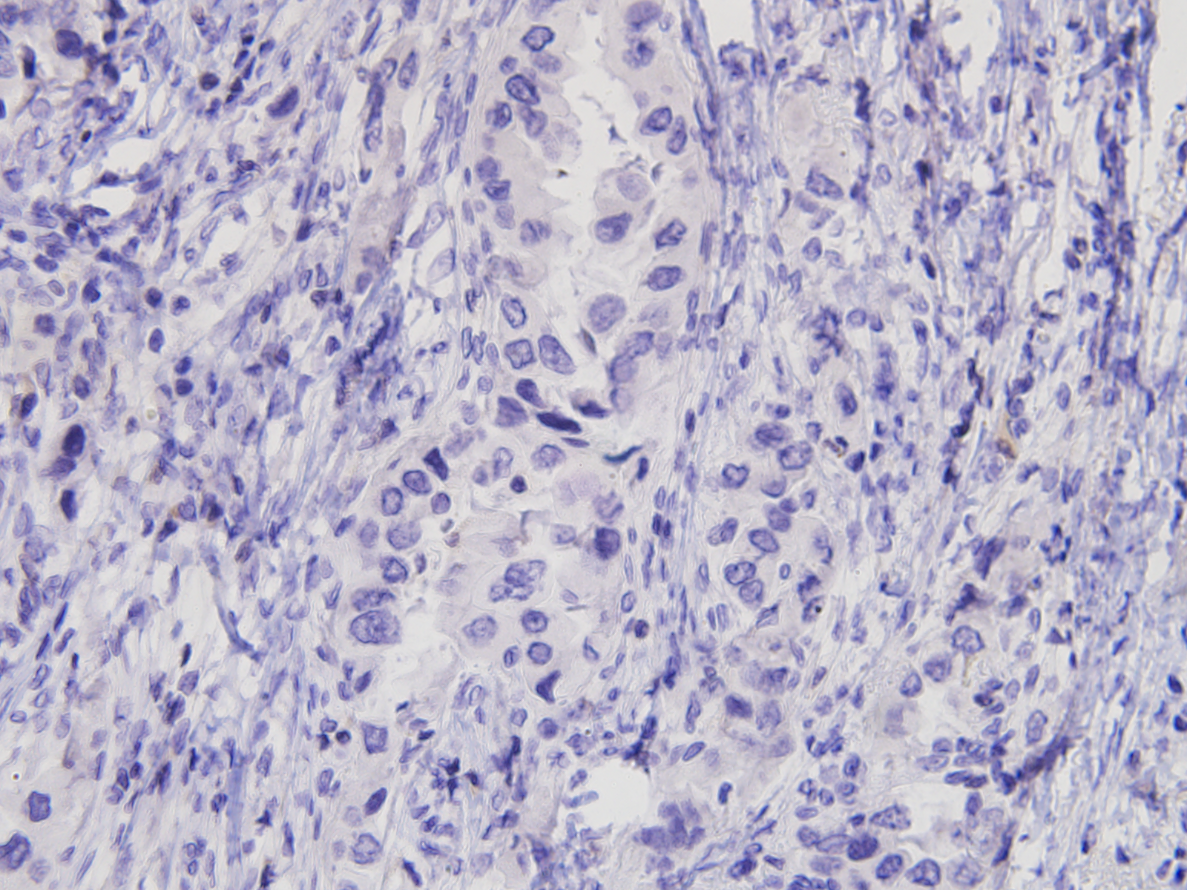

Supplement: S43 File — (ZIP) [file pone.0337223.s044.zip › 496547-400X-CA-N/496547-400X-CA (2).tif]

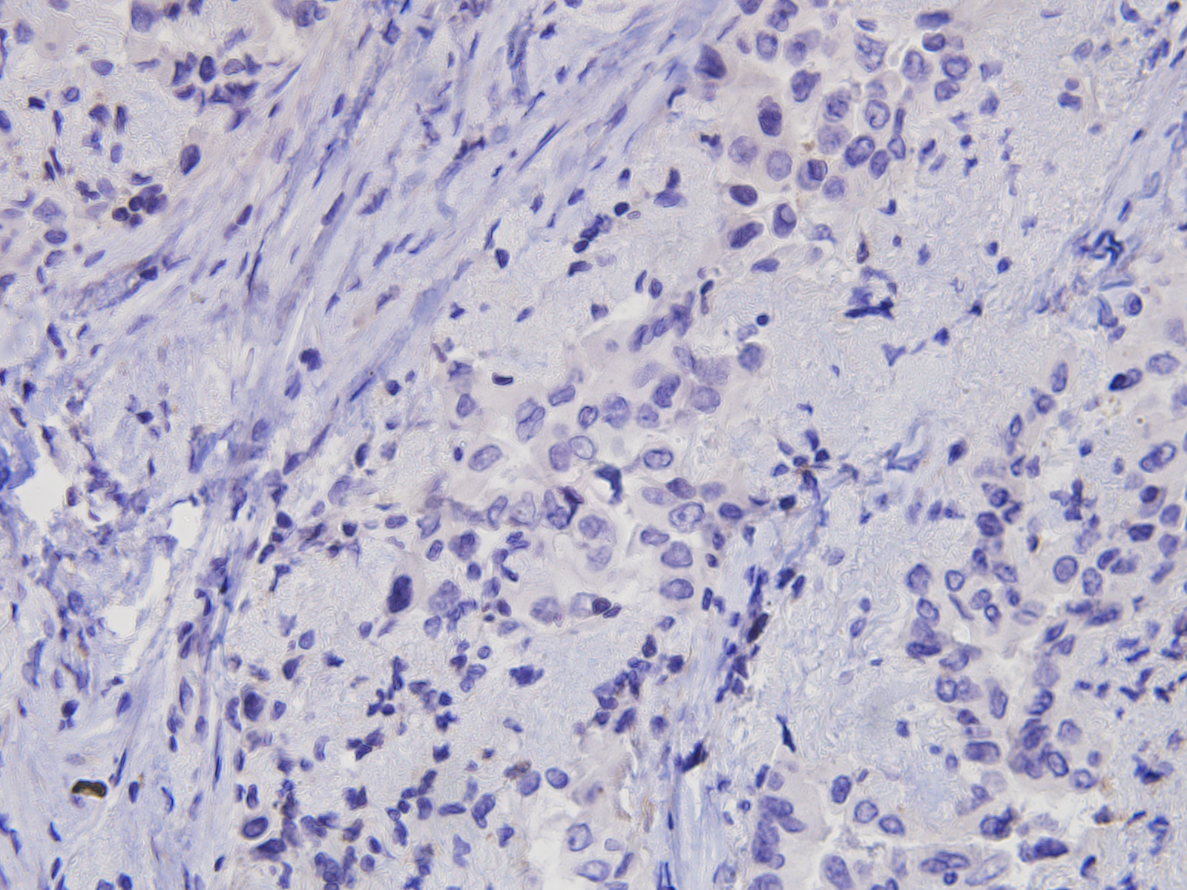

Supplement: S43 File — (ZIP) [file pone.0337223.s044.zip › 496547-400X-CA-N/496547-400X-CA (3).tif]

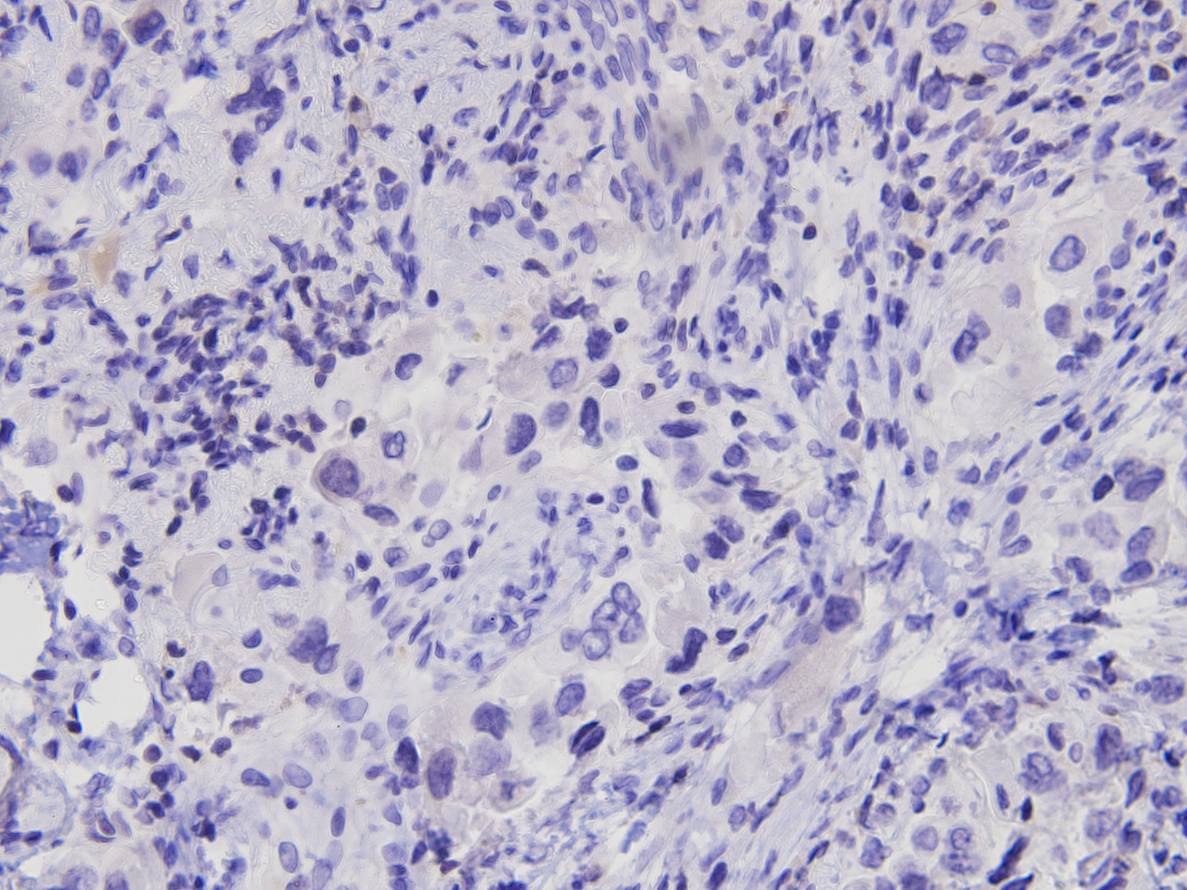

Supplement: S43 File — (ZIP) [file pone.0337223.s044.zip › 496547-400X-CA-N/496547-400X-CA (4).tif]

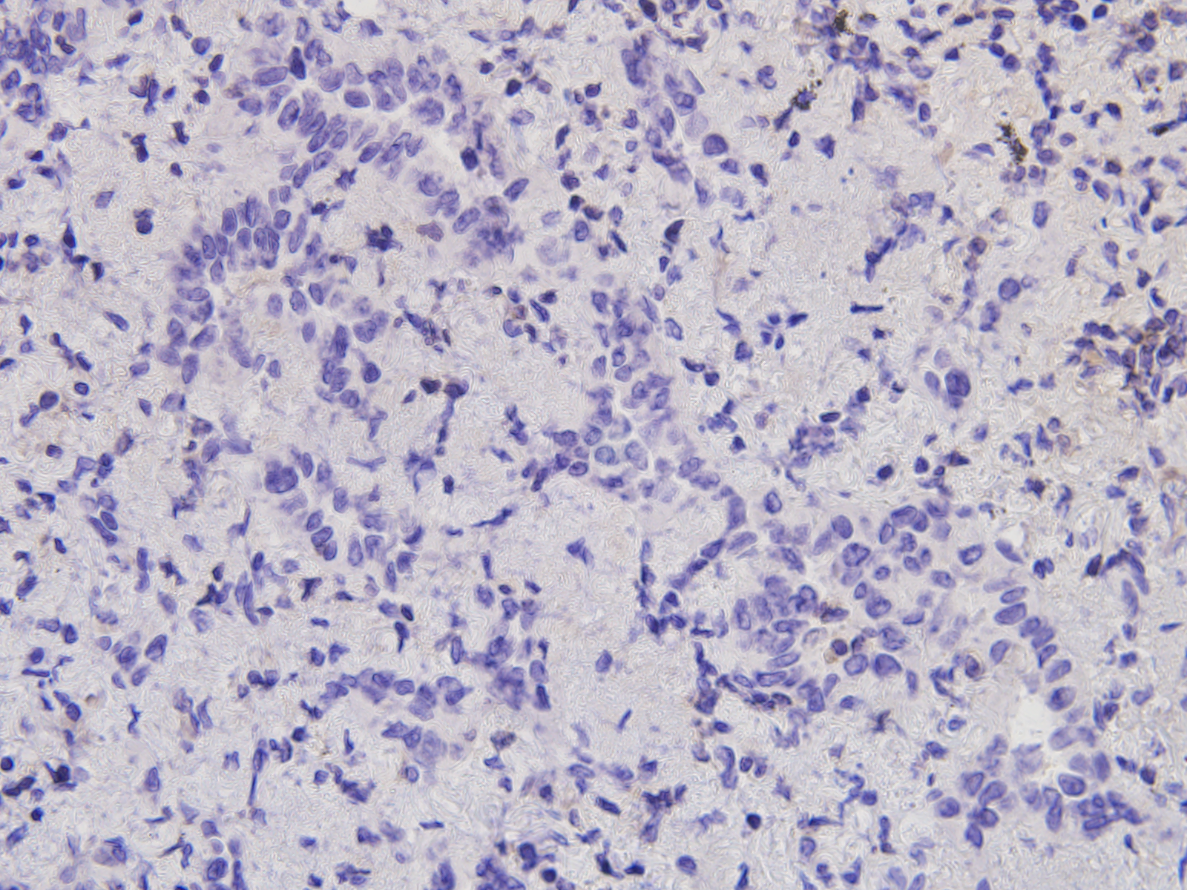

Supplement: S43 File — (ZIP) [file pone.0337223.s044.zip › 496547-400X-CA-N/496547-400X-CA (5).tif]

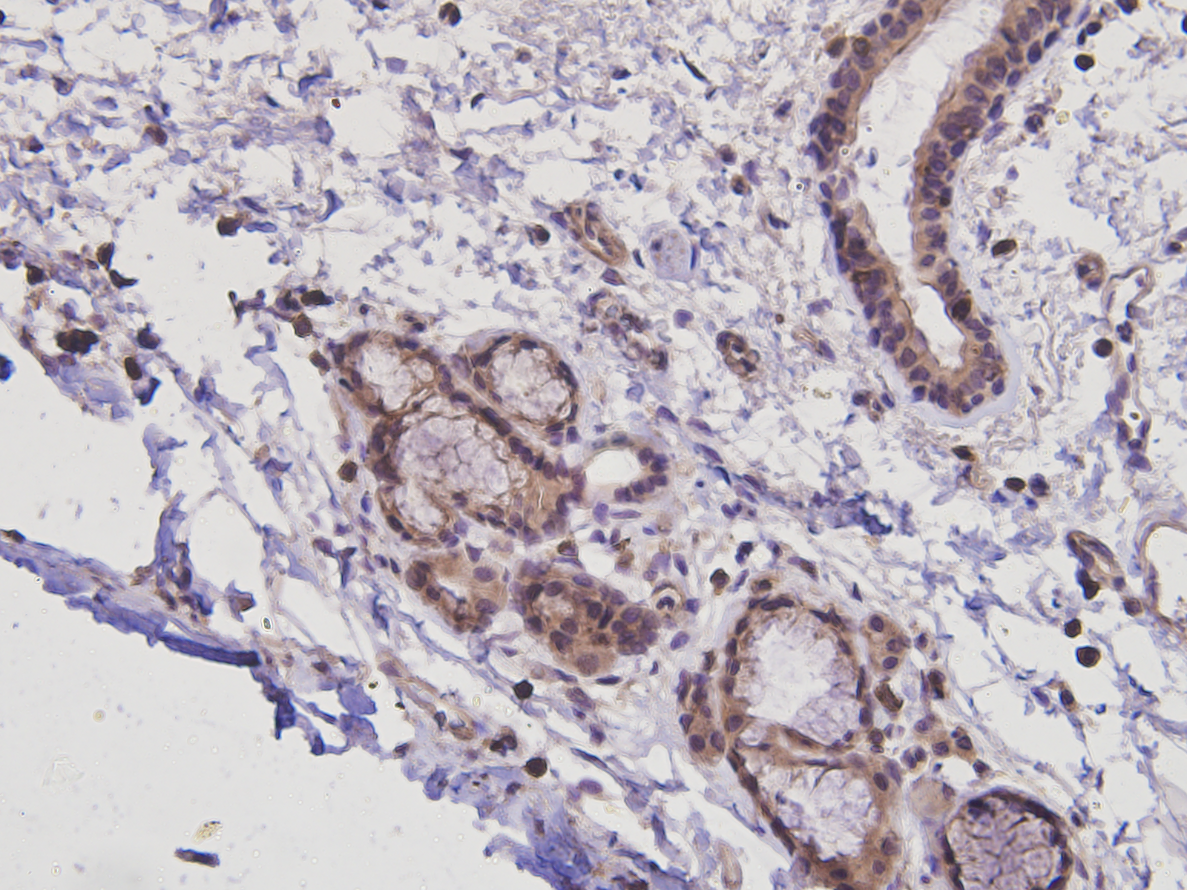

Supplement: S43 File — (ZIP) [file pone.0337223.s044.zip › 496547-400X-CA-N/496547-400X-N (1).tif]

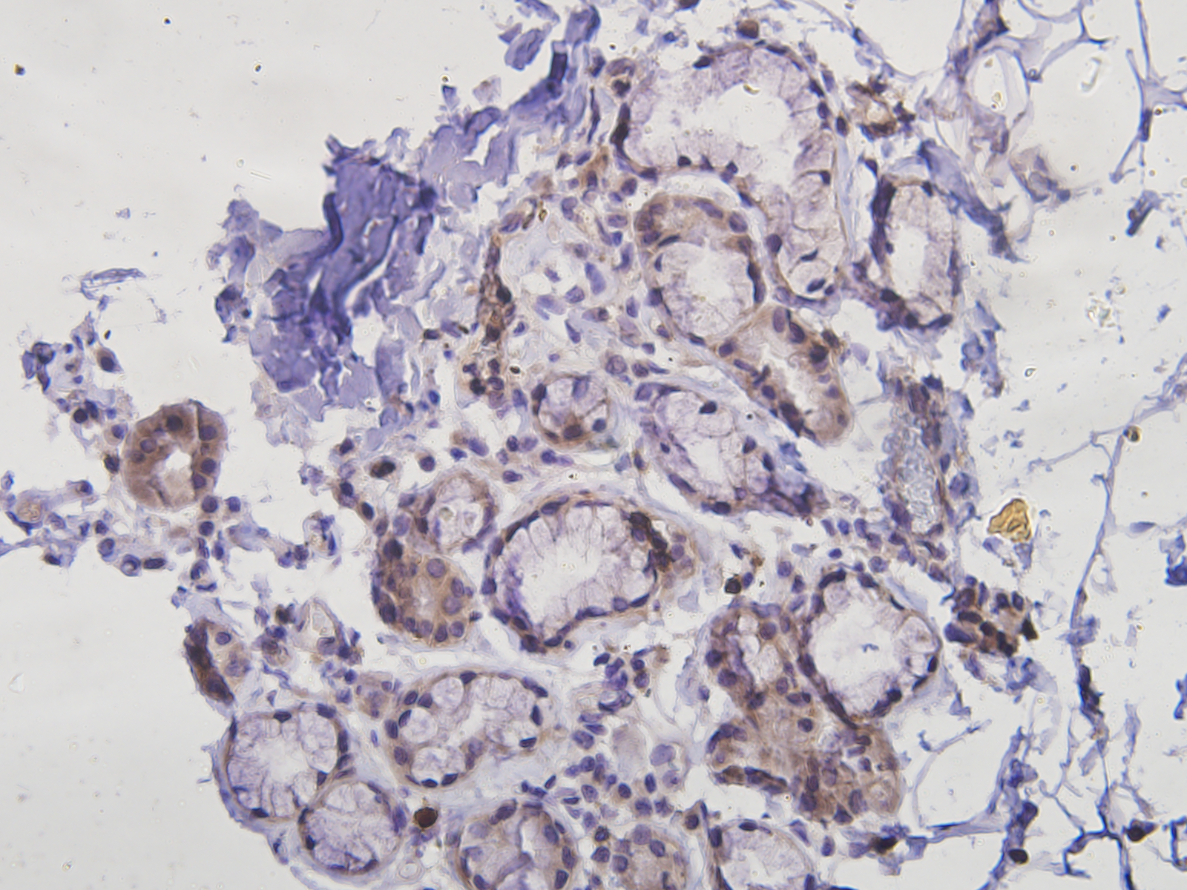

Supplement: S43 File — (ZIP) [file pone.0337223.s044.zip › 496547-400X-CA-N/496547-400X-N (2).tif]

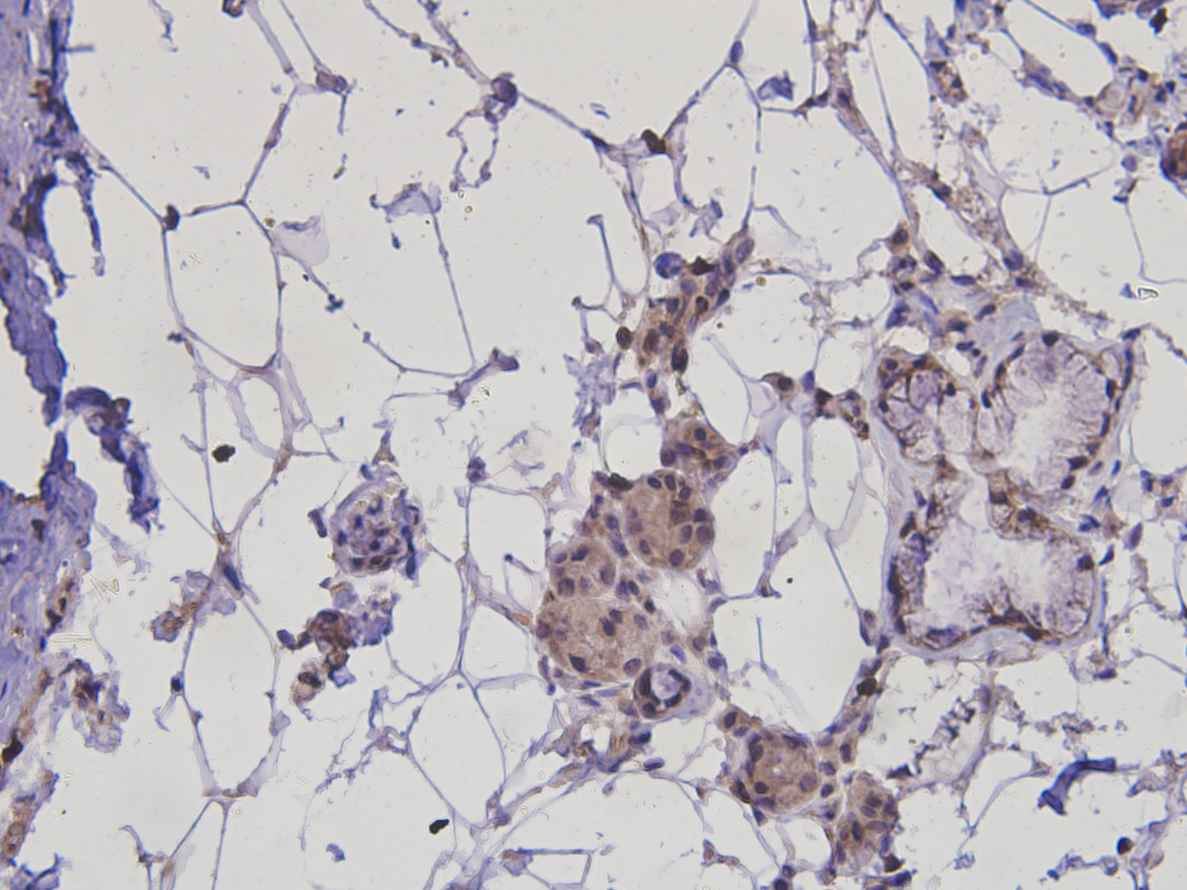

Supplement: S43 File — (ZIP) [file pone.0337223.s044.zip › 496547-400X-CA-N/496547-400X-N (3).tif]

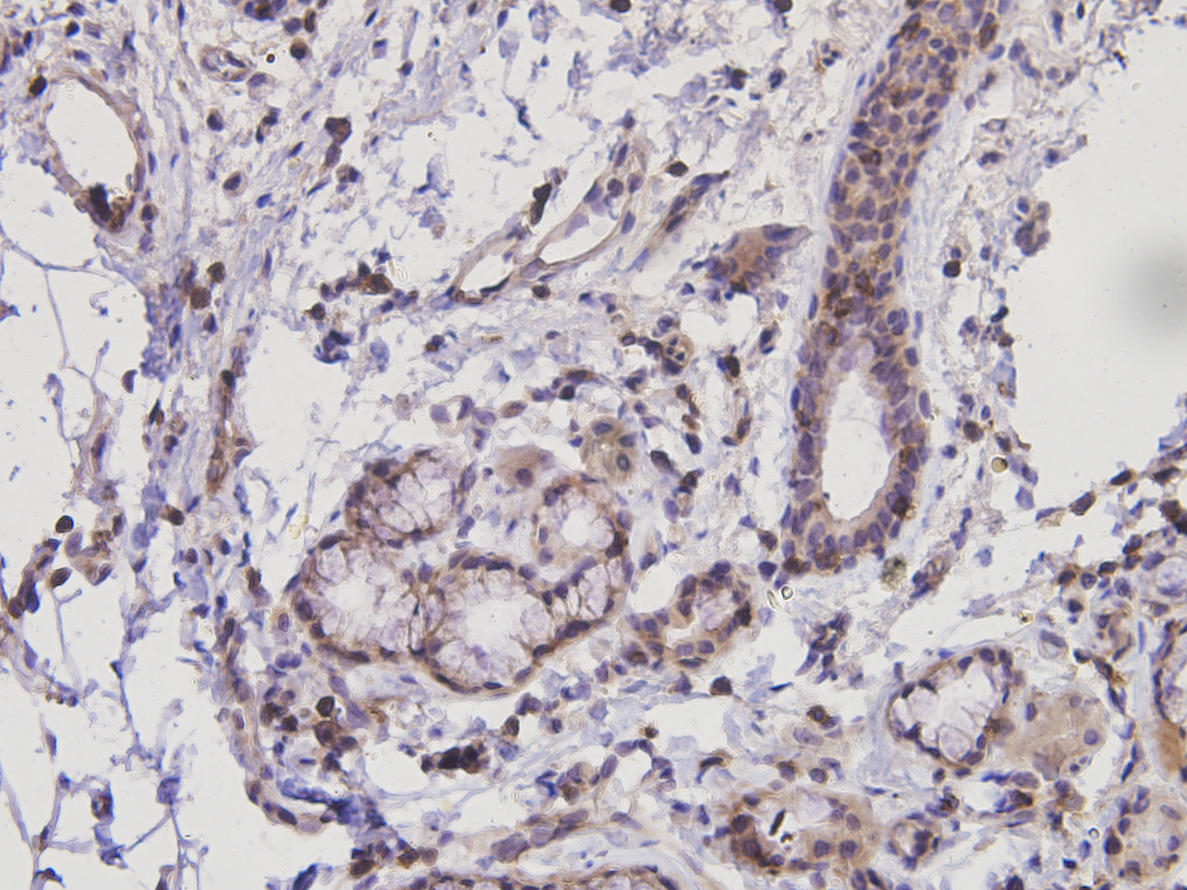

Supplement: S43 File — (ZIP) [file pone.0337223.s044.zip › 496547-400X-CA-N/496547-400X-N (4).tif]

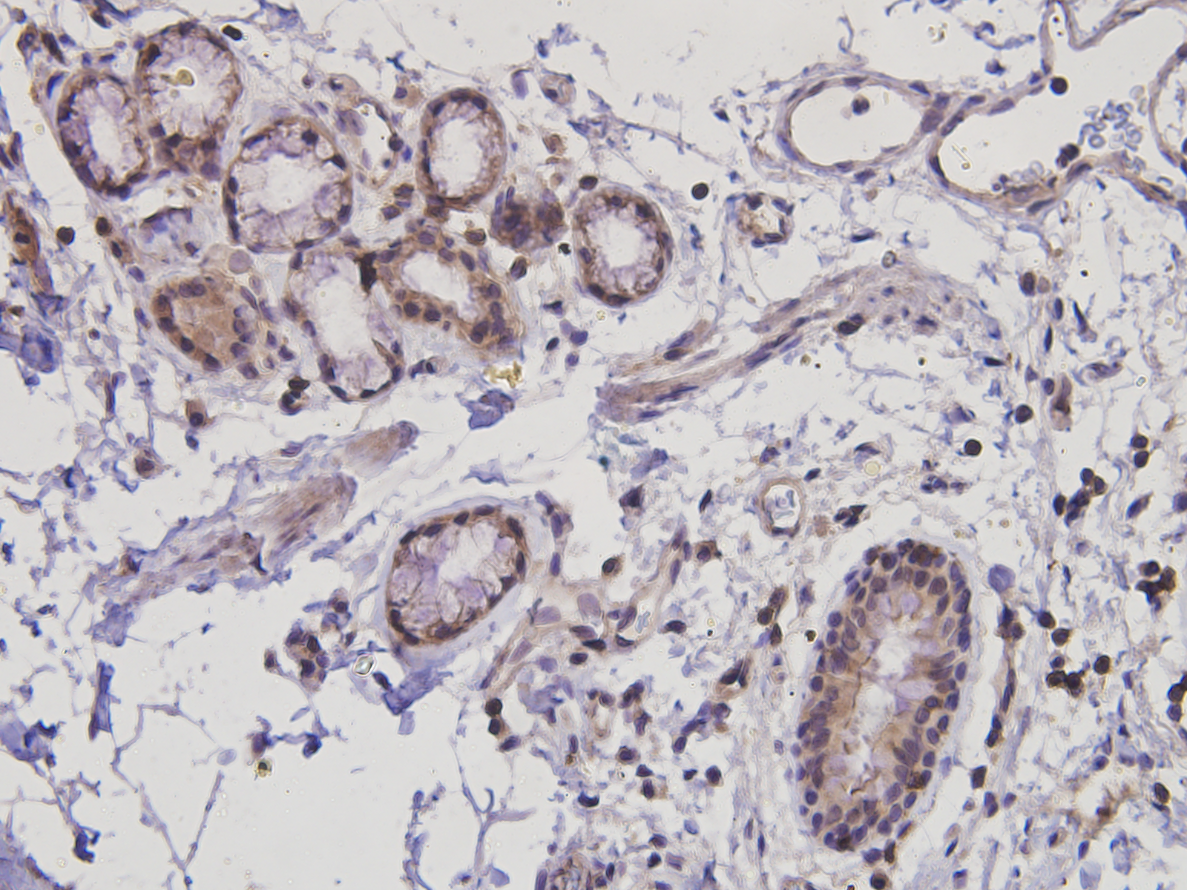

Supplement: S43 File — (ZIP) [file pone.0337223.s044.zip › 496547-400X-CA-N/496547-400X-N (5).tif]

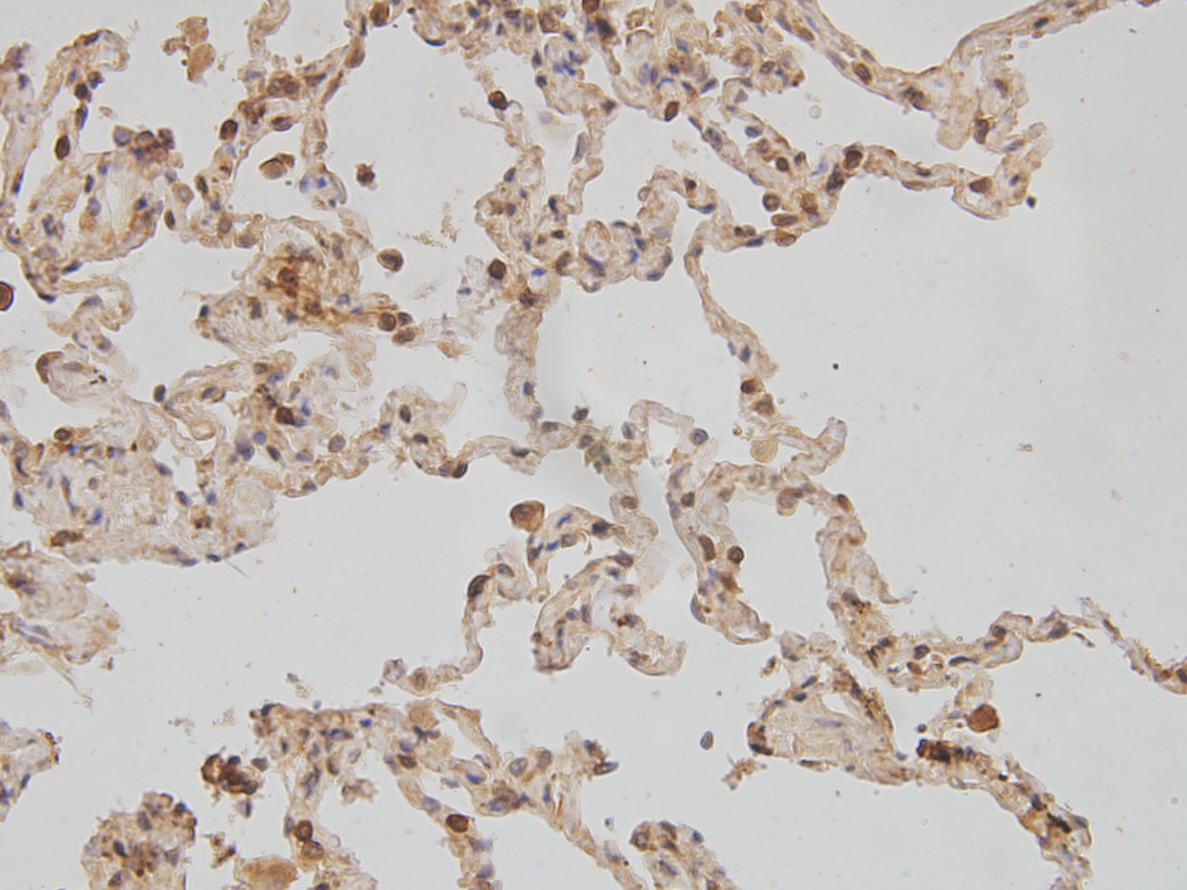

Supplement: S44 File — (ZIP) [file pone.0337223.s045.zip › 500857-400X-CA-N/500857-400X--N (1).tif]

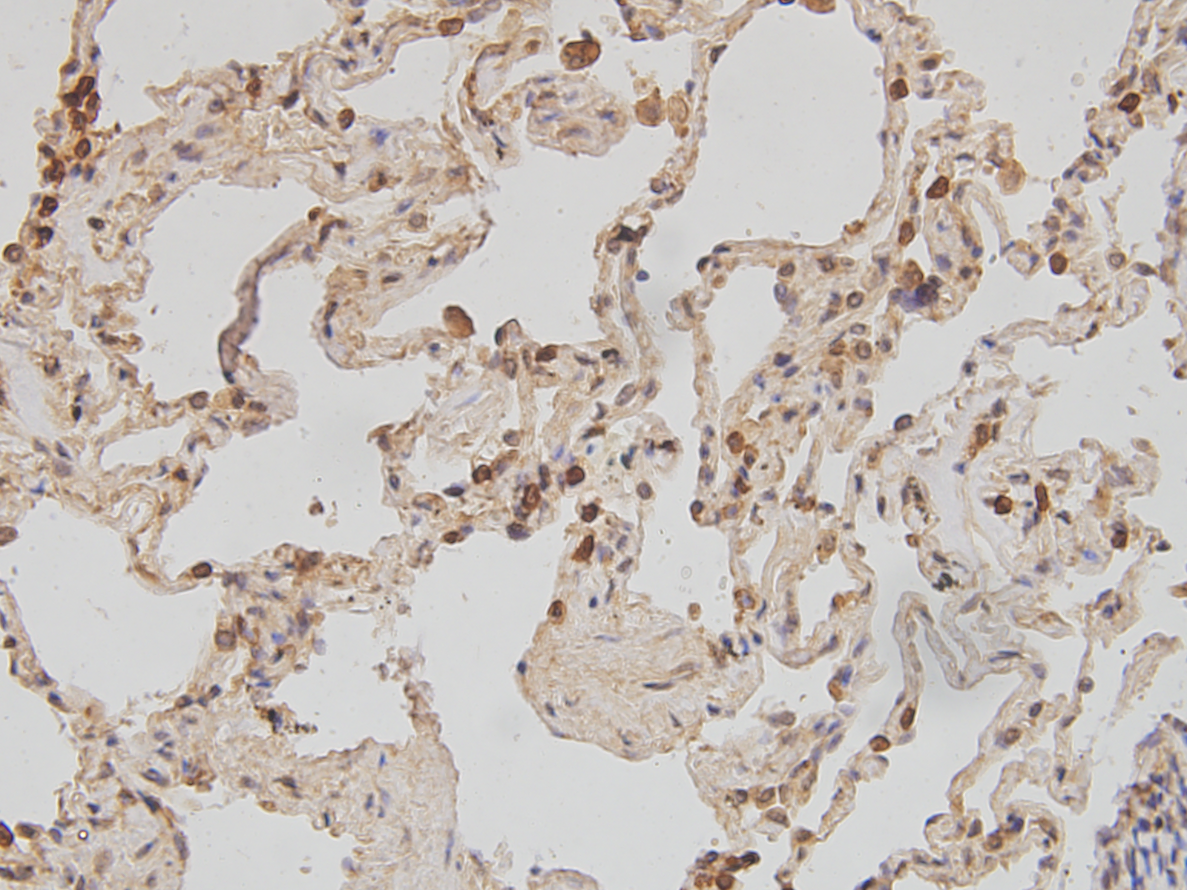

Supplement: S44 File — (ZIP) [file pone.0337223.s045.zip › 500857-400X-CA-N/500857-400X--N (2).tif]

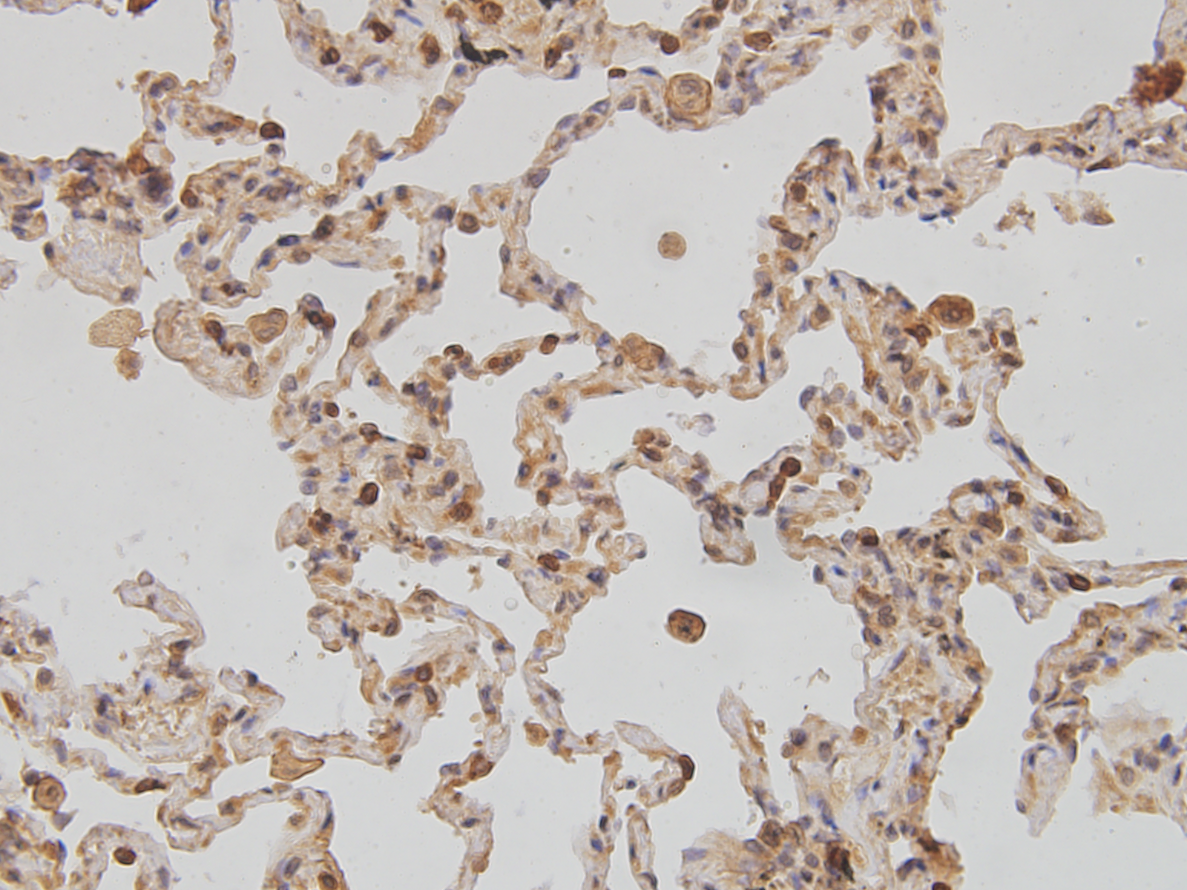

Supplement: S44 File — (ZIP) [file pone.0337223.s045.zip › 500857-400X-CA-N/500857-400X--N (3).tif]

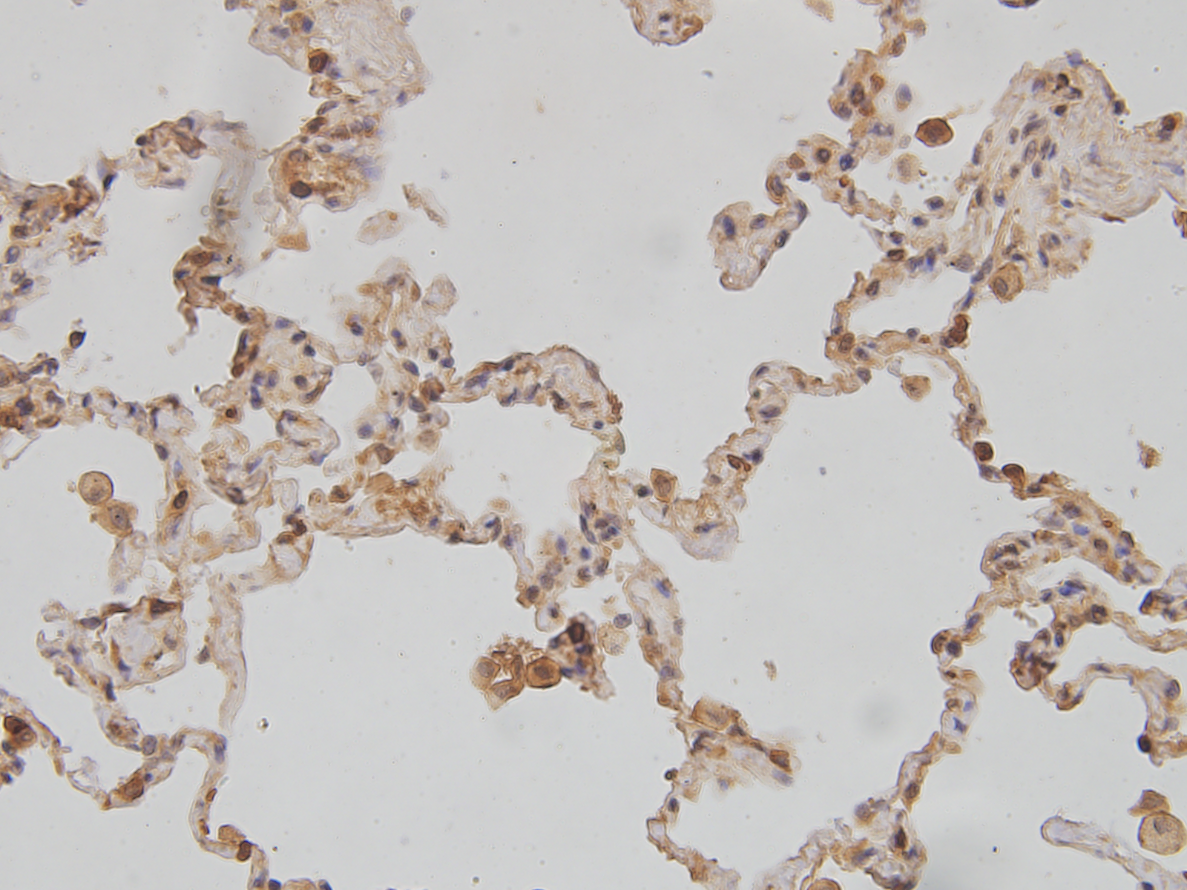

Supplement: S44 File — (ZIP) [file pone.0337223.s045.zip › 500857-400X-CA-N/500857-400X--N (4).tif]

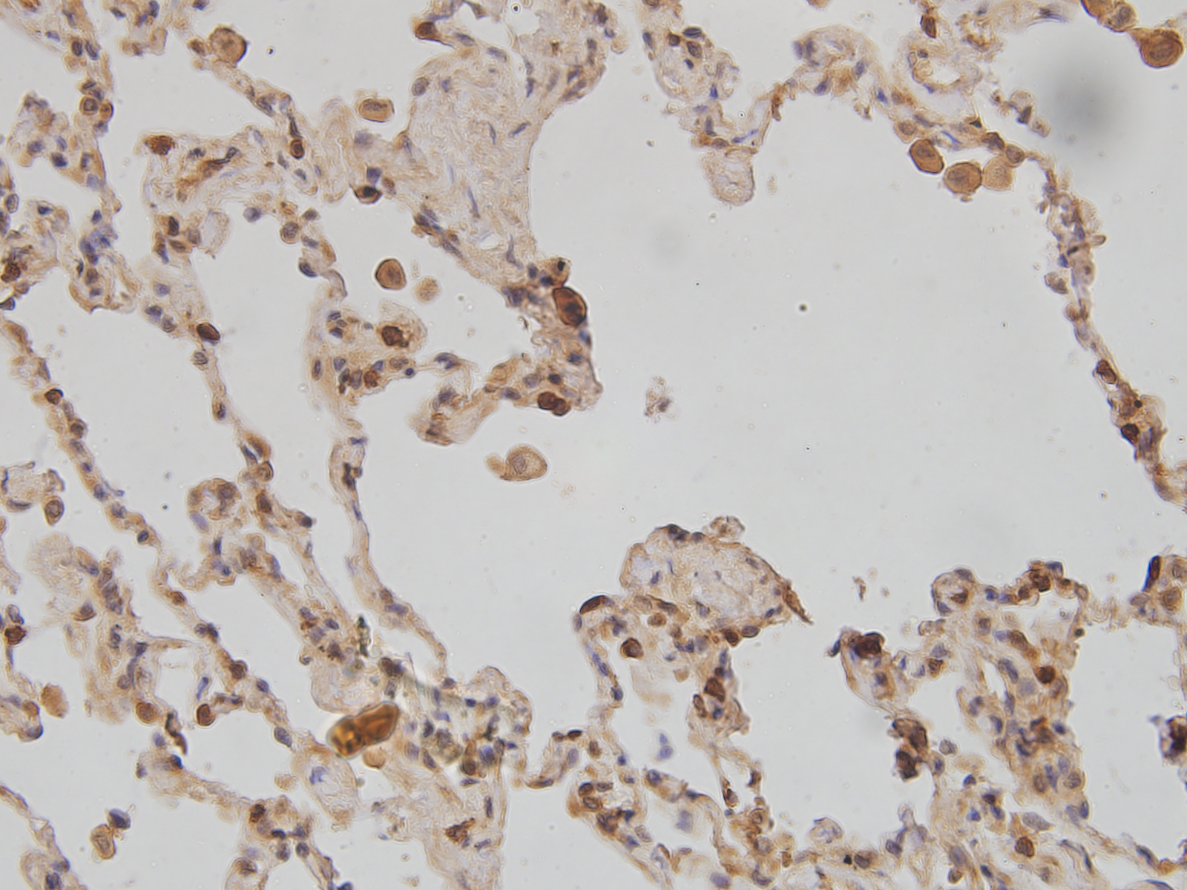

Supplement: S44 File — (ZIP) [file pone.0337223.s045.zip › 500857-400X-CA-N/500857-400X--N (5).tif]

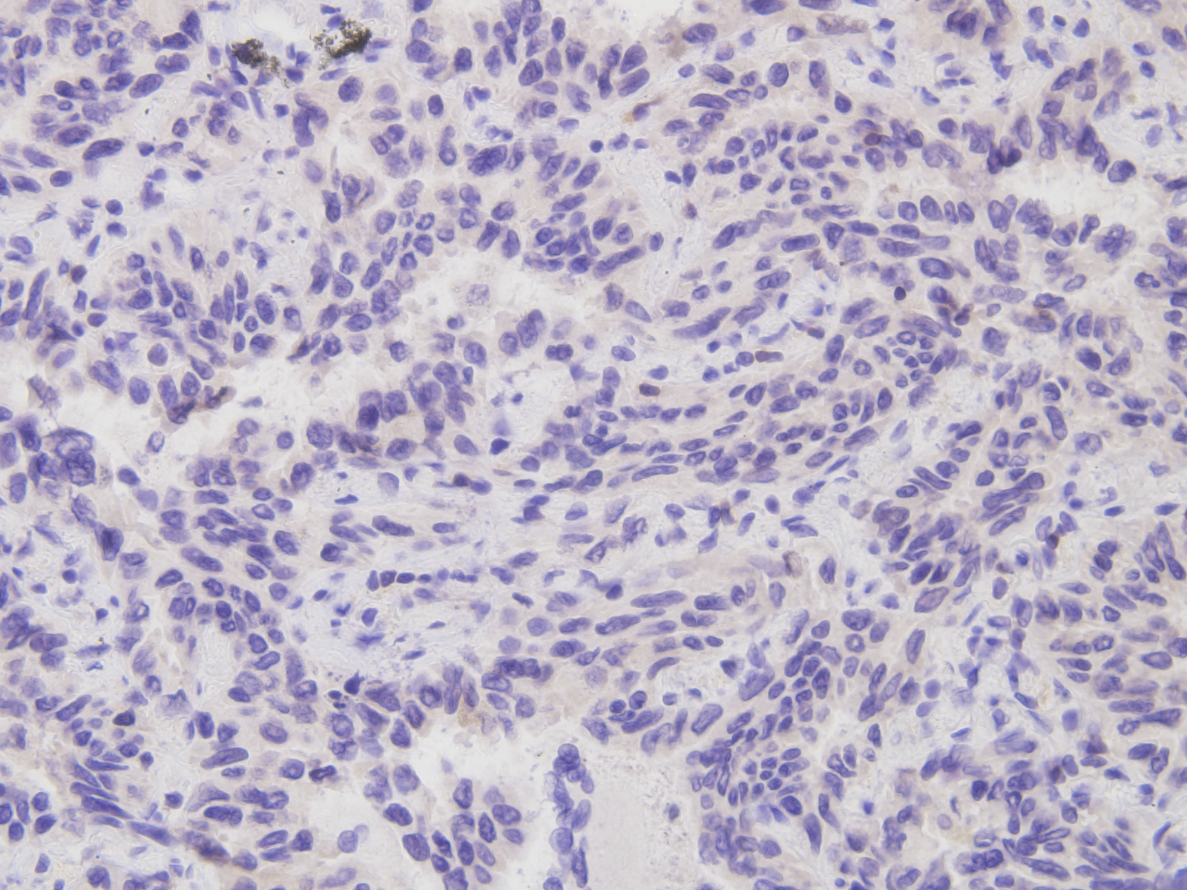

Supplement: S44 File — (ZIP) [file pone.0337223.s045.zip › 500857-400X-CA-N/500857-400X-CA (1).tif]

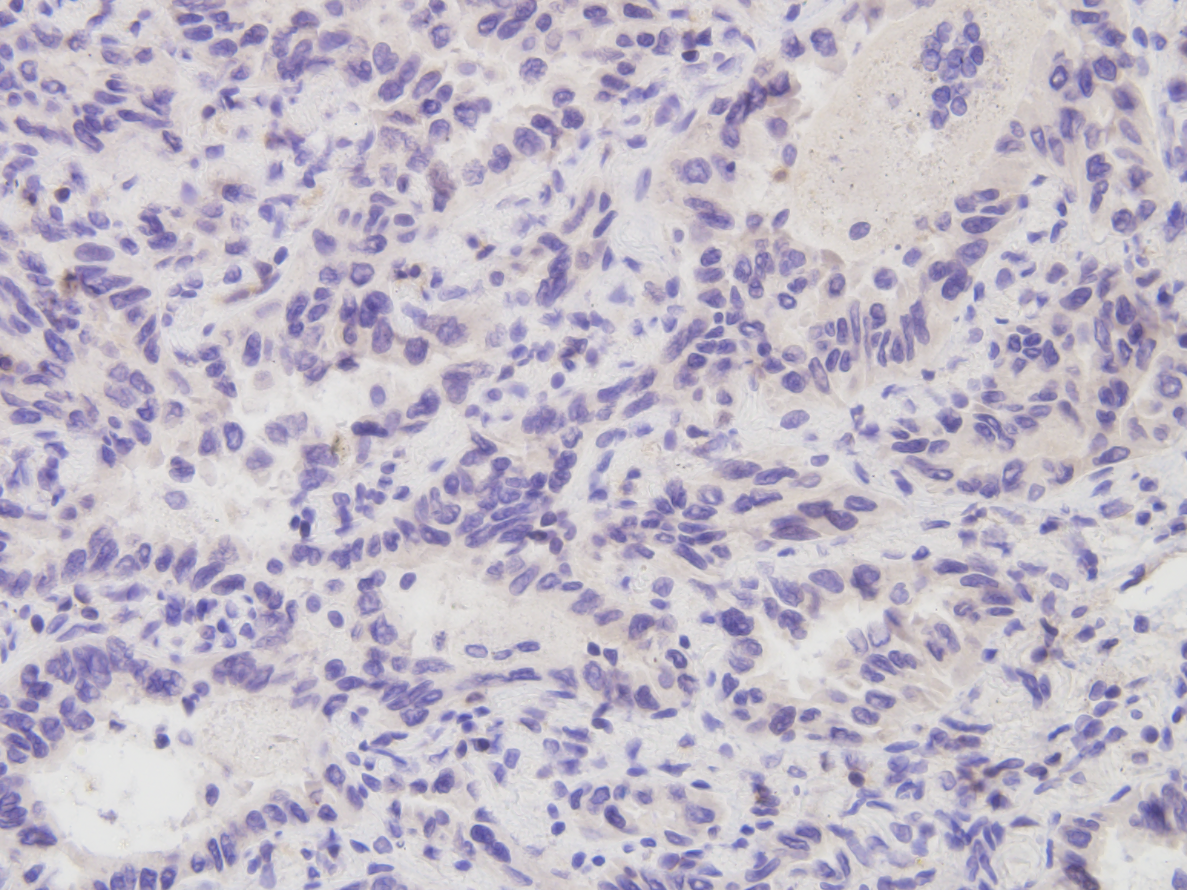

Supplement: S44 File — (ZIP) [file pone.0337223.s045.zip › 500857-400X-CA-N/500857-400X-CA (2).tif]

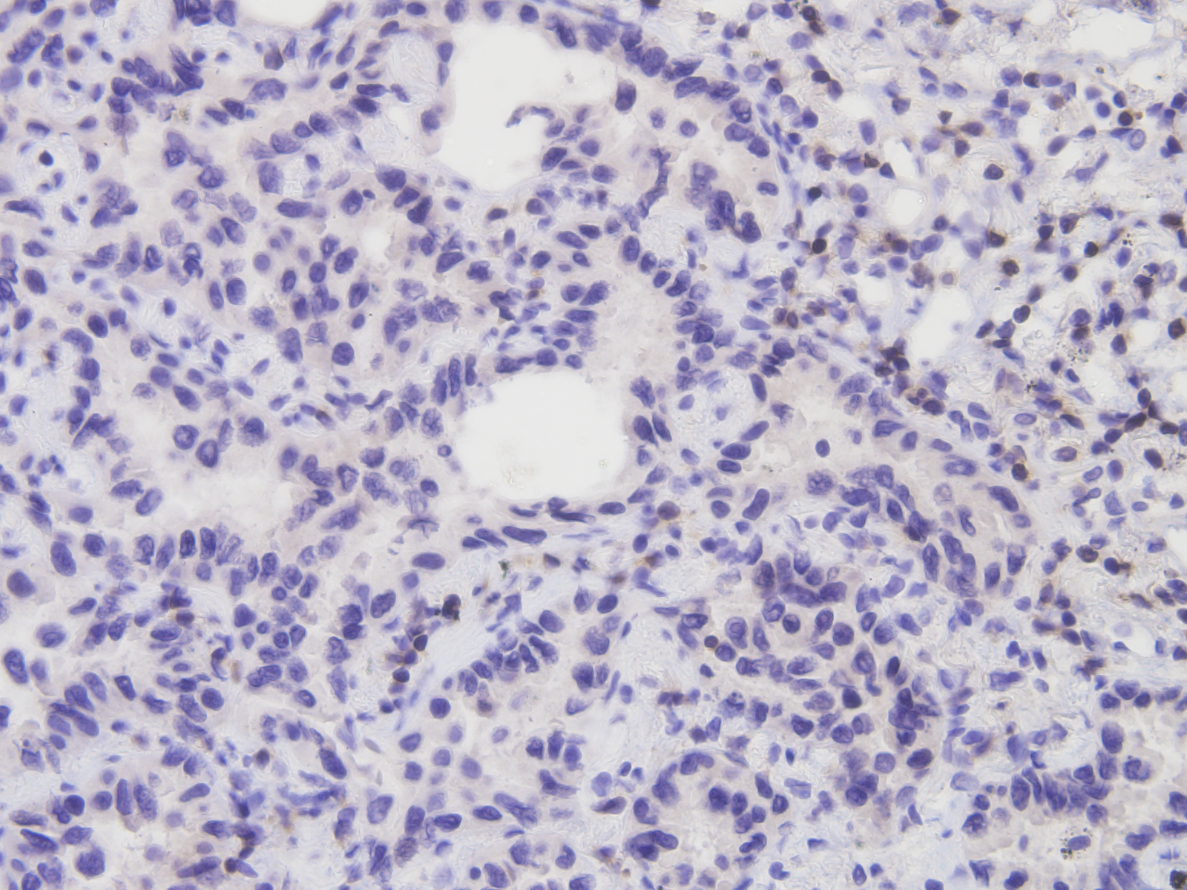

Supplement: S44 File — (ZIP) [file pone.0337223.s045.zip › 500857-400X-CA-N/500857-400X-CA (3).tif]

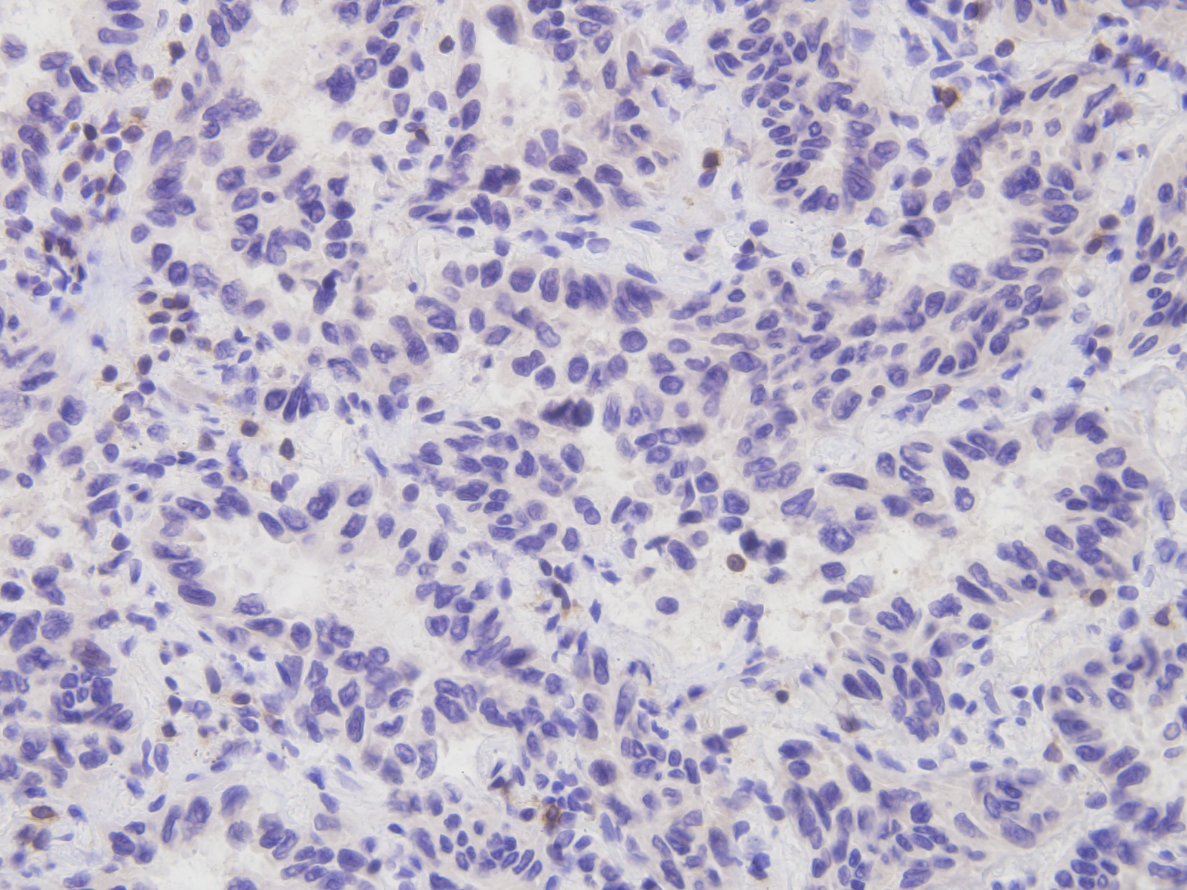

Supplement: S44 File — (ZIP) [file pone.0337223.s045.zip › 500857-400X-CA-N/500857-400X-CA (4).tif]

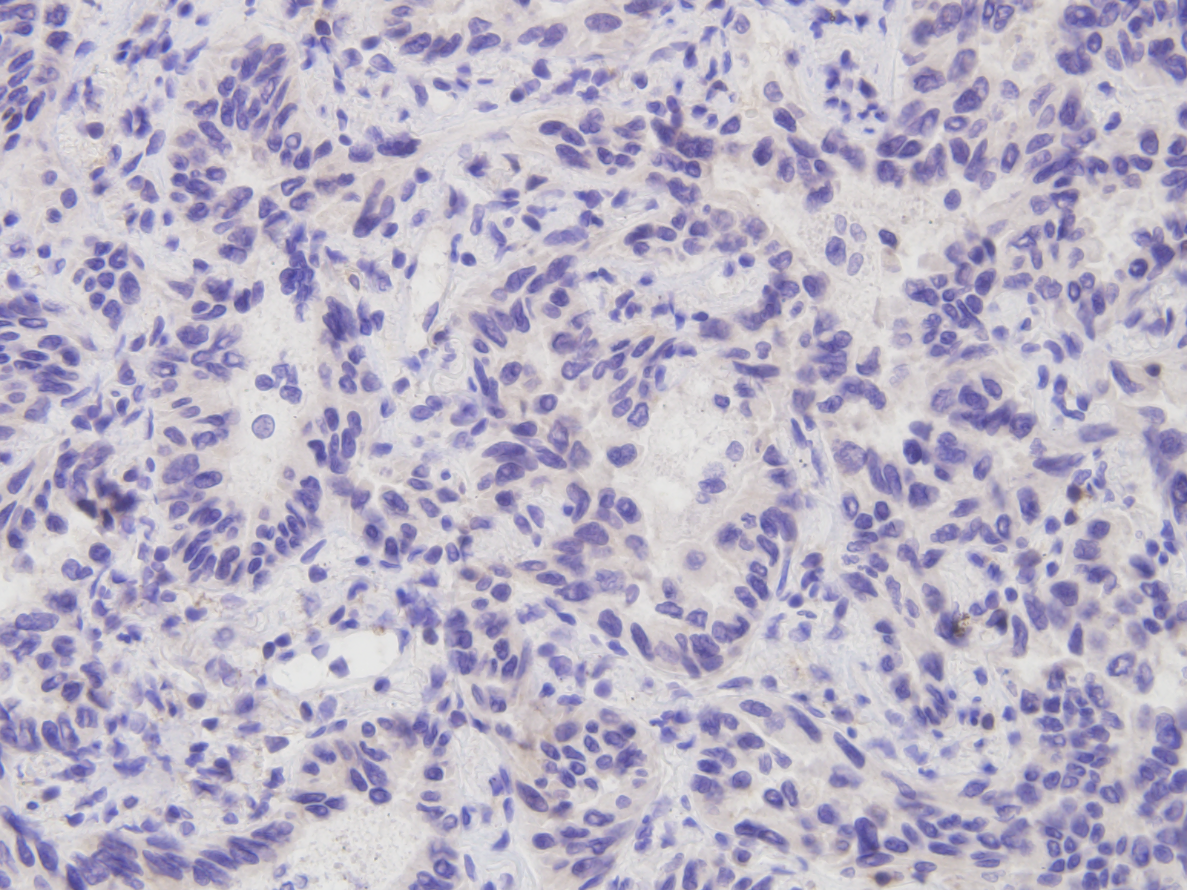

Supplement: S44 File — (ZIP) [file pone.0337223.s045.zip › 500857-400X-CA-N/500857-400X-CA (5).tif]

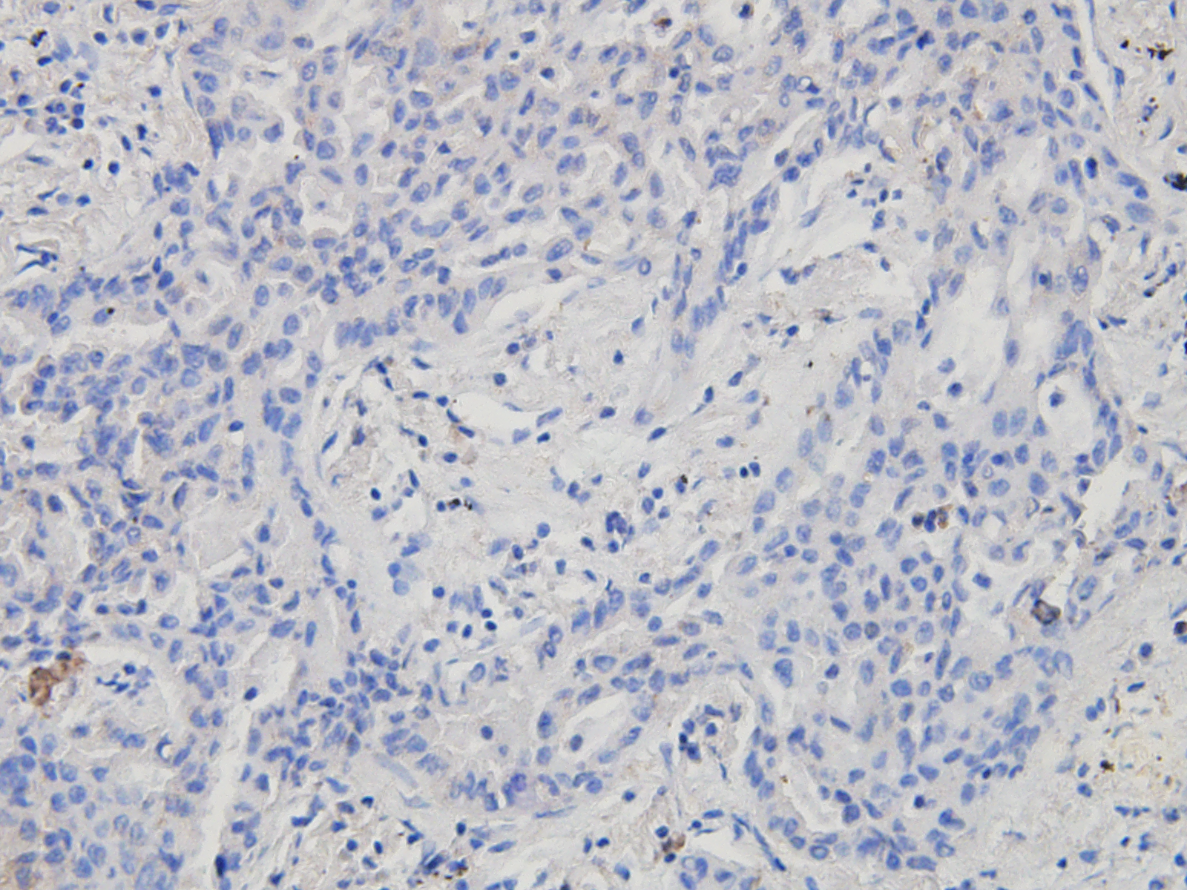

Supplement: S45 File — (ZIP) [file pone.0337223.s046.zip › 502487-400X-CA-N/502487-400X--CA (1).tif]

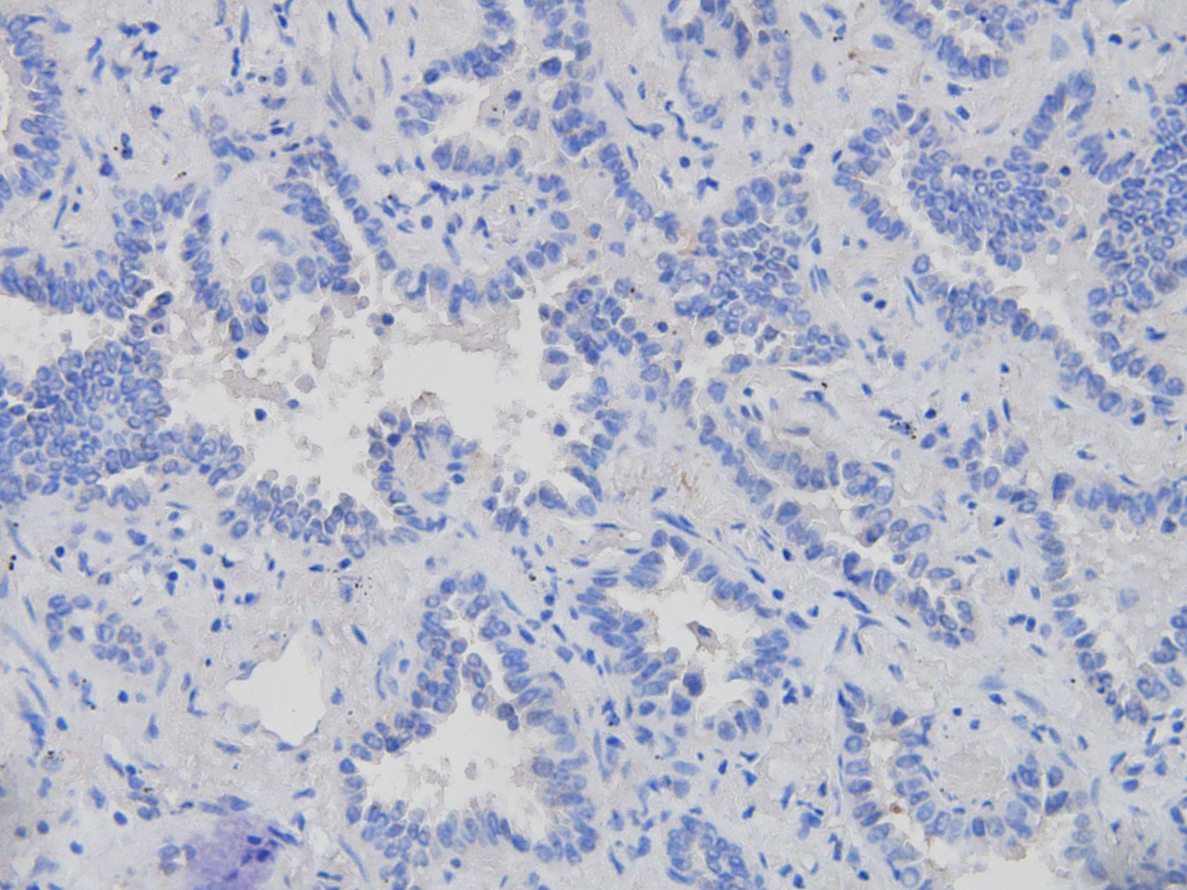

Supplement: S45 File — (ZIP) [file pone.0337223.s046.zip › 502487-400X-CA-N/502487-400X--CA (2).tif]

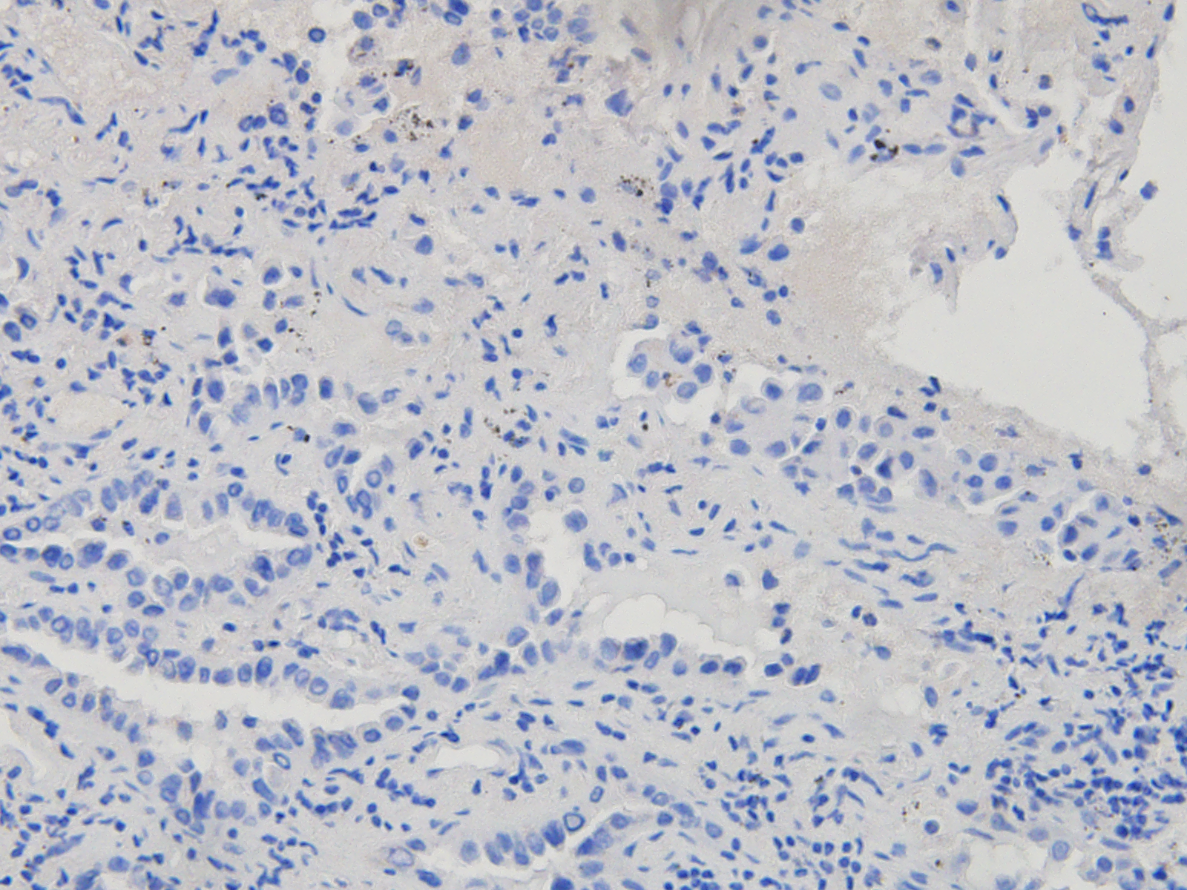

Supplement: S45 File — (ZIP) [file pone.0337223.s046.zip › 502487-400X-CA-N/502487-400X--CA (3).tif]

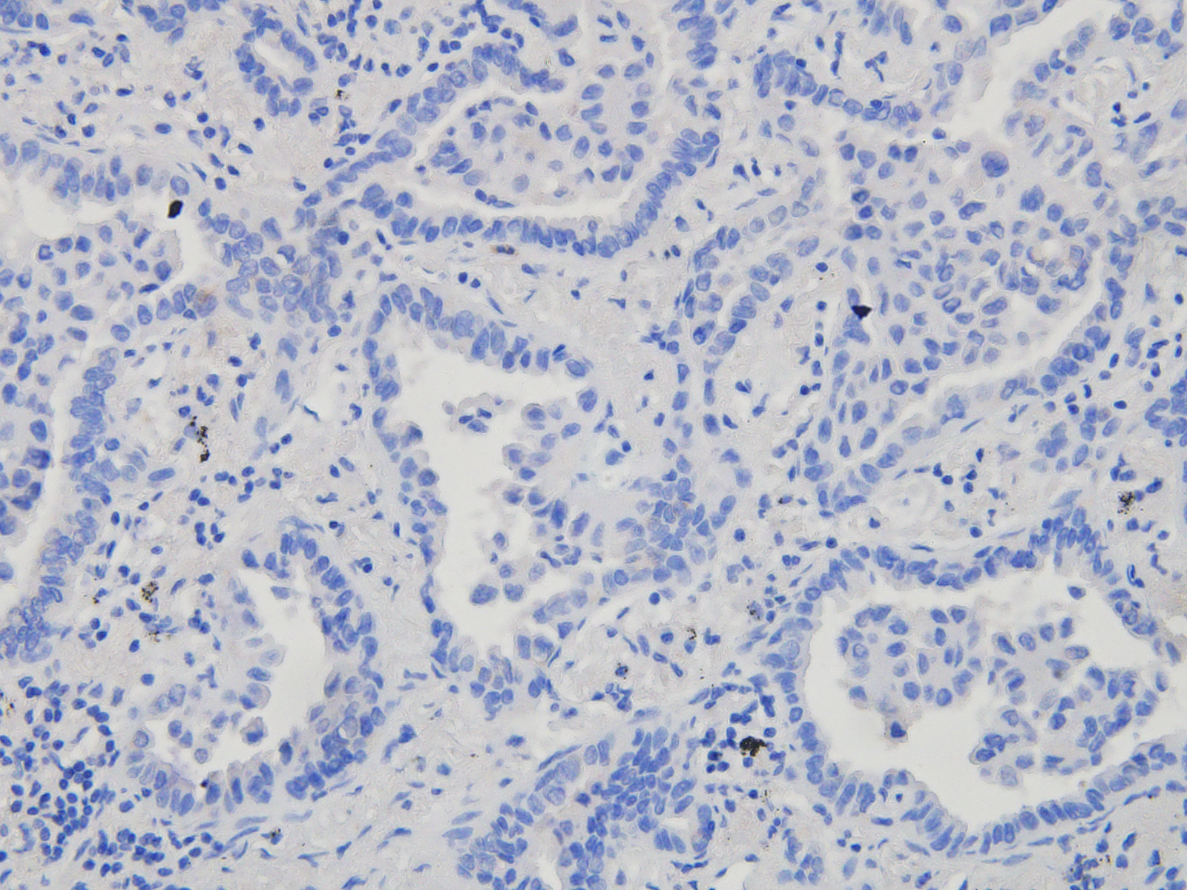

Supplement: S45 File — (ZIP) [file pone.0337223.s046.zip › 502487-400X-CA-N/502487-400X--CA (4).tif]

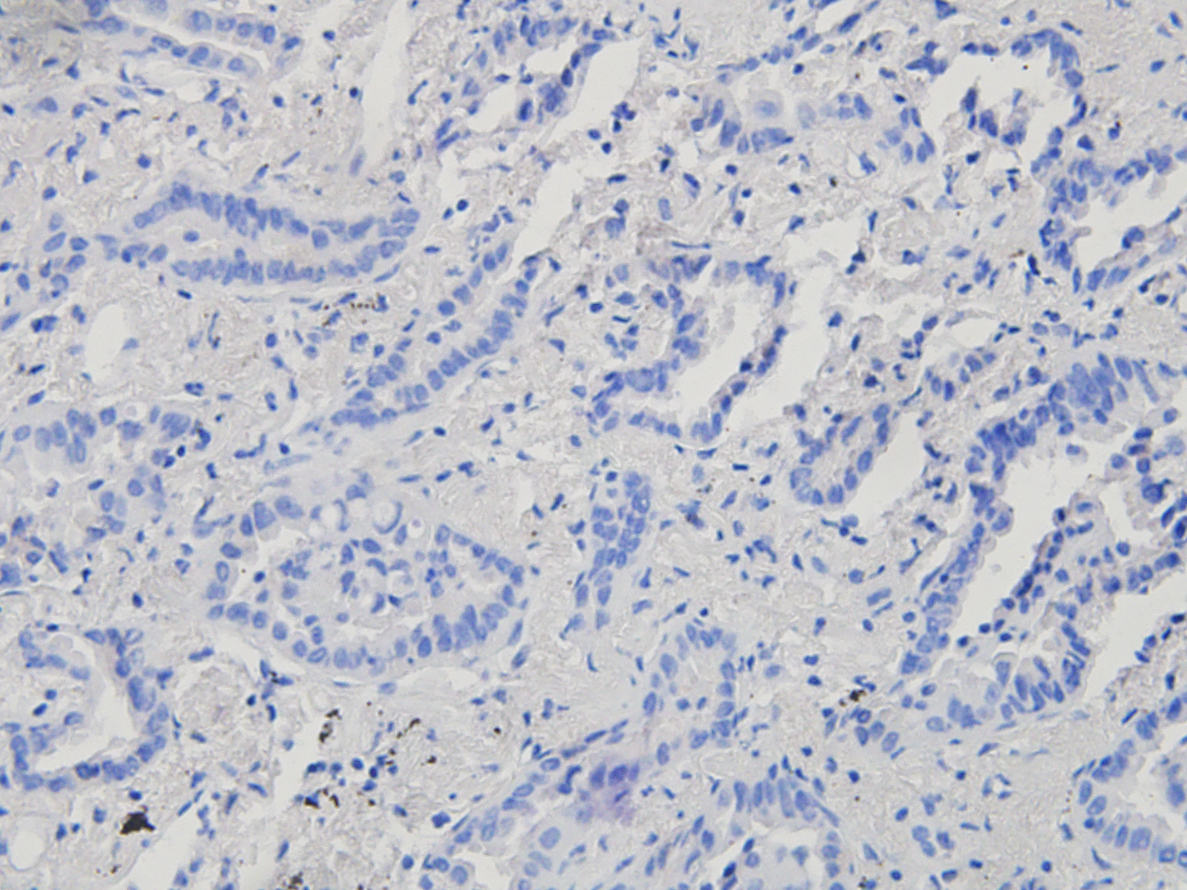

Supplement: S45 File — (ZIP) [file pone.0337223.s046.zip › 502487-400X-CA-N/502487-400X--CA (5).tif]

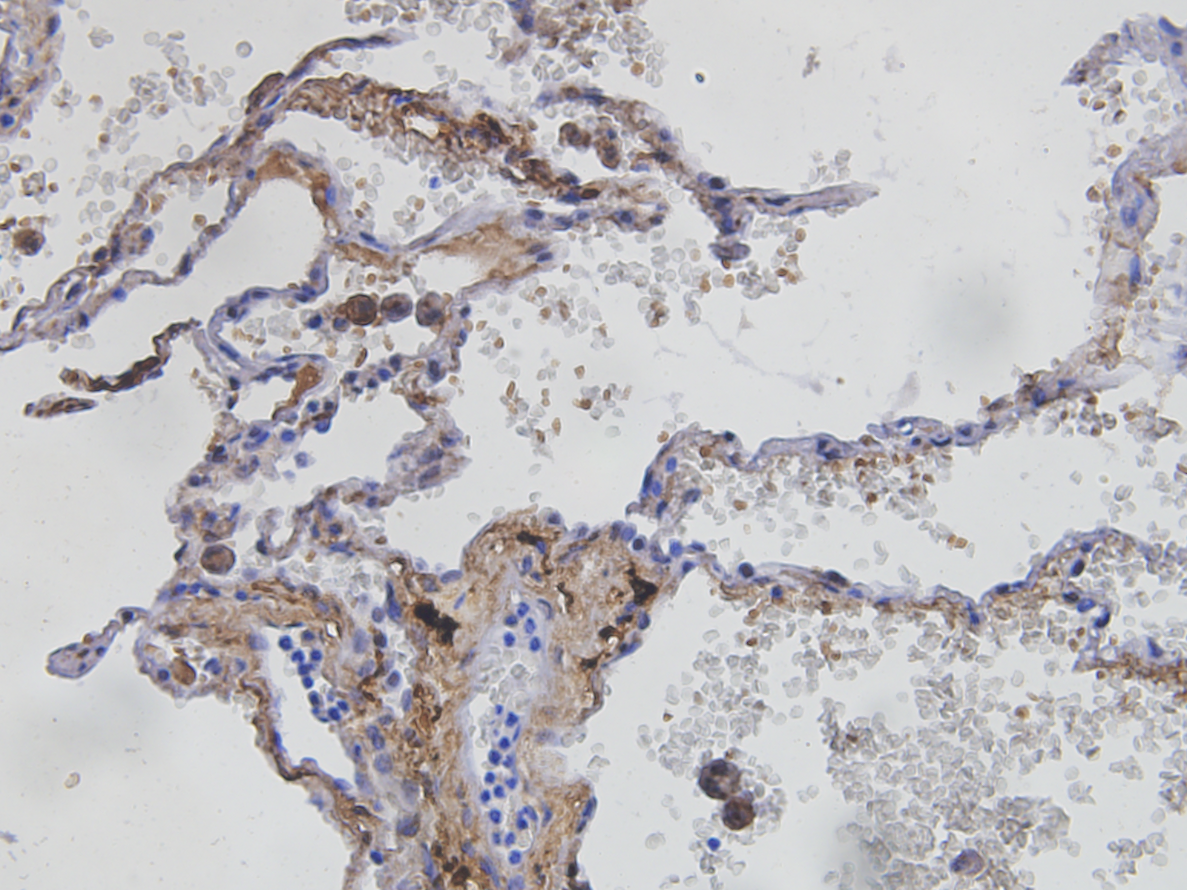

Supplement: S45 File — (ZIP) [file pone.0337223.s046.zip › 502487-400X-CA-N/502487-400X--N (1).tif]

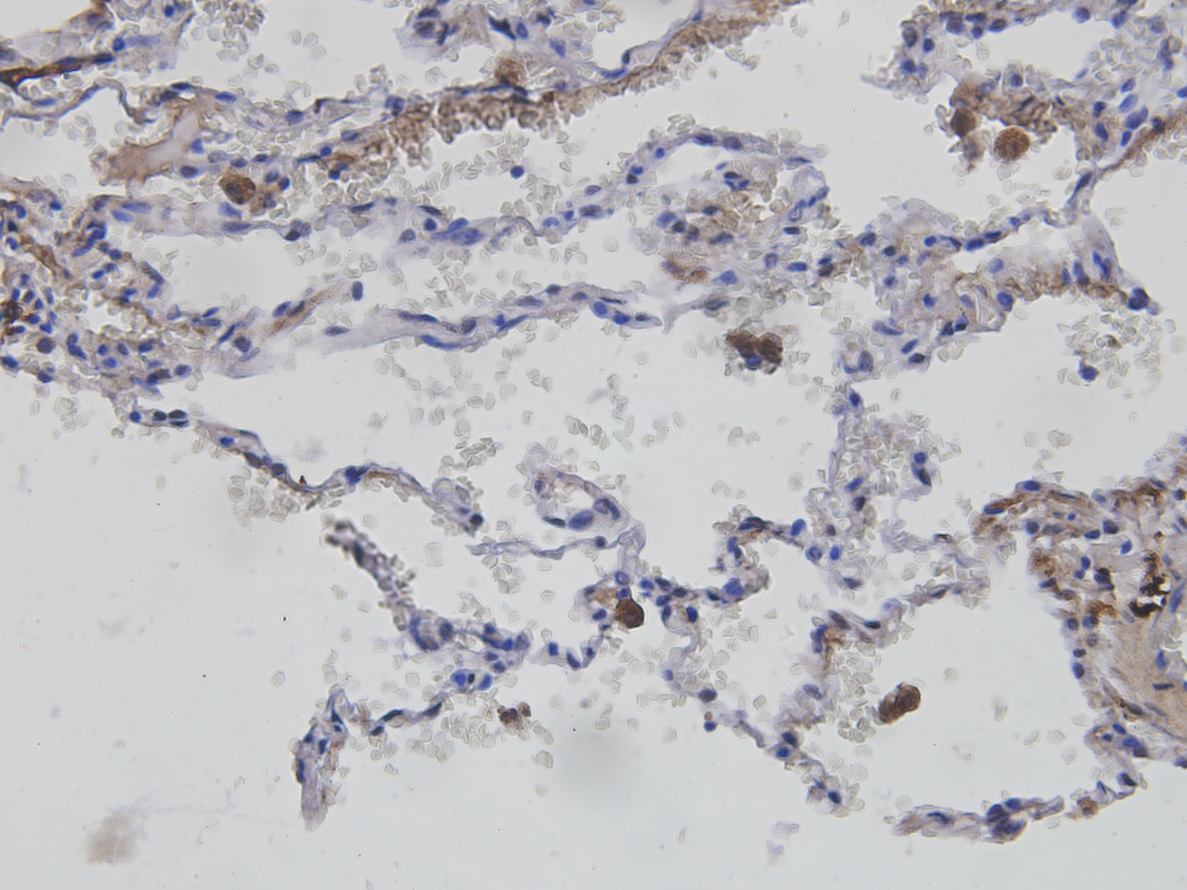

Supplement: S45 File — (ZIP) [file pone.0337223.s046.zip › 502487-400X-CA-N/502487-400X--N (2).tif]

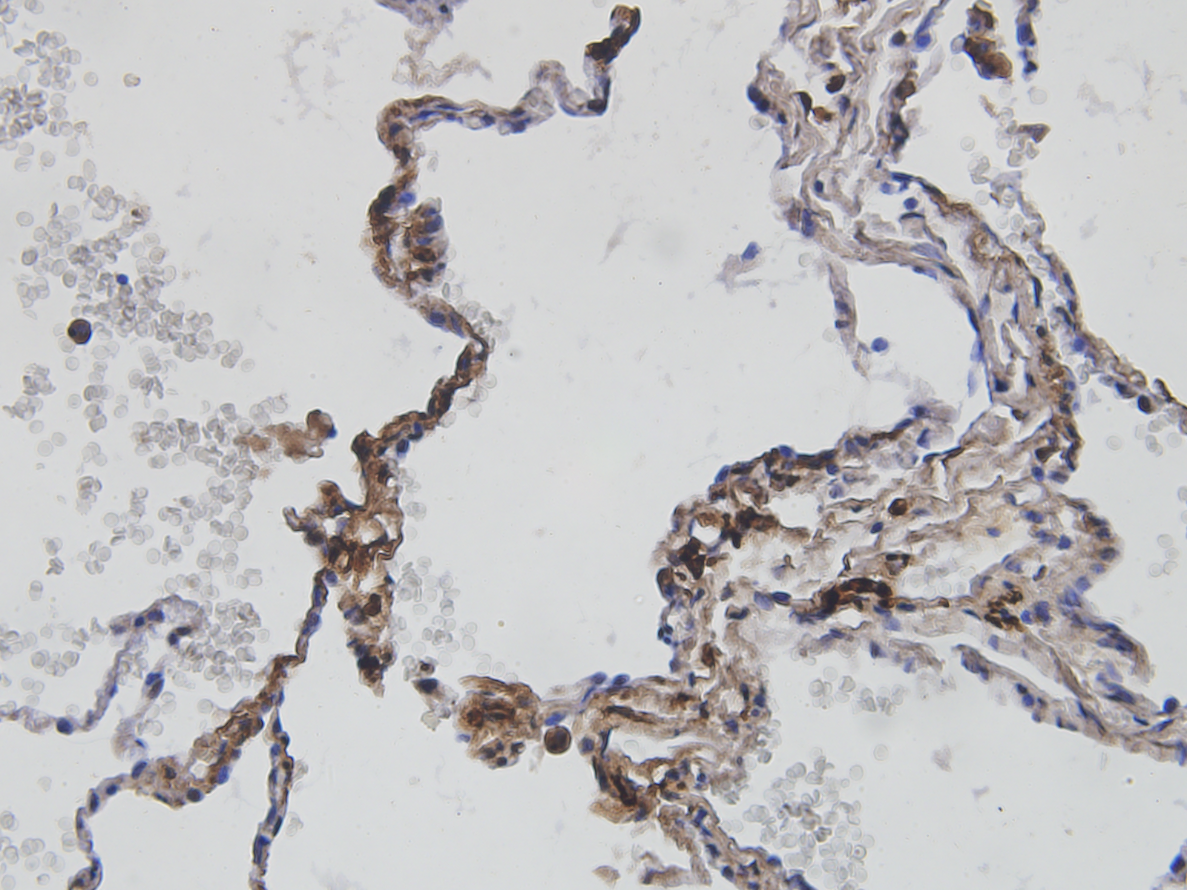

Supplement: S45 File — (ZIP) [file pone.0337223.s046.zip › 502487-400X-CA-N/502487-400X--N (3).tif]

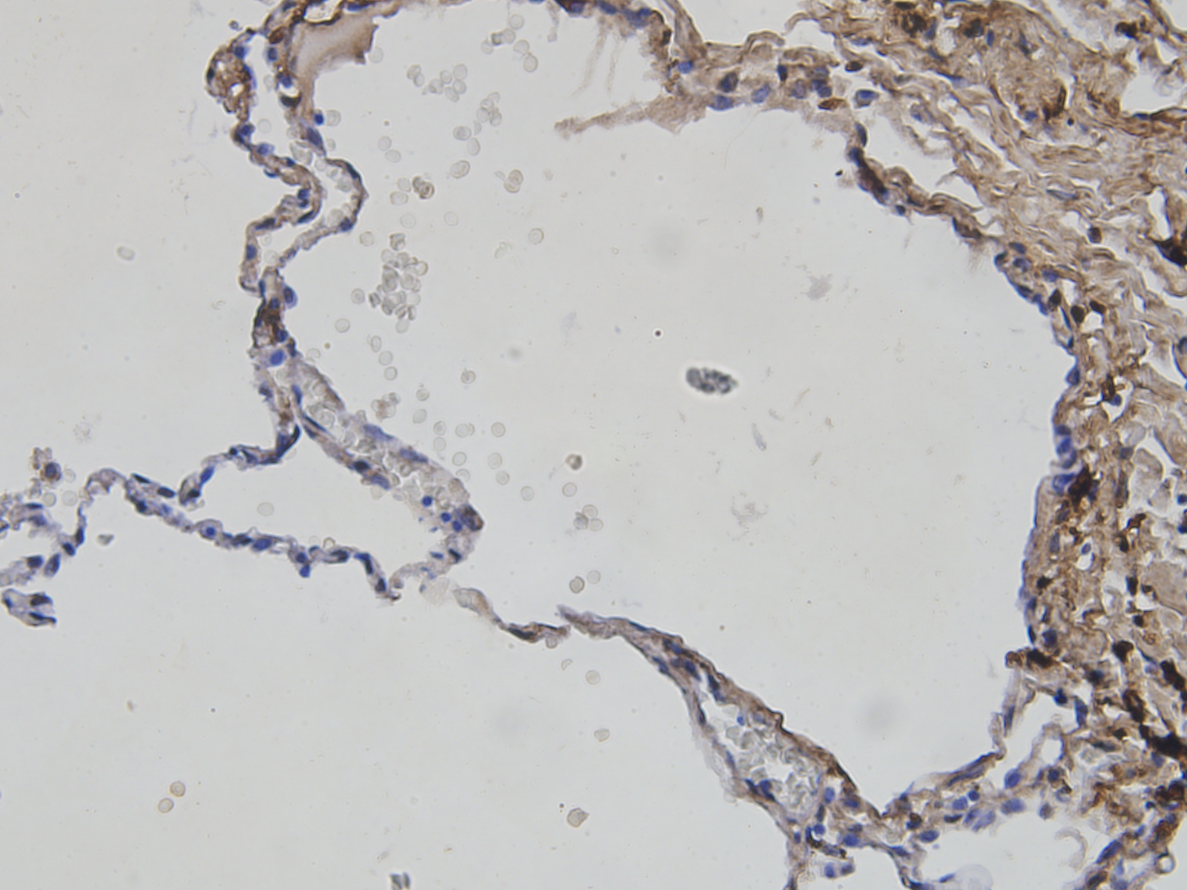

Supplement: S45 File — (ZIP) [file pone.0337223.s046.zip › 502487-400X-CA-N/502487-400X--N (4).tif]

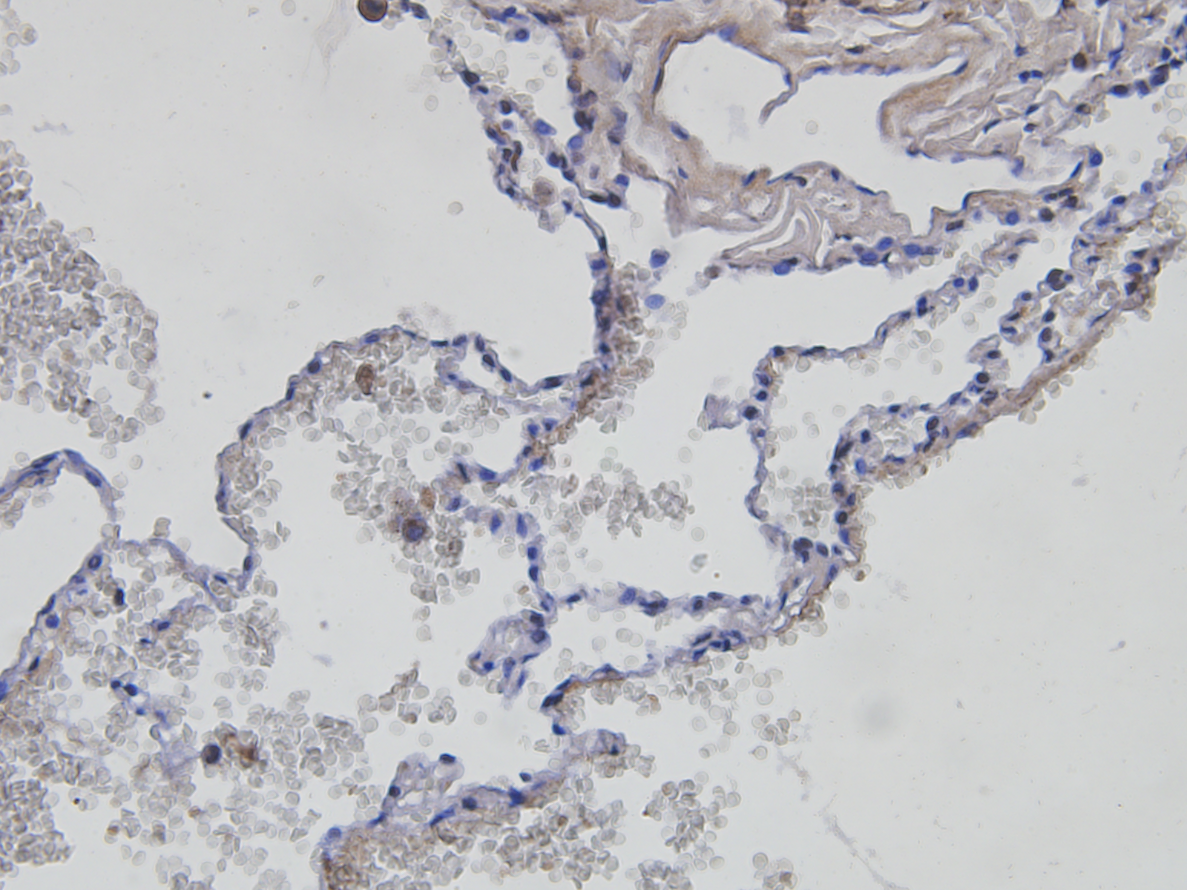

Supplement: S45 File — (ZIP) [file pone.0337223.s046.zip › 502487-400X-CA-N/502487-400X--N (5).tif]

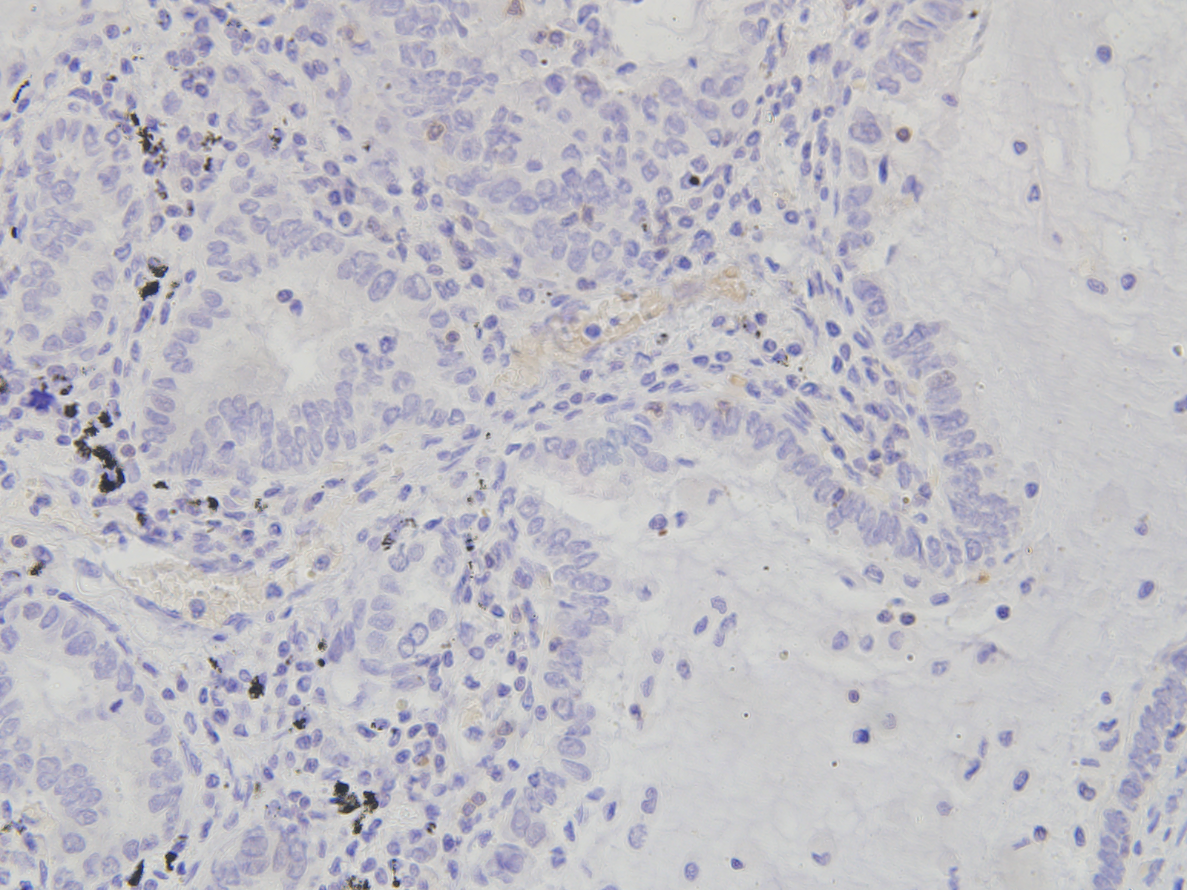

Supplement: S46 File — (ZIP) [file pone.0337223.s047.zip › 505252-400X-N-CA/505252-400X--CA (5).tif]

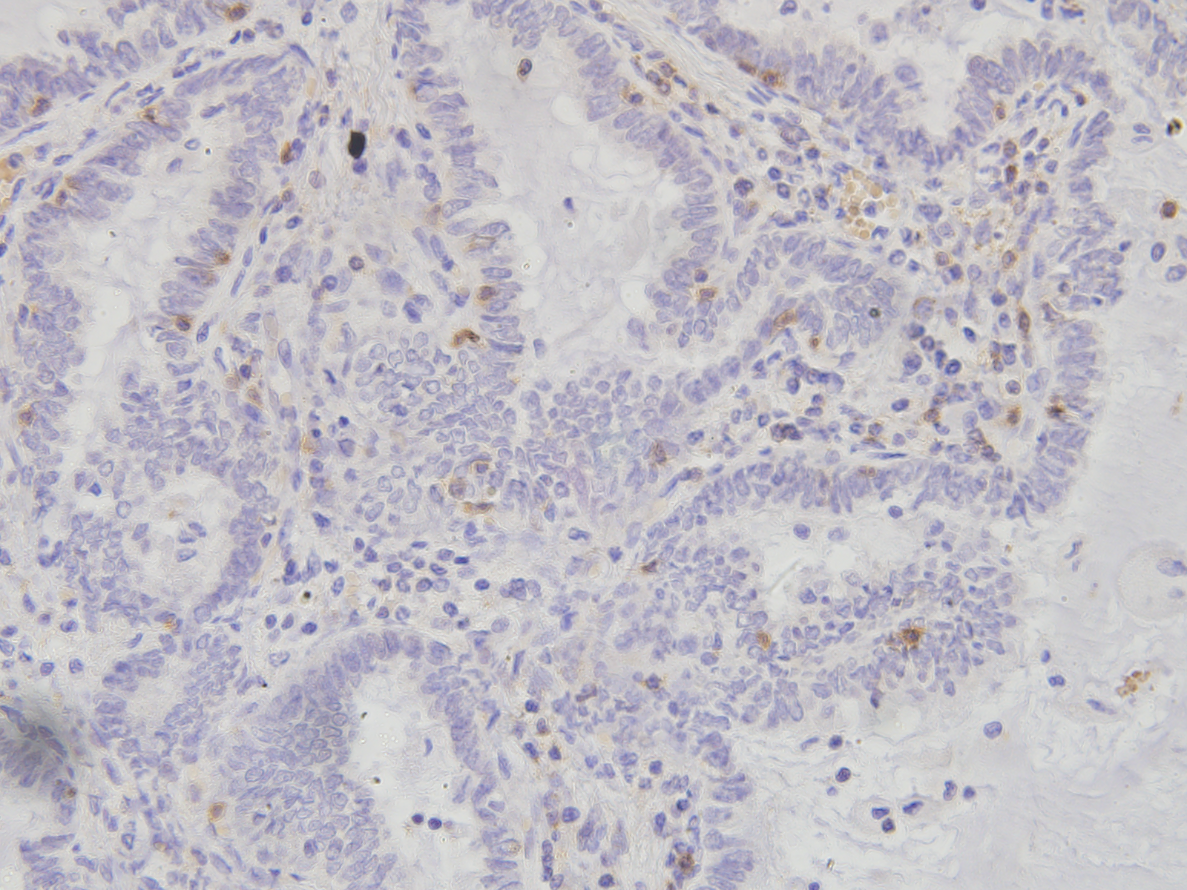

Supplement: S46 File — (ZIP) [file pone.0337223.s047.zip › 505252-400X-N-CA/505252-400X--CA (6).tif]

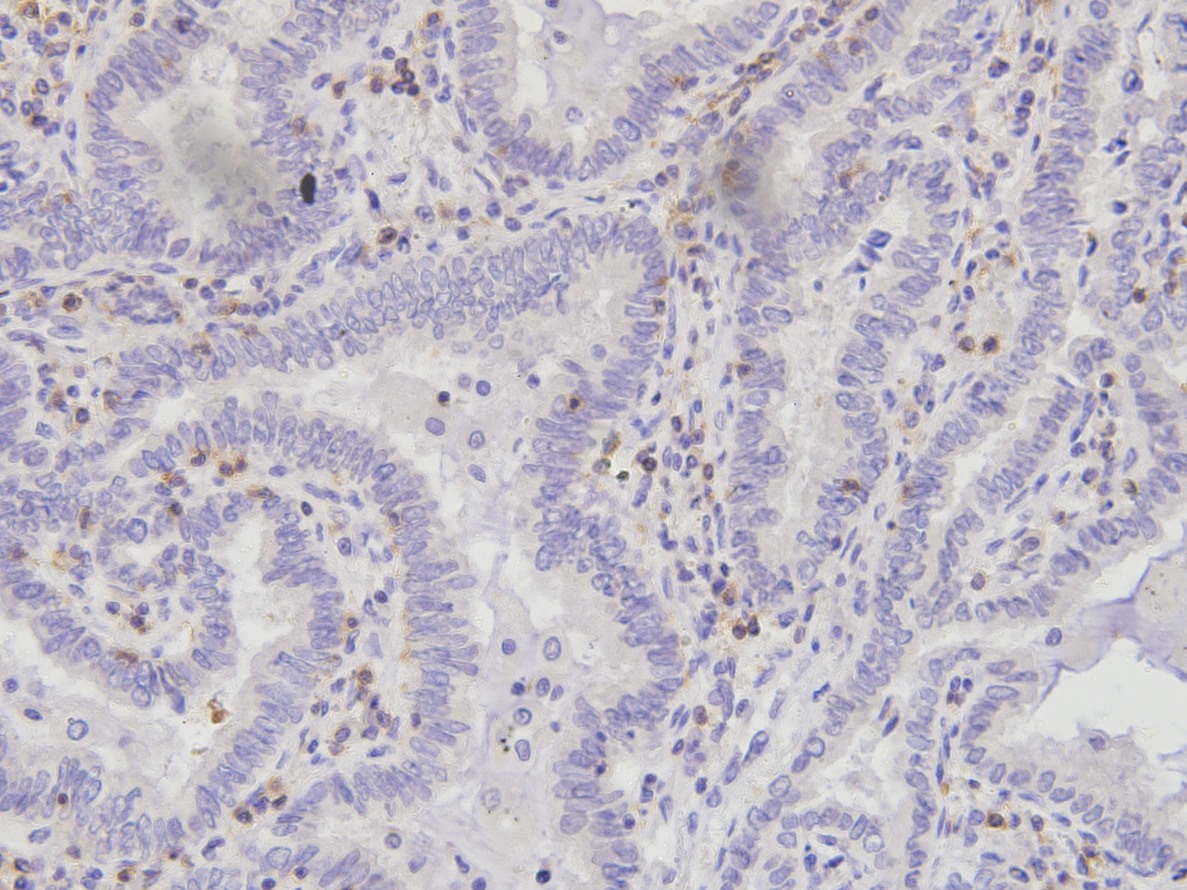

Supplement: S46 File — (ZIP) [file pone.0337223.s047.zip › 505252-400X-N-CA/505252-400X--CA (7).tif]

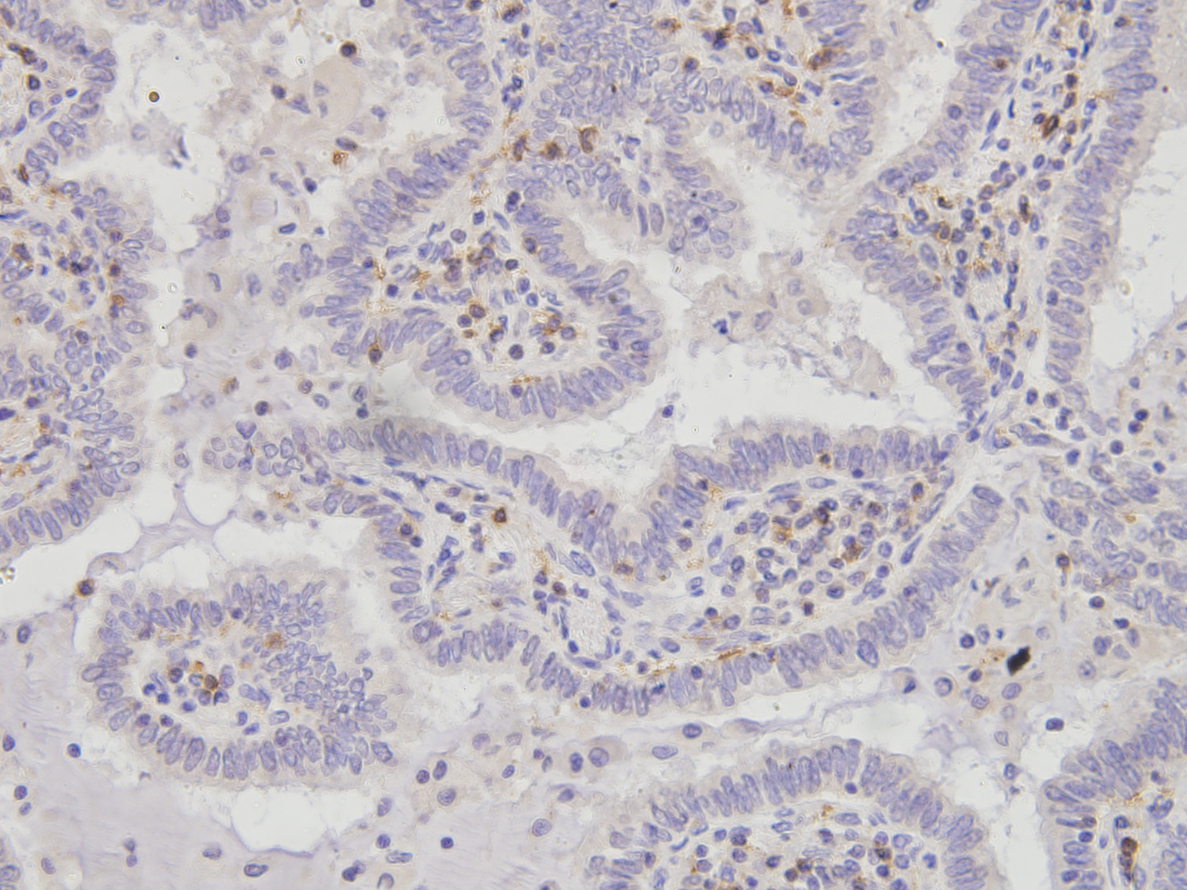

Supplement: S46 File — (ZIP) [file pone.0337223.s047.zip › 505252-400X-N-CA/505252-400X--CA (8).tif]

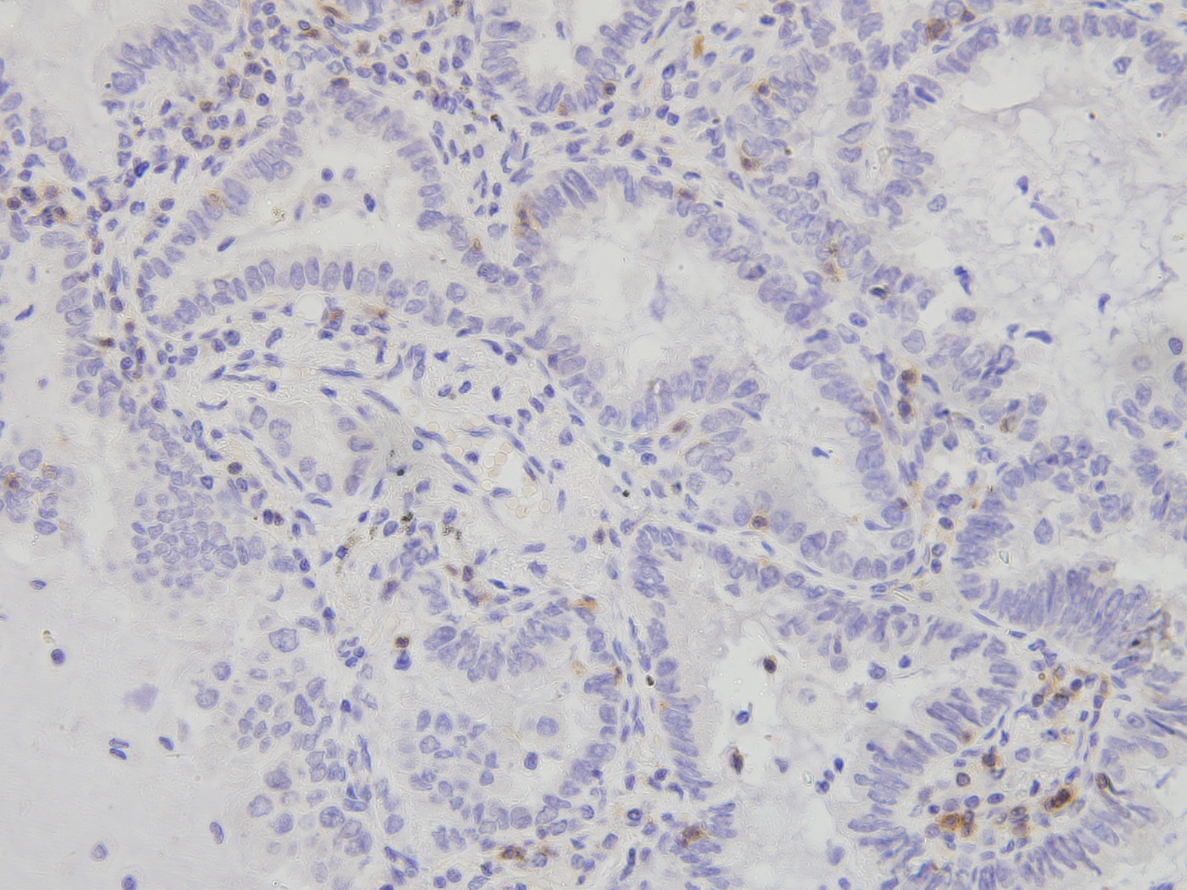

Supplement: S46 File — (ZIP) [file pone.0337223.s047.zip › 505252-400X-N-CA/505252-400X--CA (9).tif]

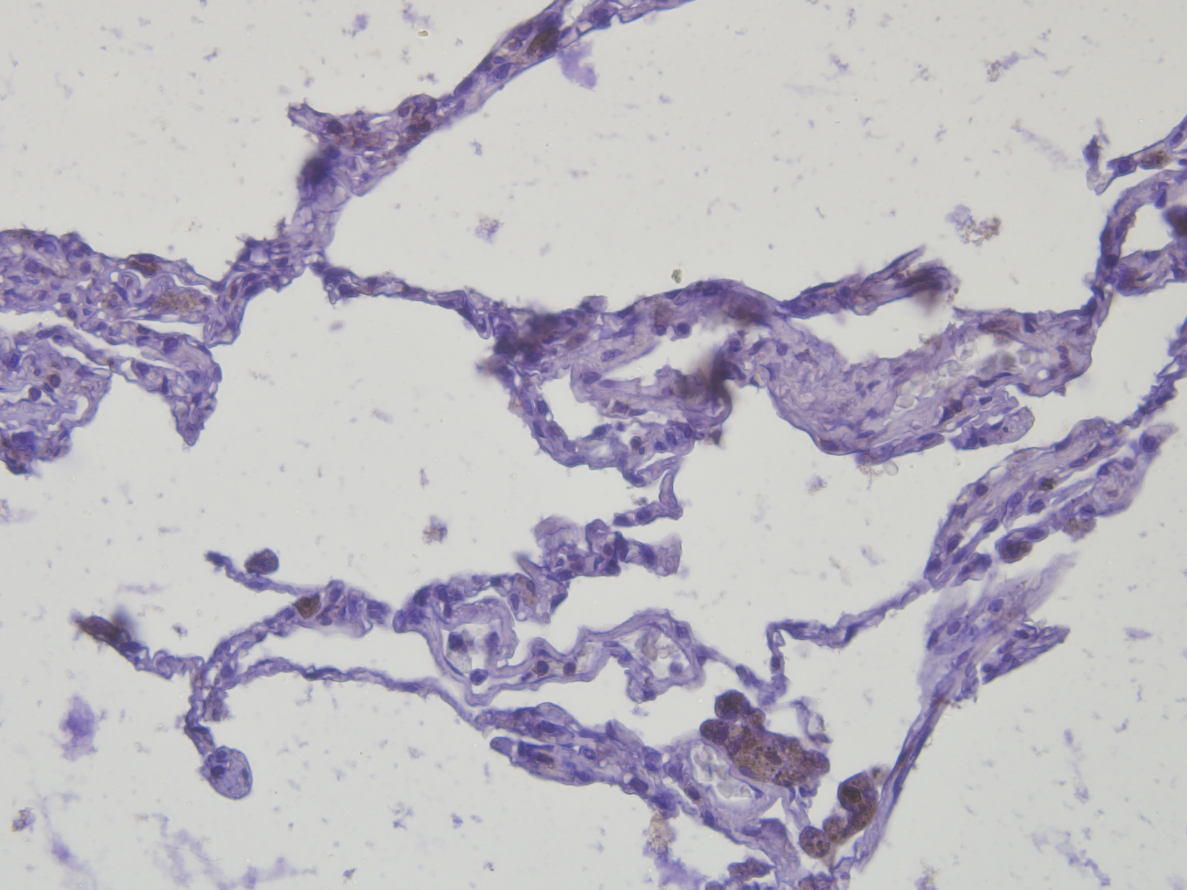

Supplement: S46 File — (ZIP) [file pone.0337223.s047.zip › 505252-400X-N-CA/505252-400X-N (5).tif]

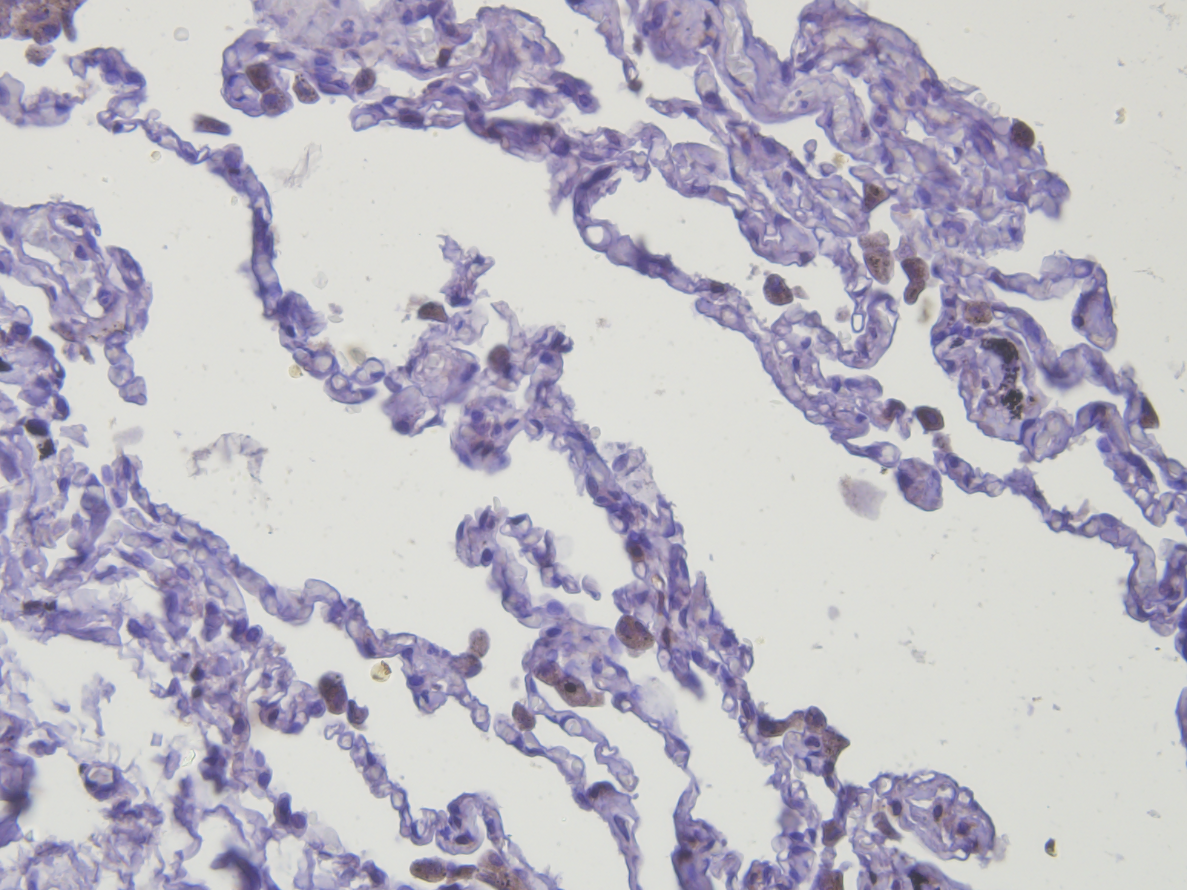

Supplement: S46 File — (ZIP) [file pone.0337223.s047.zip › 505252-400X-N-CA/505252-400X-N (6).tif]

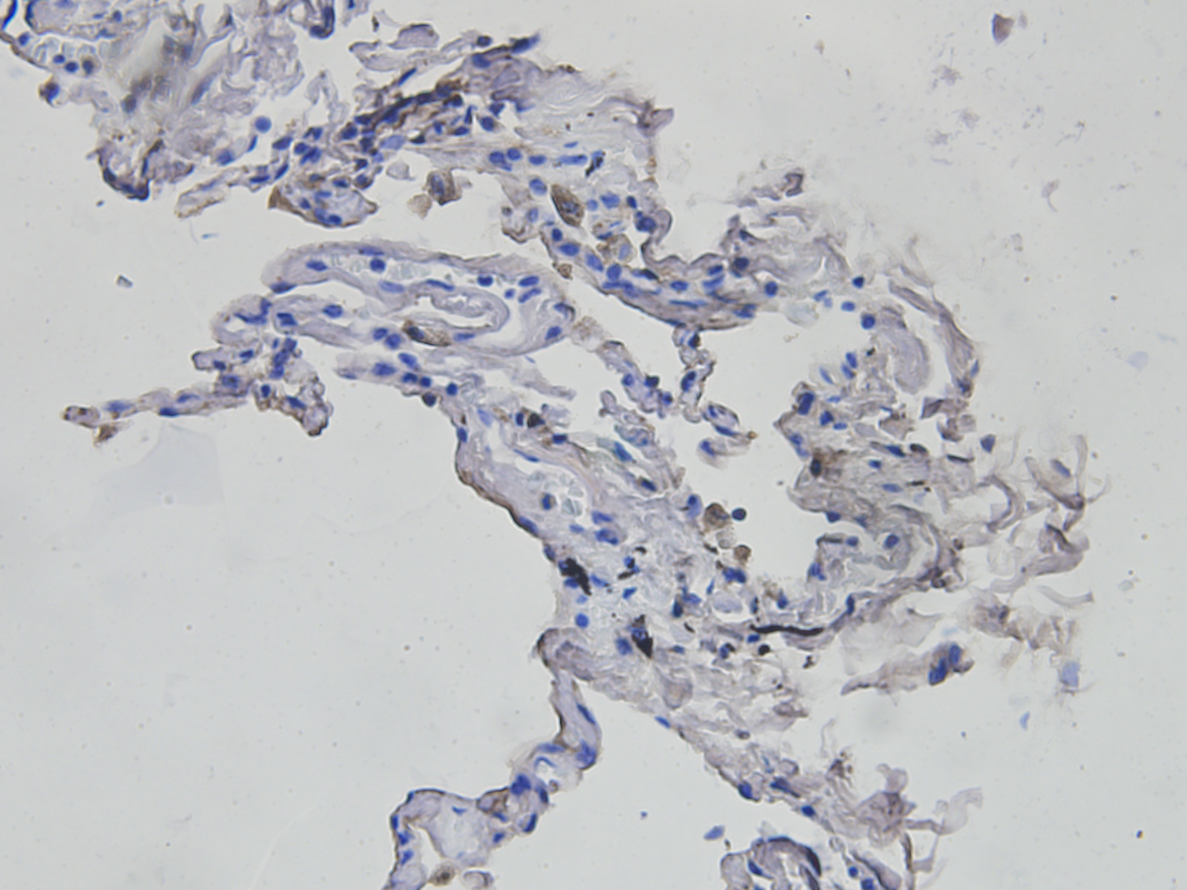

Supplement: S46 File — (ZIP) [file pone.0337223.s047.zip › 505252-400X-N-CA/505252-400X-N (7).tif]

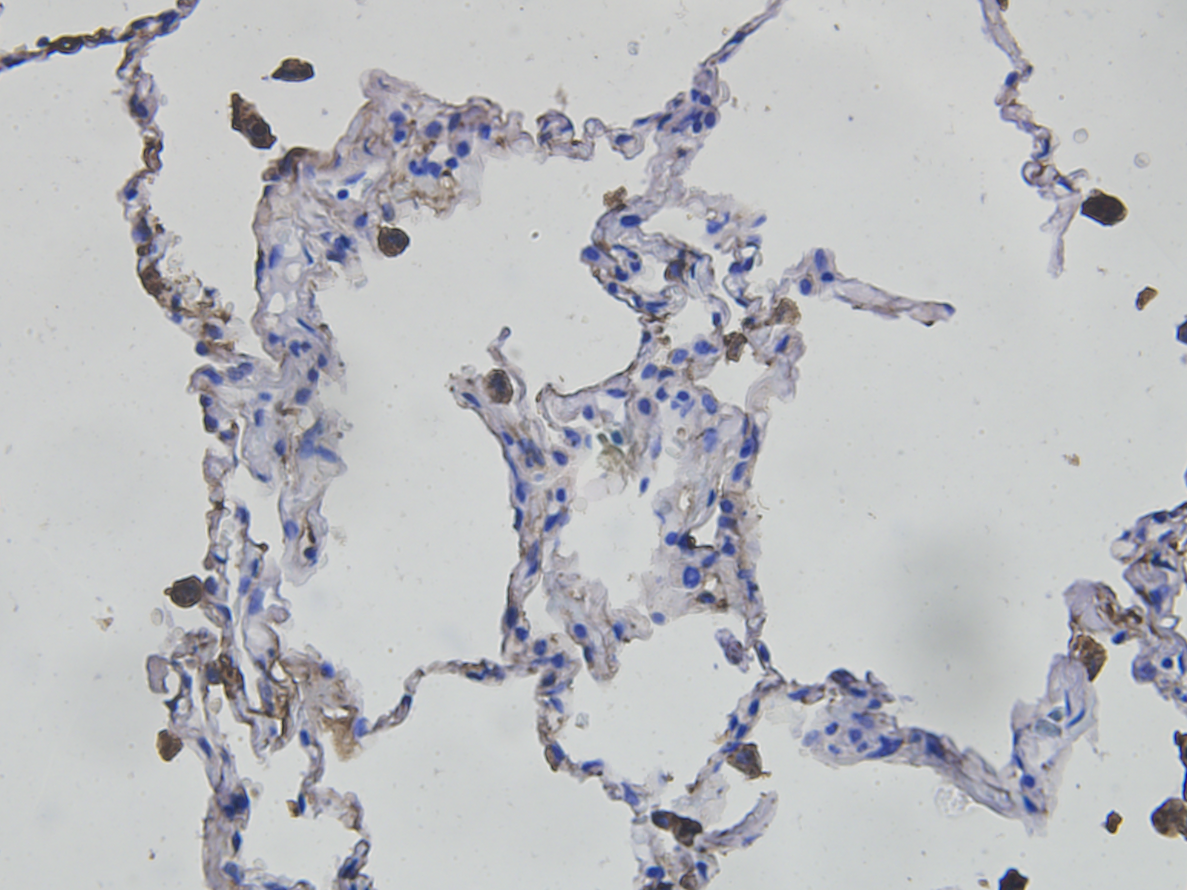

Supplement: S46 File — (ZIP) [file pone.0337223.s047.zip › 505252-400X-N-CA/505252-400X-N (8).tif]

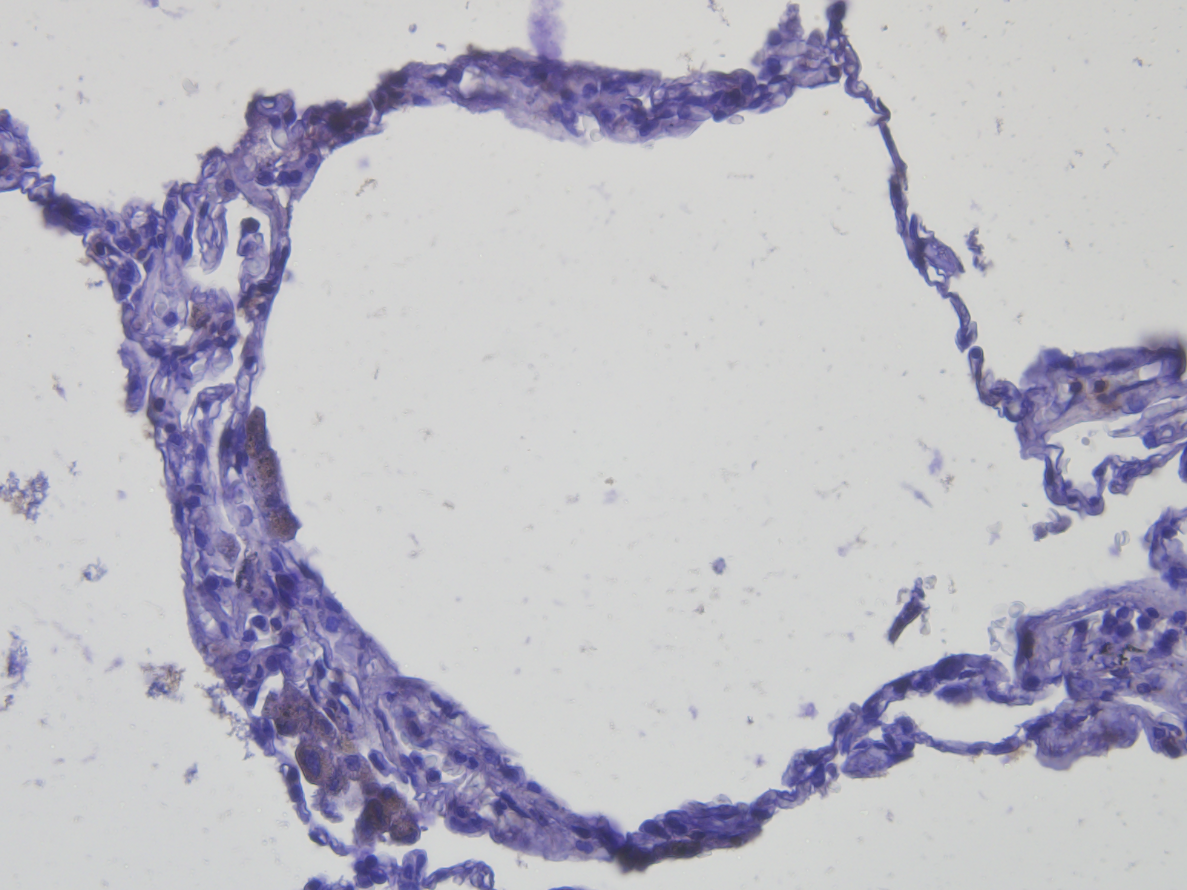

Supplement: S46 File — (ZIP) [file pone.0337223.s047.zip › 505252-400X-N-CA/505252-400X-N (9).tif]

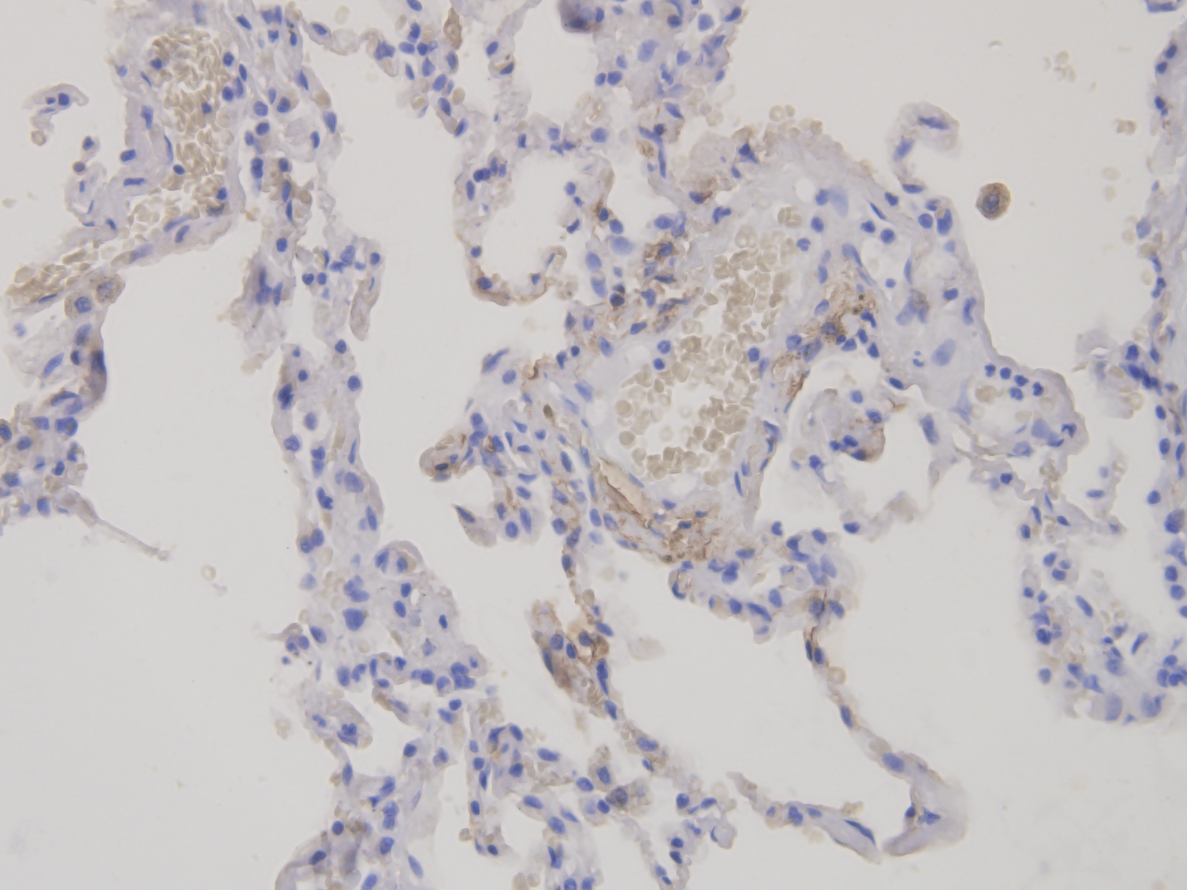

Supplement: S47 File — (ZIP) [file pone.0337223.s048.zip › 507095-400X-N-CA/507095-400X-- N (1).tif]

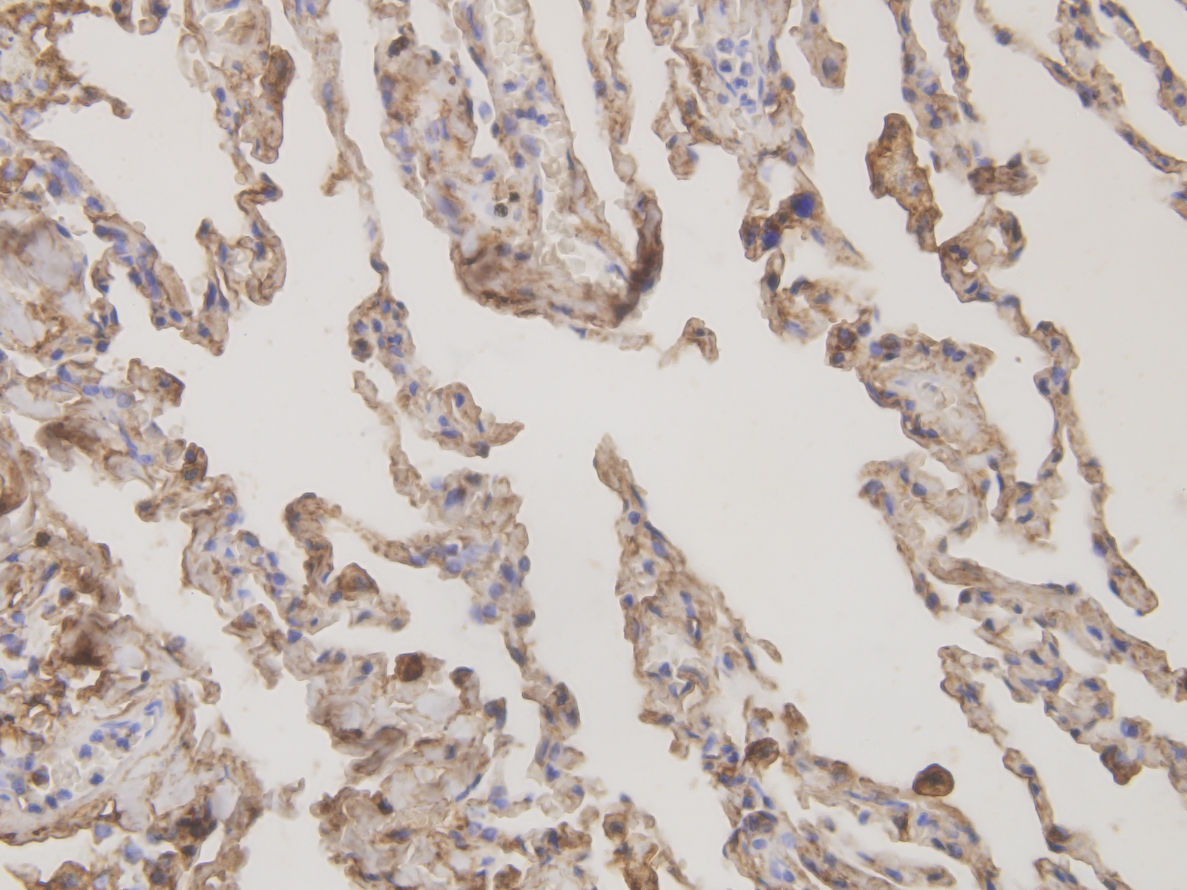

Supplement: S47 File — (ZIP) [file pone.0337223.s048.zip › 507095-400X-N-CA/507095-400X-- N (2).tif]

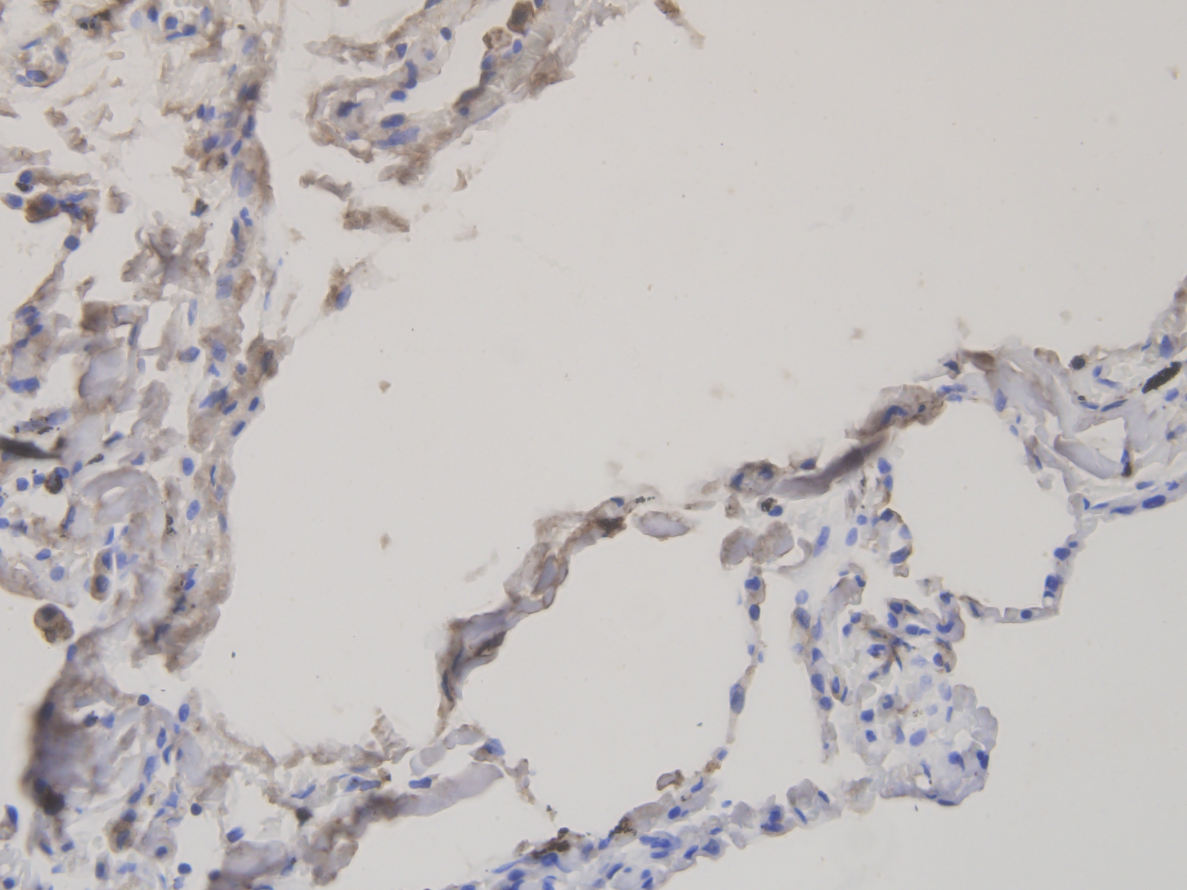

Supplement: S47 File — (ZIP) [file pone.0337223.s048.zip › 507095-400X-N-CA/507095-400X-- N (3).tif]

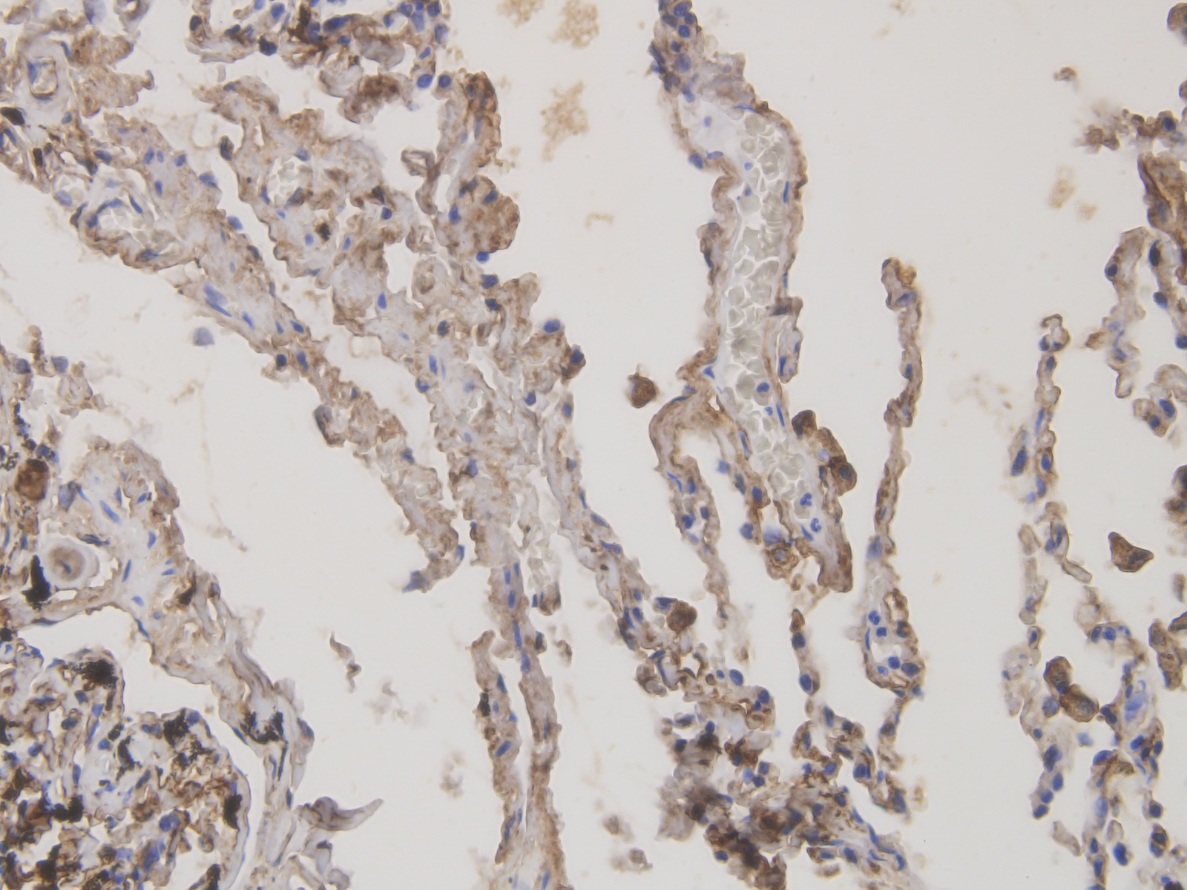

Supplement: S47 File — (ZIP) [file pone.0337223.s048.zip › 507095-400X-N-CA/507095-400X-- N (4).tif]

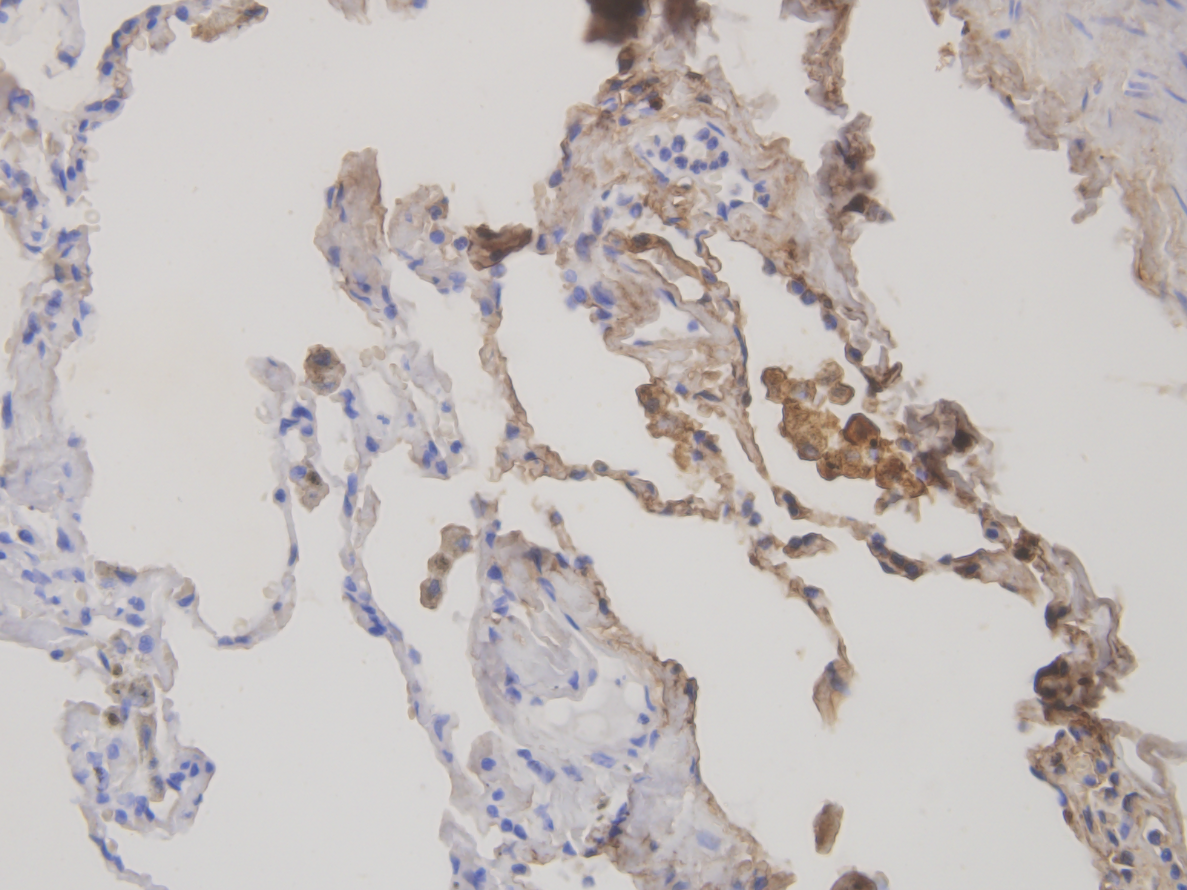

Supplement: S47 File — (ZIP) [file pone.0337223.s048.zip › 507095-400X-N-CA/507095-400X-- N (5).tif]

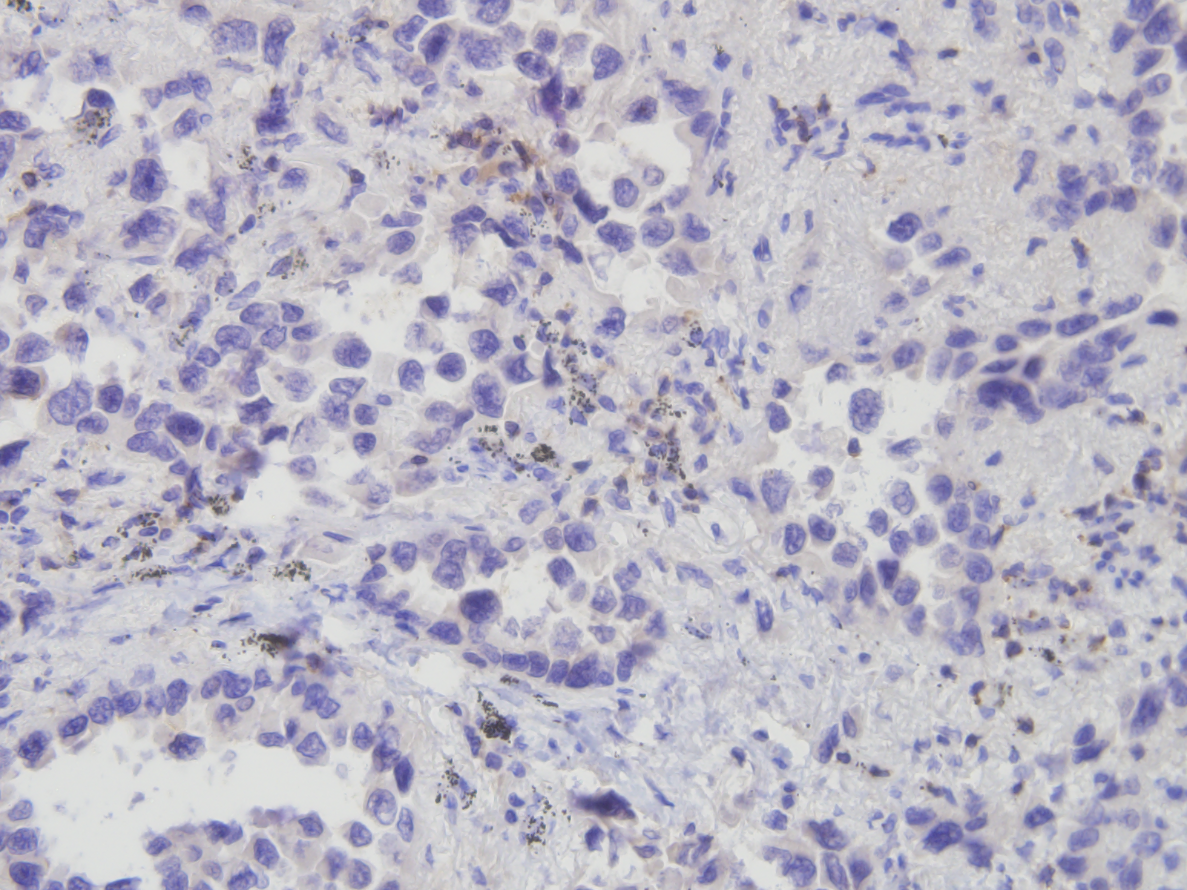

Supplement: S47 File — (ZIP) [file pone.0337223.s048.zip › 507095-400X-N-CA/507095-400X-CA (1).tif]

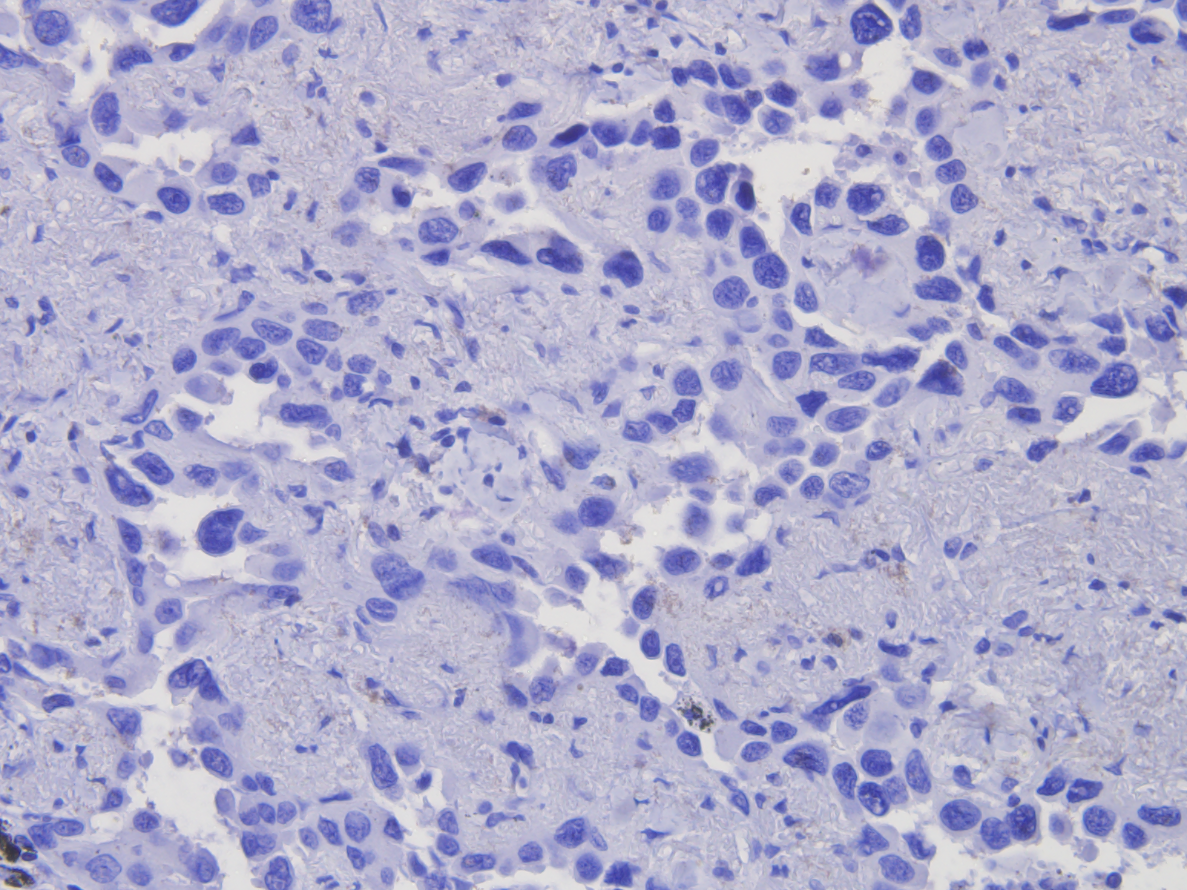

Supplement: S47 File — (ZIP) [file pone.0337223.s048.zip › 507095-400X-N-CA/507095-400X-CA (2).tif]

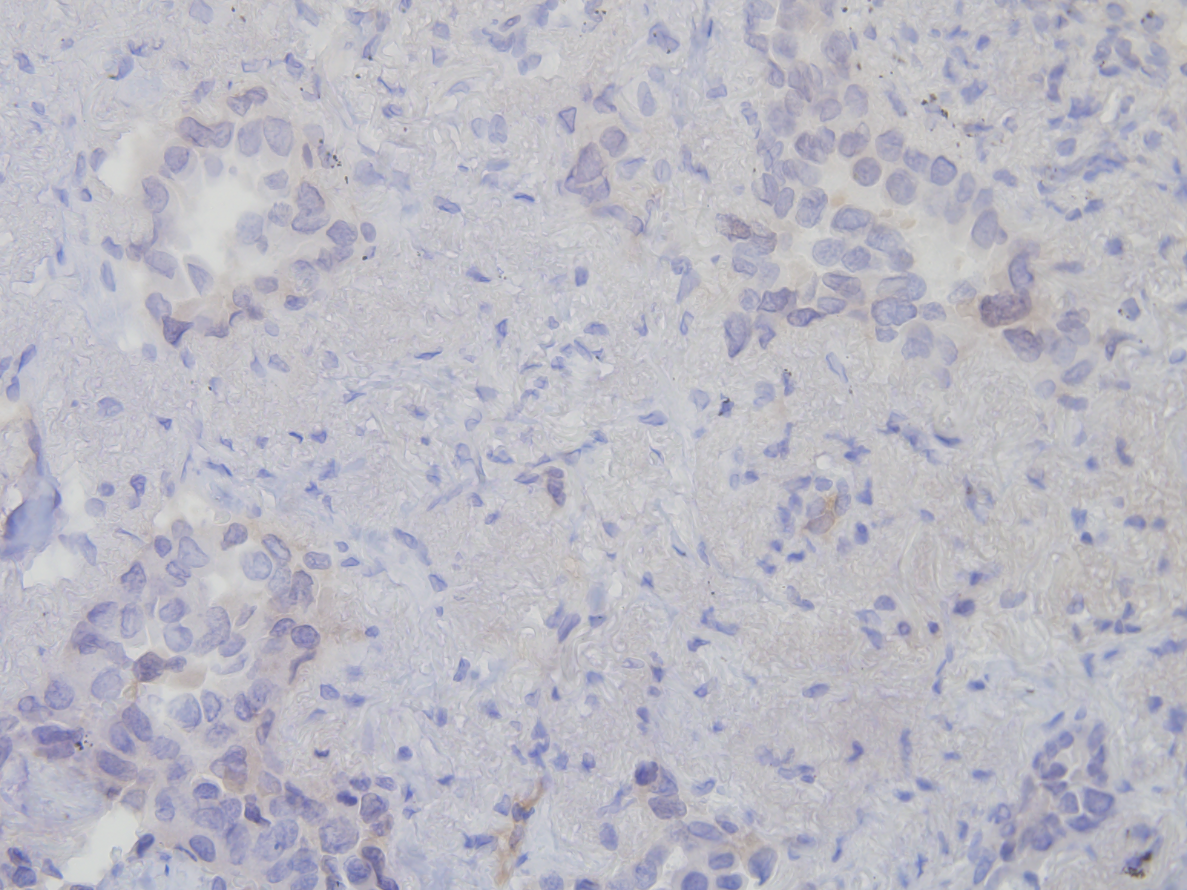

Supplement: S47 File — (ZIP) [file pone.0337223.s048.zip › 507095-400X-N-CA/507095-400X-CA (3).tif]

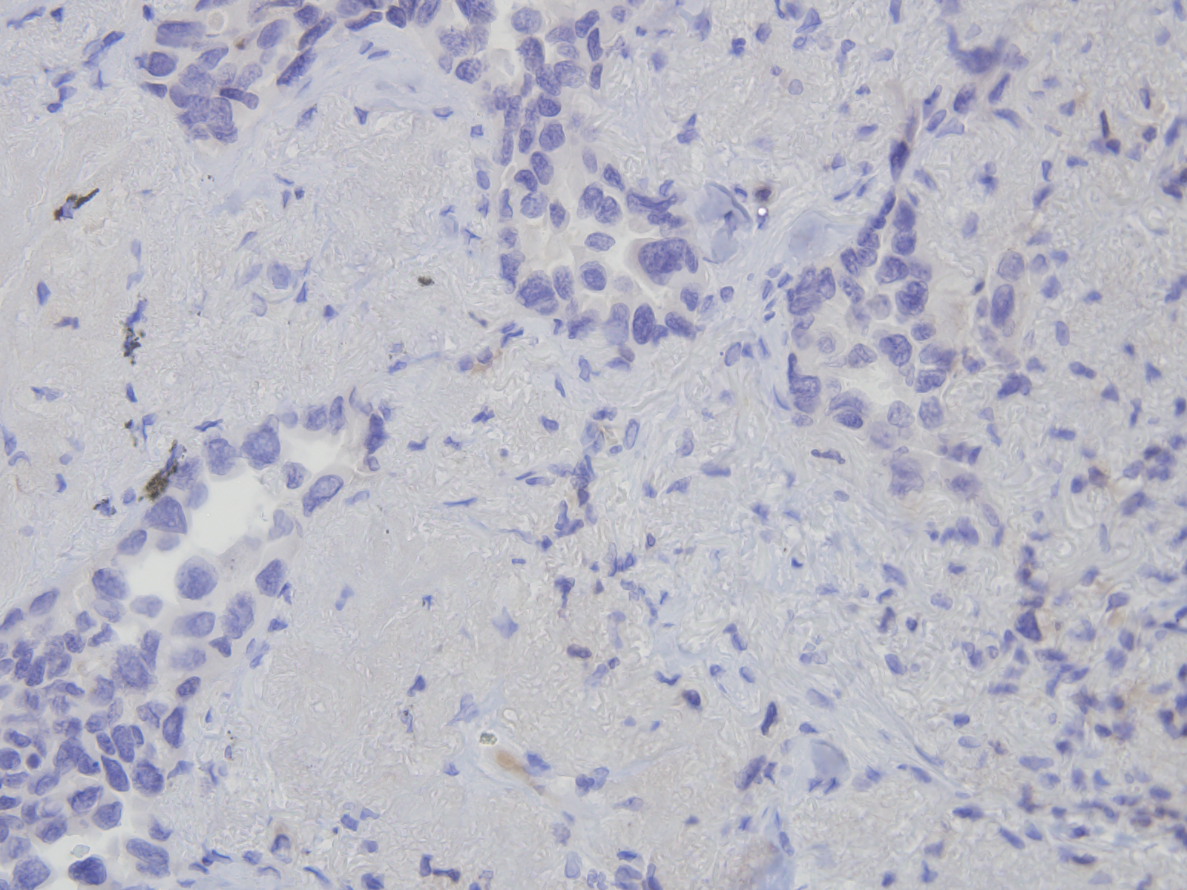

Supplement: S47 File — (ZIP) [file pone.0337223.s048.zip › 507095-400X-N-CA/507095-400X-CA (4).tif]

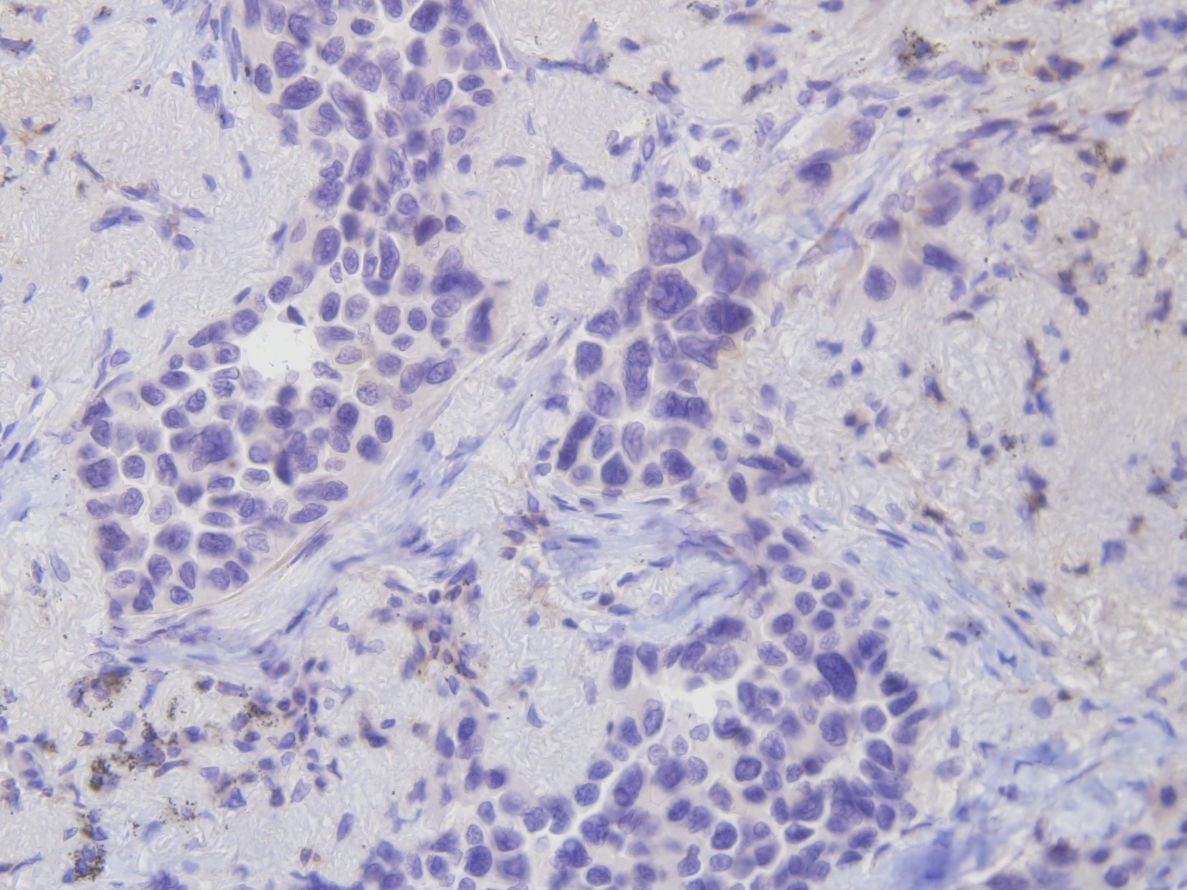

Supplement: S47 File — (ZIP) [file pone.0337223.s048.zip › 507095-400X-N-CA/507095-400X-CA (5).tif]

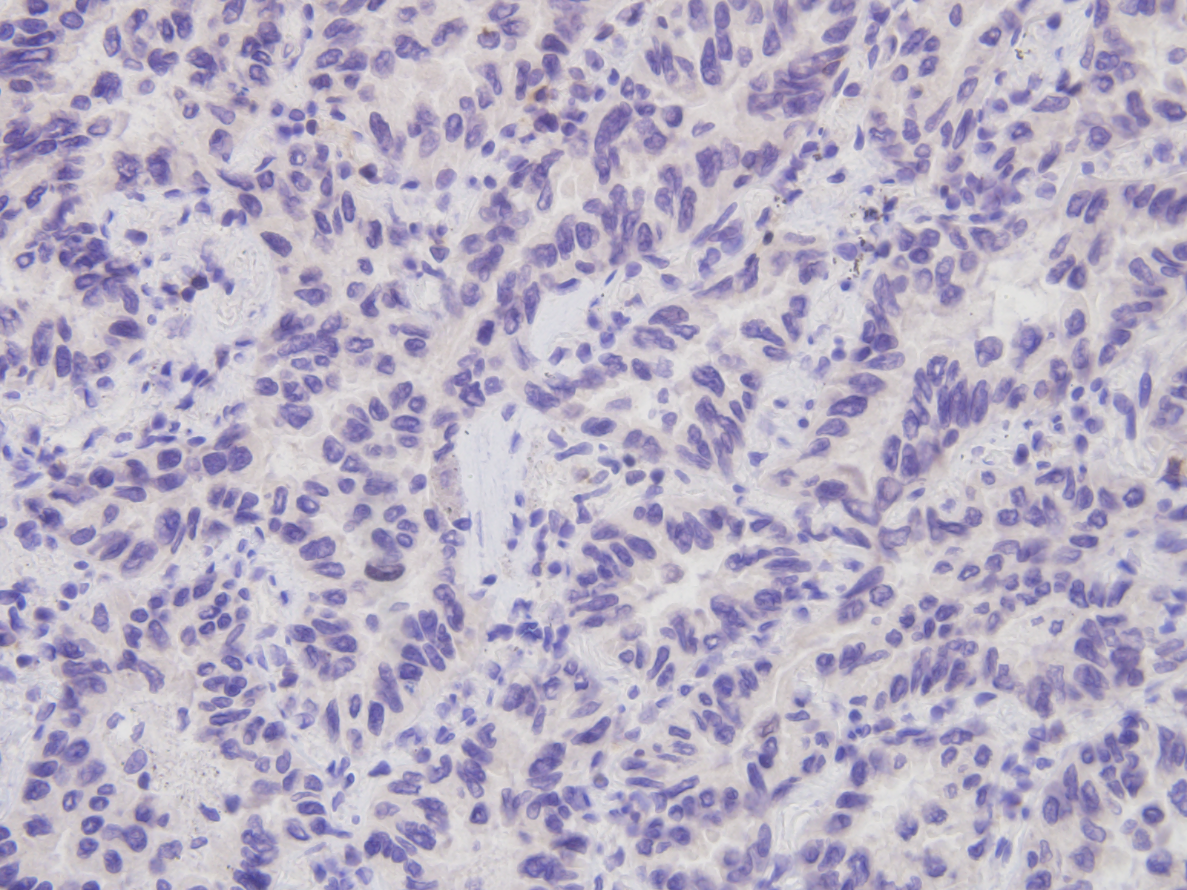

Supplement: S48 File — (ZIP) [file pone.0337223.s049.zip › 509703-400X-CA-N/509703-400X-CA (1).tif]

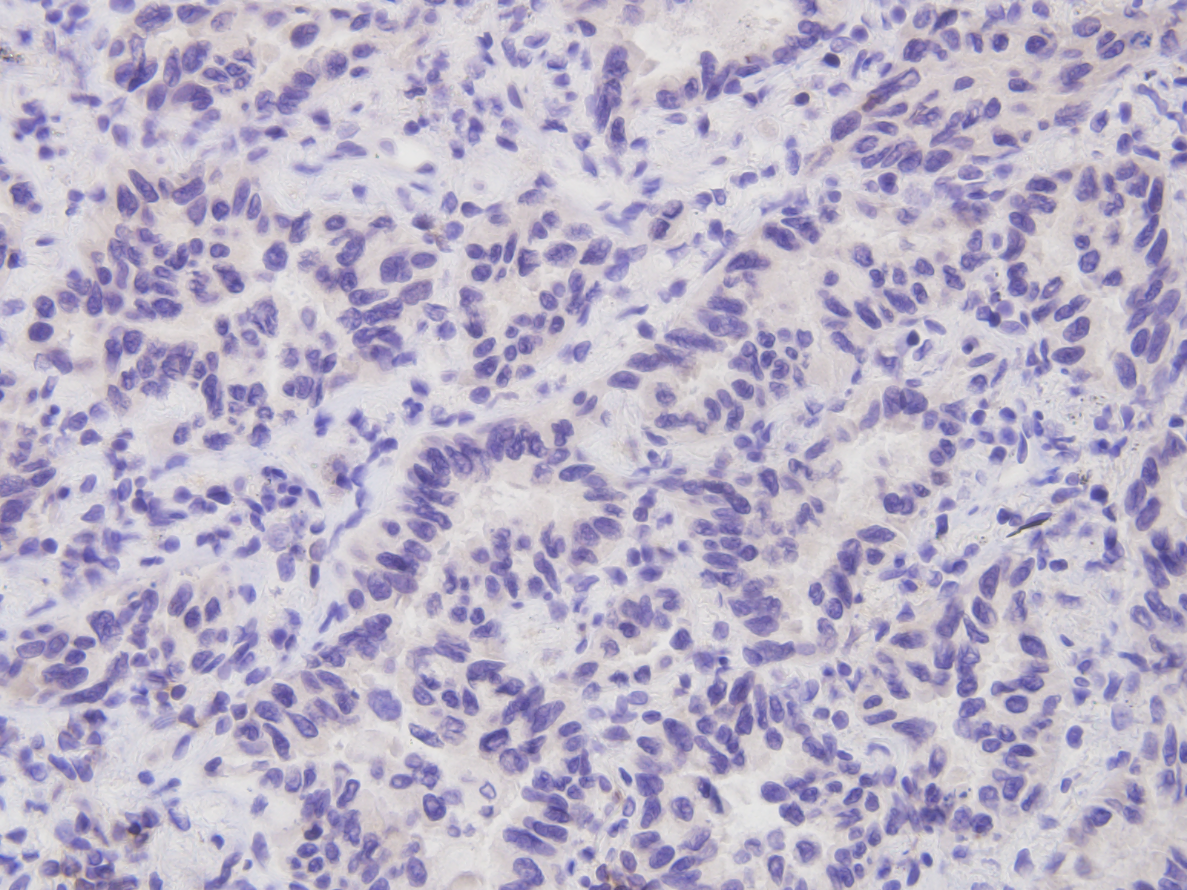

Supplement: S48 File — (ZIP) [file pone.0337223.s049.zip › 509703-400X-CA-N/509703-400X-CA (2).tif]

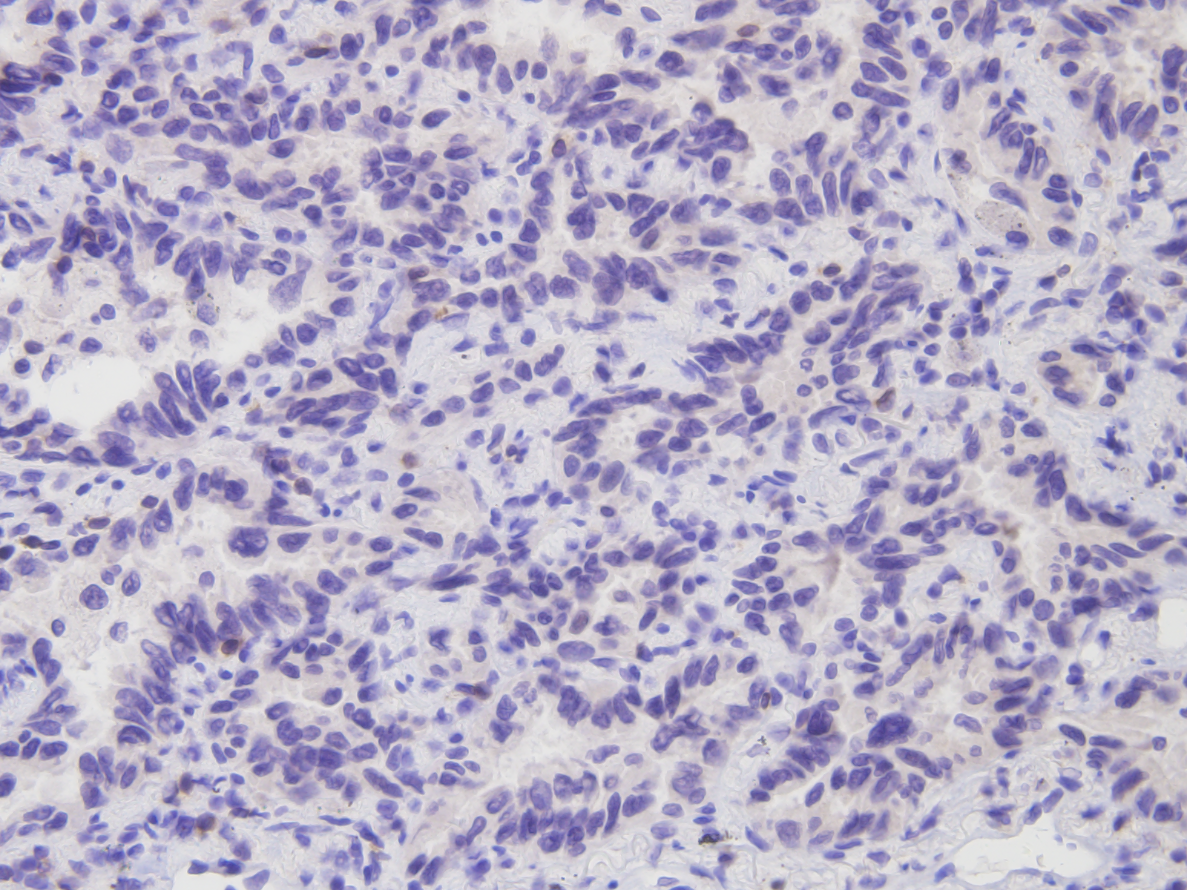

Supplement: S48 File — (ZIP) [file pone.0337223.s049.zip › 509703-400X-CA-N/509703-400X-CA (3).tif]

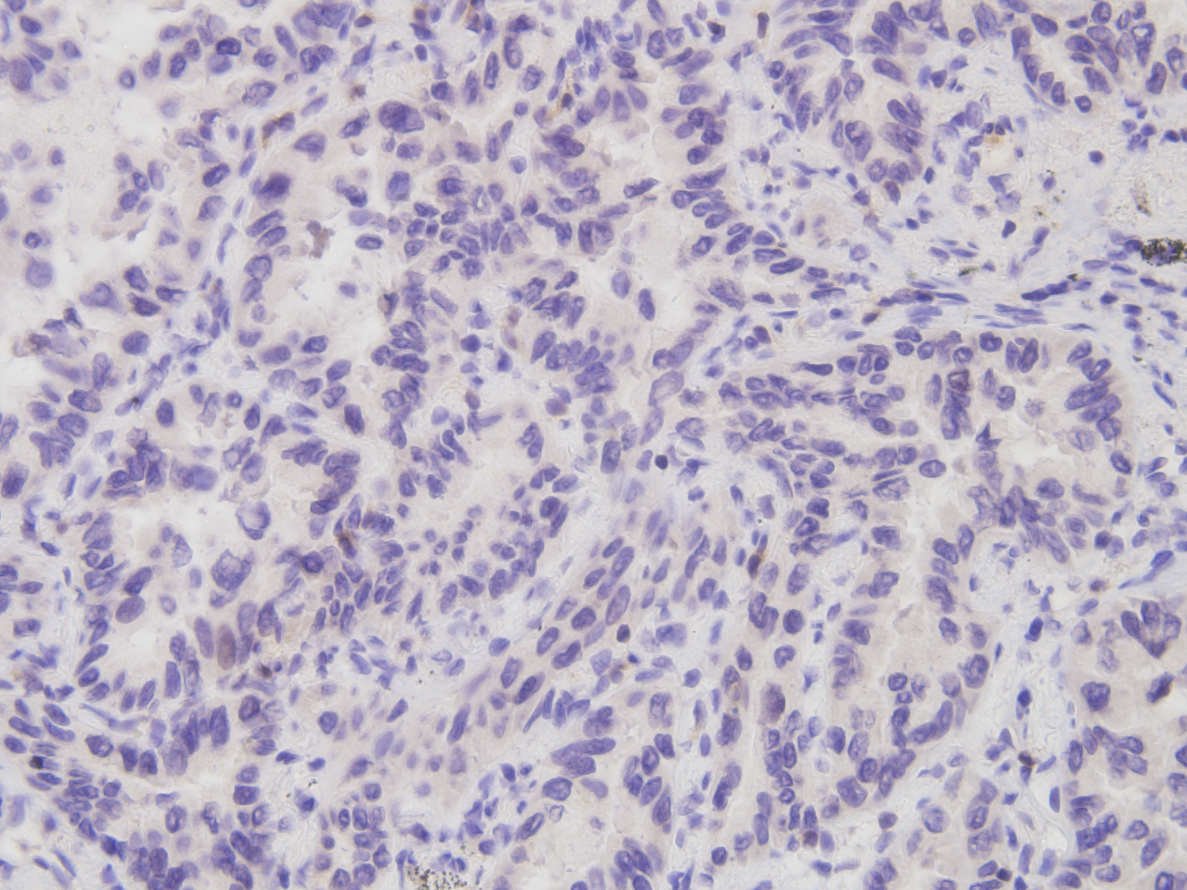

Supplement: S48 File — (ZIP) [file pone.0337223.s049.zip › 509703-400X-CA-N/509703-400X-CA (4).tif]

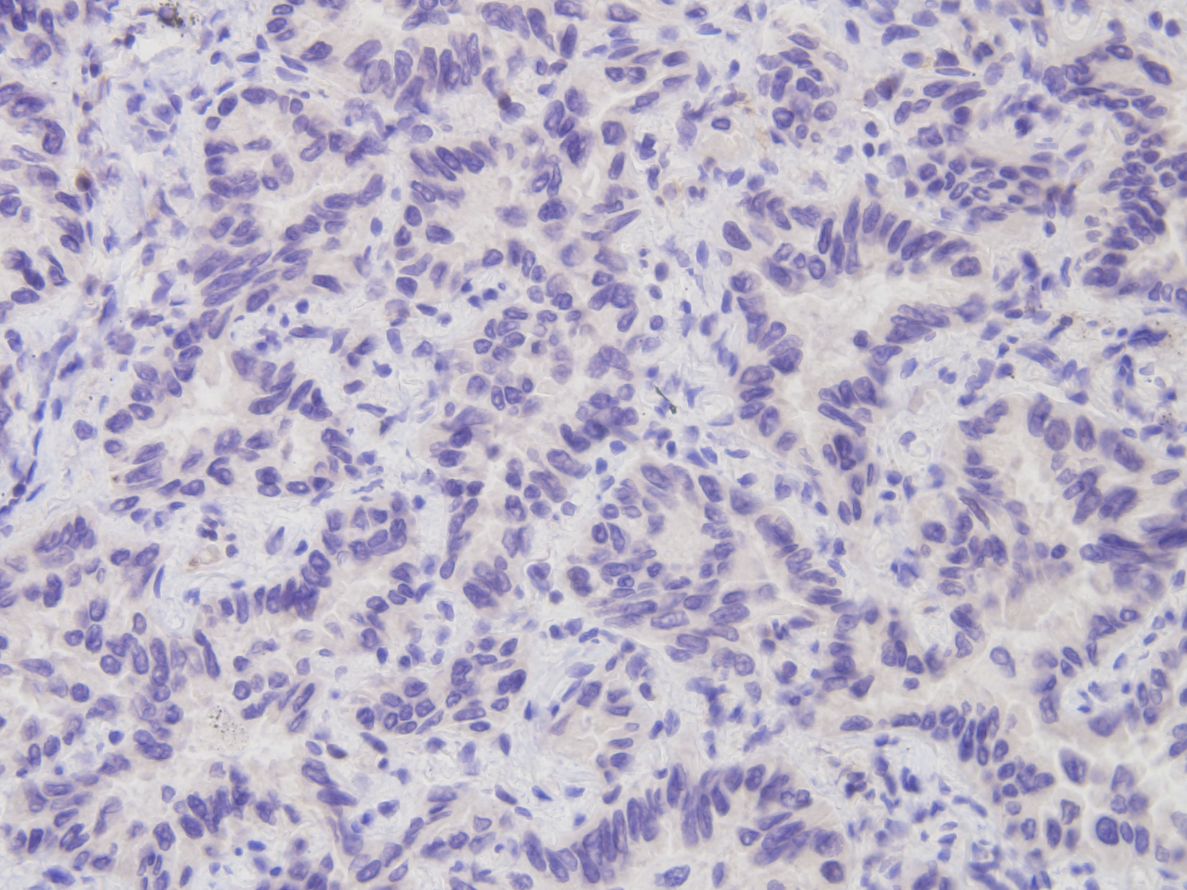

Supplement: S48 File — (ZIP) [file pone.0337223.s049.zip › 509703-400X-CA-N/509703-400X-CA (5).tif]

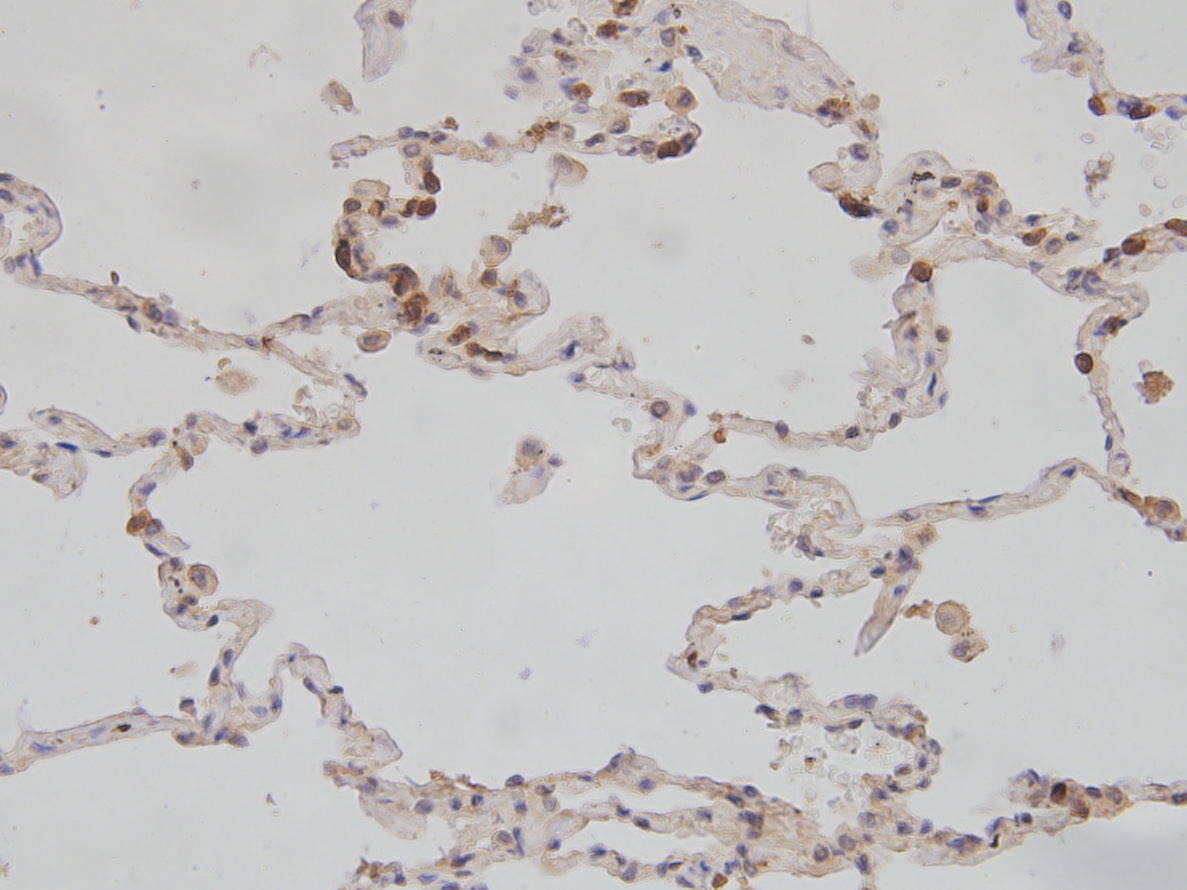

Supplement: S48 File — (ZIP) [file pone.0337223.s049.zip › 509703-400X-CA-N/509703-400X-N (1).tif]

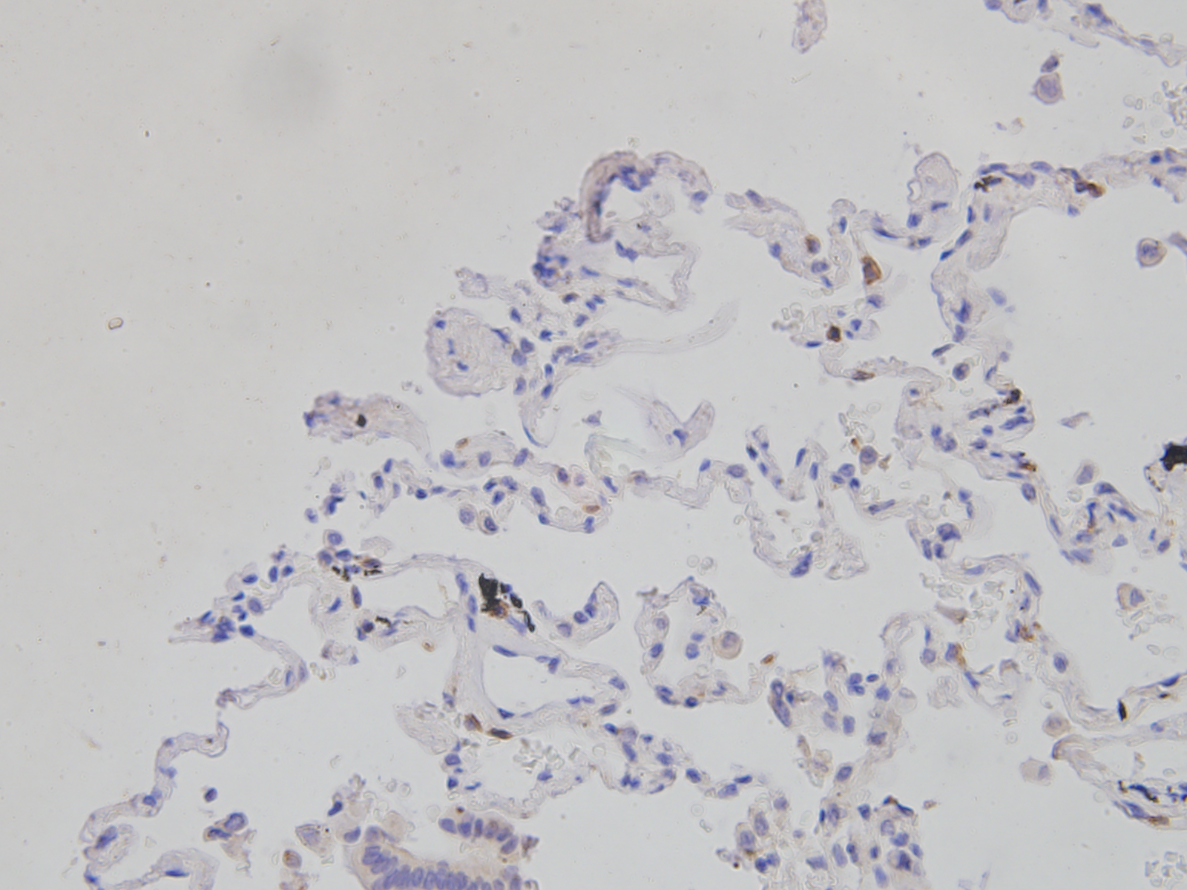

Supplement: S48 File — (ZIP) [file pone.0337223.s049.zip › 509703-400X-CA-N/509703-400X-N (2).tif]

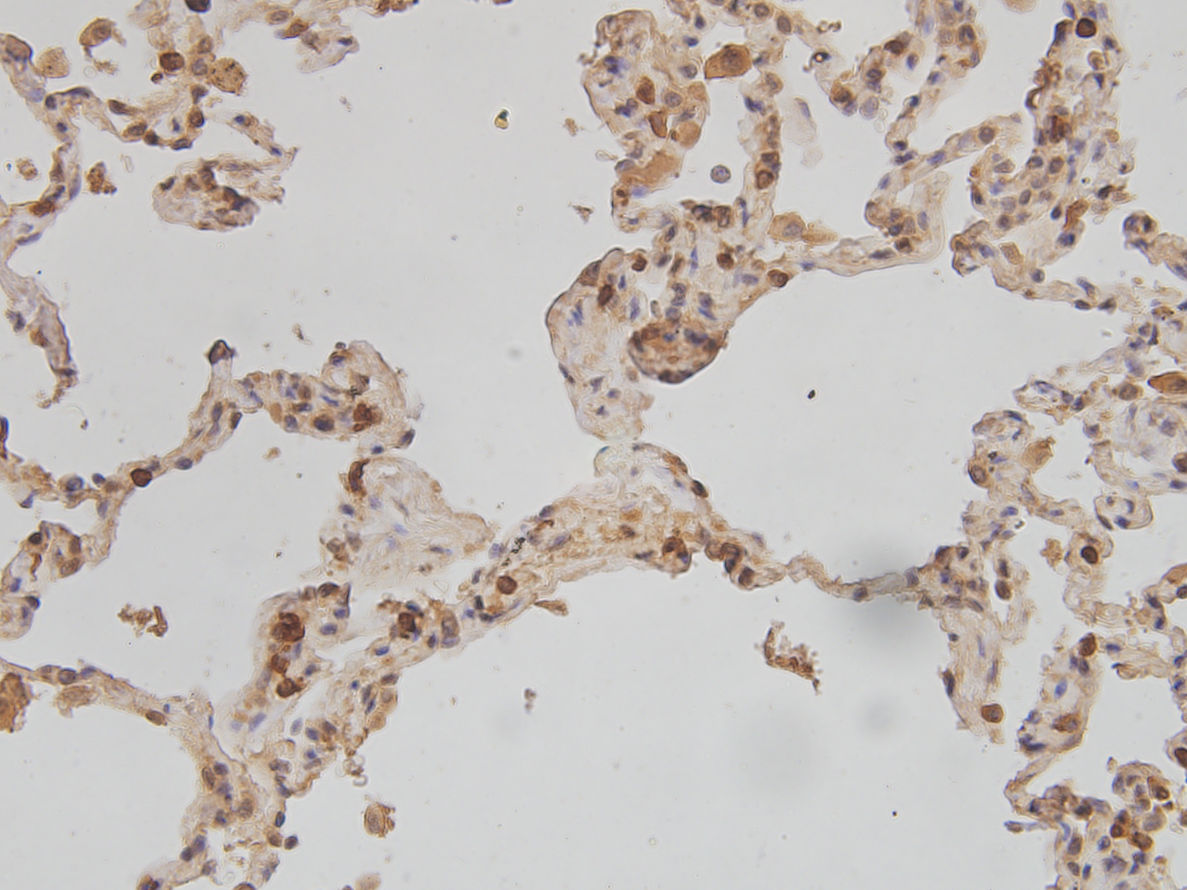

Supplement: S48 File — (ZIP) [file pone.0337223.s049.zip › 509703-400X-CA-N/509703-400X-N (3).tif]

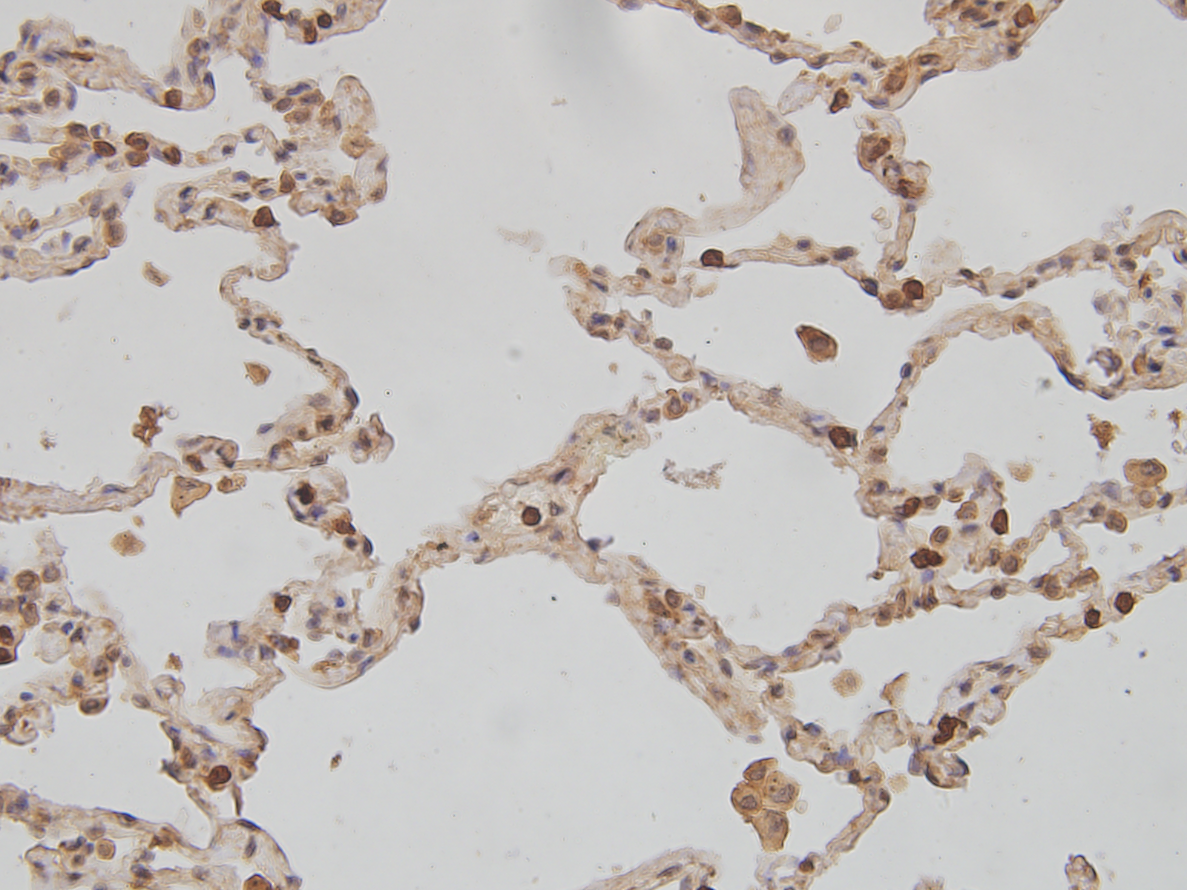

Supplement: S48 File — (ZIP) [file pone.0337223.s049.zip › 509703-400X-CA-N/509703-400X-N (4).tif]

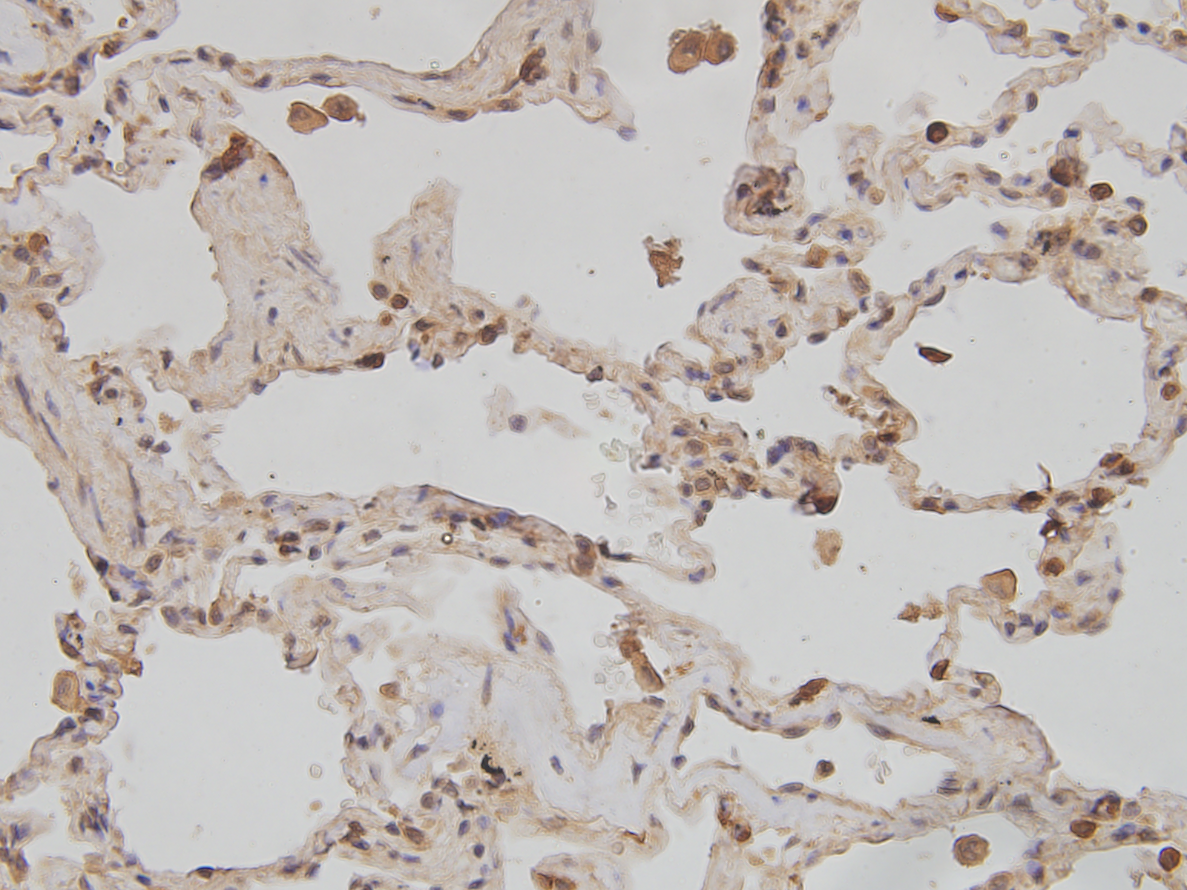

Supplement: S48 File — (ZIP) [file pone.0337223.s049.zip › 509703-400X-CA-N/509703-400X-N (5).tif]

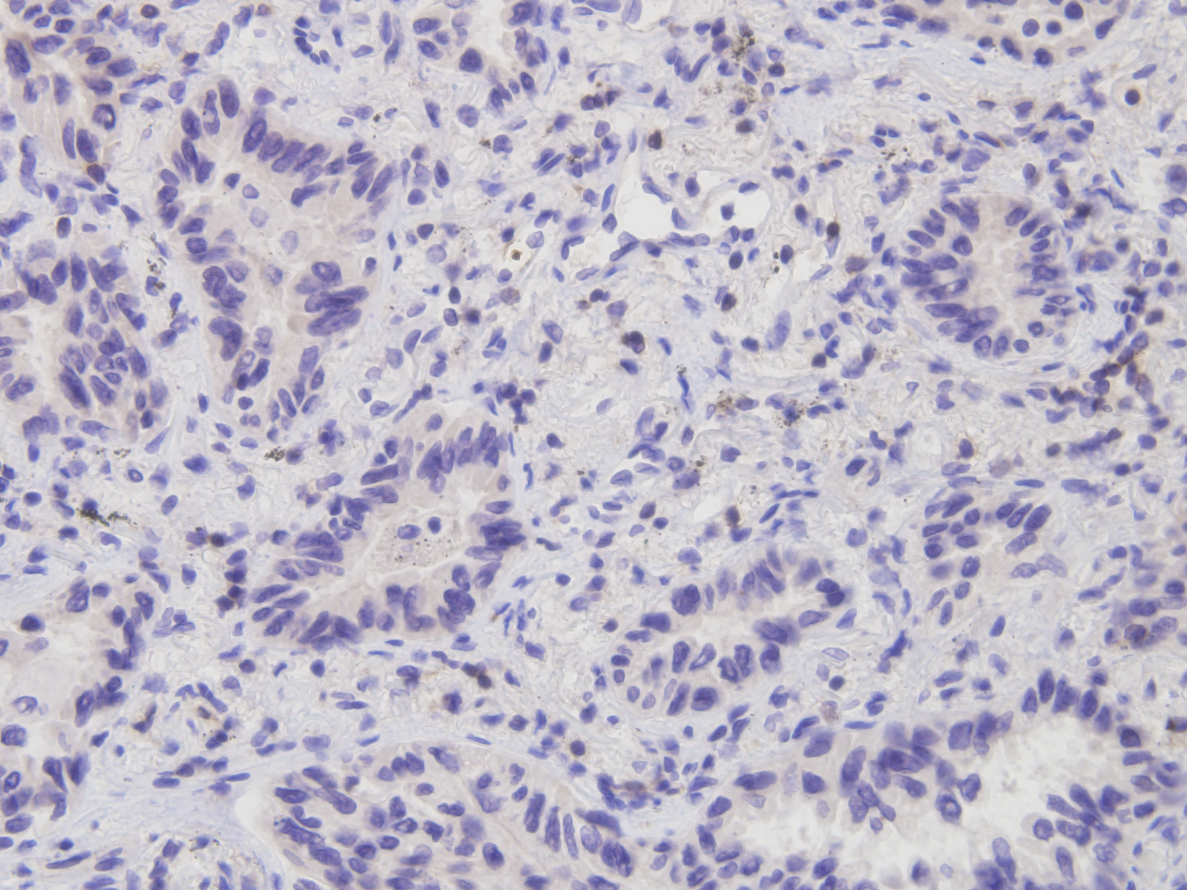

Supplement: S49 File — (ZIP) [file pone.0337223.s050.zip › 509863-400X-N-CA/509863-400X-CA (1).tif]

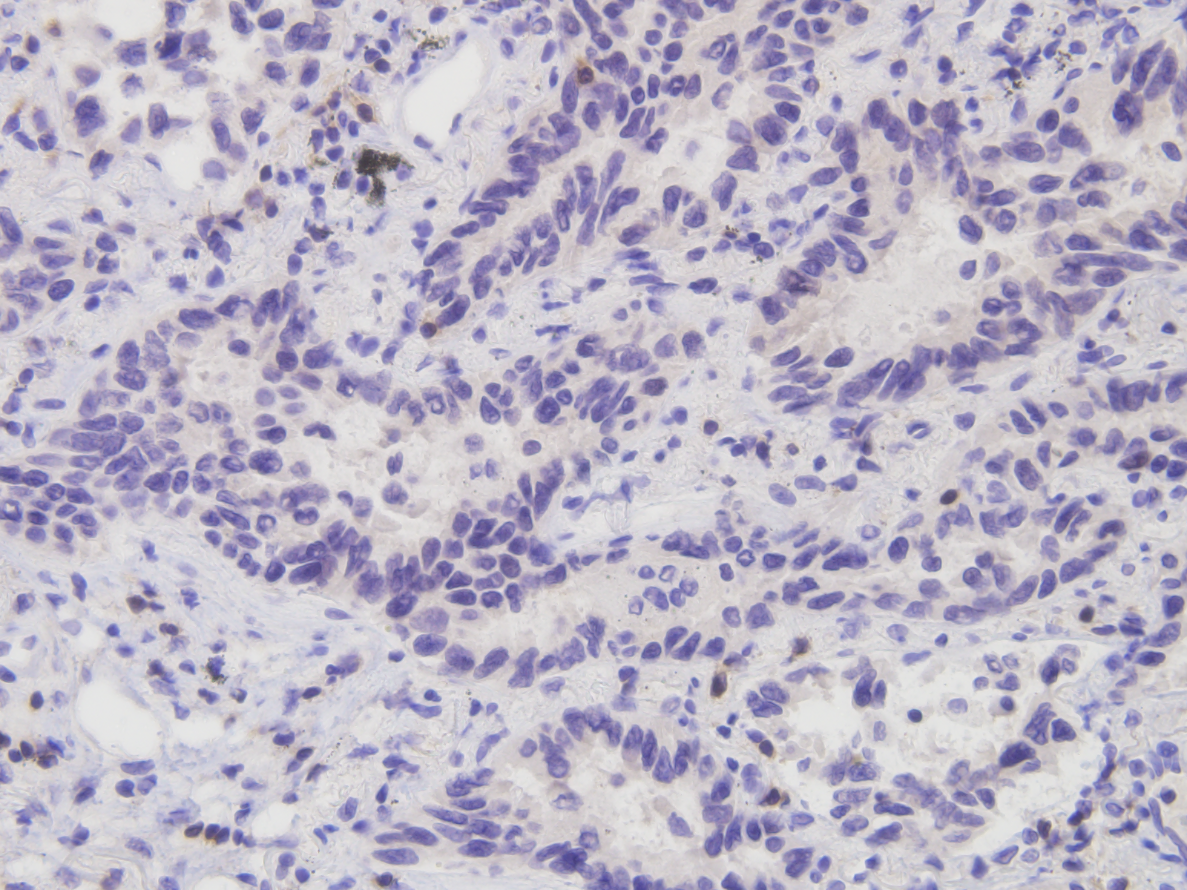

Supplement: S49 File — (ZIP) [file pone.0337223.s050.zip › 509863-400X-N-CA/509863-400X-CA (2).tif]

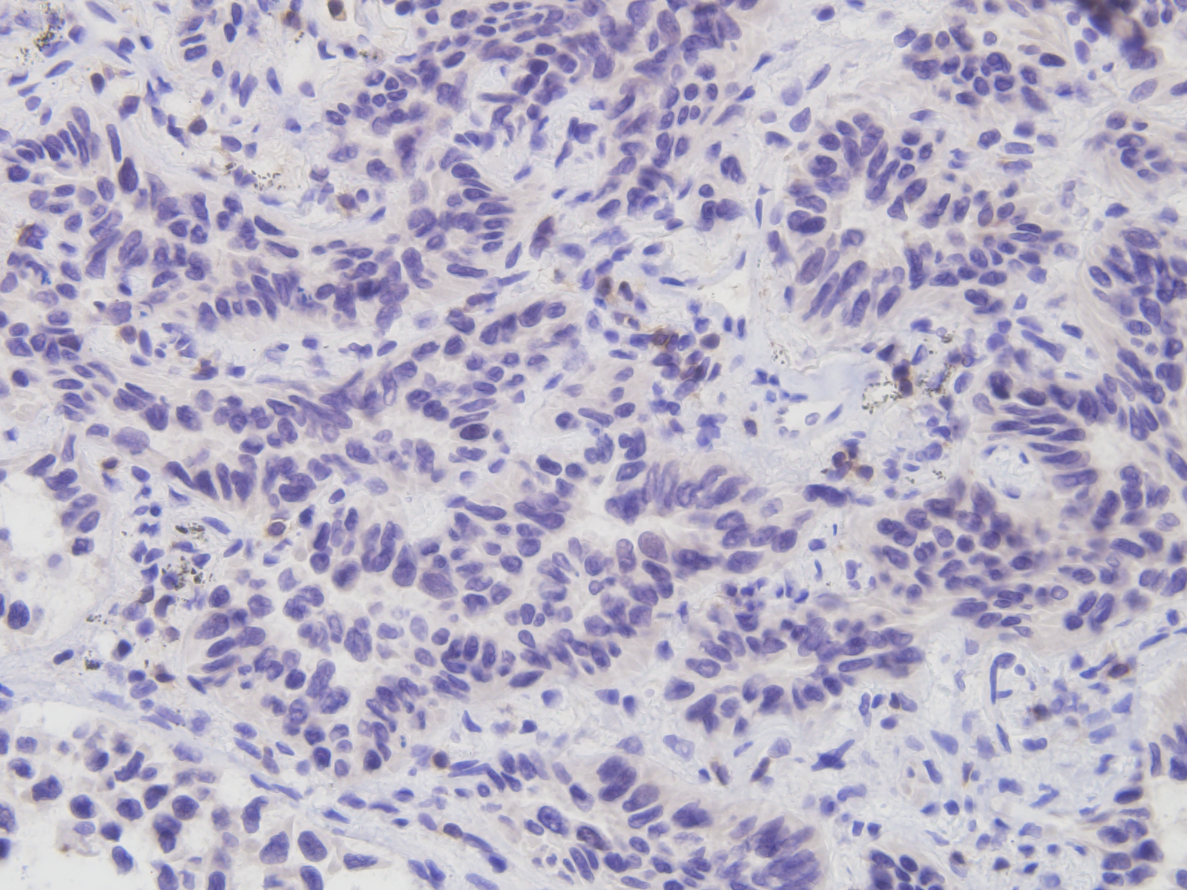

Supplement: S49 File — (ZIP) [file pone.0337223.s050.zip › 509863-400X-N-CA/509863-400X-CA (3).tif]

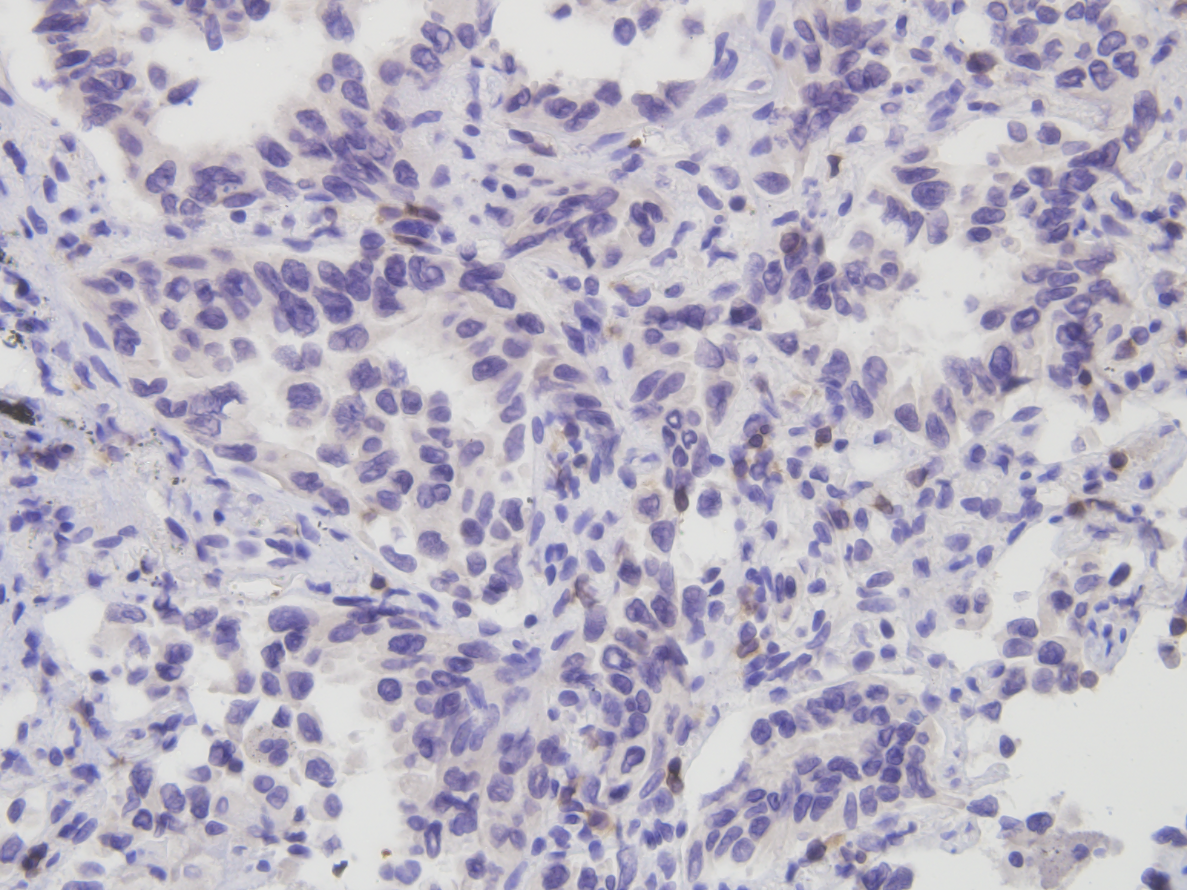

Supplement: S49 File — (ZIP) [file pone.0337223.s050.zip › 509863-400X-N-CA/509863-400X-CA (4).tif]

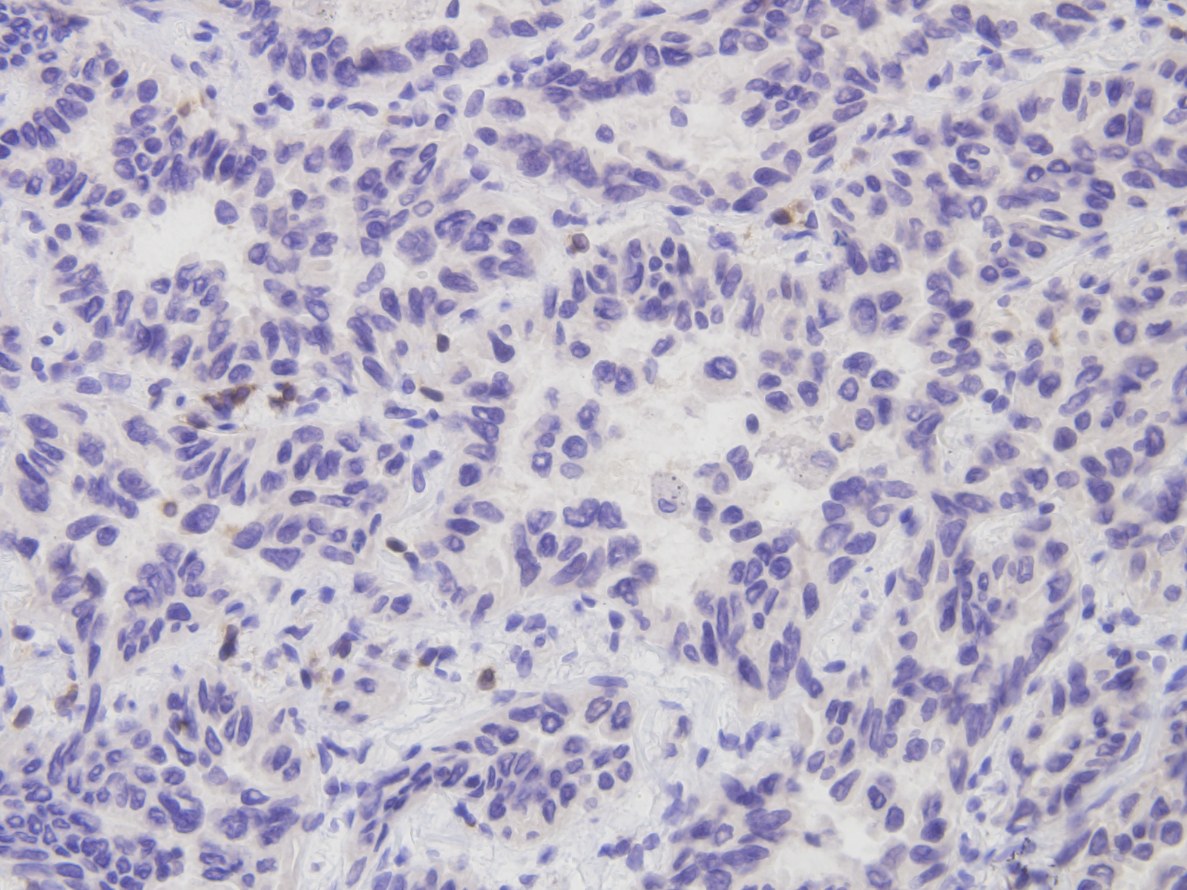

Supplement: S49 File — (ZIP) [file pone.0337223.s050.zip › 509863-400X-N-CA/509863-400X-CA (5).tif]
