# Supplementary figures and images for: Overexpression of PTPRCAP inhibits biological function of lung adenocarcinoma through apoptosis pathway (part 5 of 5)
Source: PLoS One. 2025 Dec 18;20(12):e0337223. doi: 10.1371/journal.pone.0337223 (PMC12716888; doi:10.1371/journal.pone.0337223)

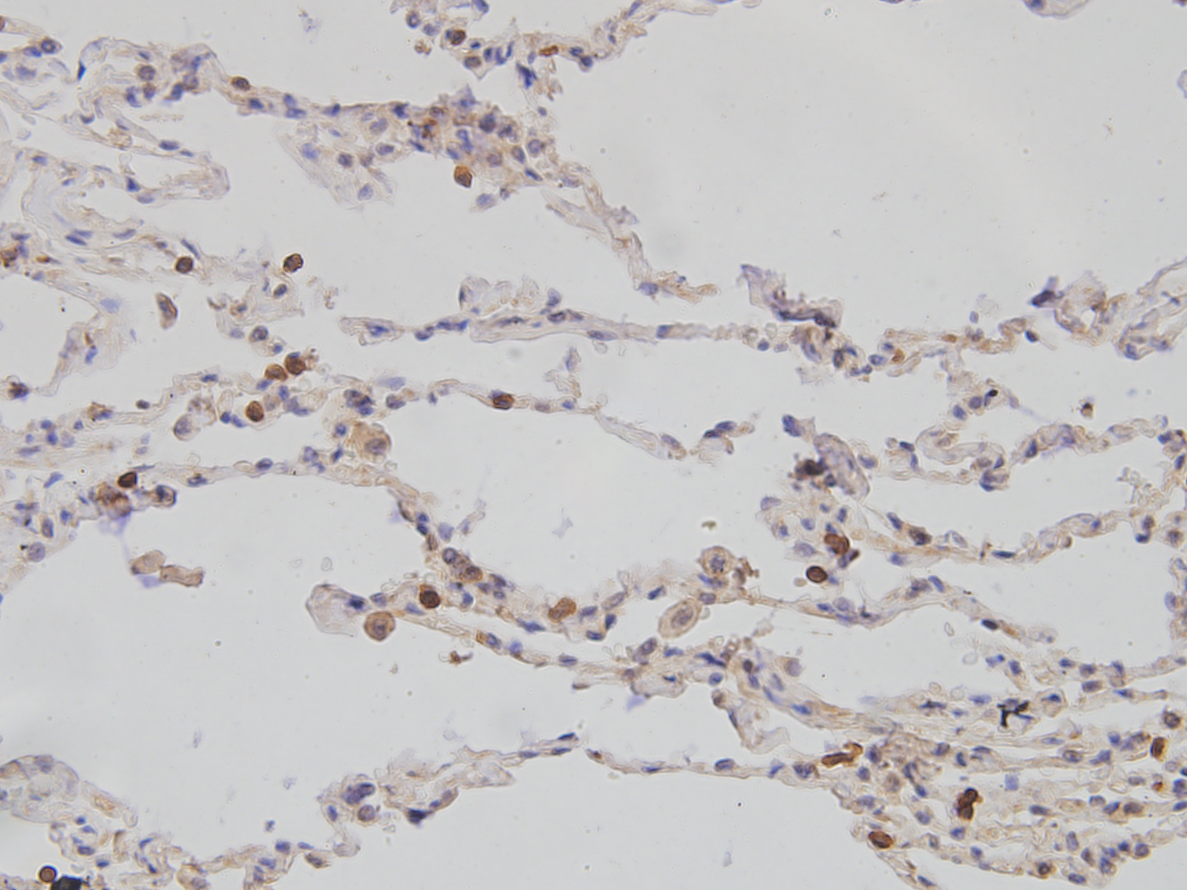

Supplement: S49 File — (ZIP) [file pone.0337223.s050.zip › 509863-400X-N-CA/509863-400X-N (1).tif]

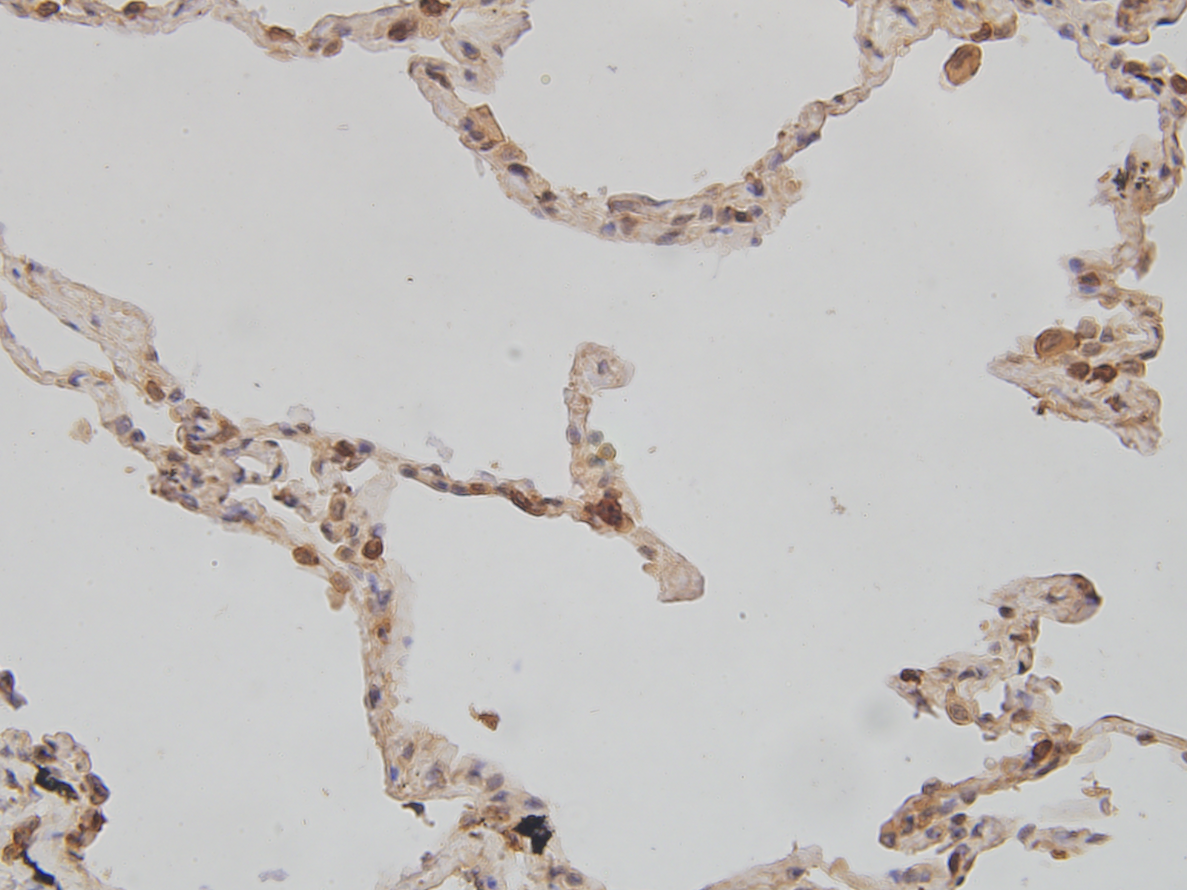

Supplement: S49 File — (ZIP) [file pone.0337223.s050.zip › 509863-400X-N-CA/509863-400X-N (2).tif]

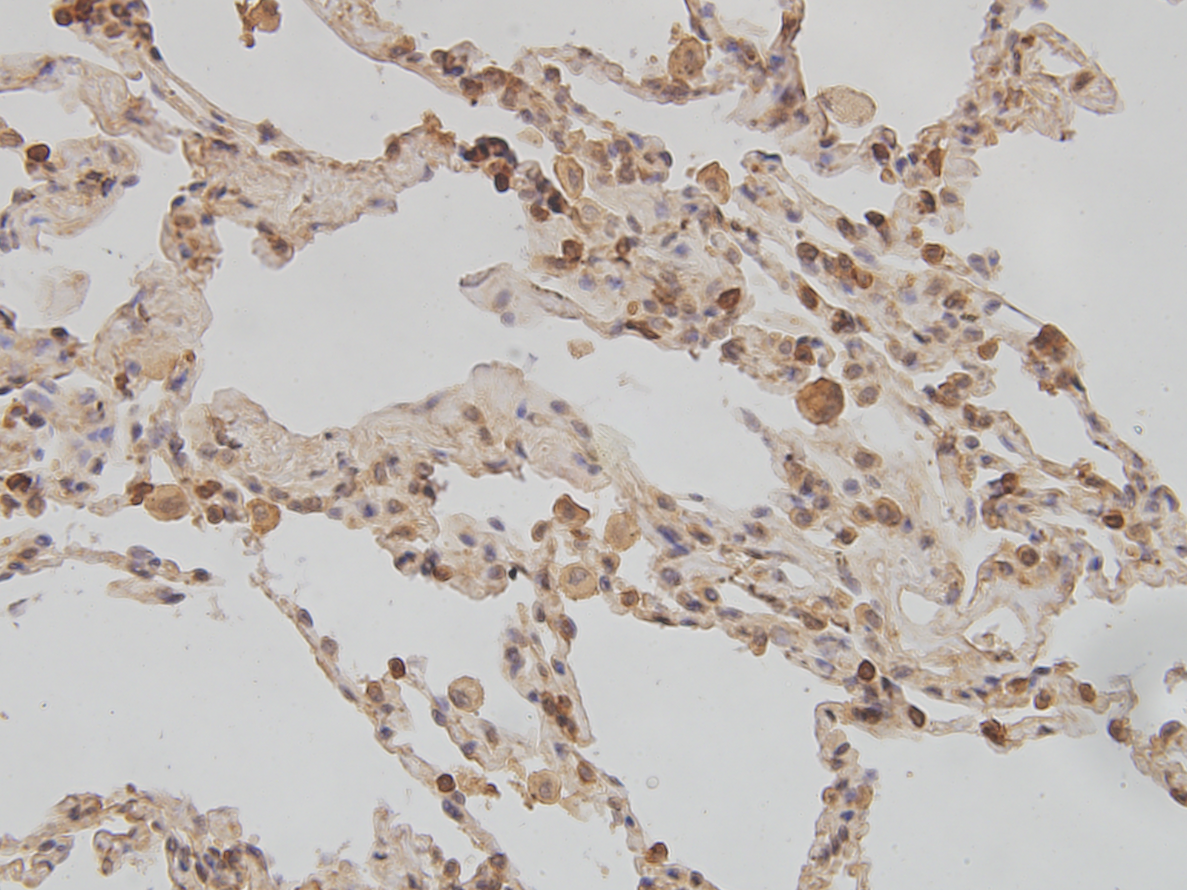

Supplement: S49 File — (ZIP) [file pone.0337223.s050.zip › 509863-400X-N-CA/509863-400X-N (3).tif]

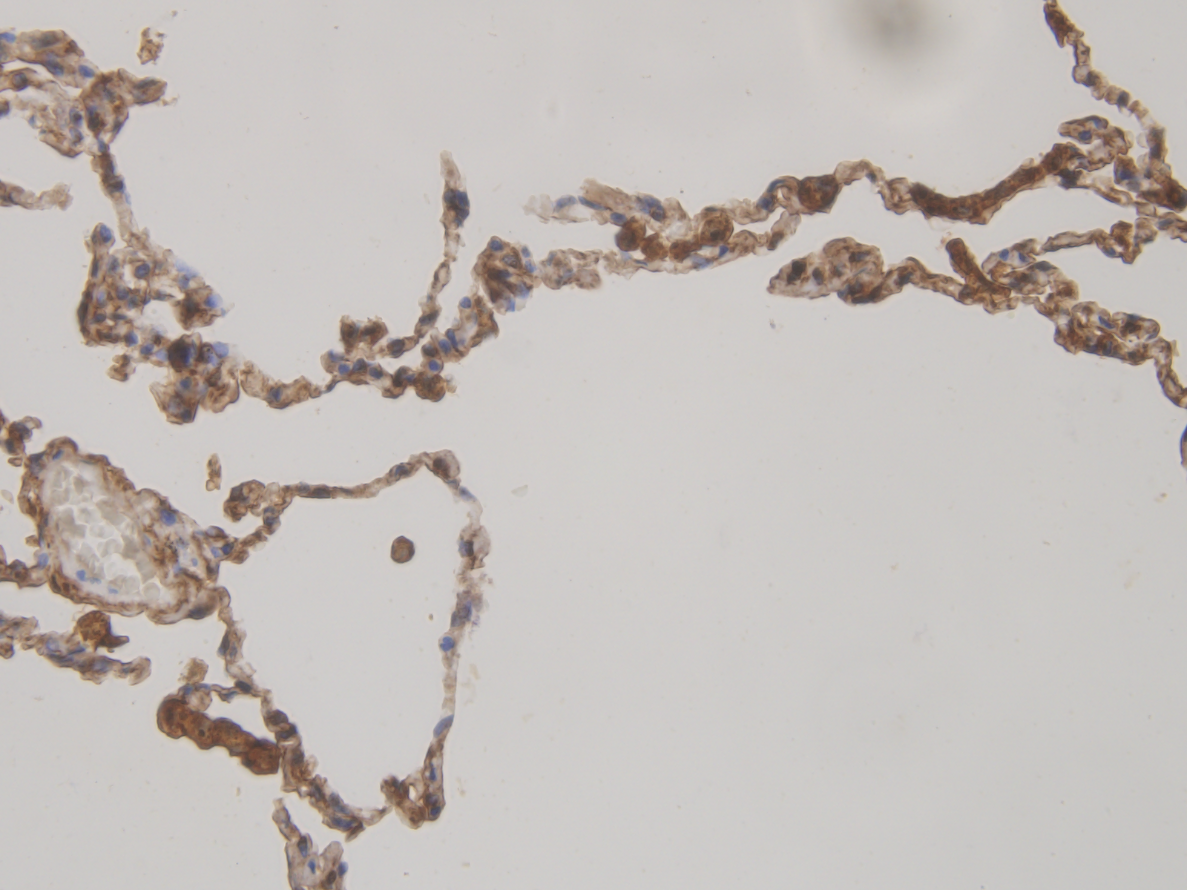

Supplement: S49 File — (ZIP) [file pone.0337223.s050.zip › 509863-400X-N-CA/509863-400X-N (4).tif]

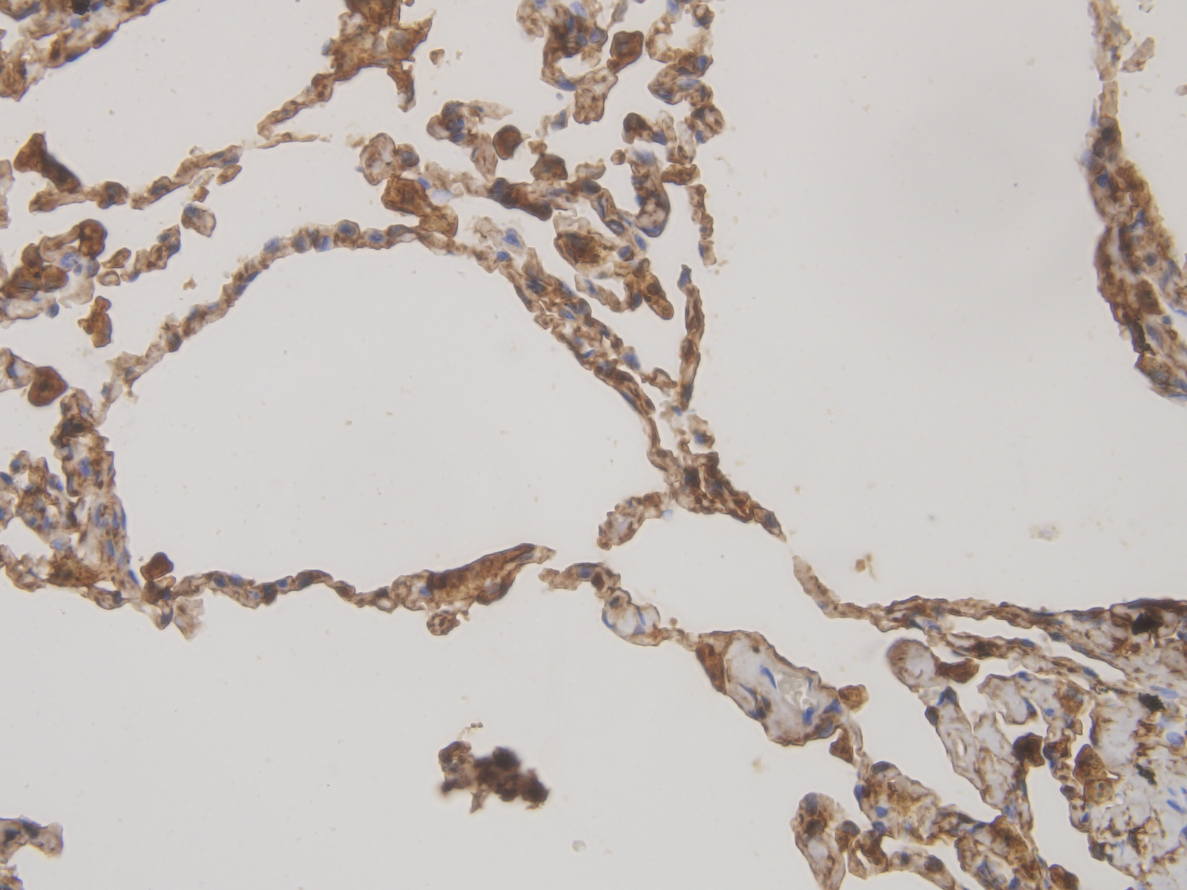

Supplement: S49 File — (ZIP) [file pone.0337223.s050.zip › 509863-400X-N-CA/509863-400X-N (5).tif]

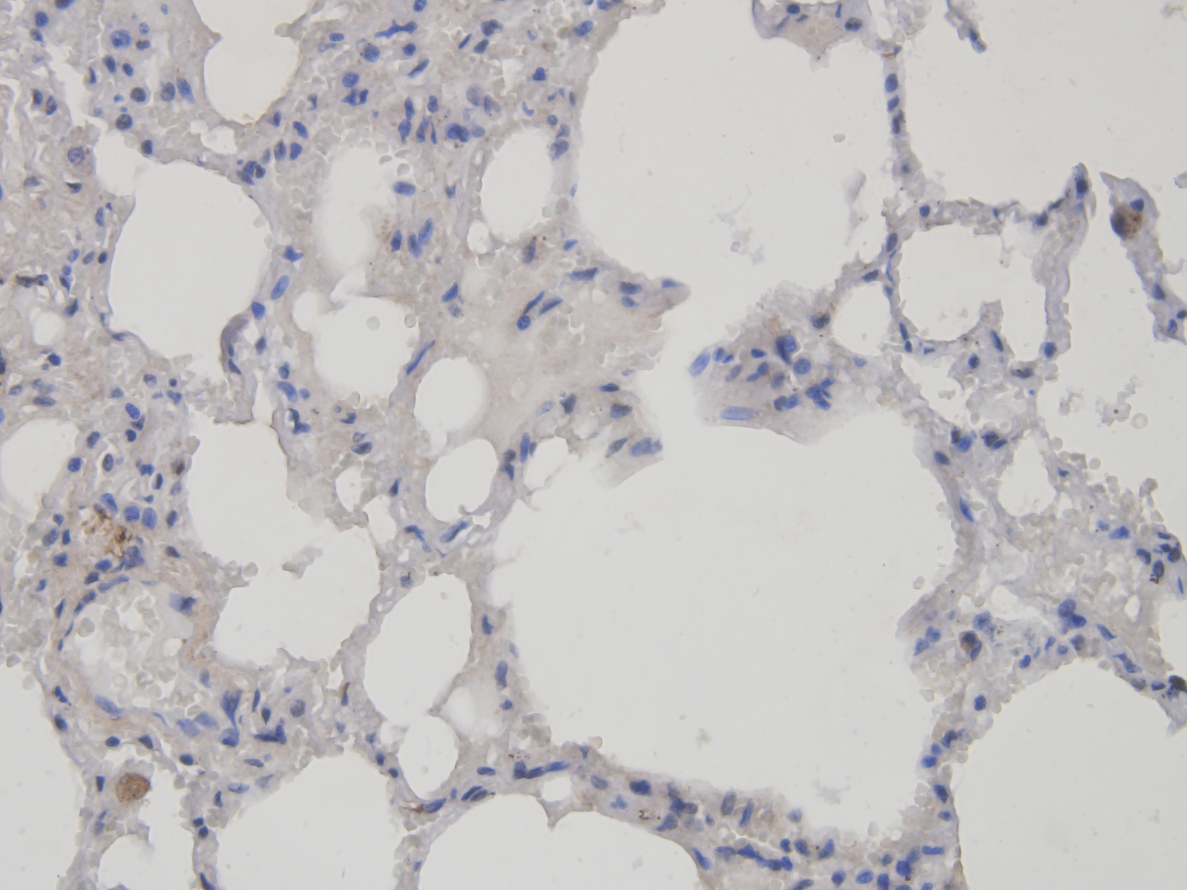

Supplement: S50 File — (ZIP) [file pone.0337223.s051.zip › 511430-400-CA-N/511430-400- N (1).tif]

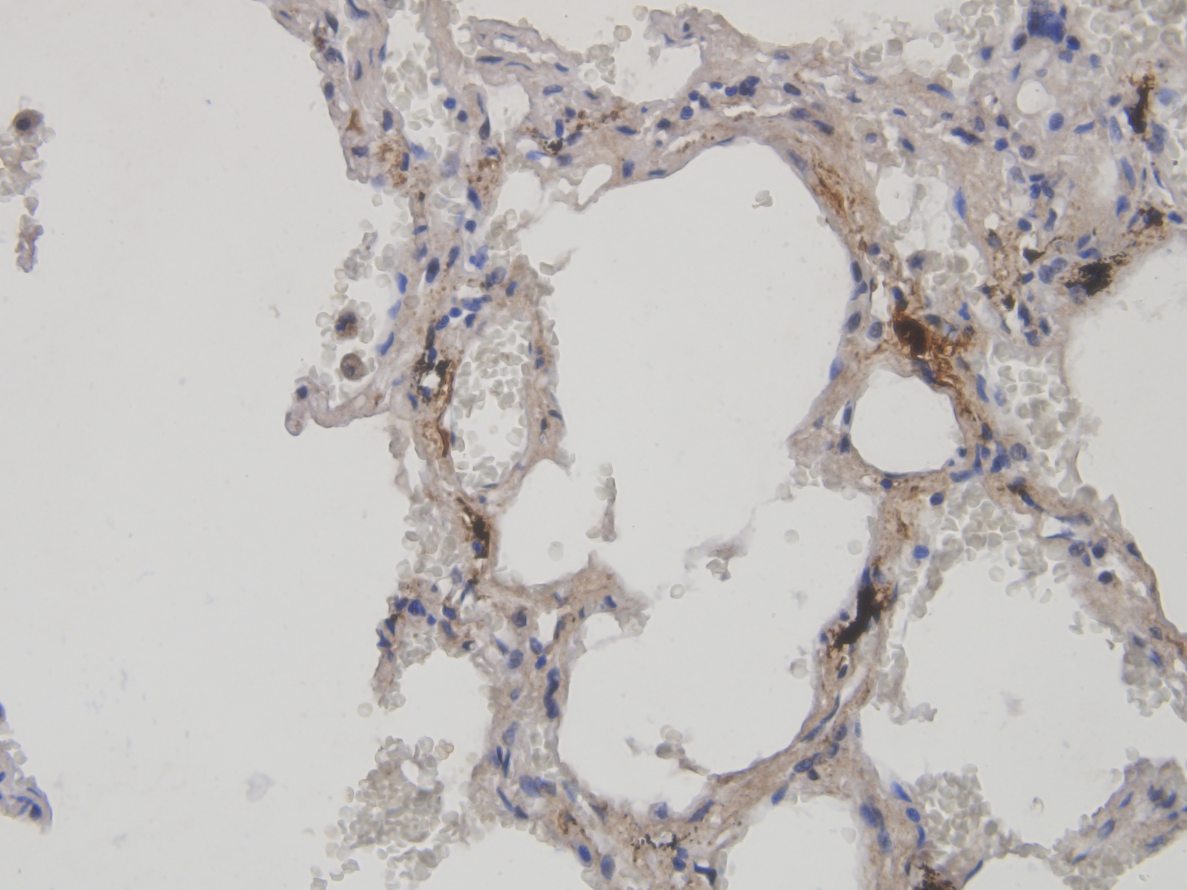

Supplement: S50 File — (ZIP) [file pone.0337223.s051.zip › 511430-400-CA-N/511430-400- N (2).tif]

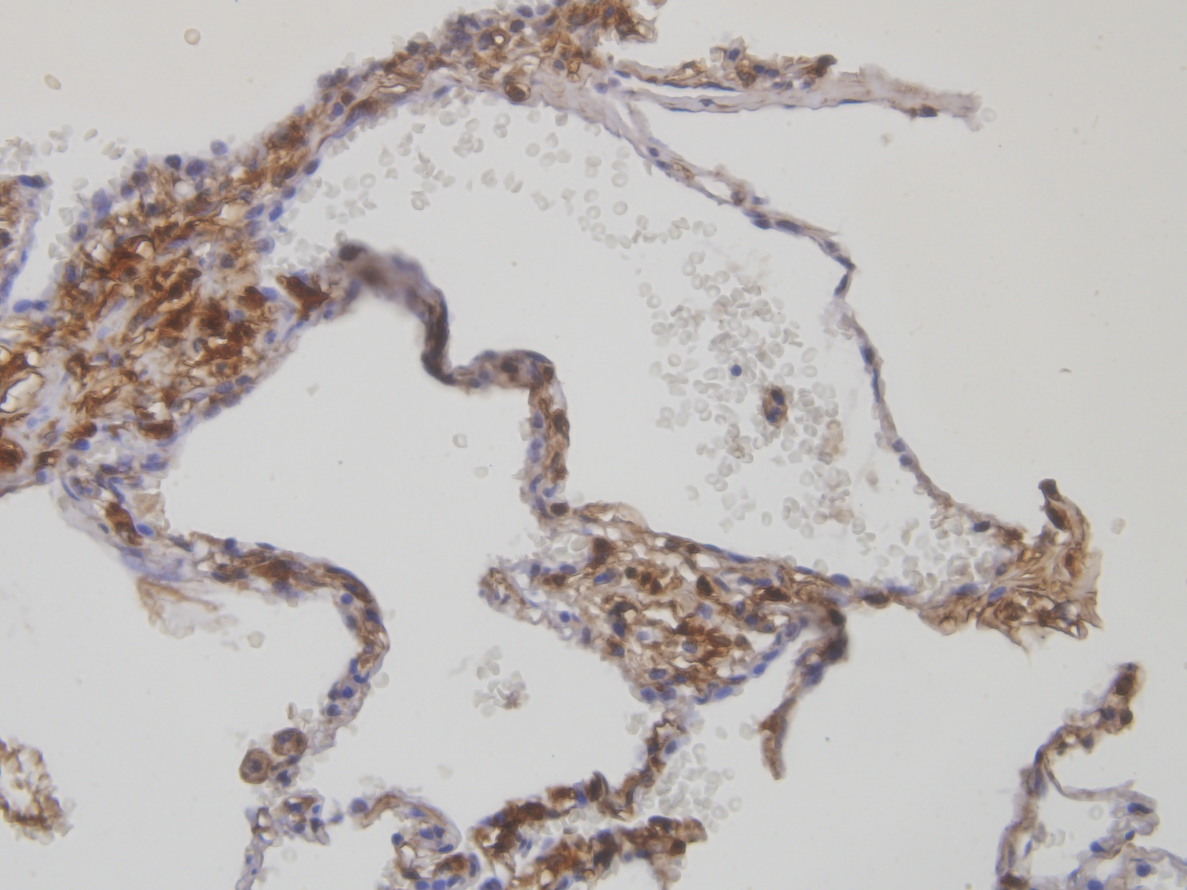

Supplement: S50 File — (ZIP) [file pone.0337223.s051.zip › 511430-400-CA-N/511430-400- N (3).tif]

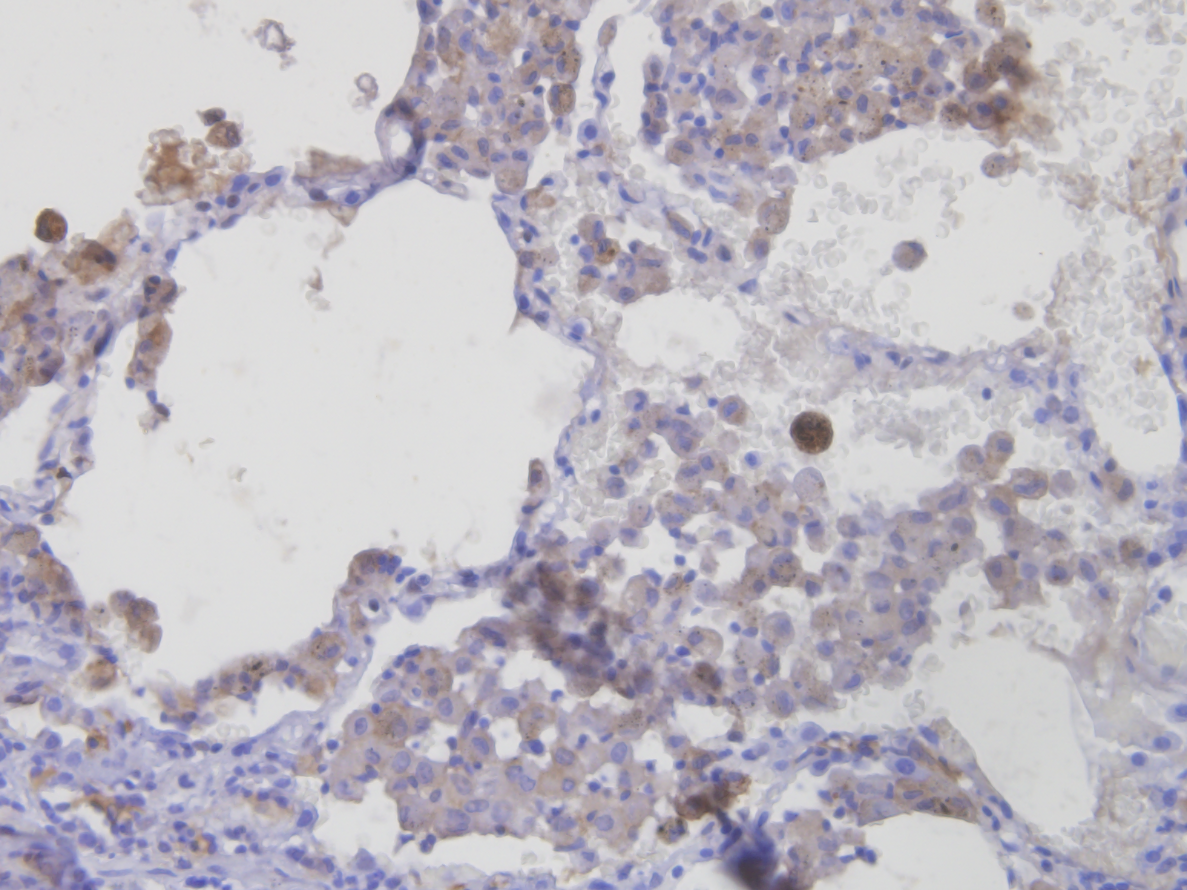

Supplement: S50 File — (ZIP) [file pone.0337223.s051.zip › 511430-400-CA-N/511430-400- N (4).tif]

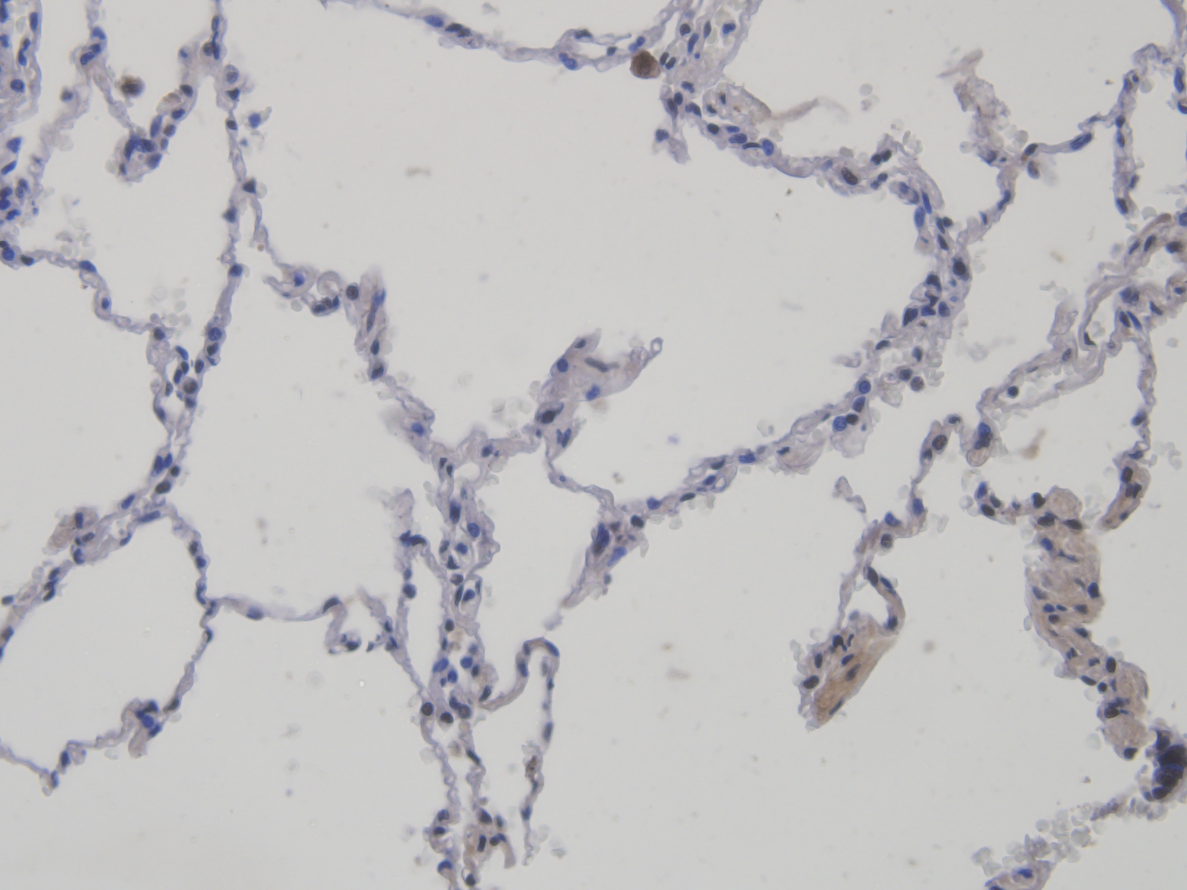

Supplement: S50 File — (ZIP) [file pone.0337223.s051.zip › 511430-400-CA-N/511430-400- N (5).tif]

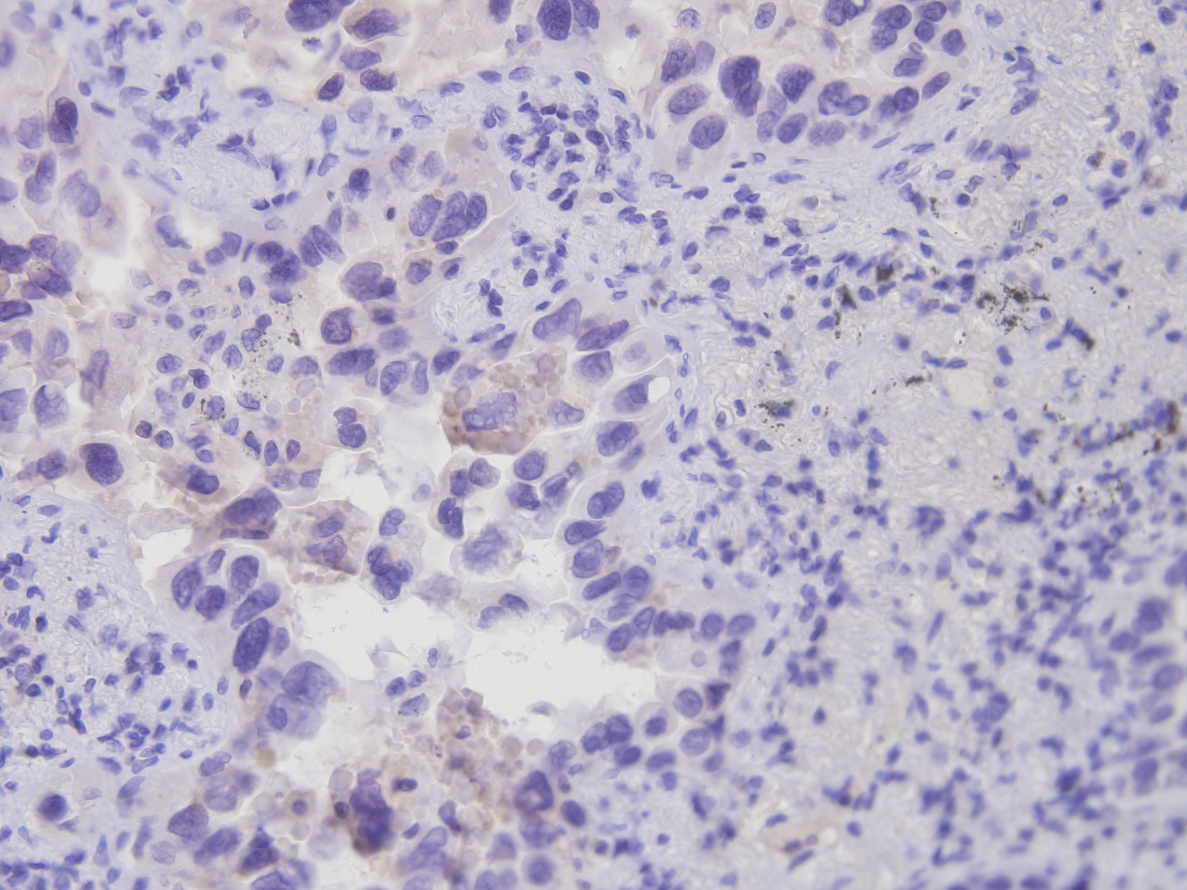

Supplement: S50 File — (ZIP) [file pone.0337223.s051.zip › 511430-400-CA-N/511430-400-CA (1).tif]

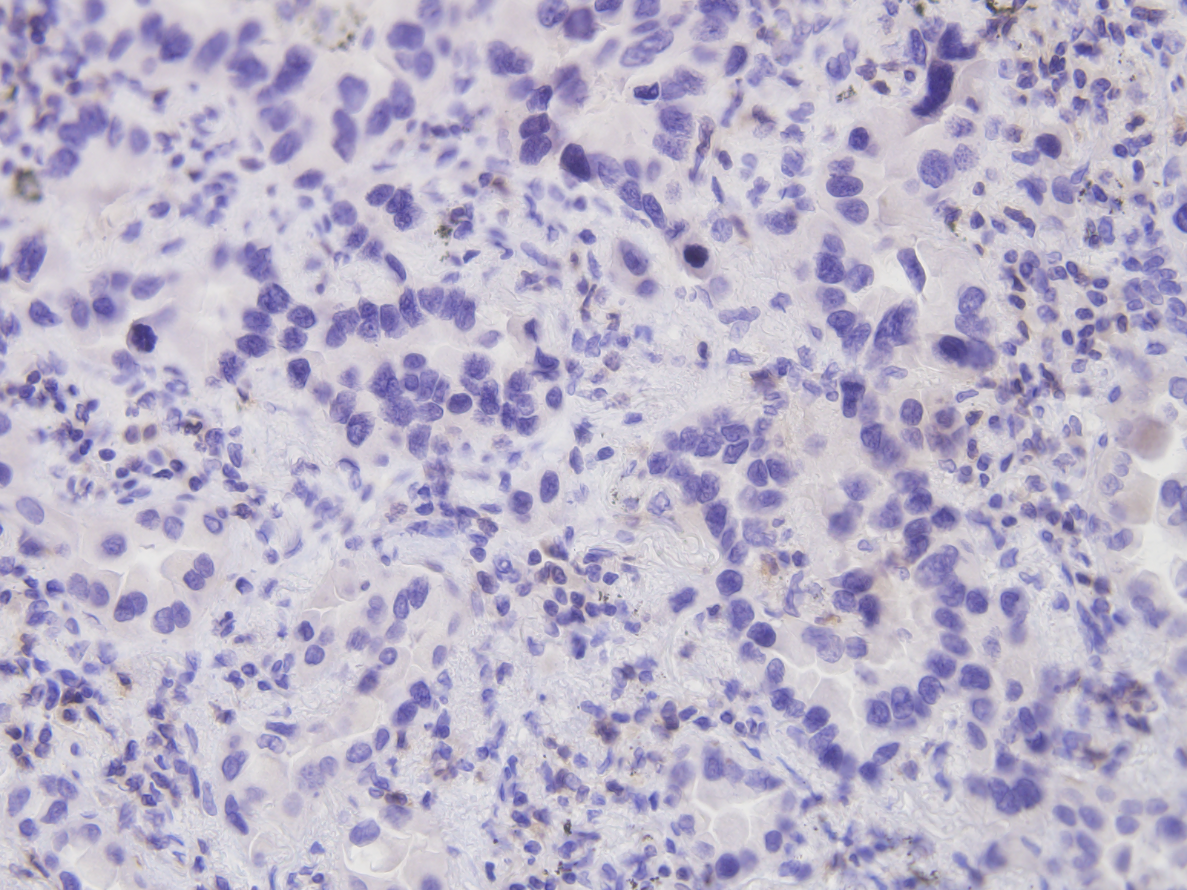

Supplement: S50 File — (ZIP) [file pone.0337223.s051.zip › 511430-400-CA-N/511430-400-CA (2).tif]

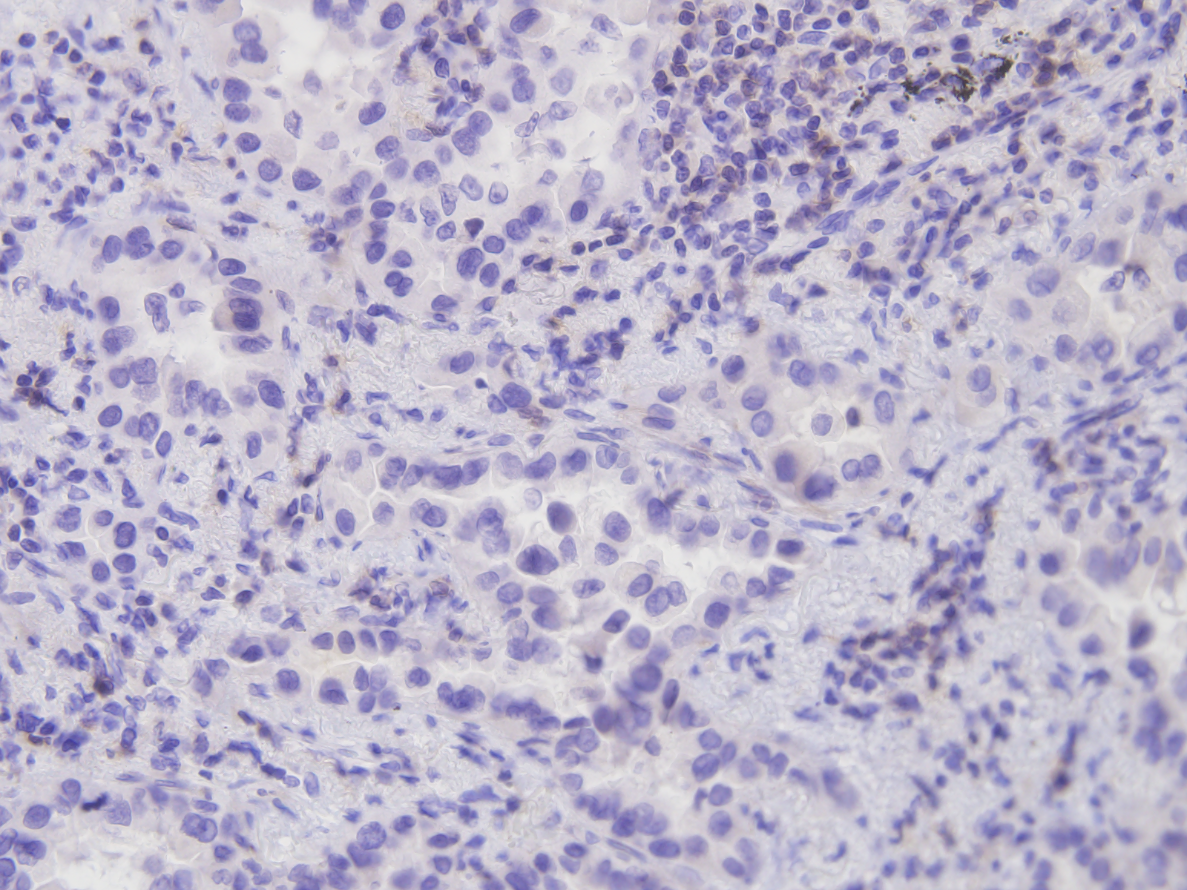

Supplement: S50 File — (ZIP) [file pone.0337223.s051.zip › 511430-400-CA-N/511430-400-CA (3).tif]

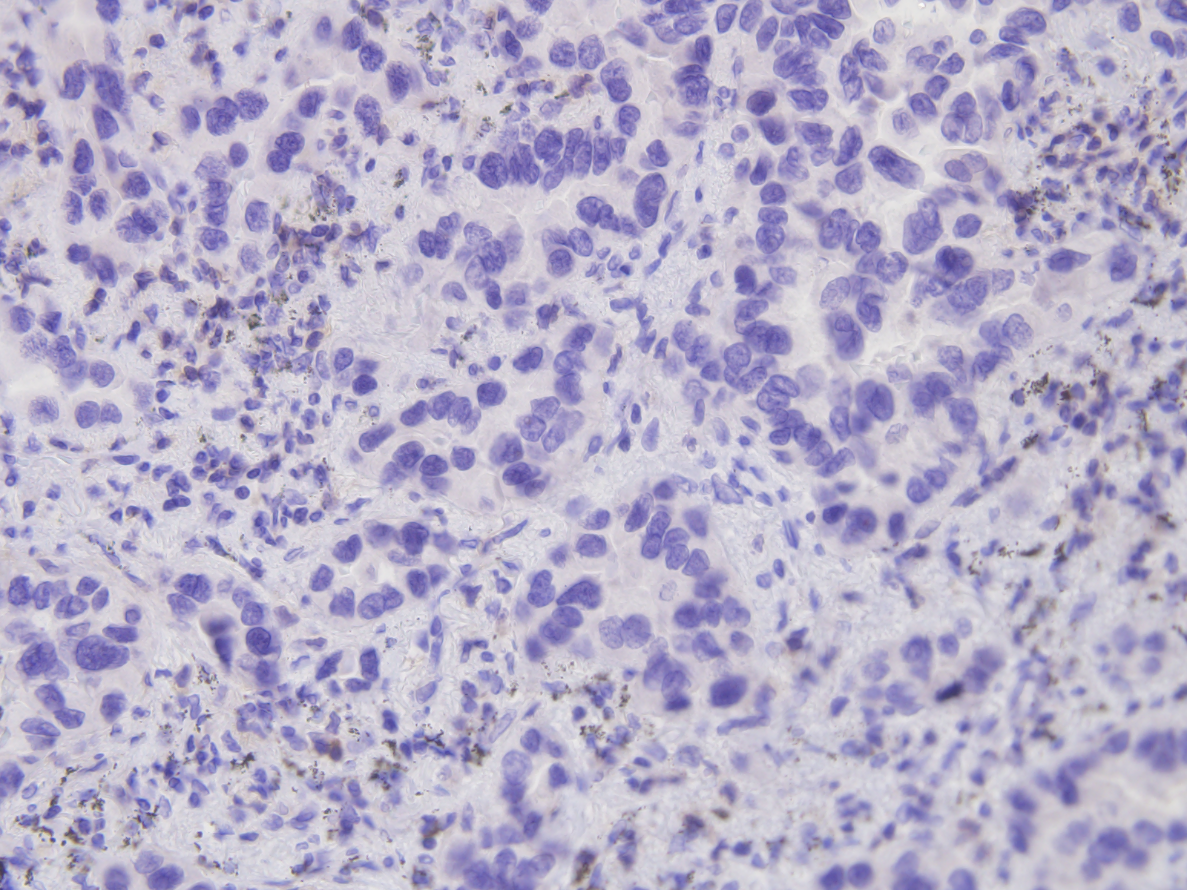

Supplement: S50 File — (ZIP) [file pone.0337223.s051.zip › 511430-400-CA-N/511430-400-CA (4).tif]

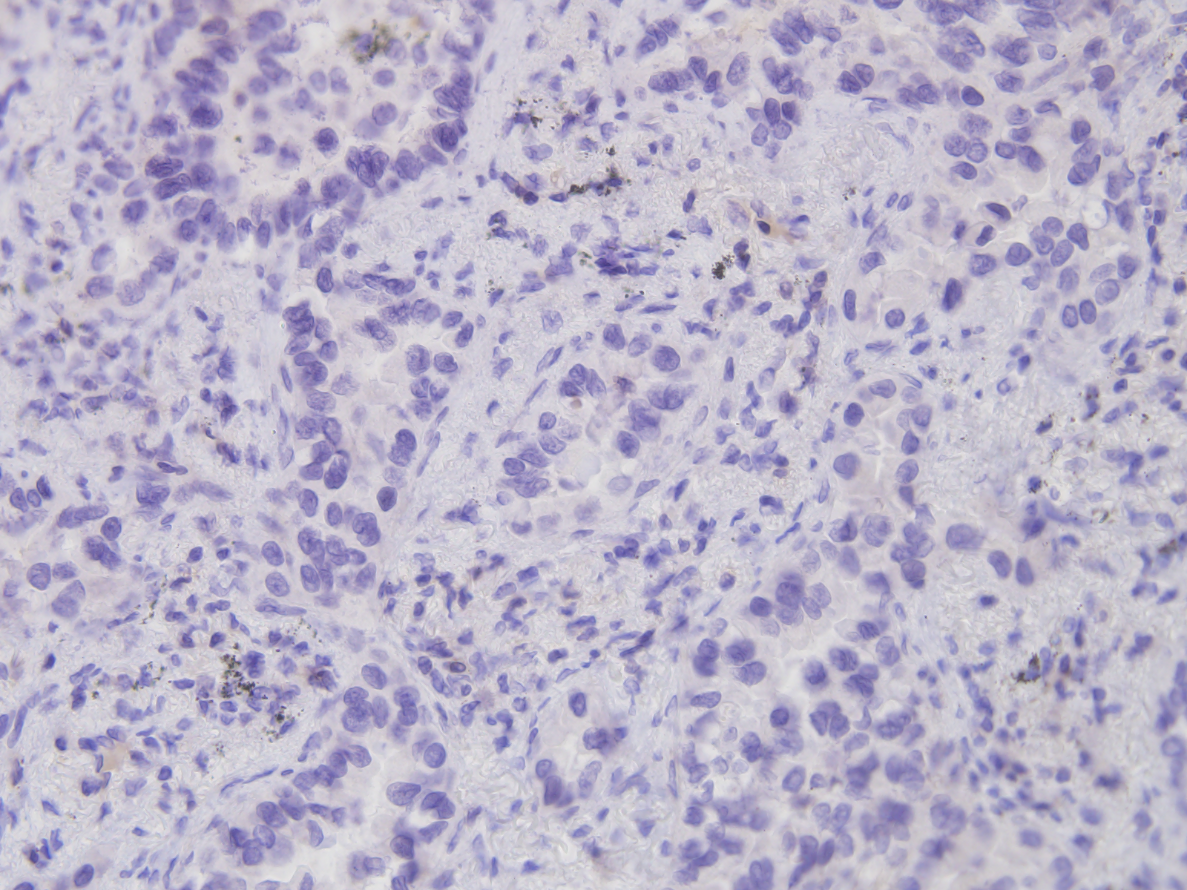

Supplement: S50 File — (ZIP) [file pone.0337223.s051.zip › 511430-400-CA-N/511430-400-CA (5).tif]

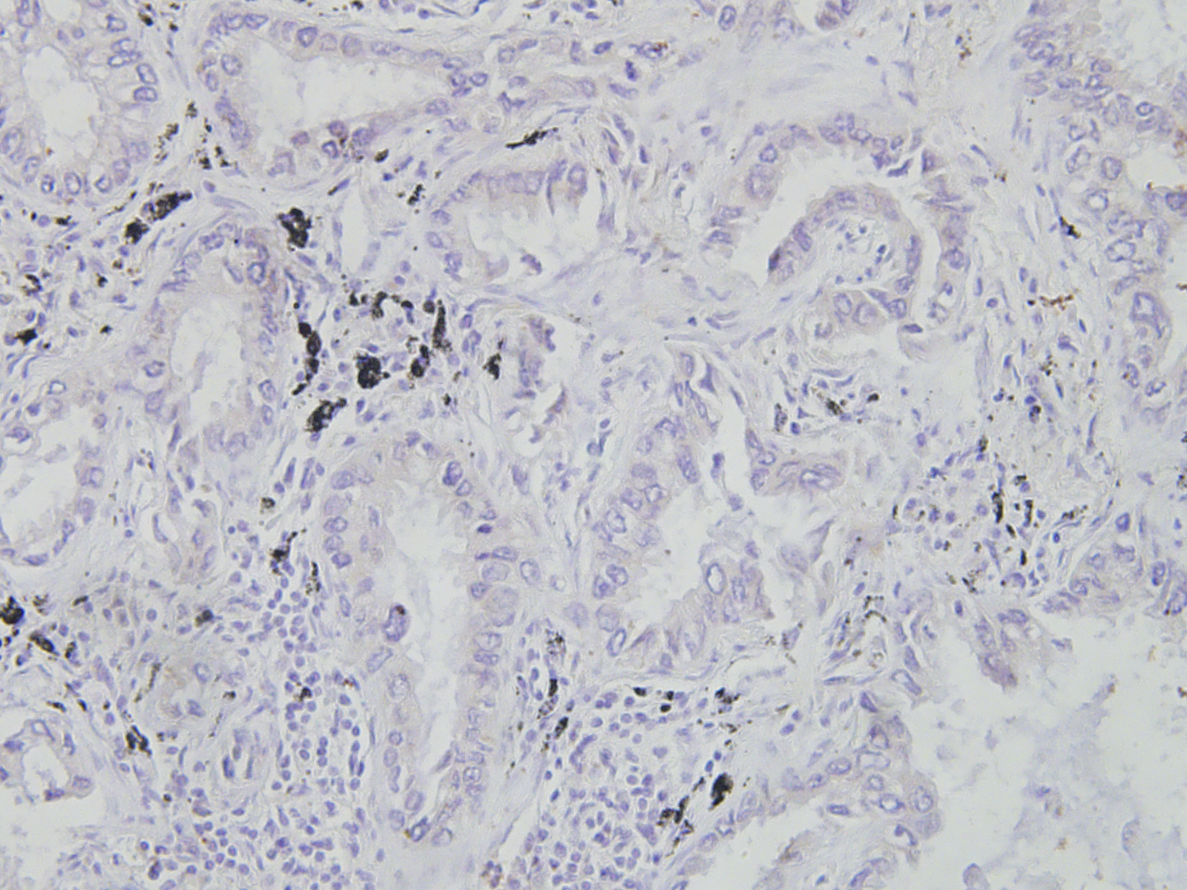

Supplement: S51 File — (ZIP) [file pone.0337223.s052.zip › 512223-400X-N-CA/512223-CA (1).tif]

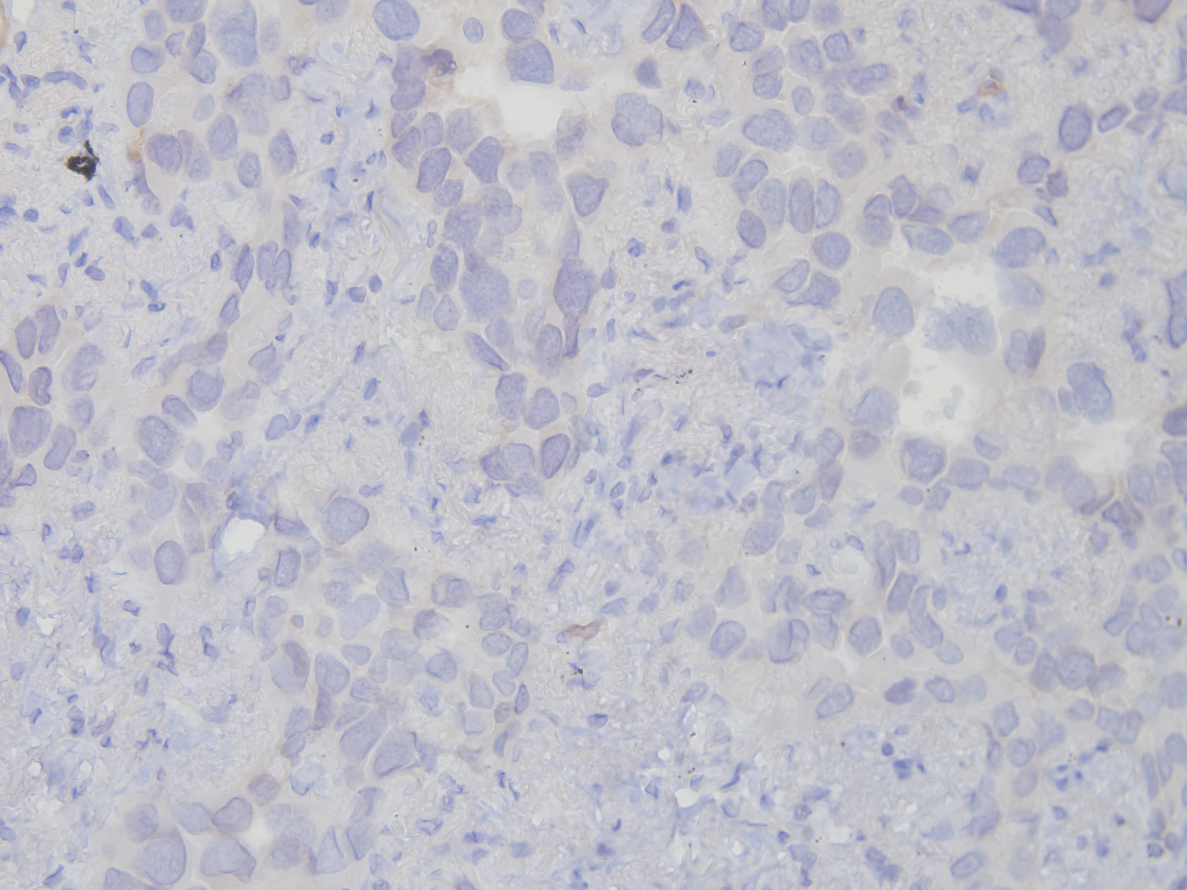

Supplement: S51 File — (ZIP) [file pone.0337223.s052.zip › 512223-400X-N-CA/512223-CA (2).tif]

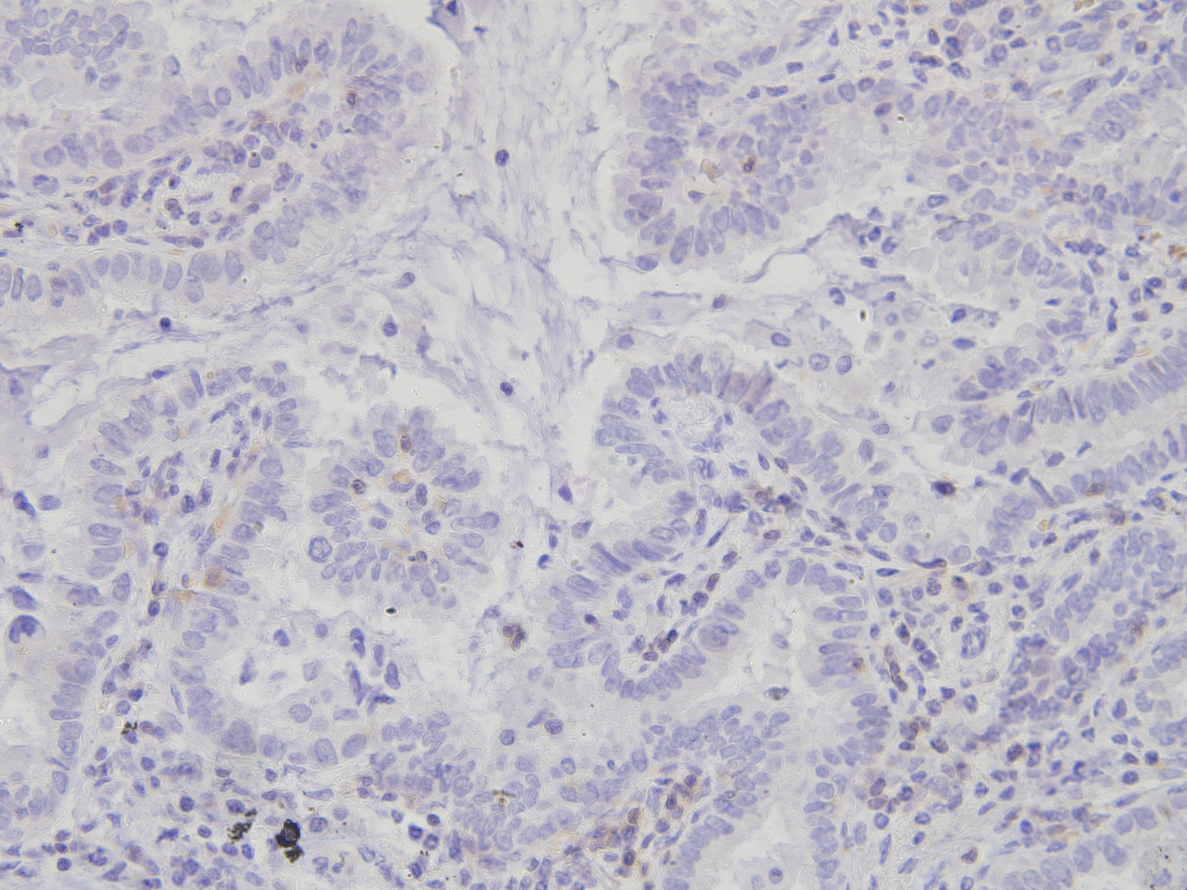

Supplement: S51 File — (ZIP) [file pone.0337223.s052.zip › 512223-400X-N-CA/512223-CA (3).tif]

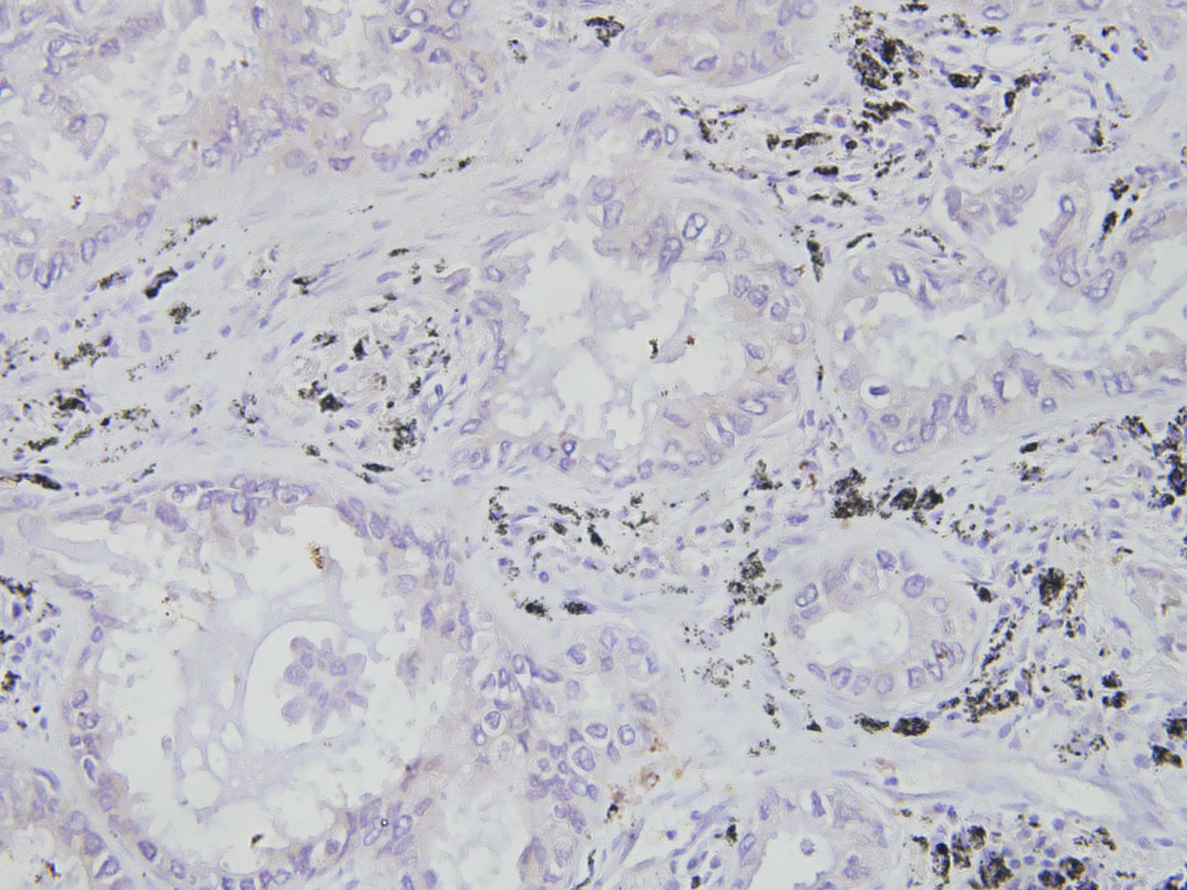

Supplement: S51 File — (ZIP) [file pone.0337223.s052.zip › 512223-400X-N-CA/512223-CA (4).tif]

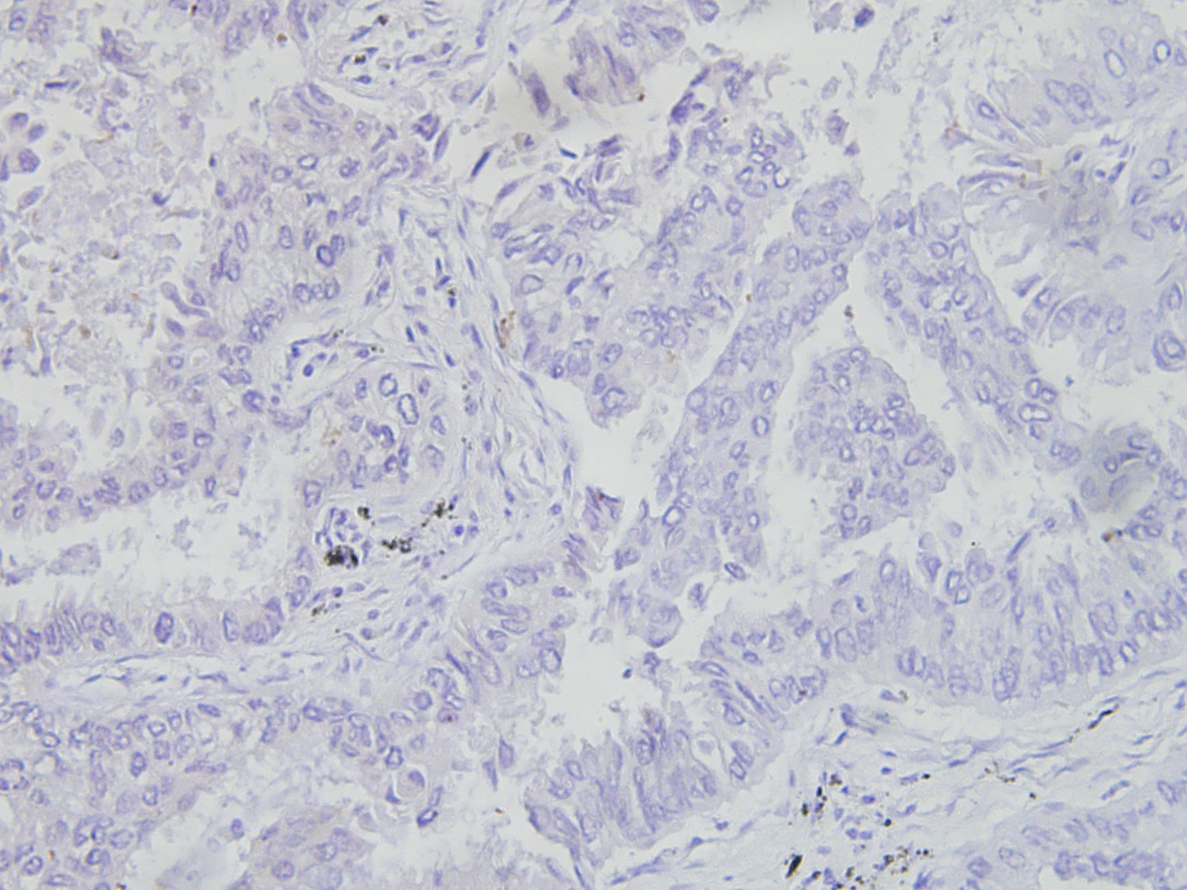

Supplement: S51 File — (ZIP) [file pone.0337223.s052.zip › 512223-400X-N-CA/512223-CA (5).tif]

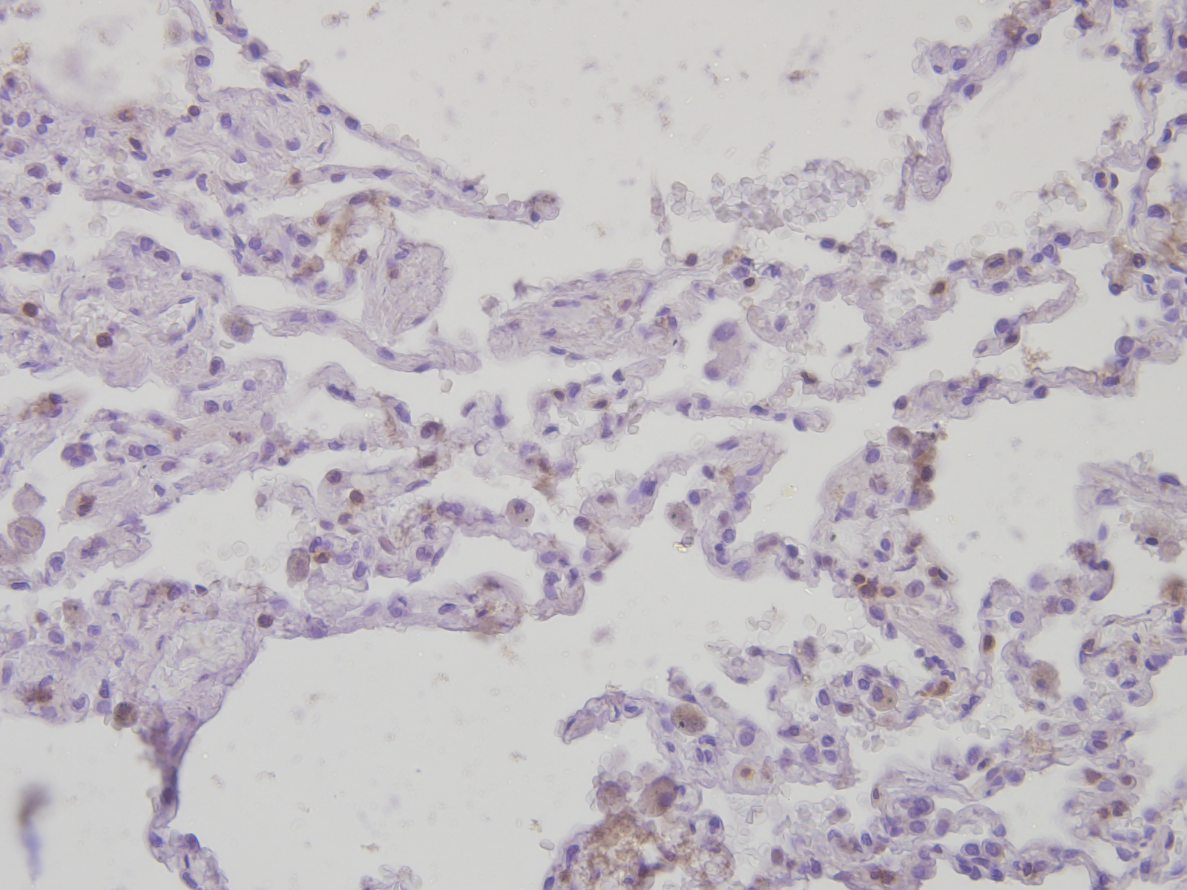

Supplement: S51 File — (ZIP) [file pone.0337223.s052.zip › 512223-400X-N-CA/512223-n (1).tif]

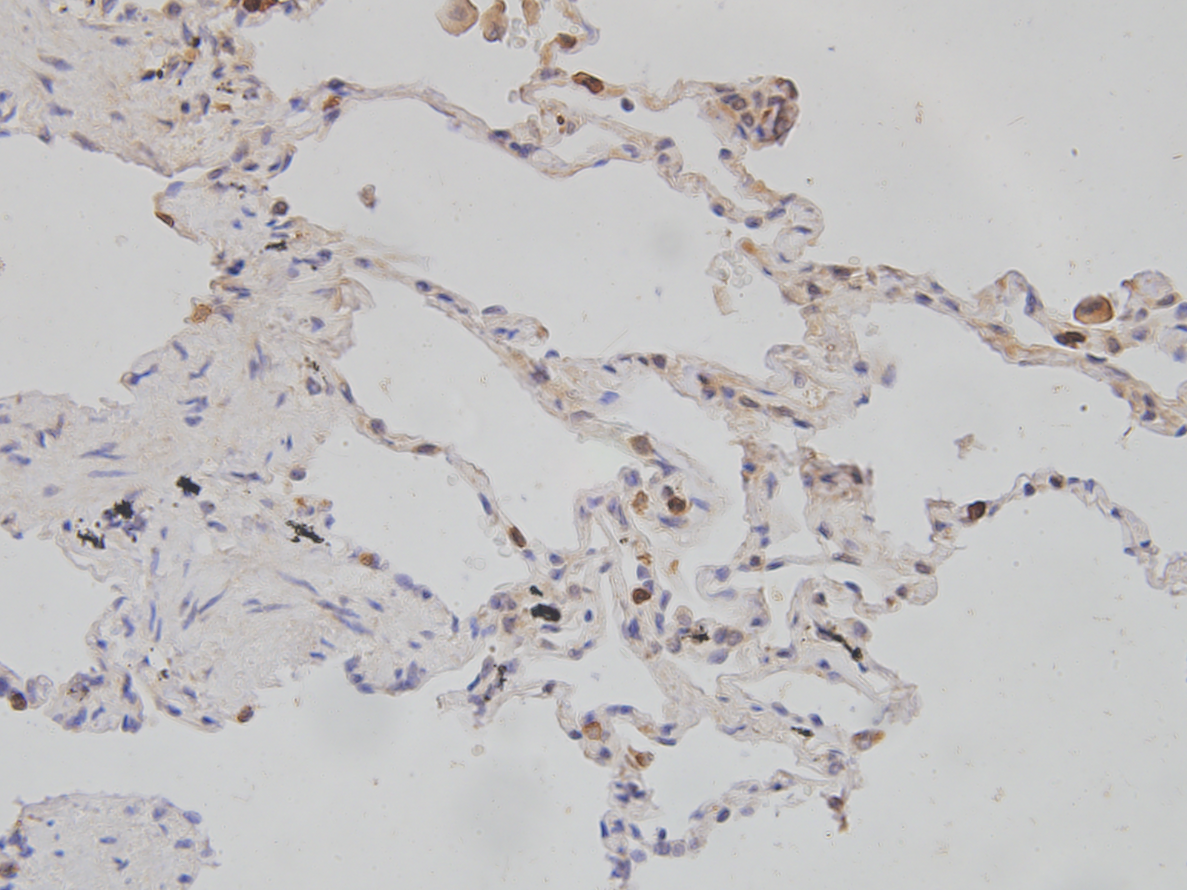

Supplement: S51 File — (ZIP) [file pone.0337223.s052.zip › 512223-400X-N-CA/512223-n (2).tif]

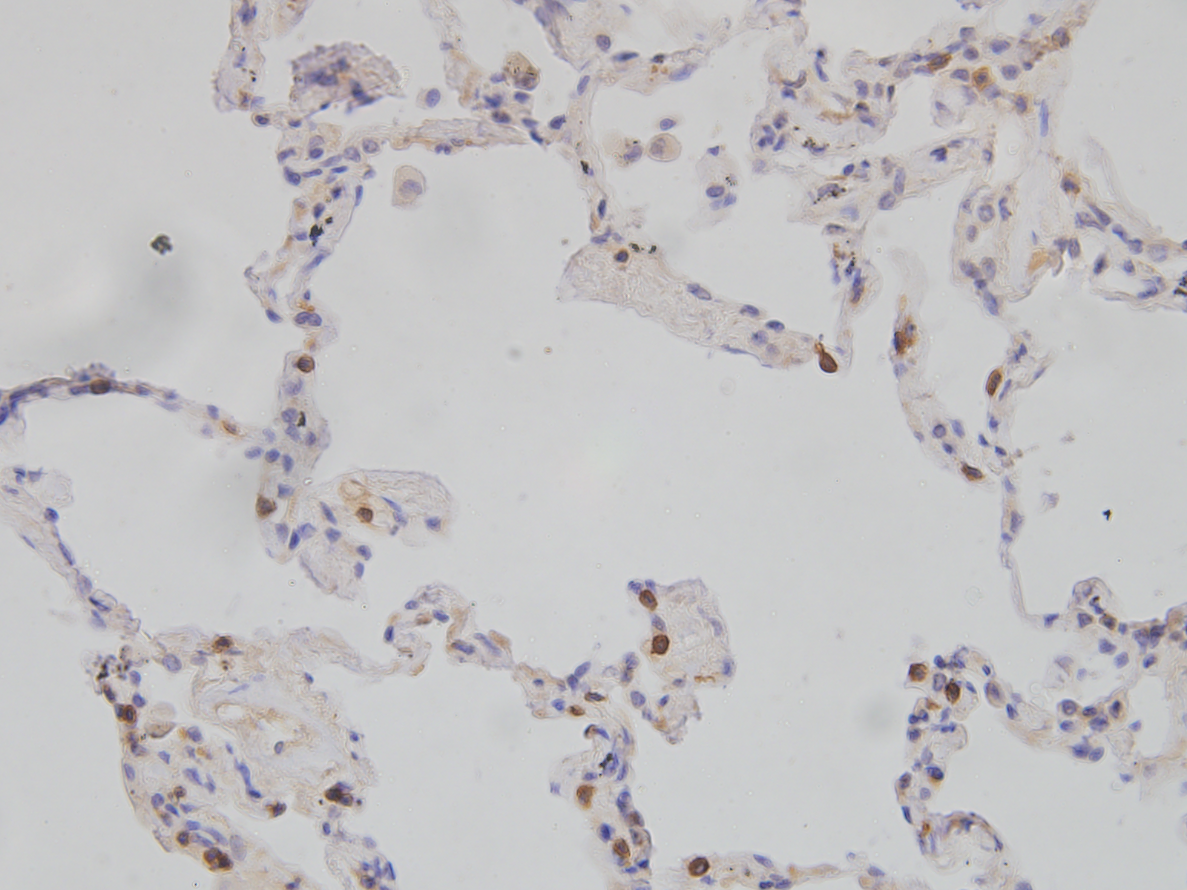

Supplement: S51 File — (ZIP) [file pone.0337223.s052.zip › 512223-400X-N-CA/512223-n (3).tif]

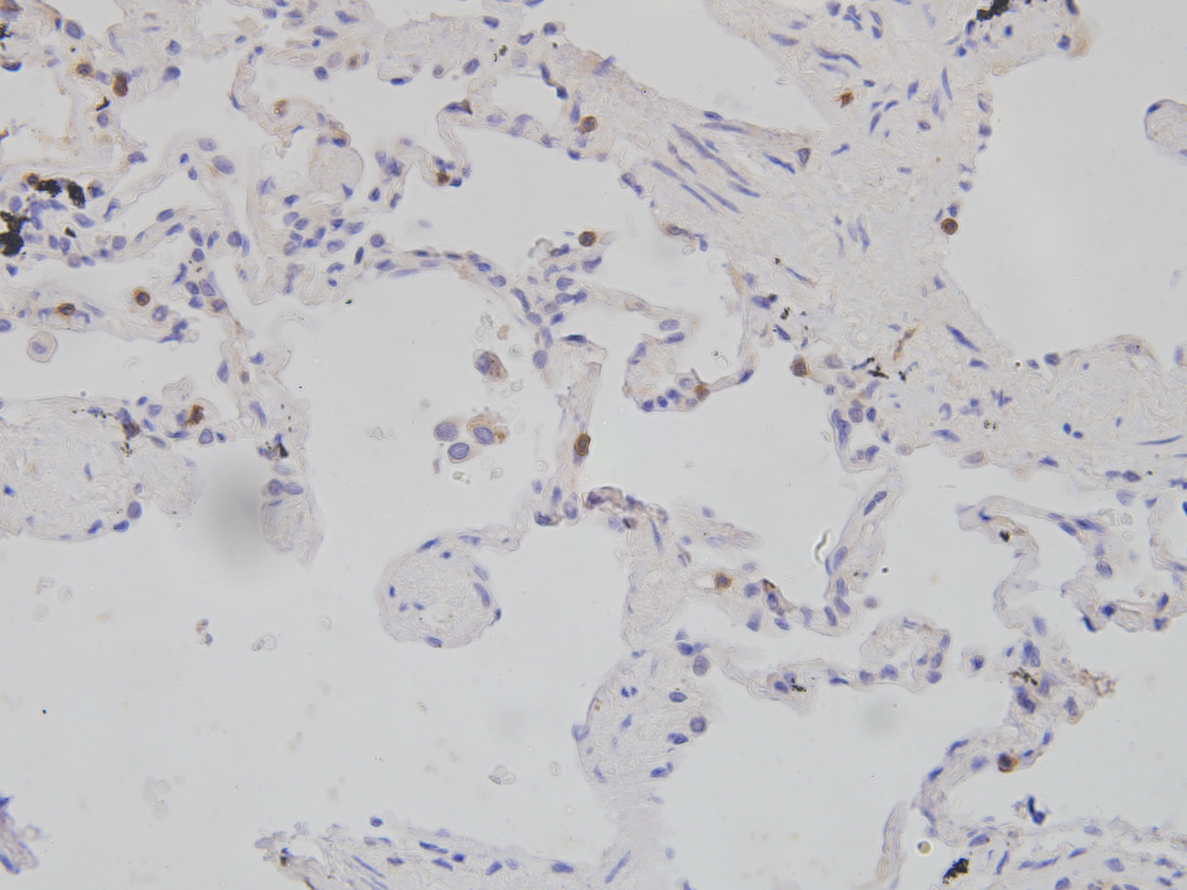

Supplement: S51 File — (ZIP) [file pone.0337223.s052.zip › 512223-400X-N-CA/512223-n (4).tif]

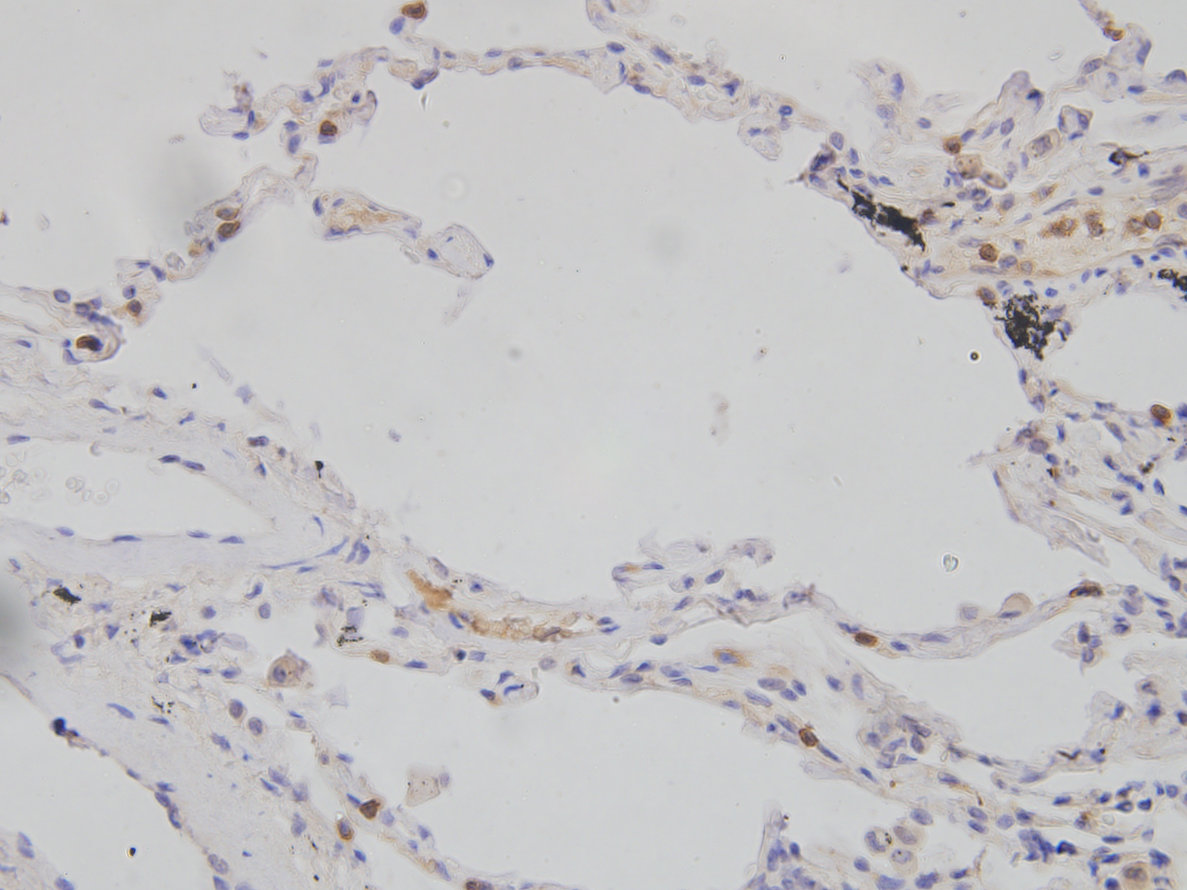

Supplement: S51 File — (ZIP) [file pone.0337223.s052.zip › 512223-400X-N-CA/512223-n (5).tif]

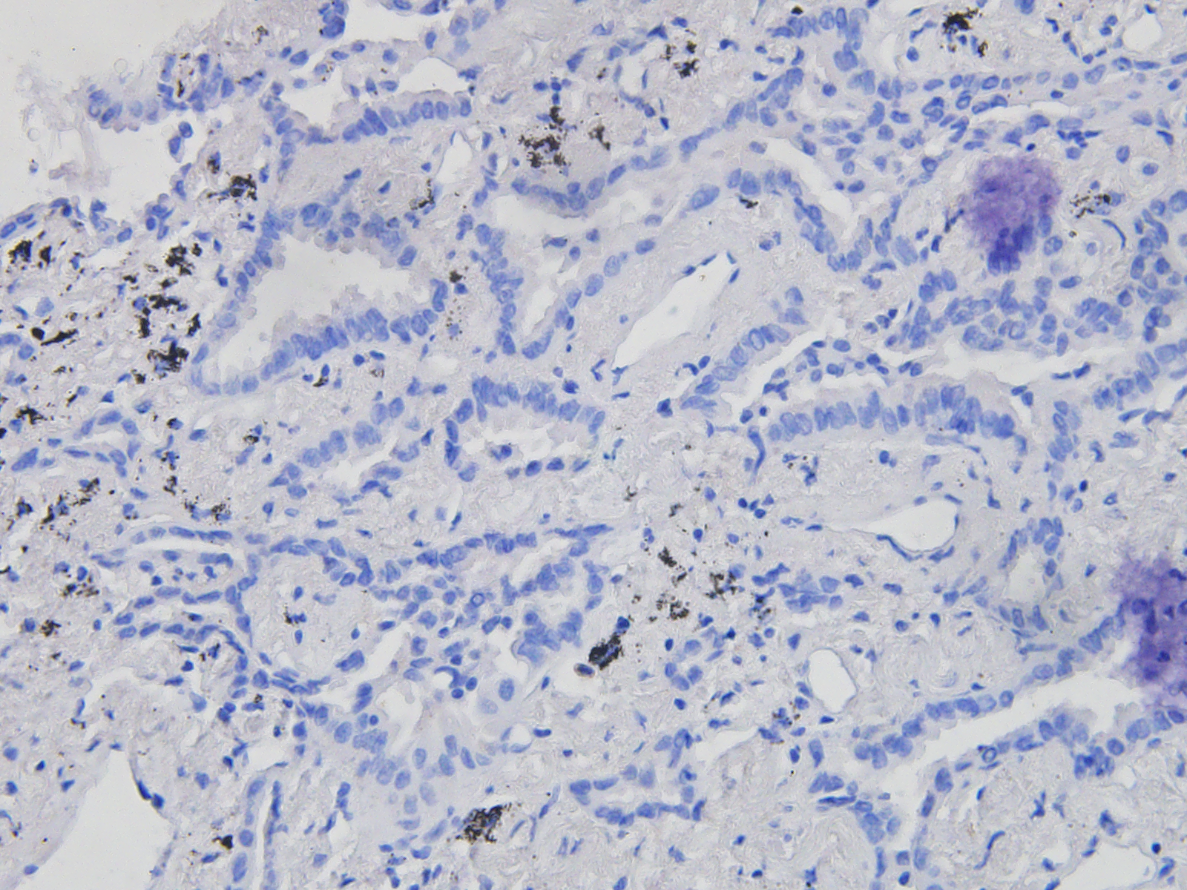

Supplement: S52 File — (ZIP) [file pone.0337223.s053.zip › 514577/514577-400x-CA (1).tif]

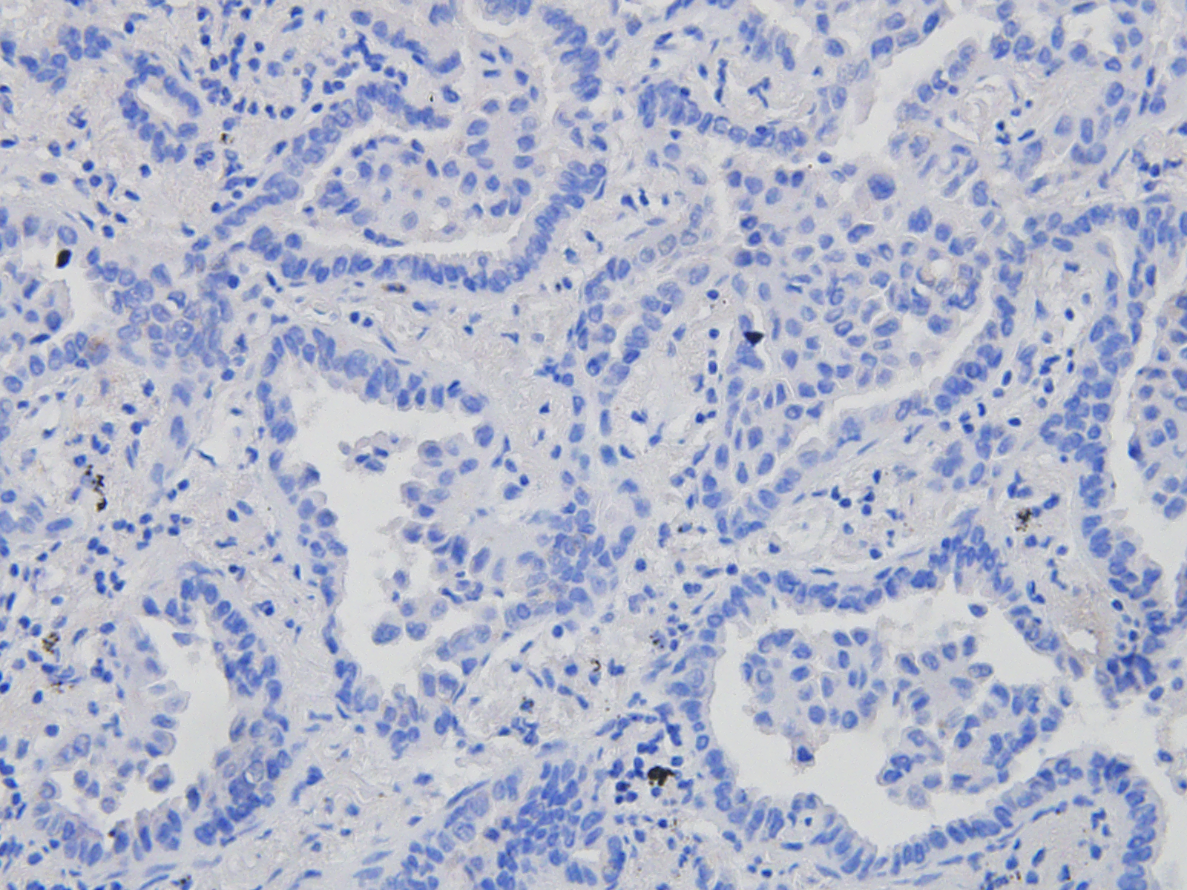

Supplement: S52 File — (ZIP) [file pone.0337223.s053.zip › 514577/514577-400x-CA (2).tif]

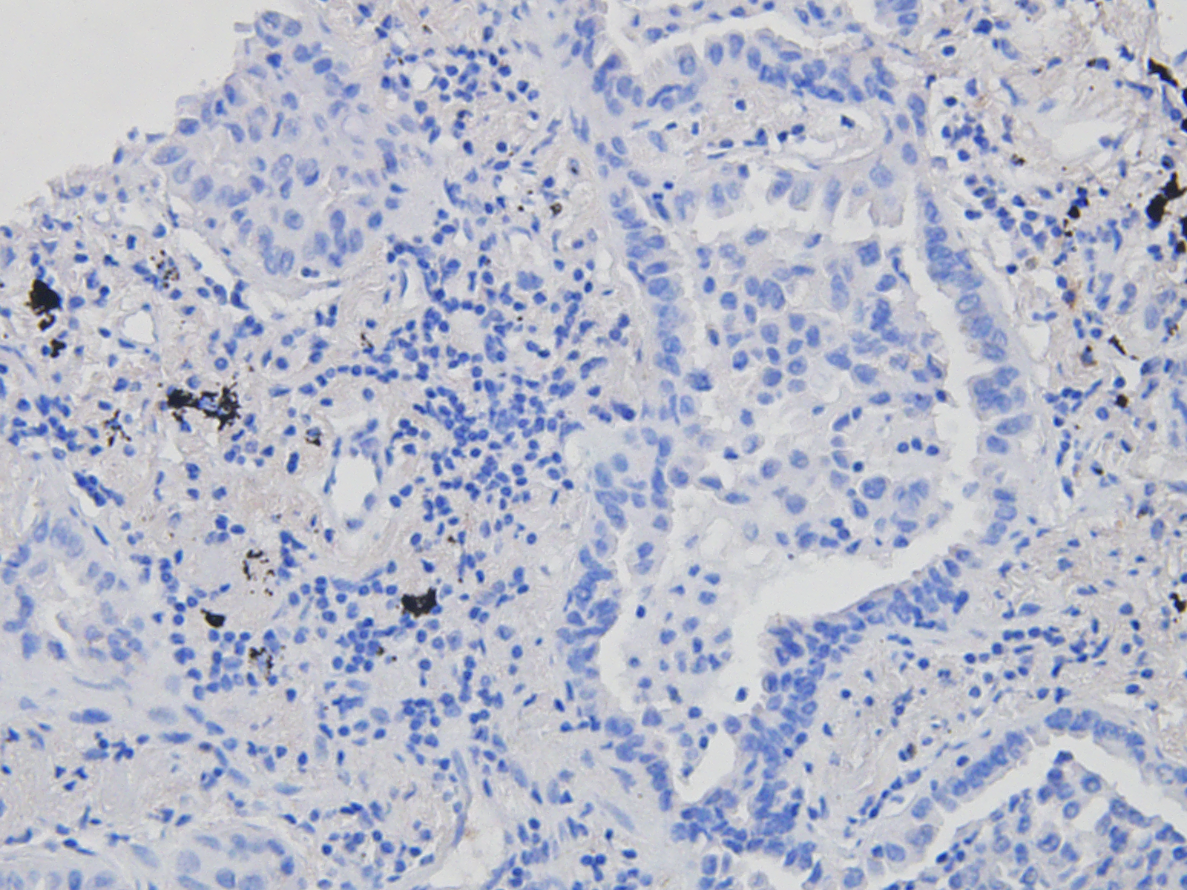

Supplement: S52 File — (ZIP) [file pone.0337223.s053.zip › 514577/514577-400x-CA (3).tif]

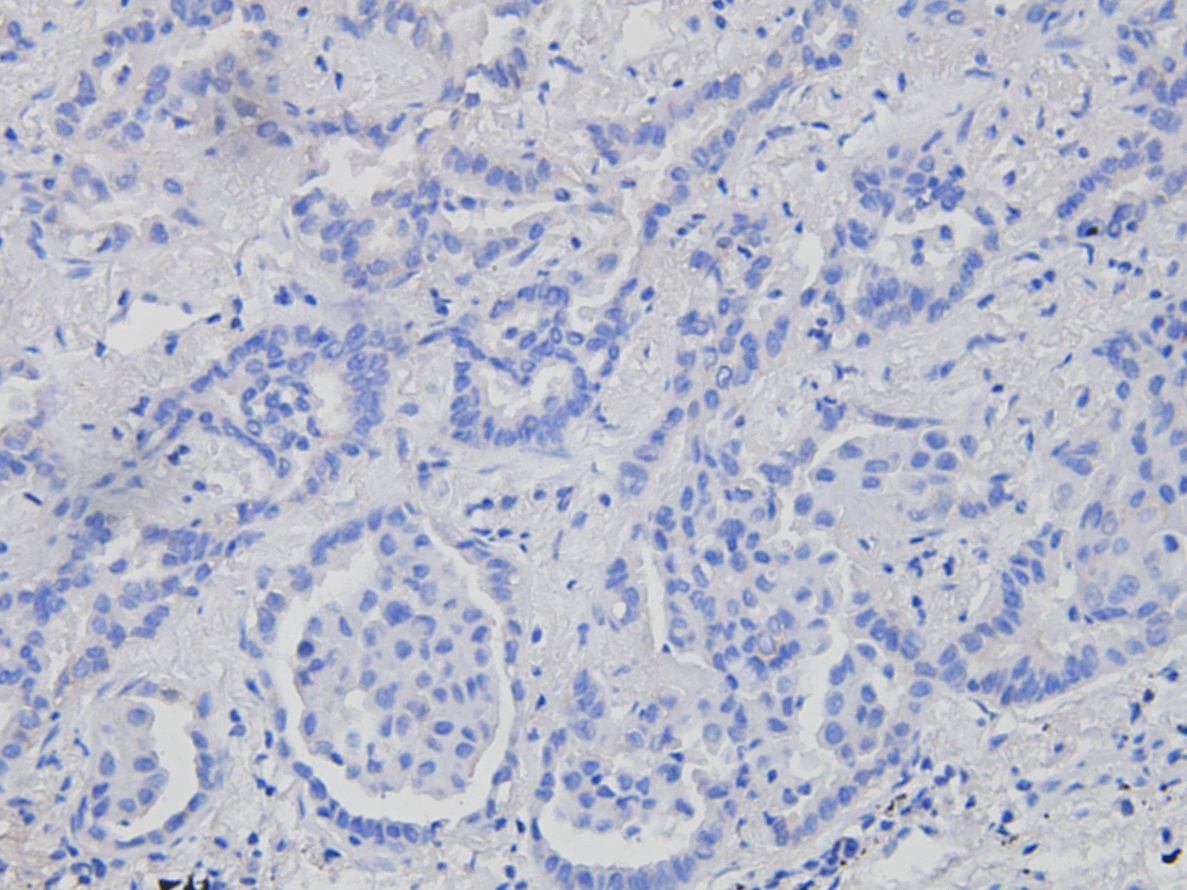

Supplement: S52 File — (ZIP) [file pone.0337223.s053.zip › 514577/514577-400x-CA (4).tif]

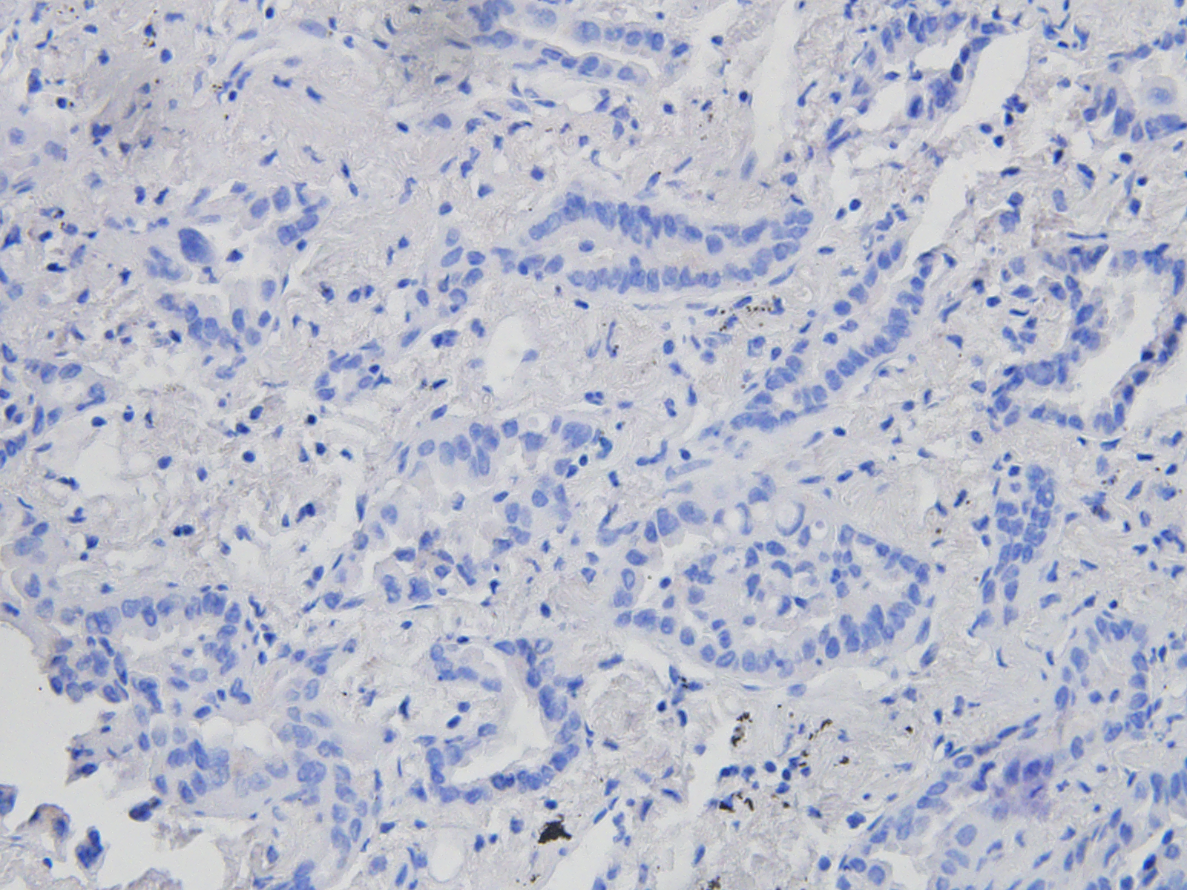

Supplement: S52 File — (ZIP) [file pone.0337223.s053.zip › 514577/514577-400x-CA (5).tif]

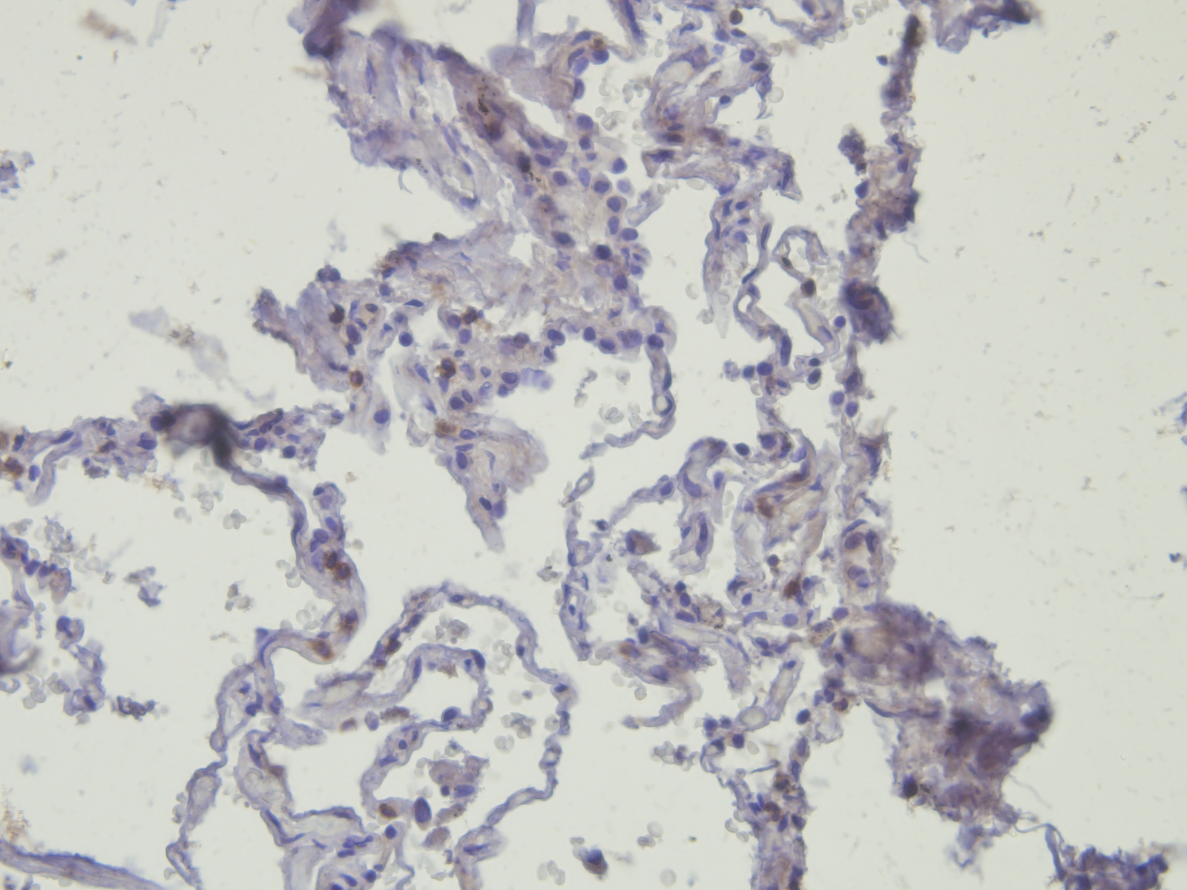

Supplement: S52 File — (ZIP) [file pone.0337223.s053.zip › 514577/514577-N (1).tif]

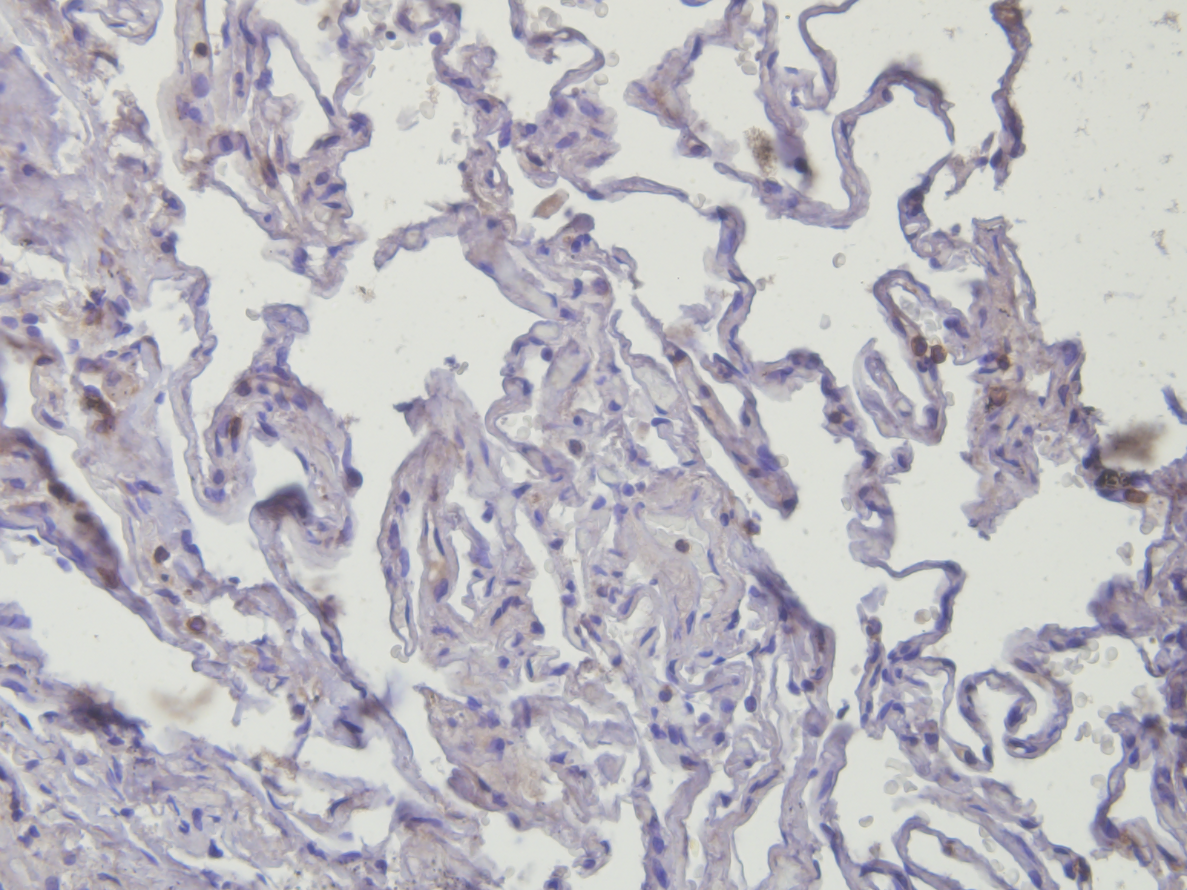

Supplement: S52 File — (ZIP) [file pone.0337223.s053.zip › 514577/514577-N (2).tif]

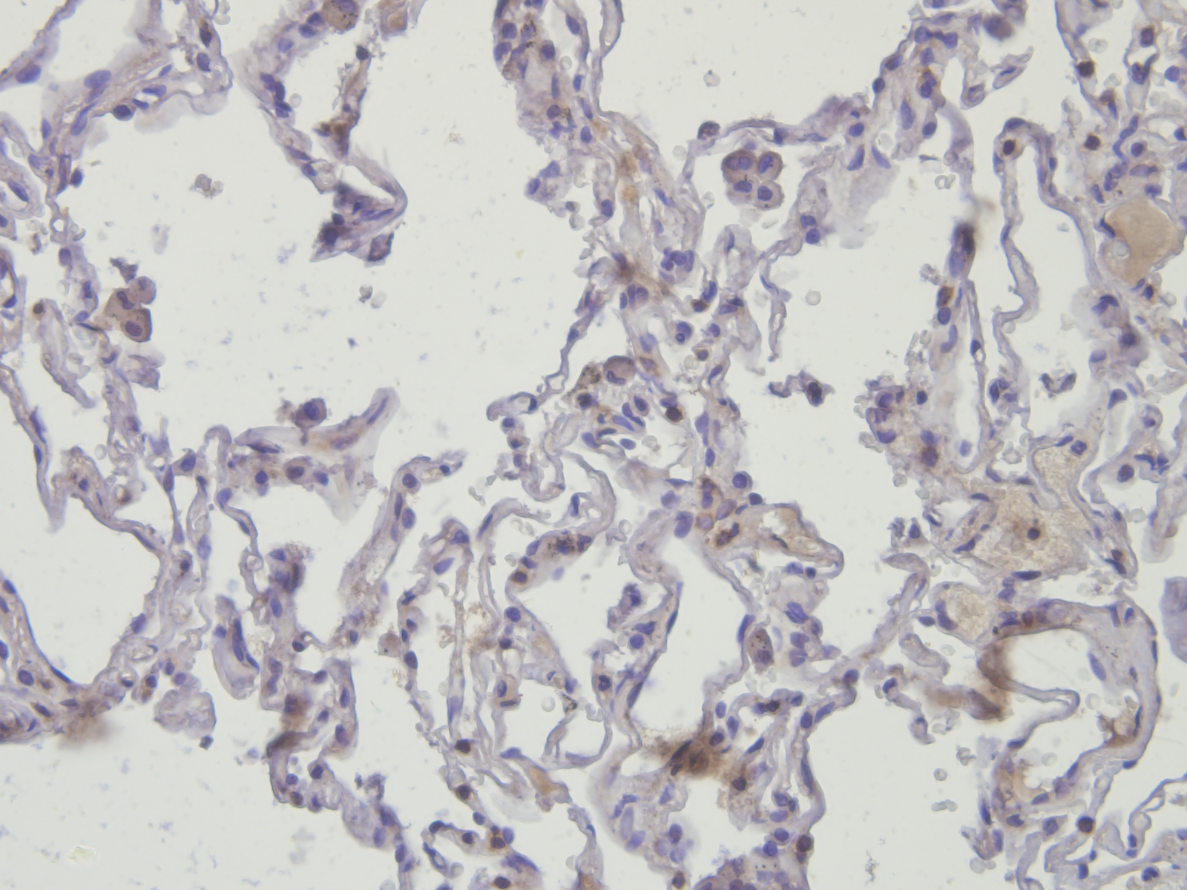

Supplement: S52 File — (ZIP) [file pone.0337223.s053.zip › 514577/514577-N (3).tif]

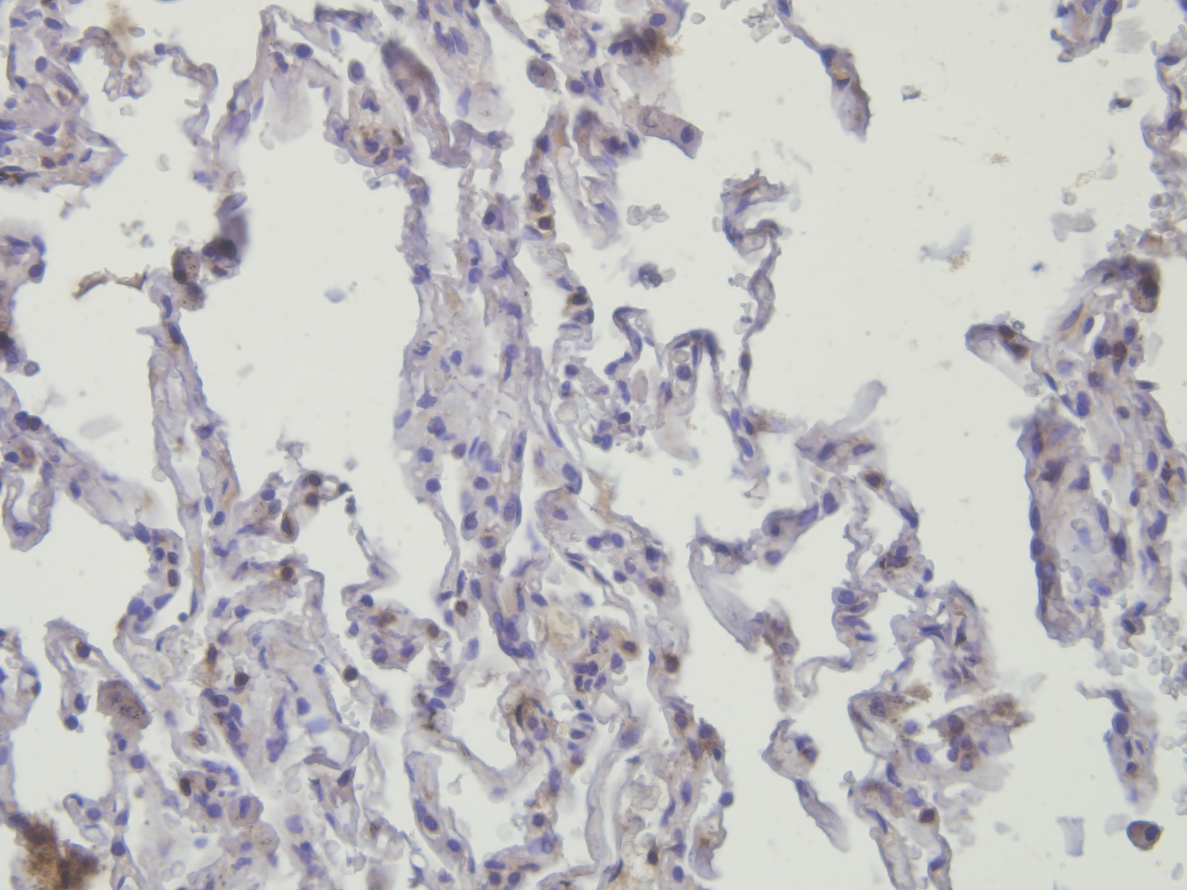

Supplement: S52 File — (ZIP) [file pone.0337223.s053.zip › 514577/514577-N (4).tif]

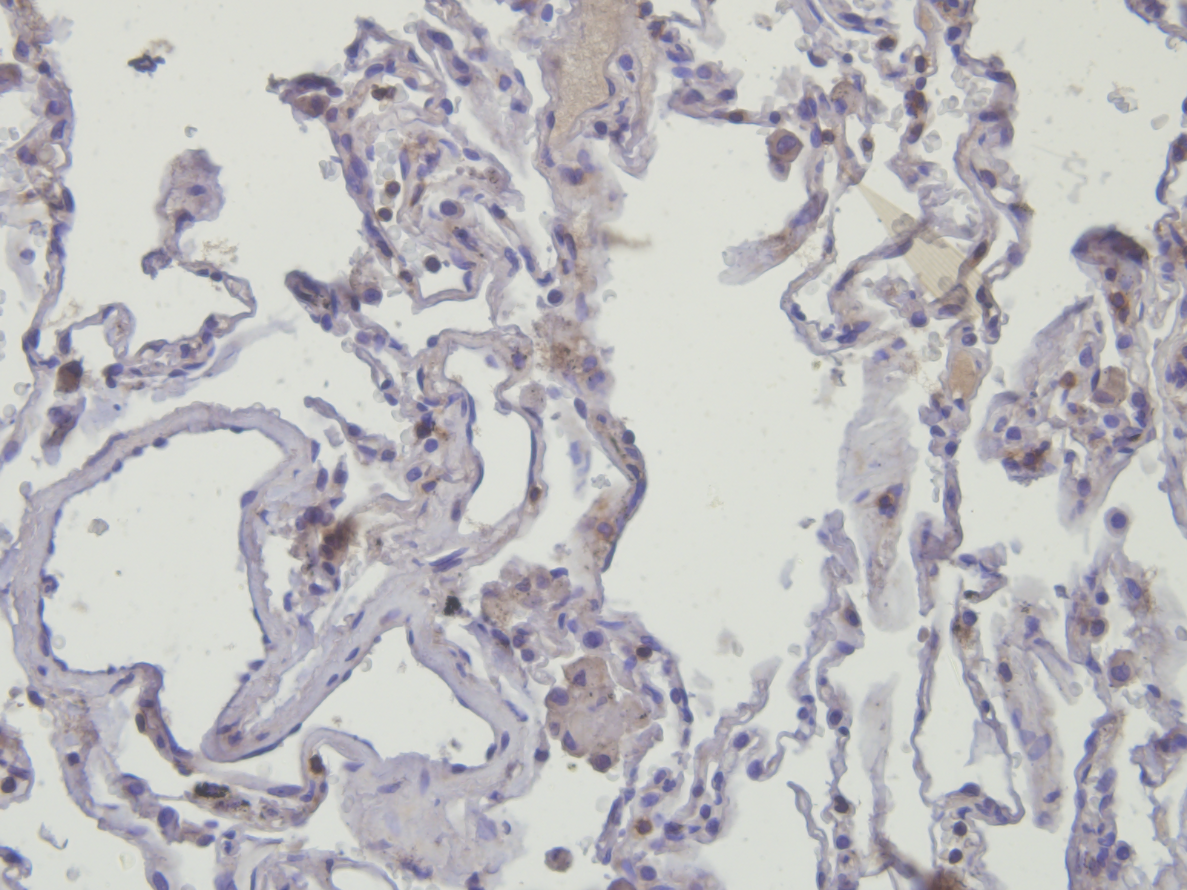

Supplement: S52 File — (ZIP) [file pone.0337223.s053.zip › 514577/514577-N (5).tif]

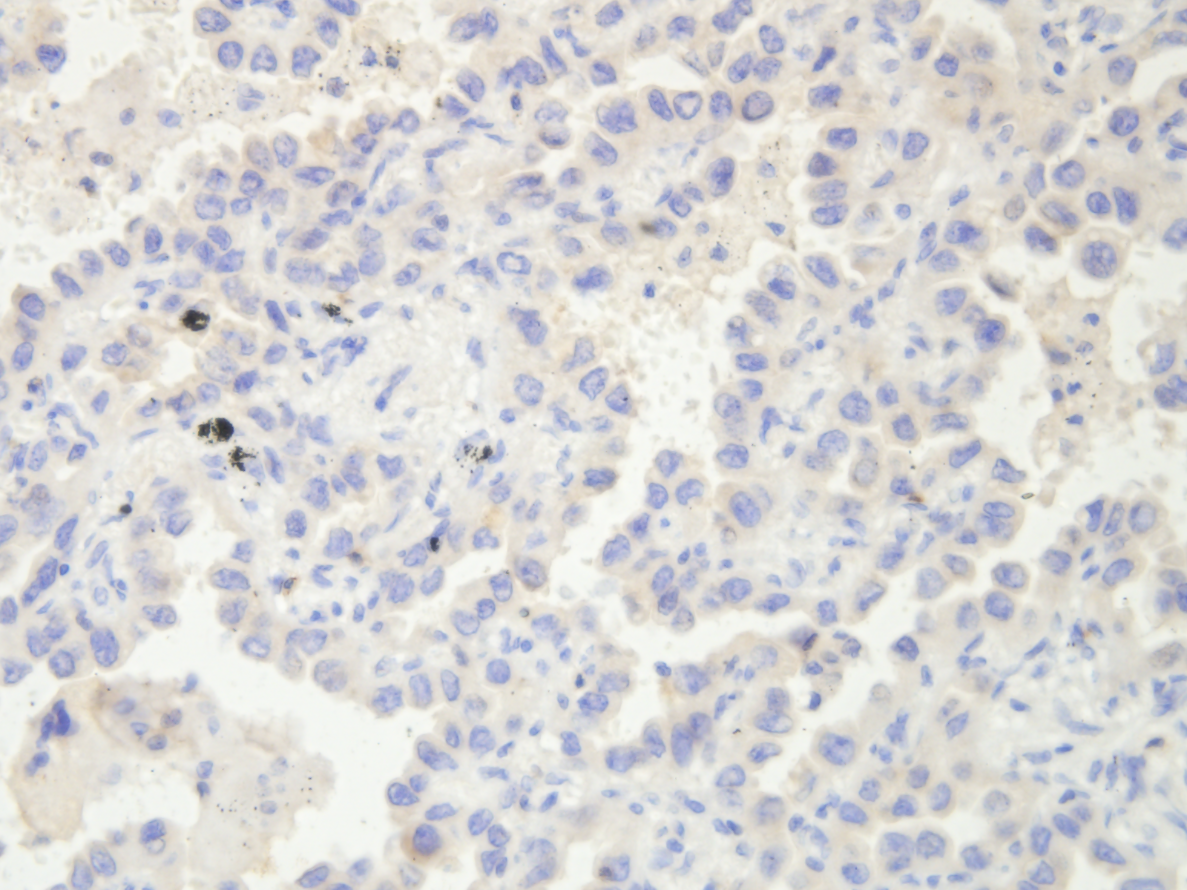

Supplement: S53 File — (ZIP) [file pone.0337223.s054.zip › 515447-400X-CA-N/515447-400X-CA (1).tif]

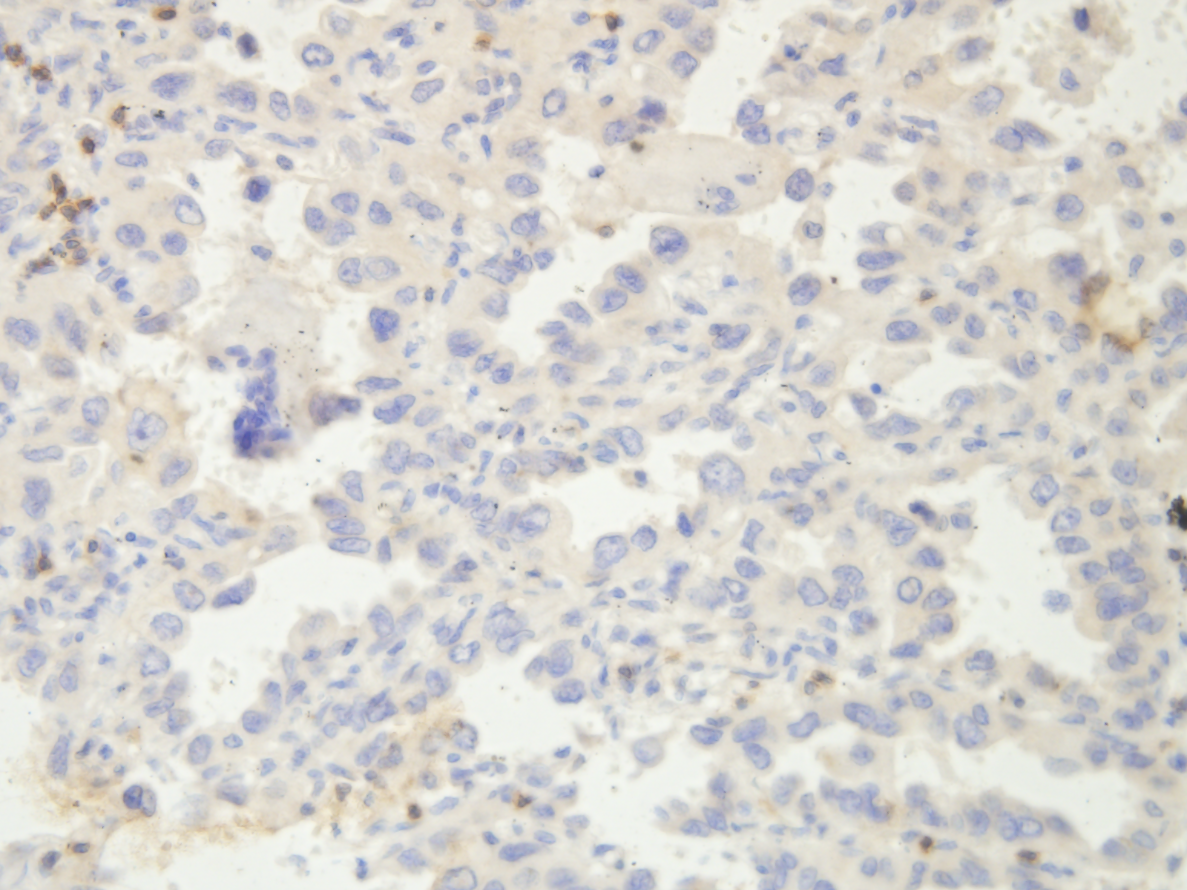

Supplement: S53 File — (ZIP) [file pone.0337223.s054.zip › 515447-400X-CA-N/515447-400X-CA (2).tif]

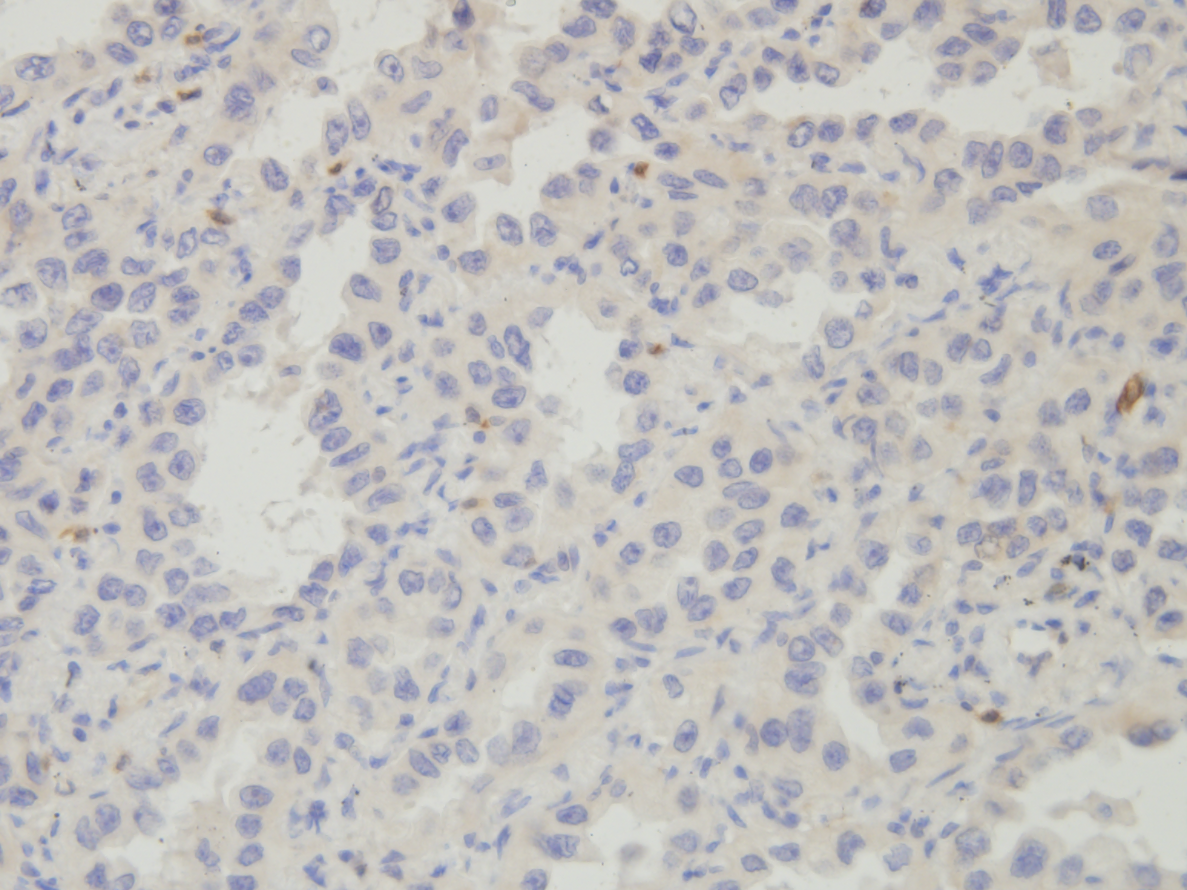

Supplement: S53 File — (ZIP) [file pone.0337223.s054.zip › 515447-400X-CA-N/515447-400X-CA (3).tif]

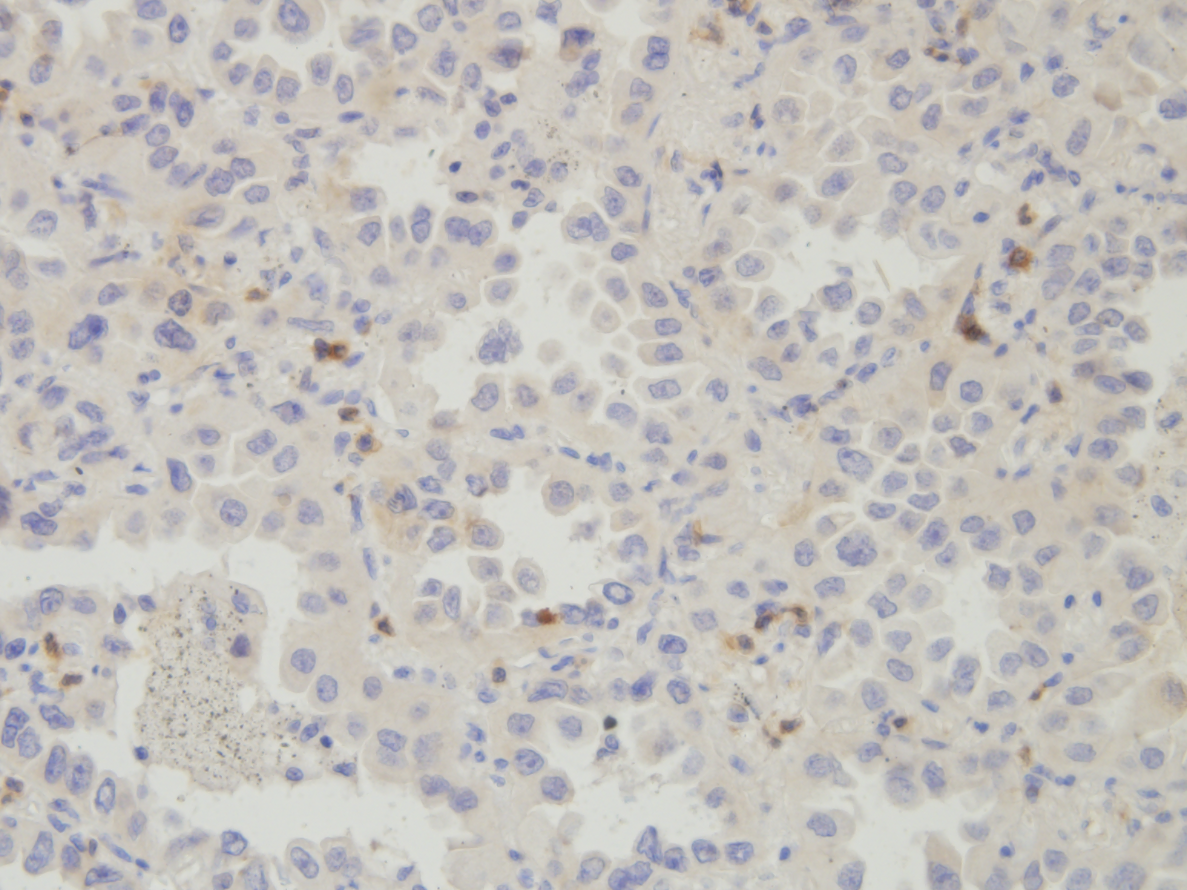

Supplement: S53 File — (ZIP) [file pone.0337223.s054.zip › 515447-400X-CA-N/515447-400X-CA (4).tif]

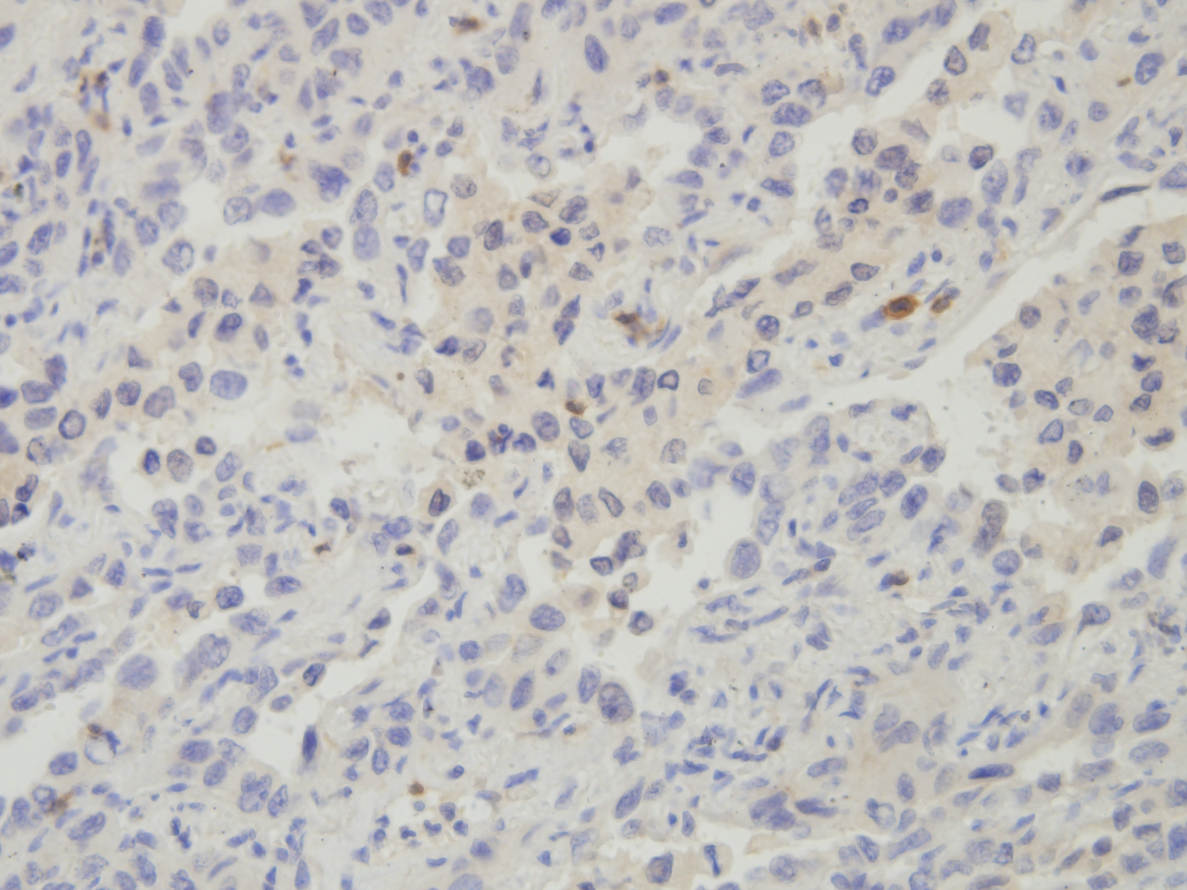

Supplement: S53 File — (ZIP) [file pone.0337223.s054.zip › 515447-400X-CA-N/515447-400X-CA (5).tif]

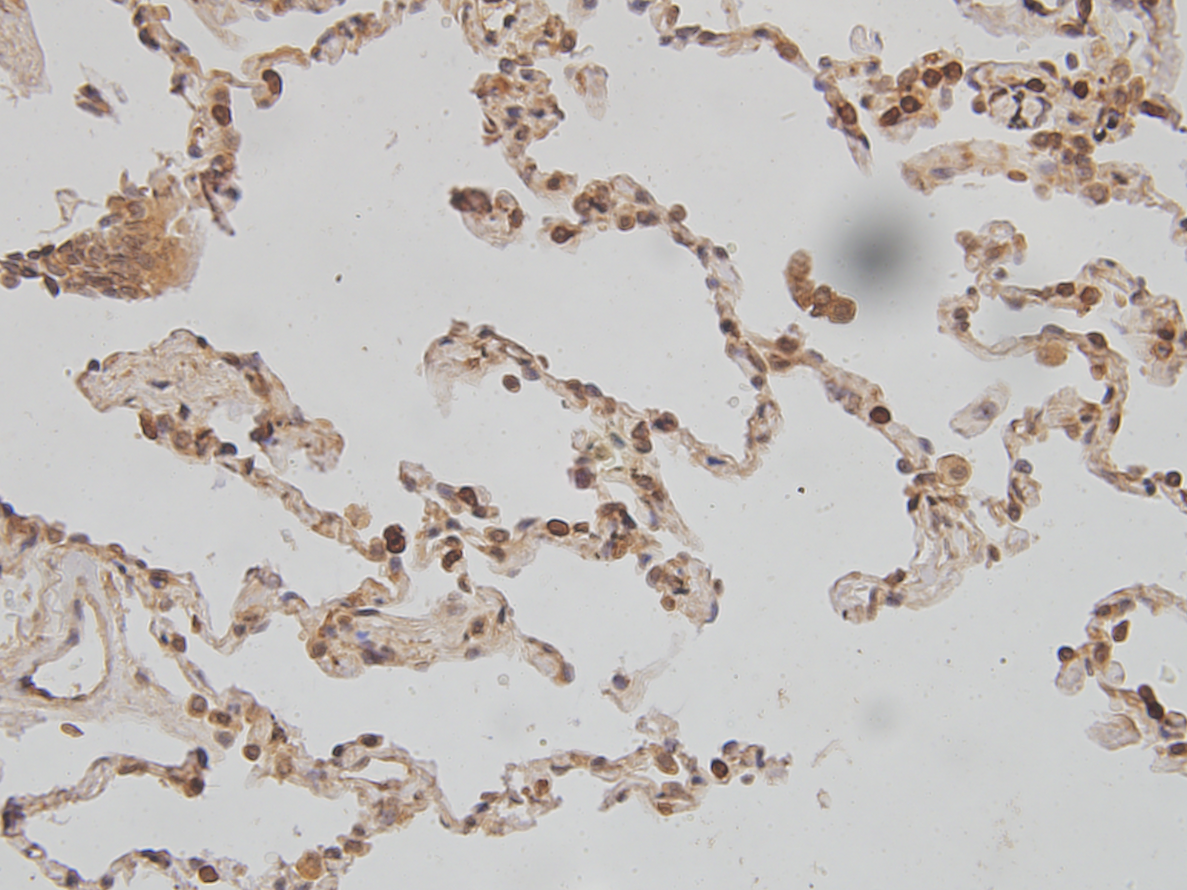

Supplement: S53 File — (ZIP) [file pone.0337223.s054.zip › 515447-400X-CA-N/515447-400X-N (1).tif]

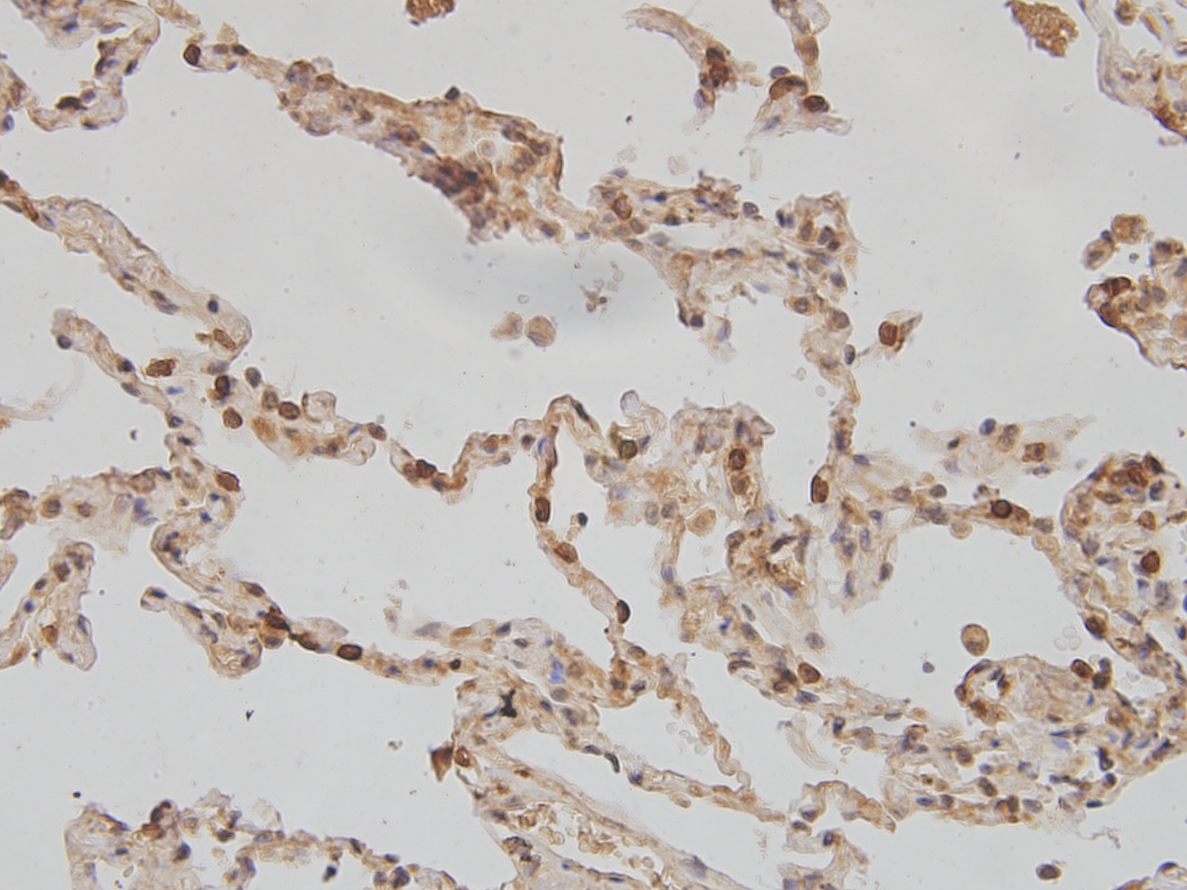

Supplement: S53 File — (ZIP) [file pone.0337223.s054.zip › 515447-400X-CA-N/515447-400X-N (2).tif]

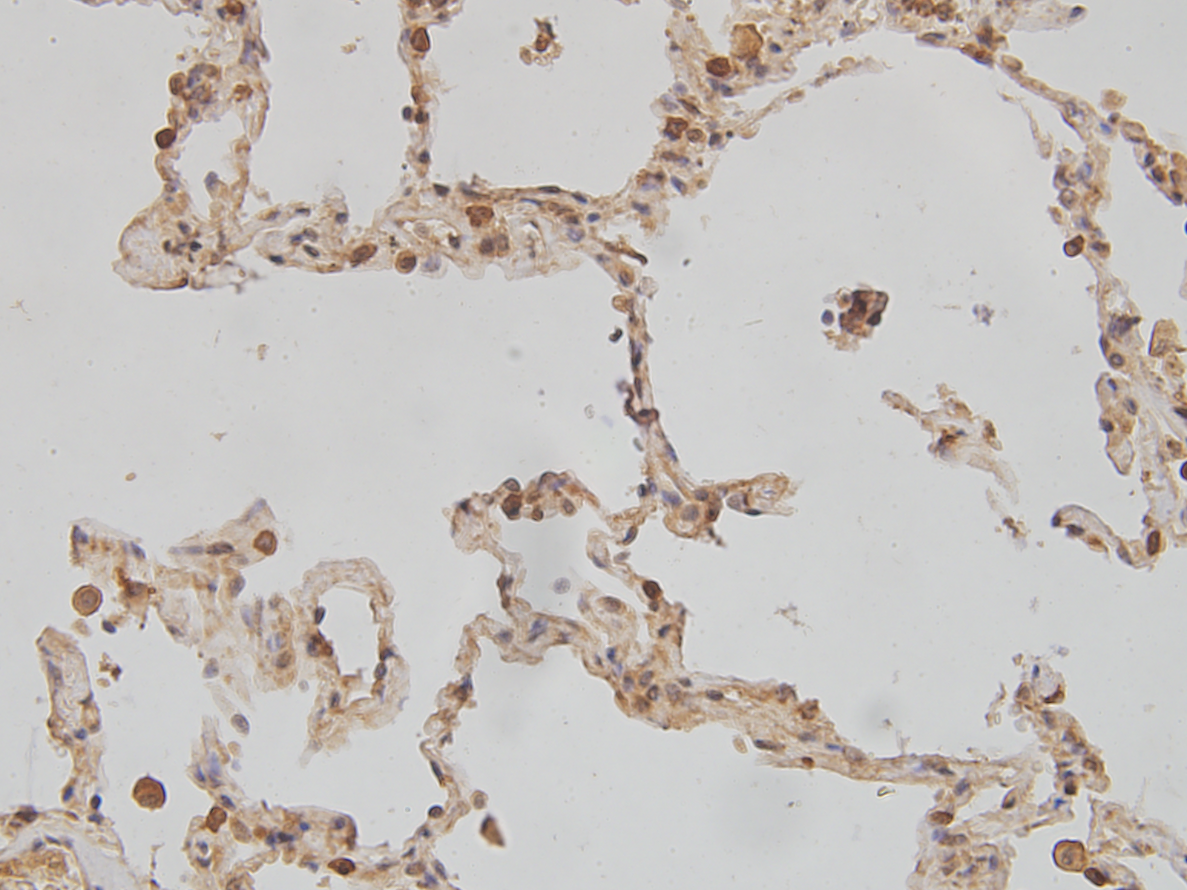

Supplement: S53 File — (ZIP) [file pone.0337223.s054.zip › 515447-400X-CA-N/515447-400X-N (3).tif]

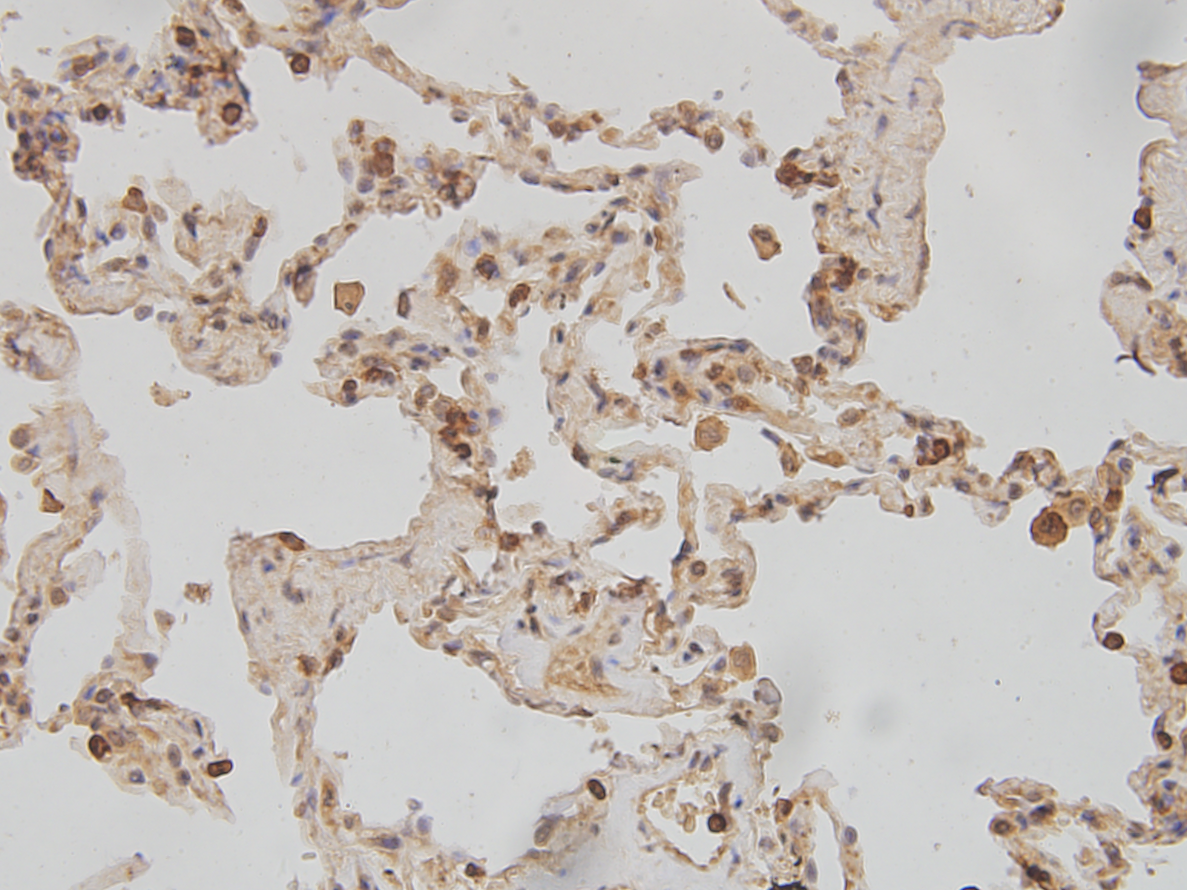

Supplement: S53 File — (ZIP) [file pone.0337223.s054.zip › 515447-400X-CA-N/515447-400X-N (4).tif]

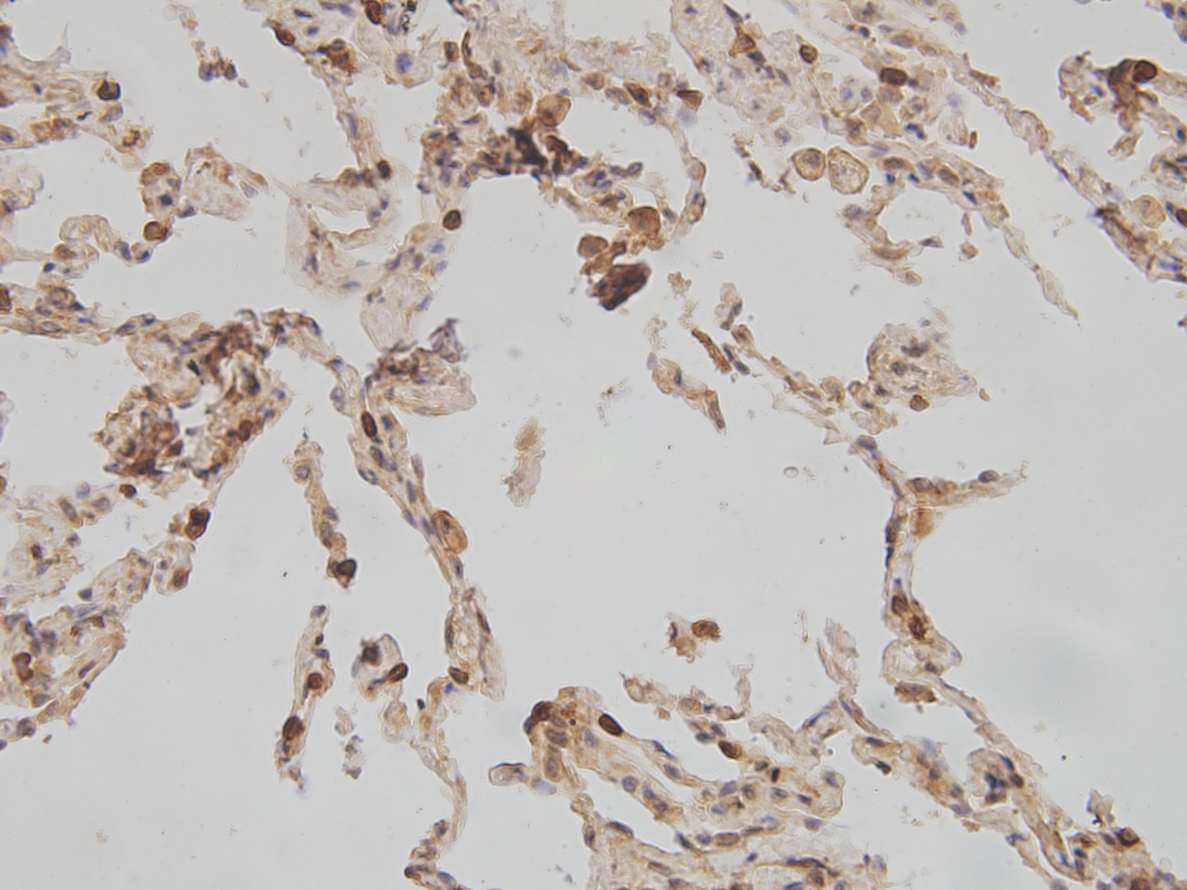

Supplement: S53 File — (ZIP) [file pone.0337223.s054.zip › 515447-400X-CA-N/515447-400X-N (5).tif]

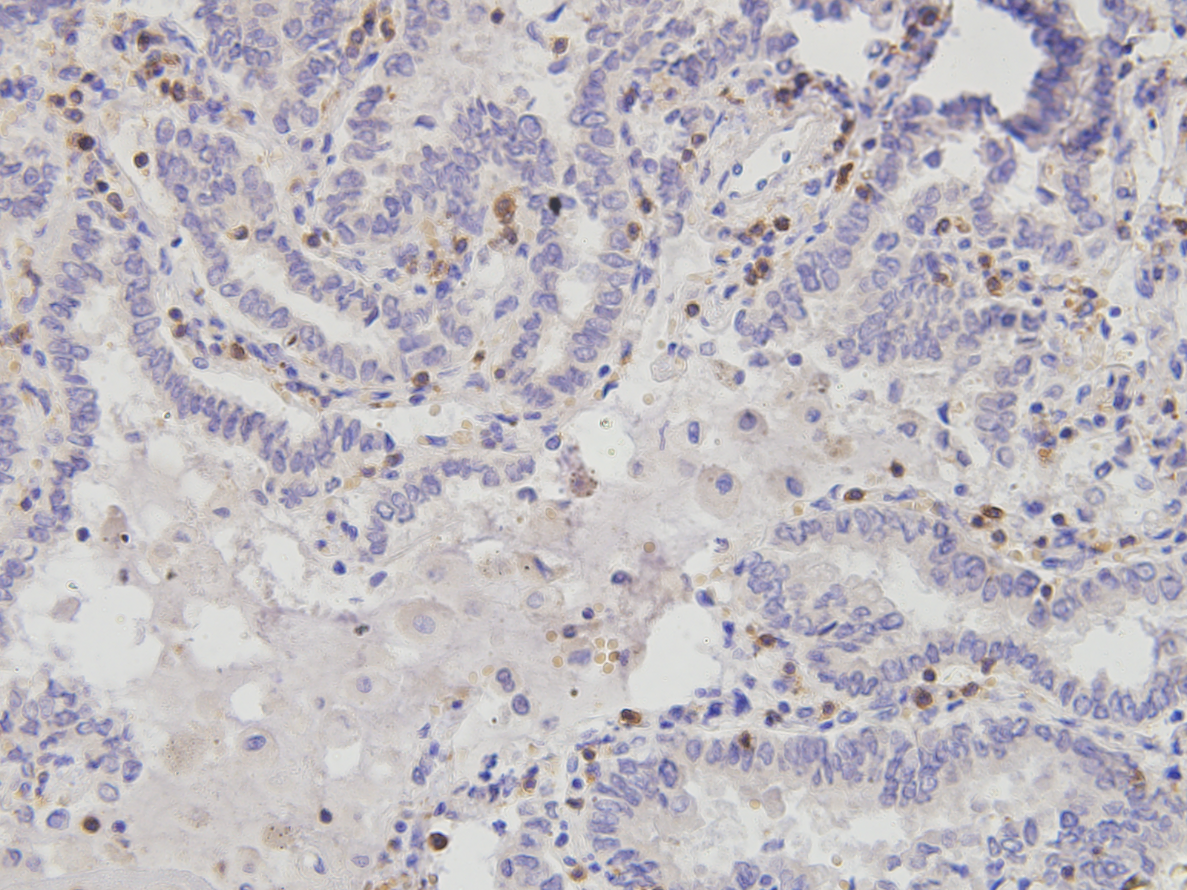

Supplement: S54 File — (ZIP) [file pone.0337223.s055.zip › 516655-400X-CA-N/516655-400X-CA (1).tif]

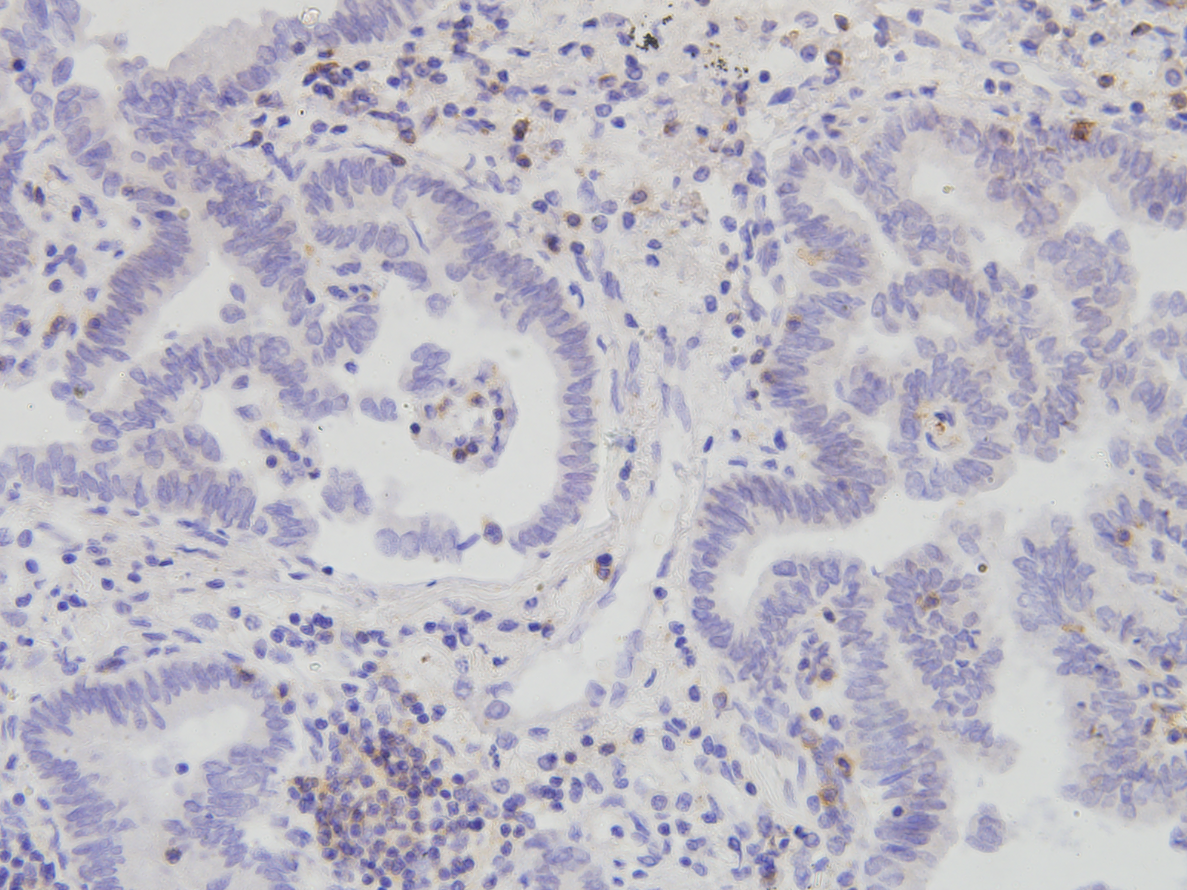

Supplement: S54 File — (ZIP) [file pone.0337223.s055.zip › 516655-400X-CA-N/516655-400X-CA (2).tif]

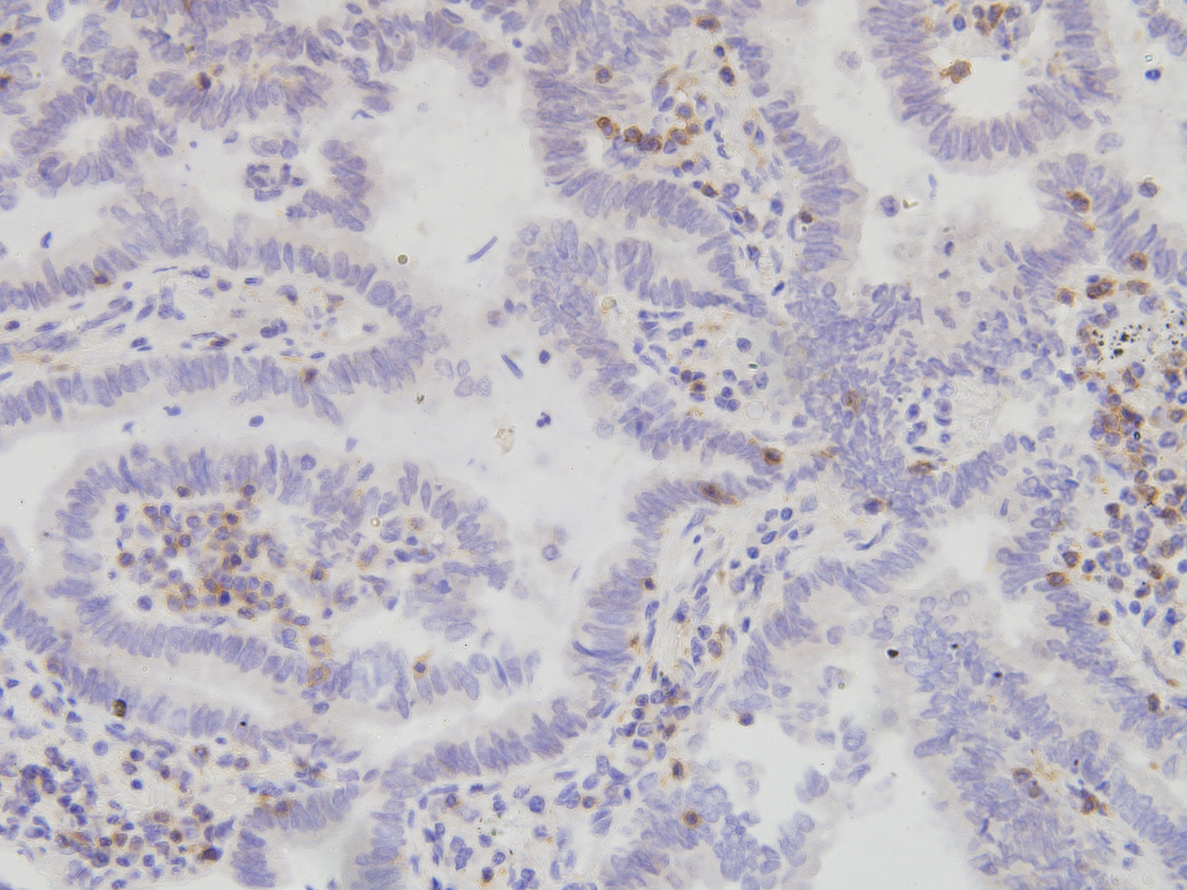

Supplement: S54 File — (ZIP) [file pone.0337223.s055.zip › 516655-400X-CA-N/516655-400X-CA (3).tif]

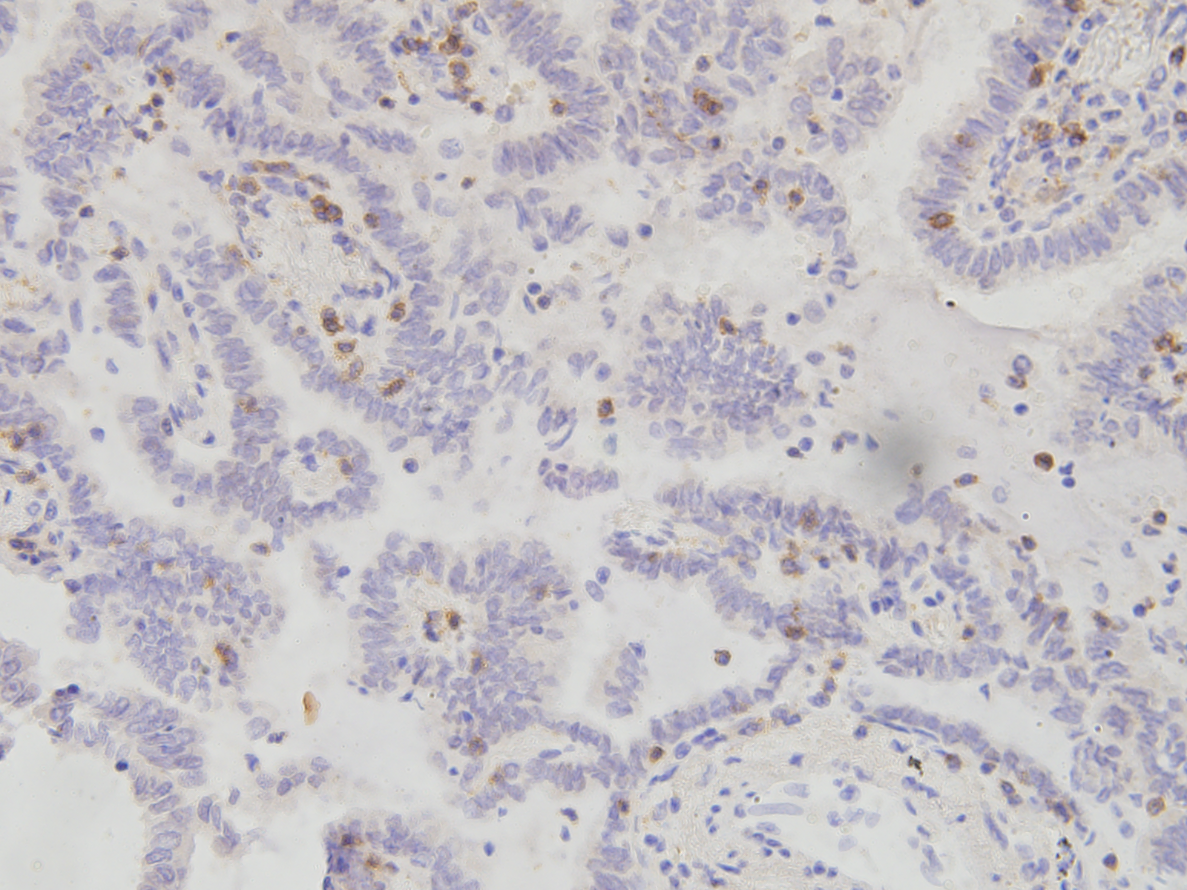

Supplement: S54 File — (ZIP) [file pone.0337223.s055.zip › 516655-400X-CA-N/516655-400X-CA (4).tif]

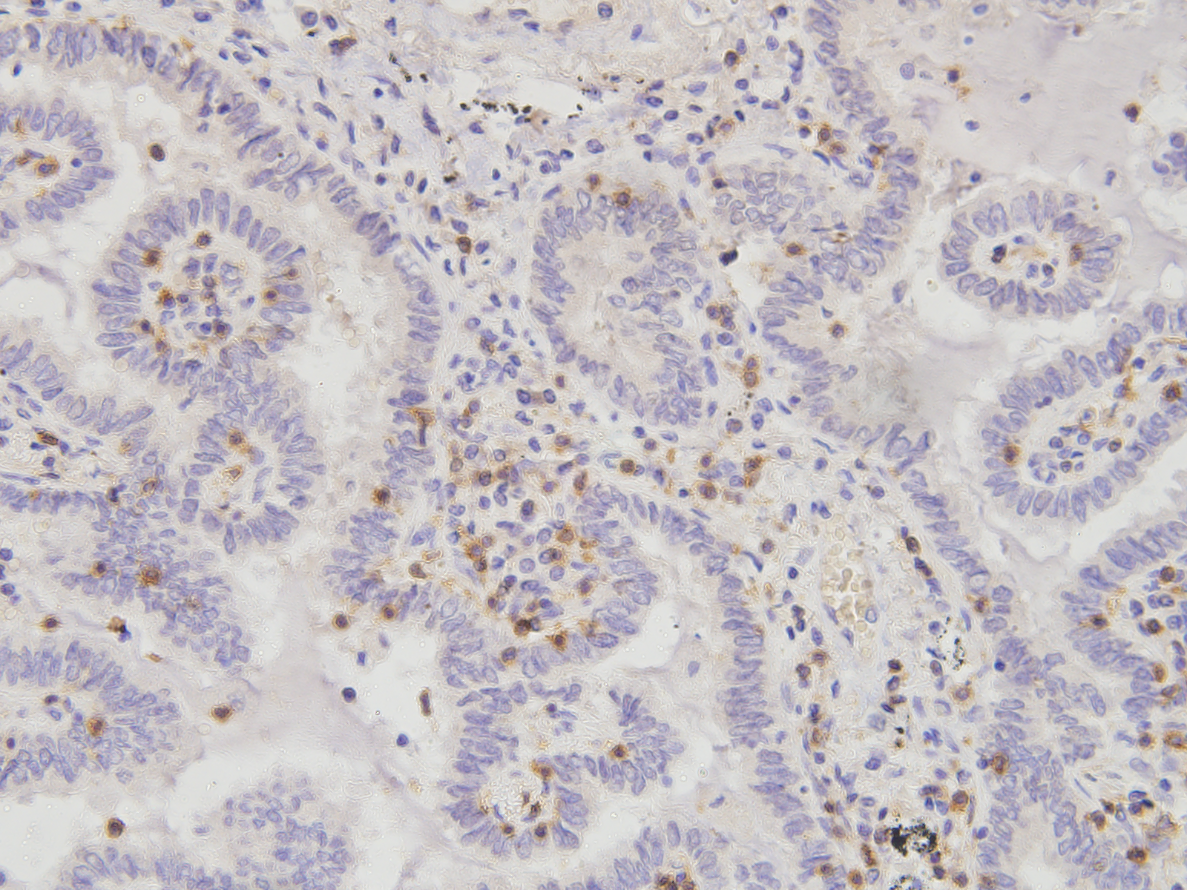

Supplement: S54 File — (ZIP) [file pone.0337223.s055.zip › 516655-400X-CA-N/516655-400X-CA (5).tif]

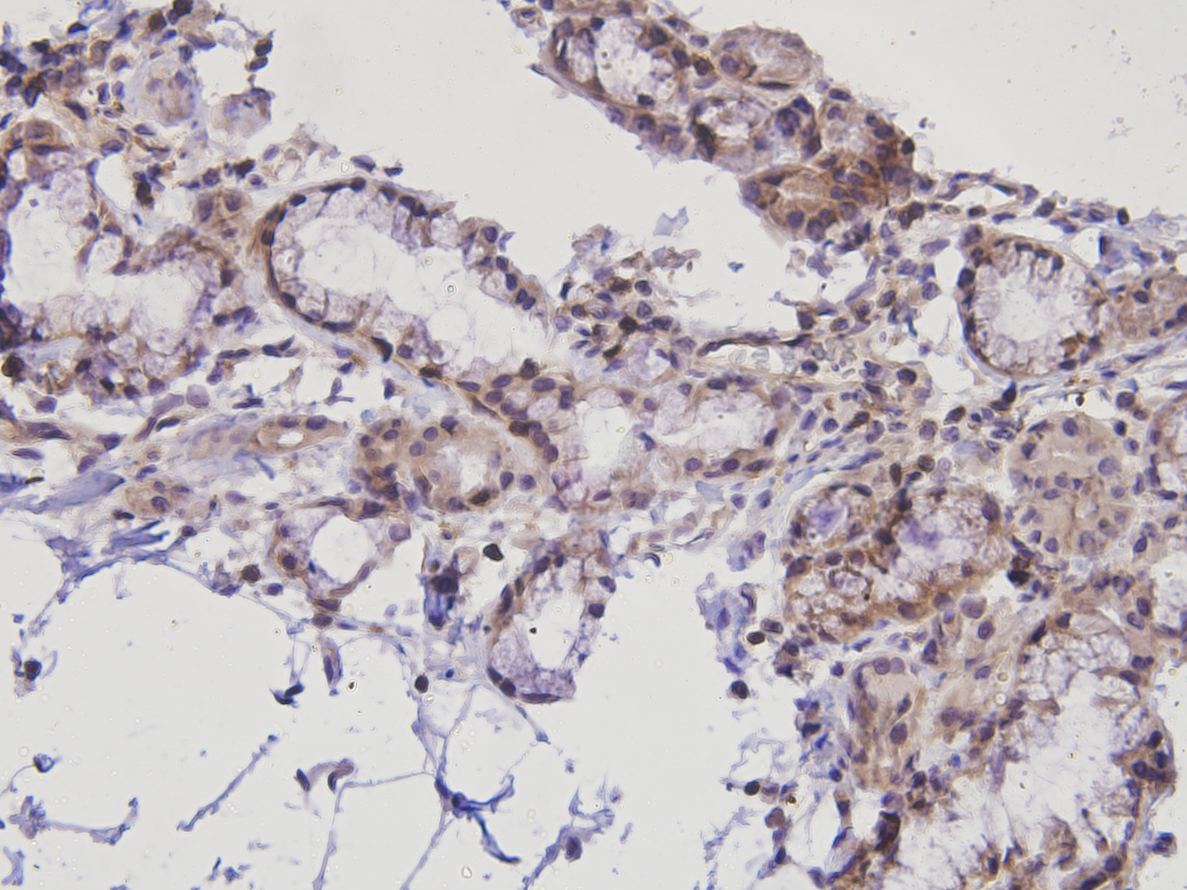

Supplement: S54 File — (ZIP) [file pone.0337223.s055.zip › 516655-400X-CA-N/516655-400X-N (5).tif]

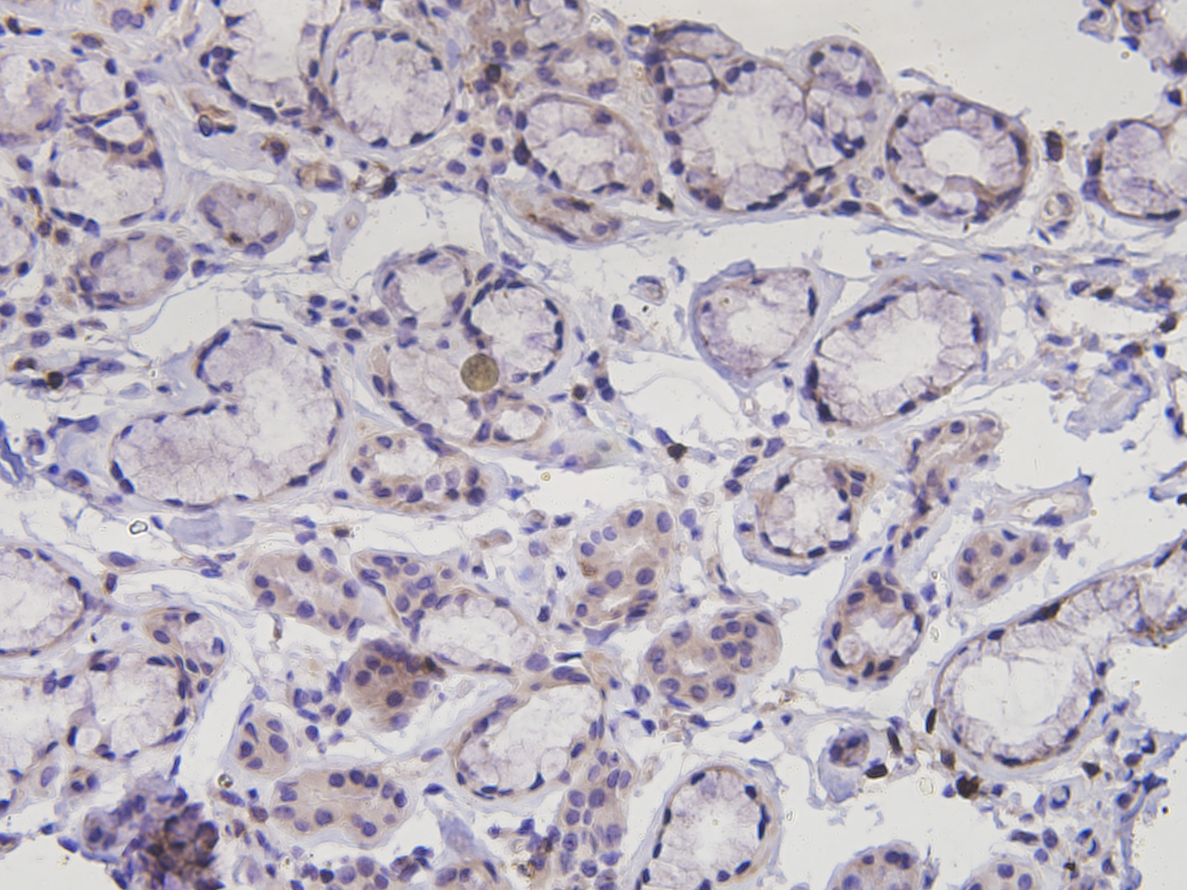

Supplement: S54 File — (ZIP) [file pone.0337223.s055.zip › 516655-400X-CA-N/516655-400X-N (6).tif]

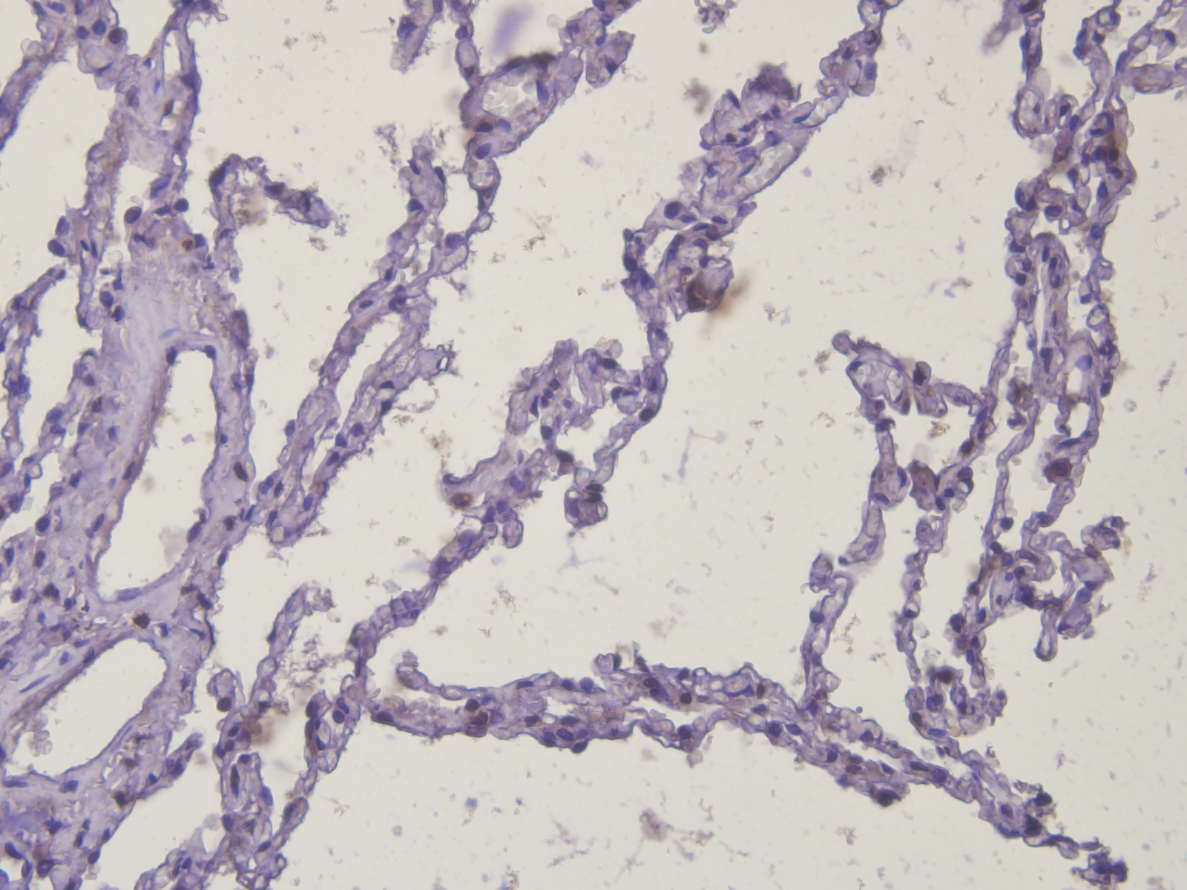

Supplement: S54 File — (ZIP) [file pone.0337223.s055.zip › 516655-400X-CA-N/516655-400X-N (7).tif]

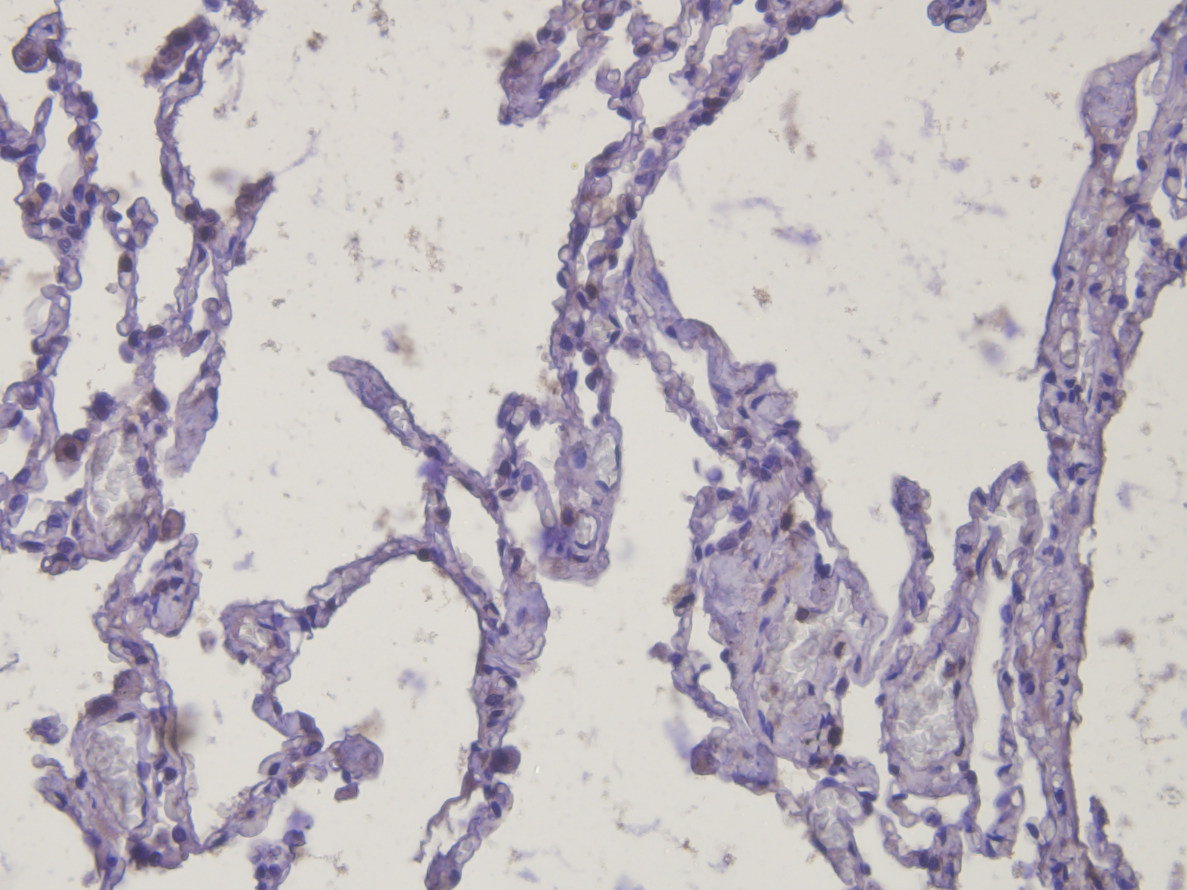

Supplement: S54 File — (ZIP) [file pone.0337223.s055.zip › 516655-400X-CA-N/516655-400X-N (8).tif]

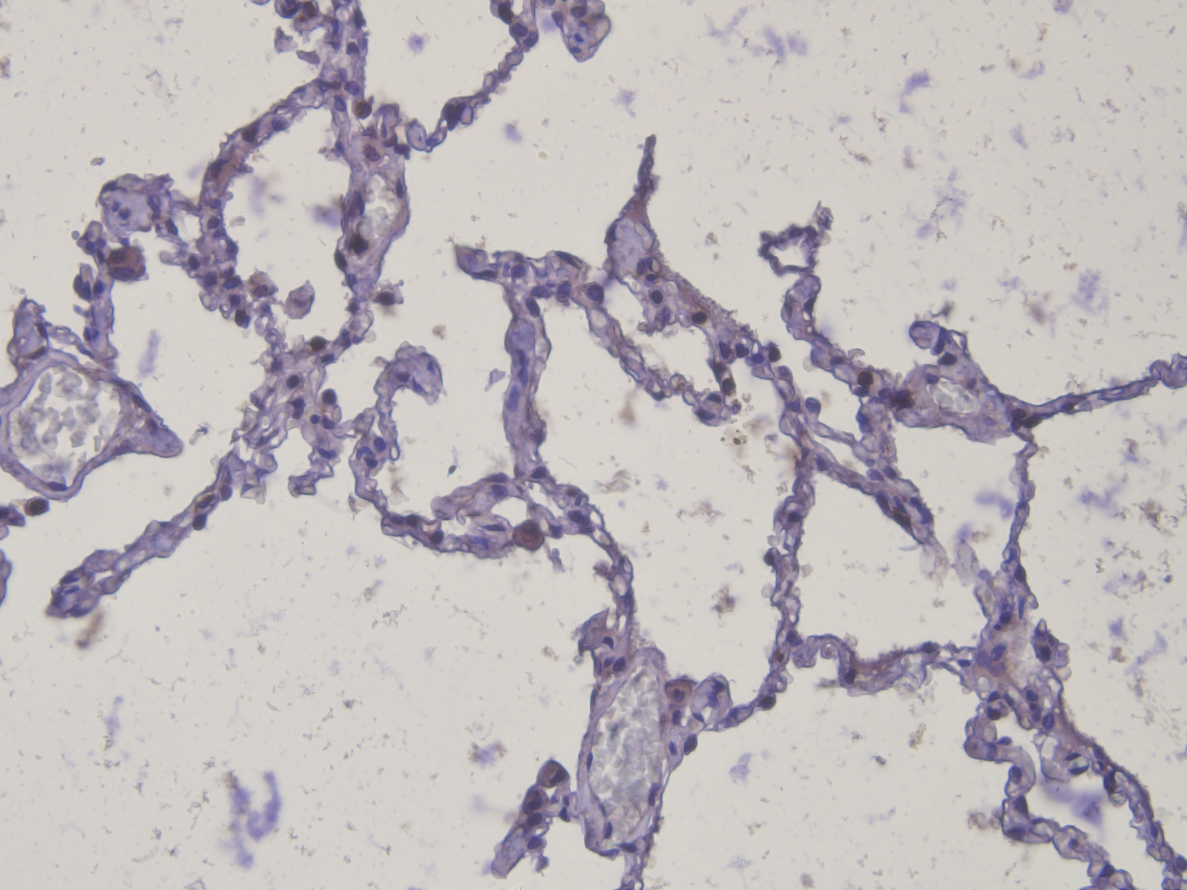

Supplement: S54 File — (ZIP) [file pone.0337223.s055.zip › 516655-400X-CA-N/516655-400X-N (9).tif]

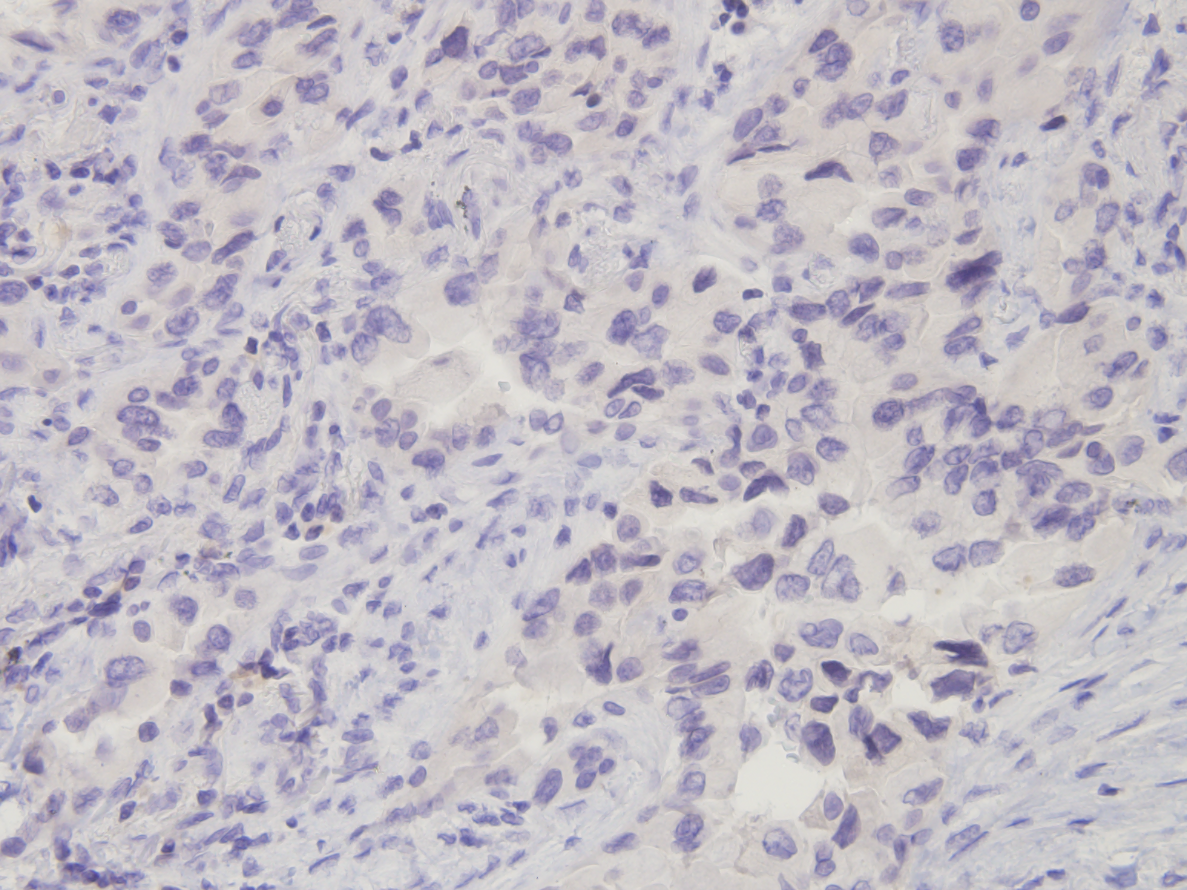

Supplement: S55 File — (ZIP) [file pone.0337223.s056.zip › 516835-400X-CA-N-/516835-400X-CA (1).tif]

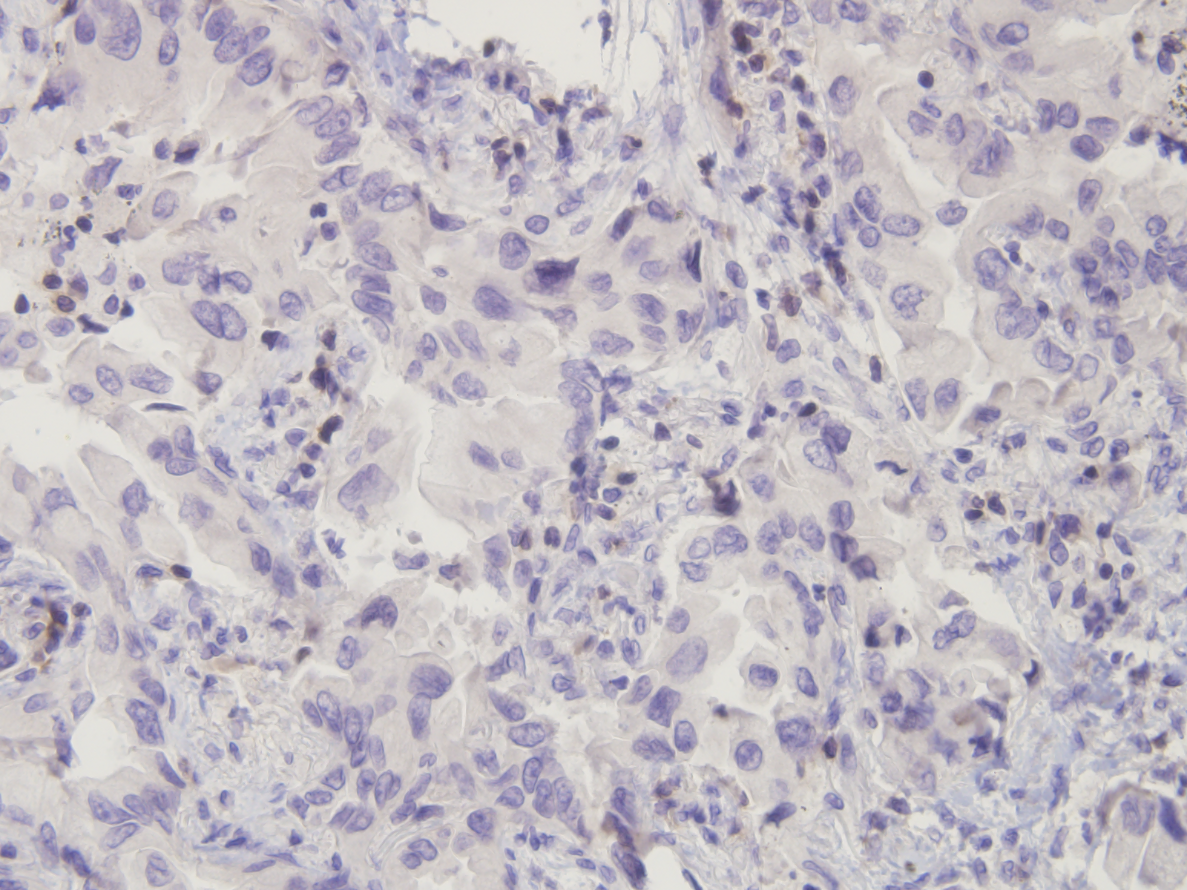

Supplement: S55 File — (ZIP) [file pone.0337223.s056.zip › 516835-400X-CA-N-/516835-400X-CA (2).tif]

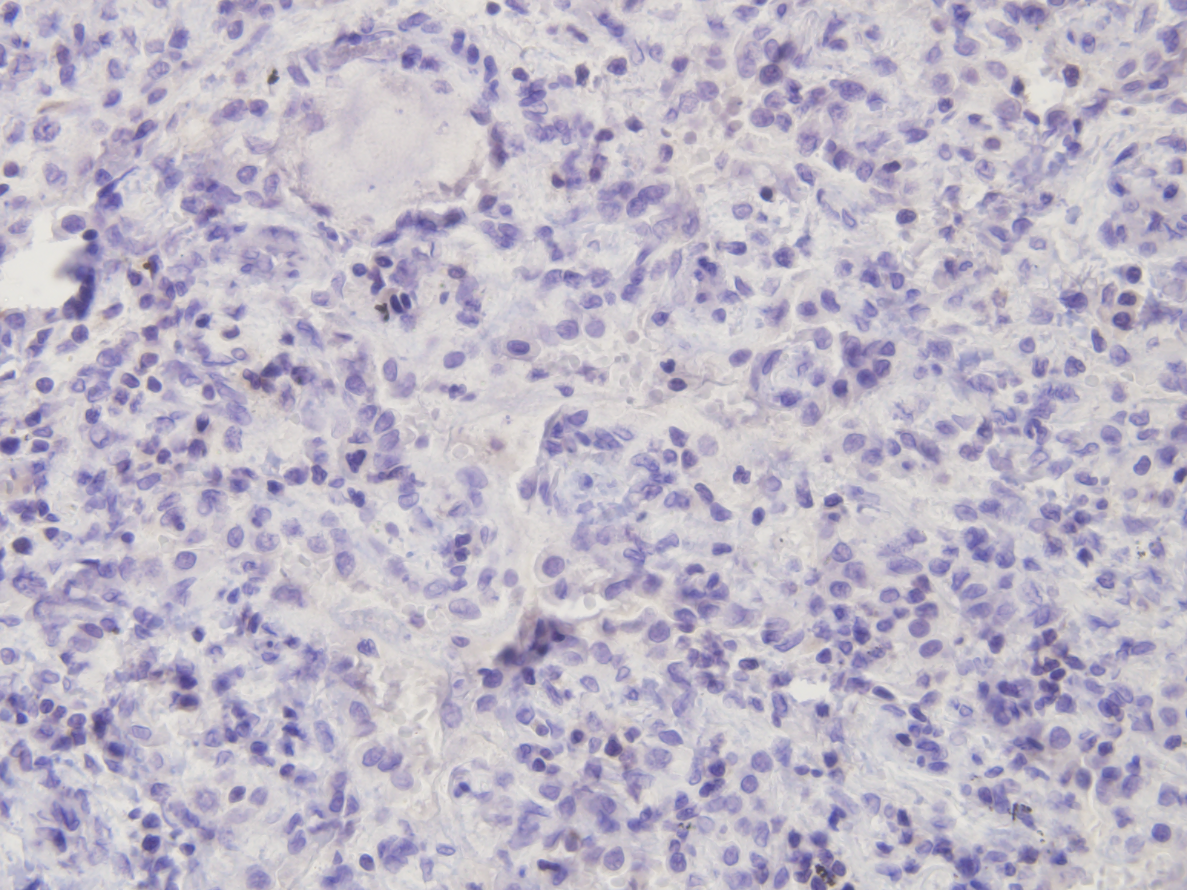

Supplement: S55 File — (ZIP) [file pone.0337223.s056.zip › 516835-400X-CA-N-/516835-400X-CA (3).tif]

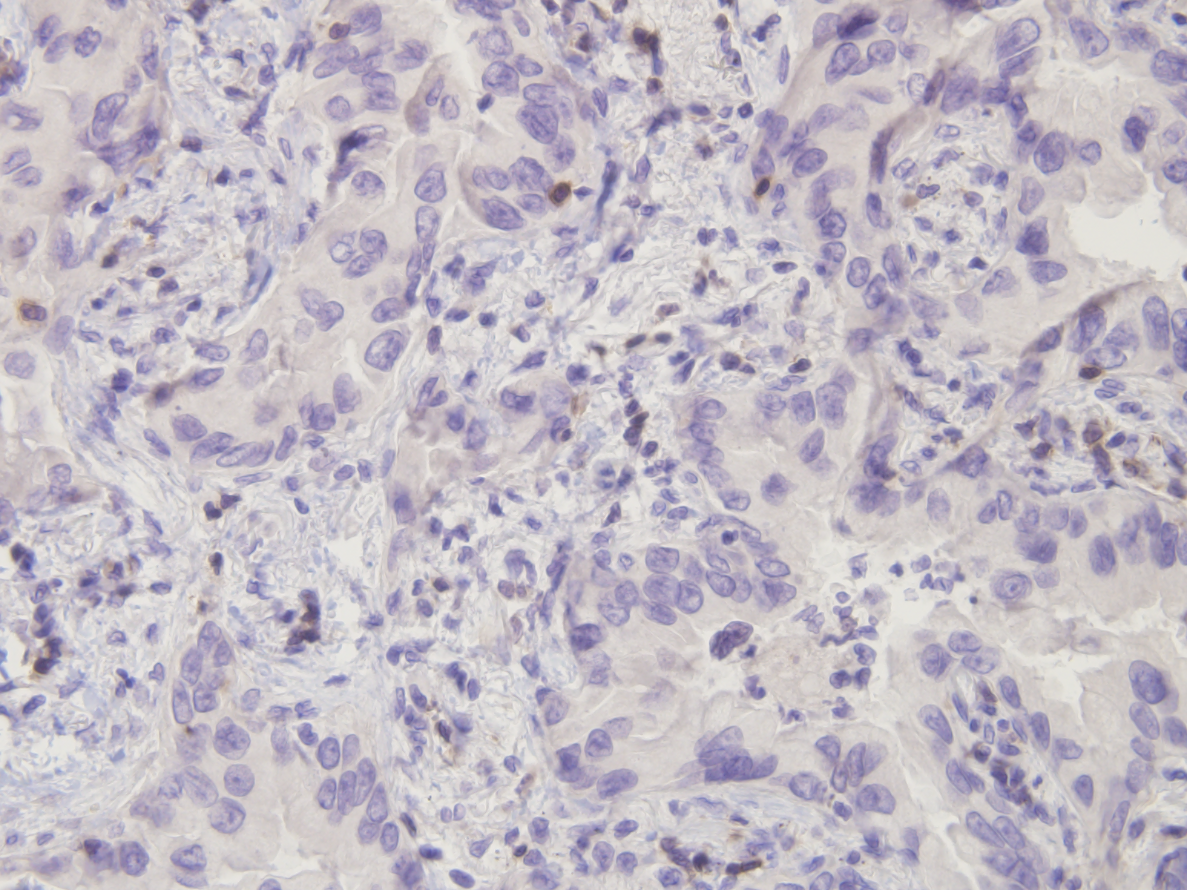

Supplement: S55 File — (ZIP) [file pone.0337223.s056.zip › 516835-400X-CA-N-/516835-400X-CA (4).tif]

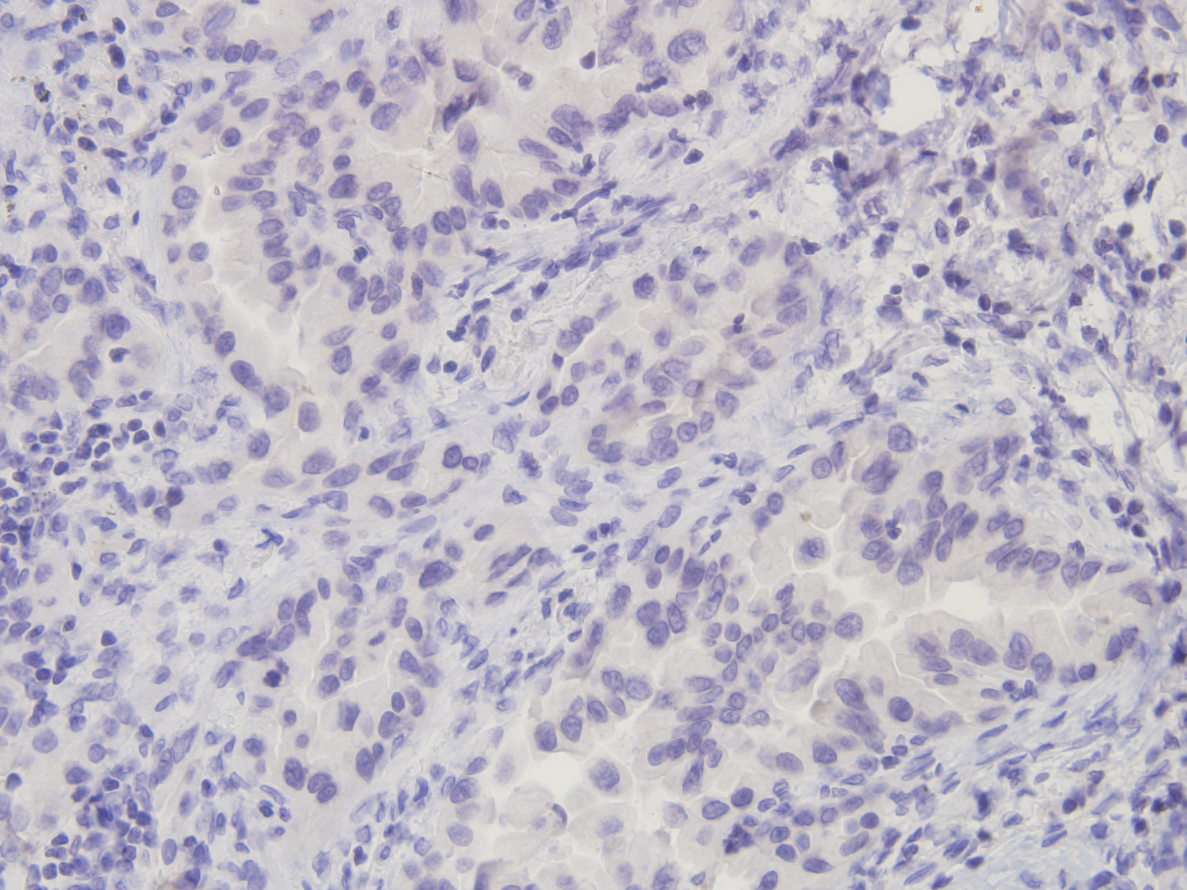

Supplement: S55 File — (ZIP) [file pone.0337223.s056.zip › 516835-400X-CA-N-/516835-400X-CA (5).tif]

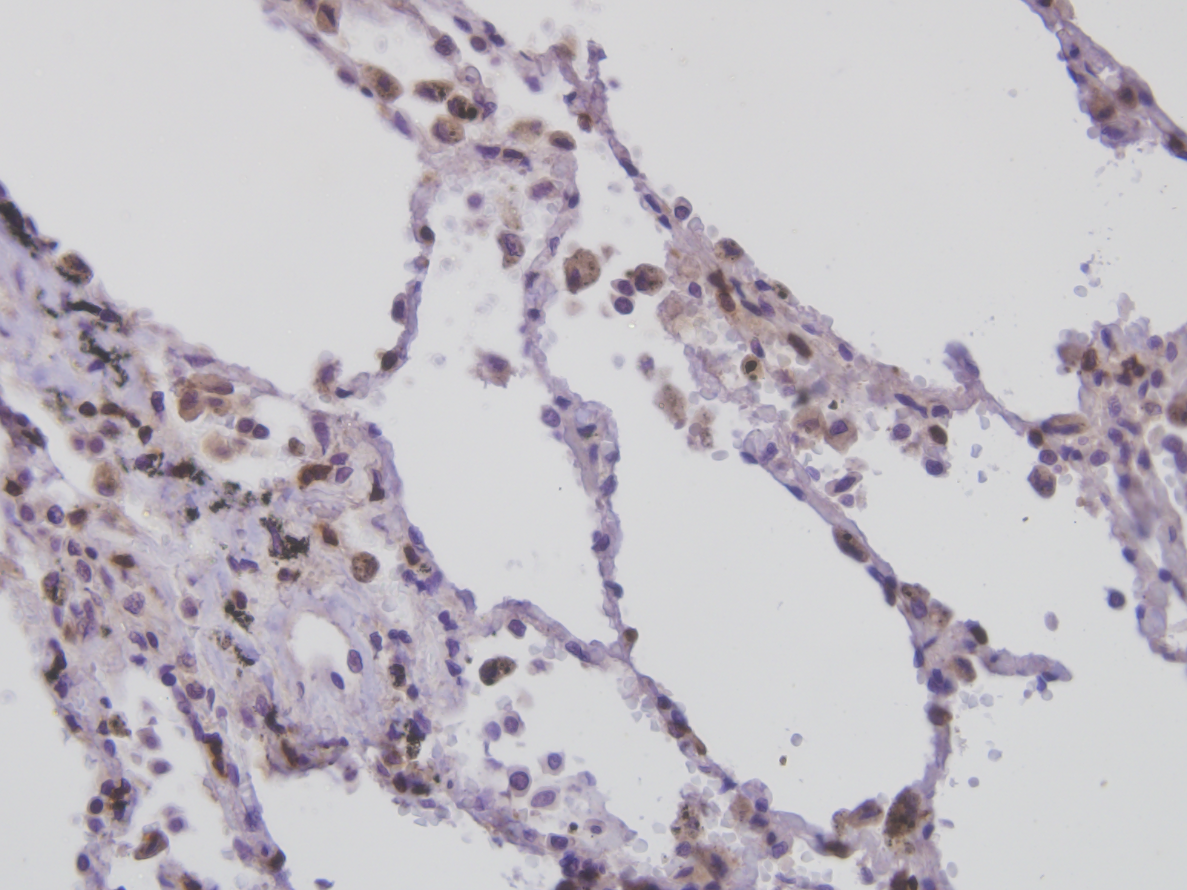

Supplement: S55 File — (ZIP) [file pone.0337223.s056.zip › 516835-400X-CA-N-/516835-400X-N (1).tif]

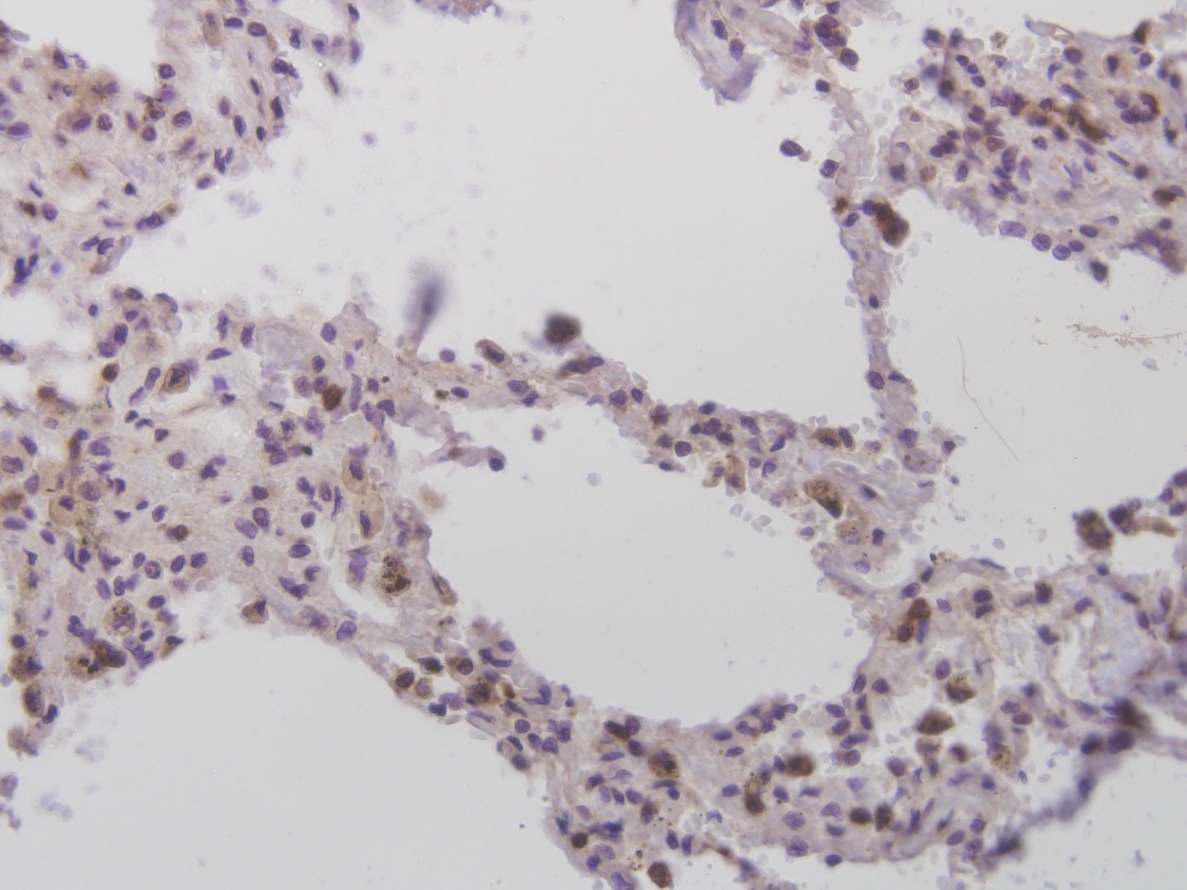

Supplement: S55 File — (ZIP) [file pone.0337223.s056.zip › 516835-400X-CA-N-/516835-400X-N (2).tif]

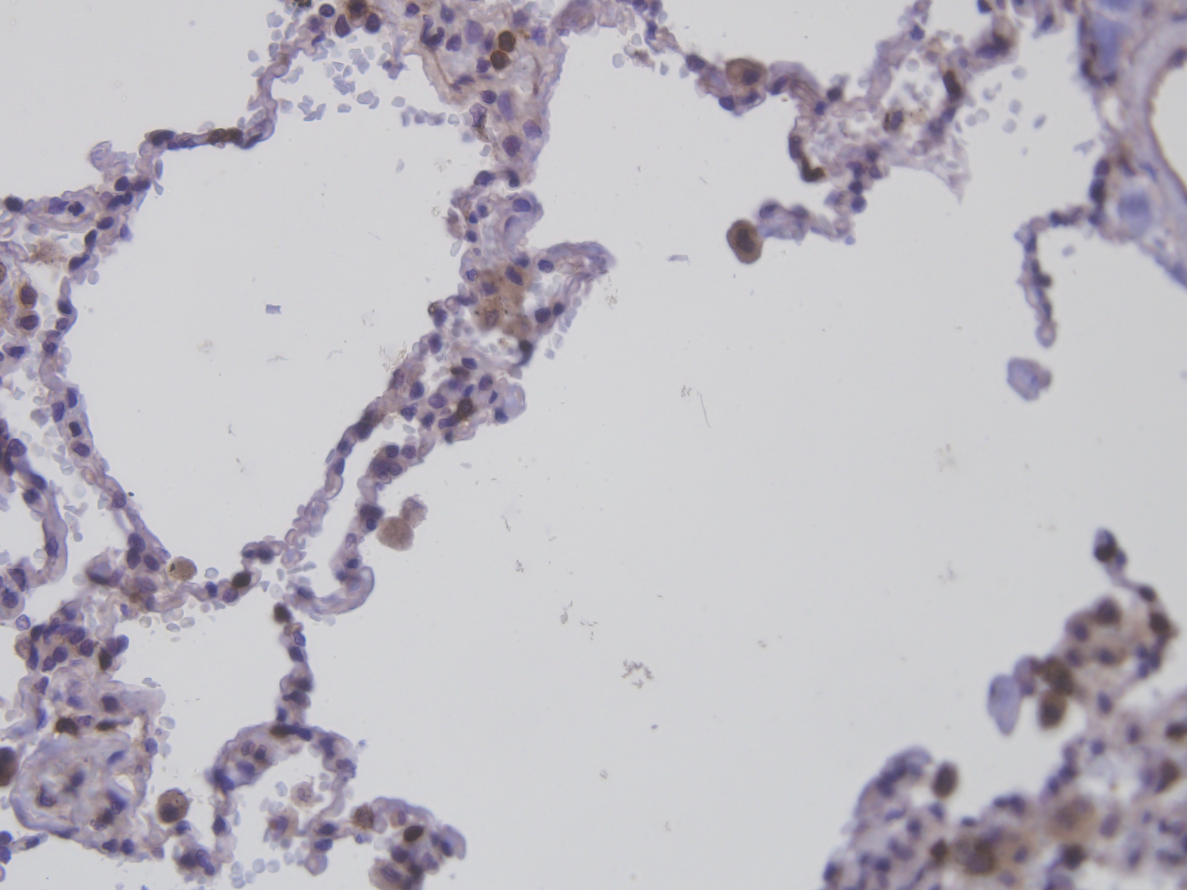

Supplement: S55 File — (ZIP) [file pone.0337223.s056.zip › 516835-400X-CA-N-/516835-400X-N (3).tif]

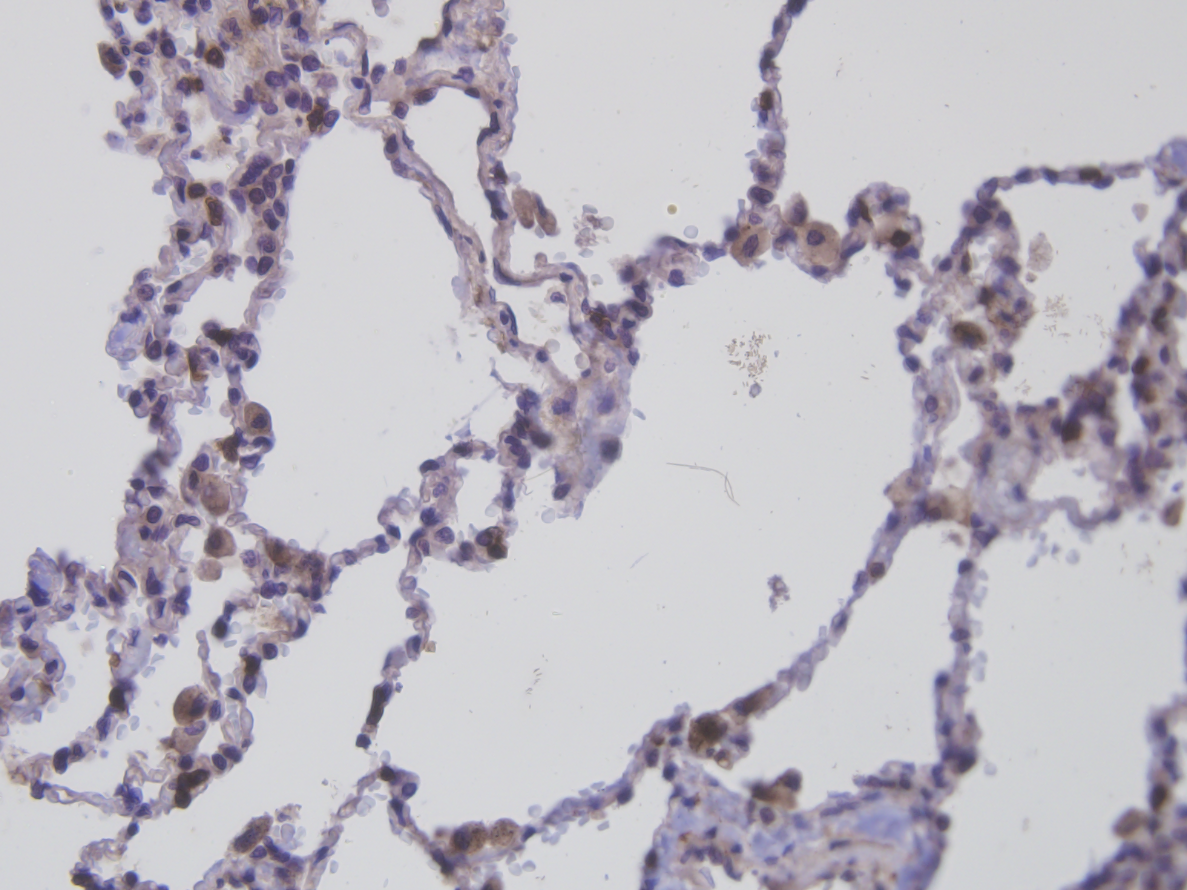

Supplement: S55 File — (ZIP) [file pone.0337223.s056.zip › 516835-400X-CA-N-/516835-400X-N (4).tif]

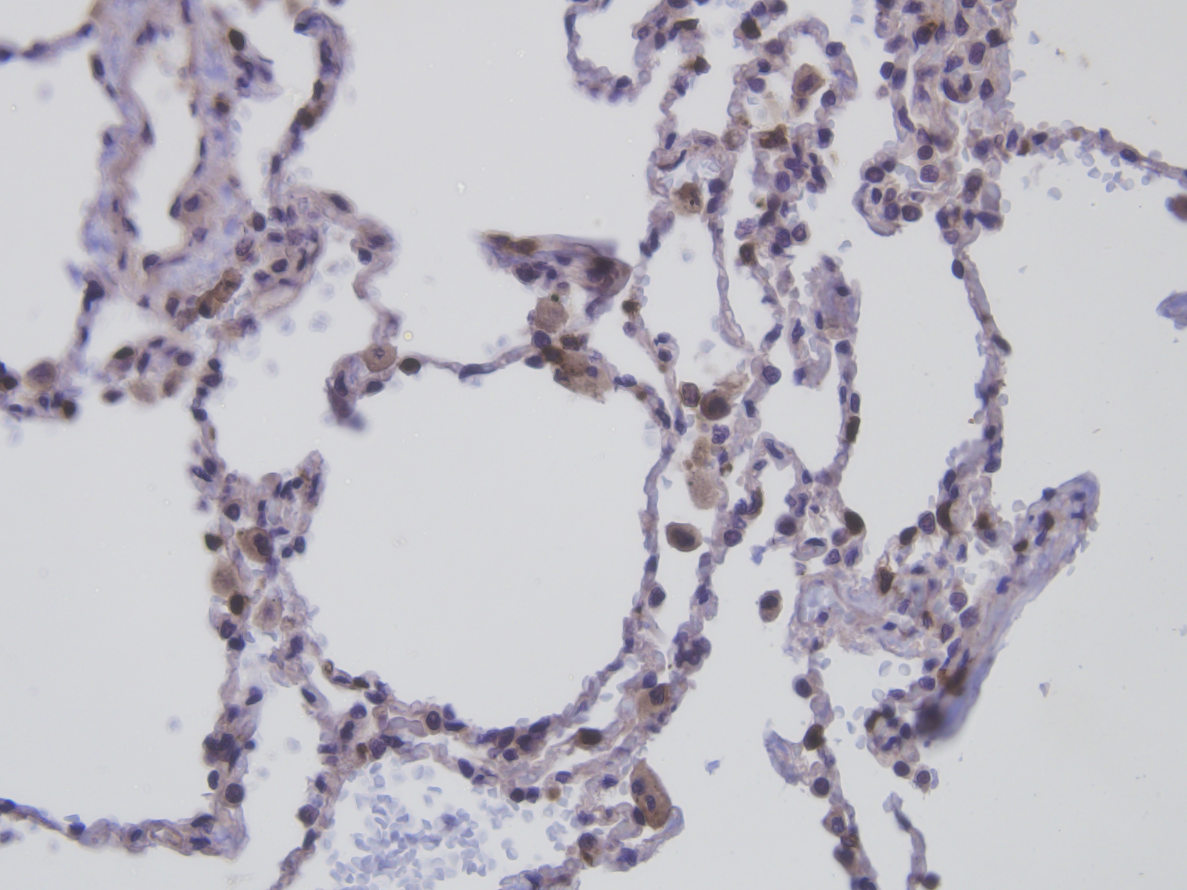

Supplement: S55 File — (ZIP) [file pone.0337223.s056.zip › 516835-400X-CA-N-/516835-400X-N (5).tif]

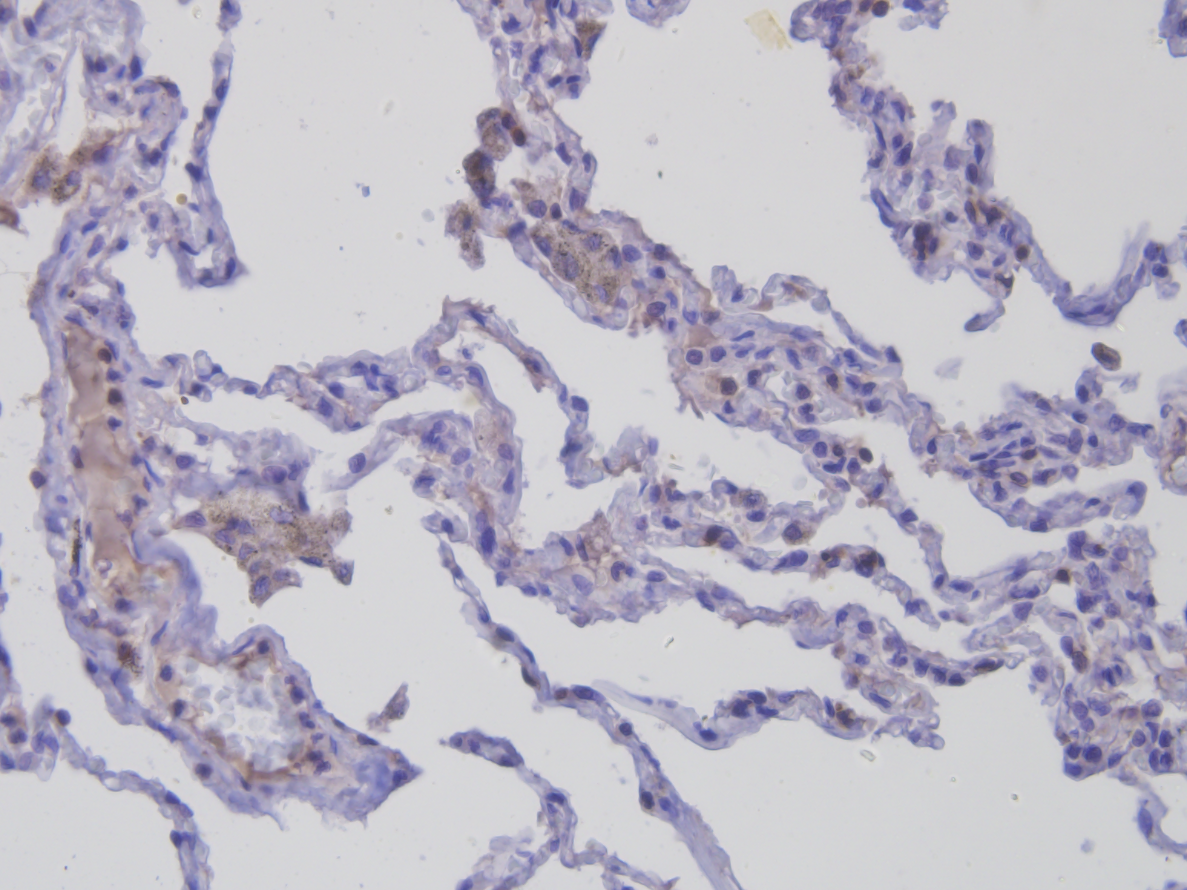

Supplement: S56 File — (ZIP) [file pone.0337223.s057.zip › 517990-400-CA-N/517990-400- N (1).tif]

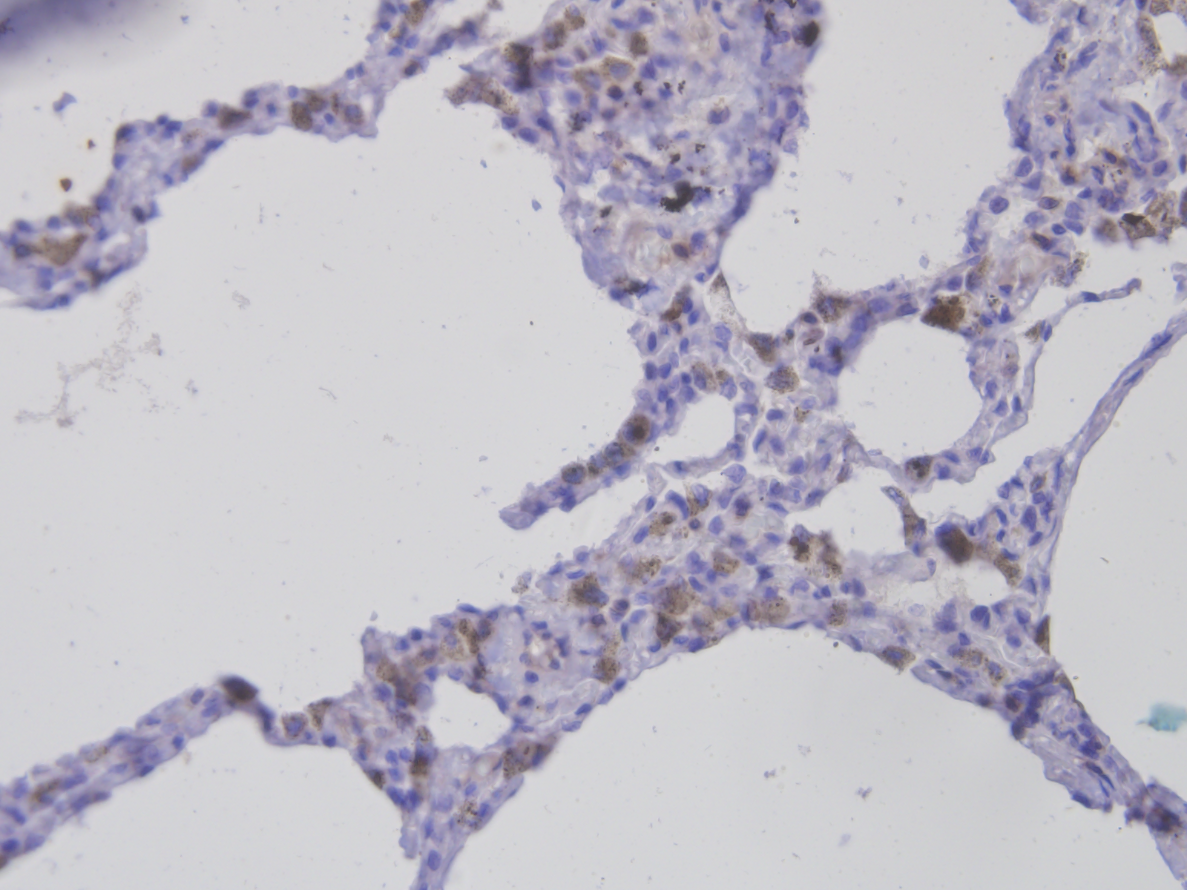

Supplement: S56 File — (ZIP) [file pone.0337223.s057.zip › 517990-400-CA-N/517990-400- N (2).tif]

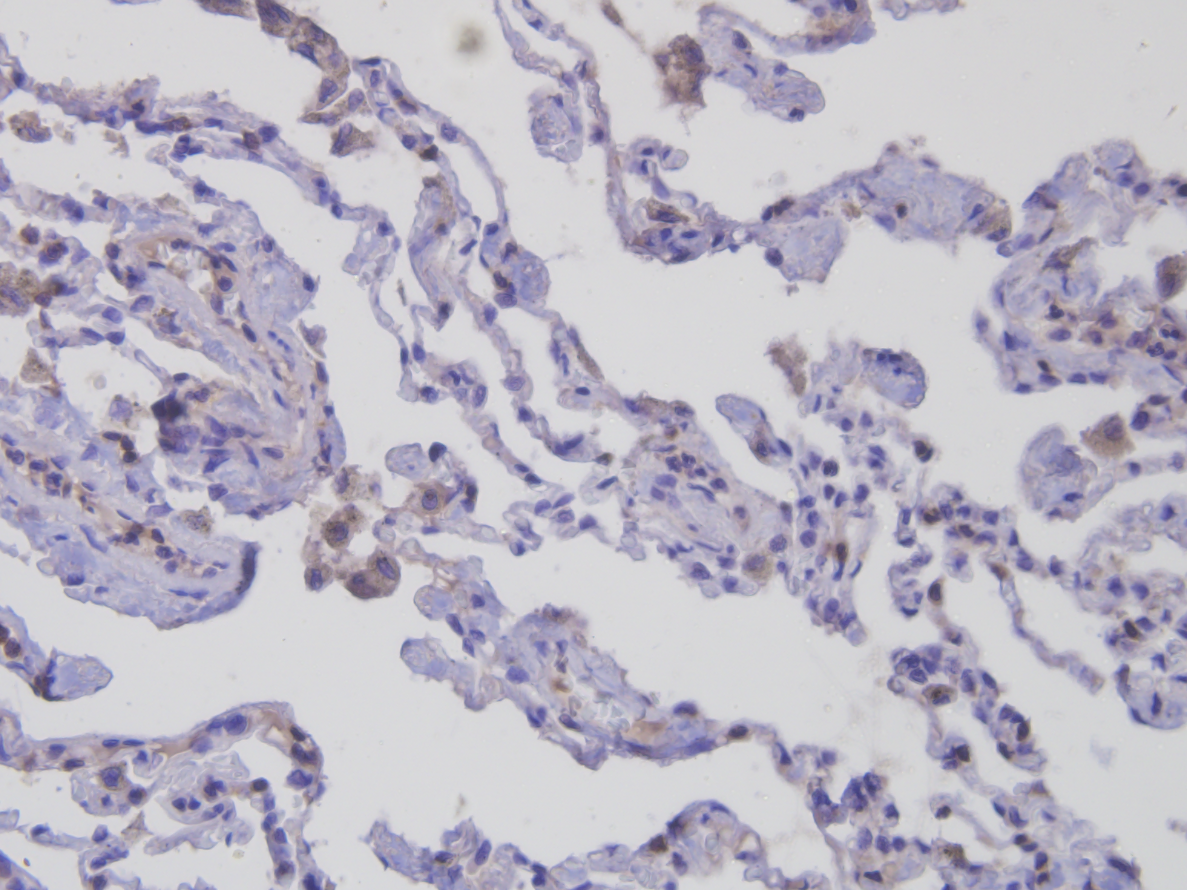

Supplement: S56 File — (ZIP) [file pone.0337223.s057.zip › 517990-400-CA-N/517990-400- N (3).tif]

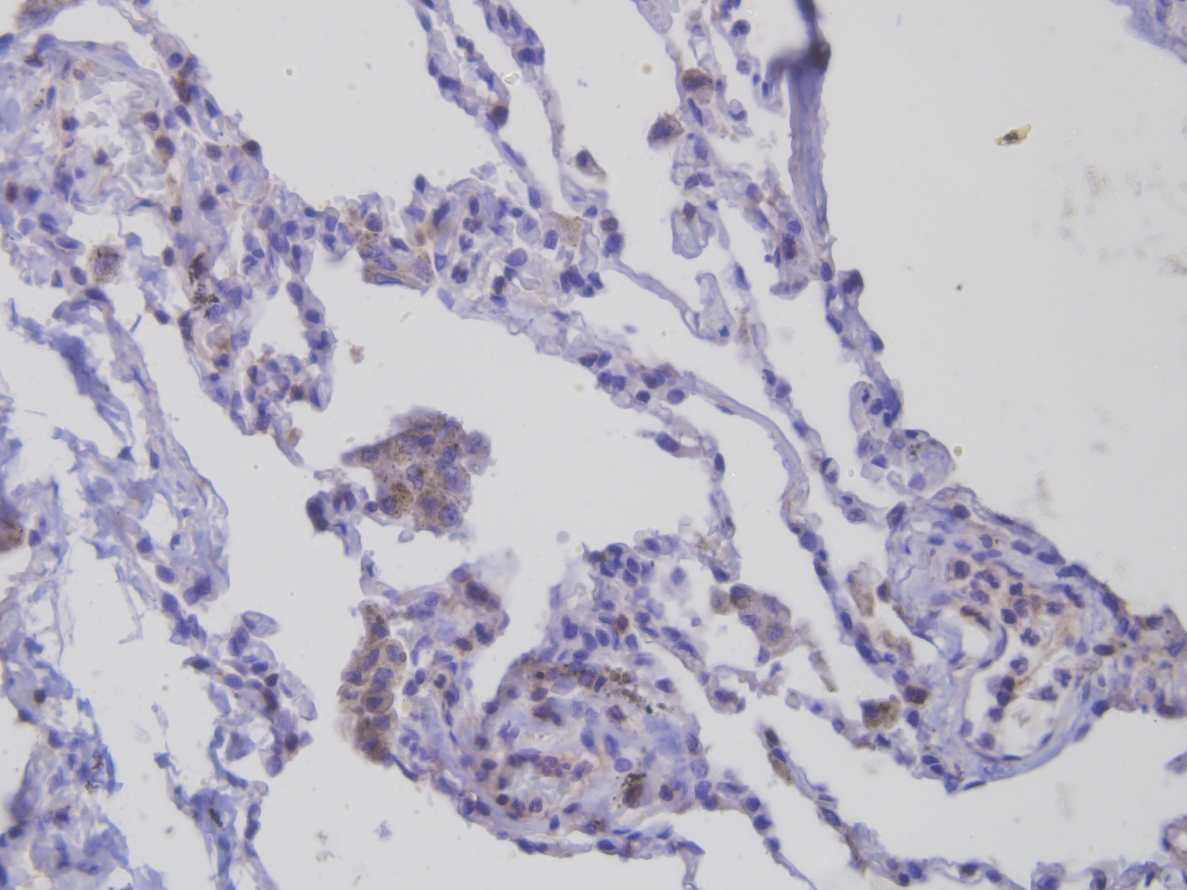

Supplement: S56 File — (ZIP) [file pone.0337223.s057.zip › 517990-400-CA-N/517990-400- N (4).tif]

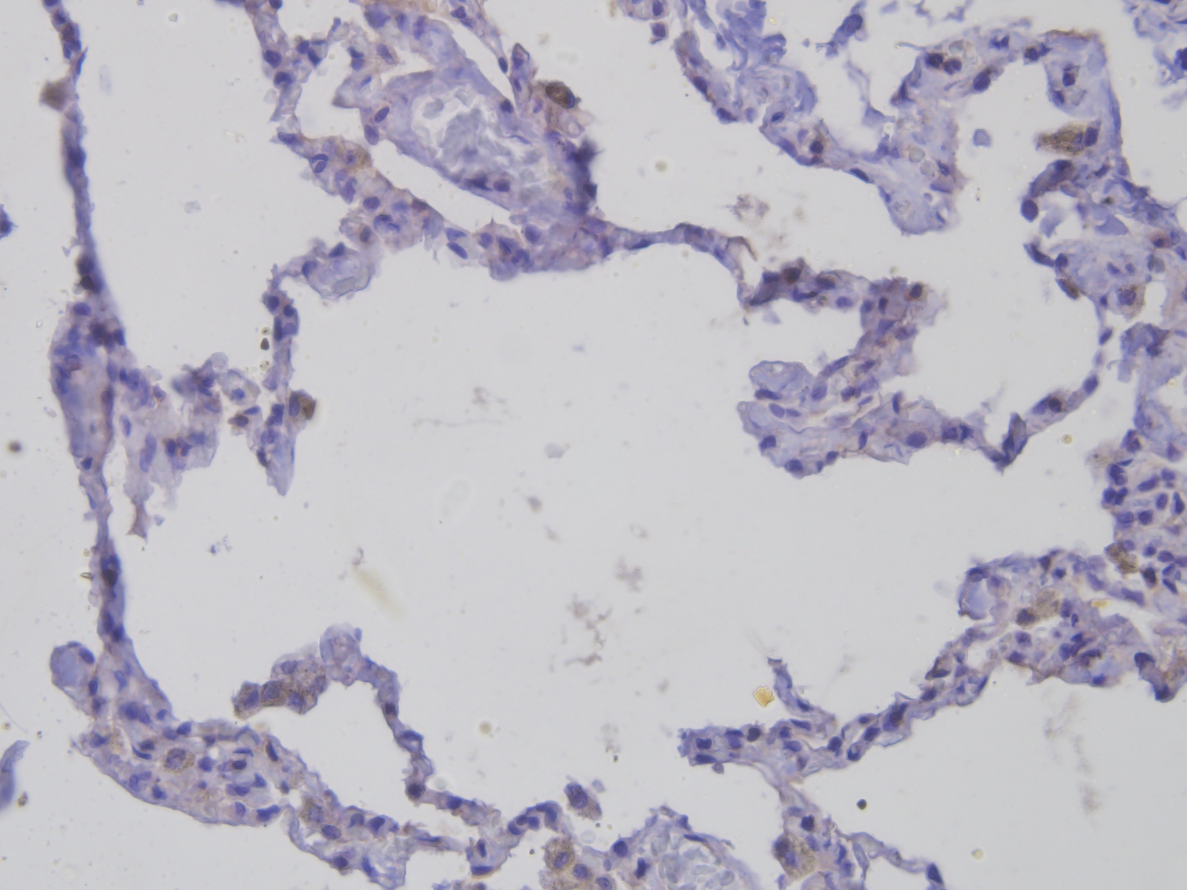

Supplement: S56 File — (ZIP) [file pone.0337223.s057.zip › 517990-400-CA-N/517990-400- N (5).tif]

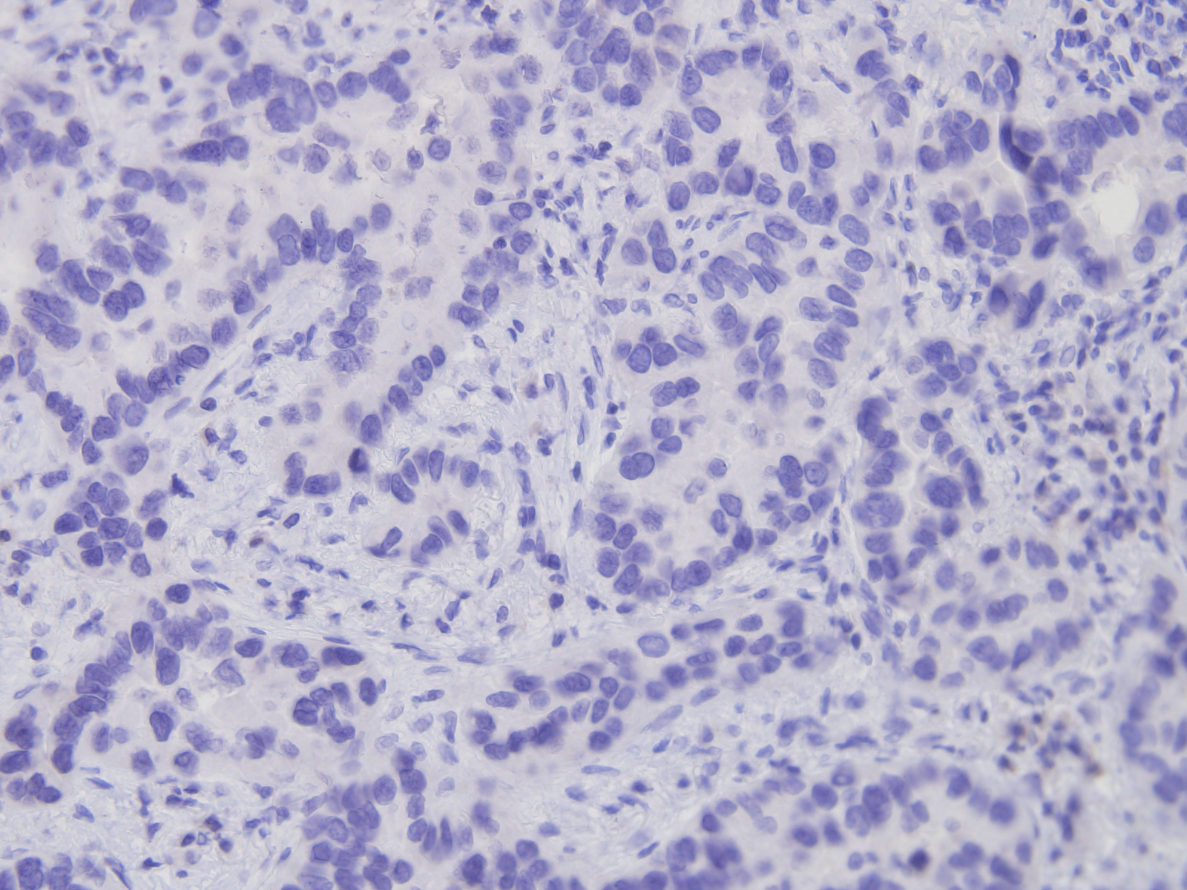

Supplement: S56 File — (ZIP) [file pone.0337223.s057.zip › 517990-400-CA-N/517990-400-CA (1).tif]

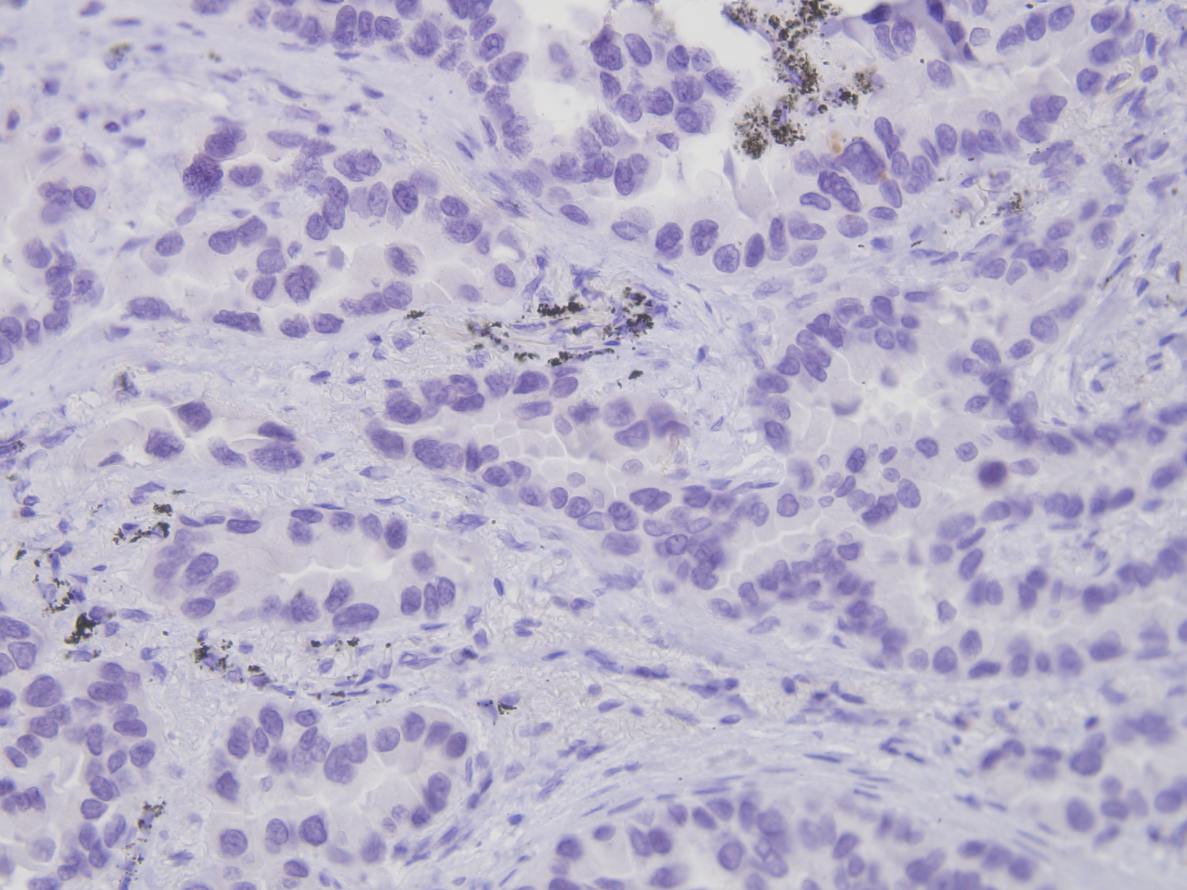

Supplement: S56 File — (ZIP) [file pone.0337223.s057.zip › 517990-400-CA-N/517990-400-CA (2).tif]

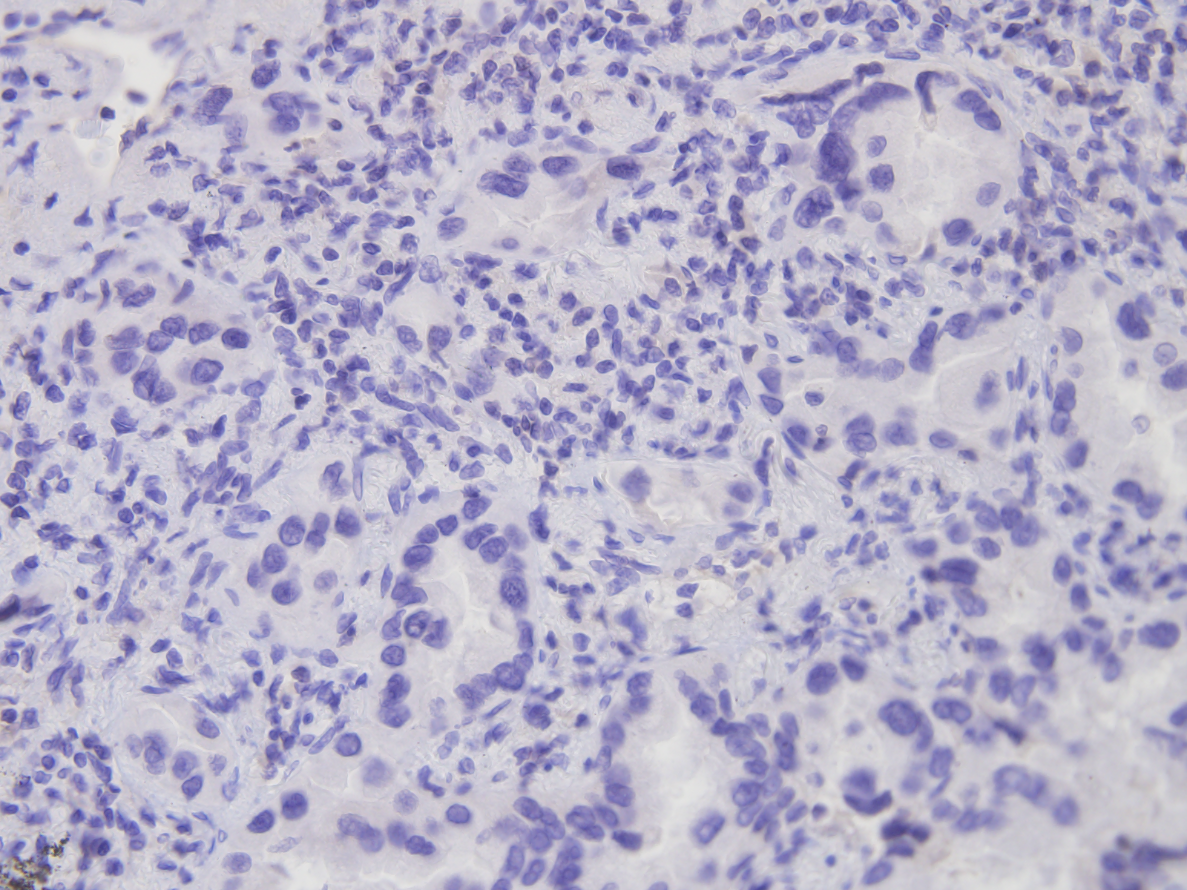

Supplement: S56 File — (ZIP) [file pone.0337223.s057.zip › 517990-400-CA-N/517990-400-CA (3).tif]

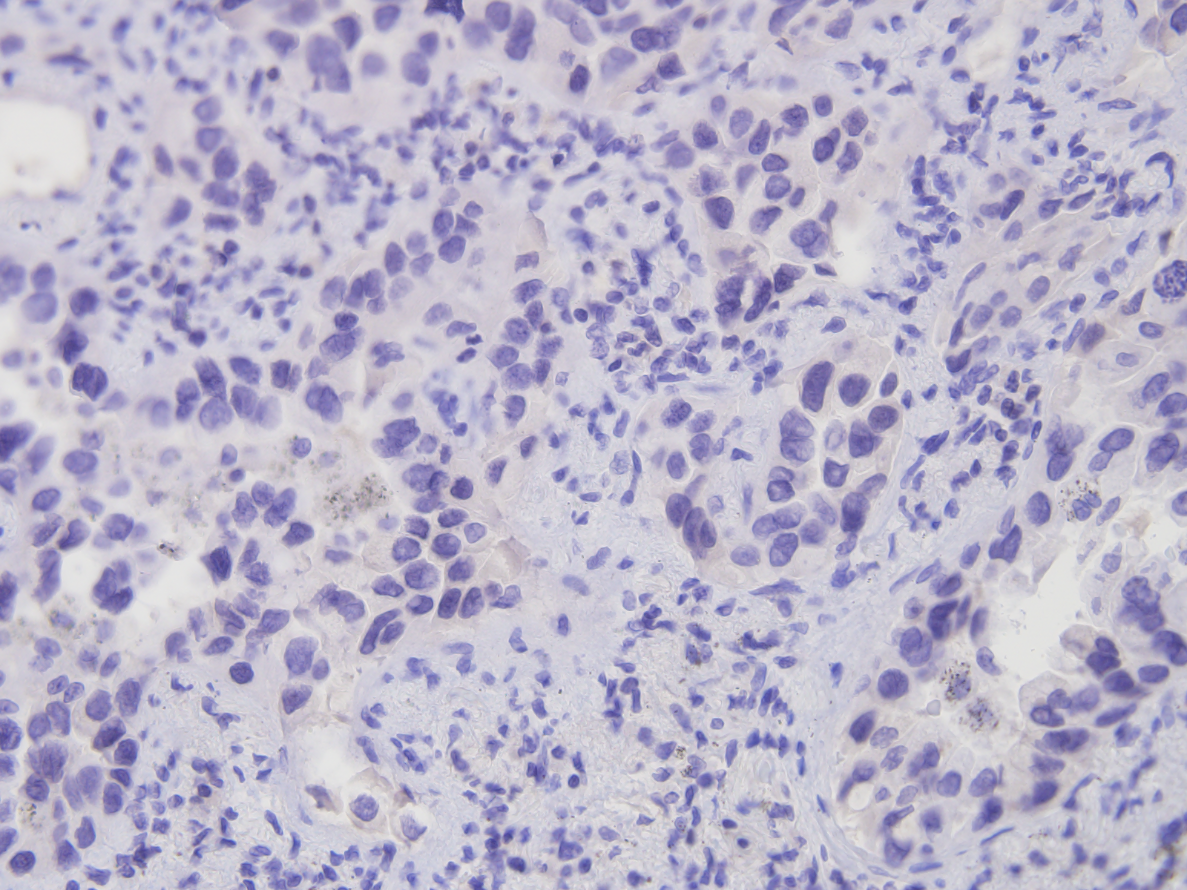

Supplement: S56 File — (ZIP) [file pone.0337223.s057.zip › 517990-400-CA-N/517990-400-CA (4).tif]

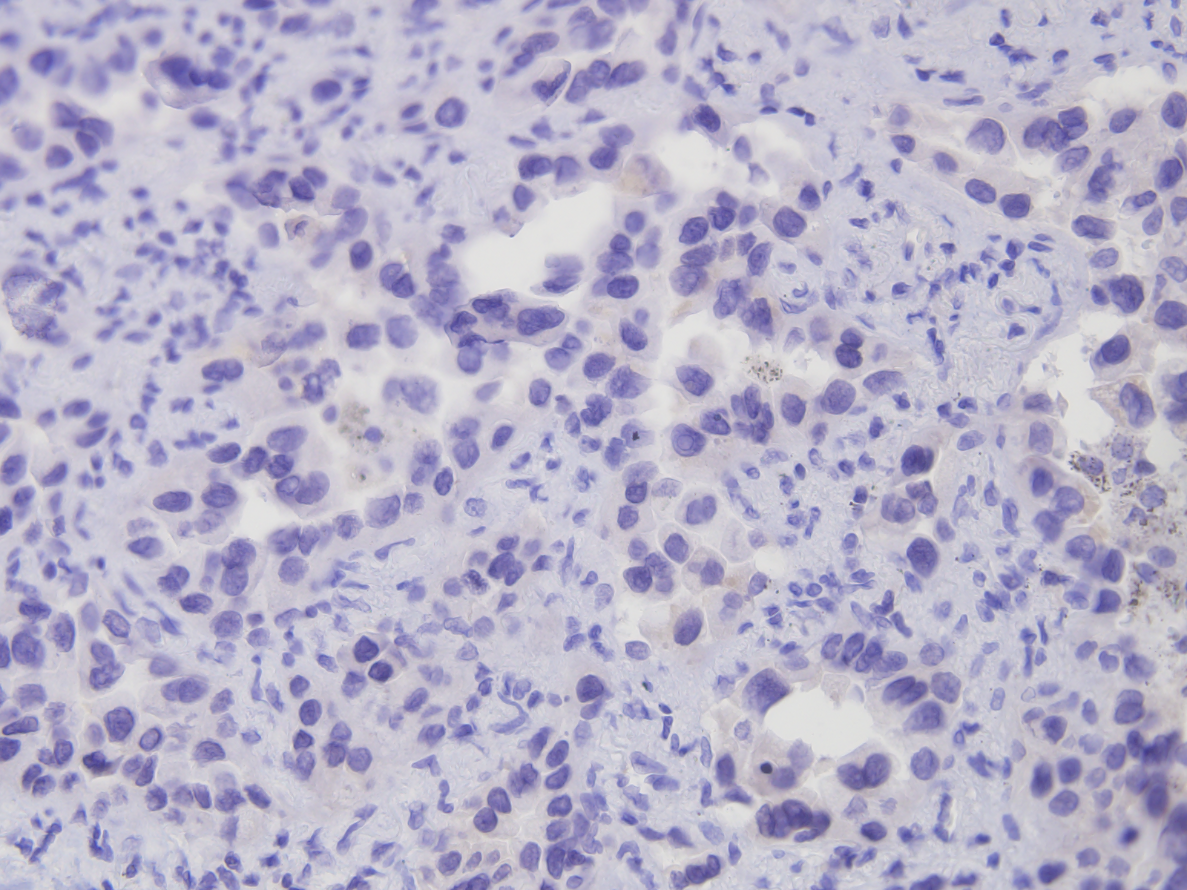

Supplement: S56 File — (ZIP) [file pone.0337223.s057.zip › 517990-400-CA-N/517990-400-CA (5).tif]

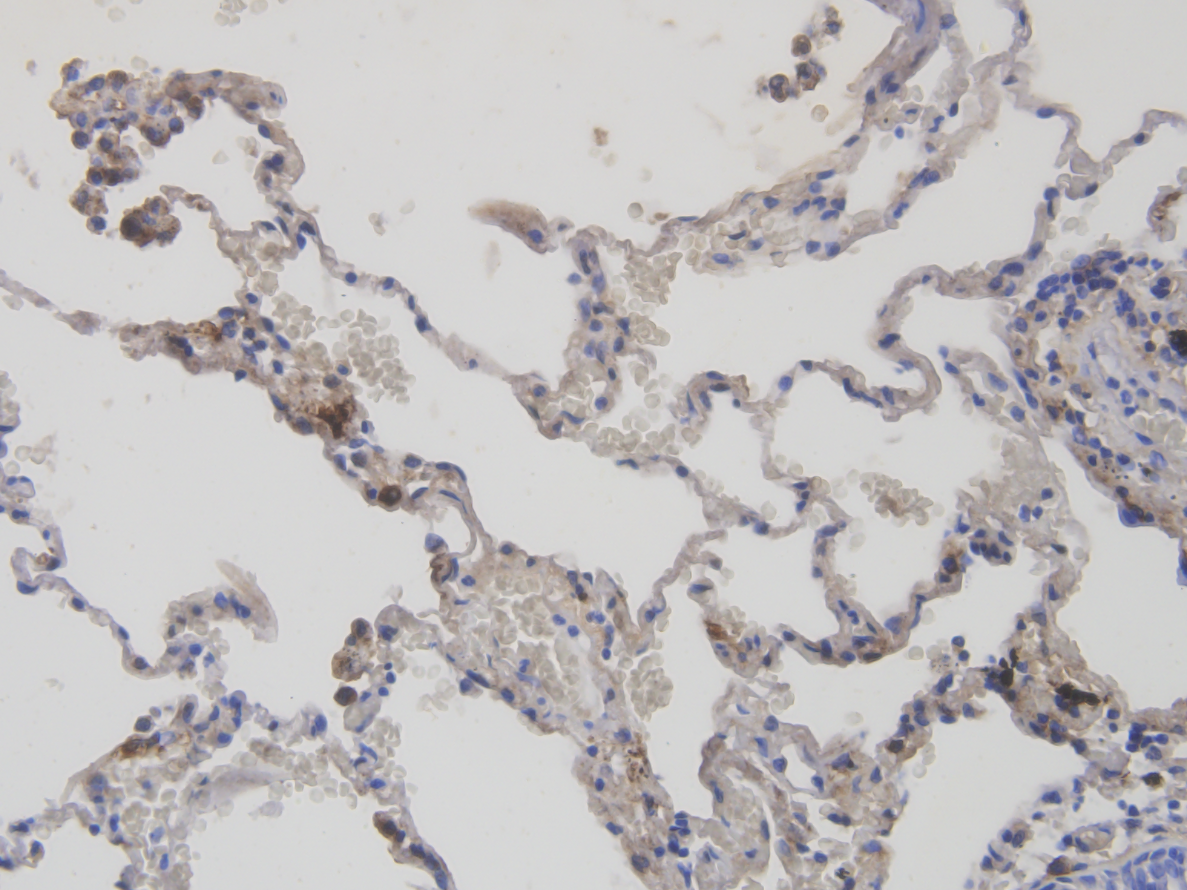

Supplement: S57 File — (ZIP) [file pone.0337223.s058.zip › 517993-400X-CA-N/517993-400X- N (1).tif]

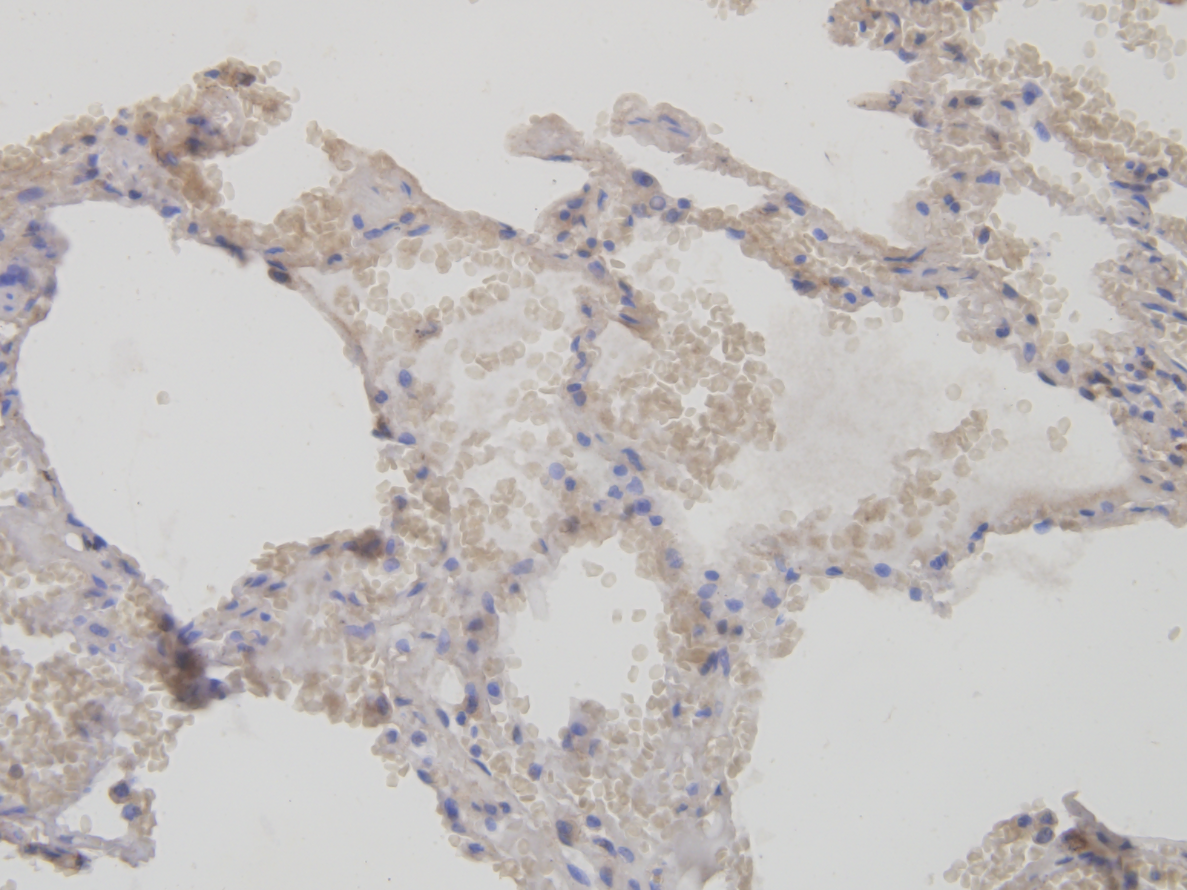

Supplement: S57 File — (ZIP) [file pone.0337223.s058.zip › 517993-400X-CA-N/517993-400X- N (2).tif]

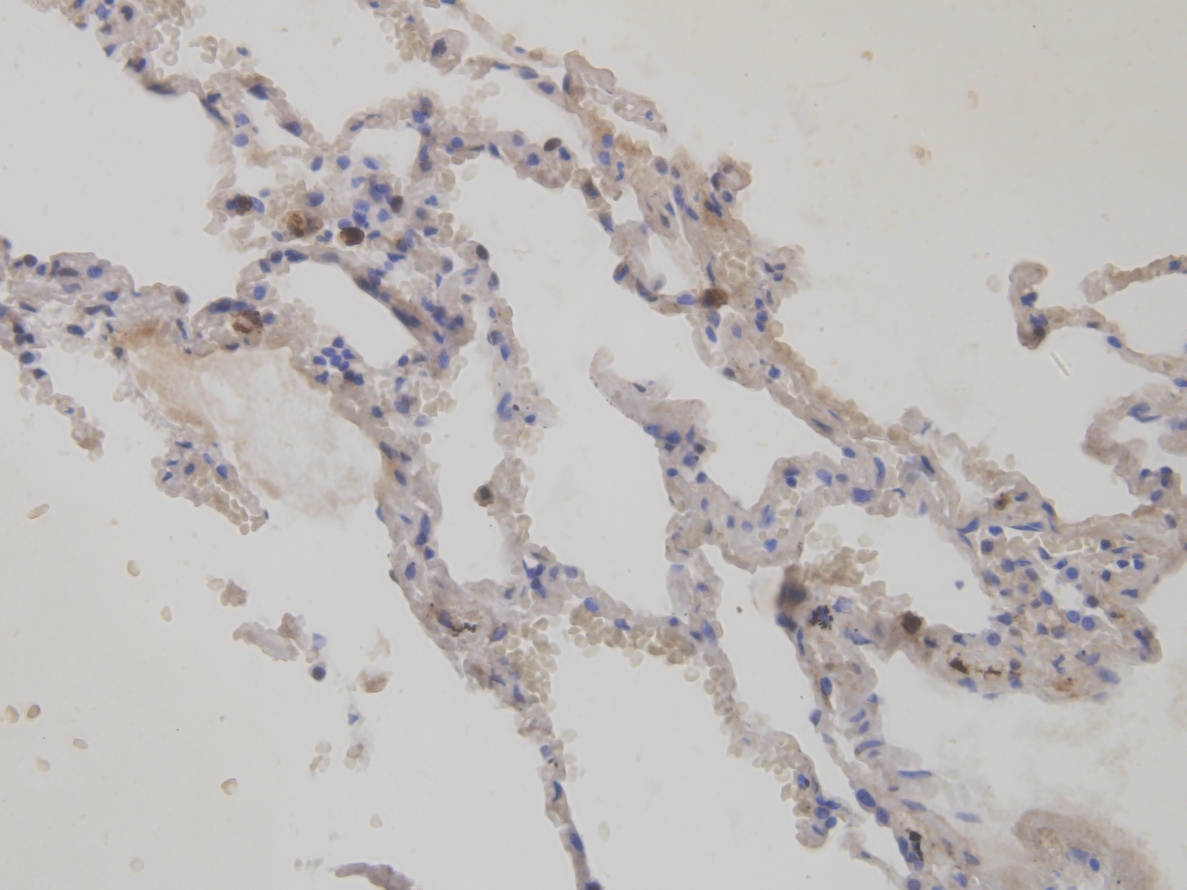

Supplement: S57 File — (ZIP) [file pone.0337223.s058.zip › 517993-400X-CA-N/517993-400X- N (3).tif]

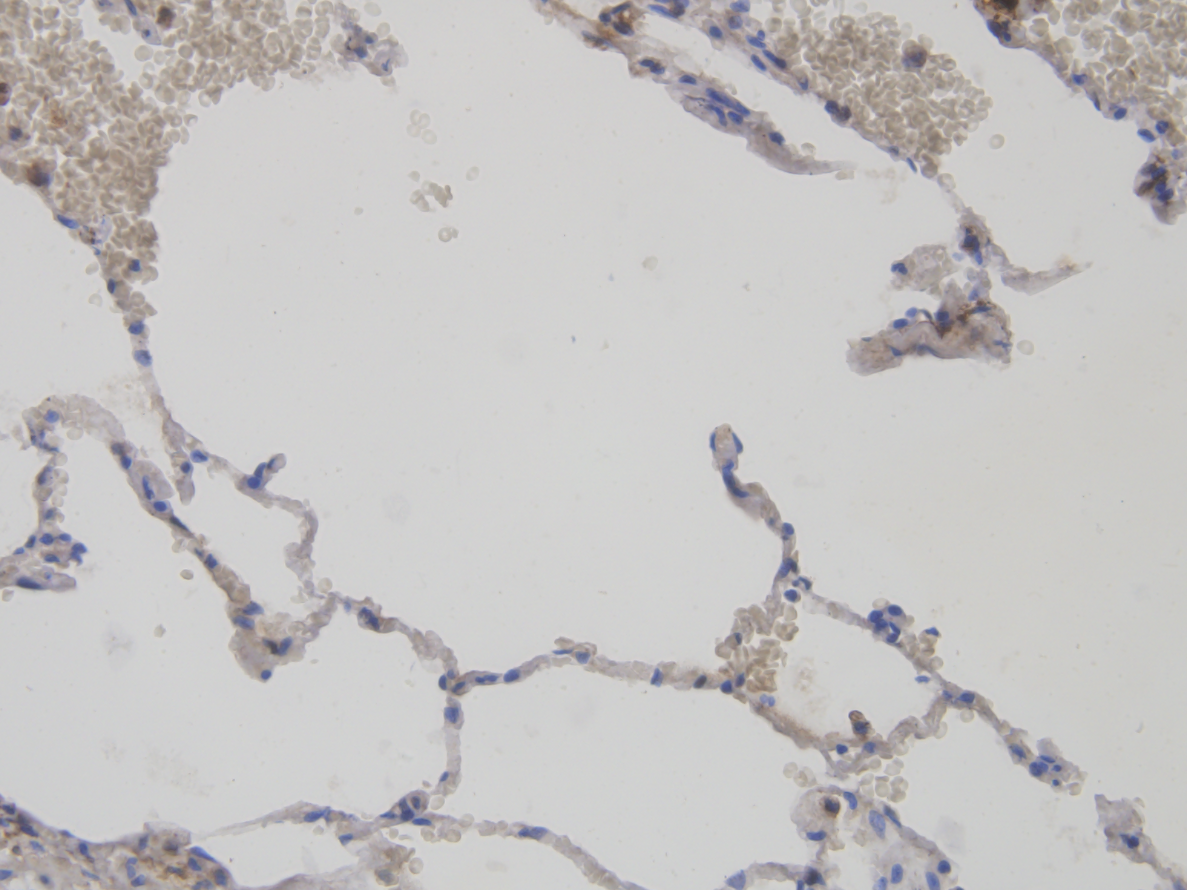

Supplement: S57 File — (ZIP) [file pone.0337223.s058.zip › 517993-400X-CA-N/517993-400X- N (4).tif]

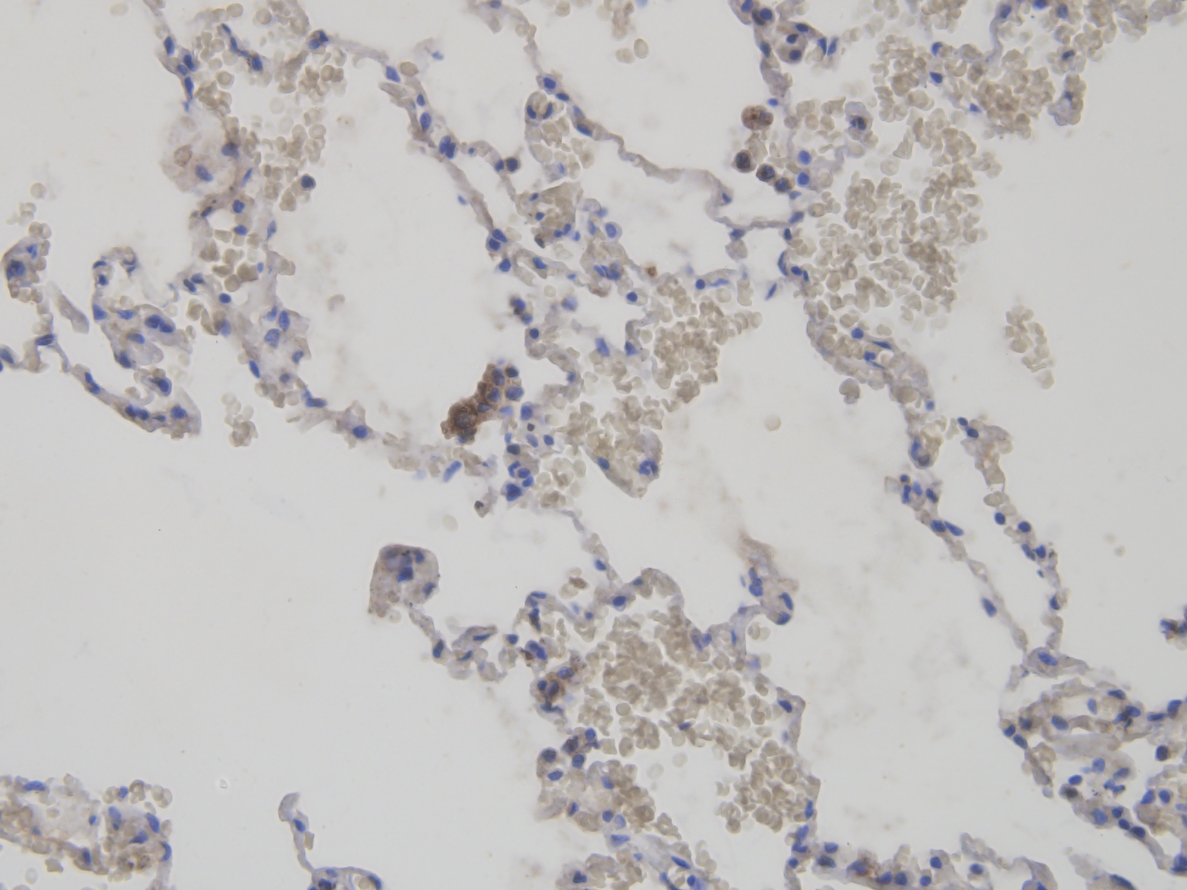

Supplement: S57 File — (ZIP) [file pone.0337223.s058.zip › 517993-400X-CA-N/517993-400X- N (5).tif]

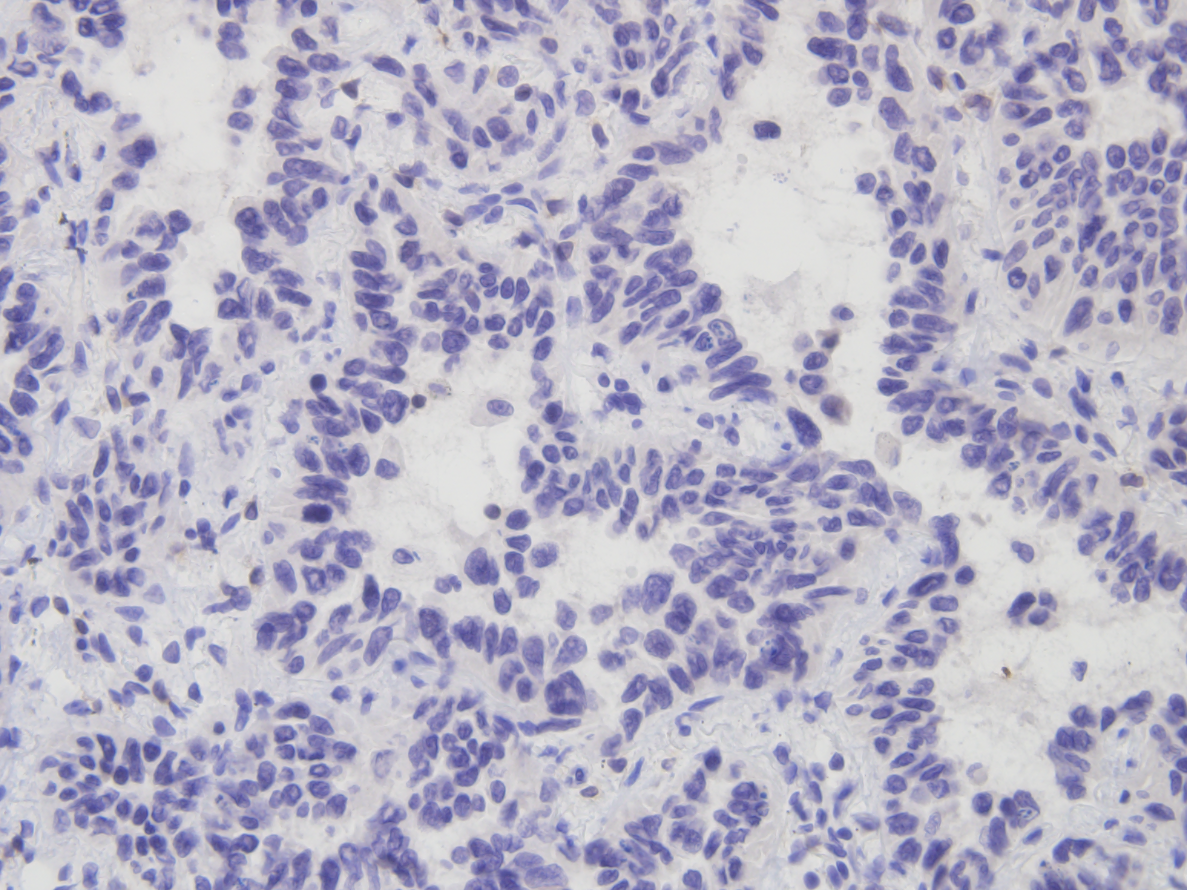

Supplement: S57 File — (ZIP) [file pone.0337223.s058.zip › 517993-400X-CA-N/517993-400X-CA (1).tif]

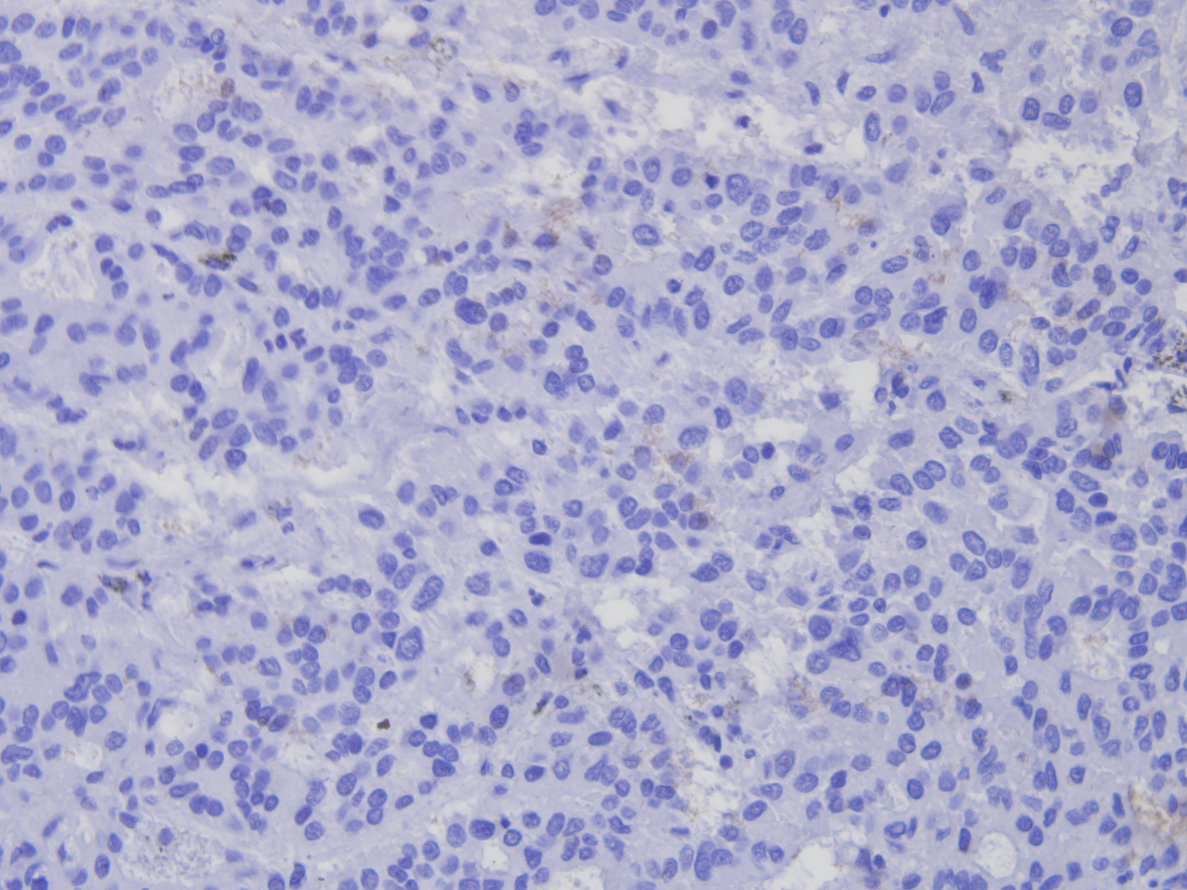

Supplement: S57 File — (ZIP) [file pone.0337223.s058.zip › 517993-400X-CA-N/517993-400X-CA (2).tif]

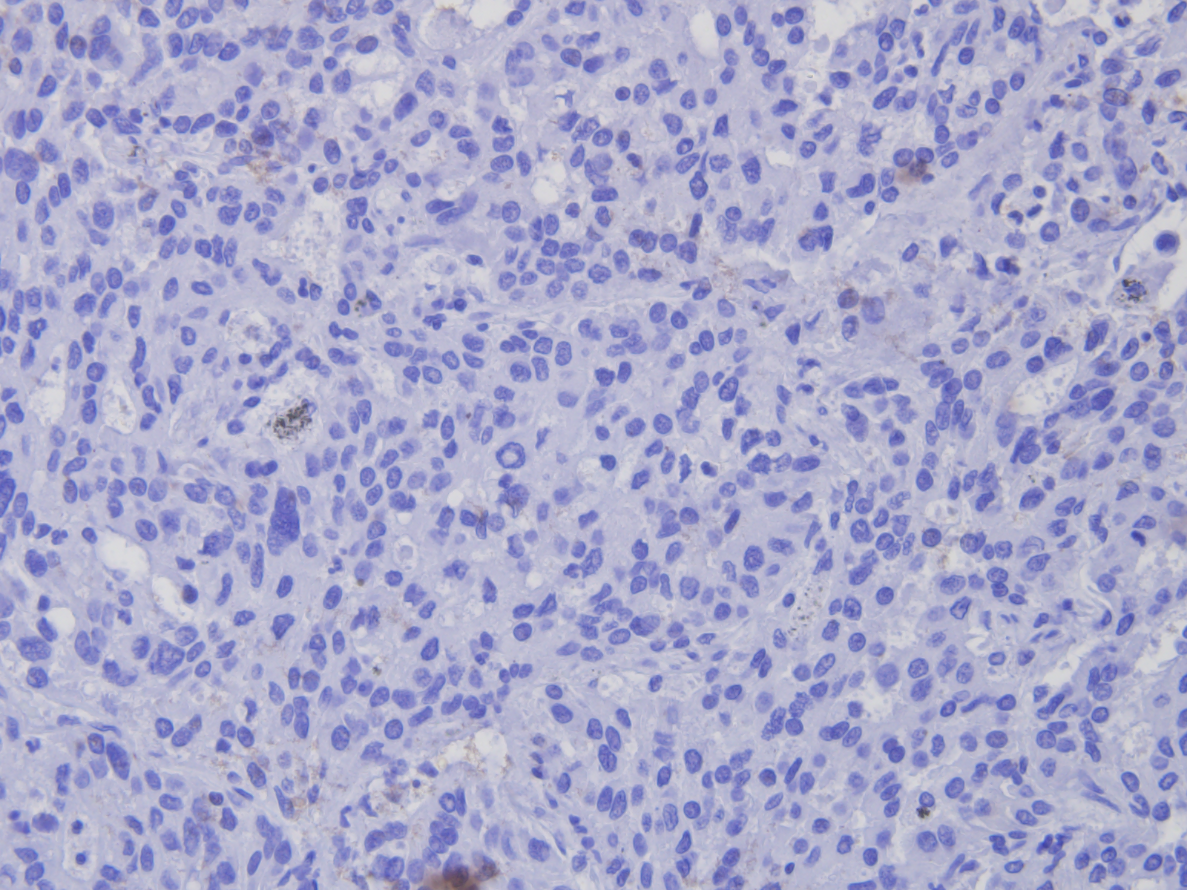

Supplement: S57 File — (ZIP) [file pone.0337223.s058.zip › 517993-400X-CA-N/517993-400X-CA (3).tif]

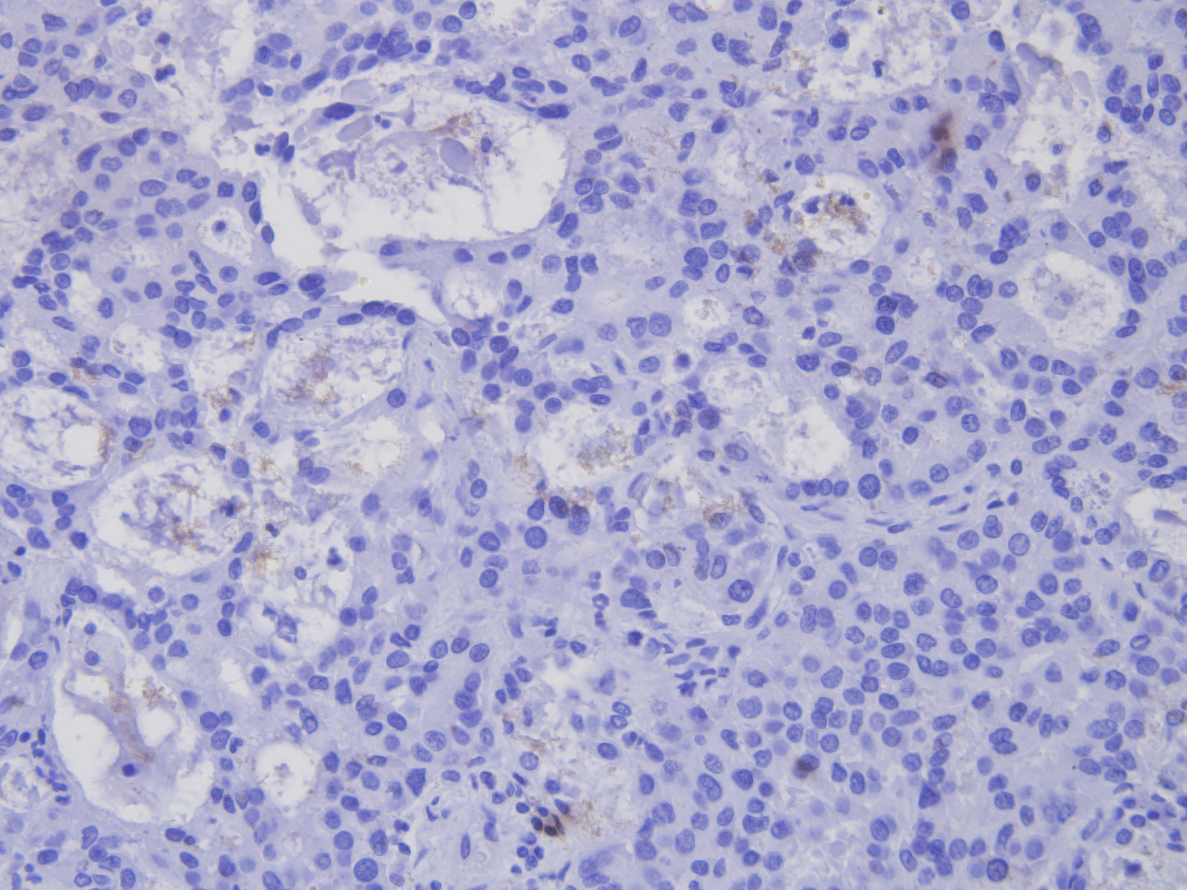

Supplement: S57 File — (ZIP) [file pone.0337223.s058.zip › 517993-400X-CA-N/517993-400X-CA (4).tif]

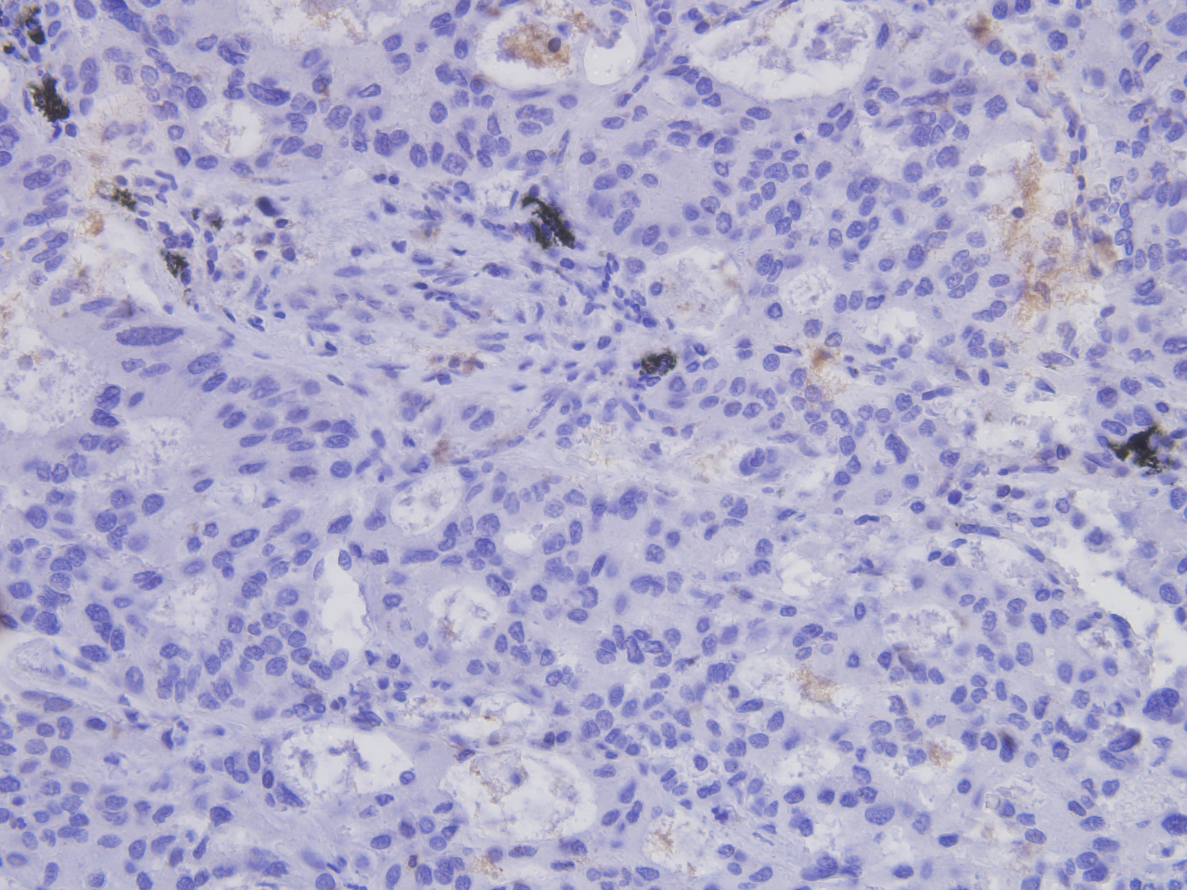

Supplement: S57 File — (ZIP) [file pone.0337223.s058.zip › 517993-400X-CA-N/517993-400X-CA (5).tif]
